# Climate vulnerability and governance impact the payoff of policy drivers for SDG 7 outcomes in BRICS countries

**DOI:** 10.1016/j.isci.2026.117700

**Published:** 2026-09-22

**Authors:** Avik Sinha, Abhinav Jindal, Shujaat Abbas

**Affiliations:** 1 Gnanam School of Business, Thanjavur, India; 2 ARUCAD Research Centre, Arkin University of Creative Arts and Design, Kyrenia, Northern Cyprus, Mersin 10, Turkey; 3 Center for Sustainable Development and Green Innovation Research, Bahçeşehir Cyprus University, Nicosia, TRNC Mersin 10, Turkey; 4 Power Management Institute, Noida, India; 5 NTPC School of Business, Noida, India; 6 Graduate School of Economics and Management, Ural Federal University, Ekaterinburg, Russian Federation

**Keywords:** BRICS, climate vulnerability, clean energy transition, energy access and efficiency, governance quality, sustainable development goal 7

## Abstract

Achieving Sustainable Development Goal 7 (SDG 7) in large emerging economies requires energy policies that operate under diverse institutional and climatic conditions. We examine BRICS countries over 2000–2022 to assess how climate vulnerability and governance quality condition the estimated marginal effects of environmental policy stringency, innovation, financial development, inequality, and demographic structure on energy access, renewable energy, and energy efficiency. A two-stage empirical framework combines moderated GLS estimation with post-estimation evaluation of marginal-effect surfaces across contrasting governance-vulnerability regimes and country-specific conditions. The results show that stronger governance and lower climate vulnerability are generally associated with more favorable estimated effects of finance, innovation, and policy stringency, while weak governance and high vulnerability can weaken or reverse these relationships. Policy priorities also differ across SDG 7 subgoals and BRICS economies. These findings support context-specific energy policies that align institutional strengthening and climate resilience with SDG 7 implementation.

## Highlights

• Governance quality conditions the effectiveness of SDG 7 policy drivers
• Climate vulnerability can weaken or reverse estimated policy effects
• Policy priorities vary across SDG 7 subgoals and BRICS economies
• Context-specific governance and resilience support SDG 7 implementation

## Graphical abstract

**Figure:**
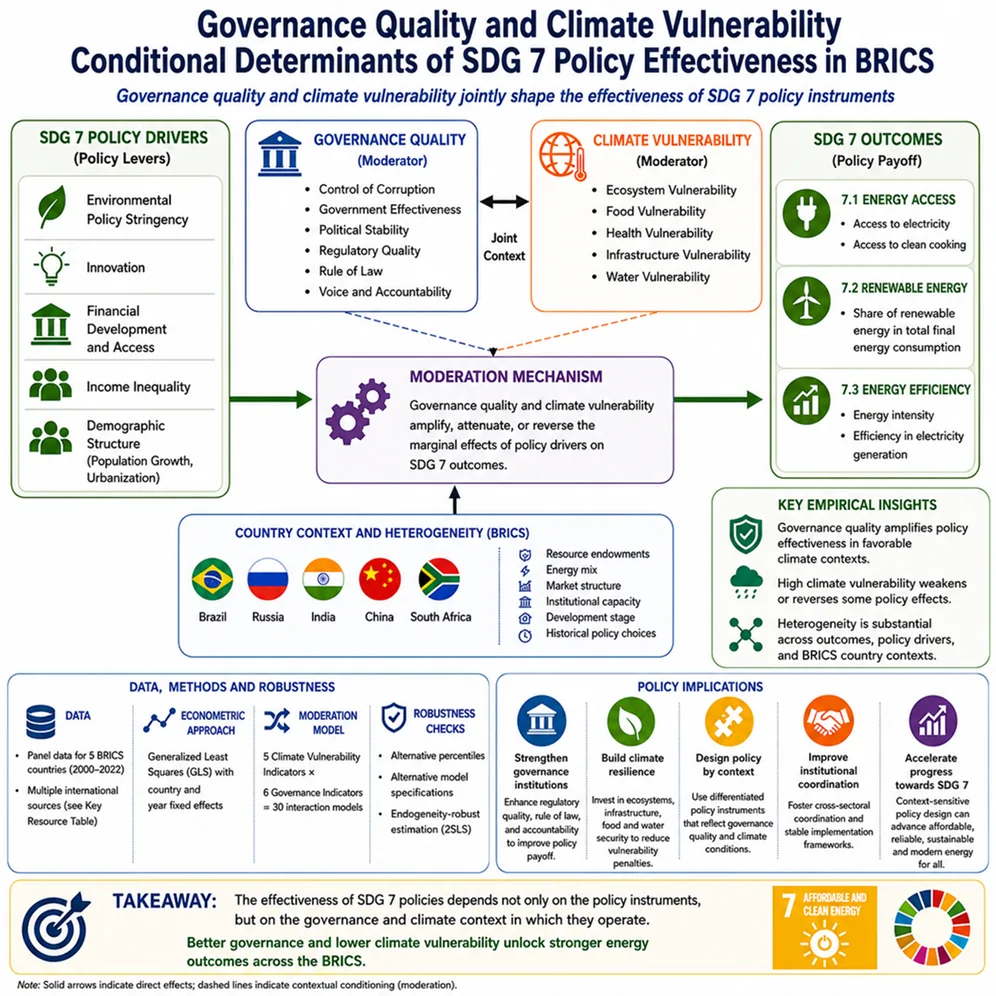

## Teaser

Economics; Environmental policy

## Introduction

Sustainable Development Goal 7 (SDG 7) targets universal access to affordable, reliable, sustainable, and modern energy (Table 1). However, most of the developing economies around the world have not been able to achieve the SDG7 objectives.^1^ Despite considerable policy efforts and growing investments in renewable energy, many nations in the Global South are falling short of this goal. The BRICS countries—Brazil, Russia, India, China, and South Africa—constitute a unique cohort within this challenge, as they represent over 40% of the global population and nearly 50% of global greenhouse gas emissions.

**Table 1:** Overview of SDG 7 subgoals and their policy relevance

SDG 7 subgoal	Official UN indicator	Measurement (Unit)	Broad policy relevance
**SDG 7.1.1**	proportion of population with access to electricity	percentage of total population (%)	measures progress toward universal electricity access and energy inclusion.
**SDG 7.1.2**	proportion of population with primary reliance on clean fuels and technologies for cooking	percentage of total population (%)	assesses access to clean cooking solutions, reducing reliance on traditional biomass and improving health and environmental outcomes.
**SDG 7.2.1**	renewable energy share in total final energy consumption	percentage of total final energy consumption (%)	captures the transition toward renewable energy and decarbonization of the energy system.
**SDG 7.3.1**	energy intensity measured in terms of primary energy and GDP	megajoules per constant 2017 PPP USD GDP (MJ/2017 PPP USD)	reflects improvements in energy efficiency and the ability to generate economic output using less energy.

Climate change is altering both supply and demand for energy: more frequent heatwaves, floods, droughts and storms directly damage infrastructure, change seasonal demand patterns, and increase the cost and uncertainty of investments in generation and transmission.^2^^,^^3^ In such a setting, regulation and policy do not operate against a neutral backdrop; in fact, they act as enablers for creating the desired impact.^4^ Weak enforcement, inconsistent rules, or fragile institutions can blunt the returns from innovation, finance, or subsidy programs, whereas strong governance can amplify the same levers.

Clearly, energy access, affordability, and sustainability are immediate development priorities for BRICS, but these priorities are now unfolding under growing climatic stress and institutional heterogeneity. Even as BRICS countries account for a large proportion of global energy demand and emissions, they differ sharply in regulatory reach, financial depth, innovation capacity, social inequality and demographic trends; these differences shape how quickly and equitably each country can deliver SDG 7—affordable, reliable and clean energy. Figure 1 illustrates the underlying heterogeneity in progress toward SDG 7 subgoals across the BRICS countries. Clearly, China and India exhibit comparatively stronger progress, particularly in expanding electricity access and clean energy deployment, whereas Brazil and South Africa show more uneven performance, and Russia lags notably in the transition toward sustainable energy. This heterogeneity underscores that progress toward affordable, reliable, and clean energy is not uniform even among large emerging economies with comparable development ambitions. Overall, Figure 1 provides the motivation to examine these divergent trajectories and sets the empirical context for analyzing how differences in governance quality and climate vulnerability influence them.

**Figure 1:**
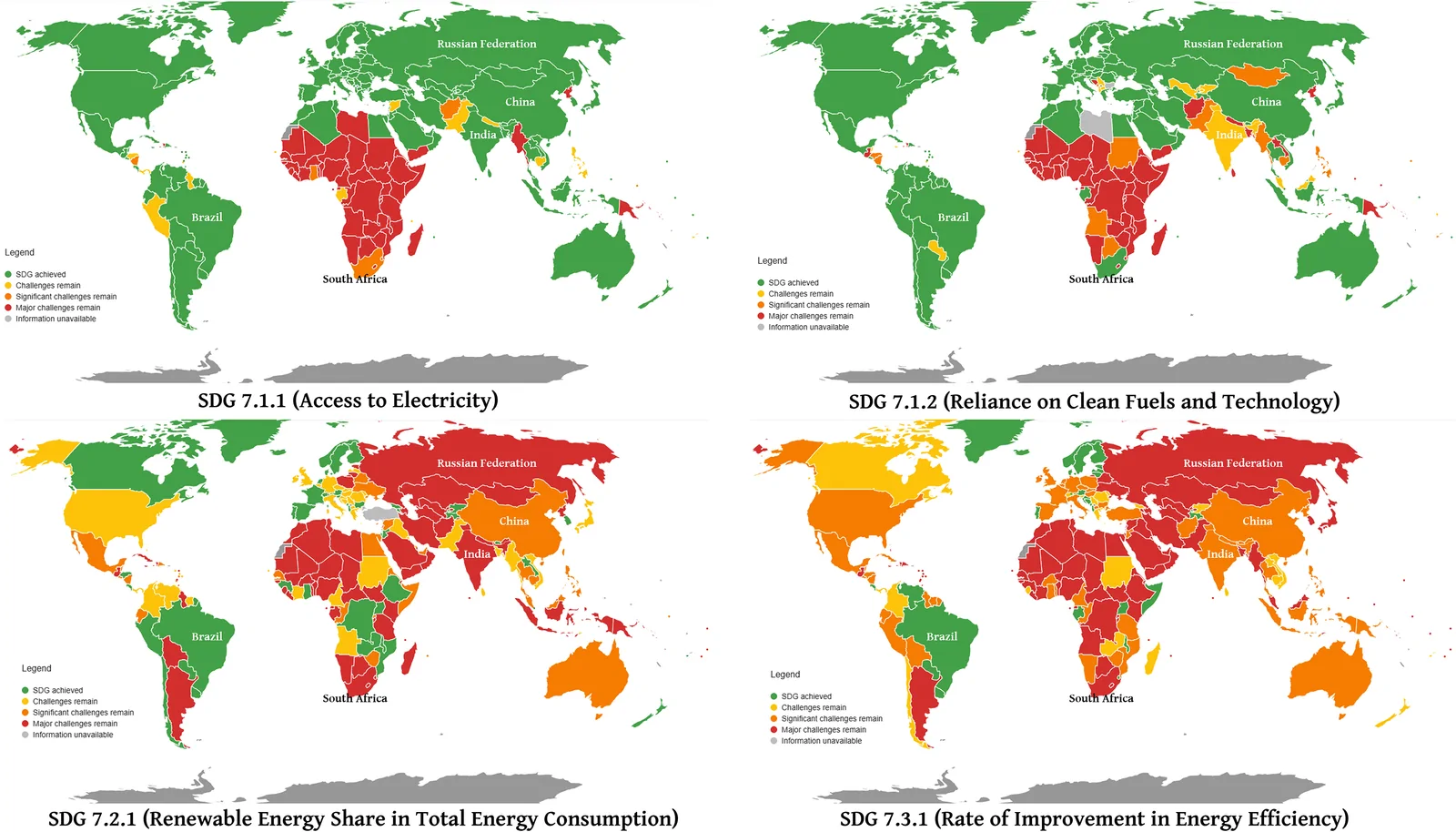
Status of SDG 7 attainment across the BRICS (Brazil, Russian Federation, India, China, and South Africa) countries Note: The maps are adopted from Sachs et al.^5^

The core problem we address is: *how do climate vulnerability and governance quality jointly shape the ability of BRICS countries to meet SDG 7 objectives*? While the existing literature has separately addressed the impact of climate risks^6^ and regulatory frameworks^7^ on energy transition, there is limited integrated empirical evidence on how these two dimensions interact and affect SDG 7 outcomes. This question matters because failure to deliver clean, affordable energy in these large economies would undermine poverty reduction, public health, and global climate goals; it also matters because average, one-size-fits-all policy prescriptions risk being ineffective or even counterproductive when climatic shocks and institutional weaknesses alter payoffs.

Empirical literature has made substantial progress on pieces of this puzzle—showing how policy stringency, innovation and finance affect renewable uptake,^7^^,^^8^^,^^9^^,^^10^^,^^11^ and how inequality and demographics influence energy demand and access^12^^,^^13^^,^^14^^,^^15^^,^^16^ —but it rarely links those levers to the context of climate vulnerability and governance as active moderators. Recent nature-family contributions underline parts of this story: urban and system-level studies show that future climate variability and extreme events can materially change optimal energy planning and reliability outcomes,^3^ while energy-system modeling that incorporates extreme weather reveals alternative investment and resilience trade-offs in storm-prone settings.^2^

Work on decent-living energy shows how country-specific needs and inequalities alter the energy required to deliver basic wellbeing,^17^ and analyses of inequality emphasize that social distributions can dramatically change the scale and character of energy transitions.^13^ Parallel policy work highlights that lowering financing costs and strategic public finance can be decisive for affordable transitions in developing regions.^18^^,^^19^

What is missing, however, is a systematic empirical account of how the effectiveness of conventional SDG 7 policy drivers changes across different institutional and climatic environments. Our study addresses this gap by treating governance quality and climate vulnerability as interacting contextual conditions rather than static background controls. This moderation perspective advances the theoretical understanding of SDG 7 implementation by demonstrating that policy effectiveness is conditional on institutional and climatic context rather than being universally constant. By combining moderated estimation with post-estimation scenario evaluation, the study moves beyond average effects to identify governance-vulnerability regimes within which the estimated marginal effectiveness of policy interventions systematically differs.

For this purpose, we use a two-stage empirical framework analyzing data on key SDG drivers from 2000–2022 focused on policy relevance.^†^ First, a static marginal impact analysis estimates how key drivers of SDG 7, i.e., environmental policy stringency, innovation, financial development, income inequality, and demographic structure affect various SDG 7 outcomes, viz. population access to electricity (SDG 7.1.1), renewable share (SDG 7.1.2 and SDG 7.2.1), or energy intensity (SDG 7.3.1) across BRICS countries, while allowing climate vulnerability and governance indicators to shape the said impacts. Figure 2 shows the conceptual framework. We compute marginal effects for each driver and then quantify how often (percentage of cases) those marginal effects fall or rise, or the impacts become positive/negative as climate vulnerability and governance quality change. This produces an intuitive sensitivity metric: which policy levers tend to lose potency under climatic stress or weak institutions, and which hold up.

**Figure 2:**
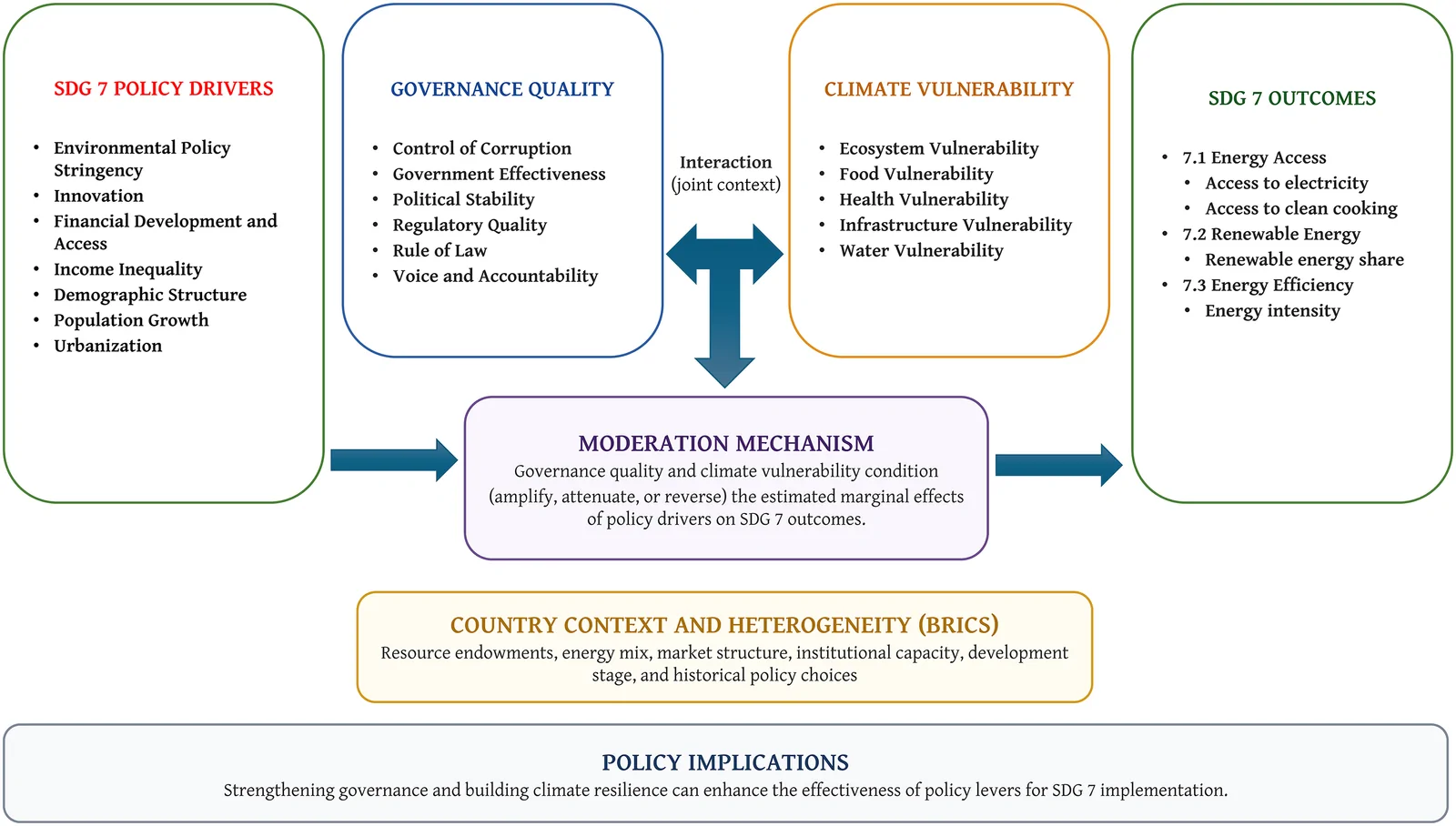
Conceptual framework of the study See Figures S1–S7; Tables S1–S6.

Second, an extreme marginal impact analysis constructs four governance-vulnerability regime combinations: low vulnerability-high governance, low vulnerability-low governance, high vulnerability-high governance, and high vulnerability-low governance. This is done by setting vulnerability and governance indicators at extreme high and low percentiles and simulating marginal responses of each SDG driver within those regimes. The extreme regime evaluation makes conditionality explicit and tangible by illustrating how the estimated marginal return to policy tightening differs between a well-governed, climatically stable regime and a climate-stressed, low-capacity regime.

The static marginal impact analysis is extended to the individual countries to understand how the priority SDG 7 subgoals differ across countries. The impacts of SDG drivers are measured, followed by the severity of counterproductive impacts. This produces a country-level sensitivity metric that summarizes which policy levers exhibit weaker estimated marginal effects under climatic stress or weak institutions, and for which countries.

Together, these methods map both average and regime-specific response surfaces for the policy, innovation, finance, inequality, and demographic levers most relevant to SDG 7. The result is a compact summary of which interventions exhibit relatively robust estimated marginal effects across contexts, which appear more fragile, and where governance or finance reforms may strengthen the effectiveness of other measures. The theoretical contribution of this framework is 2-fold. First, it conceptualizes governance quality and climate vulnerability as interacting institutional conditions that jointly shape policy effectiveness rather than as independent determinants of SDG 7 outcomes. Second, it introduces a governance-vulnerability regime perspective that translates continuous interaction effects into policy-relevant institutional environments, thereby providing a structured framework for understanding why identical policy instruments may produce different outcomes across countries facing different governance and climate conditions.

Results highlight the varying effects of different policies and socioeconomic variables under climate vulnerability and governance moderation across BRICS countries in relation to the SDG7 goals. The static marginal impact analysis shows that variables such as financial access, income inequality, health expenditure, policy stringency, and environmental innovation generally display desirable impacts under moderation, suggesting that improvements in governance or climate resilience enhance their positive effects on sustainable energy outcomes. Conversely, variables such as electricity generation, rural and urban population growth, per capita income, and energy demand often show undesirable changes under moderation, indicating that they may require stronger or more targeted policy interventions to ensure progress toward SDG7. Policy interventions are determined based on the count of policy levers having counterproductive impacts on SDG 7 subgoals. SDG 7.1.2 is the priority SDG in terms of required number of interventions, followed by SDG 7.3.1, 7.1.1, and 7.2.1.

The scenario-based analysis for stylized climate vulnerability-governance quality combinations reiterates the above finding by simulating markedly different response regimes, which lead to significant changes in the impact of policy drivers on SDG 7 outcomes. Not surprisingly, under high climate vulnerability-poor governance quality, a key attribute of several BRICS countries, several policy drivers, viz. policy stringency, financial development, and others, become ineffective, whereas under low vulnerability-high governance, many policy drivers such as innovation become highly potent and effective. This suggests that the same intervention may strengthen, weaken, or reverse its impact depending on institutional context and climatic stress. Based on the count of policy levers having counterproductive impacts on SDG 7 subgoals, SDG 7.1.1 is the priority SDG in terms of required interventions, followed by SDG 7.2.1, 7.1.2, and 7.3.1.

The country-level results reveal that BRICS countries must adopt differentiated yet coordinated policy approaches to effectively achieve SDG7 goals. The countries have their own sets of priority SDG 7 subgoals based on the severity of the required interventions, with Russia having the highest number of policy intervention points and India having the lowest.

These results offer timely evidence for the upcoming global revision of SDG 7, demonstrating that existing subgoals centered on energy access, clean cooking, renewable energy, and efficiency do not adequately reflect the structural conditions governing energy transitions in climate-vulnerable, institutionally diverse economies.

## Results

The marginal impacts of SDG 7 subgoal policy drivers on SDG 7 subgoals are first estimated for 30 climate vulnerability-governance quality indicator combinations by combining the 5 climate vulnerability indicators (ecosystem vulnerability, food vulnerability, health vulnerability, infrastructure vulnerability, and water vulnerability) and 6 governance quality indicators (voice and accountability, political stability, government effectiveness, regulatory quality, rule of law, and control of corruption) [Figure 3A]. For each evaluation, these marginal impacts are computed under the baseline specification and the moderated specification (Tables S7–S46). Comparing the two sets of marginal effects identifies how the estimated impacts of the SDG 7 drivers vary after accounting for climate vulnerability and governance quality.

**Figure 3:**
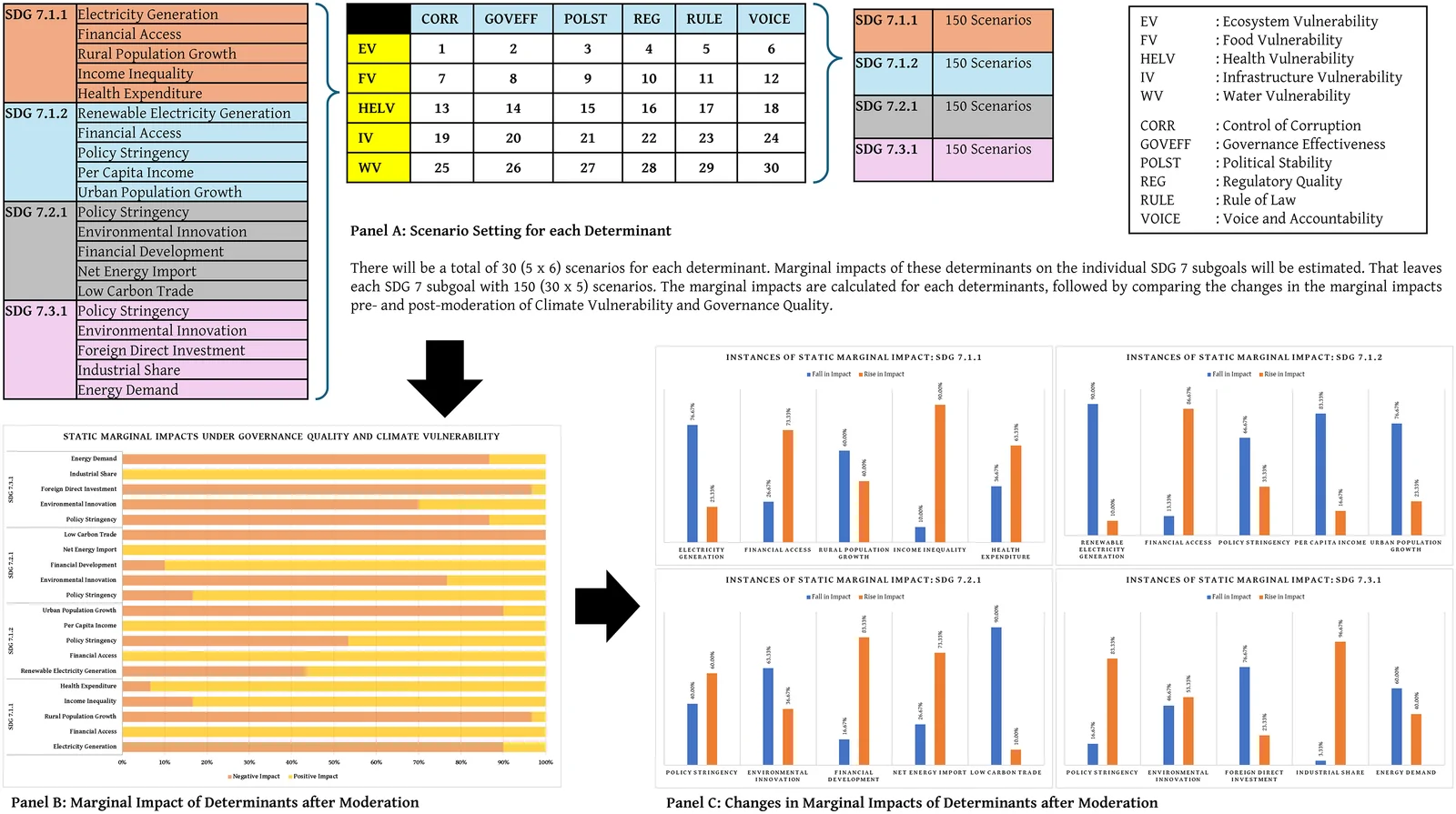
Simulation Setup and pre-post comparison of Static Marginal Impacts of SDG 7 subgoal determinants under the influence of governance quality and climate vulnerability See Tables S7–S46 and S63–S103.

### Differentiated impacts of policy drivers for SDG 7 outcomes

We start by capturing the static marginal impacts of SDG 7 subgoal drivers by allowing climate vulnerability and governance quality to moderate the impacts (Figure 3B). We observe that increases in financial access, income inequality, and health expenditure exert a positive impact on SDG 7.1.1, while rises in electricity generation and rural population growth deter it. The estimated negative marginal impact of electricity generation is consistent with the possibility that electricity distribution infrastructure is vulnerable to climatic adversities, potentially contributing to unserved energy demand,^20^ while stronger institutional capacity can make electricity less available to the poor.^21^ Also, the gradually declining trend of health expenditure in BRICS enables people to have more access to electricity.^22^

The rise in SDG 7.1.2 is due to improvements in renewable electricity generation, financial access, and per capita income, while it declines with the rise in policy stringency and urban population growth. One possible explanation is that climate vulnerability affecting urban infrastructure may increase reliance on fossil fuels to meet urban energy demand.^23^ The estimated negative association should be interpreted within the conditional interaction framework adopted in this study rather than as evidence that environmental policy stringency is inherently detrimental to SDG 7 outcomes. Under relatively weak governance or limited climate-related policy urgency, stricter environmental regulations may increase compliance costs and investment uncertainty before complementary institutional capacity, financing mechanisms, and technological adaptation are fully established. Consequently, the short-run marginal effect of policy stringency may become weaker or even negative despite its expected long-run contribution to clean energy transitions. This interpretation is consistent with studies emphasizing that regulatory effectiveness depends critically on implementation capacity and institutional quality rather than policy stringency alone.^24^

Higher levels of policy stringency, financial development, and net energy imports are estimated to be positively associated with SDG 7.2.1, while a rise in environmental innovation and low-carbon trade causes its decline. One possible explanation is that governance arrangements favoring economic growth may be associated with greater renewable-energy access among higher-income groups.^25^ It is also attributed to declining energy imports, while opposite evidence exists.^26^ The estimated negative marginal effect of environmental innovation should likewise be interpreted as conditional rather than universal. Environmental innovation generally contributes to cleaner energy systems; however, its effectiveness depends on complementary conditions, including technology diffusion, financing availability, institutional coordination, and market readiness. Under high climate vulnerability or weak governance, these complementary conditions may be insufficient, delaying the translation of innovation into measurable improvements in renewable-energy deployment. Consequently, innovation investments may exhibit weaker or even temporarily adverse marginal effects despite their long-term benefits.^26^^,^^27^

An increase in industrial share is estimated to be positively associated with SDG 7.3.1, while a rise in policy stringency, environmental innovation, foreign direct investment, and energy demand are negatively associated with it. This finding differs from previous evidence.^28^ A plausible explanation is that higher energy demand under climate vulnerability may accelerate investments in renewable energy and energy-efficient technologies, thereby moderating energy intensity.^29^ This relationship, however, should be interpreted within the conditional interaction framework adopted in this study. Although previous studies generally report positive effects of environmental policy stringency and environmental innovation on energy efficiency, the present analysis evaluates conditional marginal effects after accounting for governance quality and climate vulnerability. Consequently, the observed differences should not be interpreted as contradictions of the existing literature but as evidence that the effectiveness of these policy instruments depends on the prevailing institutional and climatic context.

### Counterproductive impact of some policy drivers on SDG7 outcomes

These simulation outcomes are shown on C of Figure 3. We find the impacts of electricity generation and rural population growth fall for SDG 7.1.1, while impacts of financial access, income inequality, and health expenditure rise. Strong governance catalyzes the reduction in the impact of rural population growth.^30^ The rise in the impact of financial access is a result of affirmative governance action,^31^ while declining health expenditure helps people with additional disposable income.^22^

For SDG 7.1.2, renewable electricity generation, environmental policy stringency, per capita income, and urban population growth lose their impacts, while the impact of financial access increases. Susceptibility of urban infrastructure to climate vulnerability reduces the impacts of renewable electricity generation and urban population growth,^23^ while stronger institutional capacity reduces the impact of per capita income by prioritizing fossil fuel contracts.^32^^,^^33^

The impacts of environmental innovation and low-carbon trade fall under SDG 7.2.1, where a rise is seen for policy stringency, financial development, and net energy imports. Exposure of energy generation infrastructure to climate vulnerability reduces the impact of environmental innovation and low-carbon trade,^26^^,^^27^ while this exposure, clubbed with governance, might boost the impacts of policy stringency and energy imports.

Lastly, the impacts of foreign direct investment and energy demand decline for SDG 7.3.1, while the impacts of policy stringency, environmental innovation, and industrial share rise. Exposure to climate vulnerability reduced the impact of foreign direct investment on energy intensity,^34^ which is increased by effective governance through catalyzing policy stringency and innovation.

### Extreme governance-vulnerability stylized regimes cause change in impact of SDG 7 drivers

We simulate the marginal impacts of the SDG 7 subgoal determinants under the entire spectrum of governance quality and climate vulnerability to understand their behavior under extreme moderating conditions (Figures S8–S107). The scenario setting is shown in A of Figure 4. Then we analyze the marginal impacts of SDG 7 subgoal determinants under the joint influence of governance quality and climate vulnerability indicators, followed by segregating them.

**Figure 4:**
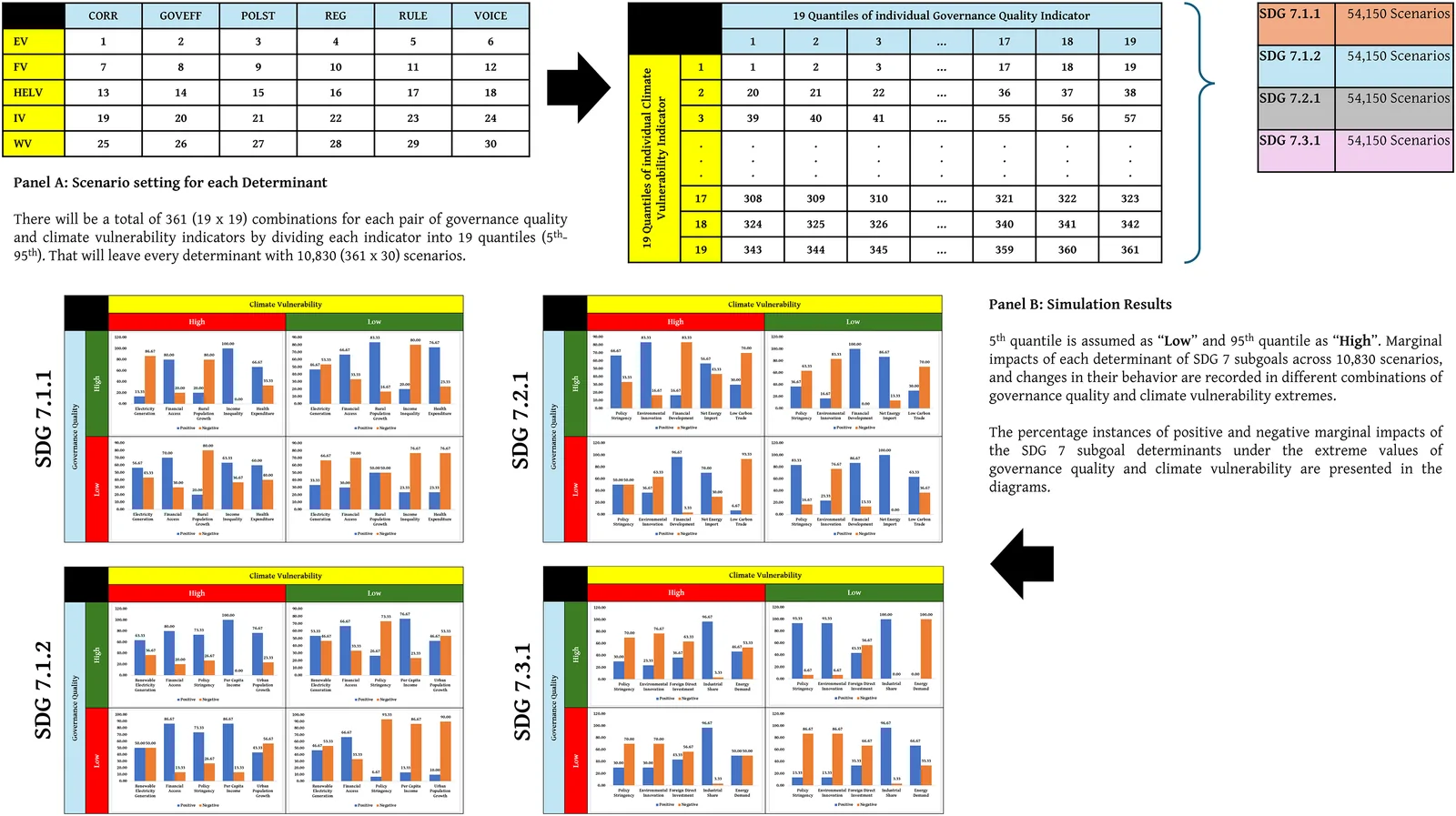
Simulation Setup and comparison of Static Marginal Impacts of SDG 7 subgoal determinants under influence of extreme conditions of Governance Quality and Climate Vulnerability See Figures S8–S107.

We start by analyzing the simulation results reported in B of Figure 4. We first analyze changes in the marginal impact of SDG 7.1.1. Electricity generation shows a more positive impact under low governance quality and high climate vulnerability. Weak governance reduces regulatory and bureaucratic obstacles and allows rapid expansion,^35^ while one possible explanation is that climate vulnerability may increase reliance on extending electricity access for adaptation needs.^36^ Financial access and health expenditure demonstrate more negative impacts under low governance quality and climate vulnerability. Weak governance enables financial access by misallocating credit mainly to commercial actors rather than to poor populations lacking electricity.^37^ Similarly, higher health expenditure fails to improve community services by diverting public resources away from basic infrastructure, such as electrification,^38^ while high climate vulnerability leads financial and health systems to reduce investment in electricity networks.^39^ Income inequality has more negative impacts under low climate vulnerability. Without climate-driven urgency to expand energy access, infrastructure investments tend to favor wealthier groups and commercially attractive regions, leaving poorer households underserved. Rural population growth has more negative impacts under high governance quality and low climate vulnerability. Regulatory requirements slow rural grid extension,^40^ and this finding differs from previous evidence.^41^ This result may reflect the fact that, under relatively low climate vulnerability, weaker adaptation incentives amplify the adverse effects of rural population growth on electricity access. Accordingly, the estimated relationship should be interpreted as conditional on the prevailing governance and climate conditions.

Moving on to SDG 7.1.2, renewable electricity generation shows a more positive impact only under high governance quality. Higher institutional quality helps strategically align energy policy with futuristic industrial development,^42^ while ensuring household renewable electricity consumption. Financial access has more positive impacts across scenarios. Availability of credit or microfinance can help overcome the costs of adopting clean fuels and equipment.^43^ We find more negative impacts of environmental policy stringency under low climate vulnerability. Low climate pressures help prioritize compliance and environmental standards over affordability, making clean fuels less accessible to low-income households.^44^ Per capita income records more negative impacts under low governance quality and climate vulnerability, and urban population growth records more positive impacts under high governance quality and climate vulnerability. Absence of strong governance can make higher income increase demand for traditional fossil fuels,^45^ while policy misalignment for climate vulnerability advances it.^46^ Strong governance prioritizes reliable energy infrastructure by enabling growing urban populations to adopt clean technologies,^47^ while high climate vulnerability further accelerates it by promoting electric or low-emission household fuels.^27^

For SDG 7.2.1, policy stringency records more negative impacts for high governance quality and low climate vulnerability. High-governance systems may enforce more rigorous environmental regulations that increase compliance requirements and project evaluation standards.^48^ Under relatively low climate vulnerability, where the urgency for rapid renewable deployment is reduced, these additional regulatory requirements may temporarily slow investment decisions and reduce the short-run marginal effectiveness of policy stringency.^49^ Accordingly, the estimated negative effect reflects transitional implementation costs under specific governance-vulnerability conditions rather than an inherent limitation of environmental regulation. Environmental innovation has more positive impacts for high governance quality and climate vulnerability, whereas financial development has more negative impacts. These findings differ from studies reporting uniformly positive effects of environmental innovation because the present analysis estimates conditional marginal effects rather than average effects. Innovation is expected to improve SDG 7 outcomes only when supported by effective governance, financing, and technology diffusion mechanisms. Where these complementary conditions are weak, innovation may require longer adjustment periods before measurable improvements become evident.^50^^,^^51^ In contrast, better governance quality channels financial resources toward conventional energy to stabilize growth,^50^ especially when climate risks intensify pressure on the economy. Net energy import records scenario-wide more positive impacts. The impact of low-carbon trade is more positive for low governance quality and climate vulnerability. Low governance makes internal policy frameworks fail to support renewable energy,^47^ but importing low-carbon technologies and cleaner energy products can help expand renewable capacity.^52^ High climate vulnerability increases demand for resilient and affordable solutions, thereby creating scope for international low-carbon goods and technology transfers.^47^

Lastly, for SDG 7.3.1, policy stringency and environmental innovation record more positive impacts for high governance quality and low climate vulnerability. This finding differs from previous evidence.^53^^,^^54^ The observed divergence may arise because strong governance under relatively low climate vulnerability prioritizes production stability and energy security, thereby weakening the expected influence of environmental policy stringency and innovation on energy intensity. This interpretation reflects the conditional interaction effects estimated in the present study. Foreign direct investment and industrial share have cross-scenario negative and positive impacts, respectively. Many FDI projects prioritize production growth and cost competitiveness rather than efficient energy use, while industries face stronger incentives to reduce energy costs.^55^ Energy demand has more positive impacts for low governance quality and climate vulnerability. In low climate vulnerability settings, energy systems face fewer disruptions, and an increase in demand pressure leads to inefficient use of available energy.^56^

### Segregated extreme moderating impacts in BRICS policy simulation reveal diverse policy intervention points

As shown in Table 2, we bifurcate these impacts into policy actions by individually focusing on how governance quality and climate vulnerability shape the impacts of the SDG 7 subgoal determinants.

**Table 2:** Percentage Instances of Positive and Negative Impacts of SDG 7 subgoal determinants in presence of Governance Quality and Climate Vulnerability

SDG 7.1.1 determinants		Governance quality		Climate vulnerability		Action required	
		High	Low	High	Low	Governance quality	Climate vulnerability
Electricity Generation	positive	30.00%	45.00%	35.00%	40.00%	high dominance	high dominance
	negative	70.00%	55.00%	65.00%	60.00%		
Financial Access	positive	73.33%	50.00%	75.00%	48.33%	low dominance	mixed dominance
	negative	26.67%	50.00%	25.00%	51.67%		
Rural Population Growth	positive	51.67%	35.00%	20.00%	66.67%	mixed dominance	mixed dominance
	negative	48.33%	65.00%	80.00%	33.33%		
Income Inequality	positive	60.00%	43.33%	81.67%	21.67%	mixed dominance	high dominance
	negative	40.00%	56.67%	18.33%	78.33%		
Health Expenditure	positive	71.67%	41.67%	63.33%	50.00%	mixed dominance	low dominance
	negative	28.33%	58.33%	36.67%	50.00%		

SDG 7.1.2 determinants		Governance Quality		Climate Vulnerability		Action Required	
		High	Low	High	Low	Governance Quality	Climate Vulnerability
Renewable Electricity Generation	positive	58.33%	48.33%	56.67%	50.00%	mixed dominance	low dominance
	negative	41.67%	51.67%	43.33%	50.00%		
Financial Access	positive	73.33%	76.67%	83.33%	66.67%	low dominance	low dominance
	negative	26.67%	23.33%	16.67%	33.33%		
Policy Stringency	positive	50.00%	40.00%	73.33%	16.67%	mixed dominance	high dominance
	negative	50.00%	60.00%	26.67%	83.33%		
Per Capita Income	positive	88.33%	50.00%	93.33%	45.00%	low dominance	mixed dominance
	negative	11.67%	50.00%	6.67%	55.00%		
Urban Population Growth	positive	61.67%	26.67%	60.00%	28.33%	high dominance	high dominance
	negative	38.33%	73.33%	40.00%	71.67%		

SDG 7.2.1 determinants		Governance Quality		Climate Vulnerability		Action Required	
		High	Low	High	Low	Governance Quality	Climate Vulnerability
Policy Stringency	positive	51.67%	66.67%	58.33%	60.00%	mixed dominance	mixed dominance
	negative	48.33%	33.33%	41.67%	40.00%		
Environmental Innovation	positive	50.00%	30.00%	60.00%	20.00%	high dominance	high dominance
	negative	50.00%	70.00%	40.00%	80.00%		
Financial Development	positive	58.33%	91.67%	56.67%	93.33%	low dominance	low dominance
	negative	41.67%	8.33%	43.33%	6.67%		
Net Energy Import	positive	71.67%	85.00%	63.33%	93.33%	low dominance	low dominance
	negative	28.33%	15.00%	36.67%	6.67%		
Low Carbon Trade	positive	30.00%	35.00%	18.33%	46.67%	high dominance	high dominance
	negative	70.00%	65.00%	81.67%	53.33%		

SDG 7.3.1 determinants		Governance Quality		Climate Vulnerability		Action Required	
		High	Low	High	Low	Governance Quality	Climate Vulnerability
Policy Stringency	positive	61.67%	21.67%	30.00%	53.33%	mixed dominance	mixed dominance
	negative	38.33%	78.33%	70.00%	46.67%		
Environmental Innovation	positive	58.33%	21.67%	26.67%	53.33%	mixed dominance	mixed dominance
	negative	41.67%	78.33%	73.33%	46.67%		
Foreign Direct Investment	positive	40.00%	38.33%	40.00%	38.33%	mixed dominance	mixed dominance
	negative	60.00%	61.67%	60.00%	61.67%		
Industrial Share	positive	98.33%	96.67%	96.67%	98.33%	high dominance	high dominance
	negative	1.67%	3.33%	3.33%	1.67%		
Energy Demand	positive	23.33%	58.33%	48.33%	33.33%	mixed dominance	low dominance
	negative	76.67%	41.67%	51.67%	66.67%		

Percentages summarize the proportion of marginal-effect evaluations exhibiting positive and negative impacts over the complete governance quality-climate vulnerability evaluation grid (19 × 19 quantile combinations across 5 climate vulnerability indicators and 6 governance quality indicators; 54,150 marginal-effect evaluations). For each governance quality or climate vulnerability condition, policy intervention intensity is classified according to the proportion of unfavorable marginal-effect evaluations: high dominance (>65%), mixed dominance (50–65%), and low dominance (<50%). These thresholds apply only to the continuous marginal-effect evaluation framework summarized in this table and differ from the four-regime intervention classification used in the extreme governance-vulnerability evaluation framework.

For SDG 7.1.1, greater policy action on governance quality is required to improve the impacts of electricity generation, moderate policy attention for rural population growth, income inequality, and health expenditure, and lower policy priority for financial access. Greater policy action on climate vulnerability is required for electricity generation and income inequality, moderate policy attention for financial access and rural population growth, and lower policy priority for health expenditure.

Moving on to SDG 7.1.2, greater policy action on governance quality is required to improve the impacts of urban population growth, moderate policy attention for renewable electricity generation and environmental policy stringency, and lower policy priority for per capita income and financial access. On the other hand, greater policy action on climate vulnerability is required for policy stringency and urban population growth, moderate policy attention for per capita income, and lower policy priority for renewable electricity generation and financial access.

Next, for SDG 7.2.1, greater policy action on both governance quality and climate vulnerability is required to improve the impacts of environmental innovation and low-carbon trade, moderate policy attention for environmental policy stringency, and lower policy priority for financial development and net energy imports.

Lastly, for SDG 7.3.1, greater policy action on governance quality is required to improve the impacts of environmental policy stringency, environmental innovation, and energy demand, moderate policy attention for foreign direct investment, and lower policy priority for industrial share. On the other hand, greater policy action on climate vulnerability is required for environmental policy stringency and environmental innovation, moderate policy attention for foreign direct investment and energy demand, and lower policy priority for industrial share.

### Identifying policy intervention points reveals priority SDG 7 subgoals under BAU and extreme scenarios

Policy simulation results under the BAU and extreme evaluation settings summarize how the estimated marginal effects of SDG 7 subgoal determinants vary across governance quality and climate vulnerability conditions. These patterns provide a basis for comparing the relative policy sensitivity of the SDG 7 subgoals under the two evaluation settings (Figure 5). The extreme scenarios should not be interpreted as forecasts of future governance quality or climate vulnerability states. Instead, they represent stylized stress-test environments designed to evaluate how the estimated relationships behave under relatively favorable and unfavorable institutional conditions. Such scenario-based evaluations provide a policy-oriented assessment of whether the effectiveness of SDG 7 determinants remains robust when governance quality and climate vulnerability approach the tails of their observed distributions. Consequently, the results identify policy priorities that are resilient across contrasting governance environments rather than predicting future outcomes.

**Figure 5:**
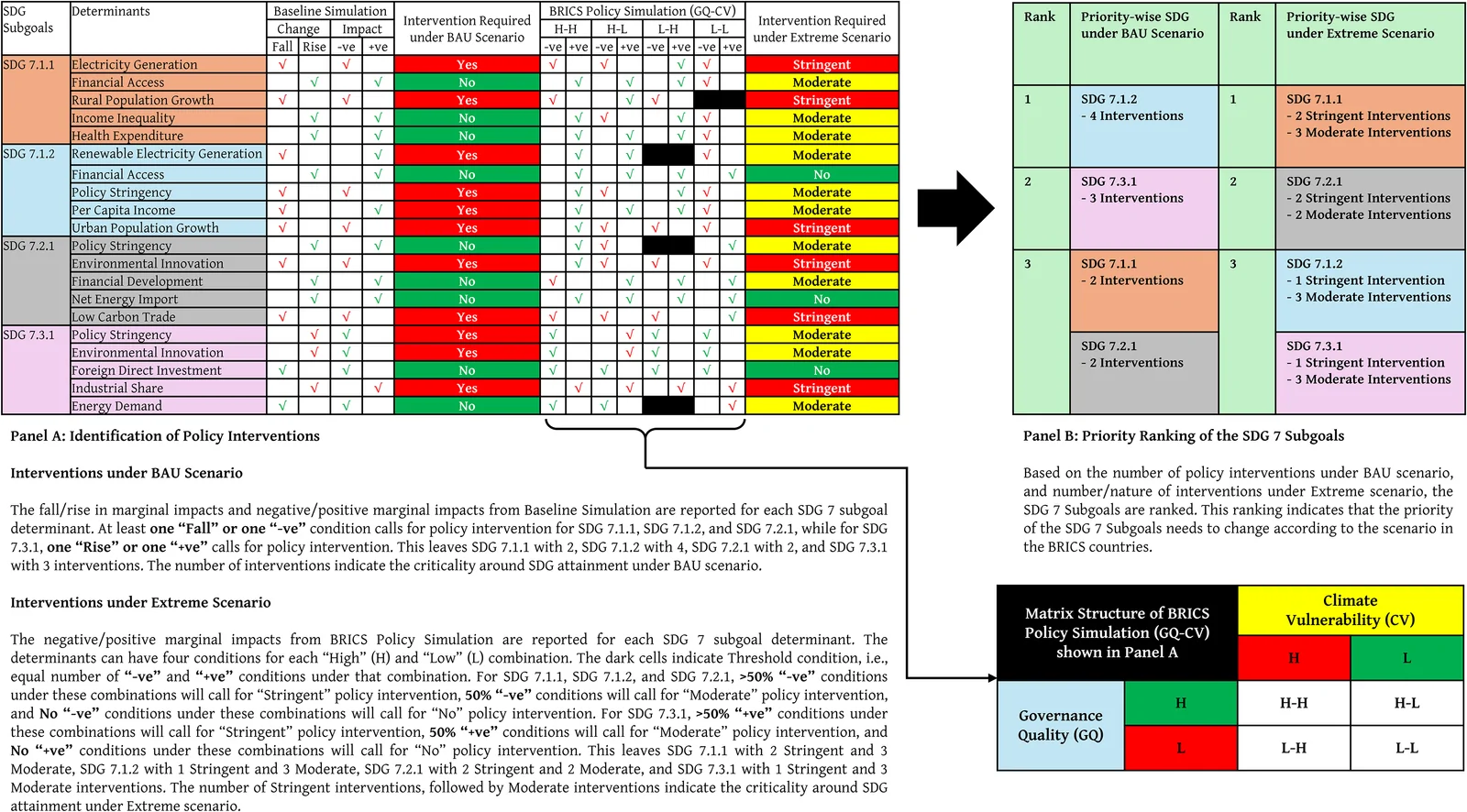
Prioritizing the Target SDG 7 Subgoals through Identification of the Policy Intervention points under BAU and extreme settings See Figures S8–S107.

The scenario analysis builds directly upon the estimated marginal-effect surface. Rather than re-estimating the empirical model, it evaluates the fitted conditional relationships under stylized governance quality and climate vulnerability settings to facilitate policy interpretation. Consequently, the scenario analysis should be interpreted as a post-estimation evaluation framework that summarizes how policy effectiveness changes across contrasting institutional environments.

The extreme evaluation framework classifies relative intervention priorities using four discrete governance quality and climate vulnerability regimes (HH, HL, LH, and LL). Since only four regime combinations exist, intervention intensity is determined directly from the number of unfavorable outcomes across these regimes. Determinants exhibiting unfavorable outcomes in more than half of the regimes (>50%) are classified as requiring greater intervention, those with an equal split between favorable and unfavorable outcomes (50%) are classified as requiring moderate intervention, and those with no unfavorable outcomes (0%) are classified as requiring lower intervention. For SDG 7.3.1, where higher energy intensity represents poorer performance, the intervention classification is based on the corresponding distribution of favorable outcomes.

Based on the proposed evaluation framework, SDG 7.1.2 exhibits the highest number of identified intervention points under the BAU setting, whereas SDG 7.1.1 and SDG 7.2.1 exhibit the fewest. Under the extreme setting, SDG 7.1.1 exhibits the highest number of identified intervention points, whereas SDG 7.1.2 and SDG 7.3.1 exhibit comparatively fewer. These comparisons may help policymakers prioritize SDG attainment objectives under alternative governance quality and climate vulnerability conditions.

### Country-wise simulation results show different policy intervention points for tailored policy design

The country-level analysis reveals distinct policy priorities across the BRICS economies. While all countries require improvements in governance quality and reductions in climate vulnerability, the simulations indicate that the relative importance of individual SDG 7 subgoals differs substantially across countries. Brazil and South Africa require stronger interventions to accelerate renewable energy deployment and energy efficiency, whereas India and China benefit more from policies targeting energy access alongside institutional strengthening. Russia exhibits comparatively lower intervention requirements under several governance and climate vulnerability scenarios, although continued improvements in energy efficiency remain important. SDG 7 subgoal determinants for individual BRICS member countries might exhibit different behavior than the group behavior. The marginal impacts of SDG 7 subgoal determinants for the individual BRICS member countries are computed (Tables S47–S62) and reported in Figures 6 and 7. Unlike the extreme evaluation framework, Figures 6 and 7 summarize the fitted marginal-effect surface over the complete climate vulnerability-governance quality evaluation grid. Figure 5 summarizes the directional sign of the estimated marginal effects as dominant positive, mixed, or dominant negative responses, whereas Figure 6 summarizes their directional response as dominant increasing, mixed, or dominant decreasing responses. Because these analyses evaluate the fitted surface over a continuous moderator space rather than four discrete regimes, directional dominance is summarized using heuristic dominance classes. A dominant response is identified when the same directional pattern is observed over more than 65% (approximately two-thirds) of the evaluation grid, a mixed response when it is observed over 35–65% of the grid, and the opposite dominant response when it is observed over less than 35% (approximately one-third) of the grid.

**Figure 6:**
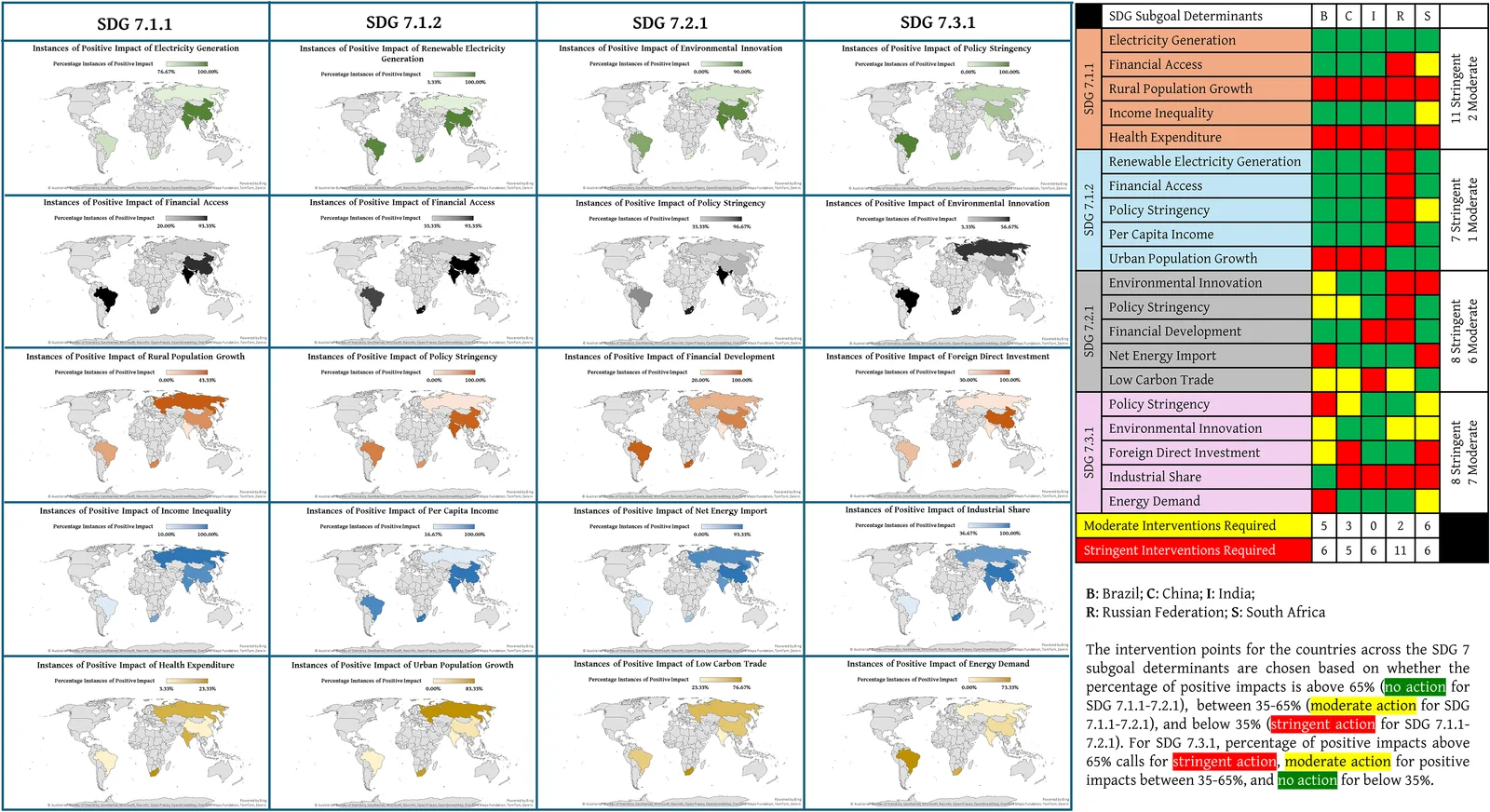
Country-wise Instances of Intervention Priority Analysis of SDG 7 subgoal determinants in the presence of Governance Quality and Climate Vulnerability See Tables S47, S48, S51, S52, S55, S56, S59, and S60.

**Figure 7:**
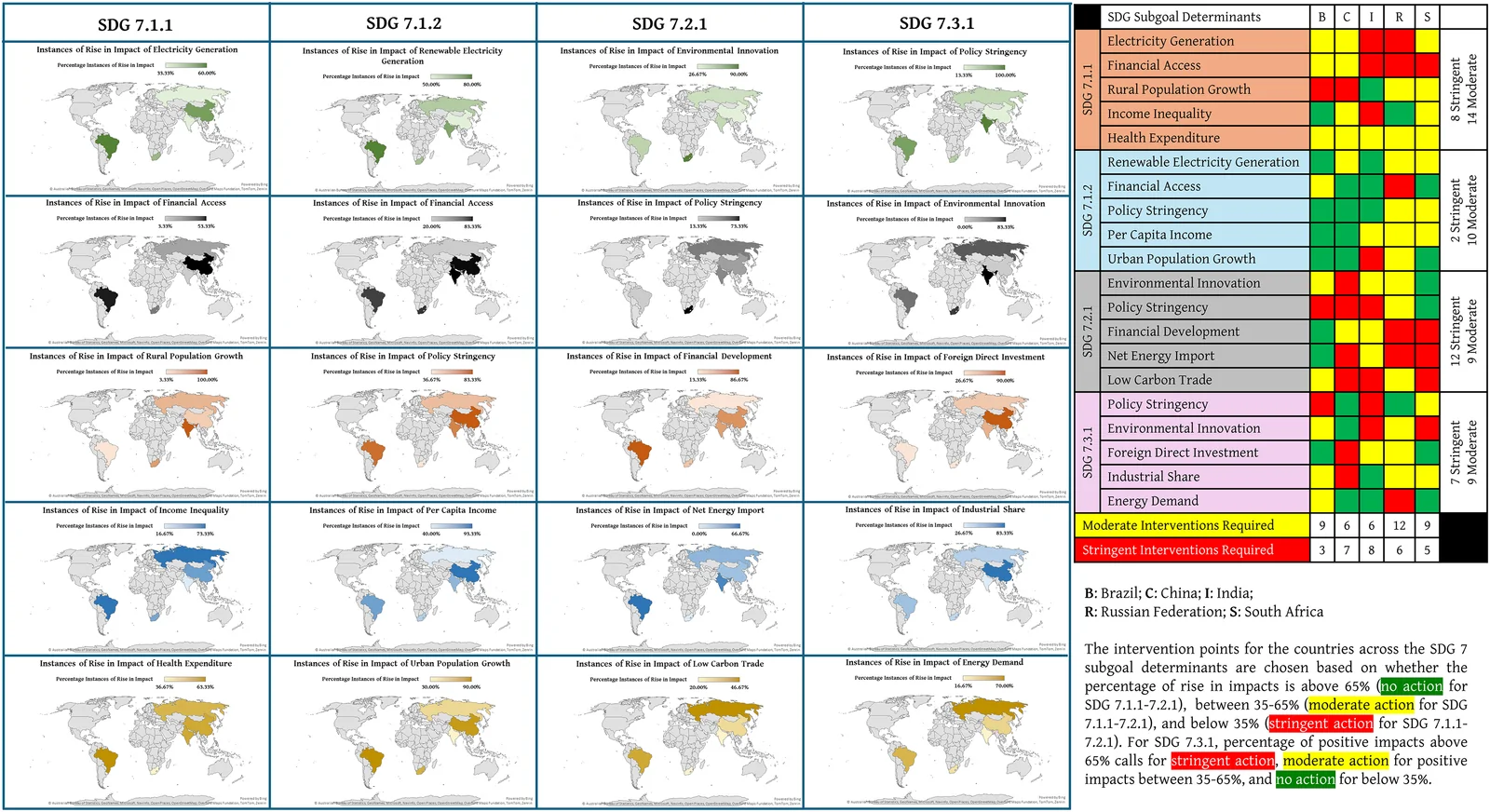
Country-wise directional dominance analysis of SDG 7 subgoal determinants in the presence of governance quality and climate vulnerability See Tables S49, S50, S53, S54, S57, S58, S61, and S62.

Based on the proposed evaluation criteria, Russia exhibits the largest number of identified intervention points, whereas India exhibits the fewest. Under the proposed evaluation framework, SDG 7.1.1 records the largest number of identified intervention points across countries, followed by SDG 7.3.1, SDG 7.2.1, and SDG 7.1.2. The country-level interventions are summarized in Table 3.

**Table 3:** Summary of country-specific SDG 7 priorities and policy implications in BRICS

Country	Priority SDG 7 subgoal(s)	Principal policy intervention areas	Policy focus
**Brazil**	SDG 7.2.1 and SDG 7.3.1	climate-resilient renewable energy infrastructure, energy efficiency, intergovernmental coordination	strengthening governance coordination and climate resilience can help sustain renewable energy deployment while improving energy efficiency across regions.
**Russia**	SDG 7.3.1	energy efficiency, regulatory transparency, diversification of the energy system	reducing dependence on fossil fuels while strengthening institutional quality is essential to improve long-term energy efficiency and accelerate the clean energy transition.
**India**	SDG 7.1.1, SDG 7.1.2 and SDG 7.2.1	renewable energy deployment, resilient electricity distribution, clean cooking access, institutional capacity	continued progress in energy access should be complemented by governance strengthening and climate-resilient infrastructure, particularly in vulnerable regions.
**China**	SDG 7.2.1 and SDG 7.3.1	renewable energy innovation, governance quality, energy efficiency	maintaining technological leadership while strengthening governance and climate resilience can sustain improvements in renewable energy deployment and energy efficiency.
**South Africa**	SDG 7.1.2, SDG 7.2.1 and SDG 7.3.1	electricity-sector reform, renewable energy investment, grid modernization, institutional capacity	accelerating clean energy deployment requires coordinated improvements in electricity-sector governance, investment certainty, and climate-resilient infrastructure.

Based on the percentage increase in estimated marginal impacts, Russia records the largest number of identified intervention points, whereas Brazil records the fewest. SDG 7.1.1 requires the most cross-country interventions, followed by SDG 7.2.1, 7.3.1, and 7.1.2.

## Discussion

Several estimated relationships differ from previous empirical evidence. In particular, the counterintuitive conditional effects associated with environmental policy stringency and environmental innovation should not be interpreted as evidence that these policy instruments are ineffective. Rather, they indicate that their estimated marginal effects depend on the interaction between governance quality and climate vulnerability, implying that institutional capacity and climatic conditions influence whether regulatory and innovation policies translate into improved SDG 7 outcomes. Previous evidence, likewise, indicates that the effectiveness of environmental policy stringency is heterogeneous rather than universal, varying across countries, environmental indicators, and different parts of the outcome distribution.^57^ Our results extend this perspective by showing that policy effectiveness also varies systematically with governance quality and climate vulnerability. These differences are interpreted as conditional associations arising from the interaction between climate vulnerability and governance quality rather than as unconditional relationships. These associations should not be interpreted as evidence of a direct causal pathway from governance quality to SDG 7 performance. Rather, governance quality captures institutional conditions under which the estimated effects of SDG 7 determinants vary, while the underlying mechanisms may operate through regulatory capacity, implementation quality, coordination, and investment conditions. The robustness analysis using panel-corrected standard errors (PCSE) indicates that the principal coefficient signs remain qualitatively stable across estimation approaches (Tables S63–S102). Therefore, where the estimated signs differ from previous studies, the discussion offers plausible contextual interpretations without implying universal mechanisms. More broadly, the findings support a context-dependent perspective of energy transition policy in which governance quality and climate vulnerability jointly determine the effectiveness of conventional SDG 7 policy instruments. These conditional patterns are consistent with policy-effectiveness channels through which governance quality can influence regulatory implementation, institutional coordination, investment certainty, and the capacity to translate policy instruments into energy-system outcomes. In this sense, the estimated relationships connect the energy-policy effectiveness literature with the broader institutional perspective that policy outcomes depend not only on the design of policy instruments but also on the institutional conditions under which they are implemented. This perspective extends the existing literature by emphasizing that institutional context influences not only energy-transition outcomes but also the marginal effectiveness of the policies intended to achieve them.

We find that, in addition to conventional drivers, e.g., policy stringency, financial development, innovation, and socioeconomic structure, the estimated relationships between these drivers and SDG 7 outcomes vary systematically with climate vulnerability and governance quality. The interaction terms indicate that the estimated marginal effects of the key energy-transition drivers differ across governance quality and climate vulnerability conditions. The study provides evidence that the effectiveness of policy levers may vary across institutional and climatic settings.^8^^,^^9^^,^^10^^,^^11^^,^^12^^,^^13^^,^^14^^,^^15^^,^^16^^,^^58^ In contrast, our findings indicate that the same intervention can have a positive, negative, and even counterproductive effect depending on the institutional context and climatic stress. These findings emphasize the importance of viewing policy effectiveness as context-dependent: policy tools are not always productive, but they are dependent on the nature of the governance-vulnerability regime in which they are applied.

Across BRICS economies, the static marginal impact analysis reveals two broad patterns of determinants. The first pattern—financial access, income inequality reduction, health expenditure, policy stringency, and environmental innovation—tends to improve SDG 7 outcomes under favorable governance and resilience conditions. These drivers exhibit stronger estimated marginal effects under environments characterized by predictable regulation, credible enforcement, and the capacity to plan and invest proactively. The second pattern—electricity generation, rural/urban population dynamics, energy demand and per capita income—performs less favorably under moderation, often producing negative marginal effects when climate vulnerability or governance weaknesses intensify. A possible explanation is that these drivers are more closely linked to the physical robustness of infrastructure, the socio-spatial design of energy systems, and the behavioral responses of households and firms to climatic shocks. The contrasting behavior across clusters underscores the importance of pairing technical or economic interventions with improvements in institutional quality and climate preparedness, especially in settings where governance or vulnerability constraints amplify implementation risks.

The scenario analysis further illustrates how different governance quality and climate vulnerability settings can lead to very different governance regimes. In the context of high vulnerability, low governance conditions, which are common in several fast-urbanizing regions in BRICS countries, their estimated marginal effects weaken substantially; environmental innovation can be fragile, and gains in energy access can stall. On the other hand, high governance, high resilience regimes are associated with stronger estimated marginal effects of the productivity of innovation, renewable generation, and industrial transition, indicating a potential for non-linear benefits for SDG 7 progress through coordinated governance and resilience improvements. These findings suggest that the energy transition is not just about technology uptake or investment in the system, but also the political-institutional context in which policies are interpreted and realized in the system.

One important policy implication of the estimated associations that has come to light from identifying intervention points is the need for differentiated governance priorities for SDG subgoals. Business-*as*-usual intervention scenarios have the greatest number of interventions for SDG 7.1.2 (clean energy access) and the most vulnerable number of interventions for SDG 7.1.1 (electricity access) under extreme conditions. This comparison suggests that access-related objectives are at risk of being directly affected by climatic extremes and institutional shortcomings, even before they affect efficiency or renewable-energy objectives. In countries such as India and South Africa, where a significant proportion of the population is already covered by electricity, the protection of SDG 7.1.1 may require targeted resilience building of distribution networks and peri-urban extension corridors that are climate-sensitive. For Brazil and China, on the other hand, the primary constraint could be the ability to maintain clean energy access gains and energy intensity gains amidst increasing climate risk.

The country-level simulation results indicate differentiated policy priorities across the BRICS economies, highlighting the need for transition strategies tailored to country-specific governance and climate conditions. Findings suggest that Brazil may benefit from strengthening climate-resilient renewable energy infrastructure and improving intergovernmental coordination to reduce regional disparities in energy access and clean energy deployment. The results indicate that greater diversification away from fossil fuel dependence may be beneficial for Russia, while improving governance transparency and regulatory certainty to facilitate investment in low-carbon energy systems. The findings suggest that strengthening institutional capacity may be particularly relevant for India for renewable energy deployment, climate-resilient electricity distribution networks, and access to clean cooking fuels, particularly in climate-vulnerable regions. China should continue integrating technological innovation with governance improvements to sustain renewable-energy expansion while addressing increasing regional climate risks. South Africa should prioritize electricity-sector reforms, investment certainty, and climate-resilient grid modernization to improve energy security and accelerate progress toward SDG 7. These differences demonstrate that the governance quality and climate vulnerability context vary substantially across BRICS economies, implying that SDG 7 strategies should be tailored to country-specific institutional and climatic conditions rather than relying on uniform regional policy approaches.

Second, the study highlights that energy access (SDG 7.1) and clean energy transitions (SDG 7.2, 7.3) are not equally sensitive to climate and institutional shocks. Our results show that access-related subgoals, particularly SDG 7.1.1, are far more vulnerable to climatic extremes, whereas efficiency and renewable penetration targets remain comparatively resilient. This differentiated sensitivity implies that the new SDG 7 framework should no longer have a single global indicator, but rather indicators with different thresholds that adjust the expectations of countries based on their exposure to climate risk. This differentiation would better reflect the SDG architecture's principles of climate justice and adaptive capacity.

Third, the country-level analysis reveals that the number and nature of interventions to remain on course for each subgoal differ significantly not only across the BRICS, but also within economies since their governance-vulnerability regime. This means that a “one size fits all” approach to SDG 7 targets is becoming less viable. Countries should reach the next SDG implementation cycle with a revised SDG 7 goal, which incorporates context-sensitive performance pathways that enable countries to work through a series of milestones that are structured based on their starting conditions, institutional maturity, and exposure to climate.

The findings of this study offer timely evidence for the forthcoming global revision of SDG 7 targets by demonstrating that the current subgoals, i.e., focused on energy access, clean cooking, renewable energy share, and energy efficiency, do not fully account for the structural realities shaping energy transitions in climate-vulnerable, institutionally diverse economies. Our results suggest that the estimated relationships underlying SDG 7 progress are shaped not only by technology adoption and investment, but also by governance quality and climate vulnerability. This suggests that the next generation of SDG 7 indicators must integrate dimensions of governance robustness, climate resilience, and system adaptability, especially for countries exposed to escalating climate hazards. This is particularly relevant as the UN considers aligning SDG targets with the Global Stocktake and just transition commitments. Our findings reinforce the need for SDG 7 to reflect not only the scale of energy deficits but the structural barriers that determine whether transitions are resilient, equitable, and attainable. In doing so, the study contributes concrete evidence supporting a more flexible, justice-oriented, and climate-resilient architecture for the post-2025 SDG revision.

In conclusion, the study provides evidence that achieving SDG 7 in large emerging economies cannot be separated from the realities of climate vulnerability and governance quality. The findings suggest that effective transition policy is likely to require not only deploying technological, financial, and regulatory tools but also understanding how their impacts shift under different institutional and climatic conditions. Recognizing this conditionality may help policymakers design interventions that are robust, context-sensitive, and ultimately capable of delivering affordable, reliable, and clean energy in an era of rising climate risk. Although derived from the BRICS economies, the findings may provide useful insights for other developing regions facing comparable governance and climate-related challenges by supporting more targeted, resilience-aware, and governance-informed energy strategies.

### Limitations of the study

At the same time, the study’s limitations provide a foundation for future research. First, the statistical moderation framework captures only the directional sensitivity of impacts and cannot fully represent or include deeper structural mechanisms, viz., cross-sectoral feedback. Second, while the extreme-scenario combination simulations offer useful insights into regime shifts, they rely on stylized percentile thresholds rather than dynamic pathways of institutional or climatic change. Third, the analysis is confined to the BRICS economies, whose governance structures, institutional capacities, energy-market characteristics, and climate vulnerability profiles may differ from those of other developing regions. Cross-country differences may also reflect unobserved institutional characteristics that are not fully captured by the governance indicators included in the analysis, such as differences in administrative capacity, policy implementation practices, or institutional coordination. Consequently, the empirical findings should not be interpreted as universally applicable beyond the study sample, and their external validity requires further evaluation using broader cross-country datasets. Future research should extend the proposed framework to additional developing economies, including low-income countries and Small Island Developing States, to examine whether similar governance-vulnerability interactions emerge under different institutional and climatic settings.

Although the empirical analysis focuses on the BRICS economies, the proposed analytical framework may offer useful insights for other emerging and developing economies facing comparable governance and climate-related challenges. However, the estimated relationships should not be interpreted as directly generalizable beyond the study sample because institutional capacity, political systems, energy-market structures, and climate vulnerability profiles differ substantially across countries. Consequently, the external validity of the empirical findings should be interpreted with appropriate caution. Nevertheless, the conceptual framework developed in this study may provide a useful basis for future empirical investigations of SDG 7 implementation in other developing-country contexts. Whether similar governance-vulnerability interactions emerge in those settings remains an empirical question requiring further validation. Future research should extend the analysis to broader country samples, including low-income economies, Small Island Developing States, and the newly expanded BRICS members, to evaluate the robustness and external validity of the proposed framework under diverse institutional and climatic conditions.

## Resource availability

### Lead contact

Requests for further information and resources should be directed to and will be fulfilled by the lead contact, Avik Sinha (f11aviks@iimidr.ac.in).

### Materials availability

This study did not generate new materials.

### Data and code availability

• All relevant data supporting the findings of this article are included in the article or can be obtained from the lead contact upon request.
• This paper does not report original code.
• Any additional information required to reanalyze the data reported in this paper is available from the lead contact upon request.

## Acknowledgments

This work is supported by the 10.13039/501100006769Russian Science Foundation under grant (RSF: 25-28-03491).

## Author contributions

Conceptualization, A.S. and A.J.; data curation, A.S.; formal analysis: A.S. and A.J.; funding acquisition, S.A.; investigation, A.S. and A.J.; methodology, A.S.; project administration, A.S.; resources, A.S. and A.J.; software, A.S.; supervision, A.S.; validation, A.S. and A.J.; visualization, A.S. and A.J.; writing – original draft, A.S. and A.J.; writing – review and editing, A.S., A.J., and S.A.

## Declaration of interests

The authors declare no competing interests.

## Declaration of generative AI and AI-assisted technologies in the writing process

During the preparation of this work, the author used ChatGPT Go to improve readability of the manuscript, structure the arguments, and maintain coherence. After using this tool, the author reviewed and edited the content as needed and takes full responsibility for the content of the publication.

## STAR★Methods

### Key resources table

**Table:** 

REAGENT or RESOURCE	SOURCE	IDENTIFIER
**Deposited data**		

SDG 7.1.1	Sustainable Development Report 2023	https://www.sustainabledevelopment.report/reports/sustainable-development-report-2023/
SDG 7.1.2		
SDG 7.2.1		
SDG 7.3.1	International Energy Agency	https://www.iea.org/data-and-statistics/data-product/sdg7-database#energy-intensity
Electricity Generation	United Nations Statistics Division	https://data.un.org/Data.aspx?d=EDATA&f=cmID%3AEL
	World Development Indicators	https://data.worldbank.org/indicator
Financial Access	World Bank	https://www.worldbank.org/en/publication/gfdr/data/global-financial-development-database
Rural Population Growth	World Development Indicators	https://data.worldbank.org/indicator
Income Inequality	World Inequality Database	https://wid.world/
Health Expenditure	World Development Indicators	https://data.worldbank.org/indicator
Renewable Electricity Generation	International Energy Agency	https://www.iea.org/data-and-statistics/data-tools/energy-statistics-data-browser?country=WORLD&fuel=Renewables%20and%20waste&indicator=RenewGenBySource
Policy Stringency	OECD Statistics	https://stats.oecd.org/
Per Capita Income	World Development Indicators	https://data.worldbank.org/indicator
Urban Population Growth	World Development Indicators	https://data.worldbank.org/indicator
Environmental Innovation	OECD Statistics	https://stats.oecd.org/
Financial Development	World Bank	https://www.worldbank.org/en/publication/gfdr/data/global-financial-development-database
Net Energy Import	United Nations Statistics Division	https://data.un.org/Data.aspx?d=EDATA&f=cmID%3AEL
Low Carbon Trade	International Monetary Fund	https://climatedata.imf.org/datasets/1d33174e9e46429d9e570d539556f66a_0/about?deliveryName=DM15392
Foreign Direct Investment	World Development Indicators	https://data.worldbank.org/indicator
Industrial Share	World Development Indicators	https://data.worldbank.org/indicator
Energy Demand	Ember	https://ember-energy.org/data/electricity-data-explorer/
Control of Corruption	Worldwide Governance Indicators	https://databank.worldbank.org/source/worldwide-governance-indicators
Governance Effectiveness		
Political Stability		
Regulatory Quality		
Rule of Law		
Voice and Accountability		
Ecosystem Vulnerability	Notre Dame Global Adaptation Initiative	https://gain.nd.edu/our-work/country-index/
Food Vulnerability		
Health Vulnerability		
Infrastructure Vulnerability		
Water Vulnerability		

**Software and algorithms**		

Stata 17	StataCorp LLC	https://www.stata.com
MATLAB 2023a	Mathworks	https://in.mathworks.com/products/new_products/release2023a.html
Microsoft Excel	Microsoft	https://www.microsoft.com/en-in/microsoft-365/excel

### Method details

#### Generalized least square

We employed Generalized Least Squares (GLS) to estimate the long-run coefficients. GLS extends the linear model **y** = **Xβ**+ε to settings in which the Gauss-Markov assumption of spherical disturbances is violated. While Ordinary Least Squares (OLS) assumes Var⁡(ε∣**X**) = σ^2^**I**, GLS replaces this with the more general structure Var⁡(ε∣**X**) = Ω, where Ω may be heteroskedastic, autocorrelated, or otherwise non-scalar. When errors exhibit such non-spherical properties, OLS no longer minimizes the variance of the estimator; GLS corrects this by transforming the model via a non-singular matrix **P**, such that **P**^⊤^**ΩP** = **I**. The transformed model becomes the following:

(Equation 1) Py=PXβ+Pε,withVar(Pε)=I

allowing OLS to be applied to the transformed variables, yielding the following:

(Equation 2) $${\hat{\beta }}_{\text{GLS}}={\left(X^{⊤}\Omega^{-1}X\right)}^{-1}X^{⊤}\Omega^{-1}y$$

Study shows is the Best Linear Unbiased Estimator (BLUE) under known Ω.^59^

GLS has certain advantages over other econometric models. First, let us take the case of heteroskedasticity, where Ω = σ^2^**D** for diagonal **D** ≠ **I**. Under OLS, the sampling variance becomes the following:

(Equation 3) $$\text{Var}\left({\hat{\beta }}_{\text{OLS}}\right)={\left(X^{⊤}X\right)}^{-1}X^{⊤}\Omega X{\left(X^{⊤}X\right)}^{-1}$$

This is large due to inefficient weight assignments to observations. On the contrary, GLS weighs each observation by the inverse of its error variance, allocating more information to precise observations, producing reduced estimator variance and valid standard errors. This efficiency recovery is beneficial in panel models with autoregressive disturbances. Moreover, iterated FGLS approaches converge to the same asymptotic efficiency as the infeasible GLS estimator but with better small-sample performance.^60^

Second, GLS provides efficiency gains under cross-equation dependence. In a Seemingly Unrelated Regressions (SUR) system,^61^ covariance across the estimation equations implies following:

(Equation 4) $$\Omega =\left[\begin{array}{ccc} \Sigma_{11} & \cdots & \Sigma_{1M}\\ \vdots & \ddots & \vdots \\ \Sigma_{M1} & \cdots & \Sigma_{\text{MM}} \end{array}\right]$$

where Σ_ij_ ≠ 0 for i ≠ j. Separate OLS regressions ignore Σ_ij_, while GLS exploits it, reducing estimator variance. Efficiency gains are proven to be strictly positive whenever regressors differ across equations and cross-equation correlations exist.^61^

Third, GLS is advantageous in econometric models with correlation manifested across units. Let’s say, the autoregressive disturbances are described as follows:

(Equation 5) $$\varepsilon =\lambda W\varepsilon +u,\text{Var}\left(u\right)=\sigma^{2}I$$

This disturbance induces covariance of $\Omega ={\left(I-\lambda W\right)}^{-1}{\left(I-\lambda W^{⊤}\right)}^{-1}\sigma^{2}$. GLS-type estimator incorporating both cross-sectional dependence and autoregressive errors yield consistent and efficient estimates, where OLS would be inefficient and potentially misleading.^62^

It is essential to distinguish these efficiency improvements from the separate issue of endogeneity. Endogeneity implies E[**X**^⊤^ε] ≠ 0, producing biased and inconsistent estimators. GLS does not alter the conditional expectation of the disturbance term and therefore cannot remove endogeneity bias. Valid causal identification requires instrumental variables (IV) or comparable strategies to restore exogeneity.^63^ Once valid instruments are in place, GLS weighing can be combined with IV addressing disturbance correlation and endogeneity simultaneously.^62^ Thus, GLS helps in maximizing efficiency conditional on proper identification but not replacing the need for instruments when regressors are endogenous.

Lastly, the data specification needed the application of GLS. GLS is employed to improve efficiency when the error structure exhibits heteroskedasticity and/or cross-correlation across panels, conditions under which OLS remains unbiased but inefficient. GLS achieves this by weighing the regression using the inverse of the disturbance covariance matrix Ω, yielding the estimator ${\hat{\beta }}_{GLS}={\left(X^{⊤}\Omega^{-1}X\right)}^{-1}X^{⊤}\Omega^{-1}y$, which is BLUE when Ω is correctly specified. In panel data, however, Ω is of dimension NT×NT, and estimating it unrestrictedly can become infeasible in finite samples due to the large number of covariance parameters. Therefore, practical suitability of GLS depends on whether the sample provides sufficient information to produce a stable and invertible estimate of Ω. In our dataset, the cross-sectional dimension (*N* = 5) is moderate relative to the temporal dimension (T = 23), which supports reliable structured covariance estimation. This justification is consistent with evidence that small-sample (<200 observations) GLS can yield underestimated standard errors,^64^ and that imposing structure improves feasibility and asymptotic validity in panels with limited observations.^65^ Lastly, barring a few cases, reverse casuality is mostly absent in the data (Table S103). Accordingly, GLS is appropriate in our setting.

### Quantification and statistical analysis

We use the following models to estimate the baseline behavior of the SDG 7 subgoals:

(Equation 6) $$Y=\alpha X+\alpha_{v}CV+\alpha_{q}\text{QG}$$

Here, **Y** is the matrix of outcome variables, i.e., the SDG 7 subgoals, **X** is the matrix of the subgoal determinants, **CV** is the matrix of climate vulnerability indicators, **QG** is the matrix of quality of governance indicators, *v* is the number of climate vulnerability indicators, and *q* is the number of governance quality indicators.

First, electricity access and clean cooking adoption depend primarily on households’ economic capacity, affordability, demographic characteristics, and public service provision. Consequently, variables such as income inequality, financial access, health expenditure, per capita income, and population distribution are included because they influence households’ ability to obtain and sustain access to modern energy services. Existing studies show that improvements in financial inclusion and income levels facilitate investments in electricity connections and clean cooking technologies, whereas higher inequality constrains access among vulnerable populations. Second, Renewable energy deployment is determined by policy incentives, technological capability, financing conditions, and integration into low carbon production networks. Environmental policy stringency creates regulatory incentives for renewable investment, environmental innovation facilitates the development and diffusion of clean technologies, financial development improves access to investment capital, while low carbon trade accelerates technology transfer and market expansion. Third, Improvements in energy efficiency arise through technological upgrading, structural transformation, and investment in productive capacity. Industrial structure, foreign direct investment, environmental innovation, and environmental policy stringency therefore capture the principal channels through which economies reduce the amount of energy required to generate economic output.

Following these mechanisms, for and *i* and *t* being the BRICS countries and study period, the empirically testable version of Equation 6 for v = {1,⋯,5} and q = {1,⋯,6} is as follows:

(Equation 7) $${Y1}_{\text{it}}=\alpha_{11}{\text{EG}}_{\text{it}}+\alpha_{12}{\text{FA}}_{\text{it}}+\alpha_{13}{\text{RP}}_{\text{it}}+\alpha_{14}{\text{INQ}}_{\text{it}}+\alpha_{15}{\text{HE}}_{\text{it}}+\alpha_{v}{\text{CV}}_{v,\text{it}}+\alpha_{q}{\text{QG}}_{q,\text{it}}$$

(Equation 8) $${Y2}_{\text{it}}=\alpha_{21}{\text{RE}}_{\text{it}}+\alpha_{22}{\text{FA}}_{\text{it}}+\alpha_{23}{\text{PS}}_{\text{it}}+\alpha_{24}{\text{PCI}}_{\text{it}}+\alpha_{25}{\text{UP}}_{\text{it}}+\alpha_{v}{\text{CV}}_{v,\text{it}}+\alpha_{q}{\text{QG}}_{q,\text{it}}$$

(Equation 9) $${Y3}_{\text{it}}=\alpha_{31}{\text{PS}}_{\text{it}}+\alpha_{32}{\text{INN}}_{\text{it}}+\alpha_{33}{\text{FD}}_{\text{it}}+\alpha_{34}{\text{NEI}}_{\text{it}}+\alpha_{35}{\text{LT}}_{\text{it}}+\alpha_{v}{\text{CV}}_{v,\text{it}}+\alpha_{q}{\text{QG}}_{q,\text{it}}$$

(Equation 10) $${Y4}_{\text{it}}=\alpha_{41}{\text{PS}}_{\text{it}}+\alpha_{42}{\text{INN}}_{\text{it}}+\alpha_{43}{\text{FDI}}_{\text{it}}+\alpha_{44}{\text{IS}}_{\text{it}}+\alpha_{45}{\text{ED}}_{\text{it}}+\alpha_{v}{\text{CV}}_{v,\text{it}}+\alpha_{q}{\text{QG}}_{q,\text{it}}$$

Here, **Y** = {Y1, Y2,Y3,Y4} are SDG 7.1.1, SDG 7.1.2, SDG 7.2.1, SDG 7.3.1, EG is electricity generation, FA is financial access, RP is rural population growth, HE is out-of-pocket health expenditure, RE is renewable electricity generation, PS in environmental policy stringency, PCI is per capita income, UP is urban population growth, INN is environmental innovation, FD is financial development, NEI is net energy import, LT is low carbon trade, IS is industrial share in gross domestic product, and ED is energy demand. The variable descriptions and respective data sources are mentioned in Table S1. Their descriptive statistics, trend, and initial model diagnostics are reported in Tables S2–S6; Figures S1–S7. The five climate vulnerability indicators were harmonized across the BRICS countries by matching the indicators to the same country-year observations over the study period. The indicators were retained as separate dimensions of climate vulnerability rather than aggregated into a composite measure, allowing each dimension to enter the moderated estimation independently.

The way climate vulnerability and governance quality change the behavior of **X** is captured by allowing their moderation in Equation 6 as the following:

(Equation 11) $$Y=X*\left(\beta +\beta_{v}CV+\beta_{q}\text{QG}+\beta_{q+v}\text{CV}*\text{QG}\right)$$

The empirically testable version of Equation 11 for v = {1,⋯,5} and q = {1,⋯,6} is as follows:

(Equation 12) $${Y1}_{\text{it}}=\beta_{11}{\text{EG}}_{\text{it}}+\beta_{12}{\text{FA}}_{\text{it}}+\alpha_{13}{\text{RP}}_{\text{it}}+\alpha_{14}{\text{INQ}}_{\text{it}}+\alpha_{15}{\text{HE}}_{\text{it}}+\alpha_{v}{\text{CV}}_{v,\text{it}}+\alpha_{q}{\text{QG}}_{q,\text{it}}+\alpha_{v}{\text{CV}}_{v,\text{it}}*\left({\text{EG}}_{\text{it}}+{\text{FA}}_{\text{it}}+{\text{RP}}_{\text{it}}+{\text{INQ}}_{\text{it}}+{\text{HE}}_{\text{it}}\right)+\alpha_{q}{\text{QG}}_{q,\text{it}}*\left({\text{EG}}_{\text{it}}+{\text{FA}}_{\text{it}}+{\text{RP}}_{\text{it}}+{\text{INQ}}_{\text{it}}+{\text{HE}}_{\text{it}}\right)+\alpha_{v+q}{\text{CV}}_{v,\text{it}}*{\text{QG}}_{q,\text{it}}*\left({\text{EG}}_{\text{it}}+{\text{FA}}_{\text{it}}+{\text{RP}}_{\text{it}}+{\text{INQ}}_{\text{it}}+{\text{HE}}_{\text{it}}\right)$$

(Equation 13) $${Y2}_{\text{it}}=\alpha_{21}{\text{RE}}_{\text{it}}+\alpha_{22}{\text{FA}}_{\text{it}}+\alpha_{23}{\text{PS}}_{\text{it}}+\alpha_{24}{\text{PCI}}_{\text{it}}+\alpha_{25}{\text{UP}}_{\text{it}}+\alpha_{v}{\text{CV}}_{v,\text{it}}+\alpha_{q}{\text{QG}}_{q,\text{it}}+\alpha_{v}{\text{CV}}_{v,\text{it}}*\left({\text{RE}}_{\text{it}}+{\text{FA}}_{\text{it}}+{\text{PS}}_{\text{it}}+{\text{PCI}}_{\text{it}}+{\text{UP}}_{\text{it}}\right)+\alpha_{q}{\text{QG}}_{q,\text{it}}*\left({\text{RE}}_{\text{it}}+{\text{FA}}_{\text{it}}+{\text{PS}}_{\text{it}}+{\text{PCI}}_{\text{it}}+{\text{UP}}_{\text{it}}\right)+\alpha_{v+q}{\text{CV}}_{v,\text{it}}*{\text{QG}}_{q,\text{it}}*\left({\text{RE}}_{\text{it}}+{\text{FA}}_{\text{it}}+{\text{PS}}_{\text{it}}+{\text{PCI}}_{\text{it}}+{\text{UP}}_{\text{it}}\right)$$

(Equation 14) $${Y3}_{\text{it}}=\alpha_{31}{\text{PS}}_{\text{it}}+\alpha_{32}{\text{INN}}_{\text{it}}+\alpha_{33}{\text{FD}}_{\text{it}}+\alpha_{34}{\text{NEI}}_{\text{it}}+\alpha_{35}{\text{LT}}_{\text{it}}+\alpha_{v}{\text{CV}}_{v,\text{it}}+\alpha_{q}{\text{QG}}_{q,\text{it}}+\alpha_{v}{\text{CV}}_{v,\text{it}}*\left({\text{PS}}_{\text{it}}+{\text{INN}}_{\text{it}}+{\text{FD}}_{\text{it}}+{\text{NEI}}_{\text{it}}+{\text{LT}}_{\text{it}}\right)+\alpha_{q}{\text{QG}}_{q,\text{it}}*\left({\text{PS}}_{\text{it}}+{\text{INN}}_{\text{it}}+{\text{FD}}_{\text{it}}+{\text{NEI}}_{\text{it}}+{\text{LT}}_{\text{it}}\right)+\alpha_{v+q}{\text{CV}}_{v,\text{it}}*{\text{QG}}_{q,\text{it}}*\left({\text{PS}}_{\text{it}}+{\text{INN}}_{\text{it}}+{\text{FD}}_{\text{it}}+{\text{NEI}}_{\text{it}}+{\text{LT}}_{\text{it}}\right)$$

(Equation 15) $${Y4}_{\text{it}}=\beta_{41}{\text{PS}}_{\text{it}}+\beta_{42}{\text{INN}}_{\text{it}}+\beta_{43}{\text{FDI}}_{\text{it}}+\beta_{44}{\text{IS}}_{\text{it}}+\beta_{45}{\text{ED}}_{\text{it}}+\beta_{v}{\text{CV}}_{v,\text{it}}+\beta_{q}{\text{QG}}_{q,\text{it}}+\beta_{v}{\text{CV}}_{v,\text{it}}*\left({\text{PS}}_{\text{it}}+{\text{INN}}_{\text{it}}+{\text{FDI}}_{\text{it}}+{\text{IS}}_{\text{it}}+{\text{ED}}_{\text{it}}\right)+\beta_{q}{\text{QG}}_{q,\text{it}}*\left({\text{PS}}_{\text{it}}+{\text{INN}}_{\text{it}}+{\text{FDI}}_{\text{it}}+{\text{IS}}_{\text{it}}+{\text{ED}}_{\text{it}}\right)+\beta_{v+q}{\text{CV}}_{v,\text{it}}*{\text{QG}}_{q,\text{it}}*\left({\text{PS}}_{\text{it}}+{\text{INN}}_{\text{it}}+{\text{FDI}}_{\text{it}}+{\text{IS}}_{\text{it}}+{\text{ED}}_{\text{it}}\right)$$

The marginal impact of **X** derived from Equations 6 and 11 are:

(Equation 16) $$\frac{\delta Y}{\delta X}=\alpha$$

(Equation 17) $$\frac{\delta Y}{\delta X}=\beta +\beta_{v}\text{CV}+\beta_{q}\text{QG}+\beta_{q+v}\text{CV}*\text{QG}$$

The conditions outlined in Equations 16 and 17 correspond to the marginal impacts before and after considering the effects of climate vulnerability and governance quality. Now, comparing these marginal impacts yields the condition **α**≷**β**+β_**v**_**CV+β**_**q**_**QG+β**_**q**+**v**_**CV∗QG, ∀q,v**. This difference in the marginal impacts depends on **CV** and **QG**. The mean and extreme values of these two factors will determine the value of $\frac{\delta Y}{\delta X}$.^66^

#### Two-stage empirical framework

The empirical framework consists of two complementary stages designed to address distinct but interconnected analytical objectives. In the first stage, moderated generalized least squares (GLS) estimation quantifies the average conditional associations between each SDG 7 determinant, climate vulnerability, governance quality, and their interaction terms while accounting for heteroskedasticity and serial correlation. This stage establishes whether governance quality systematically moderates the relationship between climate vulnerability and SDG 7 outcomes and provides the estimated coefficients that form the basis of the subsequent analysis.

The second stage translates these estimated interaction effects into policy-oriented interpretations through post-estimation marginal-effect evaluation. Specifically, the estimated marginal-effect surface is evaluated across the observed governance quality and climate vulnerability distributions to examine how the magnitude and direction of the conditional effects evolve under contrasting institutional environments. Building on these marginal effects, the extreme-scenario analysis summarizes the estimated relationships under stylized combinations of low and high governance quality and climate vulnerability, thereby identifying determinants whose effectiveness remains robust or becomes vulnerable under adverse institutional conditions. Consequently, the second stage is interpretative rather than a separate estimation procedure, as it derives entirely from the coefficients estimated in the moderated GLS model and provides an operational framework for translating statistical interactions into policy-relevant evidence. The proposed framework is intended to interpret continuous interaction effects through post-estimation marginal-effect evaluation rather than to estimate dynamic adjustment, discrete threshold effects, or conditional quantile relationships, which represent different empirical objectives. Accordingly, the proposed framework should be viewed as a theoretical extension of moderation analysis for policy evaluation. Rather than focusing solely on whether governance or climate vulnerability influence SDG 7 outcomes, it explicitly evaluates how these institutional conditions alter the effectiveness of conventional policy instruments across contrasting governance-vulnerability regimes, thereby providing a policy-oriented interpretation of conditional interaction effects.

#### Scenario setting

Following the estimation long-run coefficients for Equations 7, 8, 9, 10, 12, 13, 14, and 15 using GLS, the marginal impacts are assessed using Equations 16 and 17. Now, these marginal impacts will be used to develop two scenarios, i.e., BAU and Extreme scenarios.

##### BAU Scenario is designed using individual SDG 7 Subgoal determinants, climate vulnerability indicators, and governance quality indicators

Each SDG 7 Subgoal has 5 determinants, while climate vulnerability and governance quality have 5 and 6 indicators, respectively. So, there are 150 marginal effect evaluations (5 × 5 × 6 combinations) for each SDG 7 Subgoal determinant. The comparison is carried out by evaluating the marginal effects obtained from the baseline specification (Equation 16) and the moderated specification (Equation 17). Equation 17 clearly shows that the marginal effects $\frac{\delta Y_{\text{it}}}{\delta X_{\text{it}}}$ are dependent on the values of **CV**_**v**,**it**_ and **QG**_**v**,**it**_.

Assuming the climatic and governance conditions in country are likely to follow a central tendency, BAU scenario is developed at the respective means of **CV**_**v**,**it**_ and **QG**_**v**,**it**_, i.e., μ_CV_ and μ_QG_. These means are the respective sample means to reflect the central tendency, so that **E[CV] = μ**_**CV**_, **E[QG] = μ**_**QG**_. While evaluating the country-specific BAU setting, central tendencies become country-specific, i.e., **E[CV**_**i**_**] =** **E[CV]**_**i**_ **= μ**_**CV,i**_, **E[QG**_**i**_**] =** **E[QG]**_**i**_ **= μ**_**QG**,**i**_. This gives Equation 17 the form:

(Equation 18) $$\frac{\delta Y}{\delta X}=\alpha +\alpha_{v}\mu_{\text{CV}}+\alpha_{q}\mu_{\text{QG}}+\alpha_{q+v}\mu_{\text{CV}}*\mu_{\text{QG}}$$

The conditions are explained as follows:

**α < α + α**_**v**_**μ_CV_** **+ α**_**q**_**μ**_**QG**_ **+ α**_**q**+**v**_**μ**_**CV**_**∗μ**_**QG**_ means the marginal impact of subgoal indicators is increased under joint influence of climate vulnerability and governance quality.

**α > α + α**_**v**_**μ**_**CV**_ **+ α**_**q**_**μ**_**QG**_ **+ α**_**q+v**_**μ**_**CV**_**∗μ**_**QG**_ means the marginal impact of subgoal indicators is decreased under joint influence of climate vulnerability and governance quality.

##### Extreme Scenario has extended the BAU scenario into extremes

Unlike the BAU Scenario, the Extreme Scenario is developed by taking the quantile values of each climate vulnerability and governance quality indicator. Each SDG 7 Subgoal has 5 determinants, while climate vulnerability and governance quality have 5 and 6 indicators, respectively. These indicators are divided into 19 quantiles (5^th^-95^th^), and that gives the fitted marginal-effect surface is evaluated for 54,150 climate vulnerability-governance quality combinations (19 × 19 × 5 x 5 × 6). The comparison is carried out by evaluating the marginal effects obtained from the baseline and moderated specifications. However, rather than taking the mean values, quantile combinations of climate vulnerability and governance quality indicators are used. If only highest (H: 95^th^ quantile) and lowest (L: 5^th^ quantile) values of these indicators are considered, i.e., {CV_**H**_, CV_**L**_} and {QG_**H**_, QG_**L**_}, then the conditions are:

(Equation 19) $$\begin{array}{c} \alpha ≷\alpha +\alpha_{v}{\text{CV}}_{L}+\alpha_{q}{\text{QG}}_{L}+\alpha_{q+v}{\text{CV}}_{L}*{\text{QG}}_{L}\\ \alpha ≷\alpha +\alpha_{v}{\text{CV}}_{H}+\alpha_{q}{\text{QG}}_{L}+\alpha_{q+v}{\text{CV}}_{H}*{\text{QG}}_{L}\\ \alpha ≷\alpha +\alpha_{v}{\text{CV}}_{L}+\alpha_{q}{\text{QG}}_{H}+\alpha_{q+v}{\text{CV}}_{L}*{\text{QG}}_{H}\\ \alpha ≷\alpha +\alpha_{v}{\text{CV}}_{H}+\alpha_{q}{\text{QG}}_{H}+\alpha_{q+v}{\text{CV}}_{H}*{\text{QG}}_{H} \end{array}\}$$

The marginal impacts under the Extreme Scenario are analyzed following these 8 conditions outlines in Equation 19.

To facilitate the interpretation of the interaction effects, the estimated marginal-effect surface was evaluated under stylized combinations of climate vulnerability and governance quality. Conditional effects were assessed at representative locations of the moderator distribution rather than at the observed minimum and maximum values. Whereas previous studies evaluated conditional effects at the 15th and 85th percentiles to represent relatively low and high moderator conditions,^66^ the present study employs the 5th and 95th percentiles to define extreme governance quality and climate vulnerability environments. This extension follows an extreme-scenario framework in which tail percentiles are used to evaluate the stability of the estimated relationships under adverse and favorable institutional conditions.^67^ Using percentile-based thresholds reduces the influence of isolated extreme observations^68^^,^^69^ while providing consistent stress-test settings across all moderator combinations.

## Supplemental information

Document S1. Figures S1–S107 and Tables S1–S103

Data S1. Dataset used for the analysis
