# Supplementary material for: Climate vulnerability and governance impact the payoff of policy drivers for SDG 7 outcomes in BRICS countries
Source: iScience. 2026 Sep 22;29(10):117700. doi: 10.1016/j.isci.2026.117700 (PMC13673360; doi:10.1016/j.isci.2026.117700)
Supplement: Supplementary file 1 [file mmc1.pdf]

iScience, Volume *29*

Supplemental information

Climate vulnerability and governance impact the payoff of policy drivers for SDG 7 outcomes in BRICS countries

Avik Sinha, Abhinav Jindal, and Shujaat Abbas

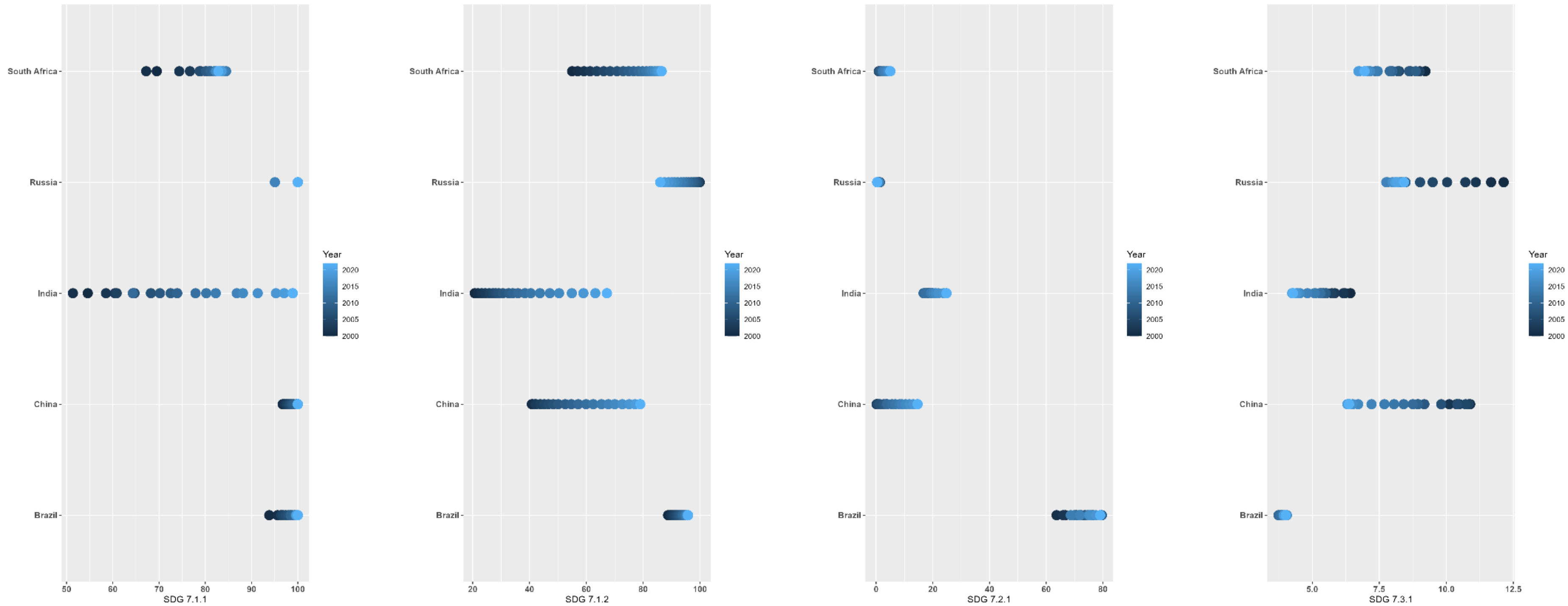

Figure S1: Distribution of the SDG 7 subgoals across the BRICS countries, Related to Figure 2

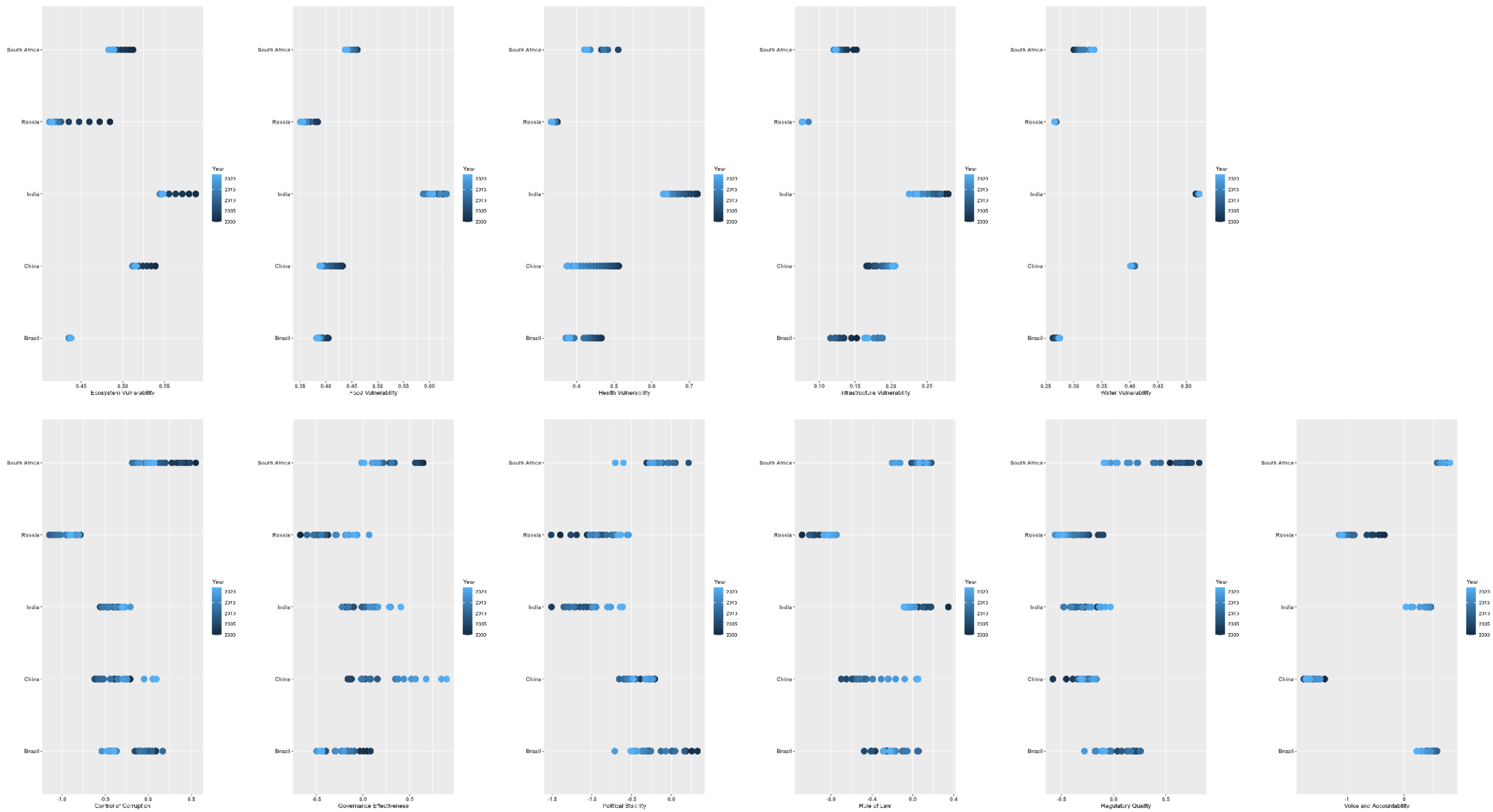

Figure S2: Distribution of the Climate Vulnerability and Governance Quality Indicators across the BRICS countries, Related to Figure 2

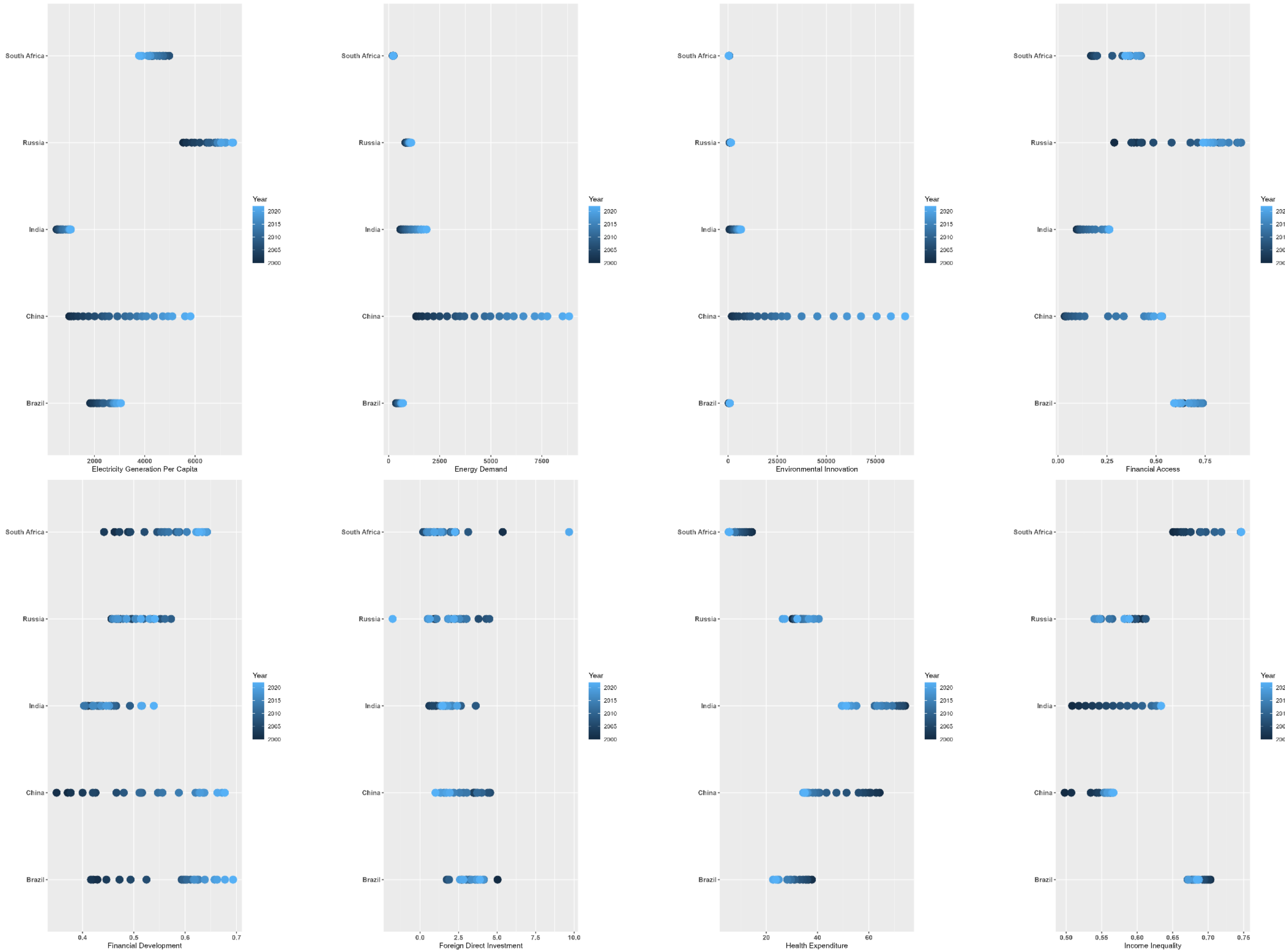
South Africa
Russia
India
China
Brazil
Year
2020
2015
2010
2005
2000
Electricity Generation Per Capita
Energy Demand
Environmental Innovation
Financial Access
Financial Development
Foreign Direct Investment
Health Expenditure
Income Inequality

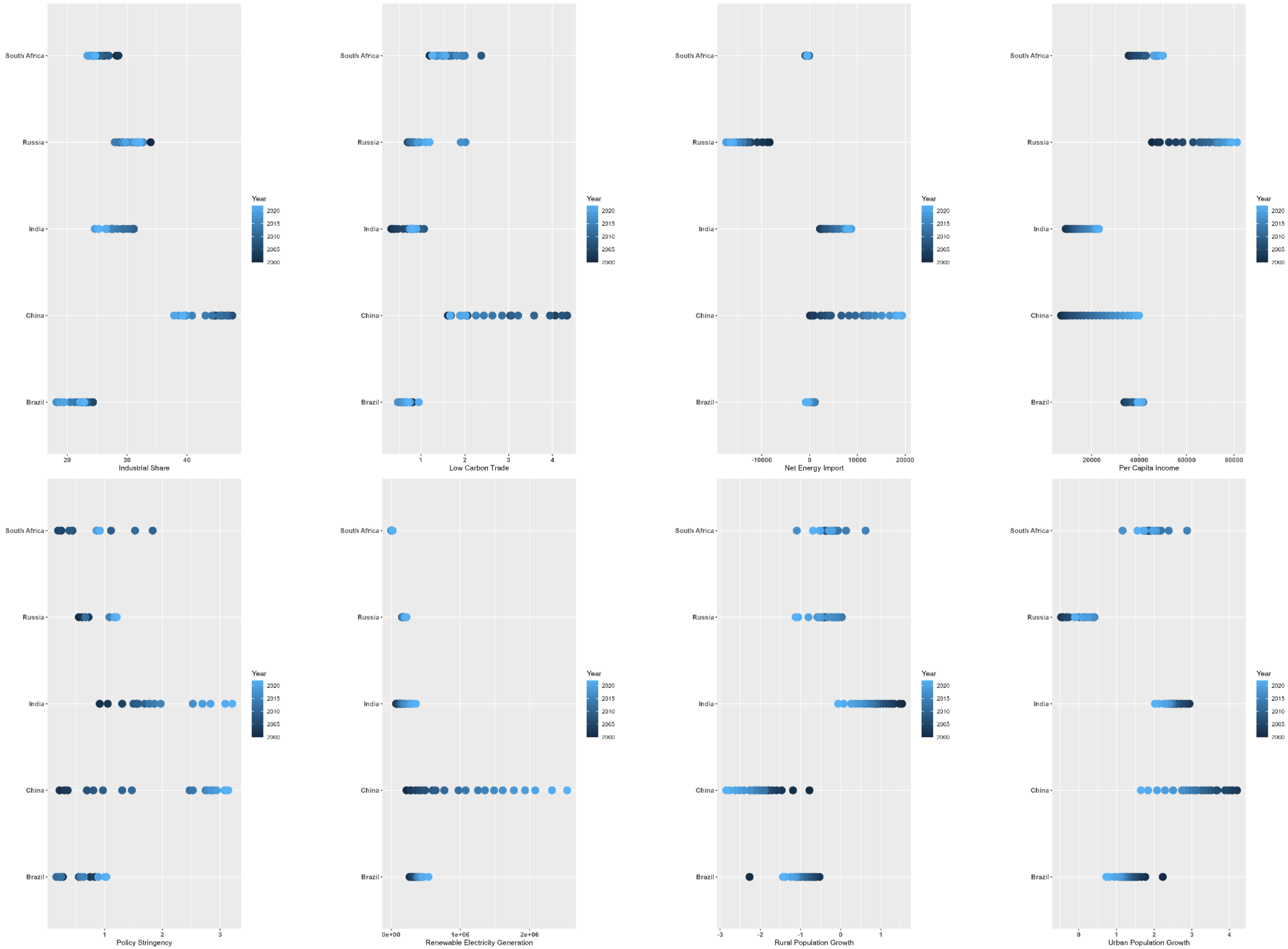

Figure S3: Distribution of the SDG 7 subgoal Determinants across the BRICS countries, Related to Figure 2

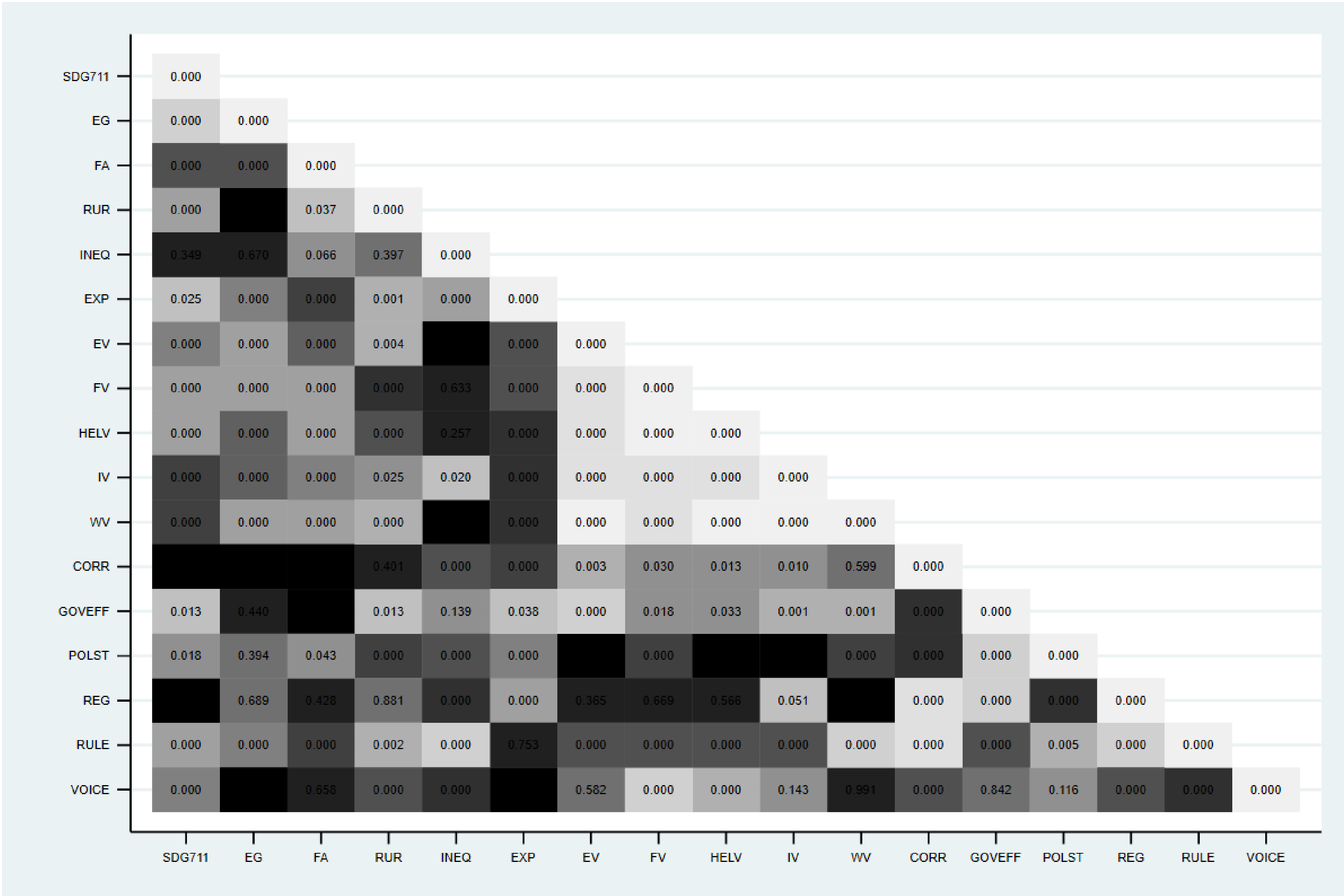

Figure S4: Correlation Matrix for SDG 7.1.1 Determinants, Related to Figure 2

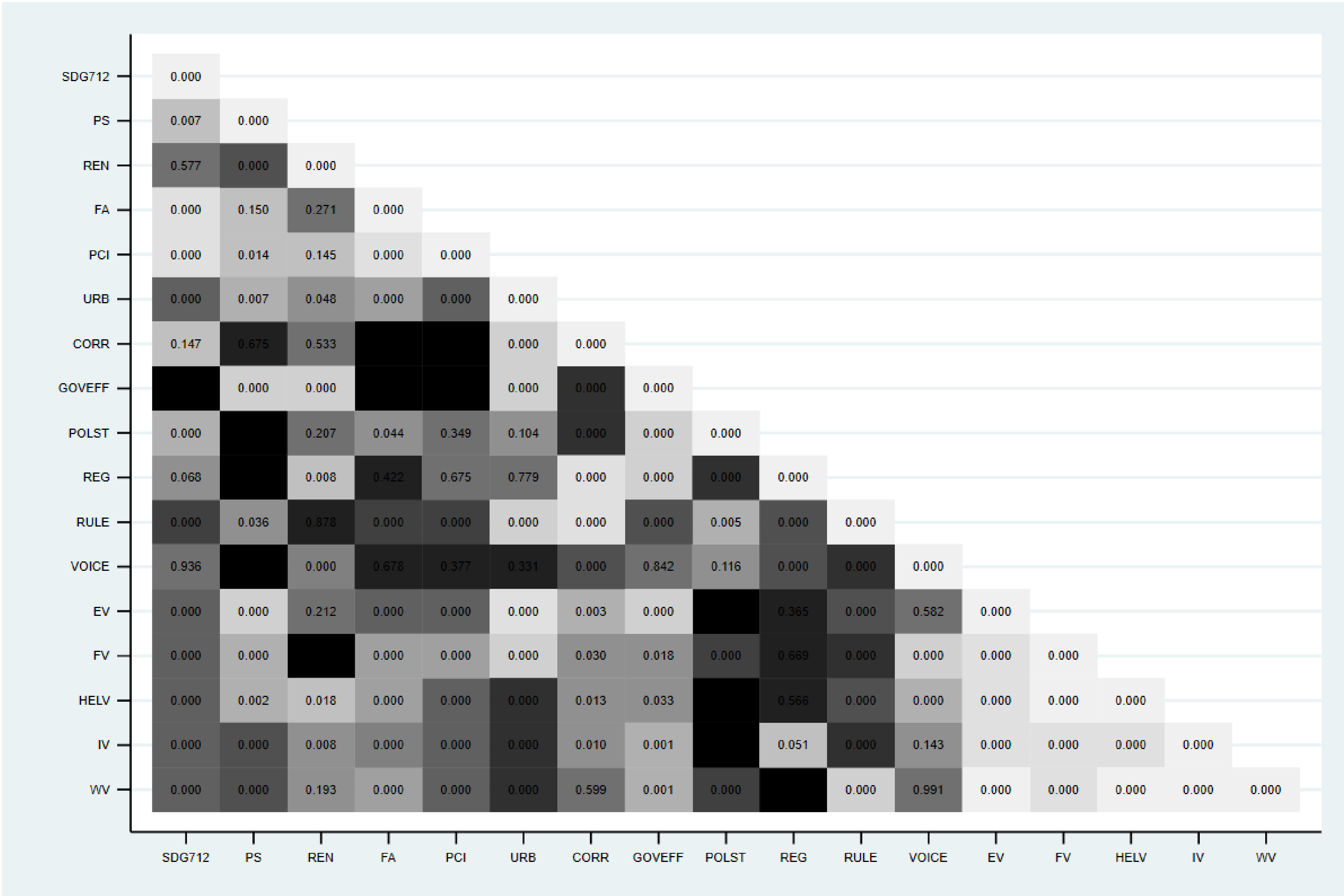

Figure S5: Correlation Matrix for SDG 7.1.2 Determinants, Related to Figure 2

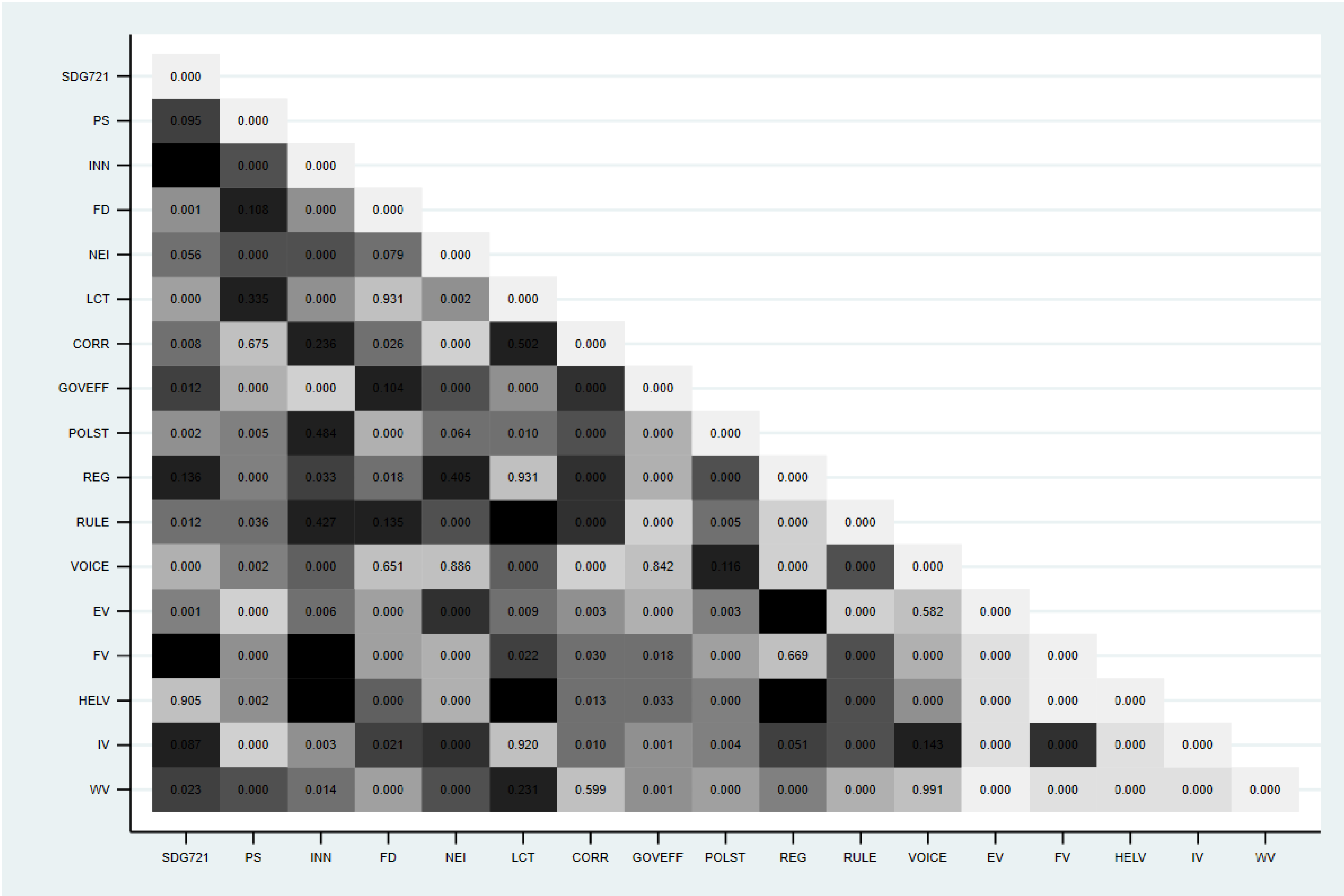

Figure S6: Correlation Matrix for SDG 7.2.1 Determinants, Related to Figure 2

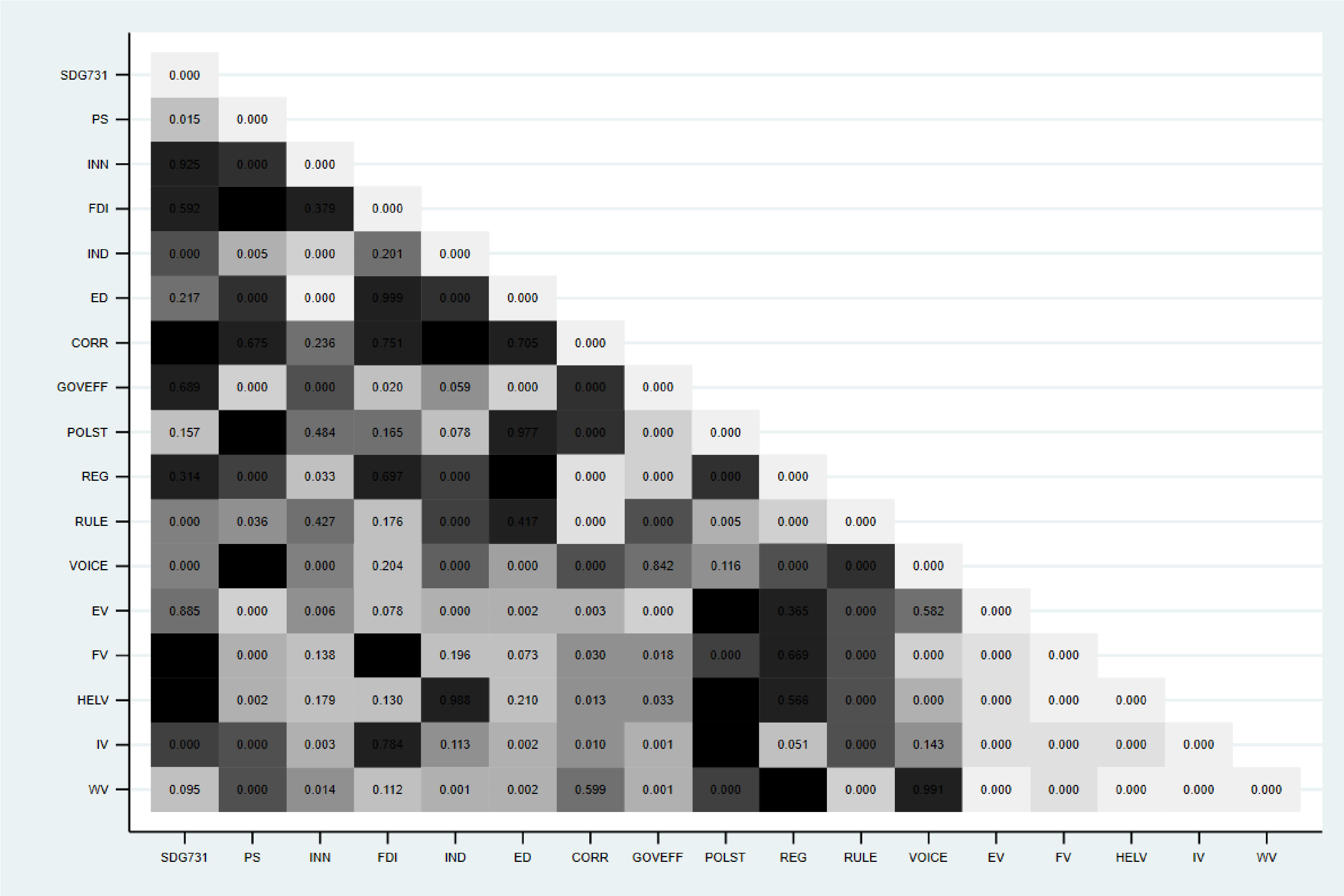

Figure S7: Correlation Matrix for SDG 7.3.1 Determinants, Related to Figure 2

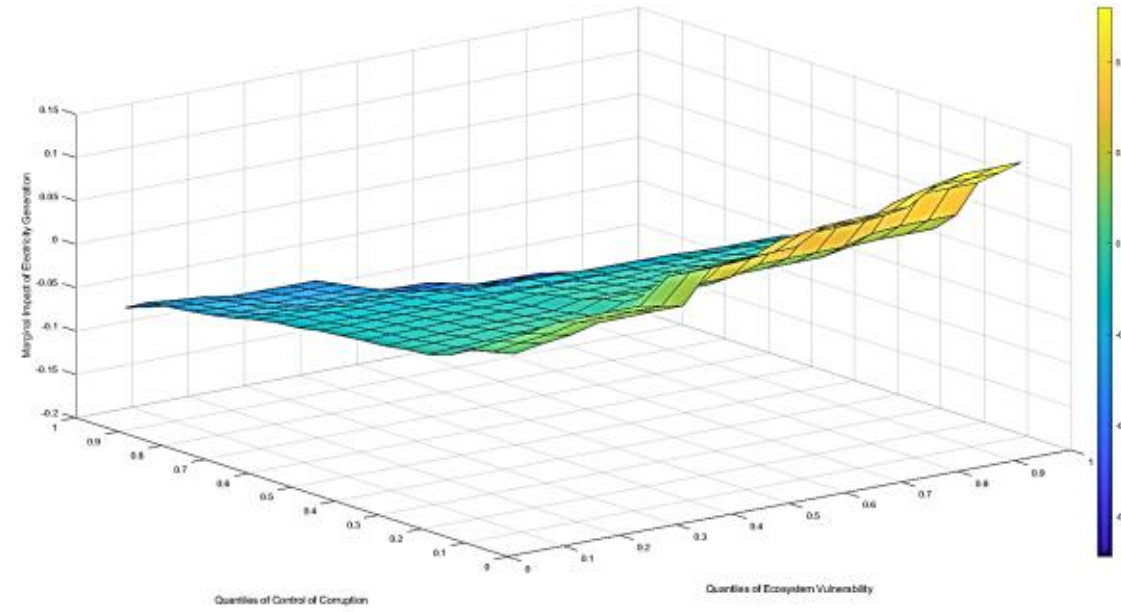

Governance Quality Indicator: Control of Corruption

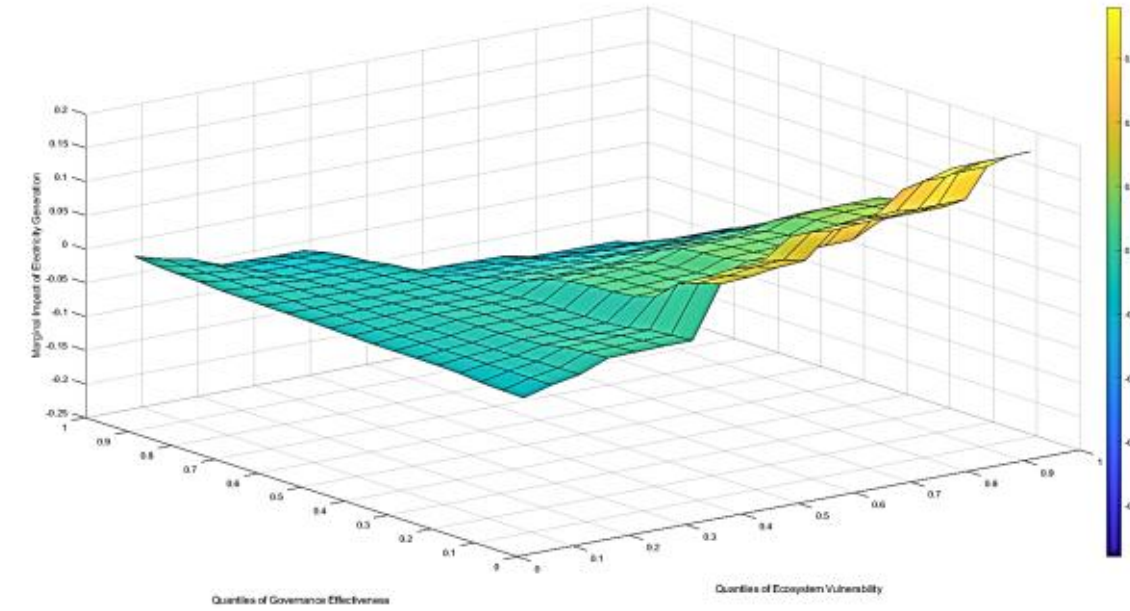

Governance Quality Indicator: Governance Effectiveness

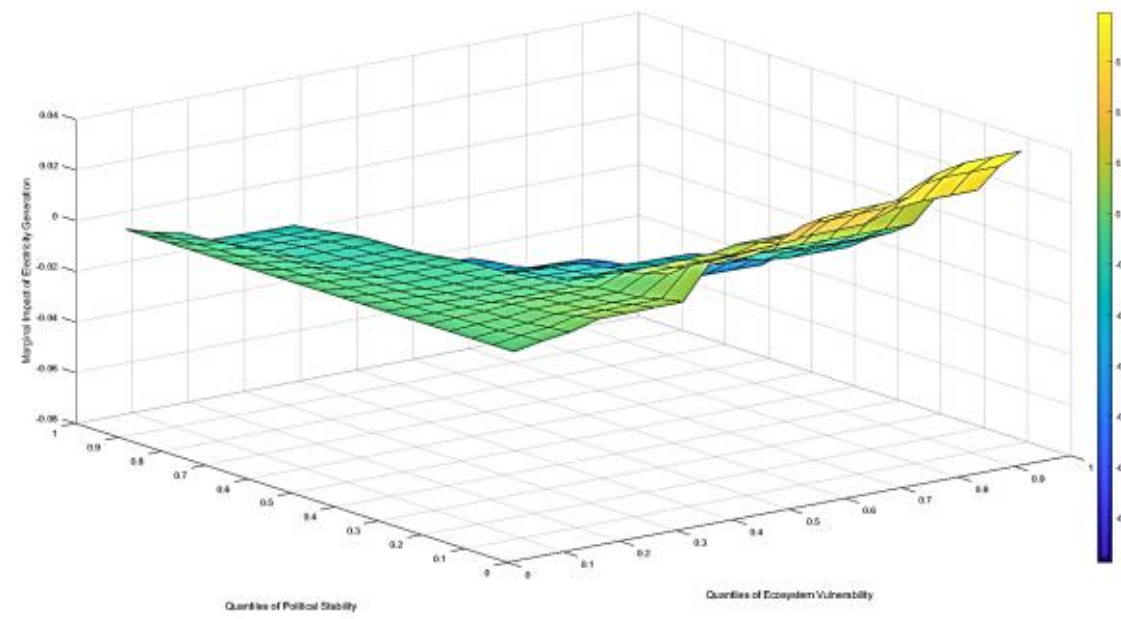

Governance Quality Indicator: Political Stability

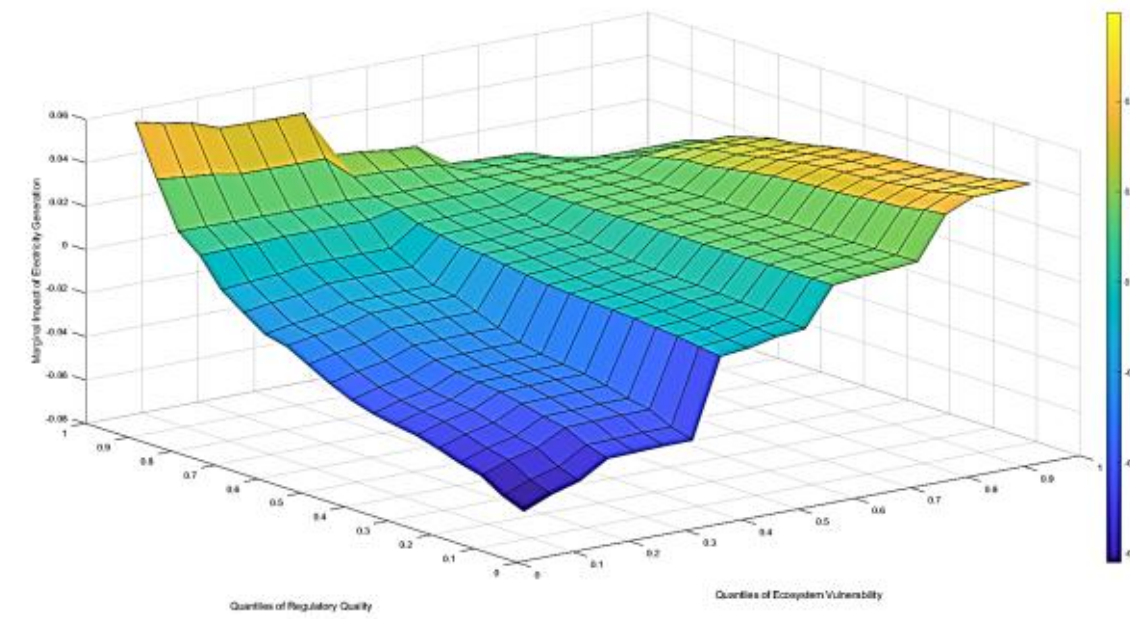

Governance Quality Indicator: Regulatory Quality

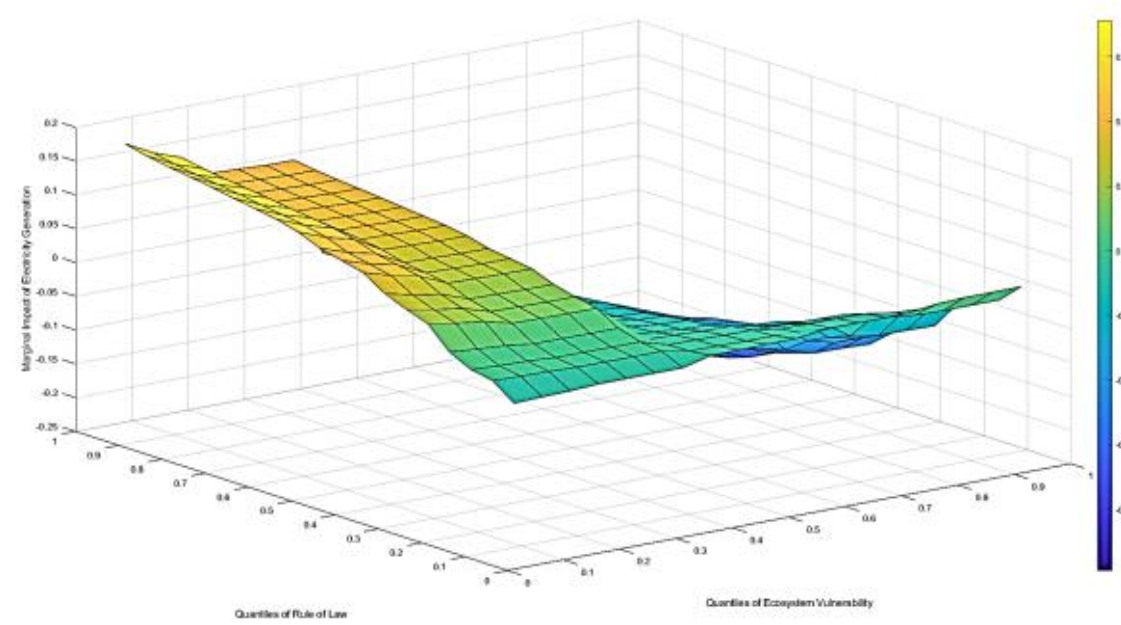

Governance Quality Indicator: Rule of Law

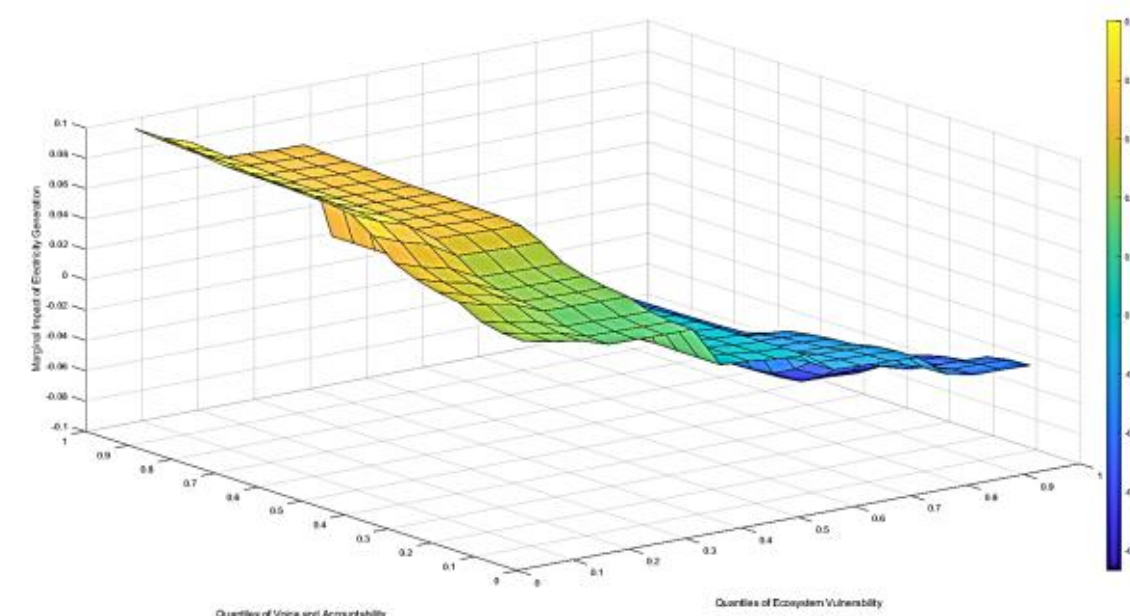

Governance Quality Indicator: Voice and Accountability

Figure S8: BRICS Policy Simulation Outcomes for the Marginal Impact of Electricity Generation on SDG 7.1.1 under Ecosystem Vulnerability and Governance Quality, Related to Figure 4-5 and Table 2

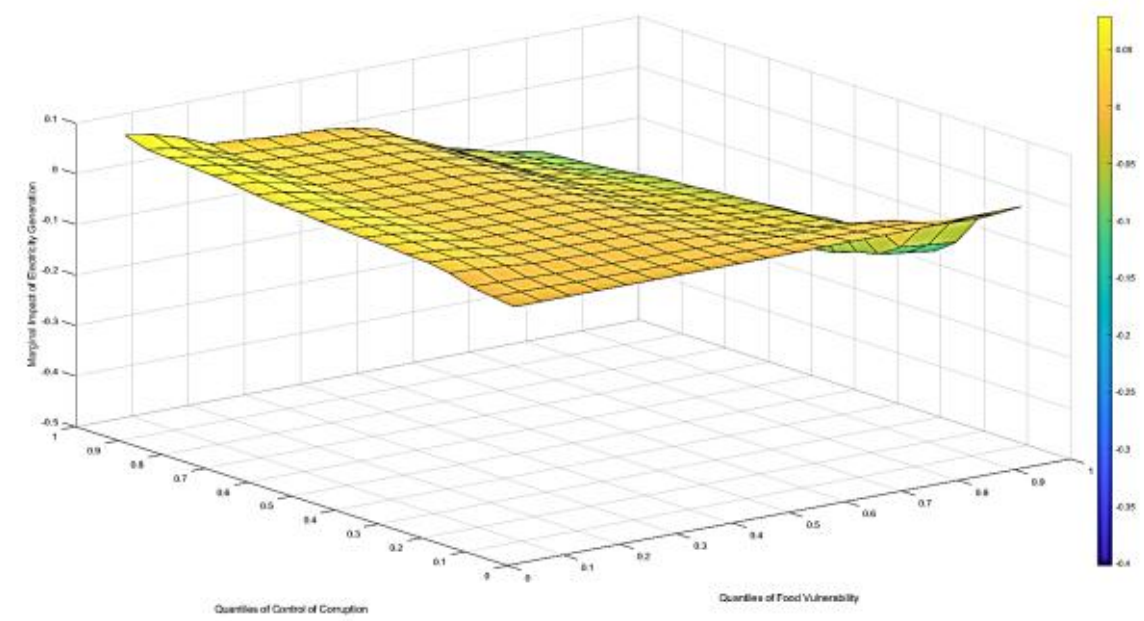

Governance Quality Indicator: Control of Corruption

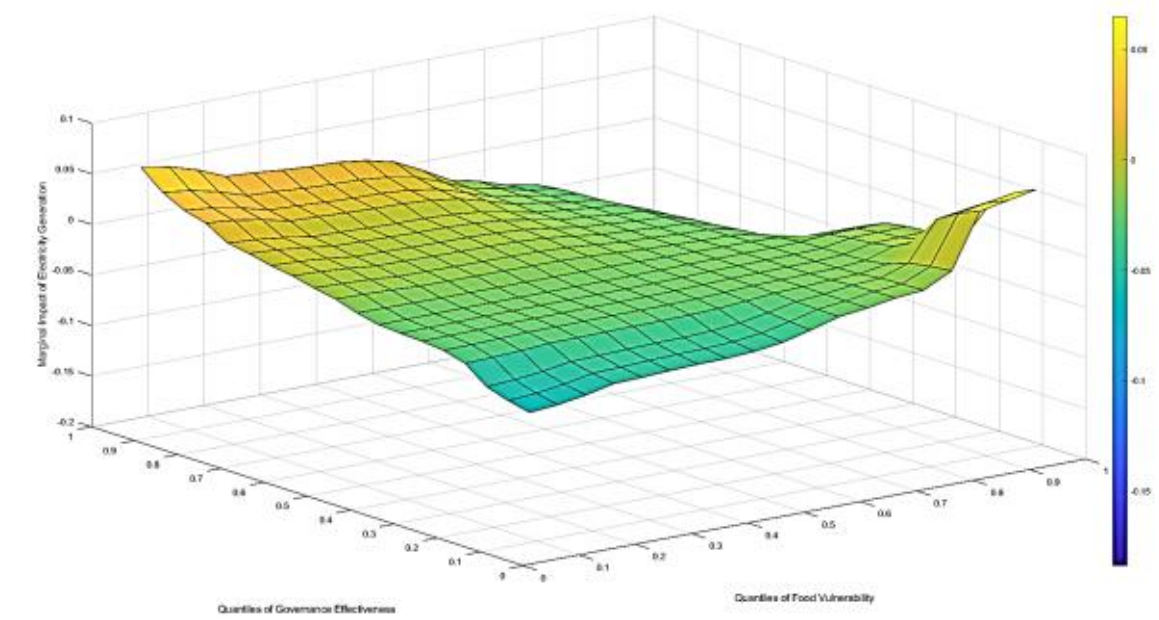

Governance Quality Indicator: Governance Effectiveness

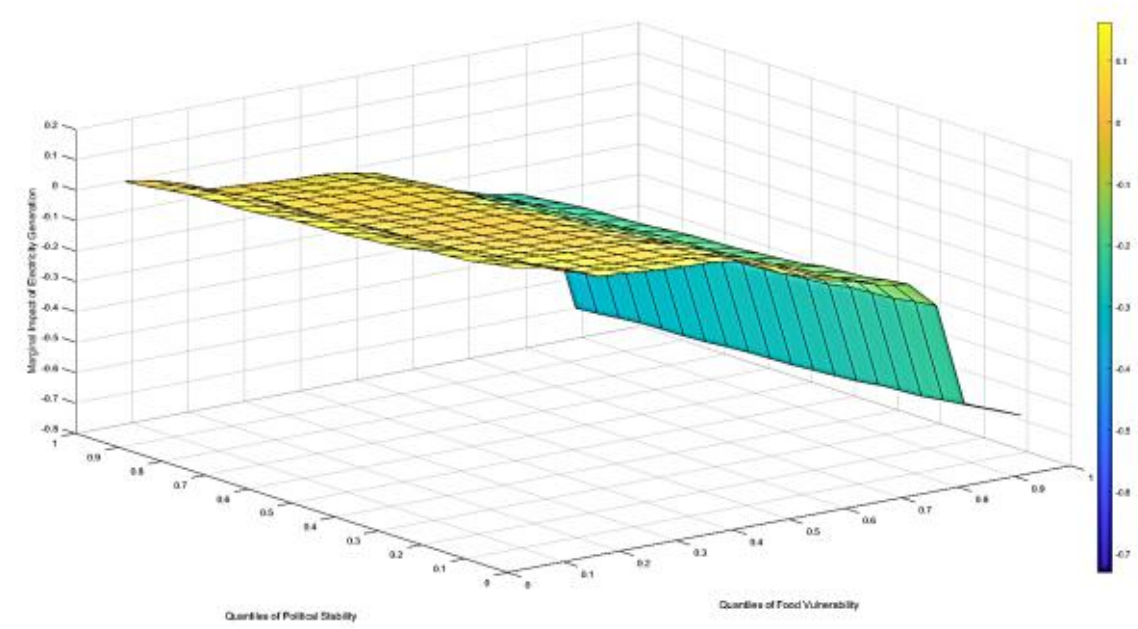

Governance Quality Indicator: Political Stability

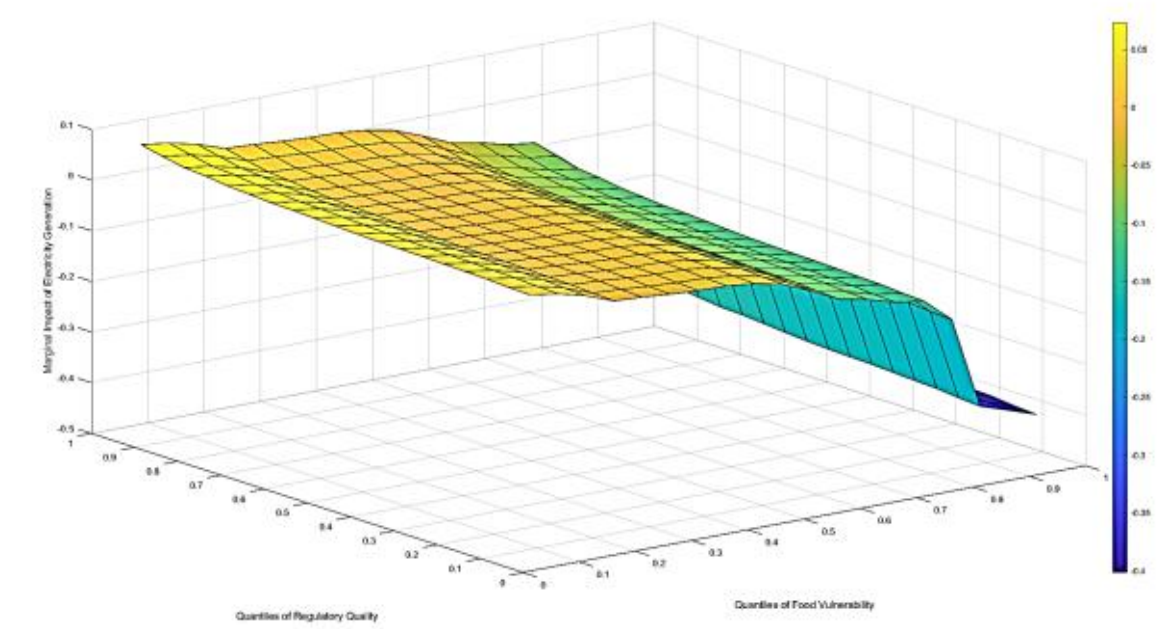

Governance Quality Indicator: Regulatory Quality

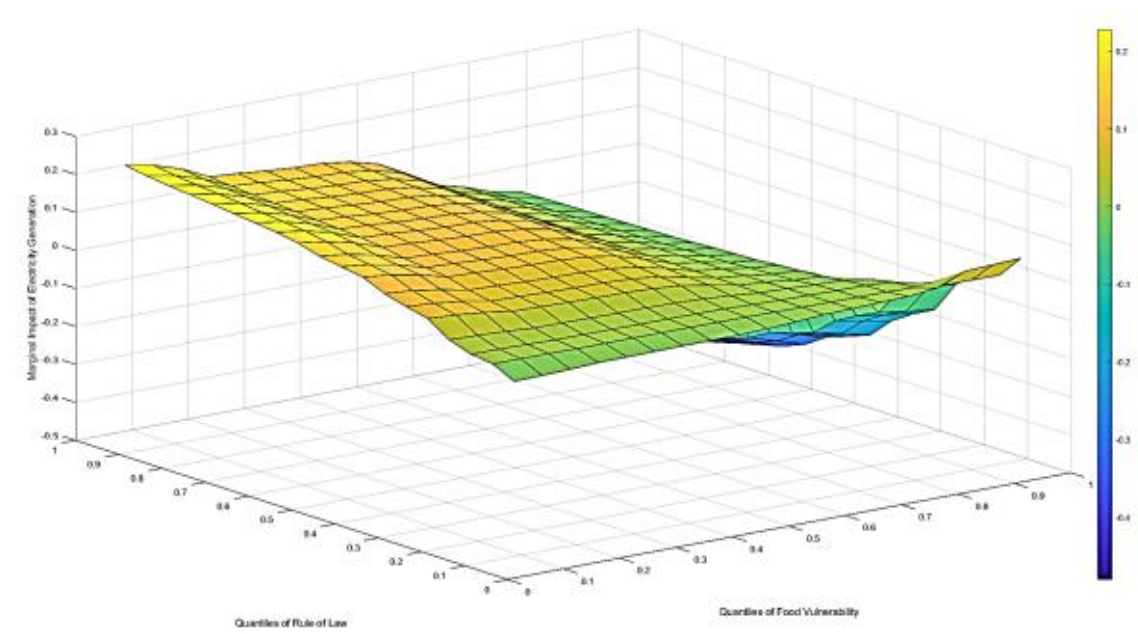

Governance Quality Indicator: Rule of Law

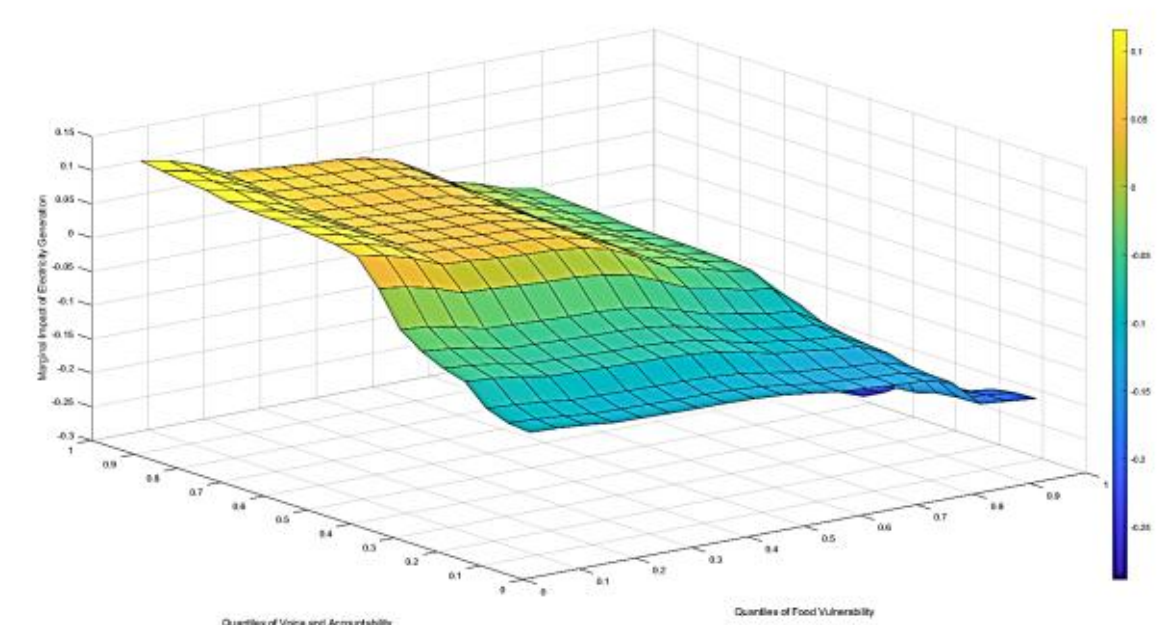

Governance Quality Indicator: Voice and Accountability

Figure S9: BRICS Policy Simulation Outcomes for the Marginal Impact of Electricity Generation on SDG 7.1.1 under Food Vulnerability and Governance Quality, Related to Figure 4-5 and Table 2

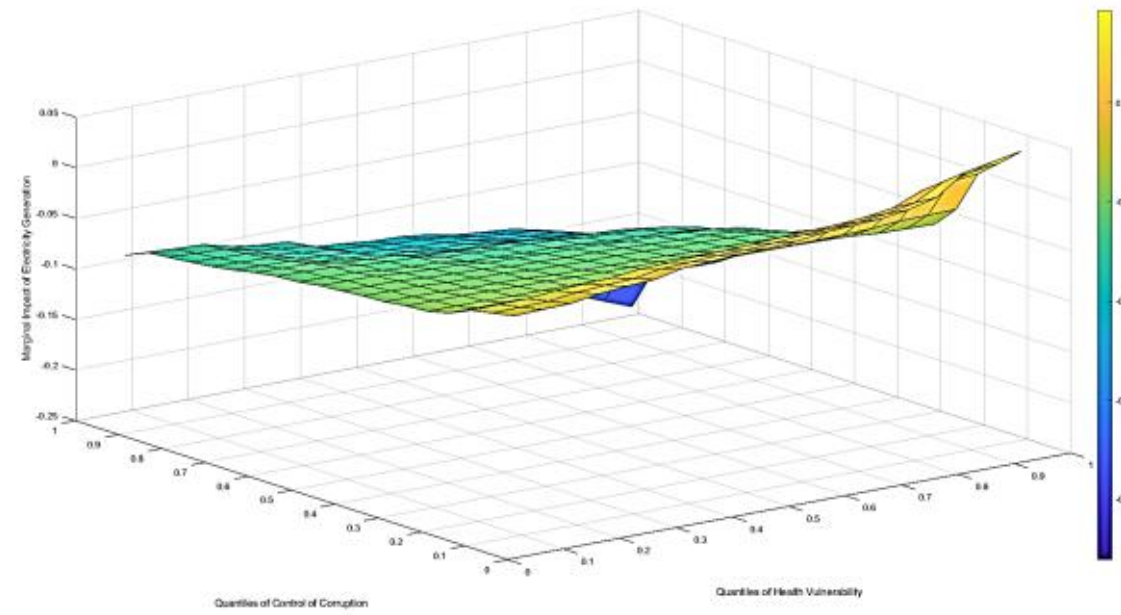

Governance Quality Indicator: Control of Corruption

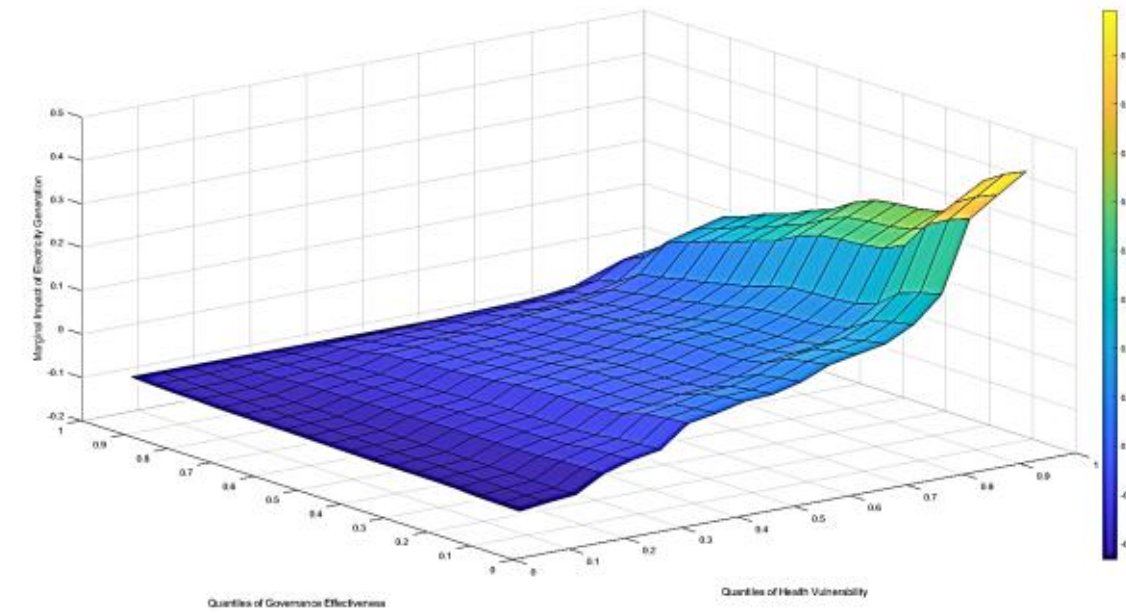

Governance Quality Indicator: Governance Effectiveness

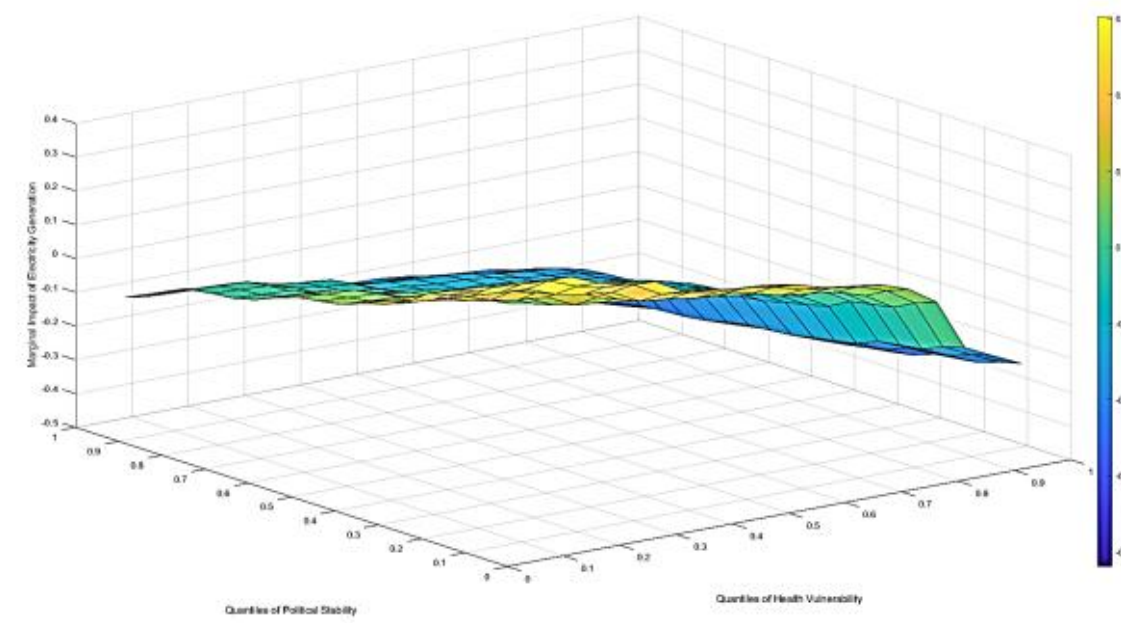

Governance Quality Indicator: Political Stability

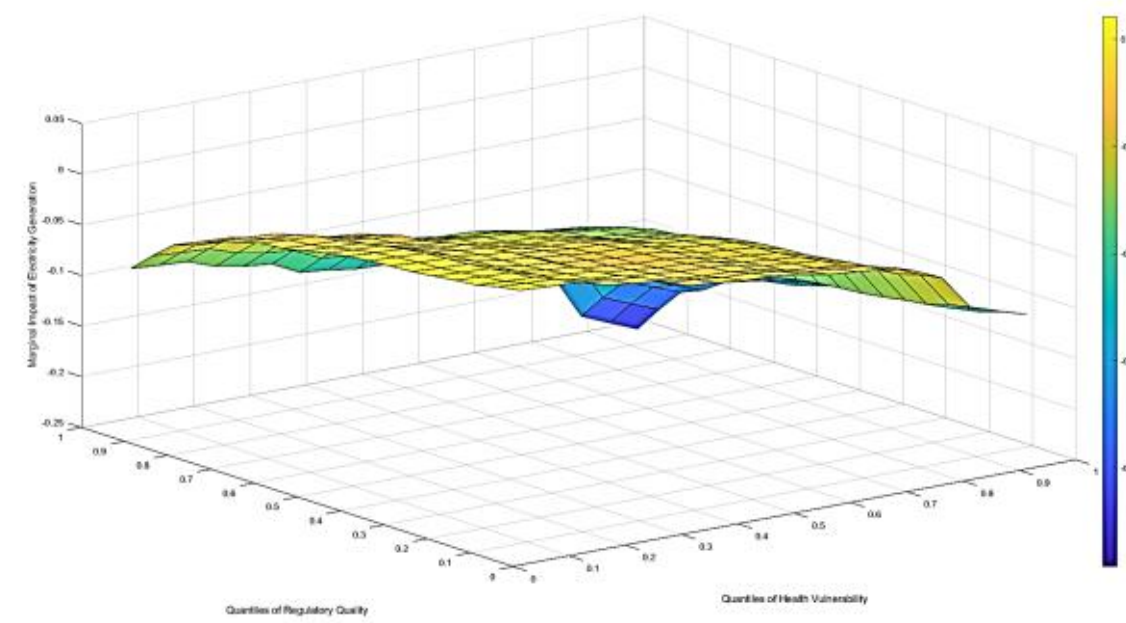

Governance Quality Indicator: Regulatory Quality

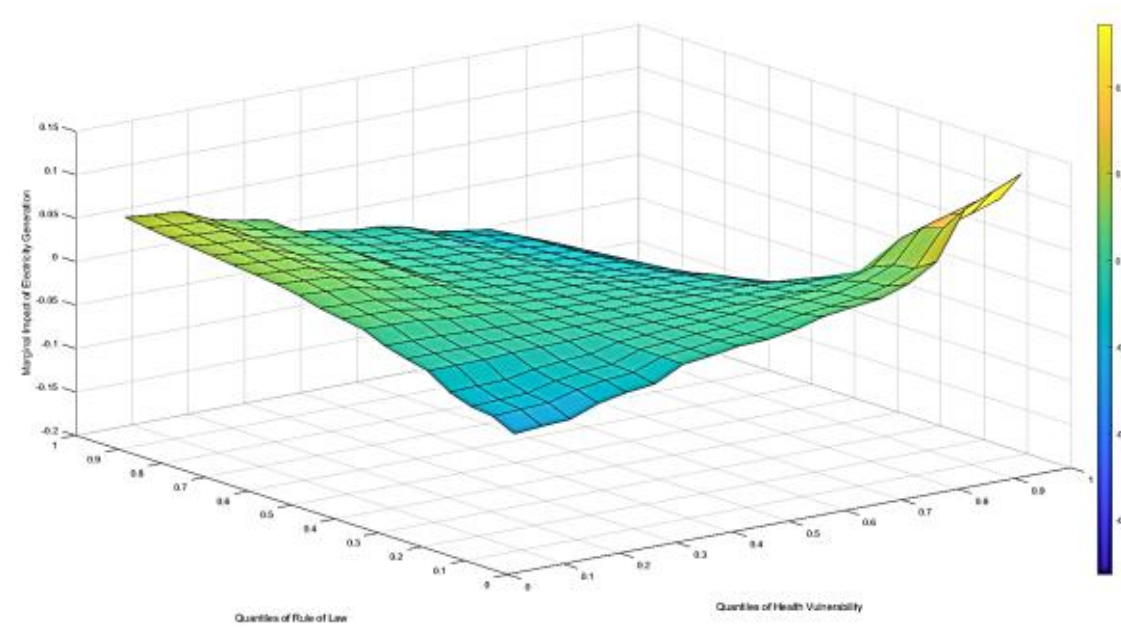

Governance Quality Indicator: Rule of Law

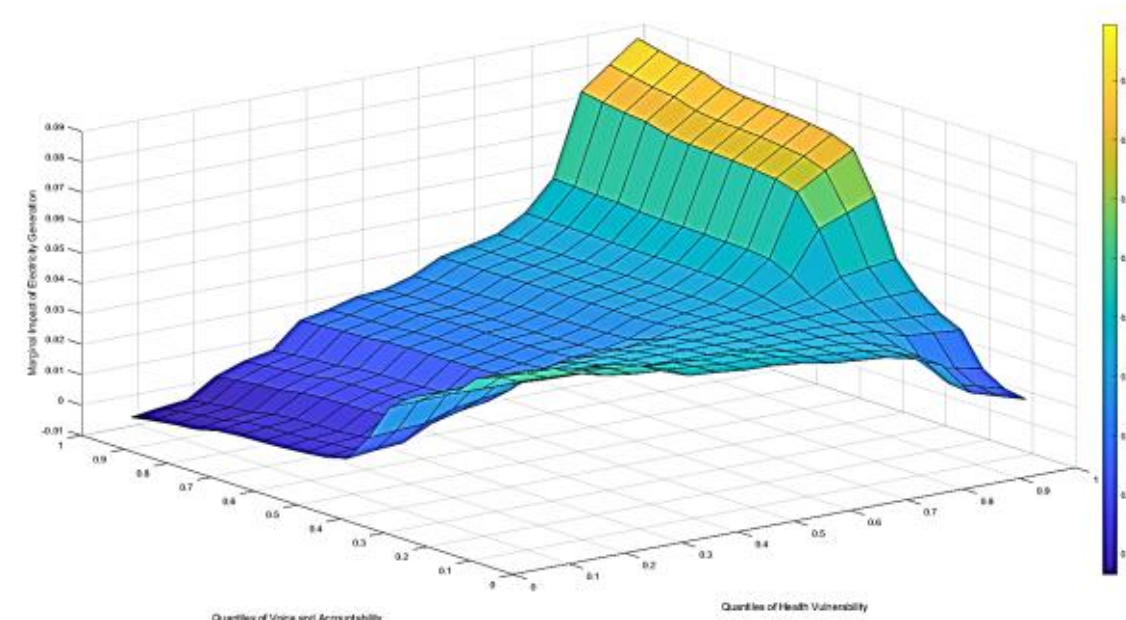

Governance Quality Indicator: Voice and Accountability

Figure S10: BRICS Policy Simulation Outcomes for the Marginal Impact of Electricity Generation on SDG 7.1.1 under Health Vulnerability and Governance Quality, Related to Figure 4-5 and Table 2

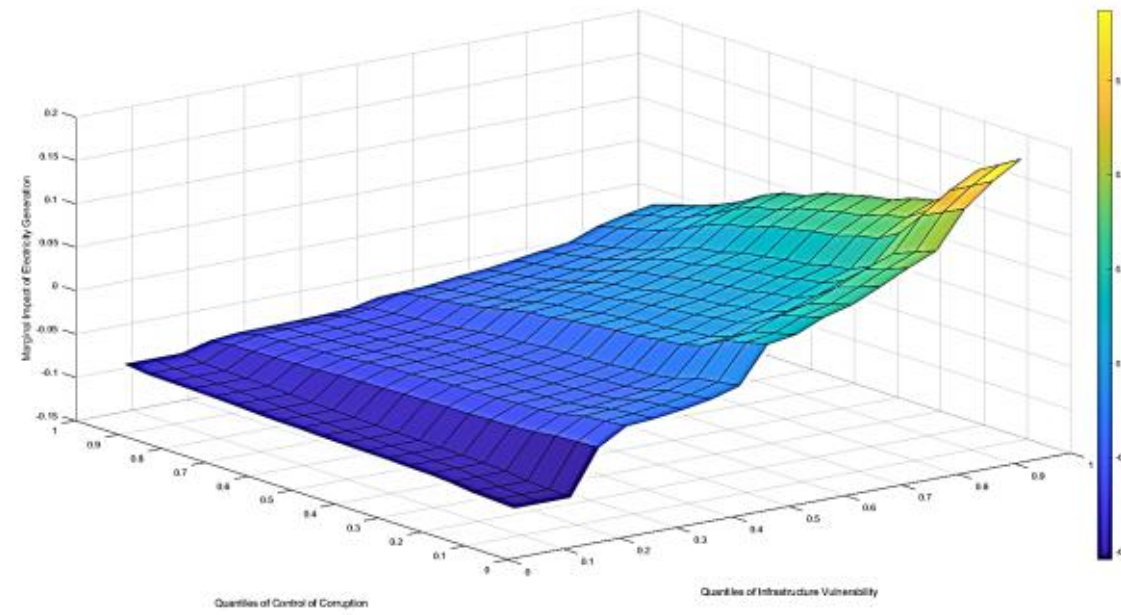

Governance Quality Indicator: Control of Corruption

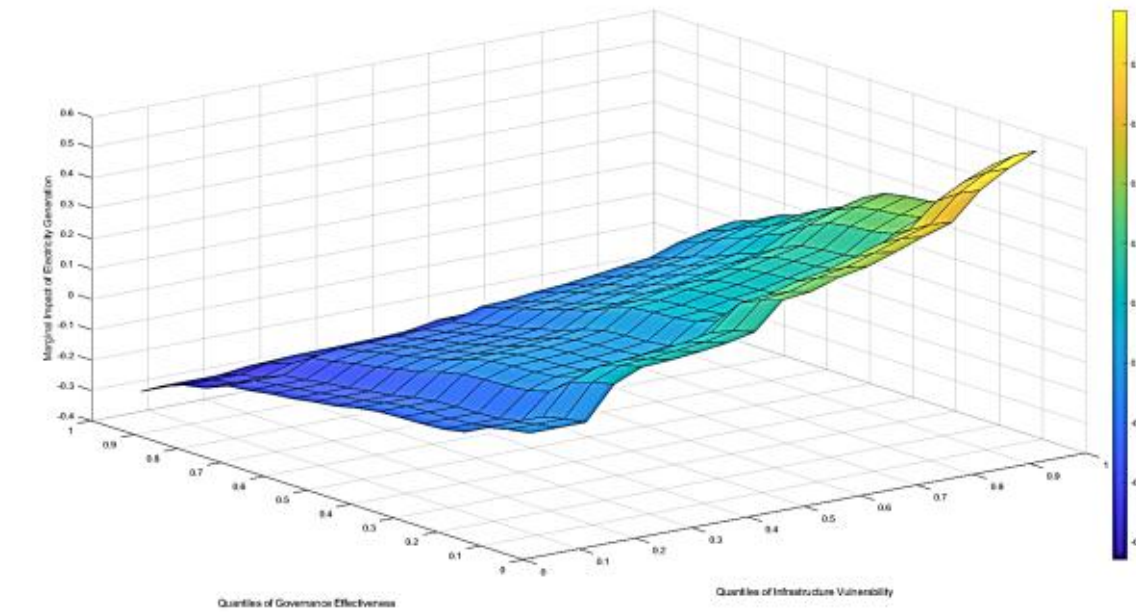

Governance Quality Indicator: Governance Effectiveness

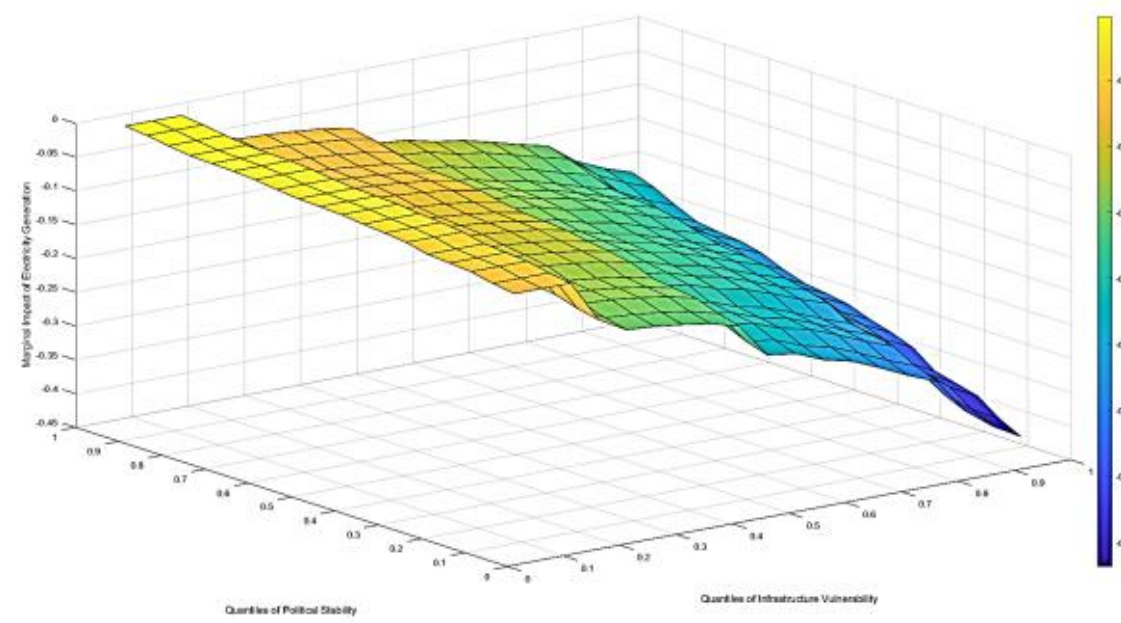

Governance Quality Indicator: Political Stability

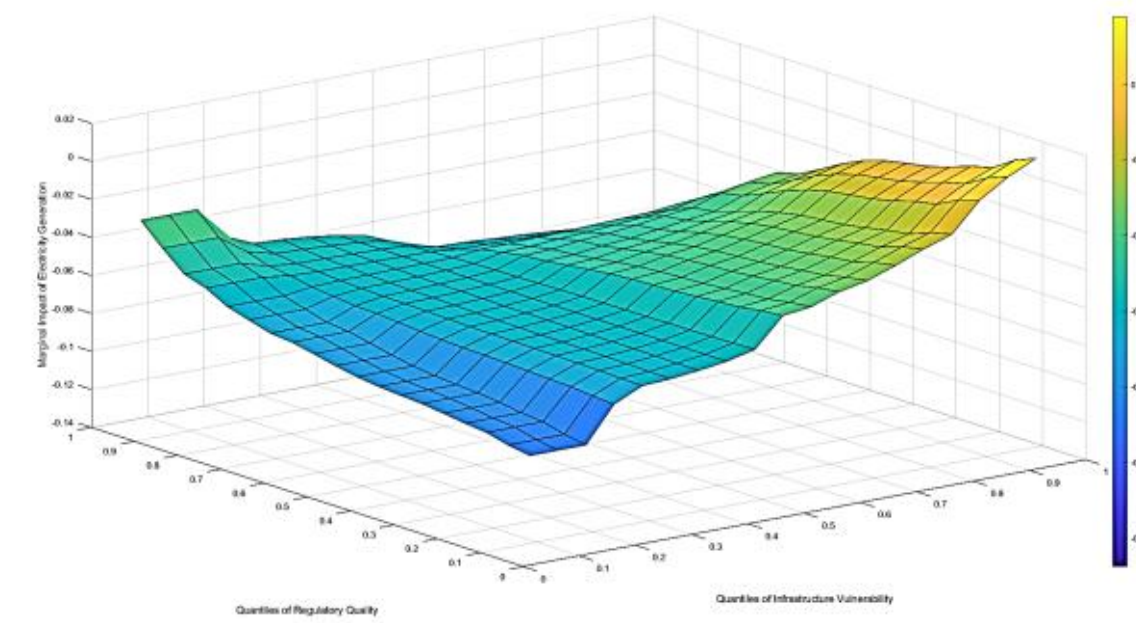

Governance Quality Indicator: Regulatory Quality

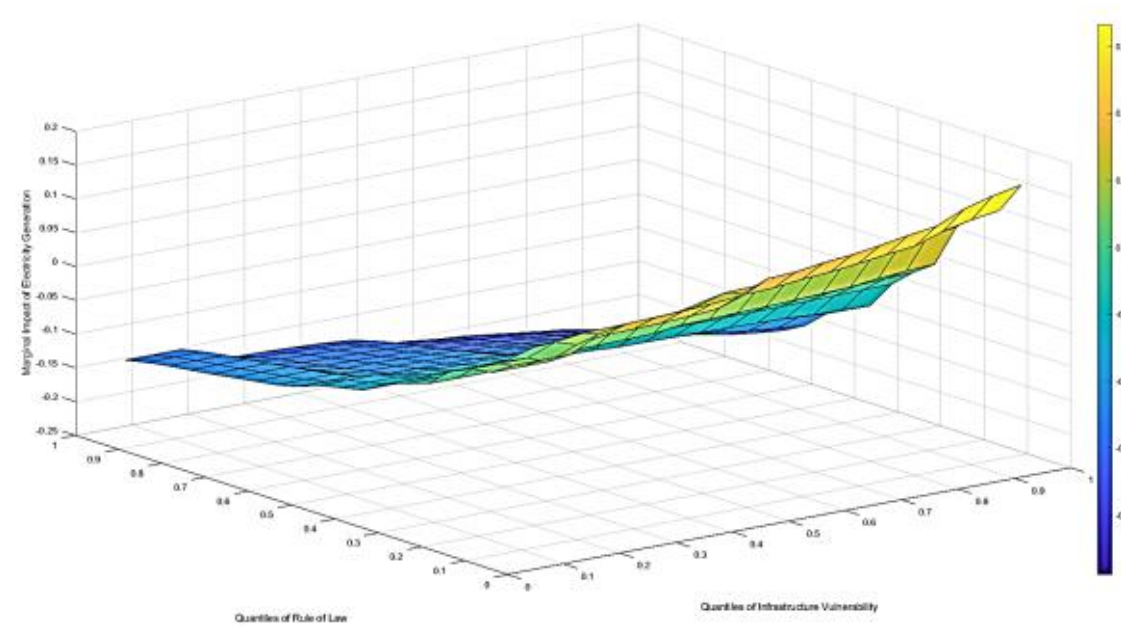

Governance Quality Indicator: Rule of Law

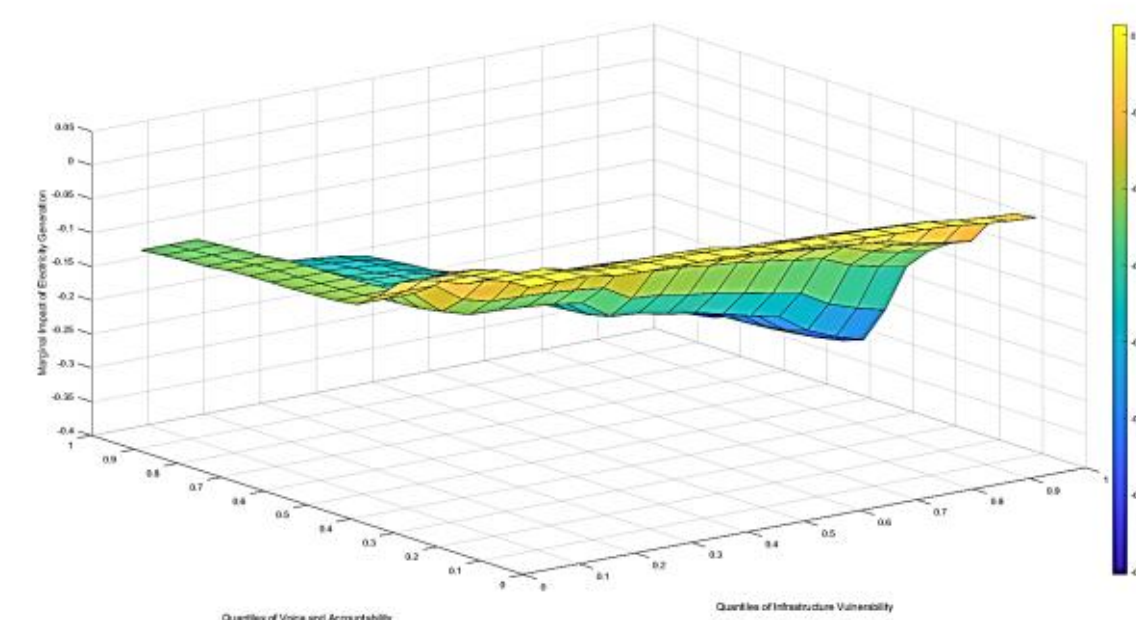

Governance Quality Indicator: Voice and Accountability

Figure S11: BRICS Policy Simulation Outcomes for the Marginal Impact of Electricity Generation on SDG 7.1.1 under Infrastructure Vulnerability and Governance Quality, Related to Figure 4-5 and Table 2

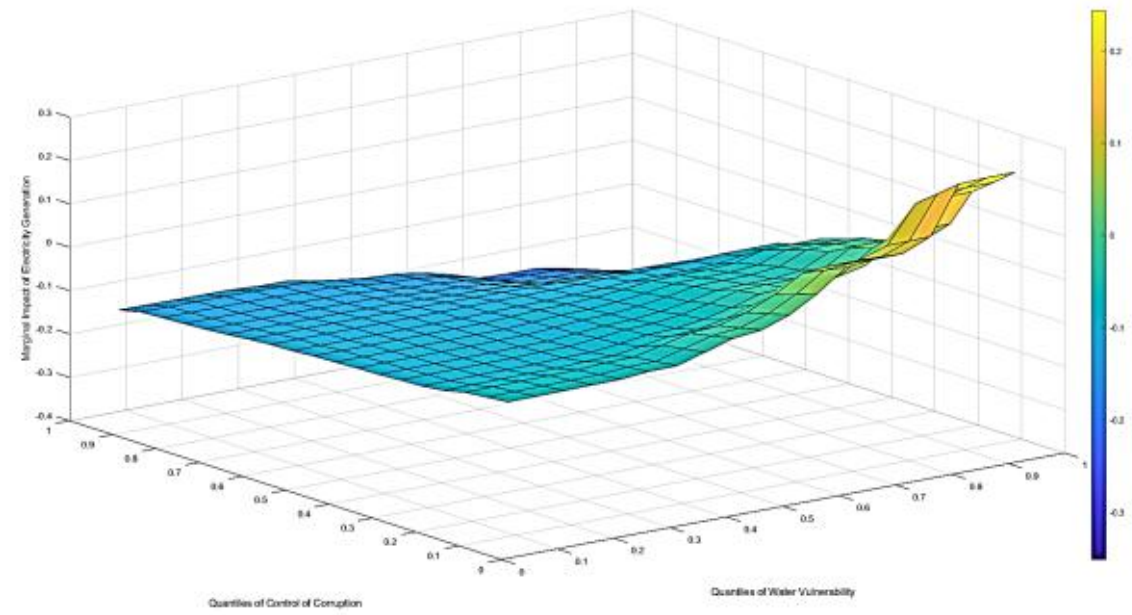

Governance Quality Indicator: Control of Corruption

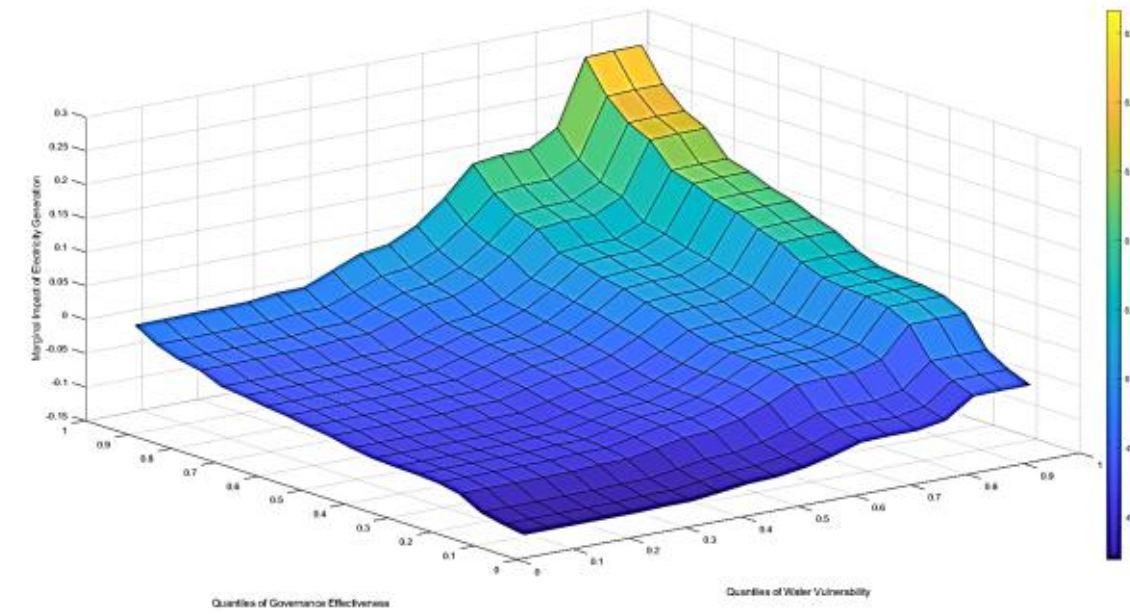

Governance Quality Indicator: Governance Effectiveness

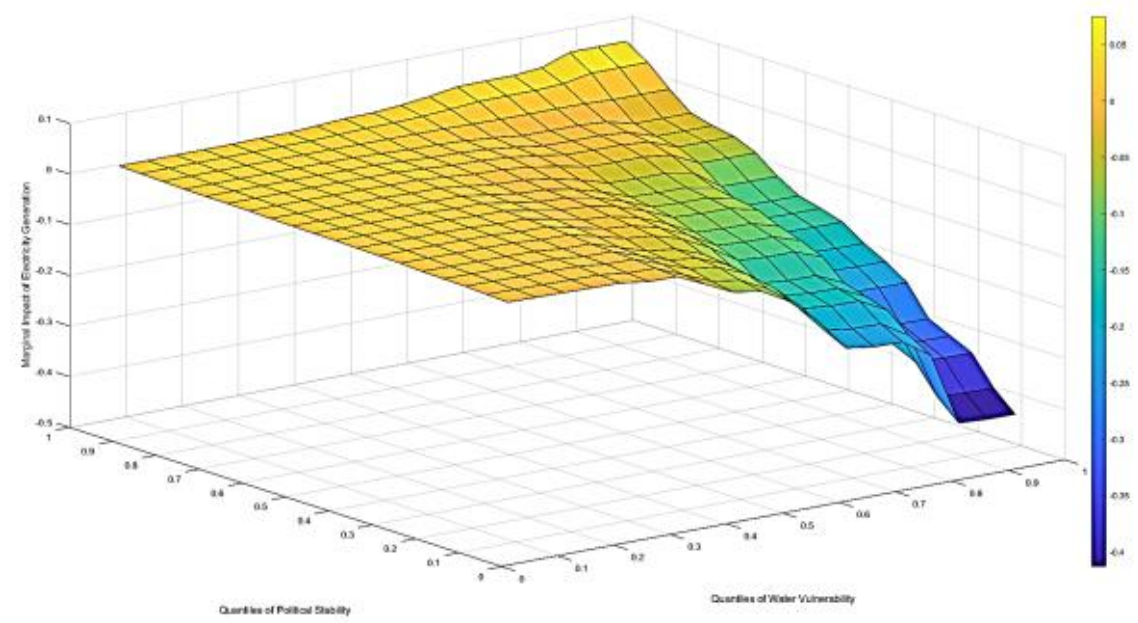

Governance Quality Indicator: Political Stability

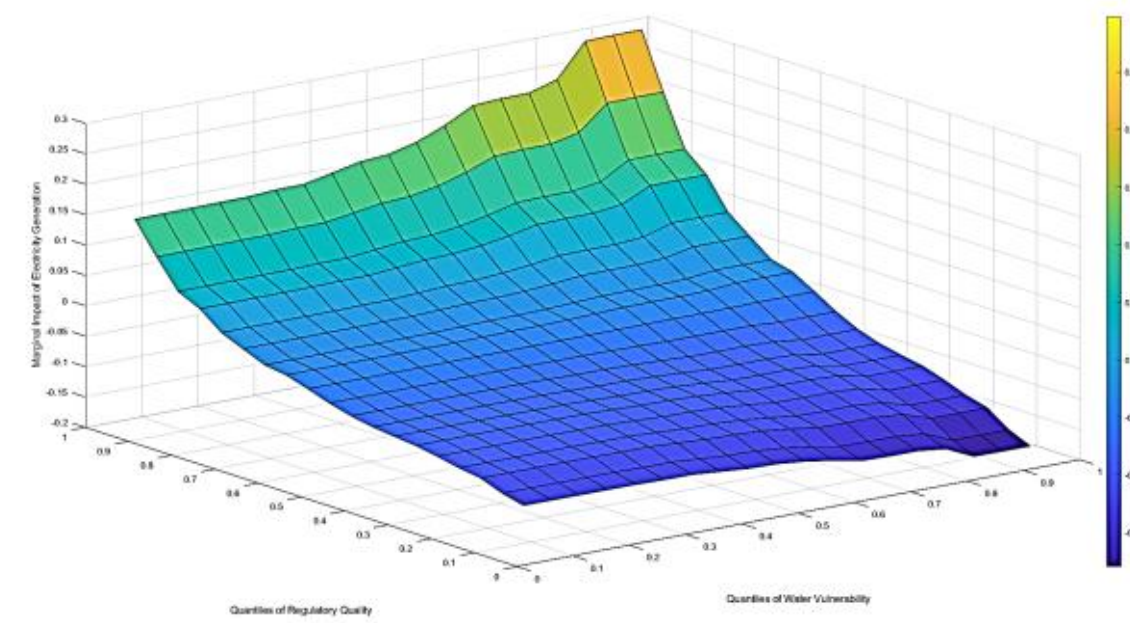

Governance Quality Indicator: Regulatory Quality

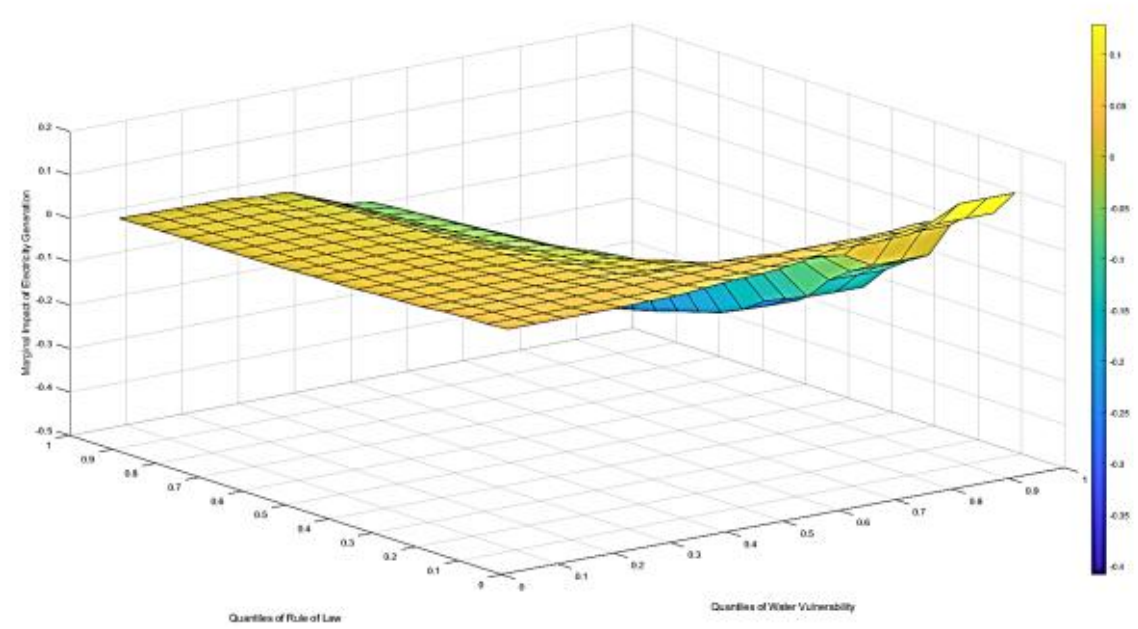

Governance Quality Indicator: Rule of Law

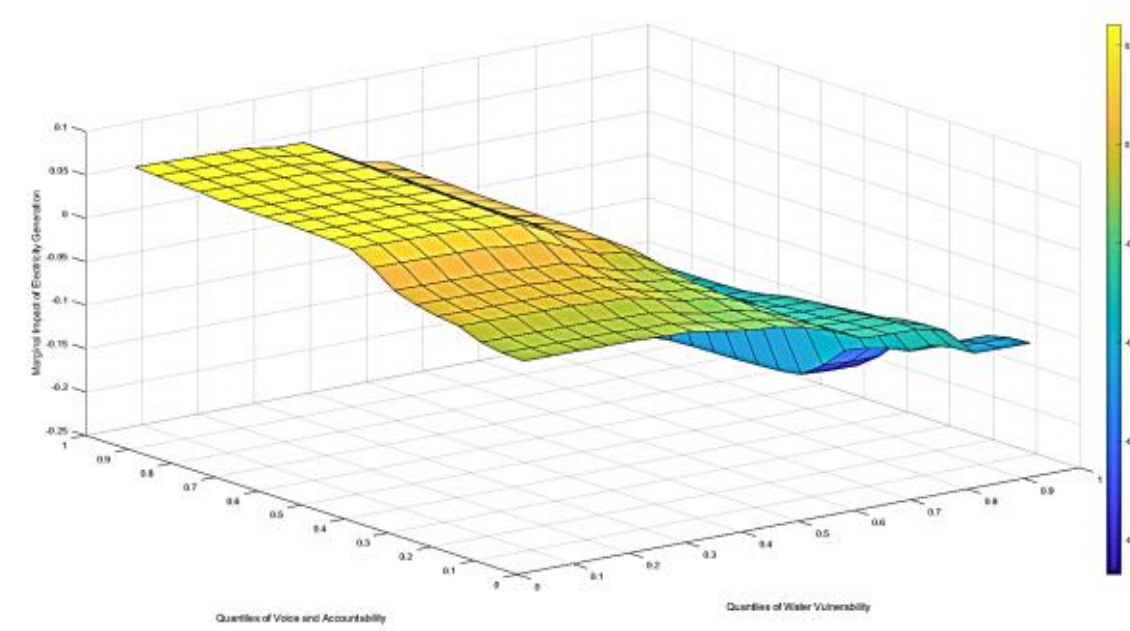

Governance Quality Indicator: Voice and Accountability

Figure S12: BRICS Policy Simulation Outcomes for the Marginal Impact of Electricity Generation on SDG 7.1.1 under Water Vulnerability and Governance Quality, Related to Figure 4-5 and Table 2

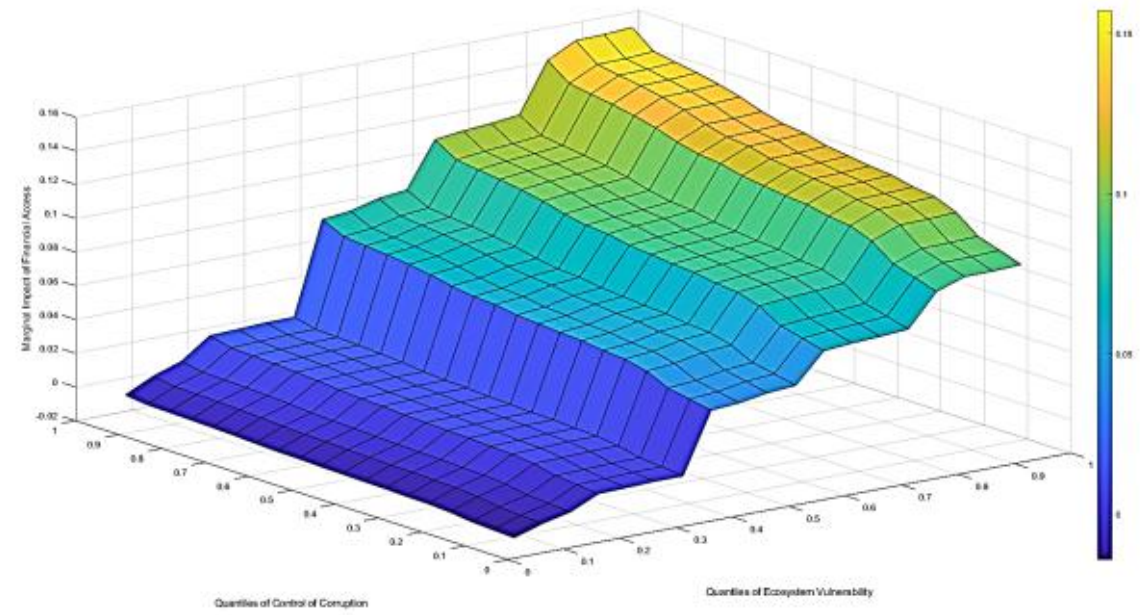

Governance Quality Indicator: Control of Corruption

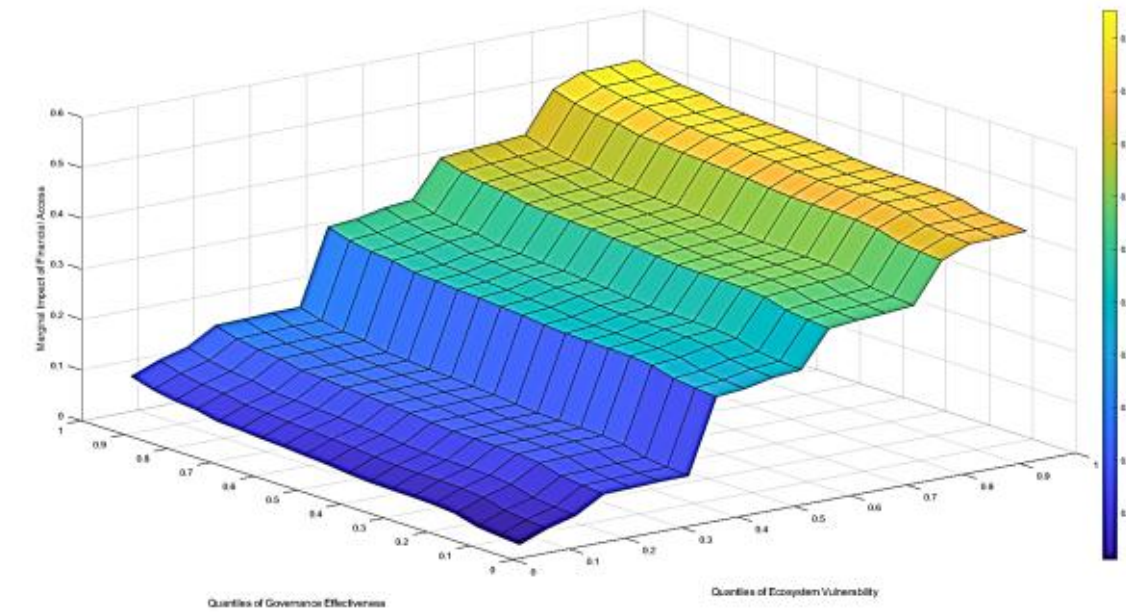

Governance Quality Indicator: Governance Effectiveness

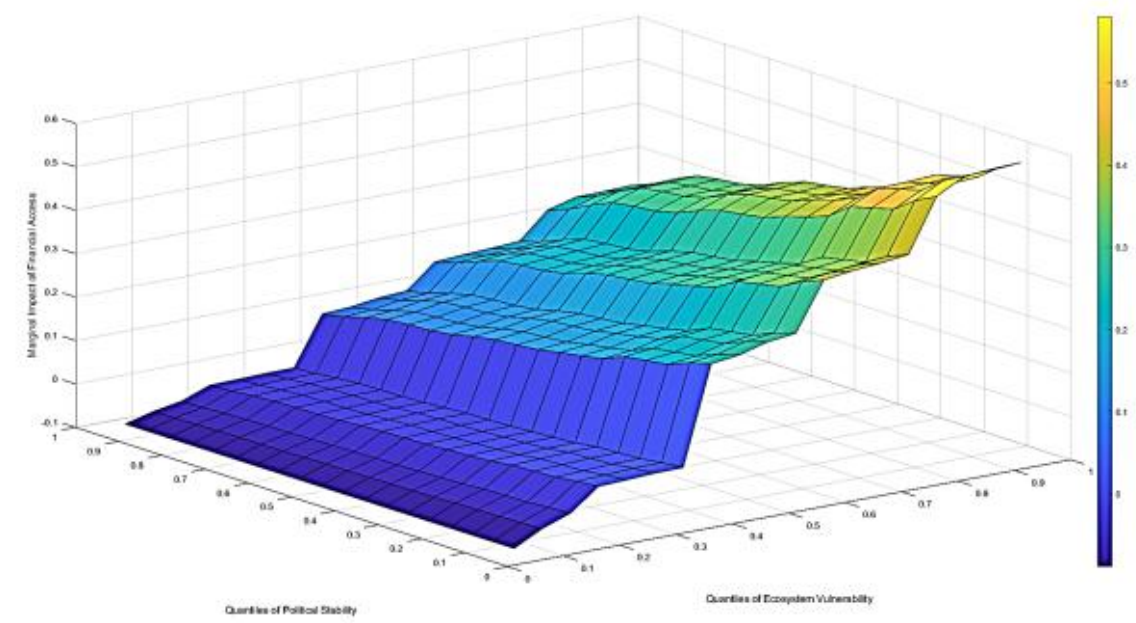

Governance Quality Indicator: Political Stability

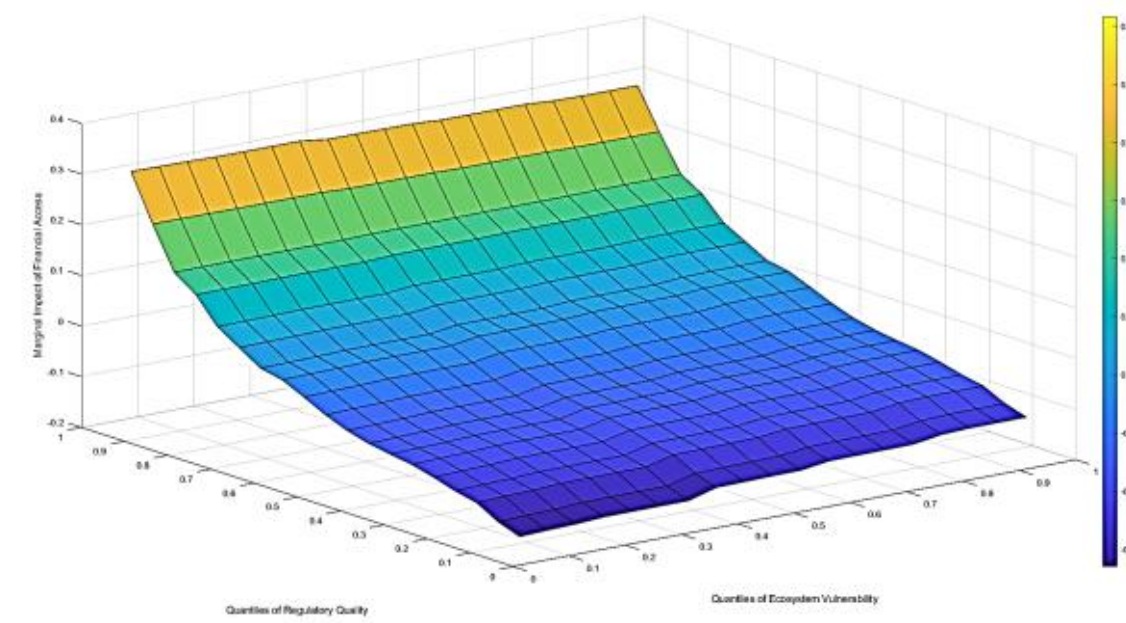

Governance Quality Indicator: Regulatory Quality

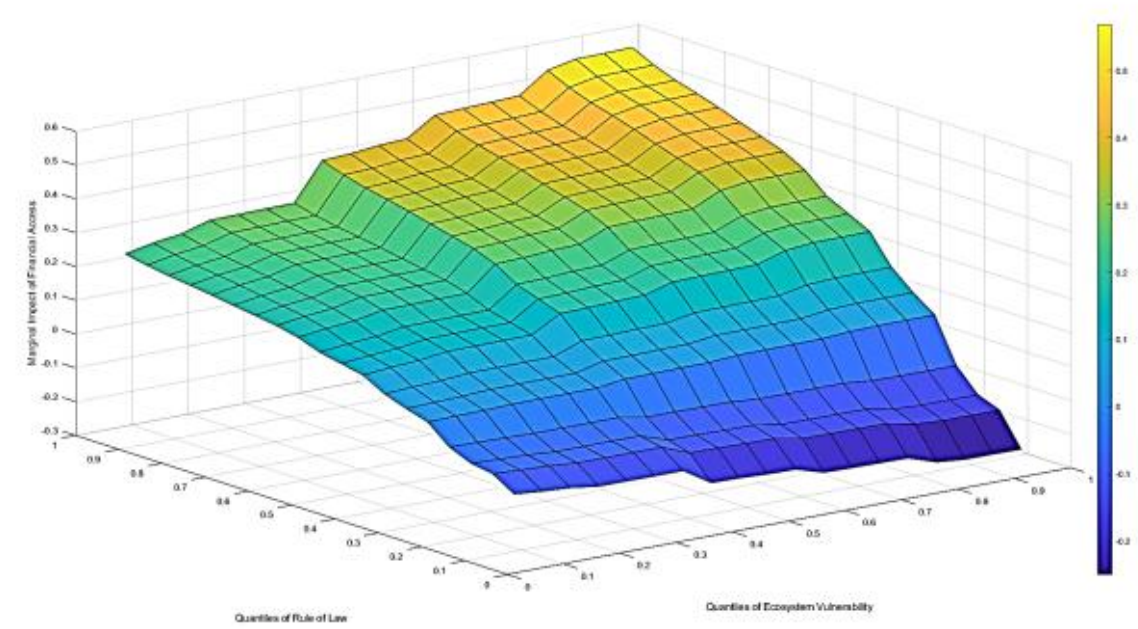

Governance Quality Indicator: Rule of Law

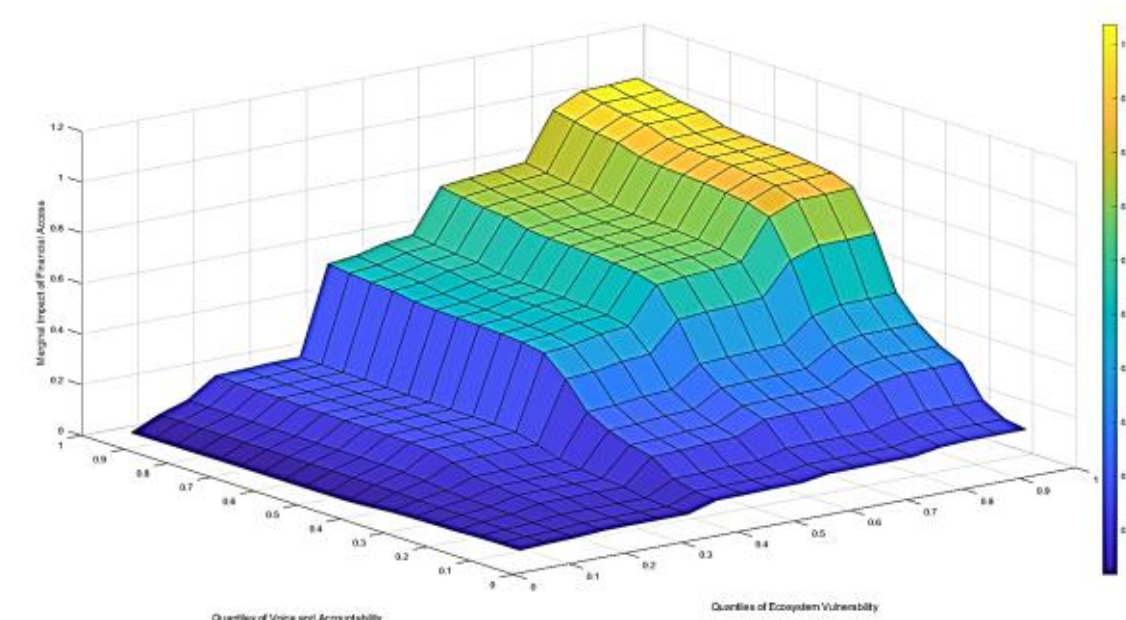

Governance Quality Indicator: Voice and Accountability

Figure S13: BRICS Policy Simulation Outcomes for the Marginal Impact of Financial Access on SDG 7.1.1 under Ecosystem Vulnerability and Governance Quality, Related to Figure 4-5 and Table 2

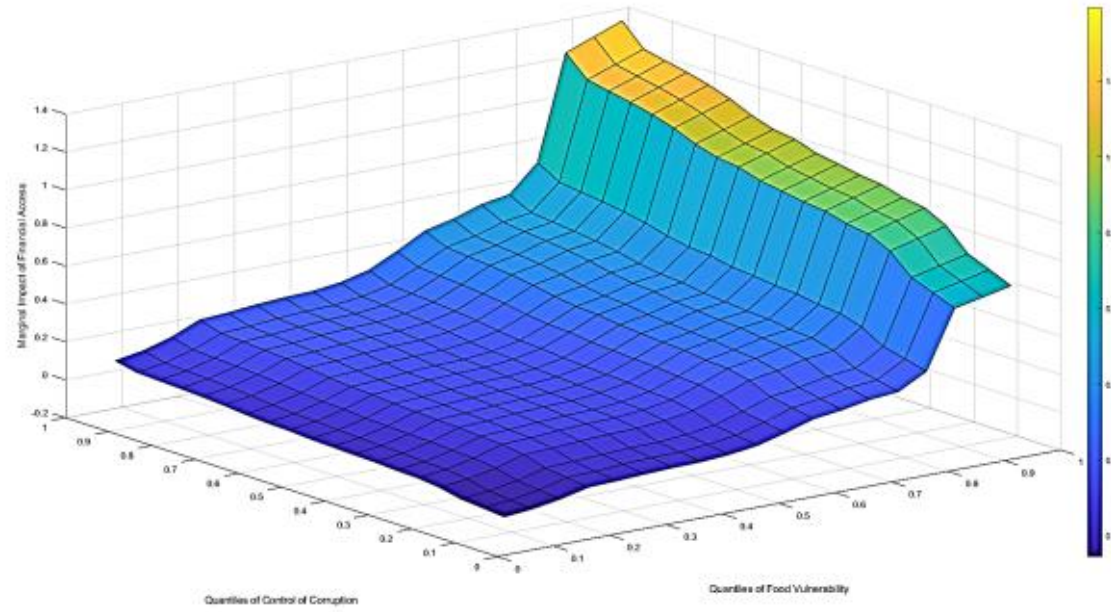

Governance Quality Indicator: Control of Corruption

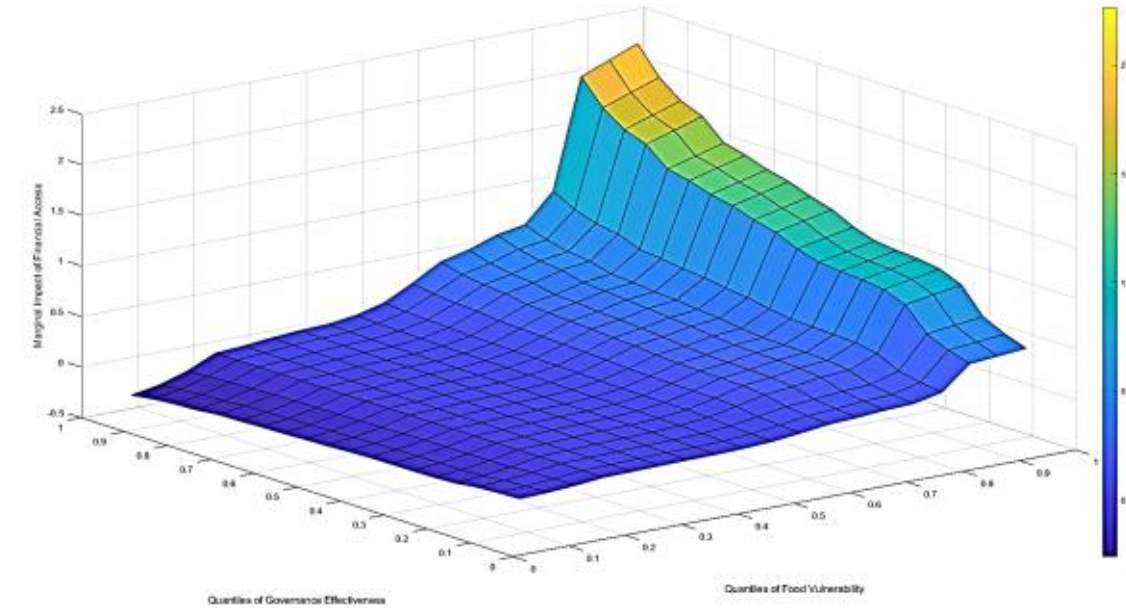

Governance Quality Indicator: Governance Effectiveness

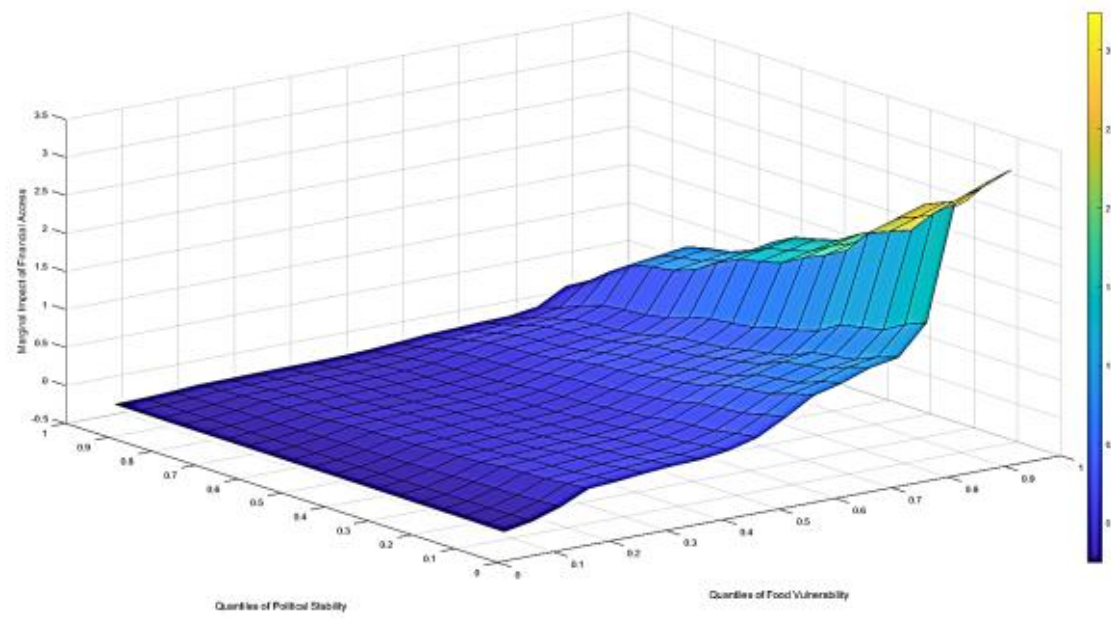

Governance Quality Indicator: Political Stability

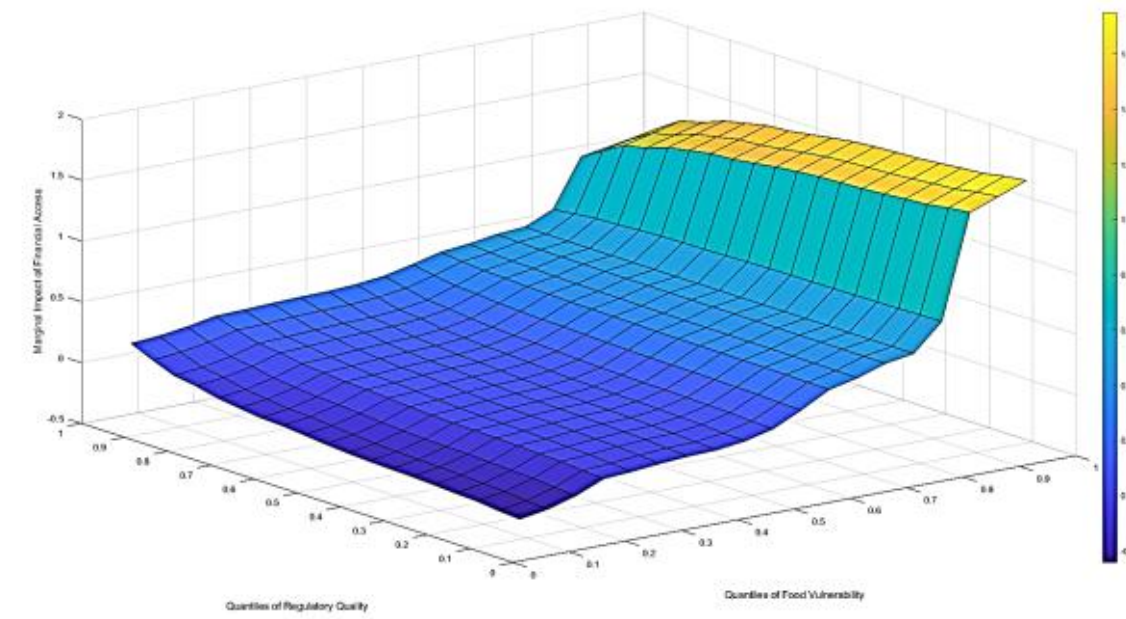

Governance Quality Indicator: Regulatory Quality

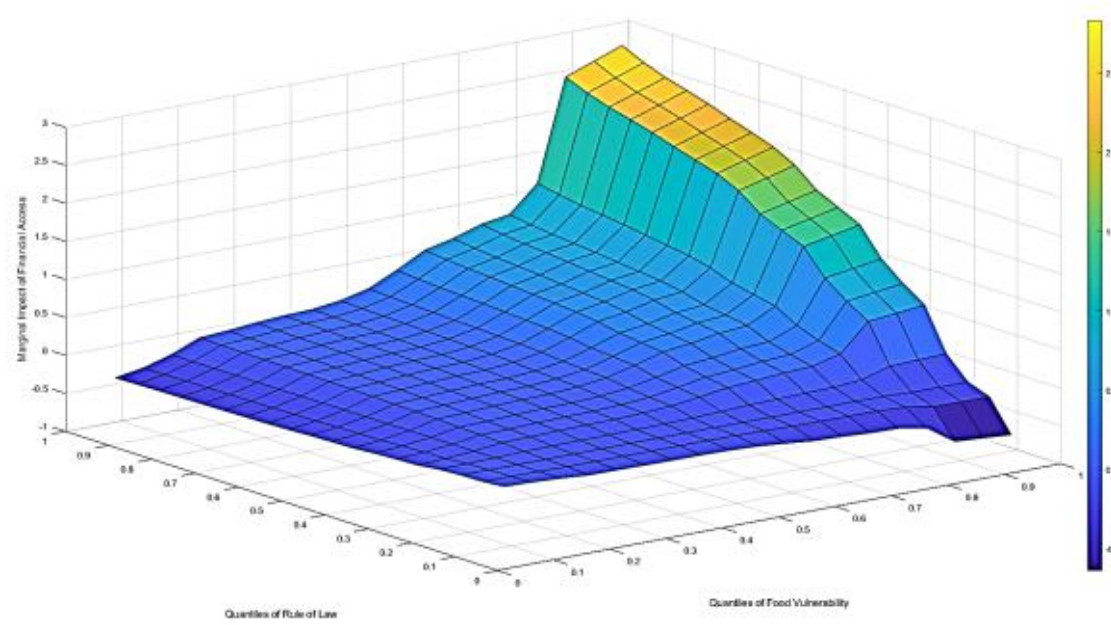

Governance Quality Indicator: Rule of Law

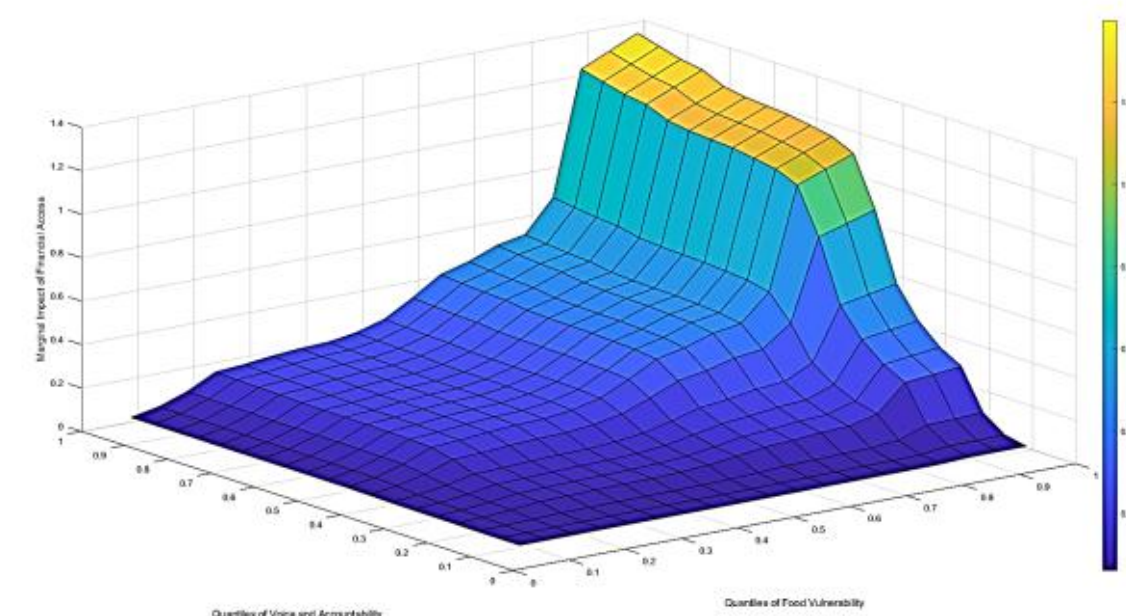

Governance Quality Indicator: Voice and Accountability

Figure S14: BRICS Policy Simulation Outcomes for the Marginal Impact of Financial Access on SDG 7.1.1 under Food Vulnerability and Governance Quality, Related to Figure 4-5 and Table 2

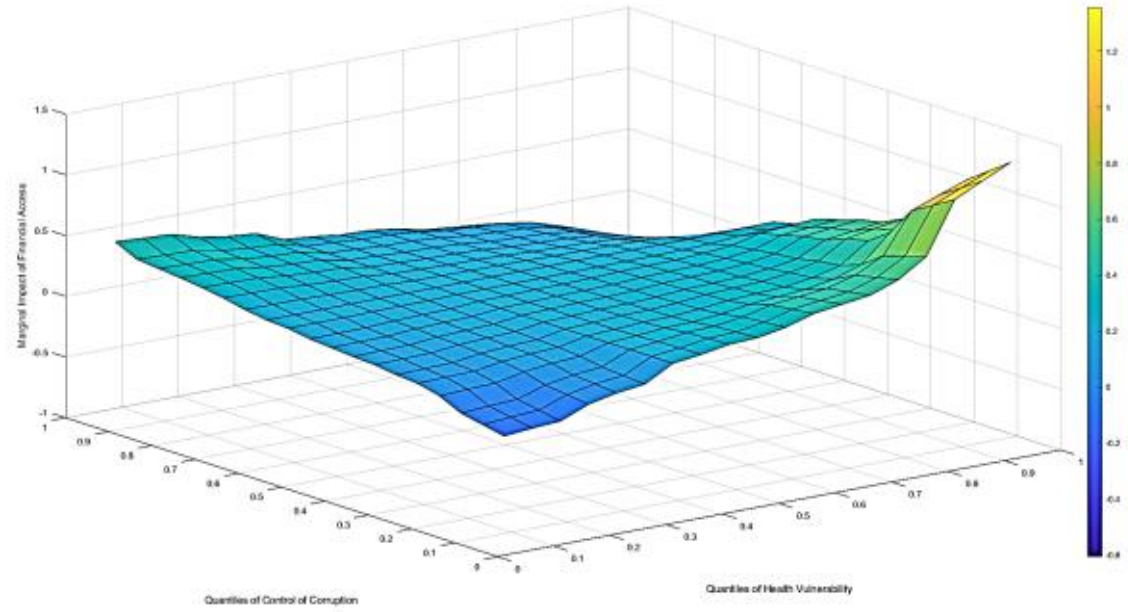

Governance Quality Indicator: Control of Corruption

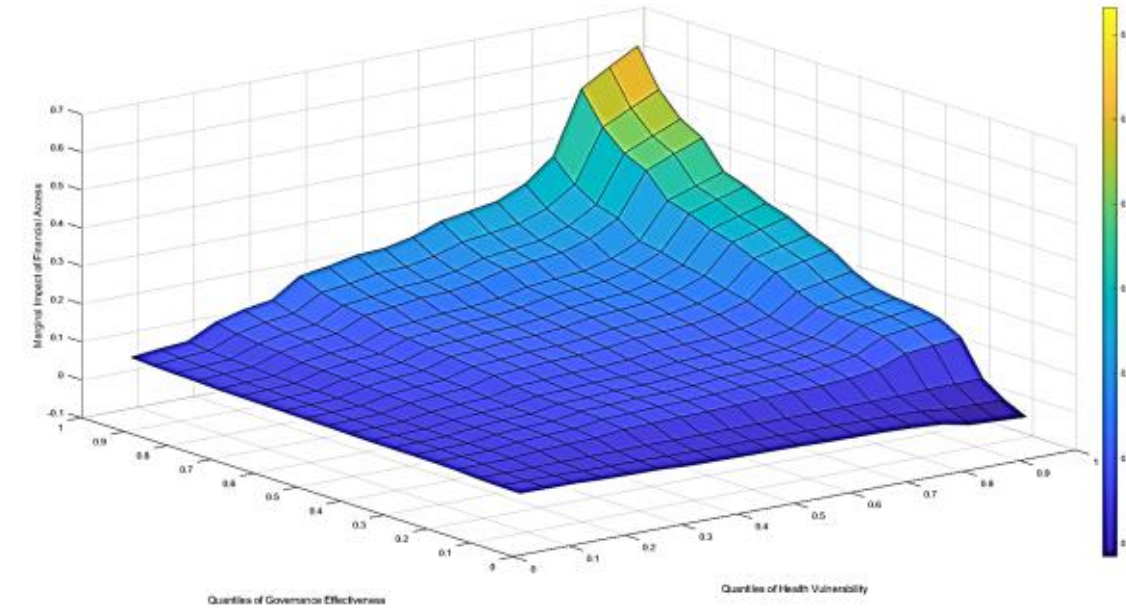

Governance Quality Indicator: Governance Effectiveness

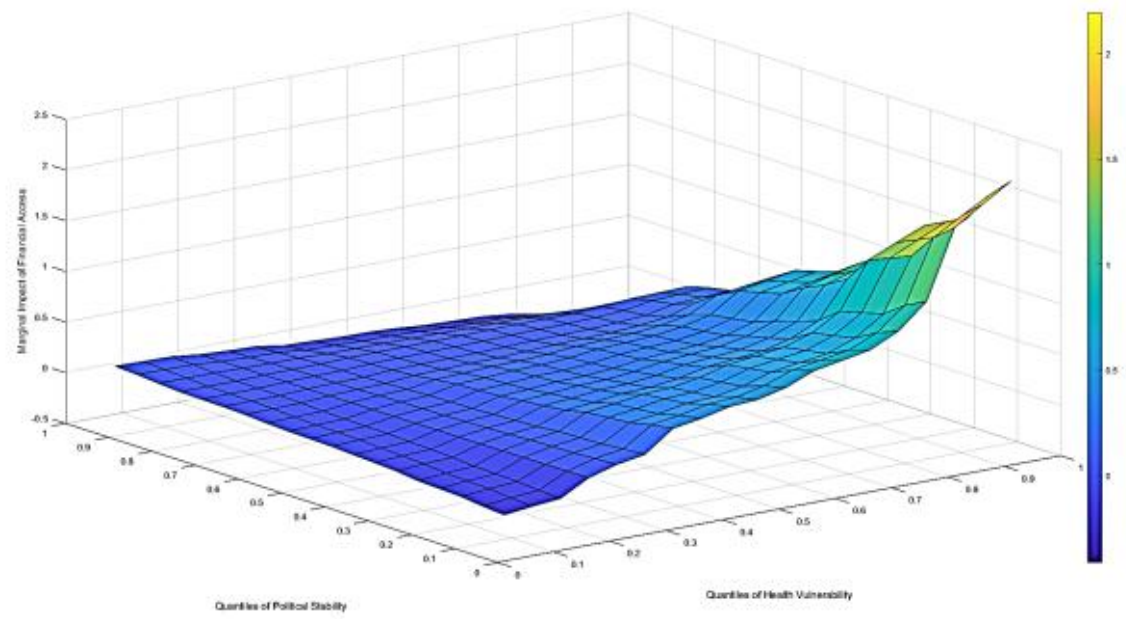

Governance Quality Indicator: Political Stability

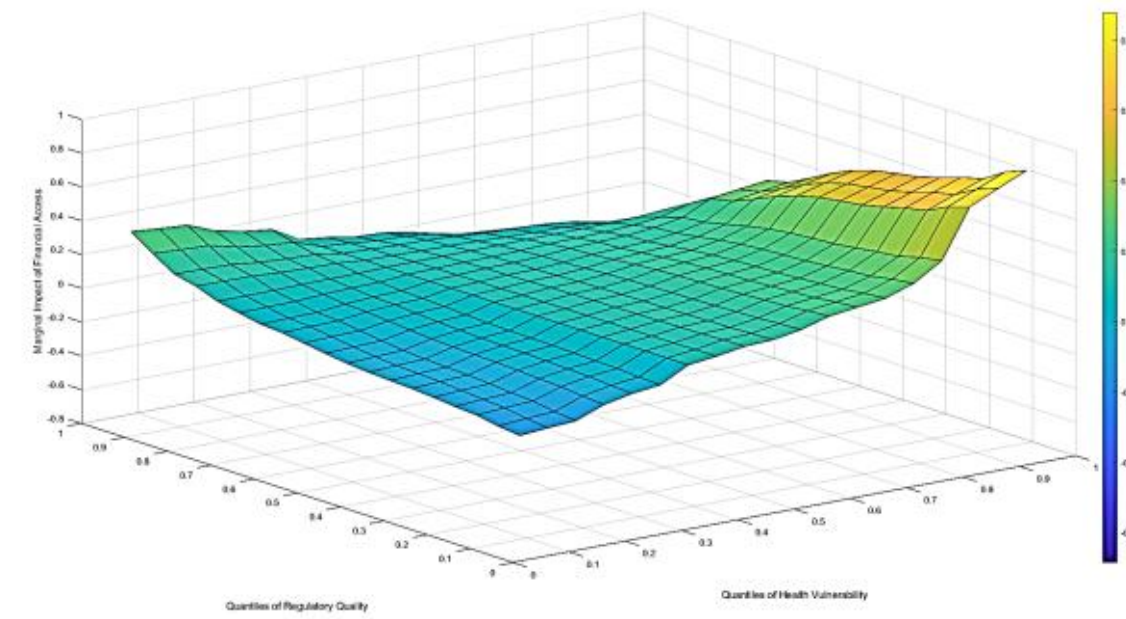

Governance Quality Indicator: Regulatory Quality

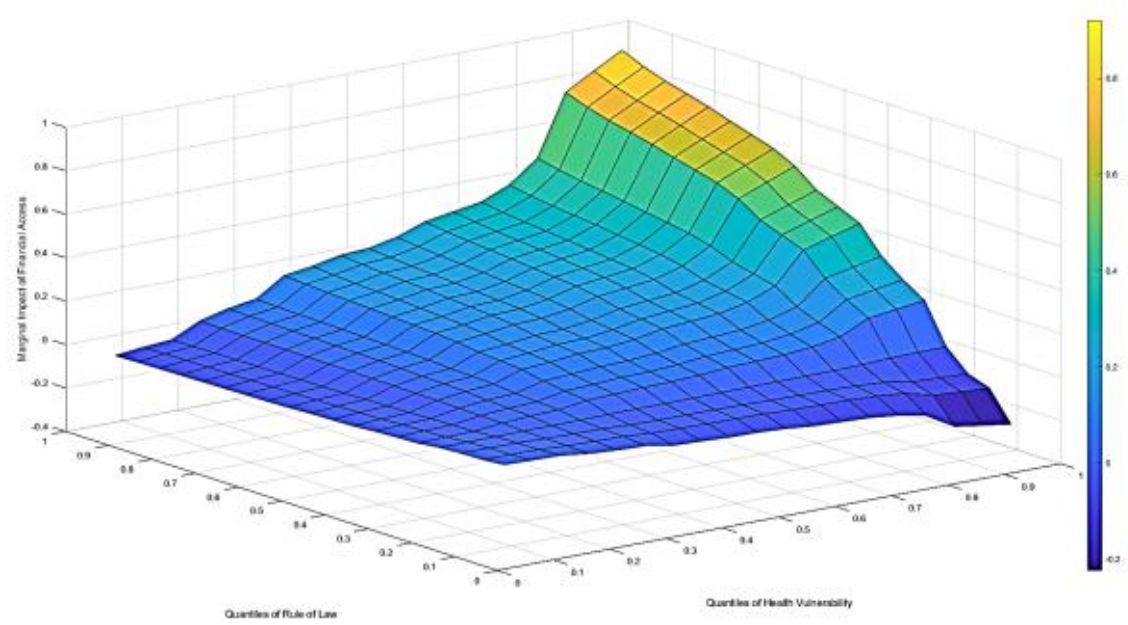

Governance Quality Indicator: Rule of Law

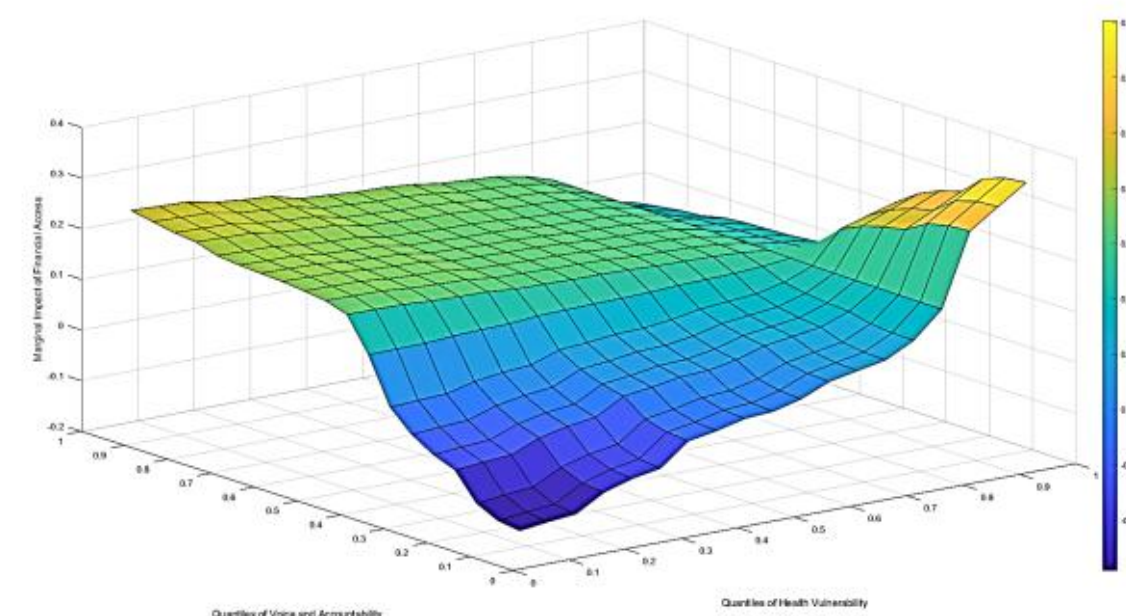

Governance Quality Indicator: Voice and Accountability

Figure S15: BRICS Policy Simulation Outcomes for the Marginal Impact of Financial Access on SDG 7.1.1 under Health Vulnerability and Governance Quality, Related to Figure 4-5 and Table 2

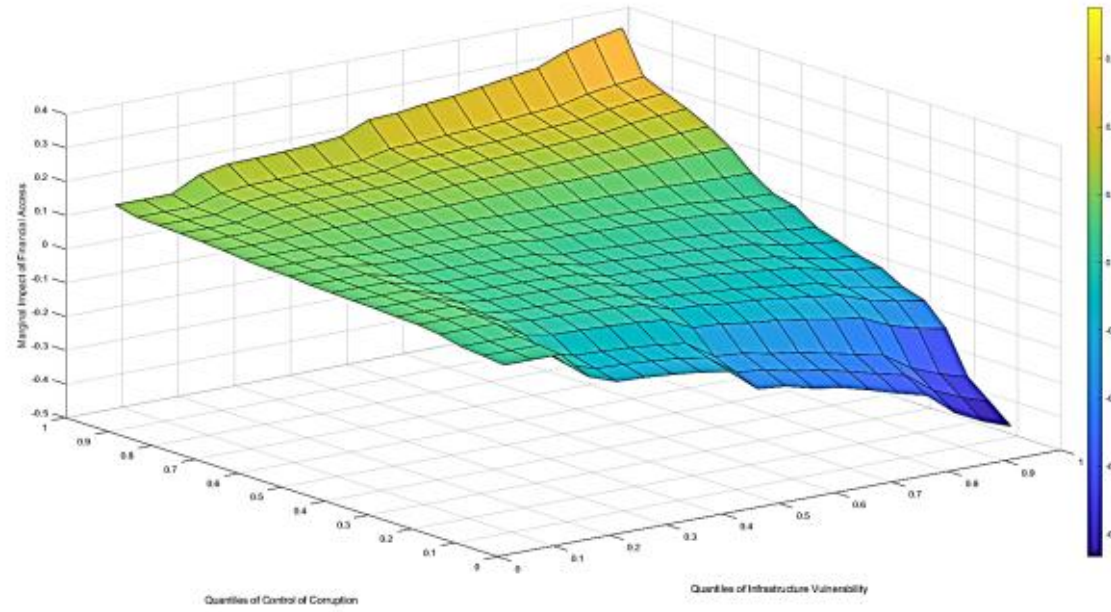

Governance Quality Indicator: Control of Corruption

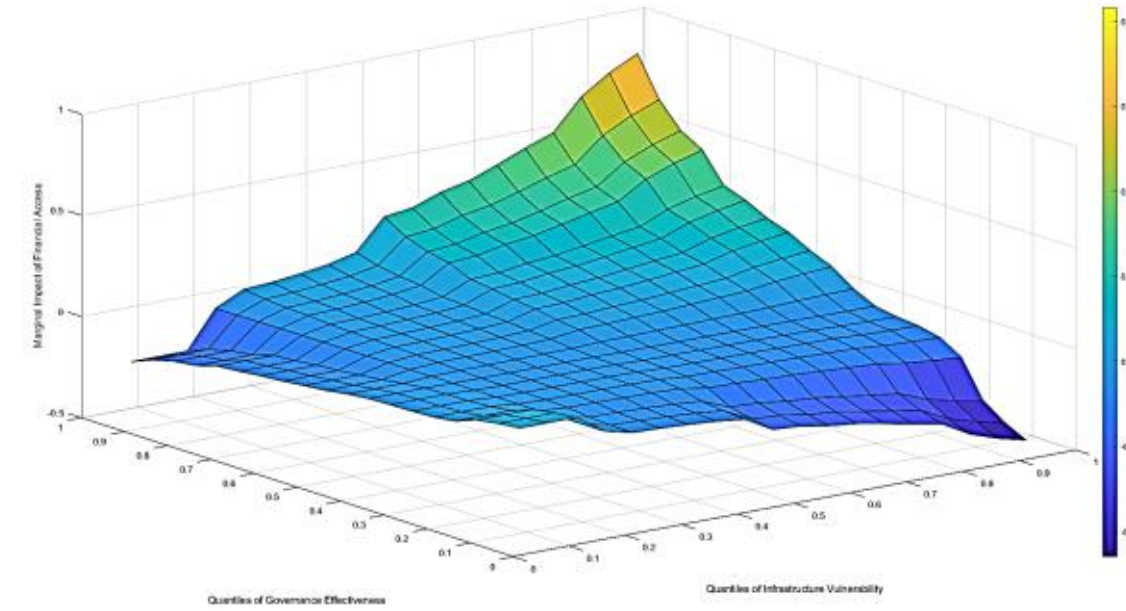

Governance Quality Indicator: Governance Effectiveness

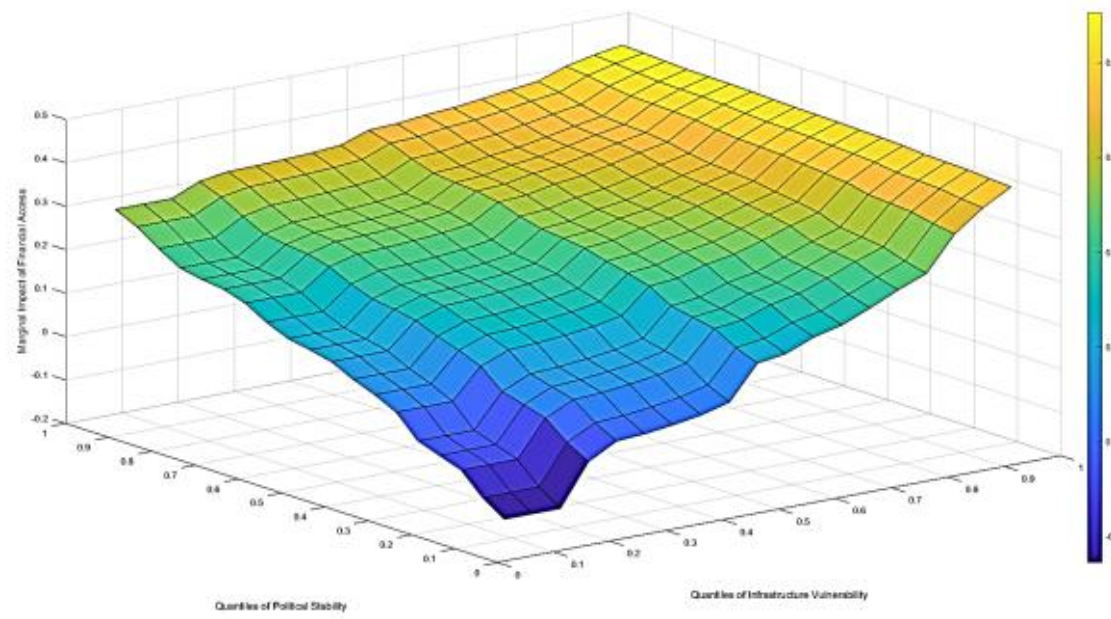

Governance Quality Indicator: Political Stability

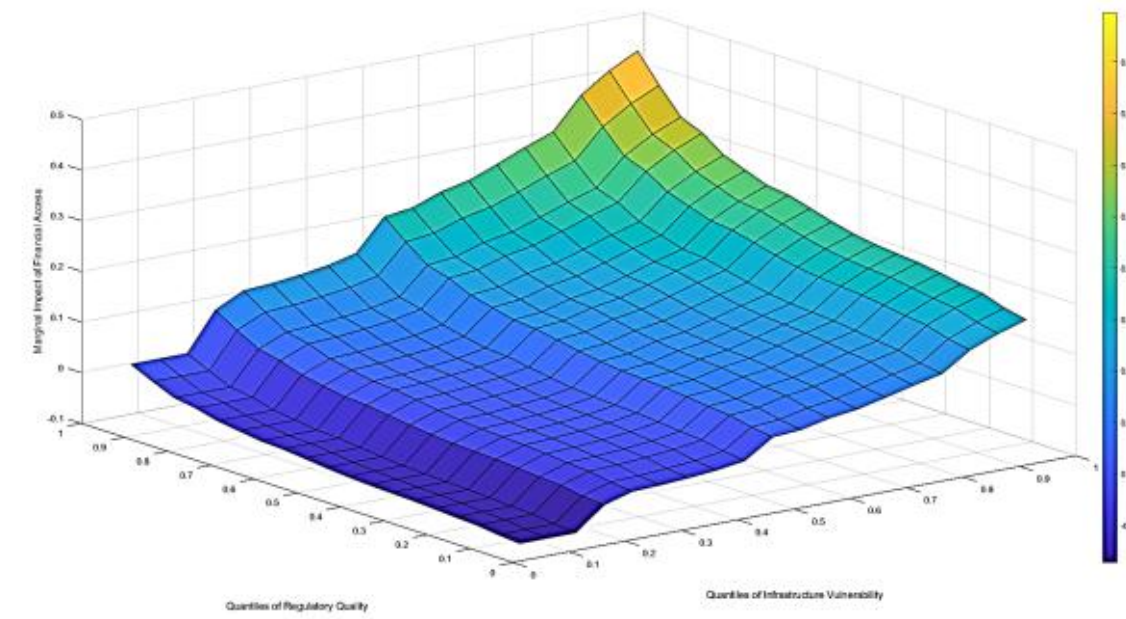

Governance Quality Indicator: Regulatory Quality

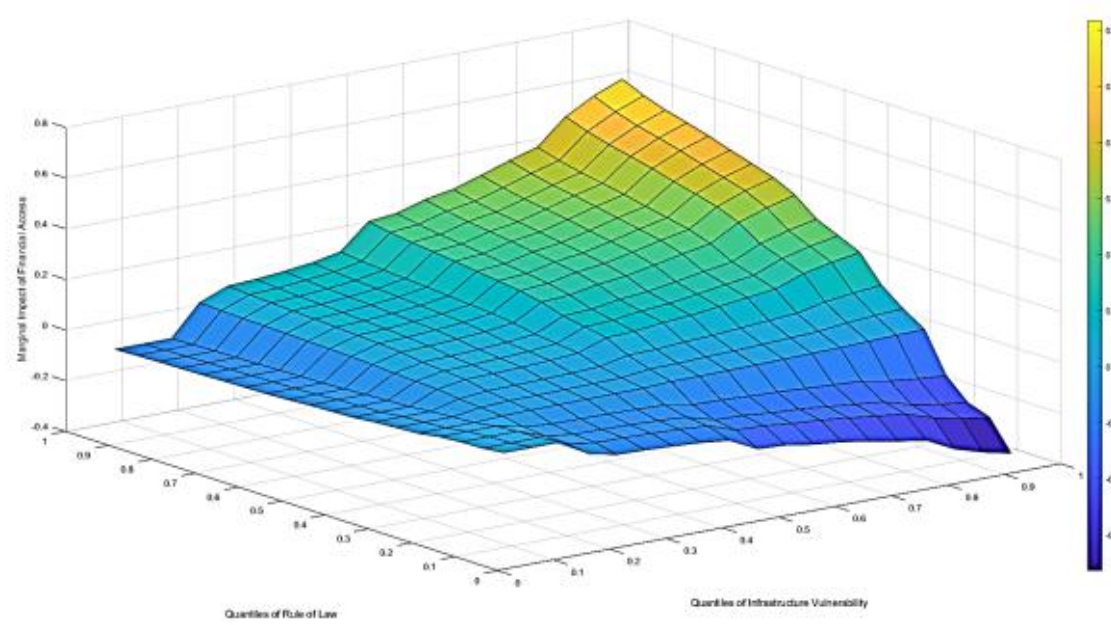

Governance Quality Indicator: Rule of Law

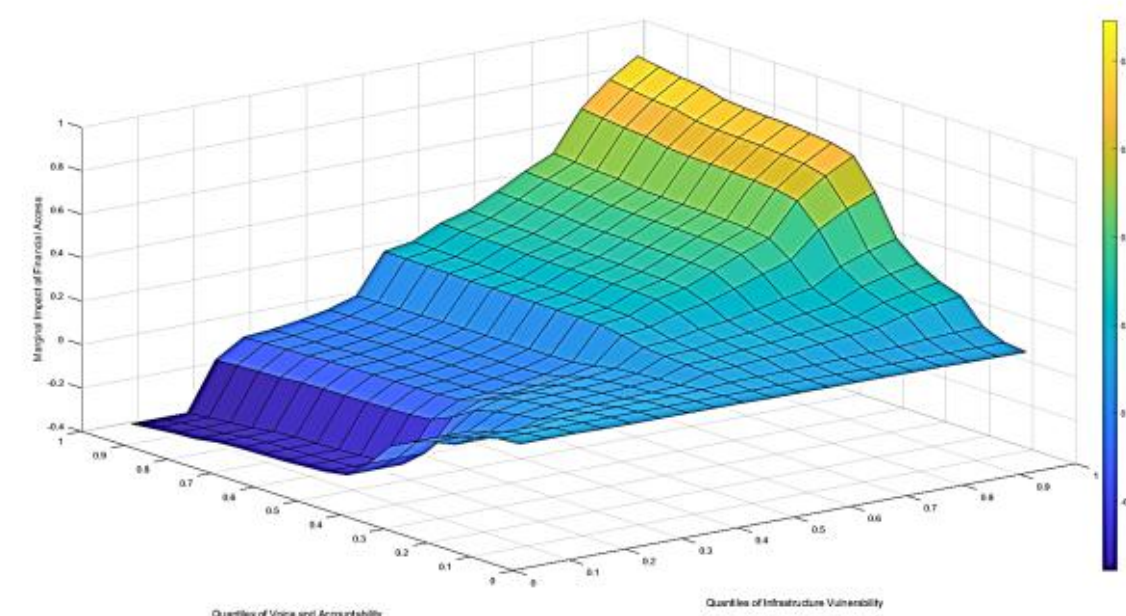

Governance Quality Indicator: Voice and Accountability

Figure S16: BRICS Policy Simulation Outcomes for the Marginal Impact of Financial Access on SDG 7.1.1 under Infrastructure Vulnerability and Governance Quality, Related to Figure 4-5 and Table 2

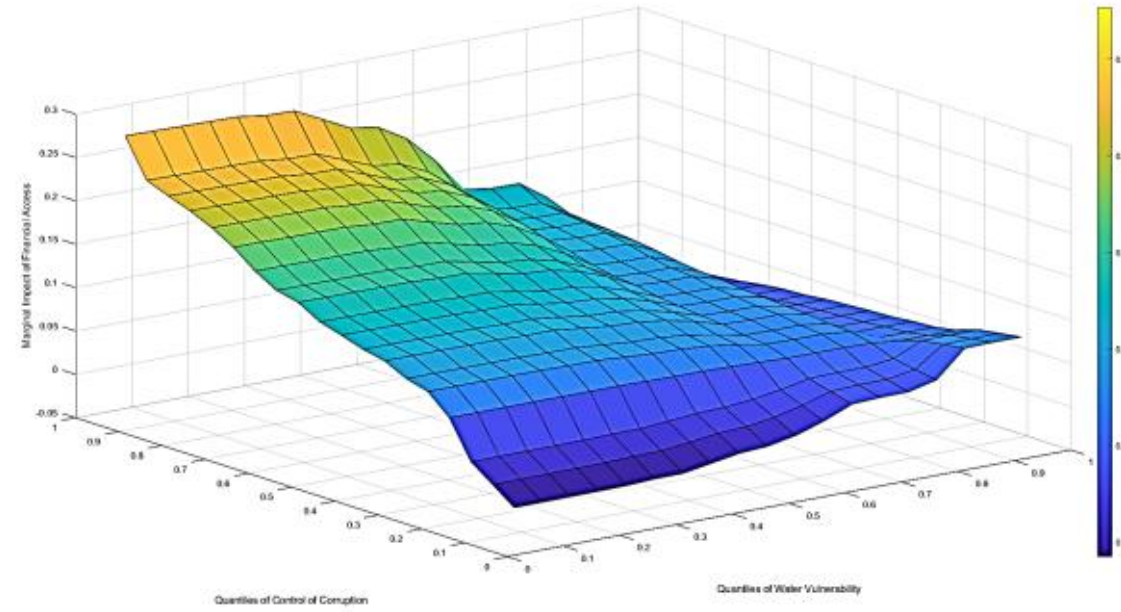

Governance Quality Indicator: Control of Corruption

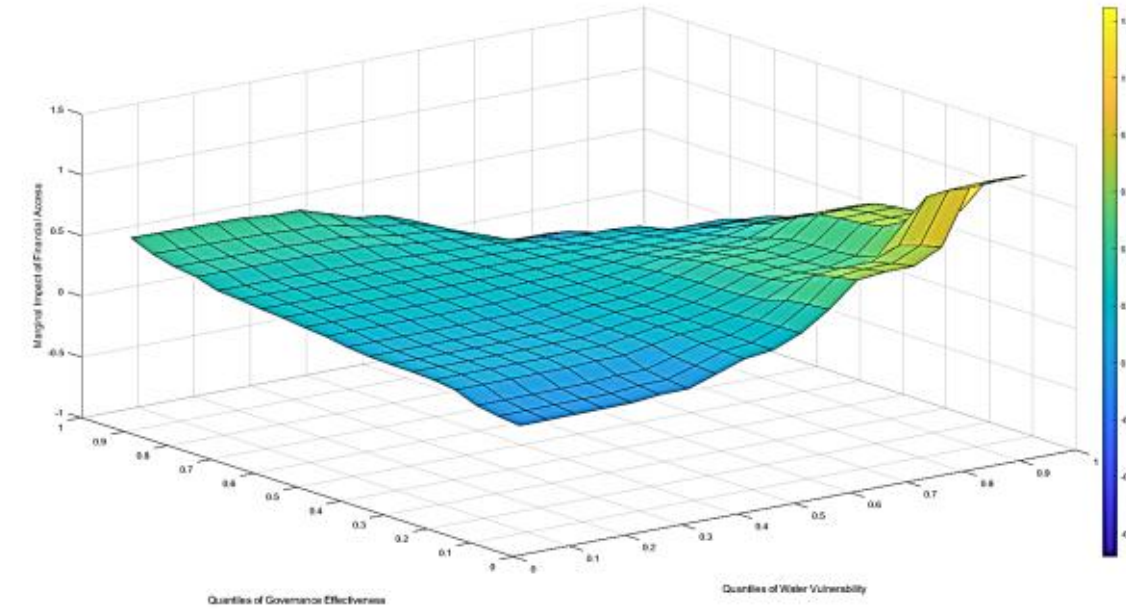

Governance Quality Indicator: Governance Effectiveness

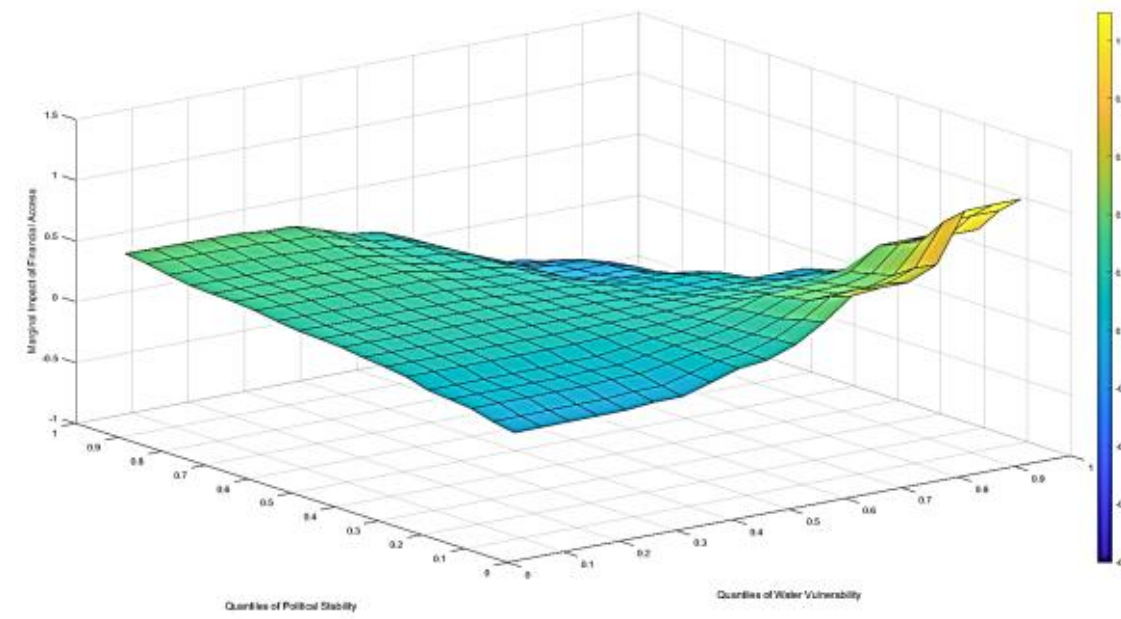

Governance Quality Indicator: Political Stability

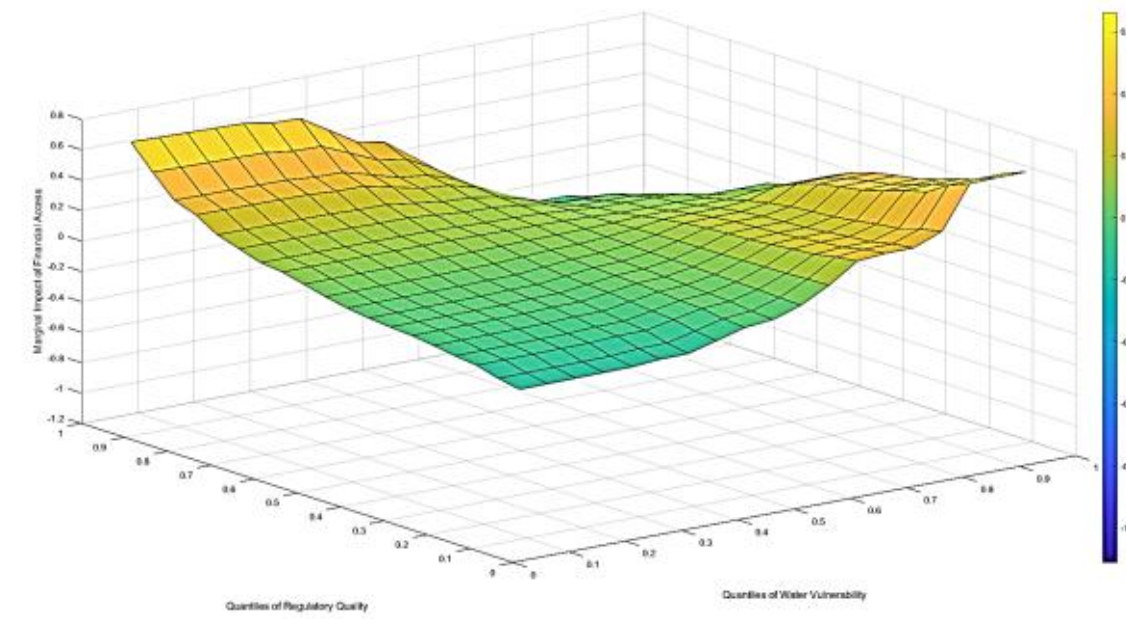

Governance Quality Indicator: Regulatory Quality

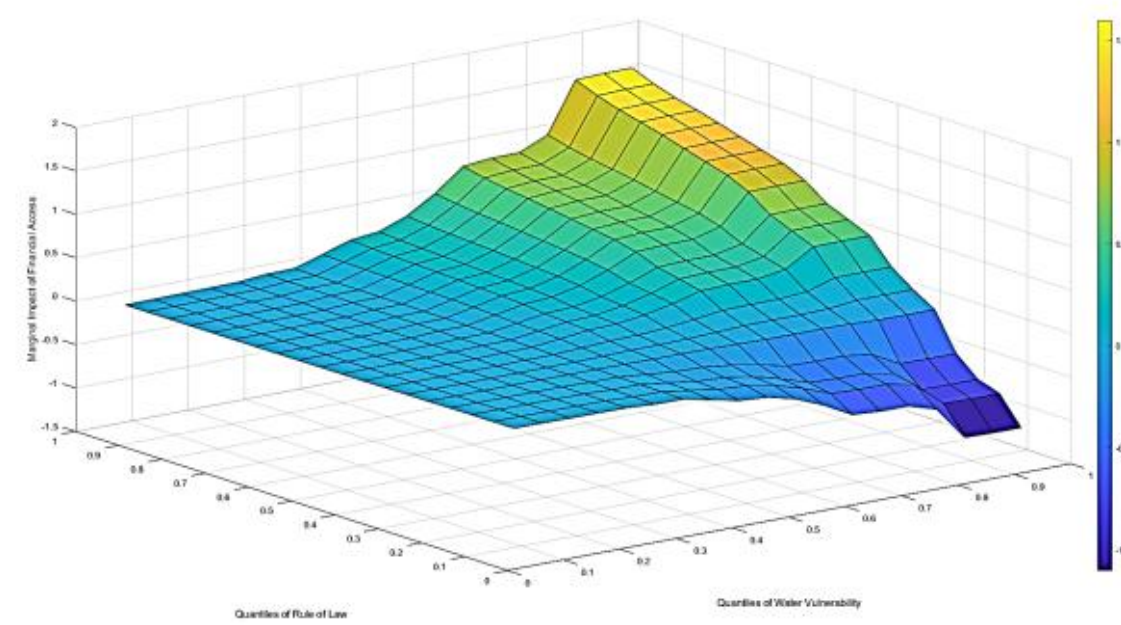

Governance Quality Indicator: Rule of Law

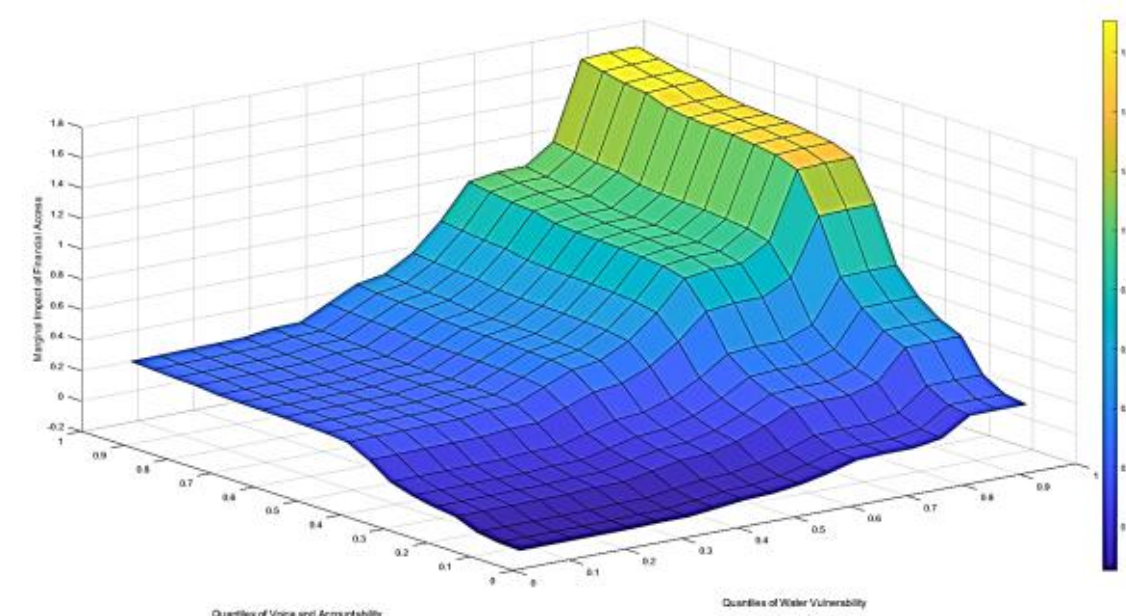

Governance Quality Indicator: Voice and Accountability

Figure S17: BRICS Policy Simulation Outcomes for the Marginal Impact of Financial Access on SDG 7.1.1 under Water Vulnerability and Governance Quality, Related to Figure 4-5 and Table 2

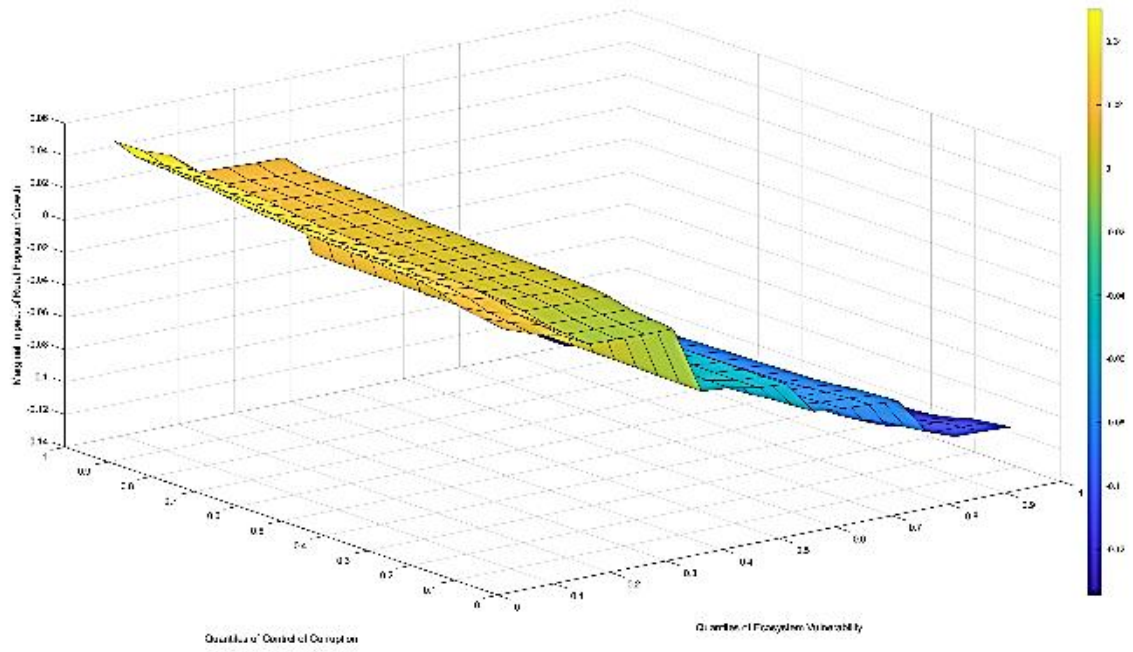

Governance Quality Indicator: Control of Corruption

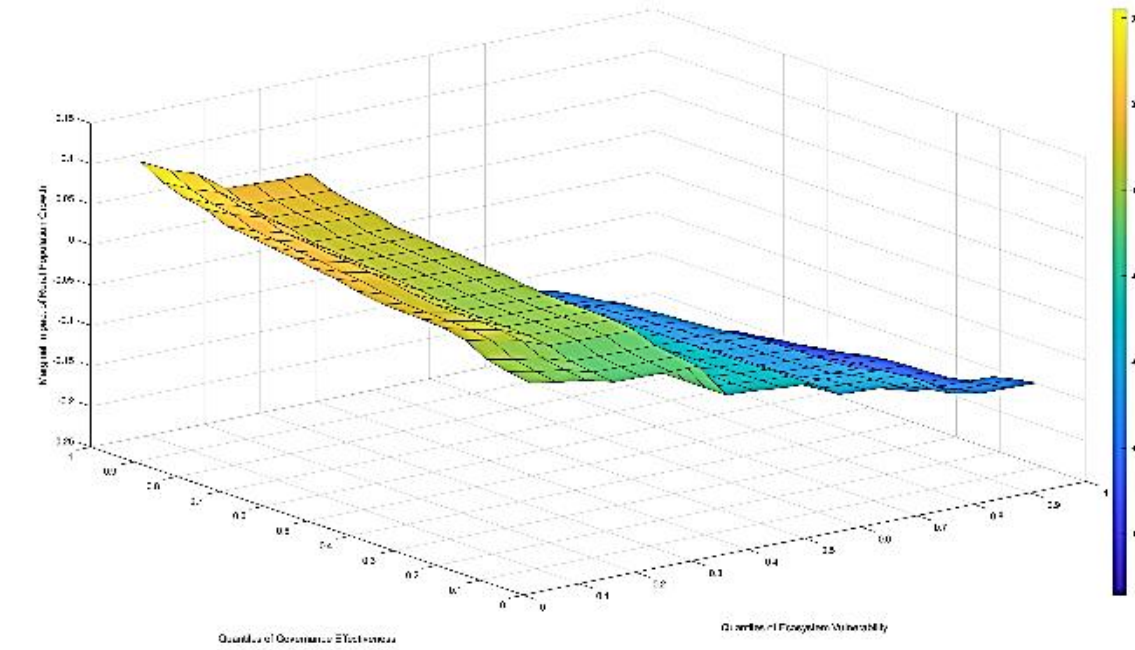

Governance Quality Indicator: Governance Effectiveness

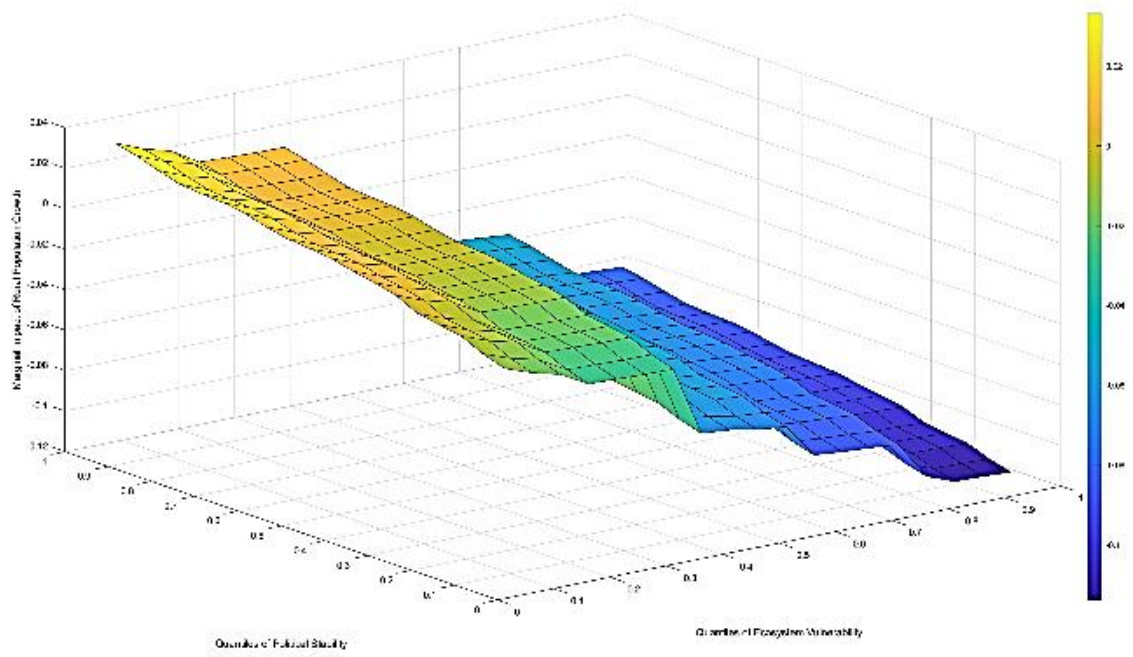

Governance Quality Indicator: Political Stability

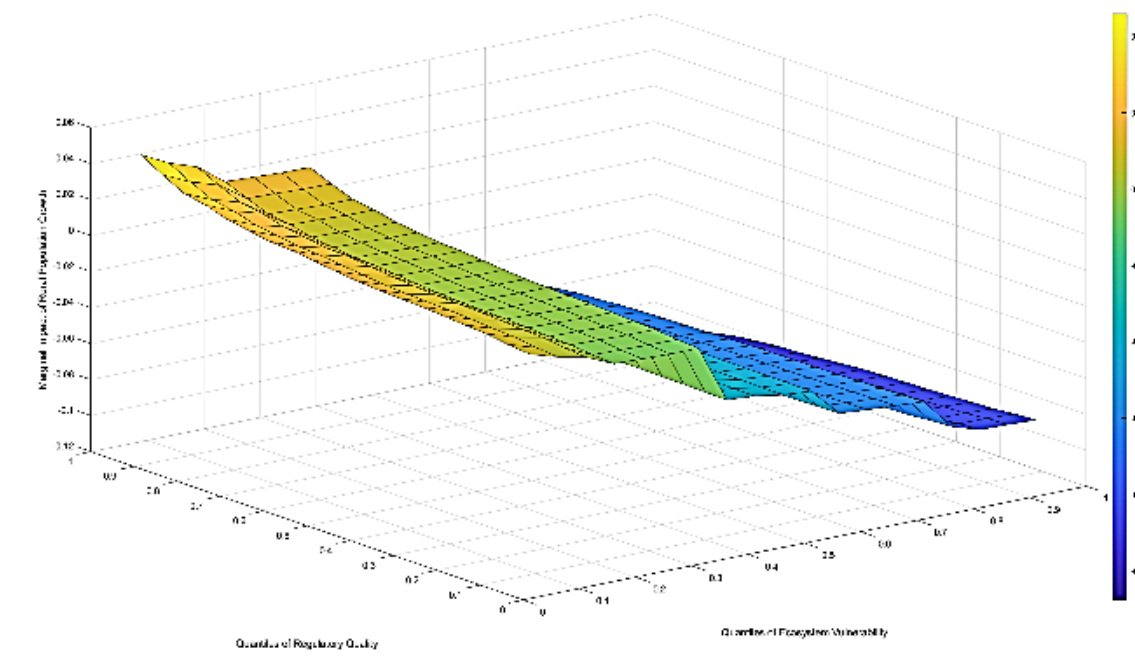

Governance Quality Indicator: Regulatory Quality

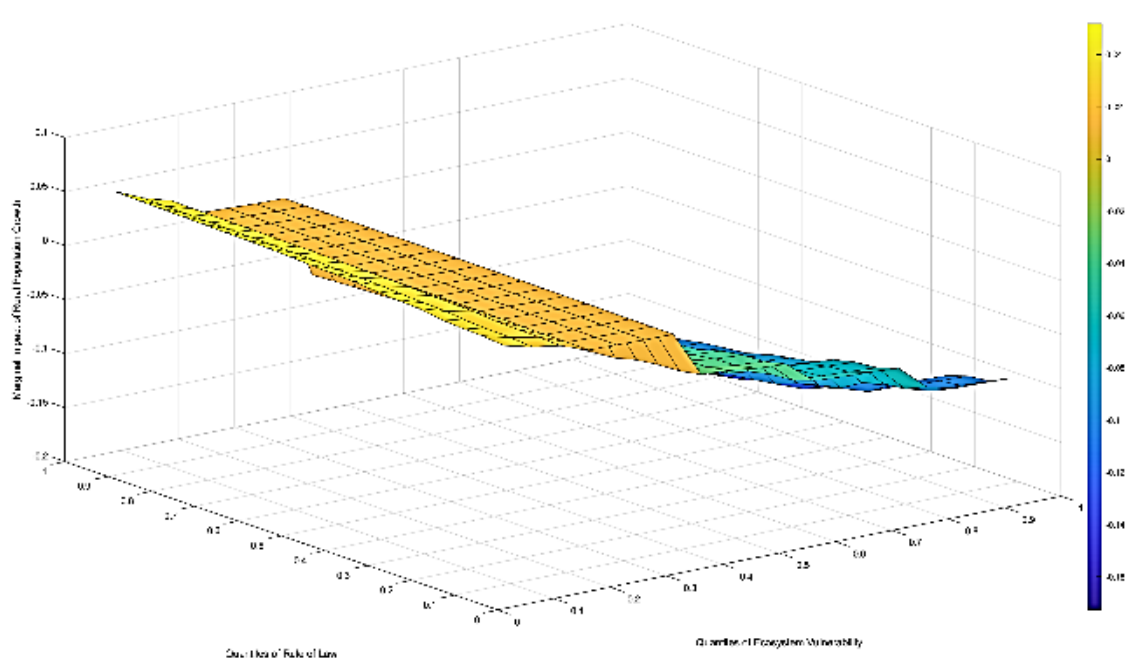

Governance Quality Indicator: Rule of Law

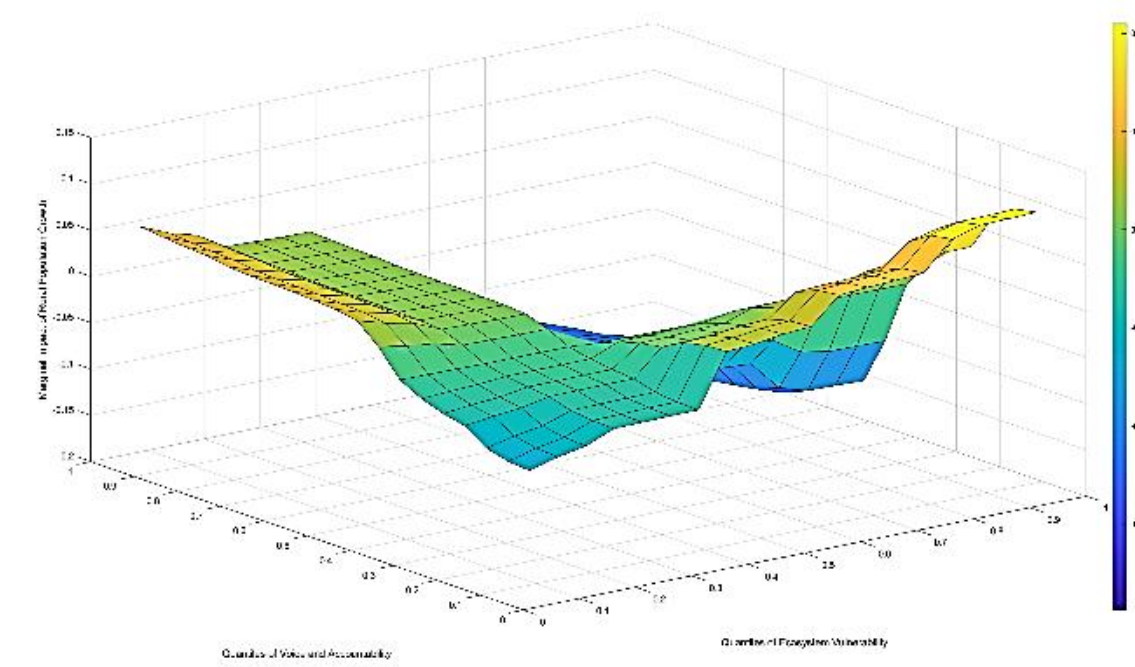

Governance Quality Indicator: Voice and Accountability

Figure S18: BRICS Policy Simulation Outcomes for the Marginal Impact of Rural Population Growth on SDG 7.1.1 under Ecosystem Vulnerability and Governance Quality, Related to Figure 4-5 and Table 2

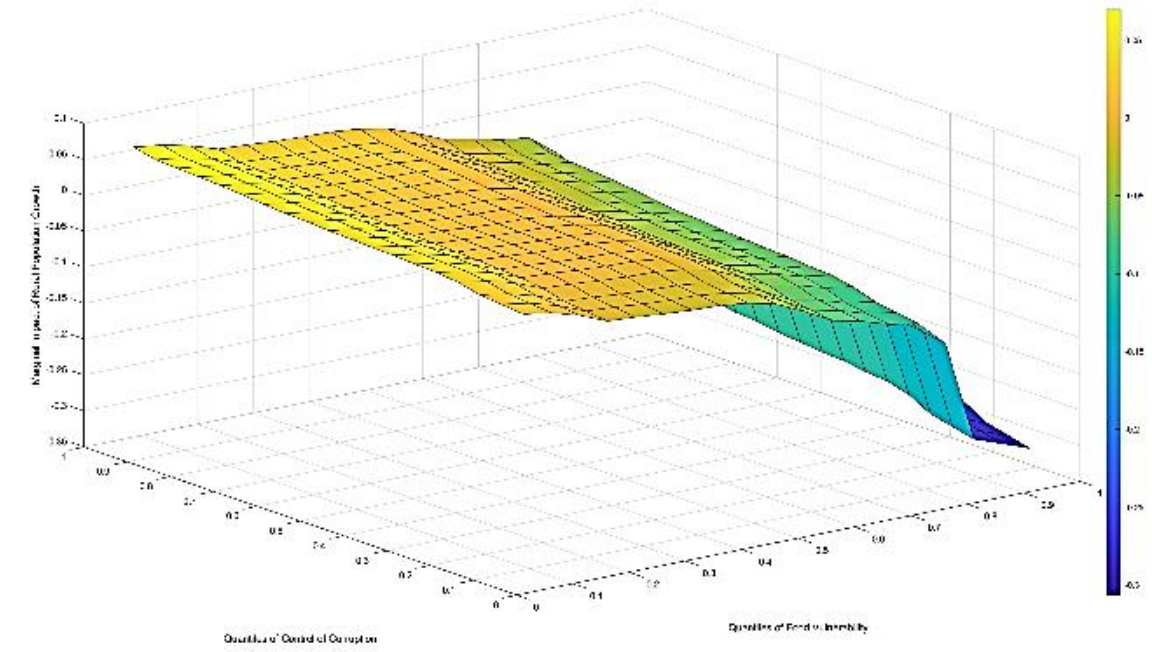

Governance Quality Indicator: Control of Corruption

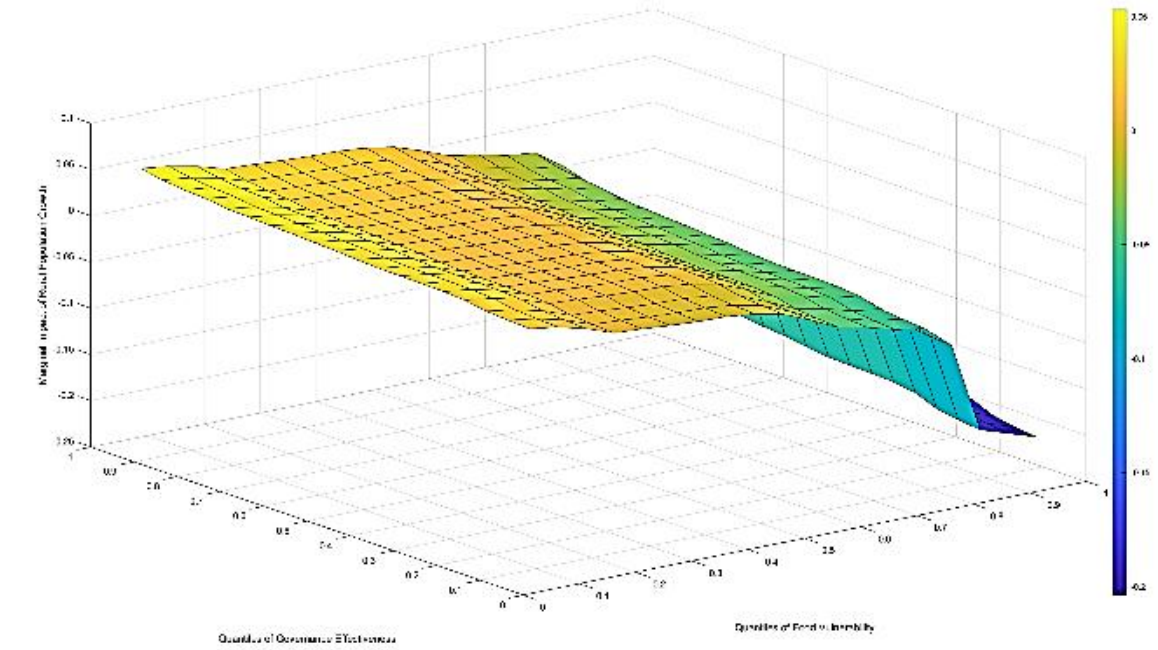

Governance Quality Indicator: Governance Effectiveness

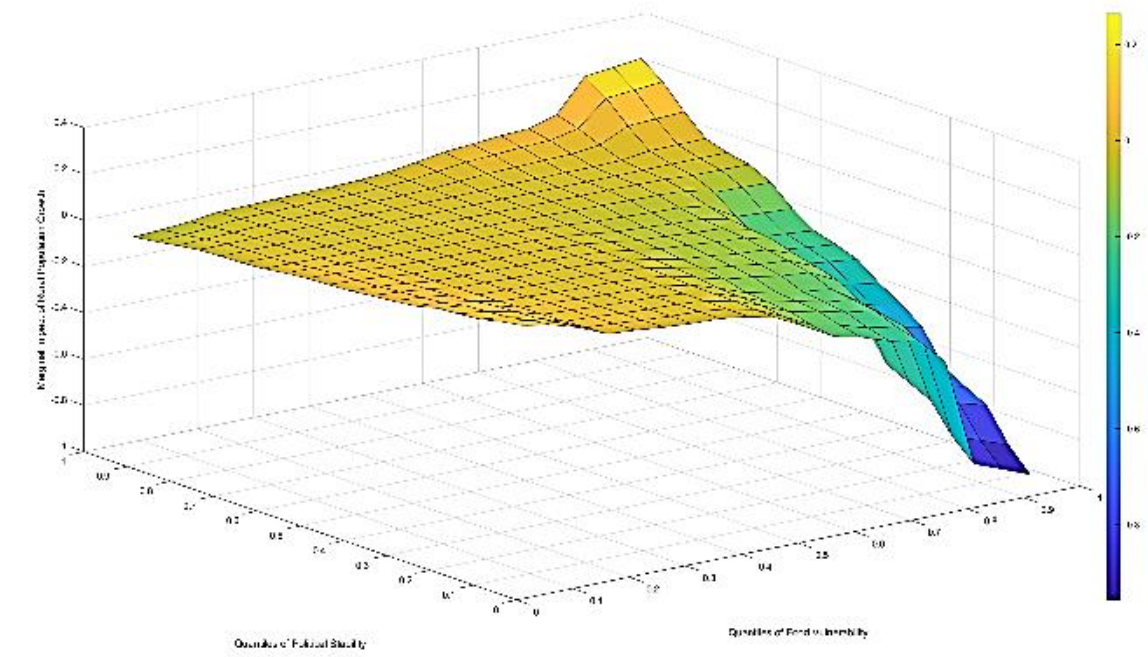

Governance Quality Indicator: Political Stability

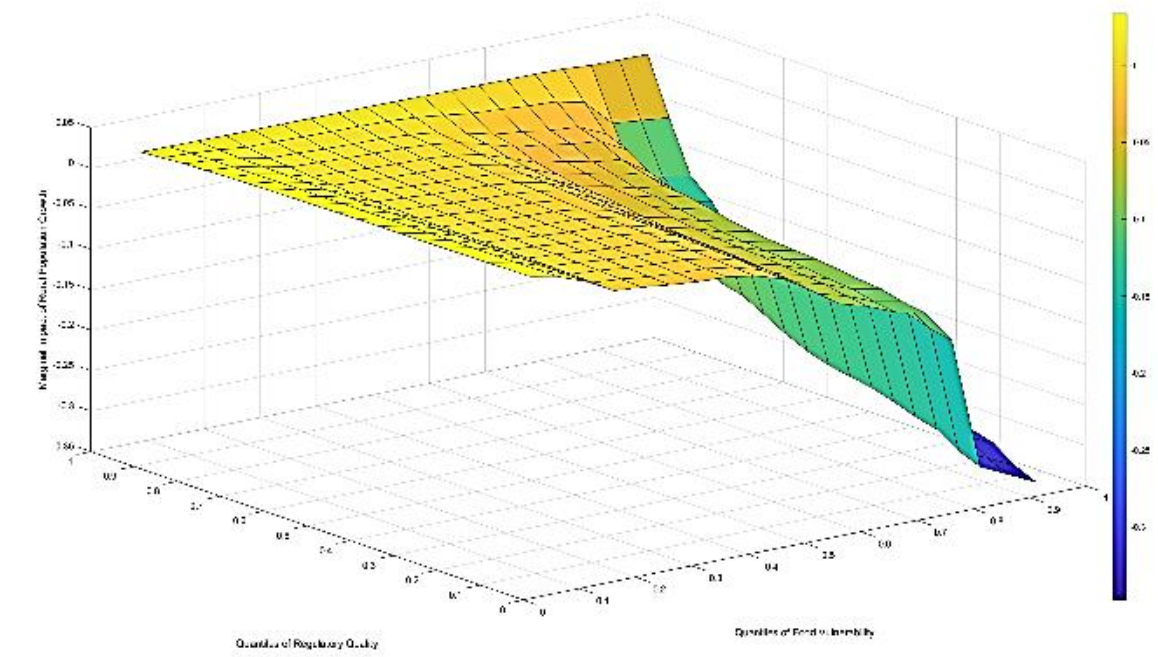

Governance Quality Indicator: Regulatory Quality

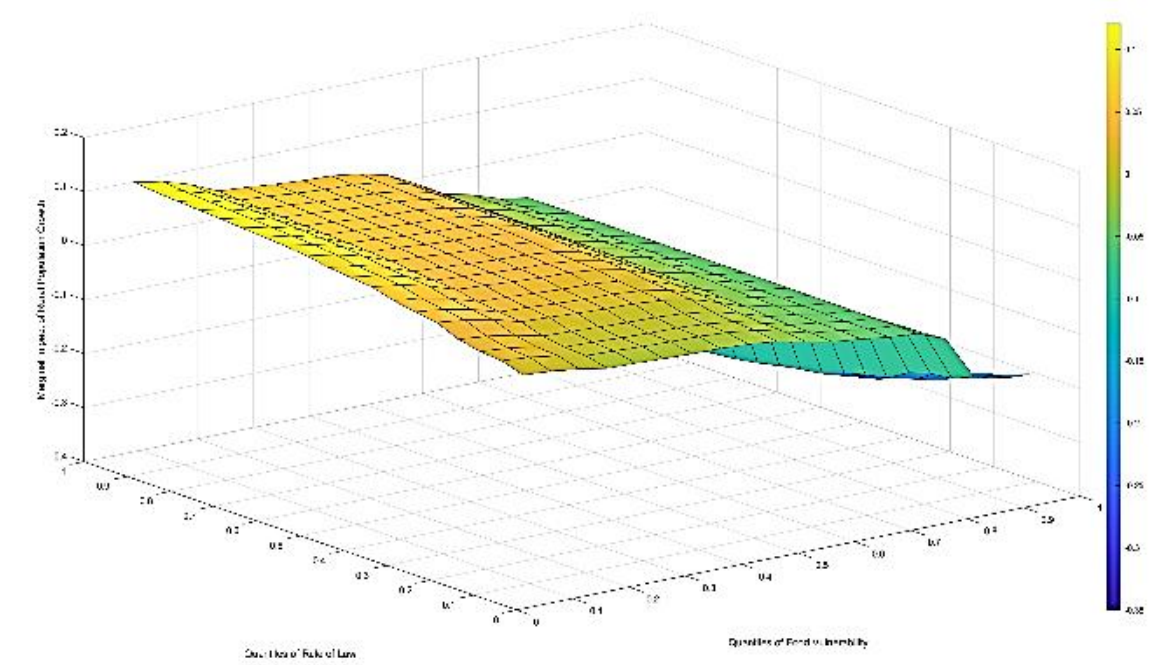

Governance Quality Indicator: Rule of Law

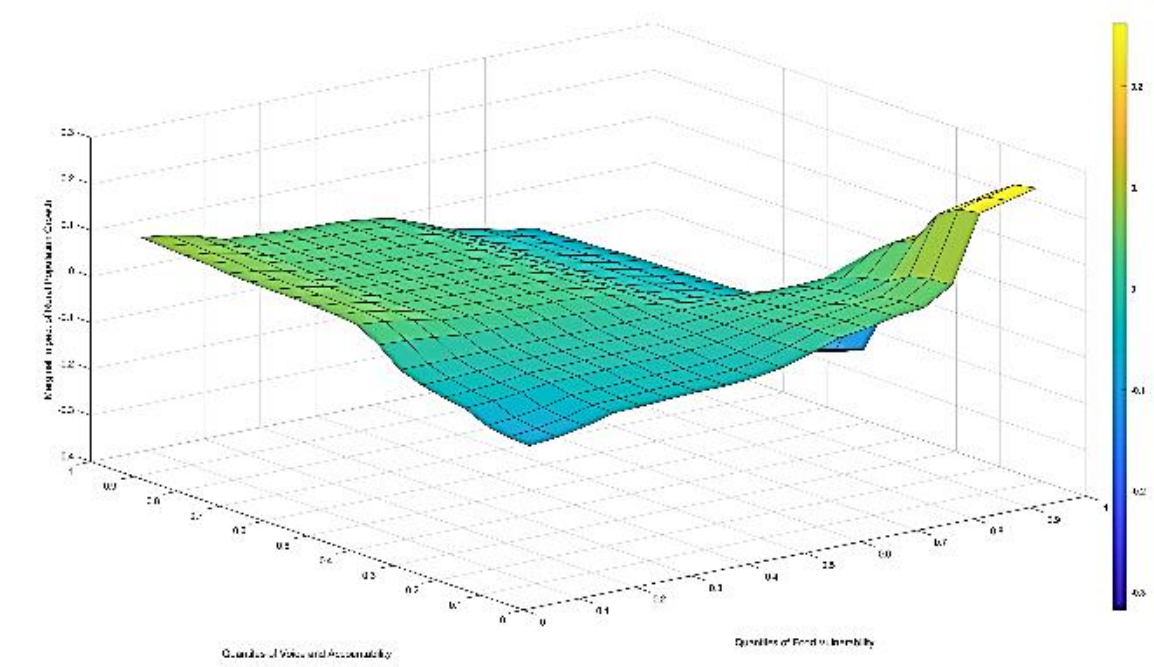

Governance Quality Indicator: Voice and Accountability

Figure S19: BRICS Policy Simulation Outcomes for the Marginal Impact of Rural Population Growth on SDG 7.1.1 under Food Vulnerability and Governance Quality, Related to Figure 4-5 and Table 2

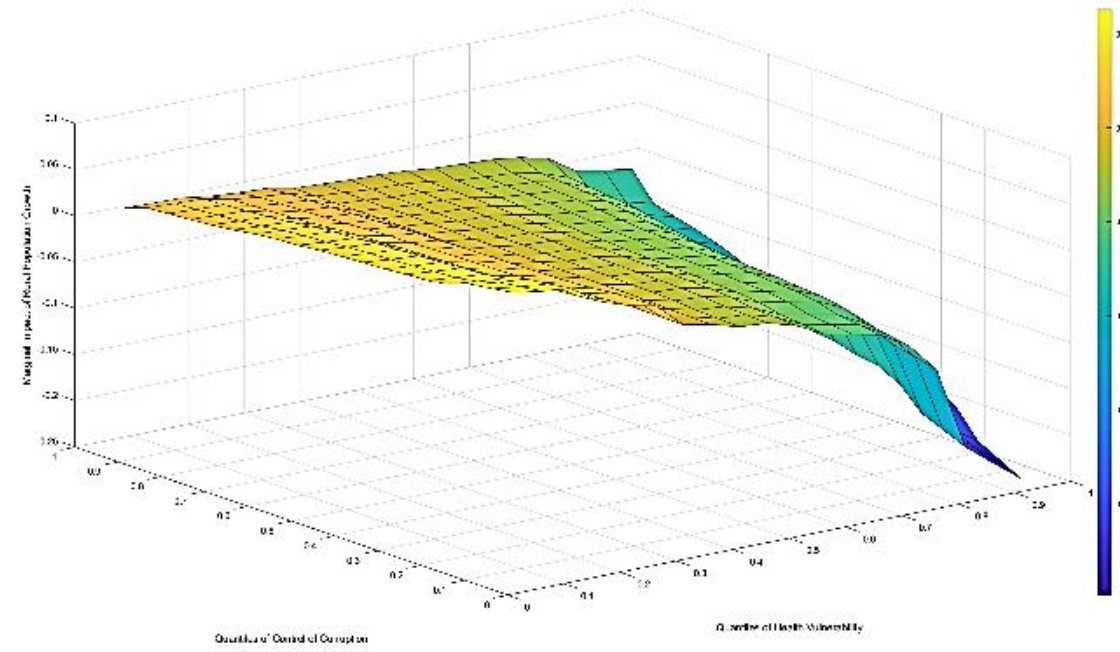

Governance Quality Indicator: Control of Corruption

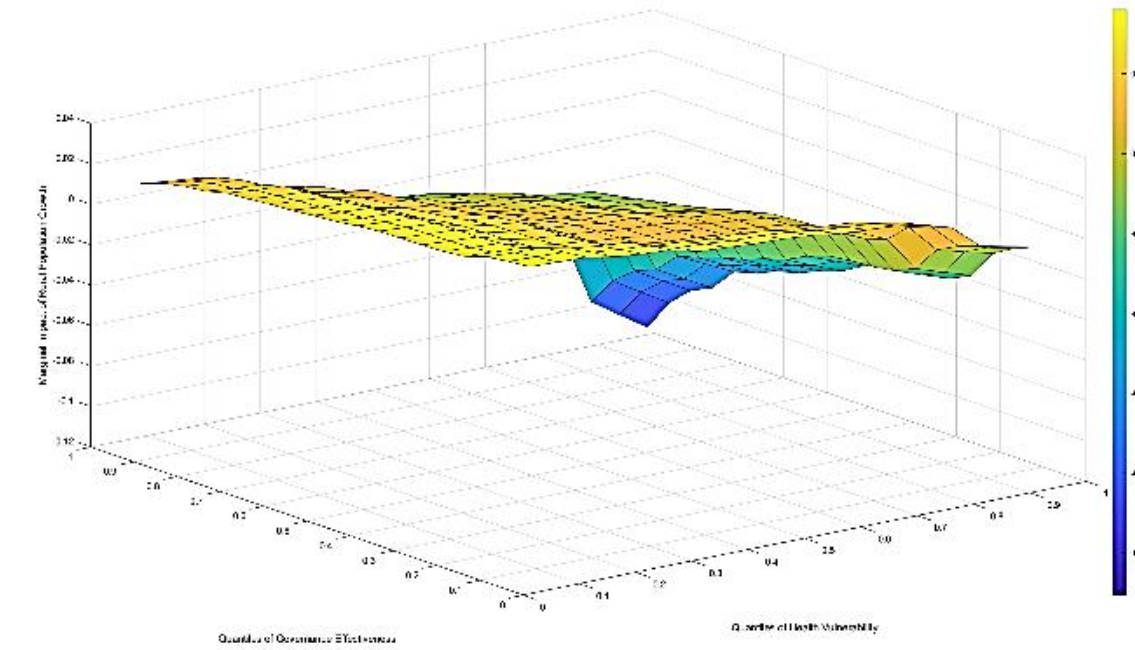

Governance Quality Indicator: Governance Effectiveness

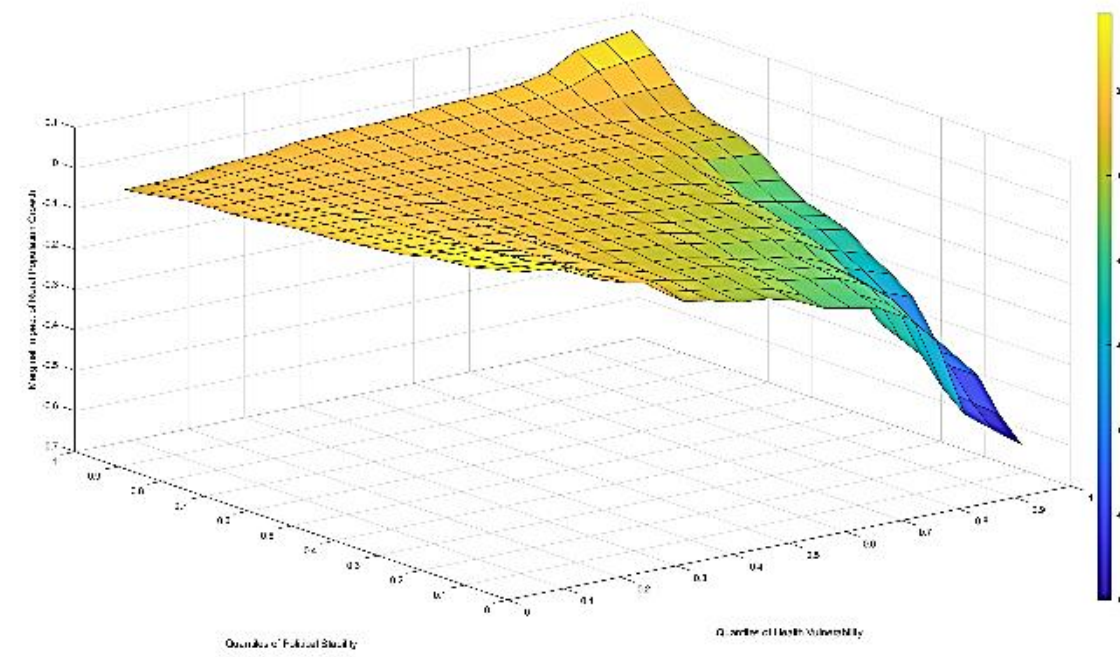

Governance Quality Indicator: Political Stability

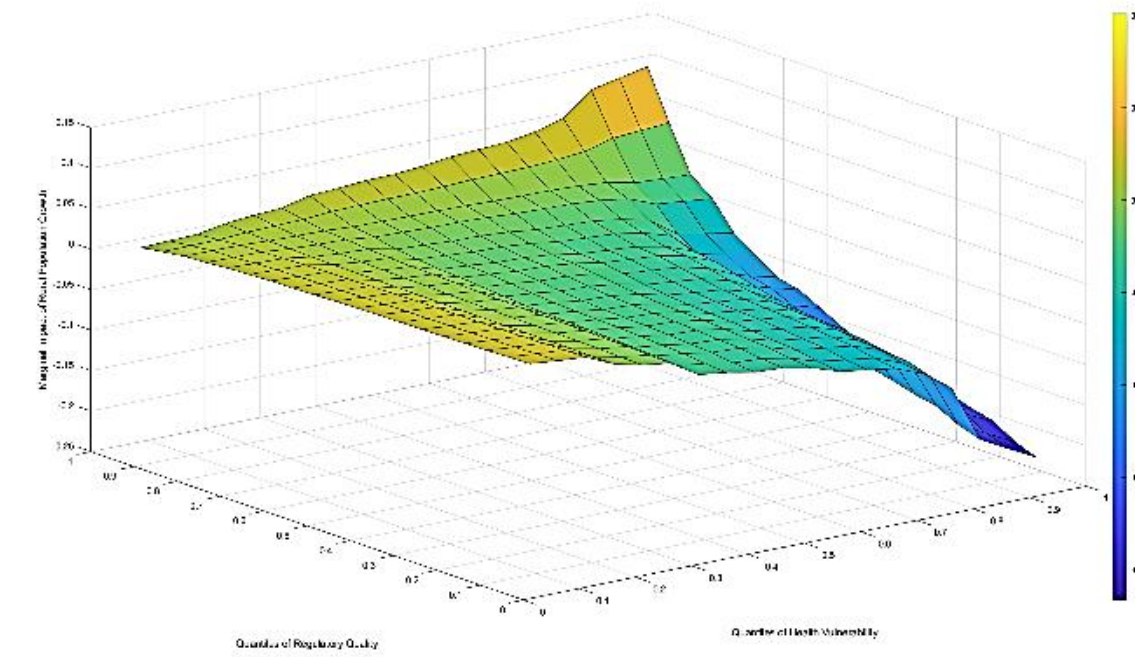

Governance Quality Indicator: Regulatory Quality

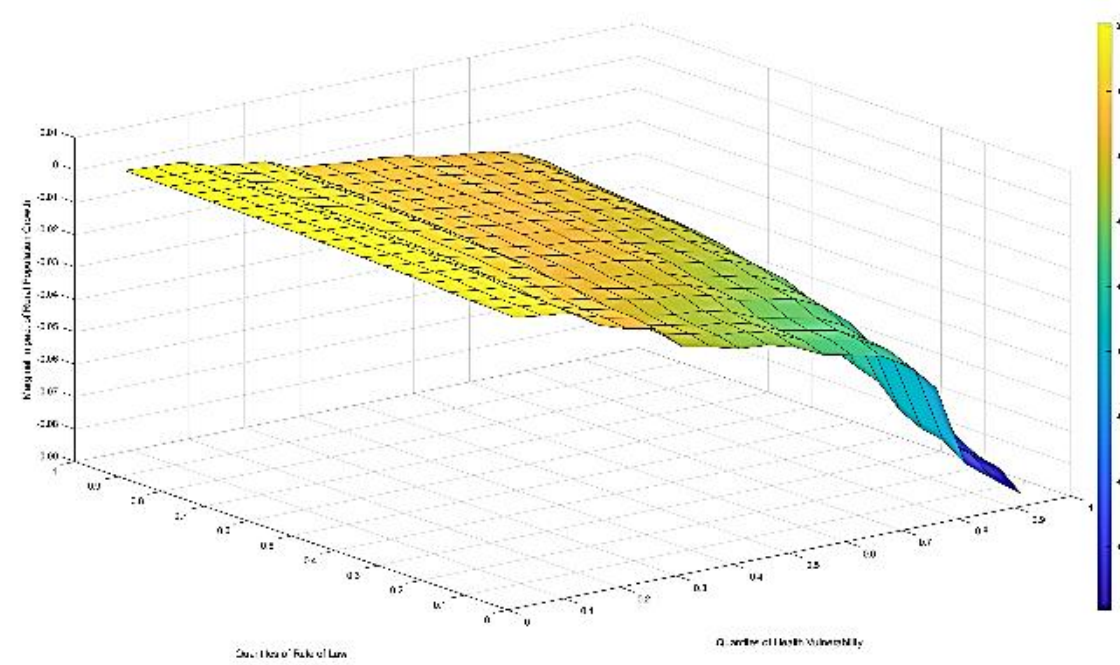

Governance Quality Indicator: Rule of Law

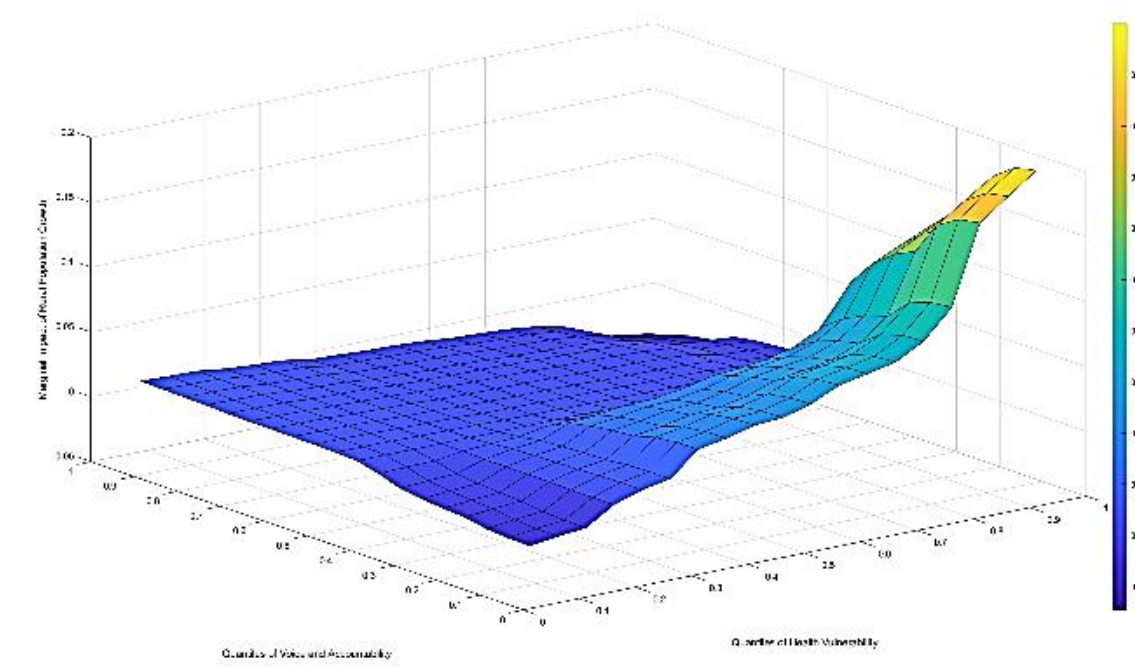

Governance Quality Indicator: Voice and Accountability

Figure S20: BRICS Policy Simulation Outcomes for the Marginal Impact of Rural Population Growth on SDG 7.1.1 under Health Vulnerability and Governance Quality, Related to Figure 4-5 and Table 2

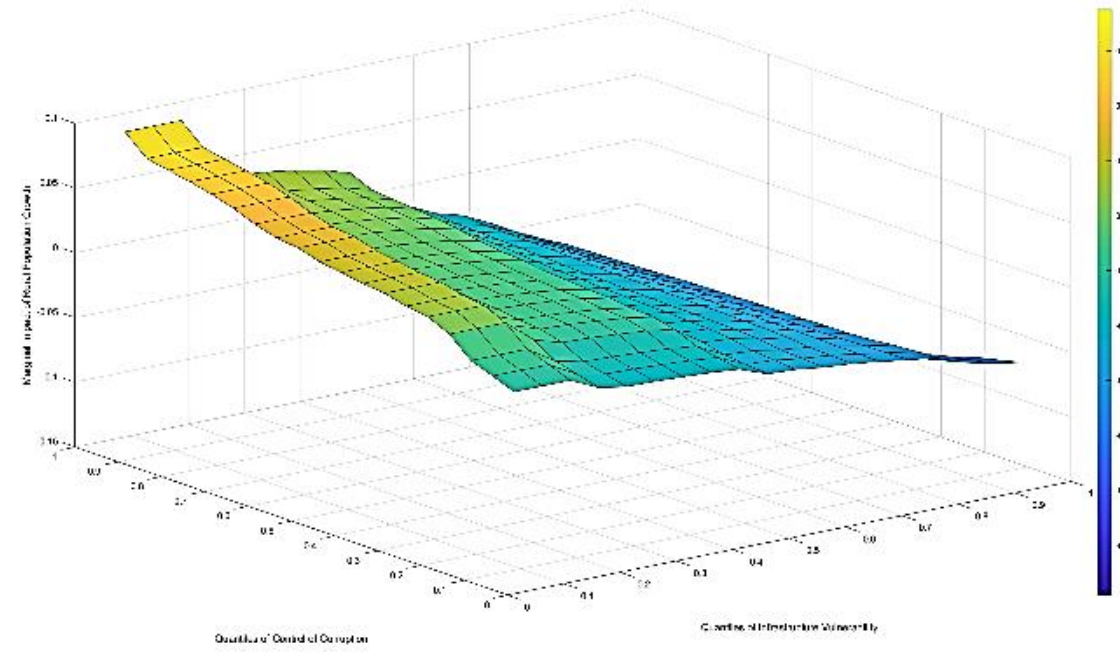

Governance Quality Indicator: Control of Corruption

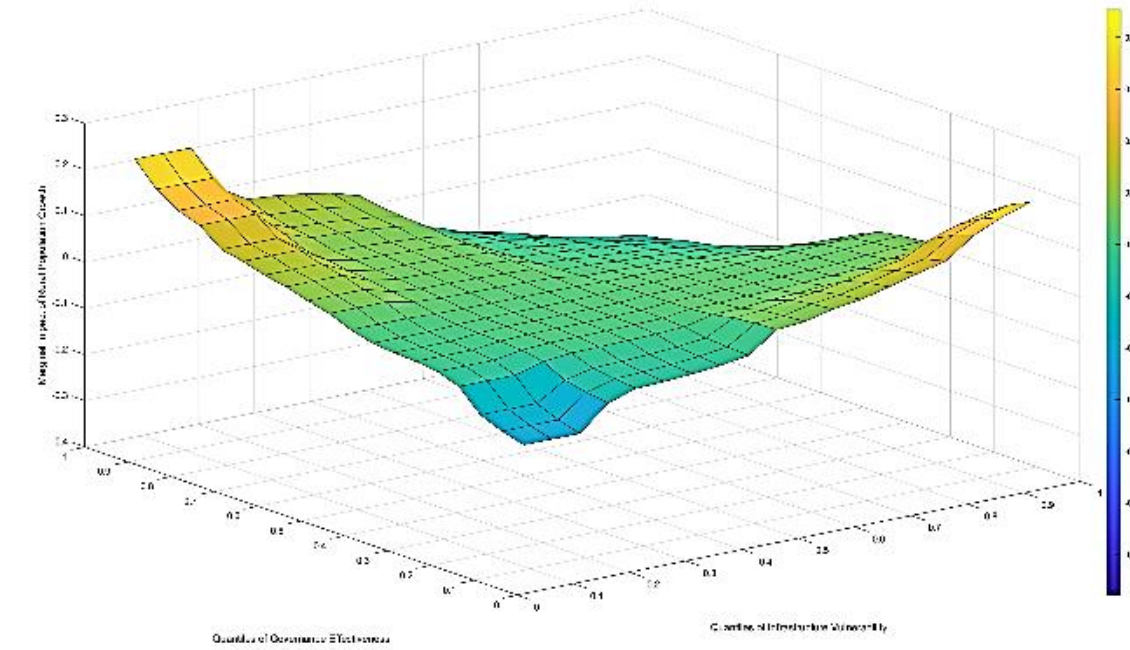

Governance Quality Indicator: Governance Effectiveness

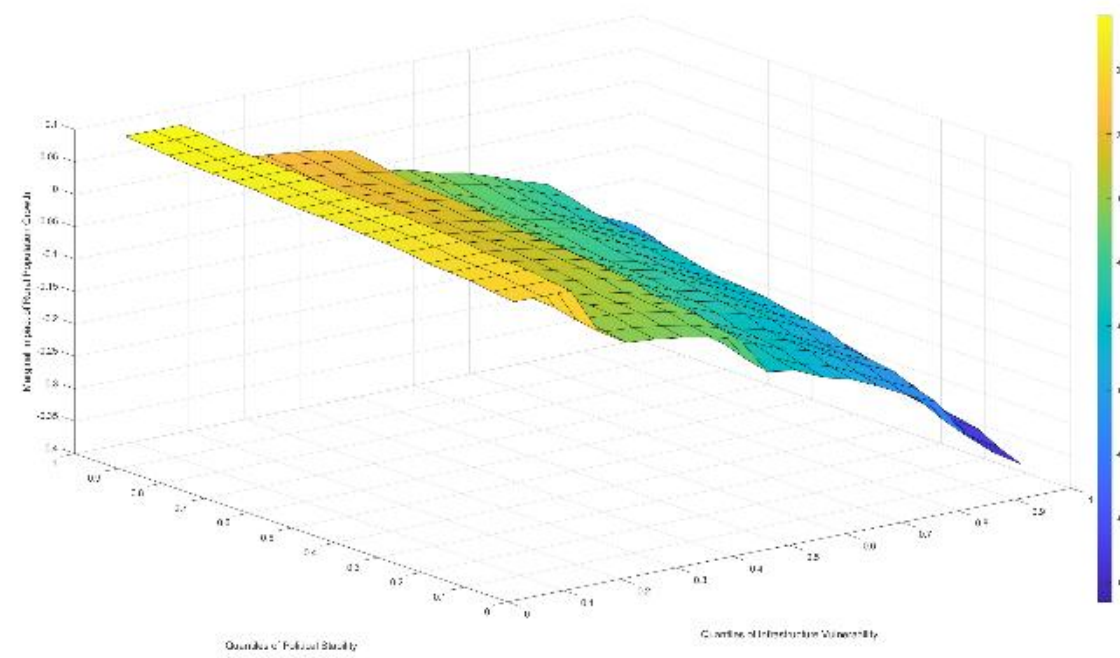

Governance Quality Indicator: Political Stability

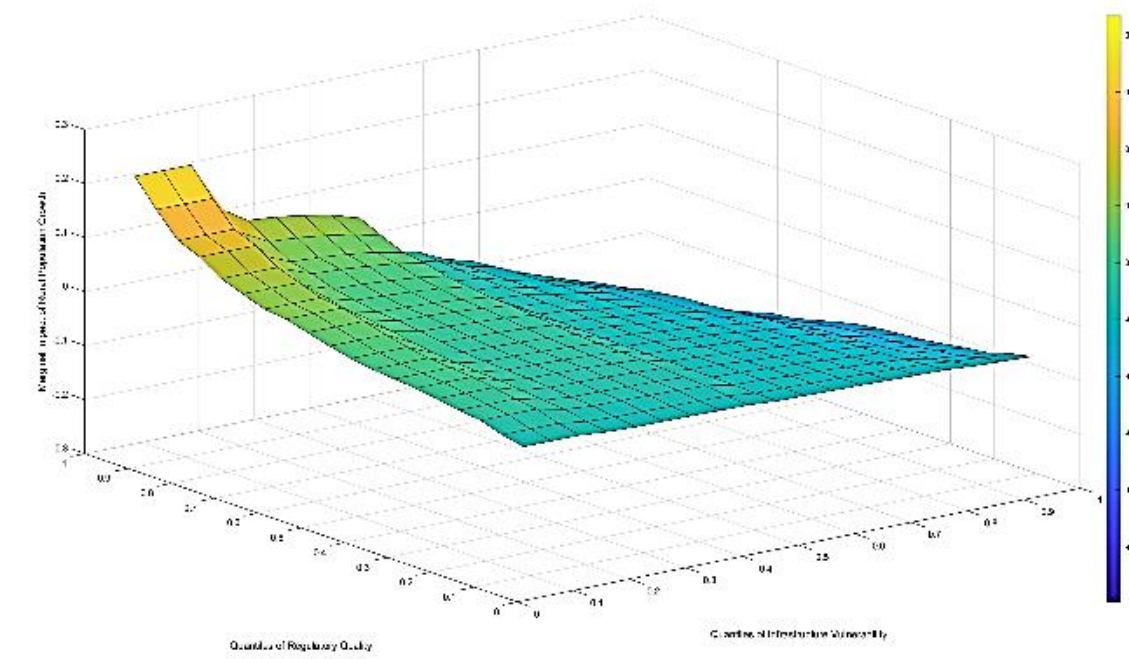

Governance Quality Indicator: Regulatory Quality

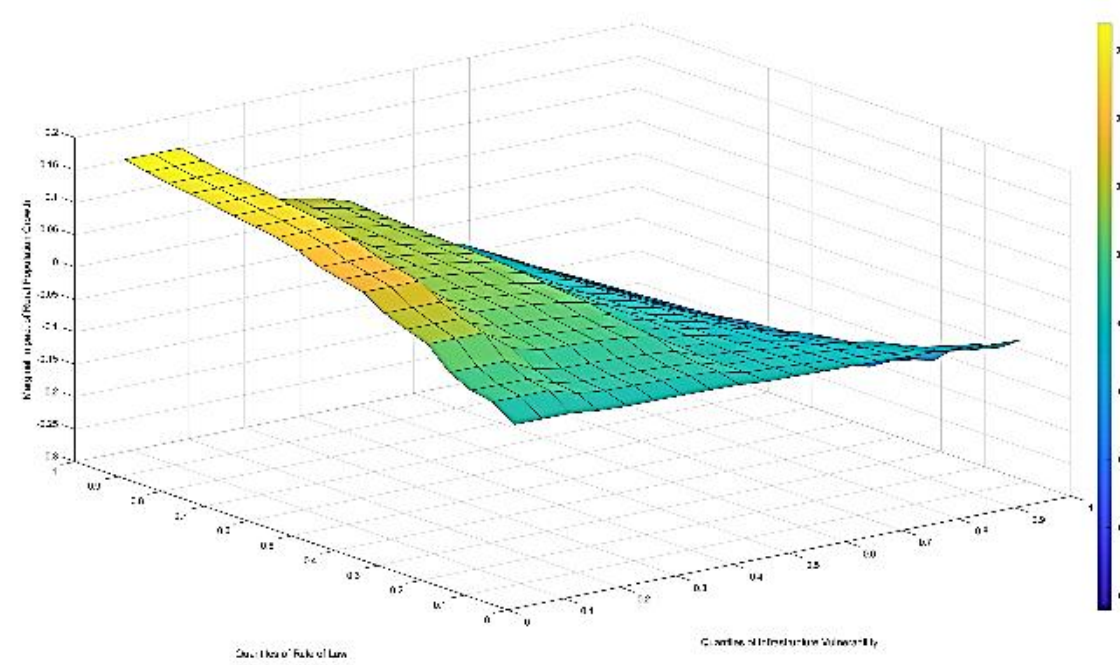

Governance Quality Indicator: Rule of Law

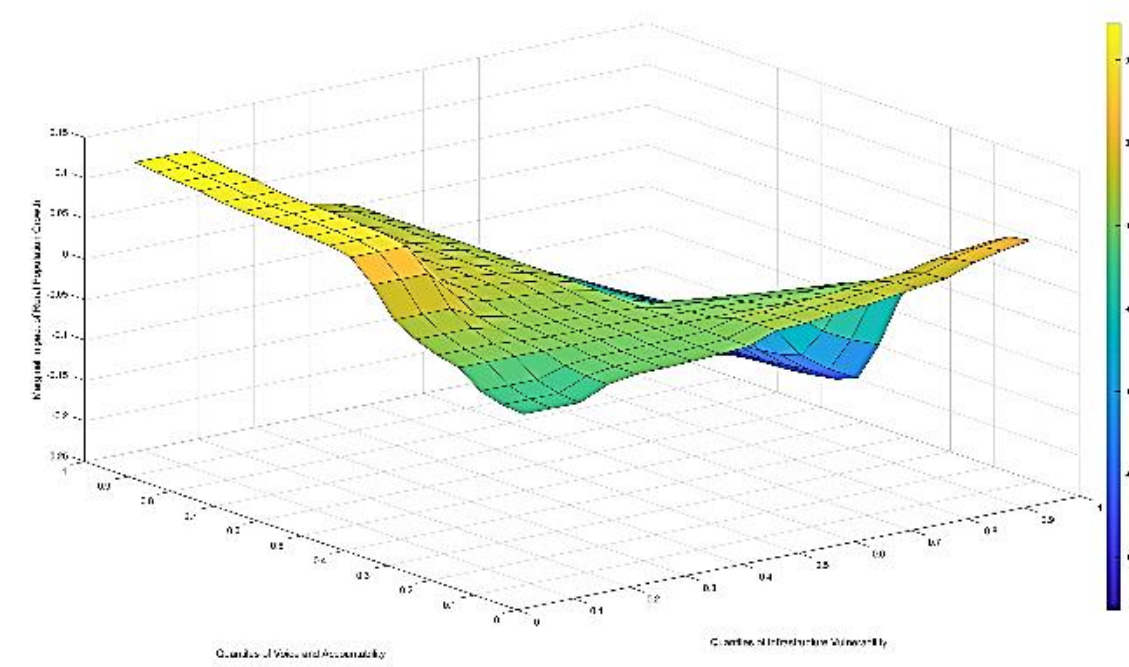

Governance Quality Indicator: Voice and Accountability

Figure S21: BRICS Policy Simulation Outcomes for the Marginal Impact of Rural Population Growth on SDG 7.1.1 under Infrastructure Vulnerability and Governance Quality, Related to Figure 4-5 and Table 2

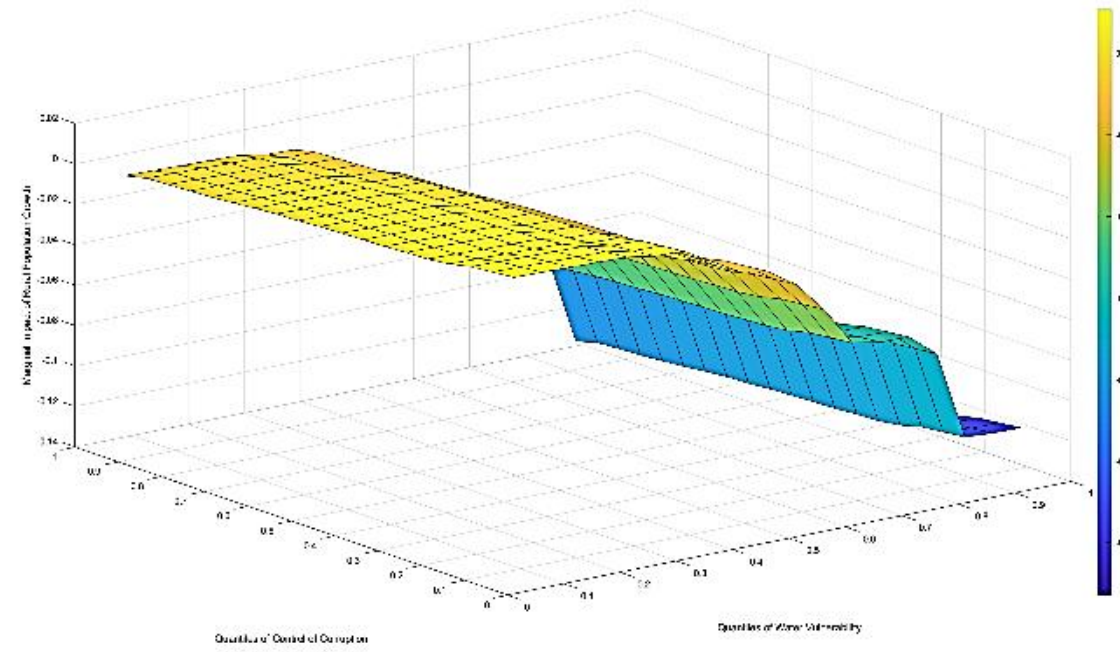

Governance Quality Indicator: Control of Corruption

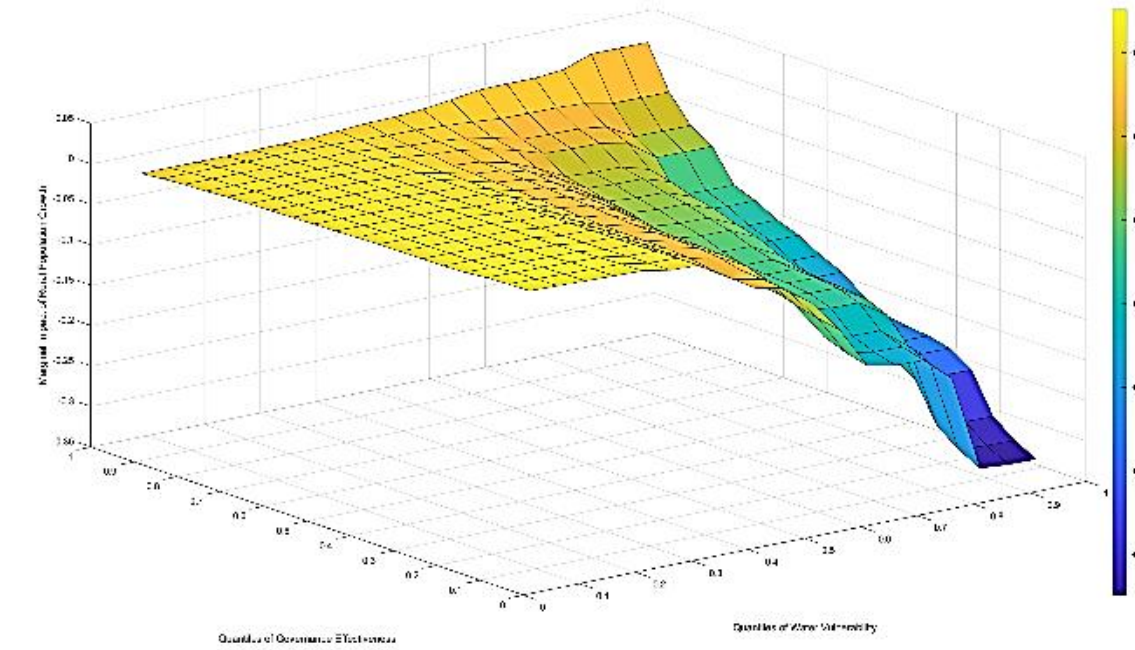

Governance Quality Indicator: Governance Effectiveness

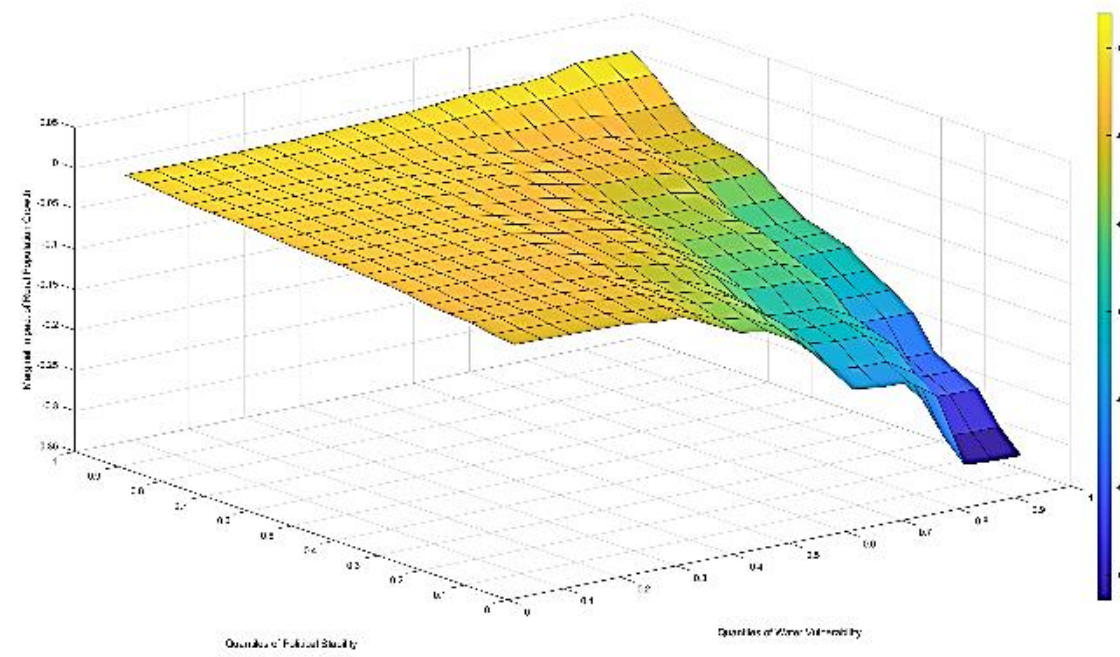

Governance Quality Indicator: Political Stability

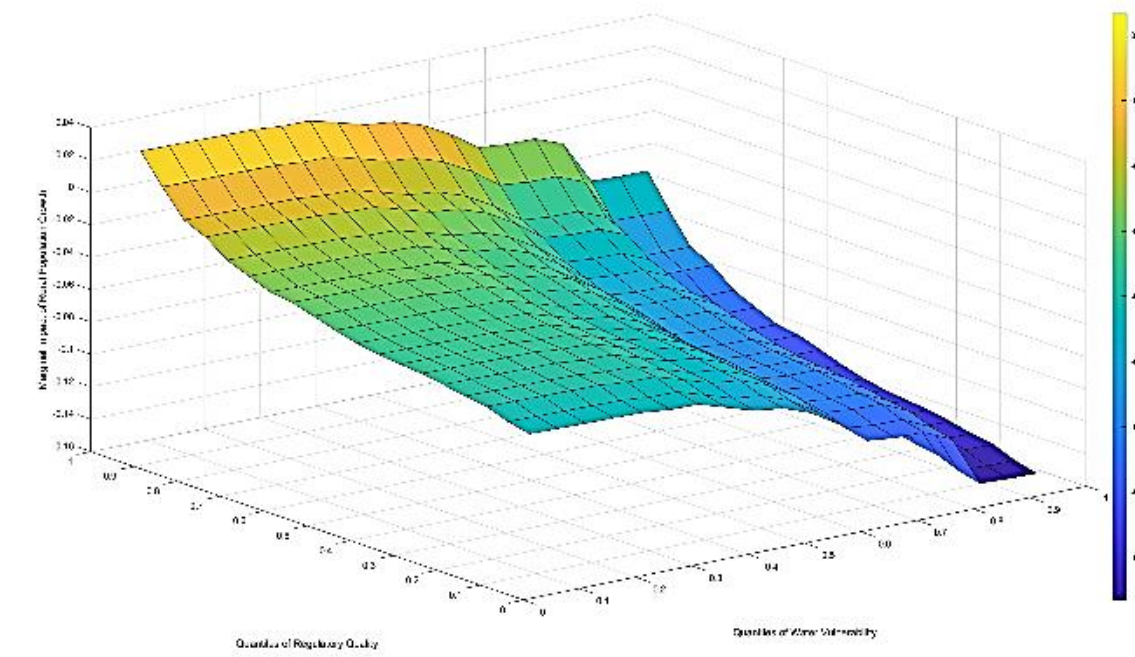

Governance Quality Indicator: Regulatory Quality

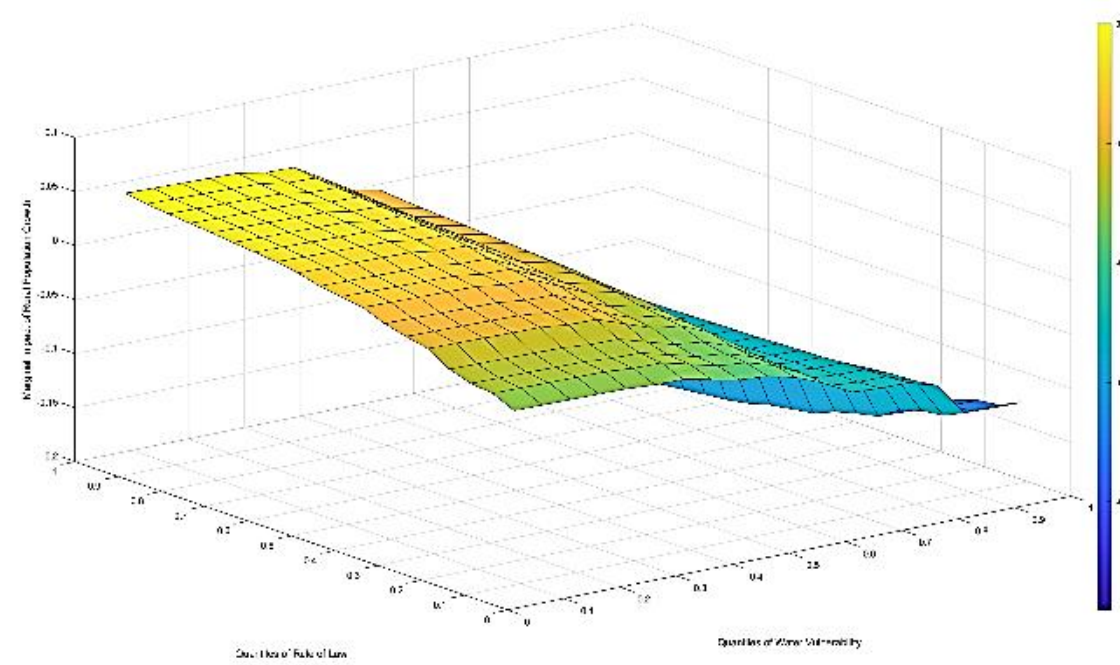

Governance Quality Indicator: Rule of Law

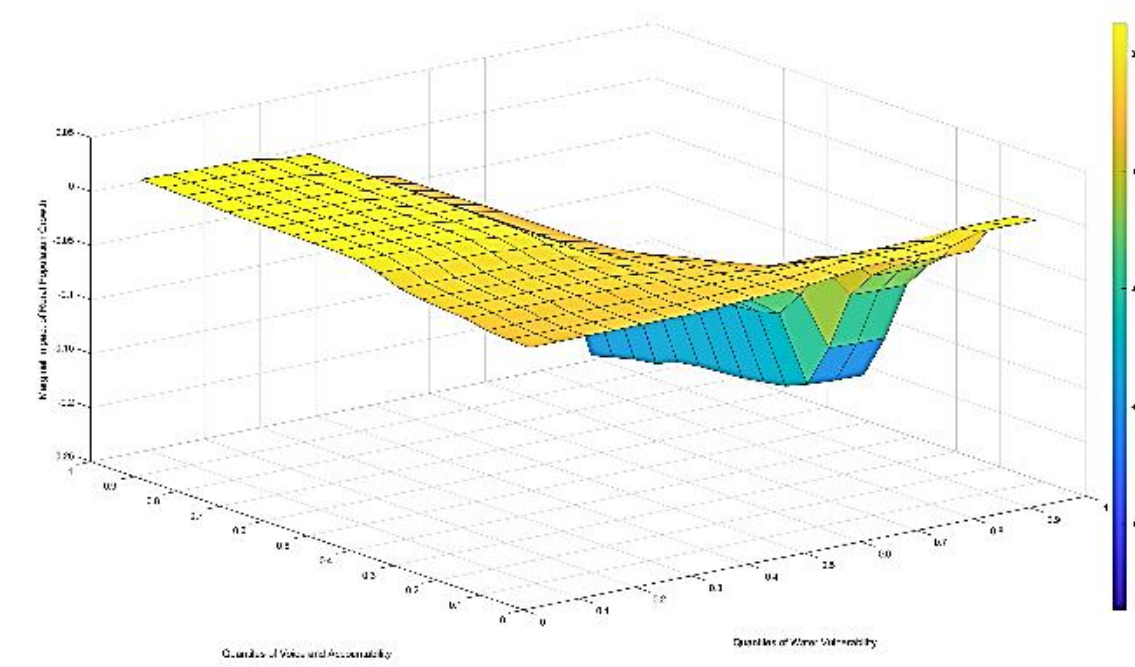

Governance Quality Indicator: Voice and Accountability

Figure S22: BRICS Policy Simulation Outcomes for the Marginal Impact of Rural Population Growth on SDG 7.1.1 under Water Vulnerability and Governance Quality, Related to Figure 4-5 and Table 2

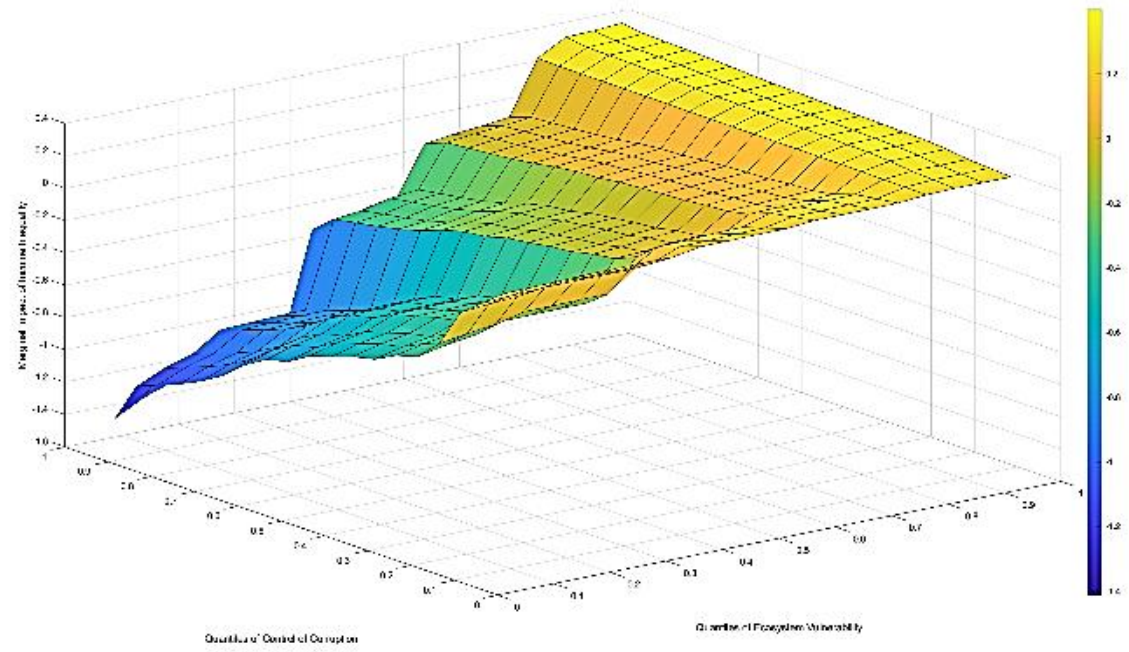

Governance Quality Indicator: Control of Corruption

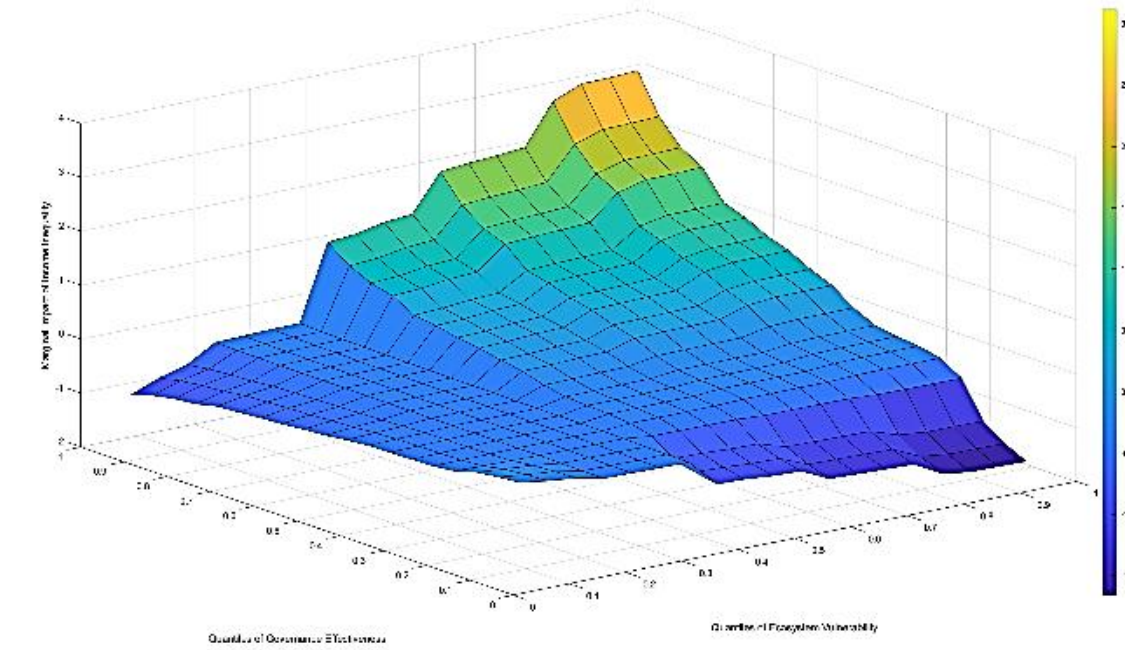

Governance Quality Indicator: Governance Effectiveness

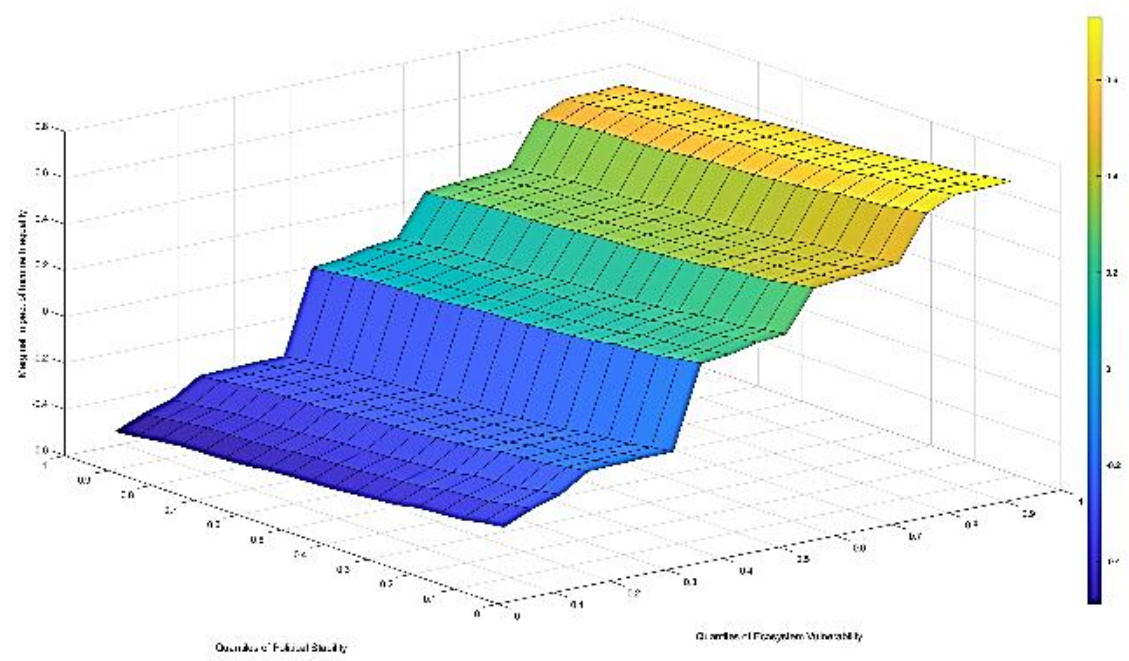

Governance Quality Indicator: Political Stability

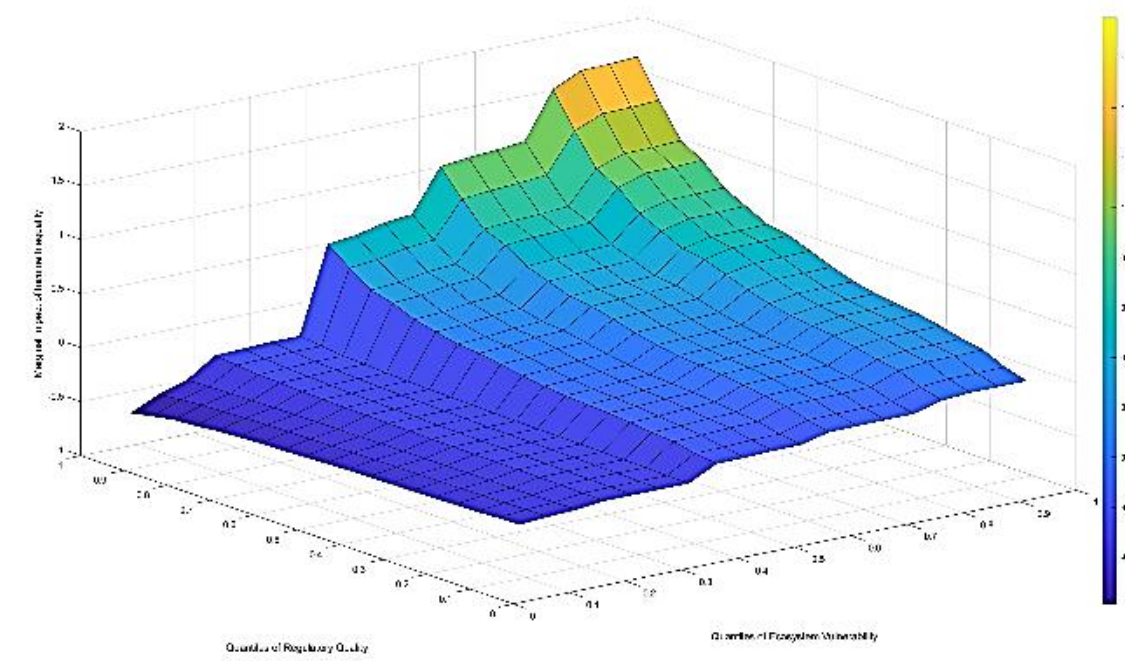

Governance Quality Indicator: Regulatory Quality

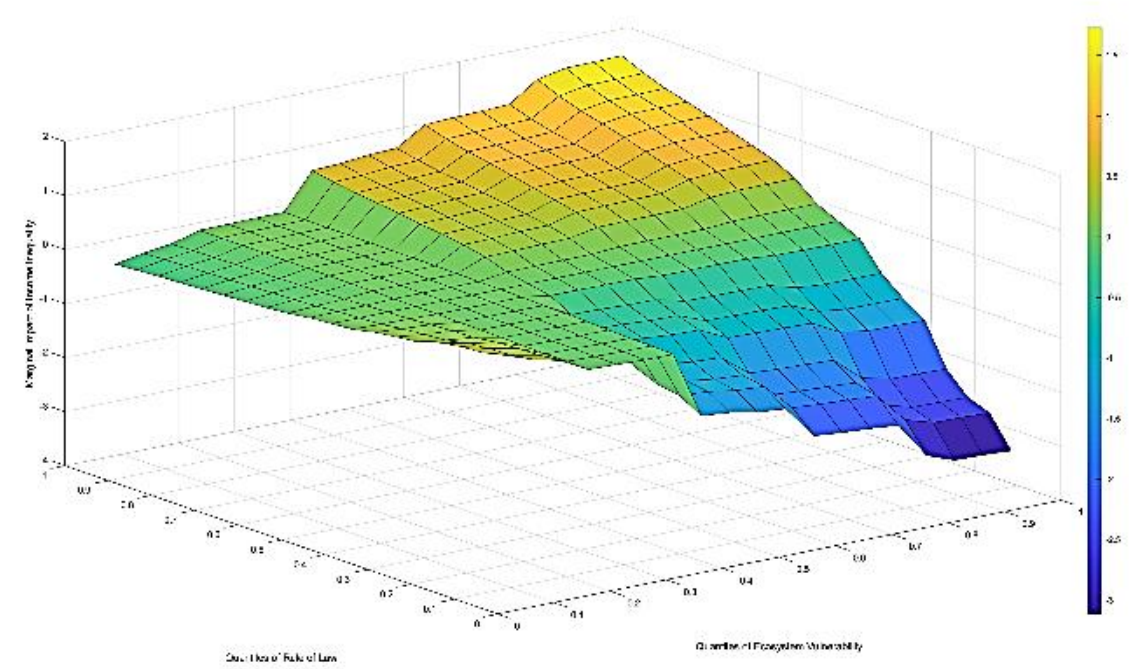

Governance Quality Indicator: Rule of Law

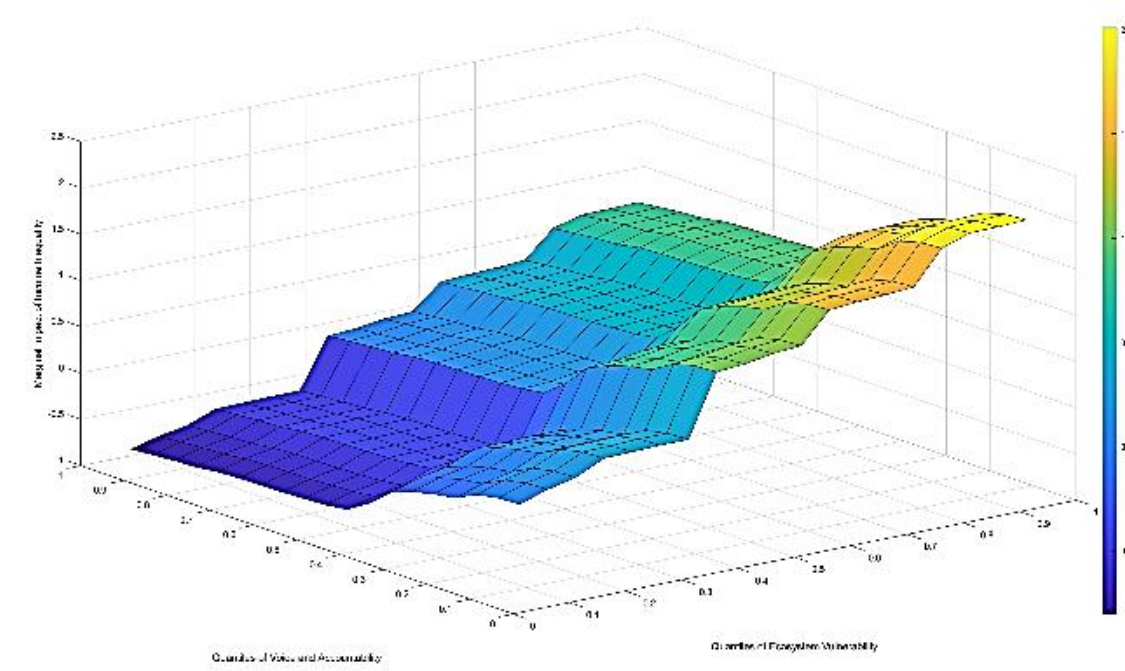

Governance Quality Indicator: Voice and Accountability

Figure S23: BRICS Policy Simulation Outcomes for the Marginal Impact of Income Inequality on SDG 7.1.1 under Ecosystem Vulnerability and Governance Quality, Related to Figure 4-5 and Table 2

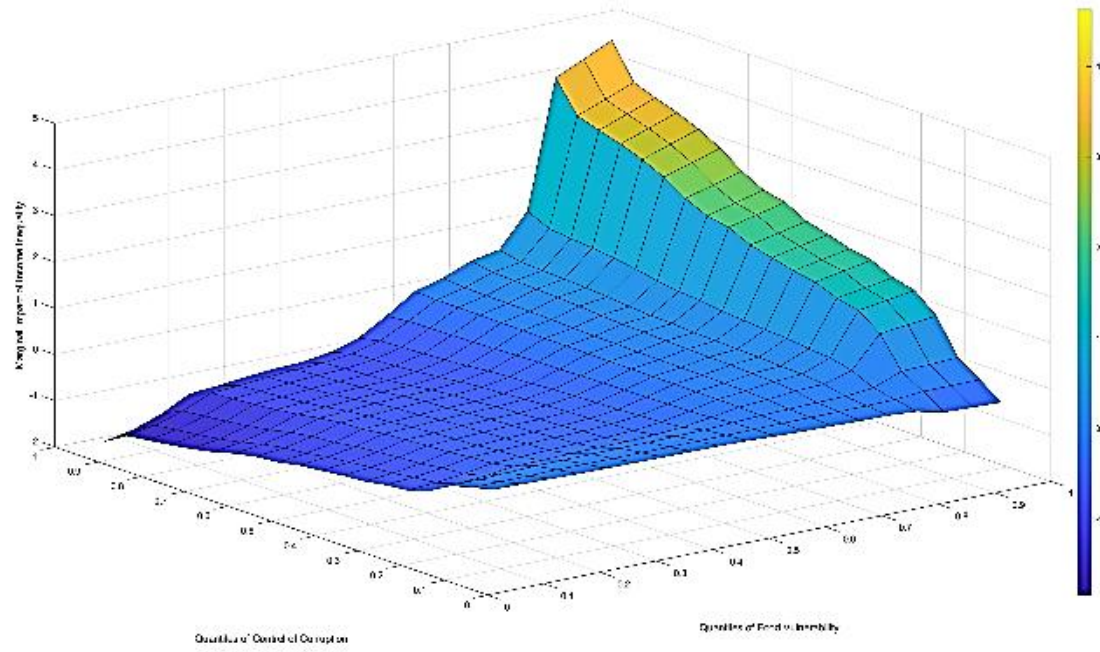

Governance Quality Indicator: Control of Corruption

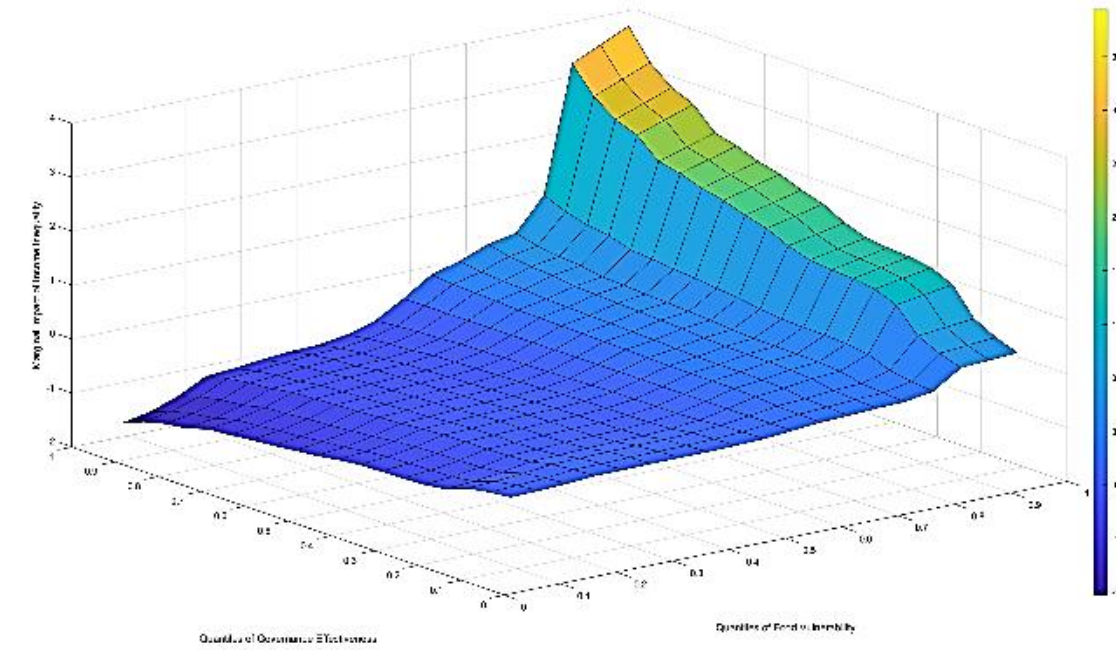

Governance Quality Indicator: Governance Effectiveness

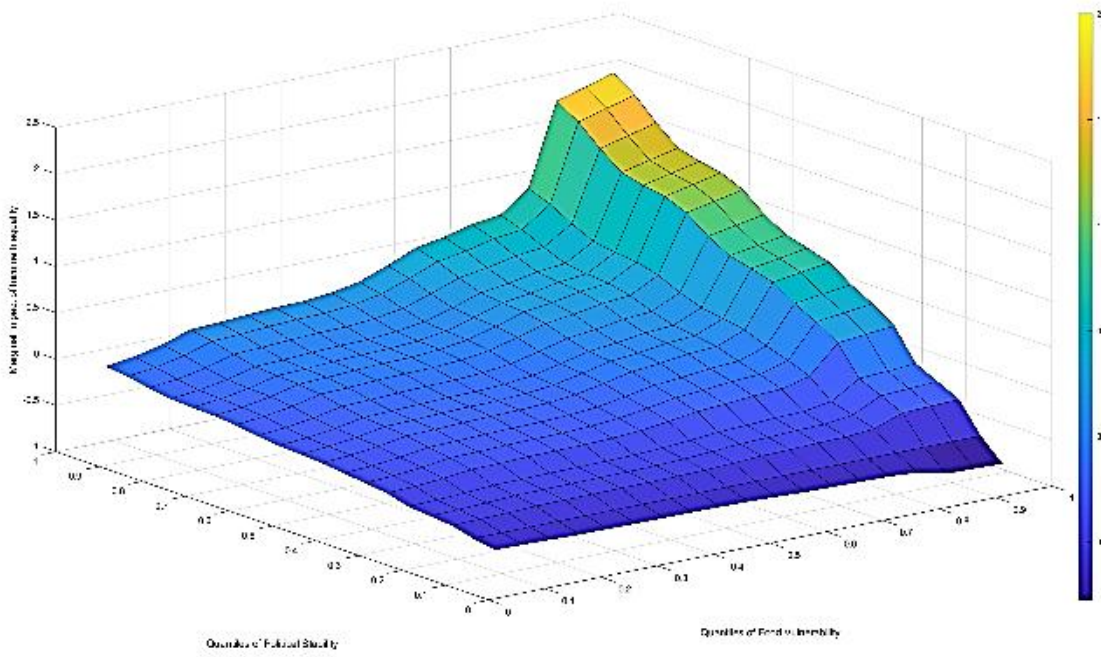

Governance Quality Indicator: Political Stability

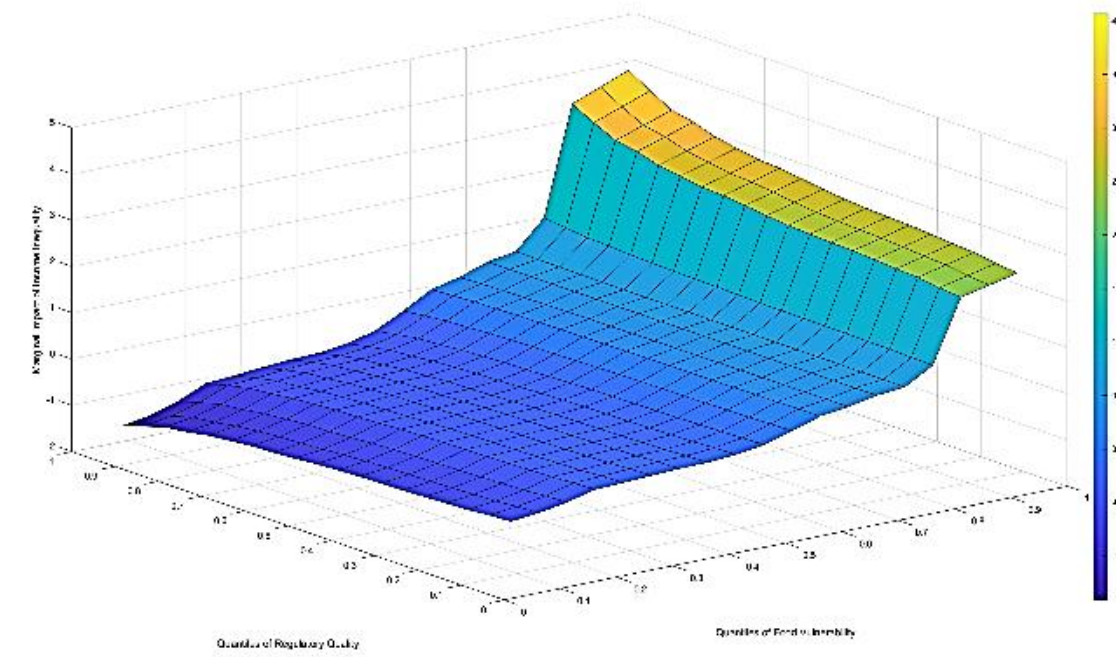

Governance Quality Indicator: Regulatory Quality

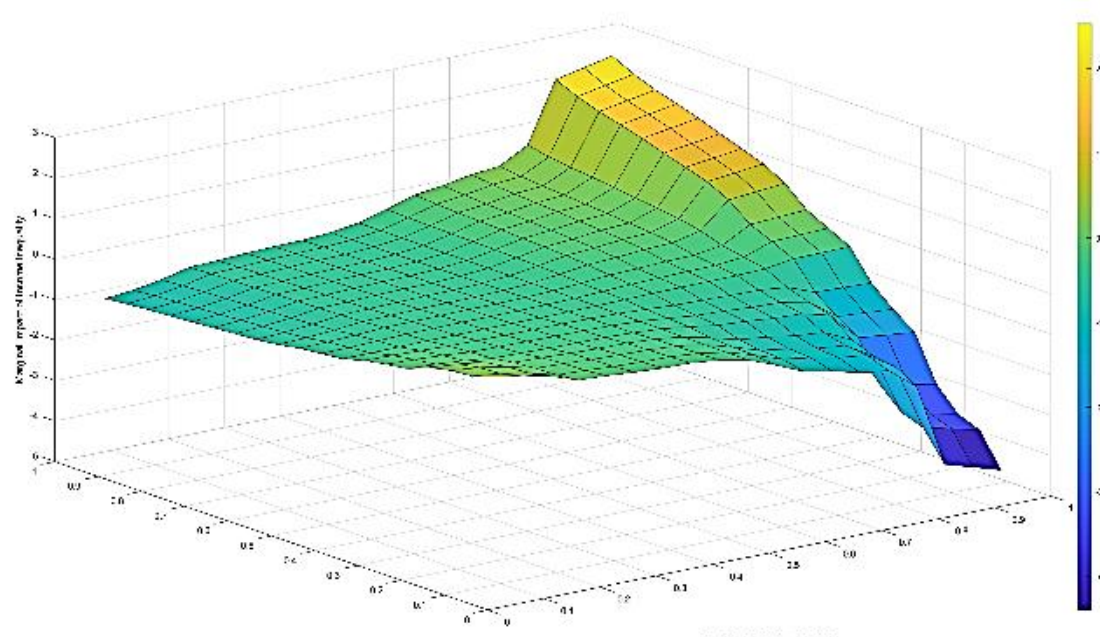

Governance Quality Indicator: Rule of Law

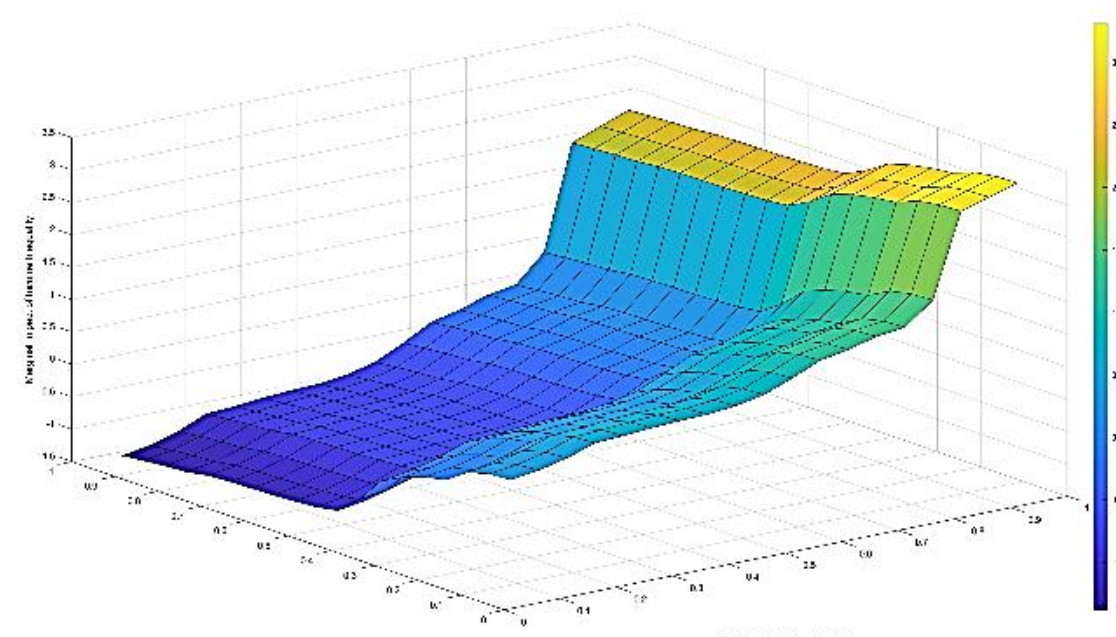

Governance Quality Indicator: Voice and Accountability

Figure S24: BRICS Policy Simulation Outcomes for the Marginal Impact of Income Inequality on SDG 7.1.1 under Food Vulnerability and Governance Quality, Related to Figure 4-5 and Table 2

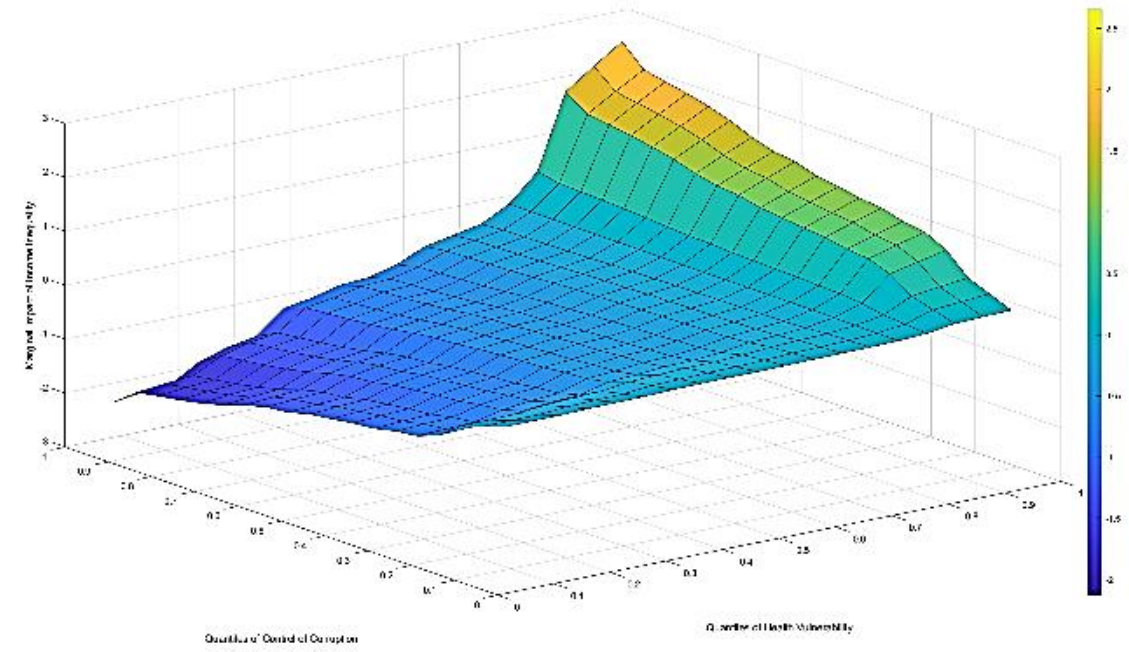

Governance Quality Indicator: Control of Corruption

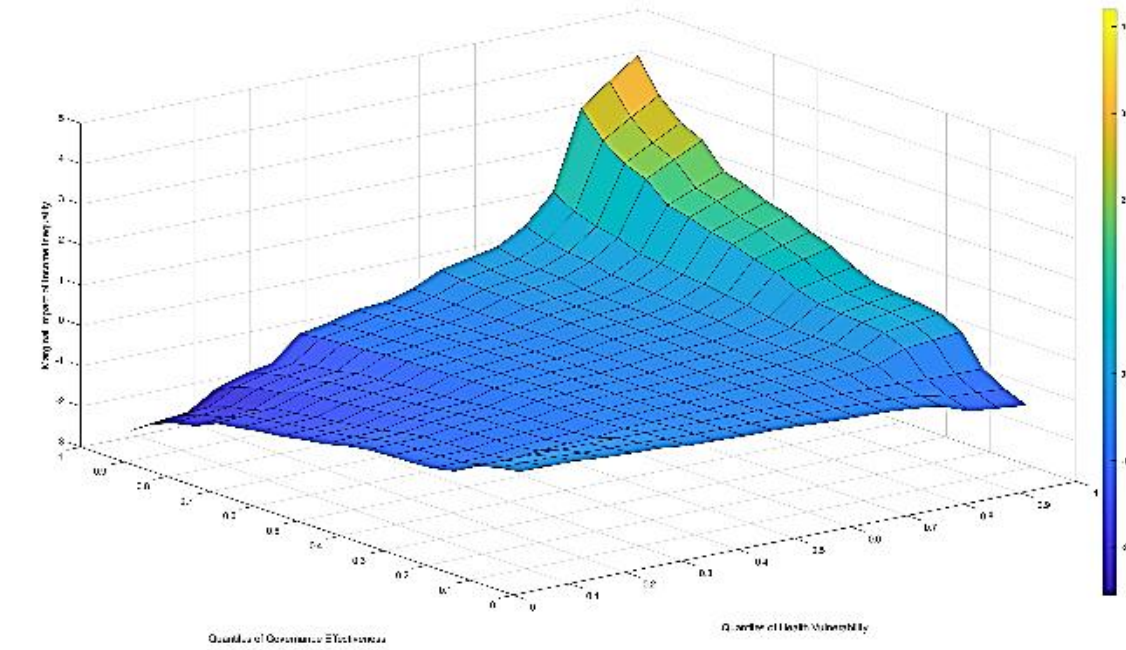

Governance Quality Indicator: Governance Effectiveness

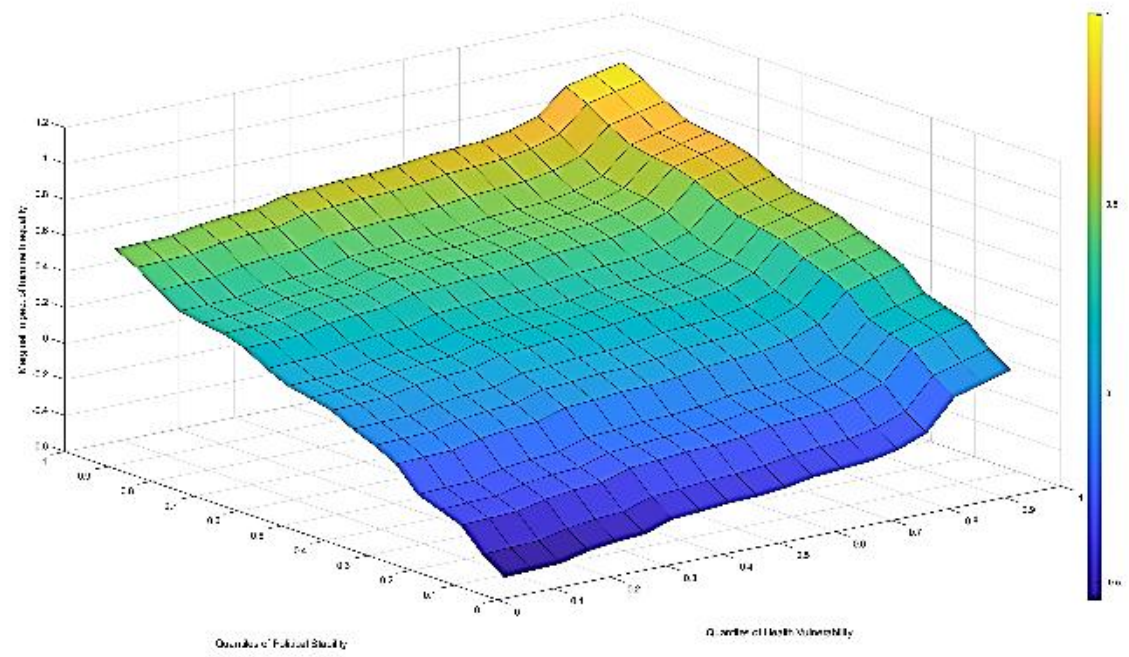

Governance Quality Indicator: Political Stability

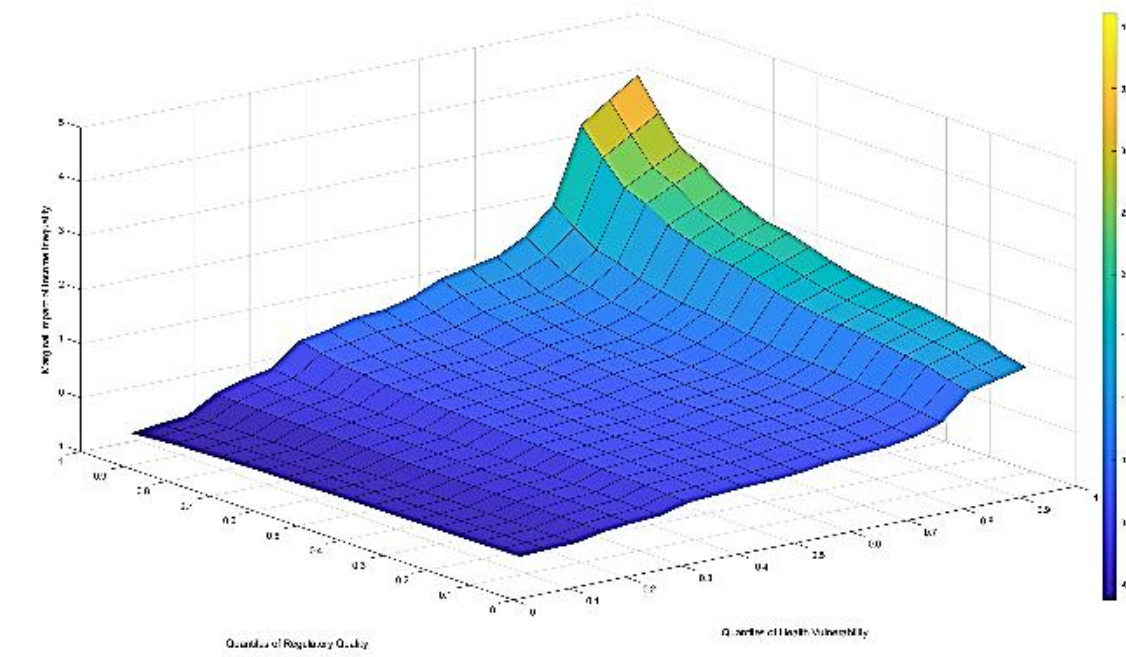

Governance Quality Indicator: Regulatory Quality

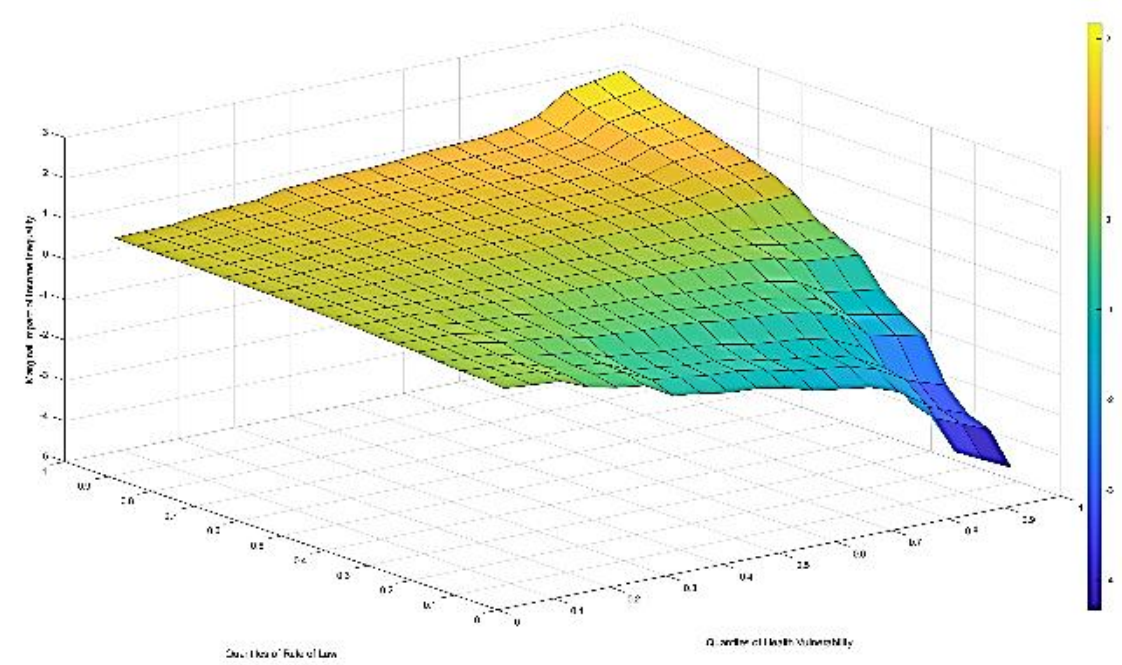

Governance Quality Indicator: Rule of Law

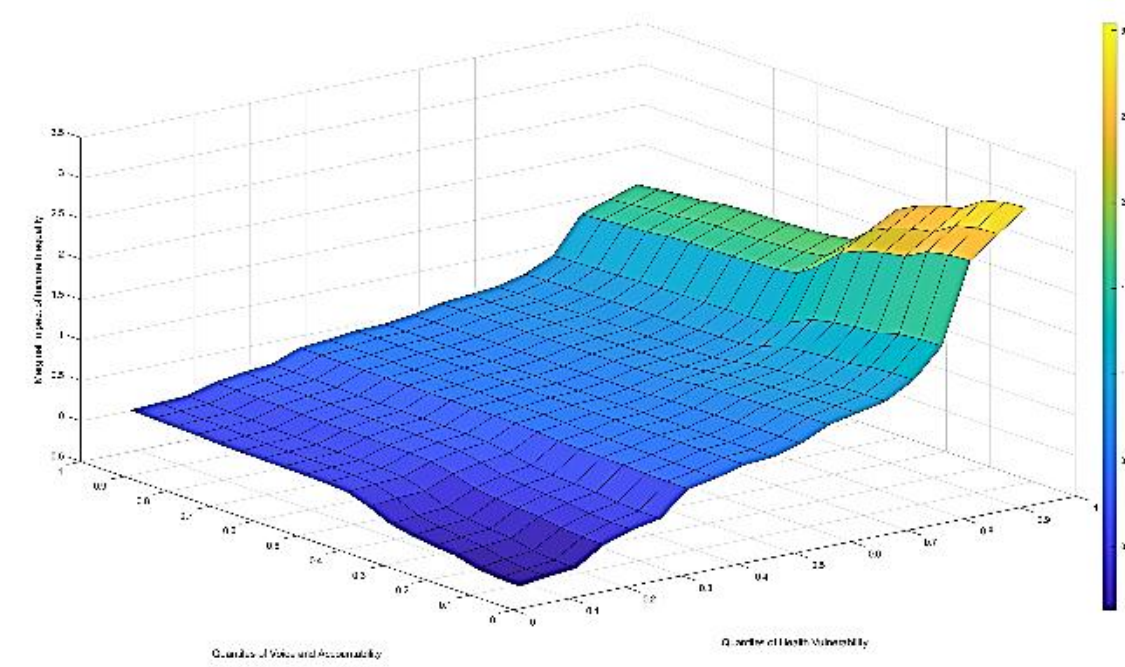

Governance Quality Indicator: Voice and Accountability

Figure S25: BRICS Policy Simulation Outcomes for the Marginal Impact of Income Inequality on SDG 7.1.1 under Health Vulnerability and Governance Quality, Related to Figure 4-5 and Table 2

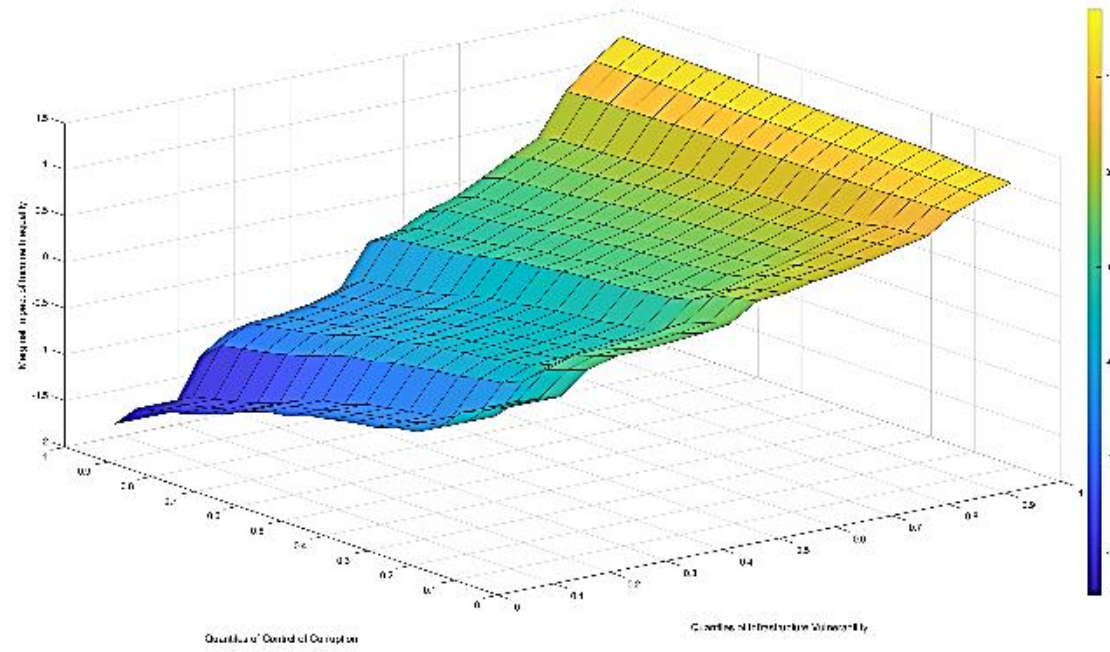

Governance Quality Indicator: Control of Corruption

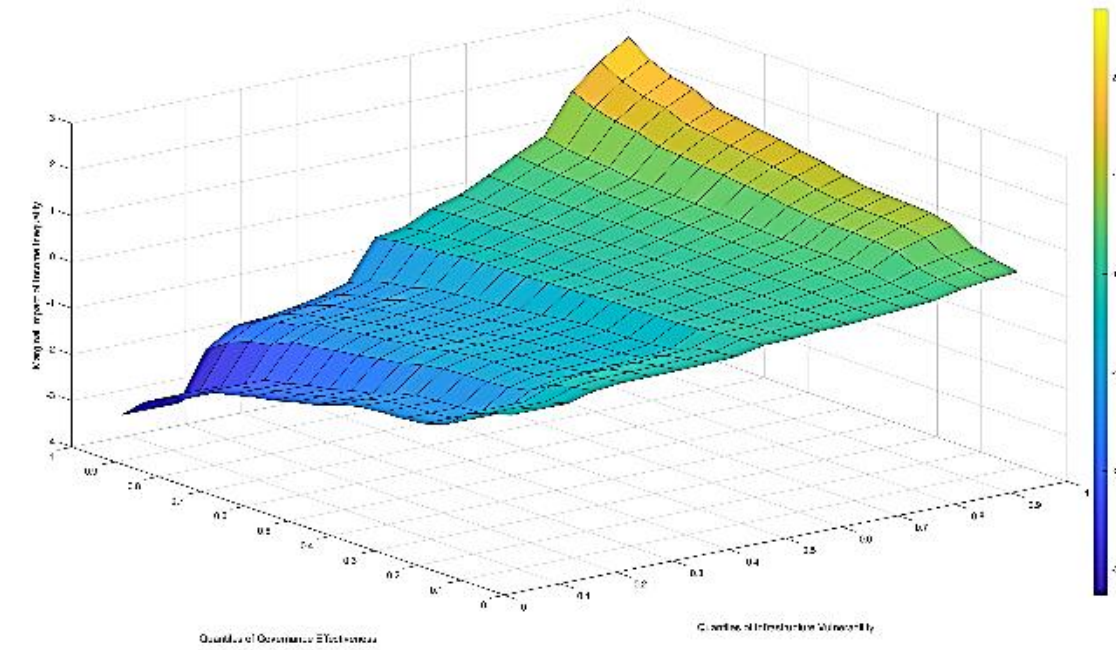

Governance Quality Indicator: Governance Effectiveness

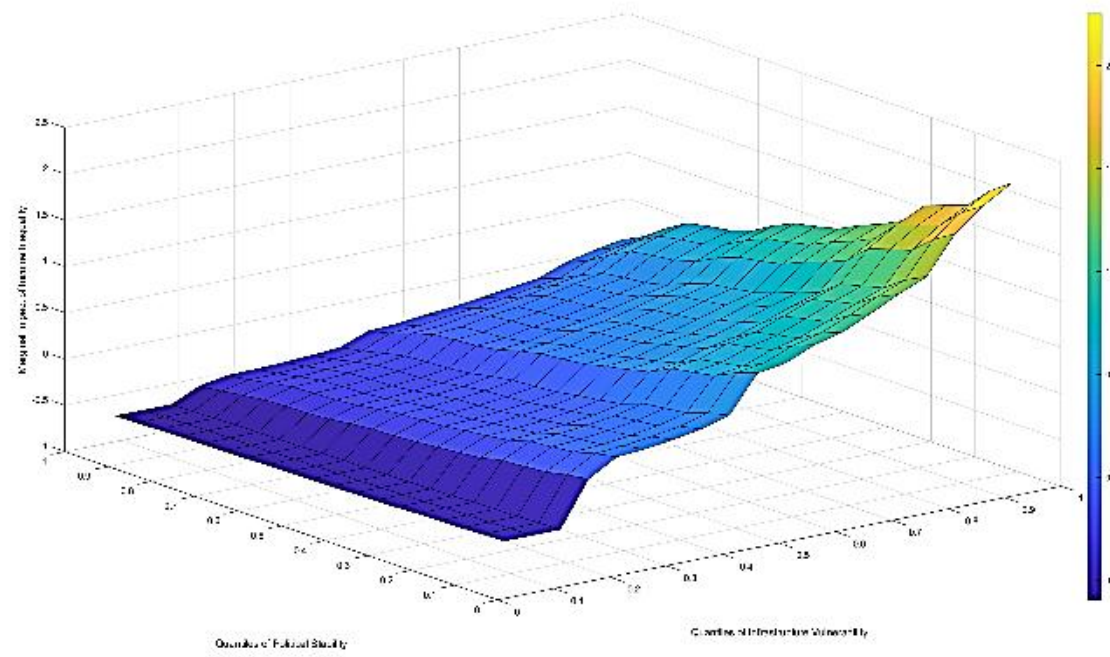

Governance Quality Indicator: Political Stability

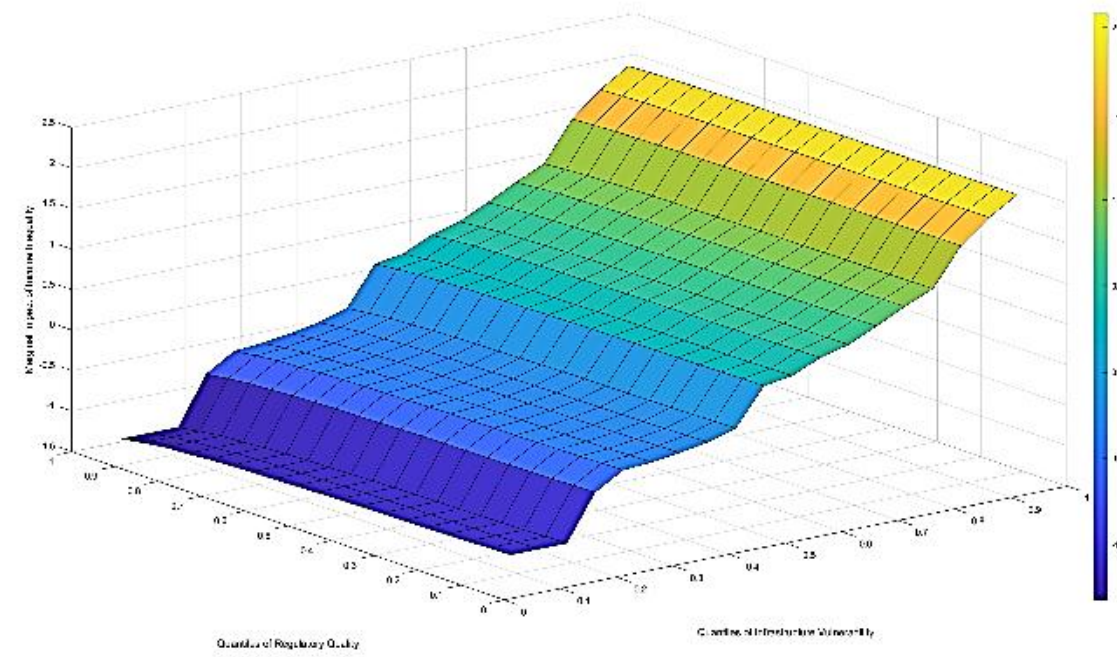

Governance Quality Indicator: Regulatory Quality

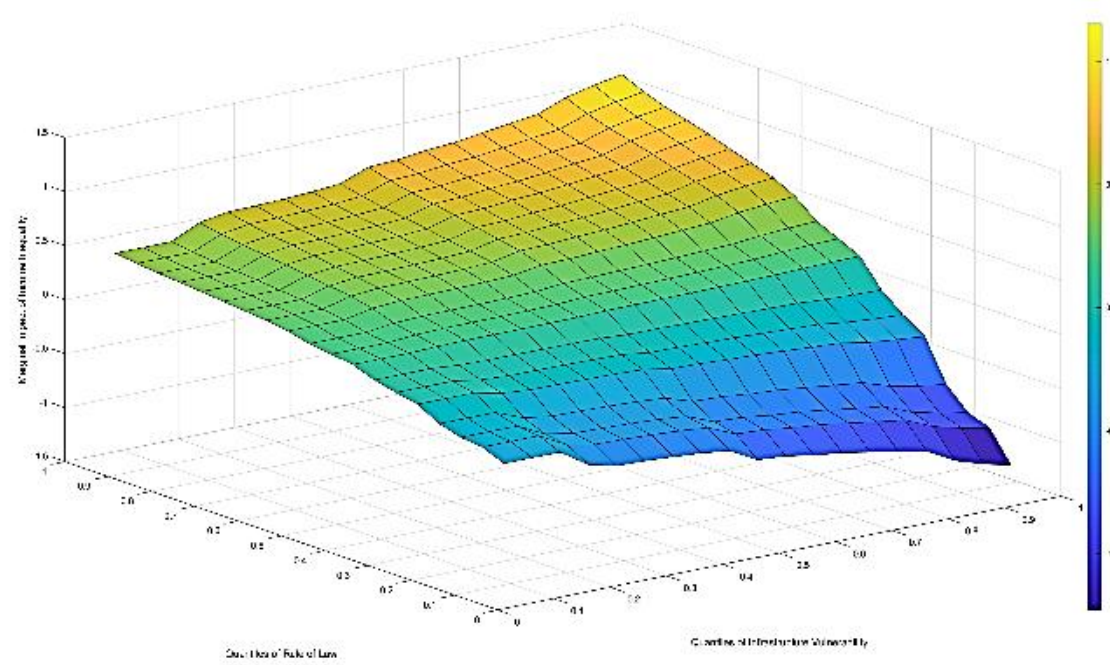

Governance Quality Indicator: Rule of Law

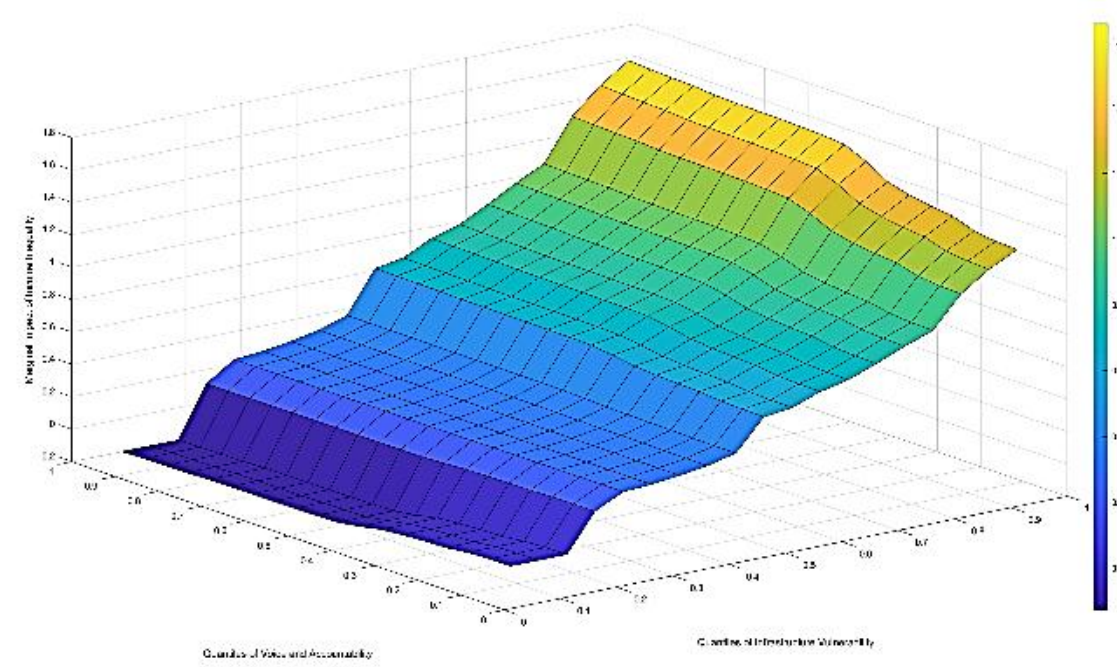

Governance Quality Indicator: Voice and Accountability

Figure S26: BRICS Policy Simulation Outcomes for the Marginal Impact of Income Inequality on SDG 7.1.1 under Infrastructure Vulnerability and Governance Quality, Related to Figure 4-5 and Table 2

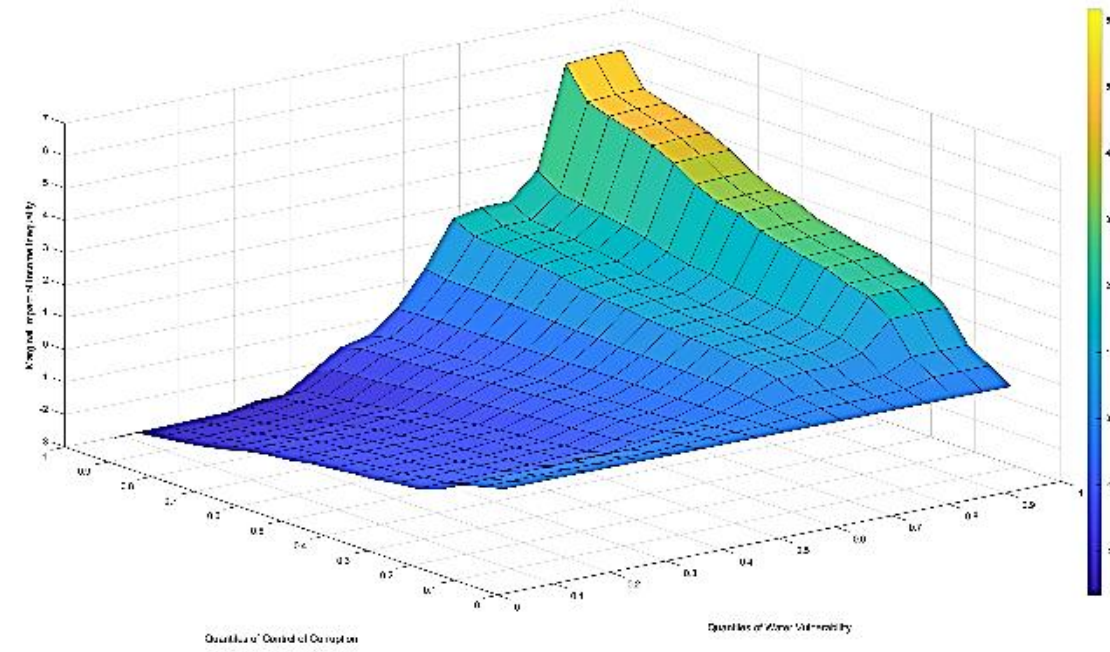

Governance Quality Indicator: Control of Corruption

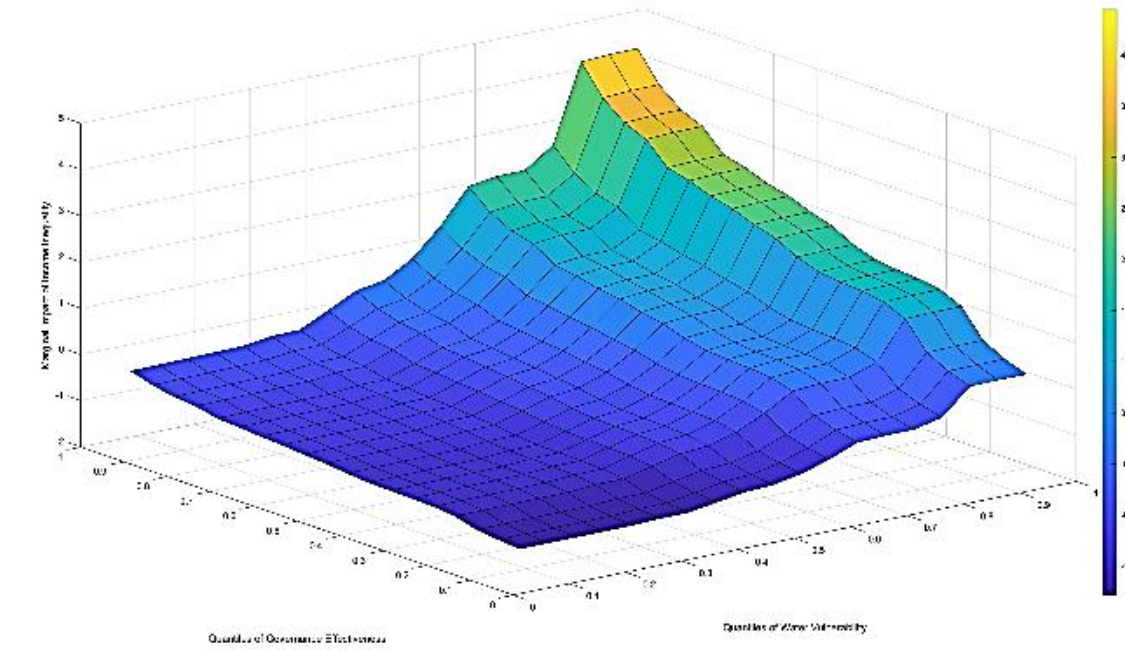

Governance Quality Indicator: Governance Effectiveness

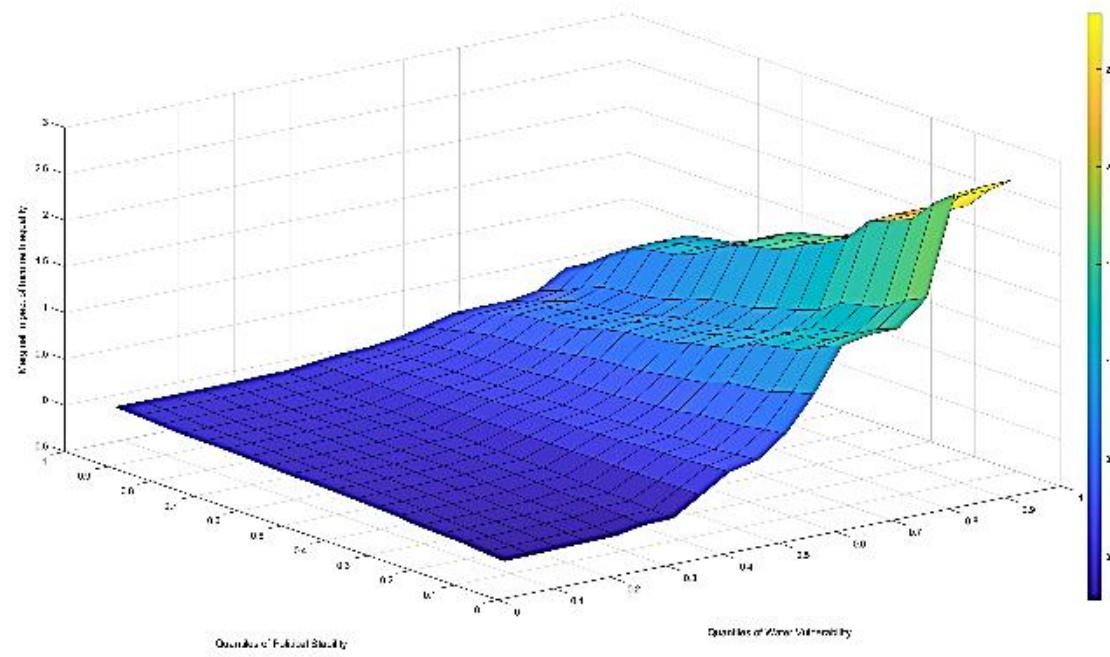

Governance Quality Indicator: Political Stability

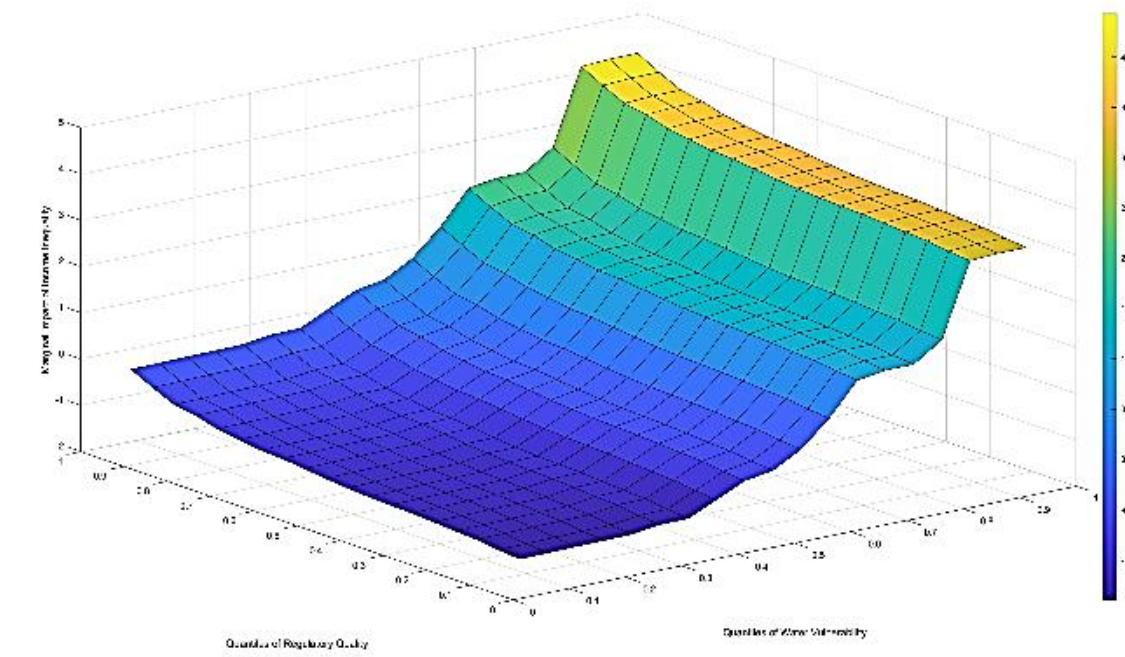

Governance Quality Indicator: Regulatory Quality

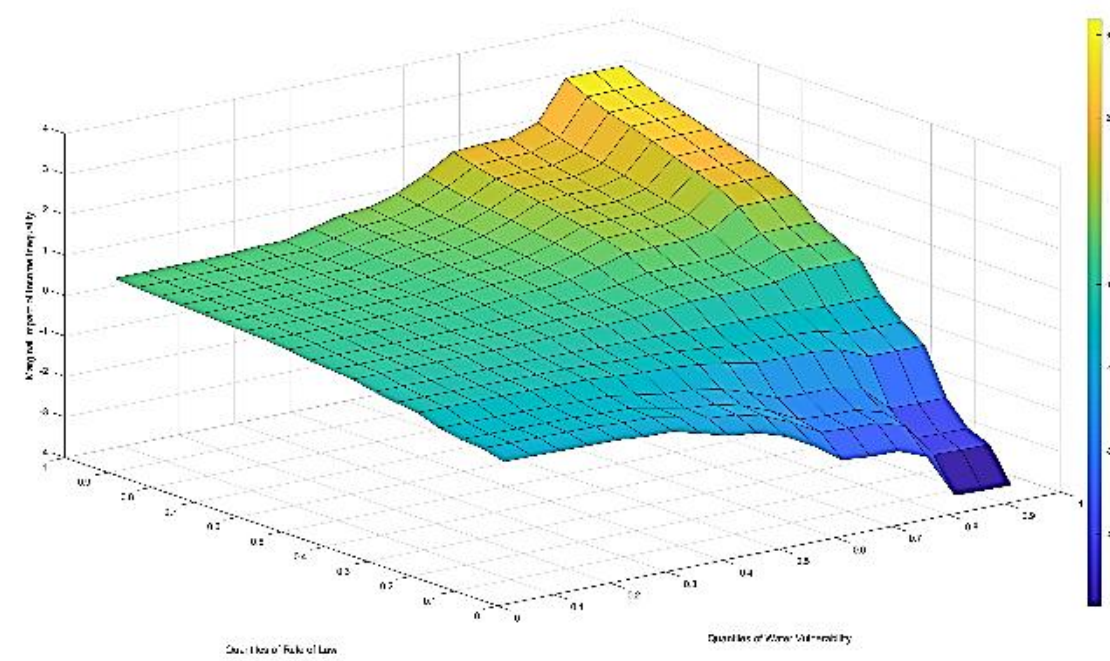

Governance Quality Indicator: Rule of Law

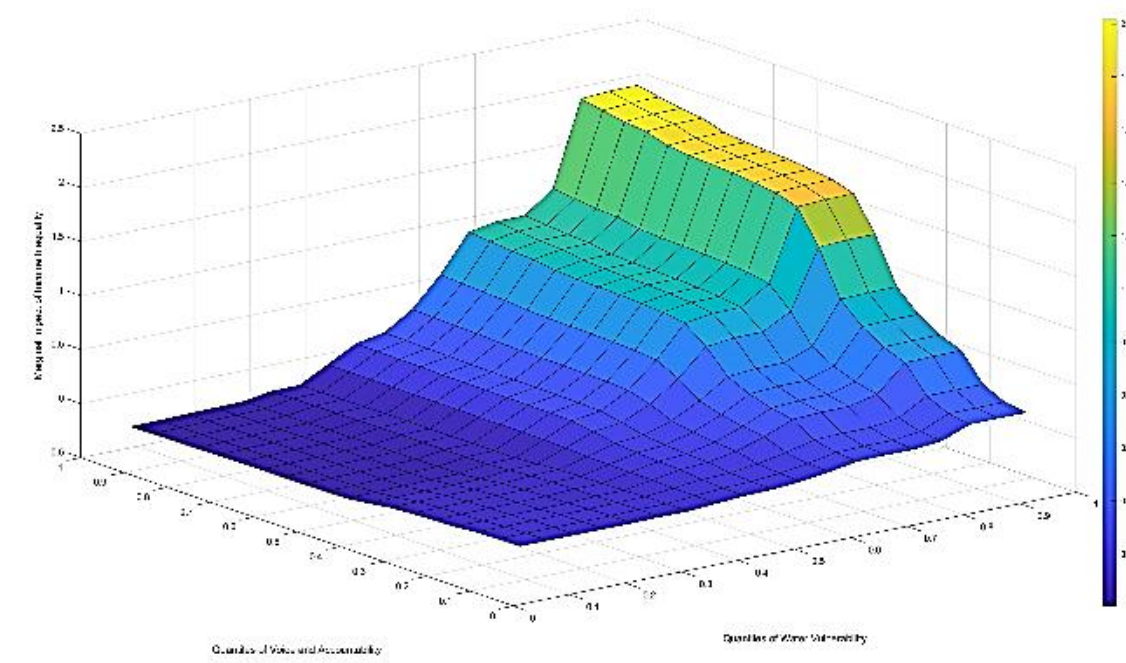

Governance Quality Indicator: Voice and Accountability

Figure S27: BRICS Policy Simulation Outcomes for the Marginal Impact of Income Inequality on SDG 7.1.1 under Water Vulnerability and Governance Quality, Related to Figure 4-5 and Table 2

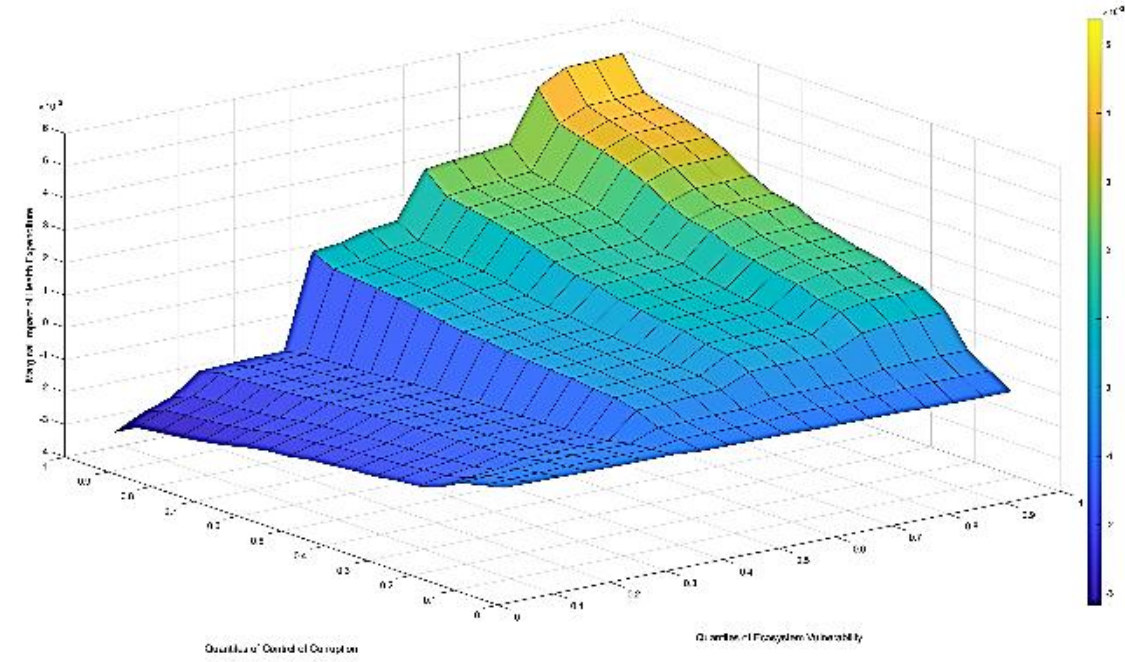

Governance Quality Indicator: Control of Corruption

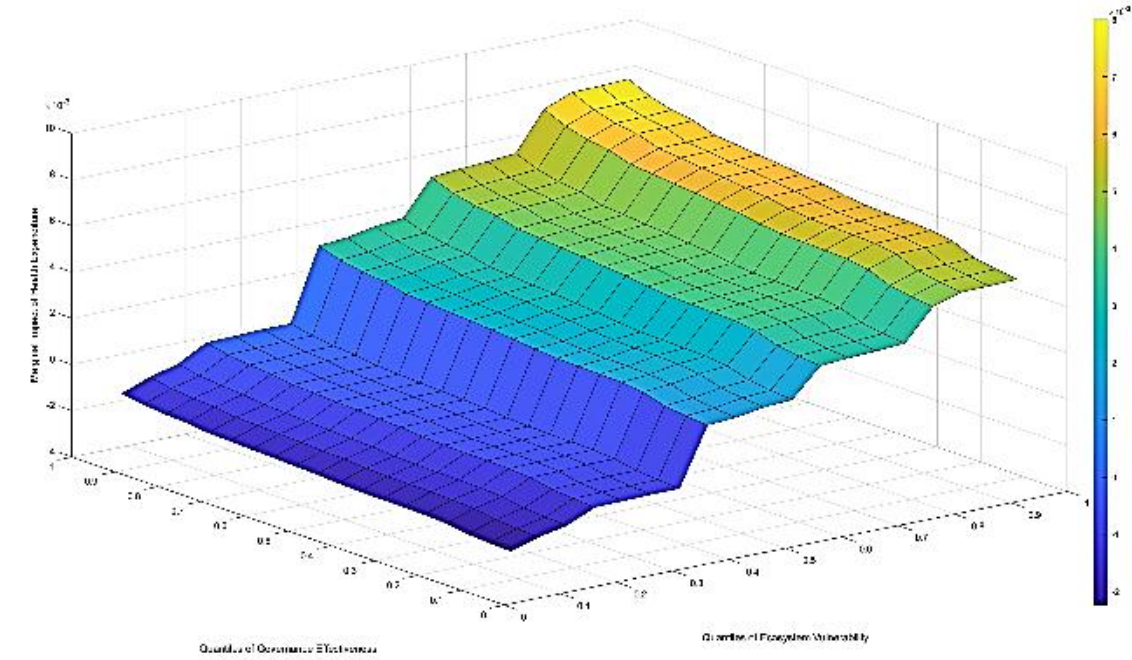

Governance Quality Indicator: Governance Effectiveness

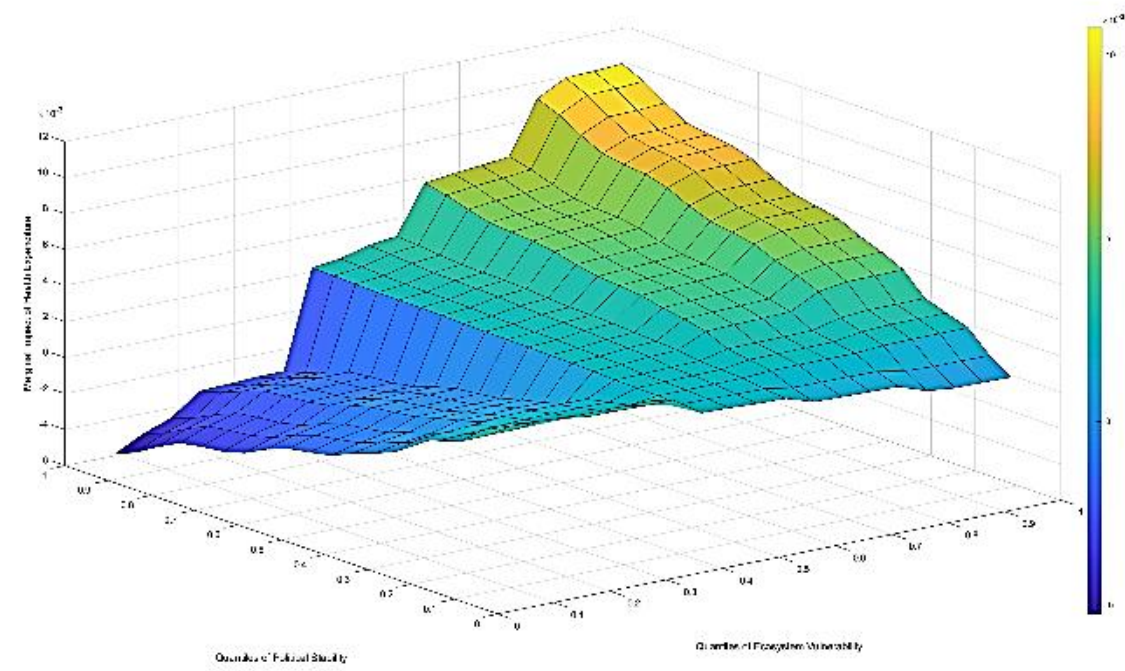

Governance Quality Indicator: Political Stability

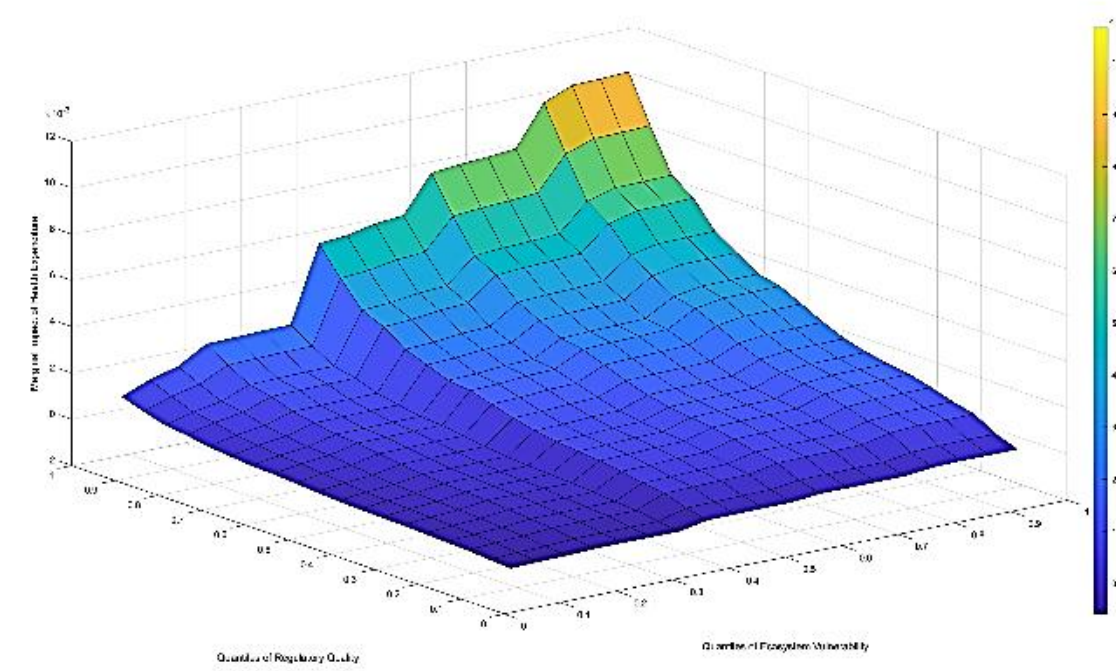

Governance Quality Indicator: Regulatory Quality

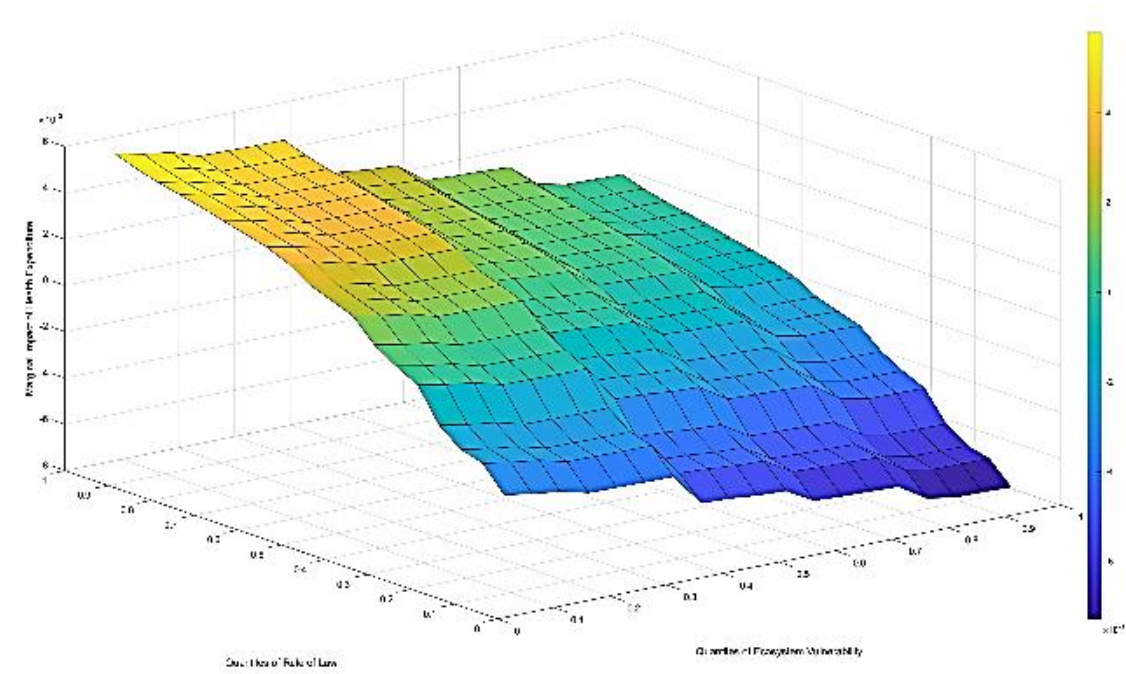

Governance Quality Indicator: Rule of Law

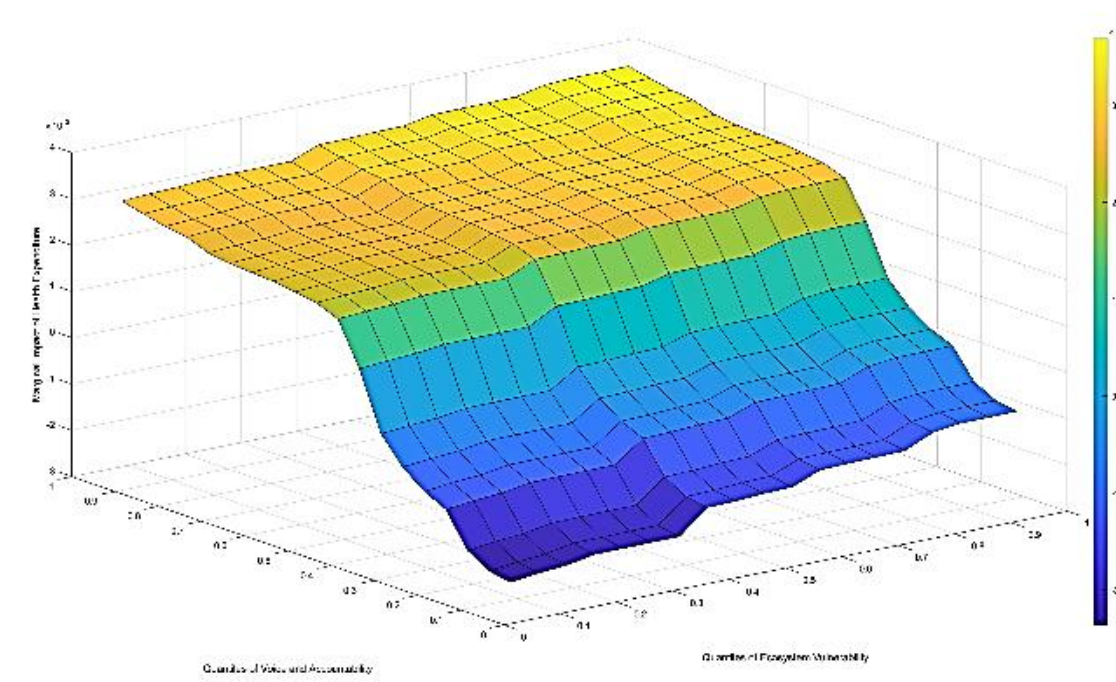

Governance Quality Indicator: Voice and Accountability

Figure S28: BRICS Policy Simulation Outcomes for the Marginal Impact of Health Expenditure on SDG 7.1.1 under Ecosystem Vulnerability and Governance Quality, Related to Figure 4-5 and Table 2

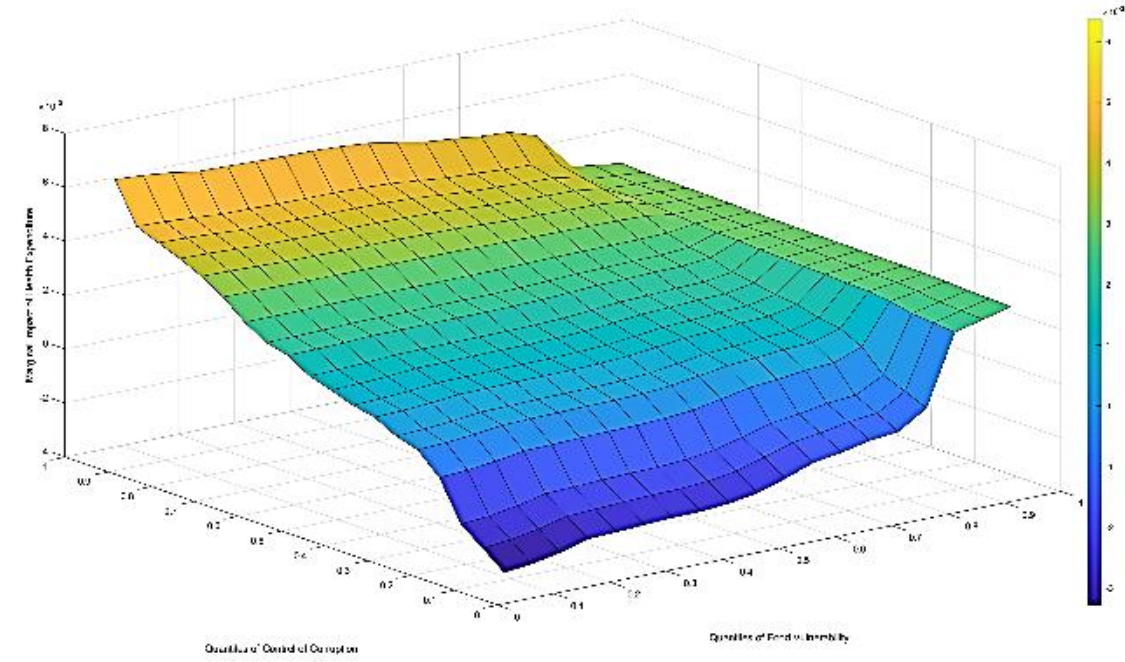

Governance Quality Indicator: Control of Corruption

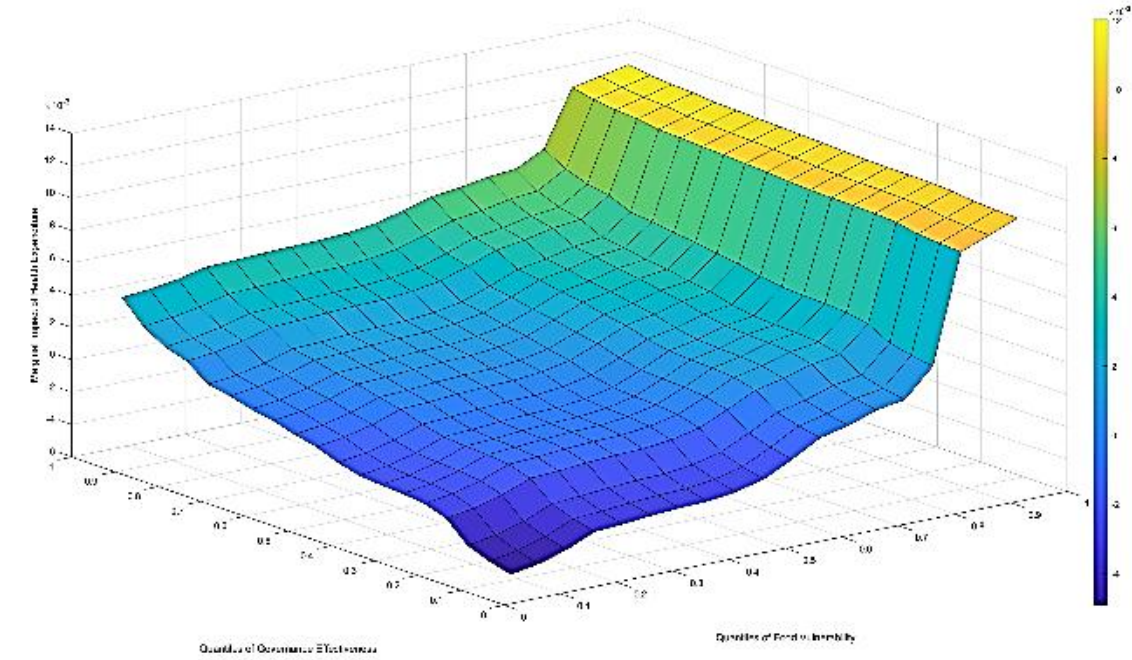

Governance Quality Indicator: Governance Effectiveness

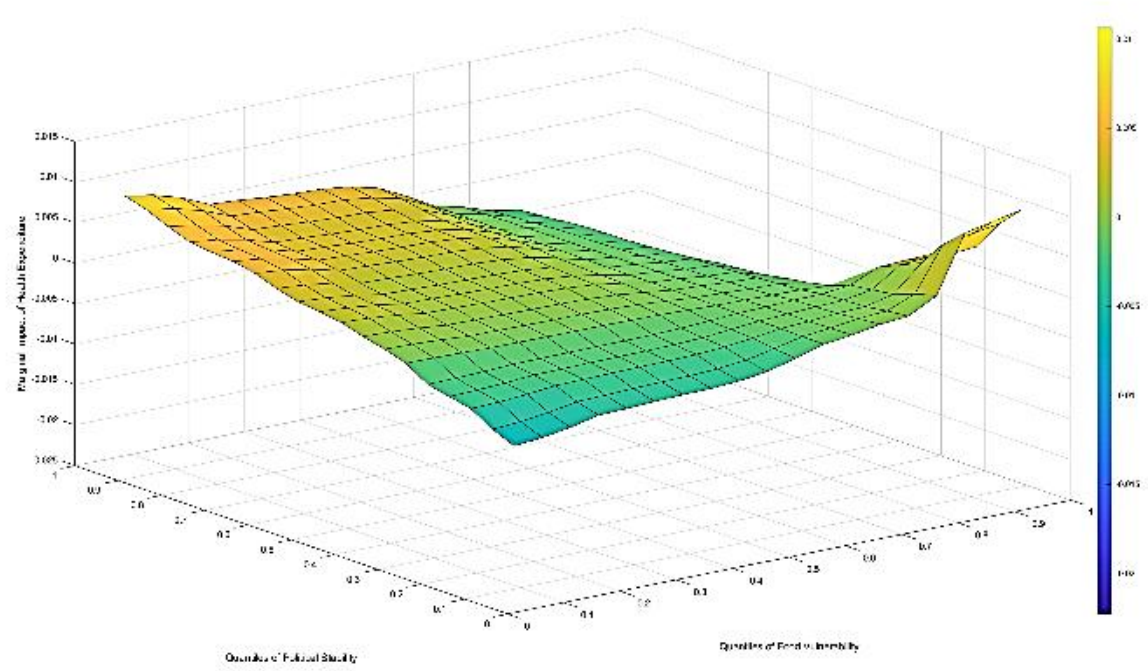

Governance Quality Indicator: Political Stability

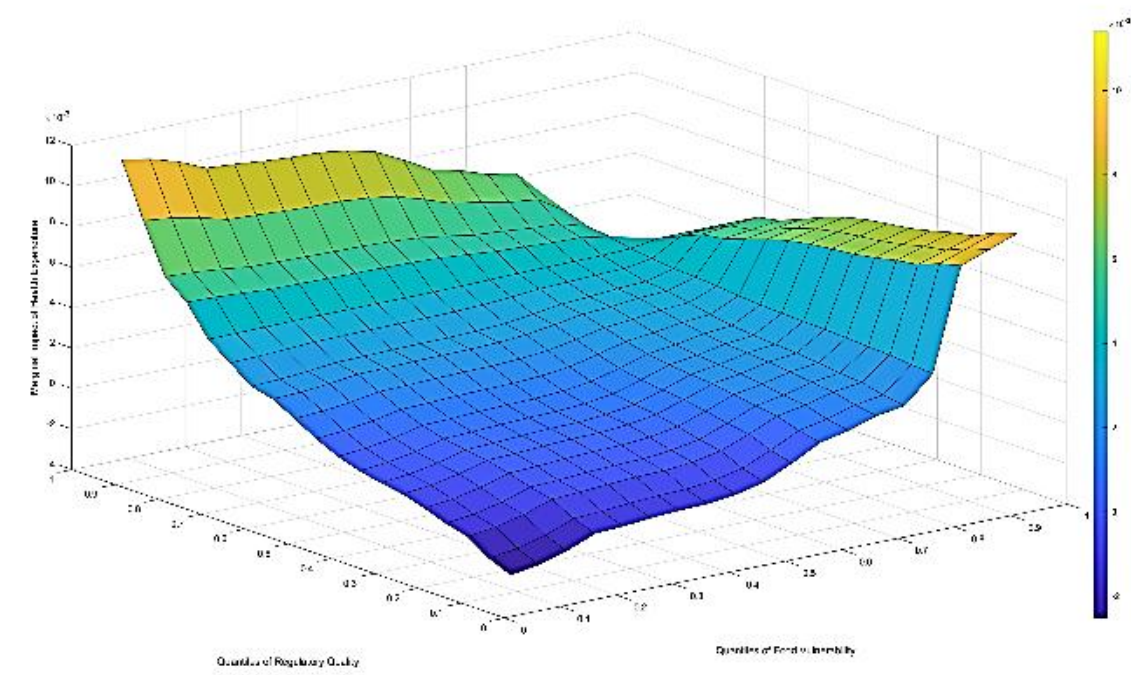

Governance Quality Indicator: Regulatory Quality

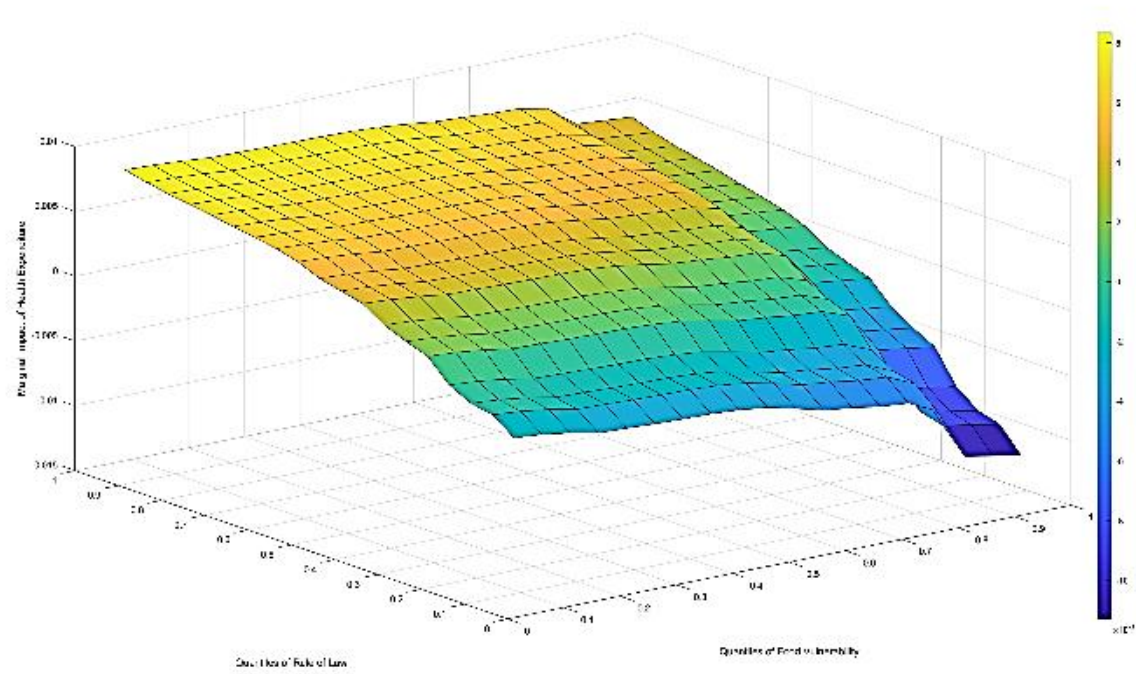

Governance Quality Indicator: Rule of Law

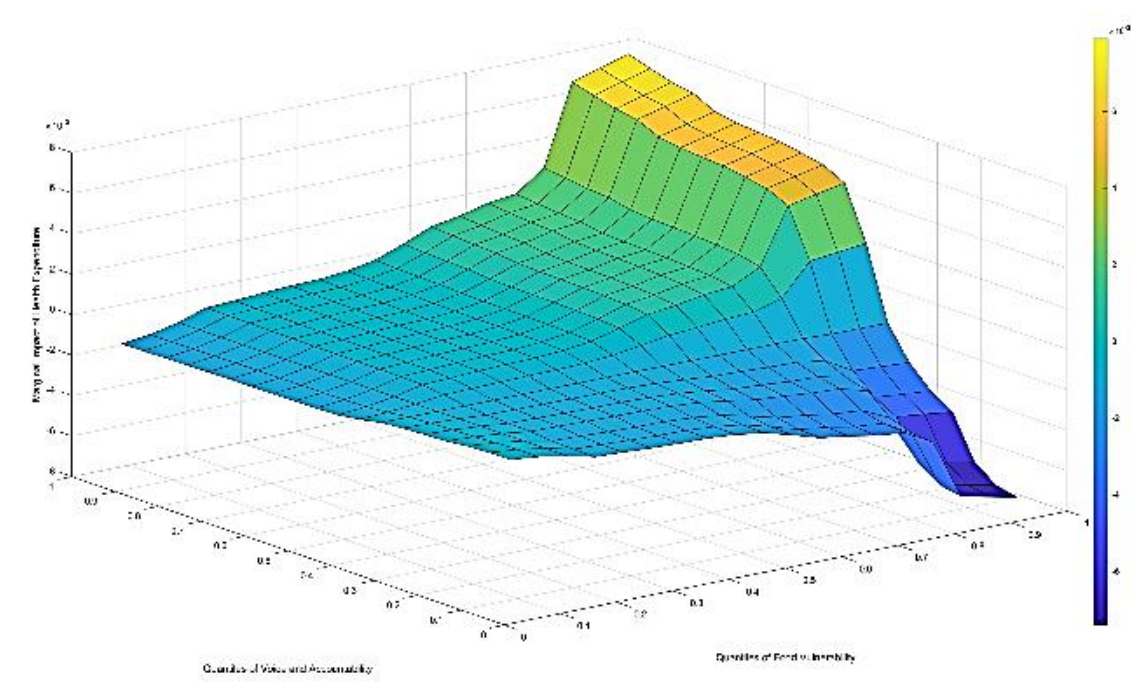

Governance Quality Indicator: Voice and Accountability

Figure S29: BRICS Policy Simulation Outcomes for the Marginal Impact of Health Expenditure on SDG 7.1.1 under Food Vulnerability and Governance Quality, Related to Figure 4-5 and Table 2

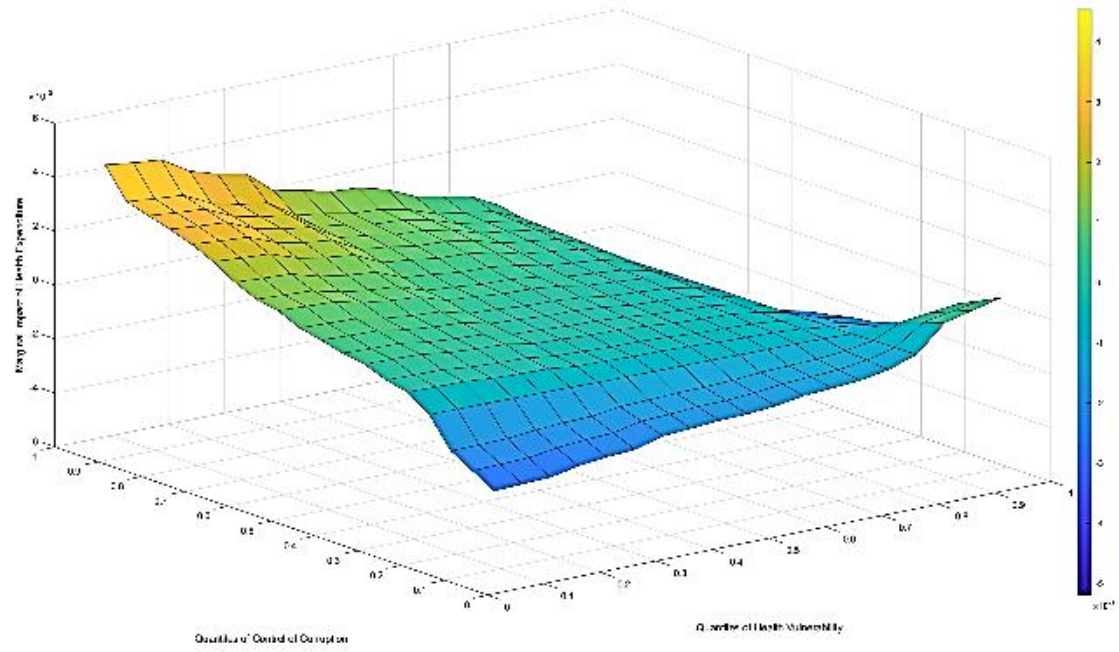

Governance Quality Indicator: Control of Corruption

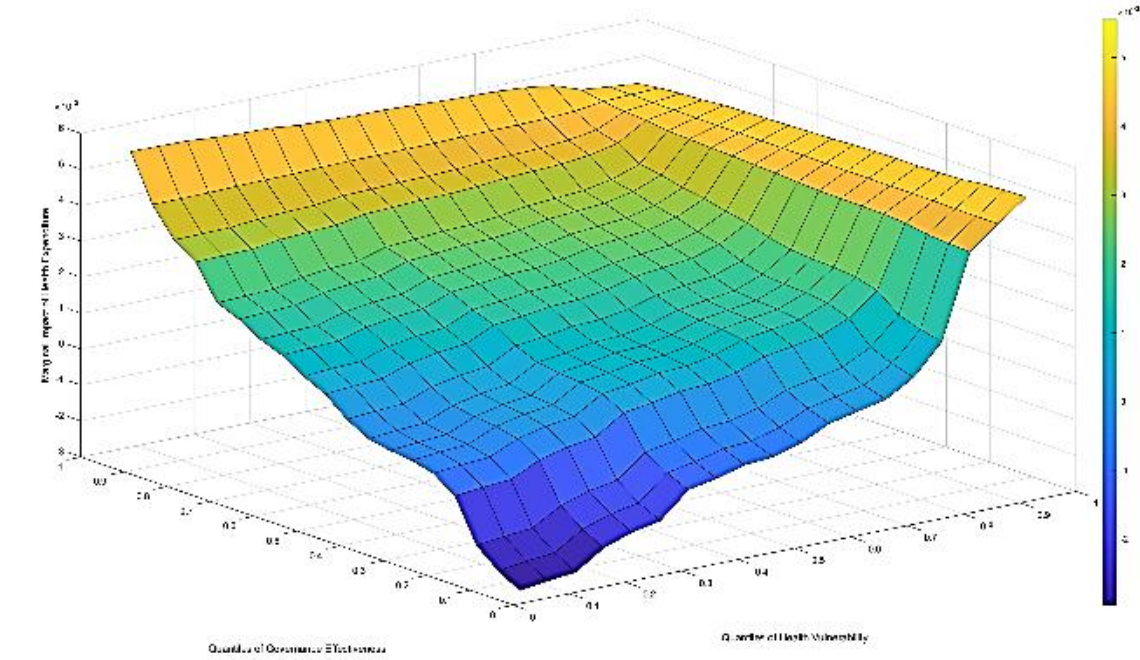

Governance Quality Indicator: Governance Effectiveness

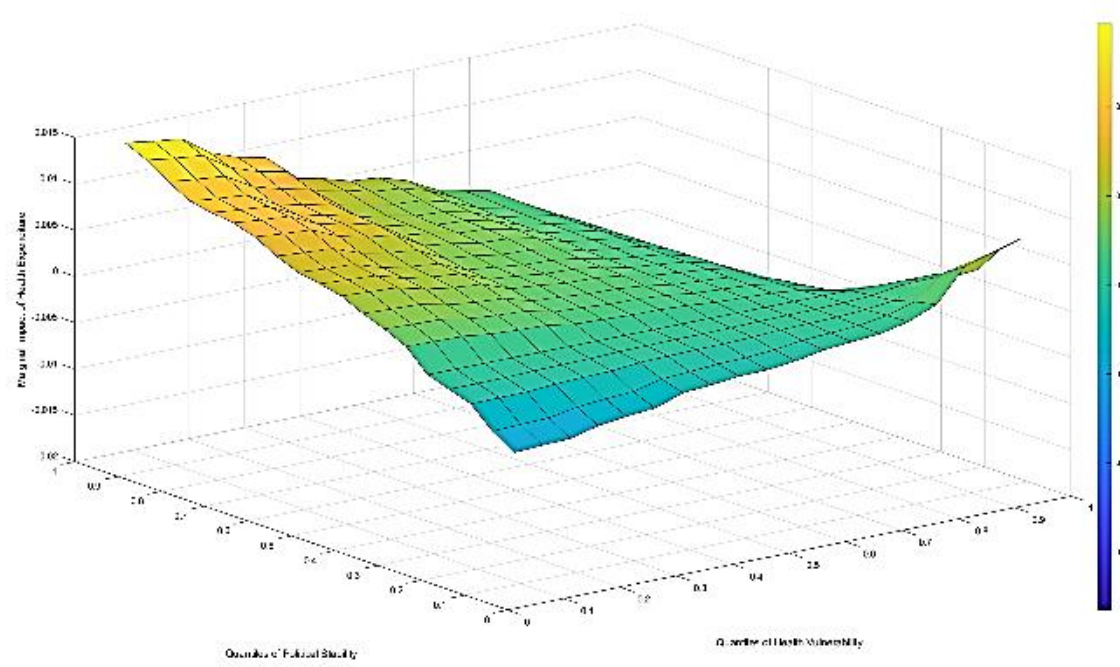

Governance Quality Indicator: Political Stability

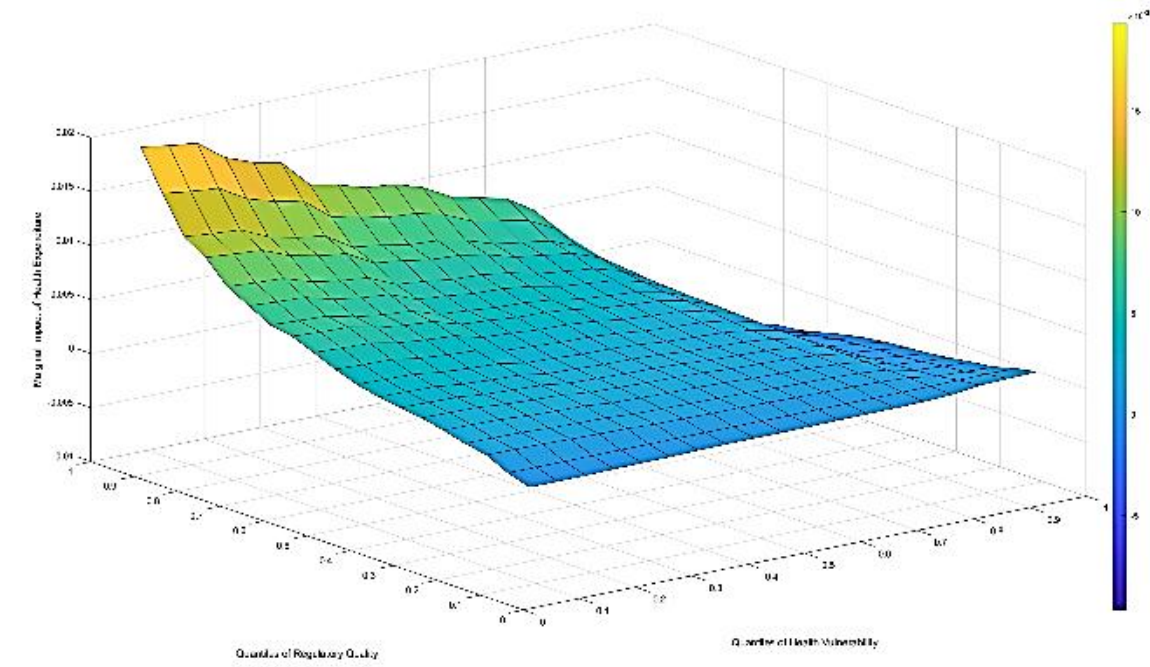

Governance Quality Indicator: Regulatory Quality

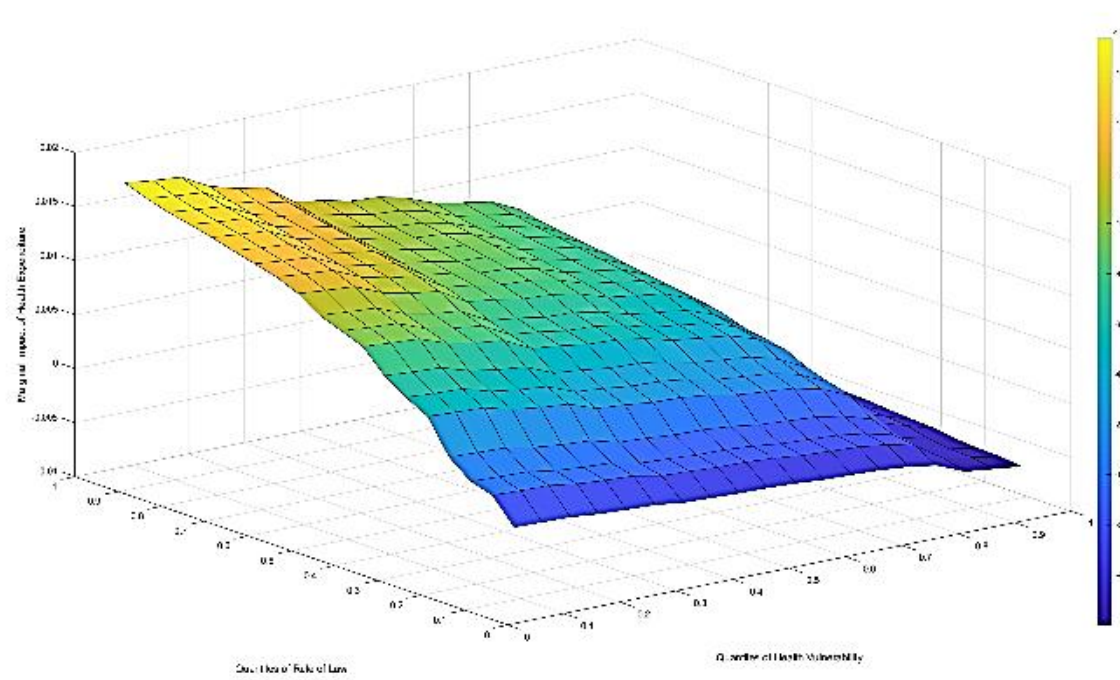

Governance Quality Indicator: Rule of Law

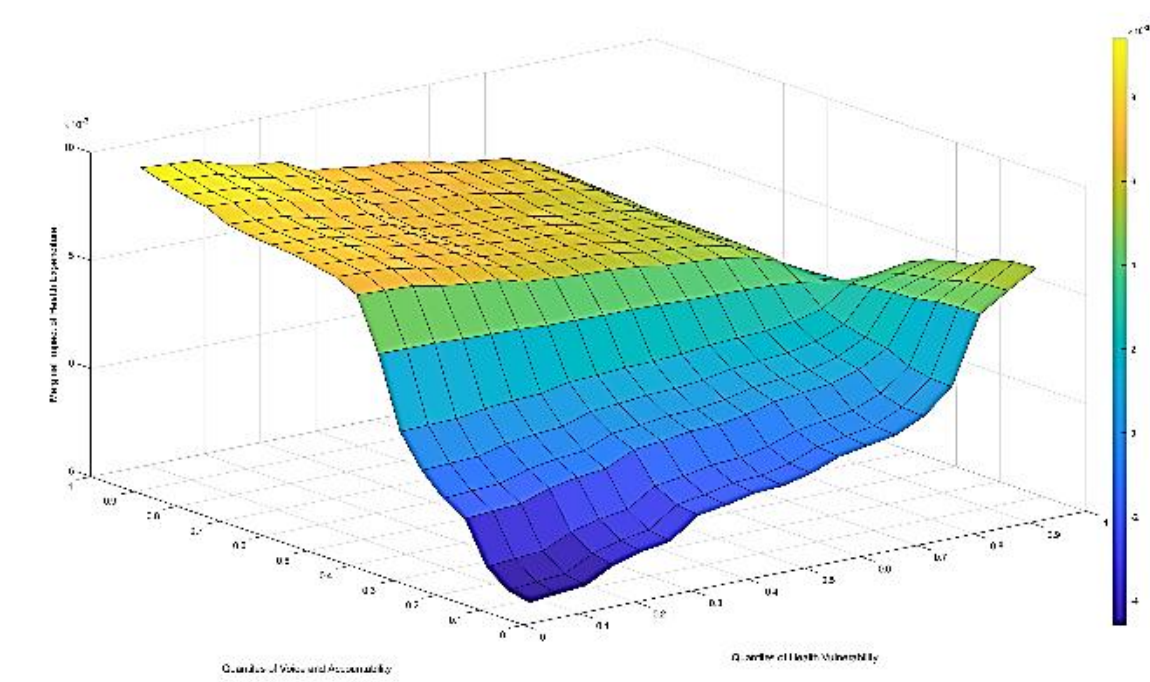

Governance Quality Indicator: Voice and Accountability

Figure S30: BRICS Policy Simulation Outcomes for the Marginal Impact of Health Expenditure on SDG 7.1.1 under Health Vulnerability and Governance Quality, Related to Figure 4-5 and Table 2

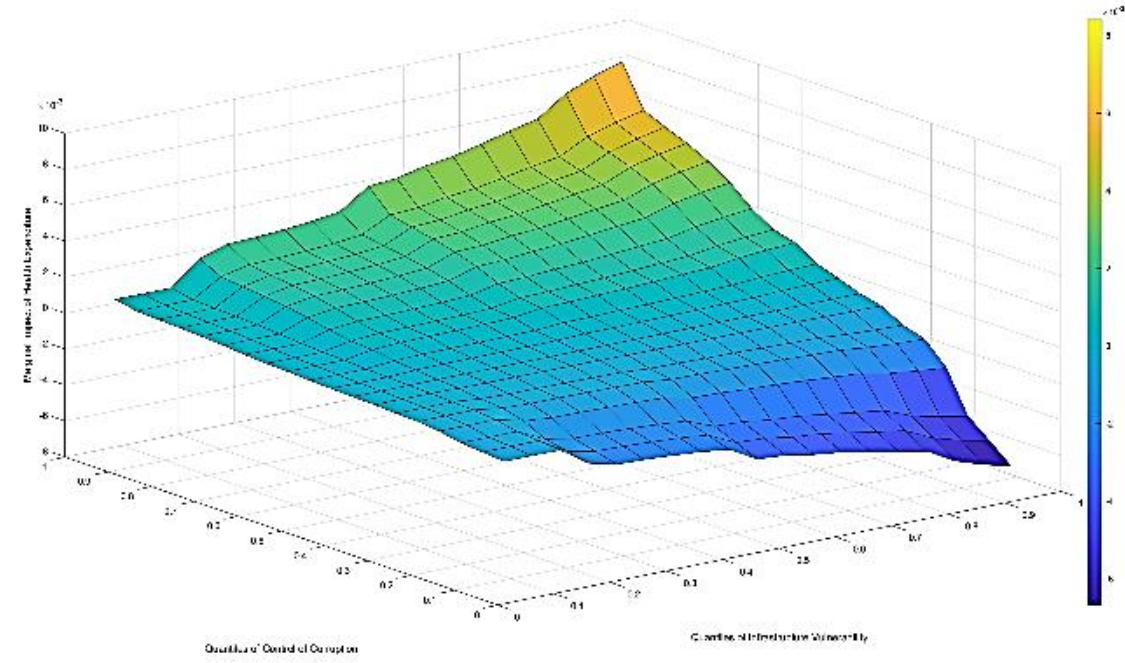

Governance Quality Indicator: Control of Corruption

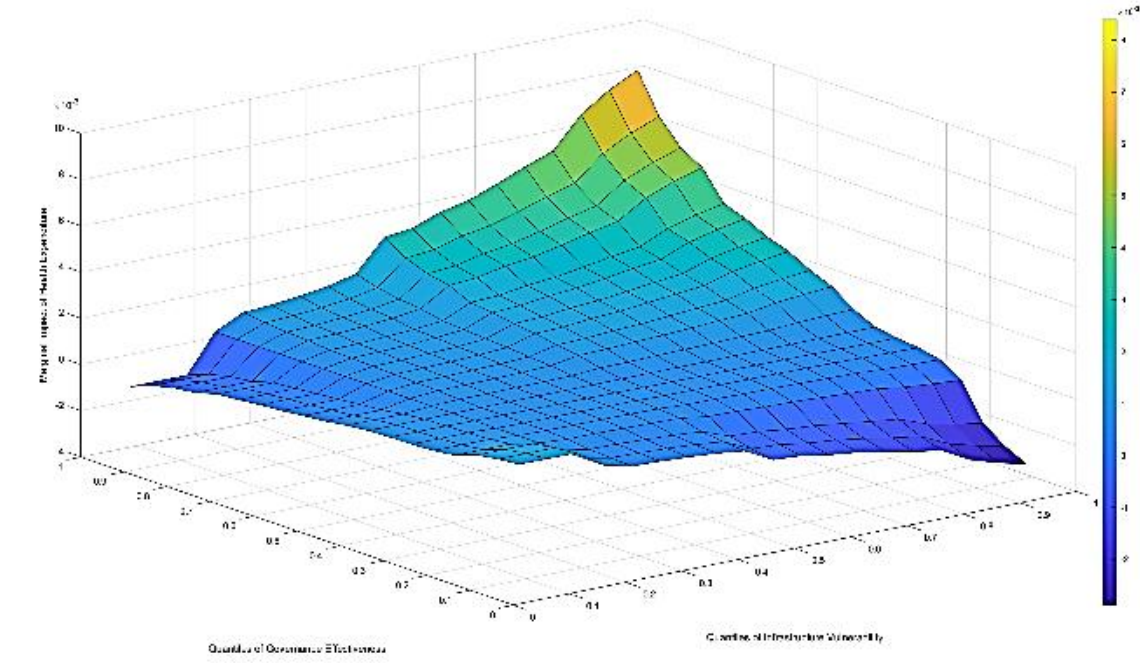

Governance Quality Indicator: Governance Effectiveness

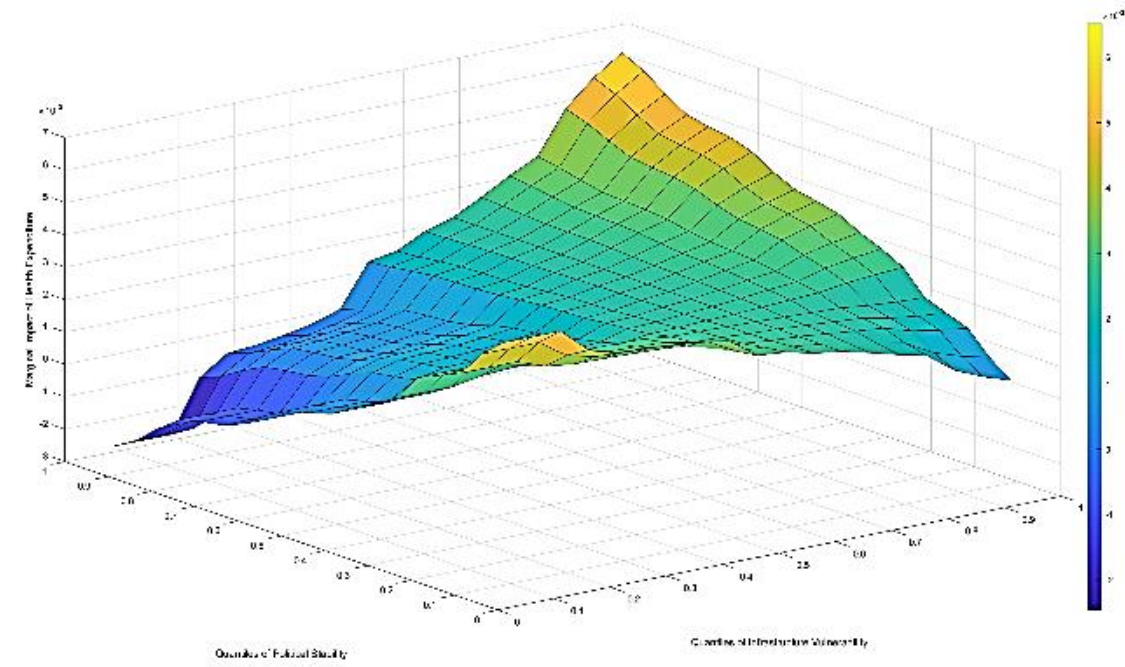

Governance Quality Indicator: Political Stability

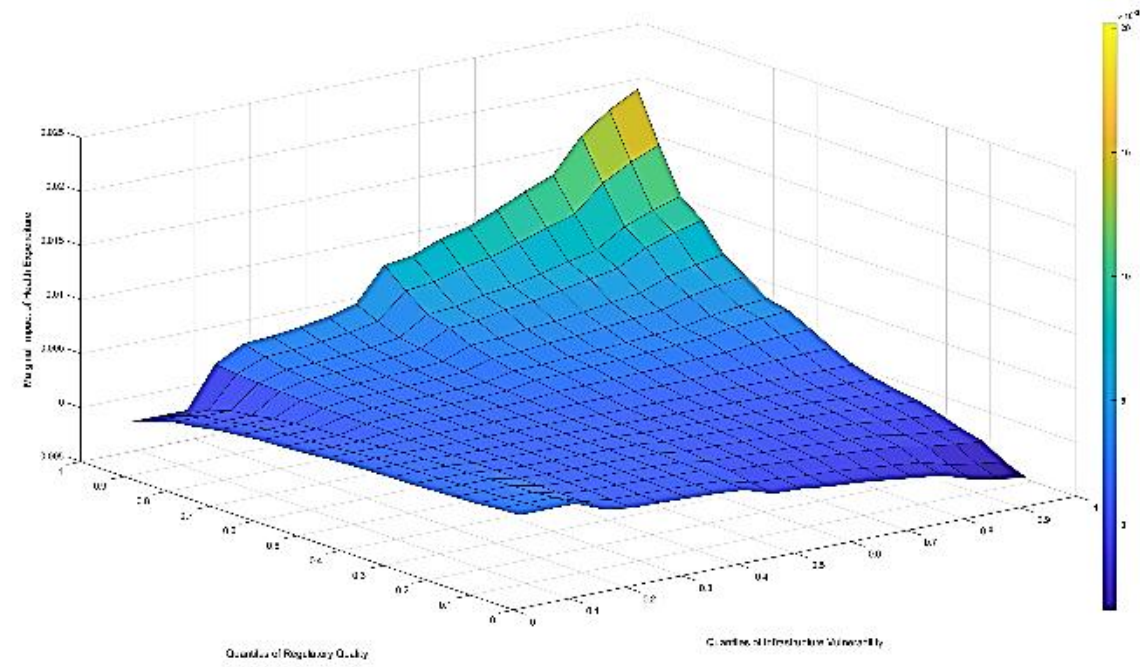

Governance Quality Indicator: Regulatory Quality

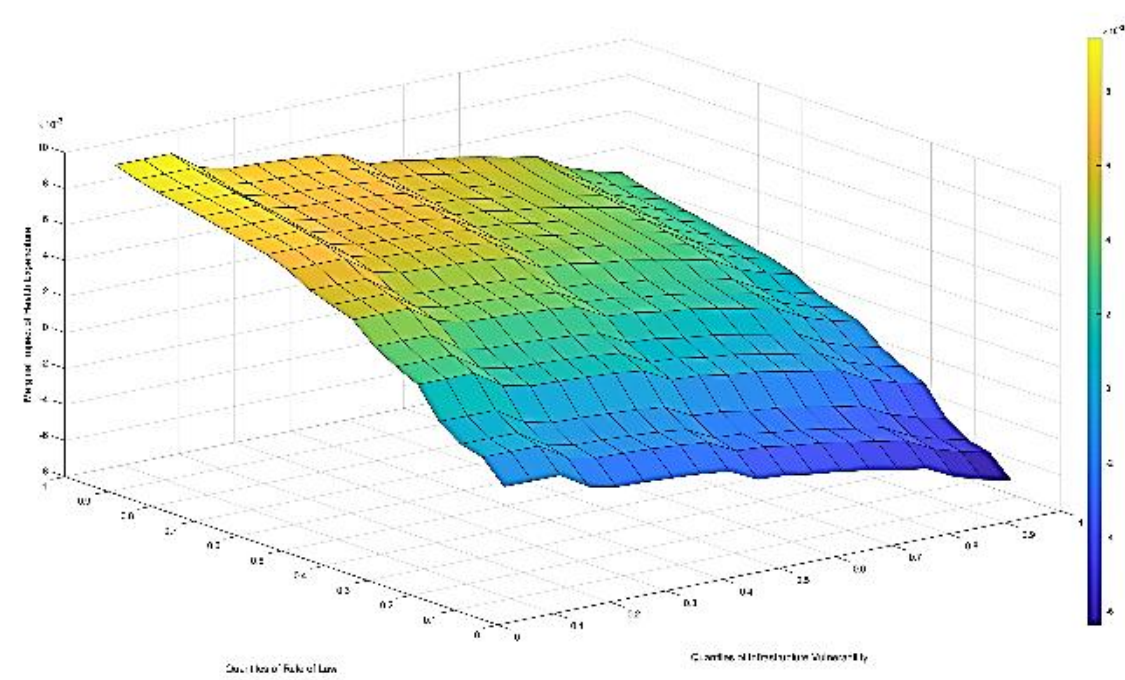

Governance Quality Indicator: Rule of Law

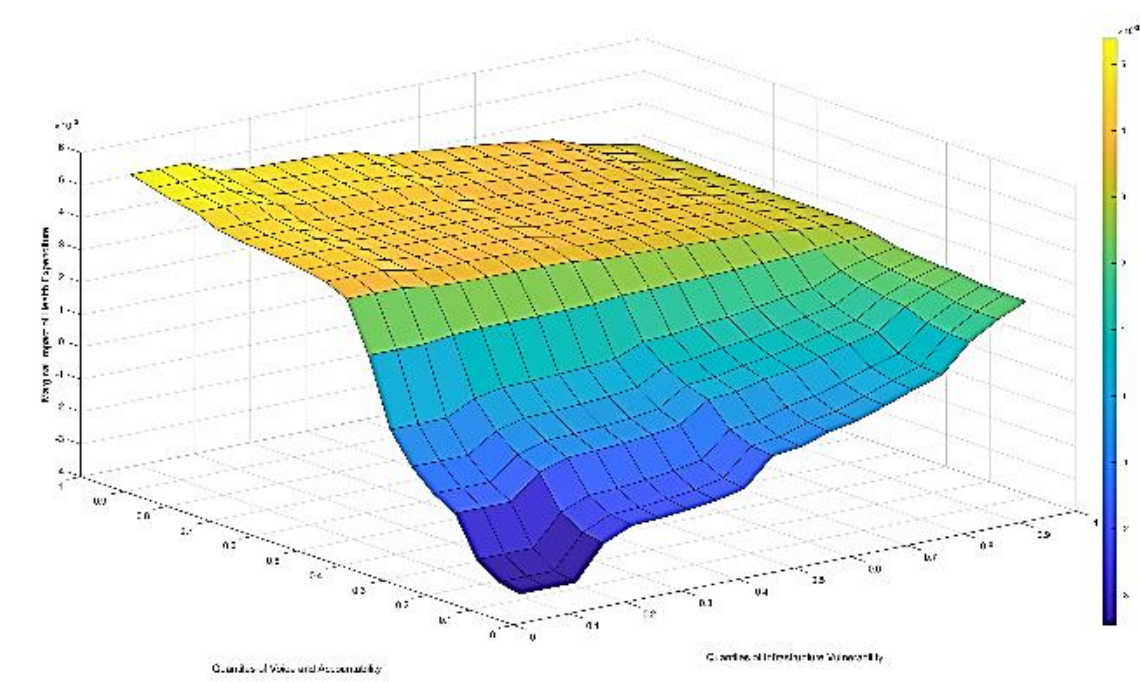

Governance Quality Indicator: Voice and Accountability

Figure S31: BRICS Policy Simulation Outcomes for the Marginal Impact of Health Expenditure on SDG 7.1.1 under Infrastructure Vulnerability and Governance Quality, Related to Figure 4-5 and Table 2

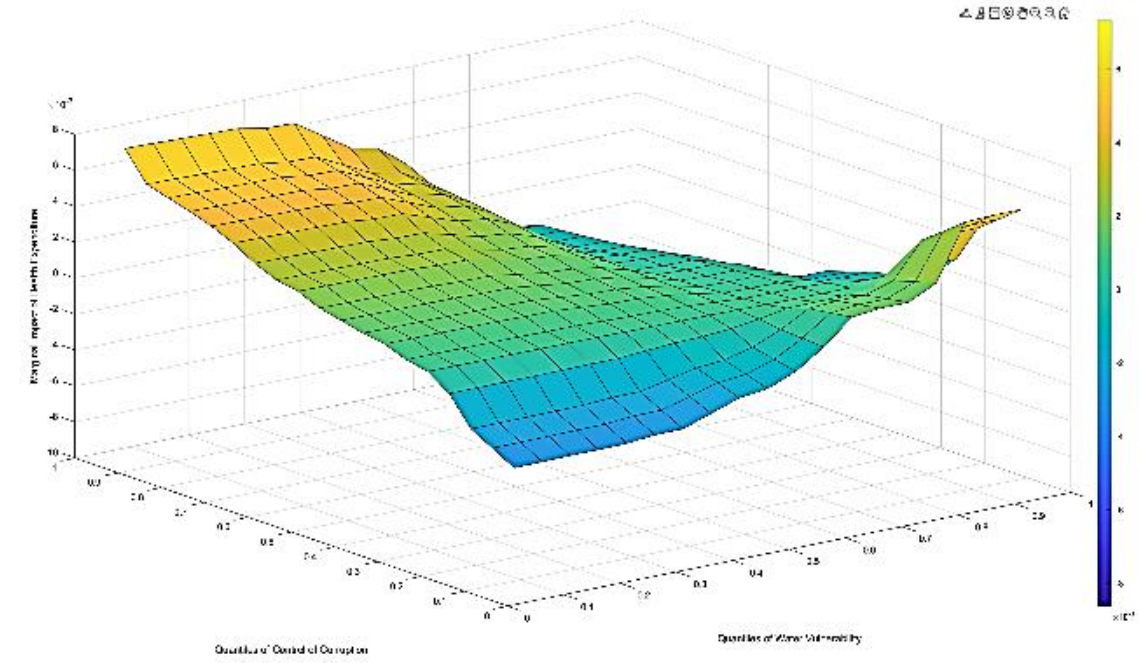

Governance Quality Indicator: Control of Corruption

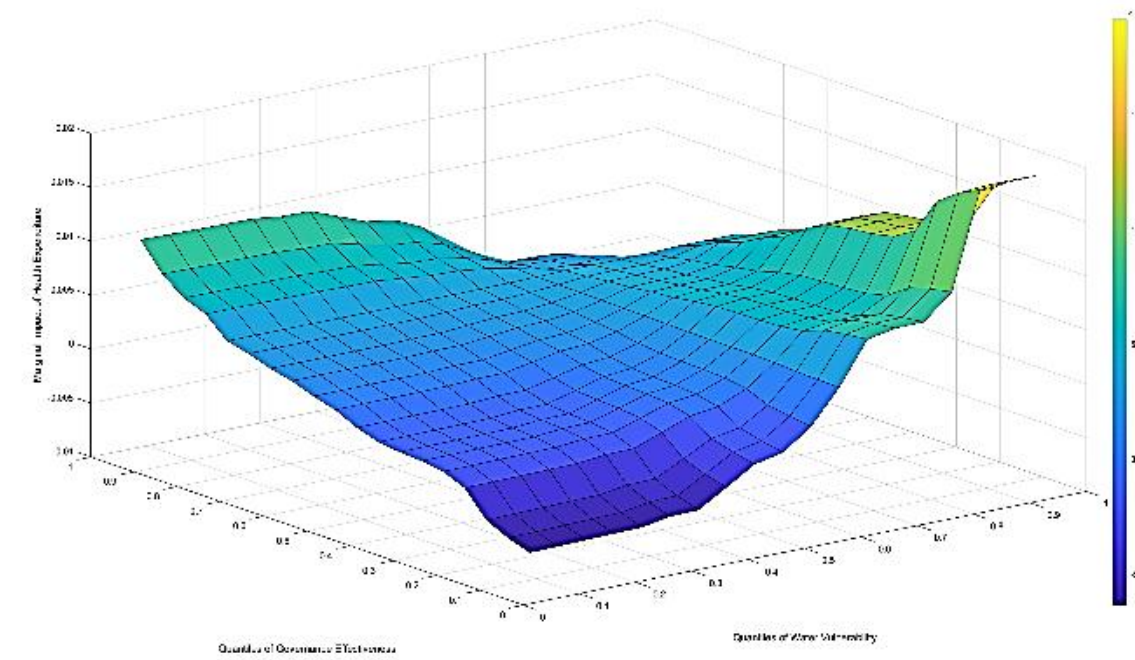

Governance Quality Indicator: Governance Effectiveness

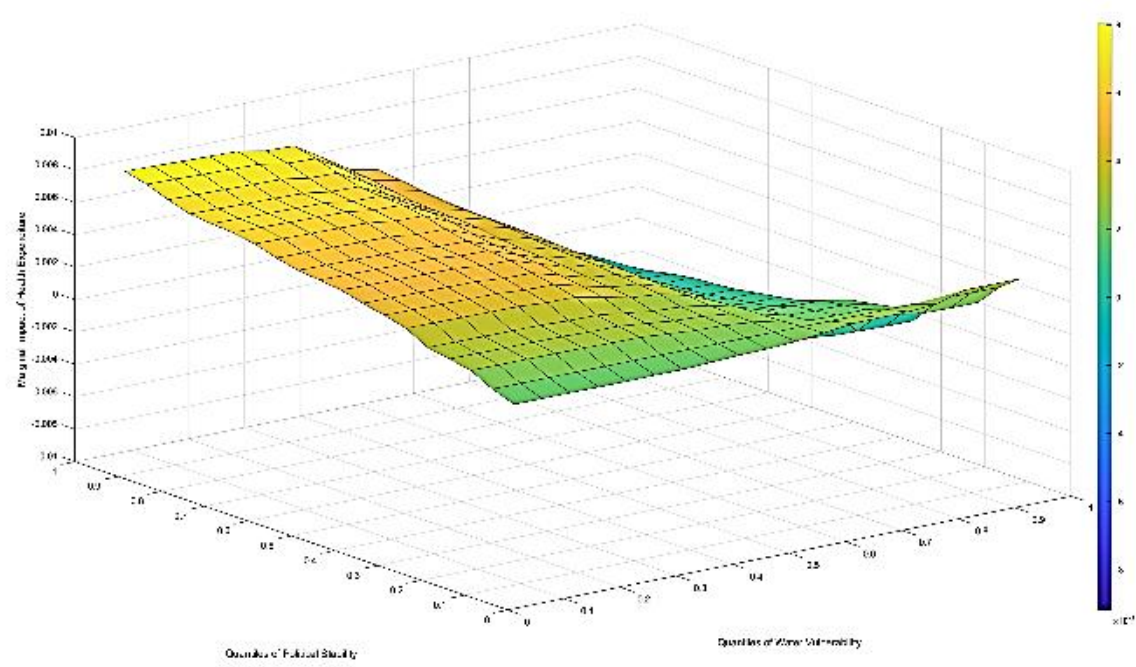

Governance Quality Indicator: Political Stability

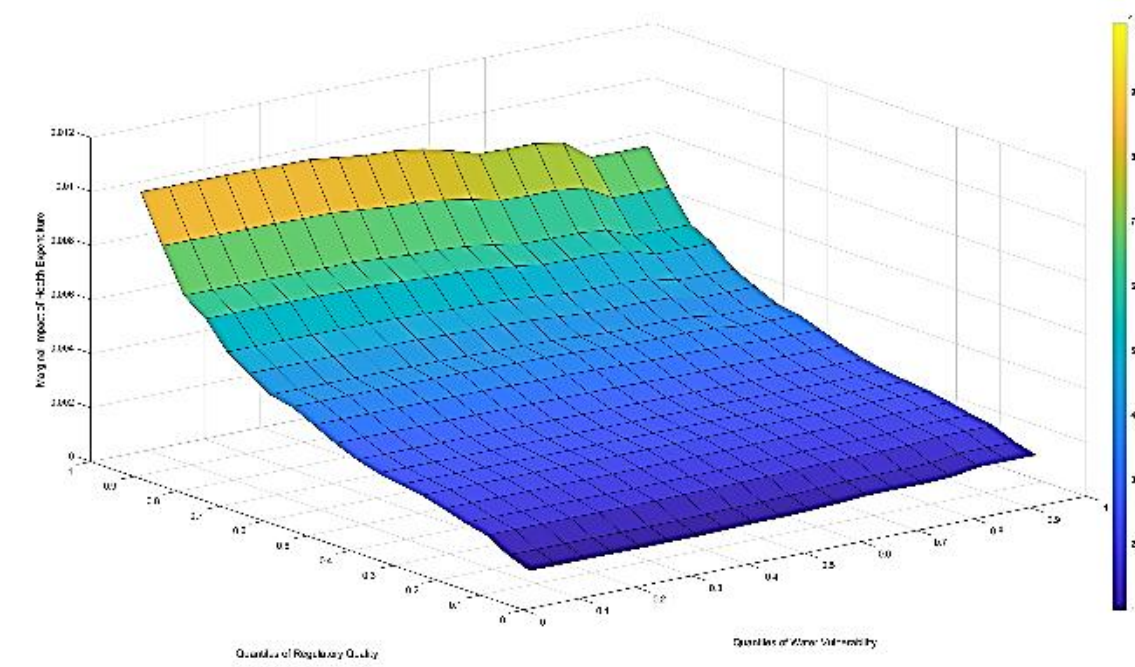

Governance Quality Indicator: Regulatory Quality

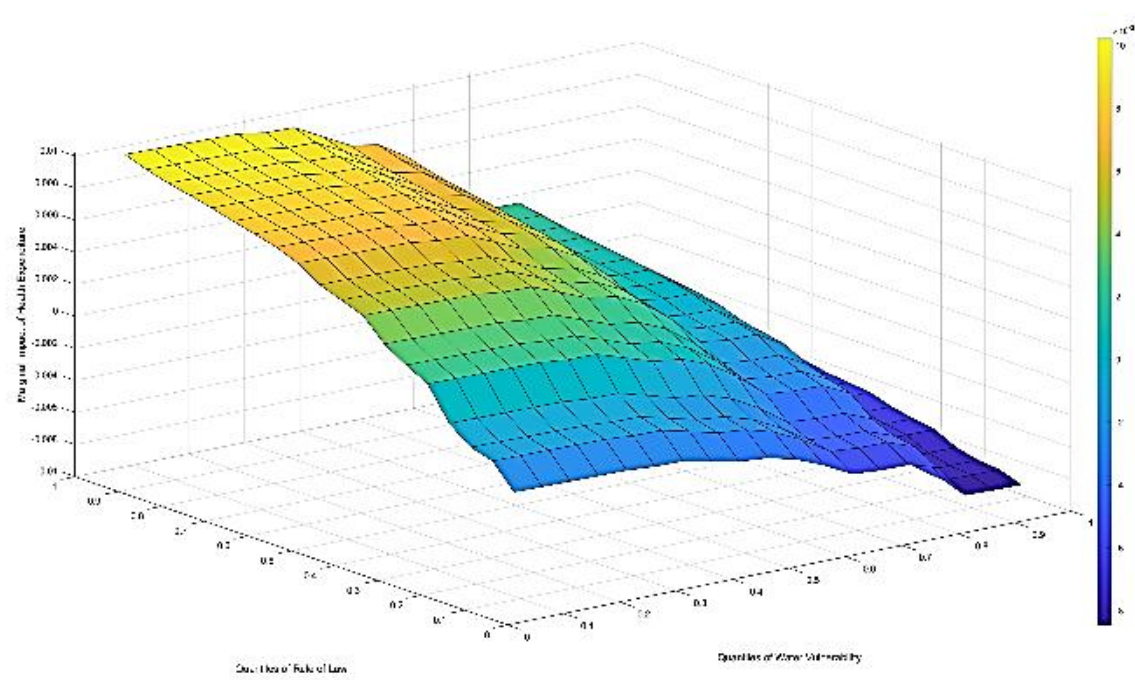

Governance Quality Indicator: Rule of Law

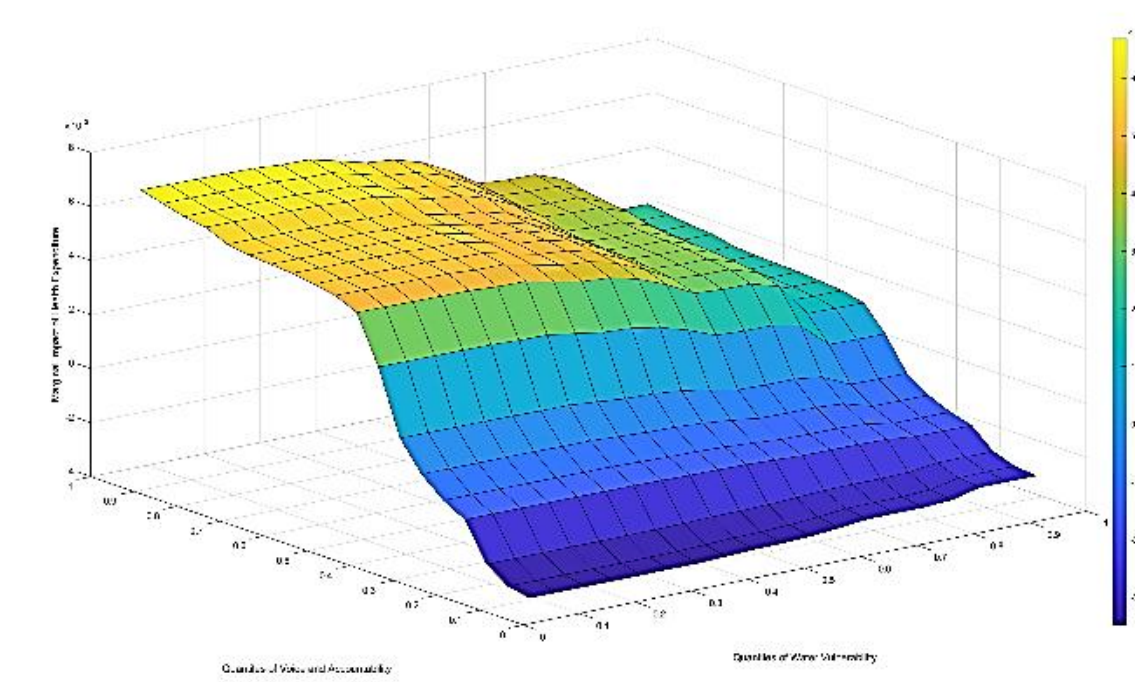

Governance Quality Indicator: Voice and Accountability

Figure S32: BRICS Policy Simulation Outcomes for the Marginal Impact of Health Expenditure on SDG 7.1.1 under Water Vulnerability and Governance Quality, Related to Figure 4-5 and Table 2

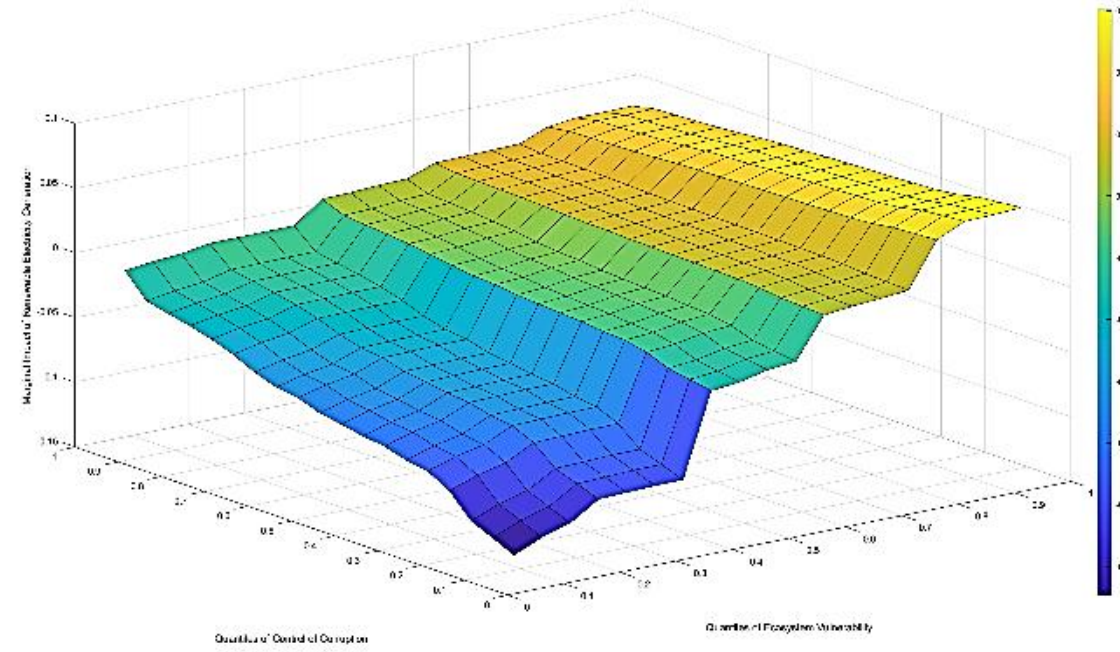

Governance Quality Indicator: Control of Corruption

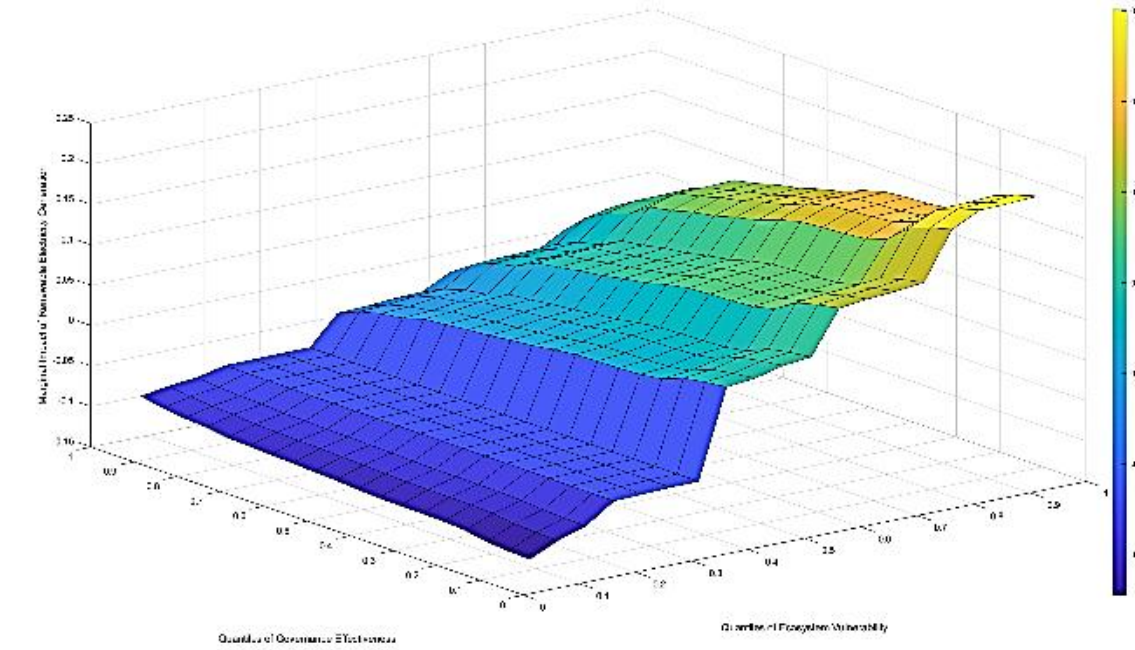

Governance Quality Indicator: Governance Effectiveness

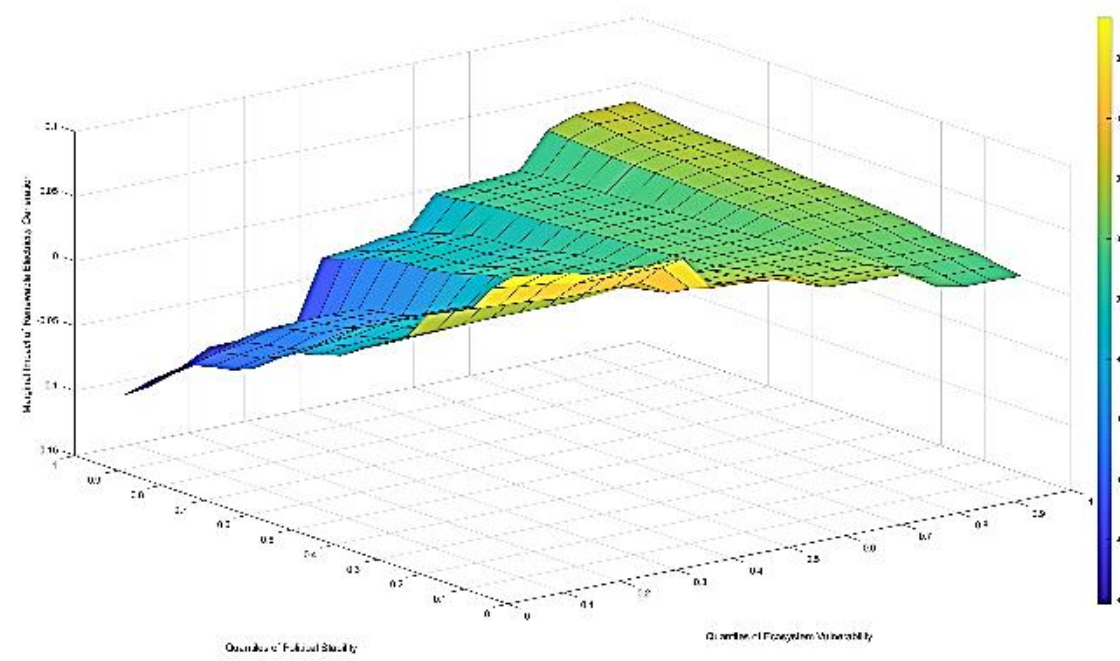

Governance Quality Indicator: Political Stability

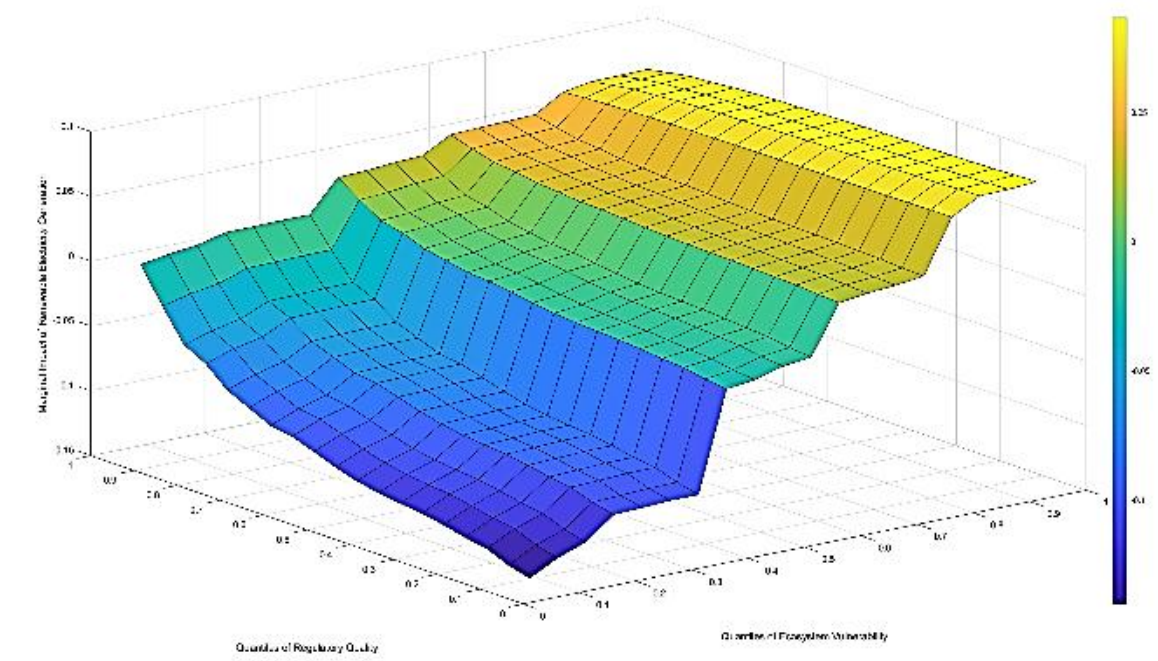

Governance Quality Indicator: Regulatory Quality

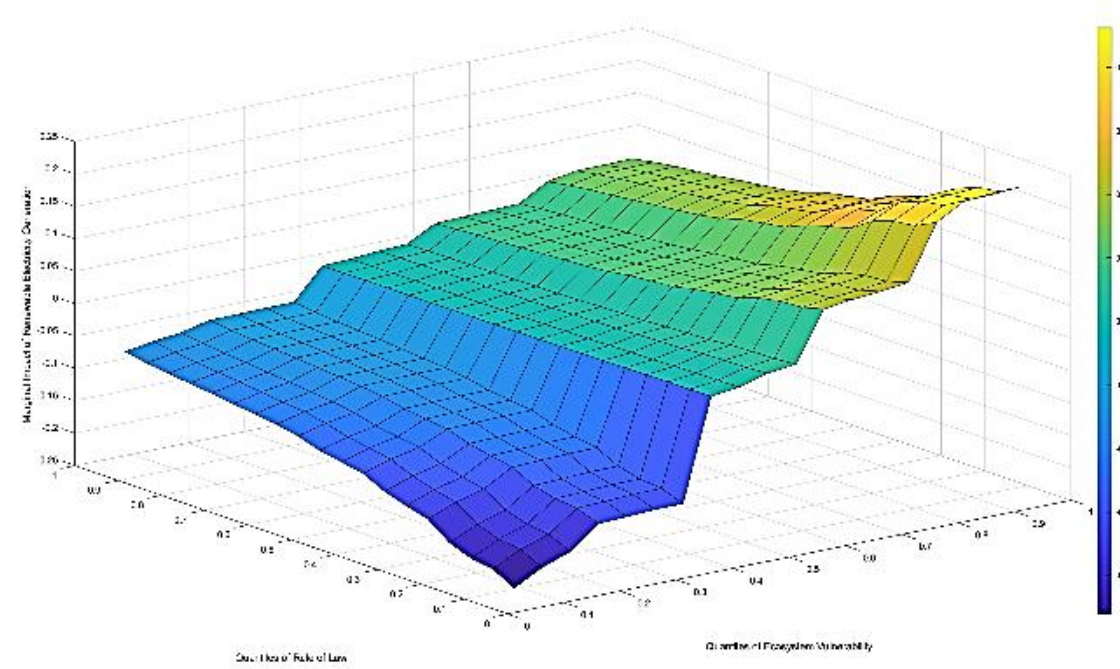

Governance Quality Indicator: Rule of Law

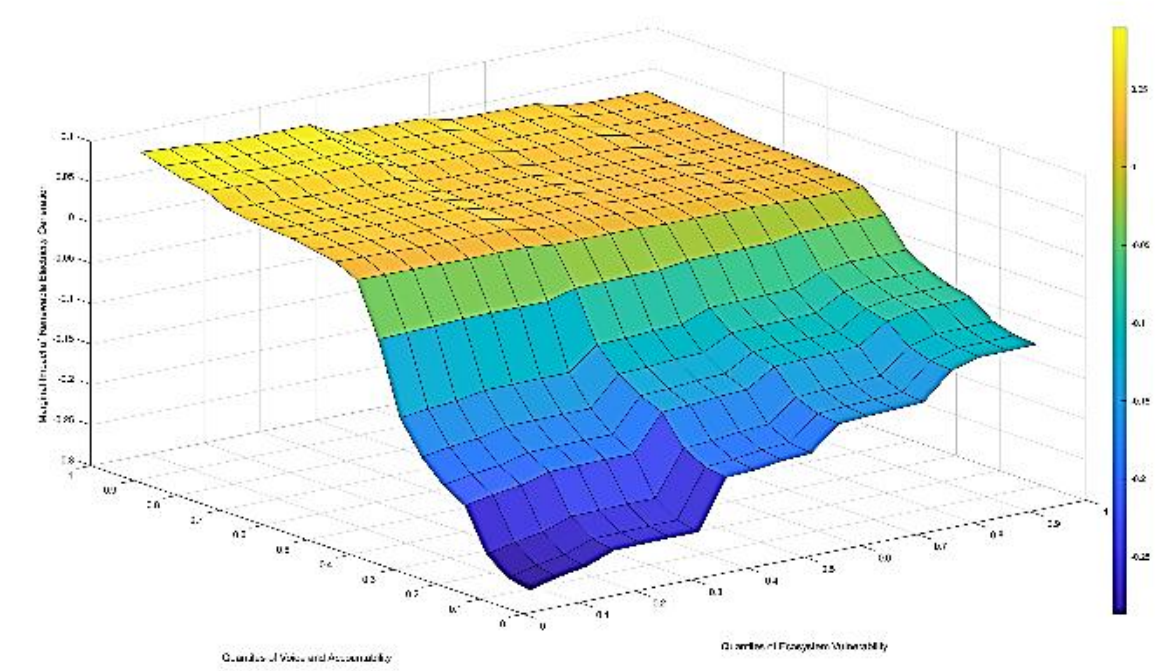

Governance Quality Indicator: Voice and Accountability

Figure S33: BRICS Policy Simulation Outcomes for the Marginal Impact of Renewable Electricity Generation on SDG 7.1.2 under Ecosystem Vulnerability and Governance Quality, Related to Figure 4-5 and Table 2

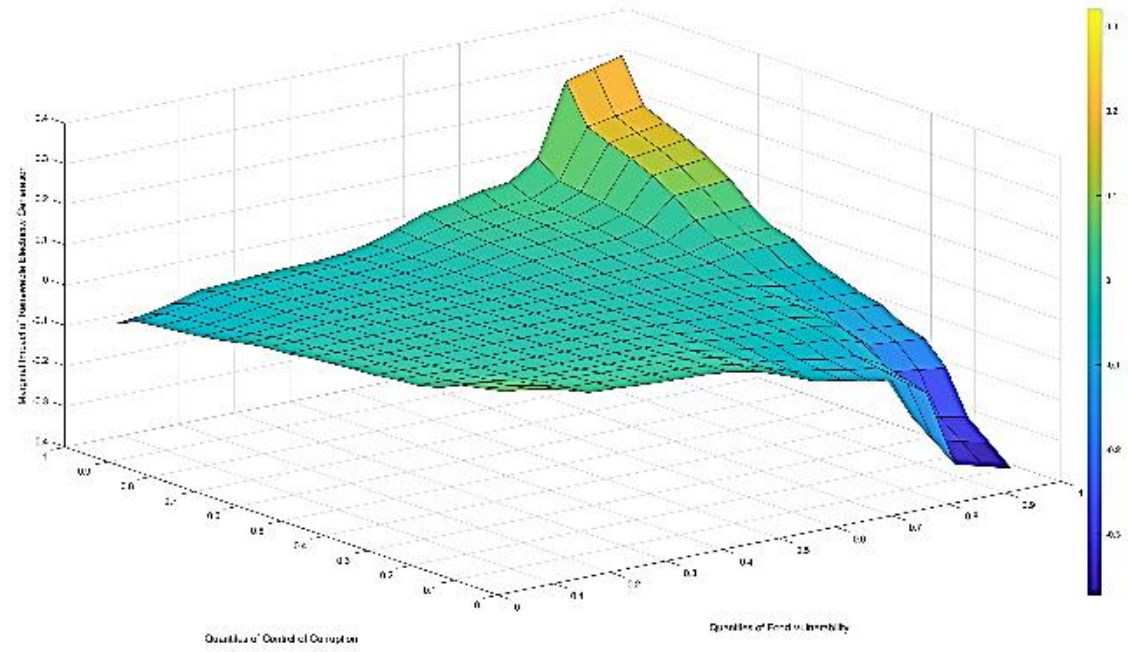

Governance Quality Indicator: Control of Corruption

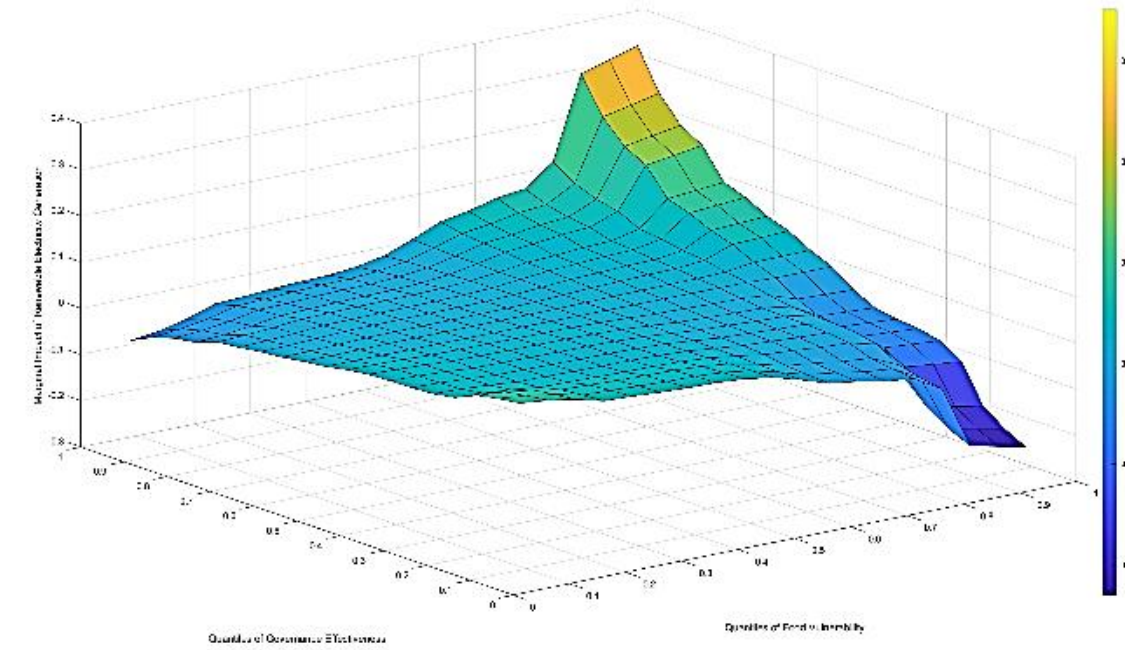

Governance Quality Indicator: Governance Effectiveness

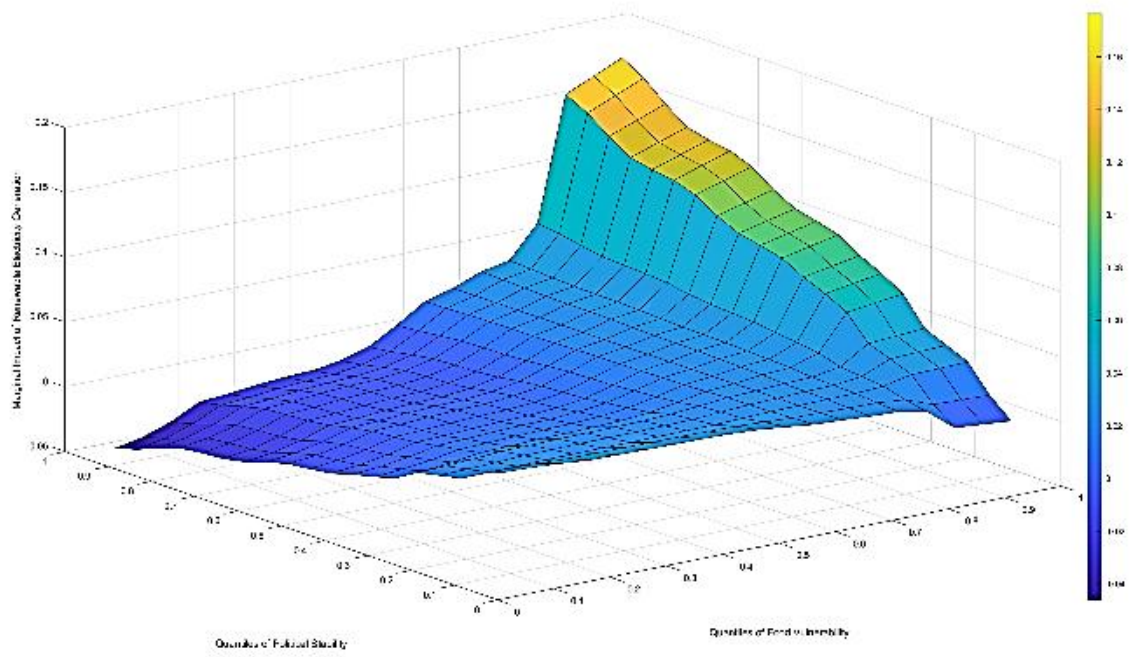

Governance Quality Indicator: Political Stability

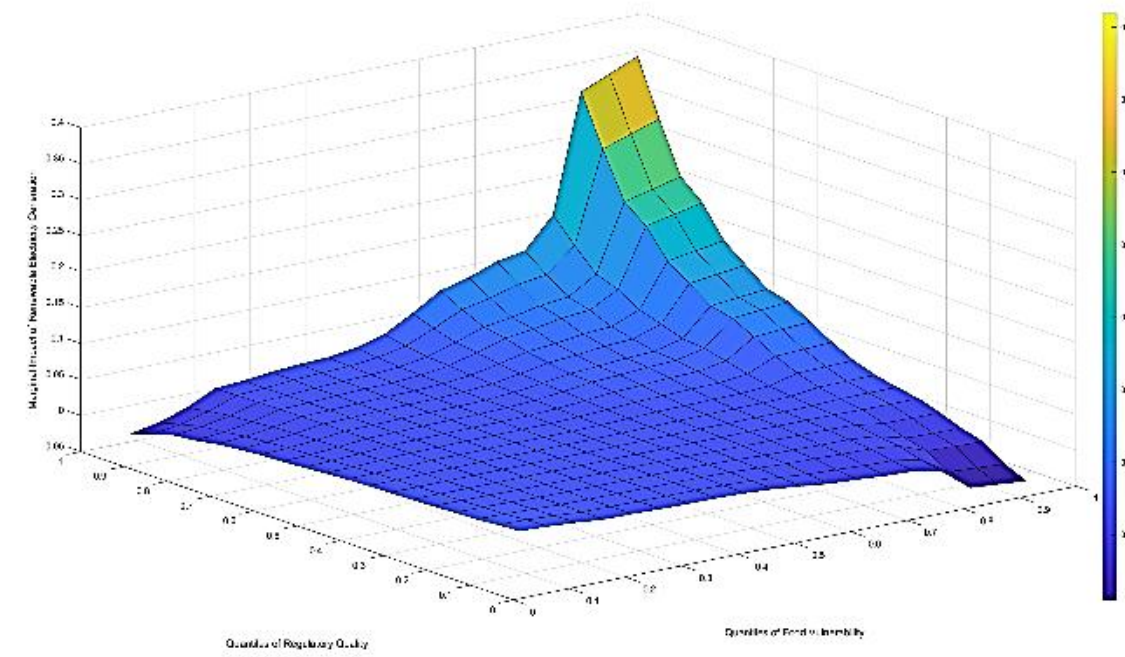

Governance Quality Indicator: Regulatory Quality

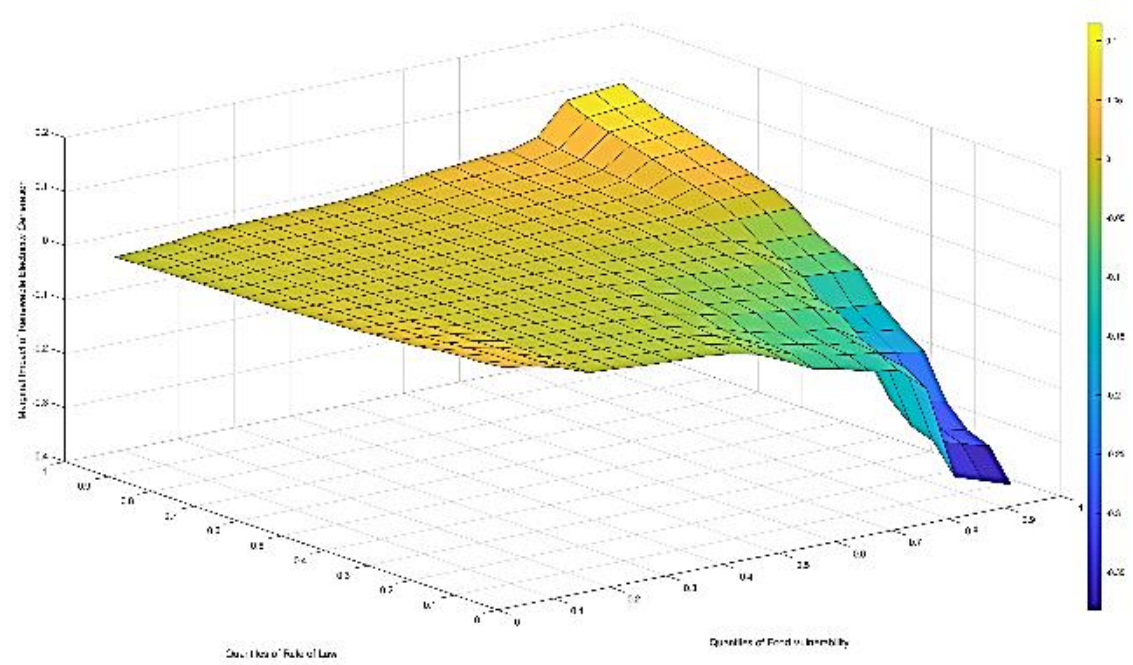

Governance Quality Indicator: Rule of Law

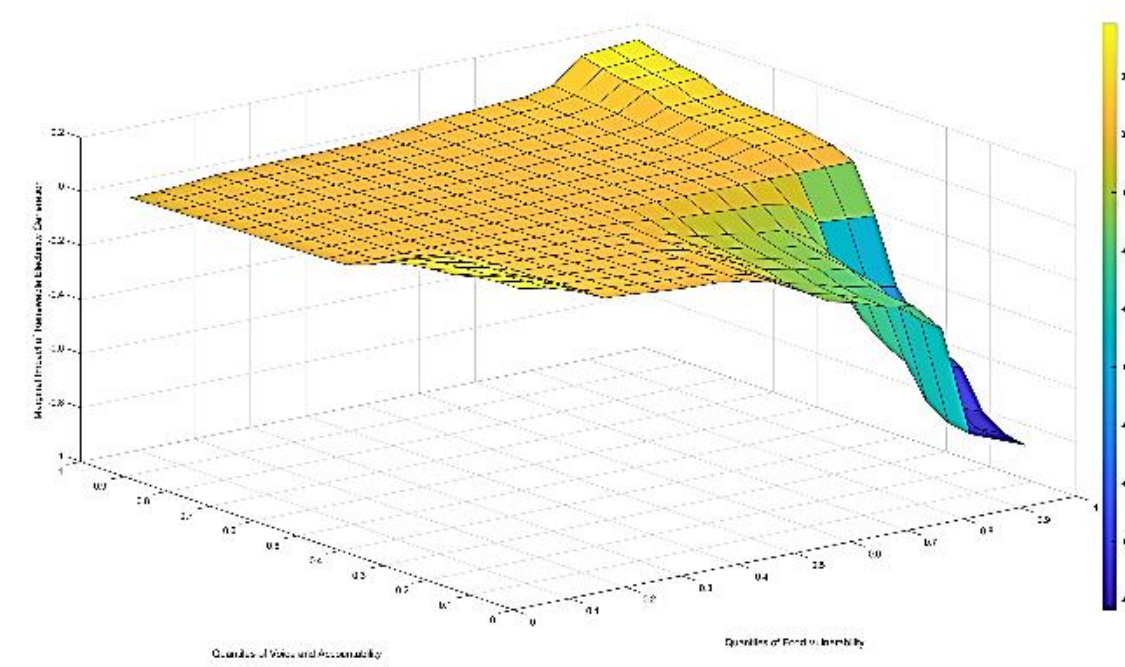

Governance Quality Indicator: Voice and Accountability

Figure S34: BRICS Policy Simulation Outcomes for the Marginal Impact of Renewable Electricity Generation on SDG 7.1.2 under Food Vulnerability and Governance Quality, Related to Figure 4-5 and Table 2

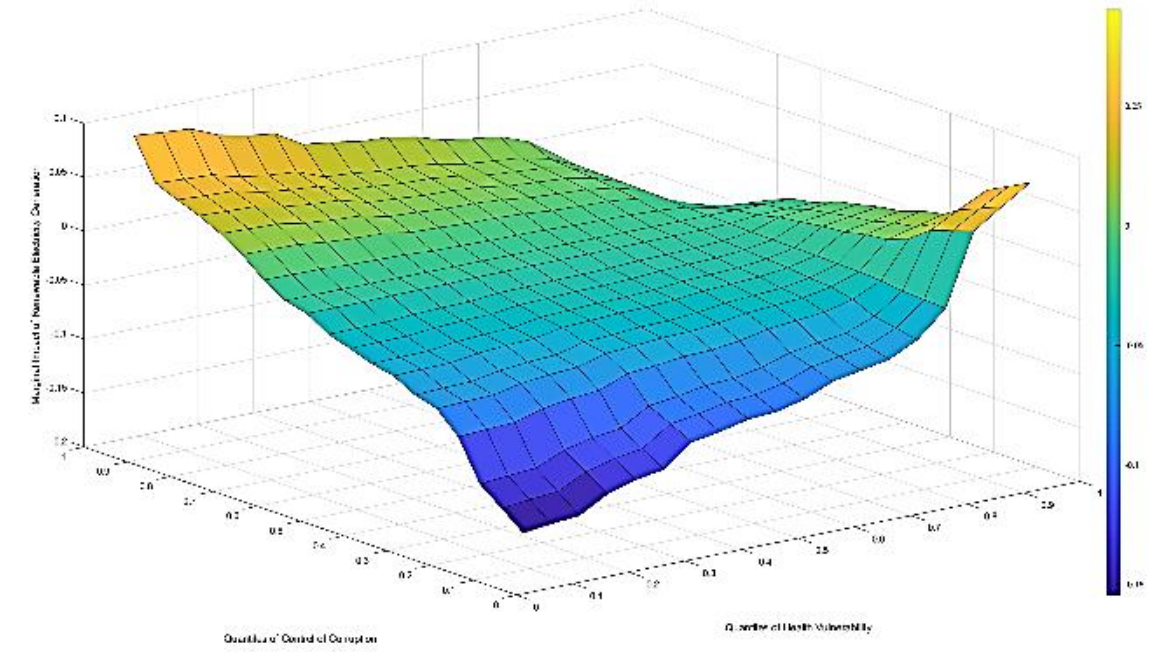

Governance Quality Indicator: Control of Corruption

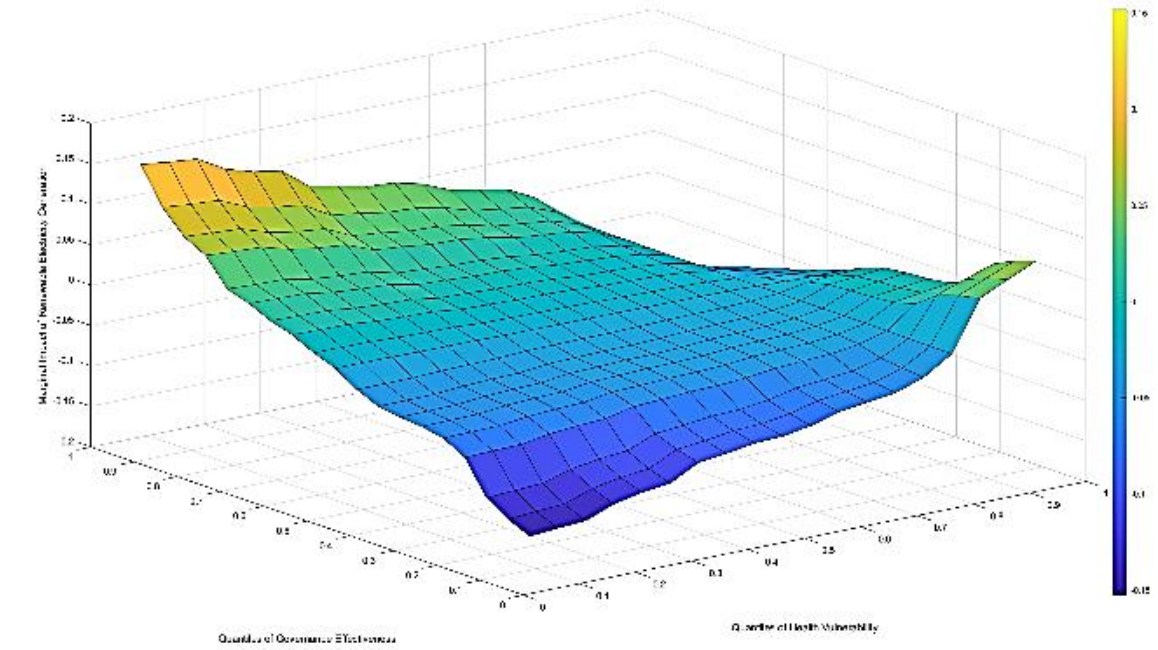

Governance Quality Indicator: Governance Effectiveness

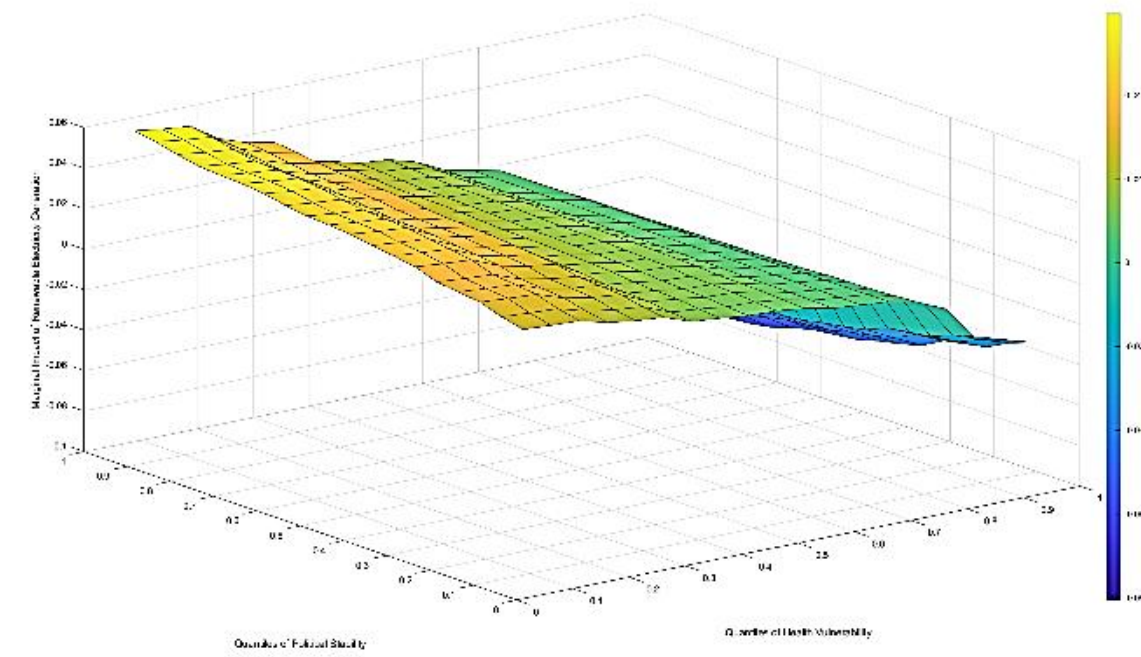

Governance Quality Indicator: Political Stability

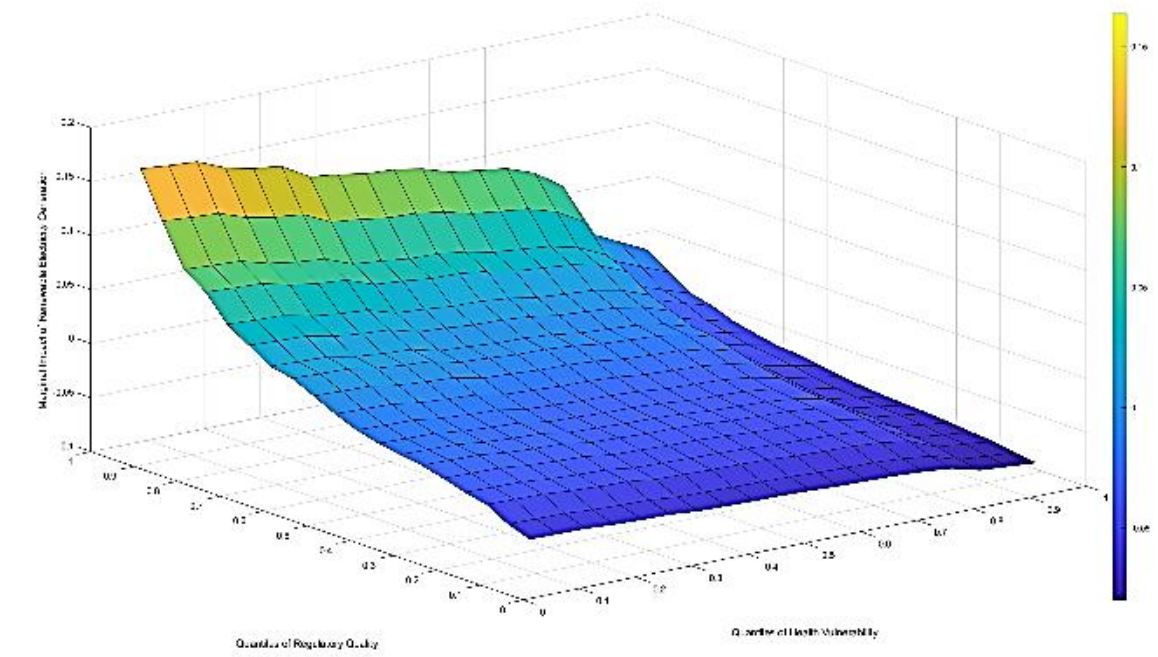

Governance Quality Indicator: Regulatory Quality

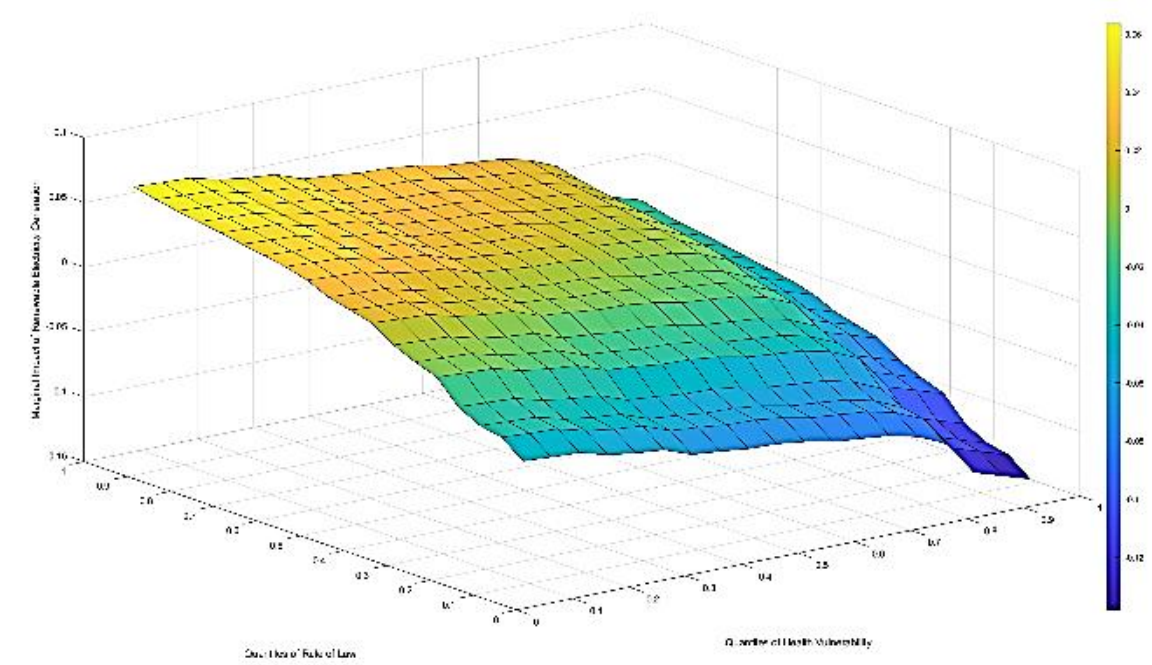

Governance Quality Indicator: Rule of Law

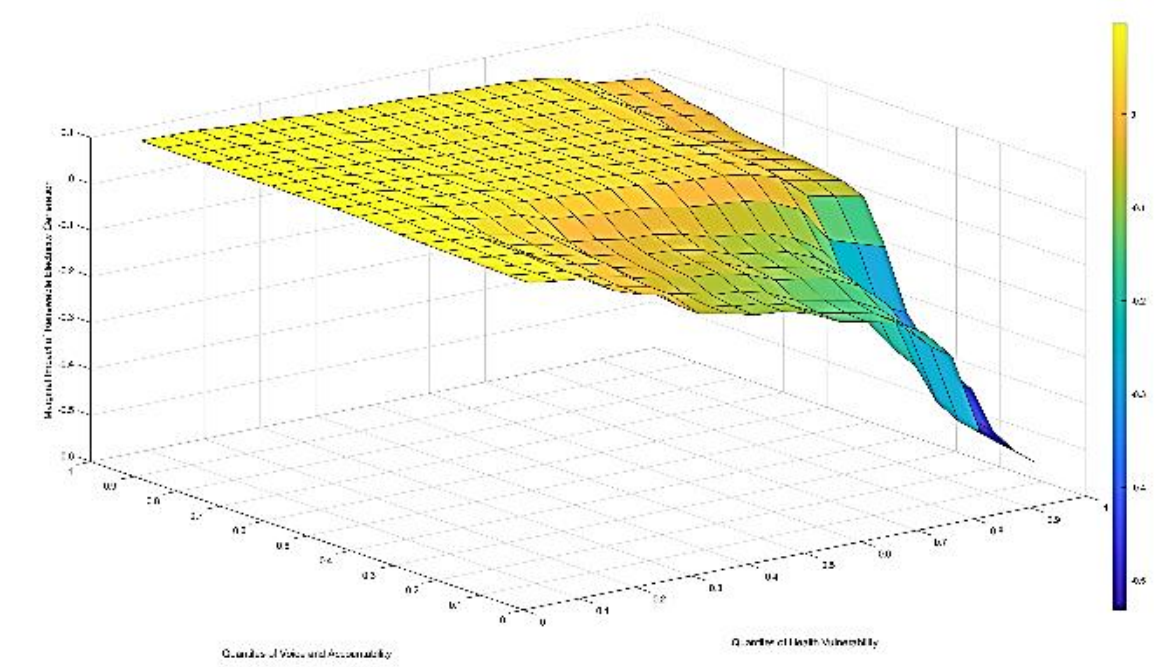

Governance Quality Indicator: Voice and Accountability

Figure S35: BRICS Policy Simulation Outcomes for the Marginal Impact of Renewable Electricity Generation on SDG 7.1.2 under Health Vulnerability and Governance Quality, Related to Figure 4-5 and Table 2

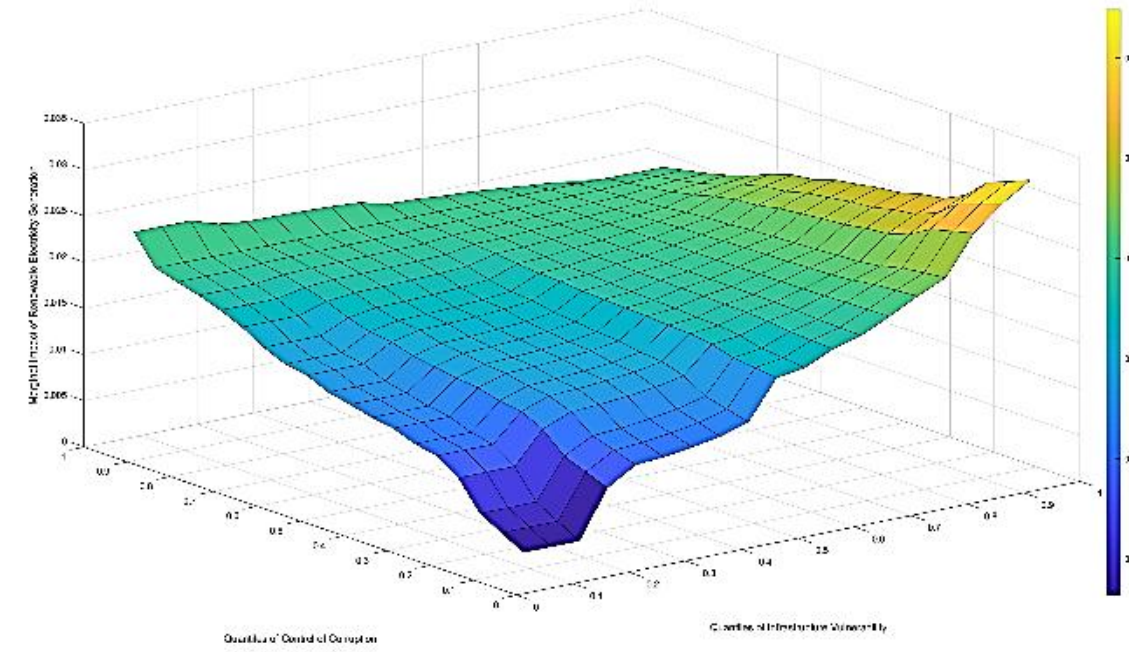

Governance Quality Indicator: Control of Corruption

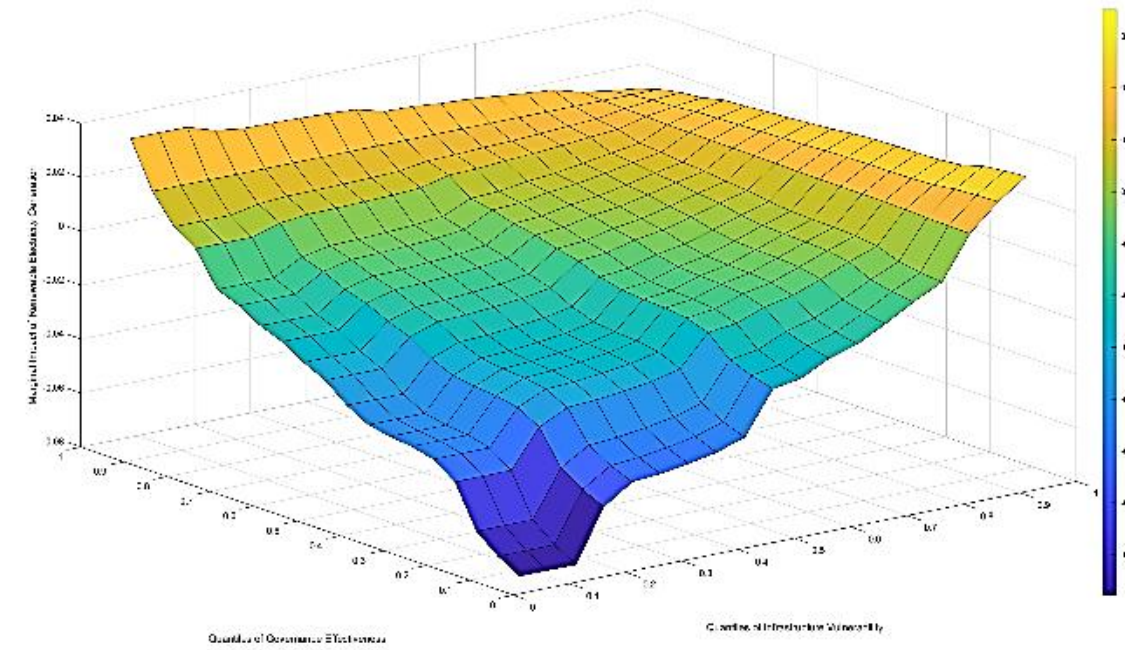

Governance Quality Indicator: Governance Effectiveness

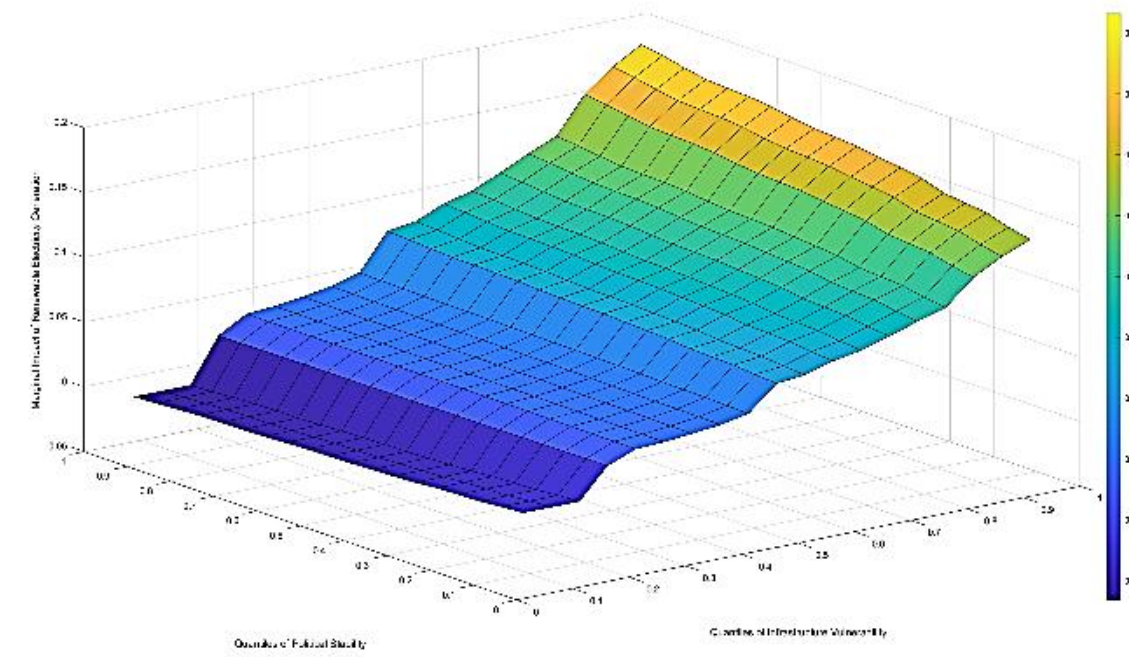

Governance Quality Indicator: Political Stability

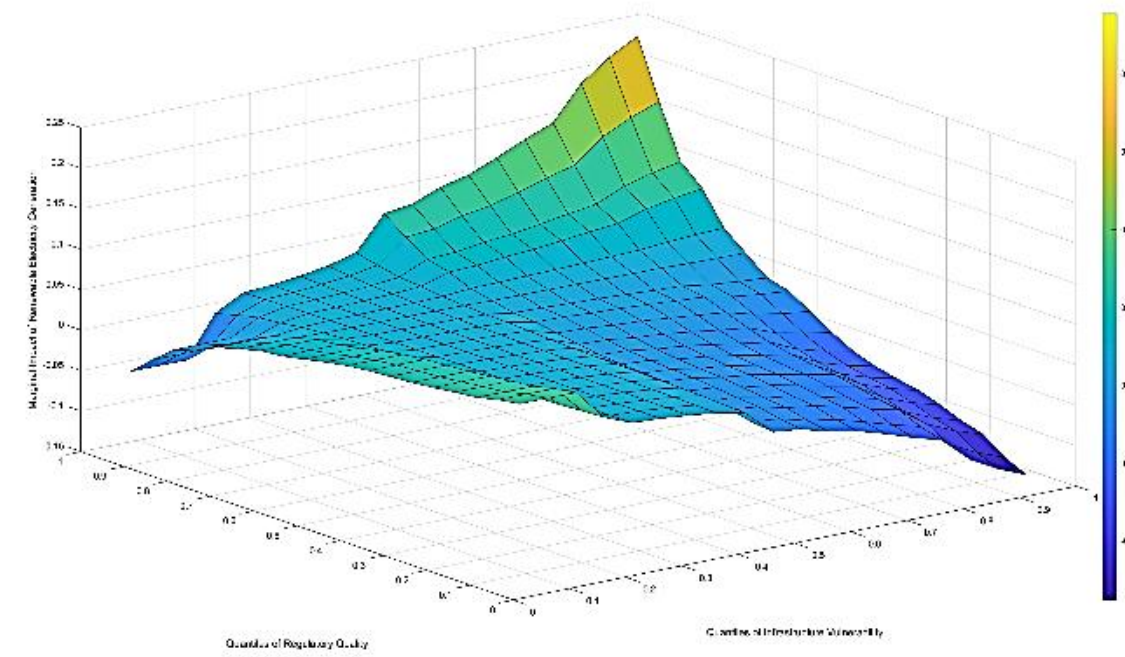

Governance Quality Indicator: Regulatory Quality

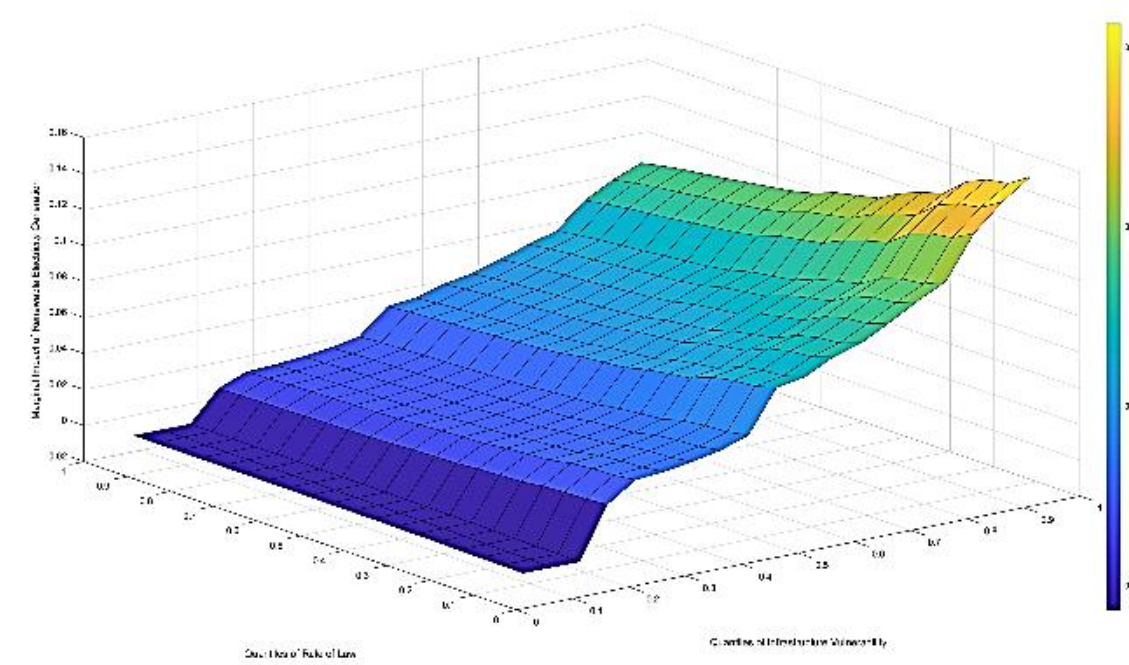

Governance Quality Indicator: Rule of Law

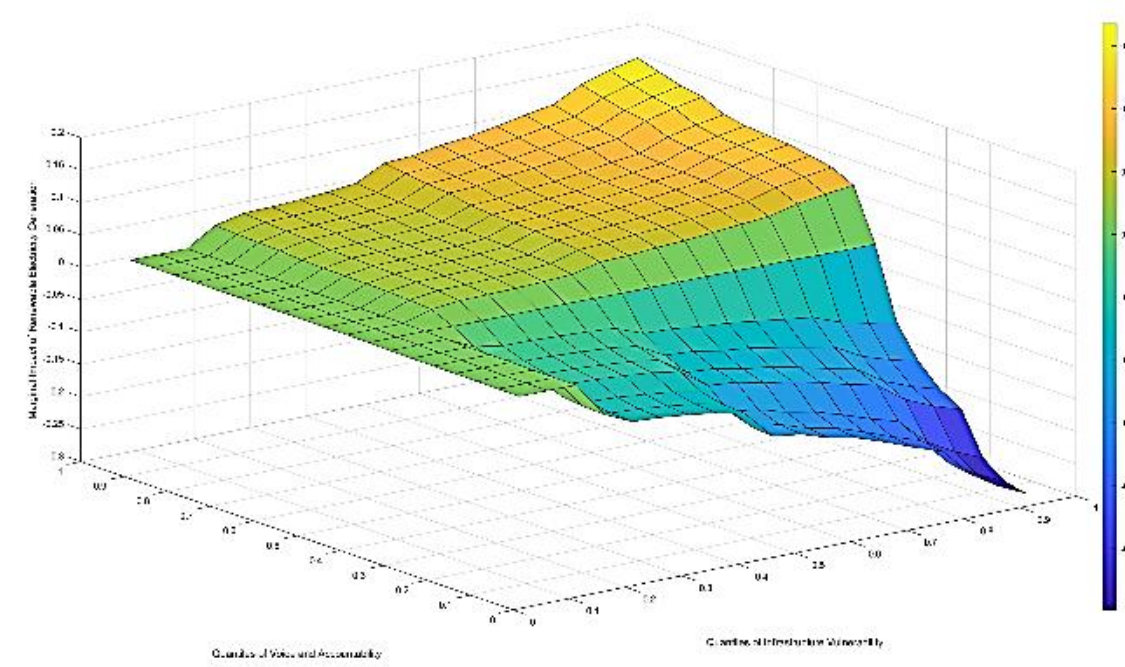

Governance Quality Indicator: Voice and Accountability

Figure S36: BRICS Policy Simulation Outcomes for the Marginal Impact of Renewable Electricity Generation on SDG 7.1.2 under Infrastructure Vulnerability and Governance Quality, Related to Figure 4-5 and Table 2

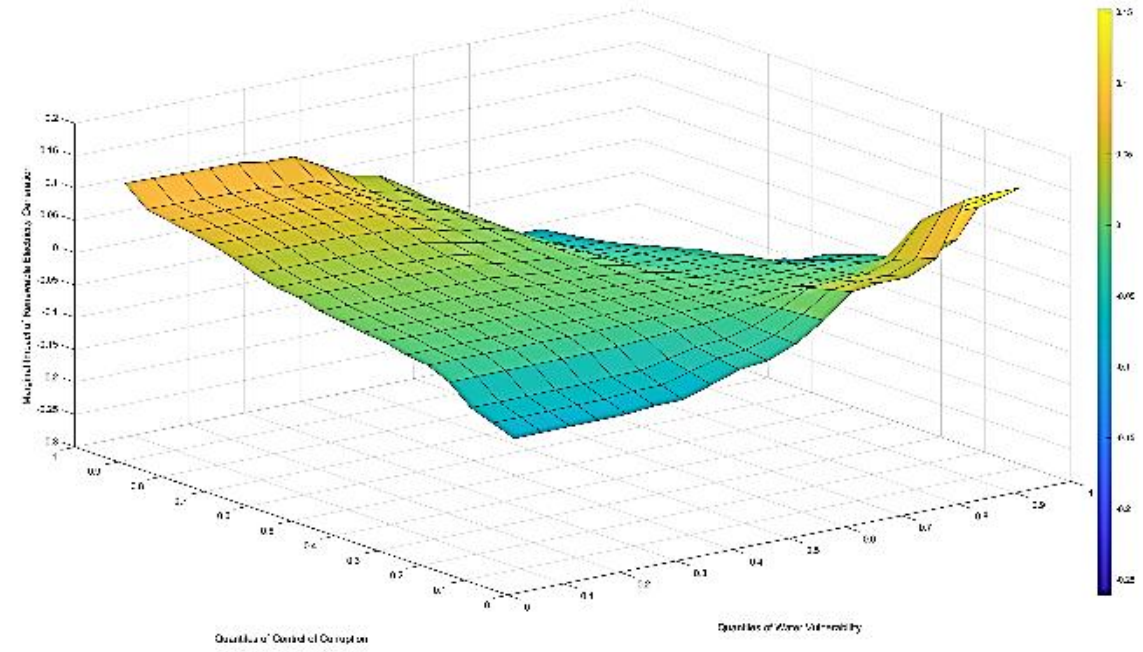

Governance Quality Indicator: Control of Corruption

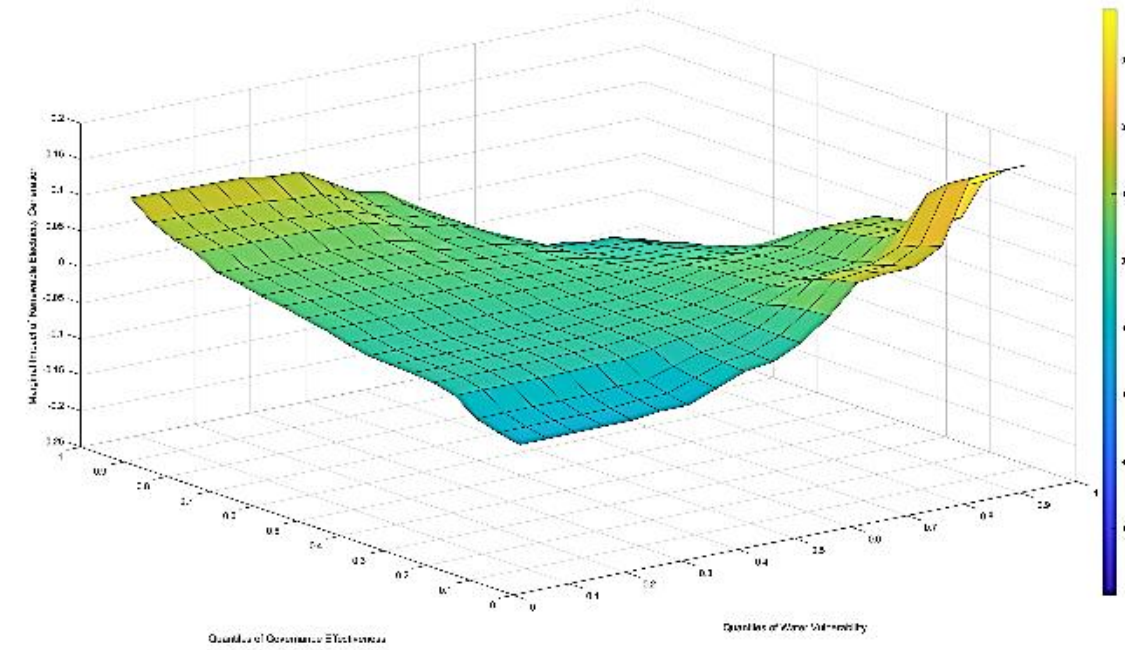

Governance Quality Indicator: Governance Effectiveness

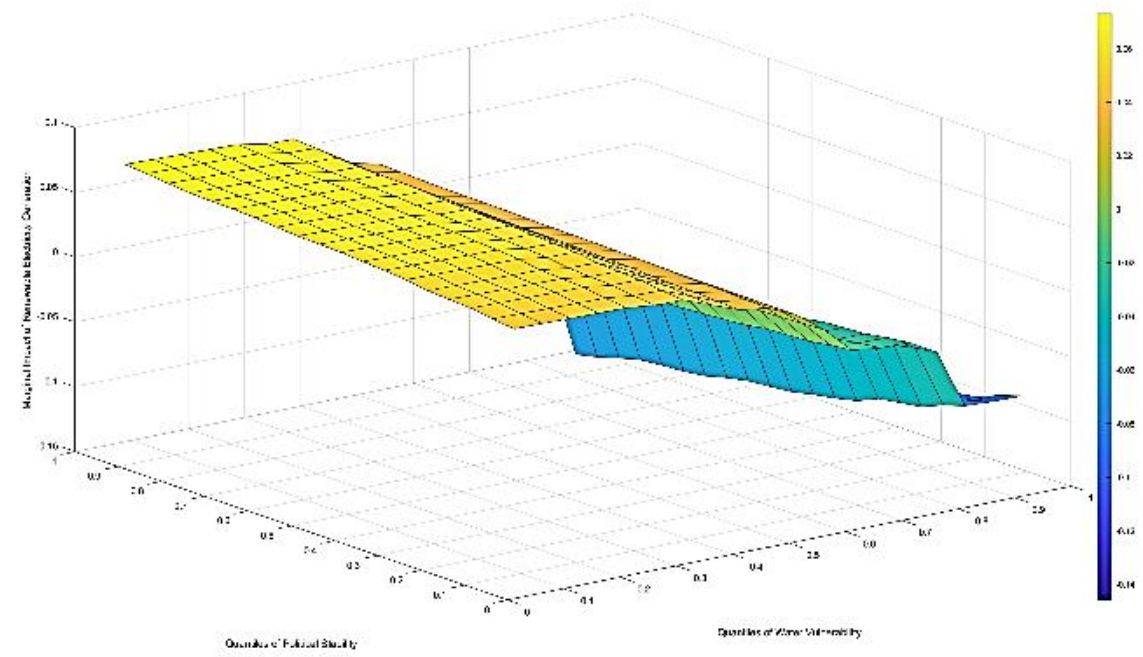

Governance Quality Indicator: Political Stability

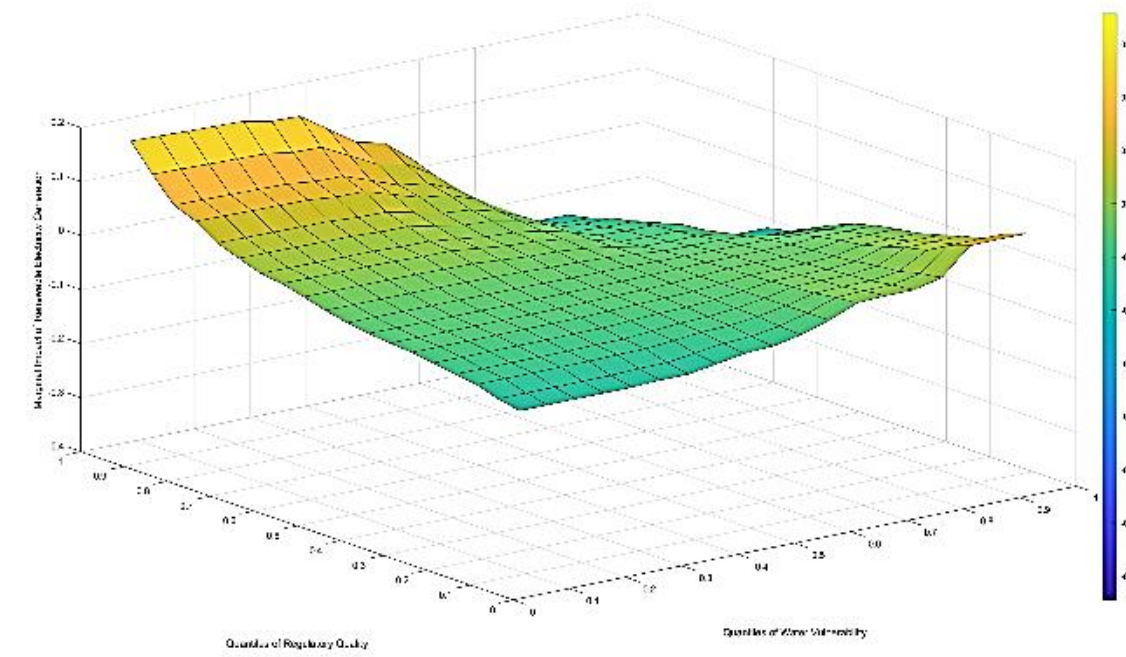

Governance Quality Indicator: Regulatory Quality

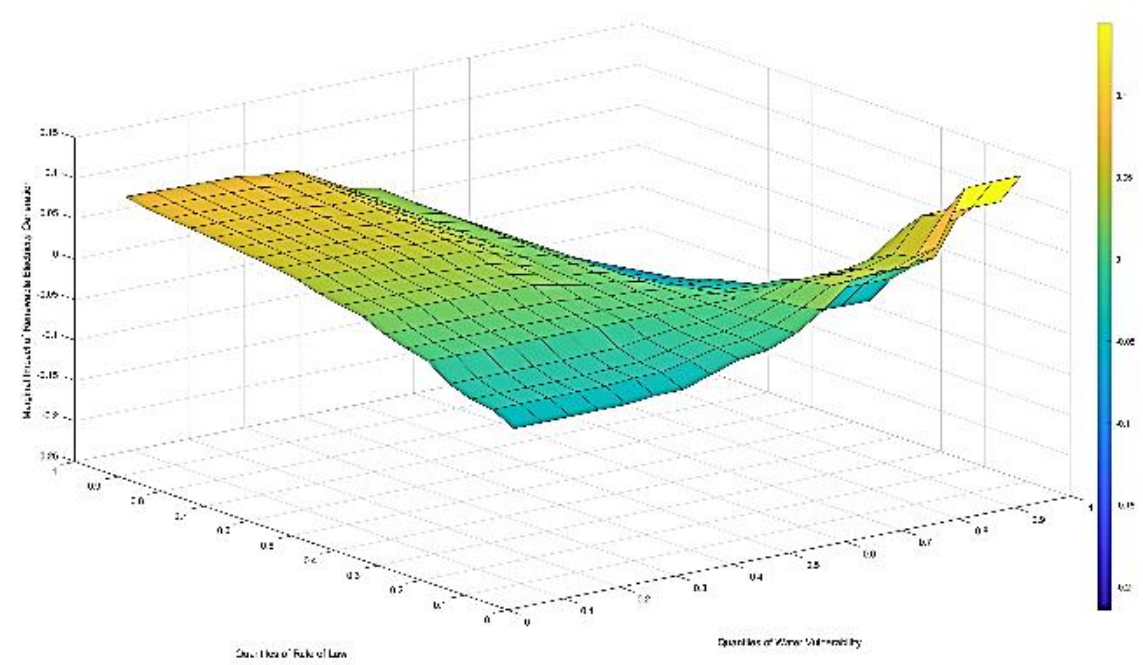

Governance Quality Indicator: Rule of Law

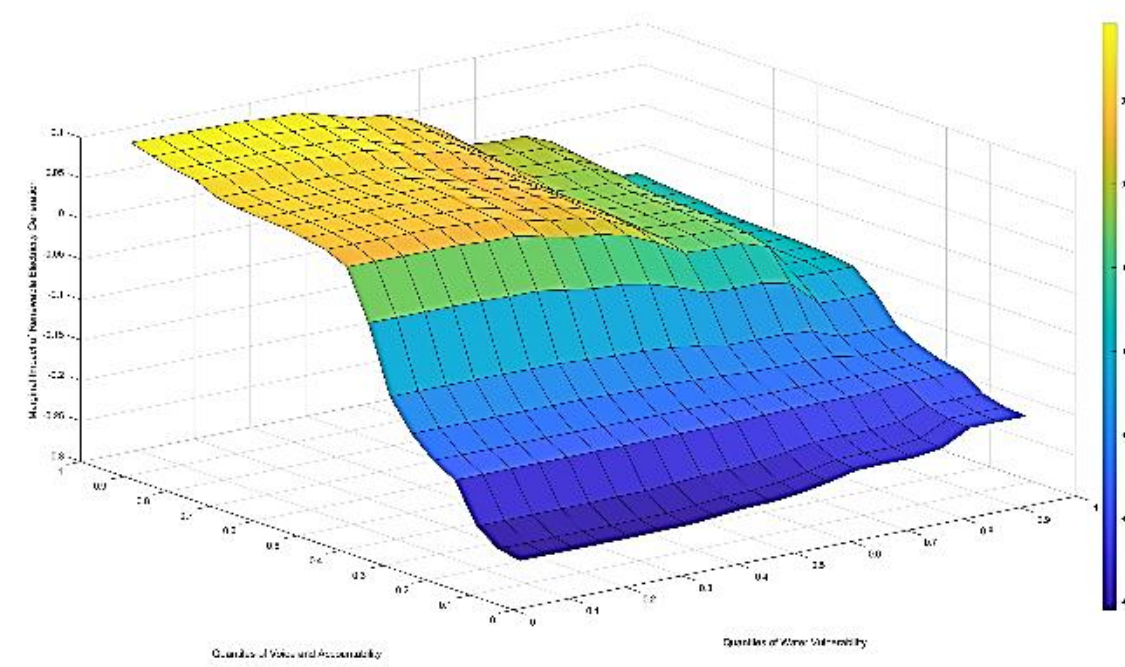

Governance Quality Indicator: Voice and Accountability

Figure S37: BRICS Policy Simulation Outcomes for the Marginal Impact of Renewable Electricity Generation on SDG 7.1.2 under Water Vulnerability and Governance Quality, Related to Figure 4-5 and Table 2

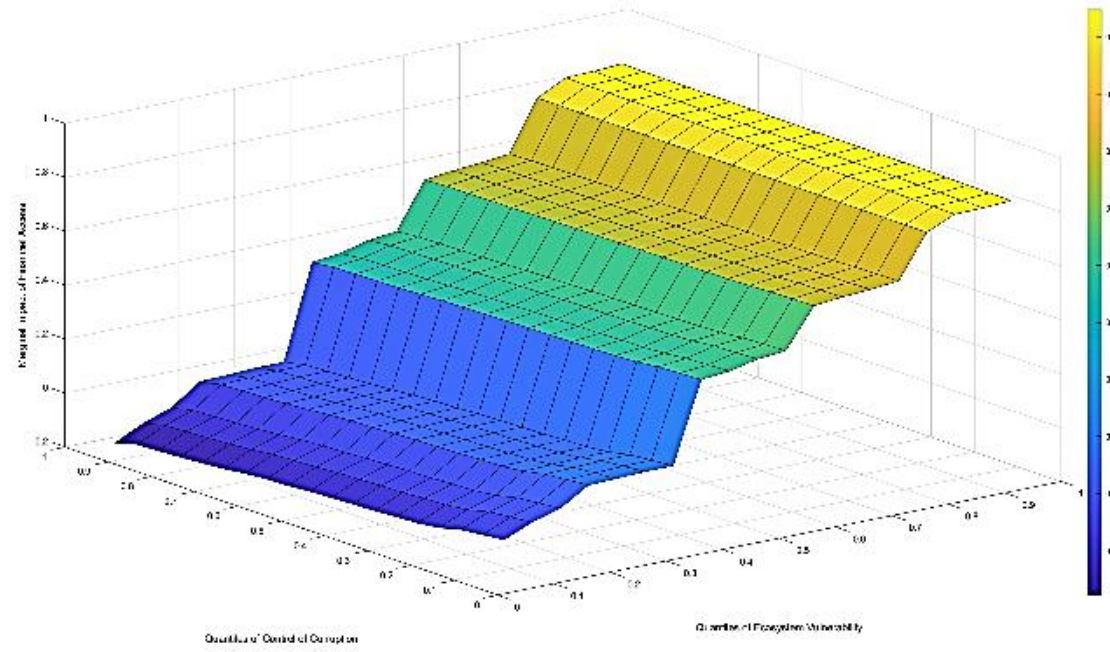

Governance Quality Indicator: Control of Corruption

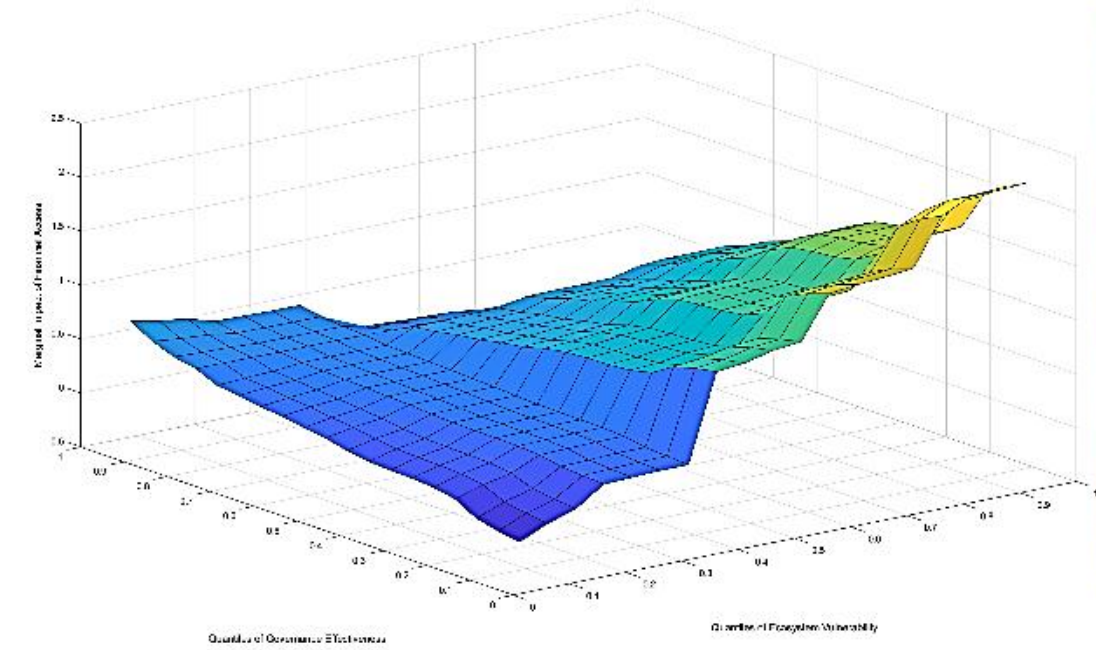

Governance Quality Indicator: Governance Effectiveness

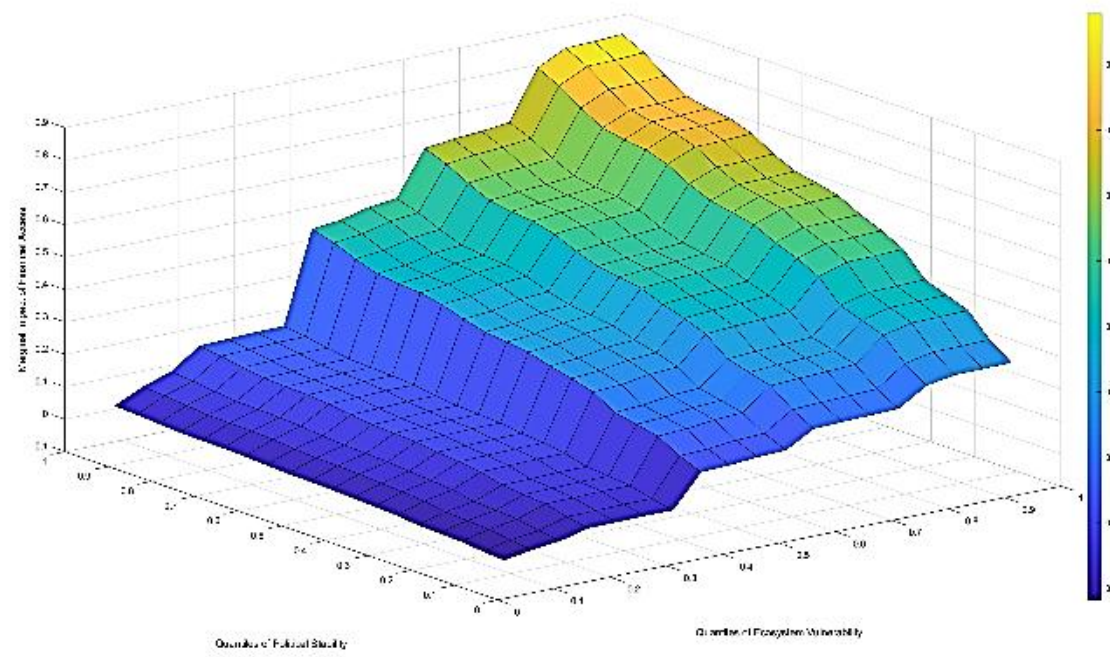

Governance Quality Indicator: Political Stability

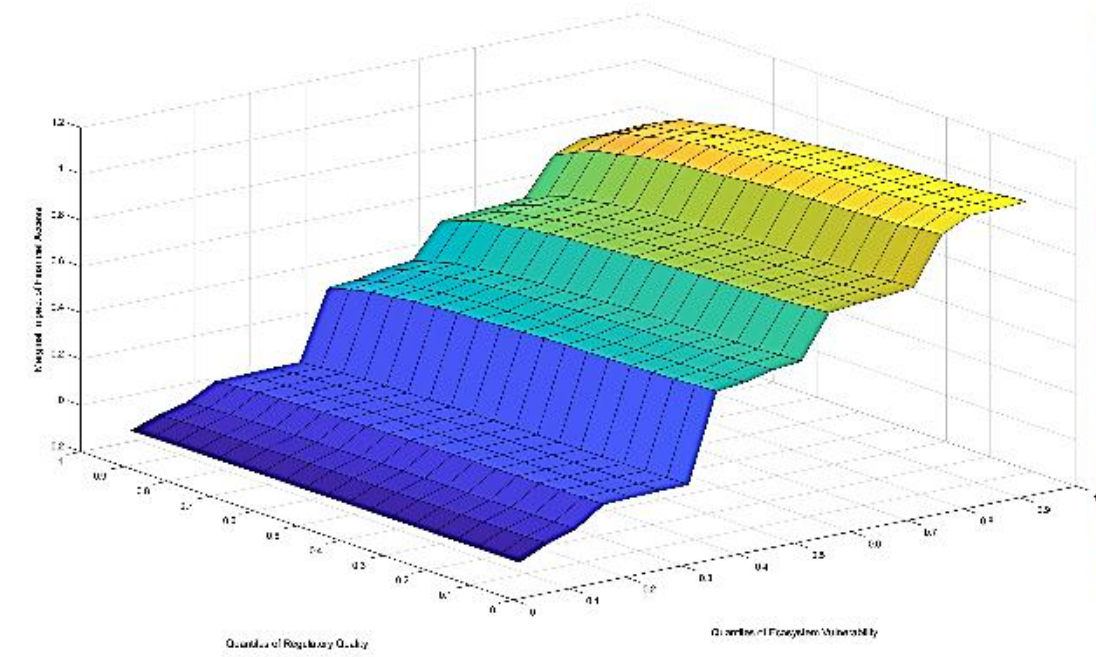

Governance Quality Indicator: Regulatory Quality

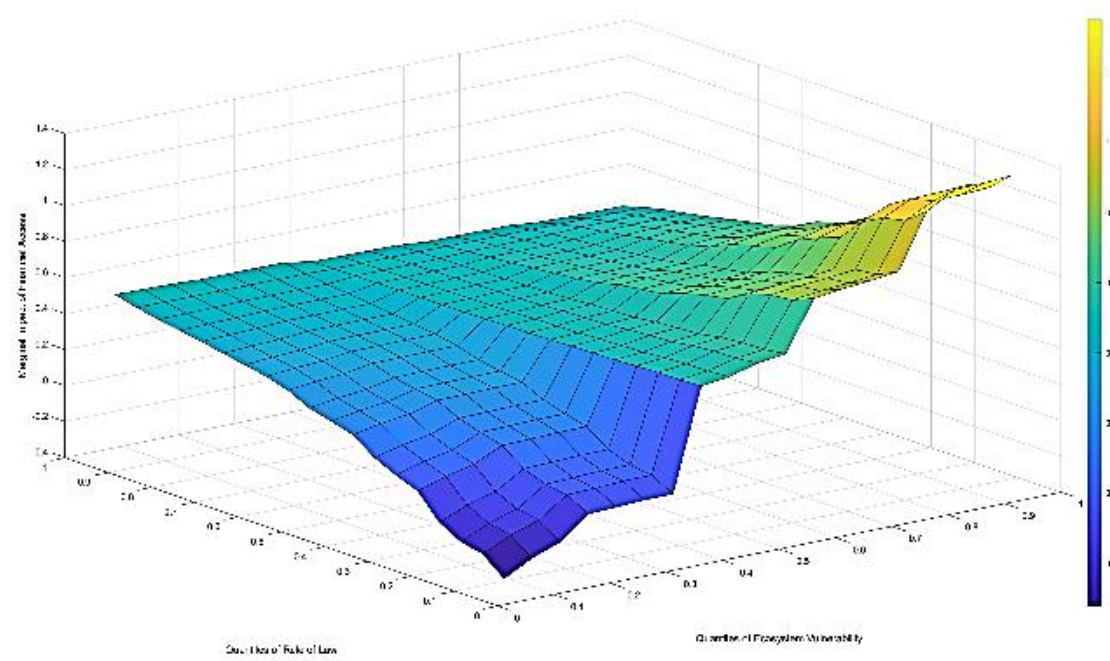

Governance Quality Indicator: Rule of Law

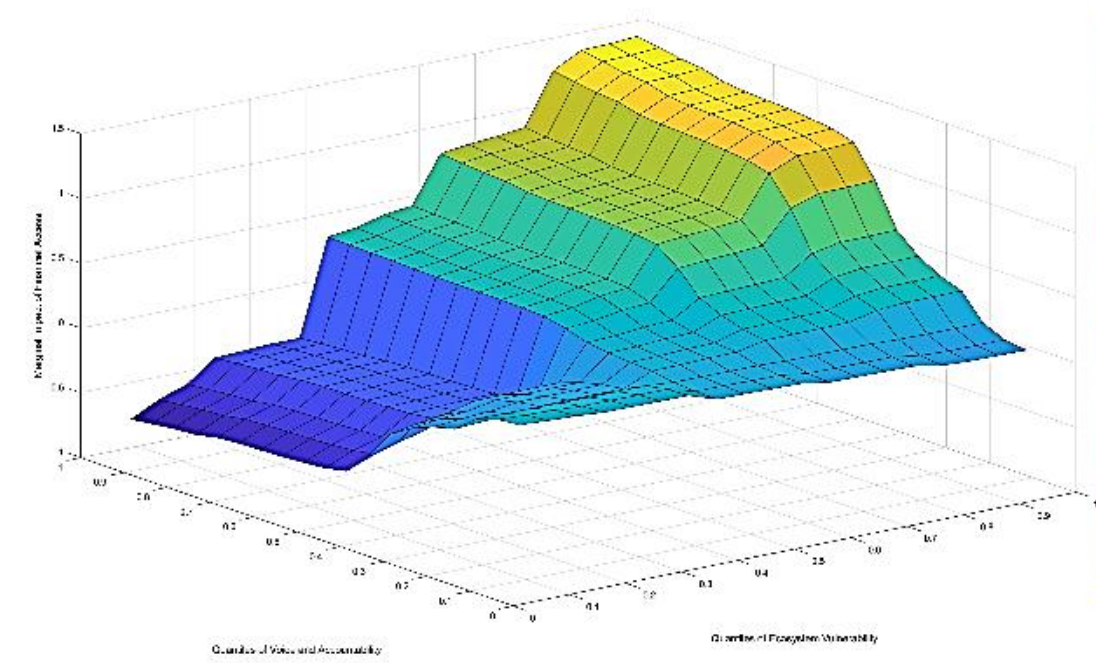

Governance Quality Indicator: Voice and Accountability

Figure S38: BRICS Policy Simulation Outcomes for the Marginal Impact of Financial Access on SDG 7.1.2 under Ecosystem Vulnerability and Governance Quality, Related to Figure 4-5 and Table 2

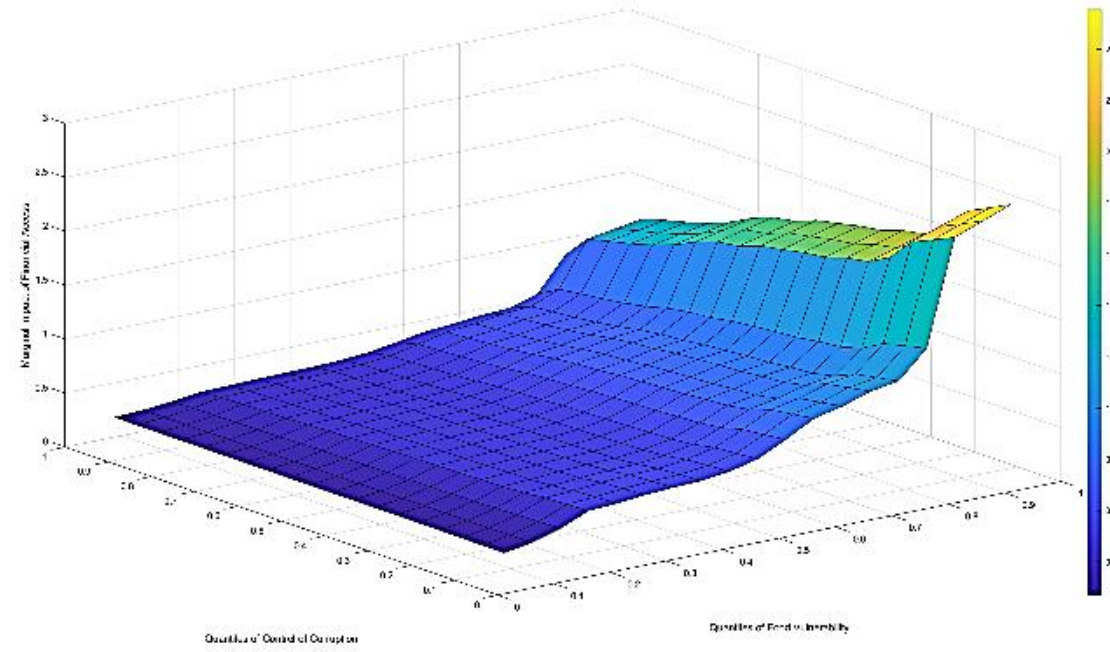

Governance Quality Indicator: Control of Corruption

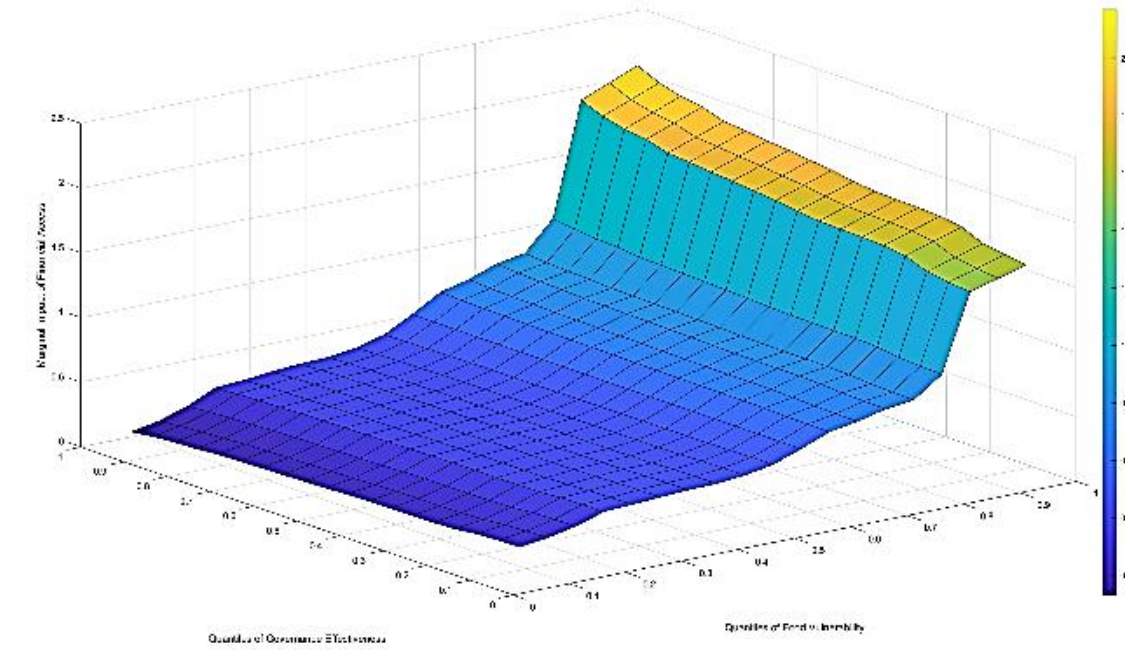

Governance Quality Indicator: Governance Effectiveness

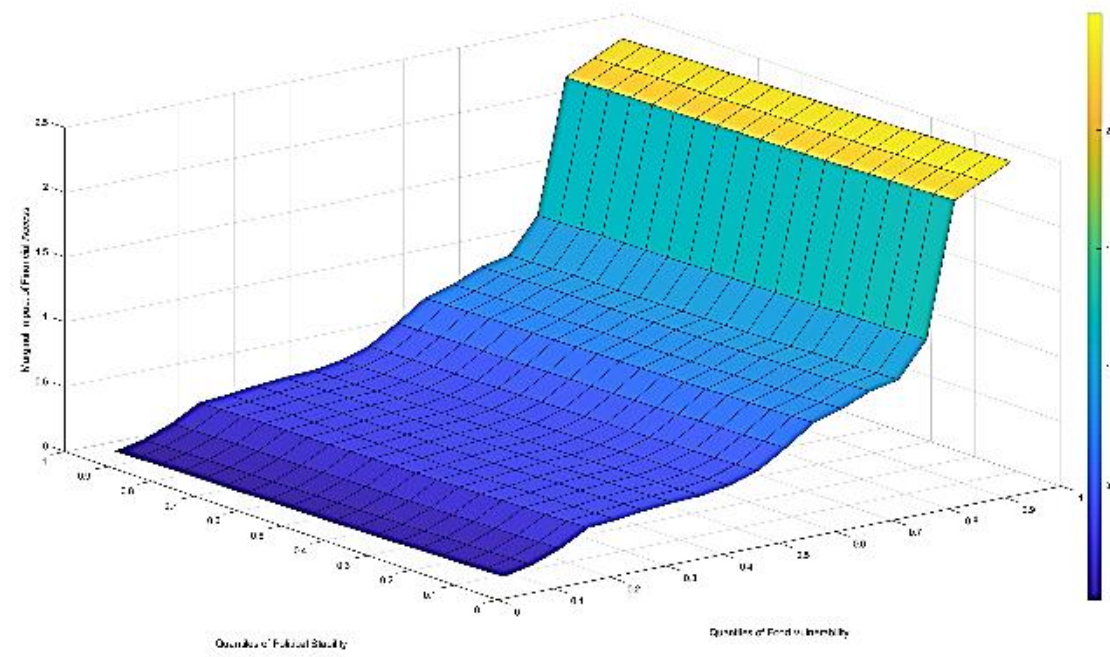

Governance Quality Indicator: Political Stability

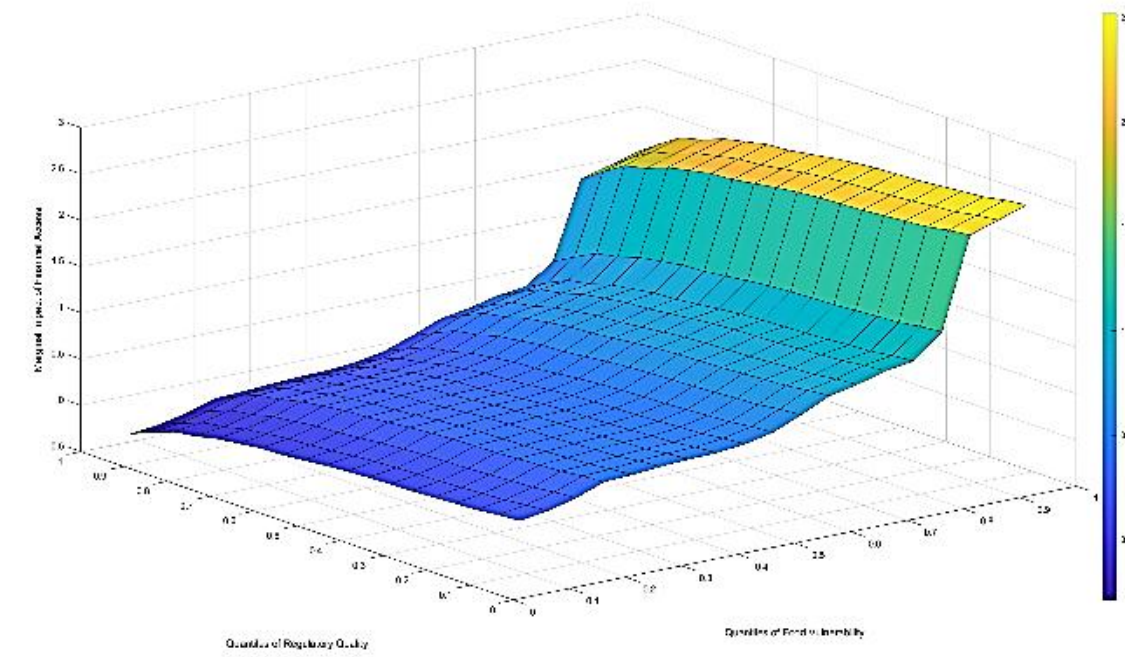

Governance Quality Indicator: Regulatory Quality

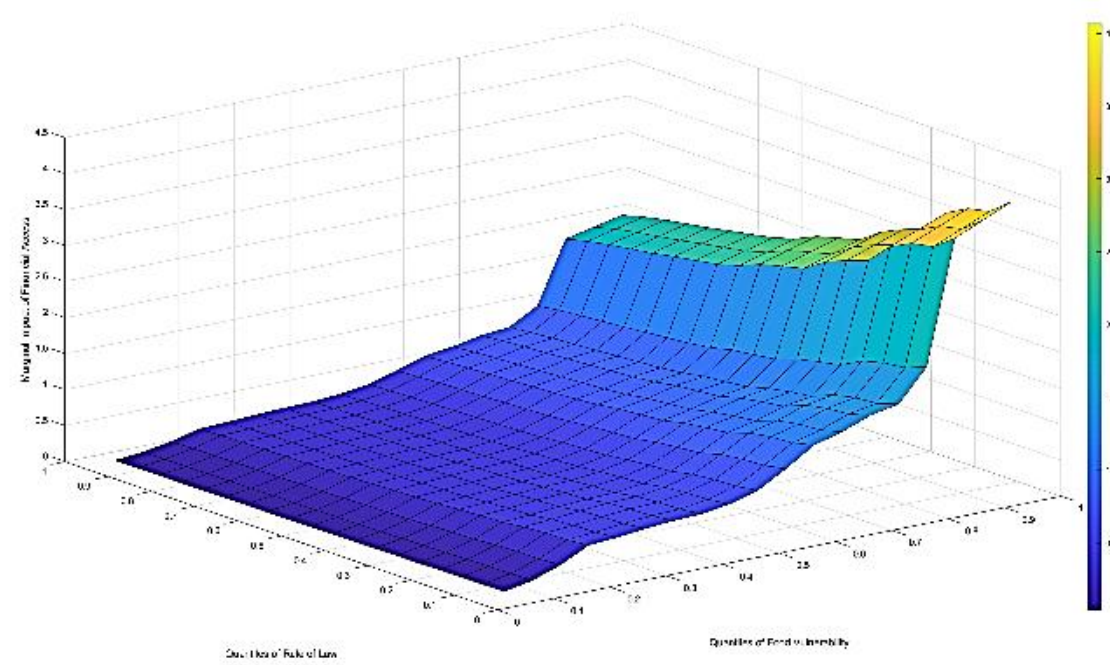

Governance Quality Indicator: Rule of Law

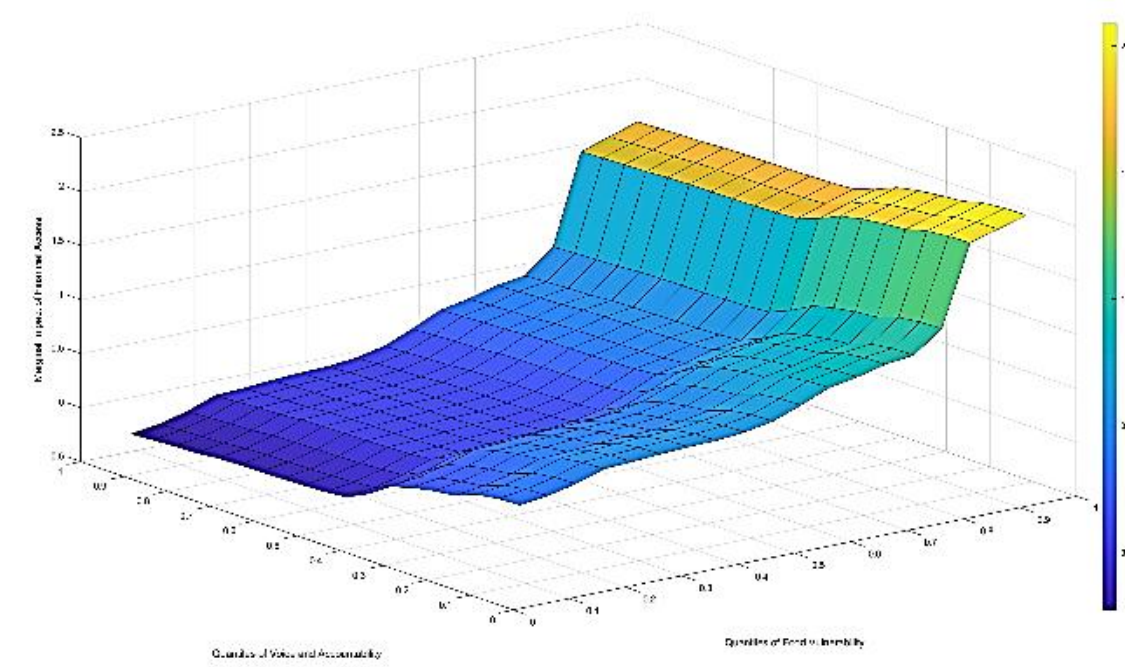

Governance Quality Indicator: Voice and Accountability

Figure S39: BRICS Policy Simulation Outcomes for the Marginal Impact of Financial Access on SDG 7.1.2 under Food Vulnerability and Governance Quality, Related to Figure 4-5 and Table 2

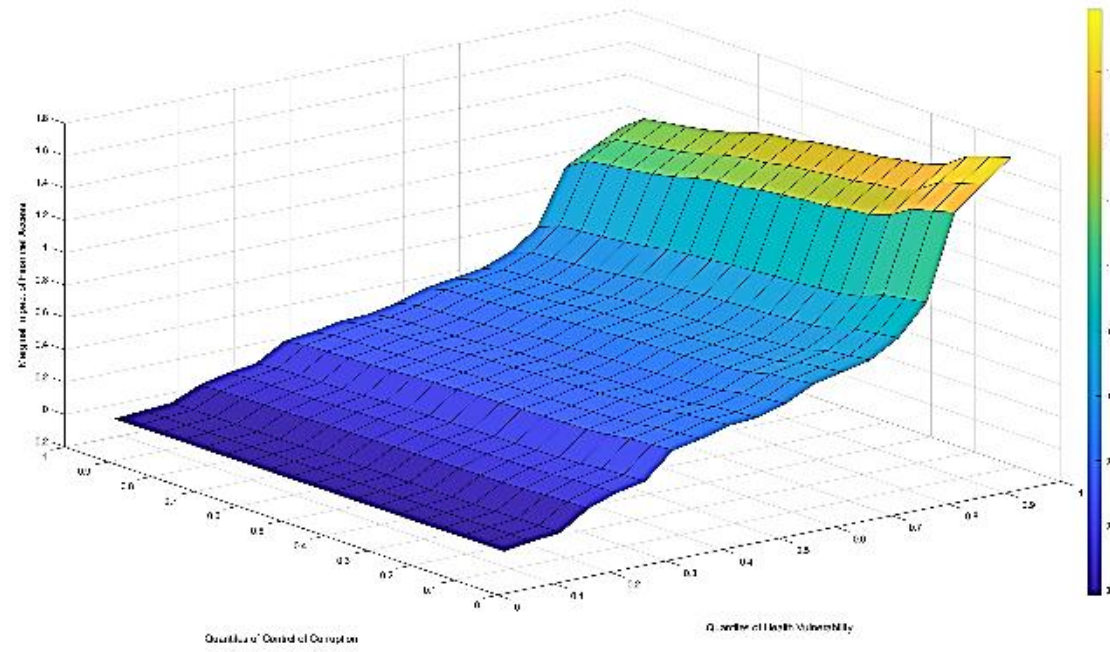

Governance Quality Indicator: Control of Corruption

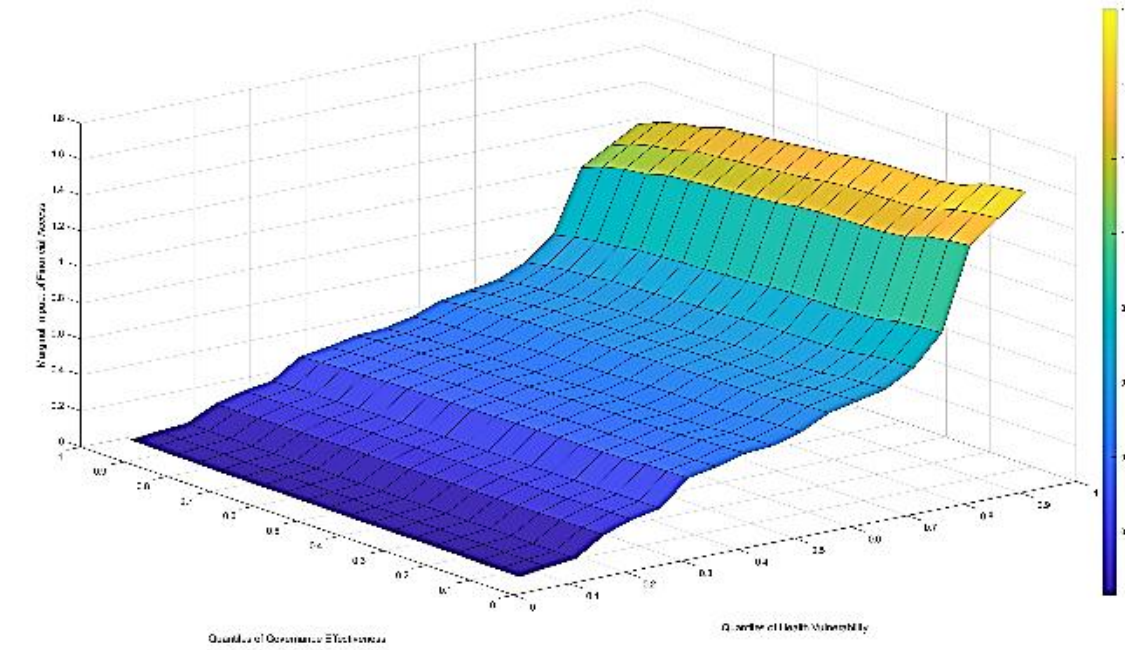

Governance Quality Indicator: Governance Effectiveness

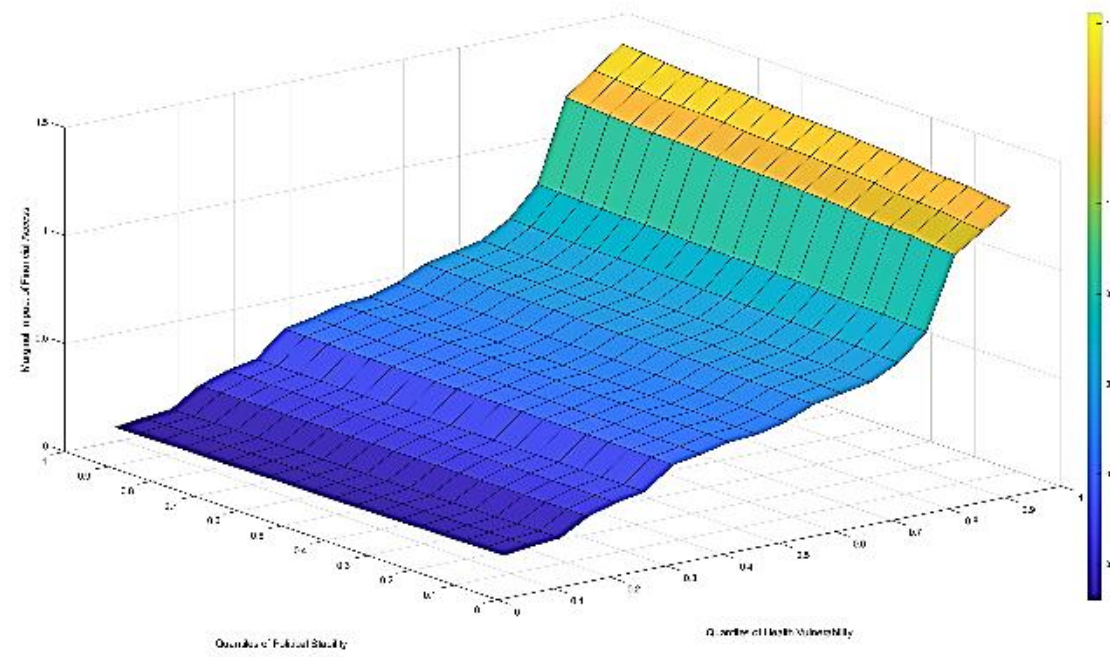

Governance Quality Indicator: Political Stability

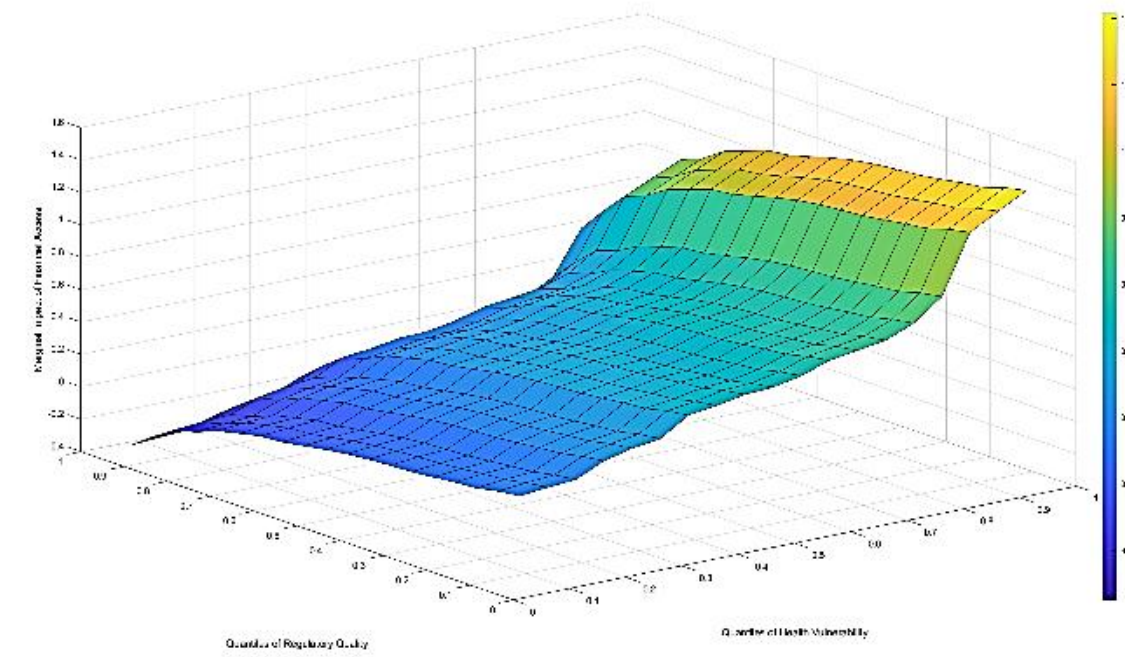

Governance Quality Indicator: Regulatory Quality

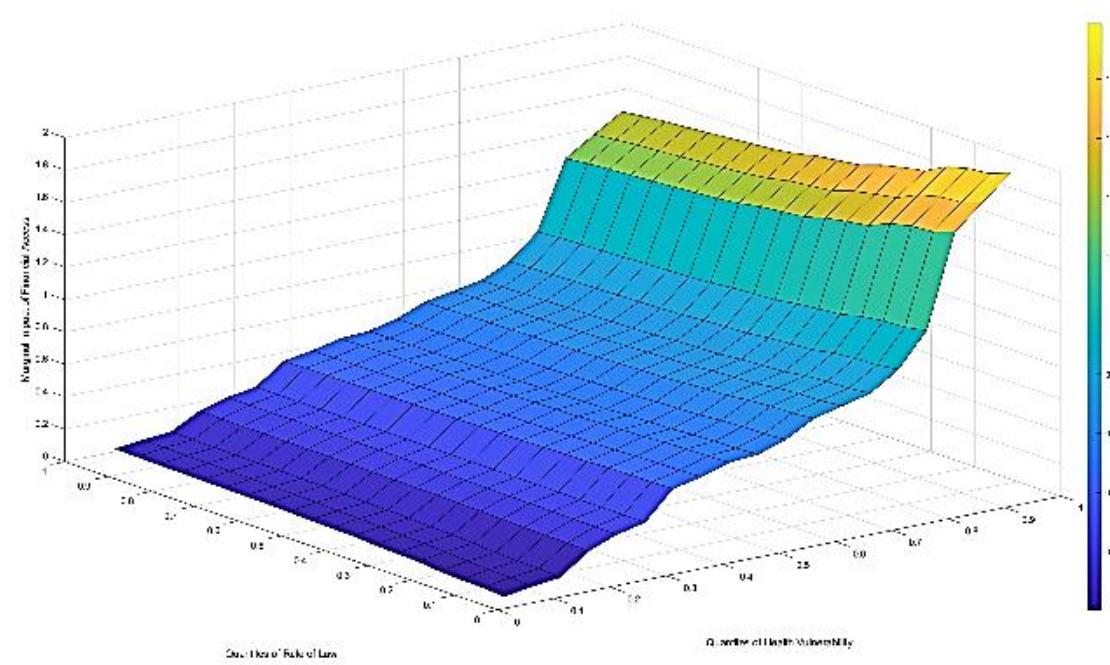

Governance Quality Indicator: Rule of Law

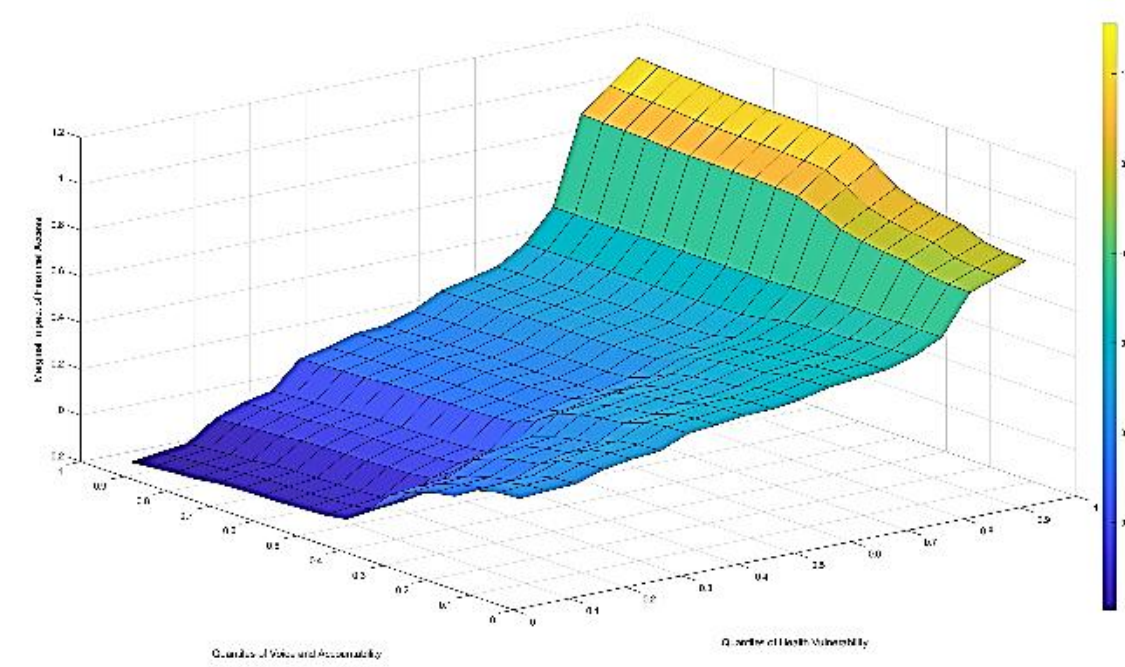

Governance Quality Indicator: Voice and Accountability

Figure S40: BRICS Policy Simulation Outcomes for the Marginal Impact of Financial Access on SDG 7.1.2 under Health Vulnerability and Governance Quality, Related to Figure 4-5 and Table 2

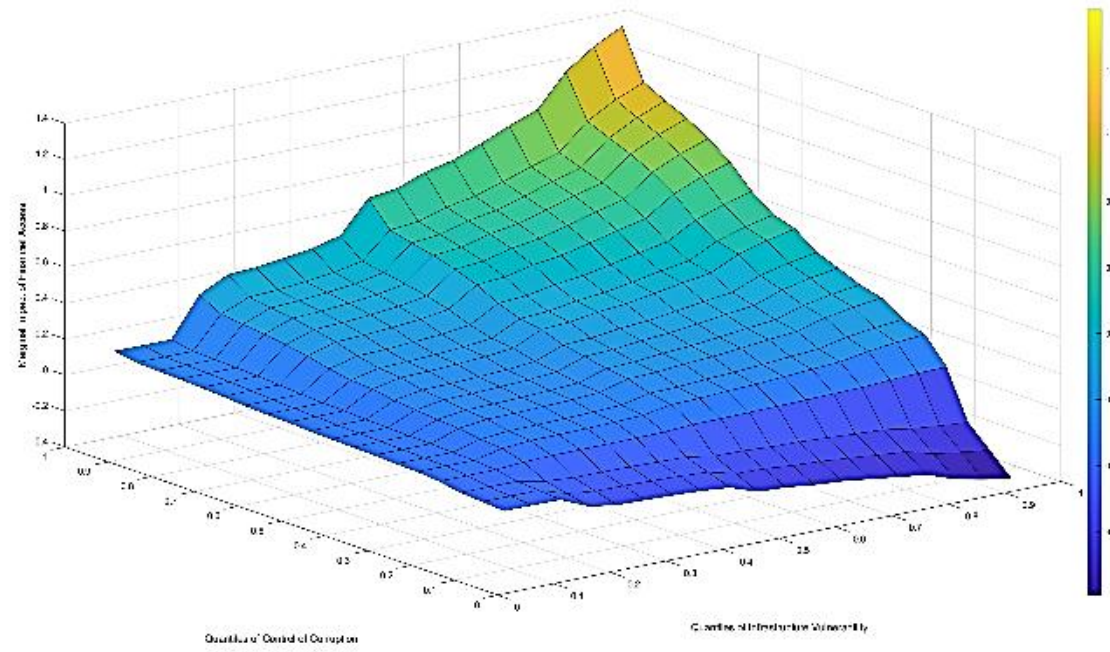

Governance Quality Indicator: Control of Corruption

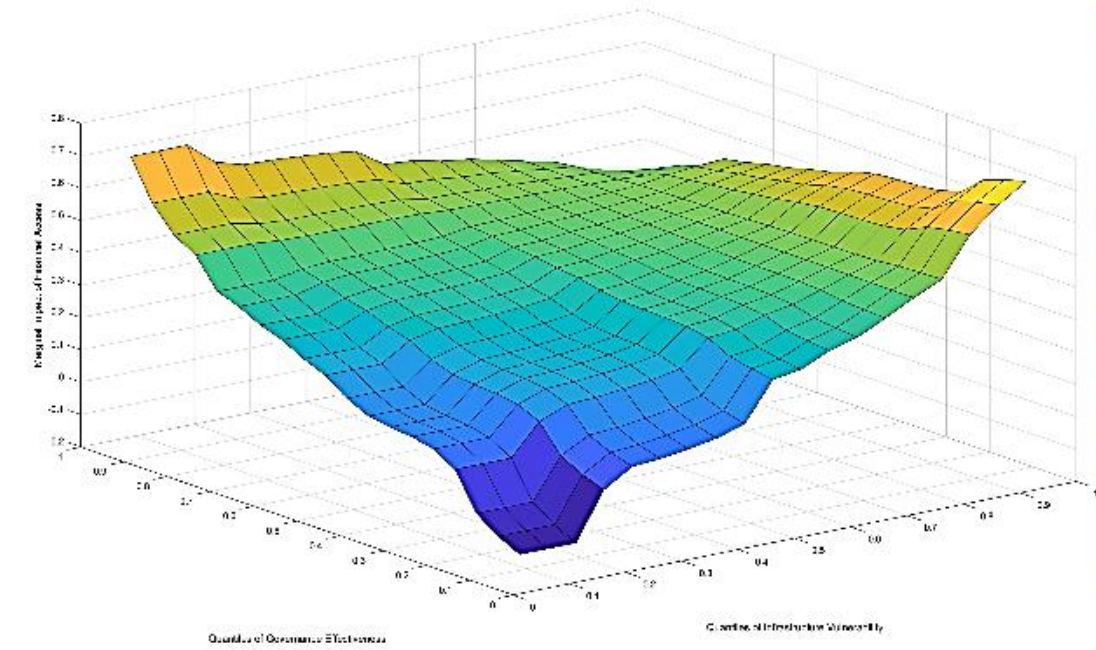

Governance Quality Indicator: Governance Effectiveness

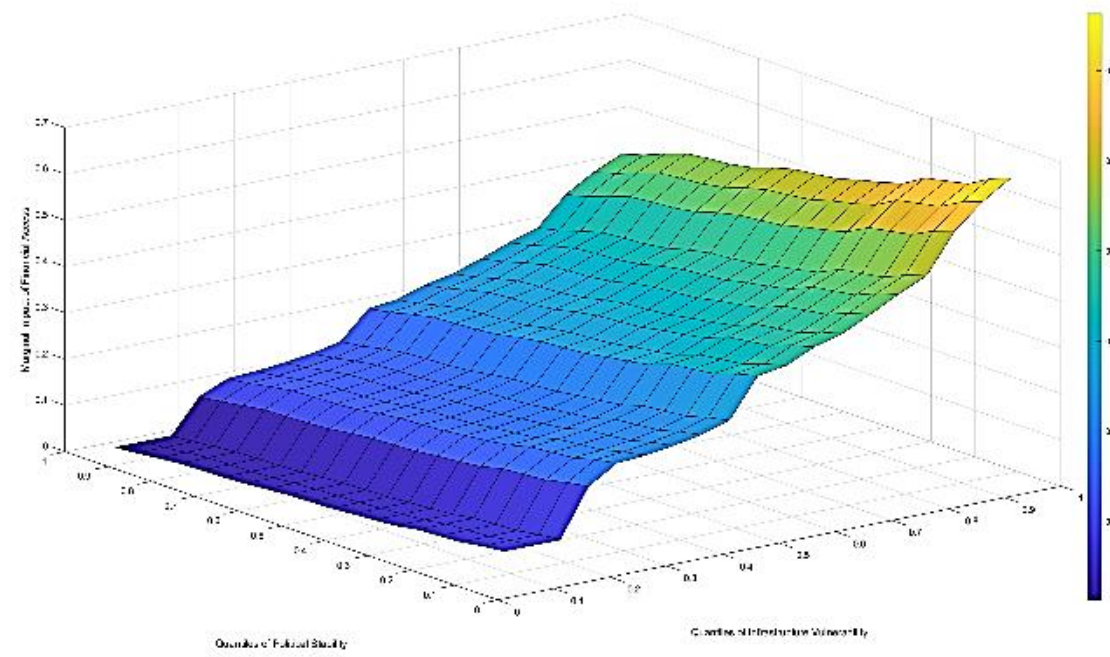

Governance Quality Indicator: Political Stability

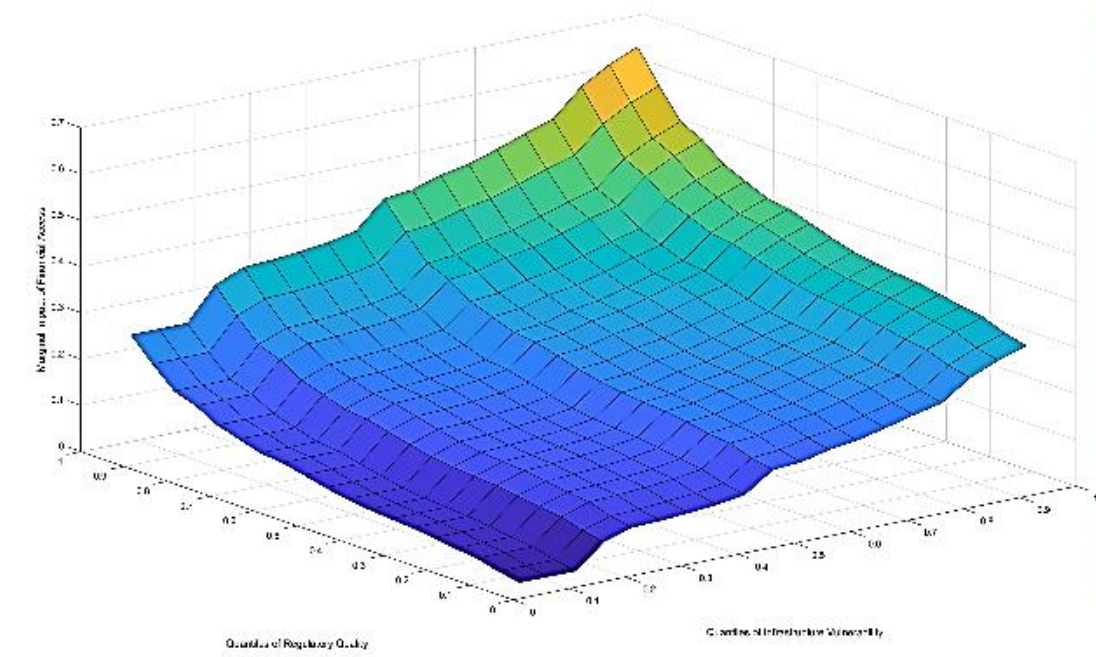

Governance Quality Indicator: Regulatory Quality

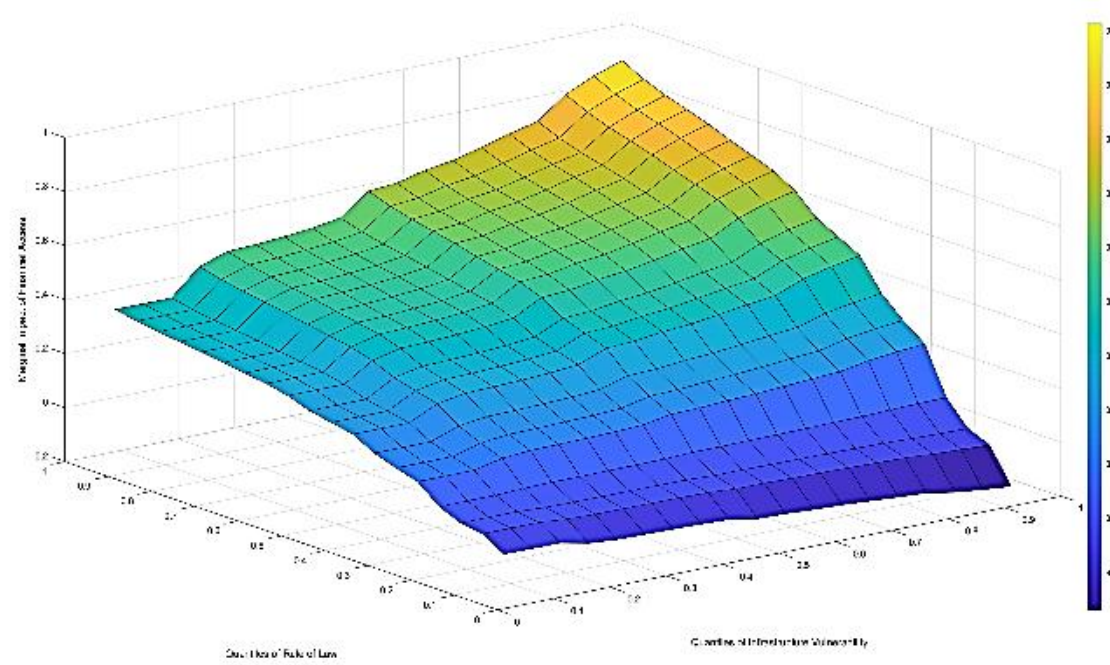

Governance Quality Indicator: Rule of Law

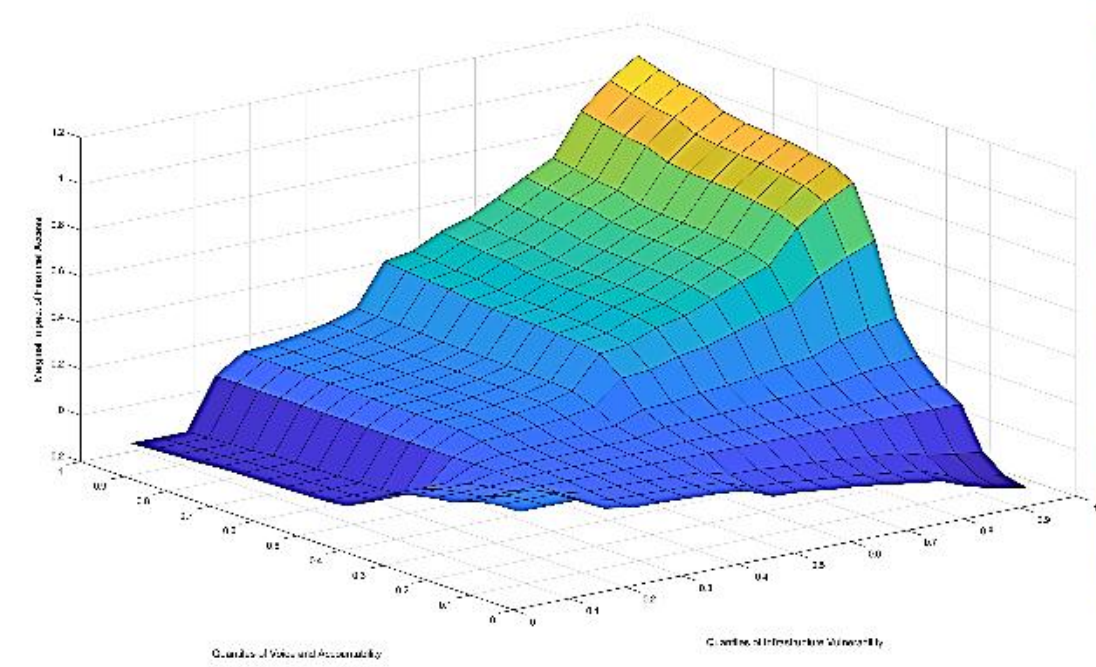

Governance Quality Indicator: Voice and Accountability

Figure S41: BRICS Policy Simulation Outcomes for the Marginal Impact of Financial Access on SDG 7.1.2 under Infrastructure Vulnerability and Governance Quality, Related to Figure 4-5 and Table 2

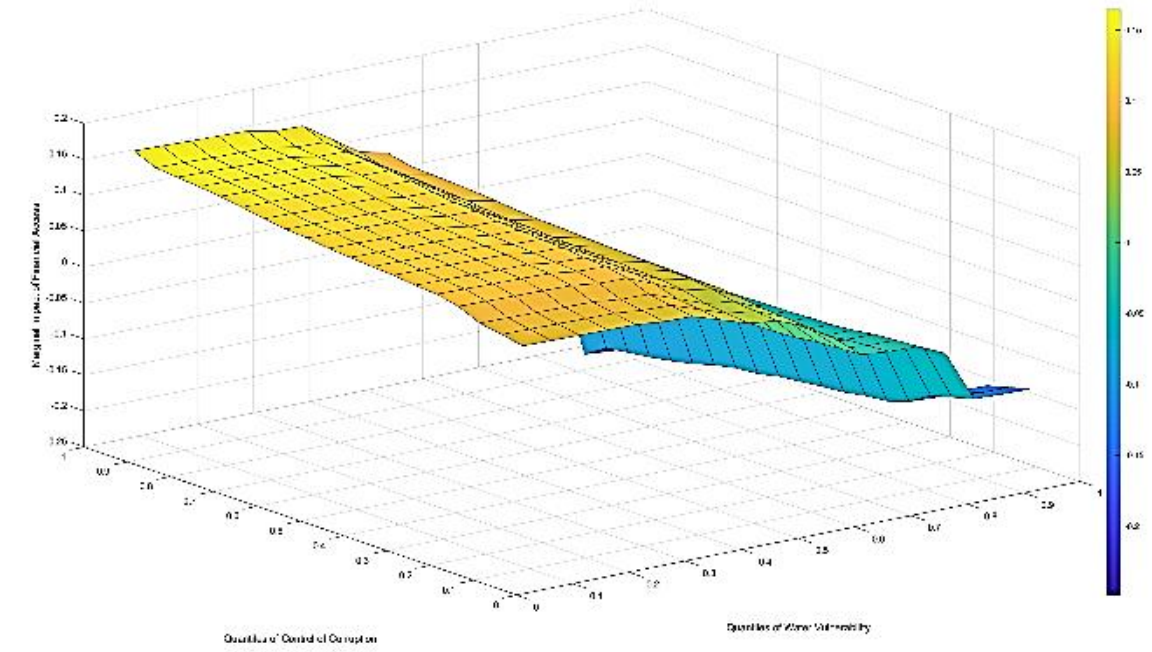

Governance Quality Indicator: Control of Corruption

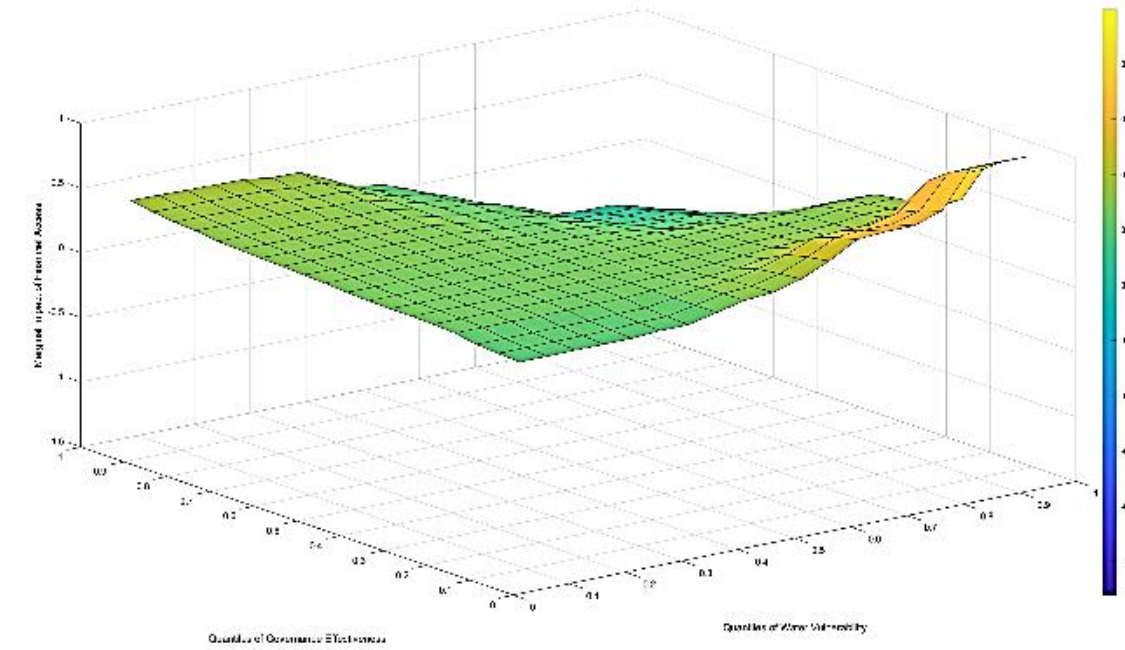

Governance Quality Indicator: Governance Effectiveness

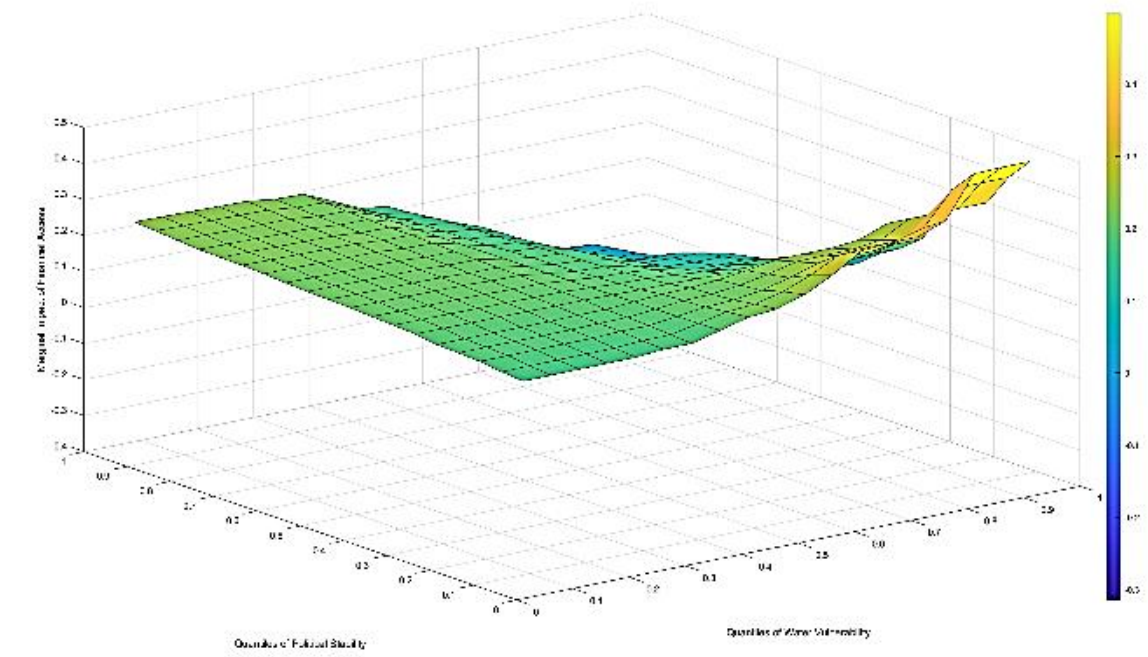

Governance Quality Indicator: Political Stability

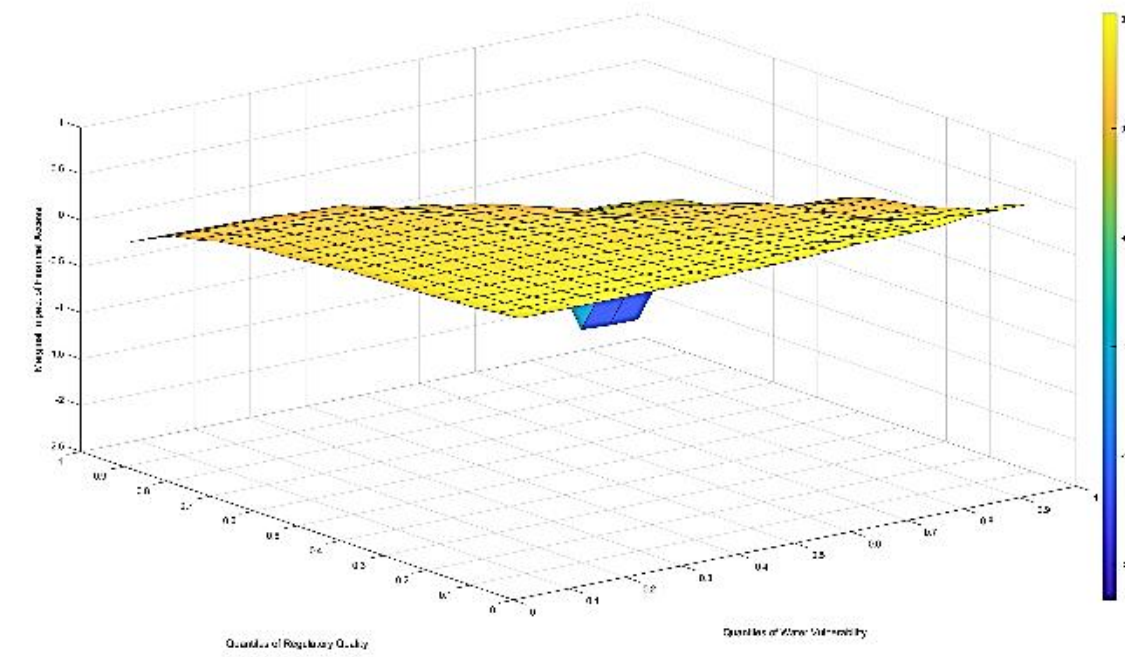

Governance Quality Indicator: Regulatory Quality

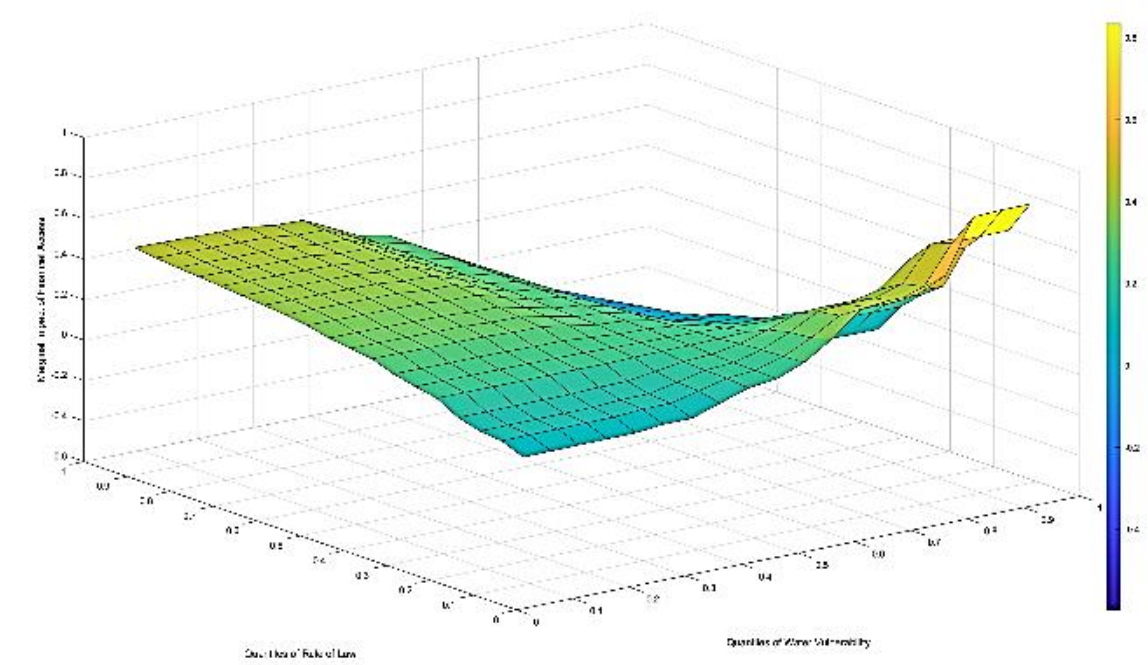

Governance Quality Indicator: Rule of Law

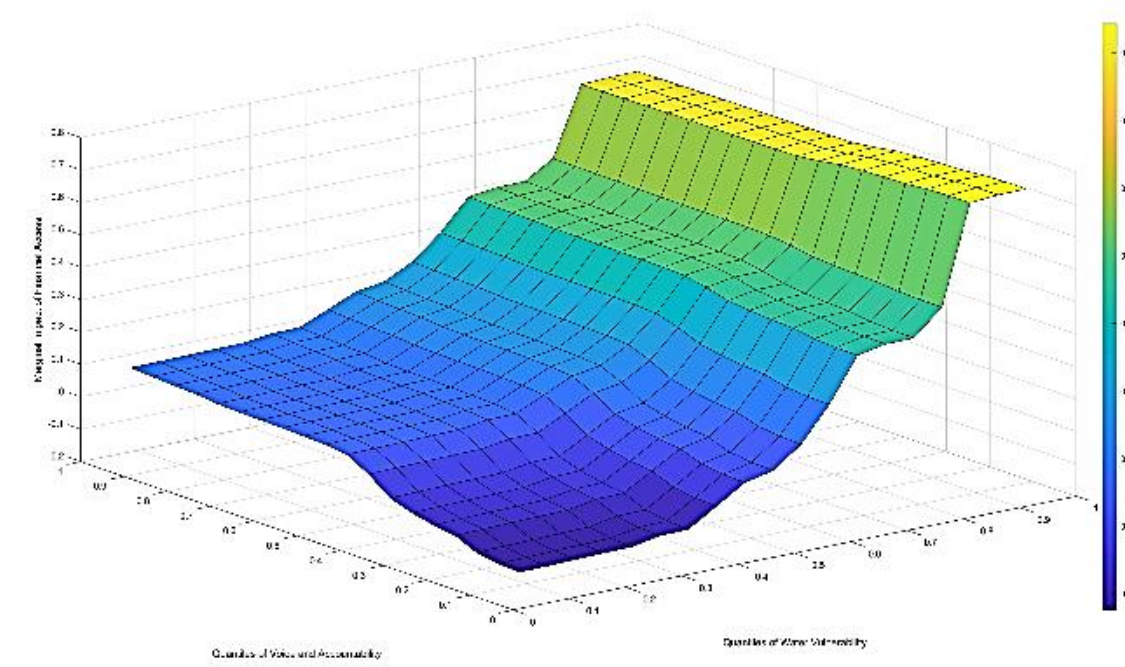

Governance Quality Indicator: Voice and Accountability

Figure S42: BRICS Policy Simulation Outcomes for the Marginal Impact of Financial Access on SDG 7.1.2 under Water Vulnerability and Governance Quality, Related to Figure 4-5 and Table 2

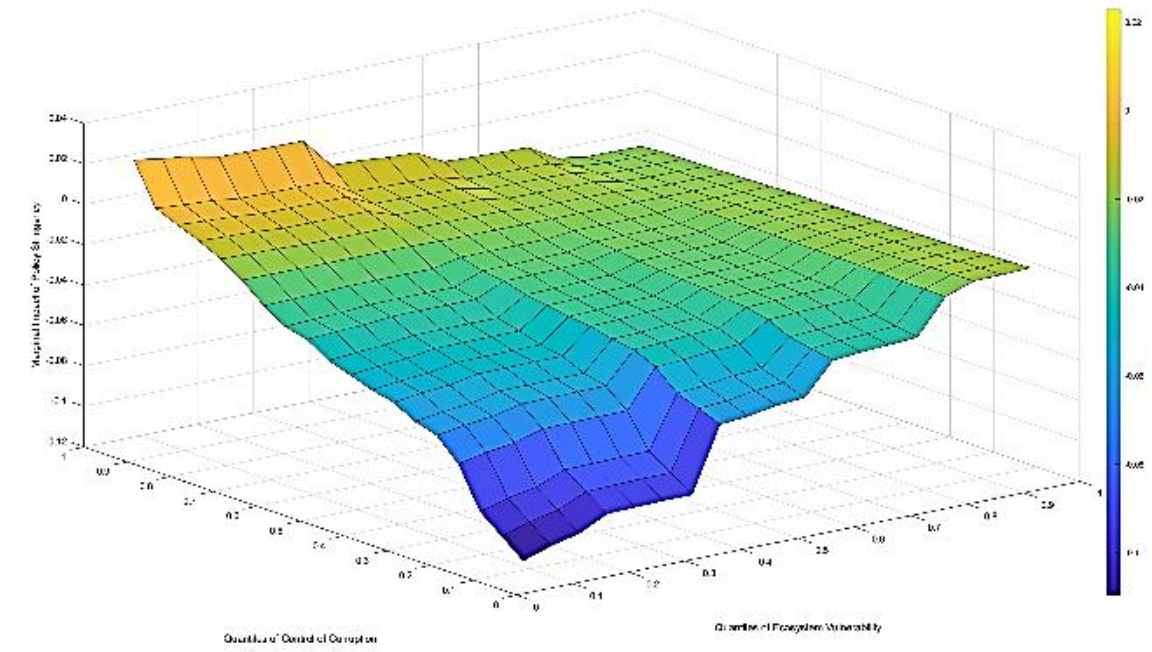

Governance Quality Indicator: Control of Corruption

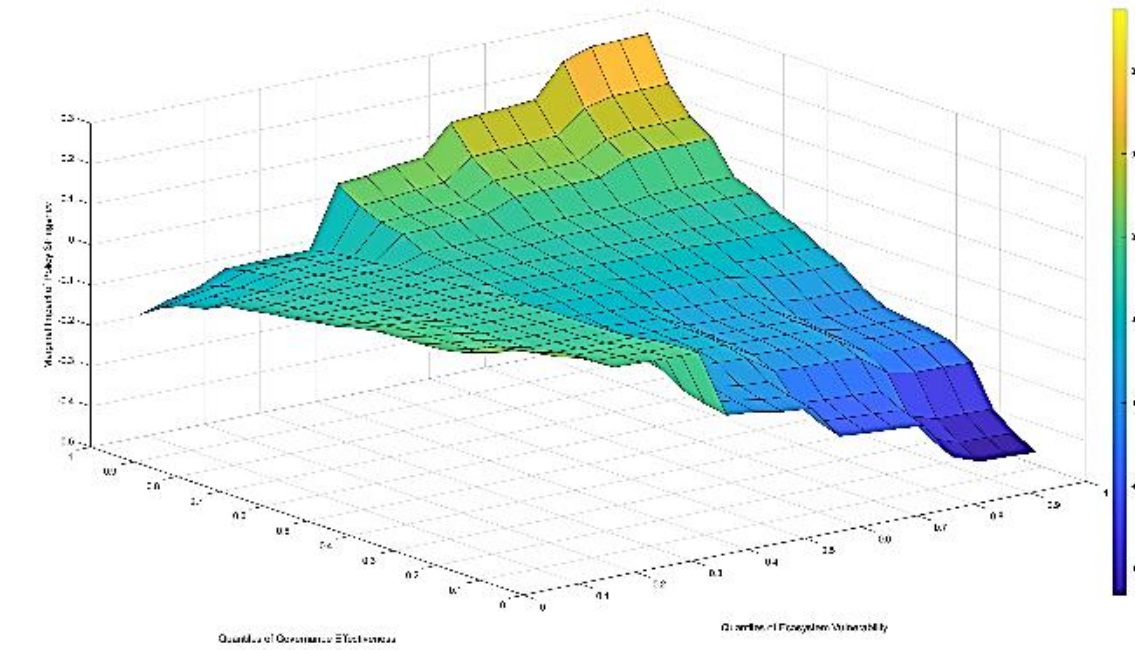

Governance Quality Indicator: Governance Effectiveness

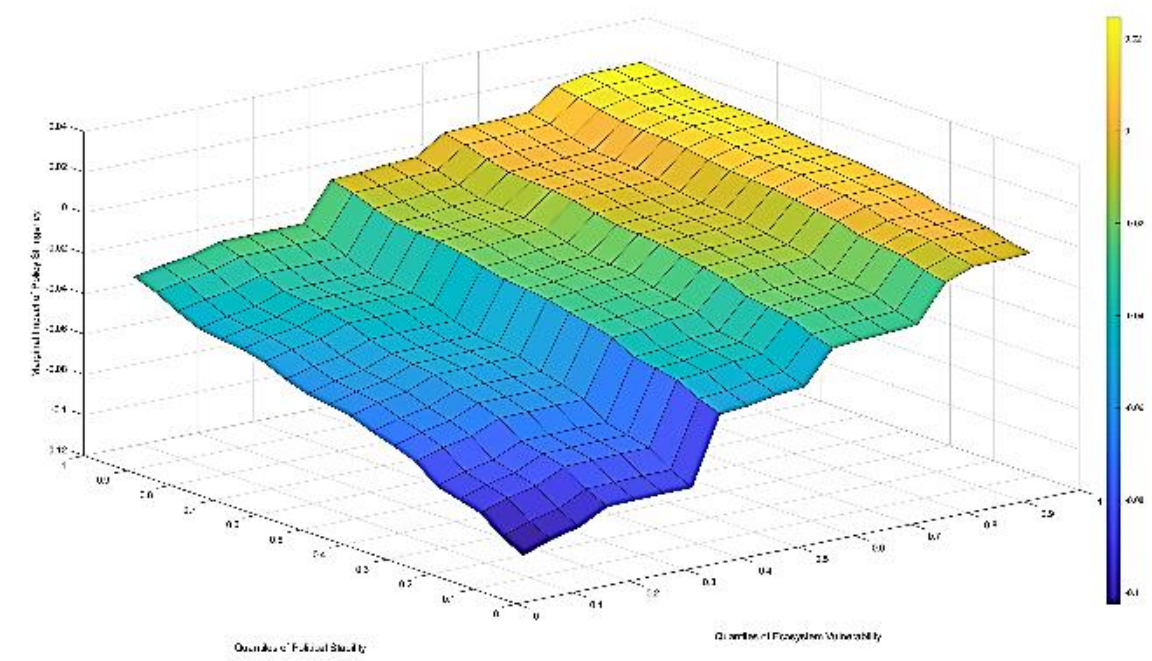

Governance Quality Indicator: Political Stability

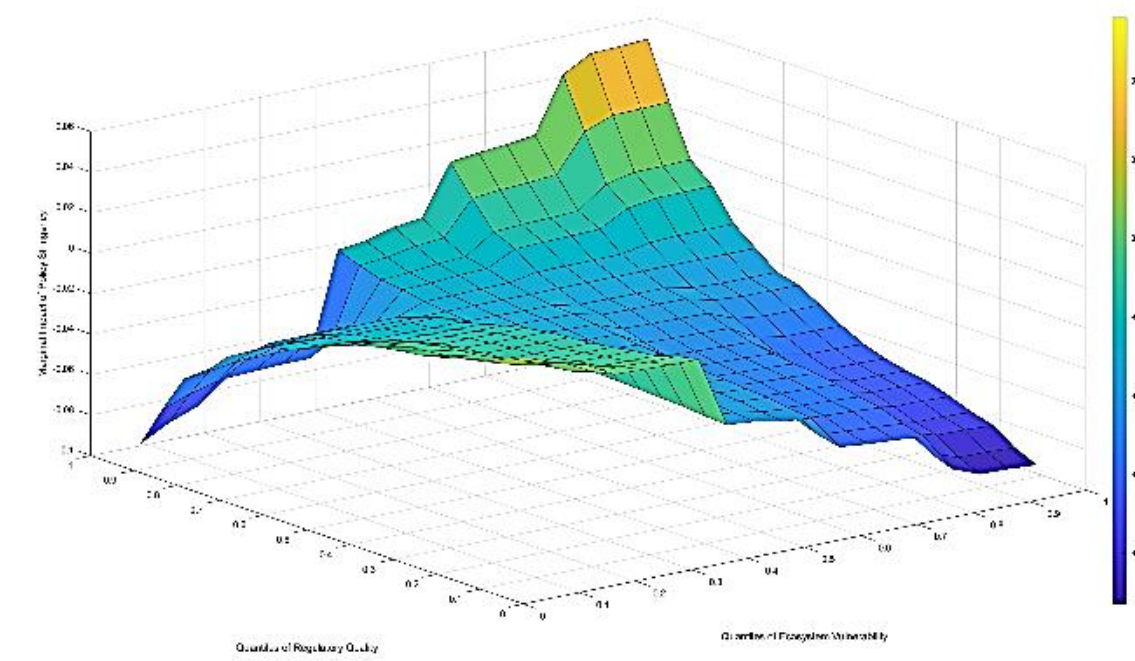

Governance Quality Indicator: Regulatory Quality

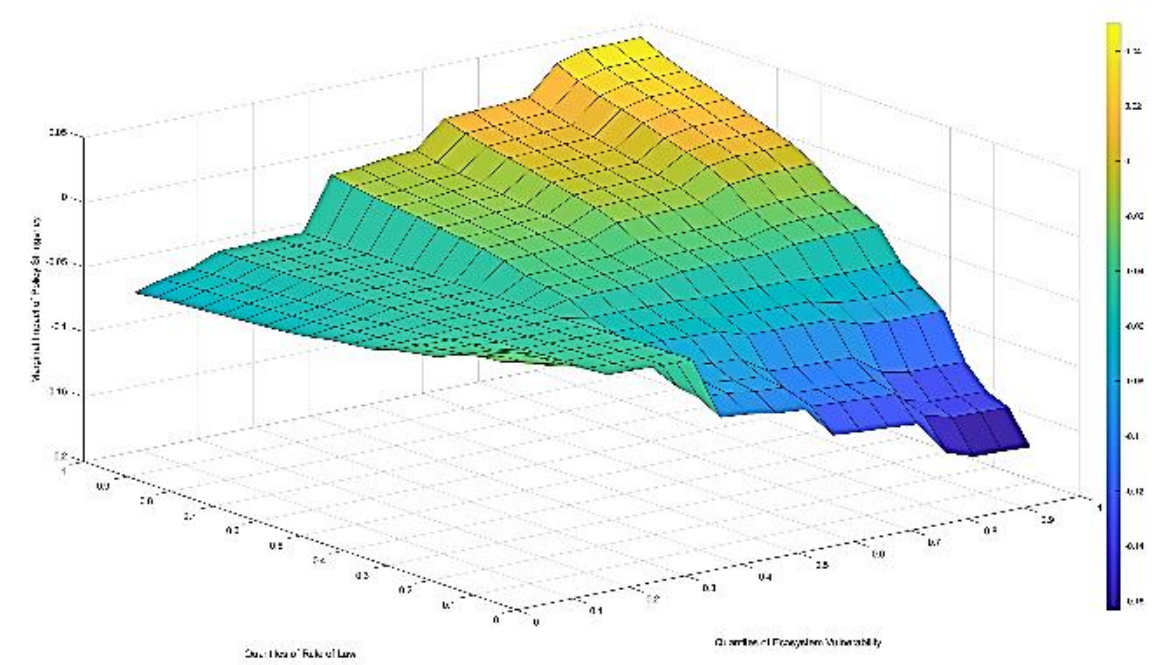

Governance Quality Indicator: Rule of Law

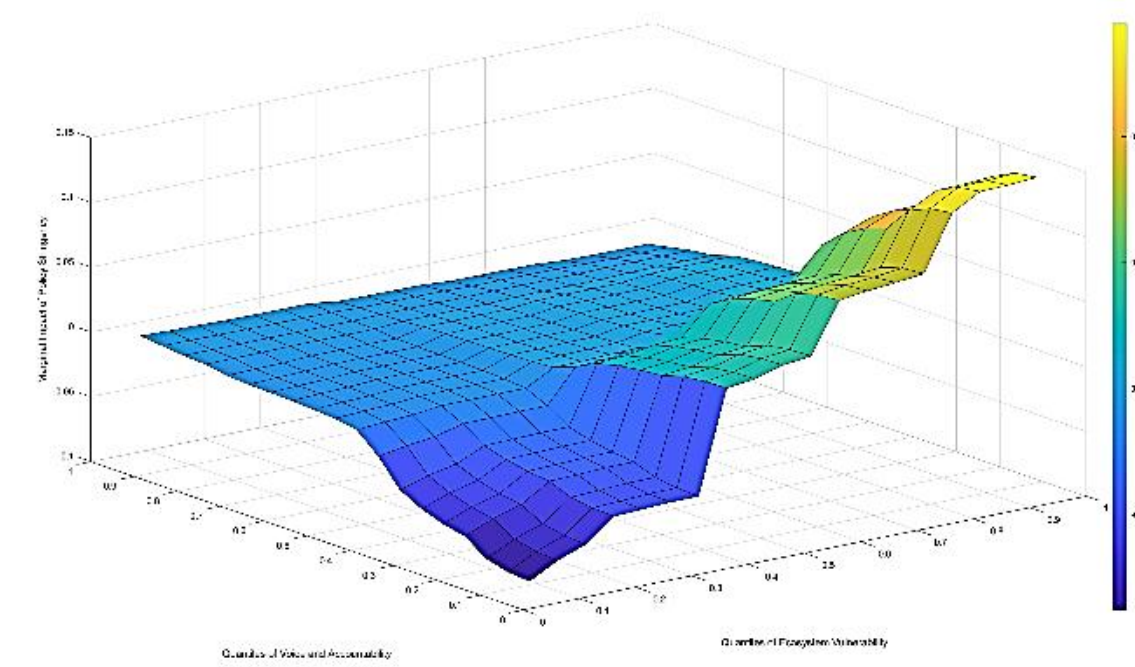

Governance Quality Indicator: Voice and Accountability

Figure S43: BRICS Policy Simulation Outcomes for the Marginal Impact of Policy Stringency on SDG 7.1.2 under Ecosystem Vulnerability and Governance Quality, Related to Figure 4-5 and Table 2

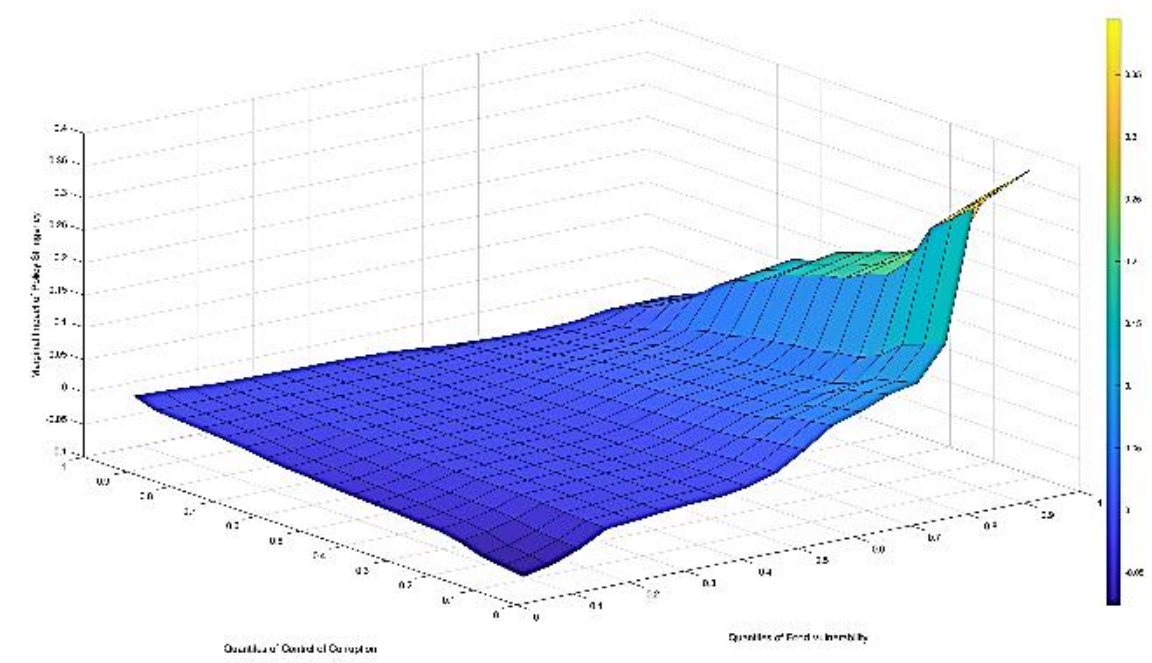

Governance Quality Indicator: Control of Corruption

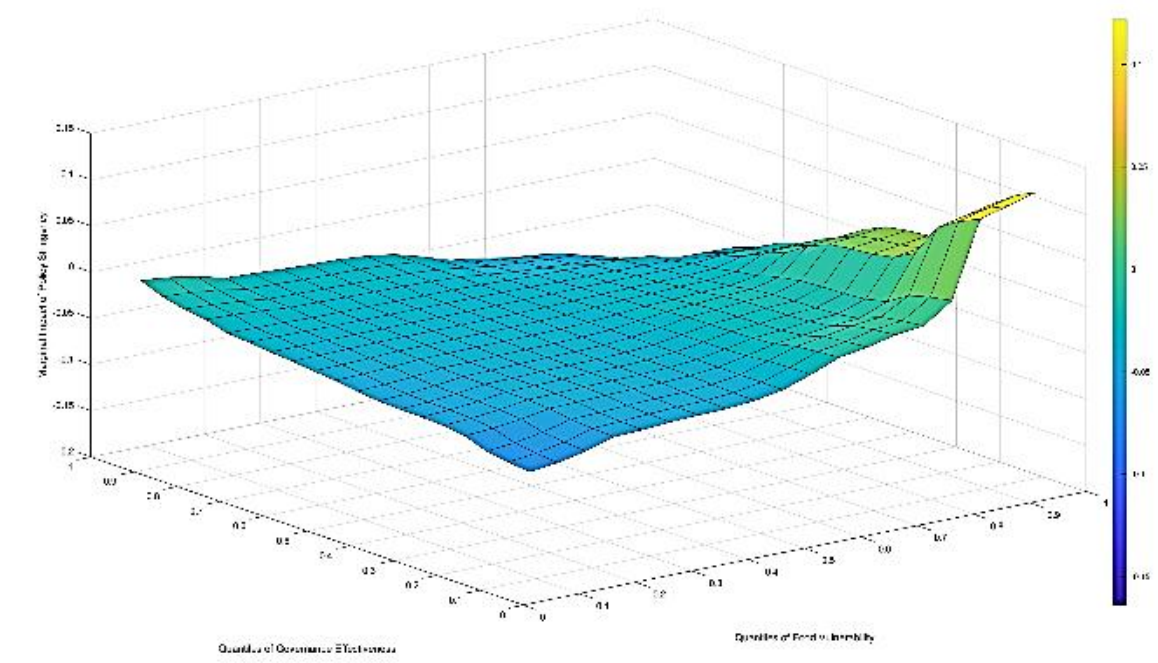

Governance Quality Indicator: Governance Effectiveness

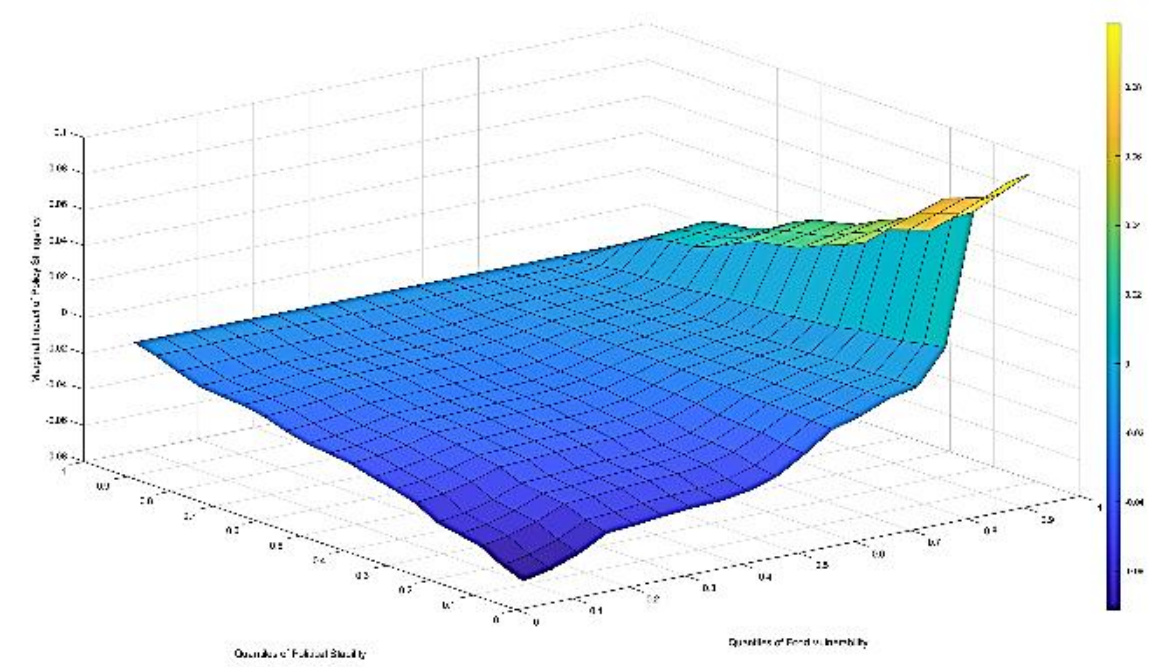

Governance Quality Indicator: Political Stability

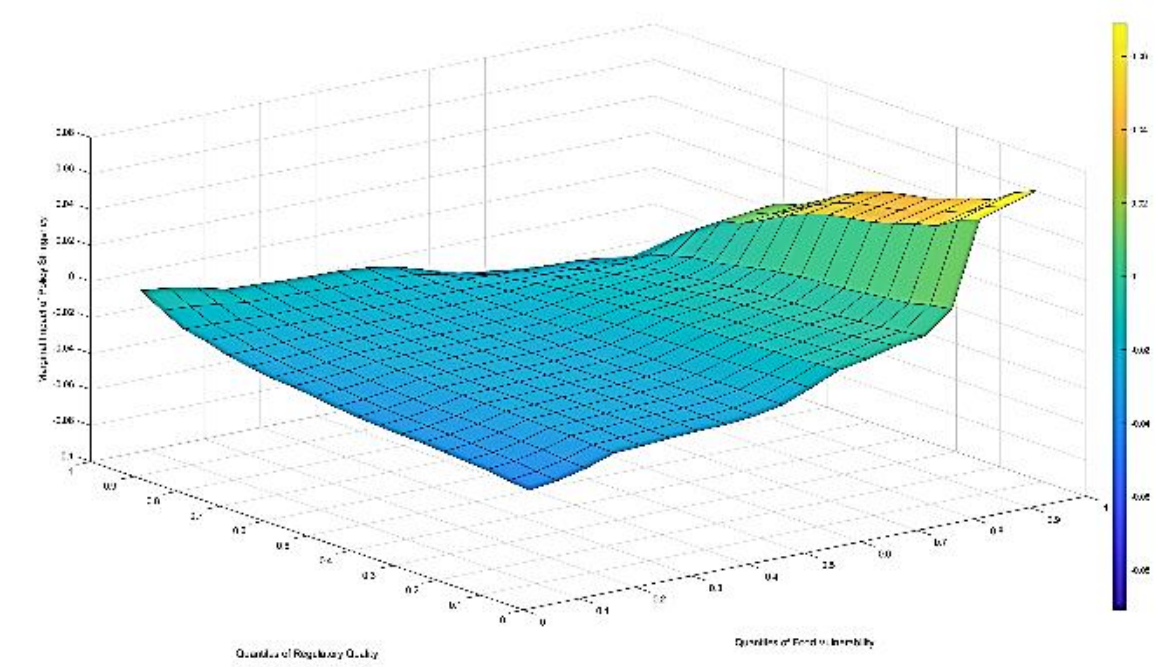

Governance Quality Indicator: Regulatory Quality

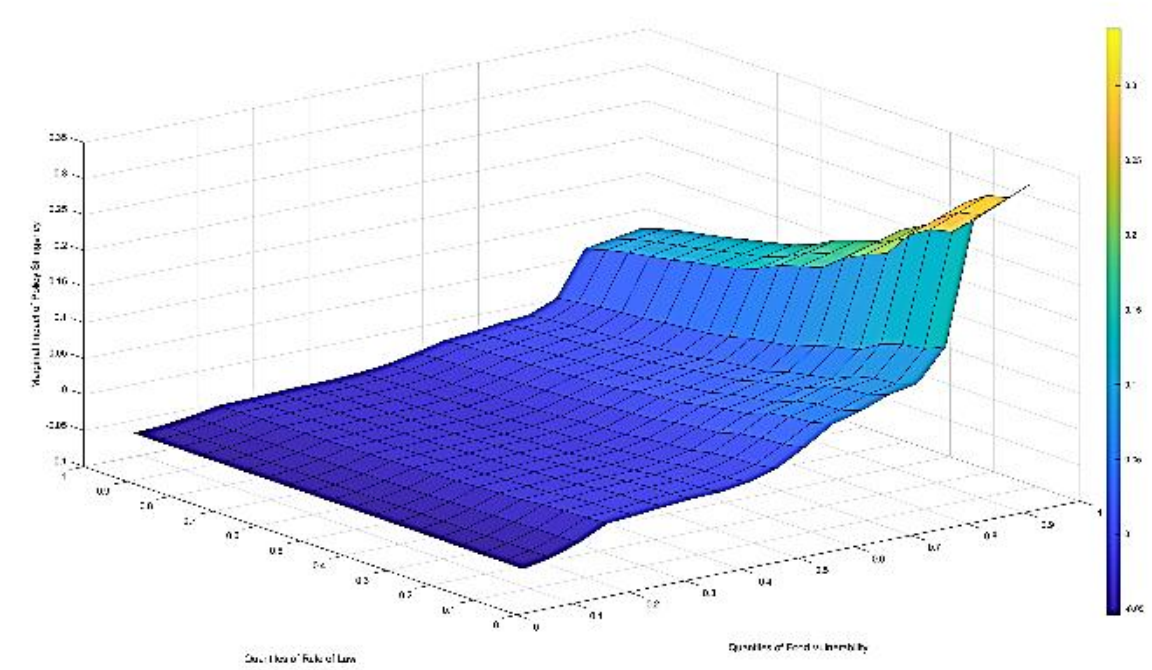

Governance Quality Indicator: Rule of Law

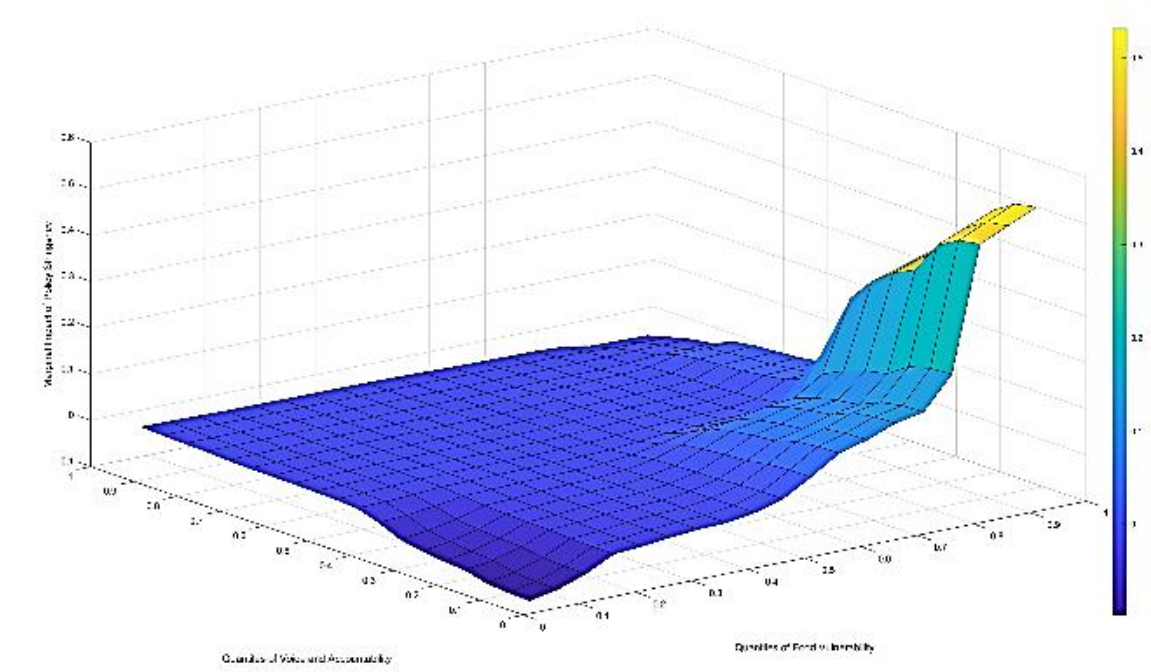

Governance Quality Indicator: Voice and Accountability

Figure S44: BRICS Policy Simulation Outcomes for the Marginal Impact of Policy Stringency on SDG 7.1.2 under Food Vulnerability and Governance Quality, Related to Figure 4-5 and Table 2

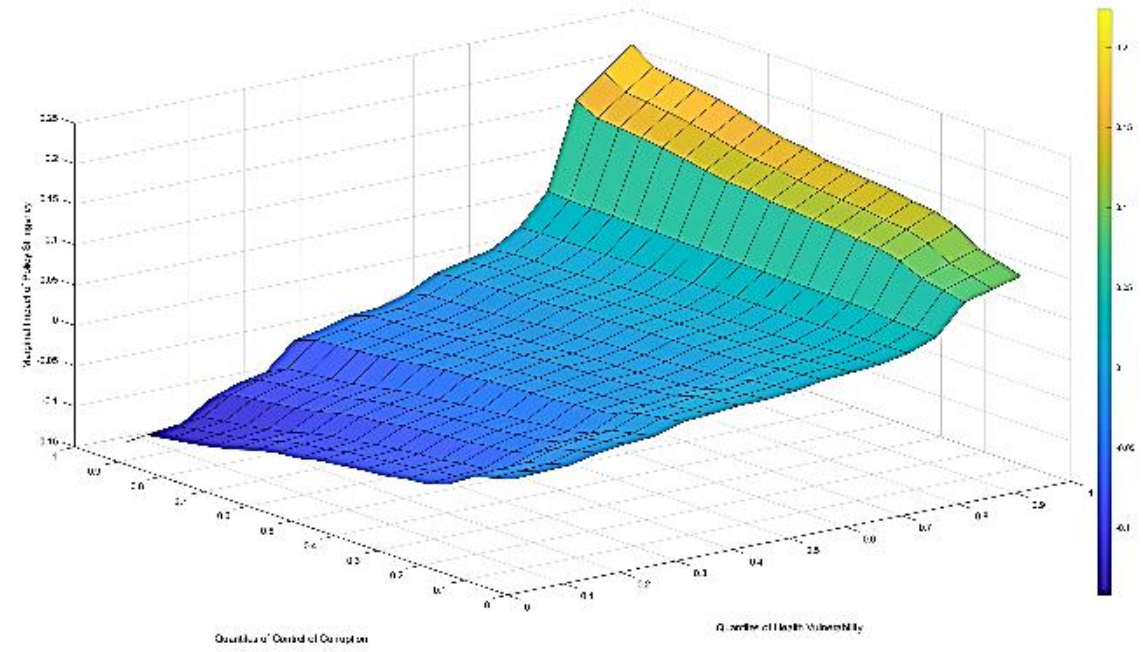

Governance Quality Indicator: Control of Corruption

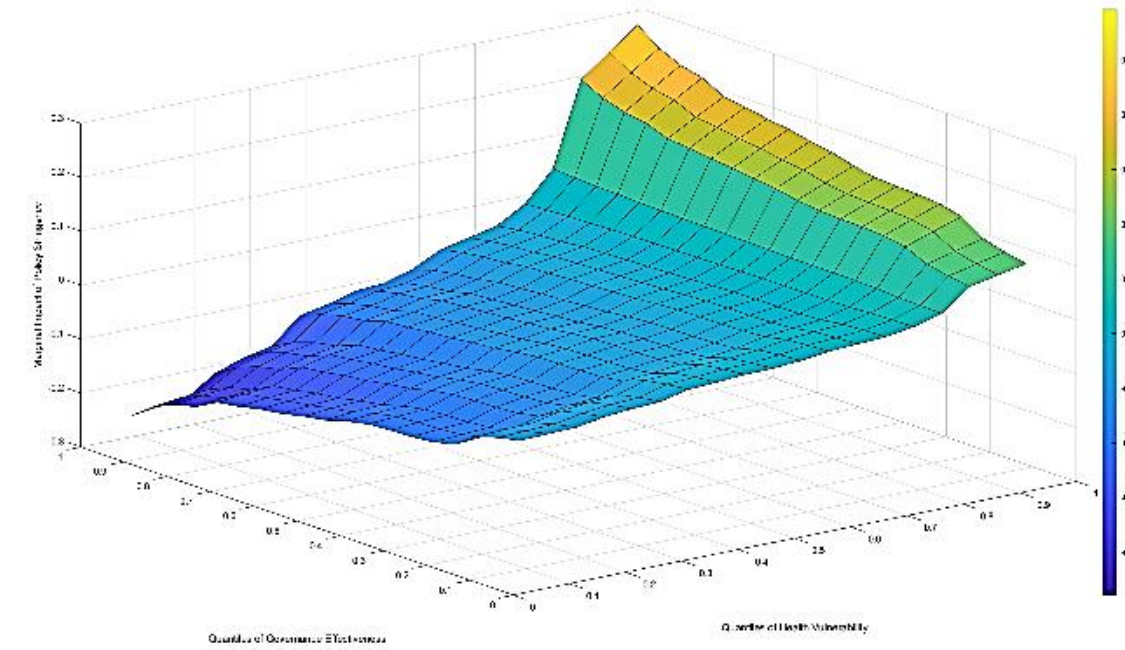

Governance Quality Indicator: Governance Effectiveness

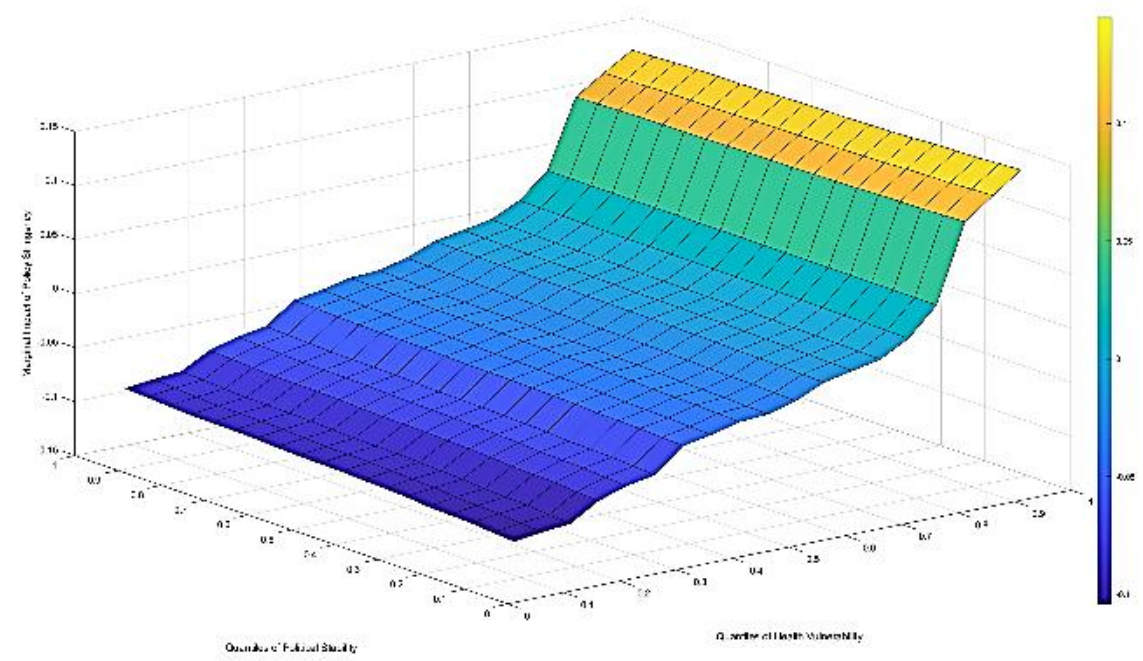

Governance Quality Indicator: Political Stability

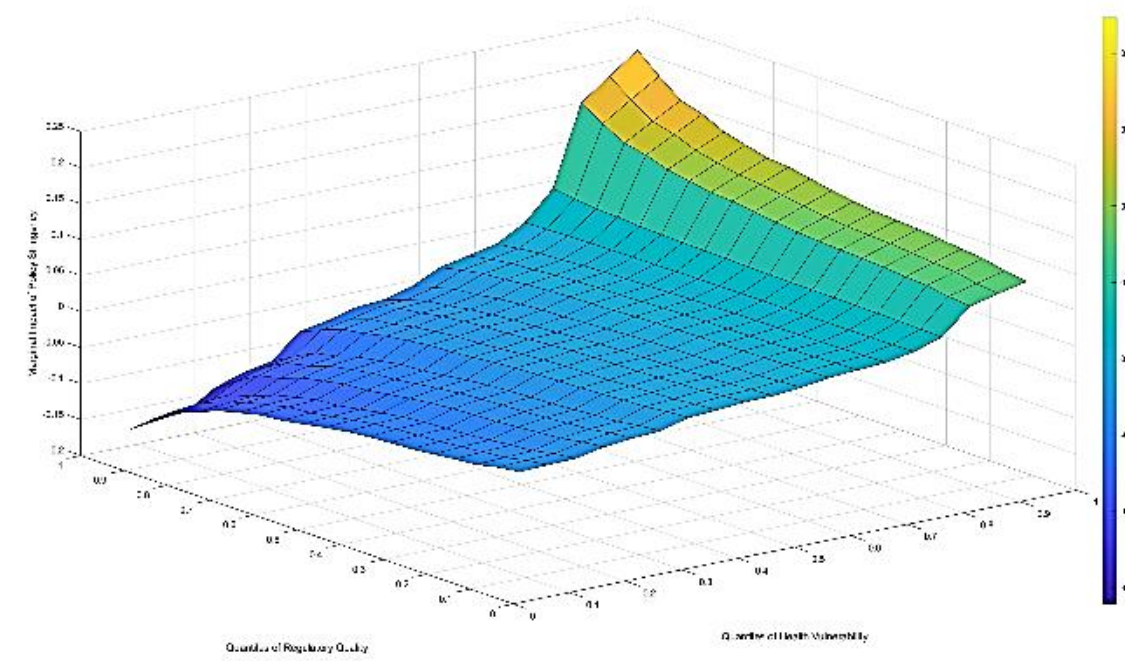

Governance Quality Indicator: Regulatory Quality

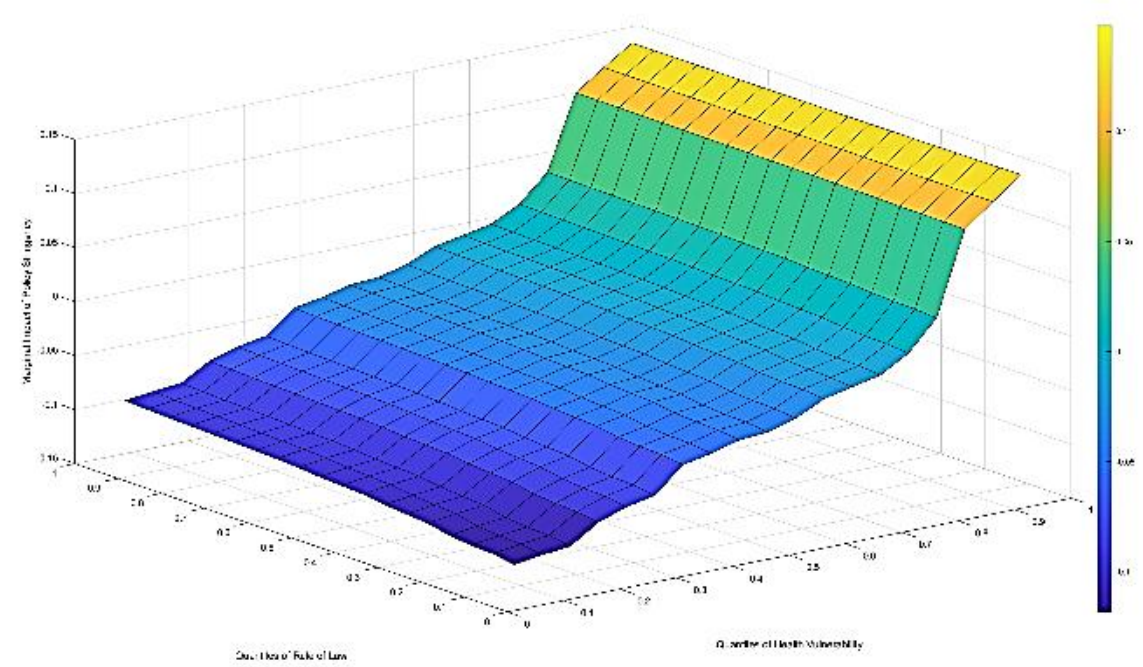

Governance Quality Indicator: Rule of Law

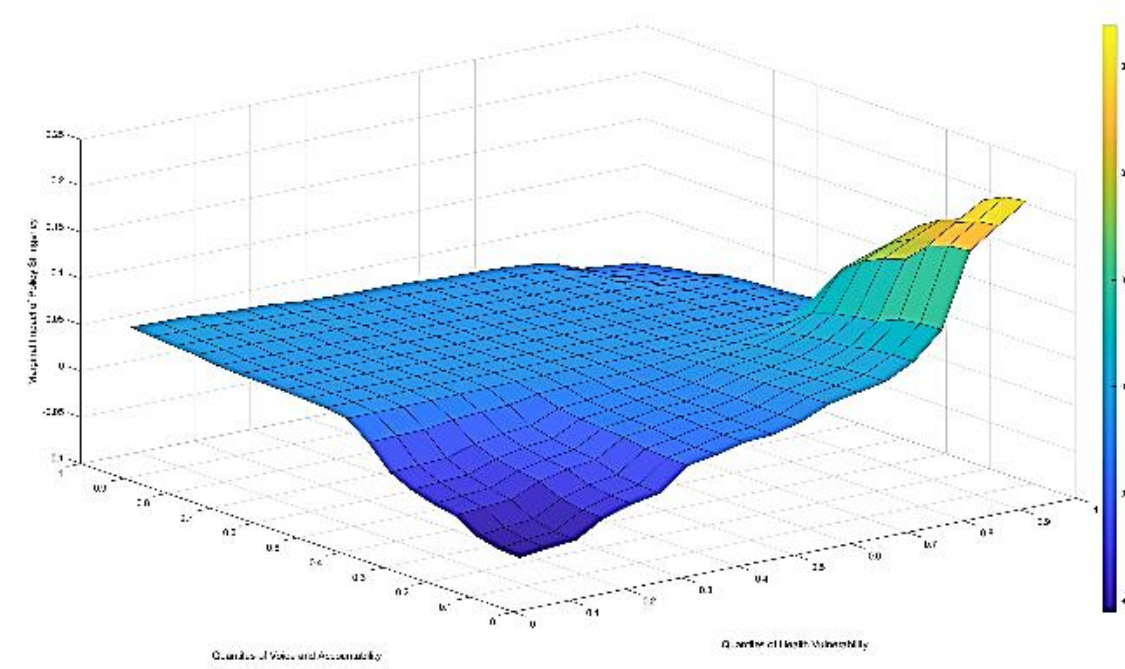

Governance Quality Indicator: Voice and Accountability

Figure S45: BRICS Policy Simulation Outcomes for the Marginal Impact of Policy Stringency on SDG 7.1.2 under Health Vulnerability and Governance Quality, Related to Figure 4-5 and Table 2

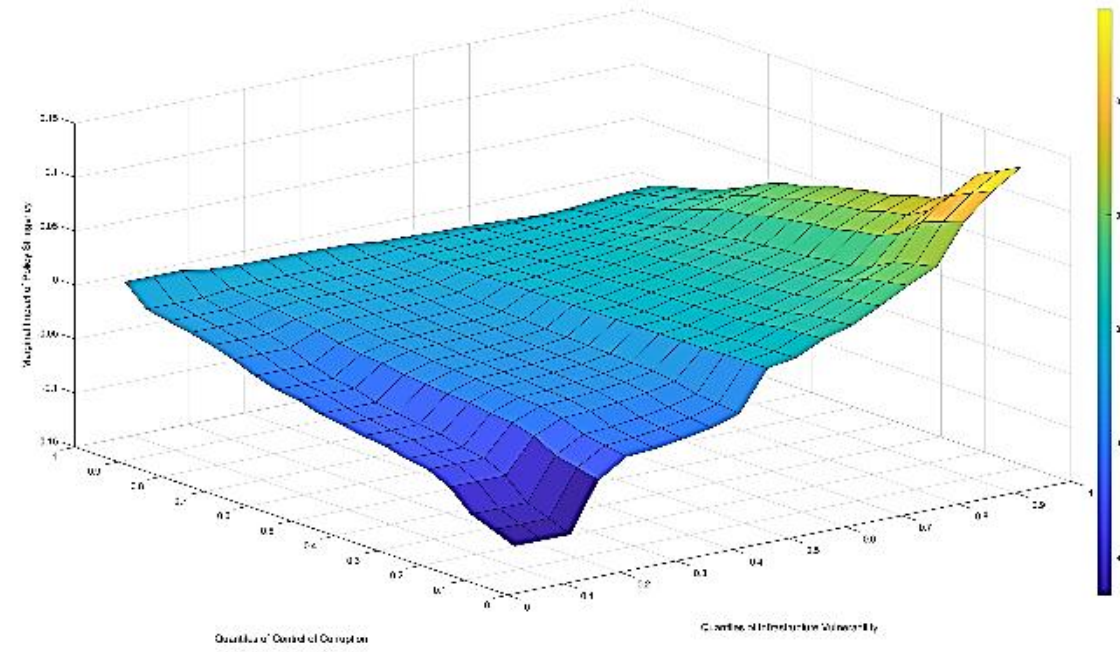

Governance Quality Indicator: Control of Corruption

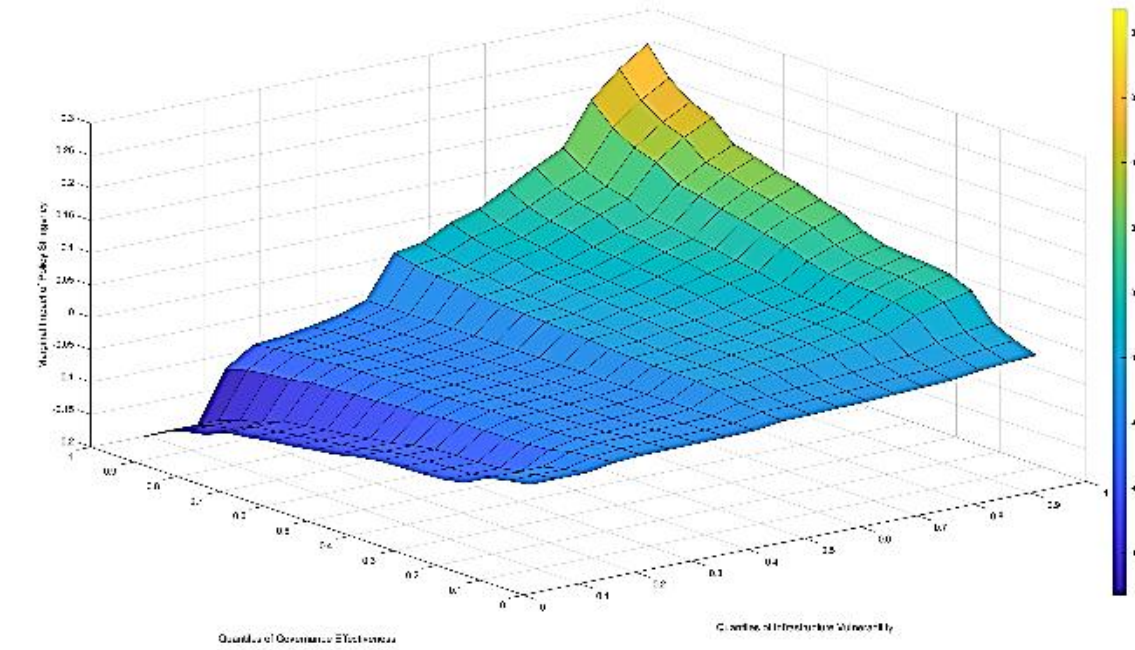

Governance Quality Indicator: Governance Effectiveness

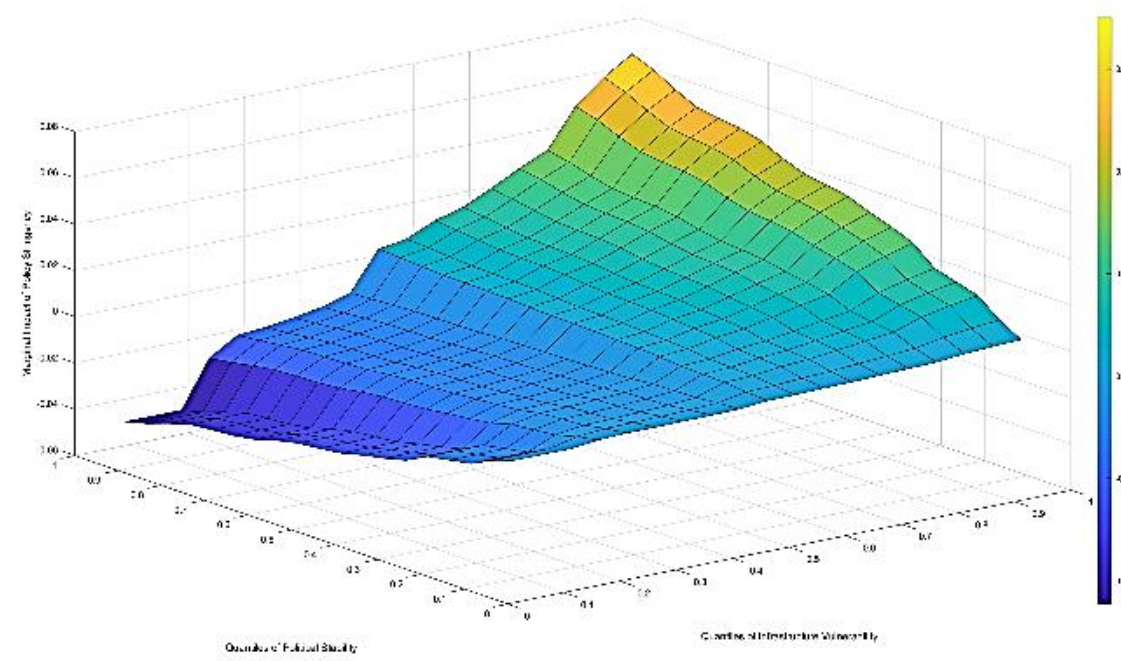

Governance Quality Indicator: Political Stability

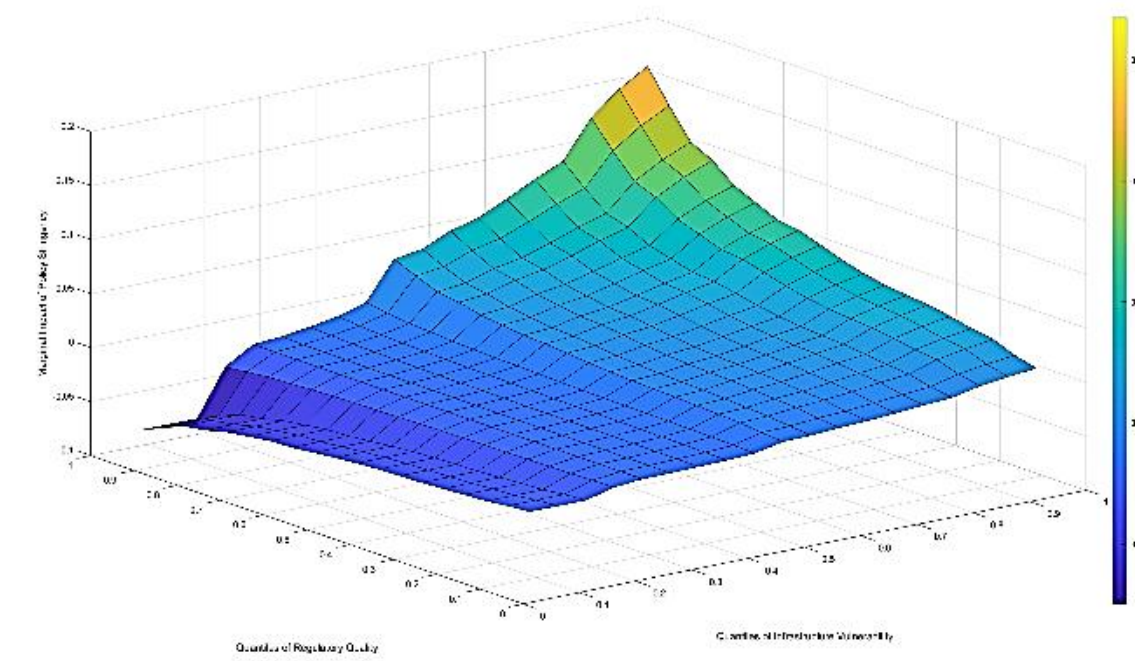

Governance Quality Indicator: Regulatory Quality

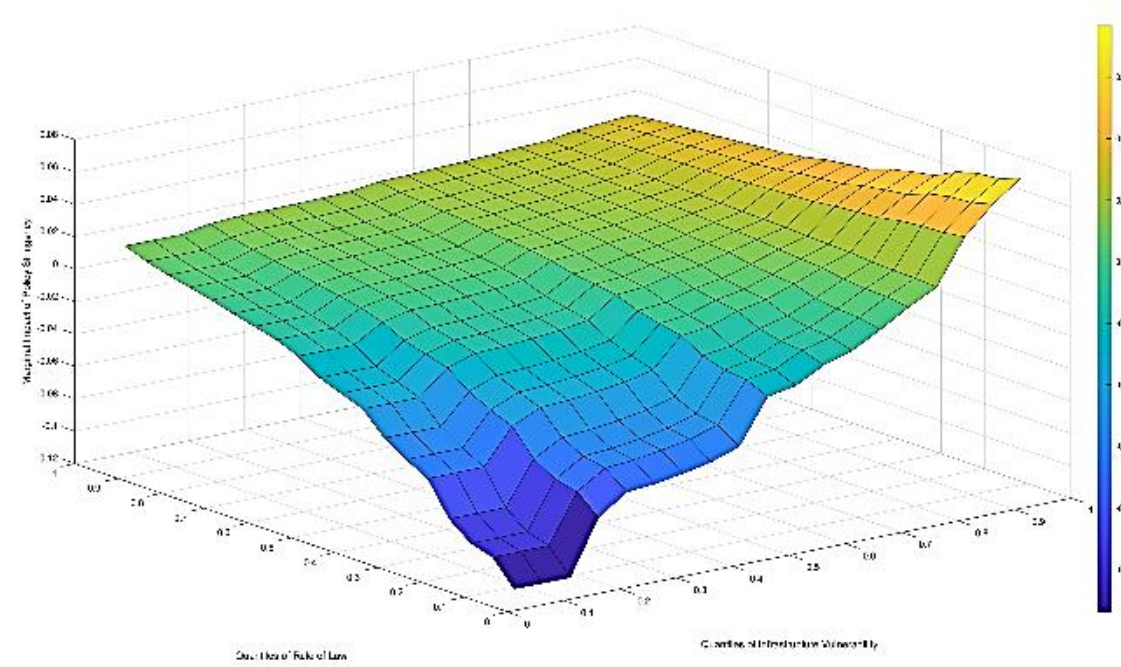

Governance Quality Indicator: Rule of Law

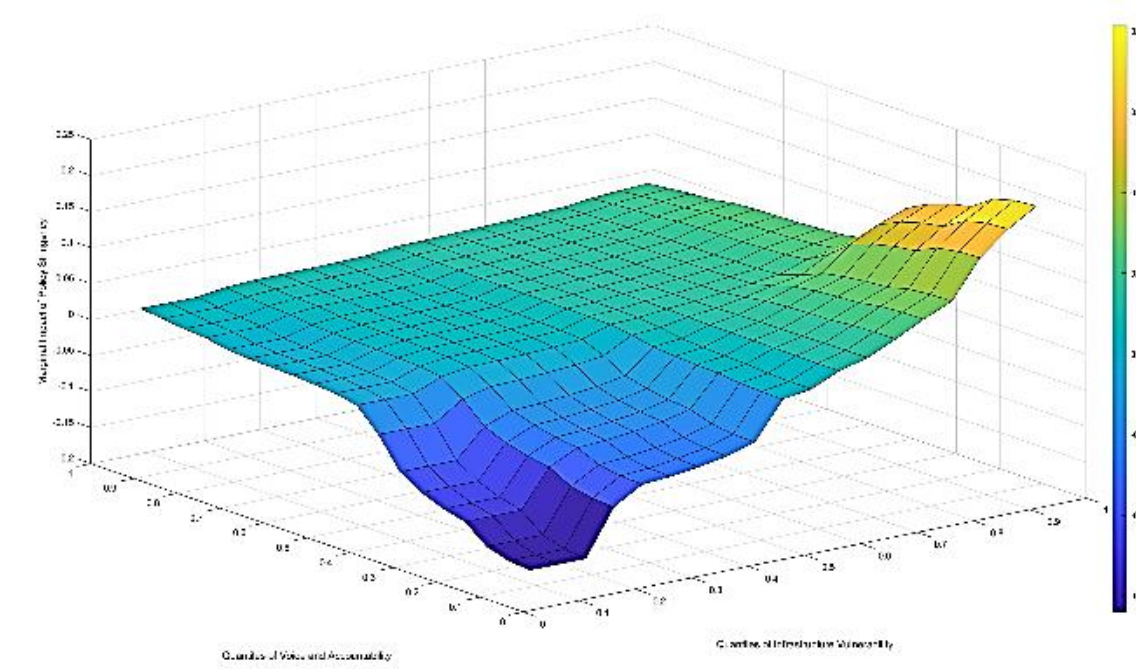

Governance Quality Indicator: Voice and Accountability

Figure S46: BRICS Policy Simulation Outcomes for the Marginal Impact of Policy Stringency on SDG 7.1.2 under Infrastructure Vulnerability and Governance Quality, Related to Figure 4-5 and Table 2

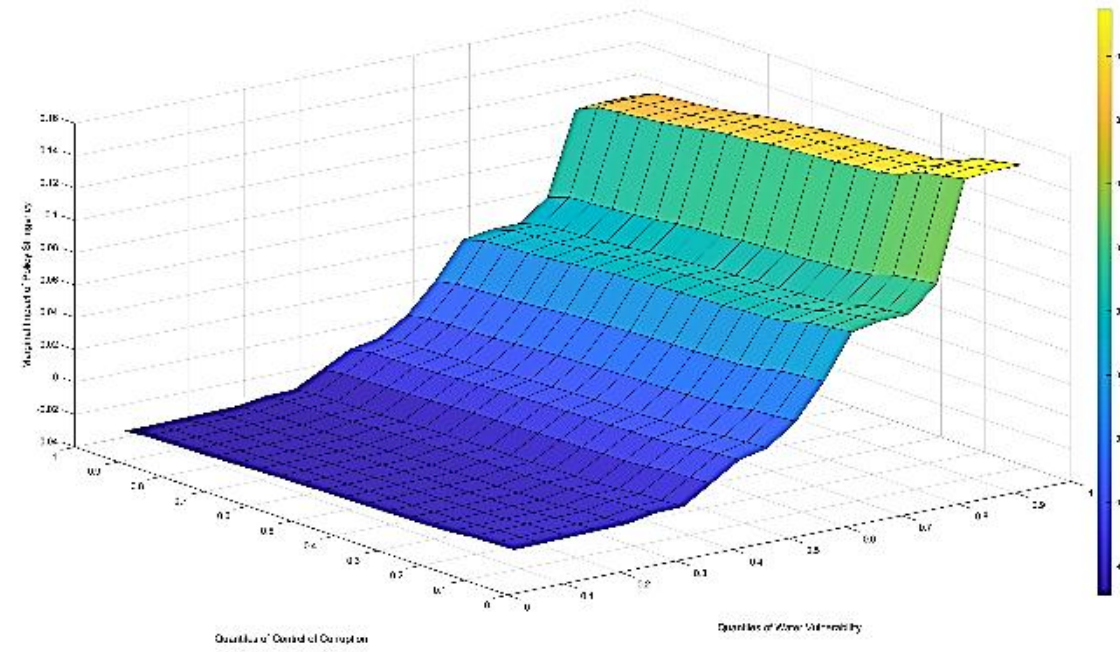

Governance Quality Indicator: Control of Corruption

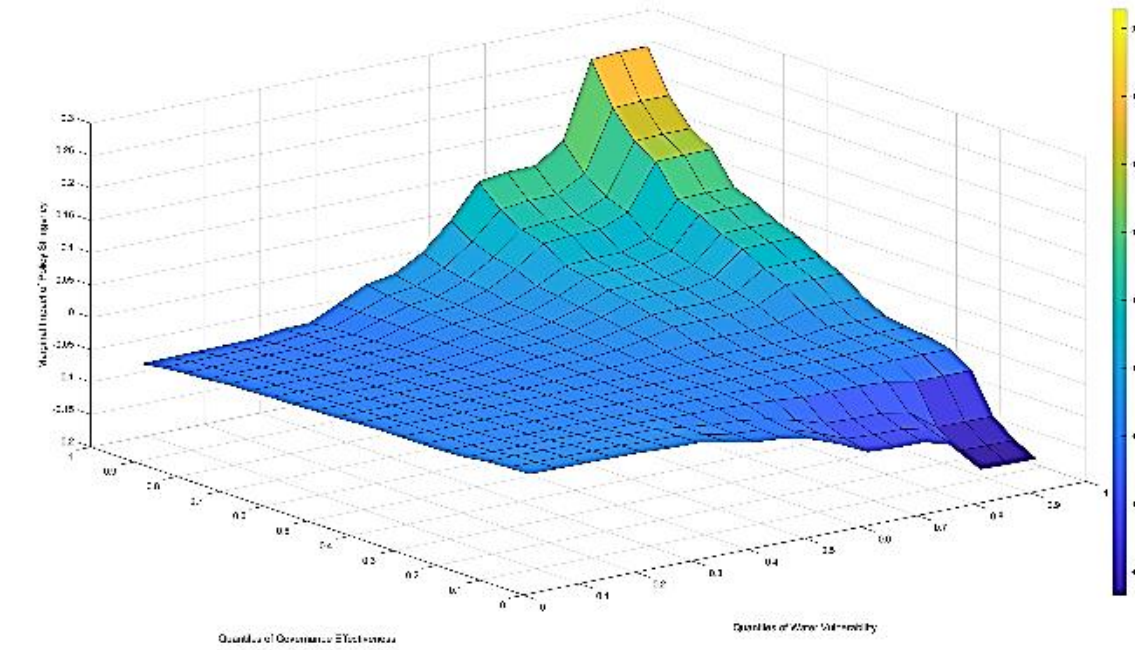

Governance Quality Indicator: Governance Effectiveness

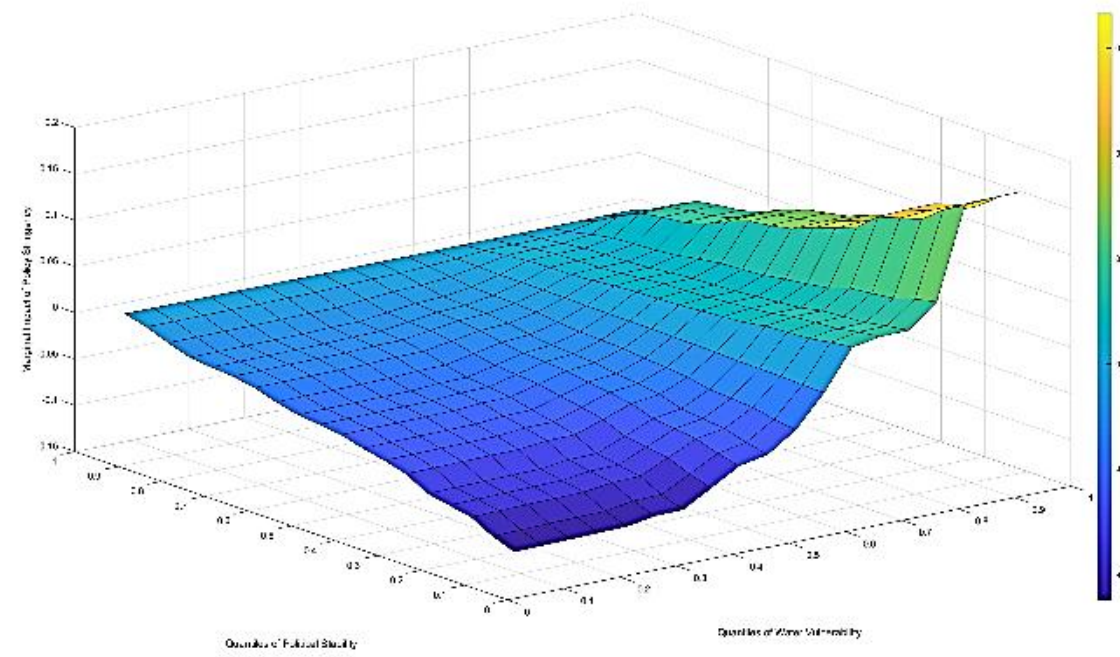

Governance Quality Indicator: Political Stability

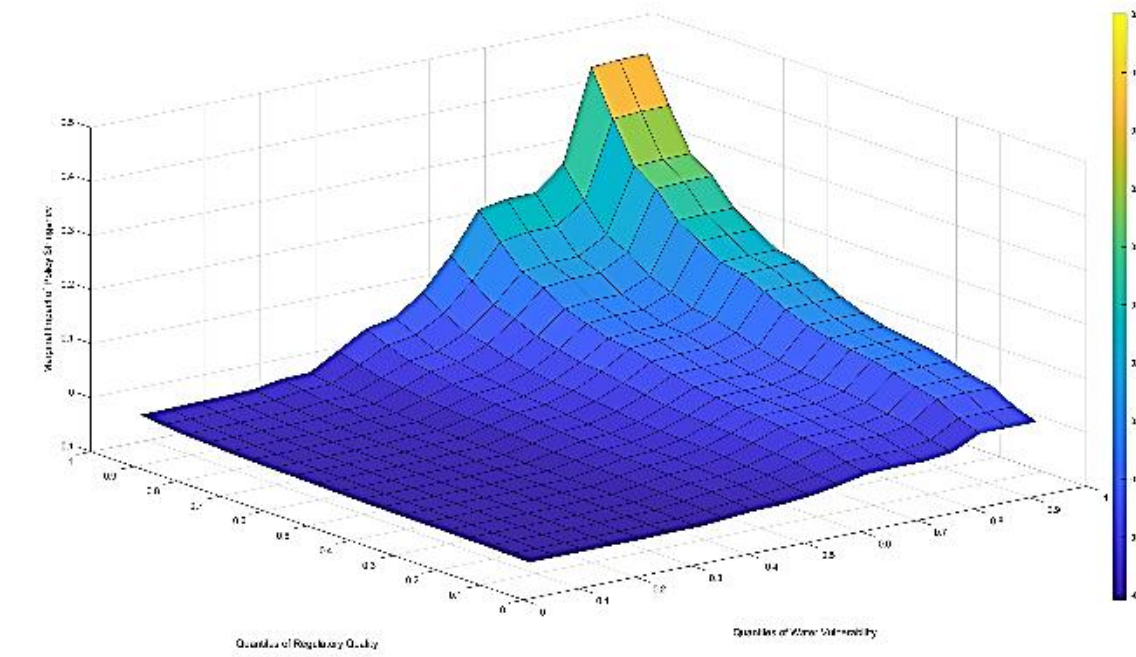

Governance Quality Indicator: Regulatory Quality

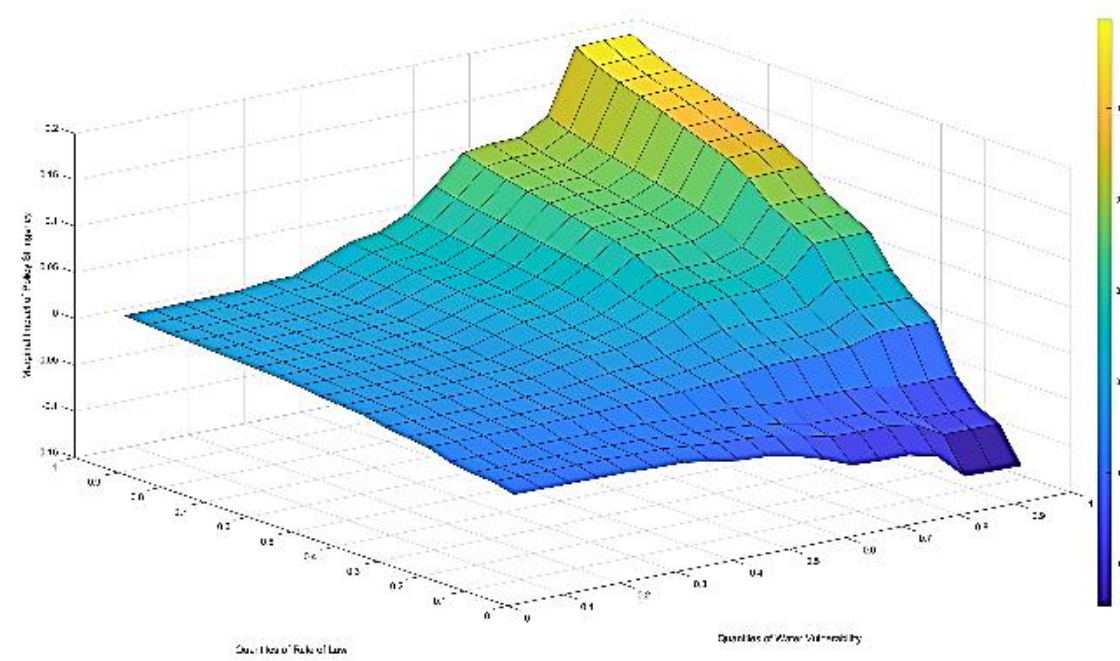

Governance Quality Indicator: Rule of Law

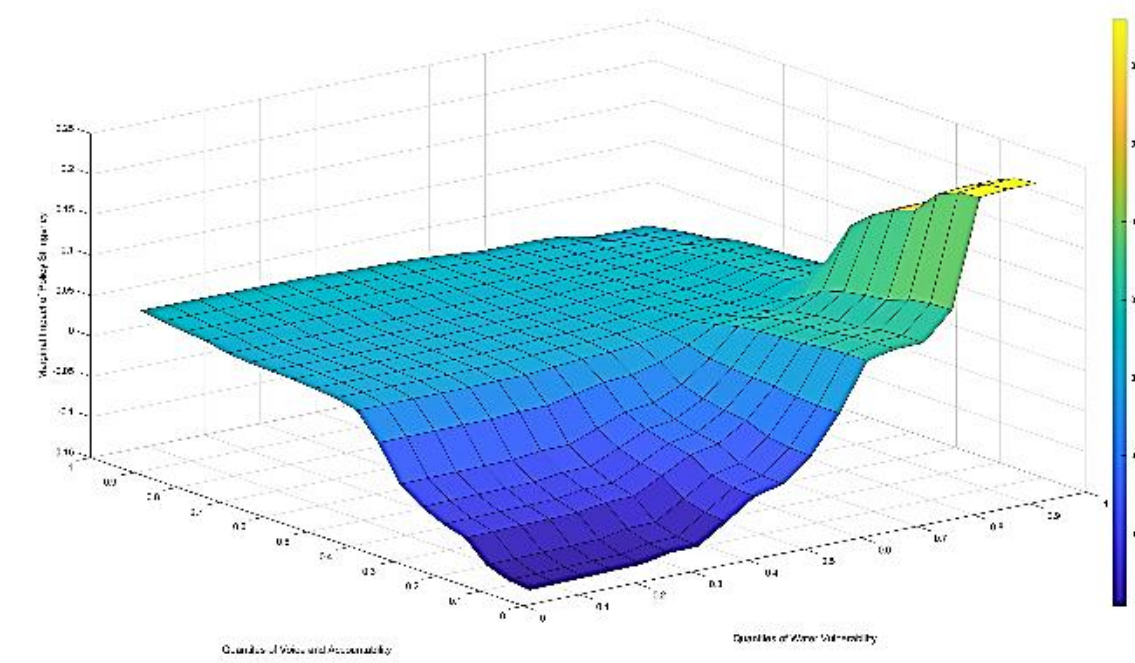

Governance Quality Indicator: Voice and Accountability

Figure S47: BRICS Policy Simulation Outcomes for the Marginal Impact of Policy Stringency on SDG 7.1.2 under Water Vulnerability and Governance Quality, Related to Figure 4-5 and Table 2

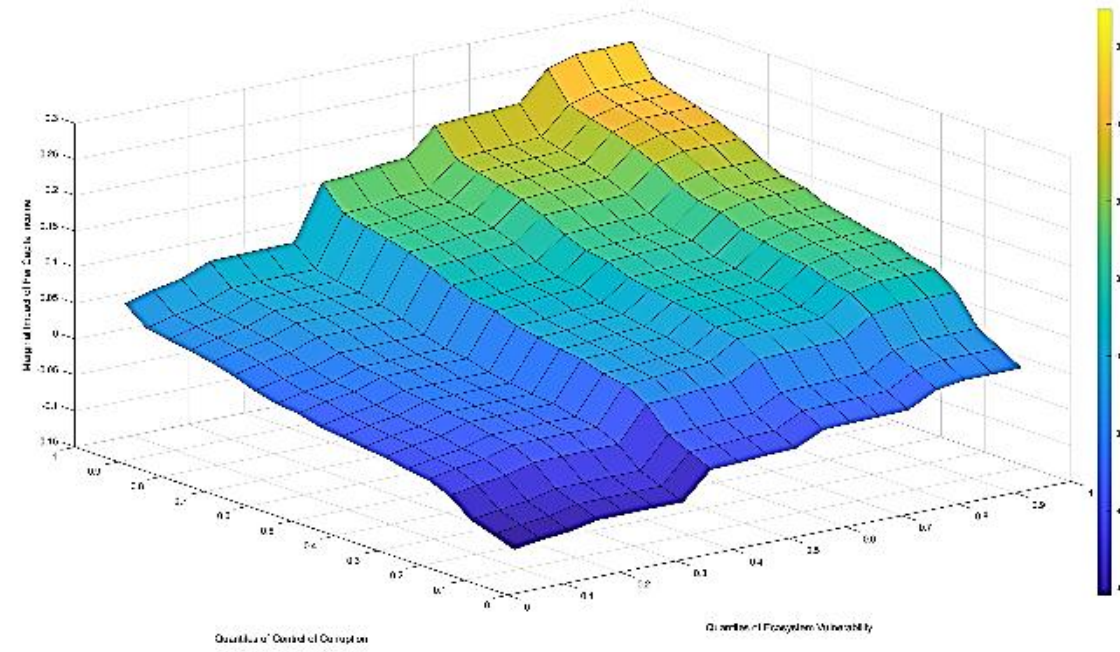

Governance Quality Indicator: Control of Corruption

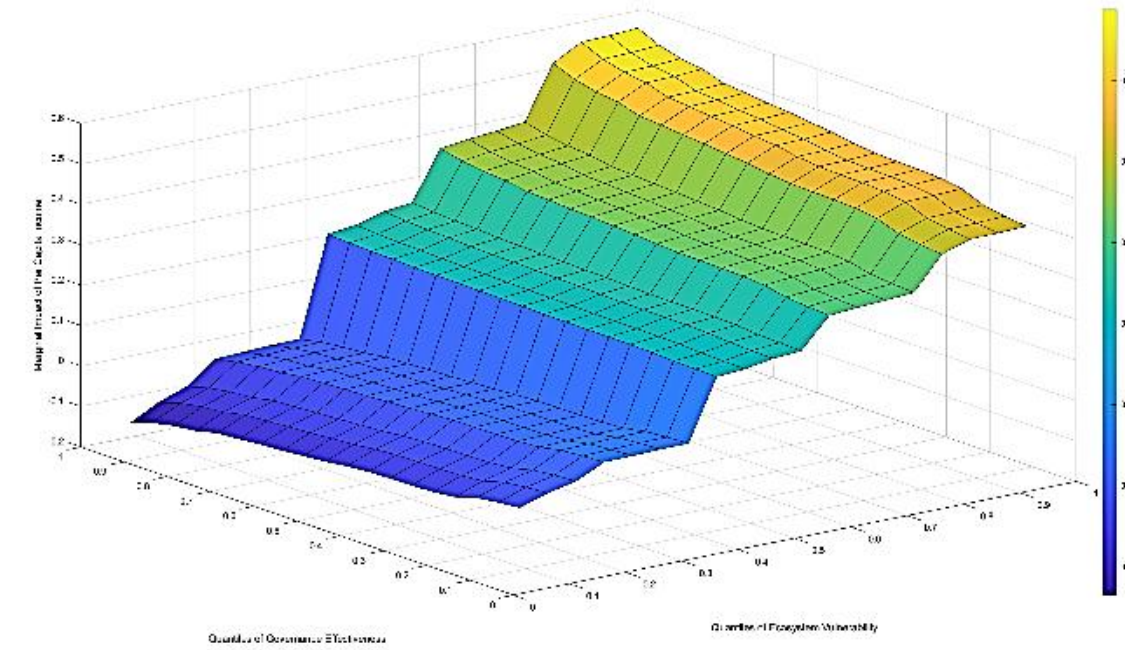

Governance Quality Indicator: Governance Effectiveness

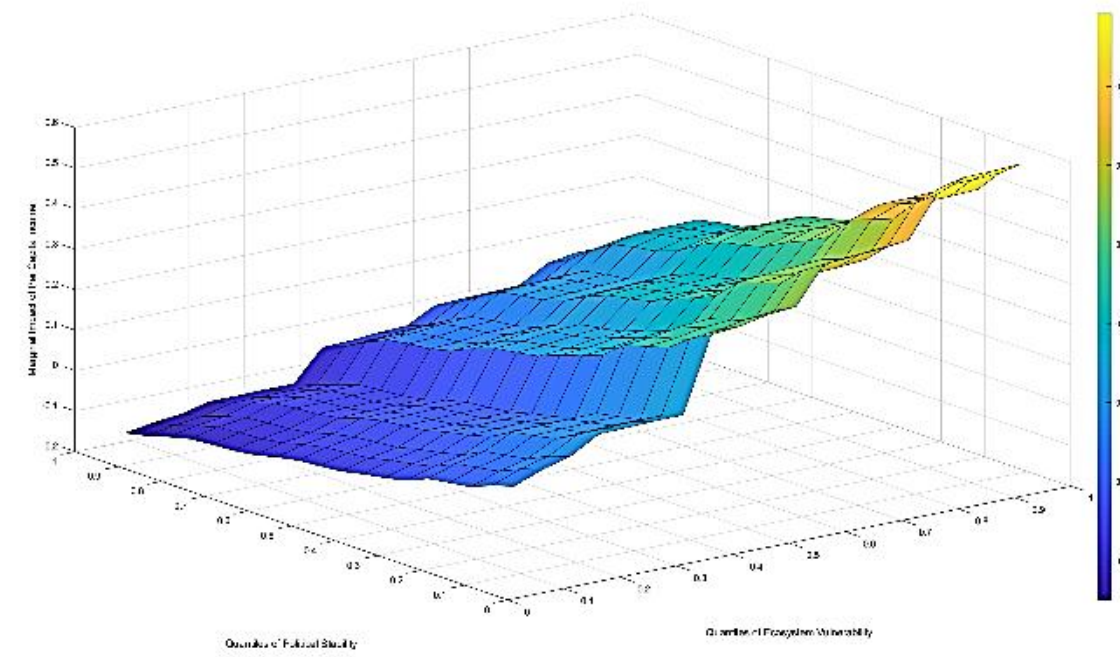

Governance Quality Indicator: Political Stability

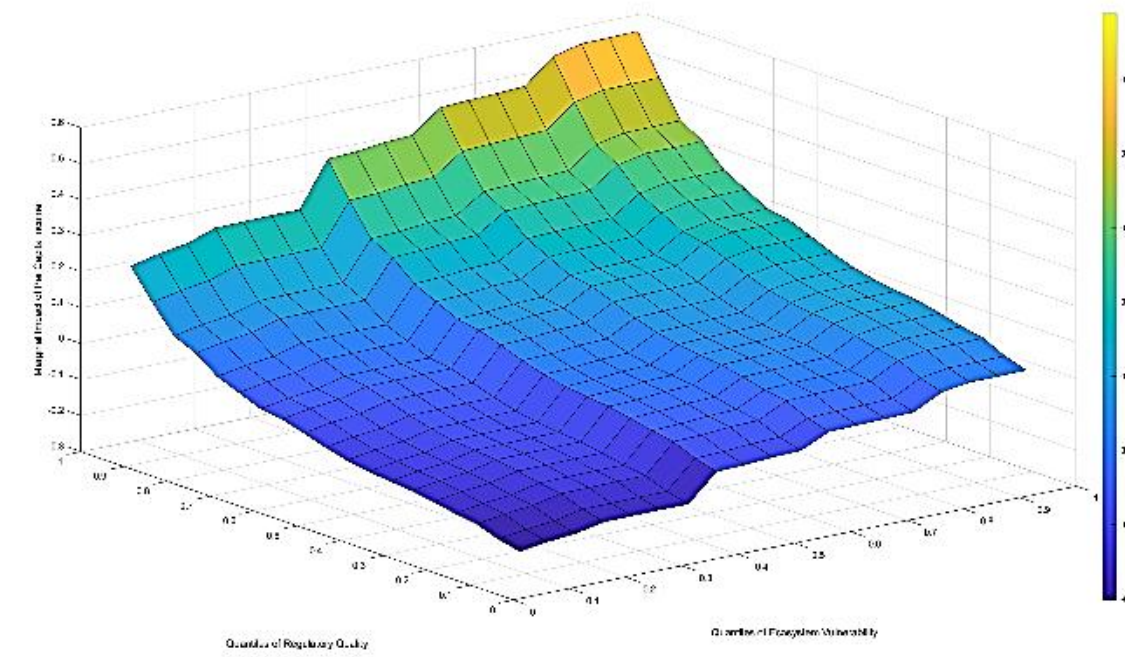

Governance Quality Indicator: Regulatory Quality

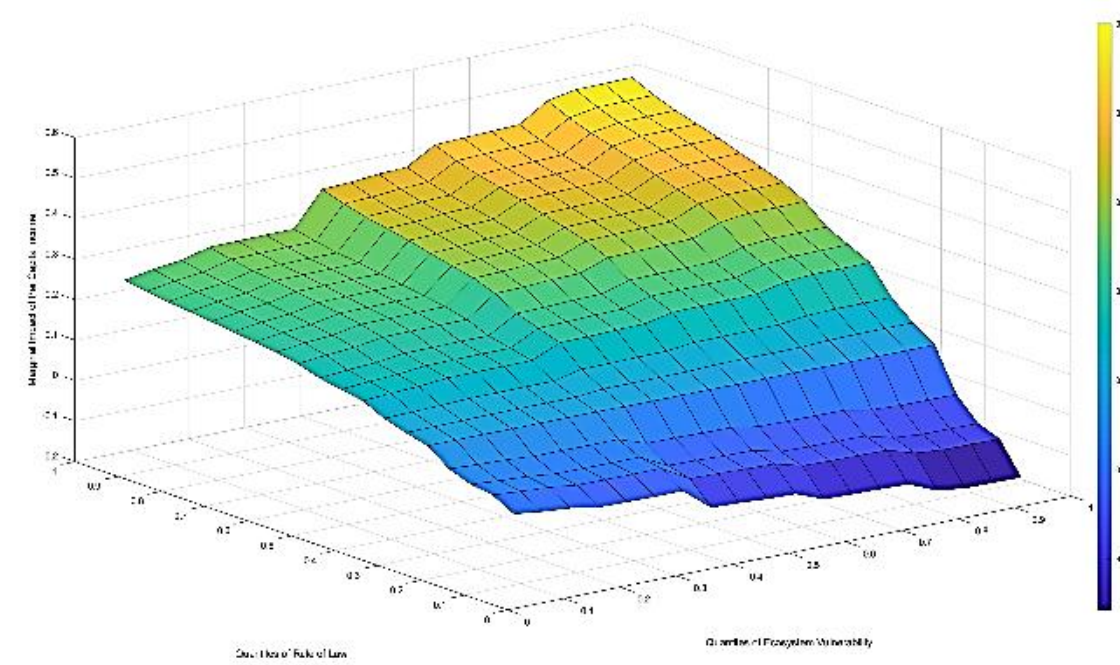

Governance Quality Indicator: Rule of Law

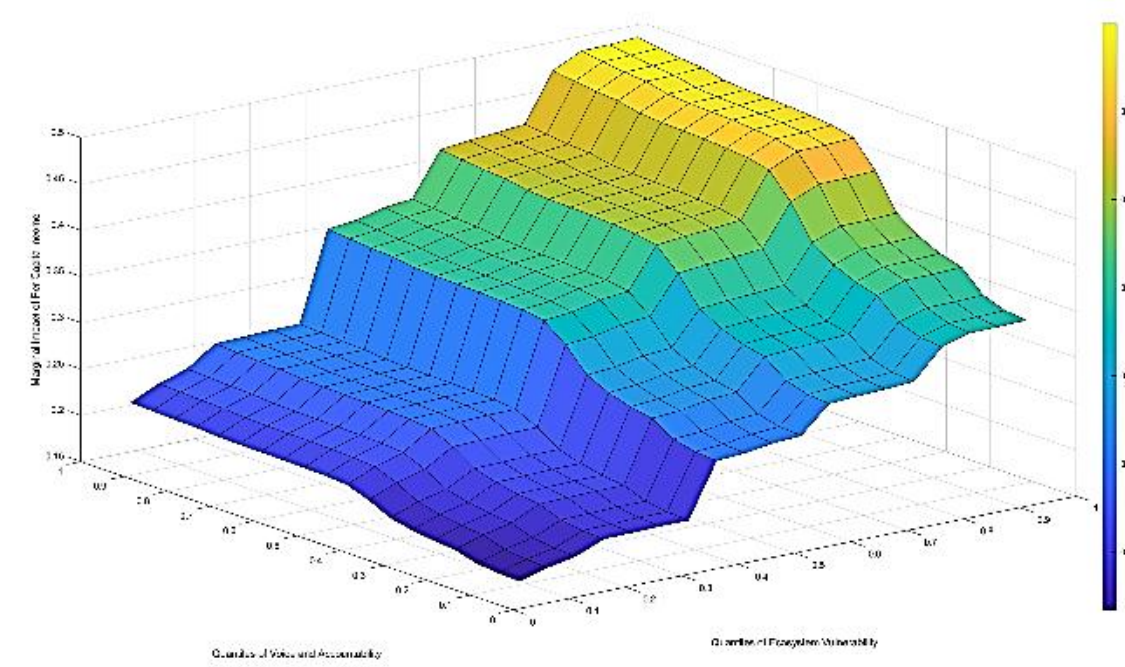

Governance Quality Indicator: Voice and Accountability

Figure S48: BRICS Policy Simulation Outcomes for the Marginal Impact of Per Capita Income on SDG 7.1.2 under Ecosystem Vulnerability and Governance Quality, Related to Figure 4-5 and Table 2

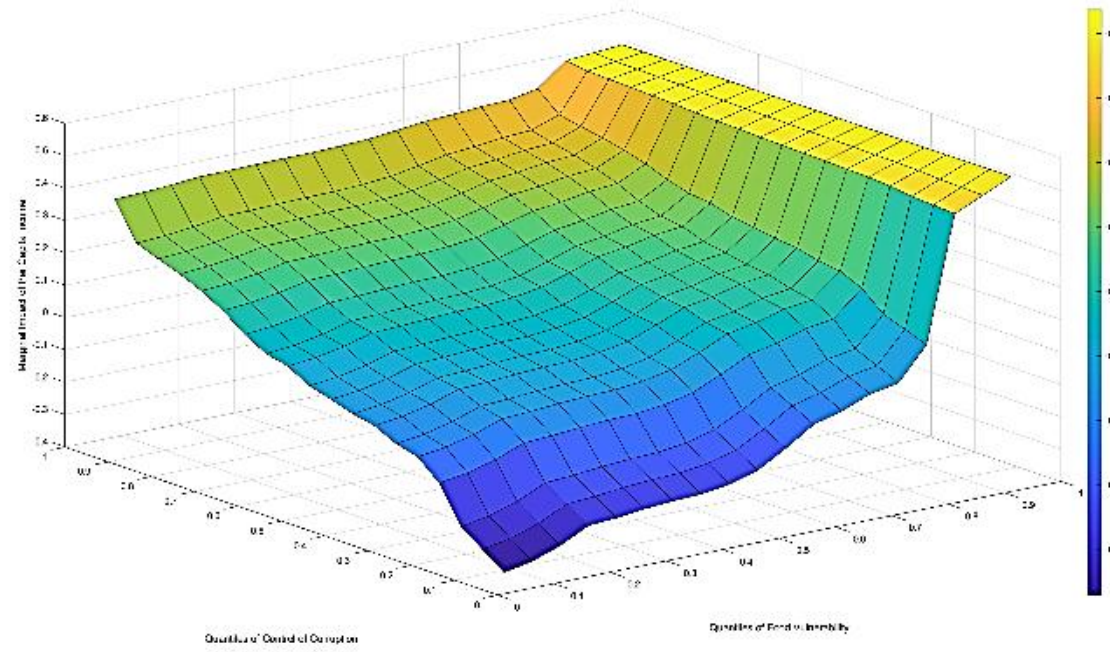

Governance Quality Indicator: Control of Corruption

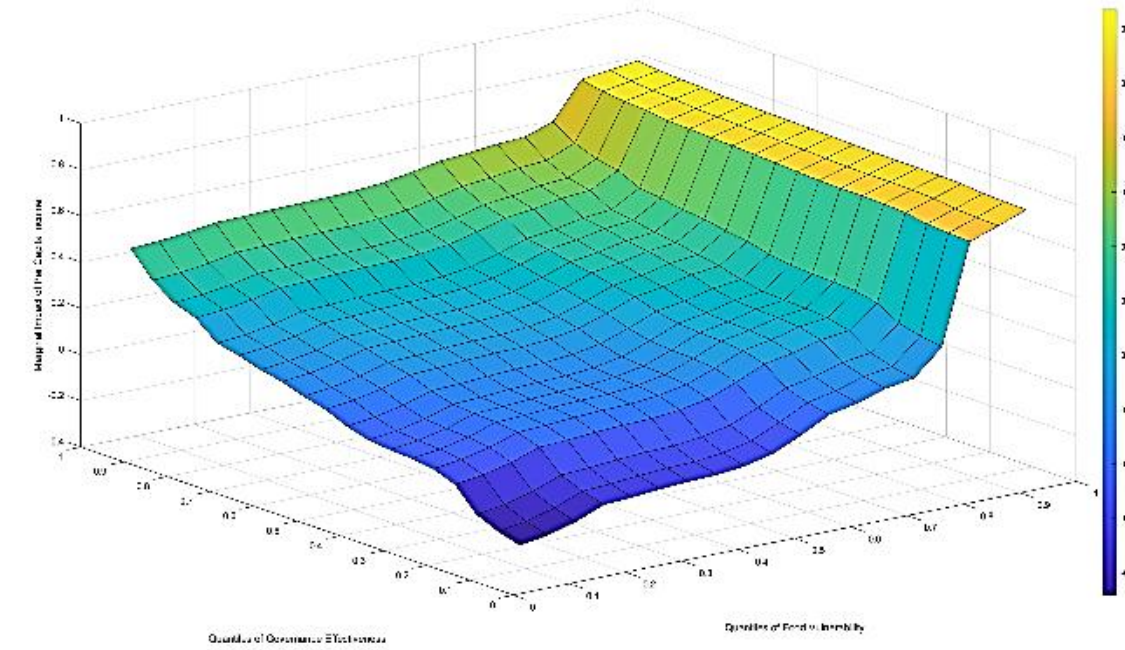

Governance Quality Indicator: Governance Effectiveness

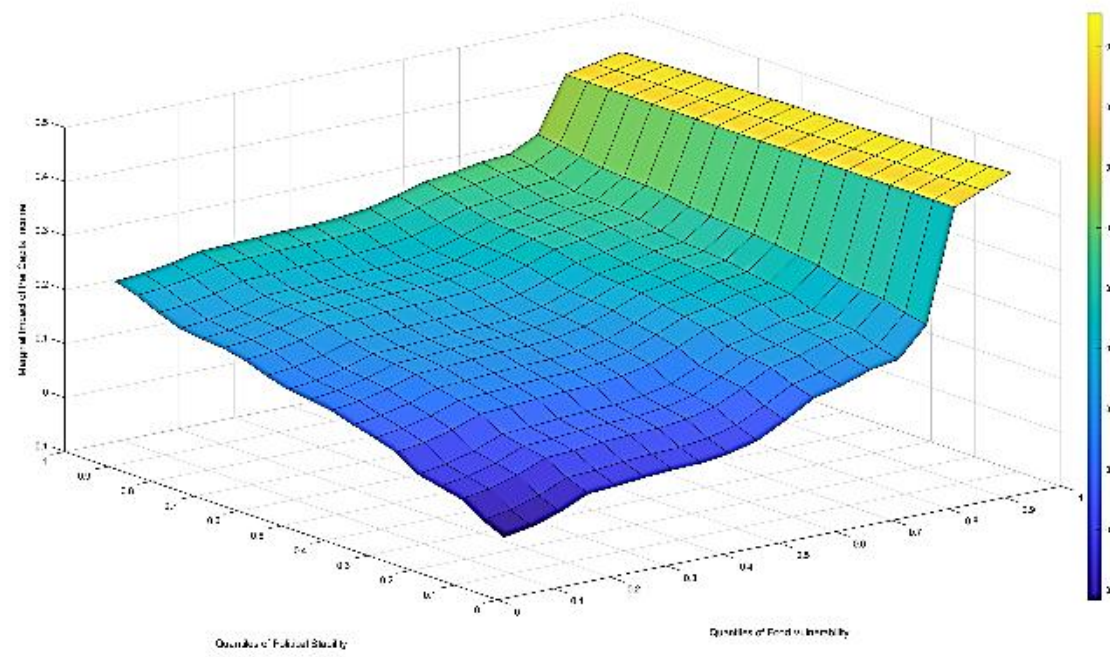

Governance Quality Indicator: Political Stability

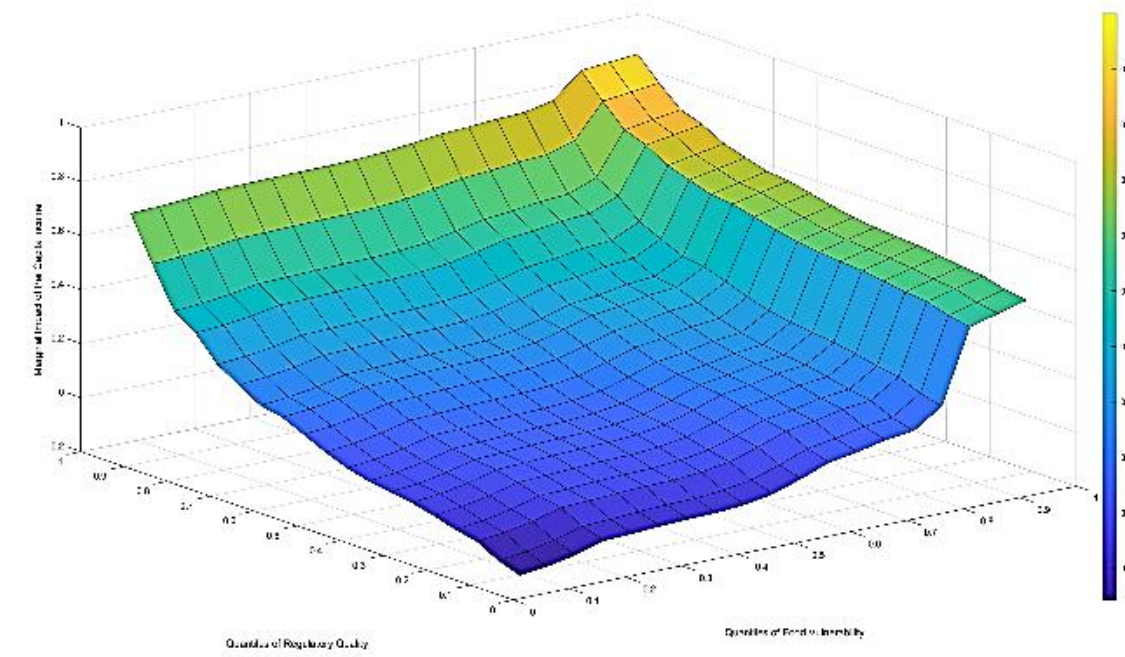

Governance Quality Indicator: Regulatory Quality

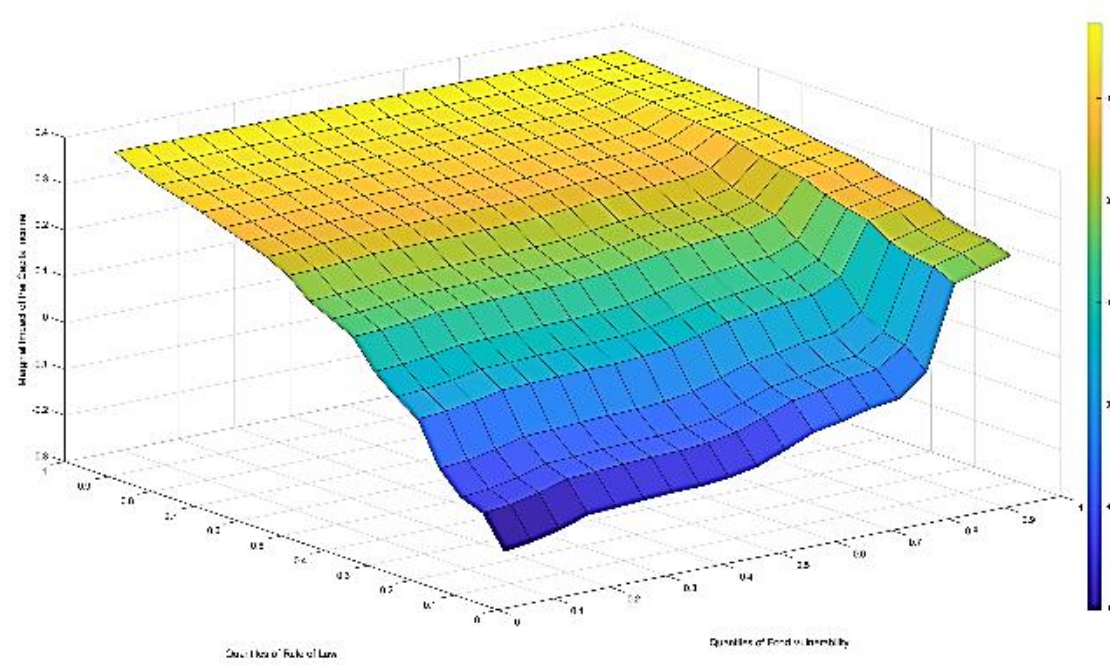

Governance Quality Indicator: Rule of Law

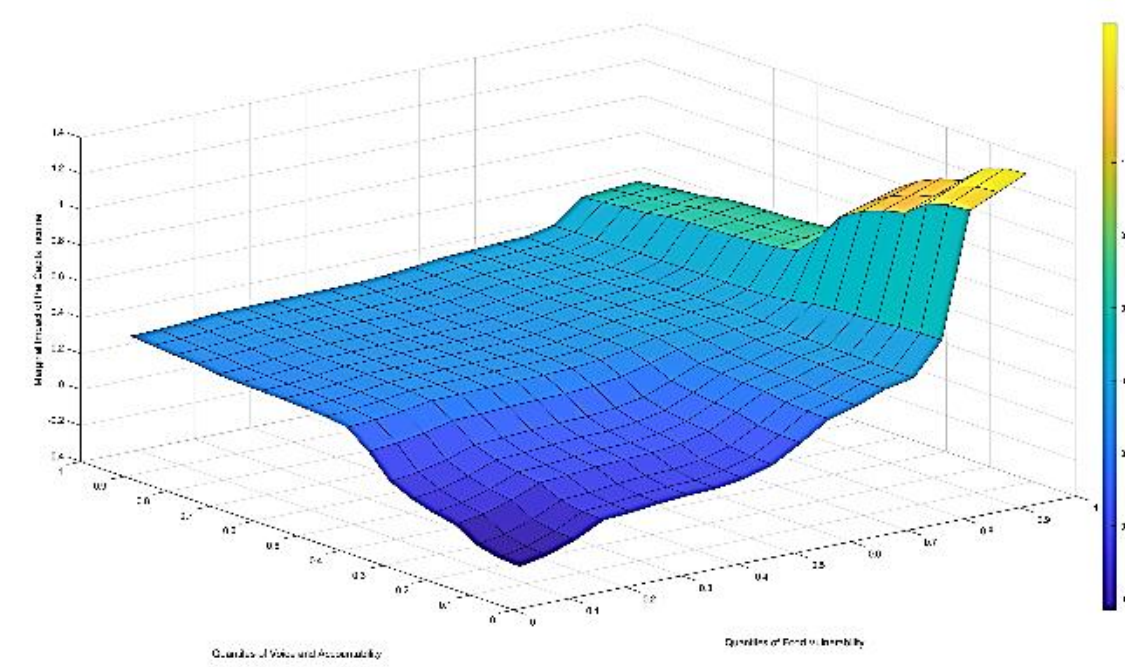

Governance Quality Indicator: Voice and Accountability

Figure S49: BRICS Policy Simulation Outcomes for the Marginal Impact of Per Capita Income on SDG 7.1.2 under Food Vulnerability and Governance Quality, Related to Figure 4-5 and Table 2

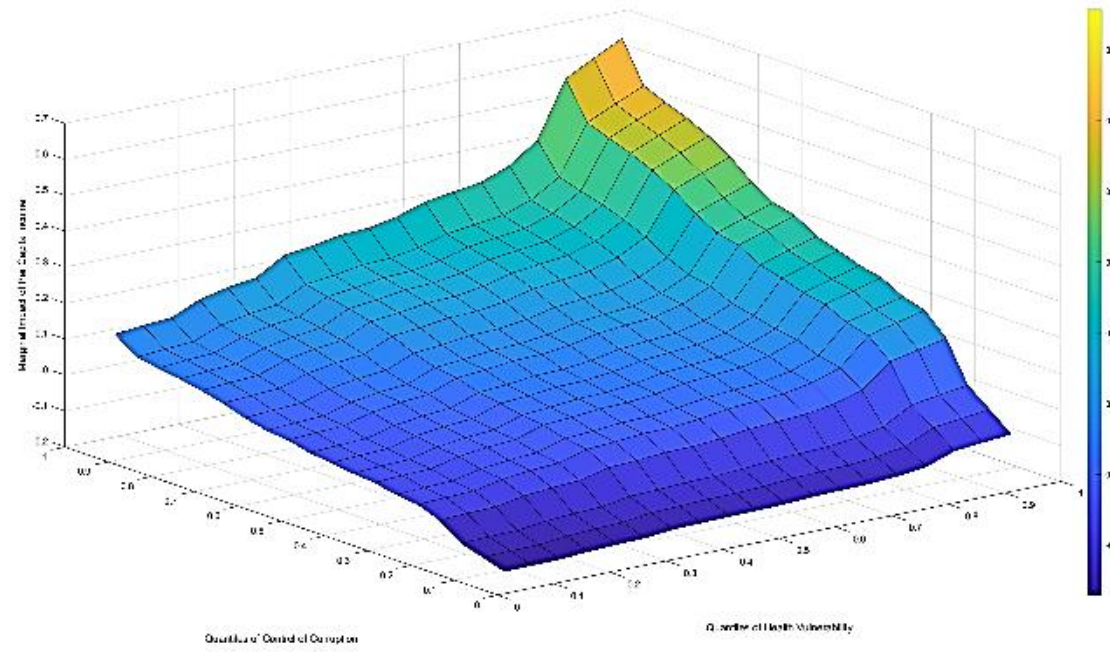

Governance Quality Indicator: Control of Corruption

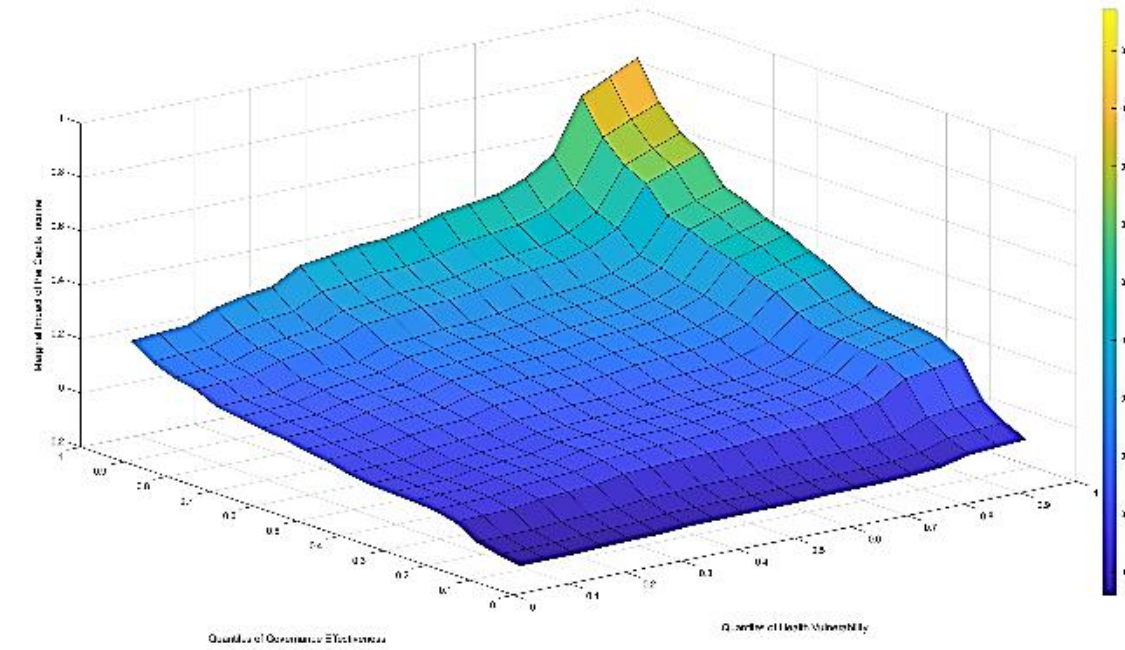

Governance Quality Indicator: Governance Effectiveness

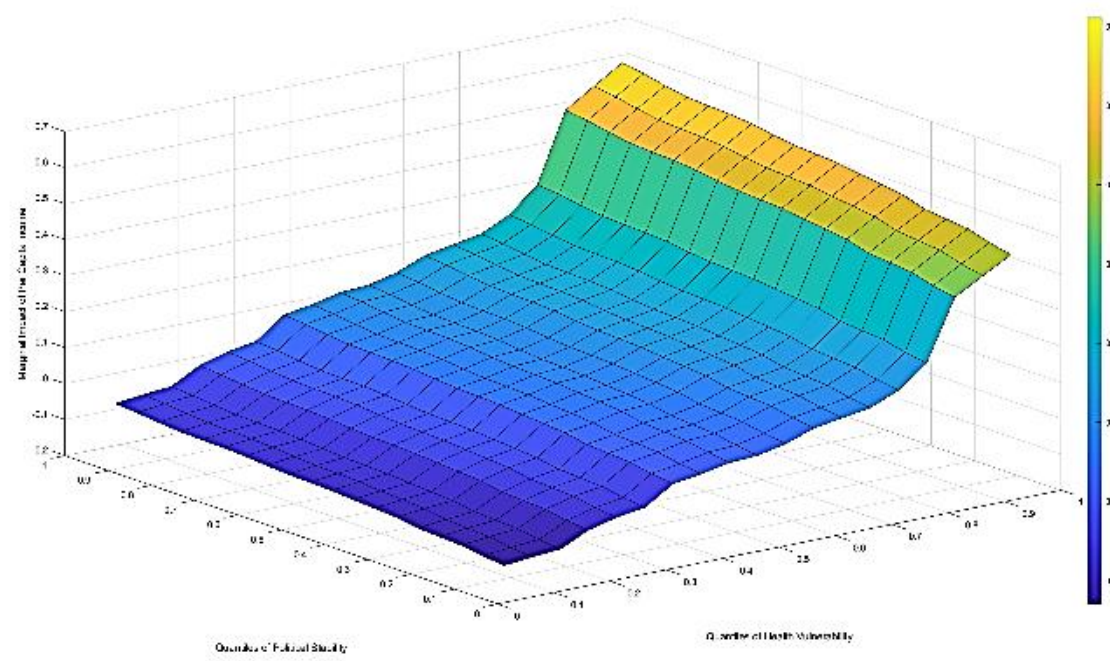

Governance Quality Indicator: Political Stability

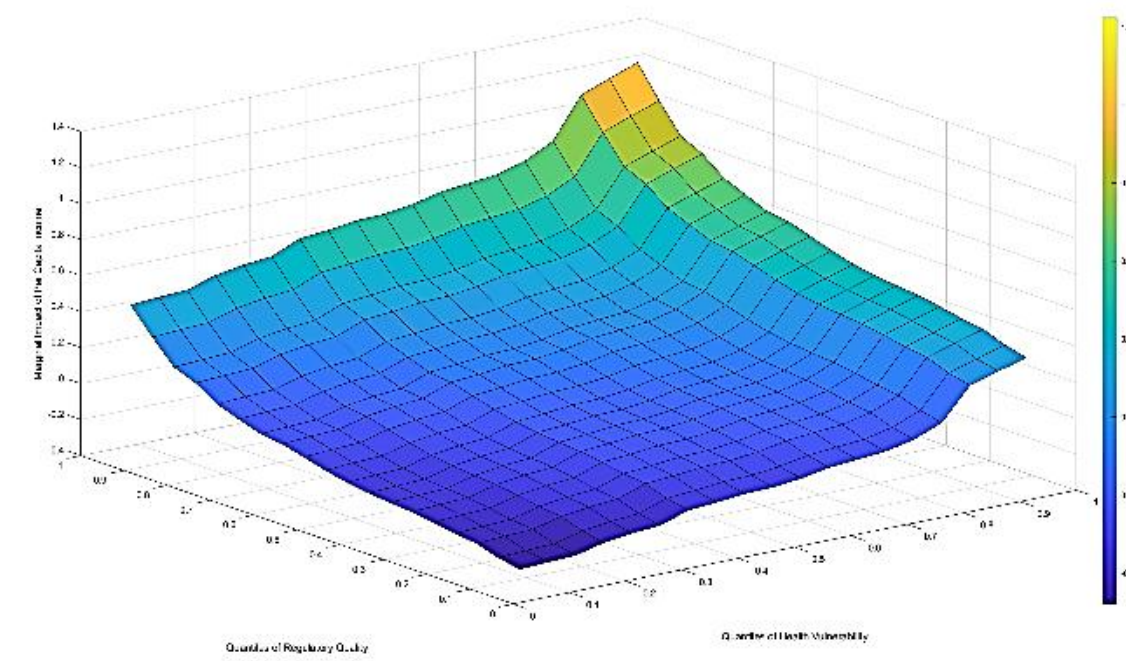

Governance Quality Indicator: Regulatory Quality

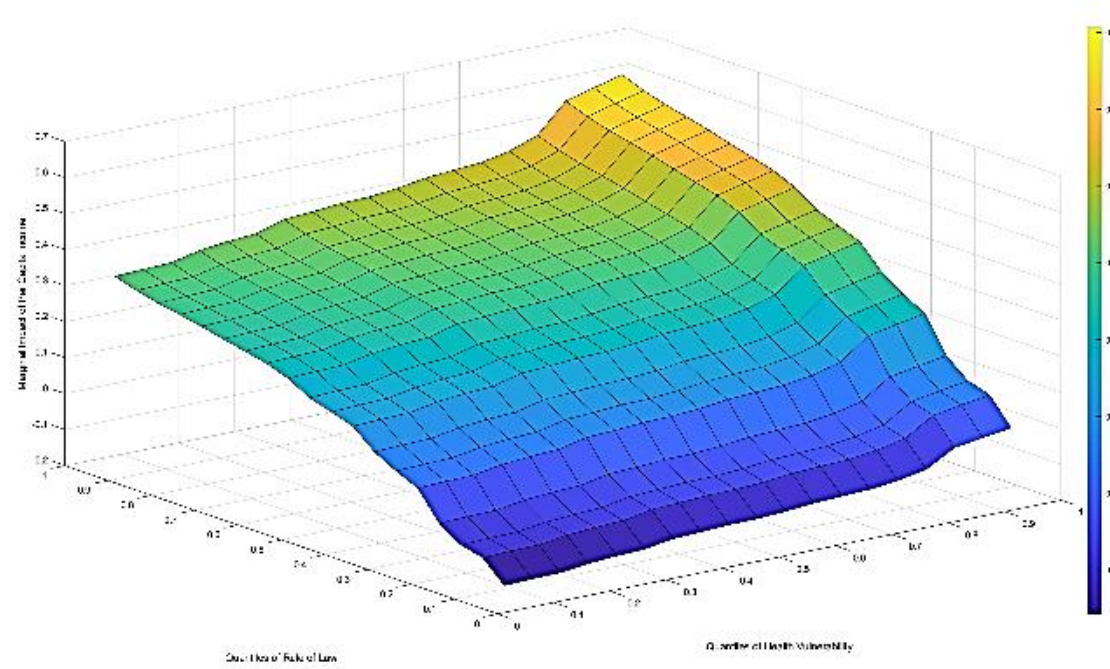

Governance Quality Indicator: Rule of Law

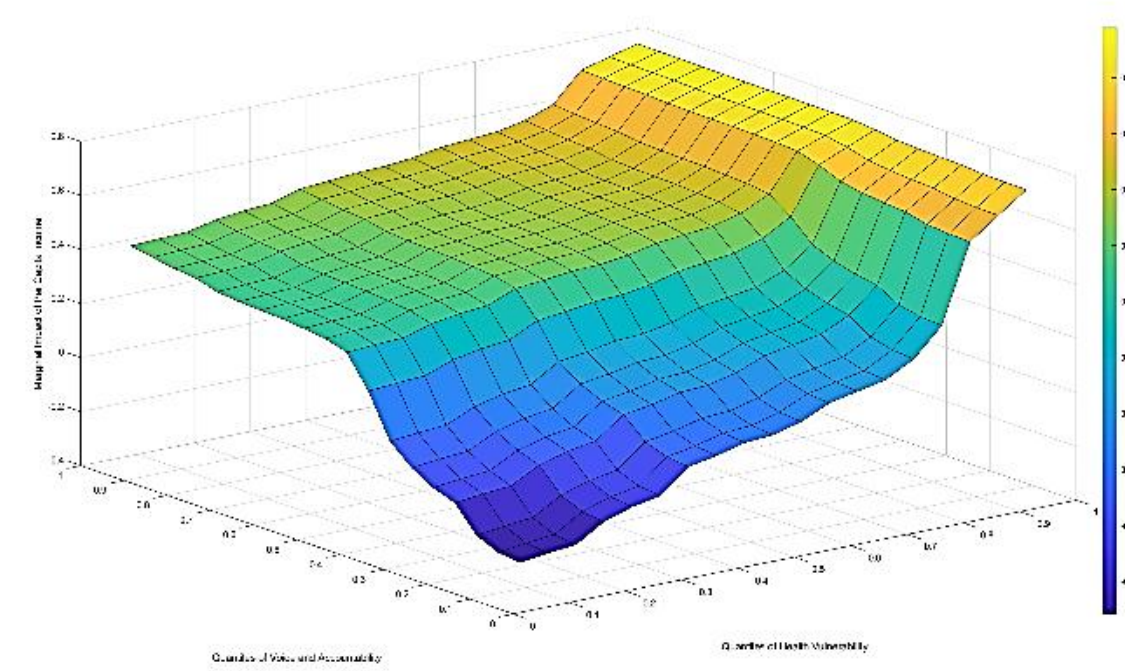

Governance Quality Indicator: Voice and Accountability

Figure S50: BRICS Policy Simulation Outcomes for the Marginal Impact of Per Capita Income on SDG 7.1.2 under Health Vulnerability and Governance Quality, Related to Figure 4-5 and Table 2

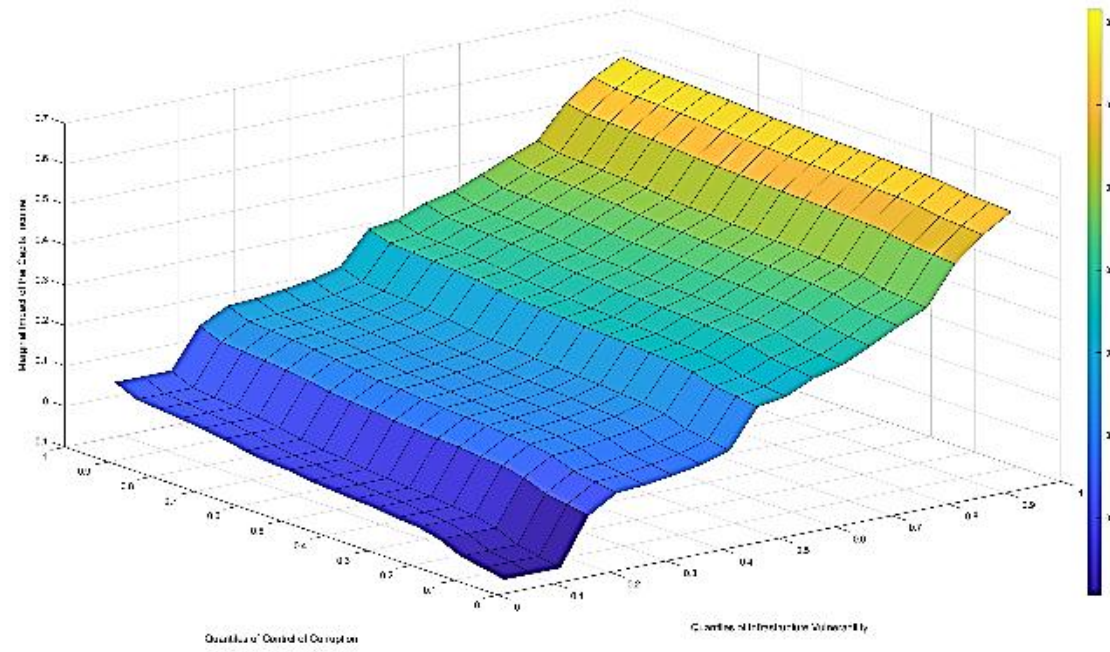

Governance Quality Indicator: Control of Corruption

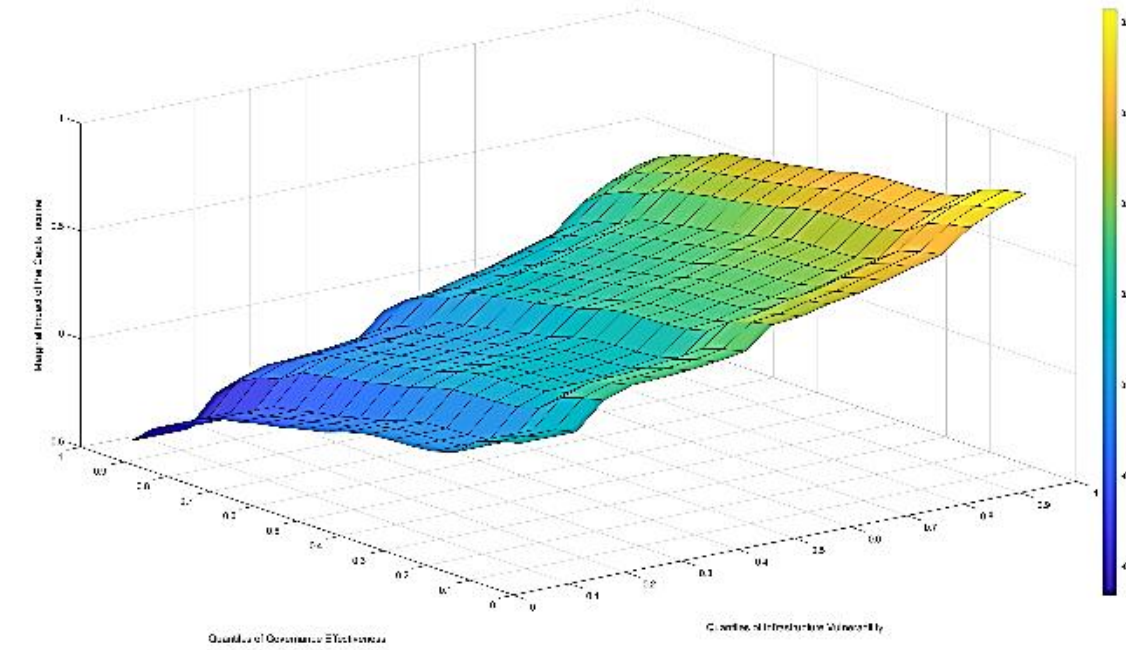

Governance Quality Indicator: Governance Effectiveness

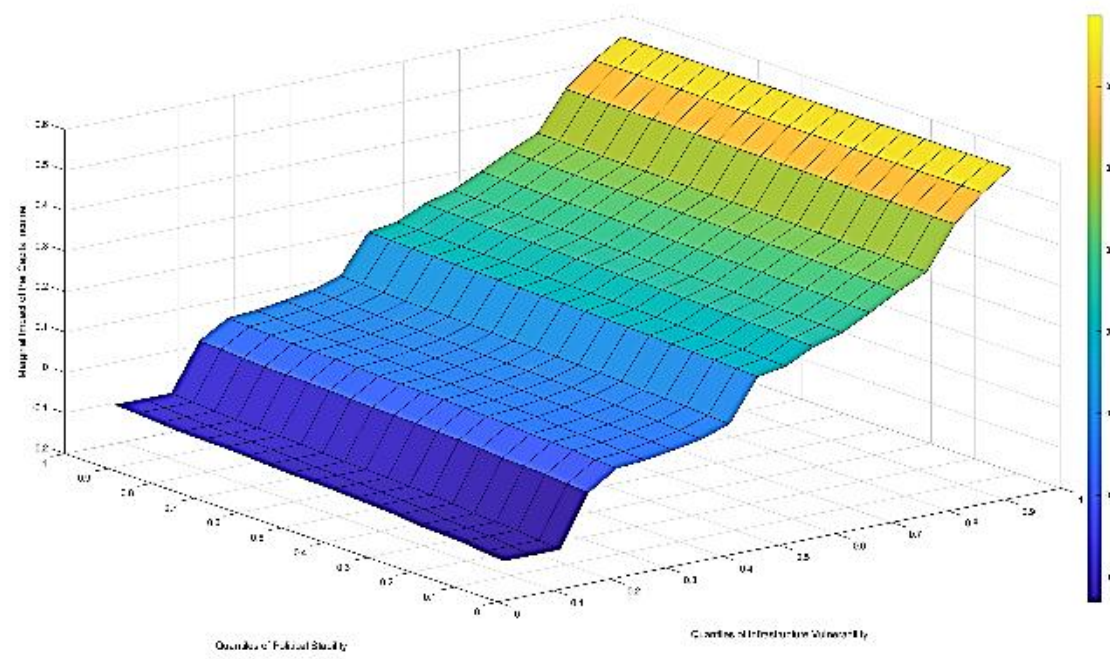

Governance Quality Indicator: Political Stability

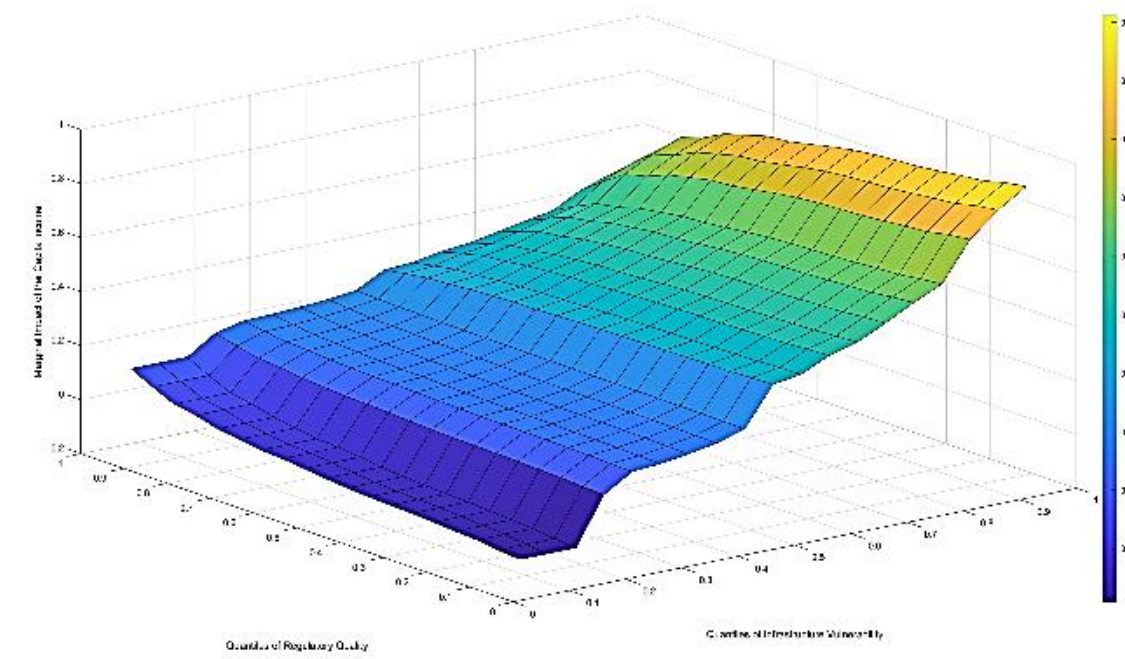

Governance Quality Indicator: Regulatory Quality

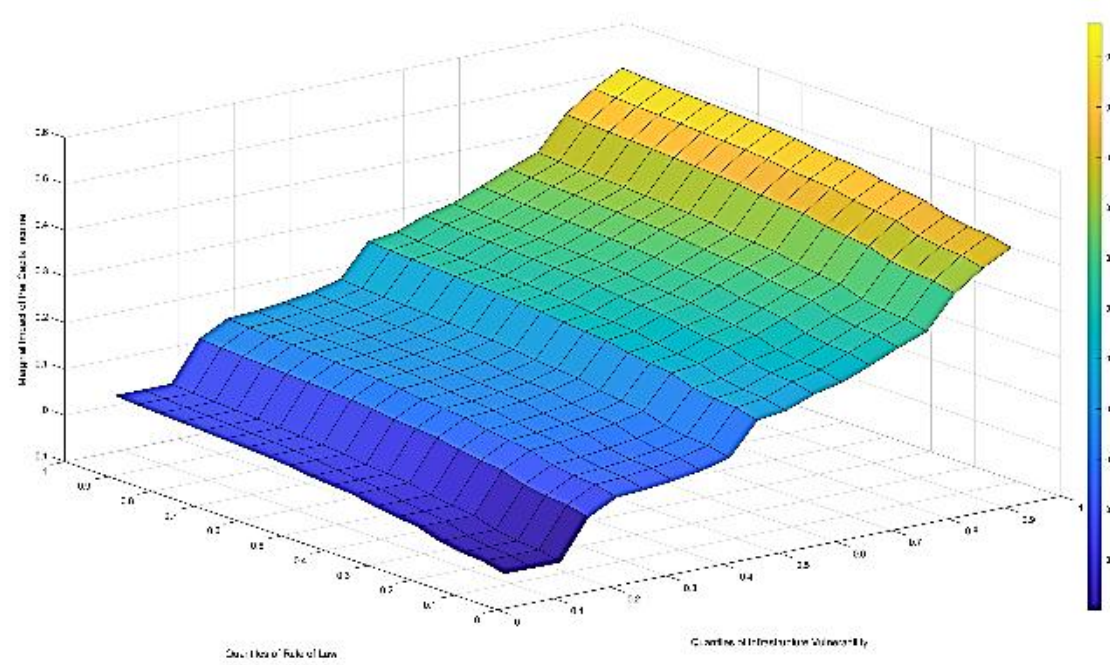

Governance Quality Indicator: Rule of Law

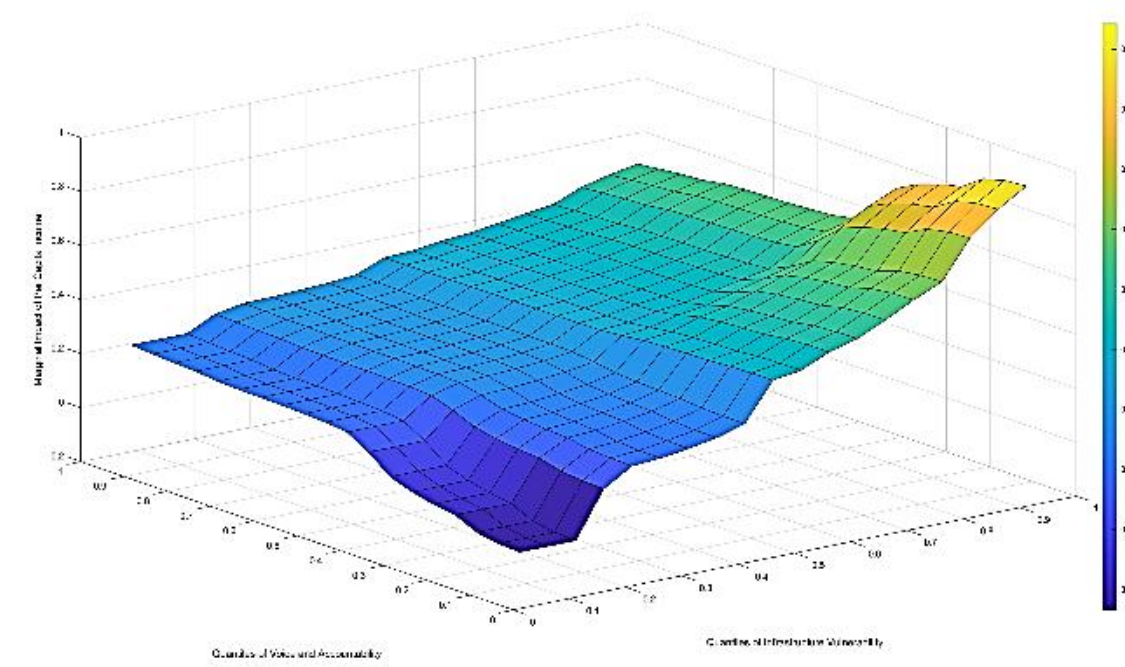

Governance Quality Indicator: Voice and Accountability

Figure S51: BRICS Policy Simulation Outcomes for the Marginal Impact of Per Capita Income on SDG 7.1.2 under Infrastructure Vulnerability and Governance Quality, Related to Figure 4-5 and Table 2

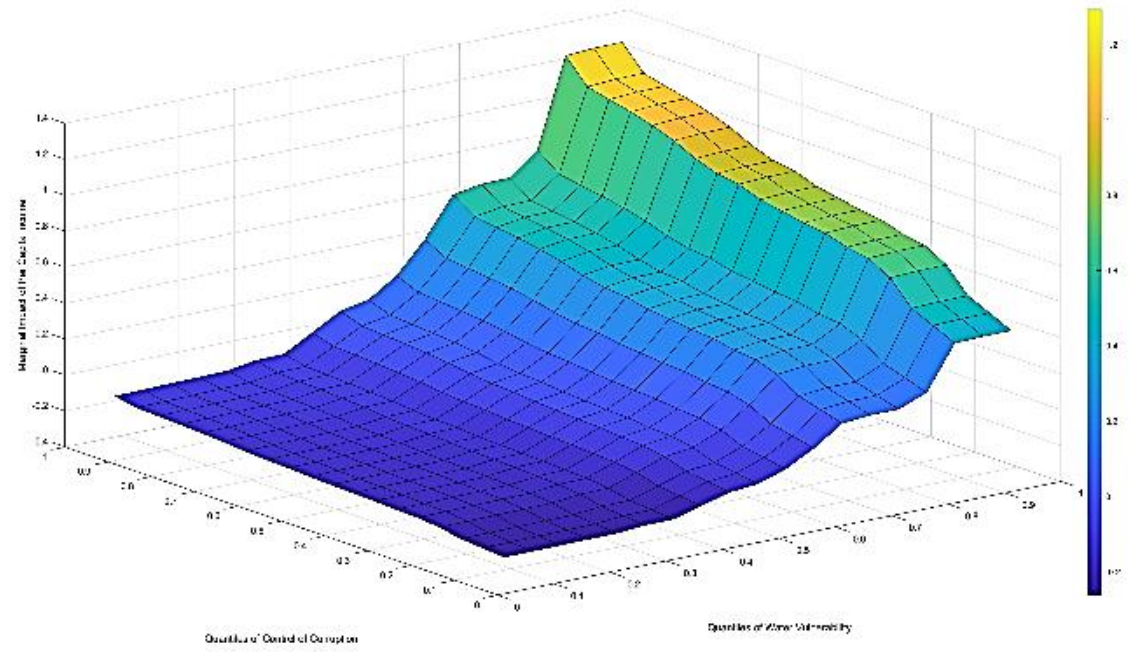

Governance Quality Indicator: Control of Corruption

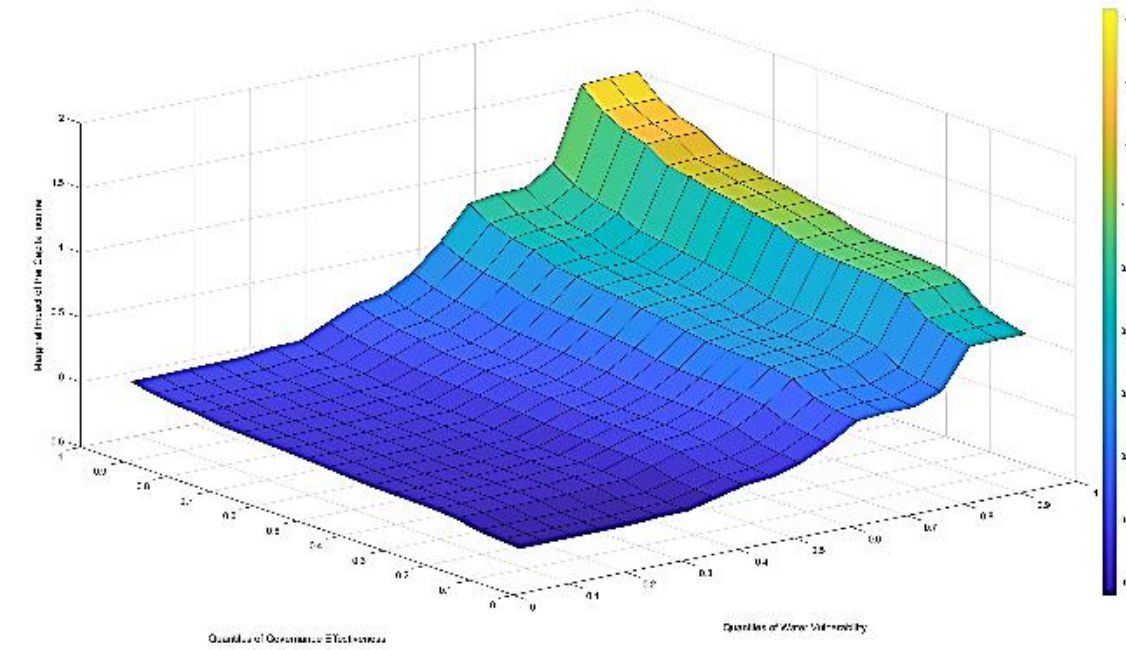

Governance Quality Indicator: Governance Effectiveness

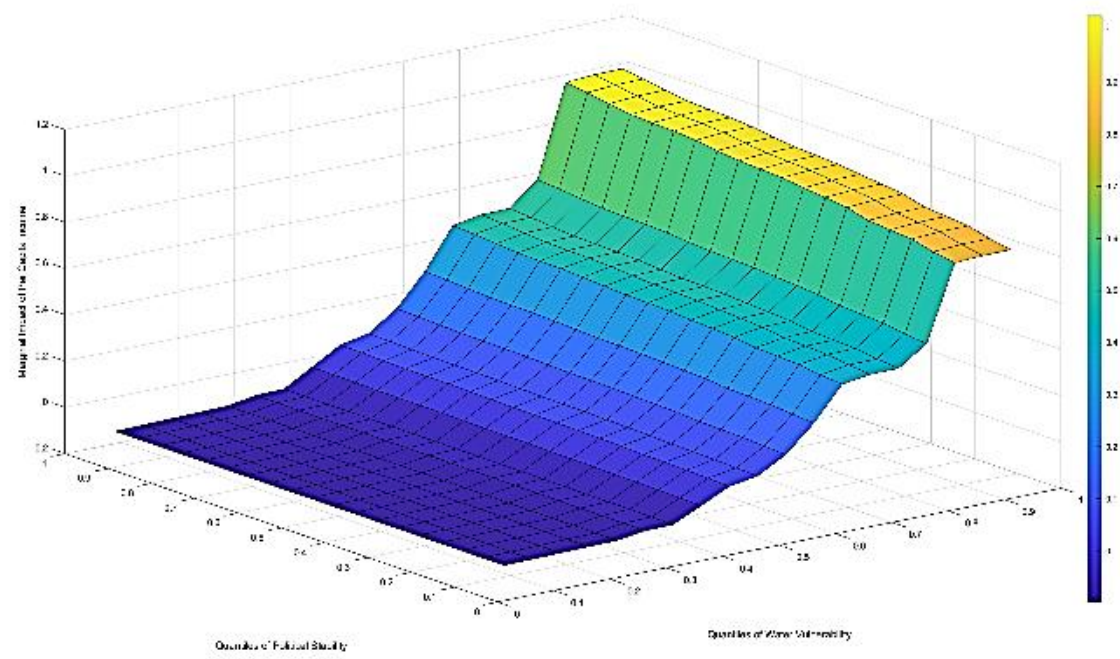

Governance Quality Indicator: Political Stability

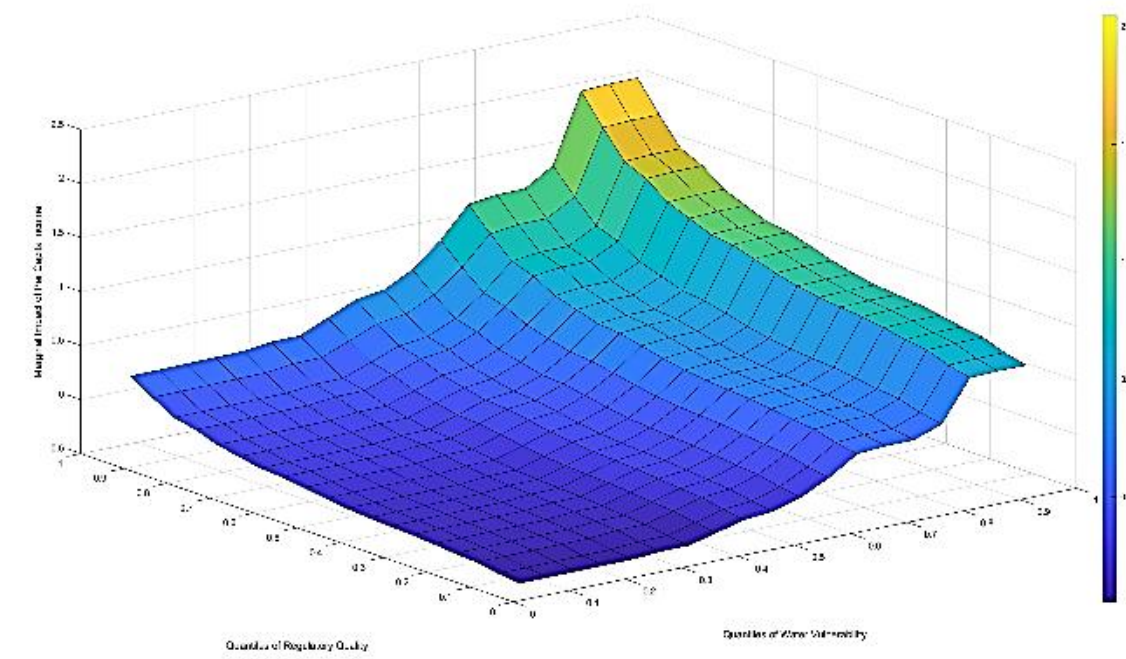

Governance Quality Indicator: Regulatory Quality

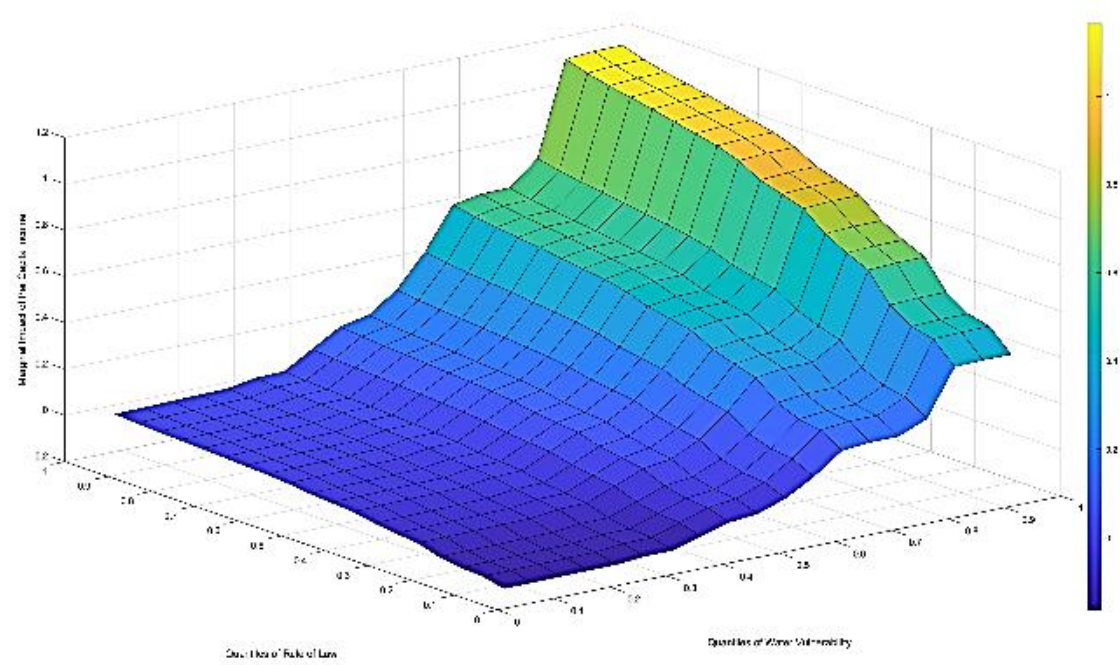

Governance Quality Indicator: Rule of Law

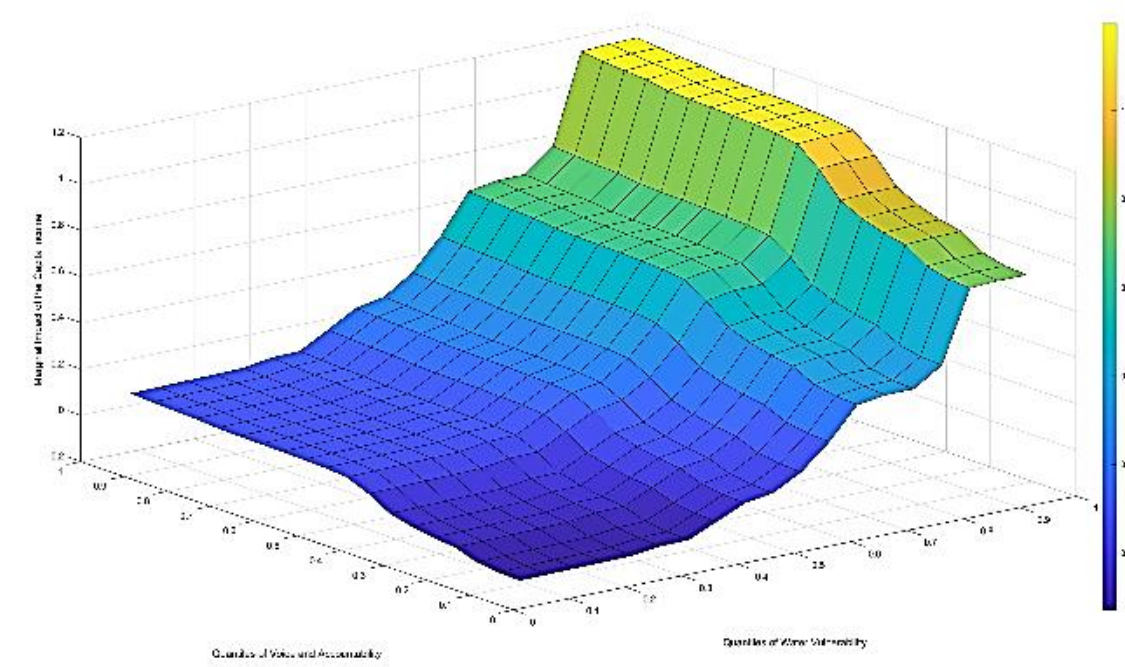

Governance Quality Indicator: Voice and Accountability

Figure S52: BRICS Policy Simulation Outcomes for the Marginal Impact of Per Capita Income on SDG 7.1.2 under Water Vulnerability and Governance Quality, Related to Figure 4-5 and Table 2

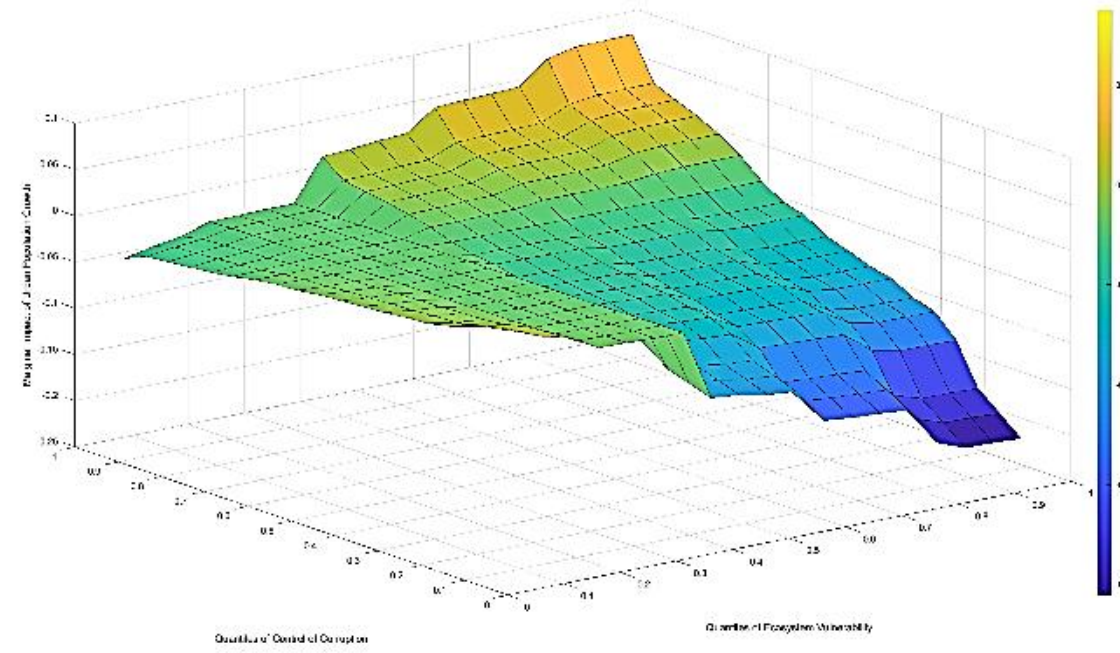

Governance Quality Indicator: Control of Corruption

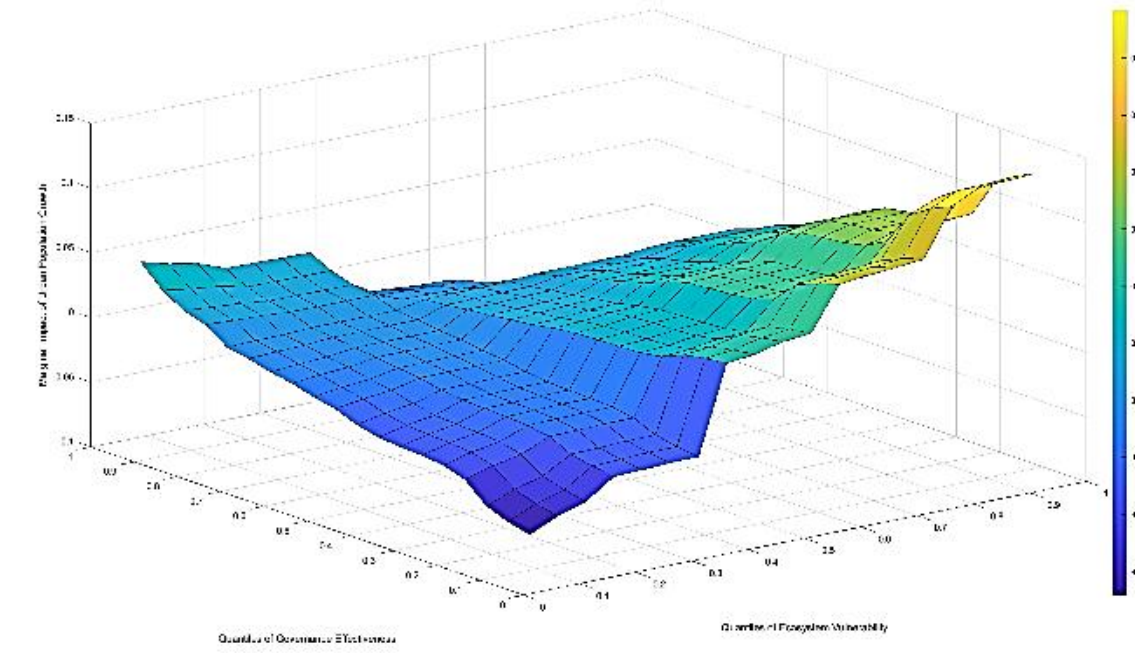

Governance Quality Indicator: Governance Effectiveness

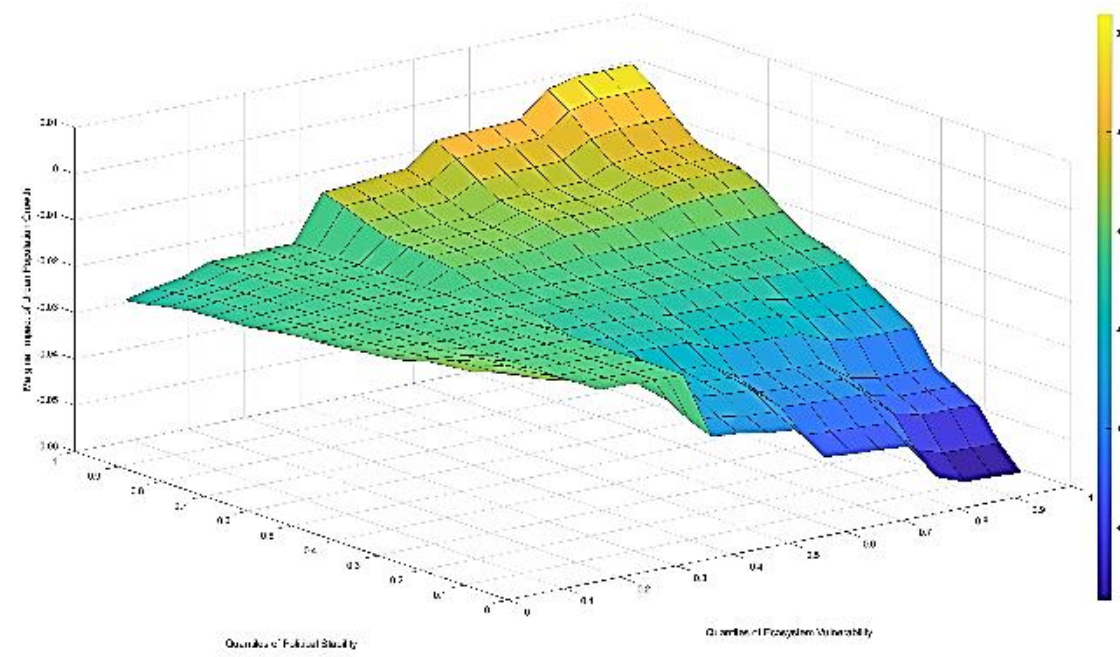

Governance Quality Indicator: Political Stability

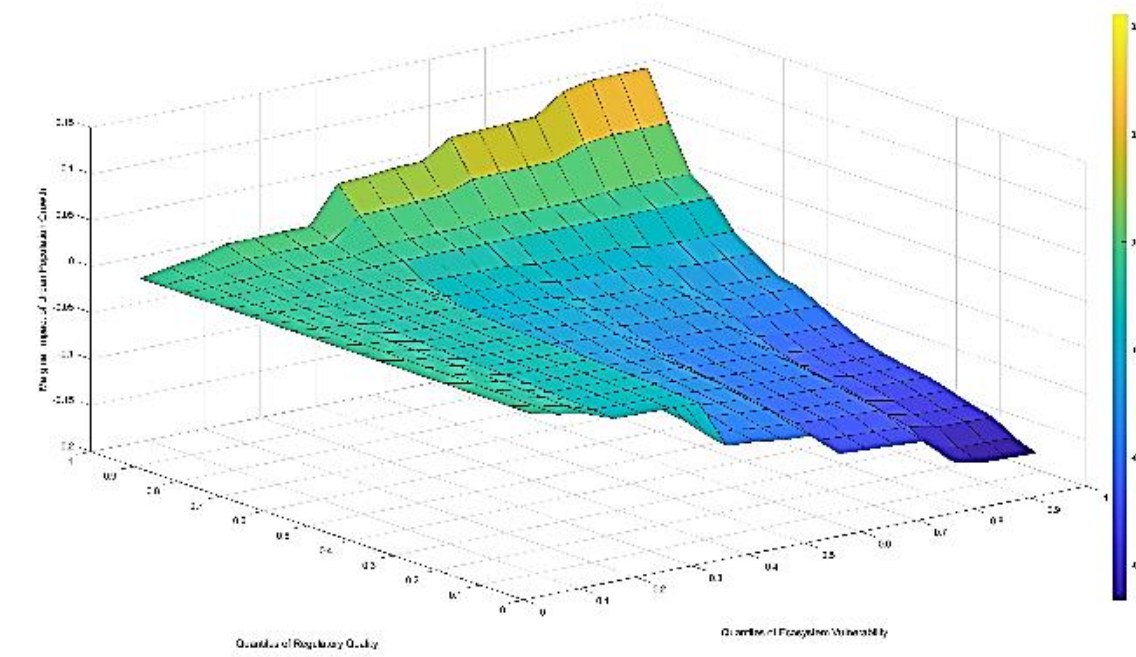

Governance Quality Indicator: Regulatory Quality

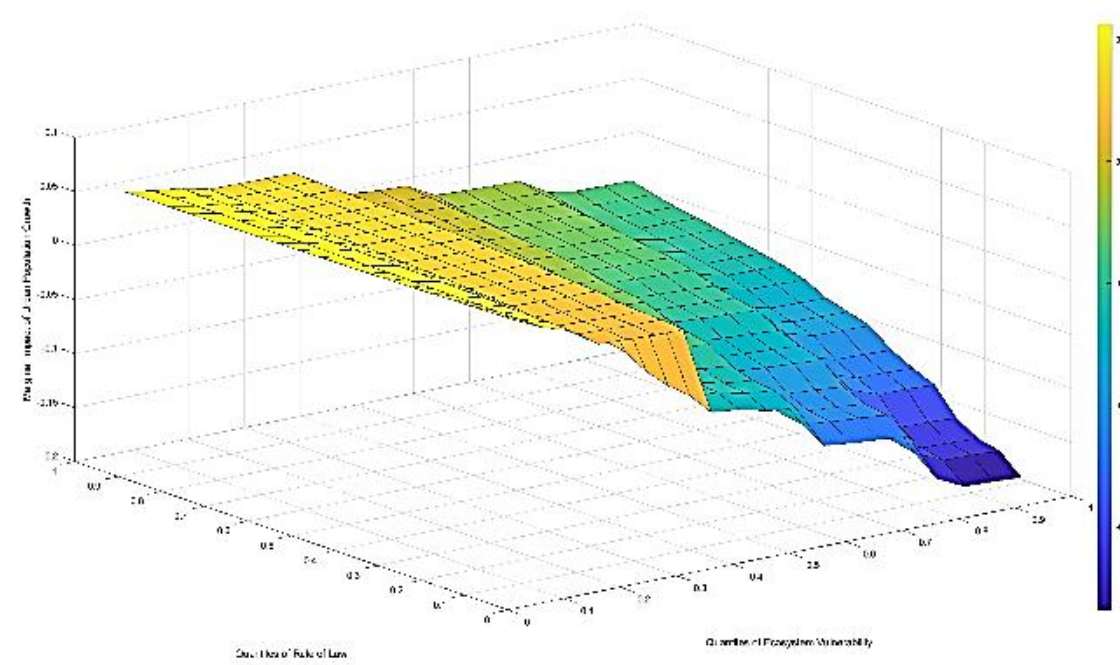

Governance Quality Indicator: Rule of Law

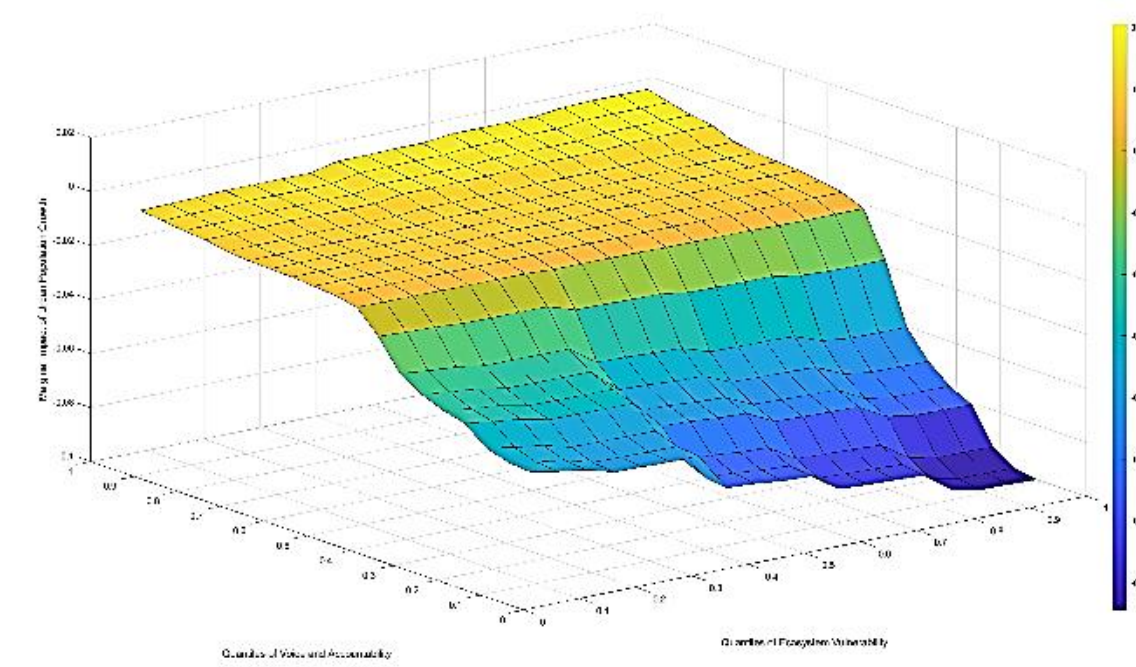

Governance Quality Indicator: Voice and Accountability

Figure S53: BRICS Policy Simulation Outcomes for the Marginal Impact of Urban Population Growth on SDG 7.1.2 under Ecosystem Vulnerability and Governance Quality, Related to Figure 4-5 and Table 2

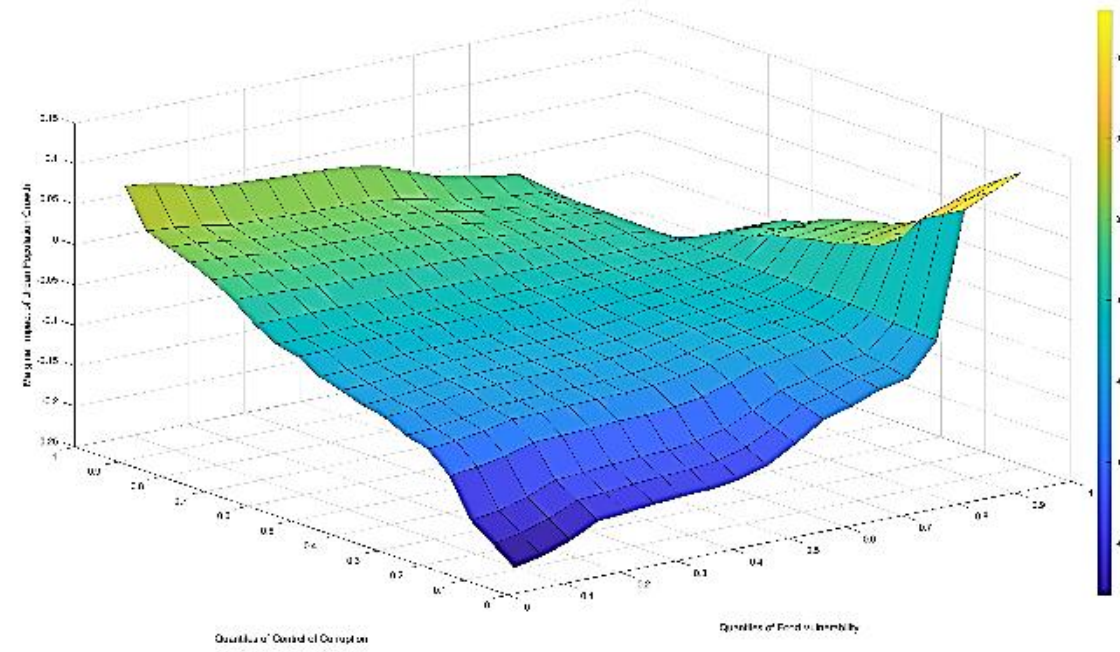

Governance Quality Indicator: Control of Corruption

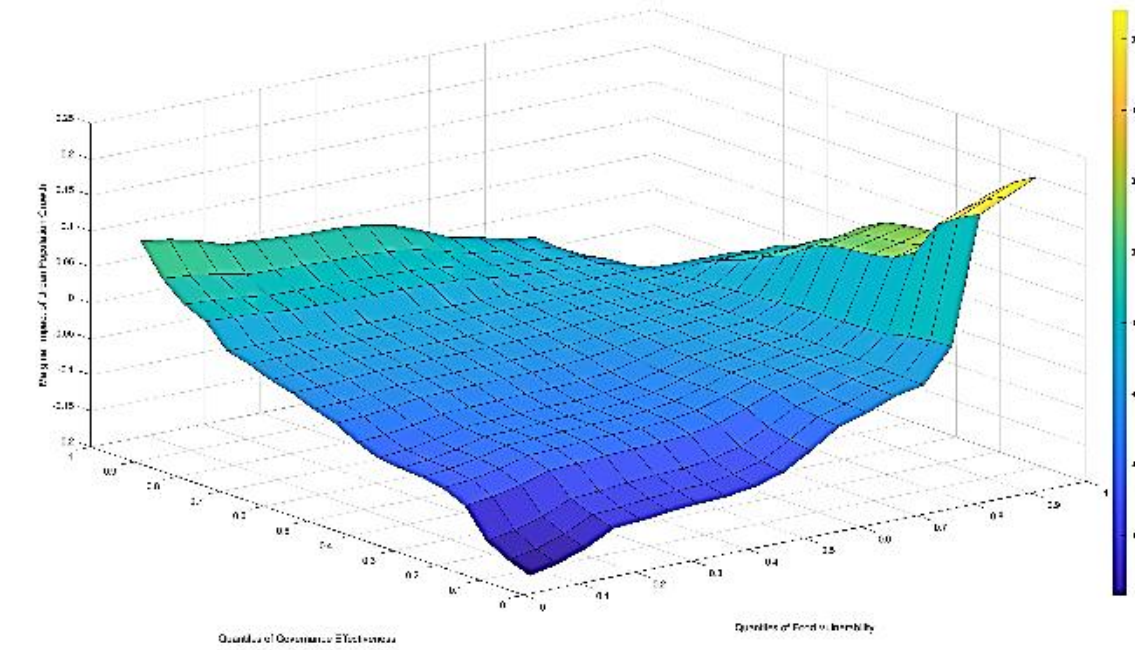

Governance Quality Indicator: Governance Effectiveness

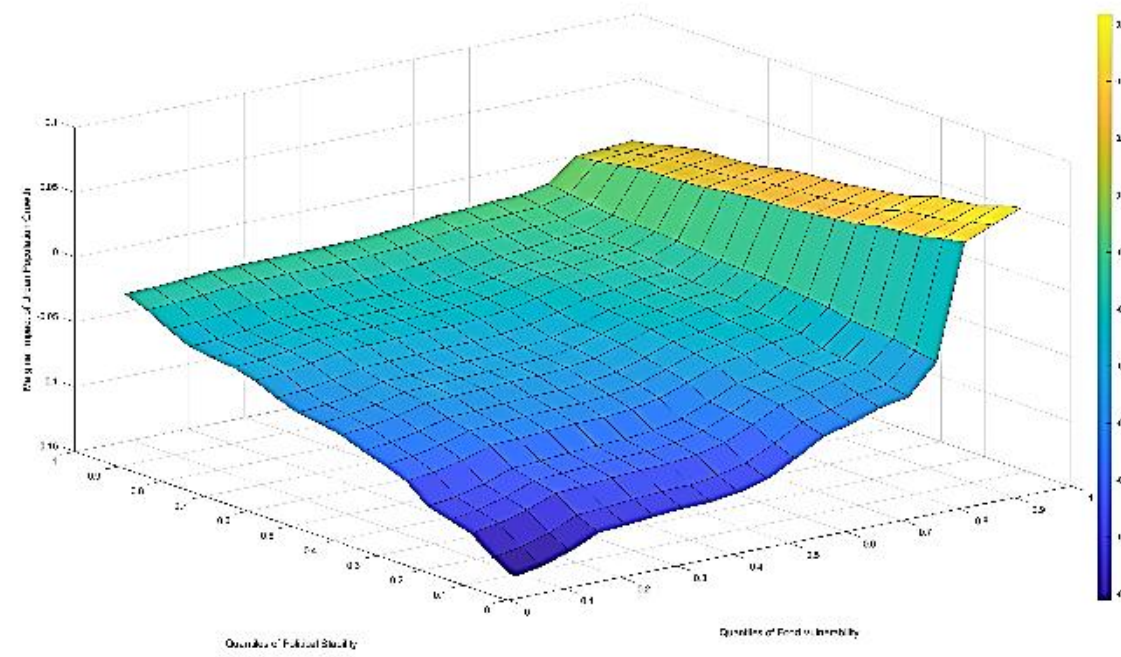

Governance Quality Indicator: Political Stability

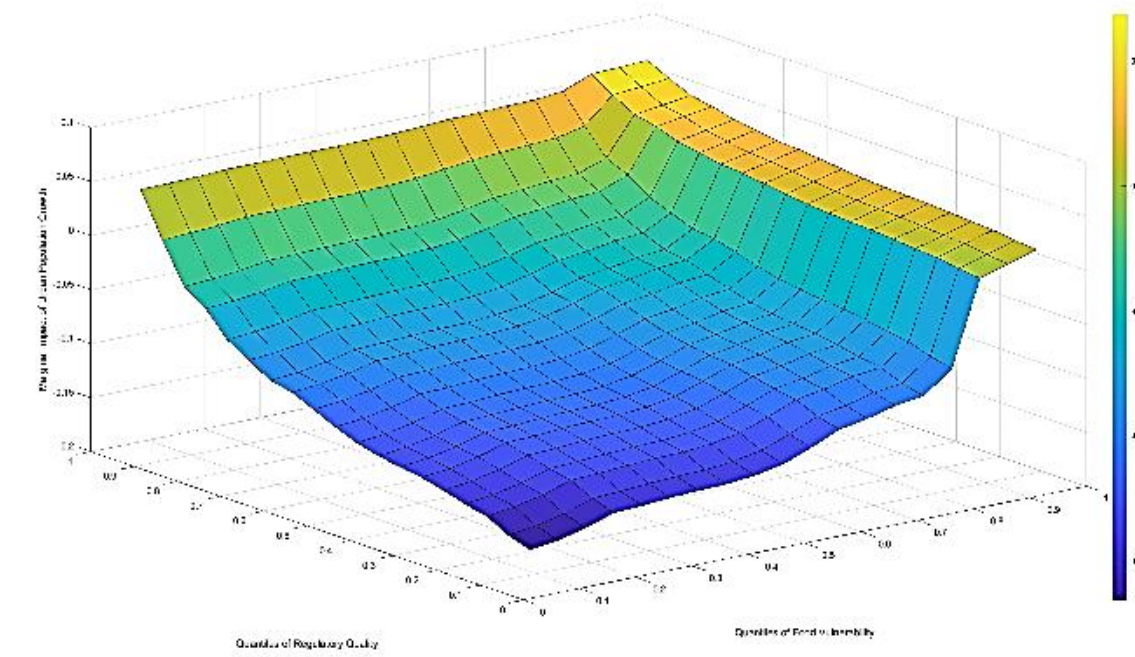

Governance Quality Indicator: Regulatory Quality

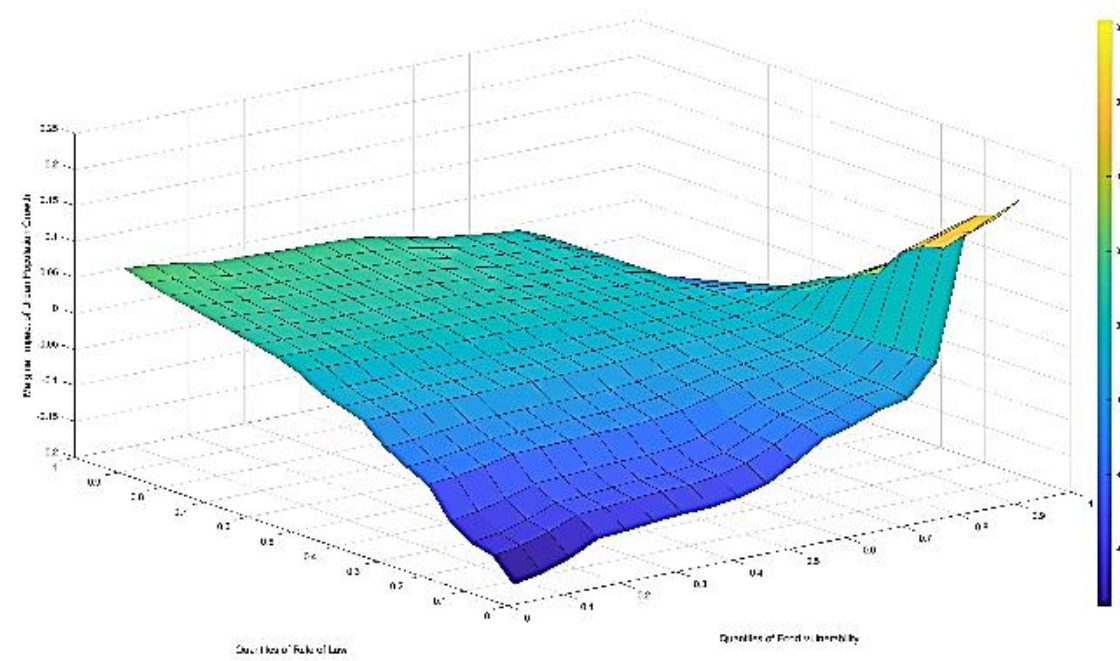

Governance Quality Indicator: Rule of Law

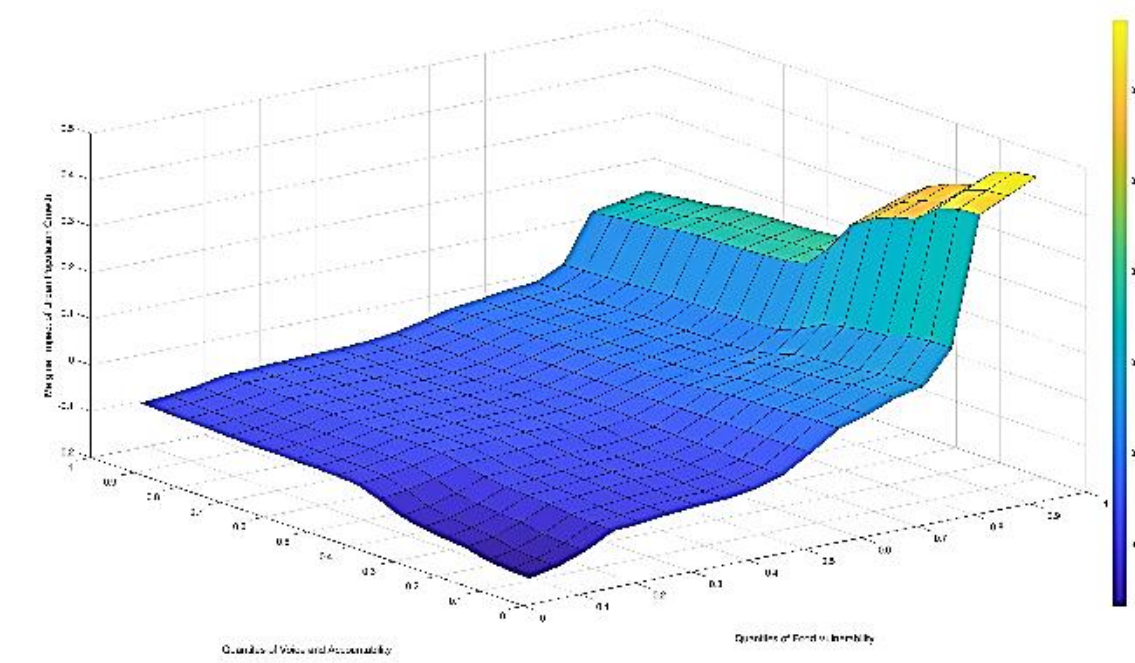

Governance Quality Indicator: Voice and Accountability

Figure S54: BRICS Policy Simulation Outcomes for the Marginal Impact of Urban Population Growth on SDG 7.1.2 under Food Vulnerability and Governance Quality, Related to Figure 4-5 and Table 2

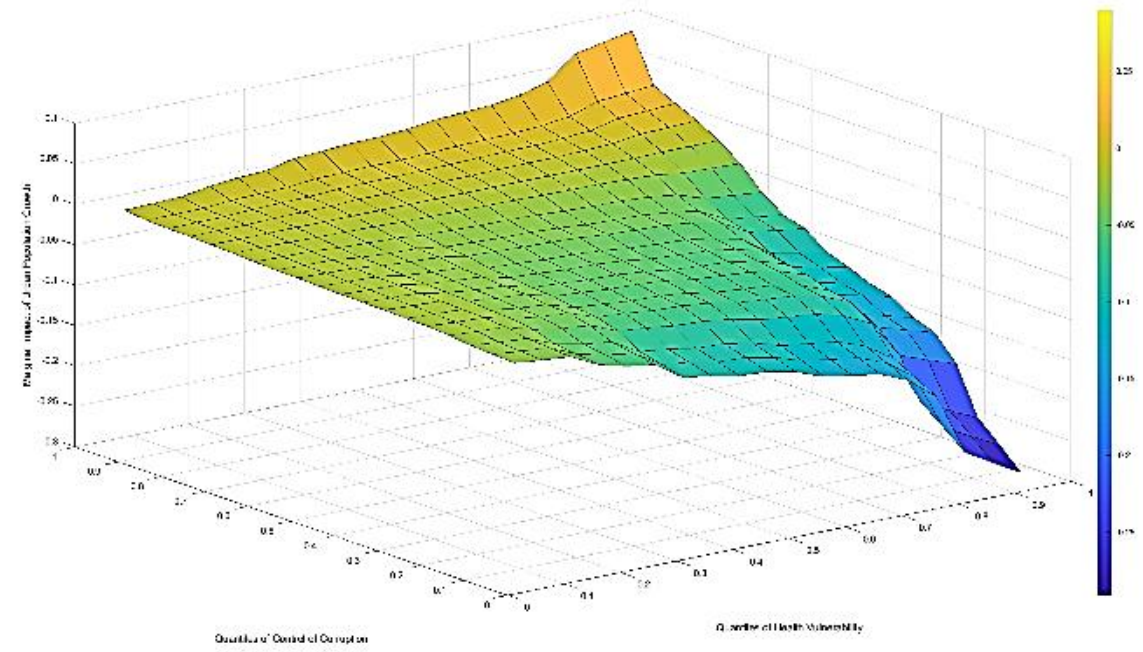

Governance Quality Indicator: Control of Corruption

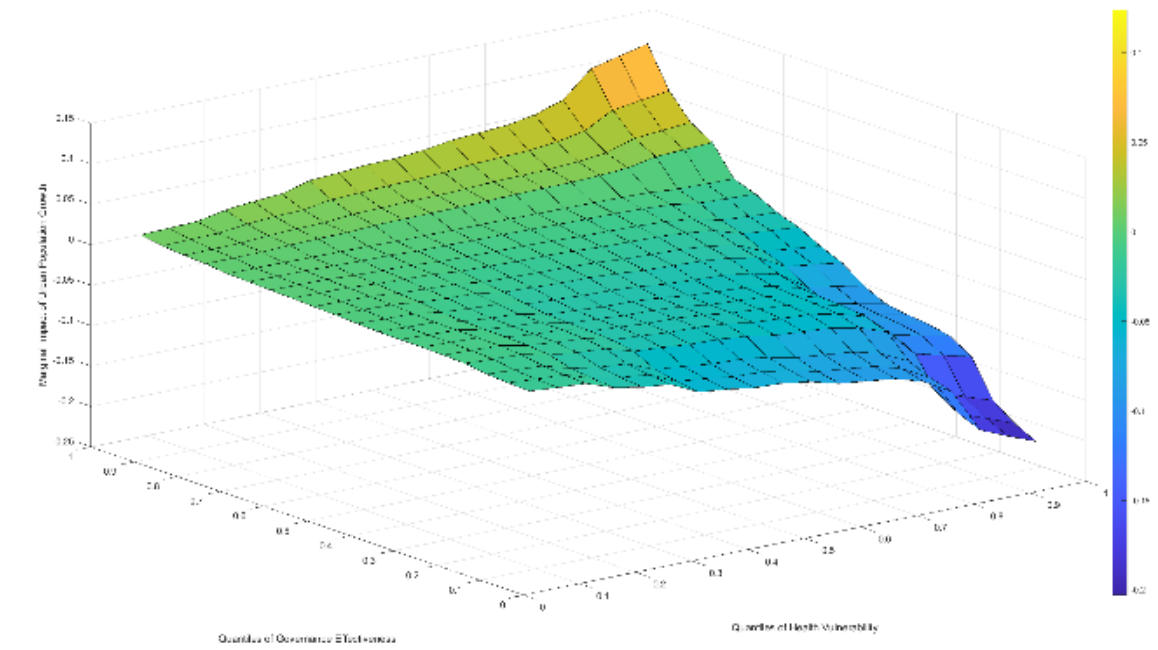

Governance Quality Indicator: Governance Effectiveness

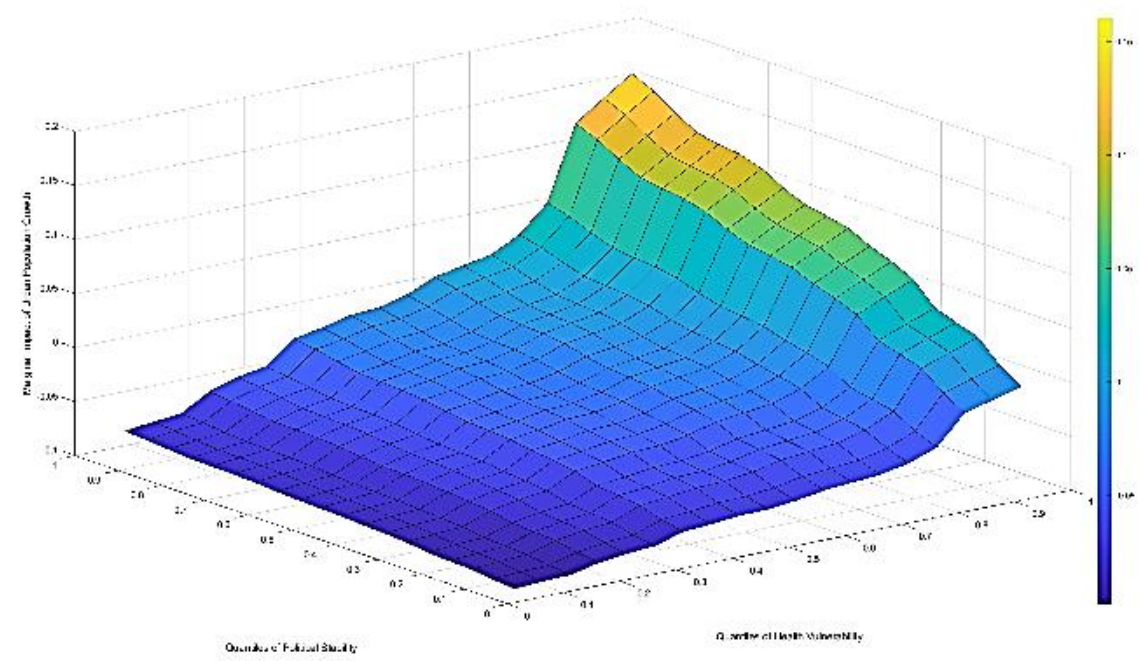

Governance Quality Indicator: Political Stability

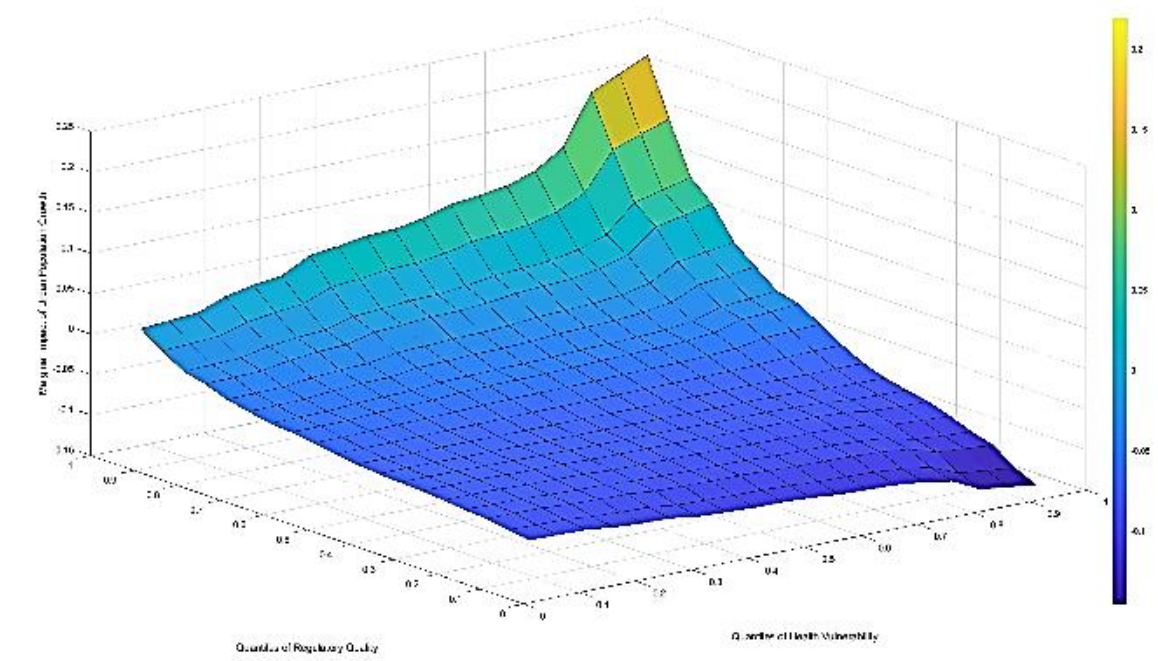

Governance Quality Indicator: Regulatory Quality

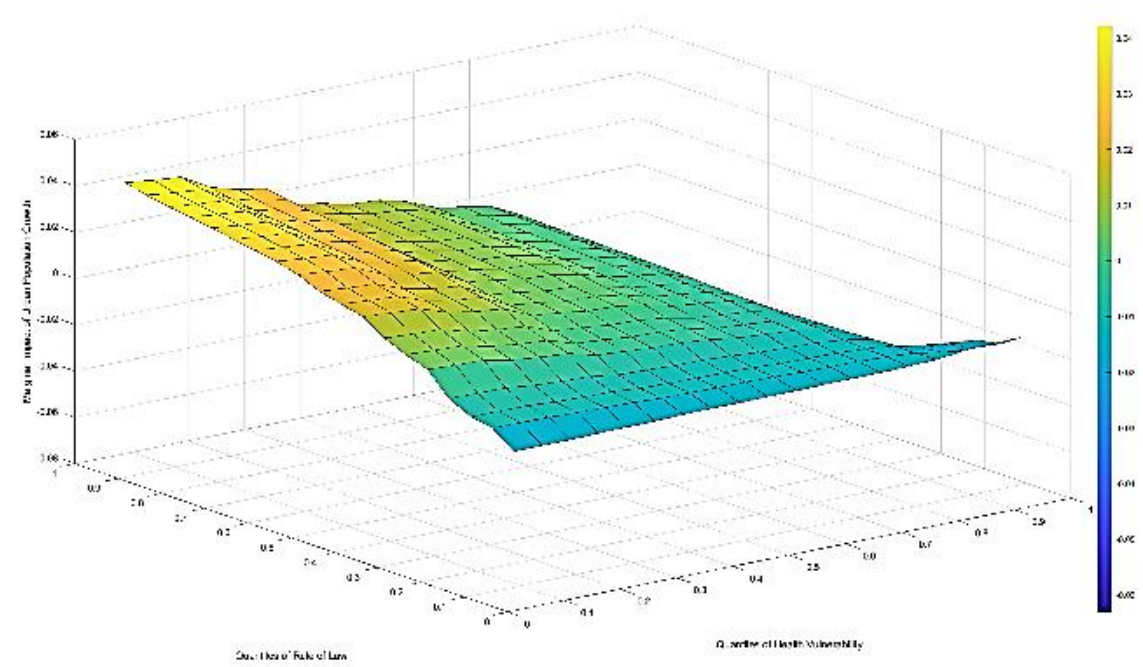

Governance Quality Indicator: Rule of Law

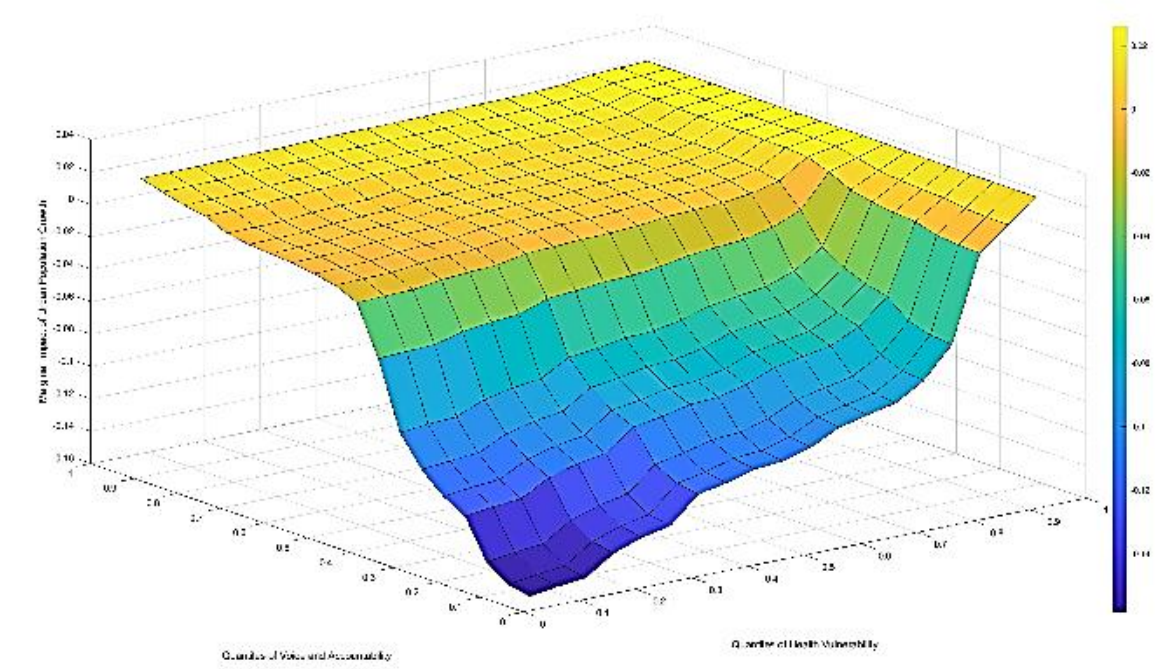

Governance Quality Indicator: Voice and Accountability

Figure S55: BRICS Policy Simulation Outcomes for the Marginal Impact of Urban Population Growth on SDG 7.1.2 under Health Vulnerability and Governance Quality, Related to Figure 4-5 and Table 2

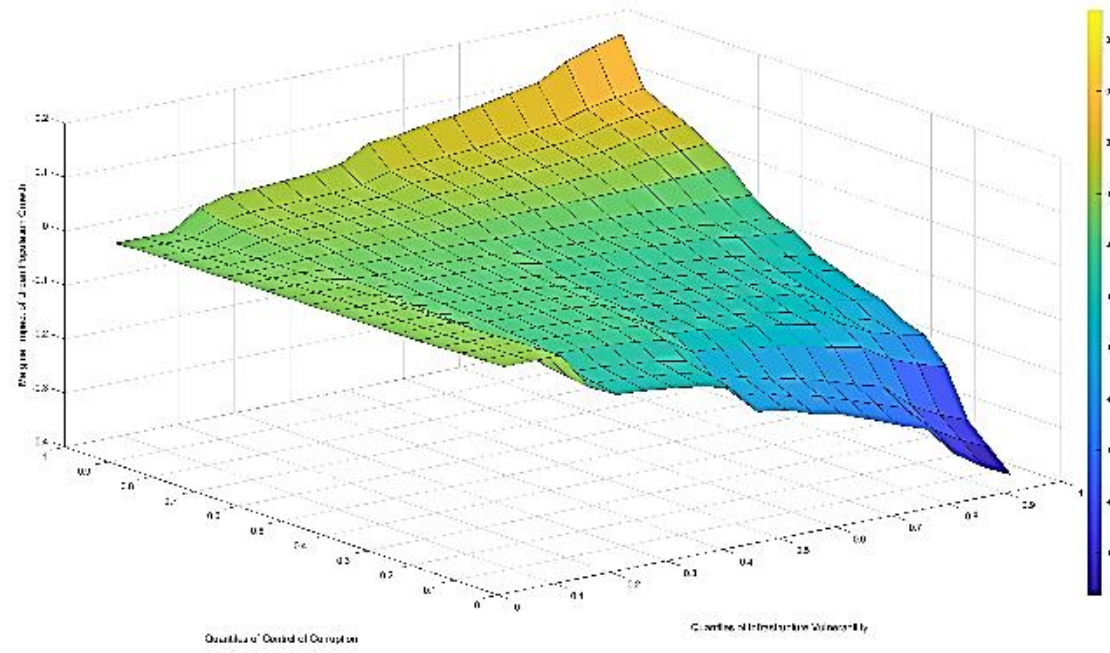

Governance Quality Indicator: Control of Corruption

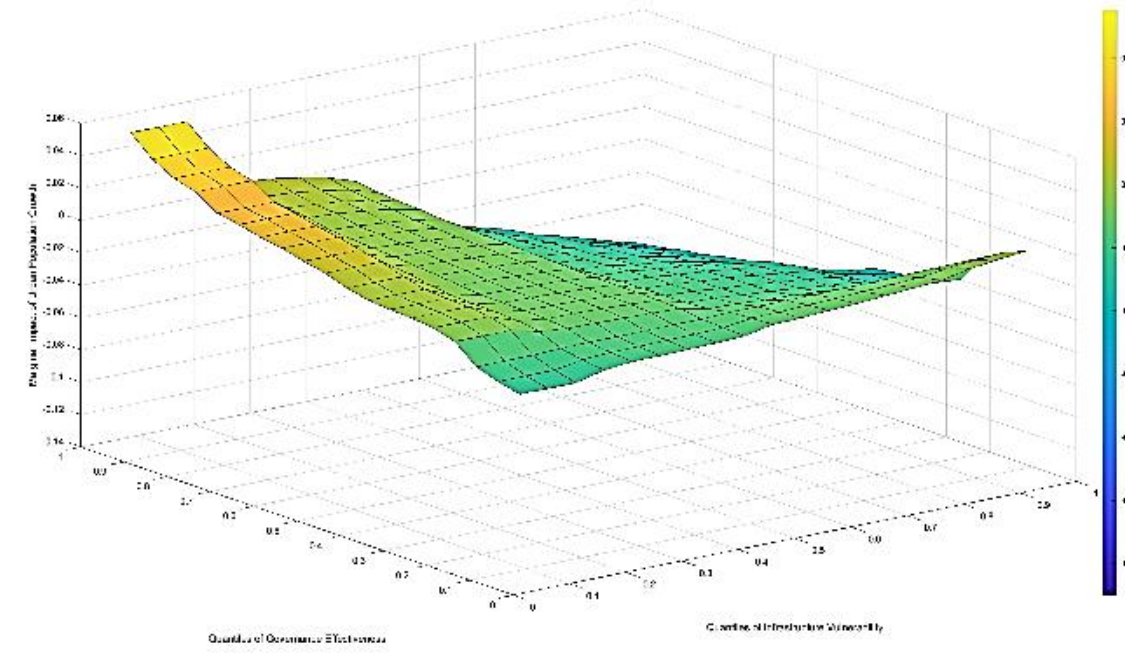

Governance Quality Indicator: Governance Effectiveness

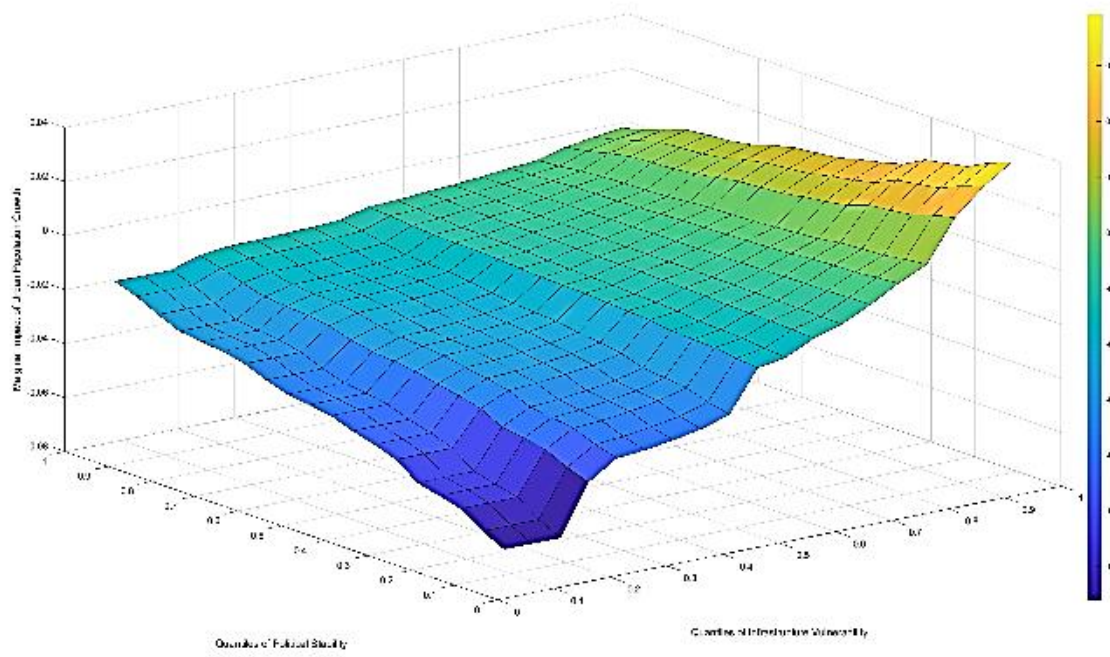

Governance Quality Indicator: Political Stability

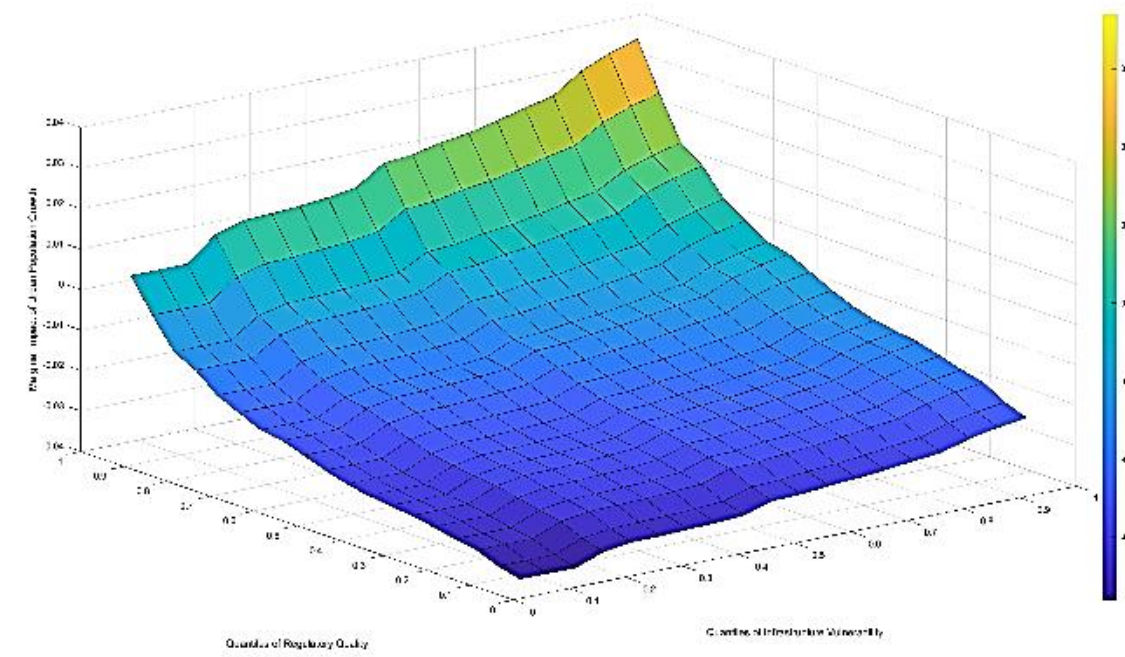

Governance Quality Indicator: Regulatory Quality

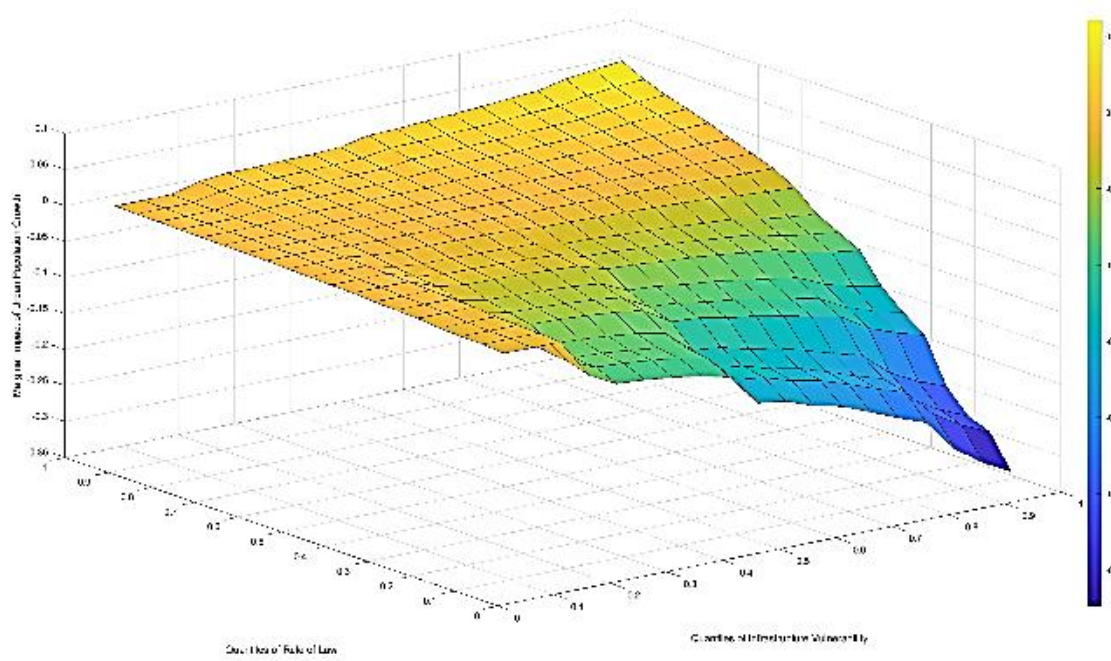

Governance Quality Indicator: Rule of Law

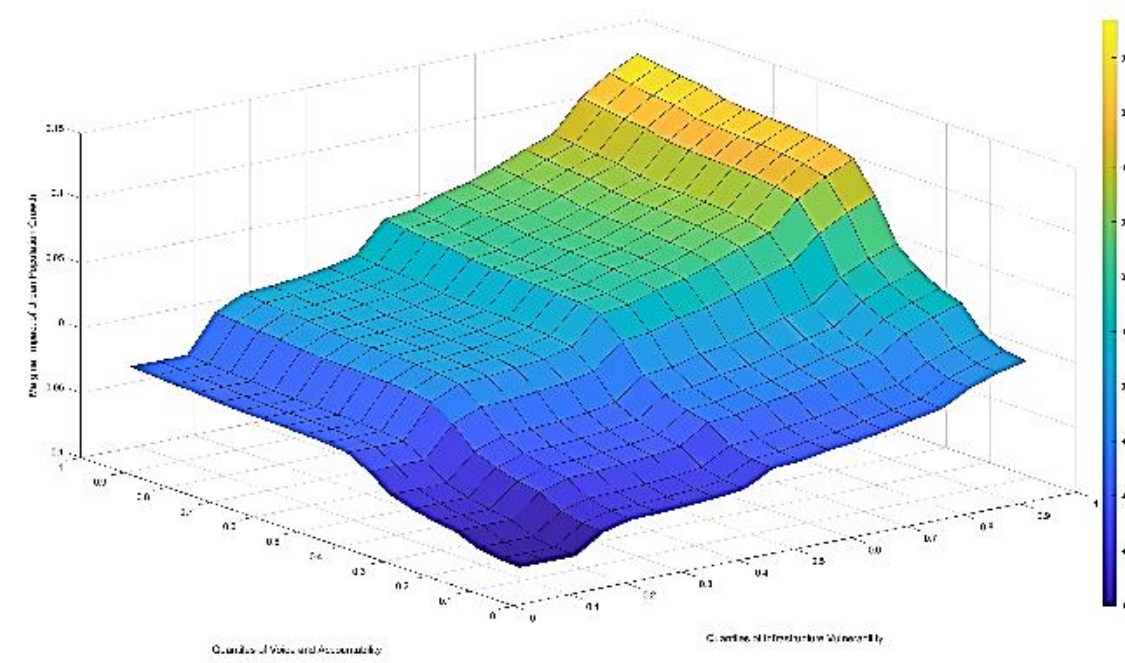

Governance Quality Indicator: Voice and Accountability

Figure S56: BRICS Policy Simulation Outcomes for the Marginal Impact of Urban Population Growth on SDG 7.1.2 under Infrastructure Vulnerability and Governance Quality, Related to Figure 4-5 and Table 2

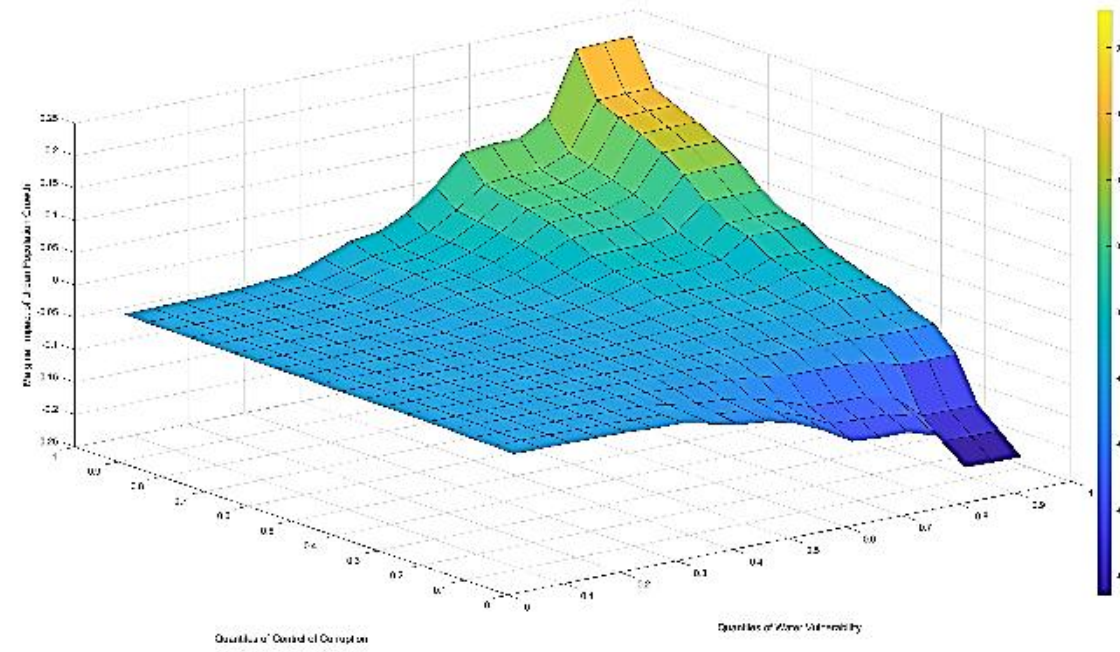

Governance Quality Indicator: Control of Corruption

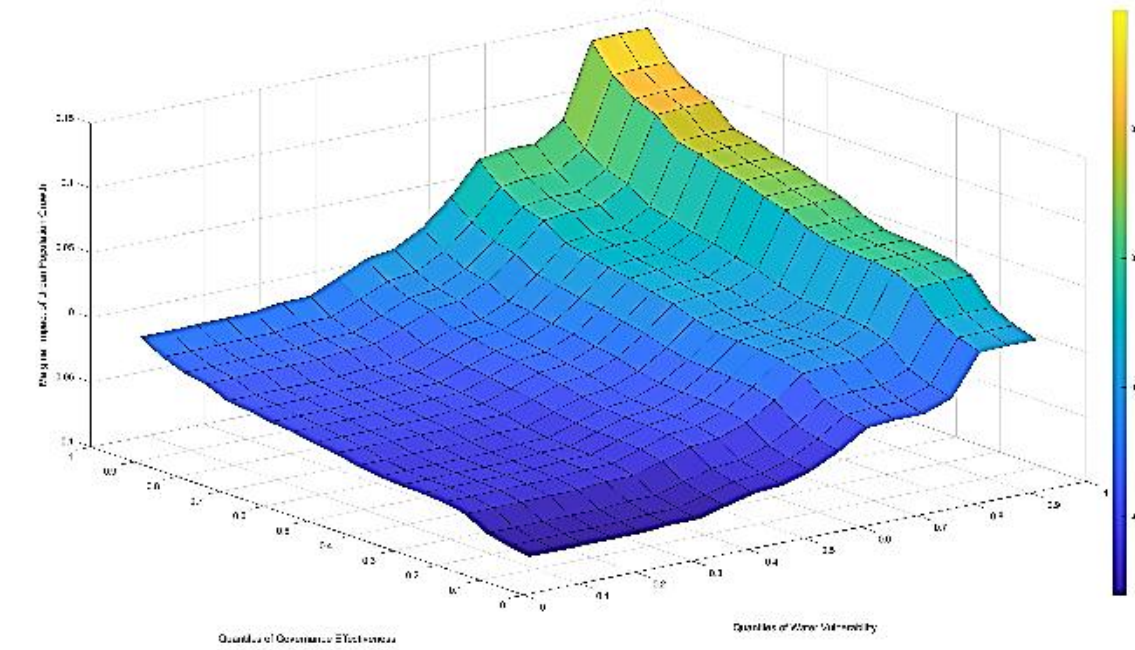

Governance Quality Indicator: Governance Effectiveness

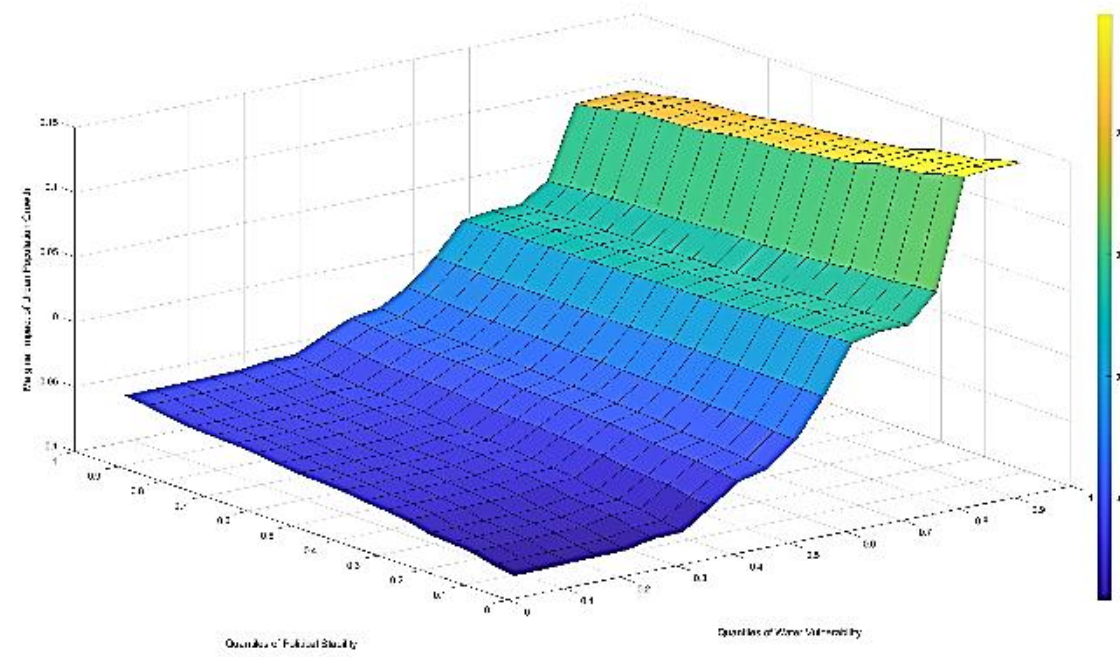

Governance Quality Indicator: Political Stability

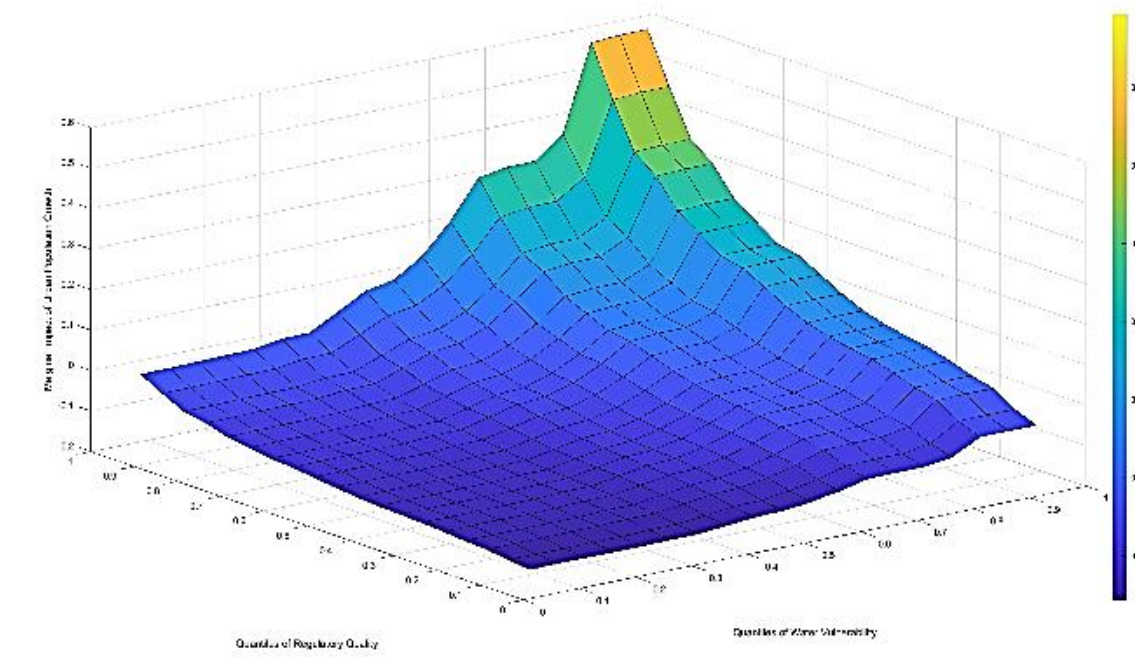

Governance Quality Indicator: Regulatory Quality

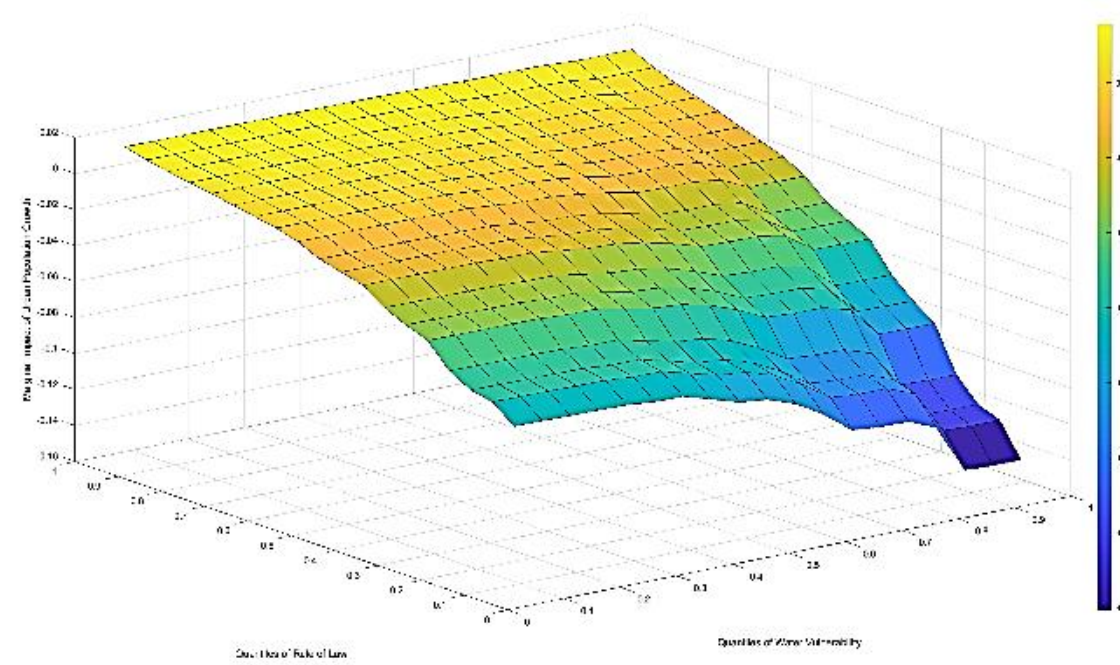

Governance Quality Indicator: Rule of Law

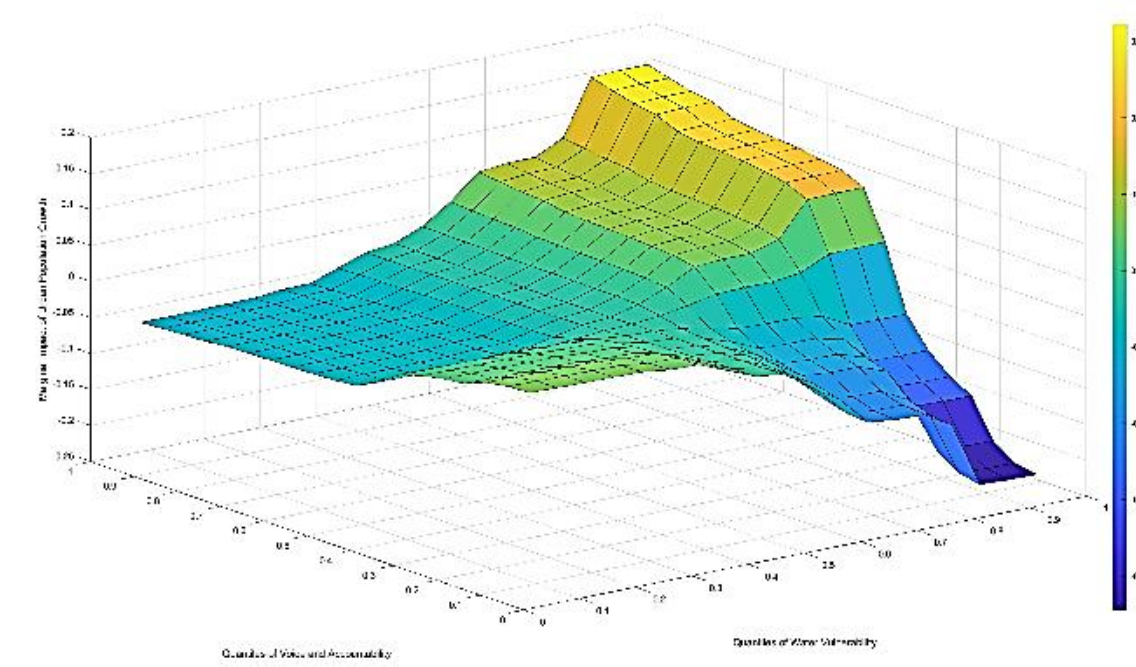

Governance Quality Indicator: Voice and Accountability

Figure S57: BRICS Policy Simulation Outcomes for the Marginal Impact of Urban Population Growth on SDG 7.1.2 under Water Vulnerability and Governance Quality, Related to Figure 4-5 and Table 2

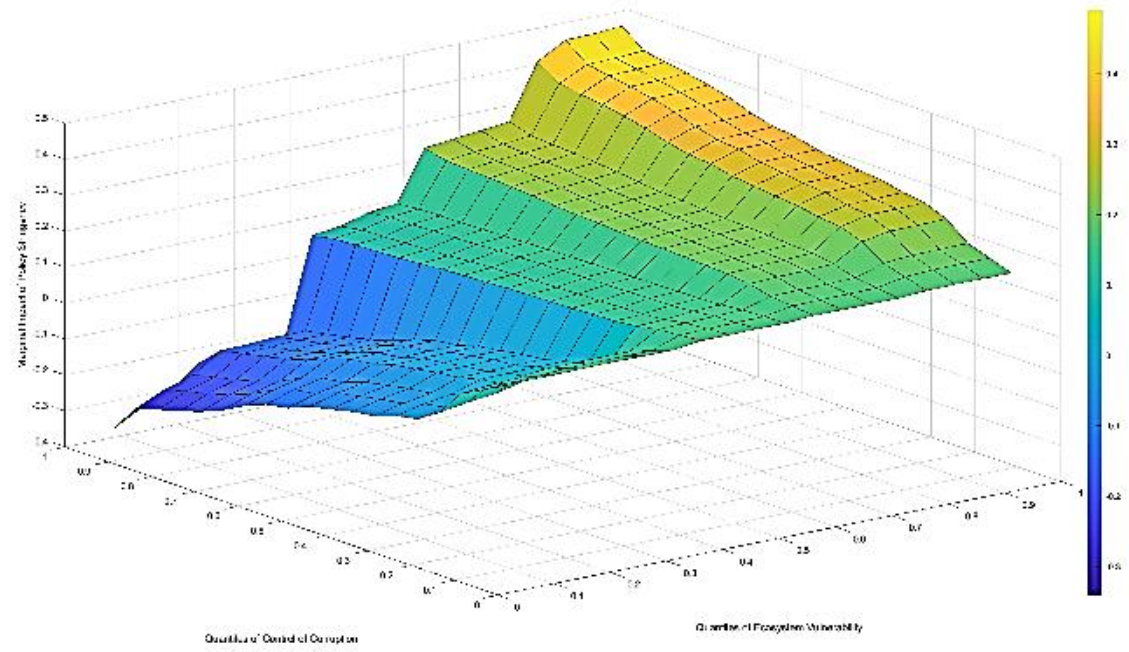

Governance Quality Indicator: Control of Corruption

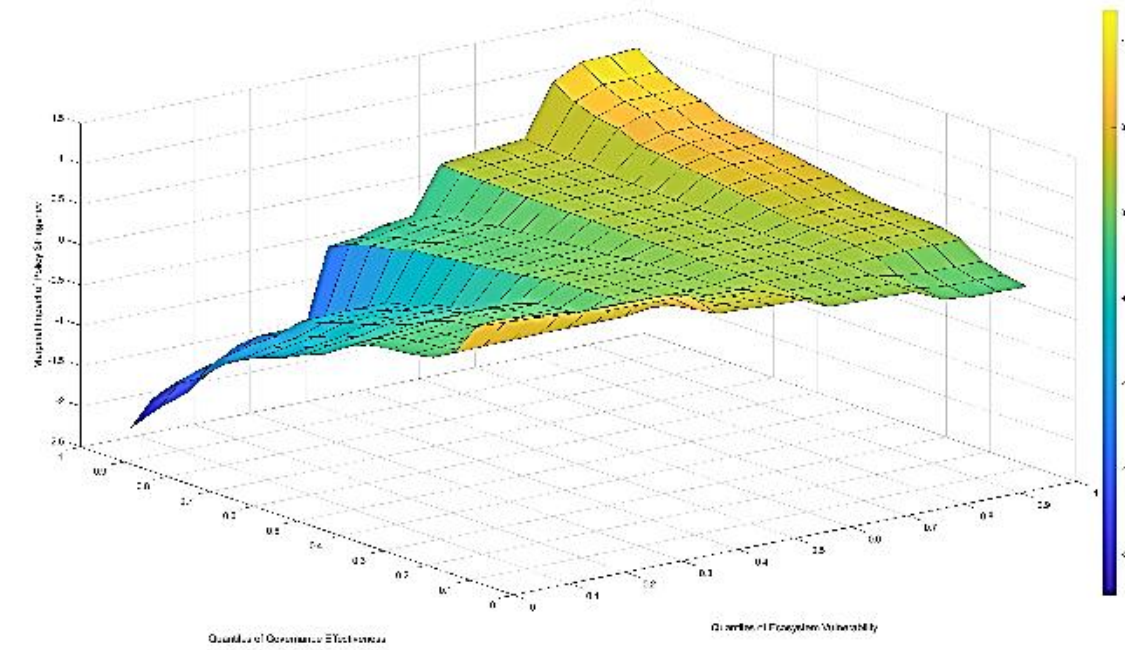

Governance Quality Indicator: Governance Effectiveness

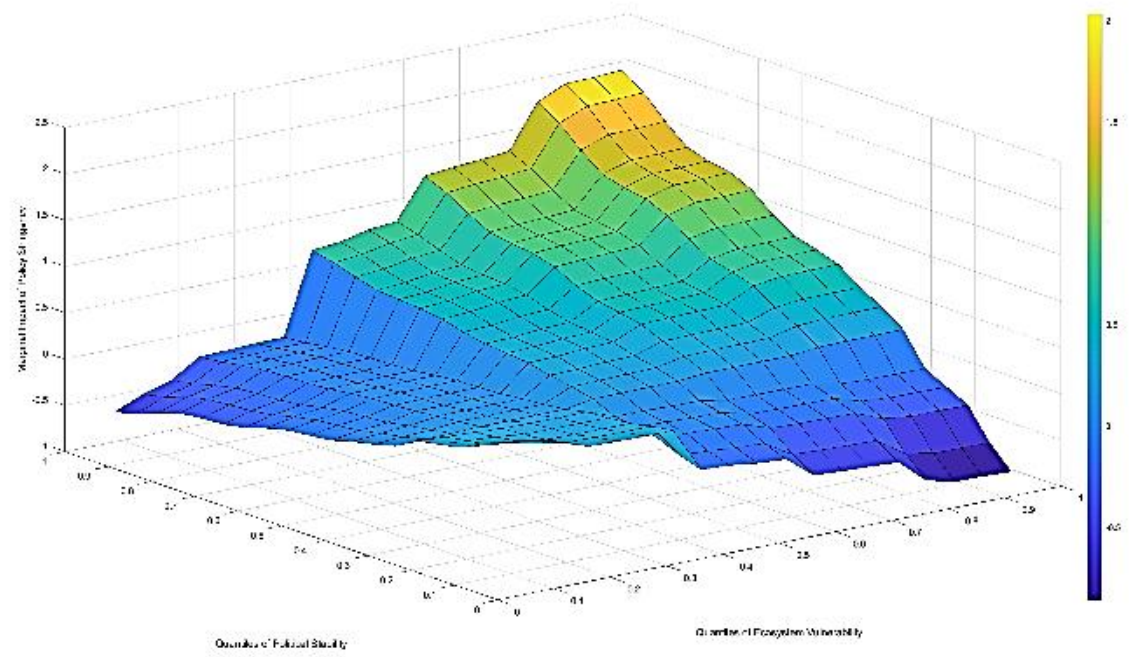

Governance Quality Indicator: Political Stability

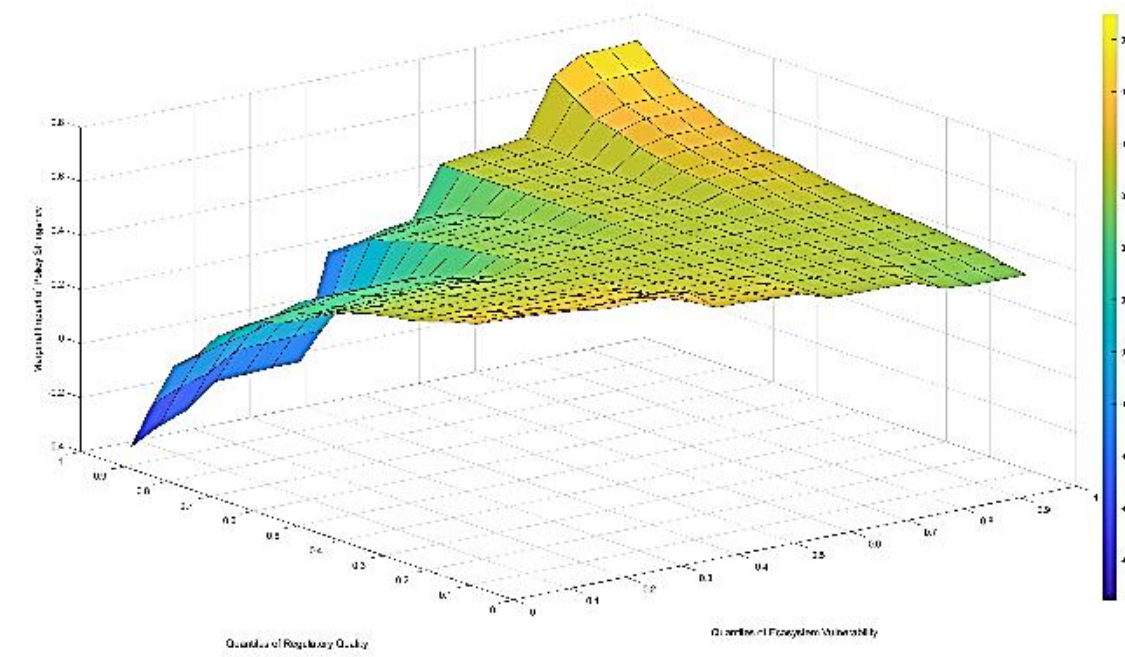

Governance Quality Indicator: Regulatory Quality

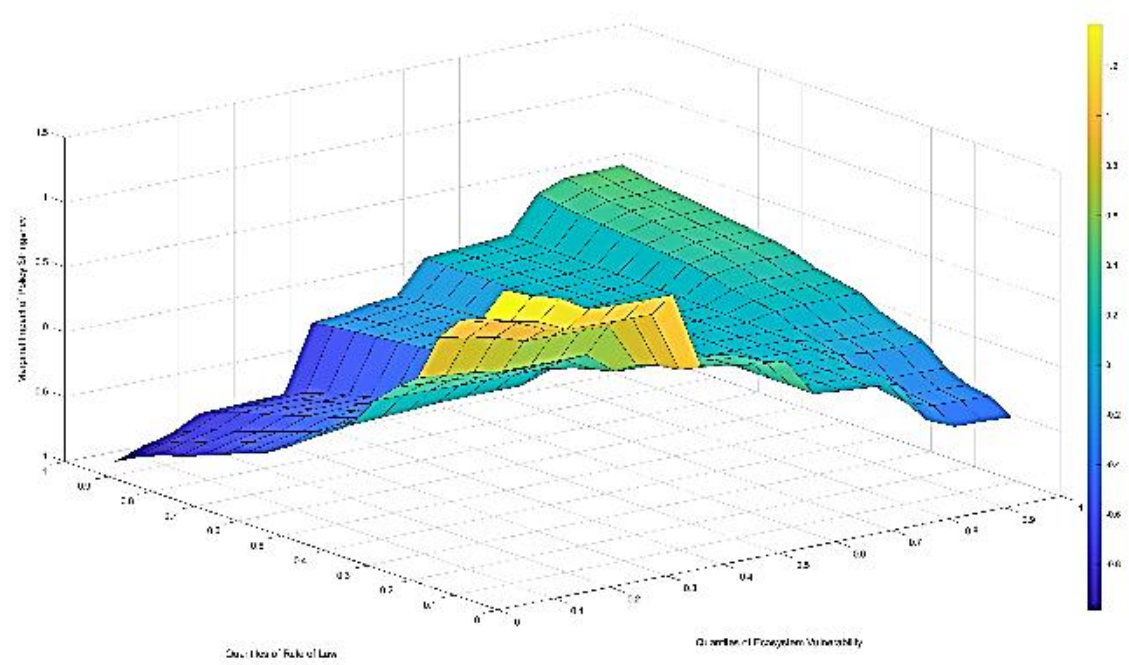

Governance Quality Indicator: Rule of Law

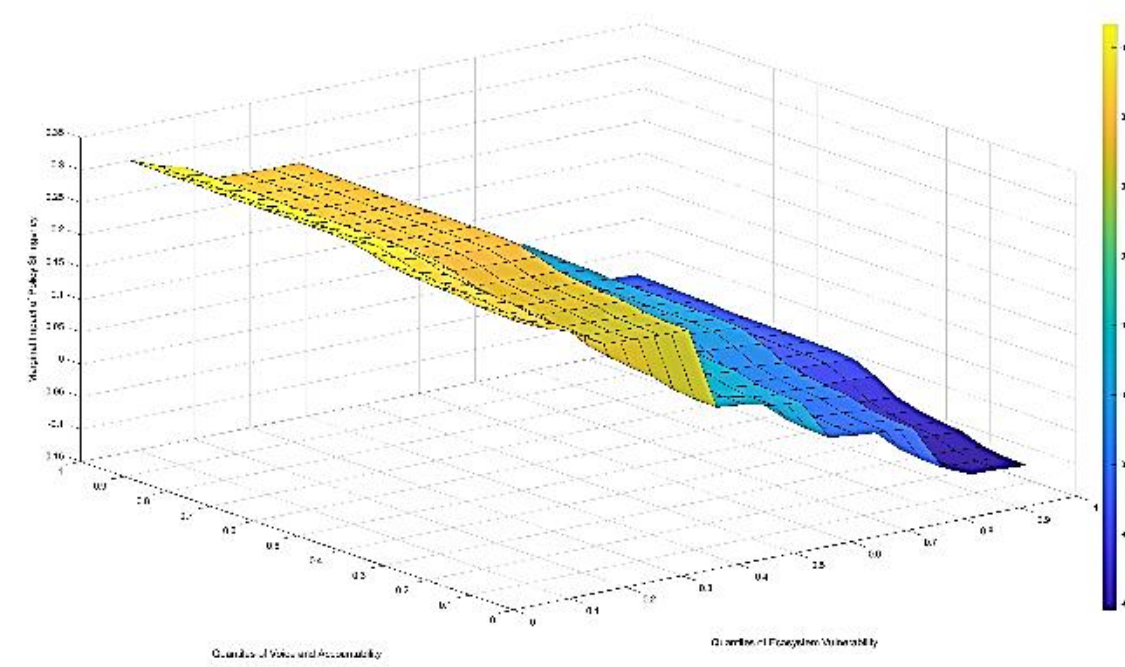

Governance Quality Indicator: Voice and Accountability

Figure S58: BRICS Policy Simulation Outcomes for the Marginal Impact of Policy Stringency on SDG 7.2.1 under Ecosystem Vulnerability and Governance Quality, Related to Figure 4-5 and Table 2

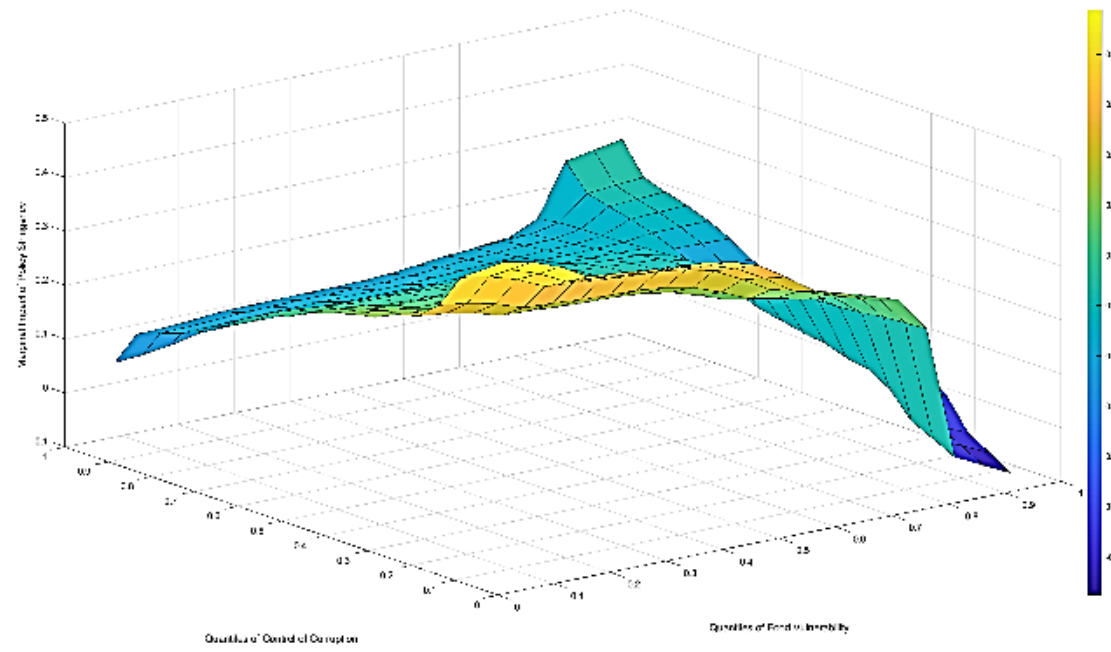

Governance Quality Indicator: Control of Corruption

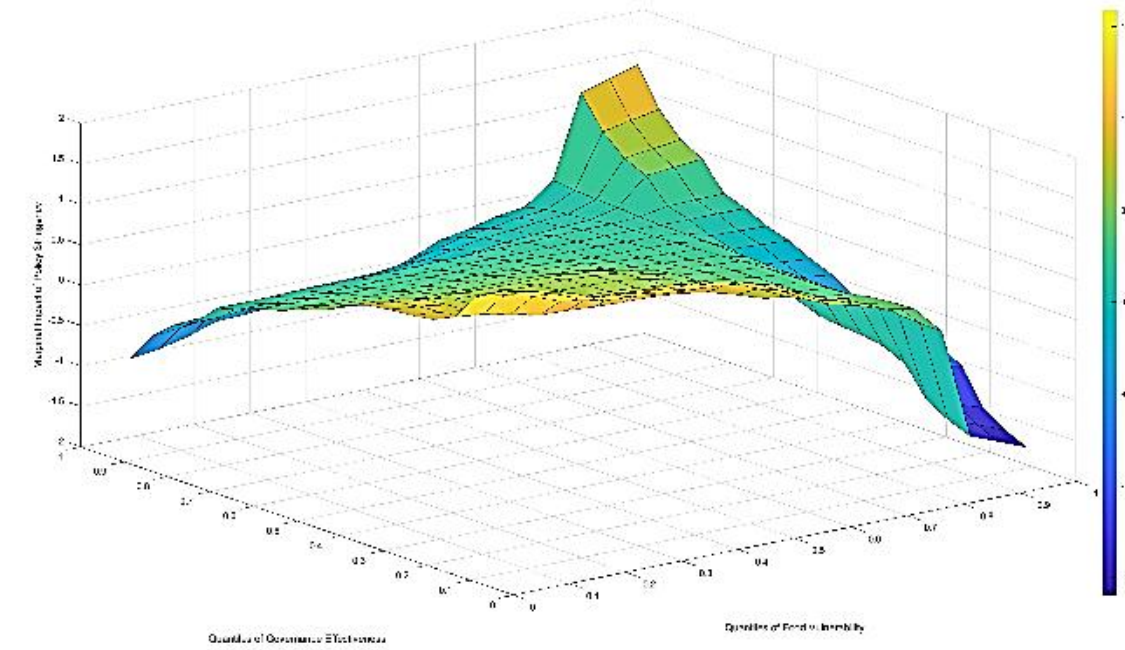

Governance Quality Indicator: Governance Effectiveness

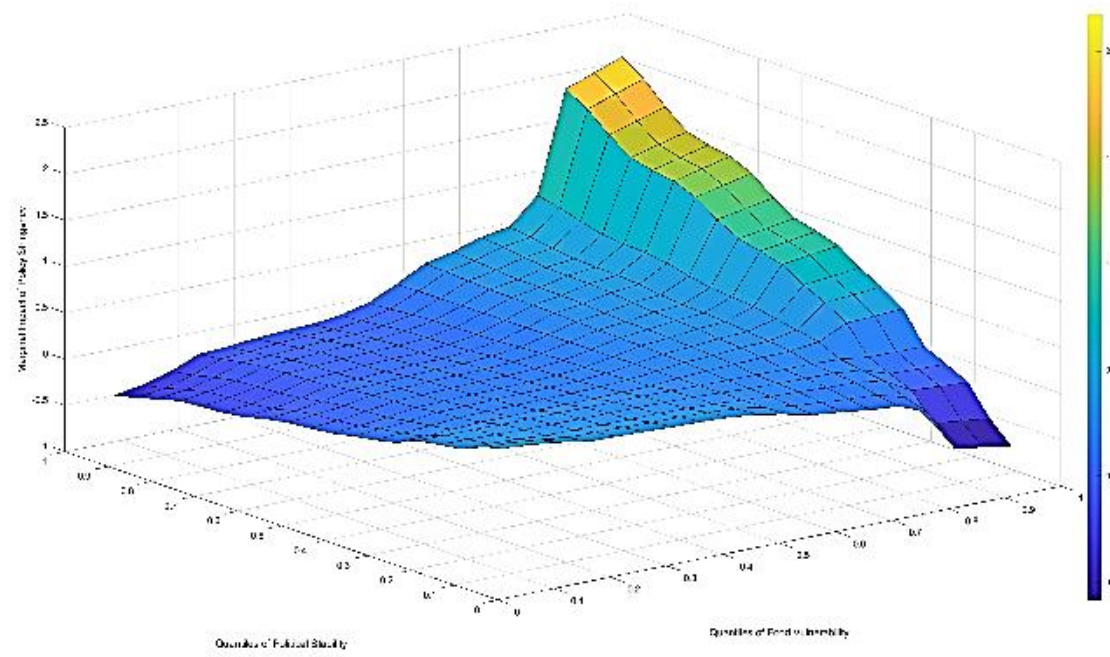

Governance Quality Indicator: Political Stability

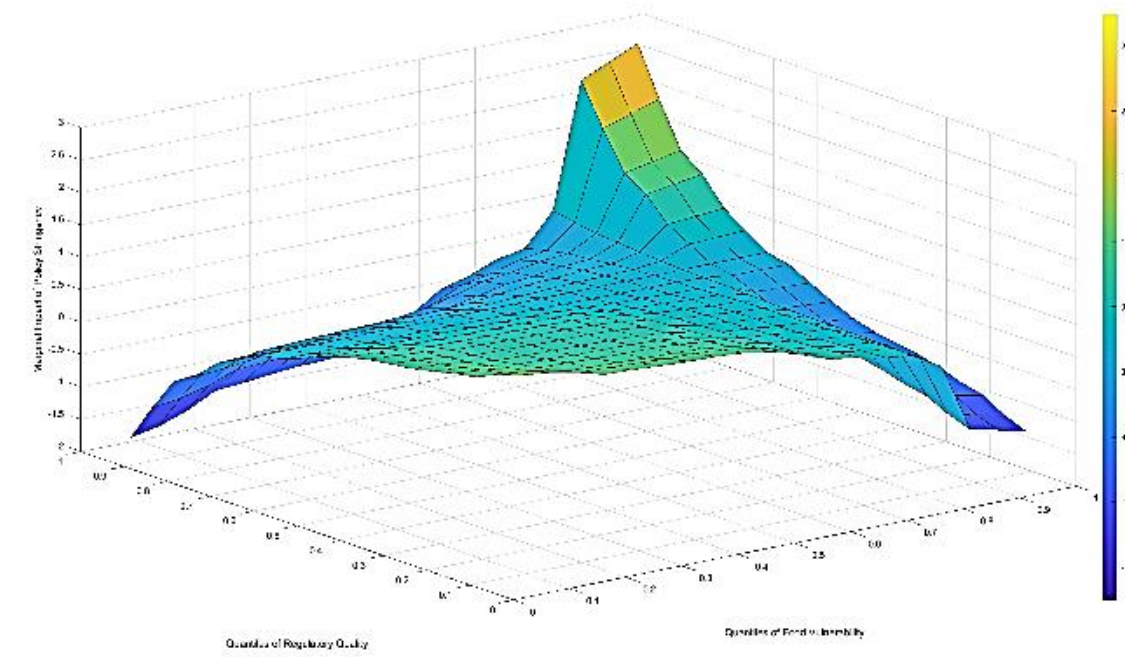

Governance Quality Indicator: Regulatory Quality

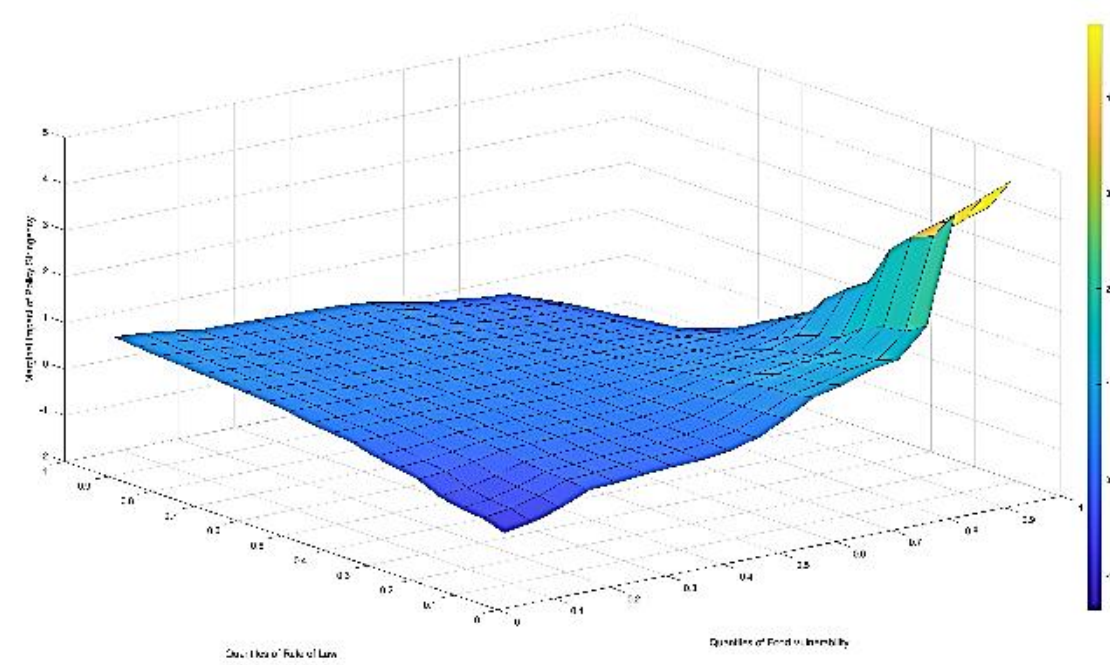

Governance Quality Indicator: Rule of Law

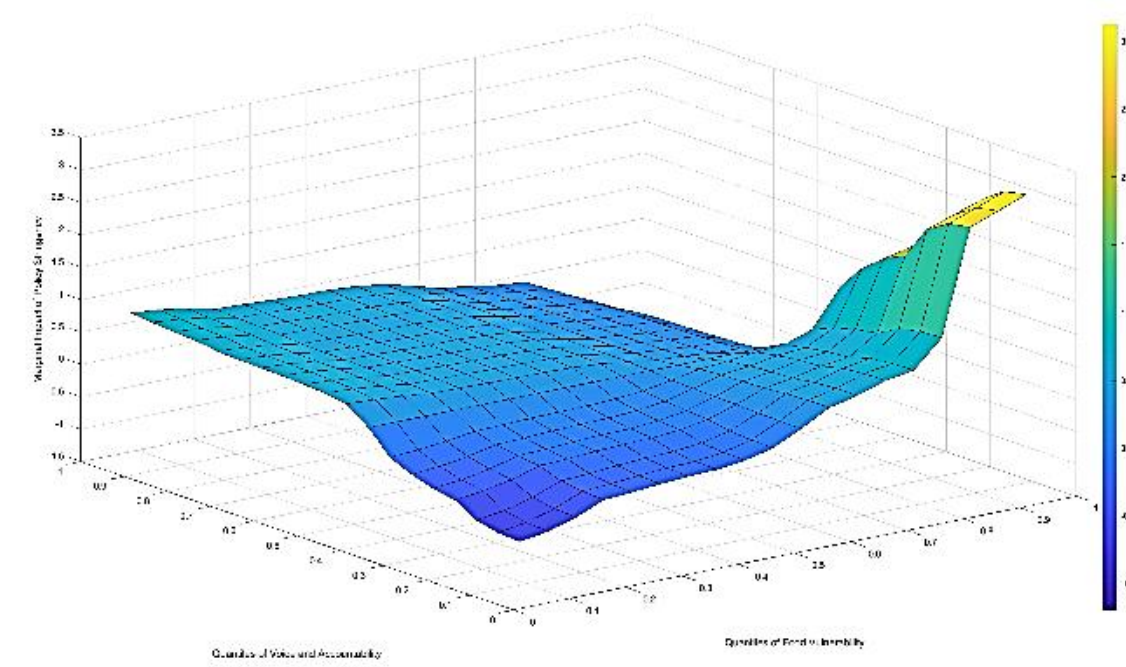

Governance Quality Indicator: Voice and Accountability

Figure S59: BRICS Policy Simulation Outcomes for the Marginal Impact of Policy Stringency on SDG 7.2.1 under Food Vulnerability and Governance Quality, Related to Figure 4-5 and Table 2

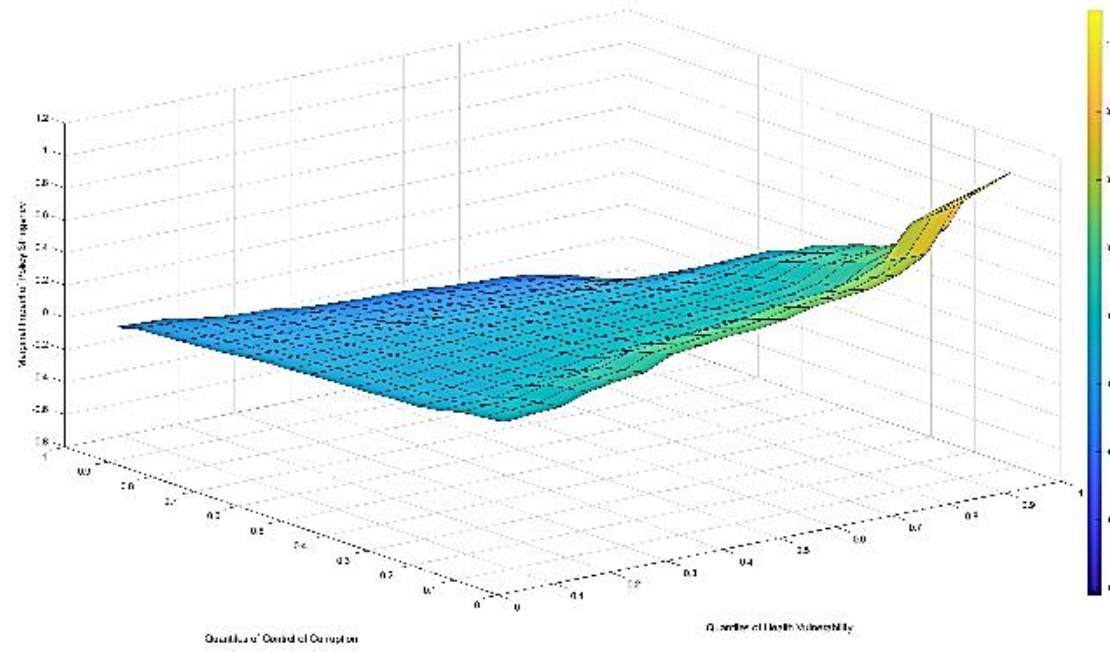

Governance Quality Indicator: Control of Corruption

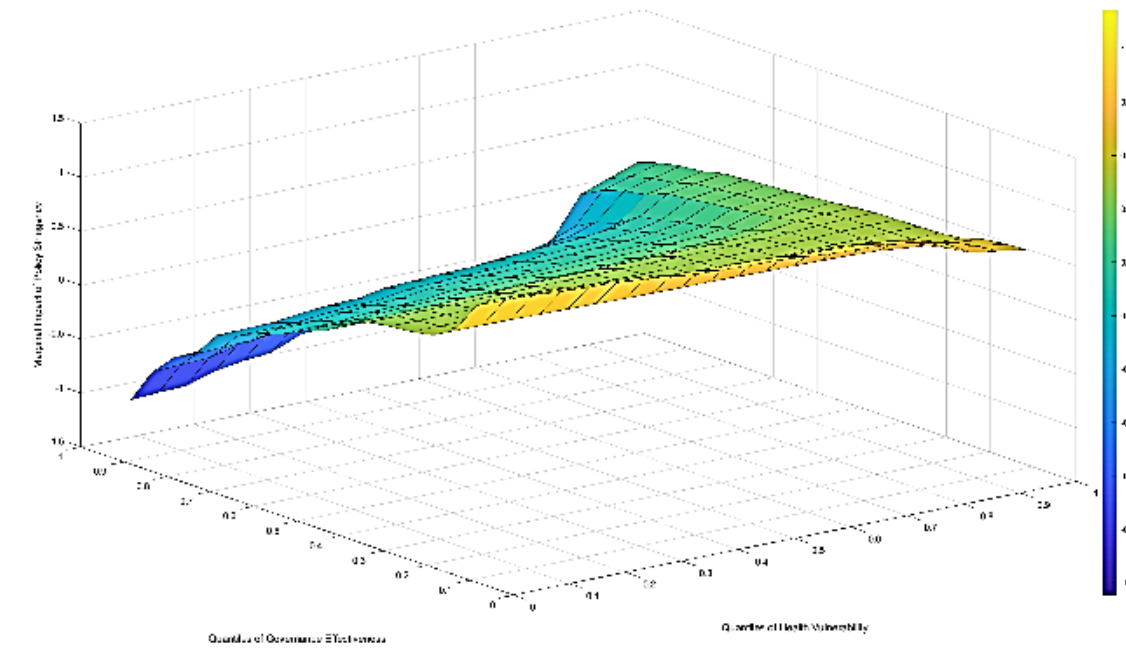

Governance Quality Indicator: Governance Effectiveness

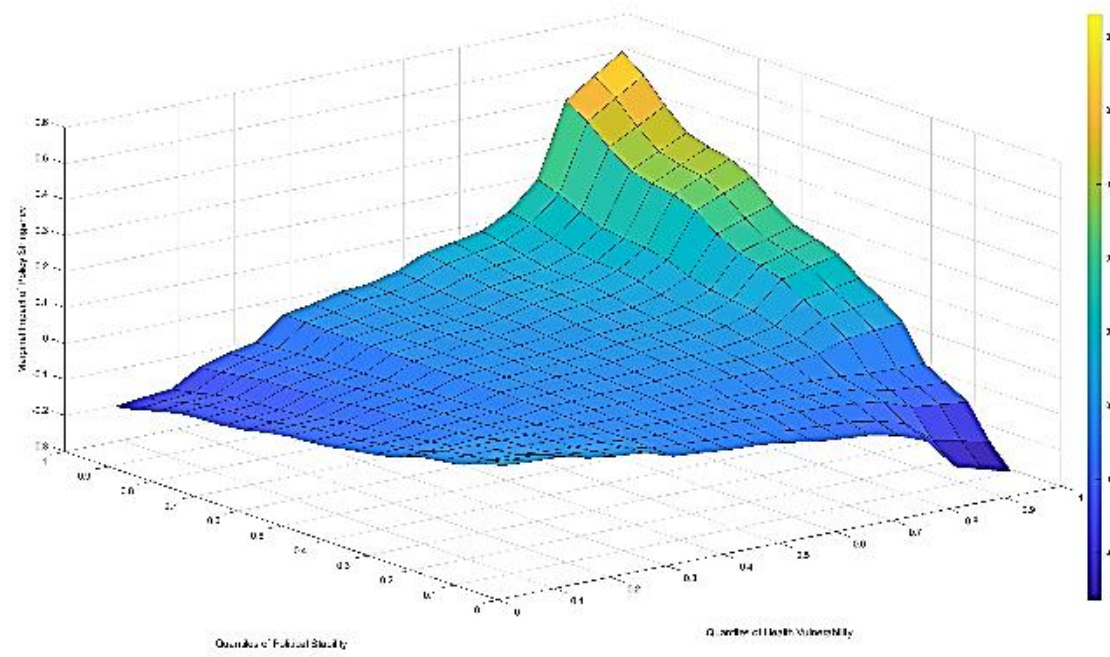

Governance Quality Indicator: Political Stability

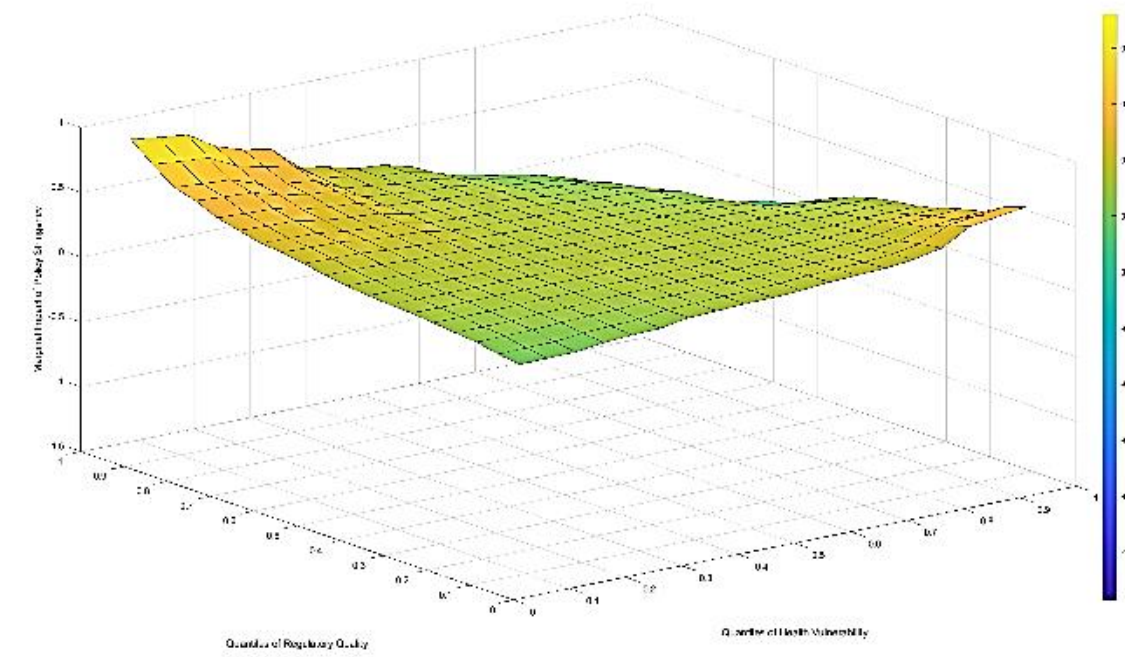

Governance Quality Indicator: Regulatory Quality

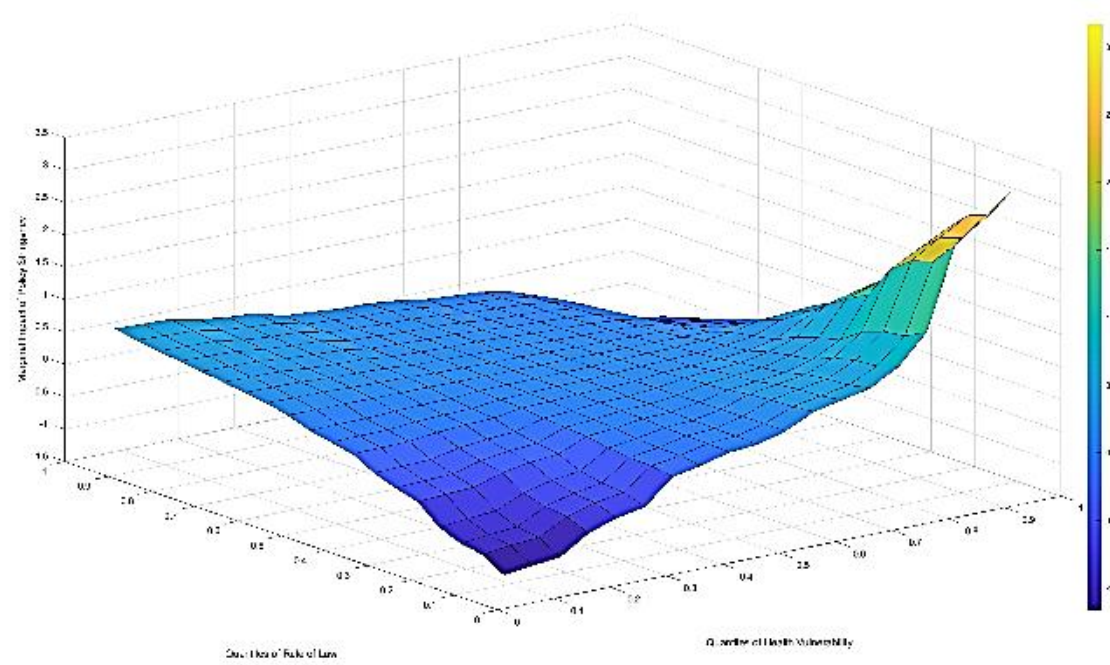

Governance Quality Indicator: Rule of Law

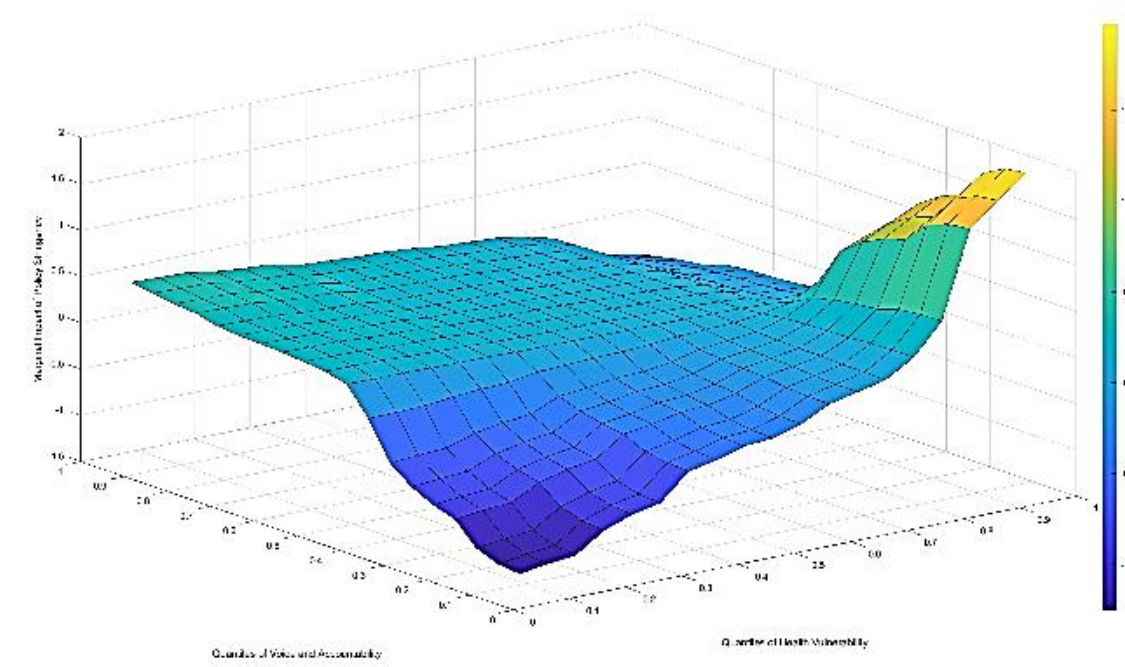

Governance Quality Indicator: Voice and Accountability

Figure S60: BRICS Policy Simulation Outcomes for the Marginal Impact of Policy Stringency on SDG 7.2.1 under Health Vulnerability and Governance Quality, Related to Figure 4-5 and Table 2

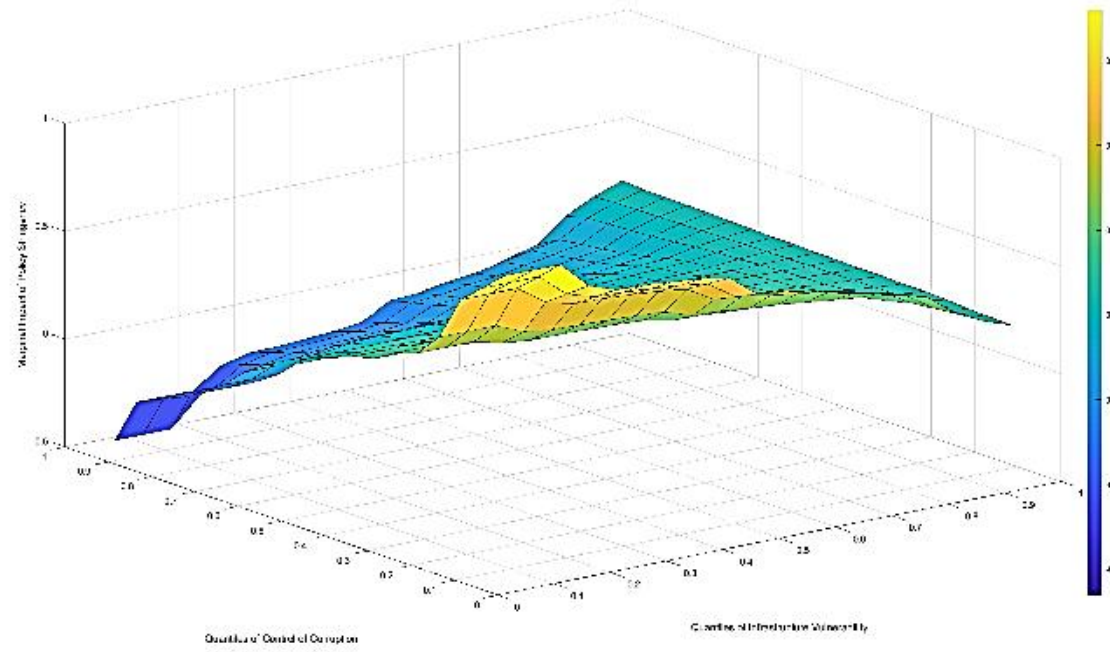

Governance Quality Indicator: Control of Corruption

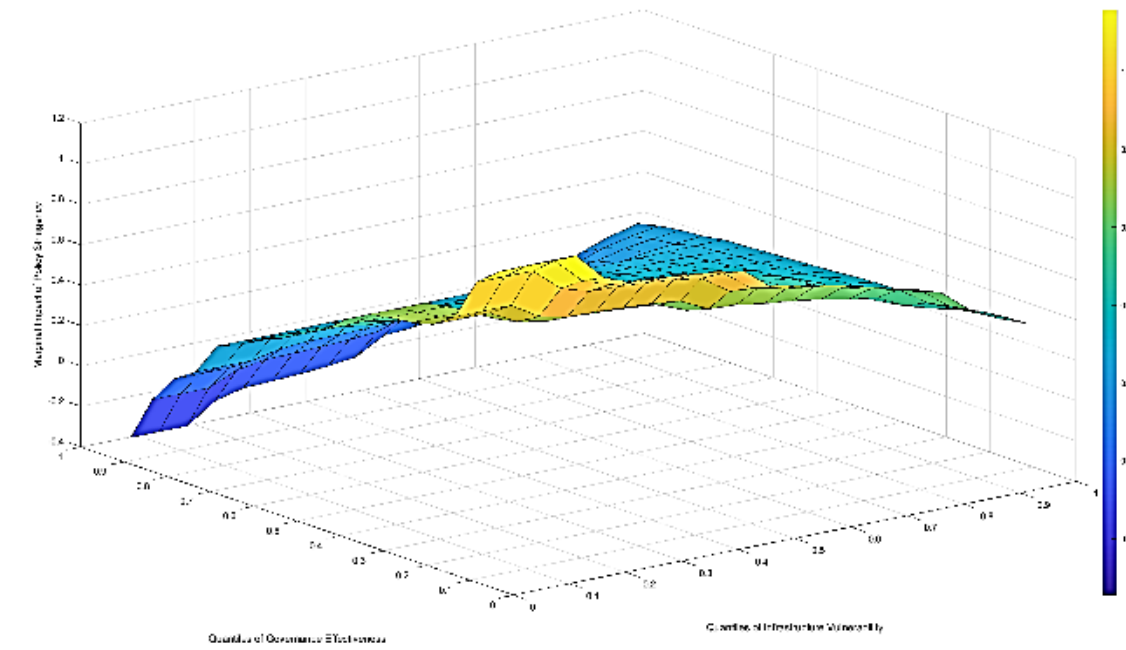

Governance Quality Indicator: Governance Effectiveness

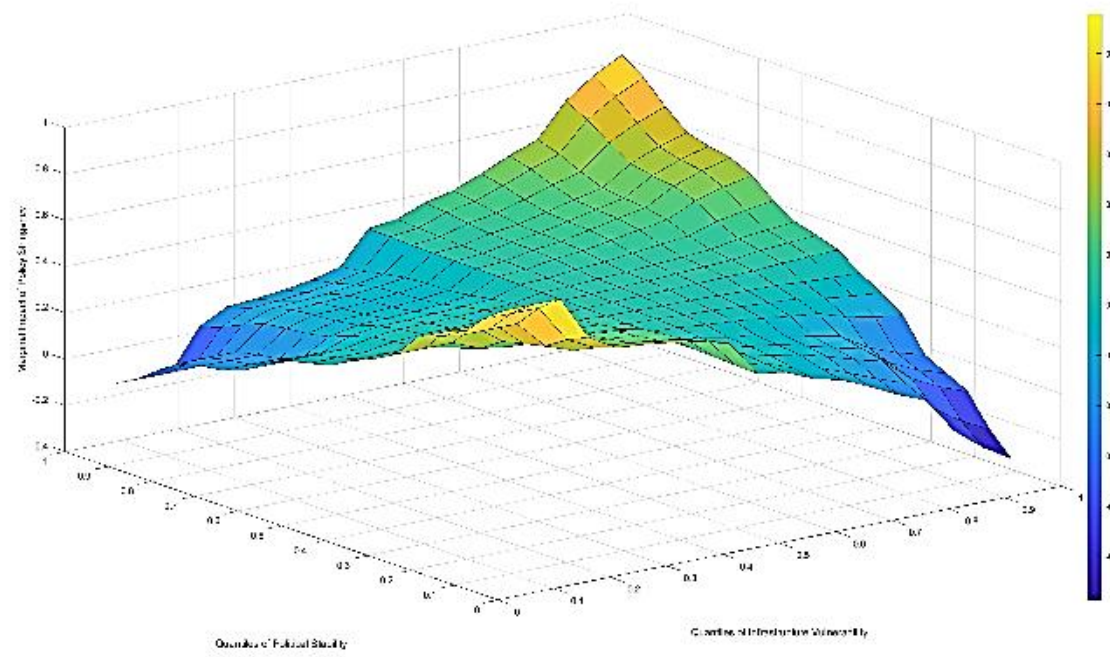

Governance Quality Indicator: Political Stability

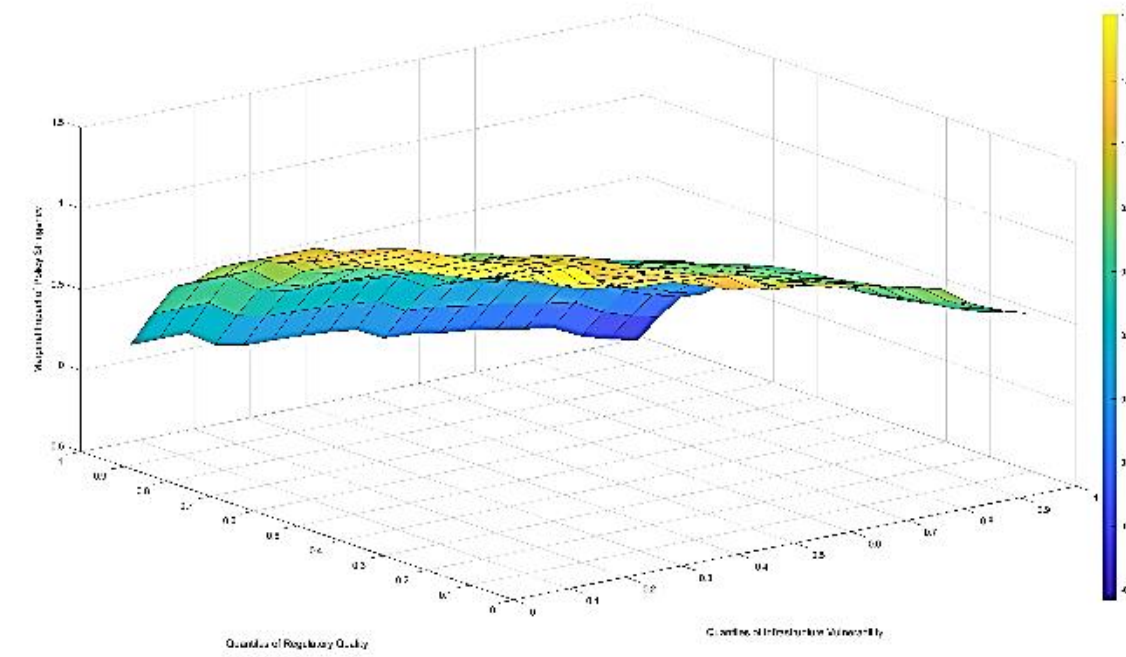

Governance Quality Indicator: Regulatory Quality

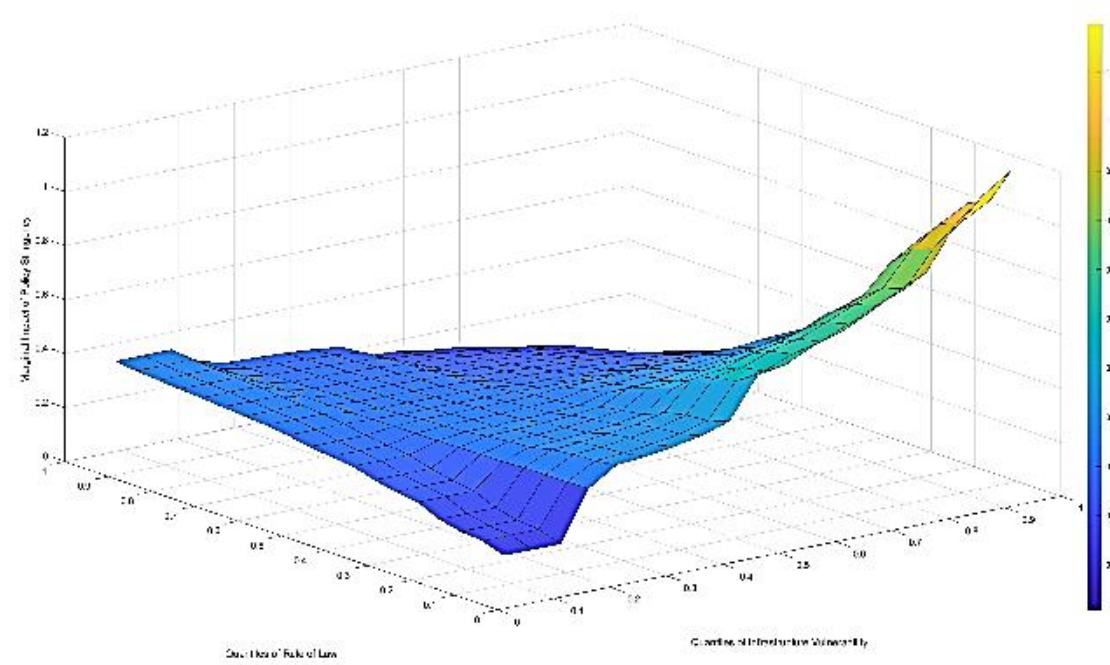

Governance Quality Indicator: Rule of Law

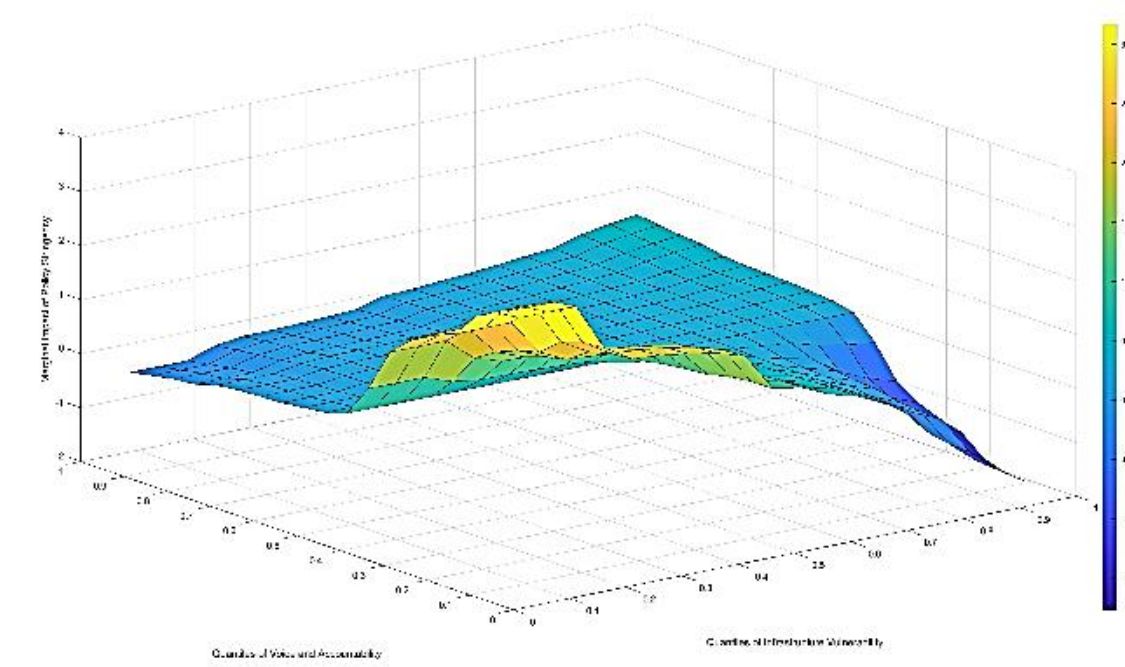

Governance Quality Indicator: Voice and Accountability

Figure S61: BRICS Policy Simulation Outcomes for the Marginal Impact of Policy Stringency on SDG 7.2.1 under Infrastructure Vulnerability and Governance Quality, Related to Figure 4-5 and Table 2

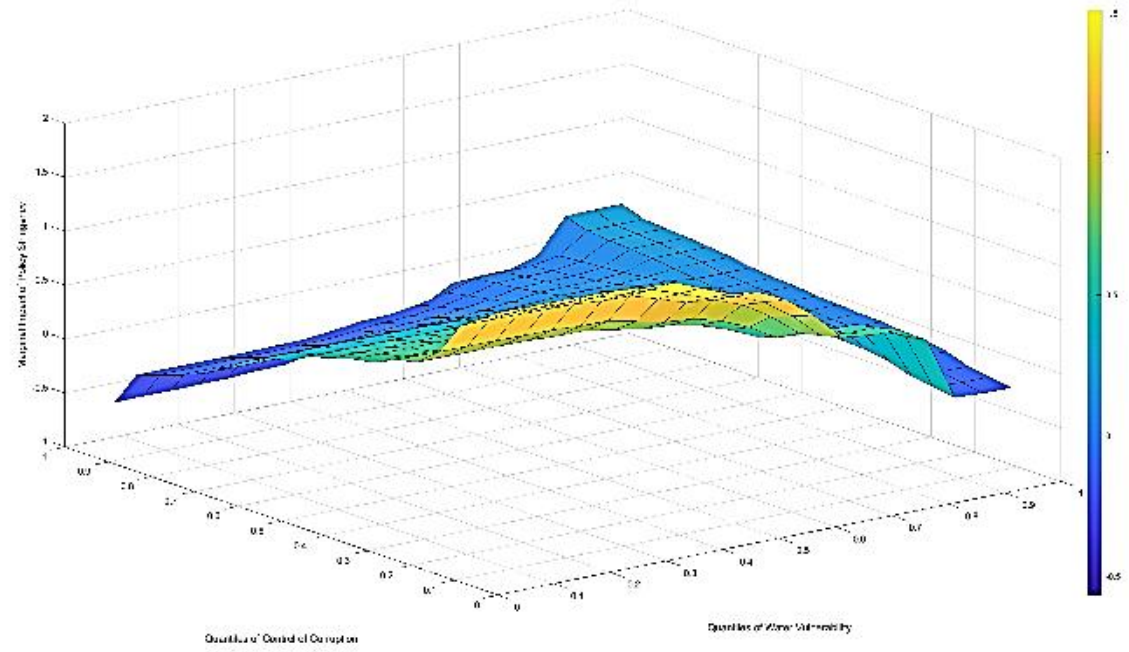

Governance Quality Indicator: Control of Corruption

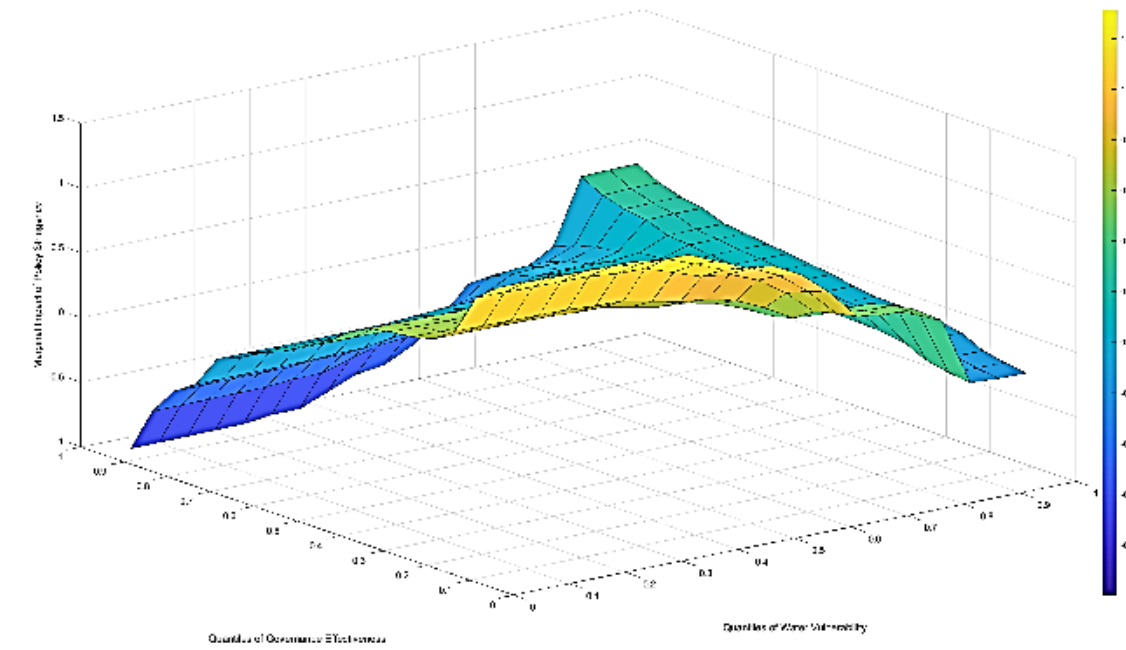

Governance Quality Indicator: Governance Effectiveness

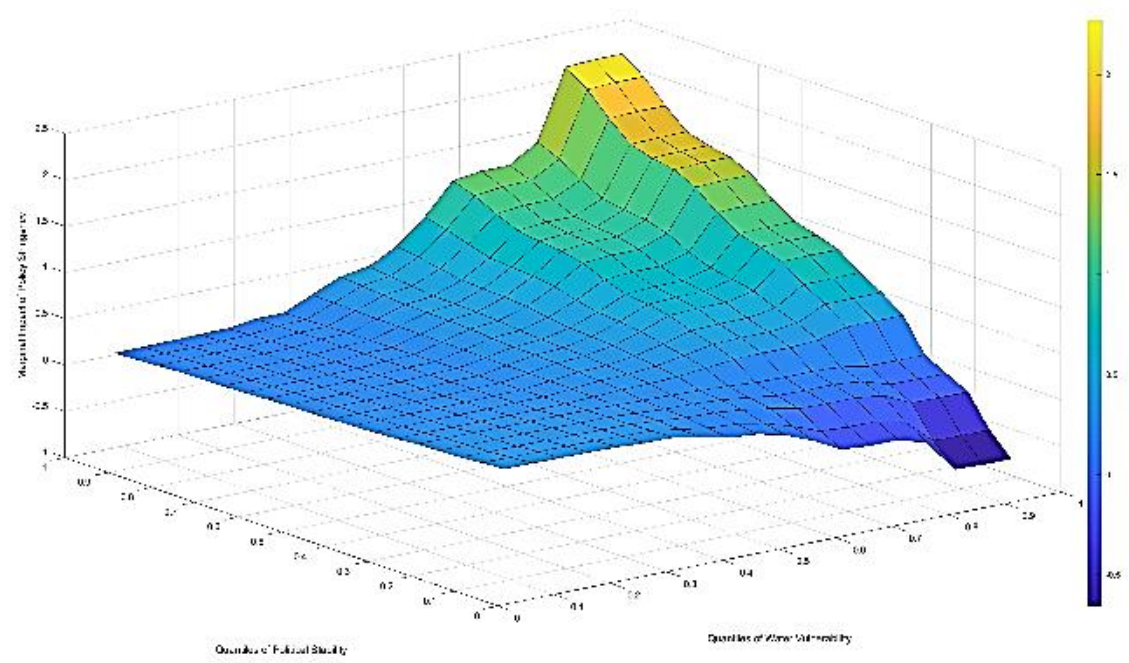

Governance Quality Indicator: Political Stability

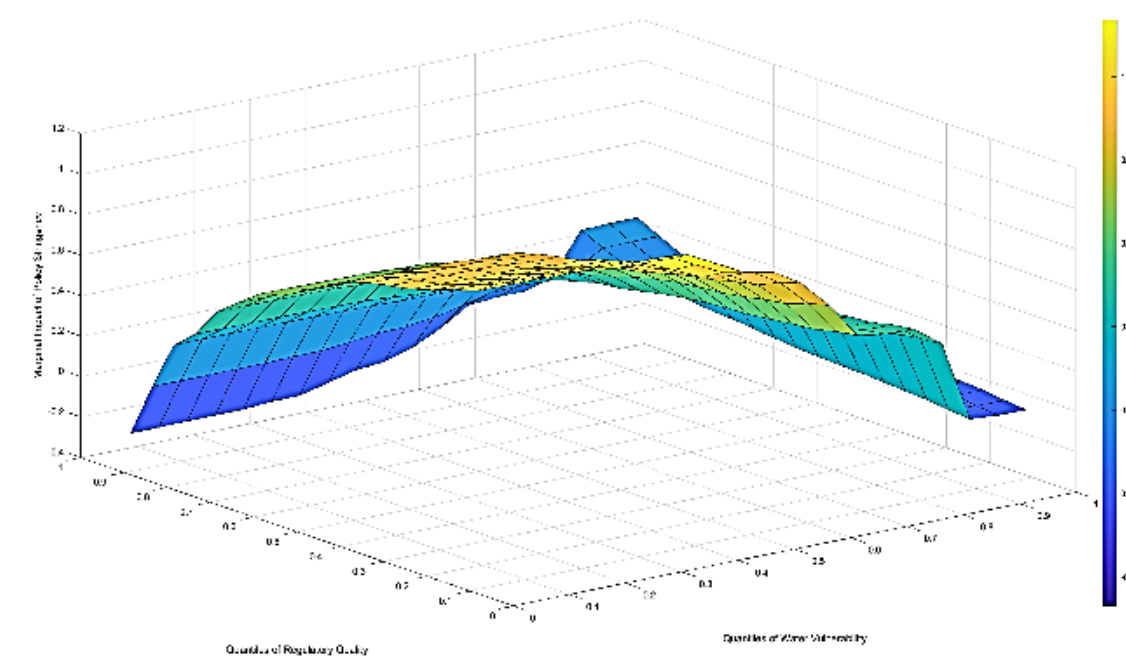

Governance Quality Indicator: Regulatory Quality

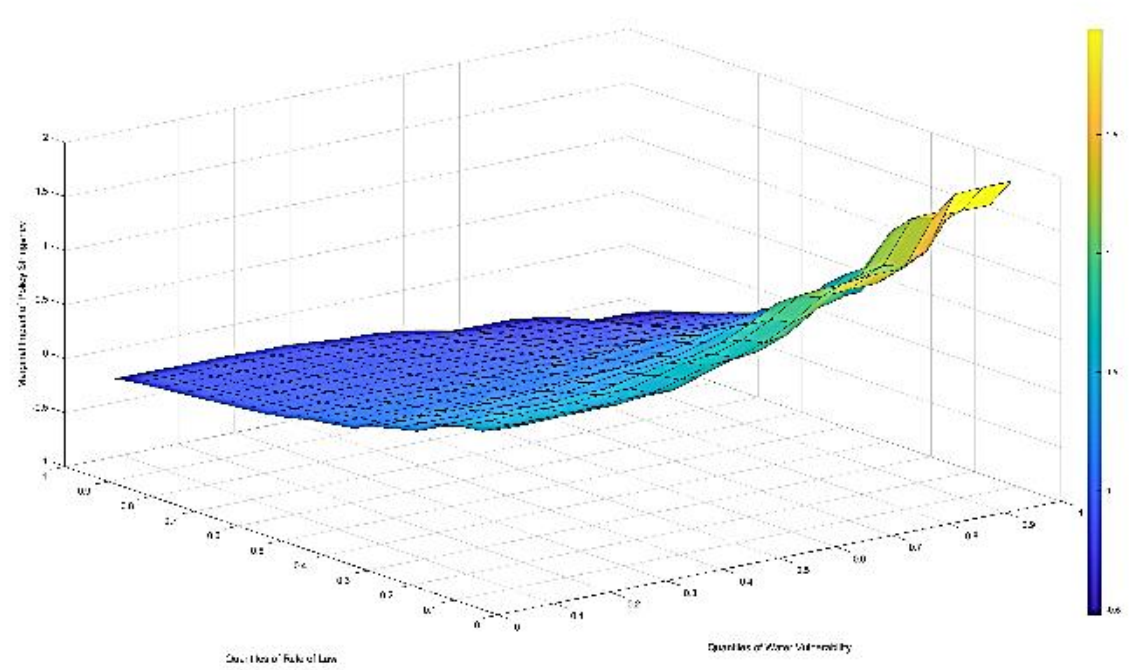

Governance Quality Indicator: Rule of Law

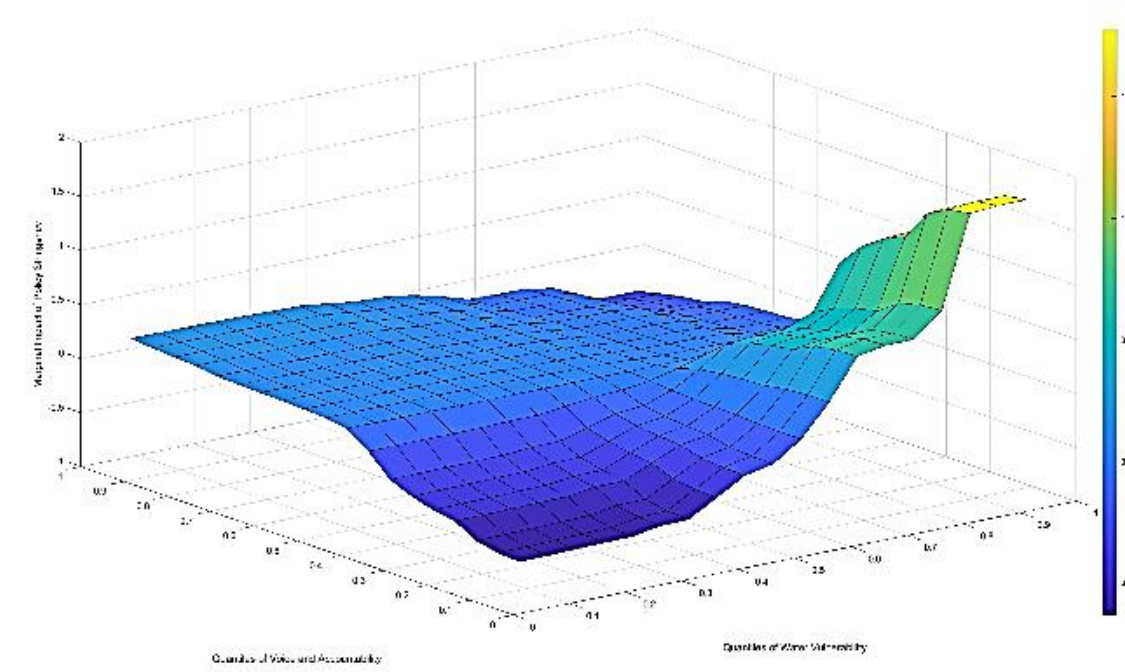

Governance Quality Indicator: Voice and Accountability

Figure S62: BRICS Policy Simulation Outcomes for the Marginal Impact of Policy Stringency on SDG 7.2.1 under Water Vulnerability and Governance Quality, Related to Figure 4-5 and Table 2

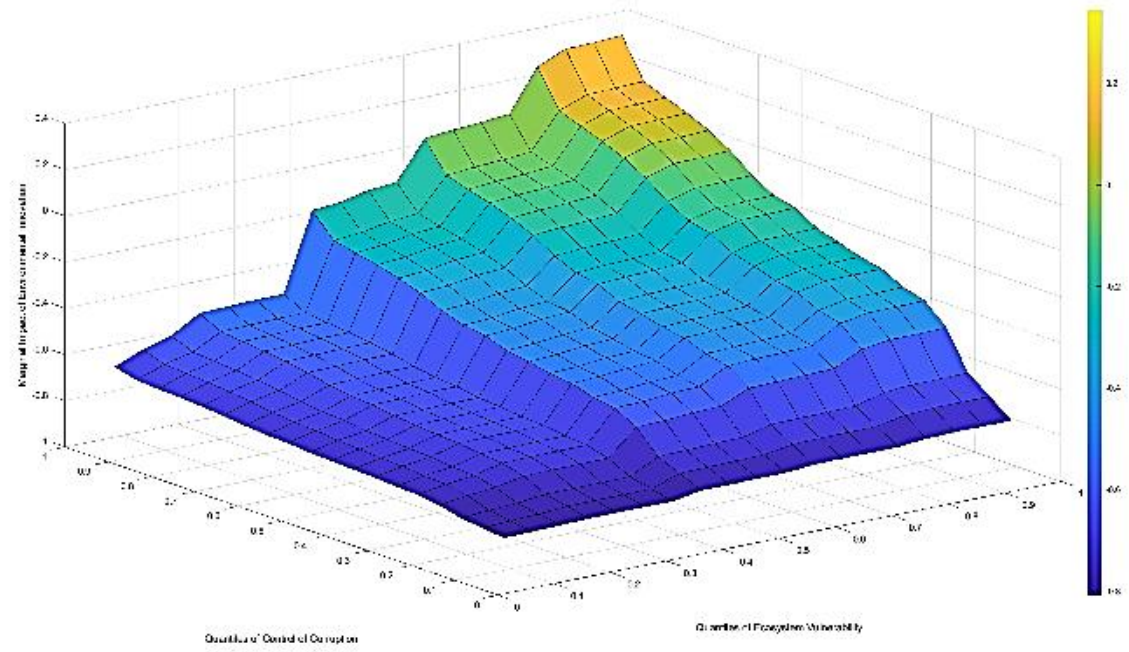

Governance Quality Indicator: Control of Corruption

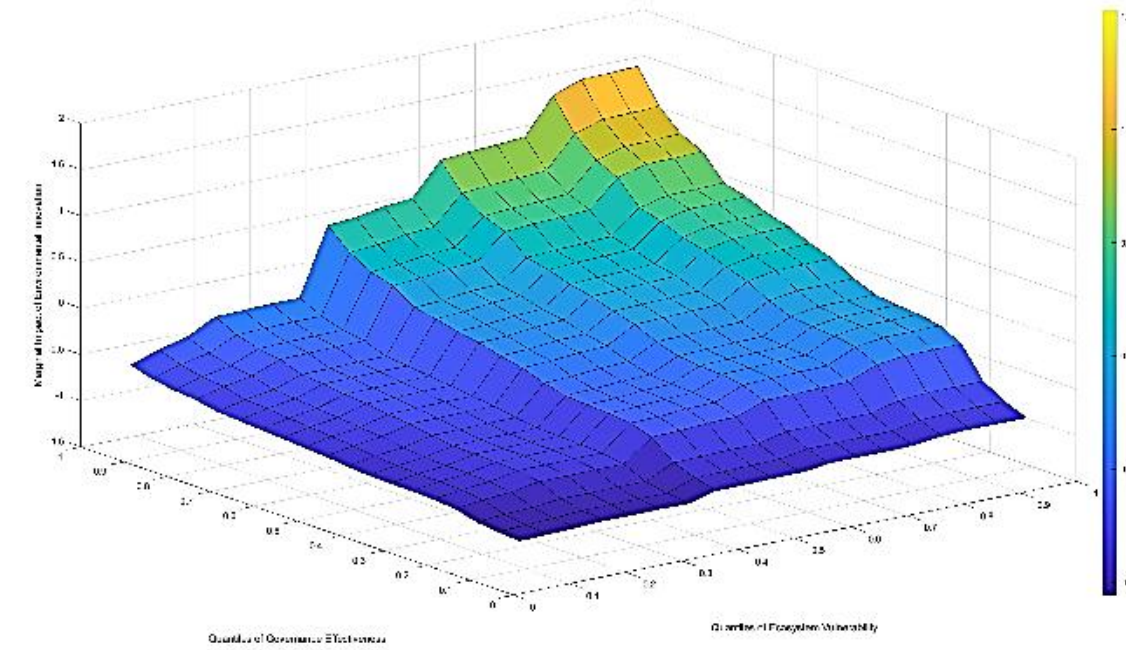

Governance Quality Indicator: Governance Effectiveness

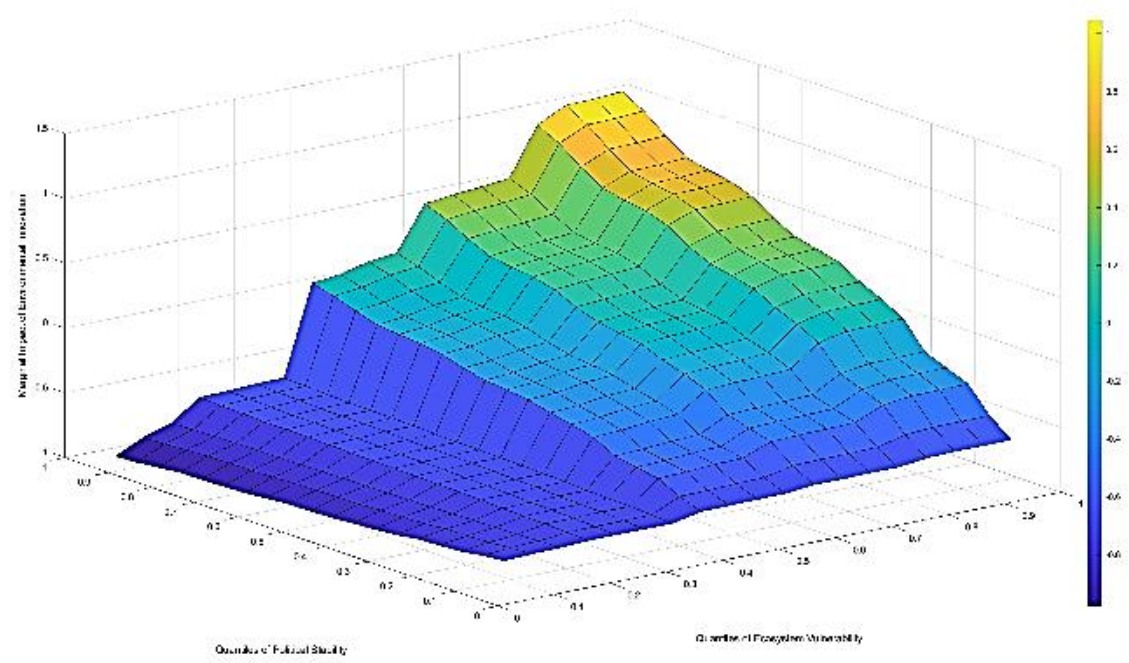

Governance Quality Indicator: Political Stability

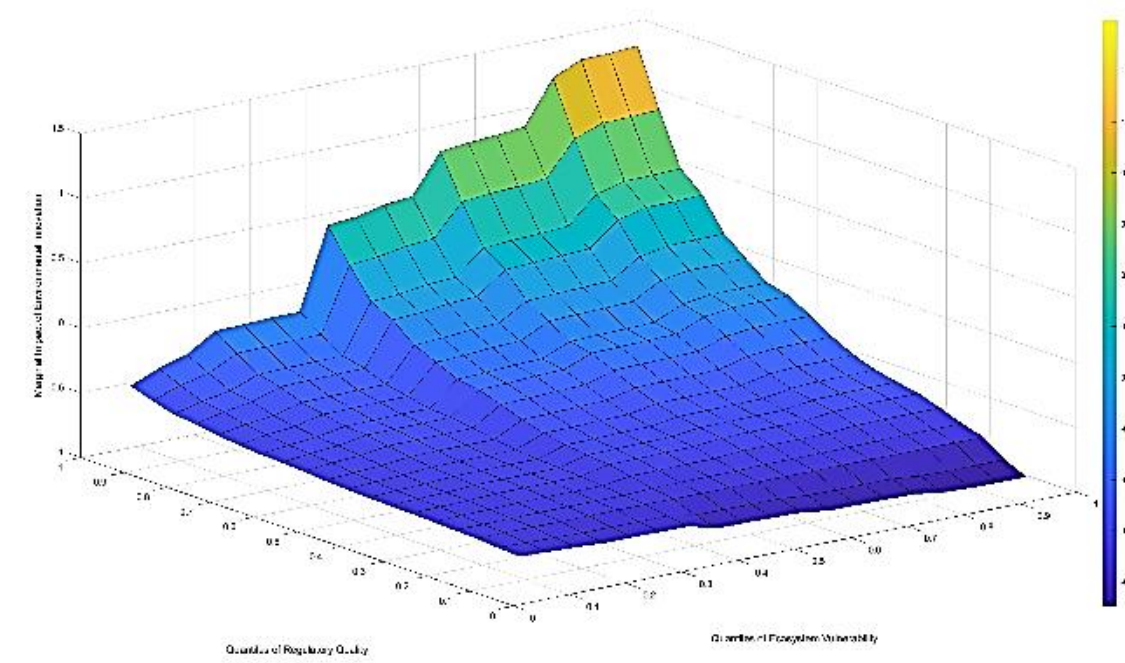

Governance Quality Indicator: Regulatory Quality

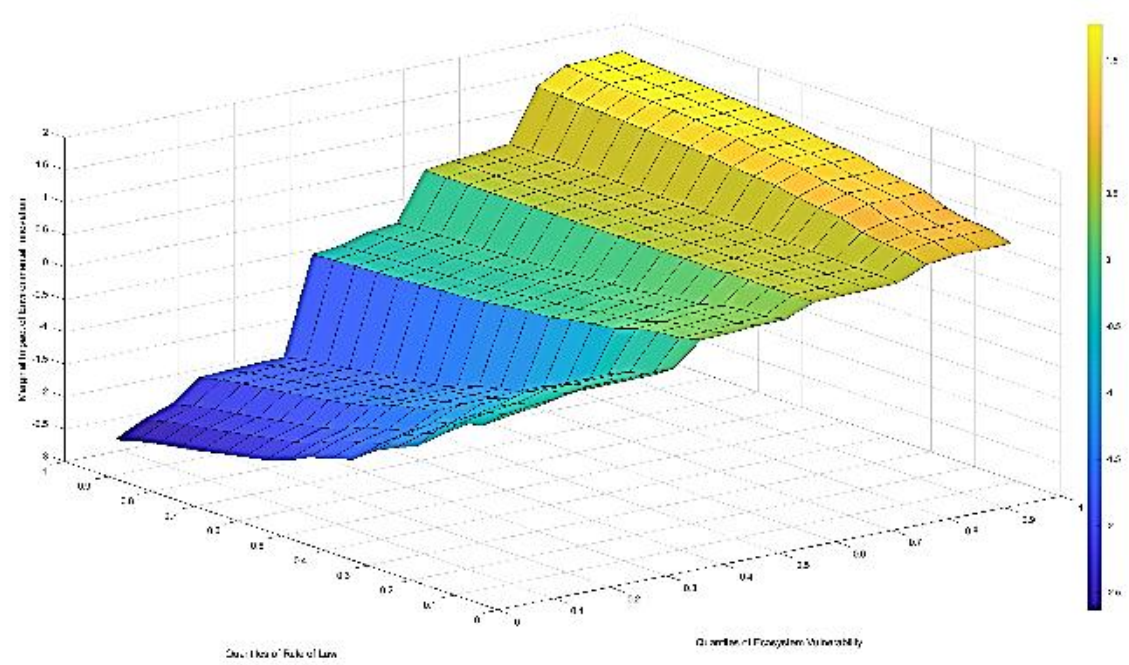

Governance Quality Indicator: Rule of Law

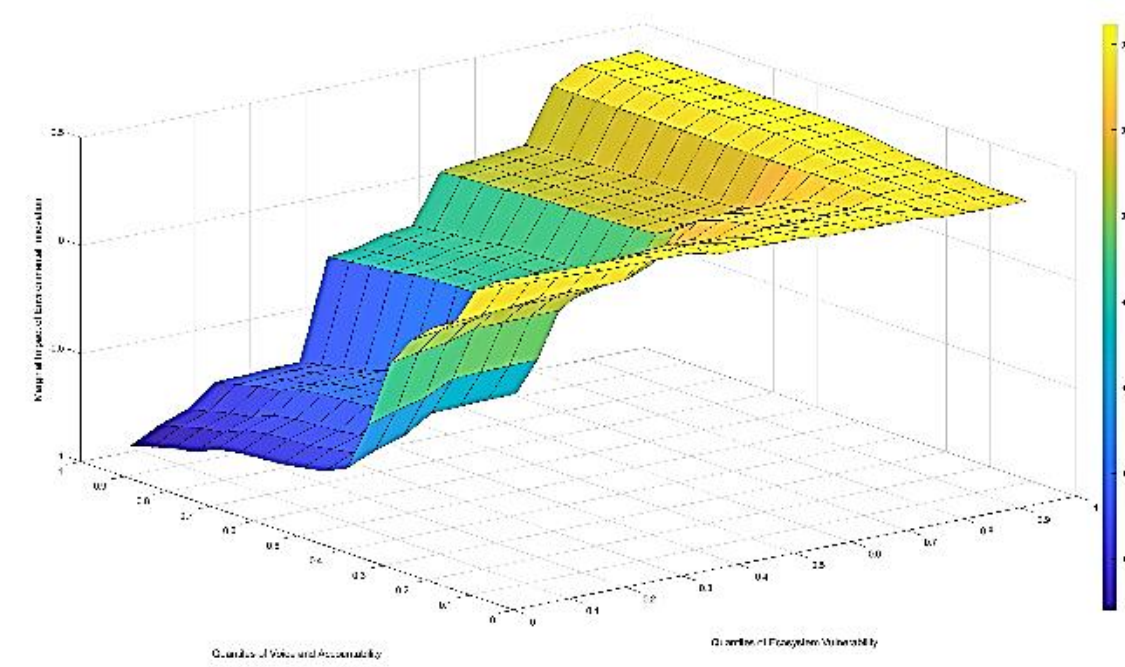

Governance Quality Indicator: Voice and Accountability

Figure S63: BRICS Policy Simulation Outcomes for the Marginal Impact of Environmental Innovation on SDG 7.2.1 under Ecosystem Vulnerability and Governance Quality, Related to Figure 4-5 and Table 2

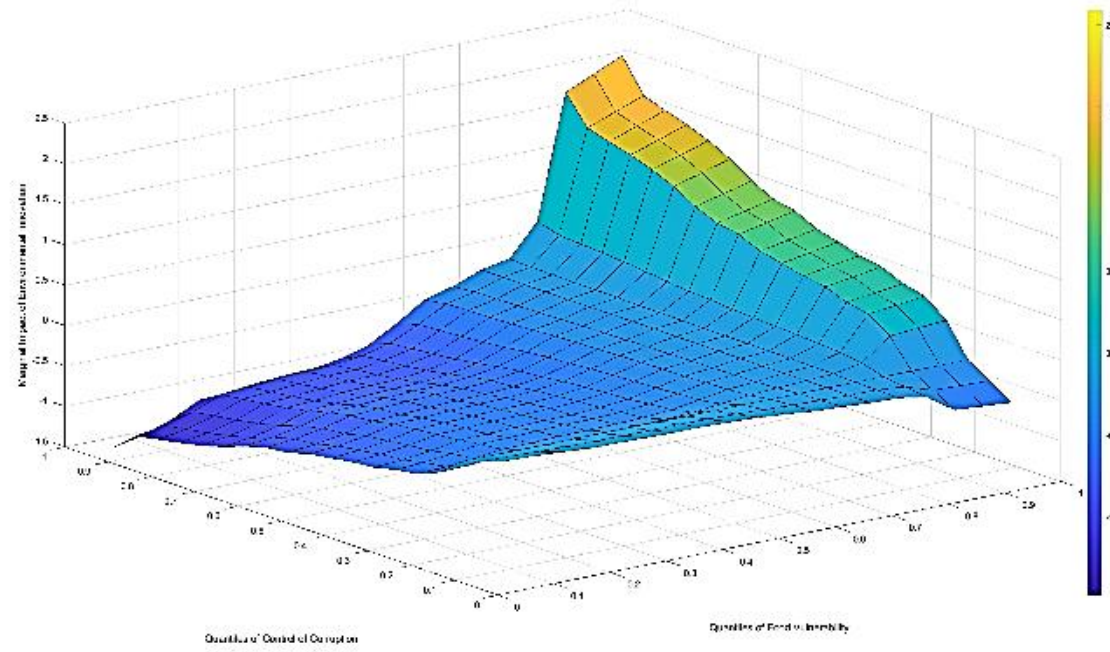

Governance Quality Indicator: Control of Corruption

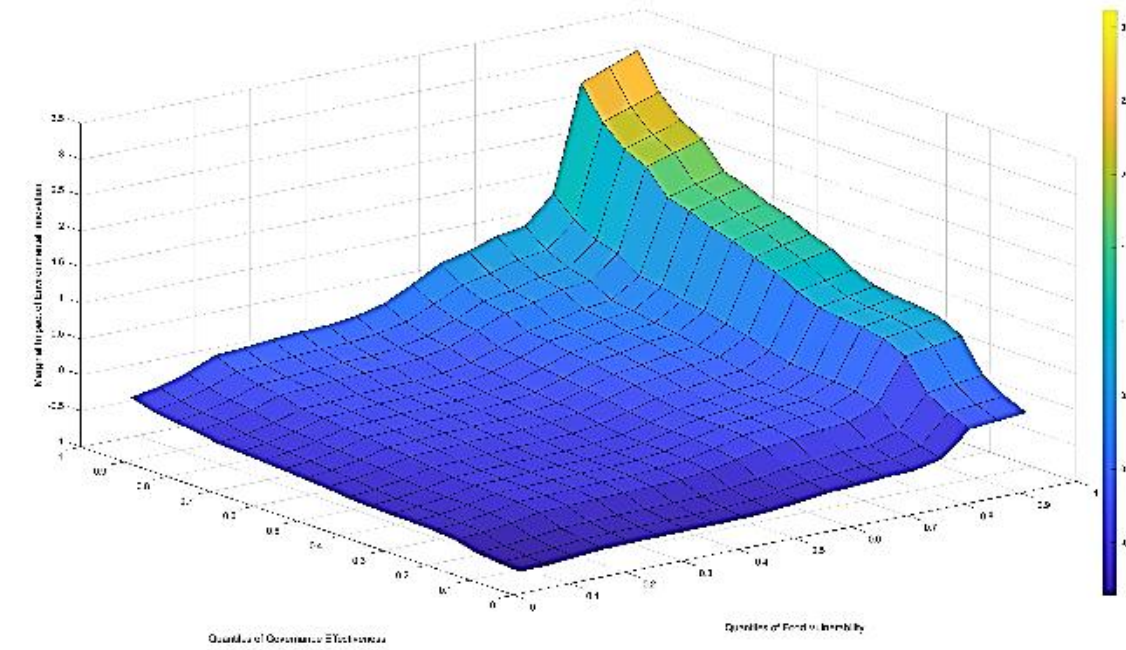

Governance Quality Indicator: Governance Effectiveness

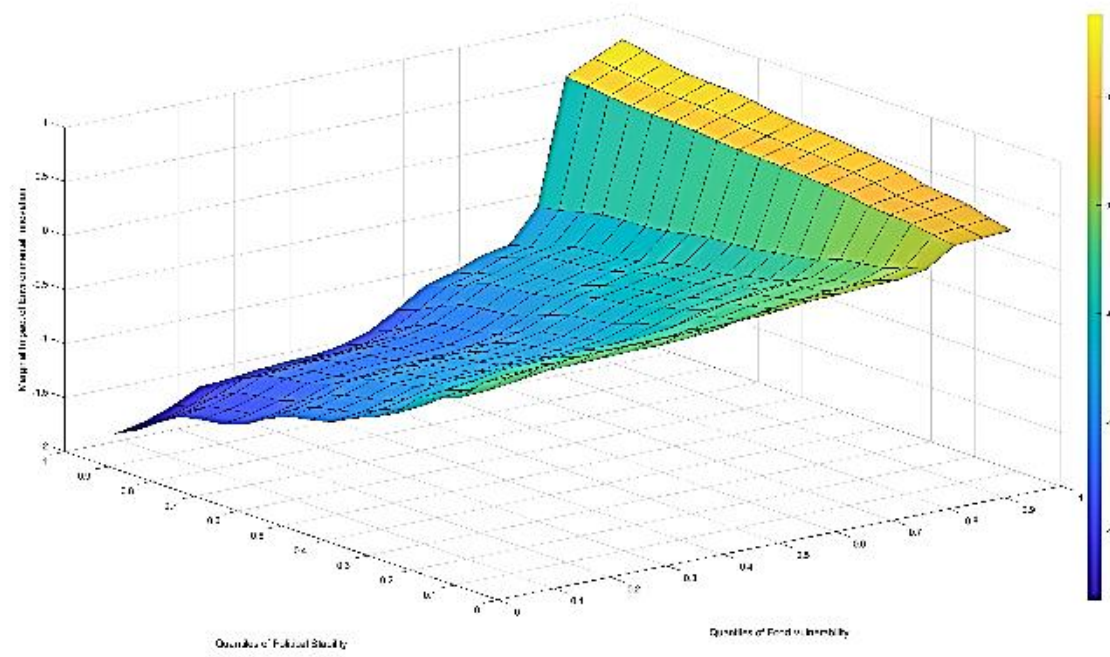

Governance Quality Indicator: Political Stability

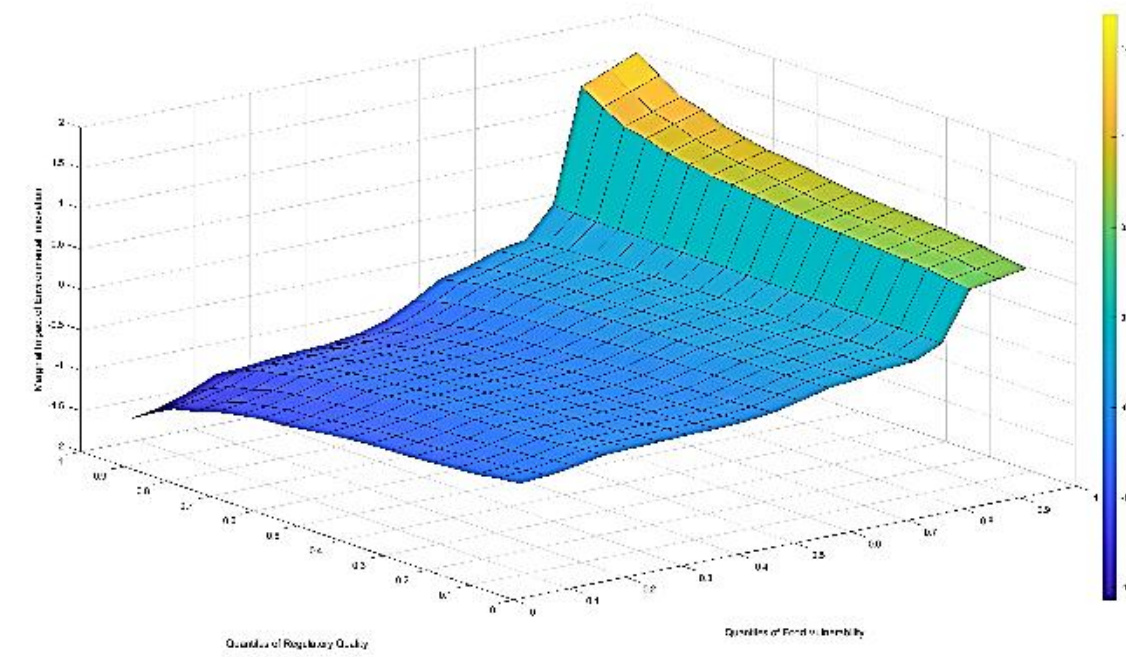

Governance Quality Indicator: Regulatory Quality

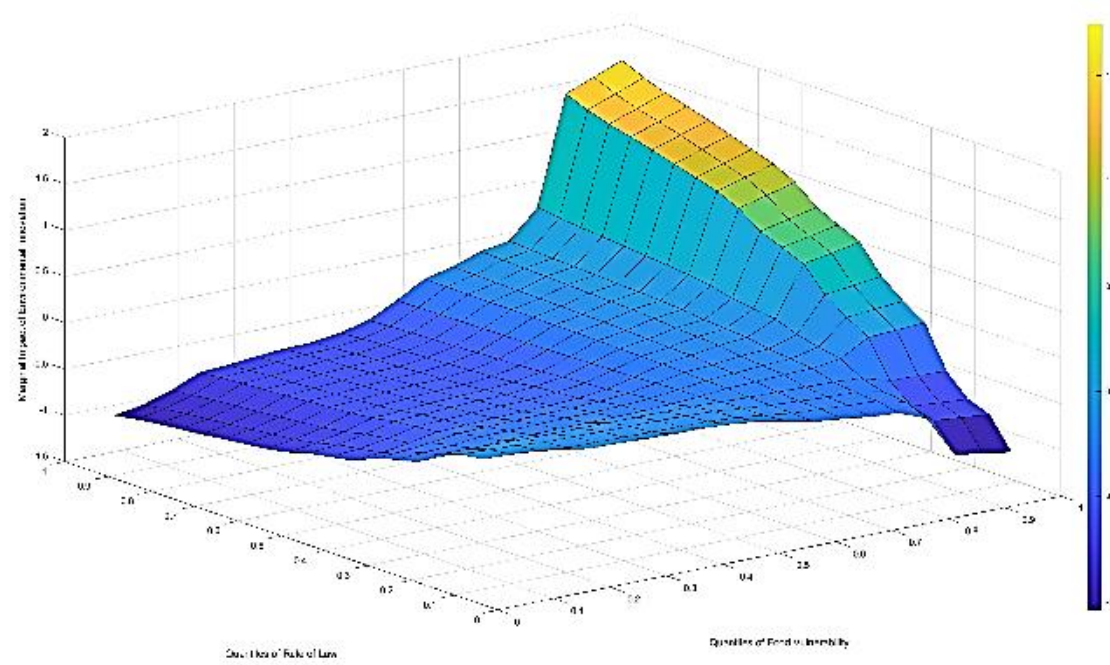

Governance Quality Indicator: Rule of Law

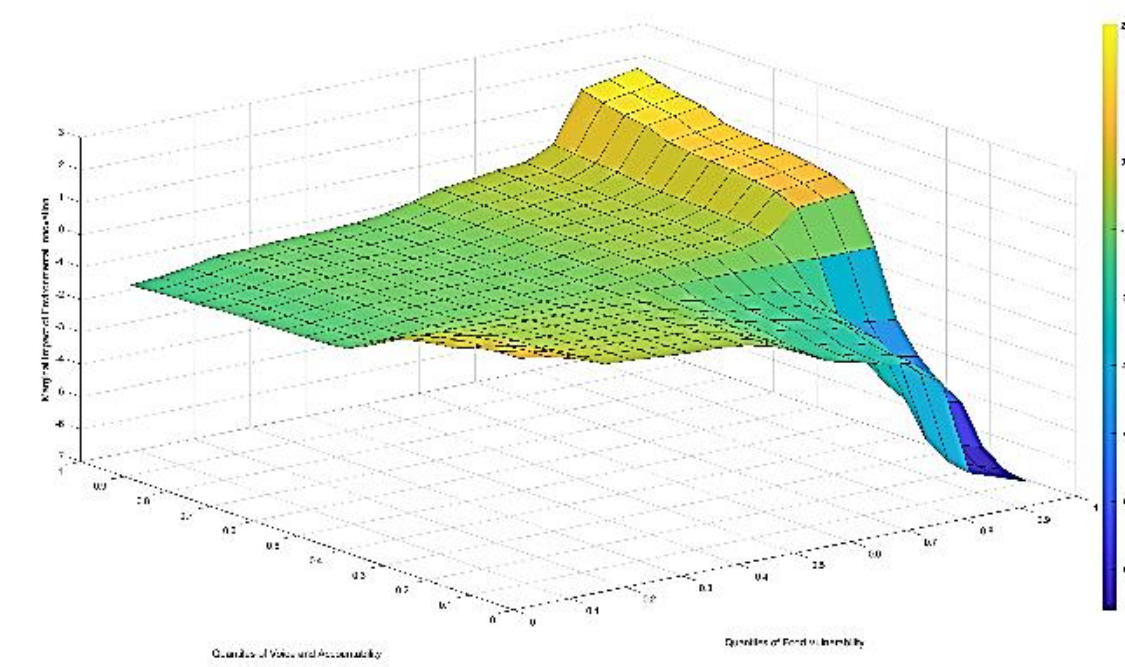

Governance Quality Indicator: Voice and Accountability

Figure S64: BRICS Policy Simulation Outcomes for the Marginal Impact of Environmental Innovation on SDG 7.2.1 under Food Vulnerability and Governance Quality, Related to Figure 4-5 and Table 2

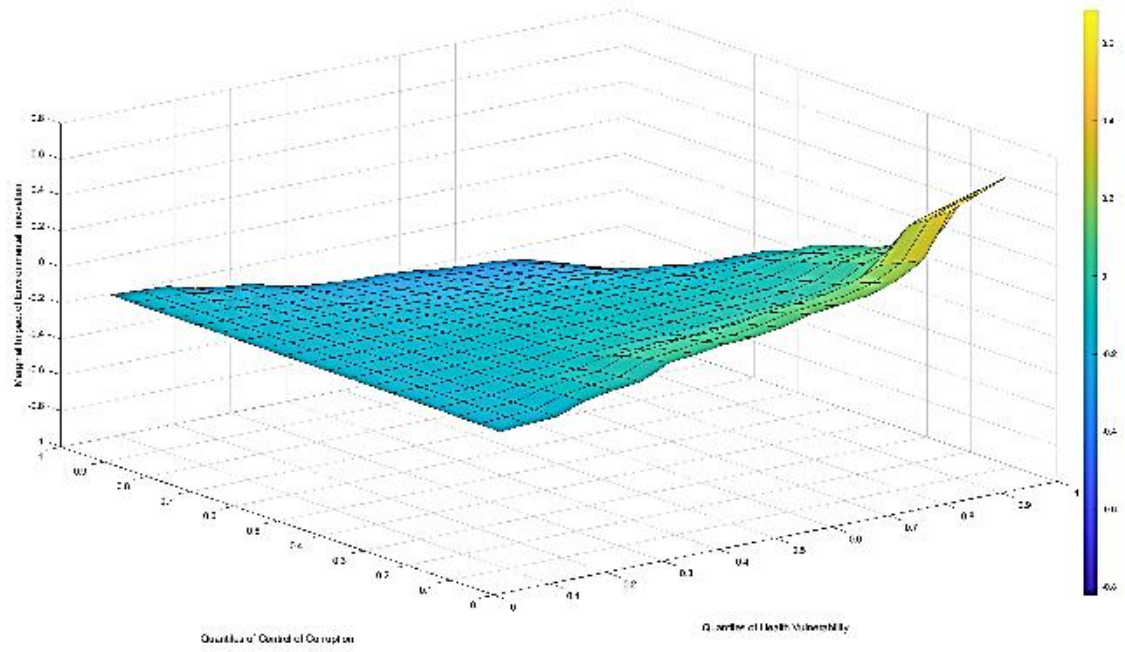

Governance Quality Indicator: Control of Corruption

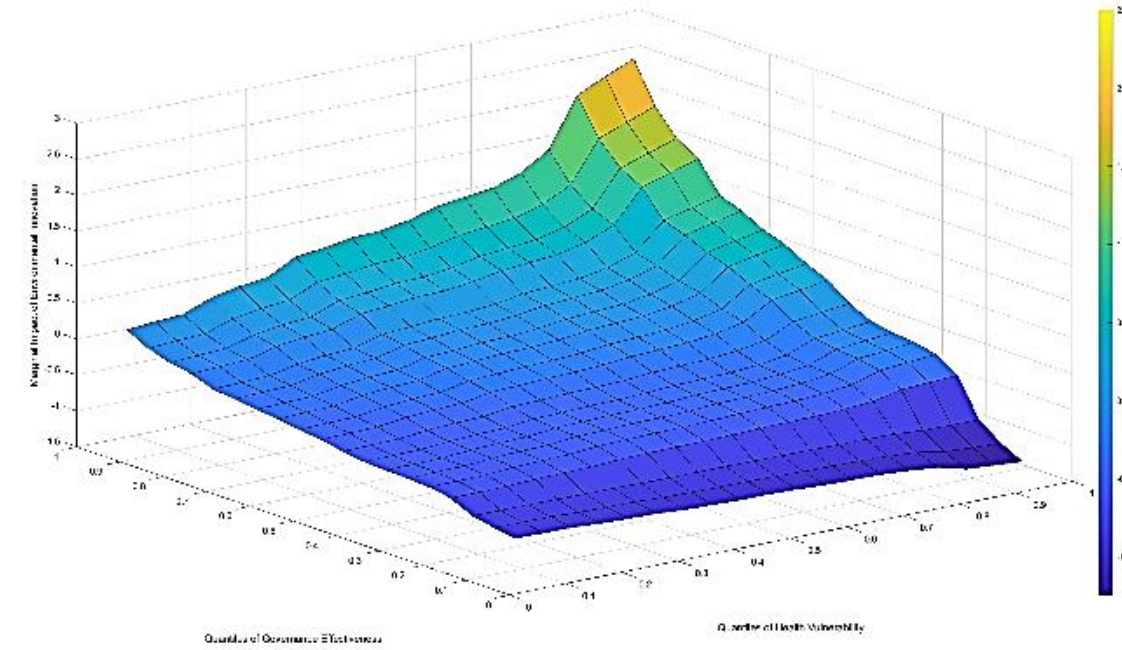

Governance Quality Indicator: Governance Effectiveness

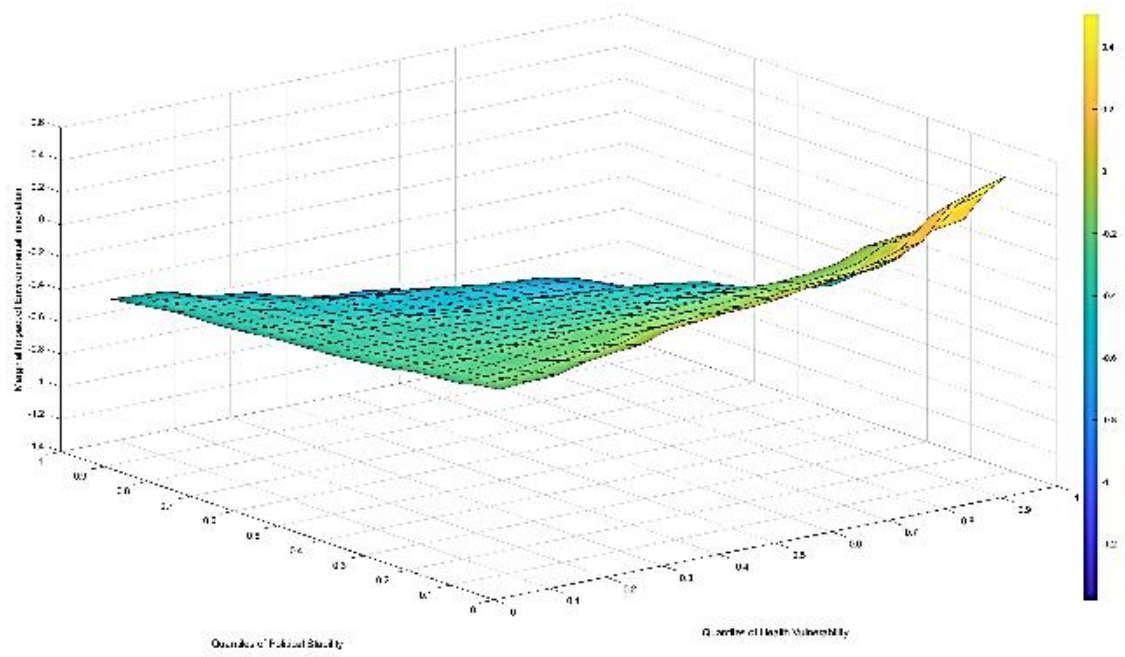

Governance Quality Indicator: Political Stability

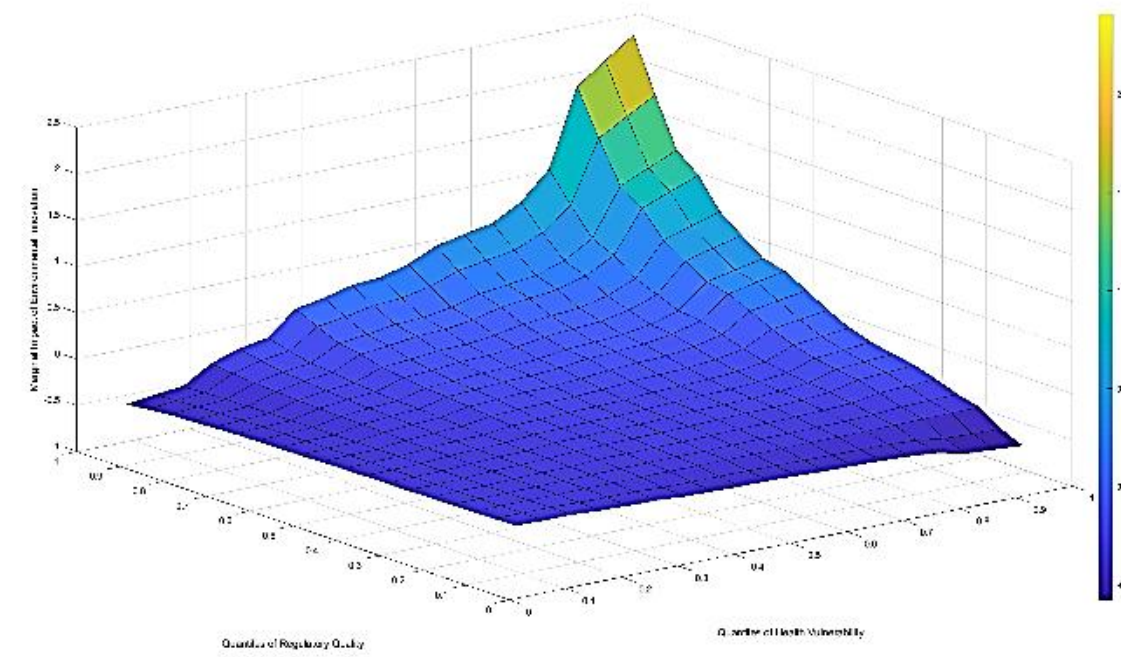

Governance Quality Indicator: Regulatory Quality

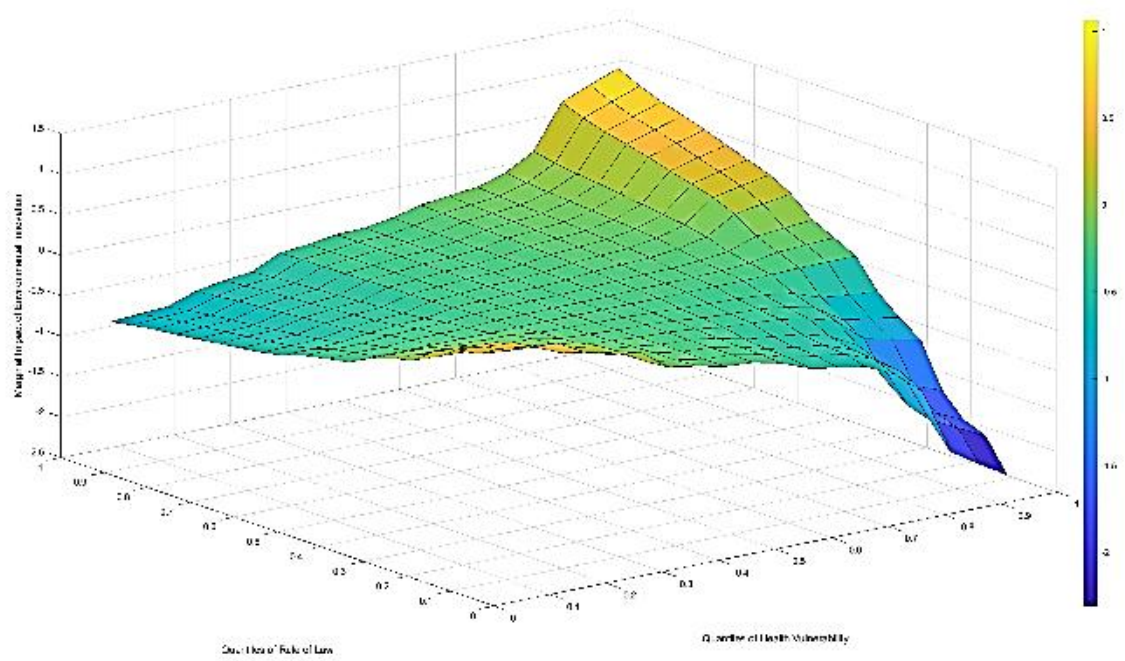

Governance Quality Indicator: Rule of Law

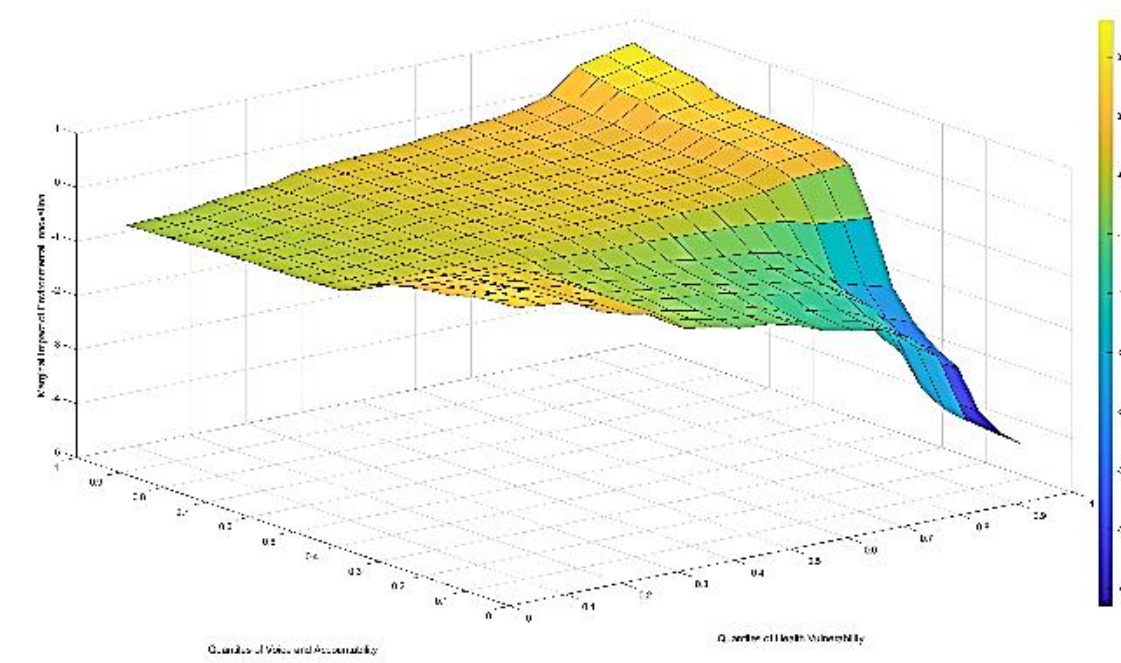

Governance Quality Indicator: Voice and Accountability

Figure S65: BRICS Policy Simulation Outcomes for the Marginal Impact of Environmental Innovation on SDG 7.2.1 under Health Vulnerability and Governance Quality, Related to Figure 4-5 and Table 2

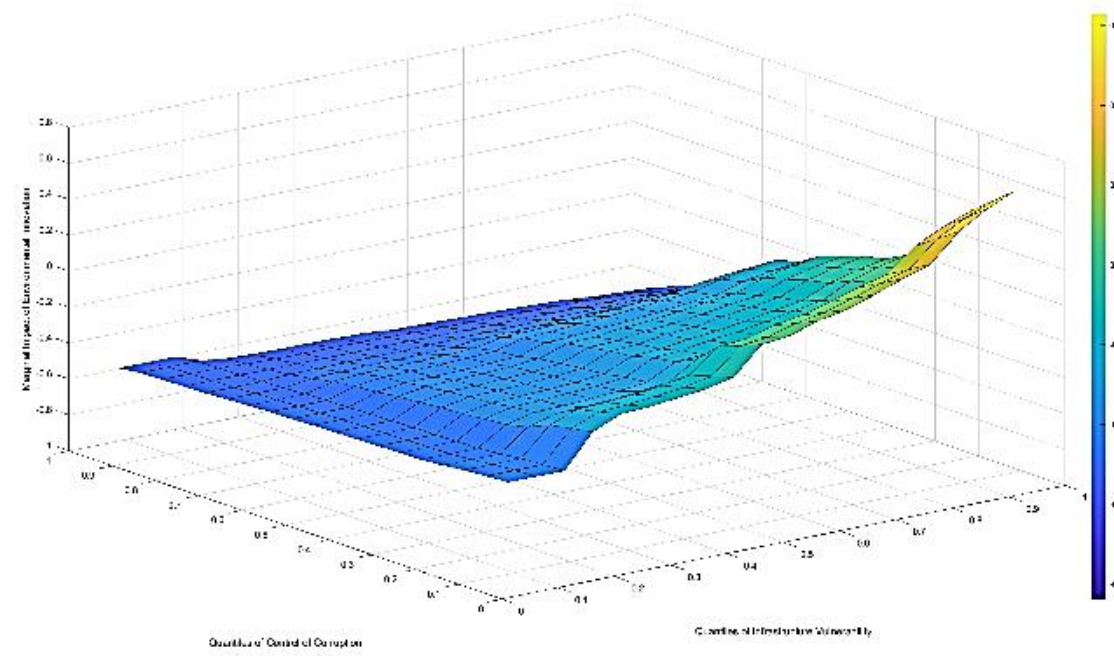

Governance Quality Indicator: Control of Corruption

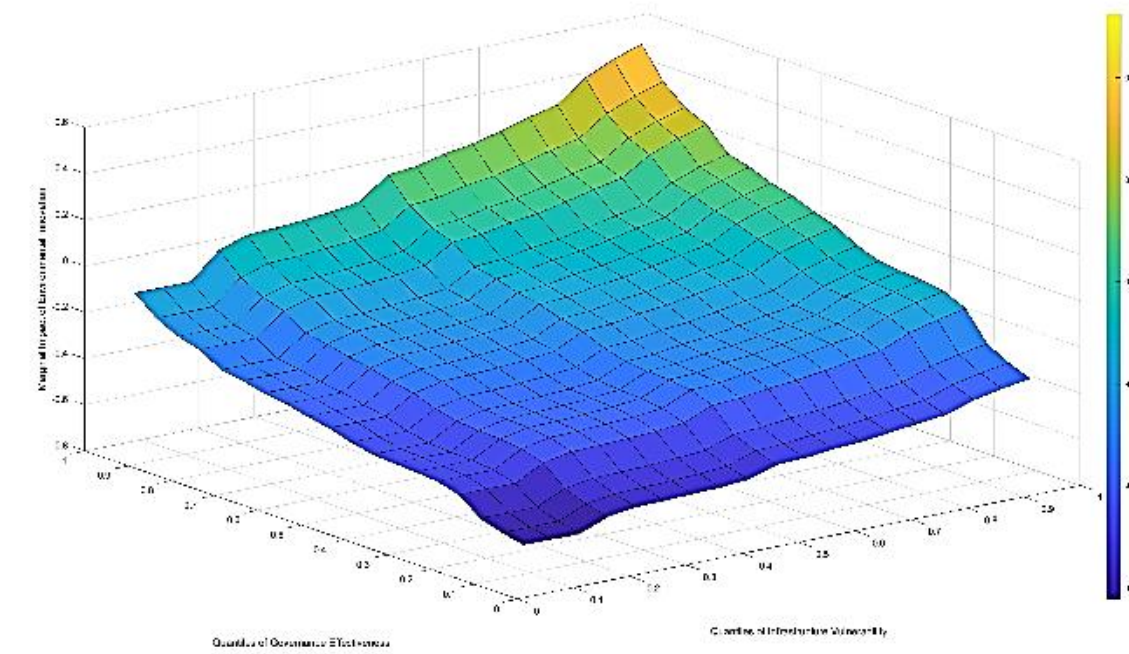

Governance Quality Indicator: Governance Effectiveness

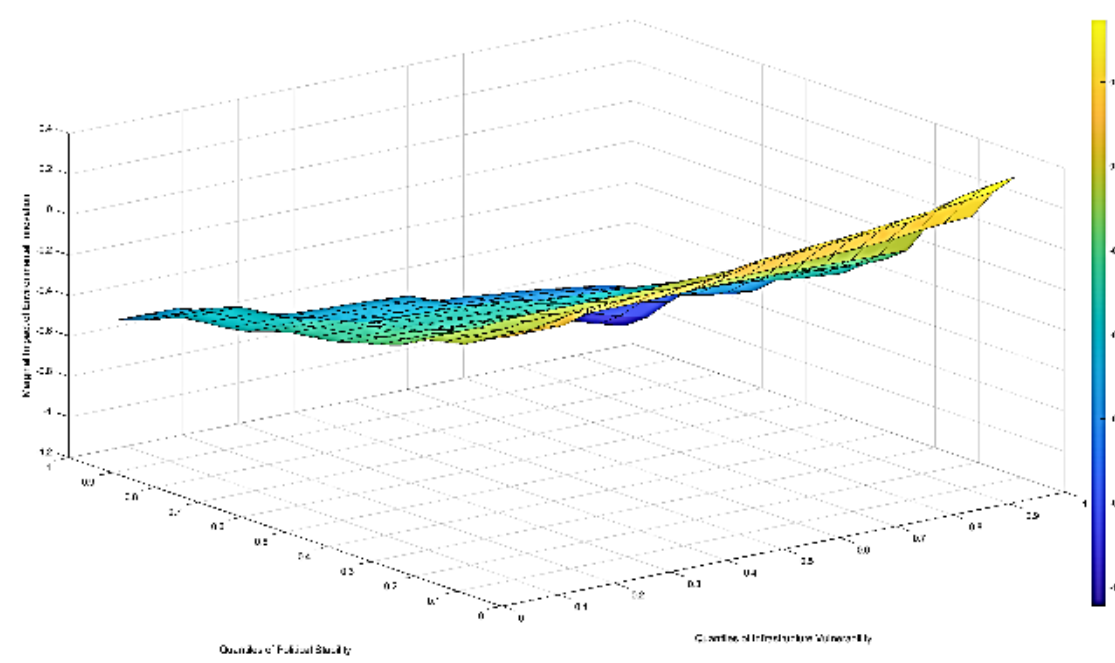

Governance Quality Indicator: Political Stability

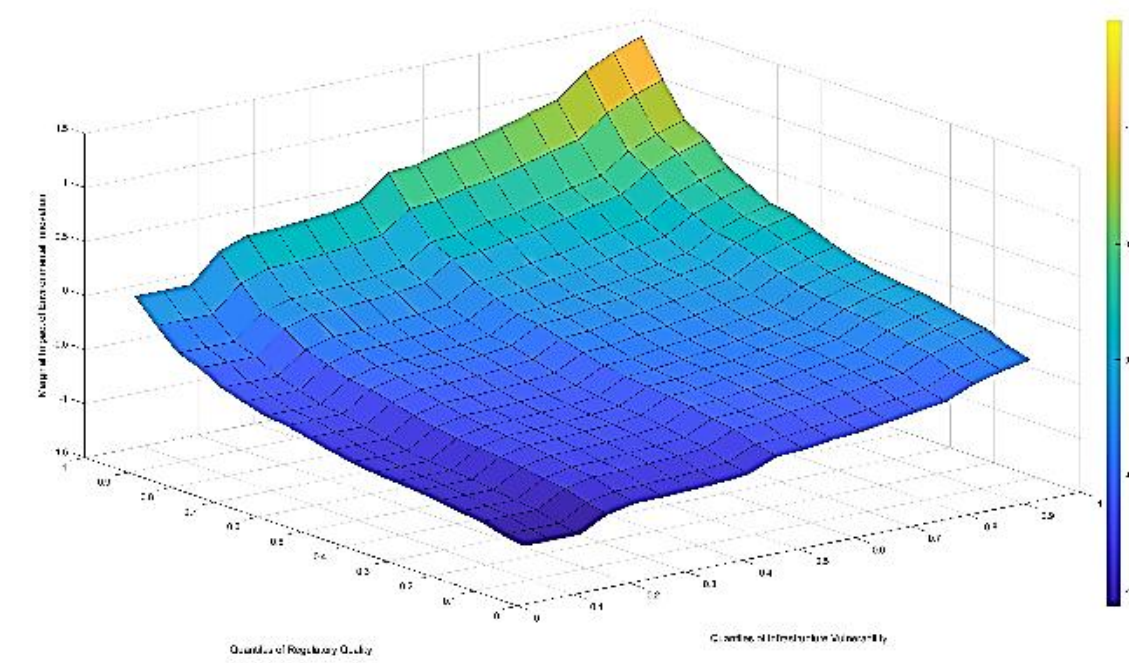

Governance Quality Indicator: Regulatory Quality

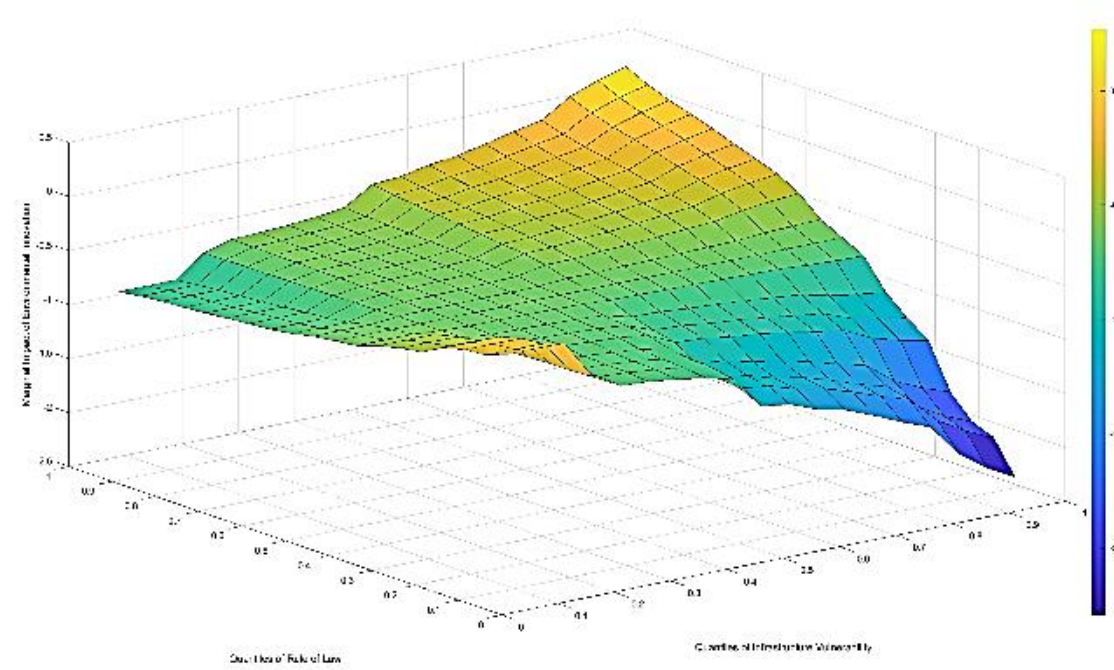

Governance Quality Indicator: Rule of Law

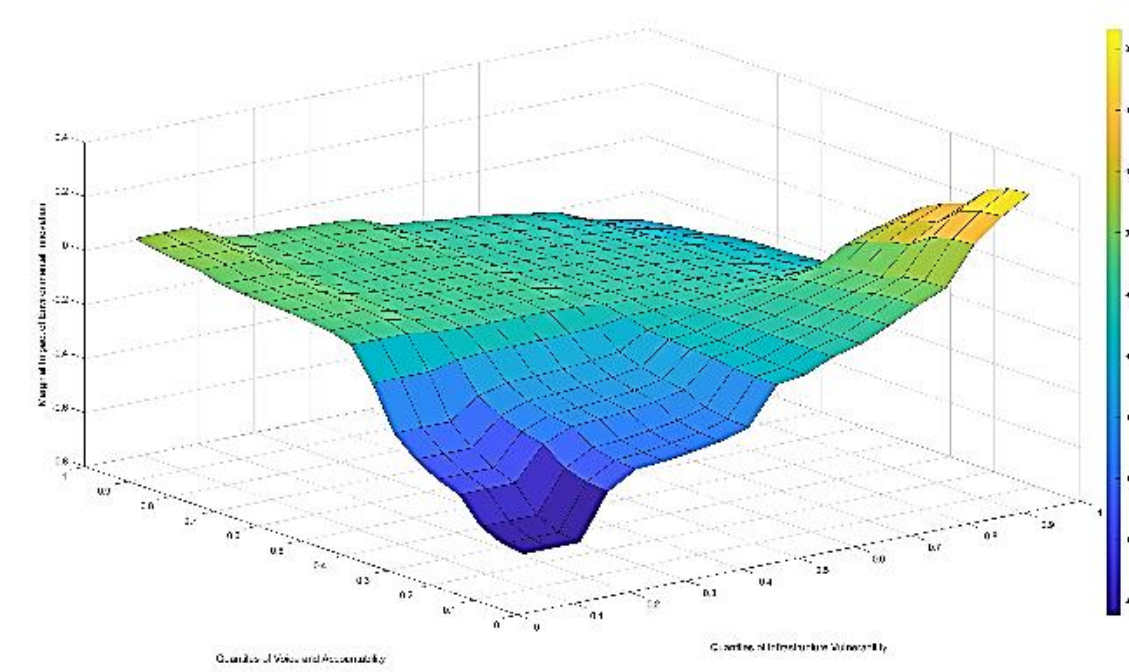

Governance Quality Indicator: Voice and Accountability

Figure S66: BRICS Policy Simulation Outcomes for the Marginal Impact of Environmental Innovation on SDG 7.2.1 under Infrastructure Vulnerability and Governance Quality, Related to Figure 4-5 and Table 2

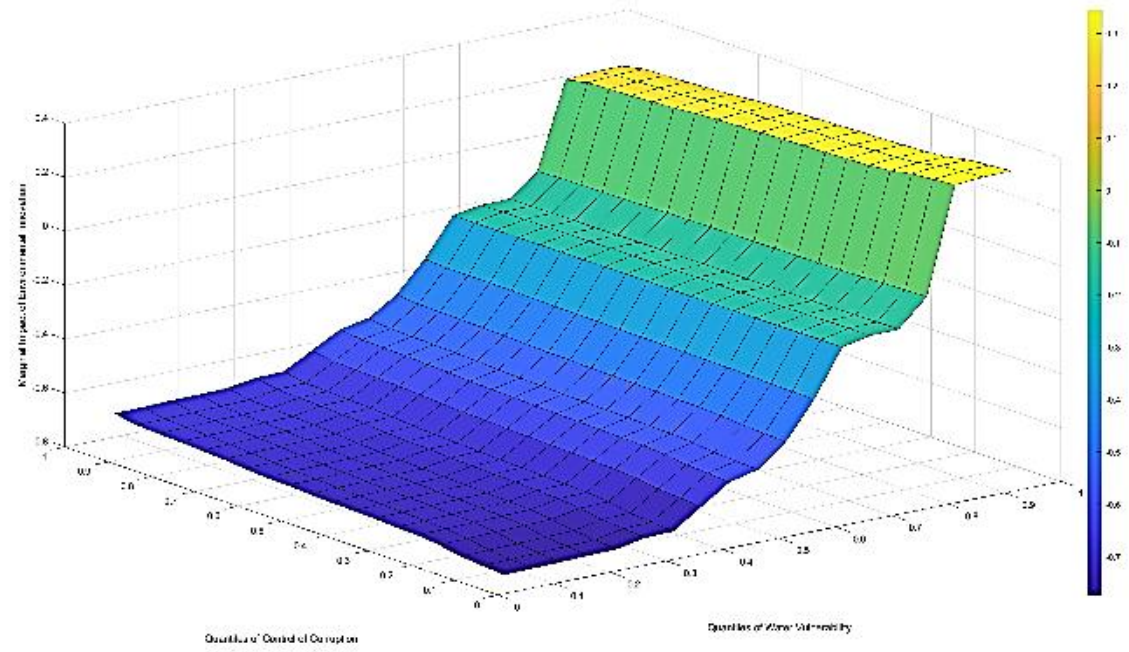

Governance Quality Indicator: Control of Corruption

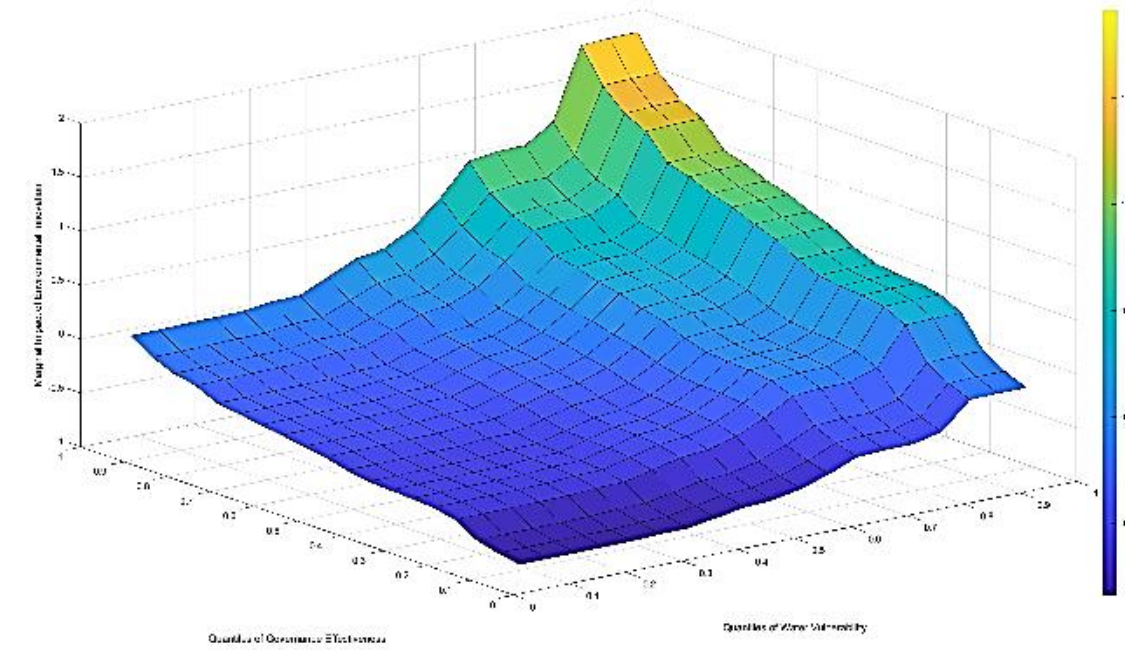

Governance Quality Indicator: Governance Effectiveness

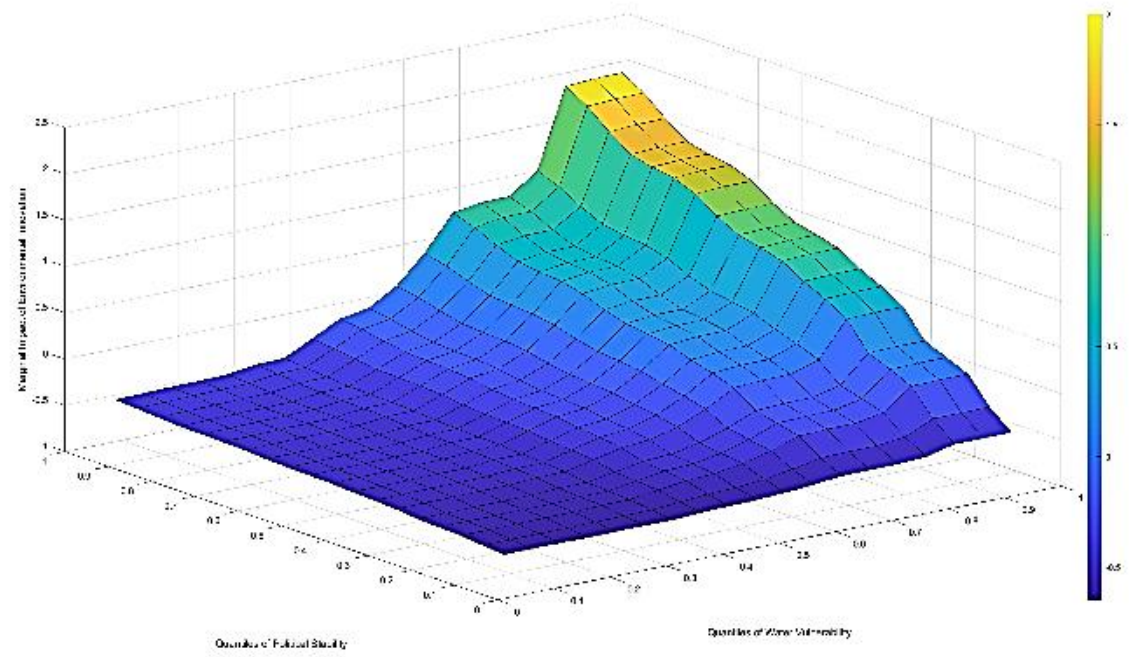

Governance Quality Indicator: Political Stability

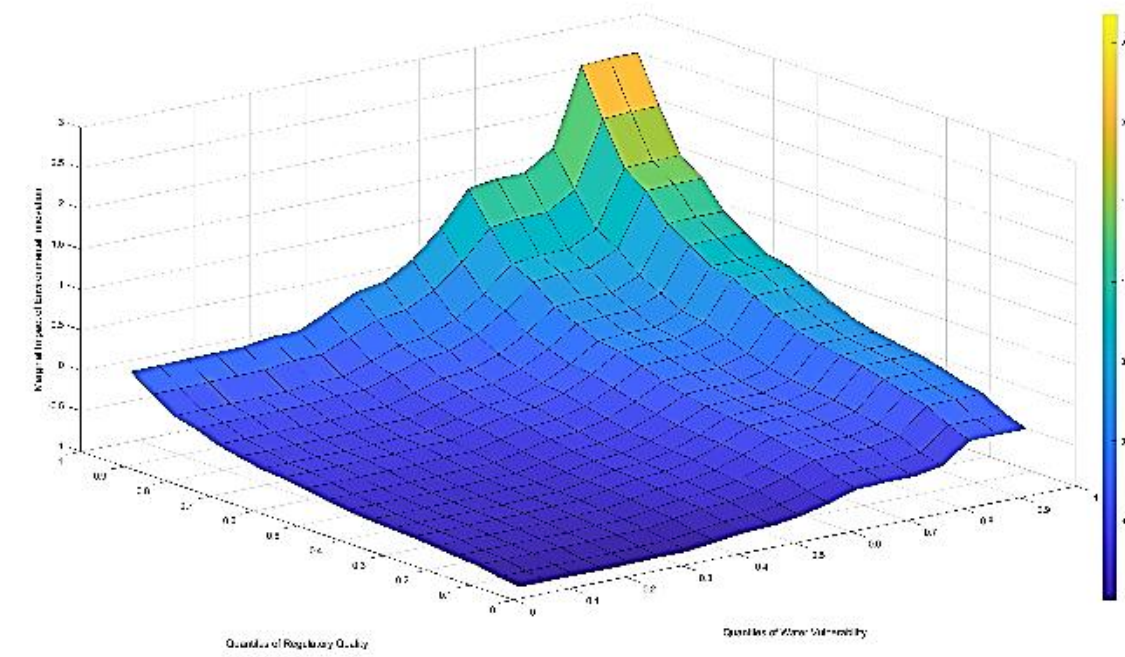

Governance Quality Indicator: Regulatory Quality

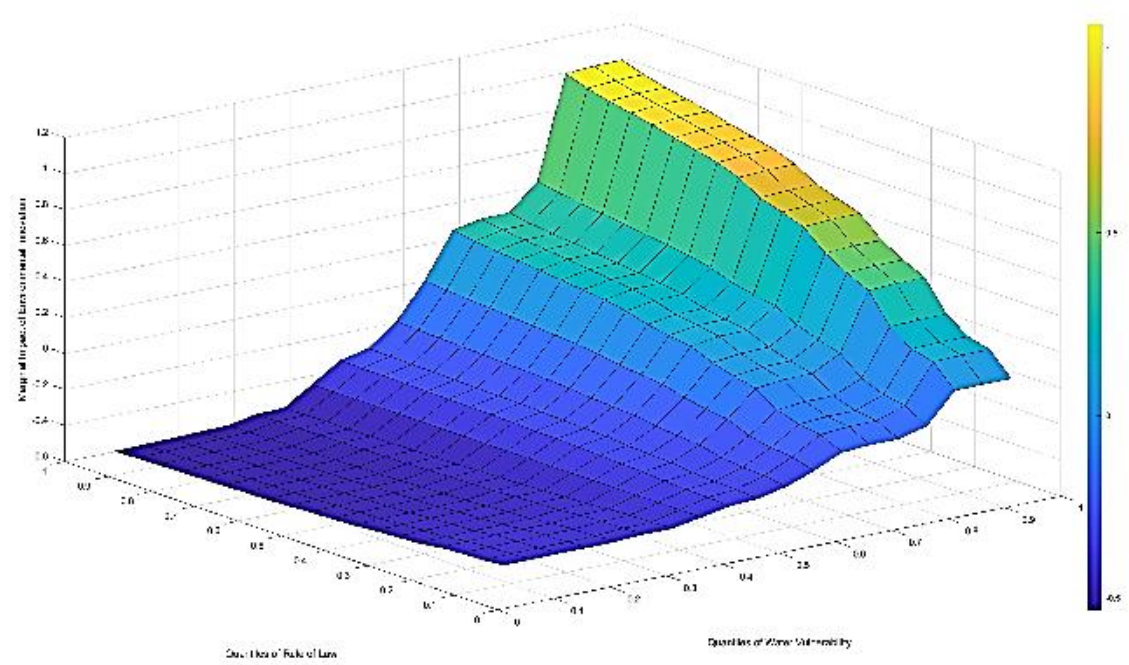

Governance Quality Indicator: Rule of Law

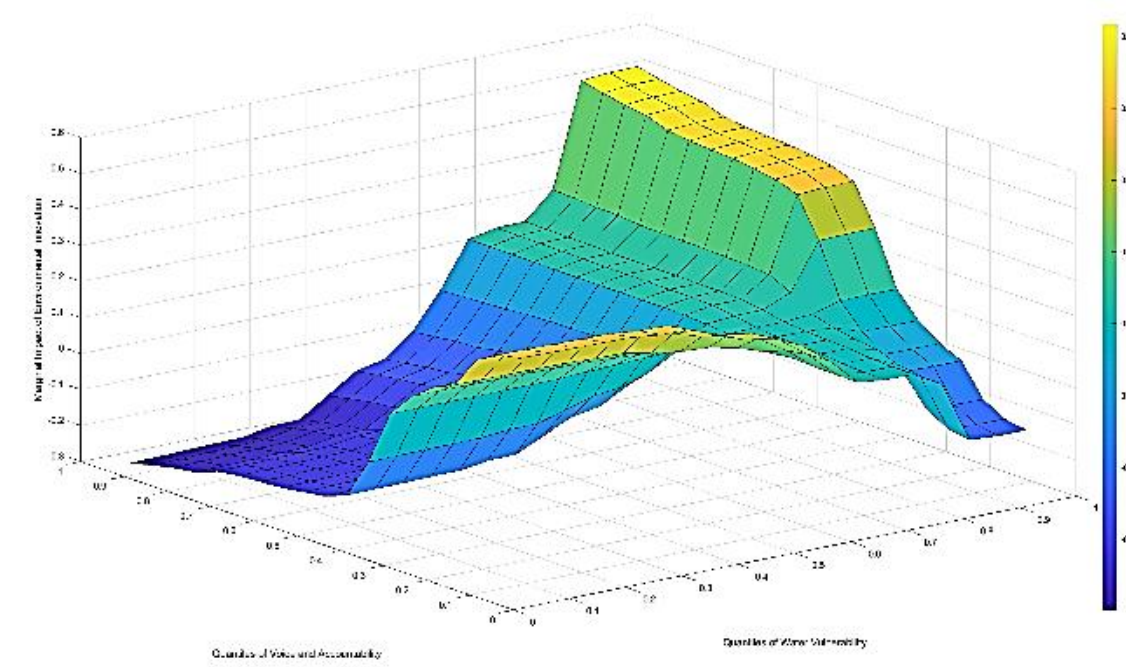

Governance Quality Indicator: Voice and Accountability

Figure S67: BRICS Policy Simulation Outcomes for the Marginal Impact of Environmental Innovation on SDG 7.2.1 under Water Vulnerability and Governance Quality, Related to Figure 4-5 and Table 2

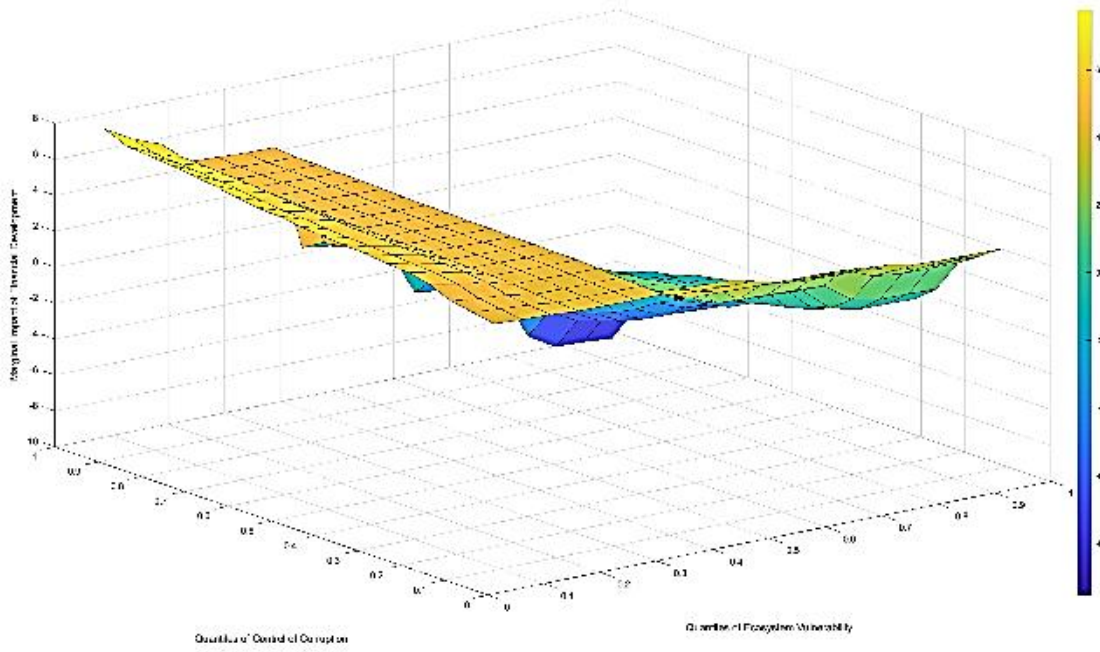

Governance Quality Indicator: Control of Corruption

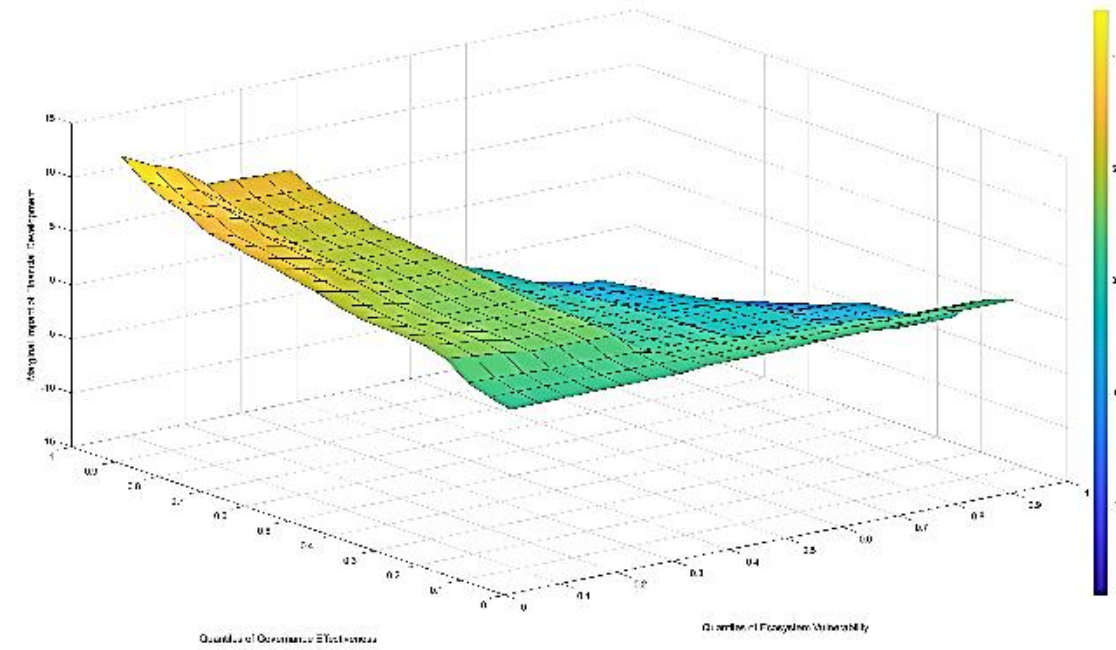

Governance Quality Indicator: Governance Effectiveness

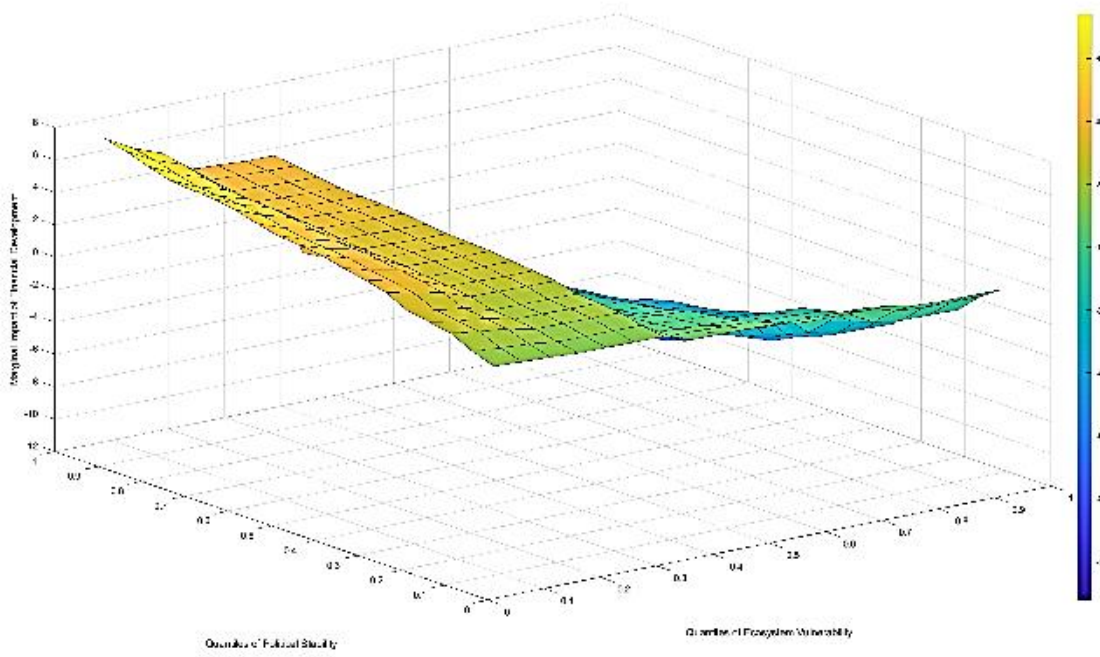

Governance Quality Indicator: Political Stability

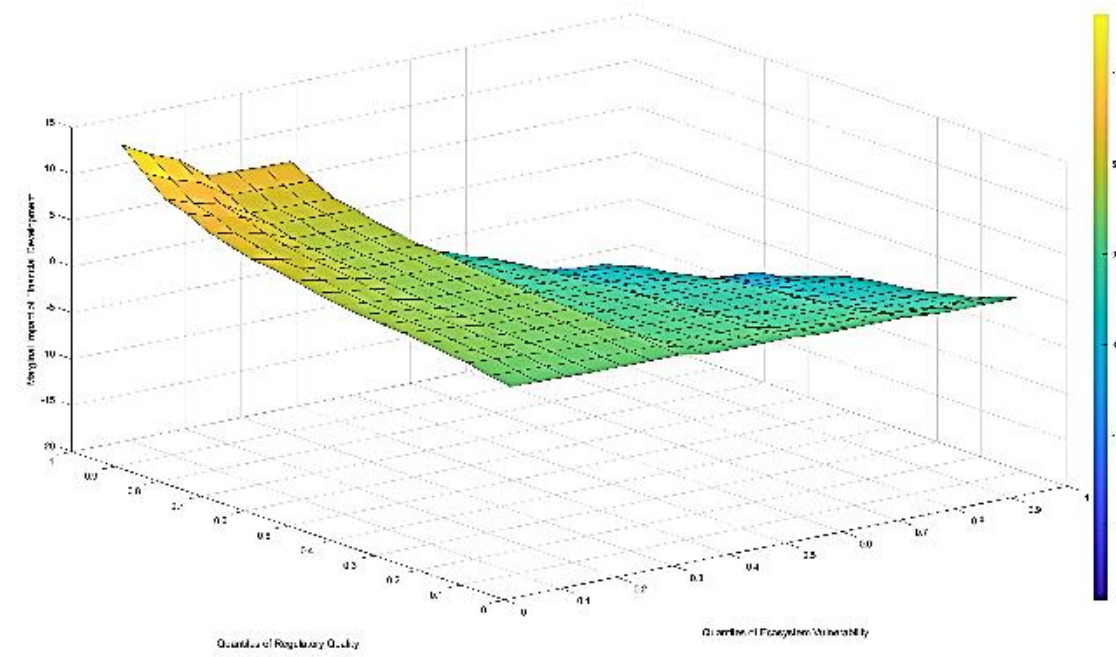

Governance Quality Indicator: Regulatory Quality

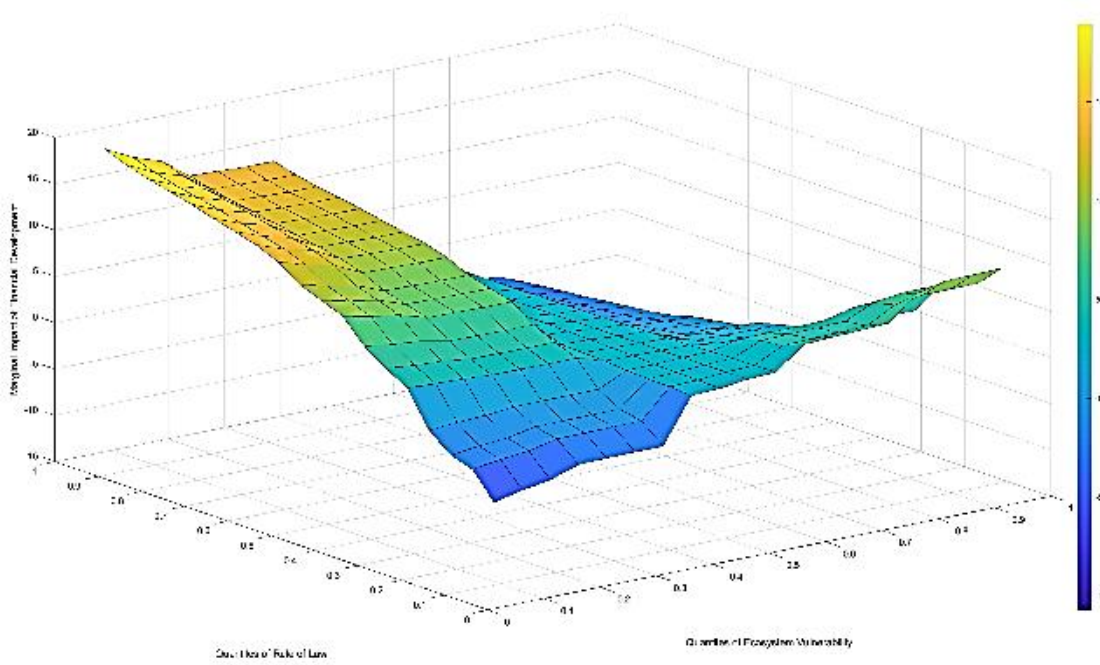

Governance Quality Indicator: Rule of Law

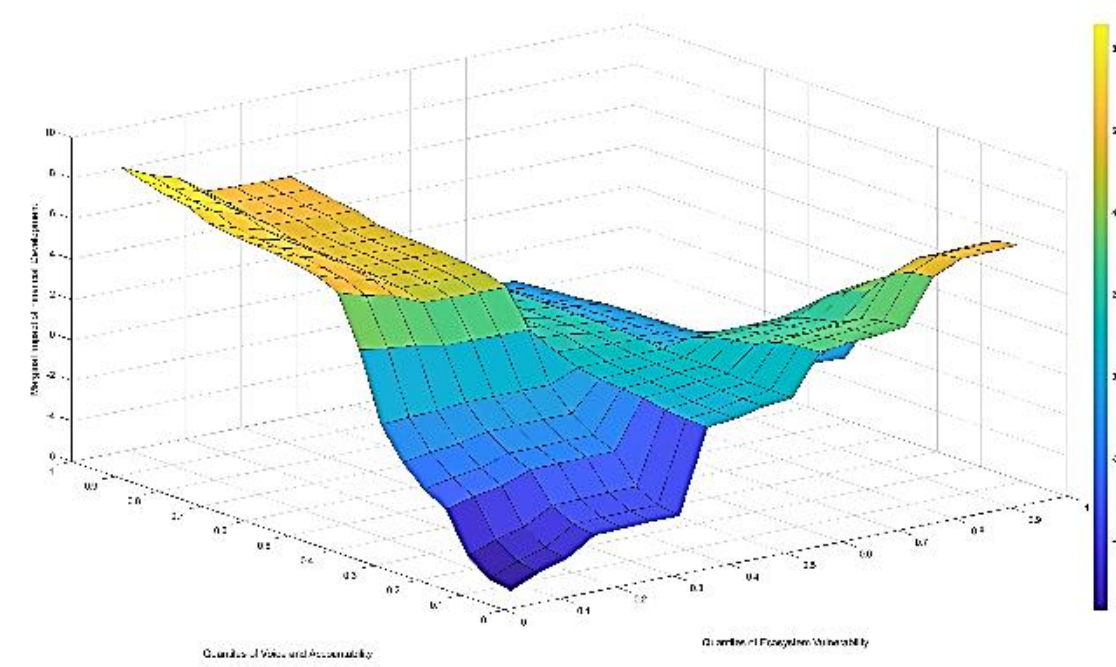

Governance Quality Indicator: Voice and Accountability

Figure S68: BRICS Policy Simulation Outcomes for the Marginal Impact of Financial Development on SDG 7.2.1 under Ecosystem Vulnerability and Governance Quality, Related to Figure 4-5 and Table 2

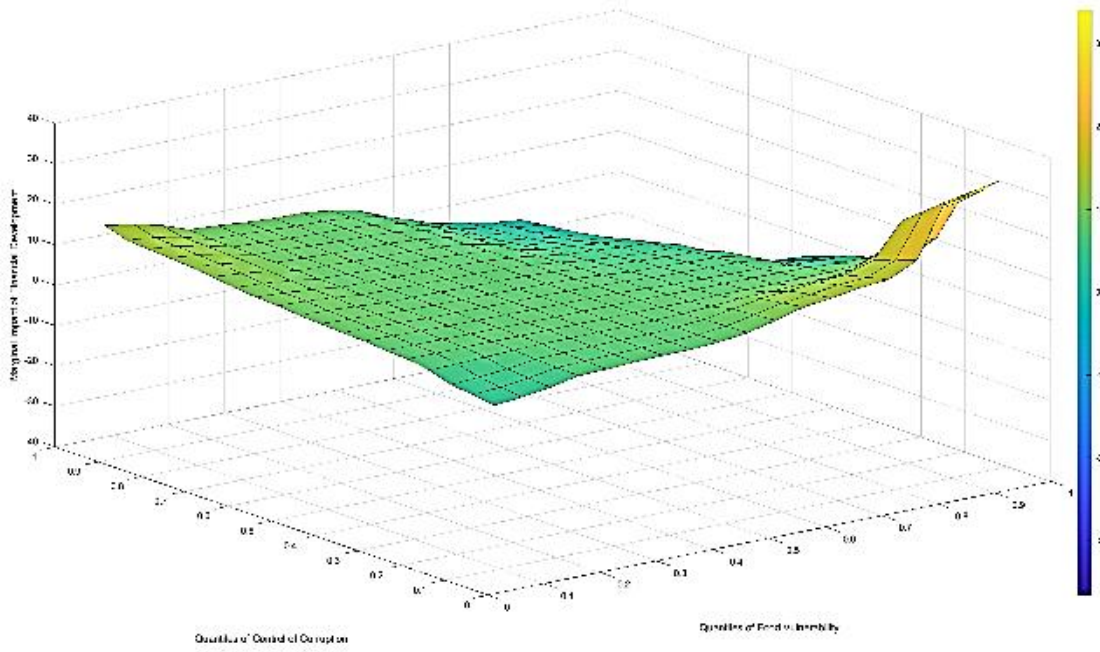

Governance Quality Indicator: Control of Corruption

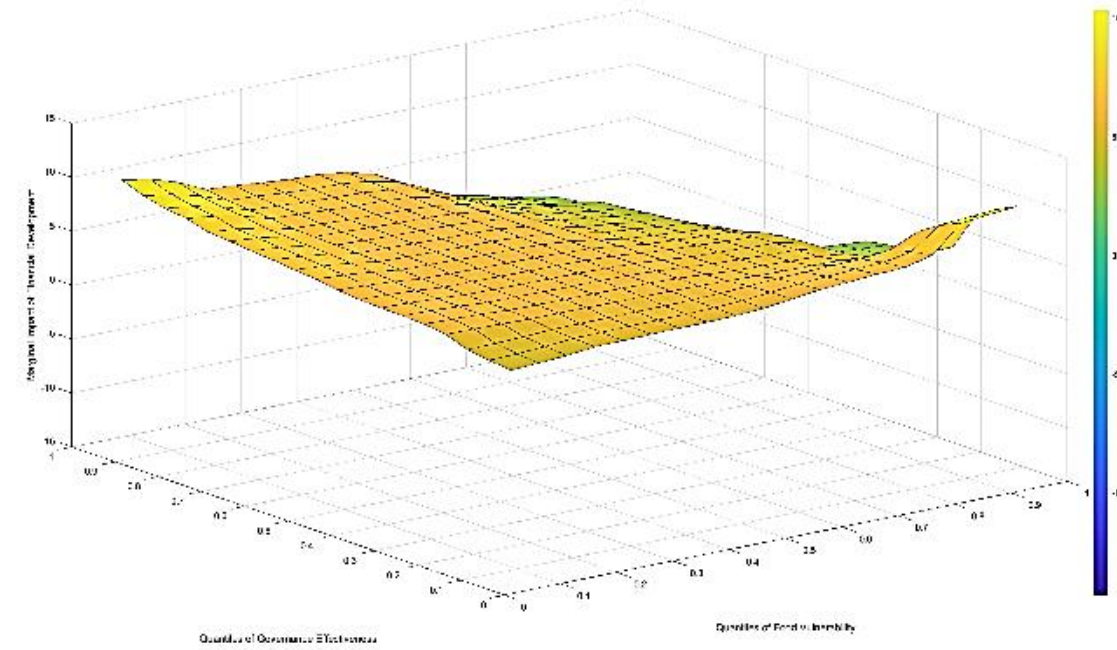

Governance Quality Indicator: Governance Effectiveness

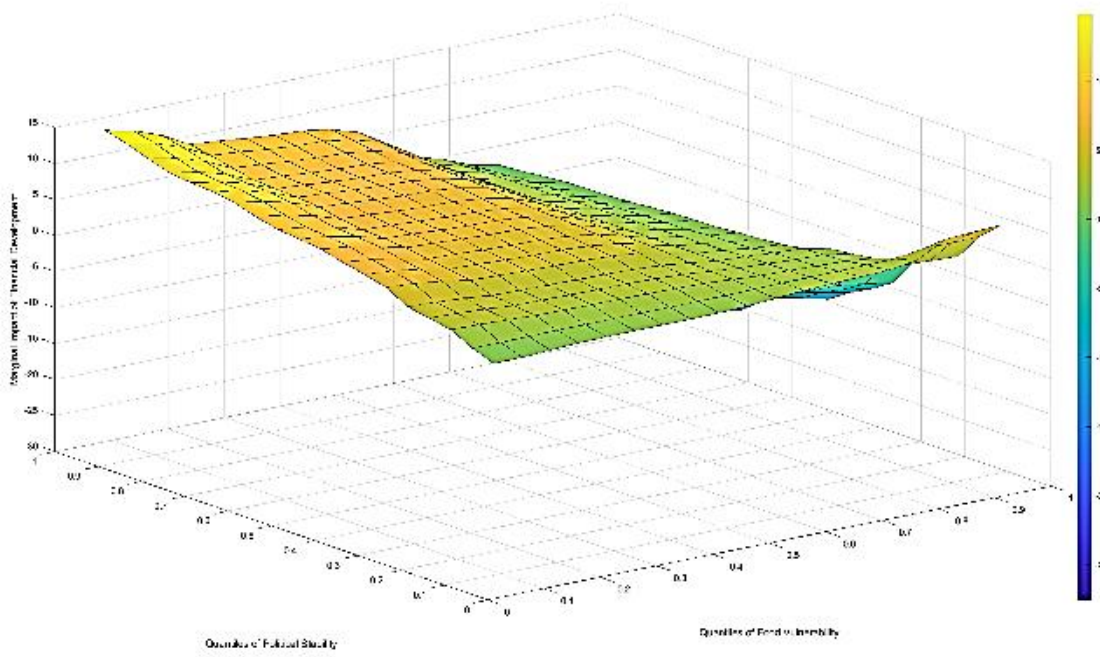

Governance Quality Indicator: Political Stability

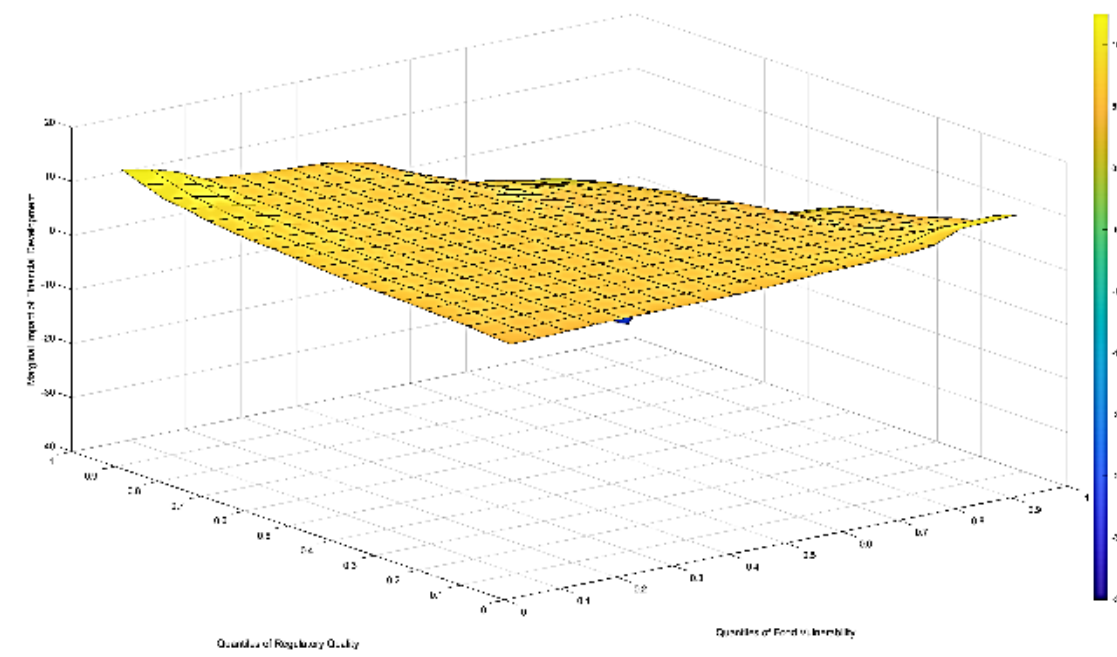

Governance Quality Indicator: Regulatory Quality

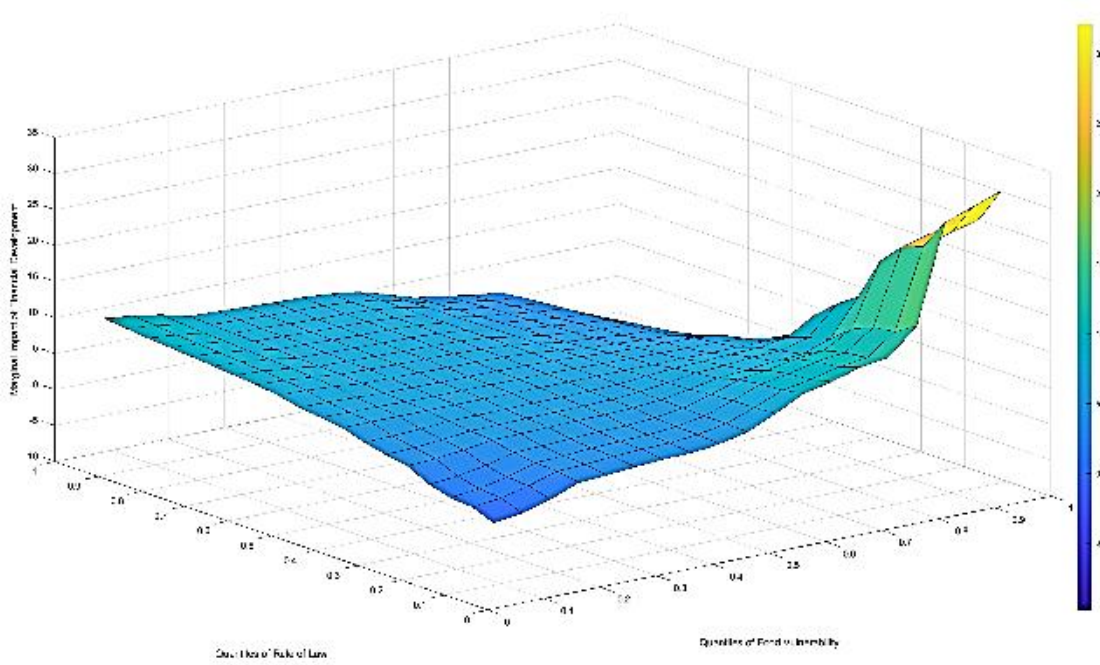

Governance Quality Indicator: Rule of Law

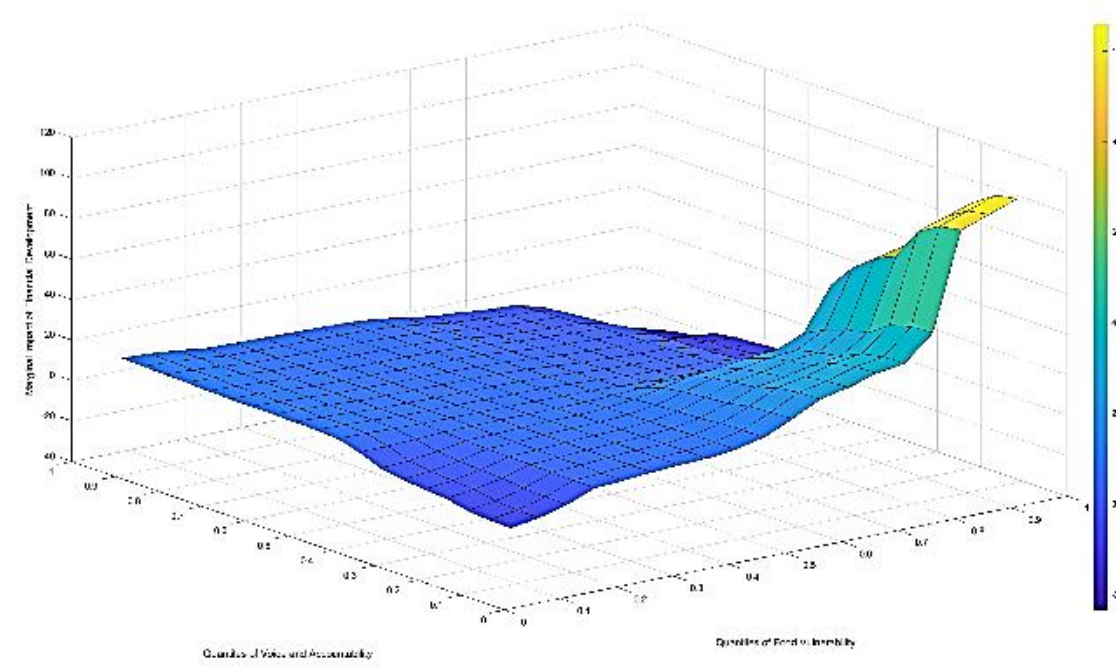

Governance Quality Indicator: Voice and Accountability

Figure S69: BRICS Policy Simulation Outcomes for the Marginal Impact of Financial Development on SDG 7.2.1 under Food Vulnerability and Governance Quality, Related to Figure 4-5 and Table 2

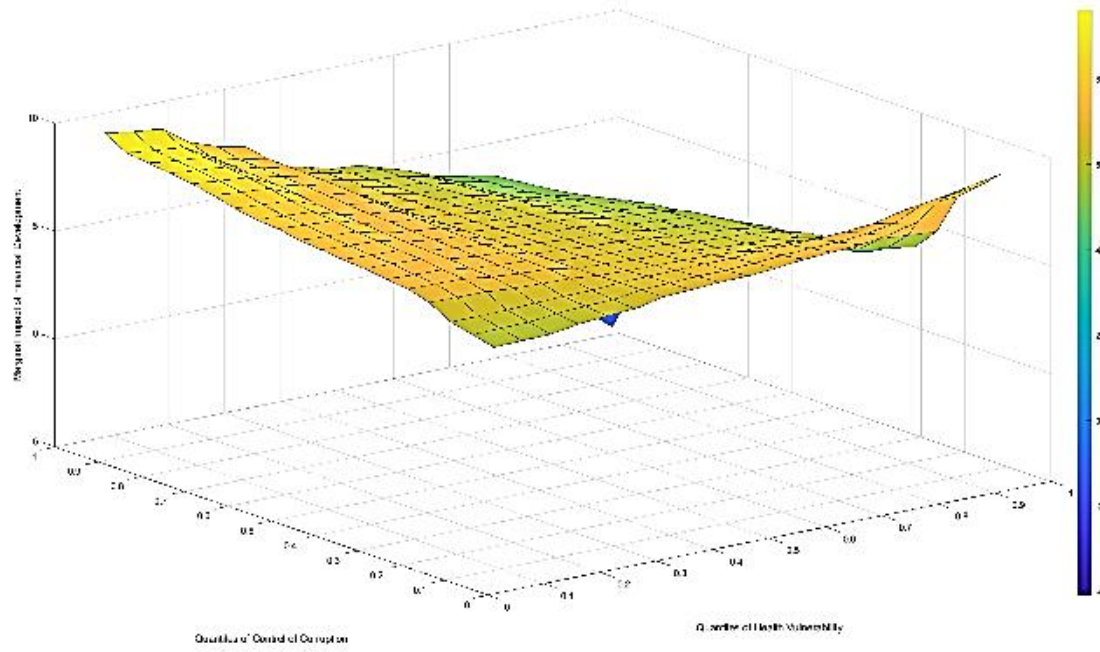

Governance Quality Indicator: Control of Corruption

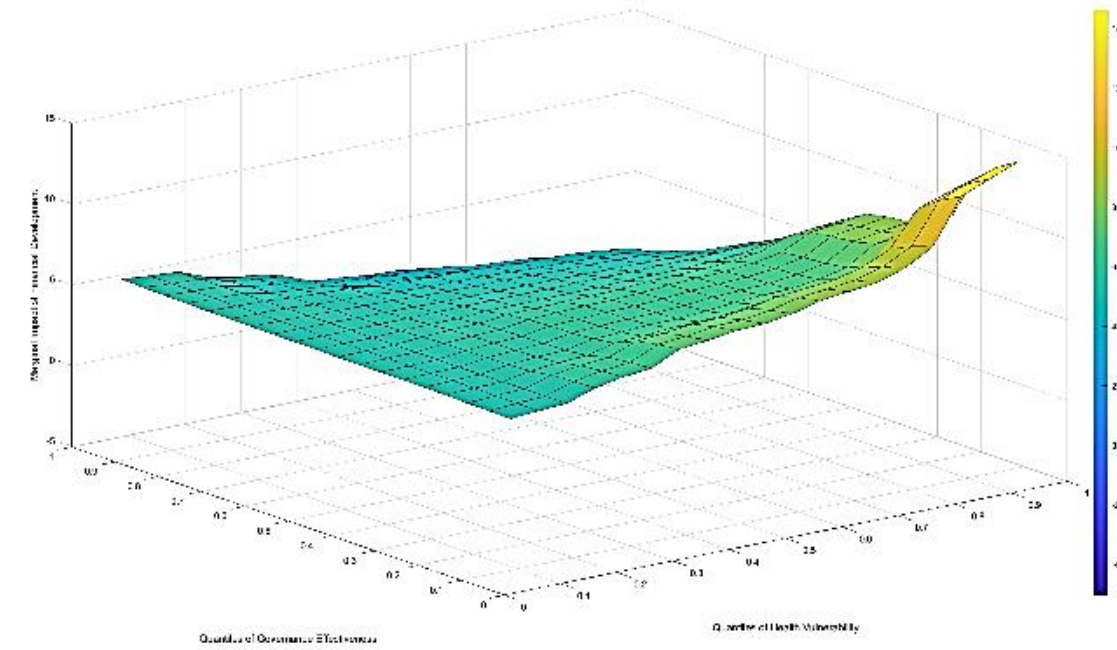

Governance Quality Indicator: Governance Effectiveness

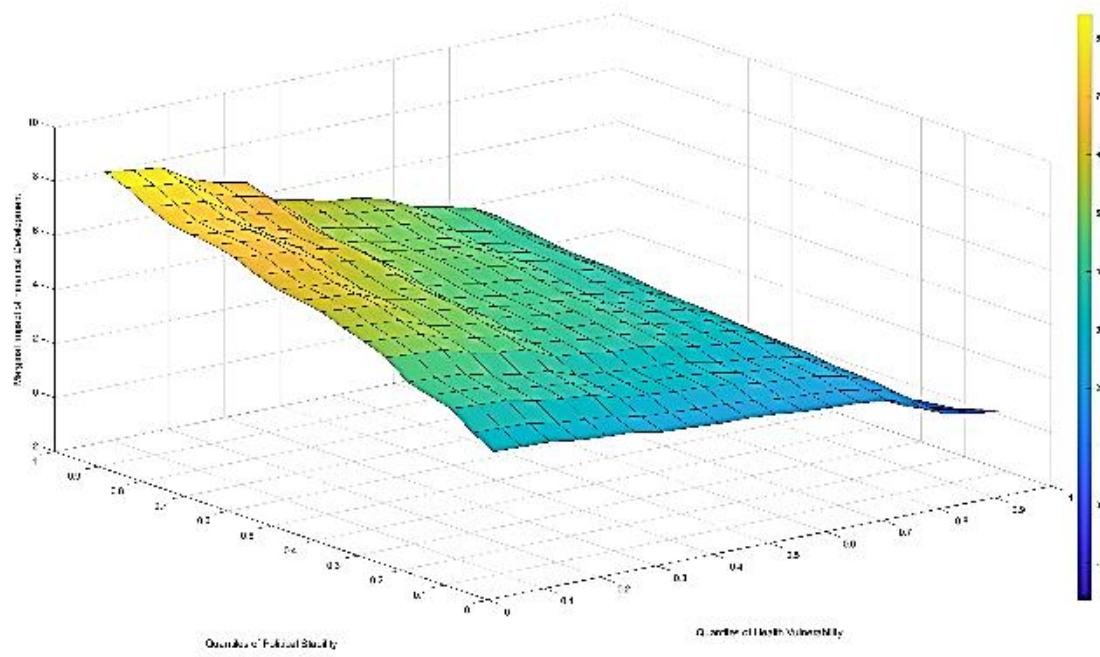

Governance Quality Indicator: Political Stability

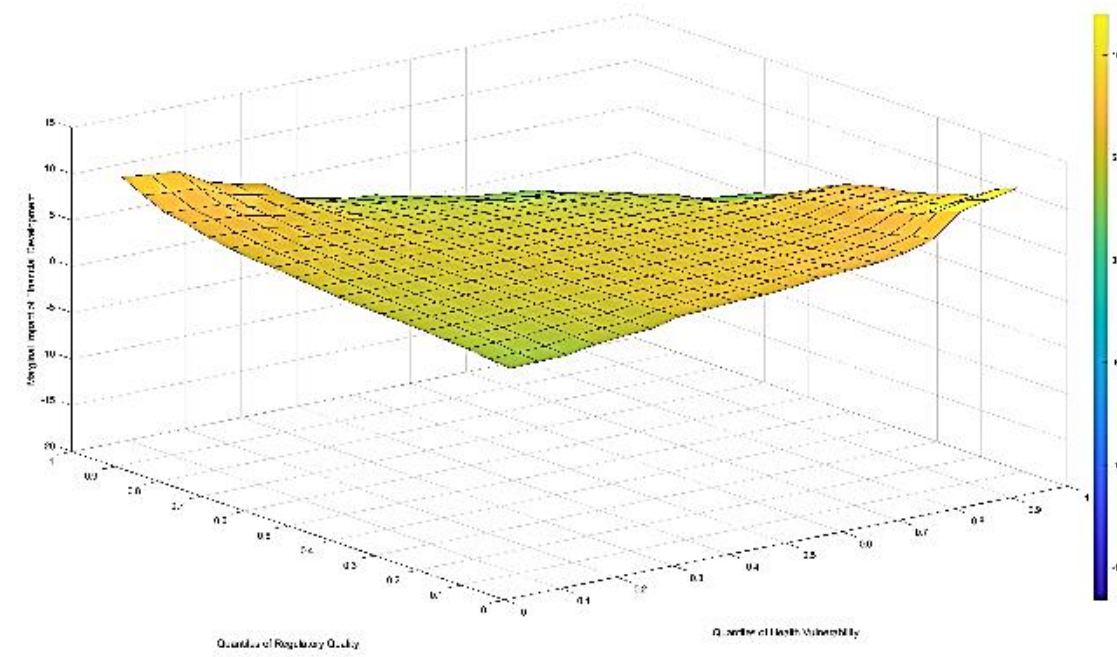

Governance Quality Indicator: Regulatory Quality

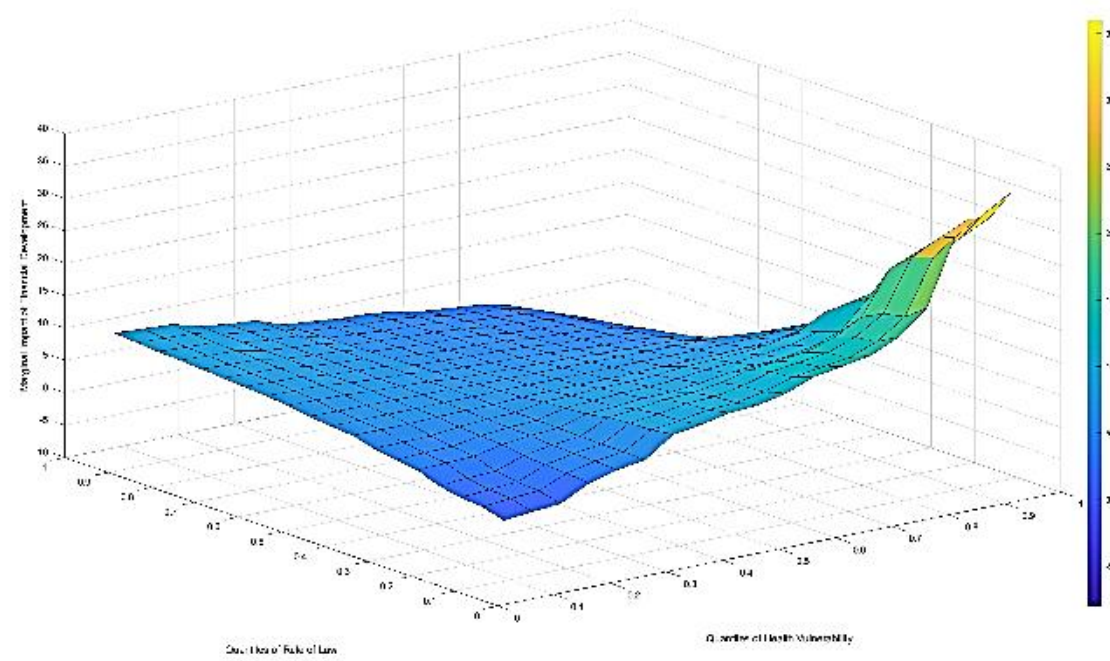

Governance Quality Indicator: Rule of Law

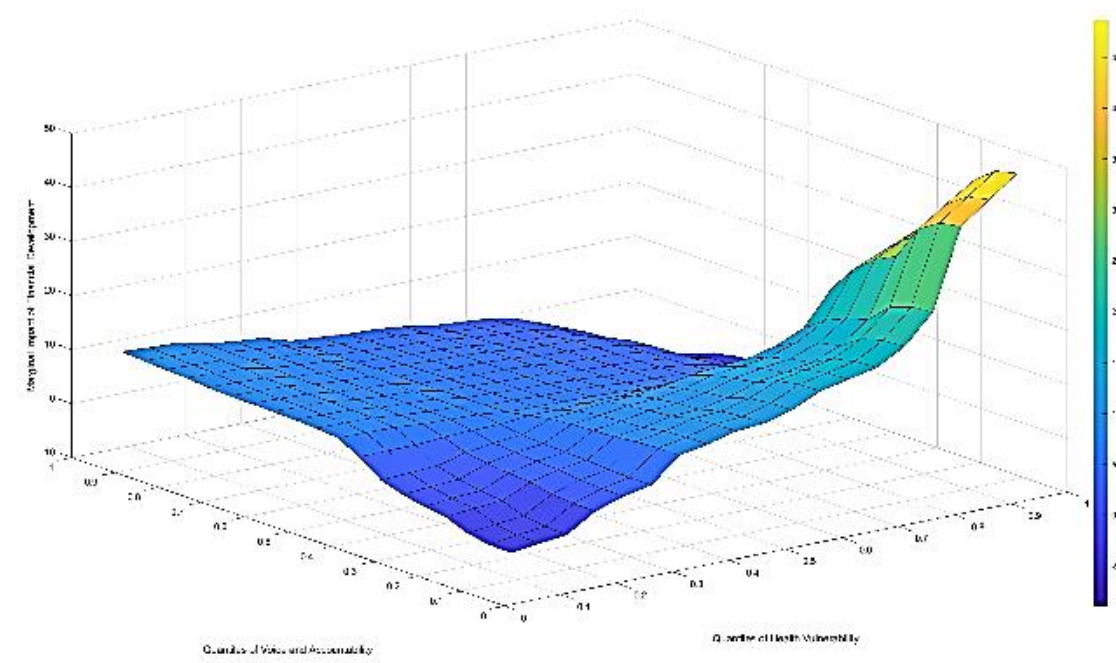

Governance Quality Indicator: Voice and Accountability

Figure S70: BRICS Policy Simulation Outcomes for the Marginal Impact of Financial Development on SDG 7.2.1 under Health Vulnerability and Governance Quality, Related to Figure 4-5 and Table 2

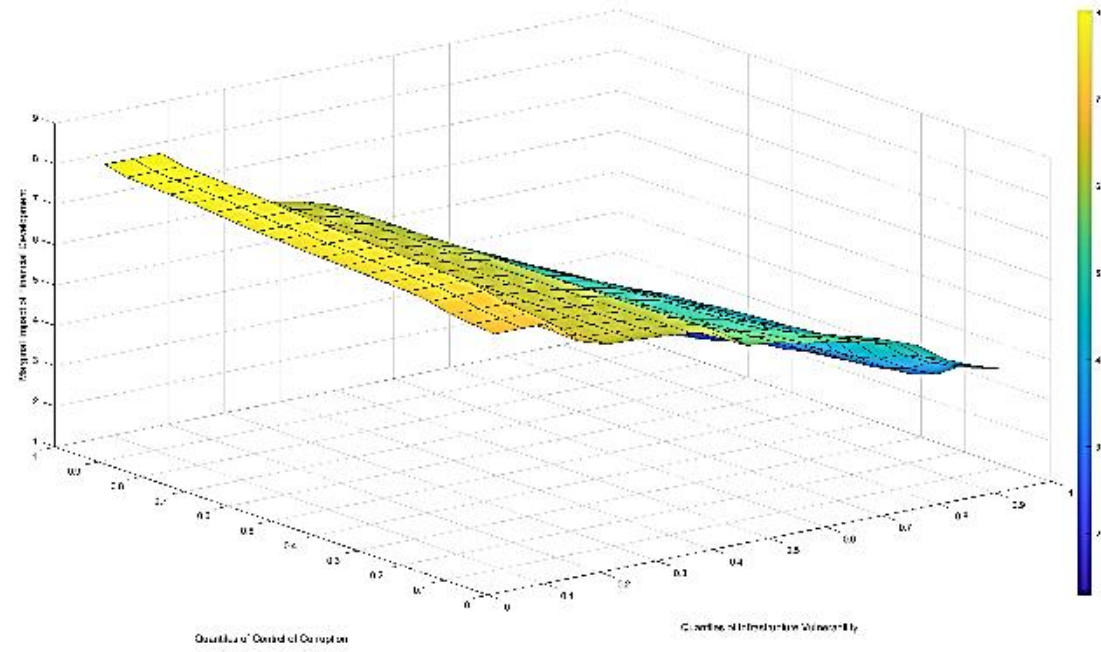

Governance Quality Indicator: Control of Corruption

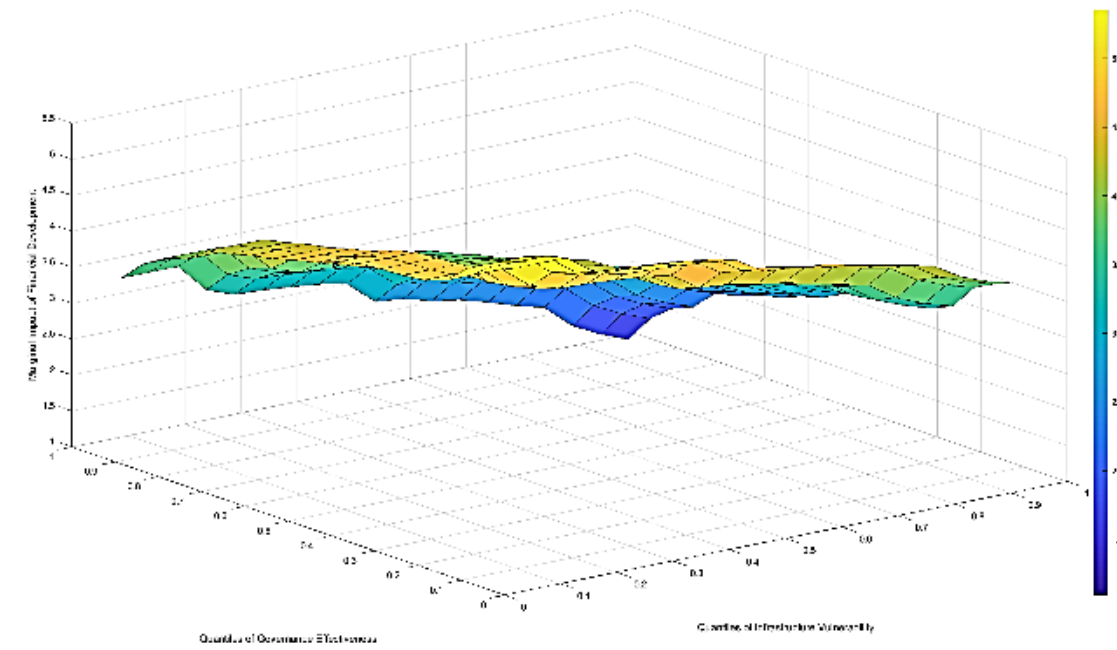

Governance Quality Indicator: Governance Effectiveness

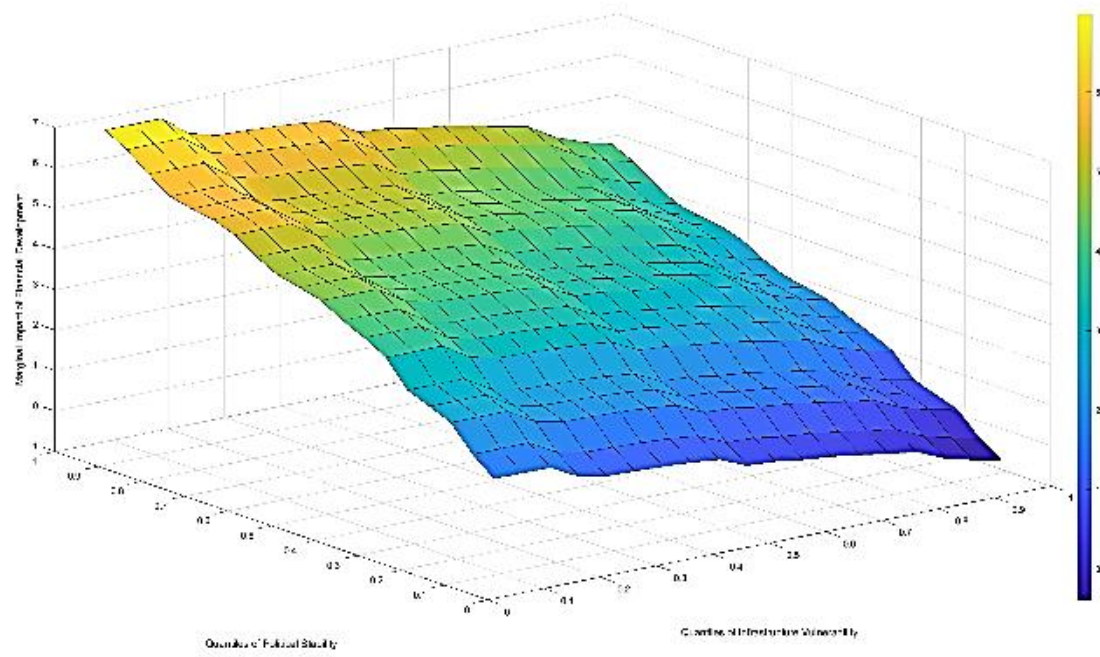

Governance Quality Indicator: Political Stability

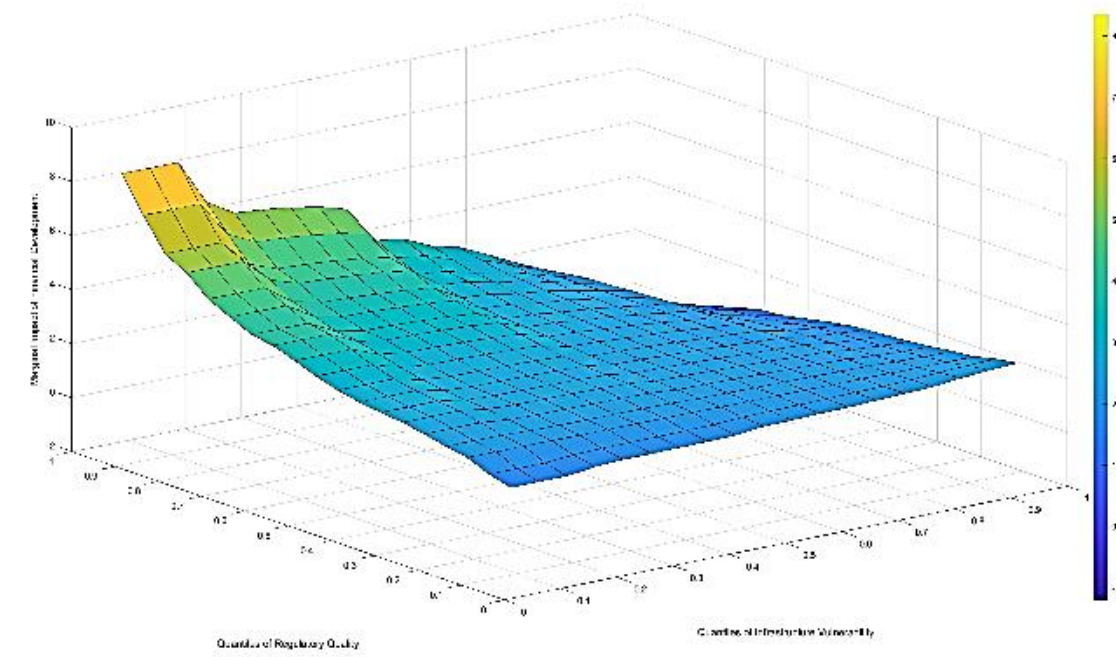

Governance Quality Indicator: Regulatory Quality

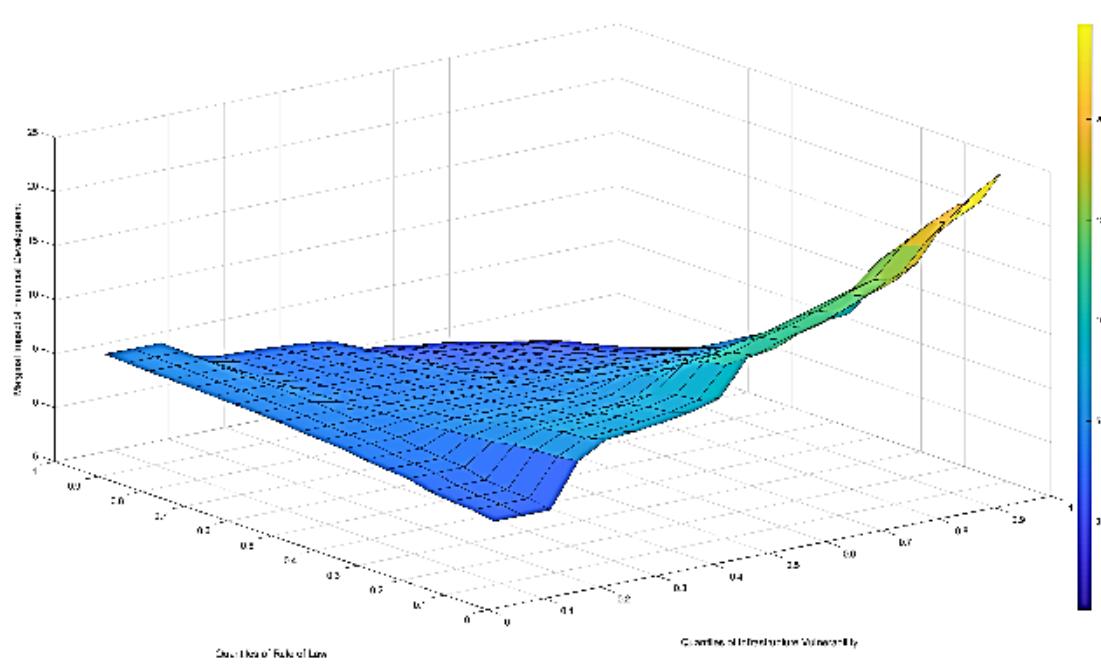

Governance Quality Indicator: Rule of Law

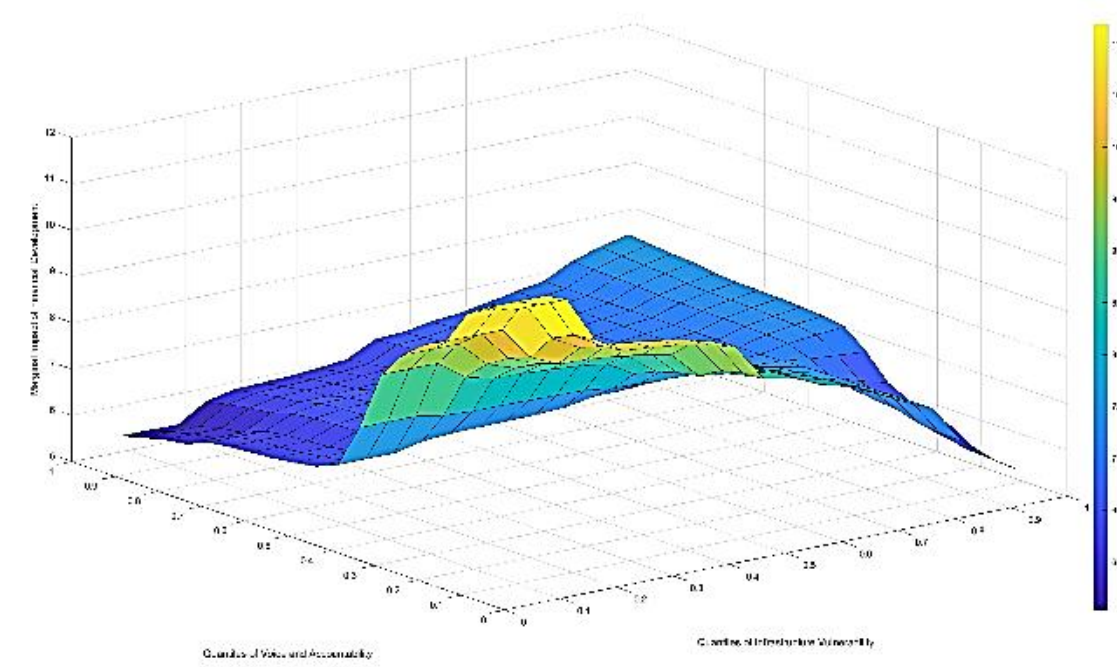

Governance Quality Indicator: Voice and Accountability

Figure S71: BRICS Policy Simulation Outcomes for the Marginal Impact of Financial Development on SDG 7.2.1 under Infrastructure Vulnerability and Governance Quality, Related to Figure 4-5 and Table 2

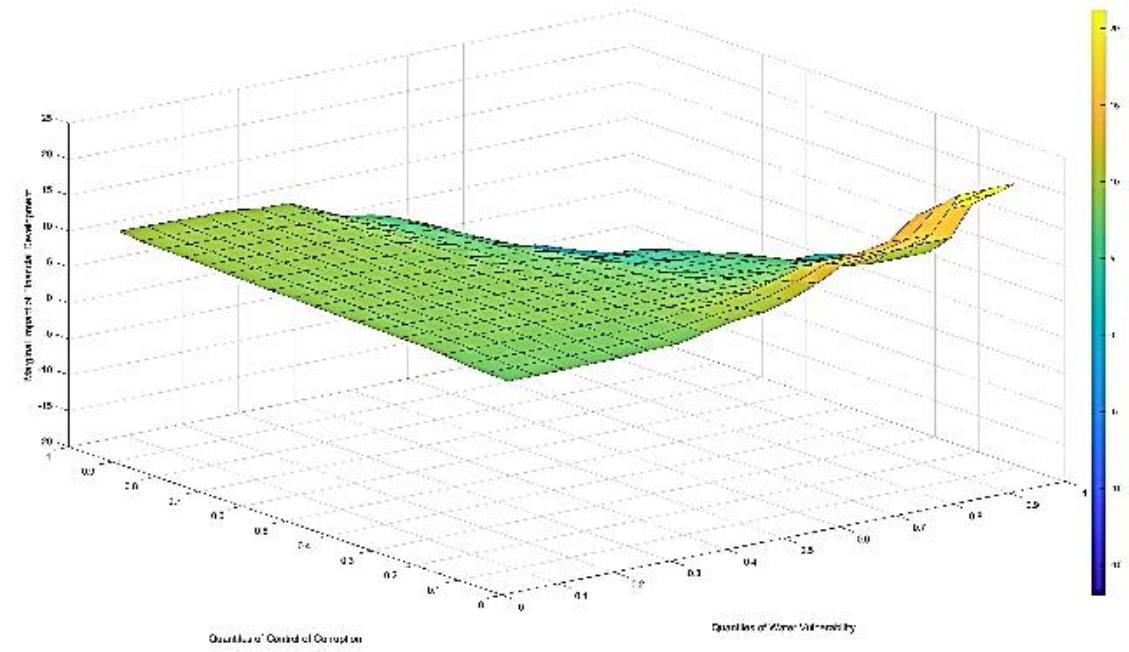

Governance Quality Indicator: Control of Corruption

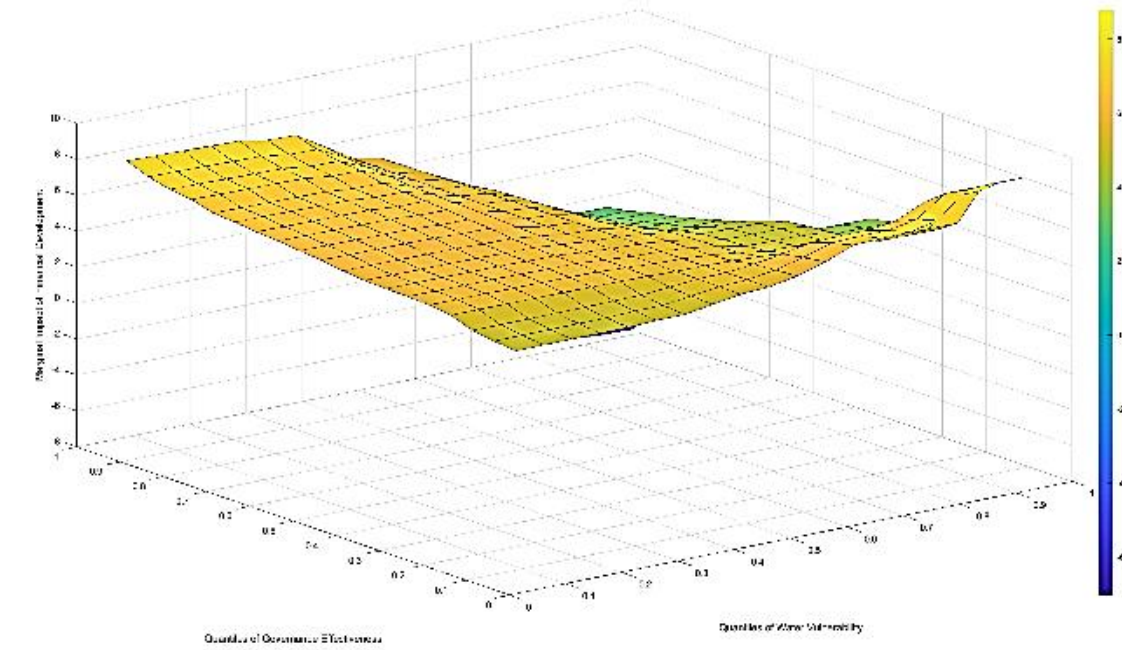

Governance Quality Indicator: Governance Effectiveness

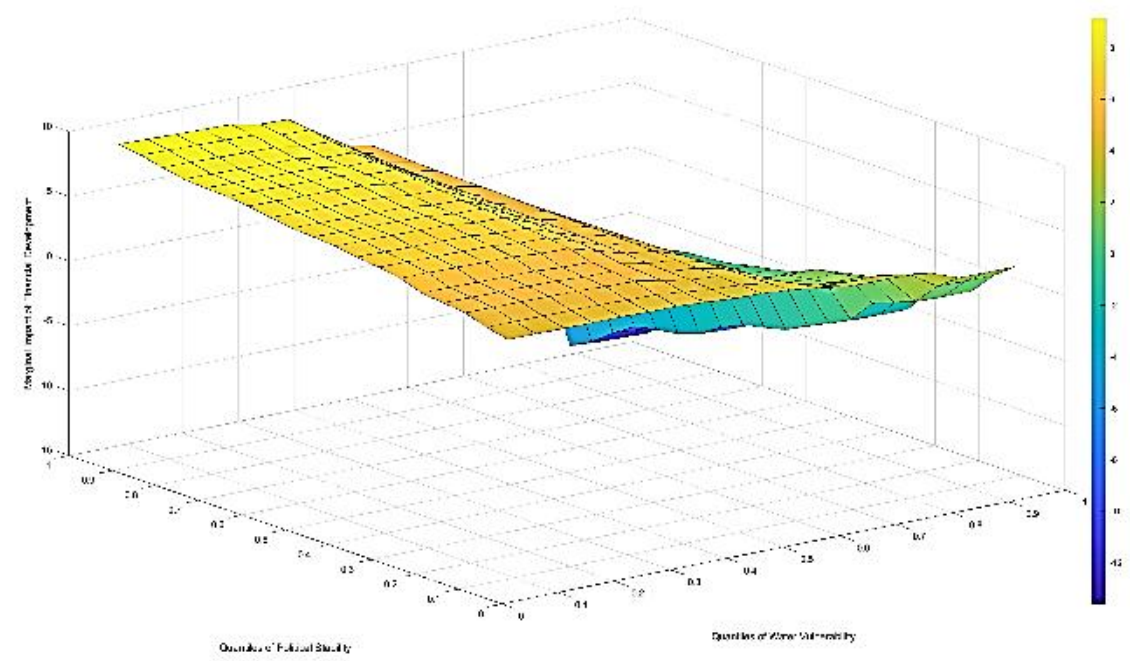

Governance Quality Indicator: Political Stability

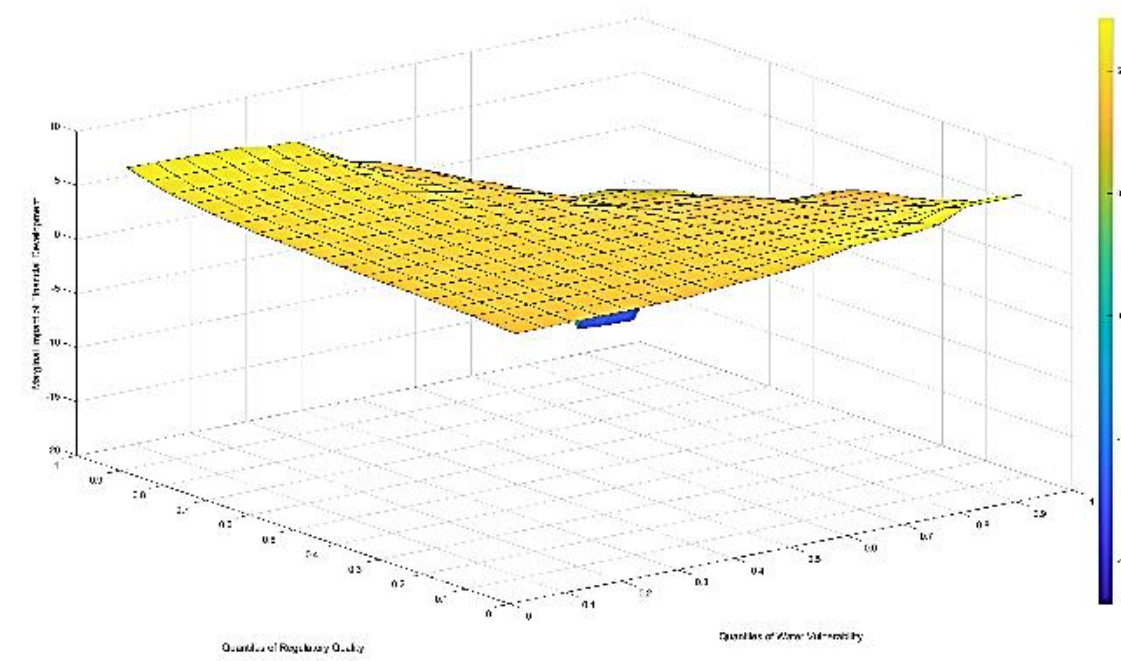

Governance Quality Indicator: Regulatory Quality

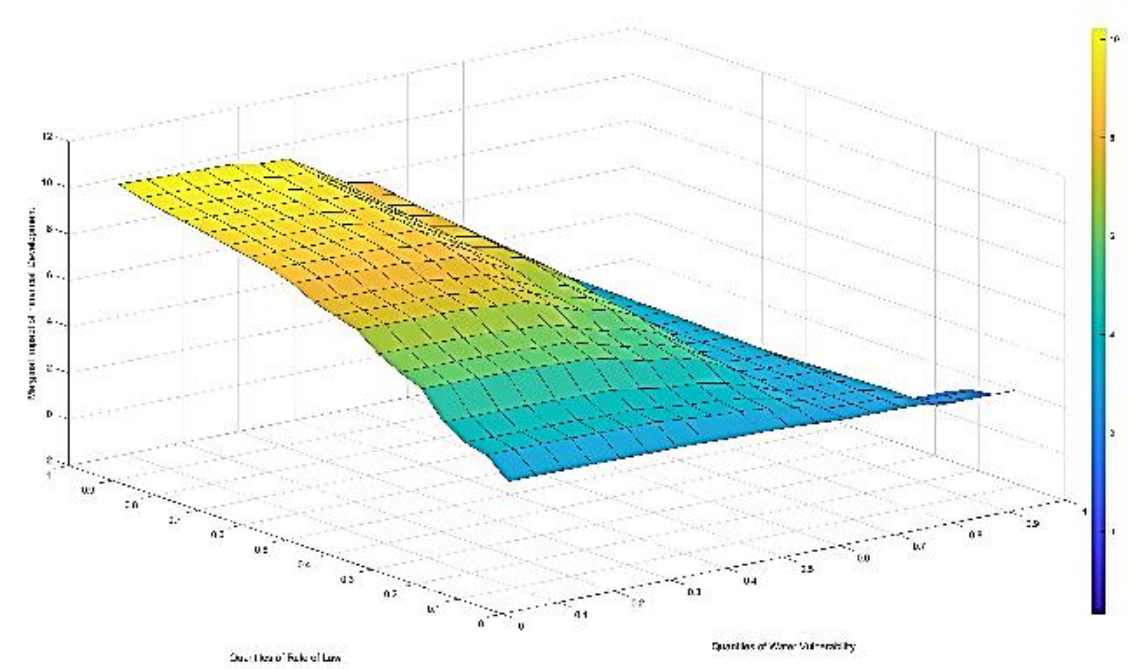

Governance Quality Indicator: Rule of Law

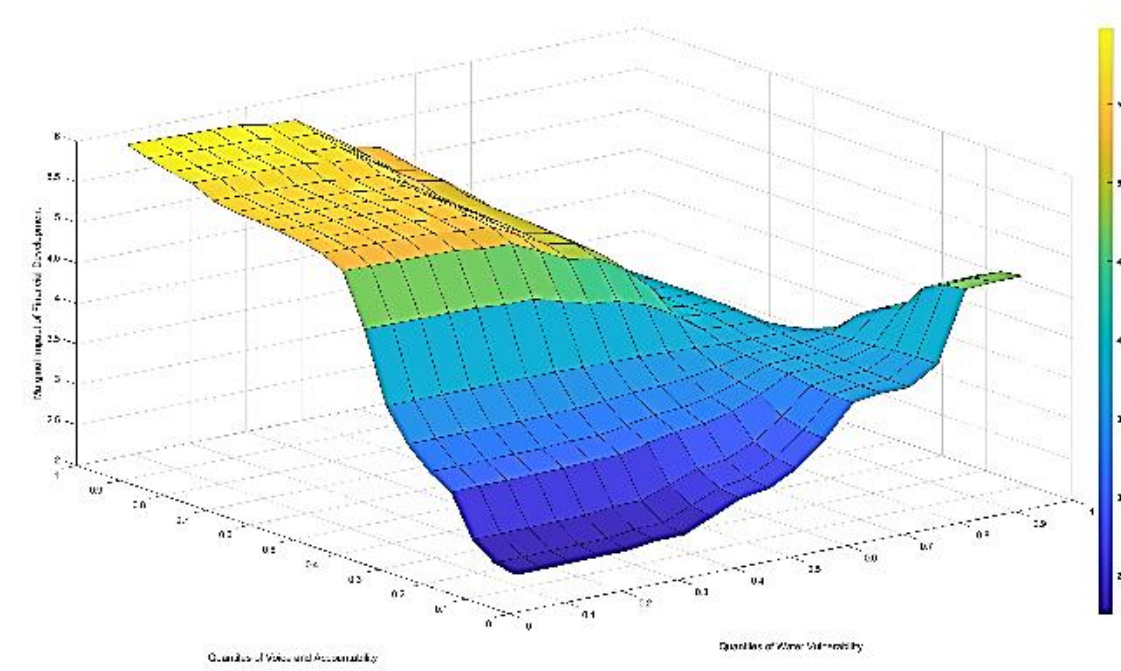

Governance Quality Indicator: Voice and Accountability

Figure S72: BRICS Policy Simulation Outcomes for the Marginal Impact of Financial Development on SDG 7.2.1 under Water Vulnerability and Governance Quality, Related to Figure 4-5 and Table 2

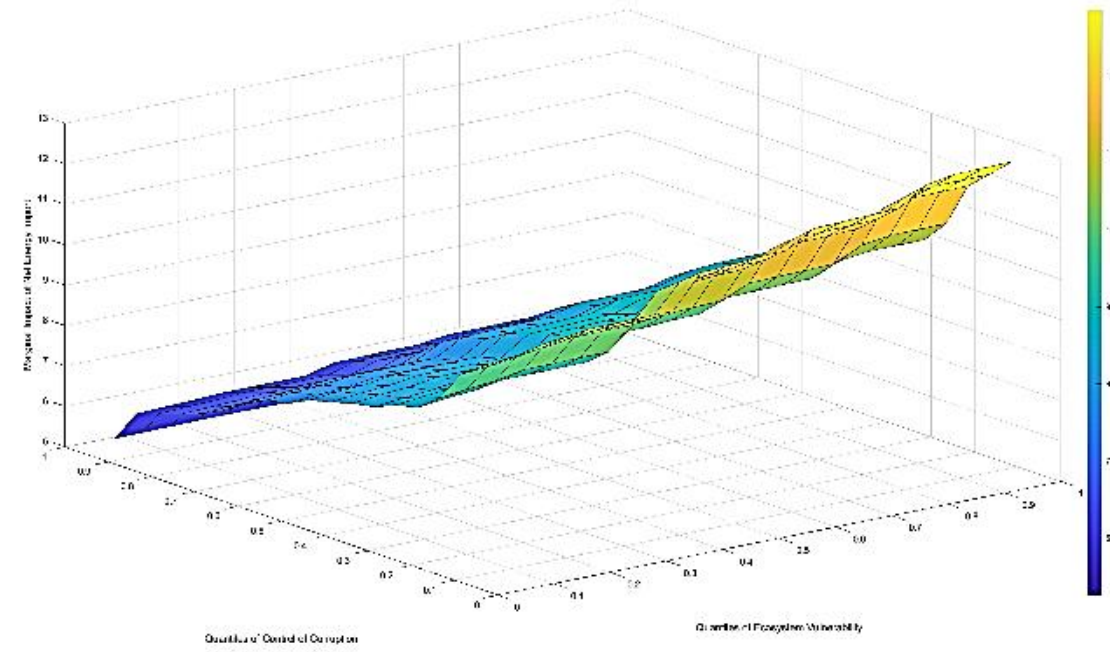

Governance Quality Indicator: Control of Corruption

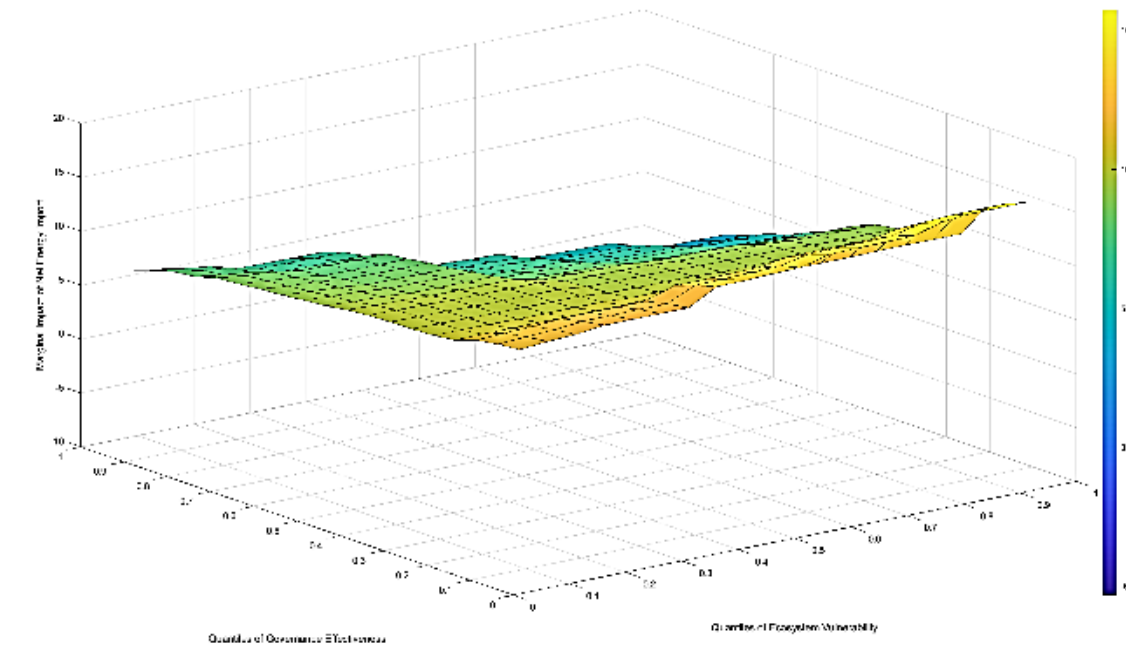

Governance Quality Indicator: Governance Effectiveness

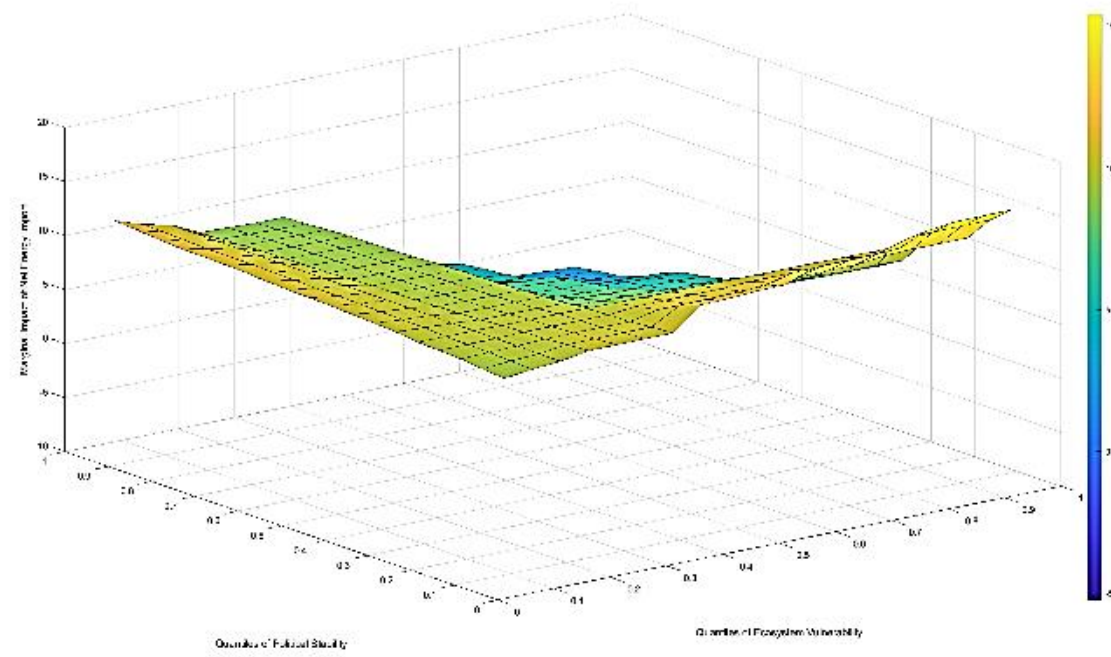

Governance Quality Indicator: Political Stability

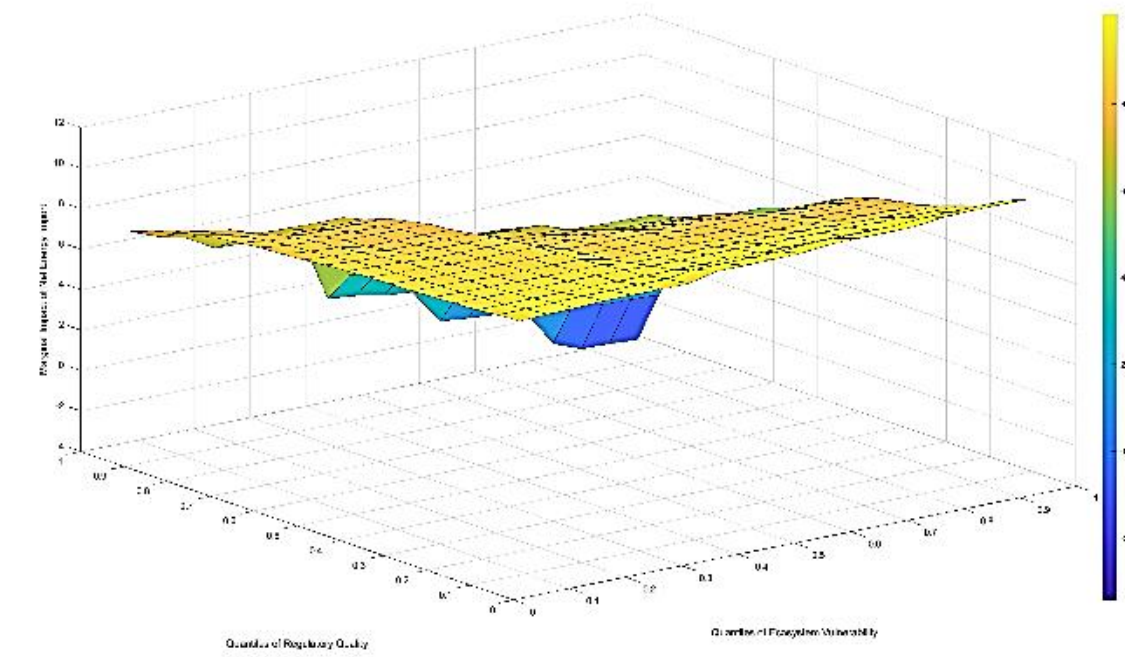

Governance Quality Indicator: Regulatory Quality

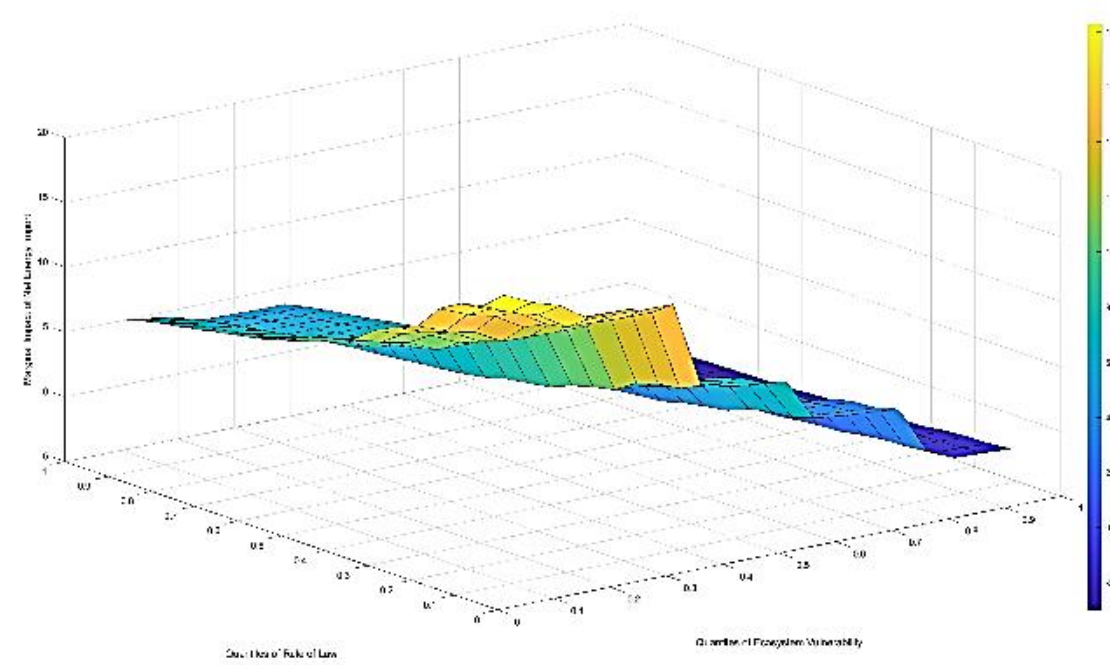

Governance Quality Indicator: Rule of Law

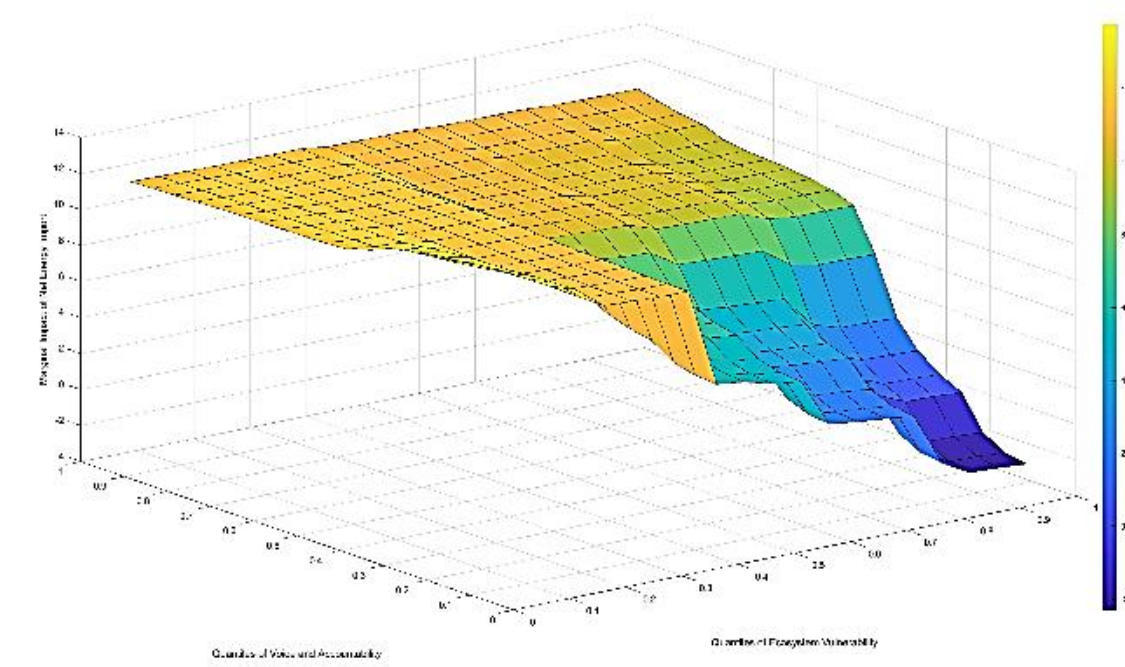

Governance Quality Indicator: Voice and Accountability

Figure S73: BRICS Policy Simulation Outcomes for the Marginal Impact of Net Energy Import on SDG 7.2.1 under Ecosystem Vulnerability and Governance Quality, Related to Figure 4-5 and Table 2

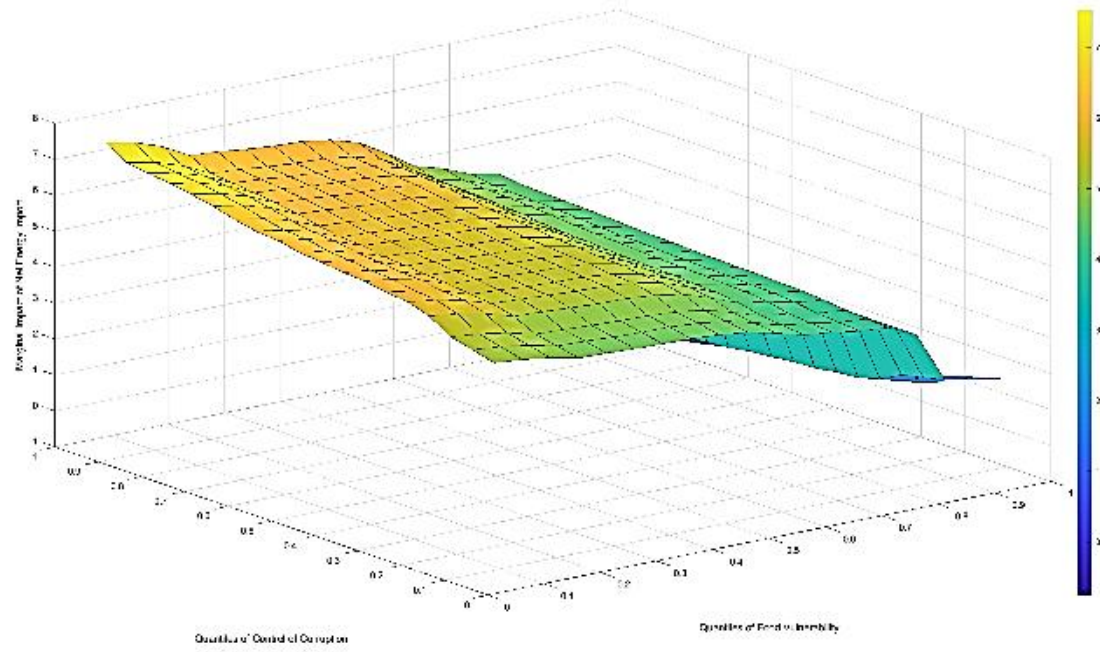

Governance Quality Indicator: Control of Corruption

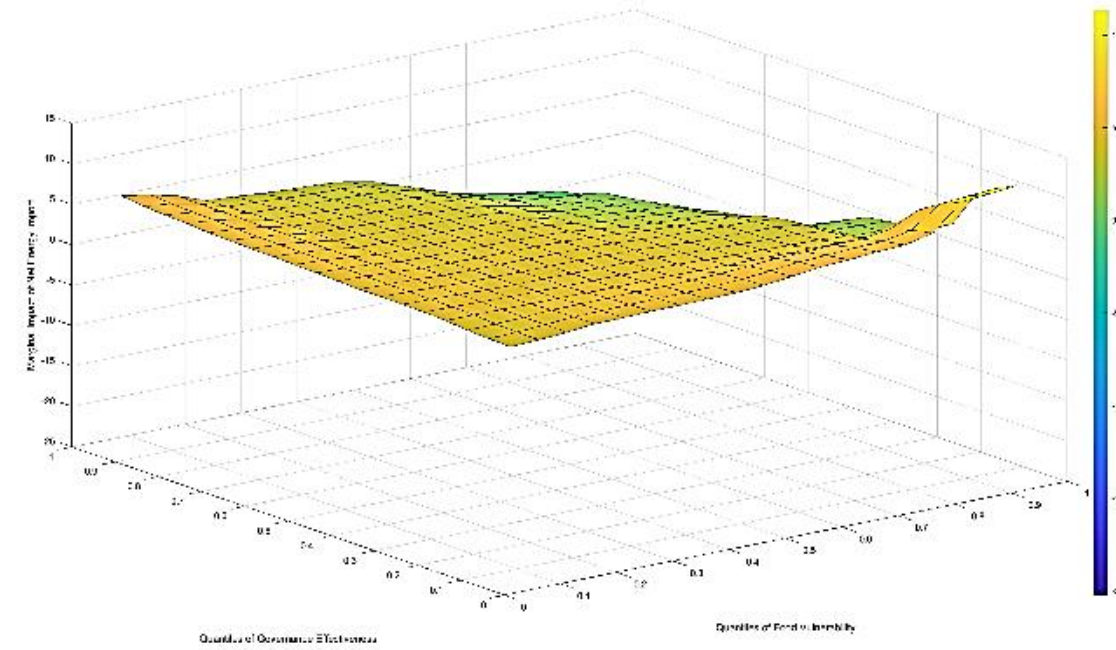

Governance Quality Indicator: Governance Effectiveness

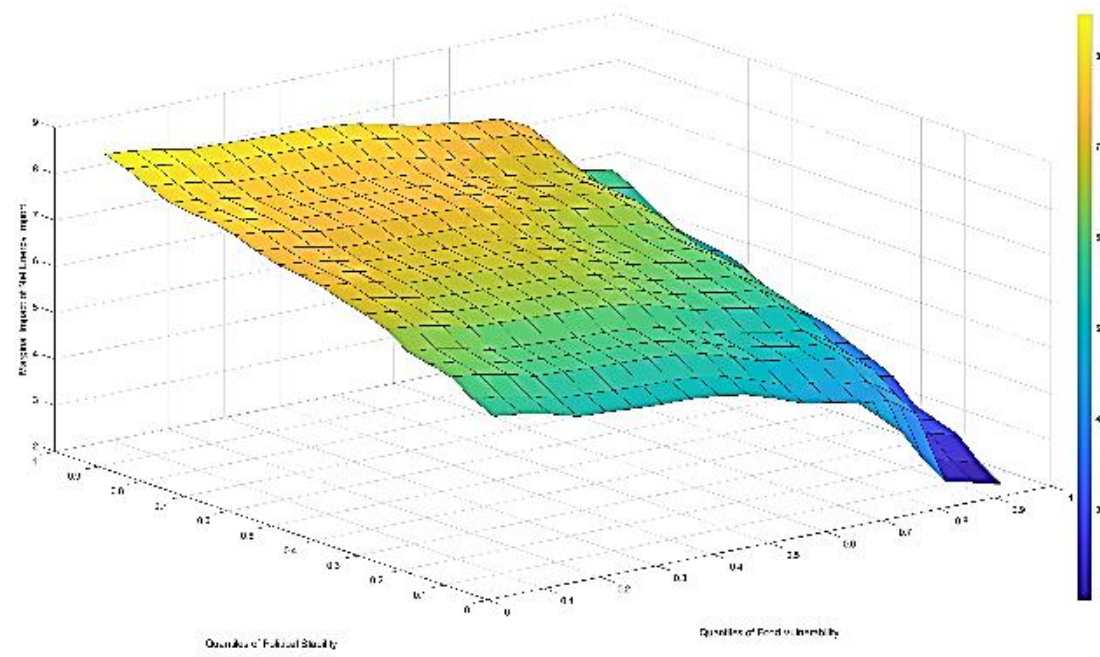

Governance Quality Indicator: Political Stability

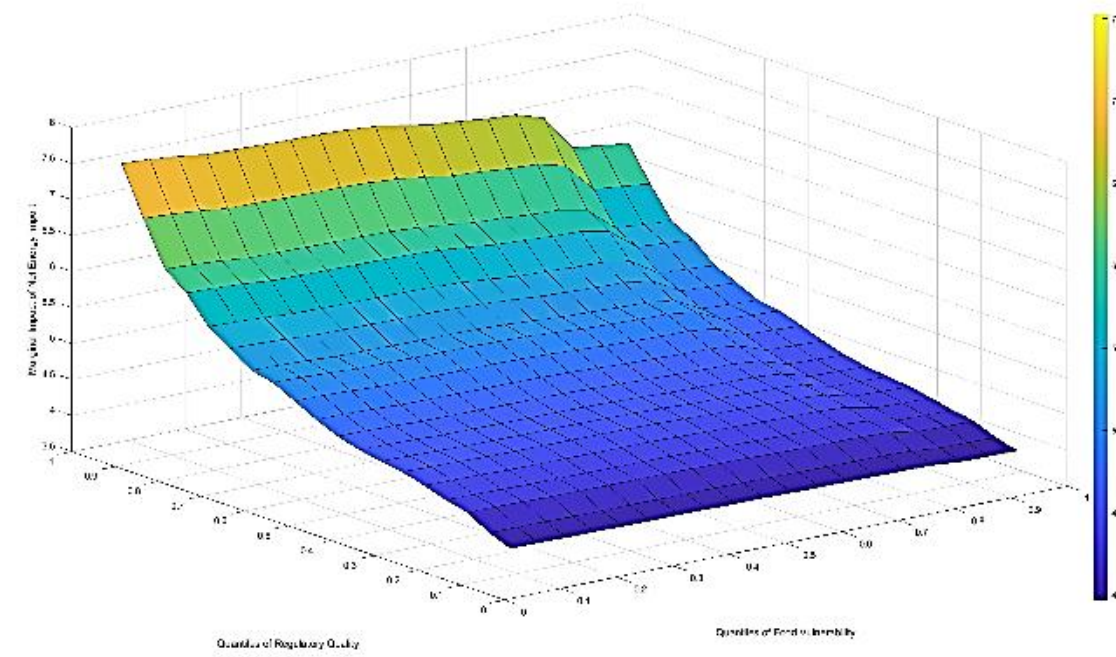

Governance Quality Indicator: Regulatory Quality

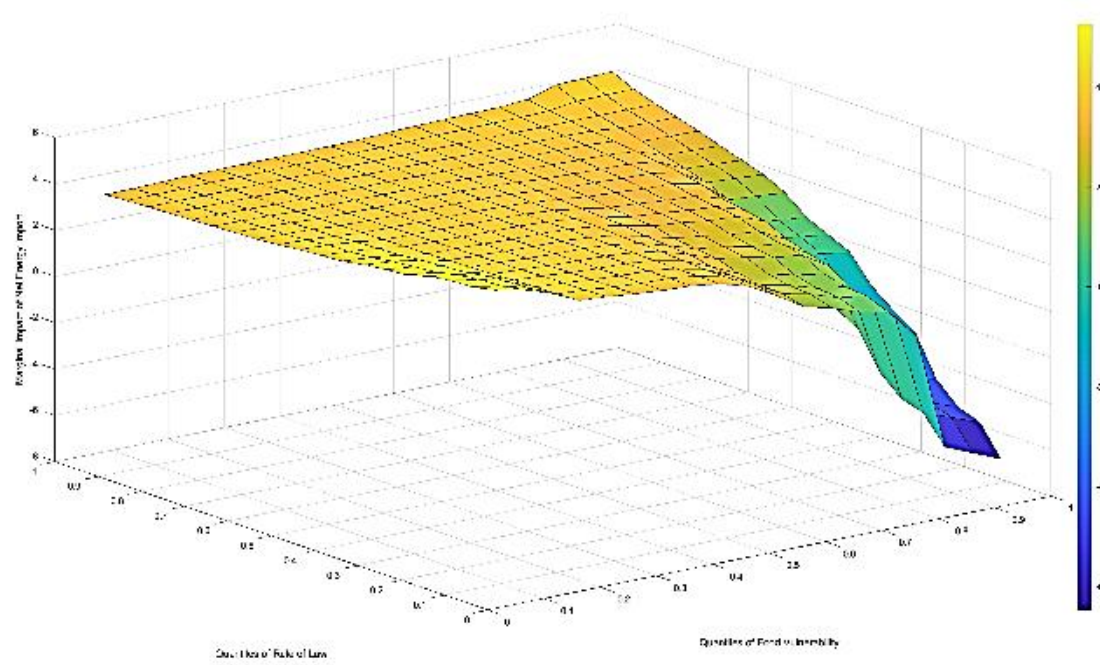

Governance Quality Indicator: Rule of Law

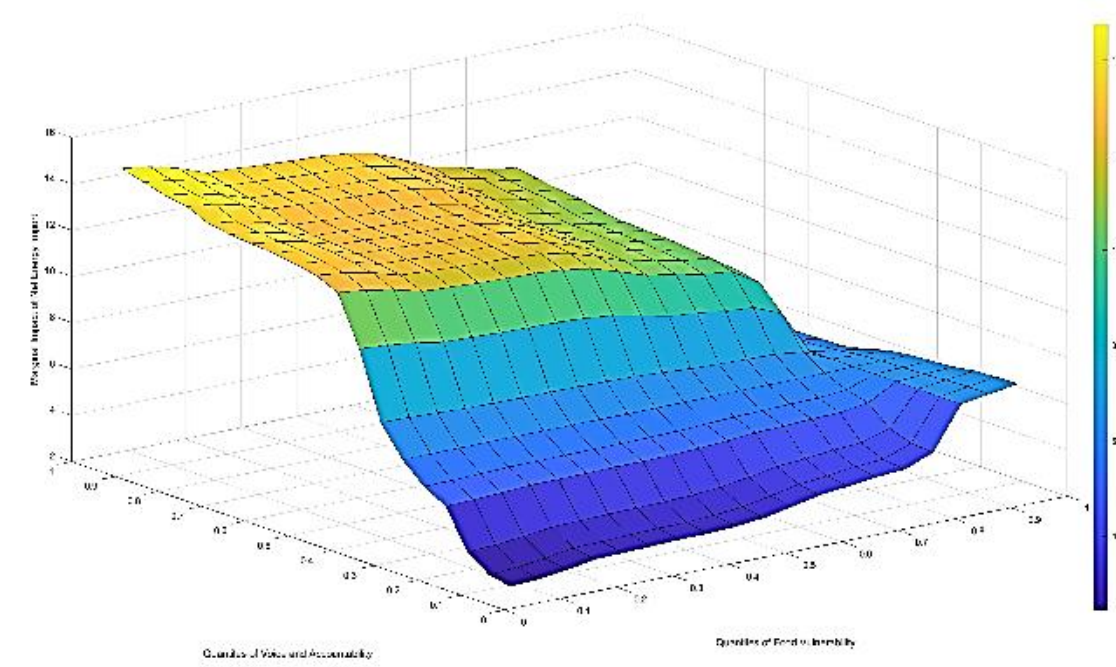

Governance Quality Indicator: Voice and Accountability

Figure S74: BRICS Policy Simulation Outcomes for the Marginal Impact of Net Energy Import on SDG 7.2.1 under Food Vulnerability and Governance Quality, Related to Figure 4-5 and Table 2

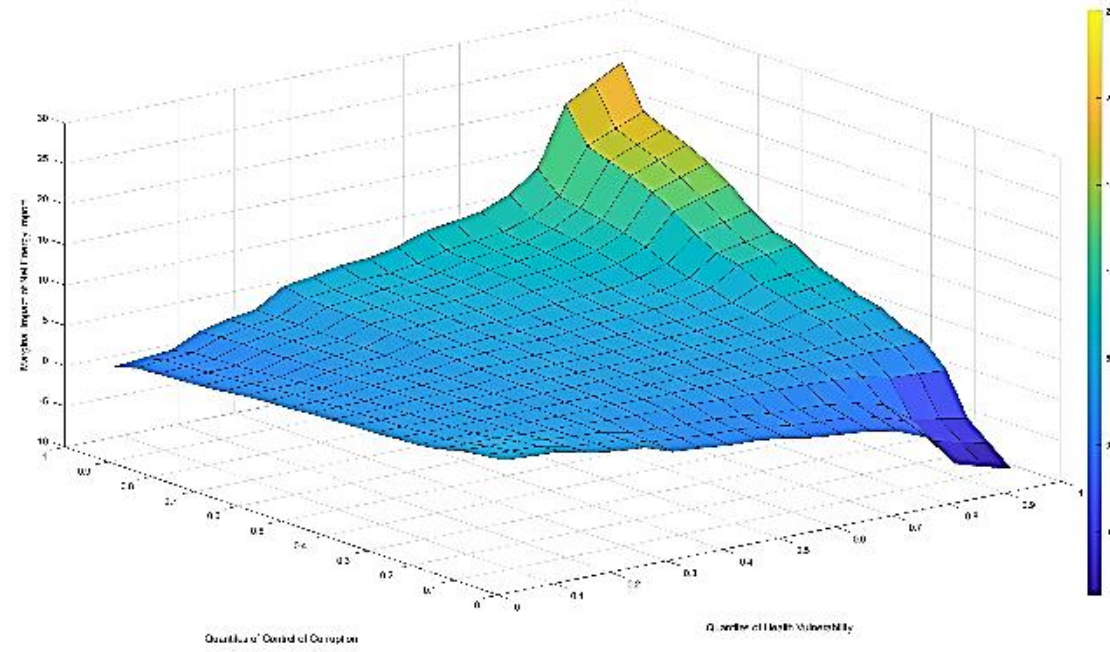

Governance Quality Indicator: Control of Corruption

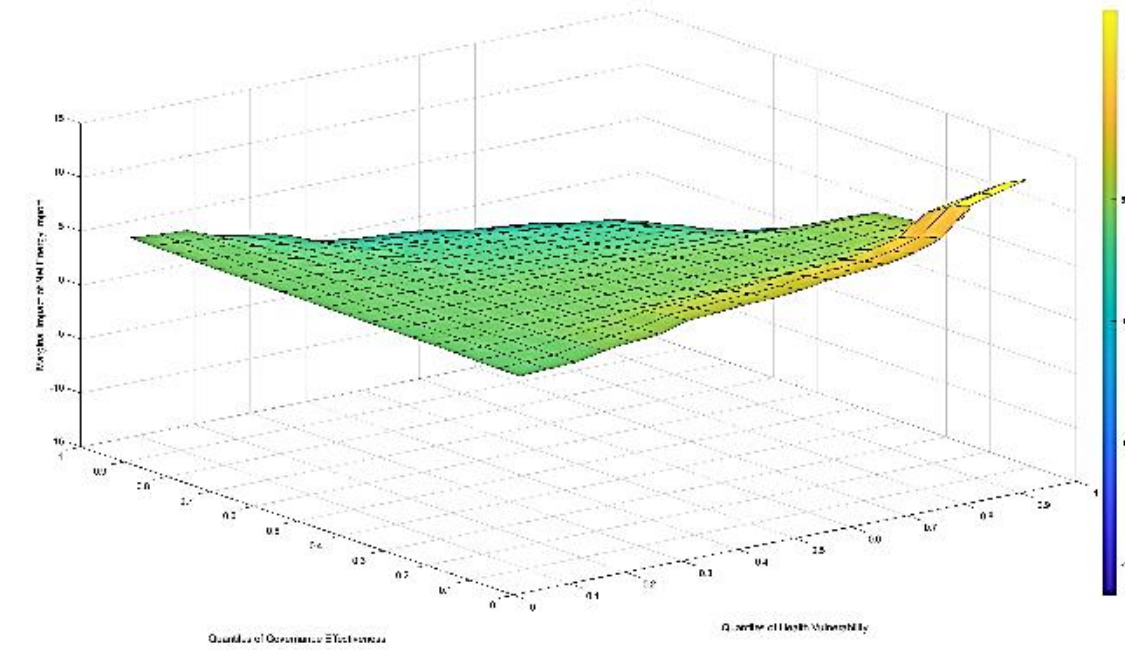

Governance Quality Indicator: Governance Effectiveness

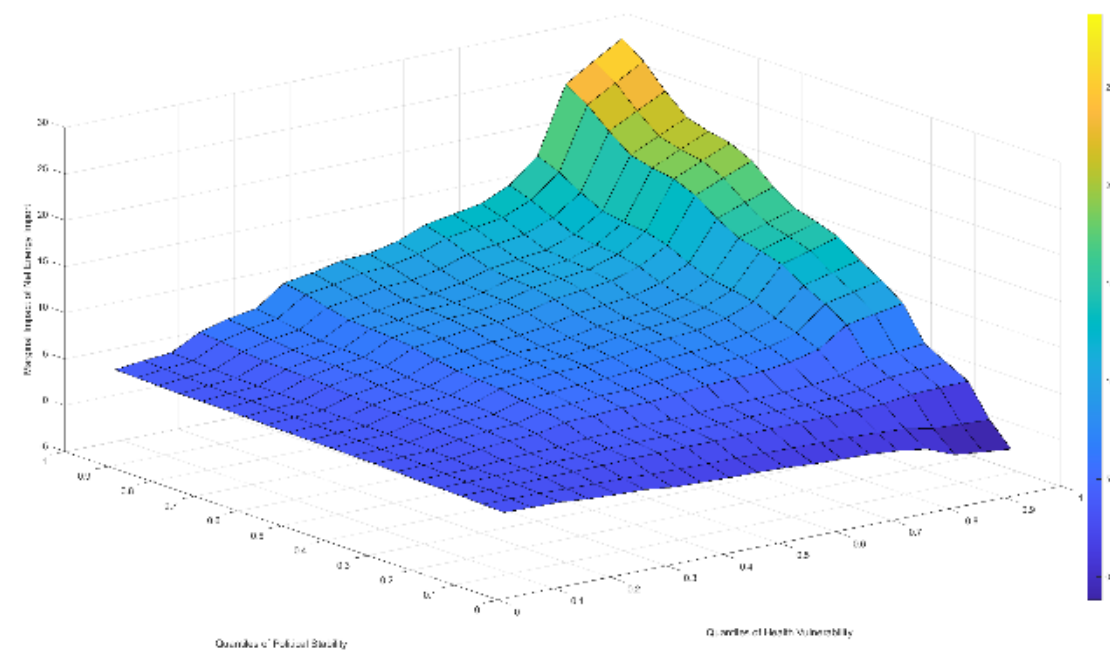

Governance Quality Indicator: Political Stability

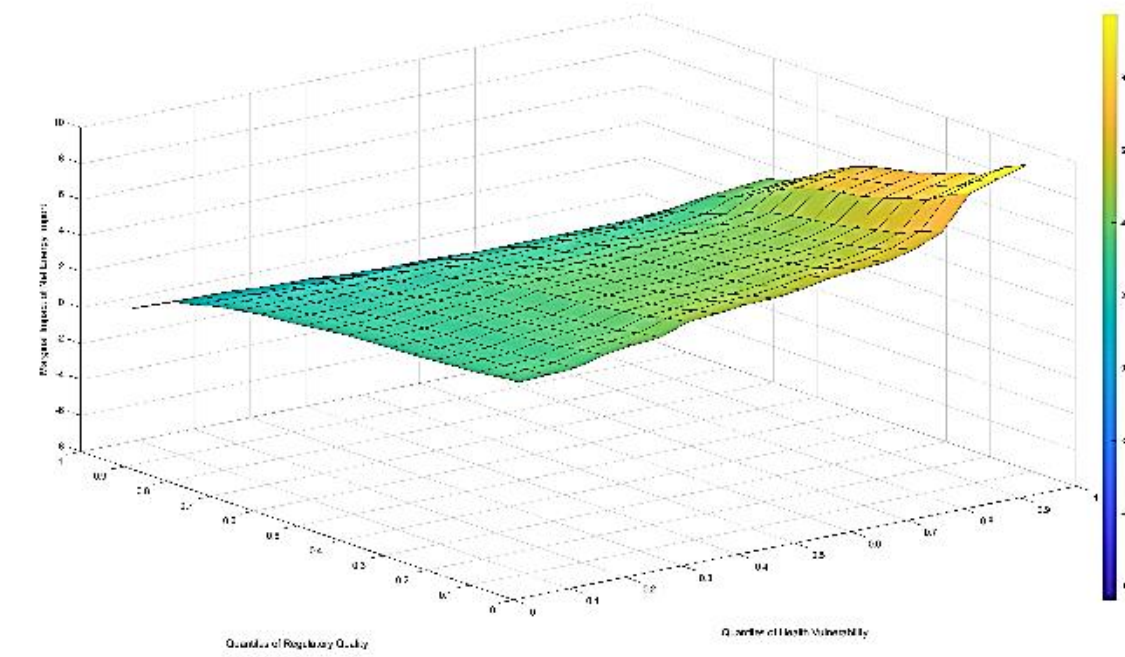

Governance Quality Indicator: Regulatory Quality

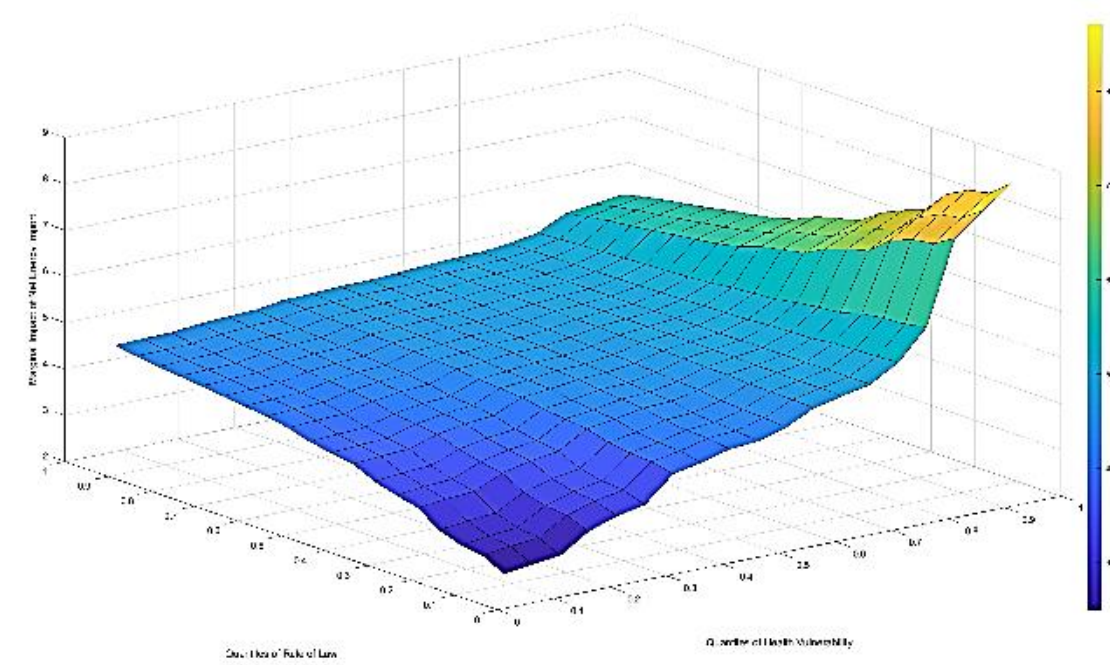

Governance Quality Indicator: Rule of Law

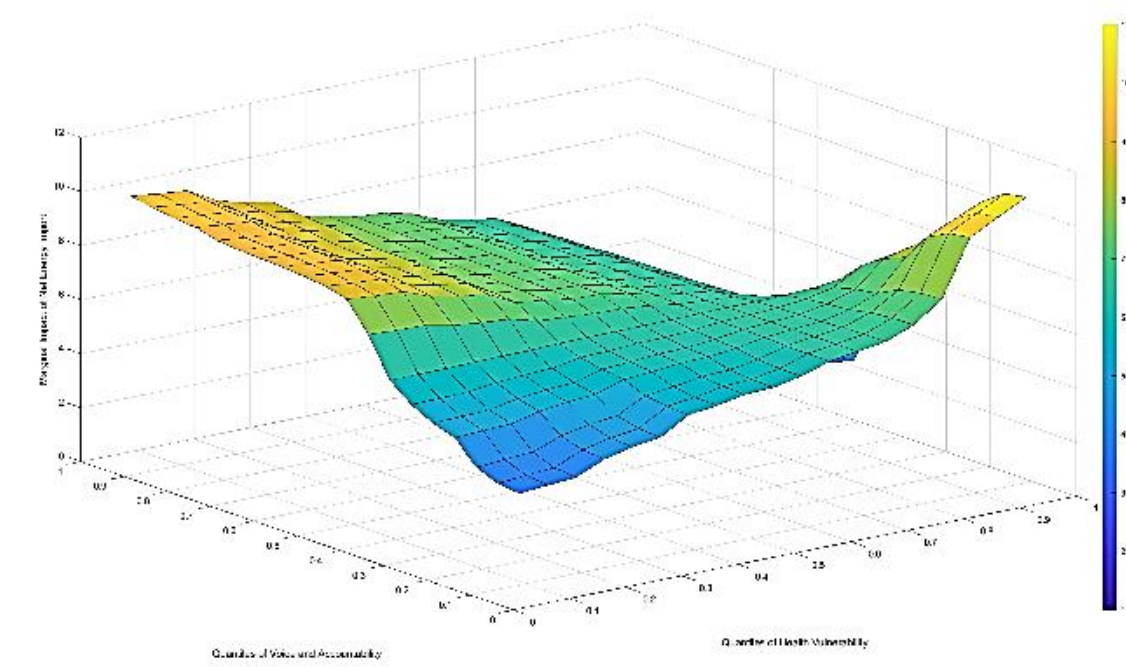

Governance Quality Indicator: Voice and Accountability

Figure S75: BRICS Policy Simulation Outcomes for the Marginal Impact of Net Energy Import on SDG 7.2.1 under Health Vulnerability and Governance Quality, Related to Figure 4-5 and Table 2

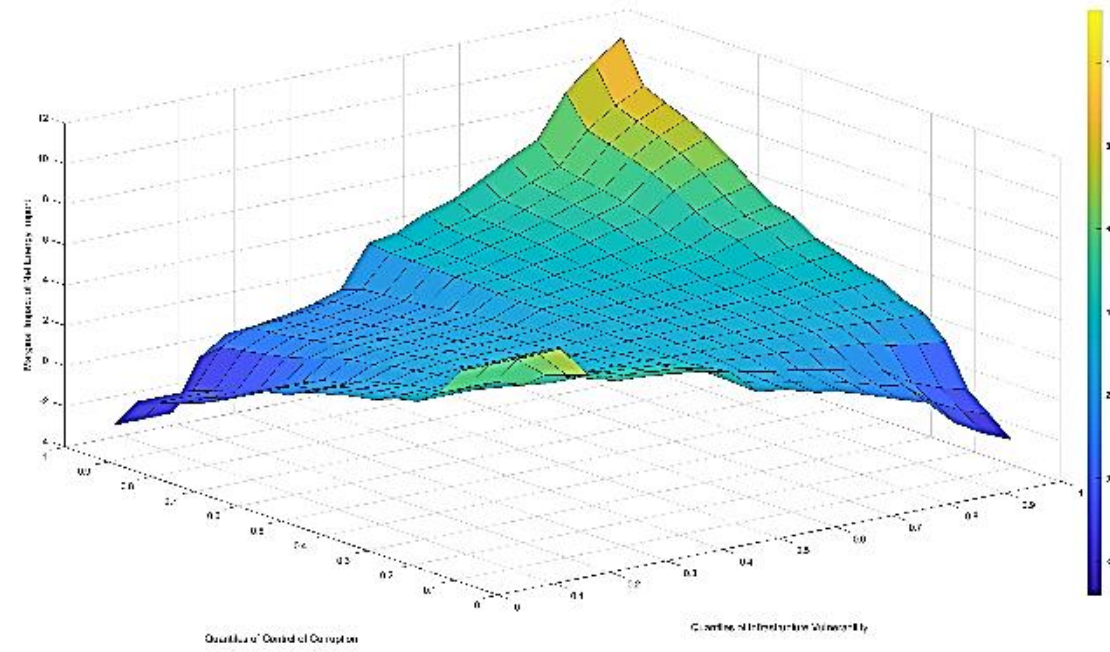

Governance Quality Indicator: Control of Corruption

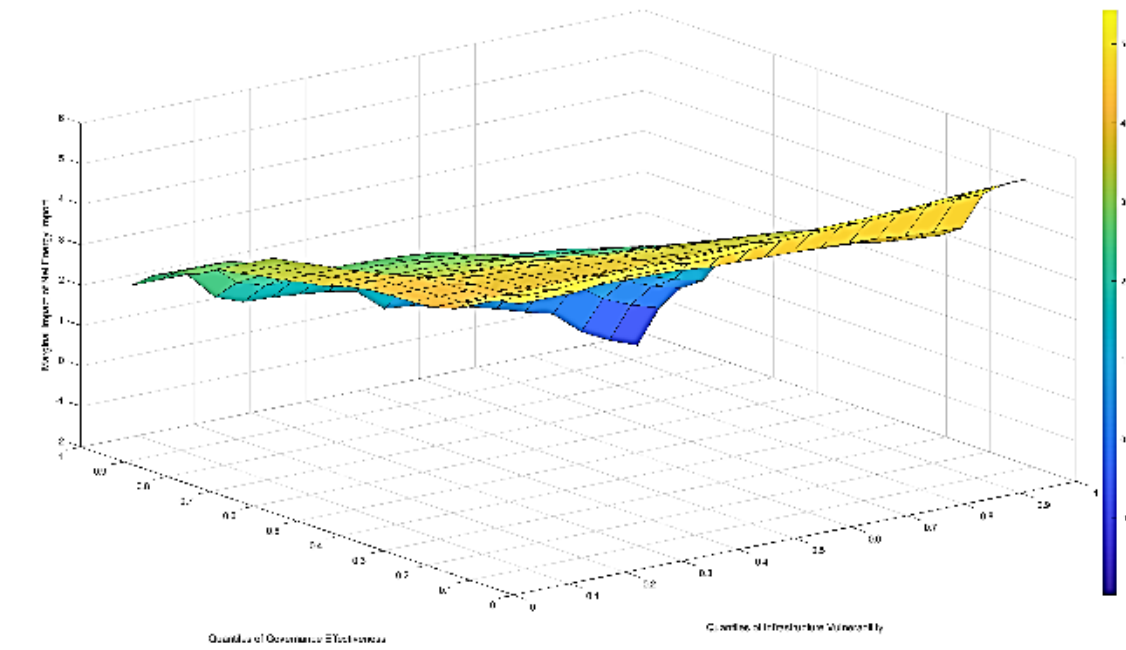

Governance Quality Indicator: Governance Effectiveness

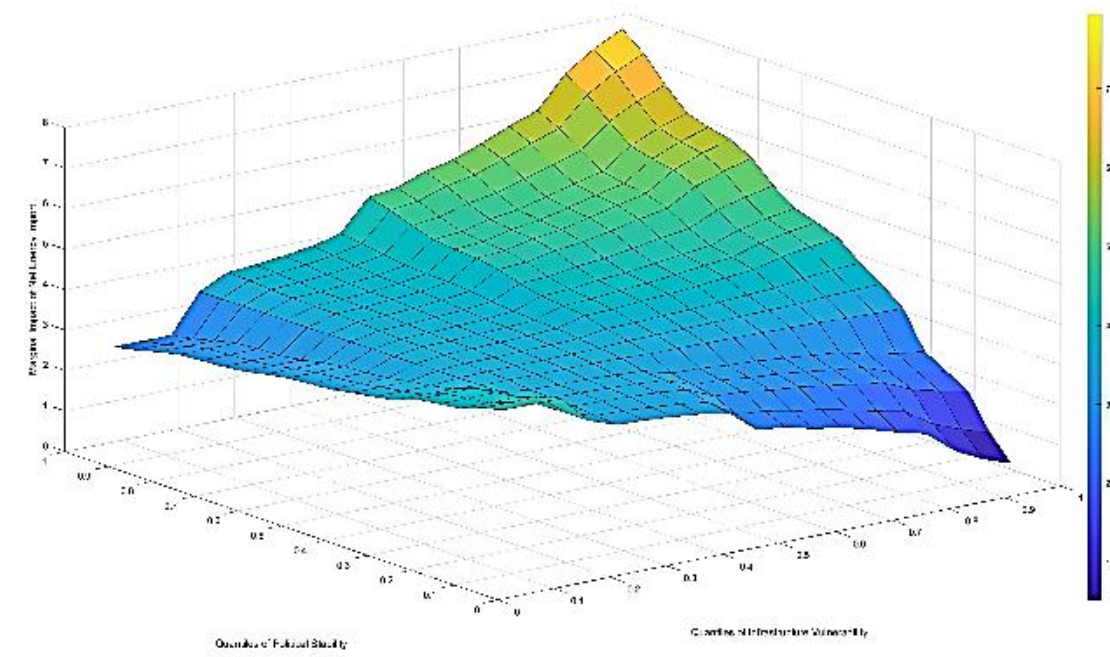

Governance Quality Indicator: Political Stability

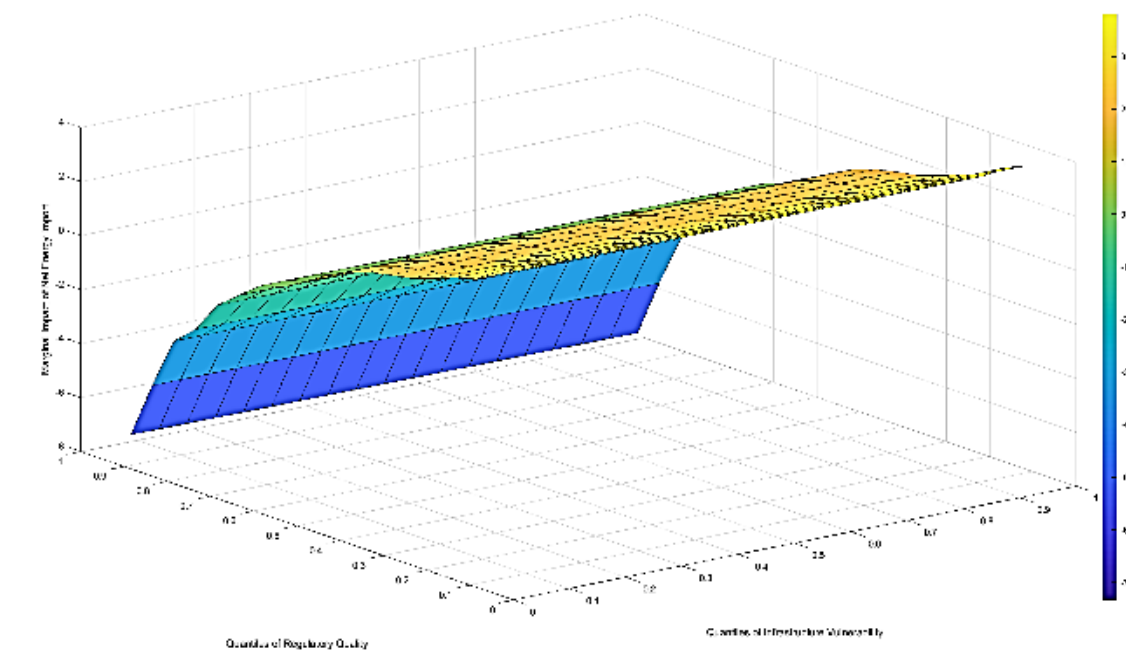

Governance Quality Indicator: Regulatory Quality

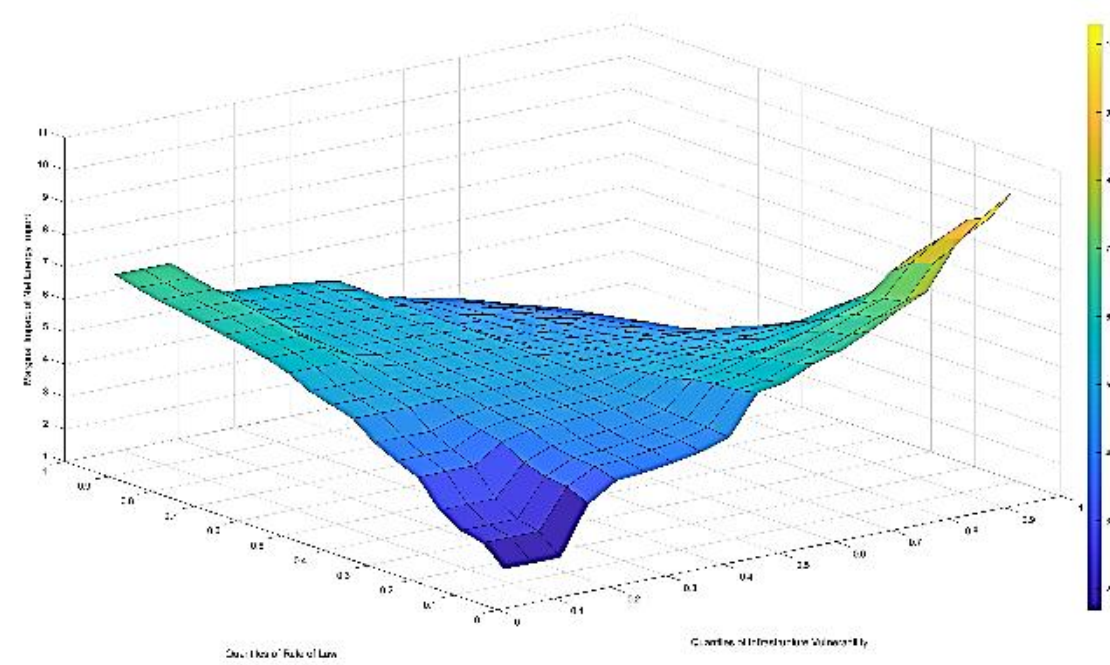

Governance Quality Indicator: Rule of Law

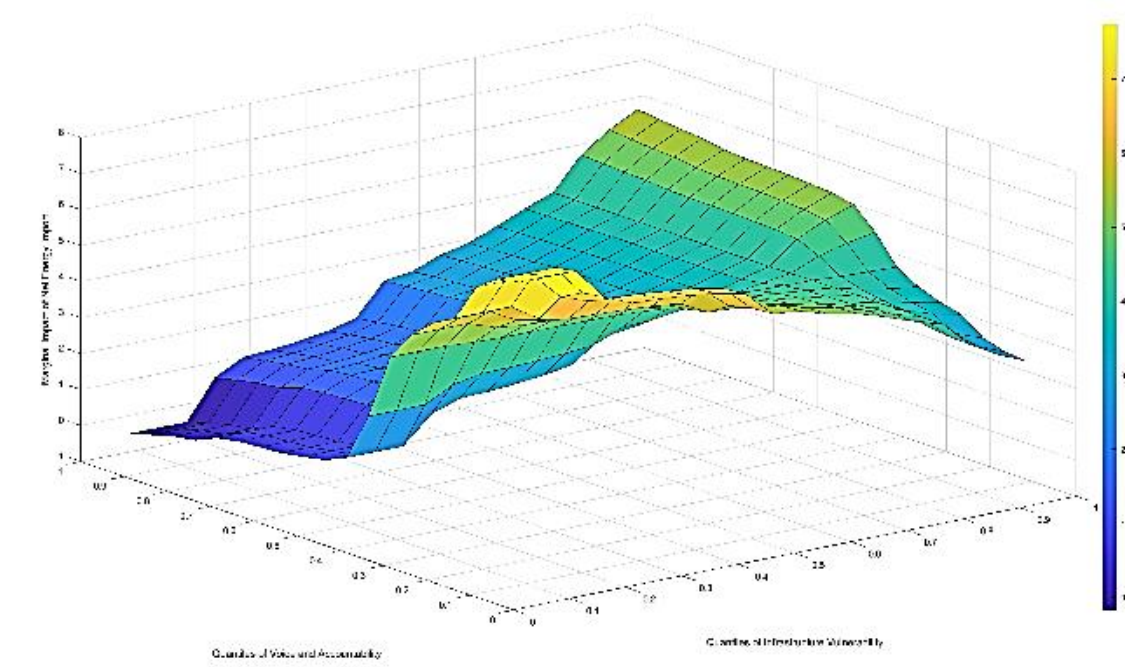

Governance Quality Indicator: Voice and Accountability

Figure S76: BRICS Policy Simulation Outcomes for the Marginal Impact of Net Energy Import on SDG 7.2.1 under Infrastructure Vulnerability and Governance Quality, Related to Figure 4-5 and Table 2

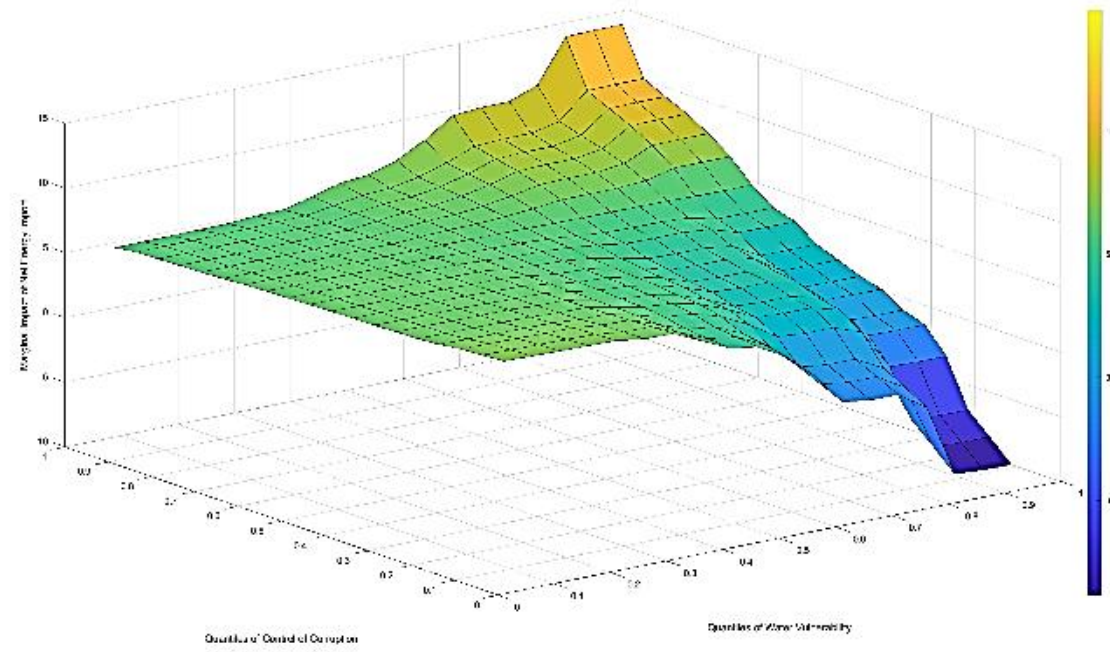

Governance Quality Indicator: Control of Corruption

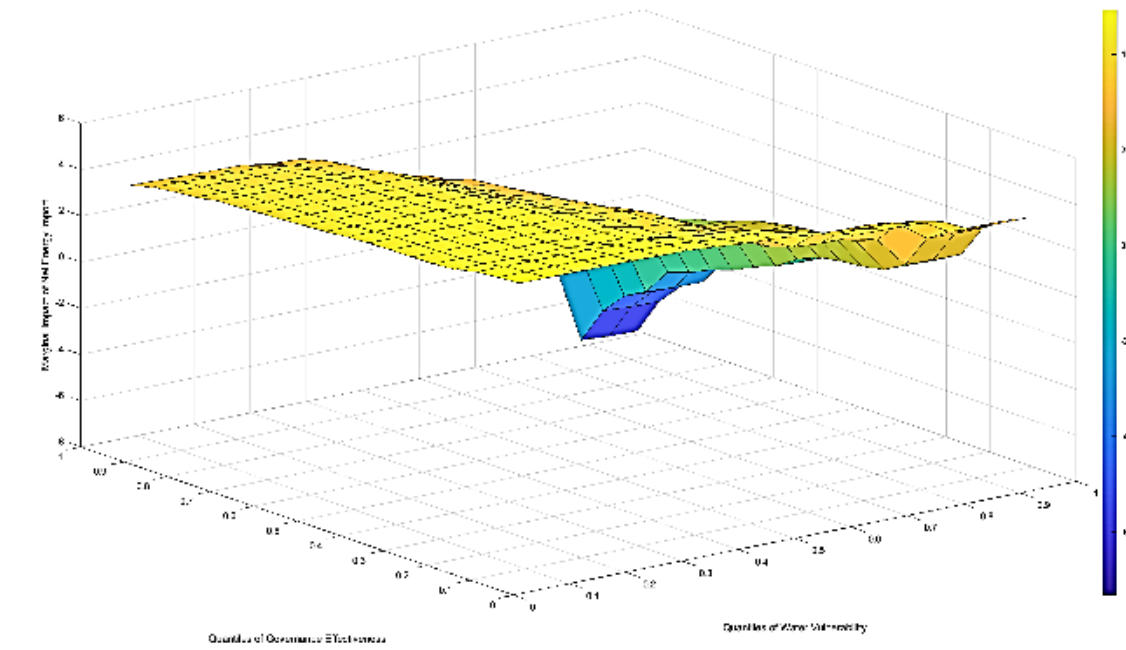

Governance Quality Indicator: Governance Effectiveness

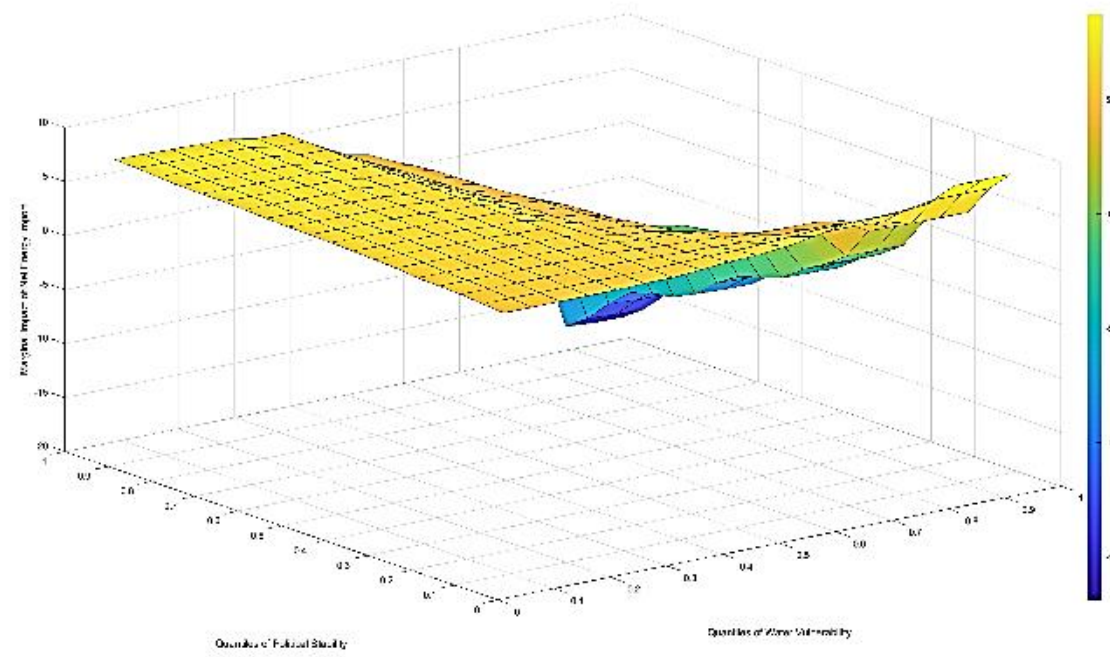

Governance Quality Indicator: Political Stability

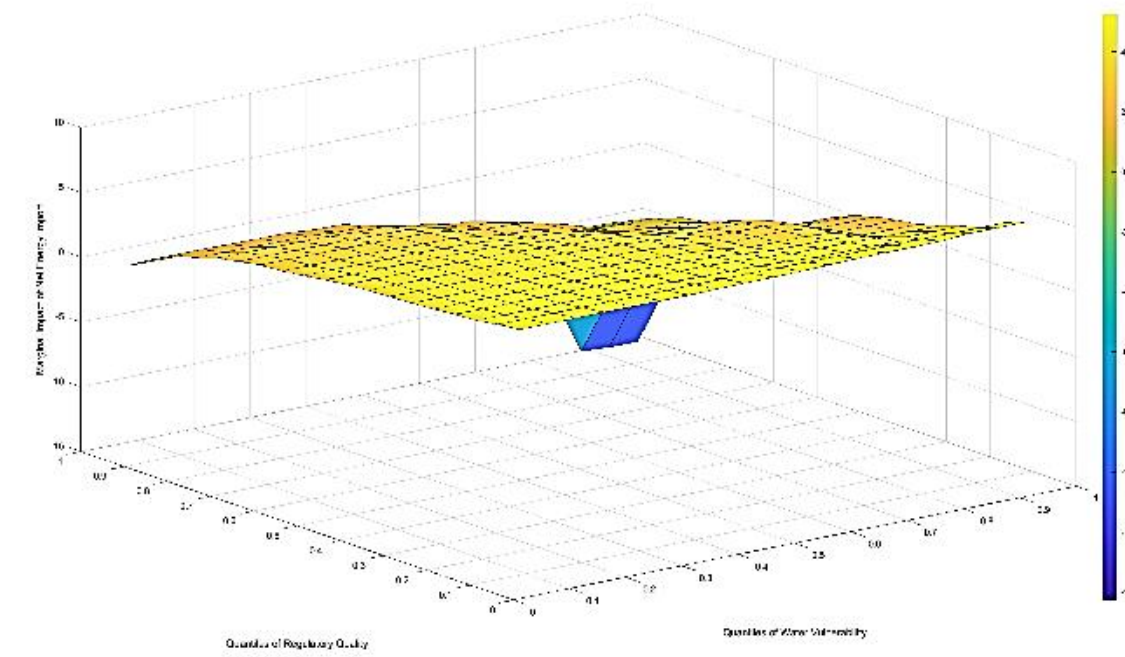

Governance Quality Indicator: Regulatory Quality

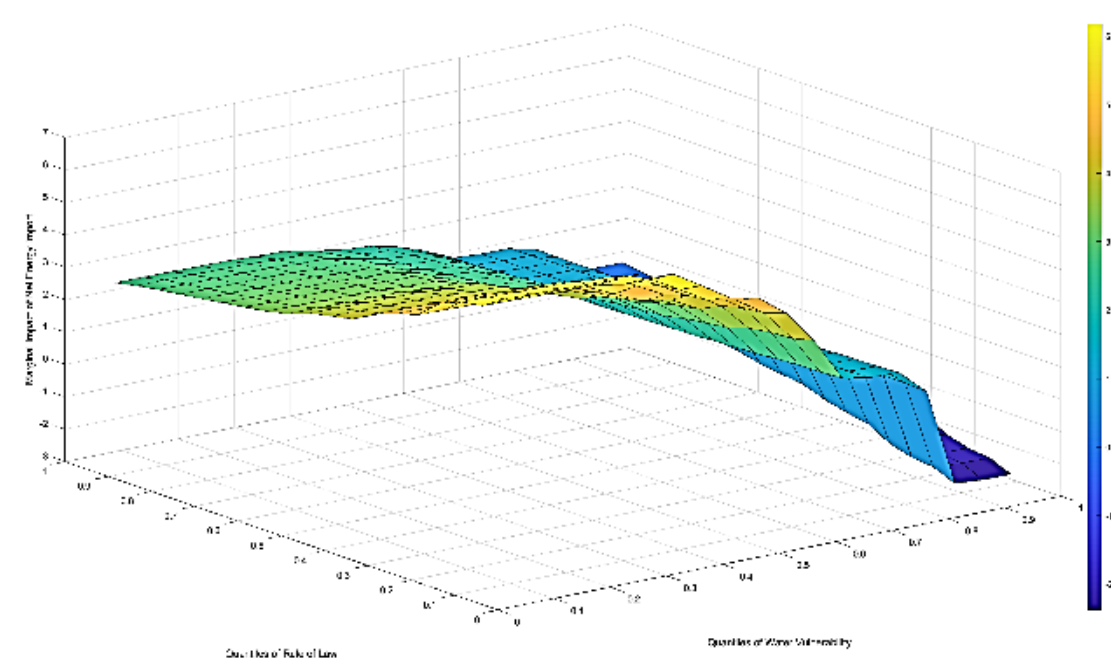

Governance Quality Indicator: Rule of Law

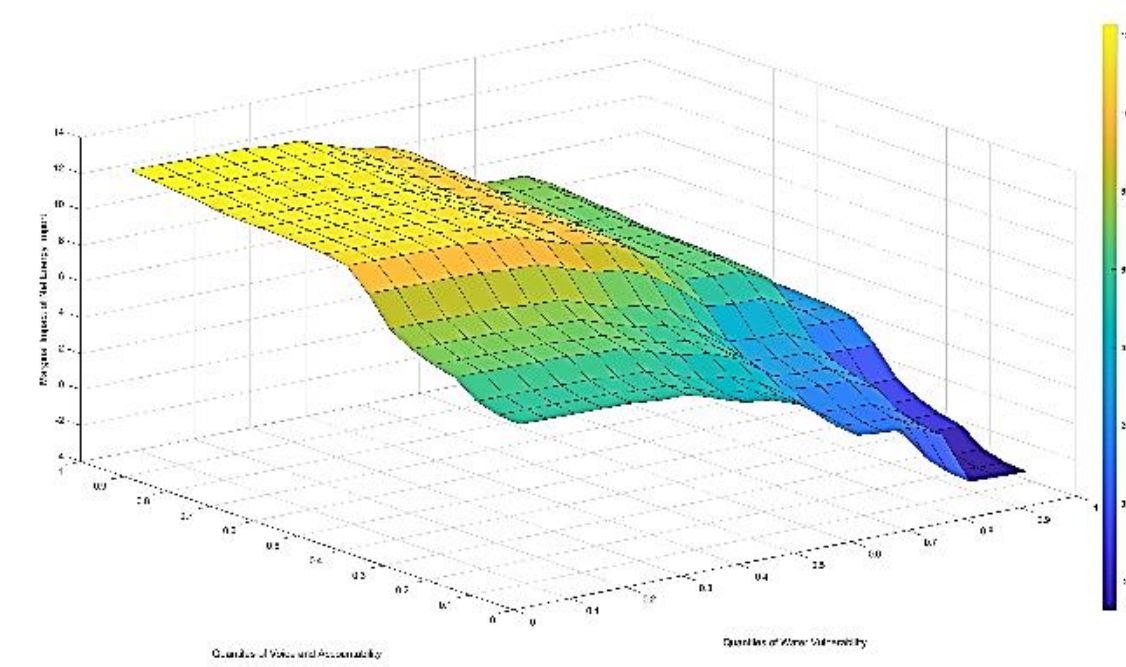

Governance Quality Indicator: Voice and Accountability

Figure S77: BRICS Policy Simulation Outcomes for the Marginal Impact of Net Energy Import on SDG 7.2.1 under Water Vulnerability and Governance Quality, Related to Figure 4-5 and Table 2

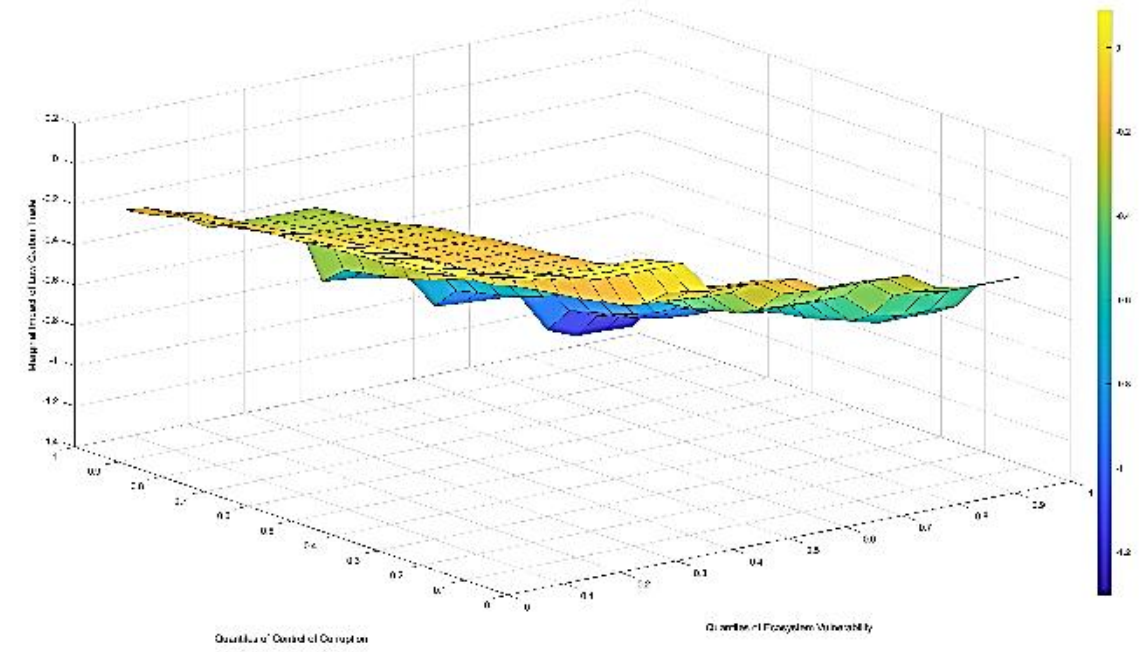

Governance Quality Indicator: Control of Corruption

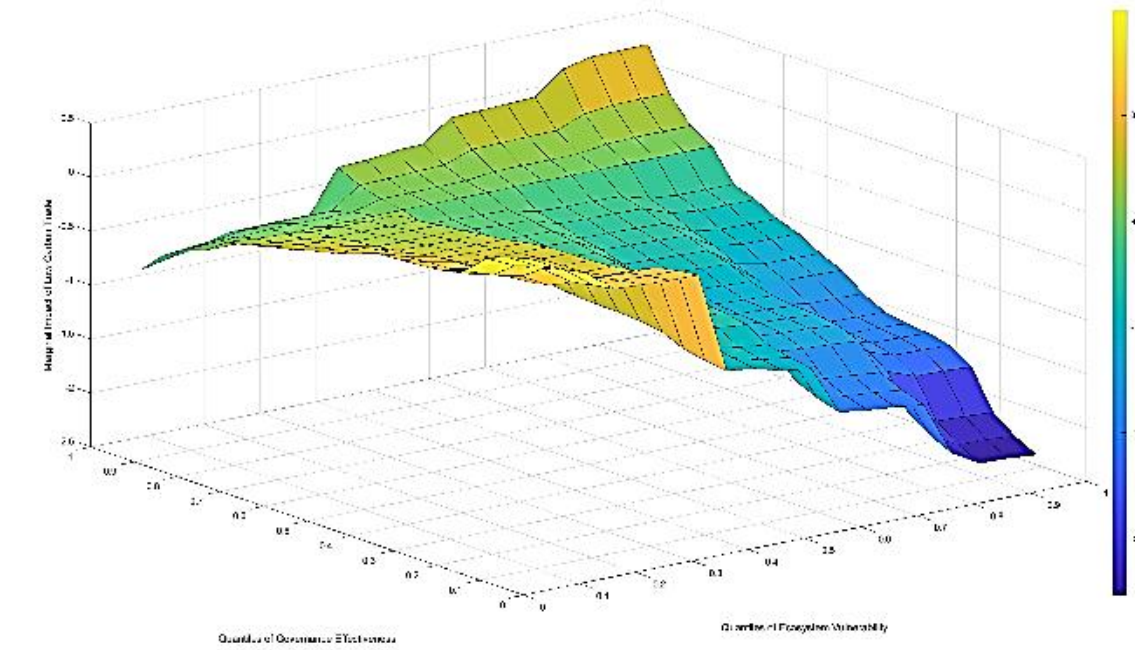

Governance Quality Indicator: Governance Effectiveness

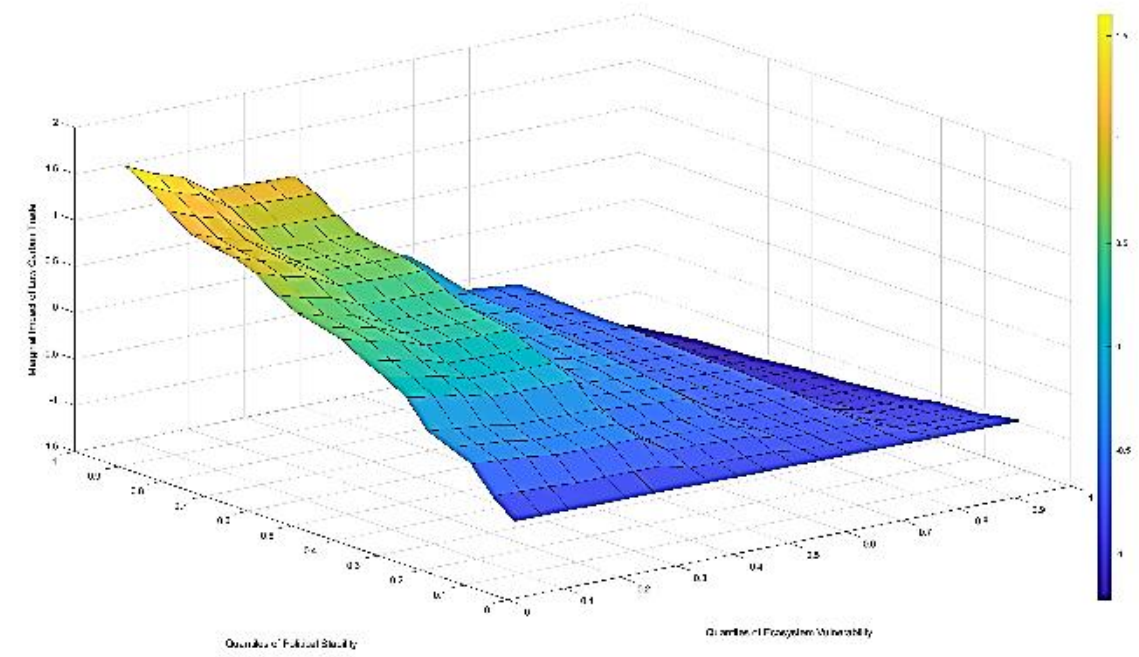

Governance Quality Indicator: Political Stability

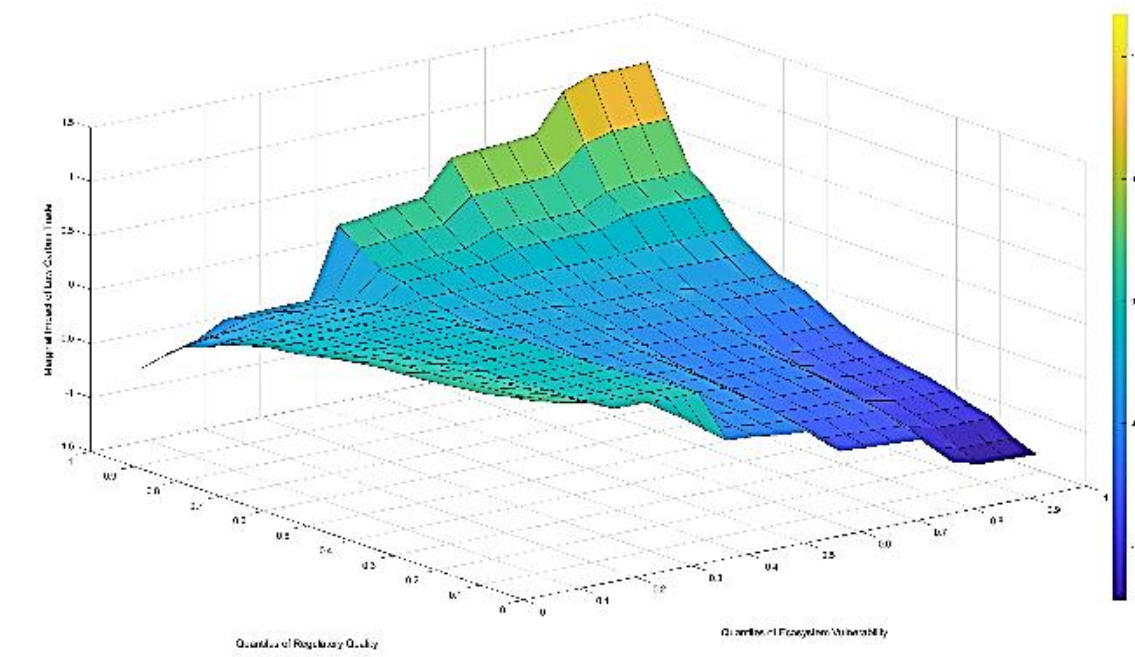

Governance Quality Indicator: Regulatory Quality

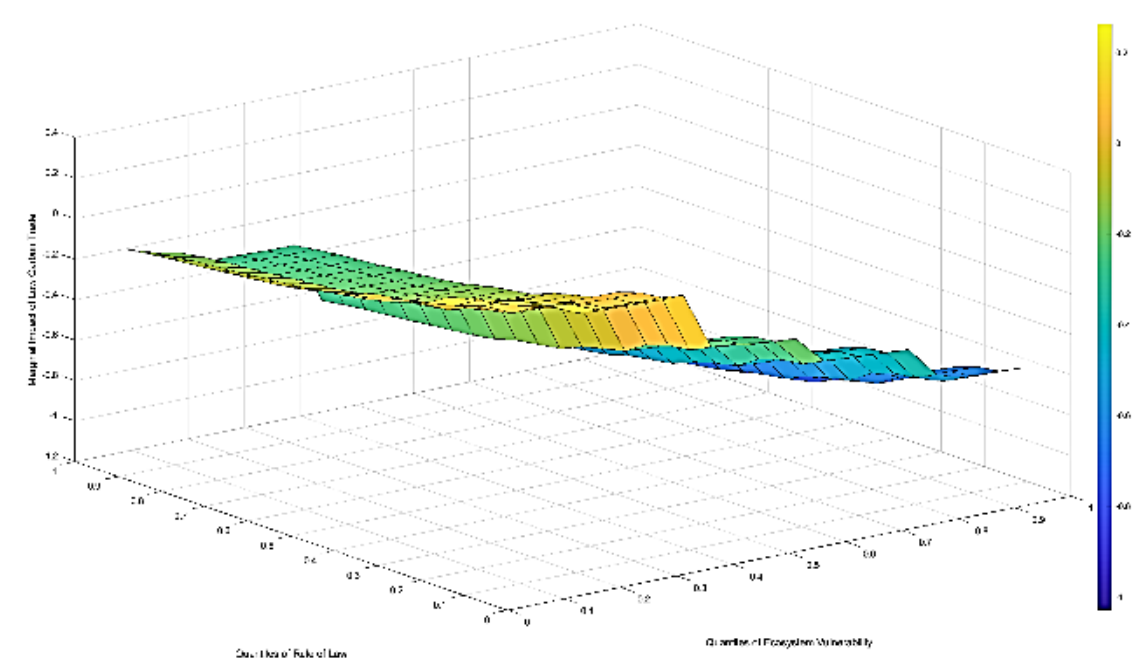

Governance Quality Indicator: Rule of Law

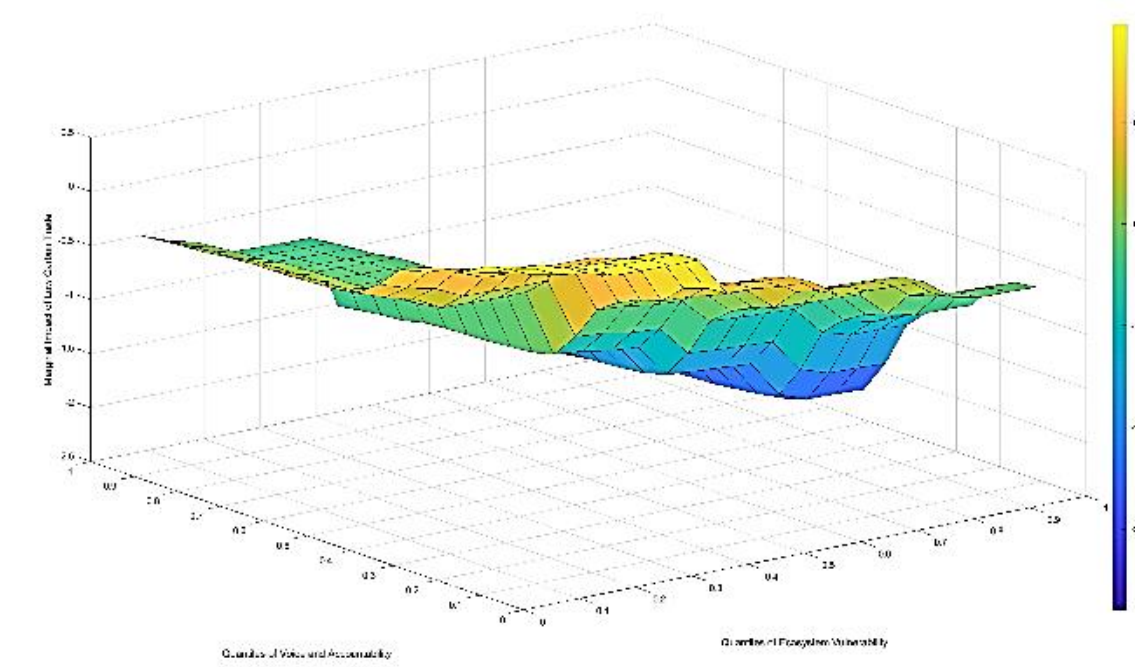

Governance Quality Indicator: Voice and Accountability

Figure S78: BRICS Policy Simulation Outcomes for the Marginal Impact of Low Carbon Trade on SDG 7.2.1 under Ecosystem Vulnerability and Governance Quality, Related to Figure 4-5 and Table 2

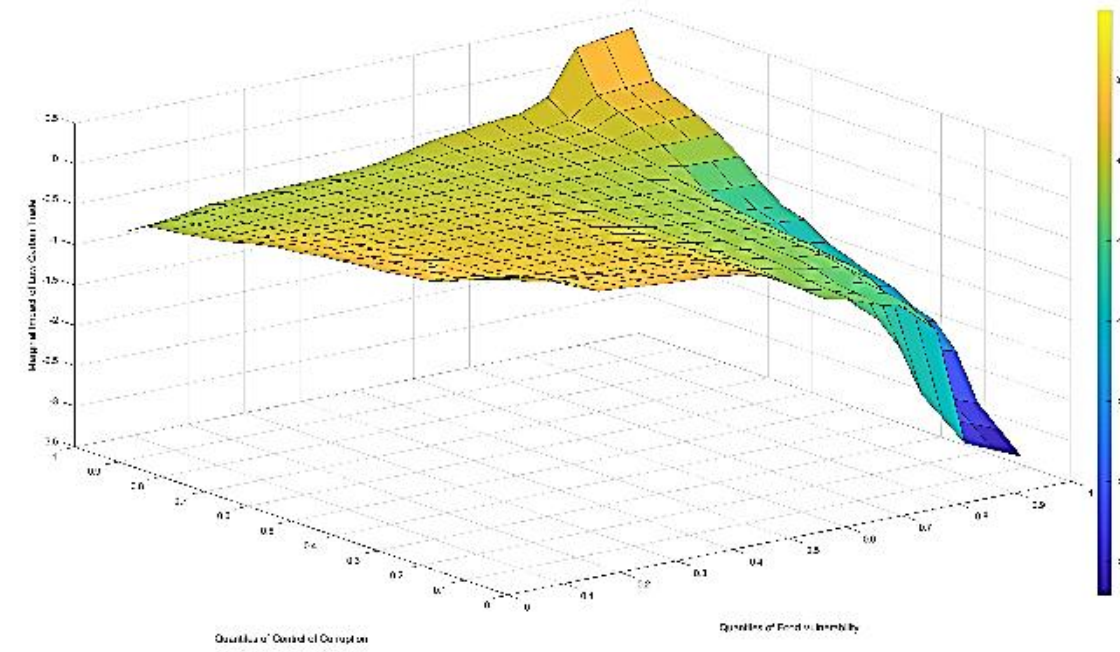

Governance Quality Indicator: Control of Corruption

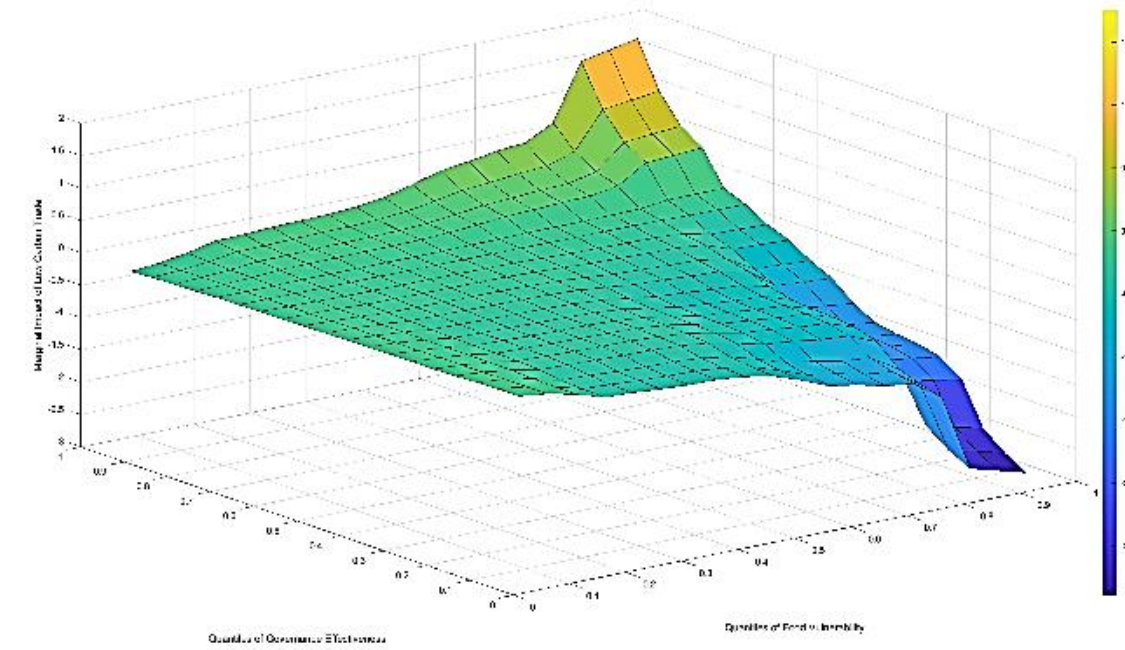

Governance Quality Indicator: Governance Effectiveness

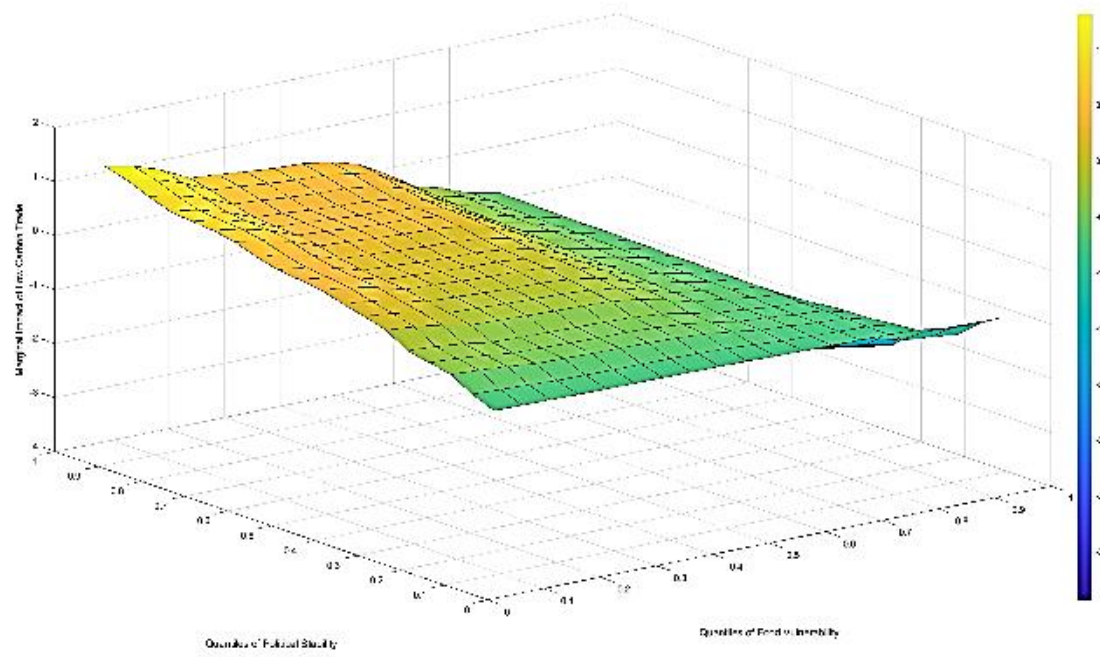

Governance Quality Indicator: Political Stability

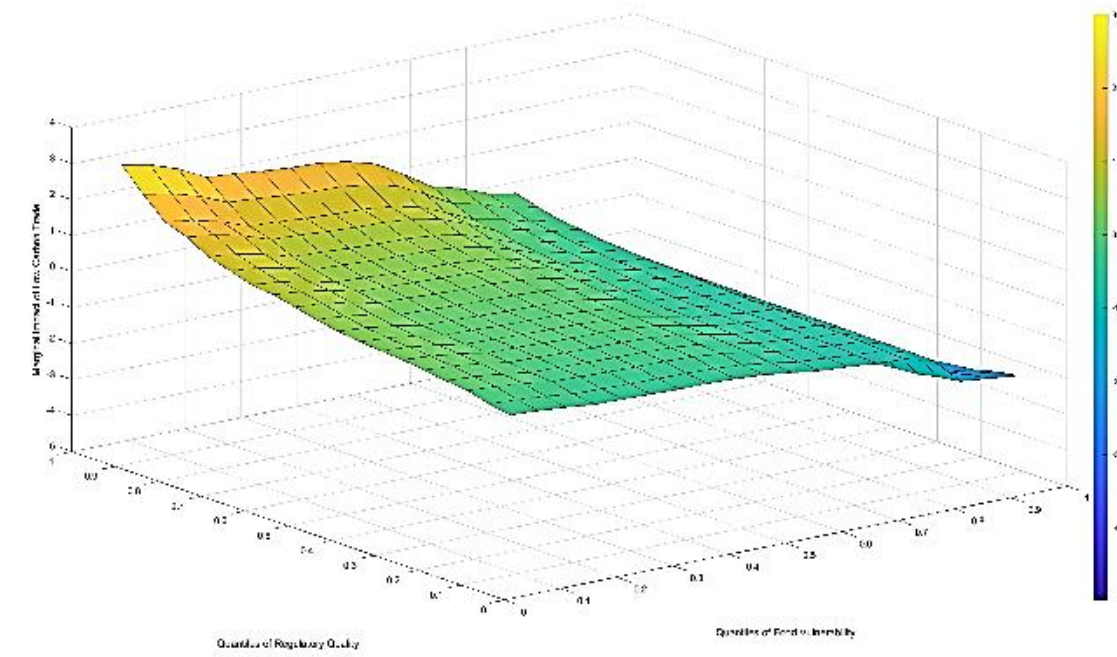

Governance Quality Indicator: Regulatory Quality

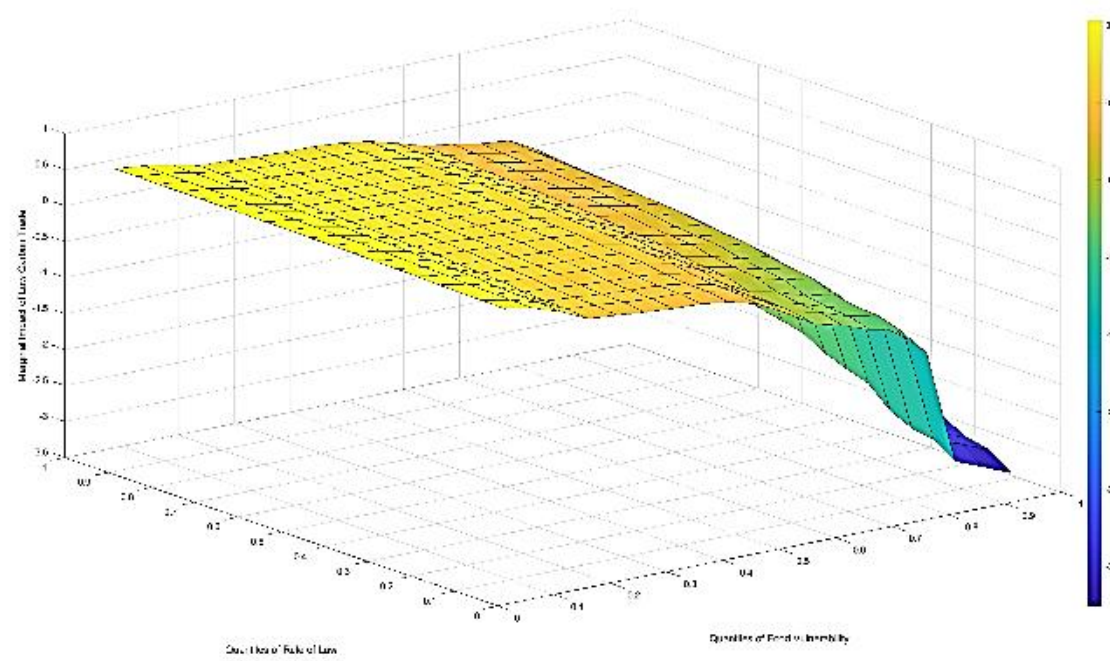

Governance Quality Indicator: Rule of Law

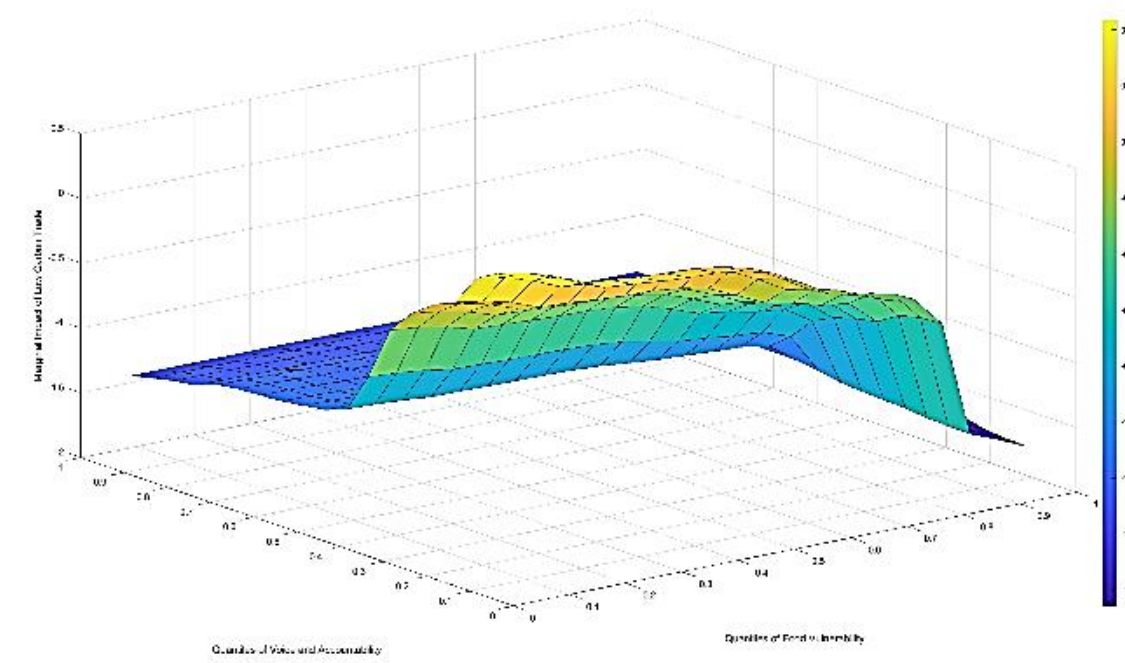

Governance Quality Indicator: Voice and Accountability

Figure S79: BRICS Policy Simulation Outcomes for the Marginal Impact of Low Carbon Trade on SDG 7.2.1 under Food Vulnerability and Governance Quality, Related to Figure 4-5 and Table 2

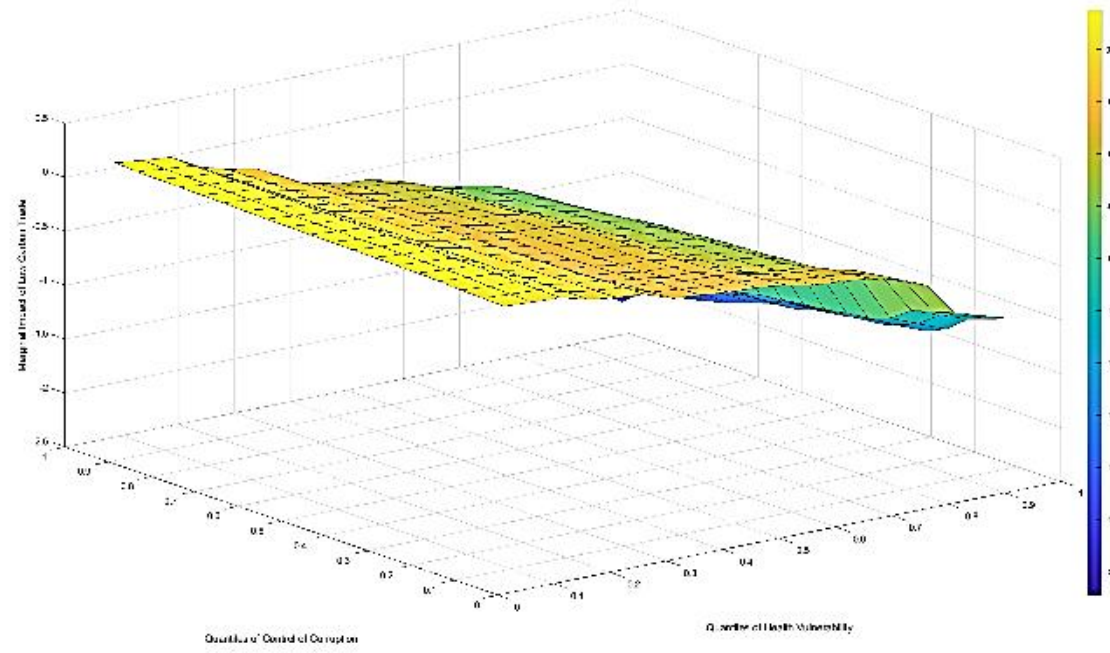

Governance Quality Indicator: Control of Corruption

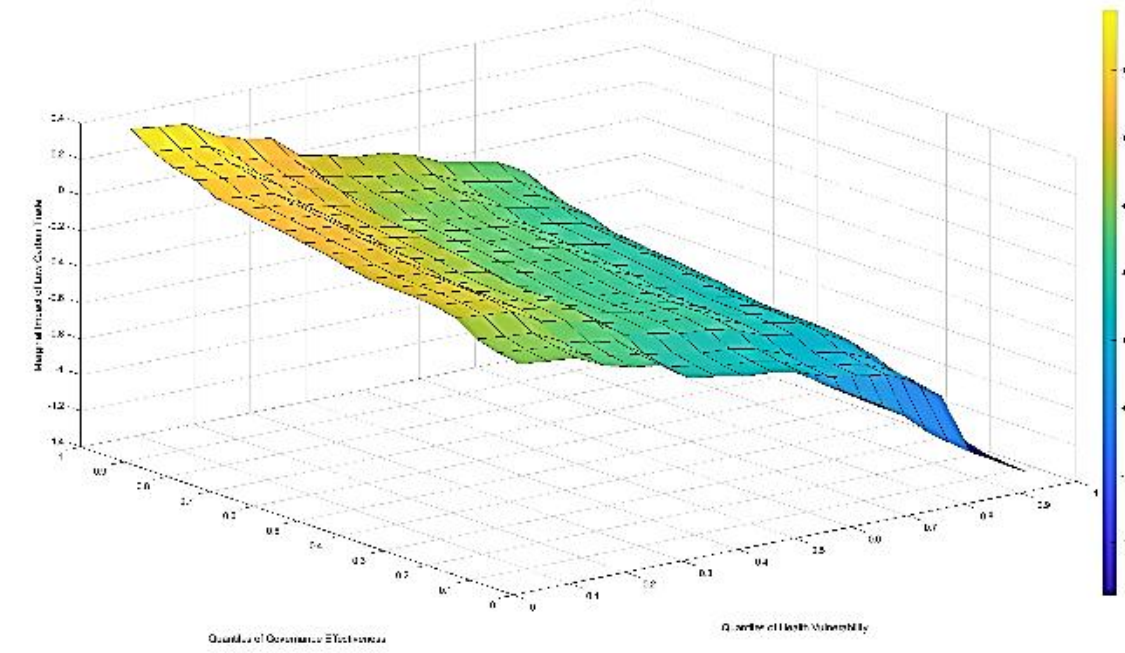

Governance Quality Indicator: Governance Effectiveness

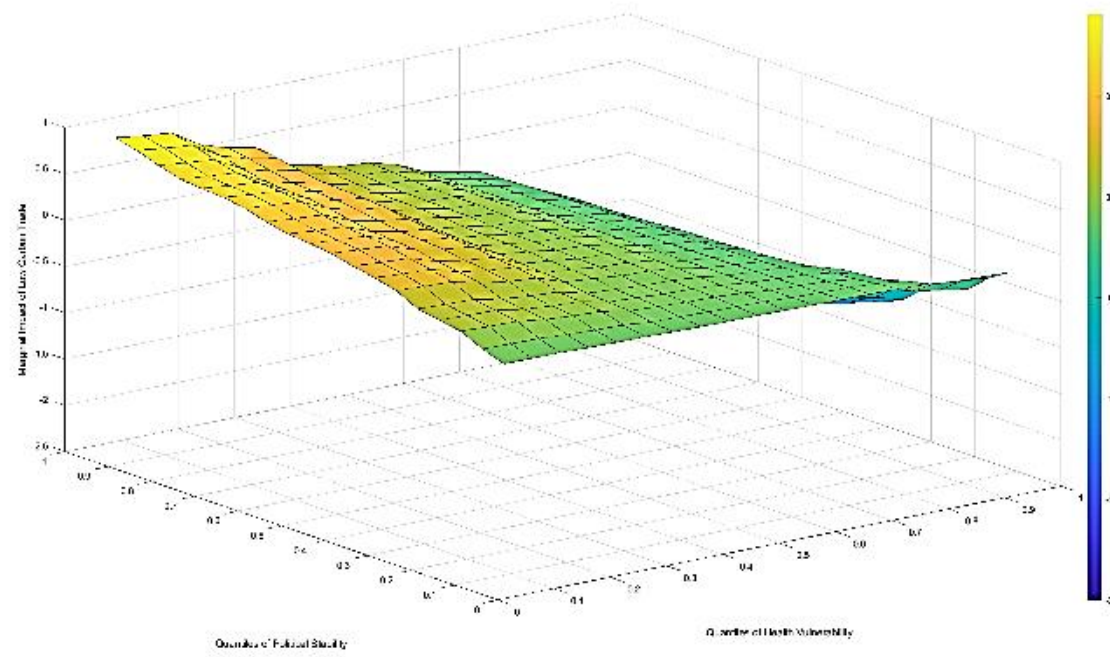

Governance Quality Indicator: Political Stability

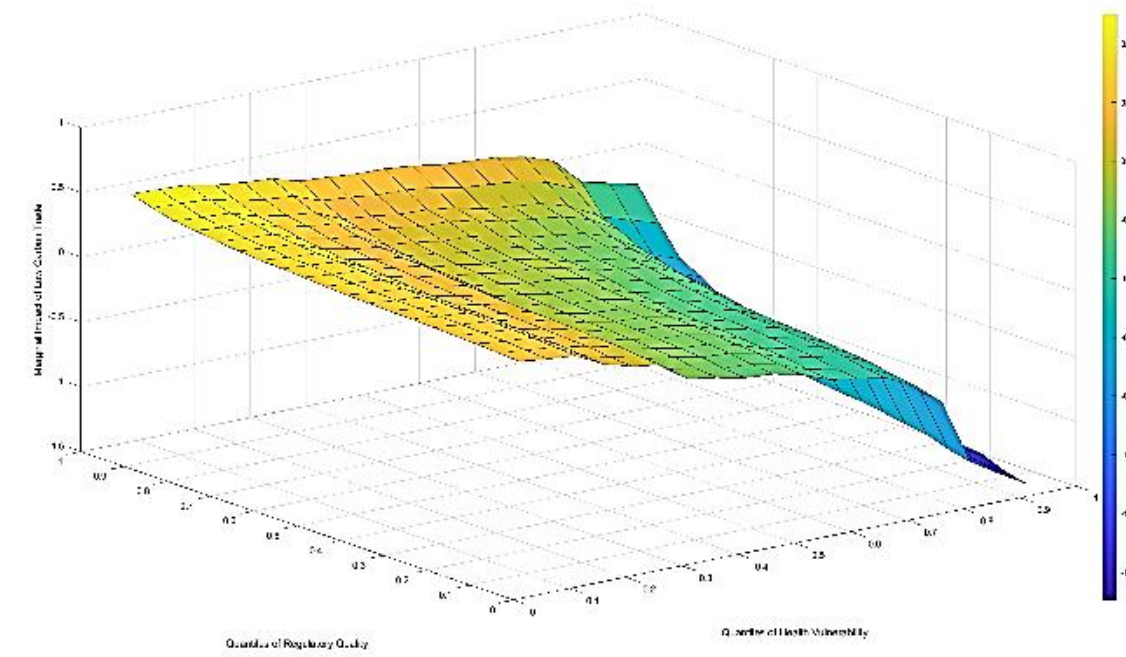

Governance Quality Indicator: Regulatory Quality

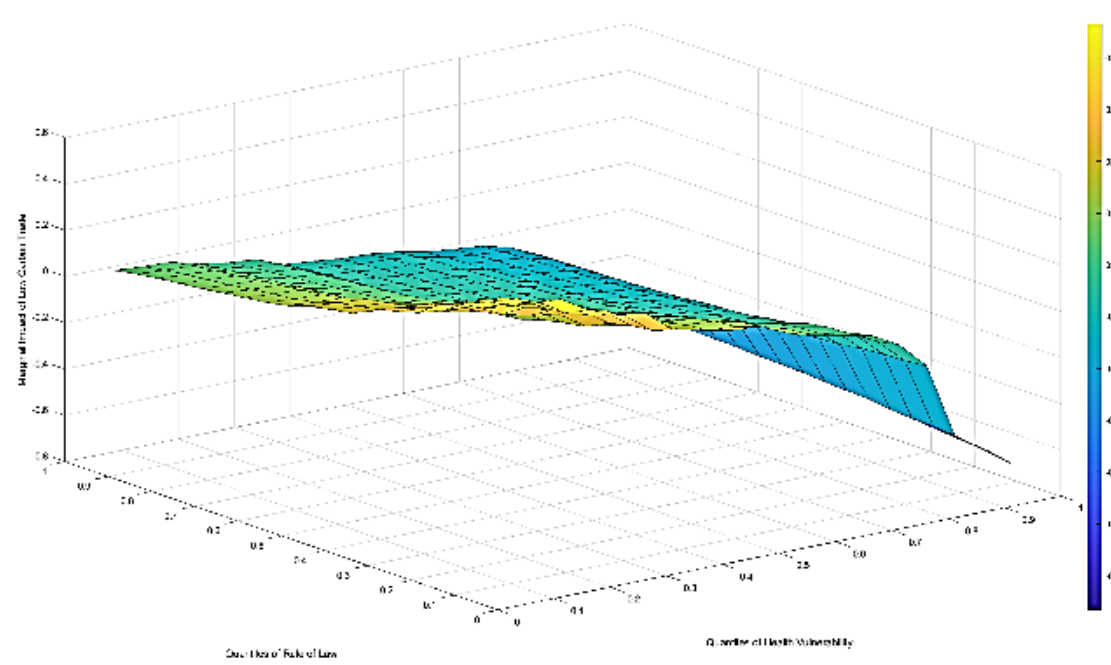

Governance Quality Indicator: Rule of Law

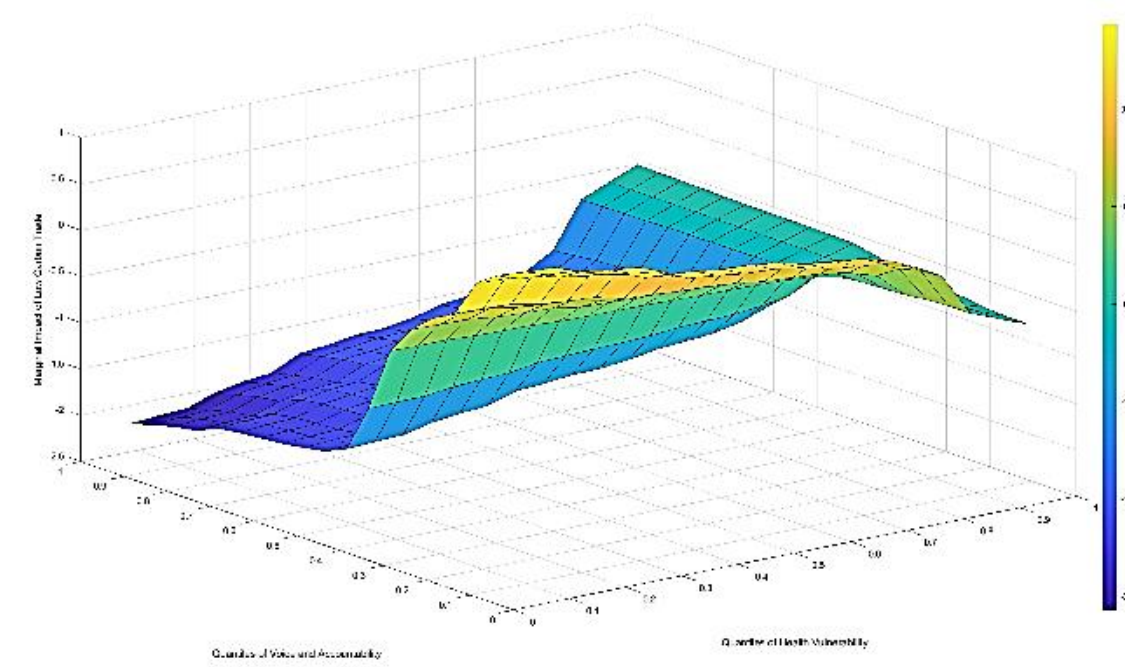

Governance Quality Indicator: Voice and Accountability

Figure S80: BRICS Policy Simulation Outcomes for the Marginal Impact of Low Carbon Trade on SDG 7.2.1 under Health Vulnerability and Governance Quality, Related to Figure 4-5 and Table 2

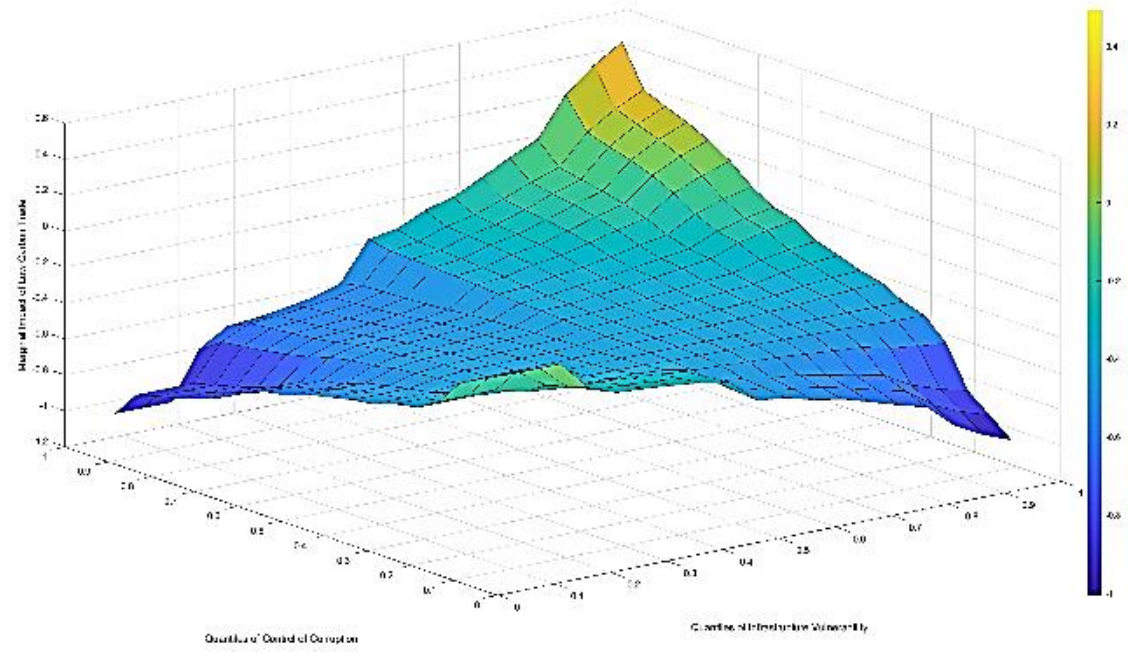

Governance Quality Indicator: Control of Corruption

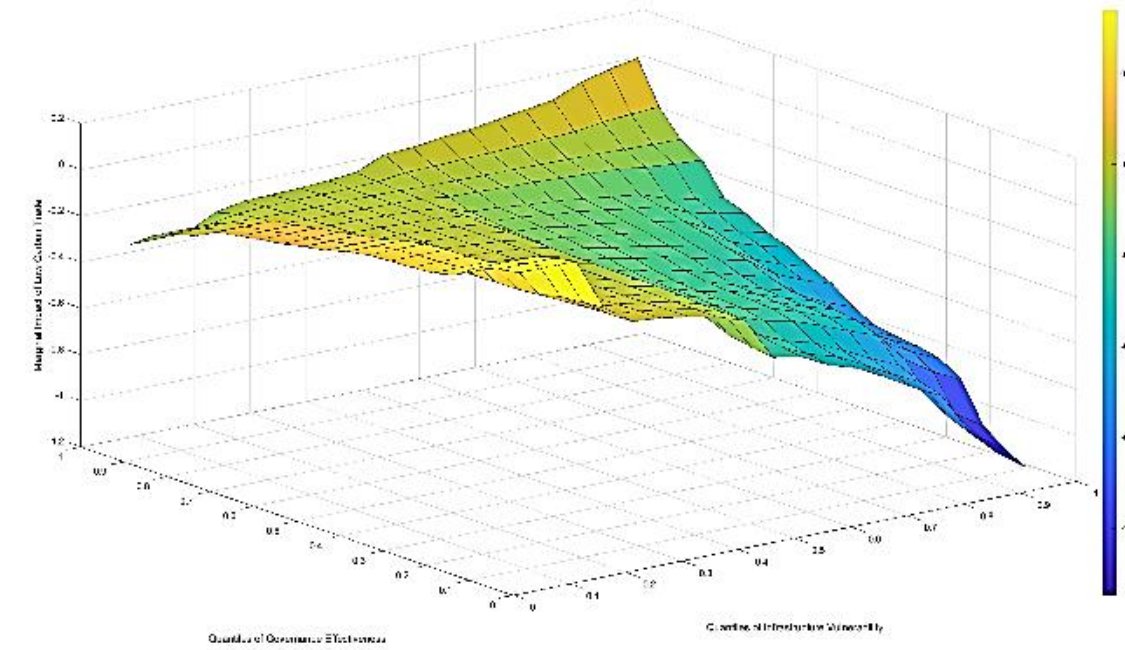

Governance Quality Indicator: Governance Effectiveness

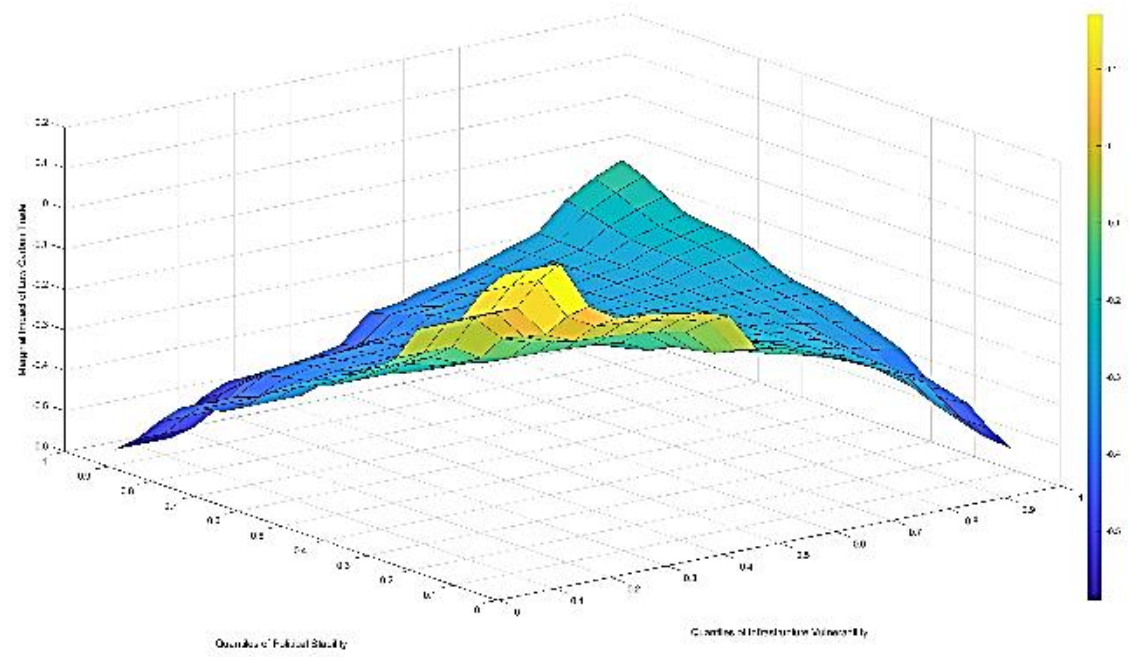

Governance Quality Indicator: Political Stability

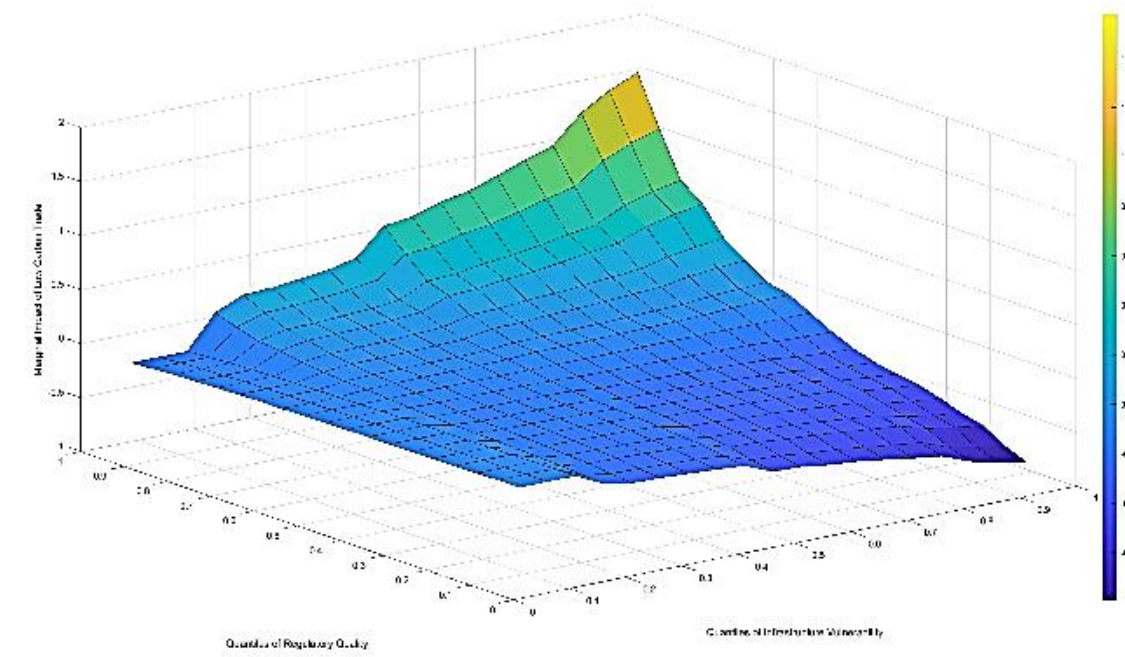

Governance Quality Indicator: Regulatory Quality

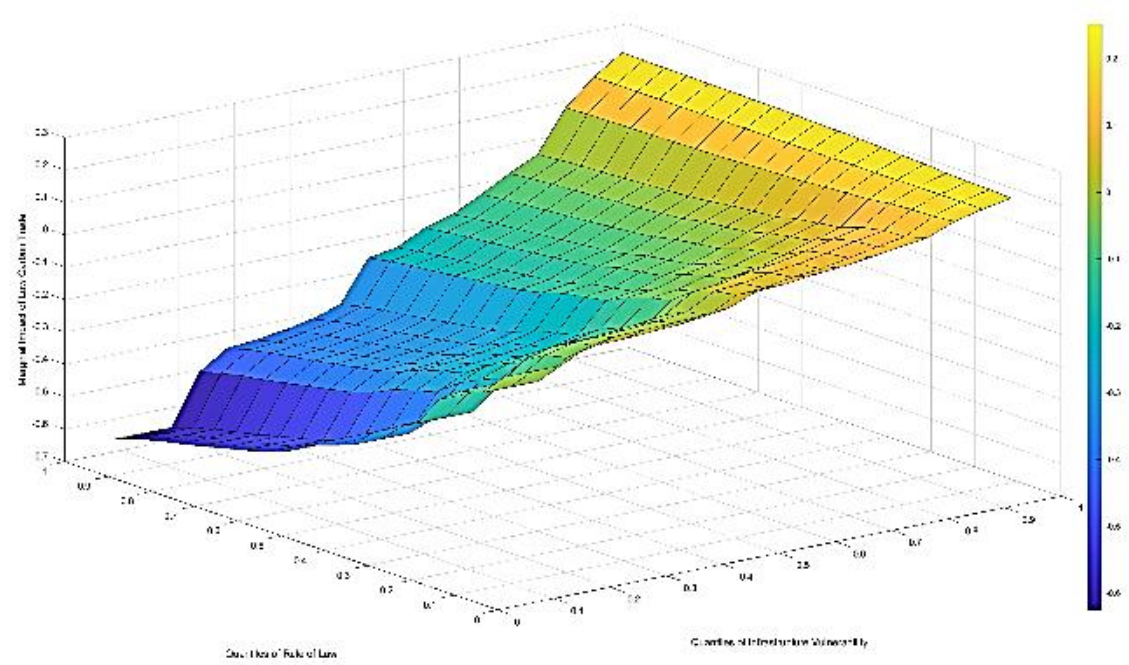

Governance Quality Indicator: Rule of Law

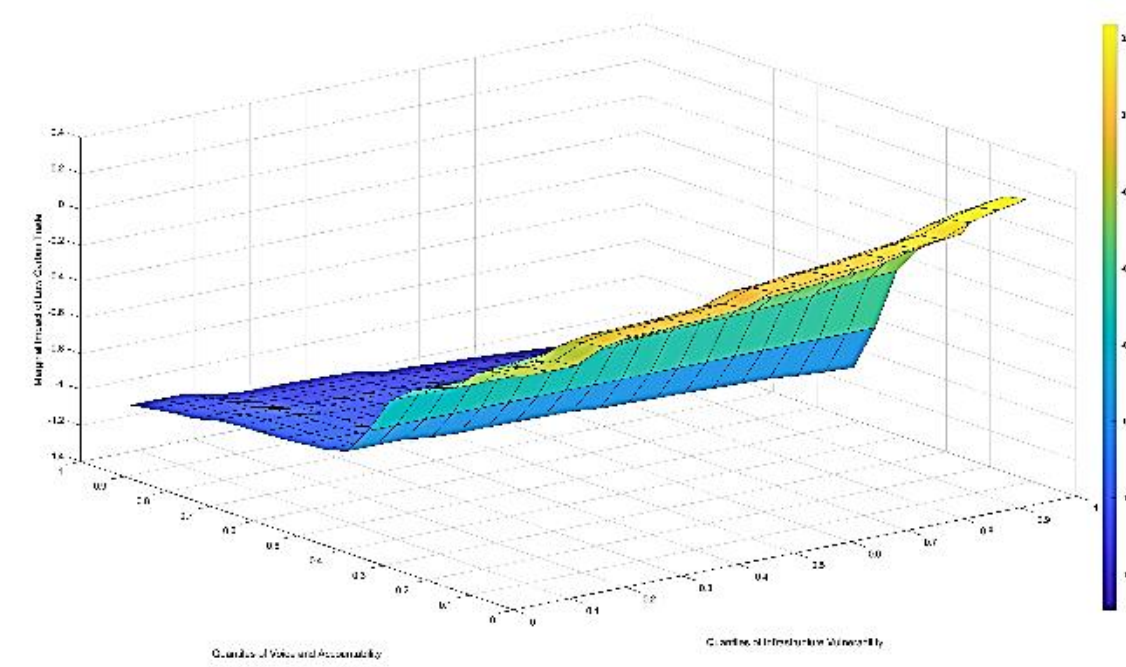

Governance Quality Indicator: Voice and Accountability

Figure S81: BRICS Policy Simulation Outcomes for the Marginal Impact of Low Carbon Trade on SDG 7.2.1 under Infrastructure Vulnerability and Governance Quality, Related to Figure 4-5 and Table 2

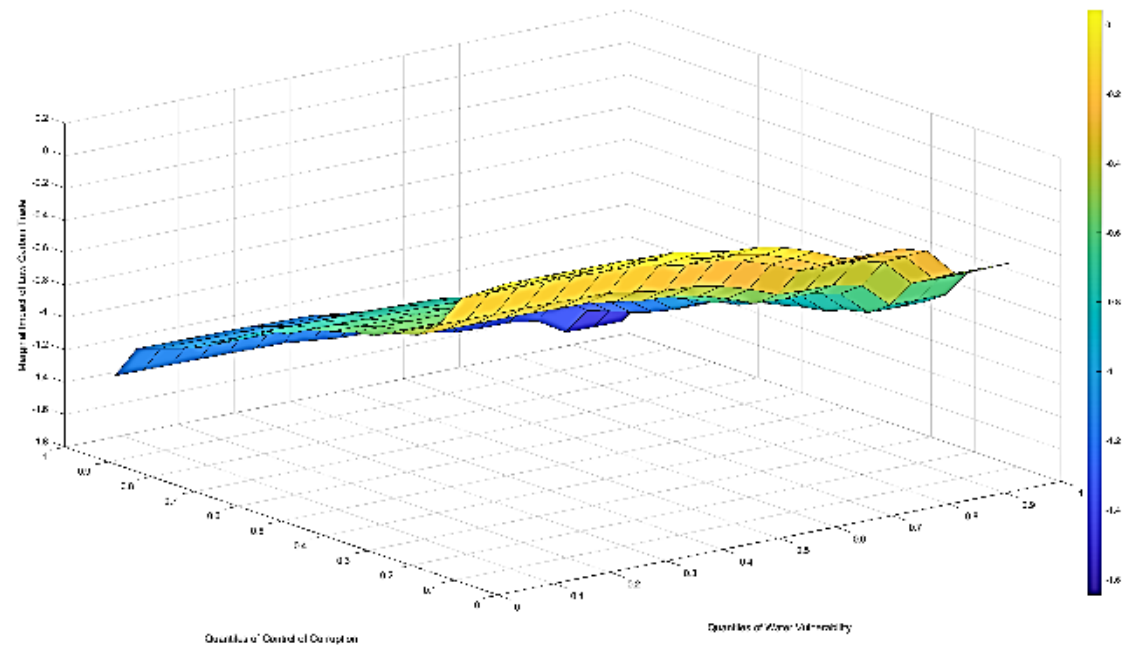

Governance Quality Indicator: Control of Corruption

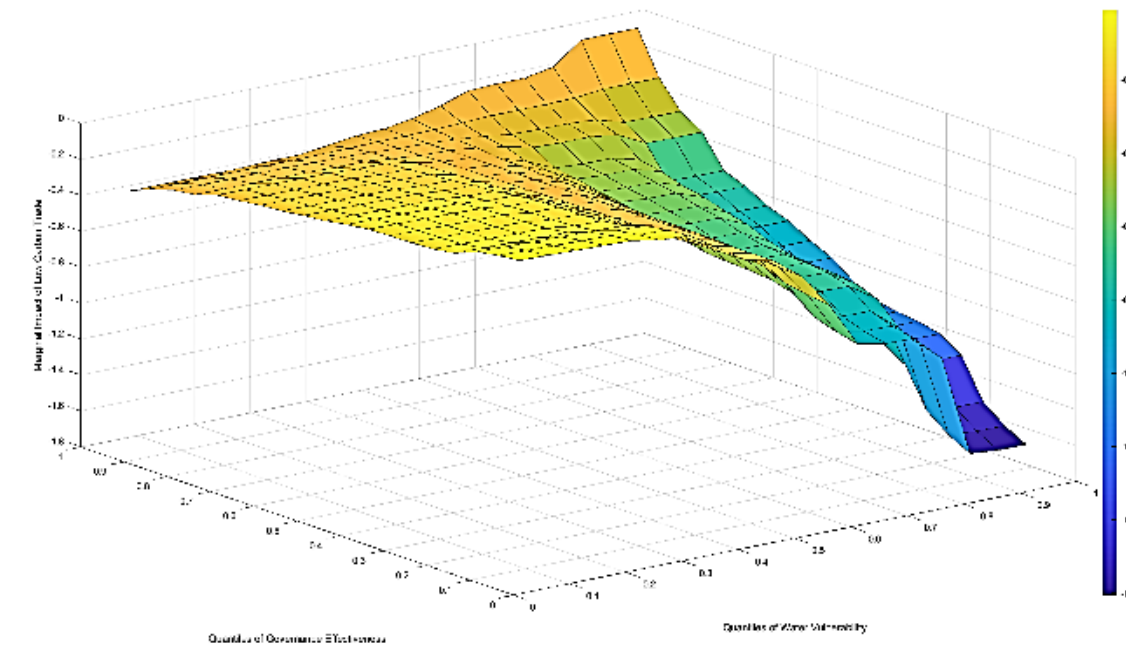

Governance Quality Indicator: Governance Effectiveness

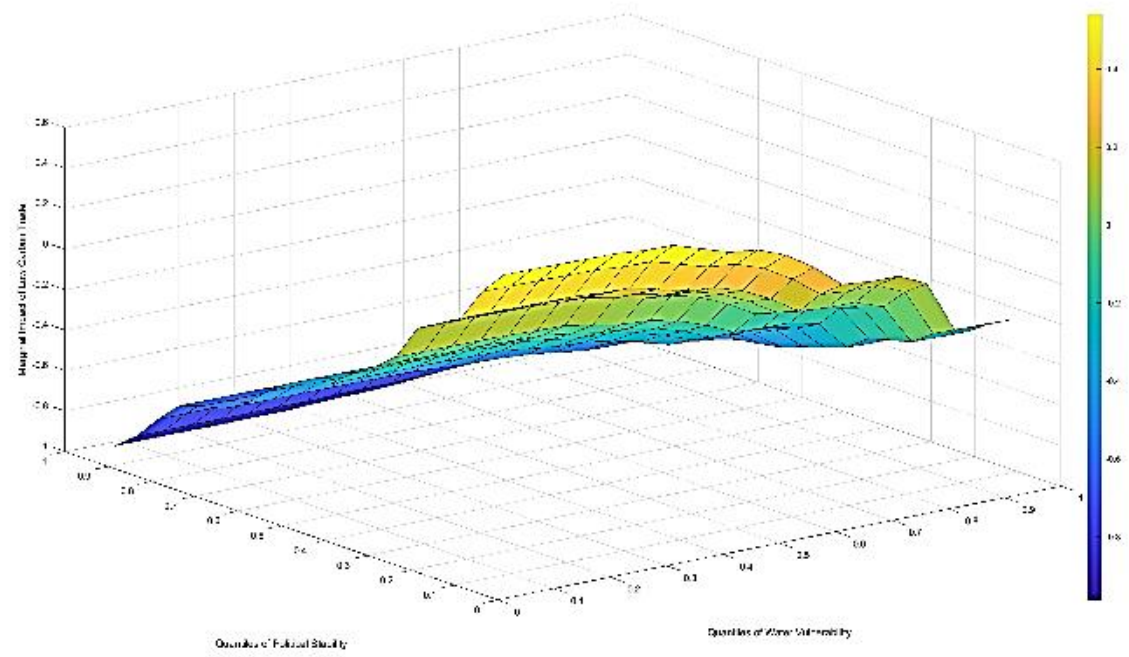

Governance Quality Indicator: Political Stability

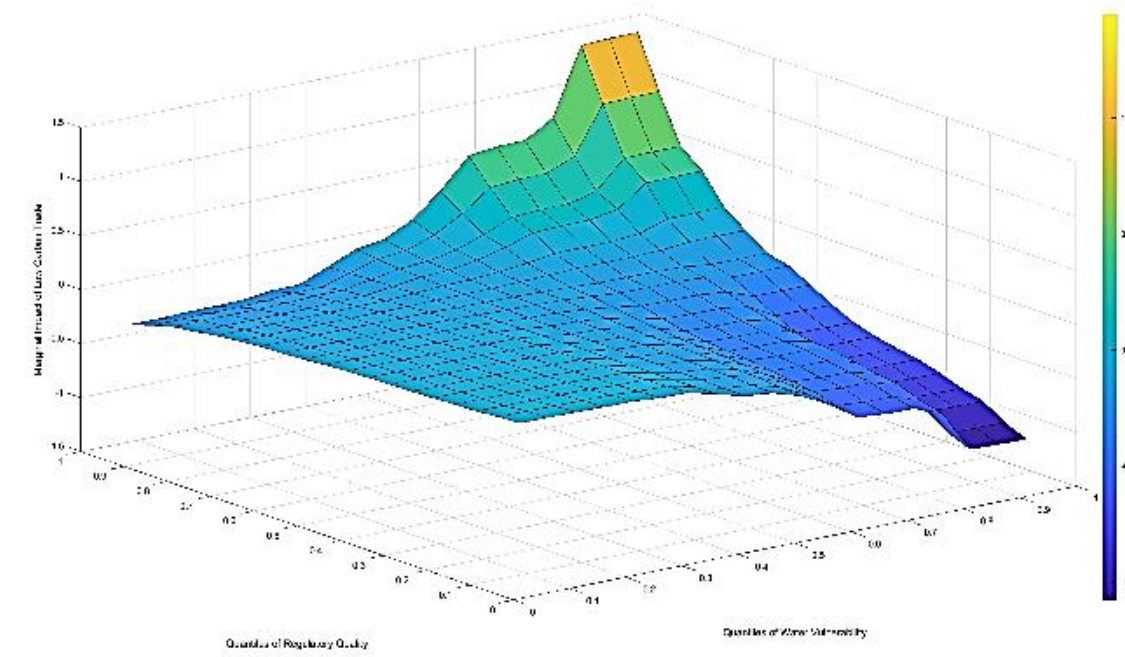

Governance Quality Indicator: Regulatory Quality

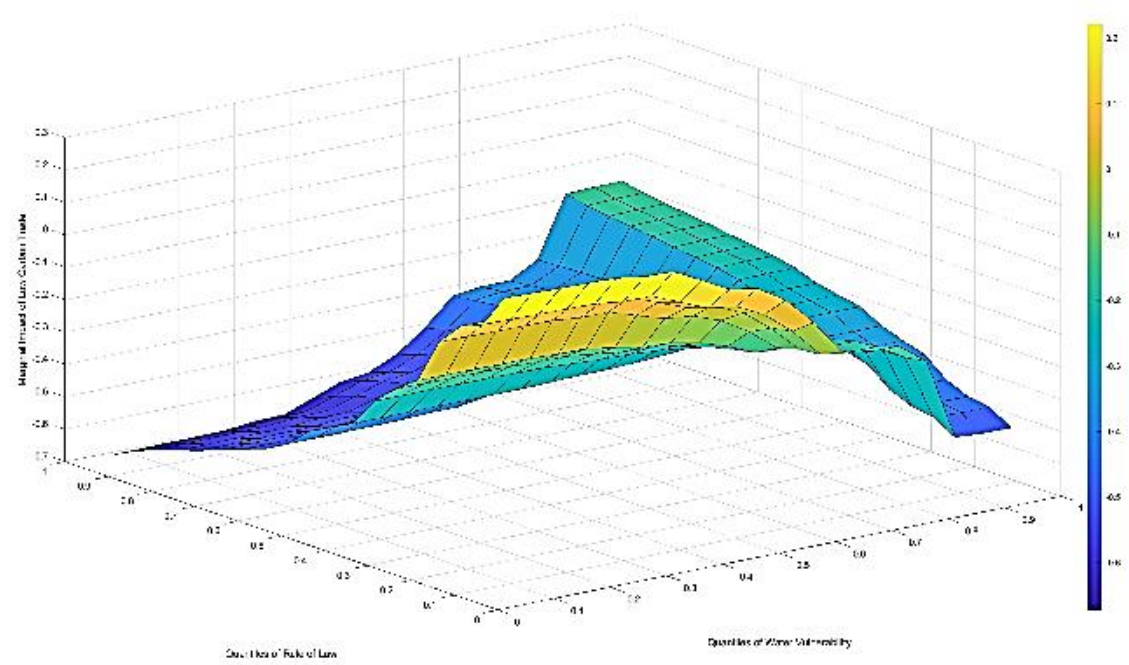

Governance Quality Indicator: Rule of Law

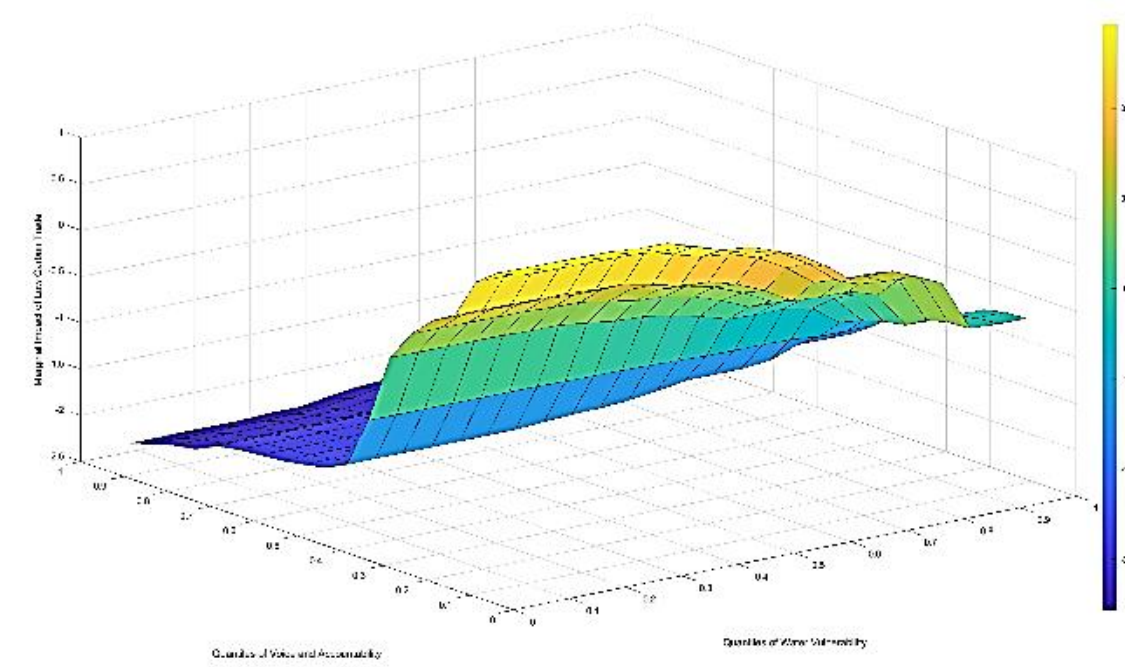

Governance Quality Indicator: Voice and Accountability

Figure S82: BRICS Policy Simulation Outcomes for the Marginal Impact of Low Carbon Trade on SDG 7.2.1 under Water Vulnerability and Governance Quality, Related to Figure 4-5 and Table 2

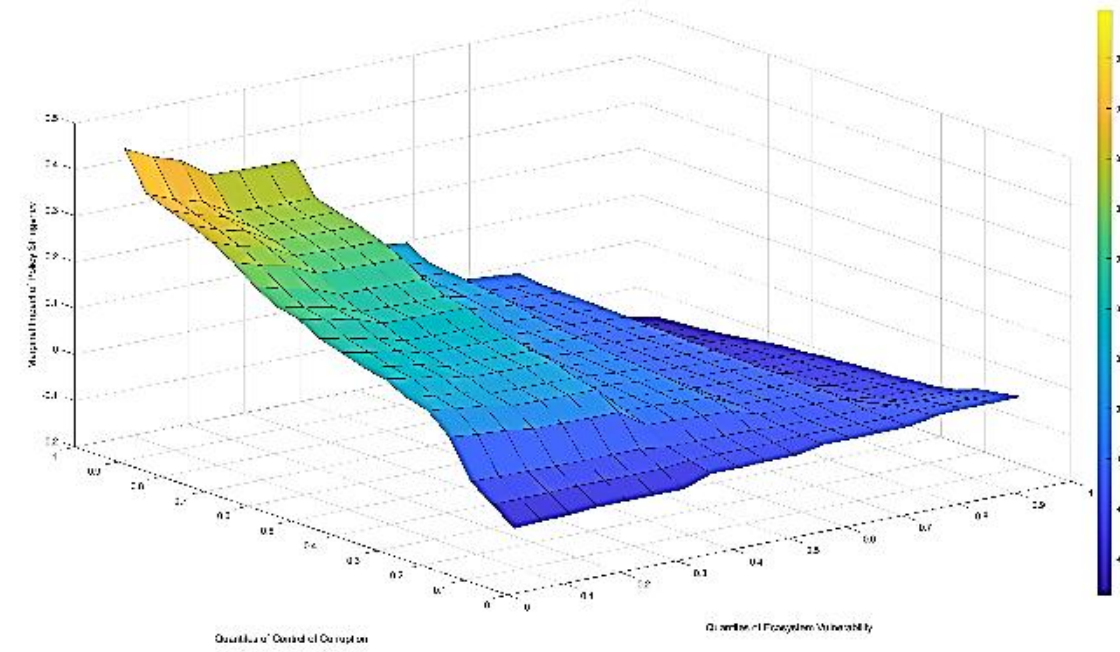

Governance Quality Indicator: Control of Corruption

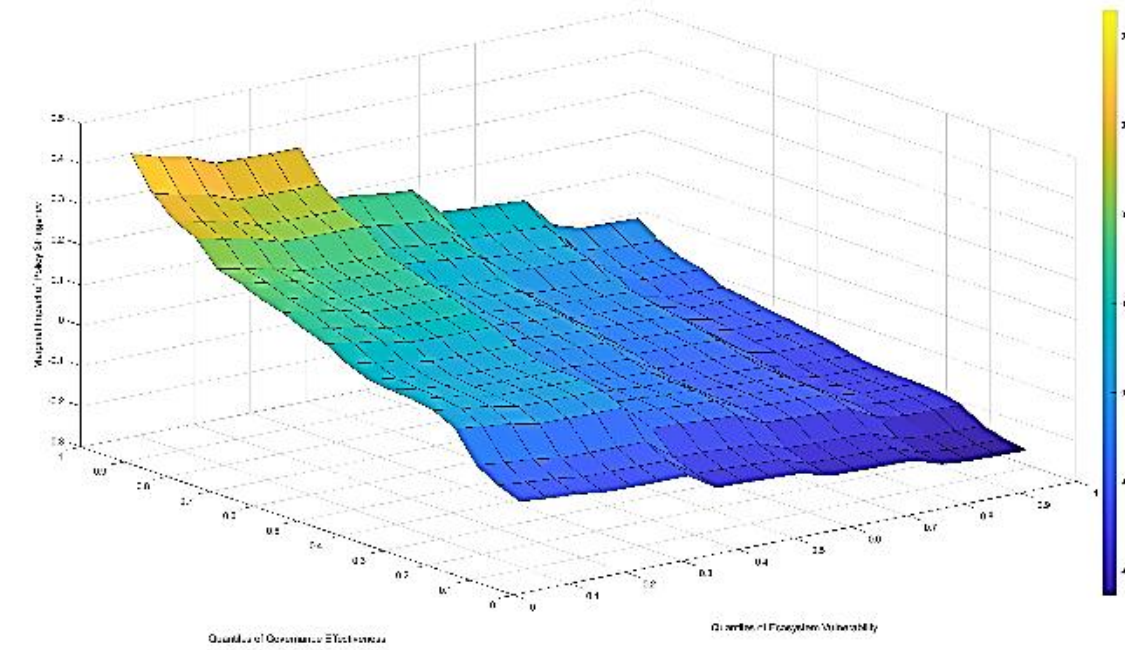

Governance Quality Indicator: Governance Effectiveness

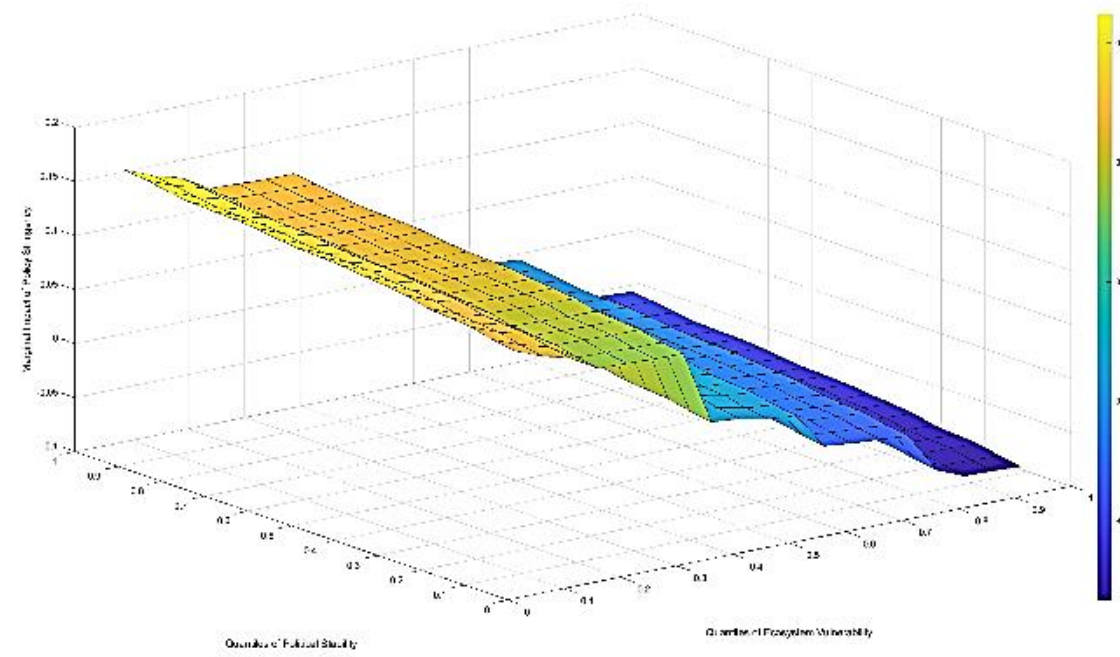

Governance Quality Indicator: Political Stability

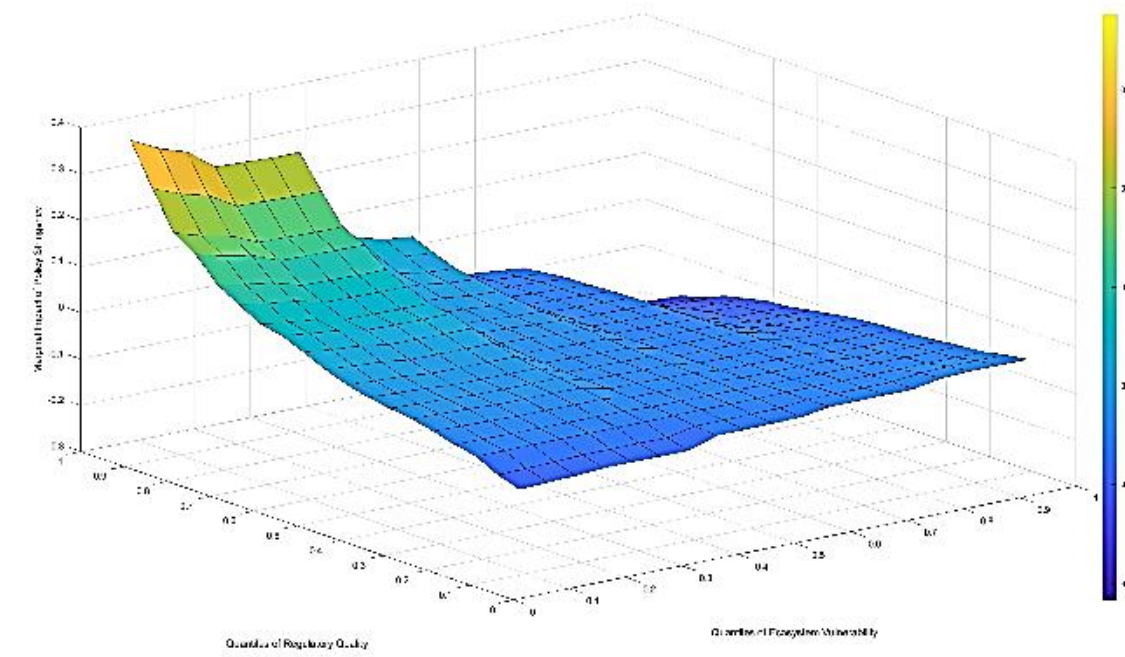

Governance Quality Indicator: Regulatory Quality

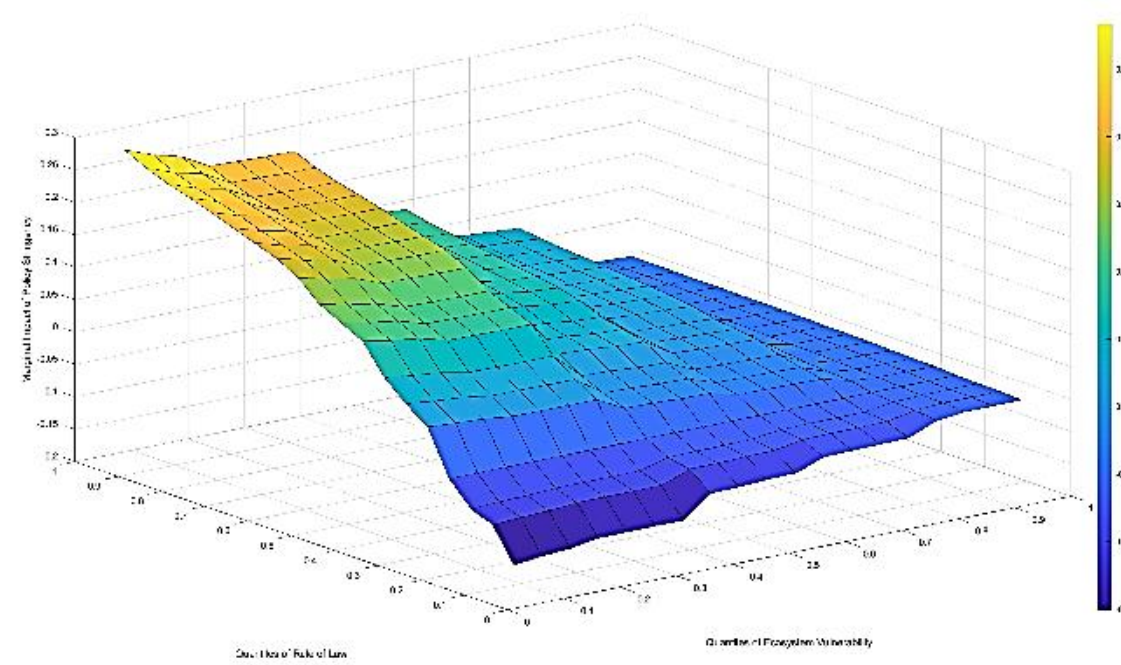

Governance Quality Indicator: Rule of Law

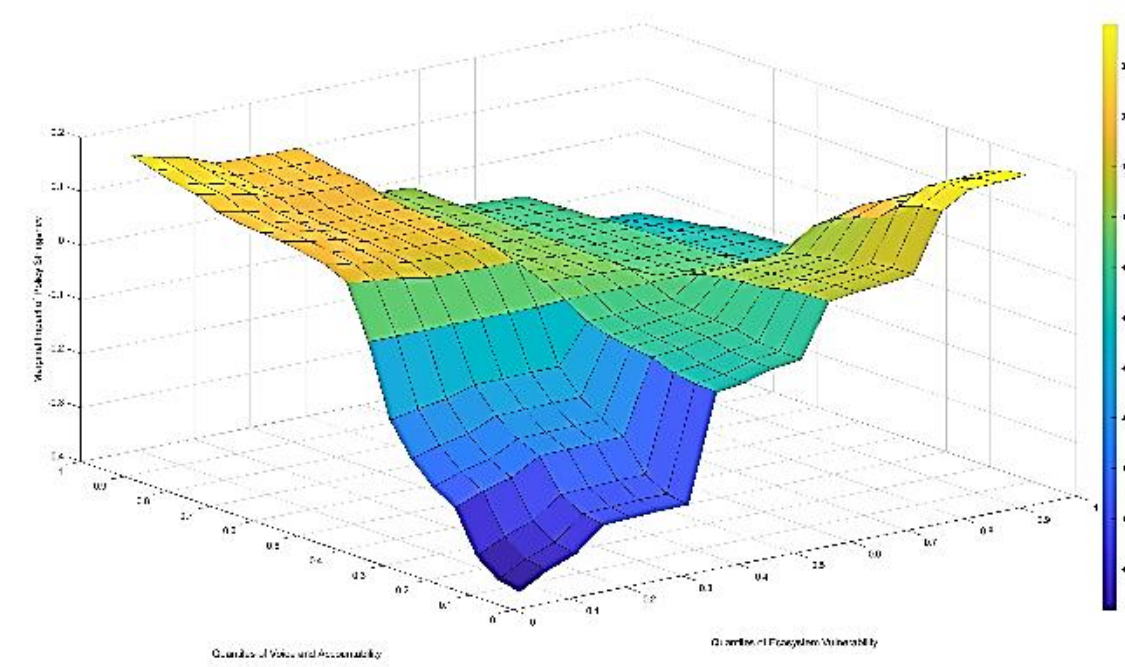

Governance Quality Indicator: Voice and Accountability

Figure S83: BRICS Policy Simulation Outcomes for the Marginal Impact of Policy Stringency on SDG 7.3.1 under Ecosystem Vulnerability and Governance Quality, Related to Figure 4-5 and Table 2

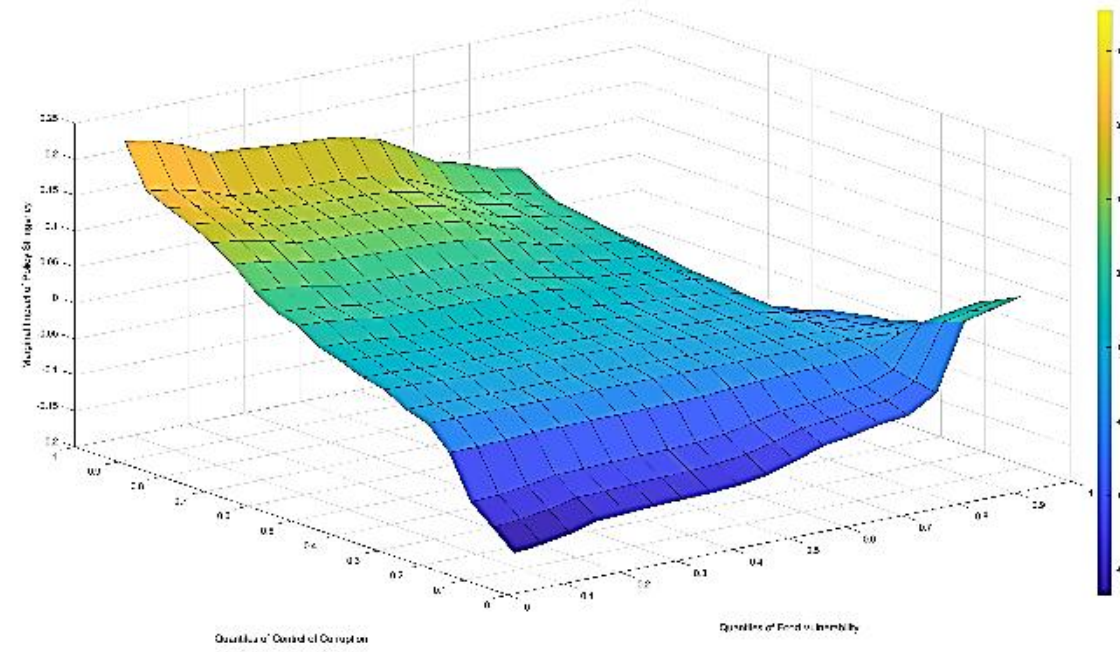

Governance Quality Indicator: Control of Corruption

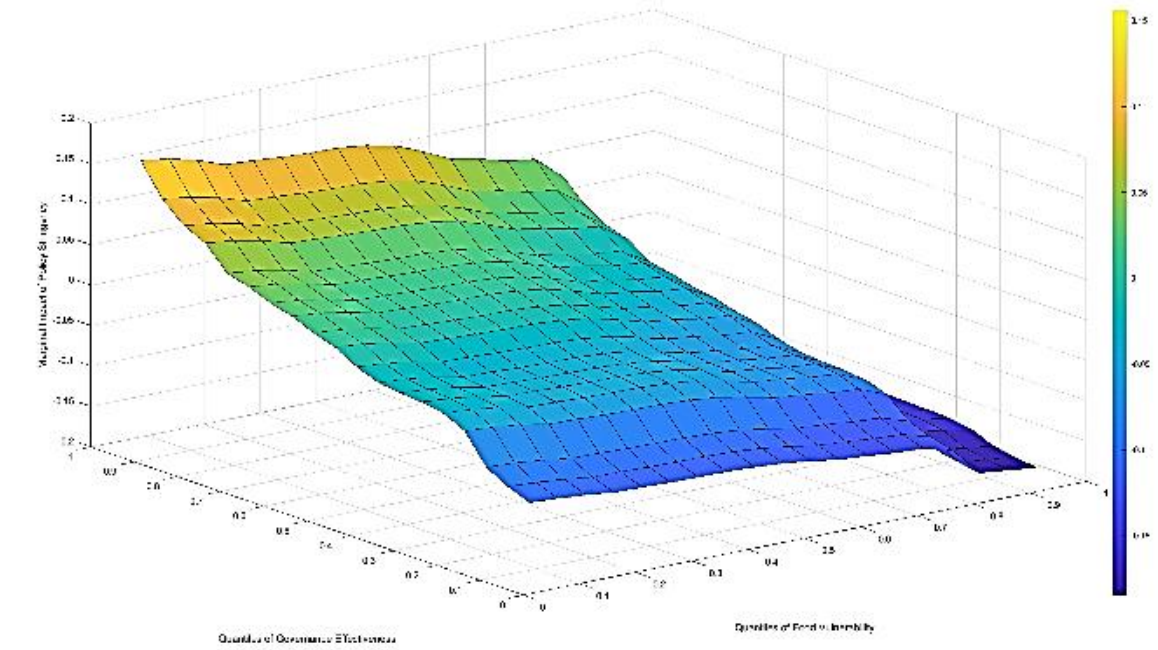

Governance Quality Indicator: Governance Effectiveness

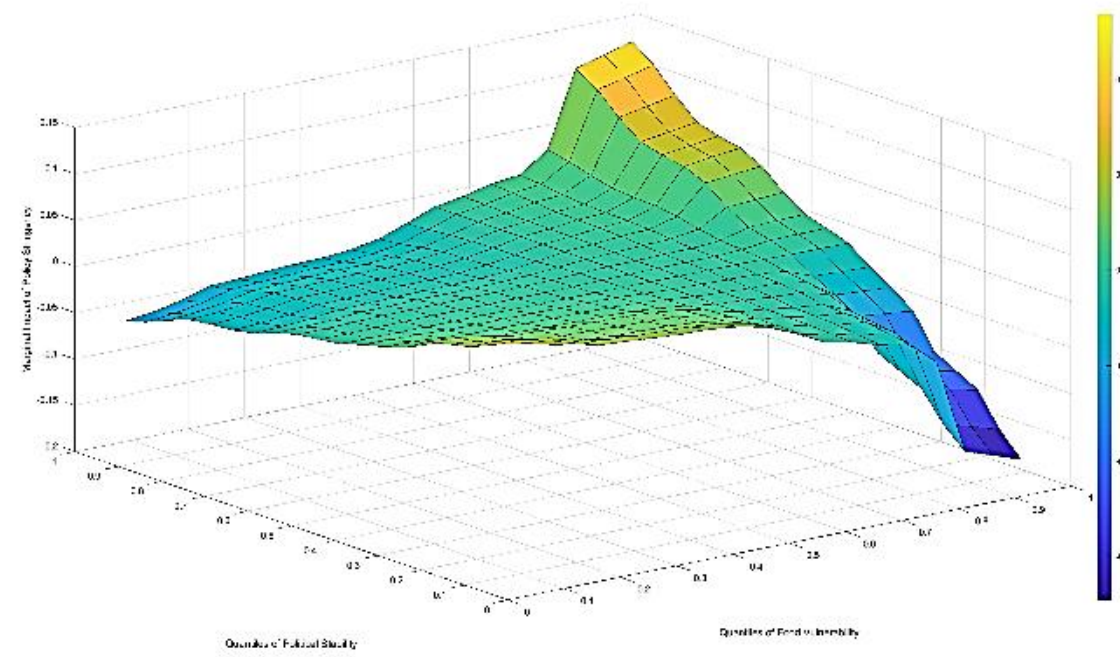

Governance Quality Indicator: Political Stability

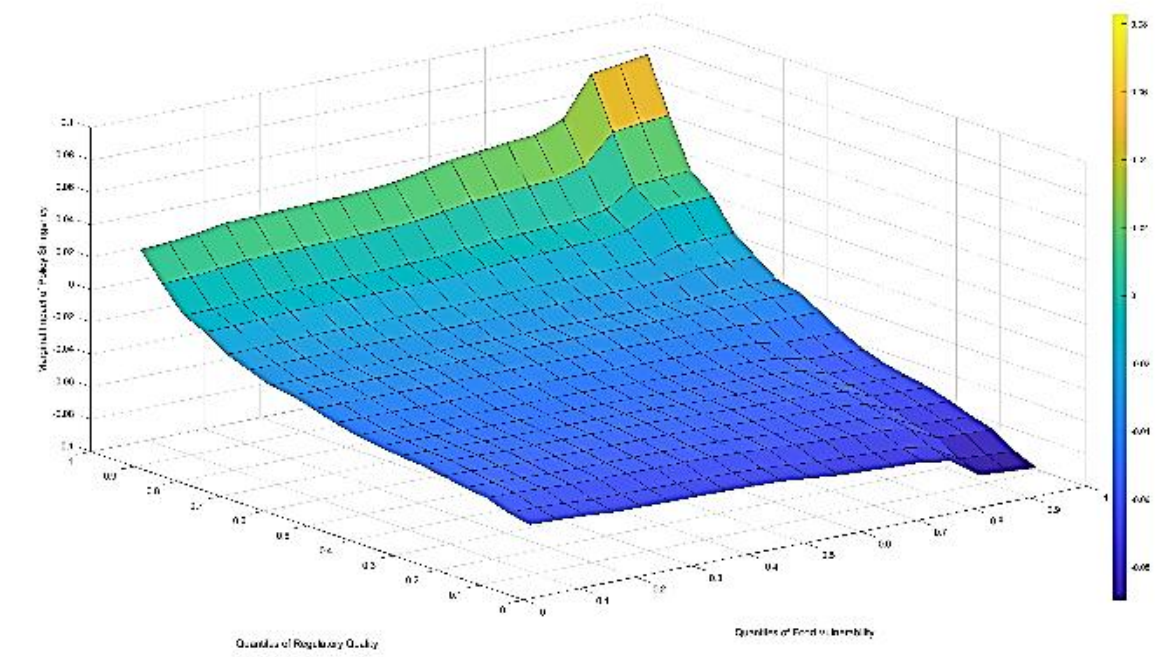

Governance Quality Indicator: Regulatory Quality

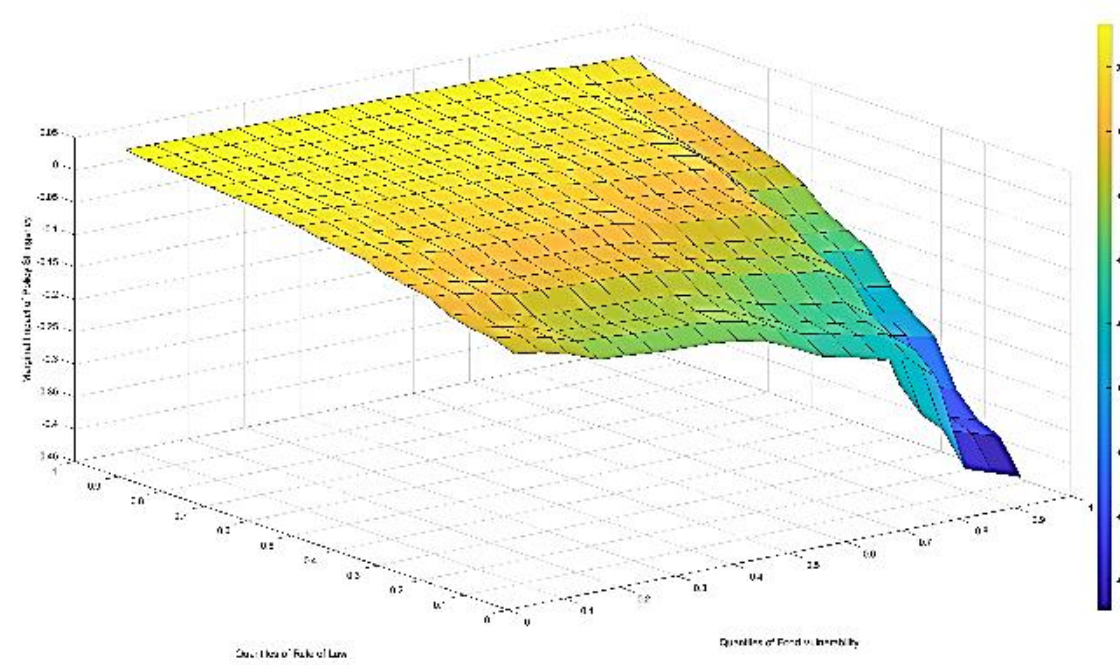

Governance Quality Indicator: Rule of Law

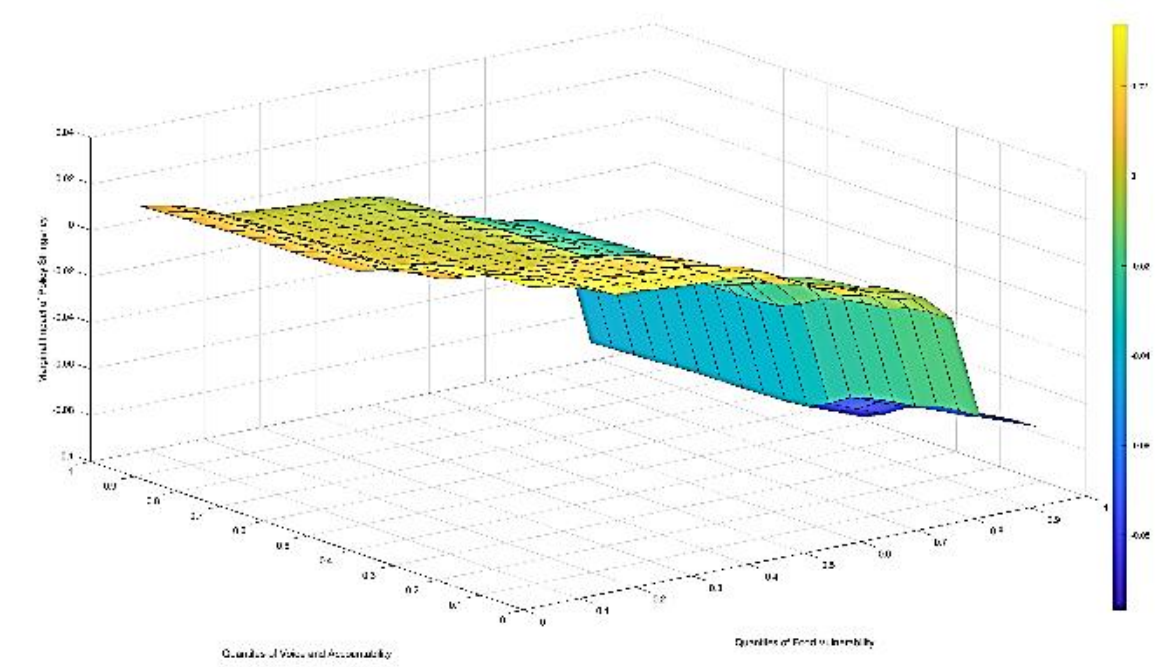

Governance Quality Indicator: Voice and Accountability

Figure S84: BRICS Policy Simulation Outcomes for the Marginal Impact of Policy Stringency on SDG 7.3.1 under Food Vulnerability and Governance Quality, Related to Figure 4-5 and Table 2

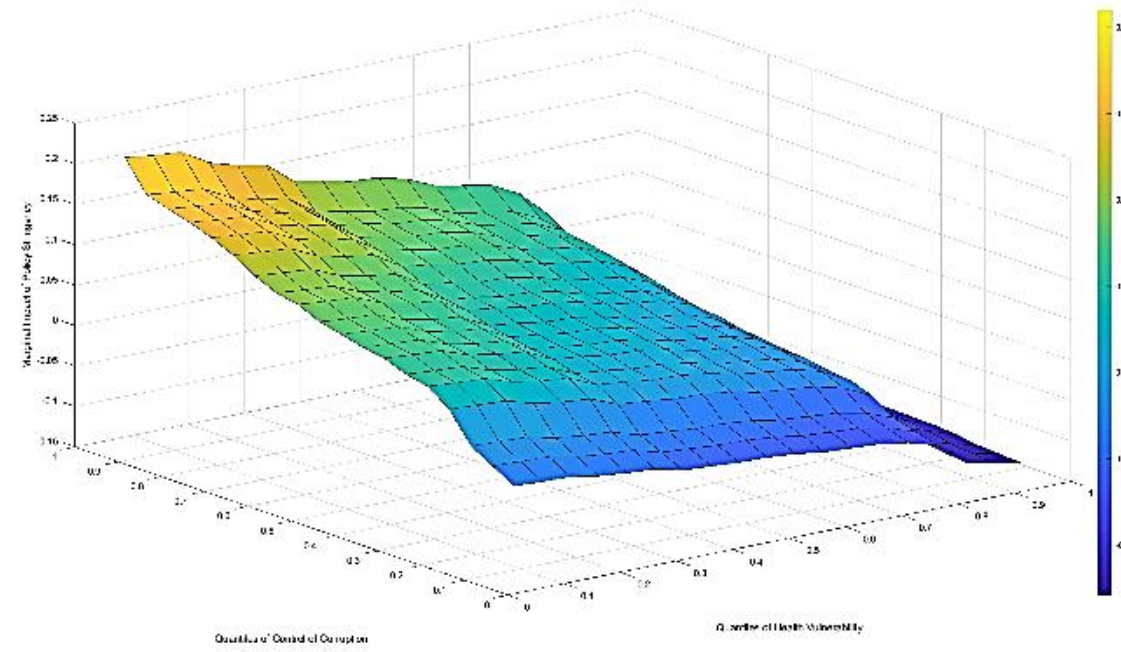

Governance Quality Indicator: Control of Corruption

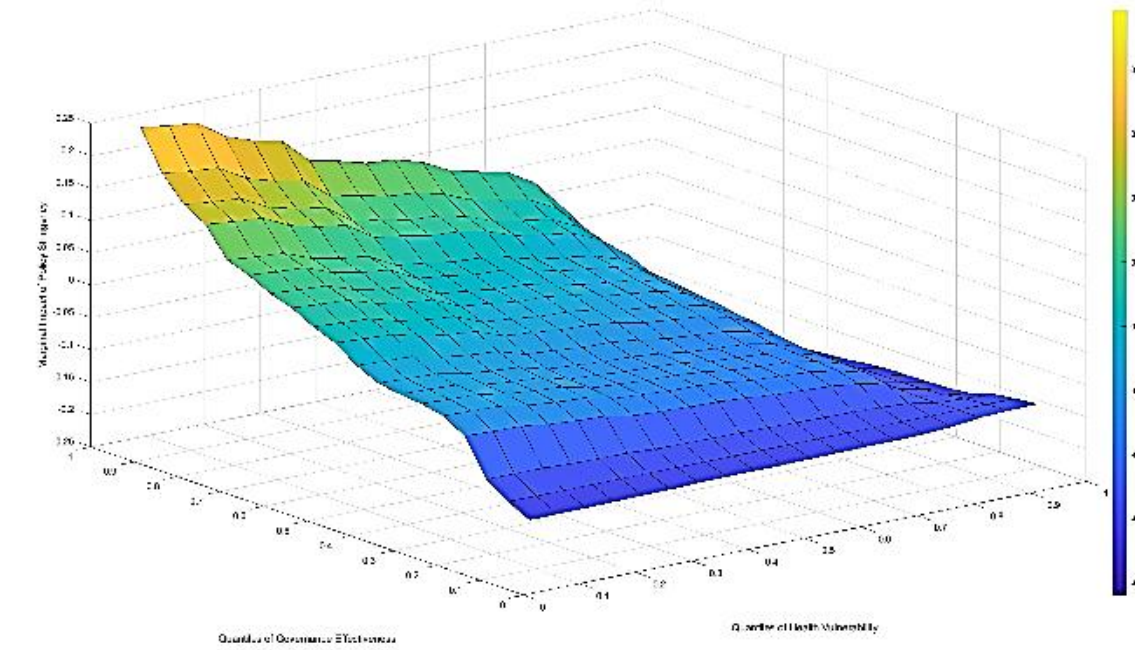

Governance Quality Indicator: Governance Effectiveness

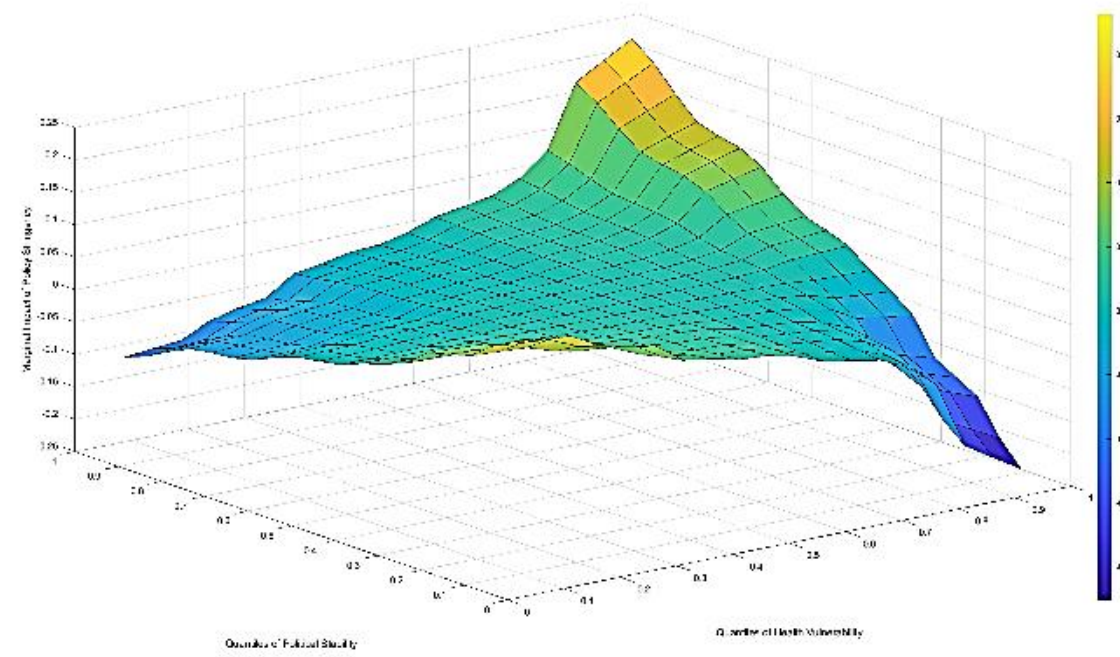

Governance Quality Indicator: Political Stability

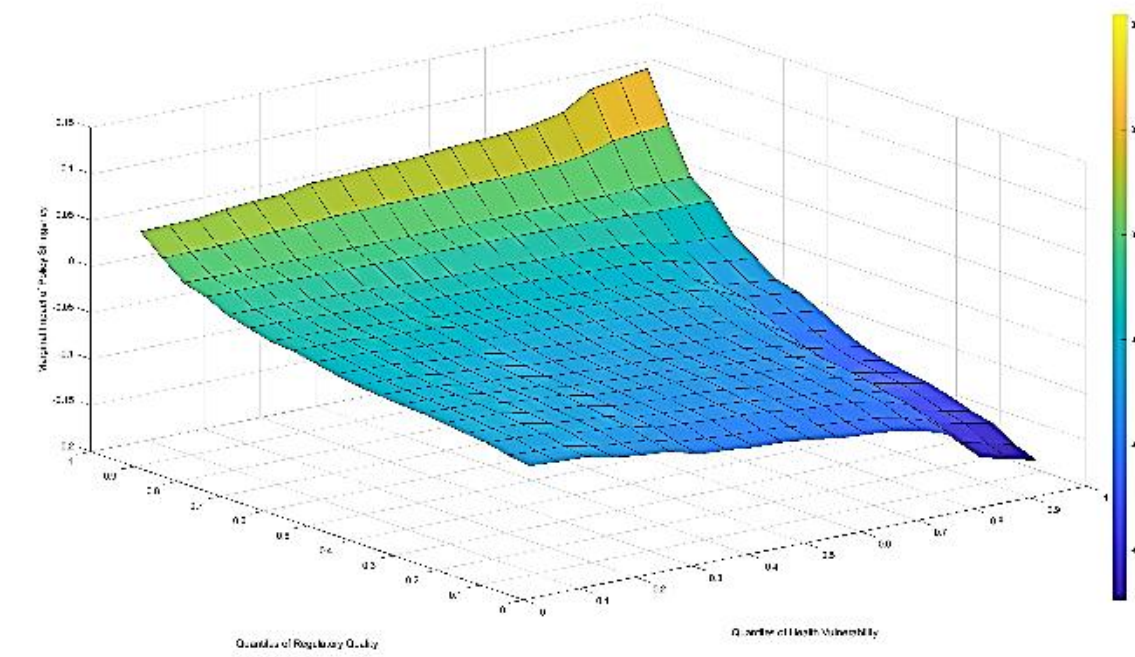

Governance Quality Indicator: Regulatory Quality

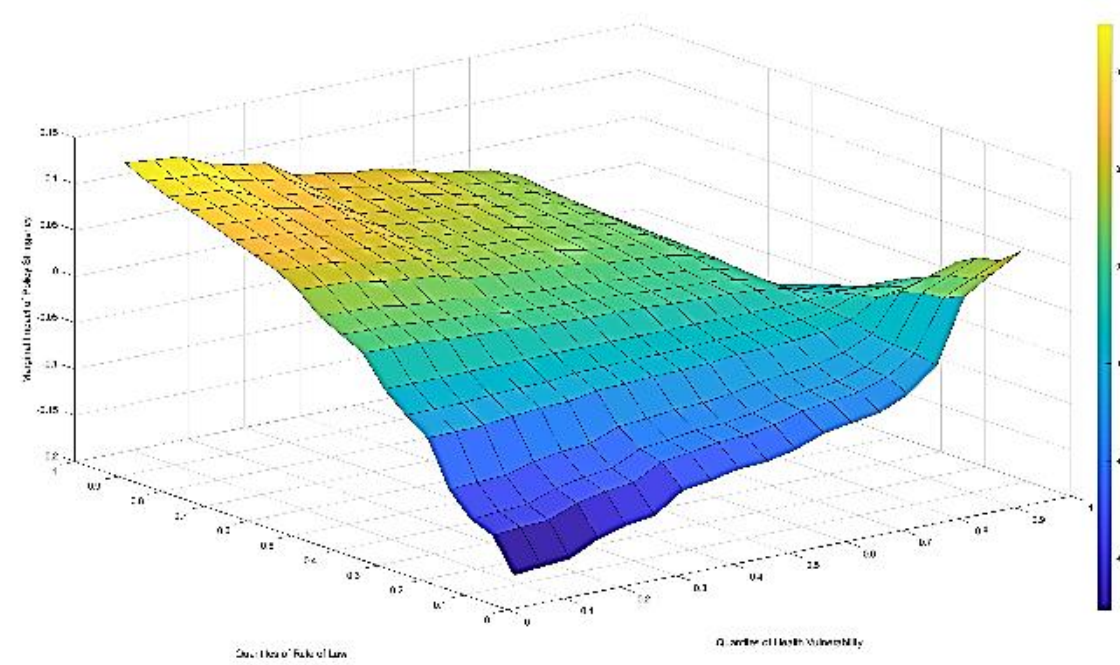

Governance Quality Indicator: Rule of Law

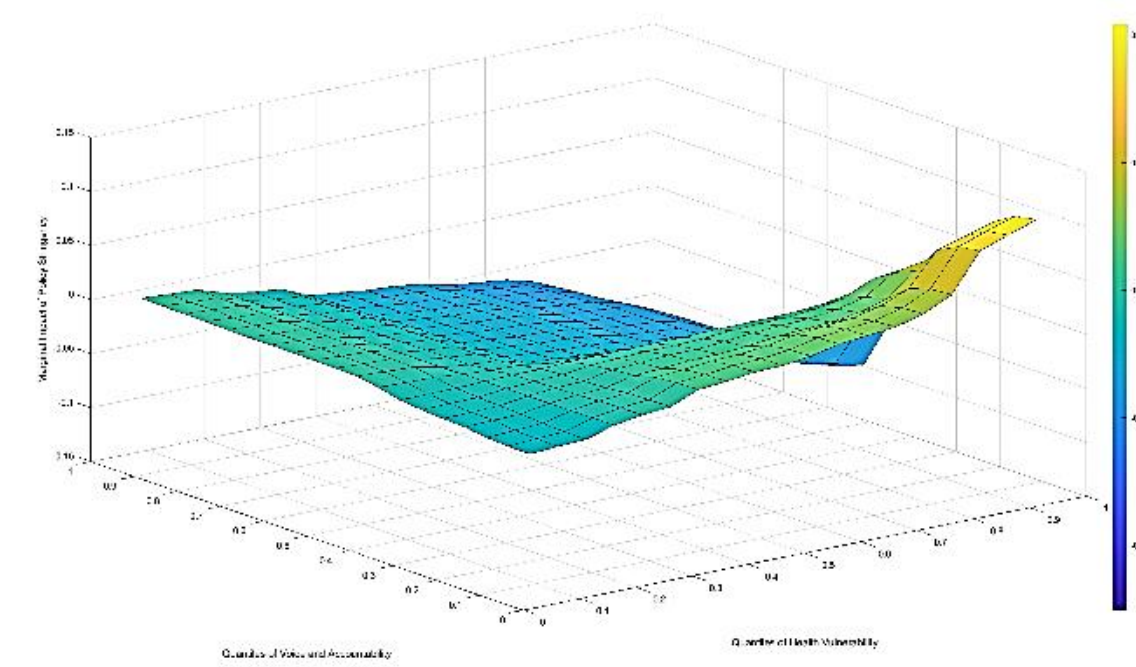

Governance Quality Indicator: Voice and Accountability

Figure S85: BRICS Policy Simulation Outcomes for the Marginal Impact of Policy Stringency on SDG 7.3.1 under Health Vulnerability and Governance Quality, Related to Figure 4-5 and Table 2

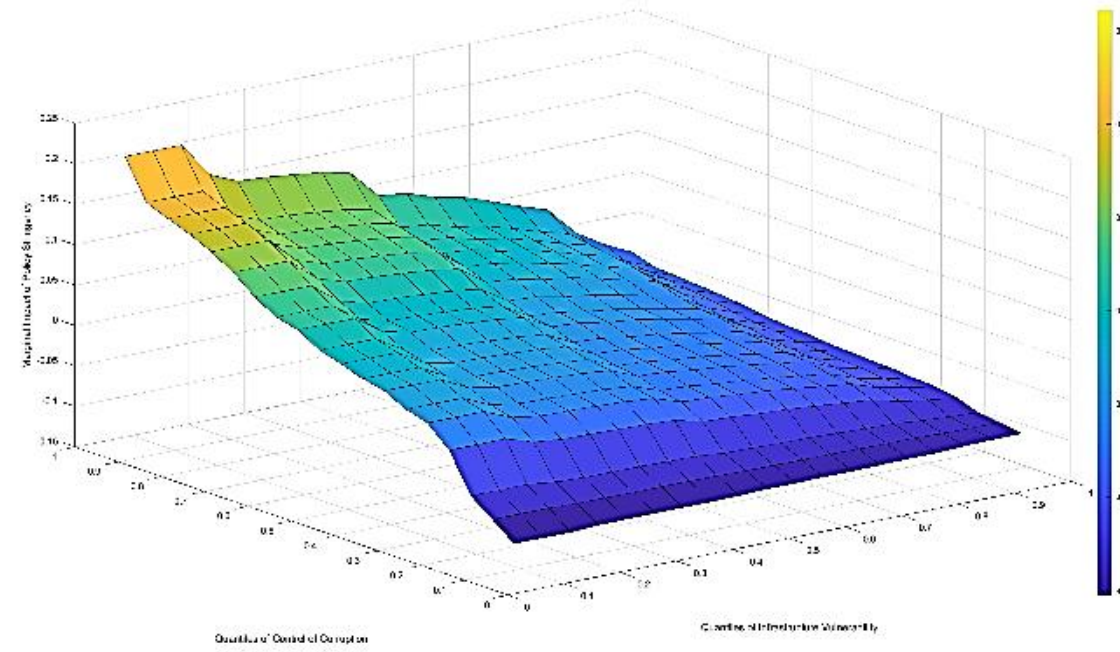

Governance Quality Indicator: Control of Corruption

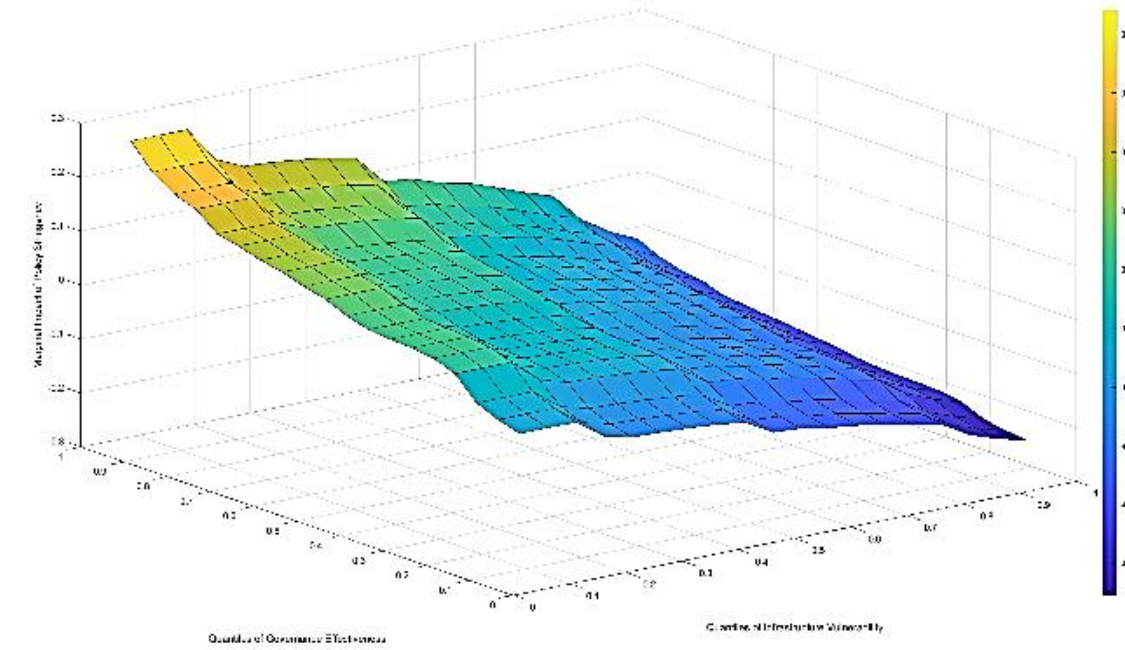

Governance Quality Indicator: Governance Effectiveness

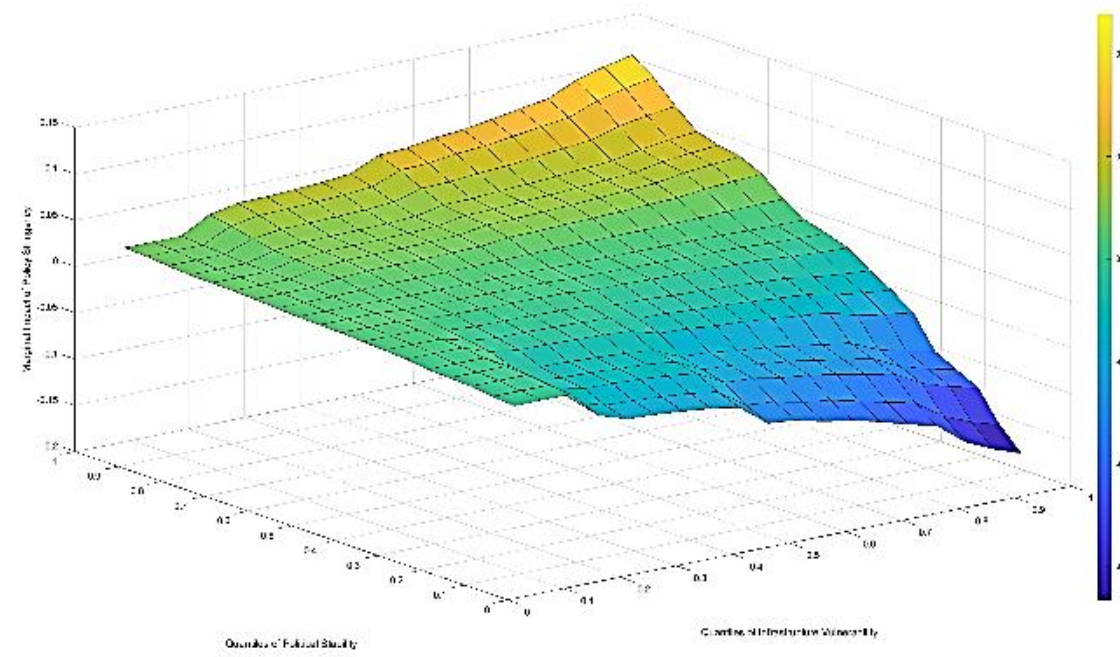

Governance Quality Indicator: Political Stability

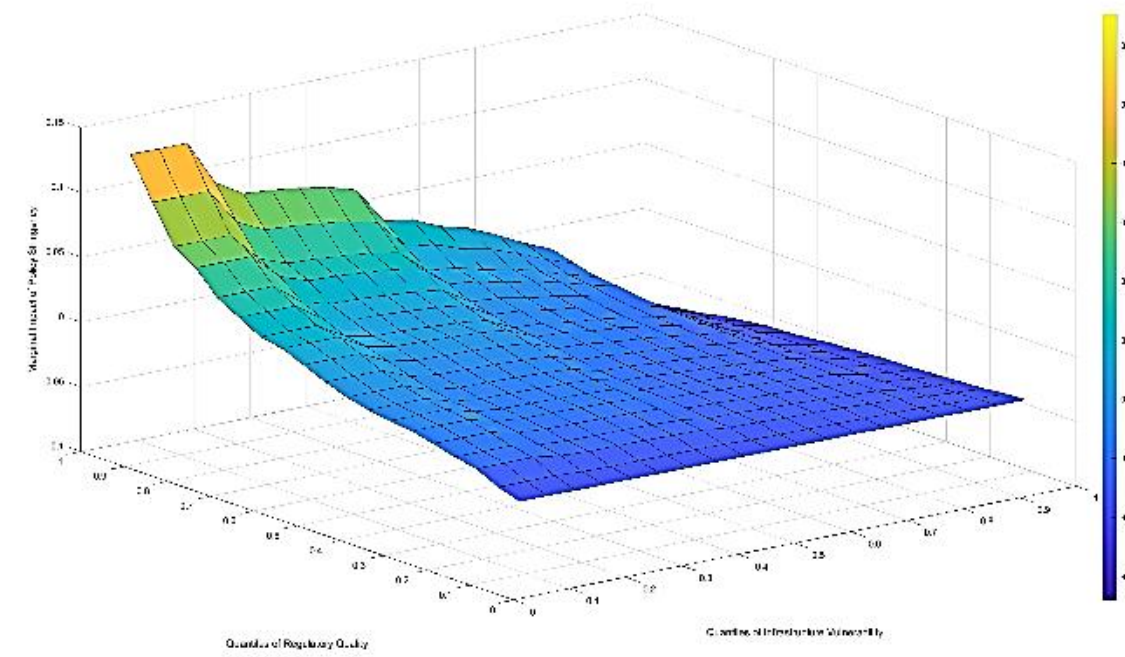

Governance Quality Indicator: Regulatory Quality

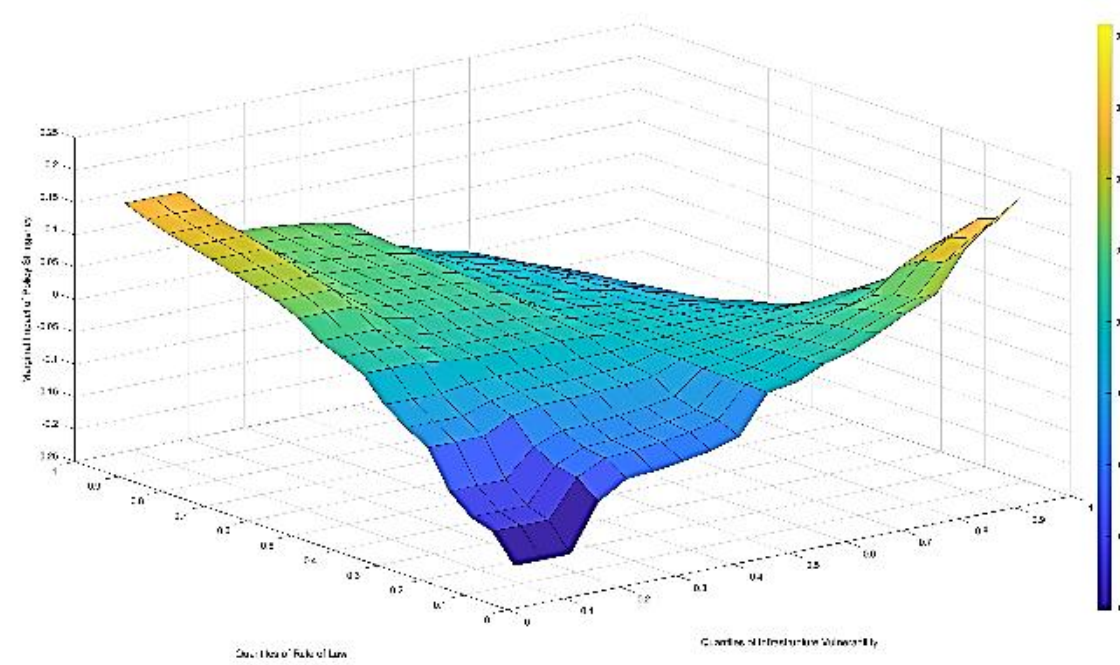

Governance Quality Indicator: Rule of Law

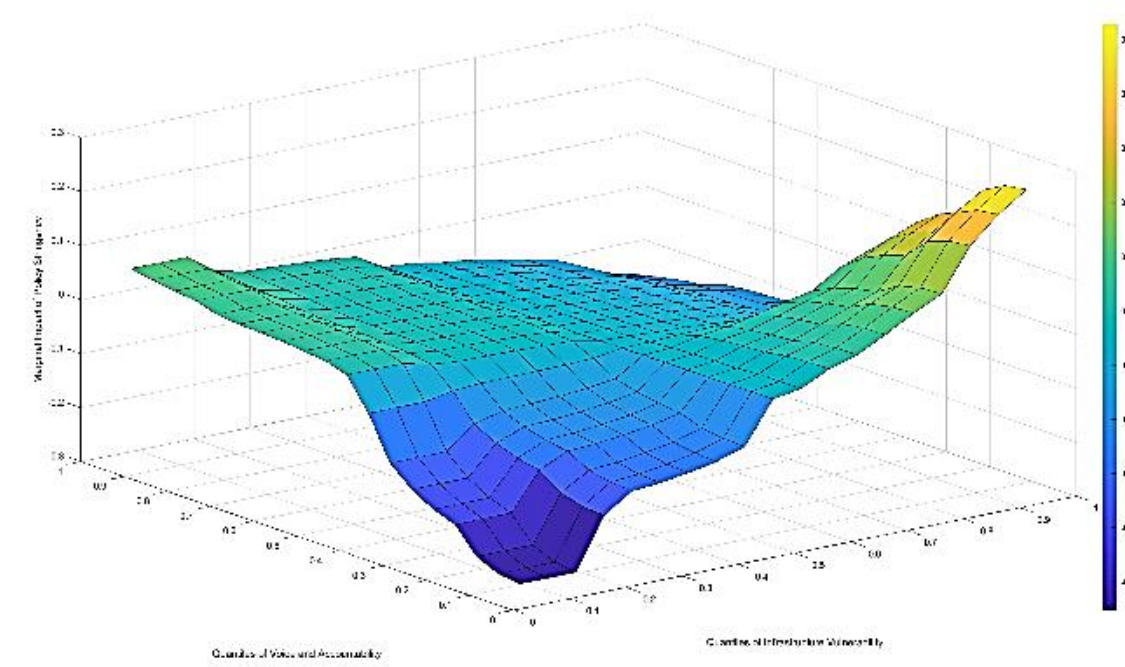

Governance Quality Indicator: Voice and Accountability

Figure S86: BRICS Policy Simulation Outcomes for the Marginal Impact of Policy Stringency on SDG 7.3.1 under Infrastructure Vulnerability and Governance Quality, Related to Figure 4-5 and Table 2

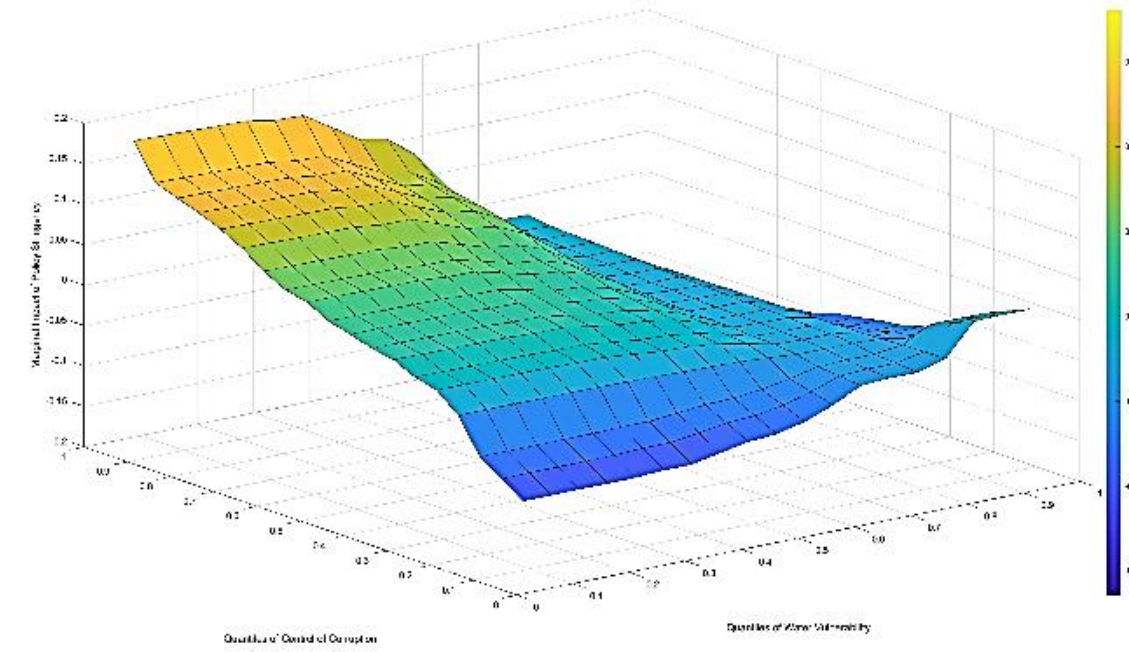

Governance Quality Indicator: Control of Corruption

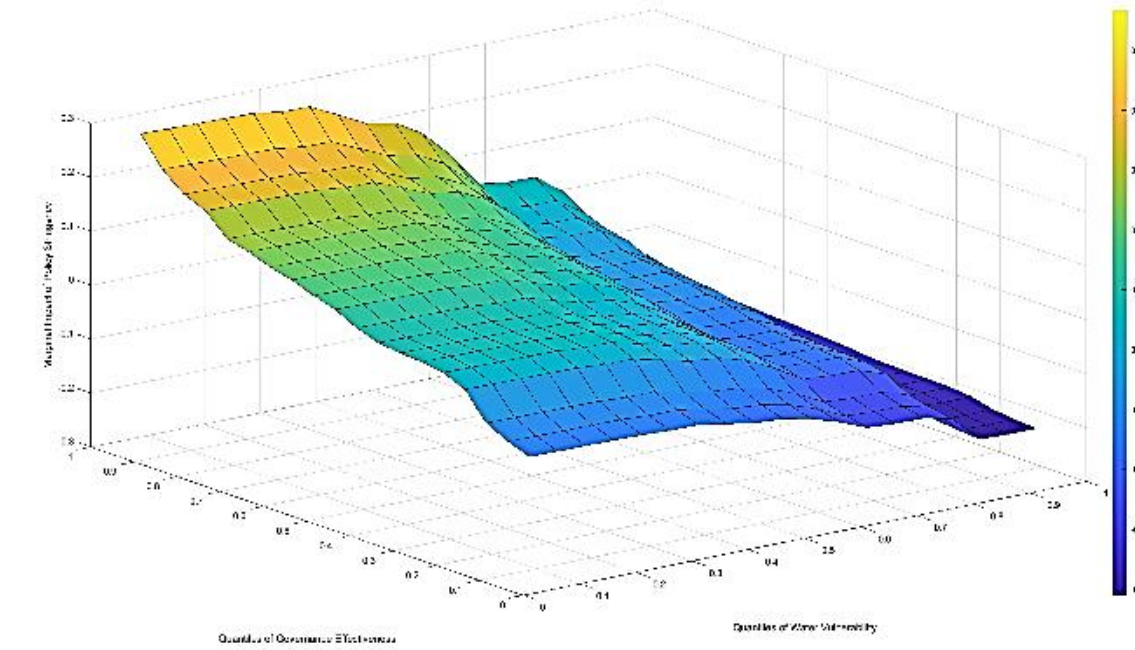

Governance Quality Indicator: Governance Effectiveness

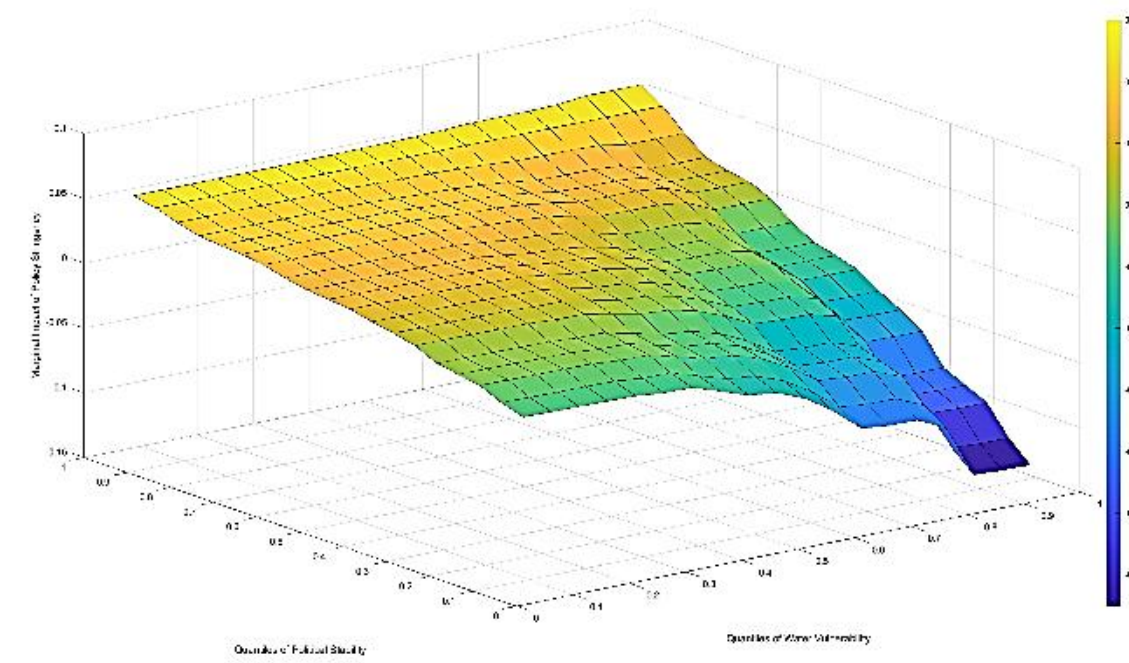

Governance Quality Indicator: Political Stability

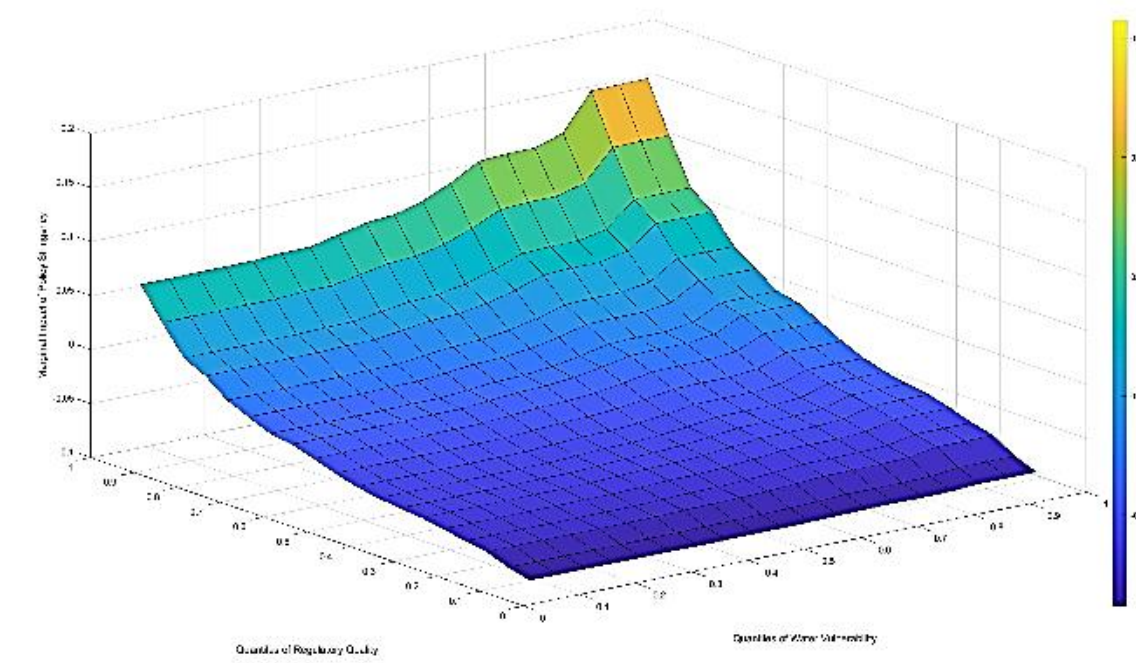

Governance Quality Indicator: Regulatory Quality

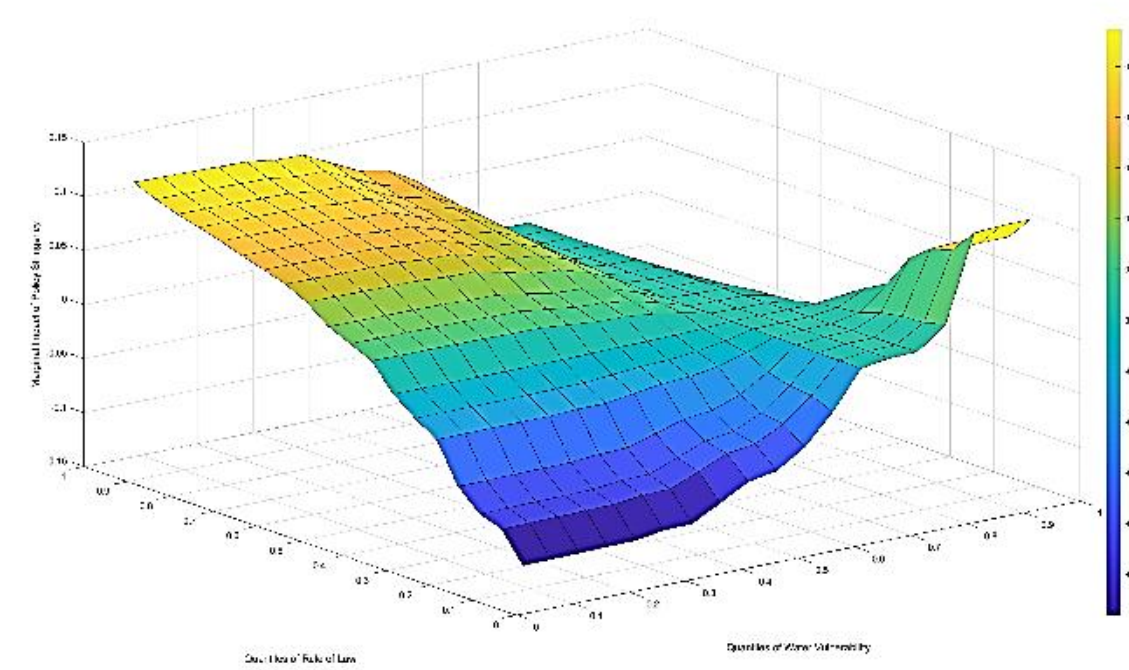

Governance Quality Indicator: Rule of Law

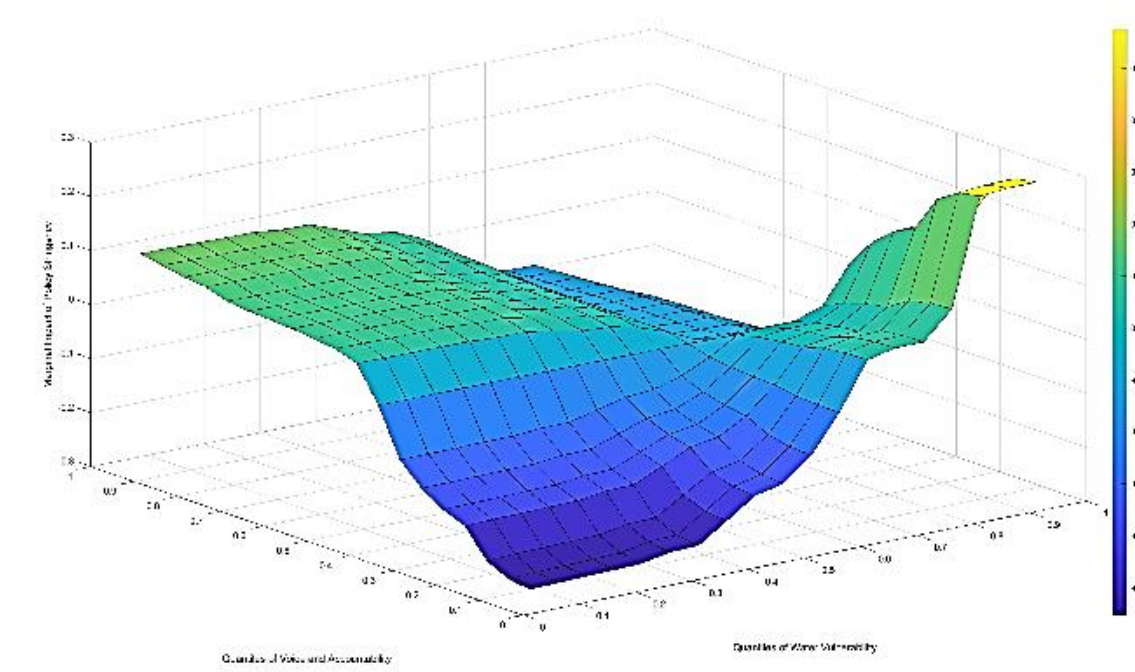

Governance Quality Indicator: Voice and Accountability

Figure S87: BRICS Policy Simulation Outcomes for the Marginal Impact of Policy Stringency on SDG 7.3.1 under Water Vulnerability and Governance Quality, Related to Figure 4-5 and Table 2

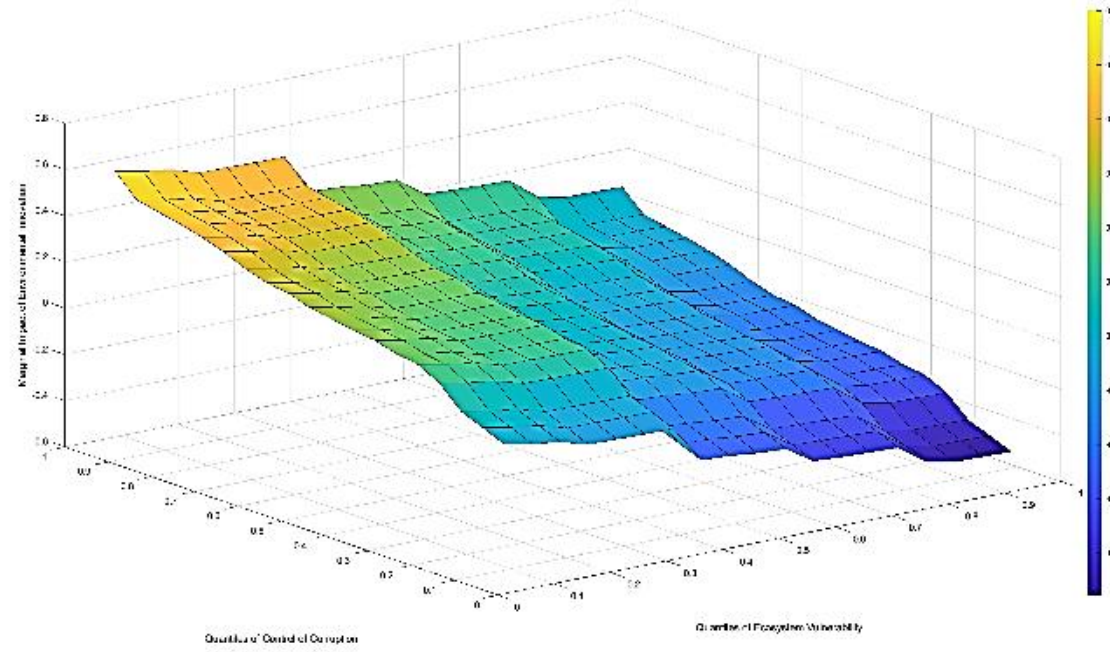

Governance Quality Indicator: Control of Corruption

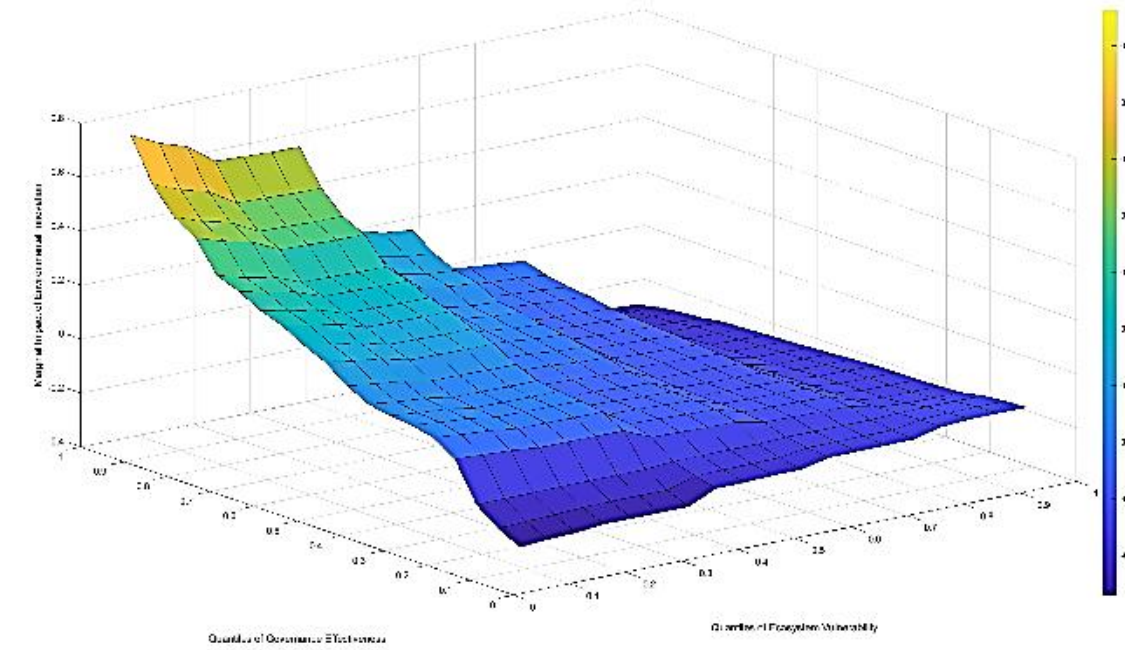

Governance Quality Indicator: Governance Effectiveness

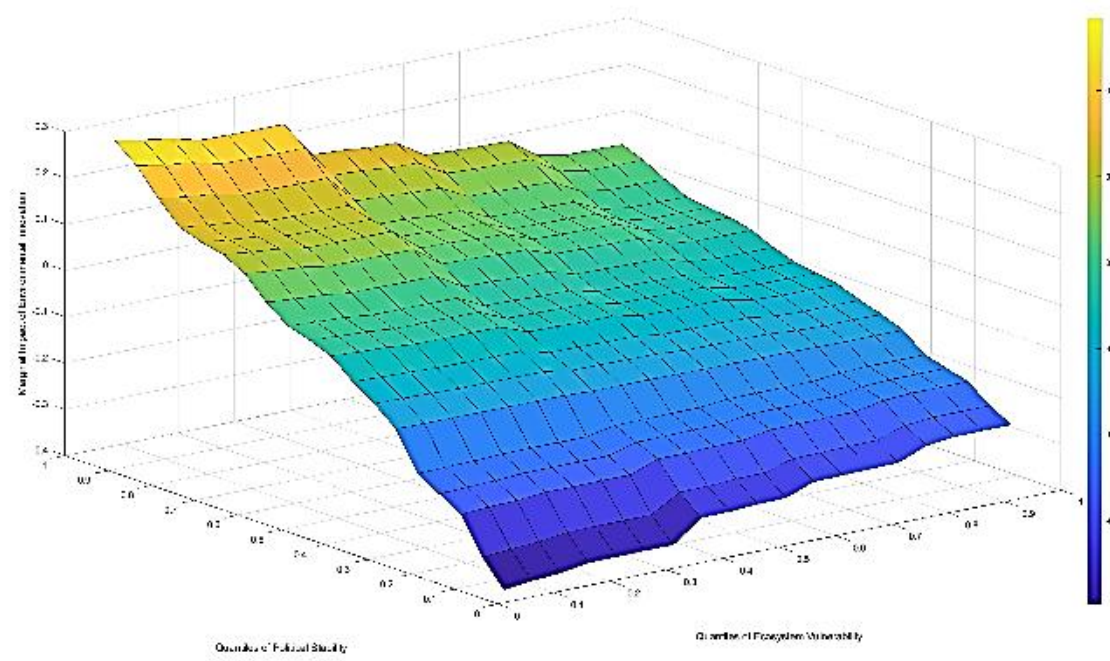

Governance Quality Indicator: Political Stability

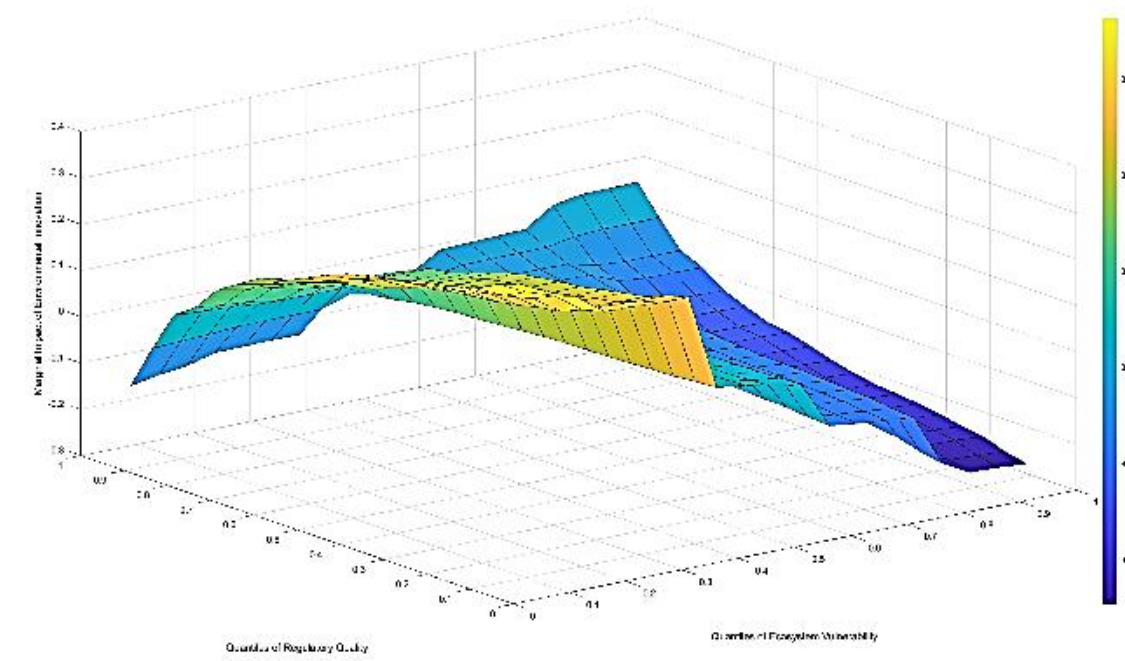

Governance Quality Indicator: Regulatory Quality

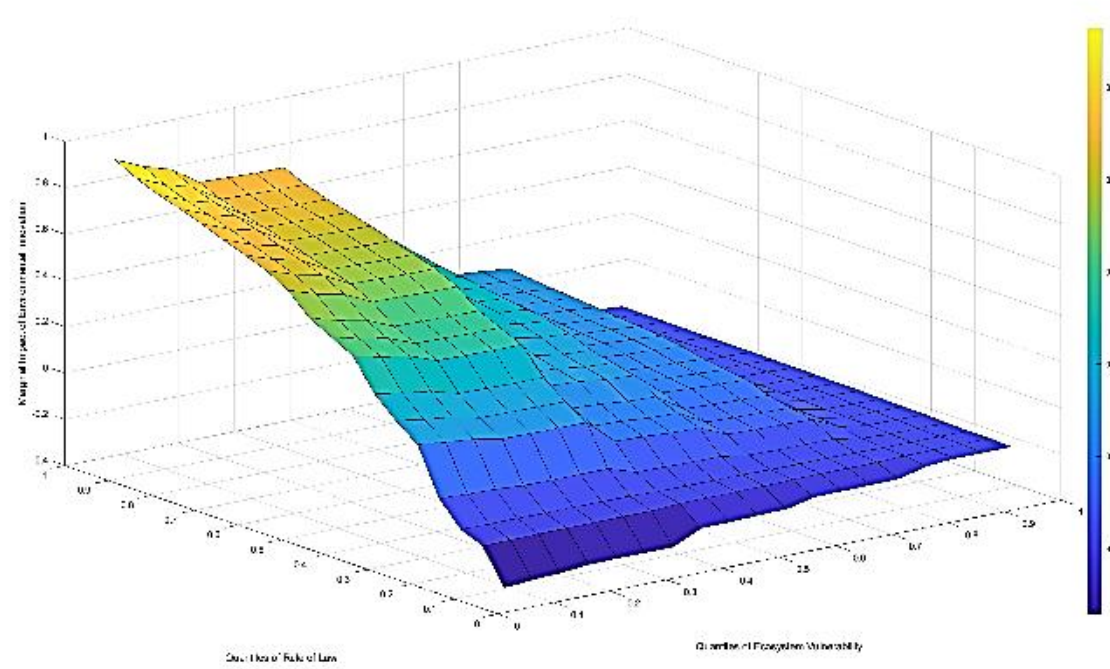

Governance Quality Indicator: Rule of Law

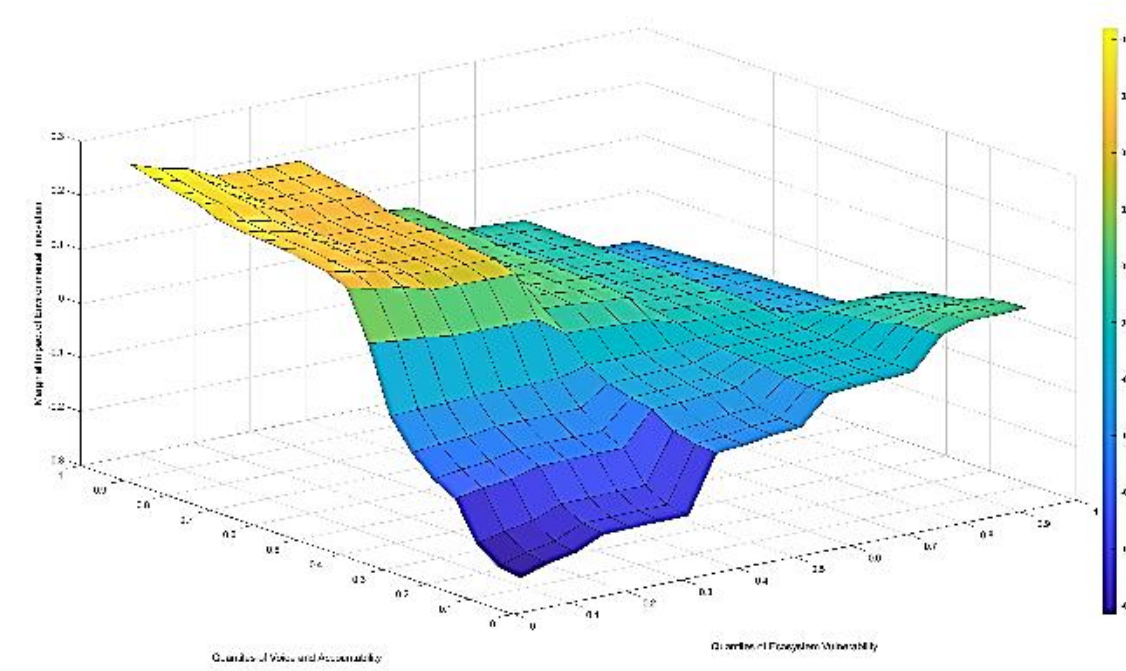

Governance Quality Indicator: Voice and Accountability

Figure S88: BRICS Policy Simulation Outcomes for the Marginal Impact of Environmental Innovation on SDG 7.3.1 under Ecosystem Vulnerability and Governance Quality, Related to Figure 4-5 and Table 2

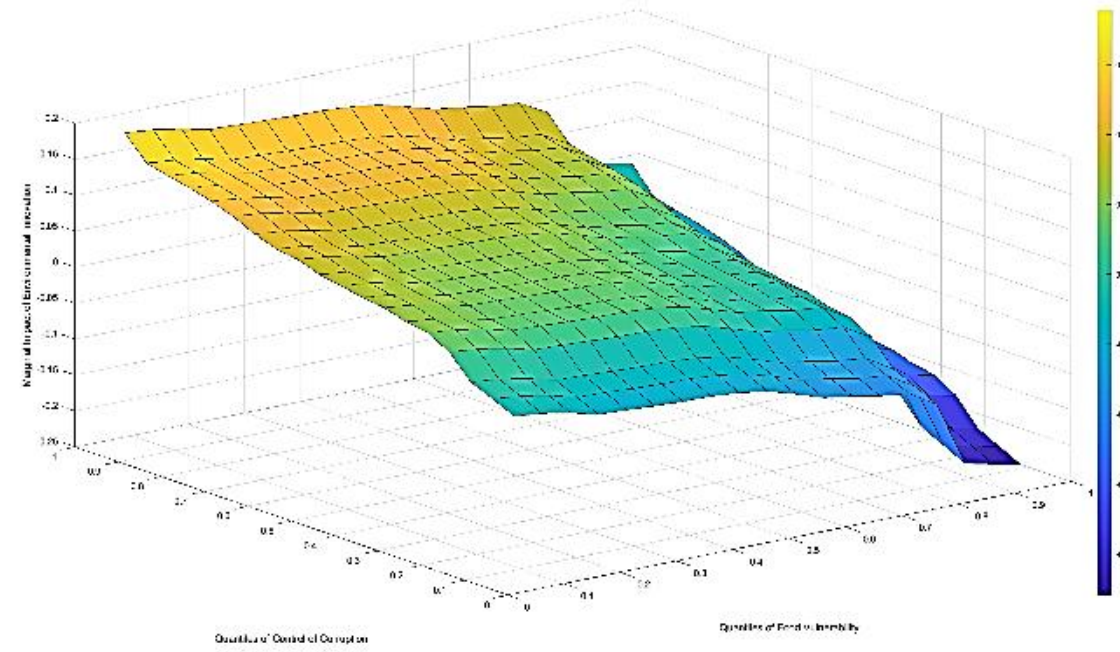

Governance Quality Indicator: Control of Corruption

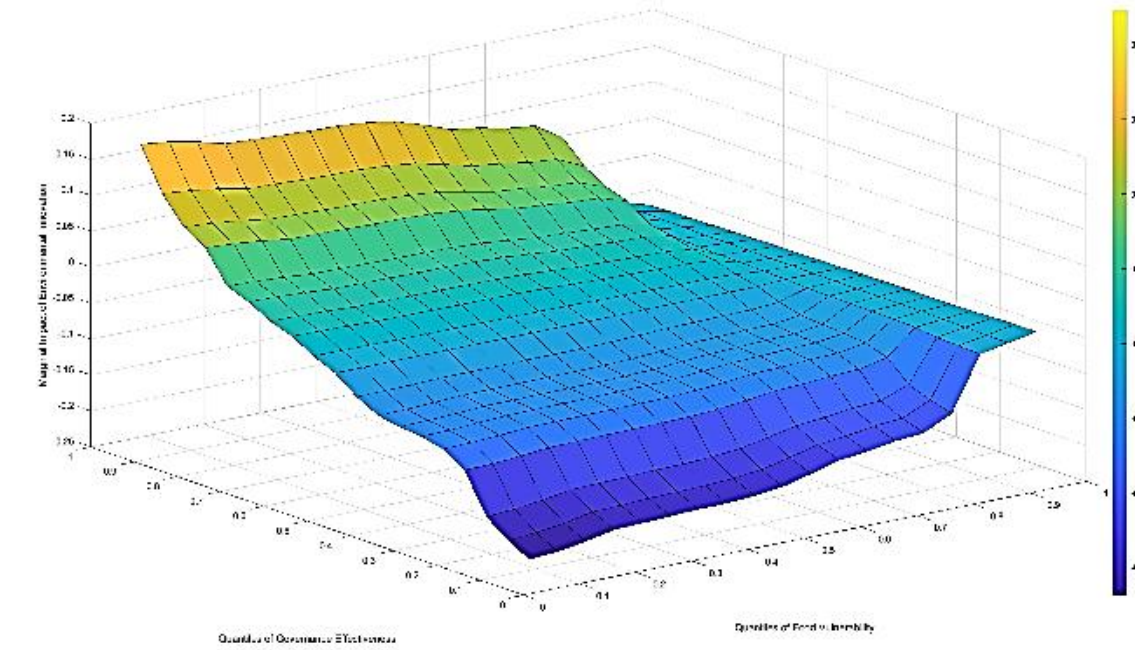

Governance Quality Indicator: Governance Effectiveness

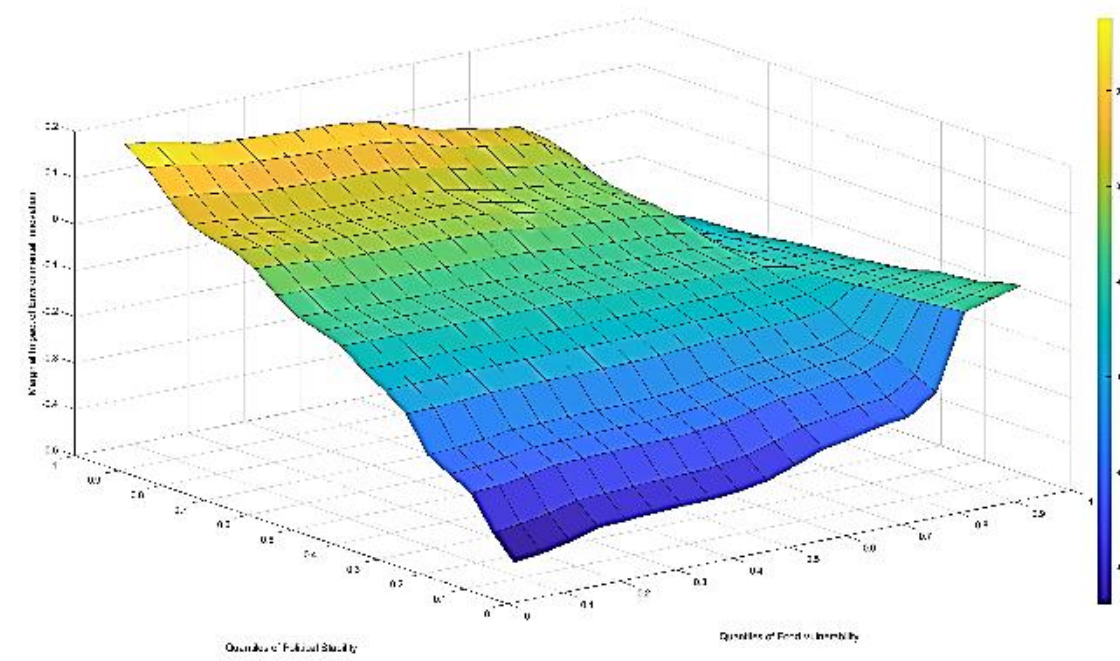

Governance Quality Indicator: Political Stability

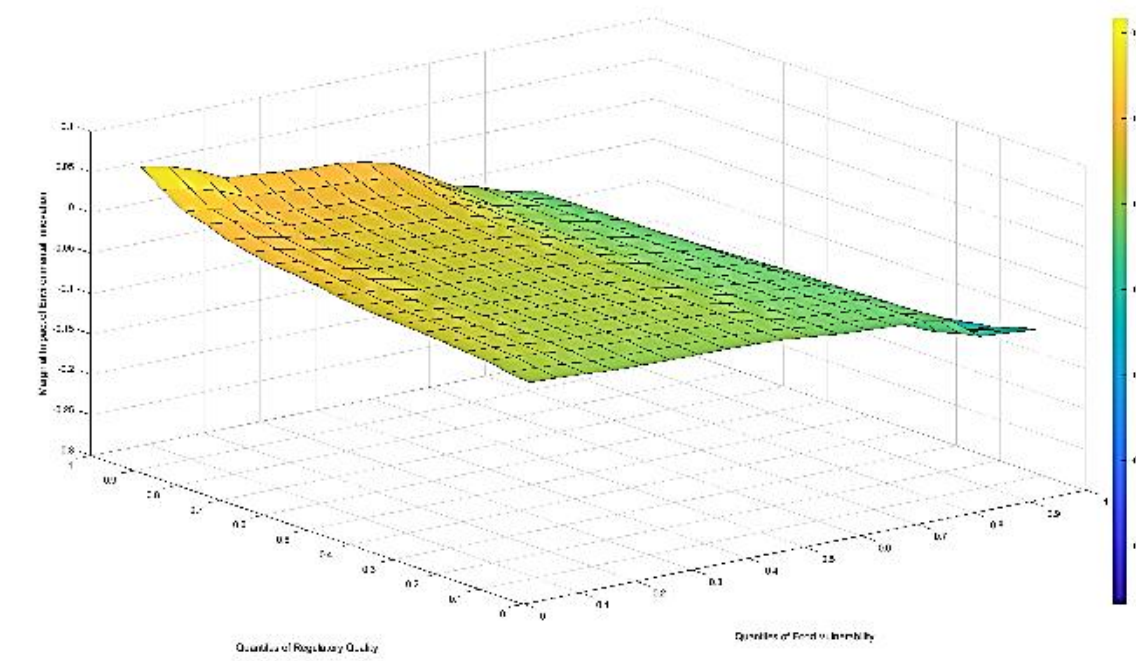

Governance Quality Indicator: Regulatory Quality

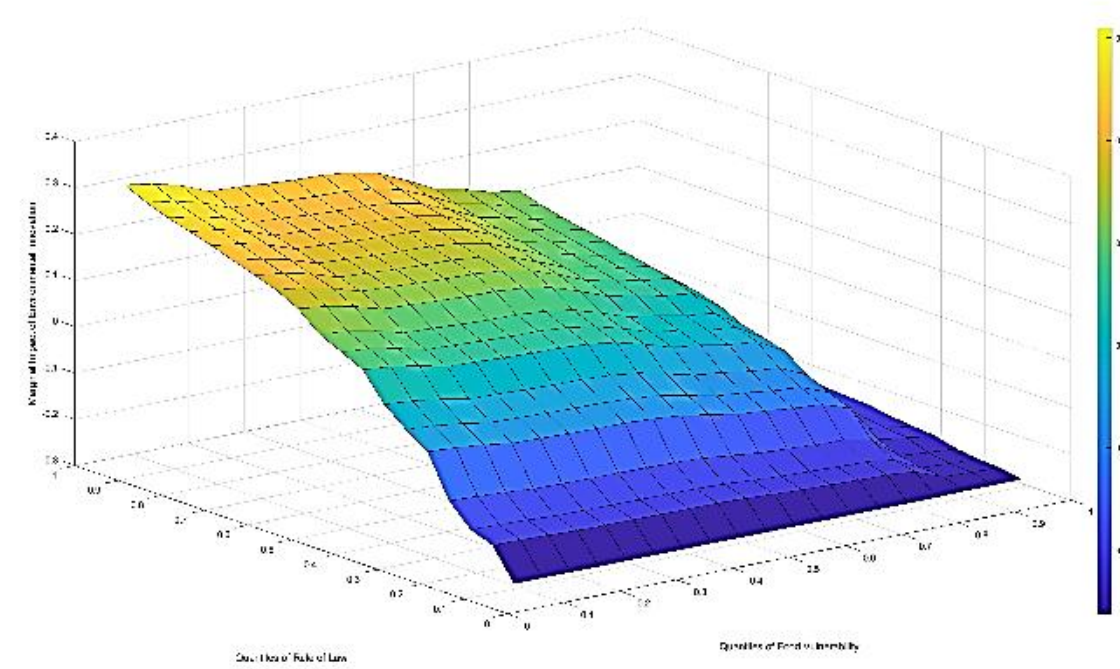

Governance Quality Indicator: Rule of Law

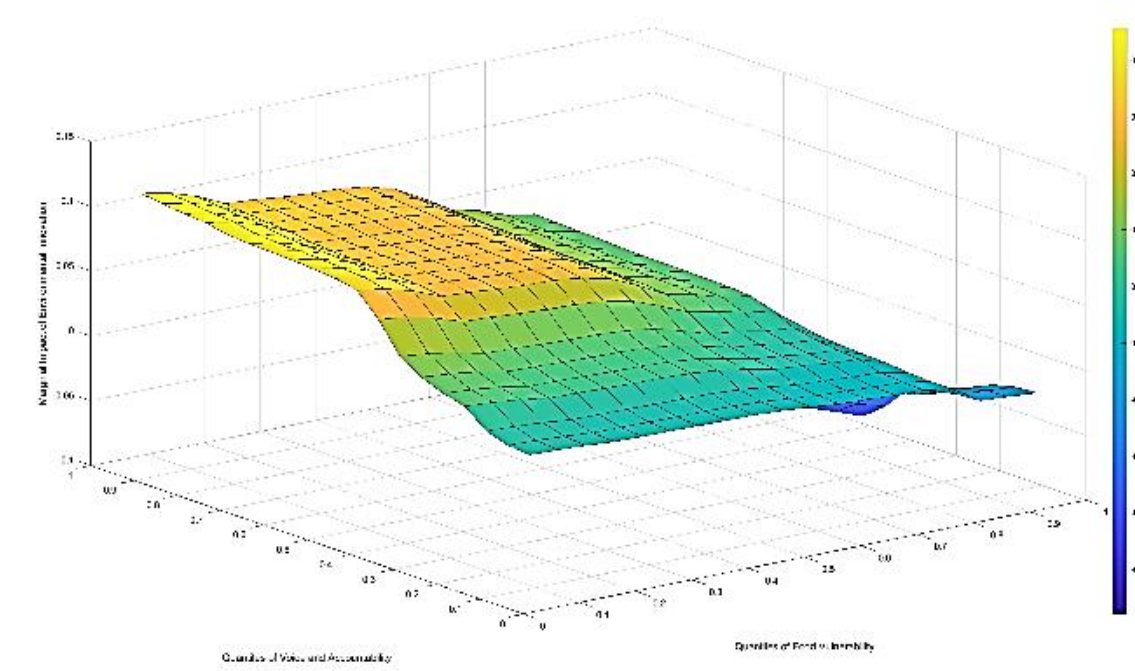

Governance Quality Indicator: Voice and Accountability

Figure S89: BRICS Policy Simulation Outcomes for the Marginal Impact of Environmental Innovation on SDG 7.3.1 under Food Vulnerability and Governance Quality, Related to Figure 4-5 and Table 2

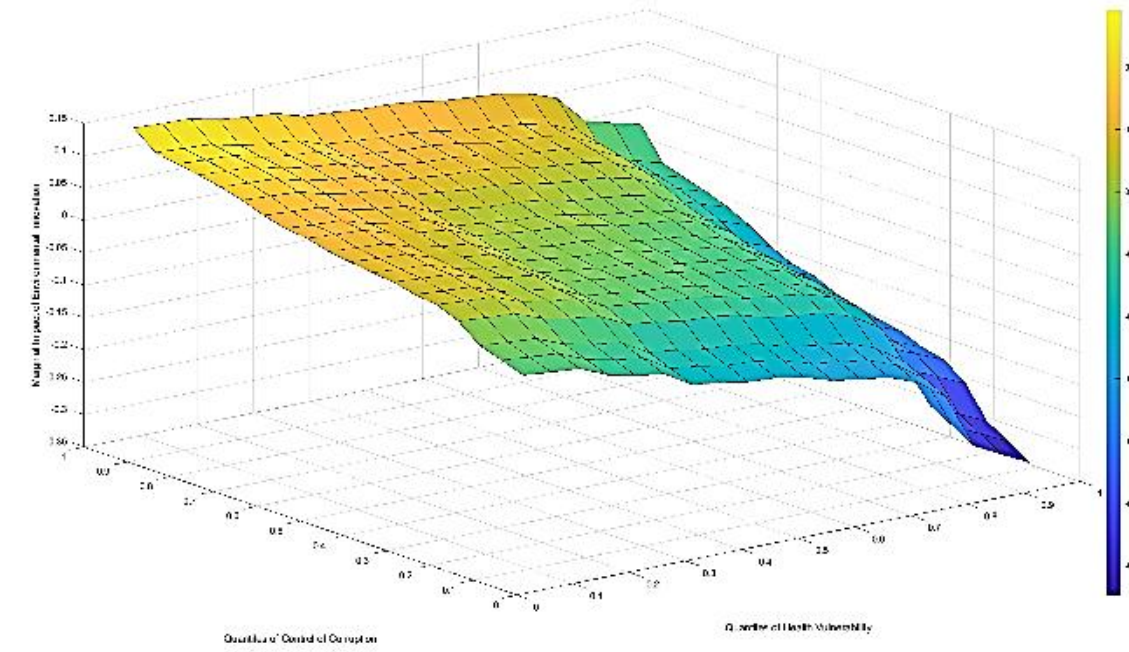

Governance Quality Indicator: Control of Corruption

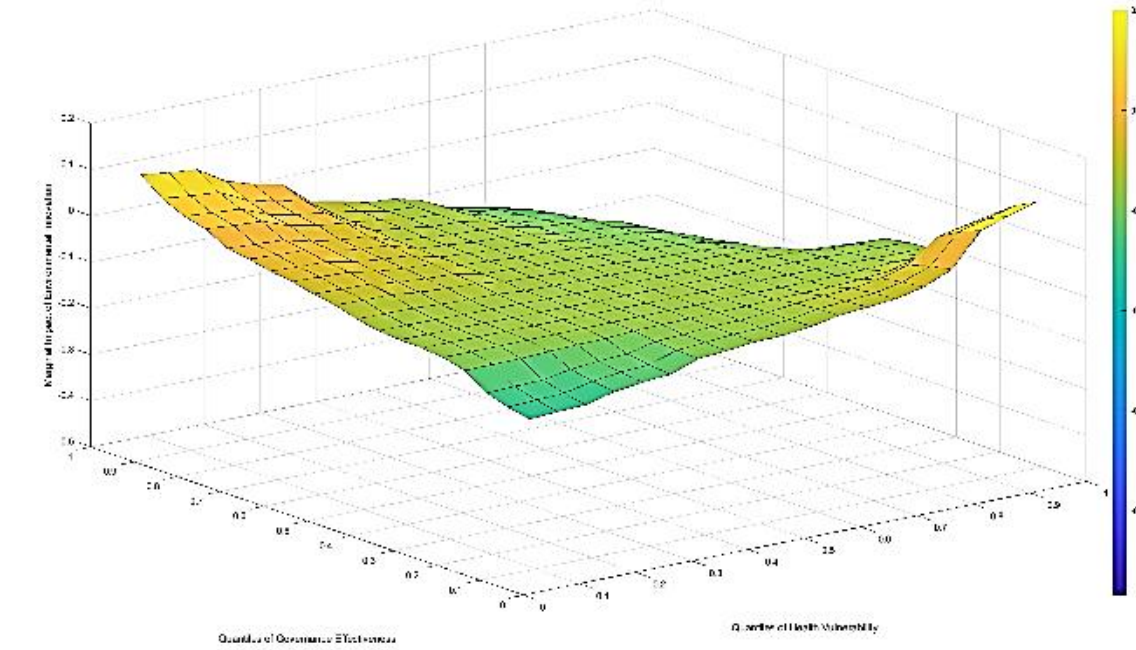

Governance Quality Indicator: Governance Effectiveness

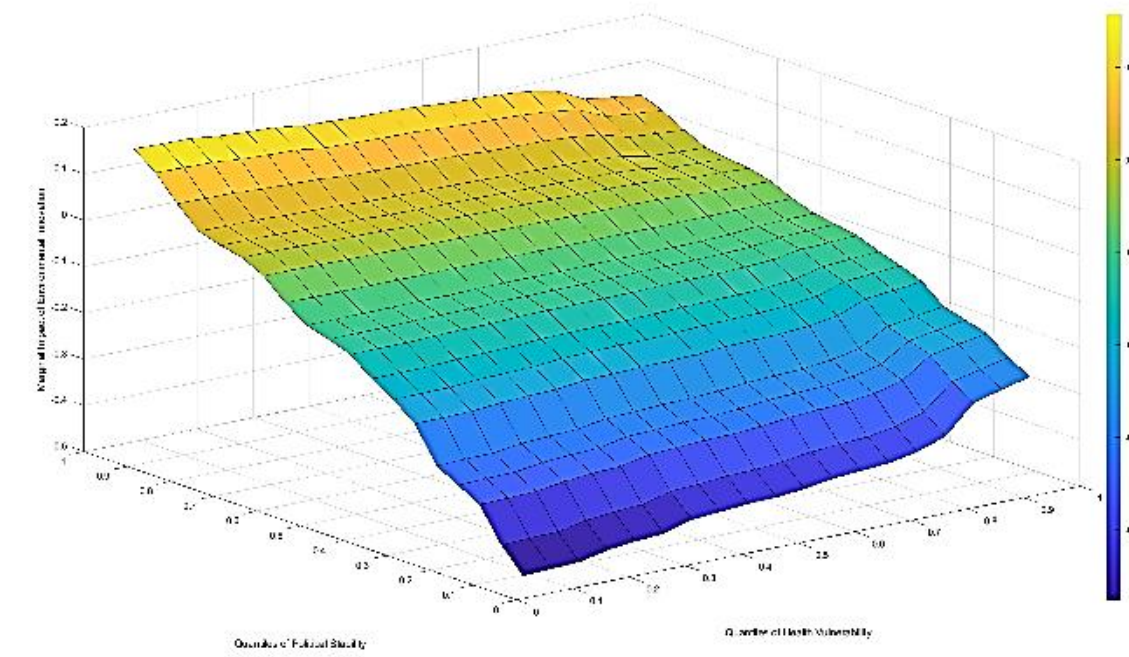

Governance Quality Indicator: Political Stability

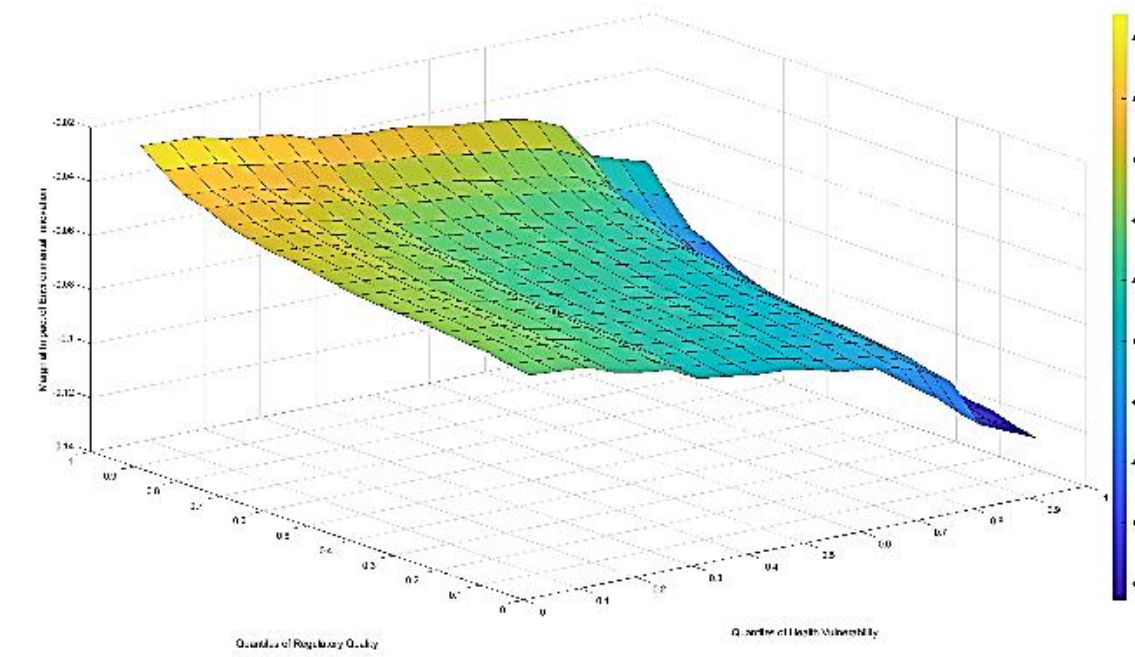

Governance Quality Indicator: Regulatory Quality

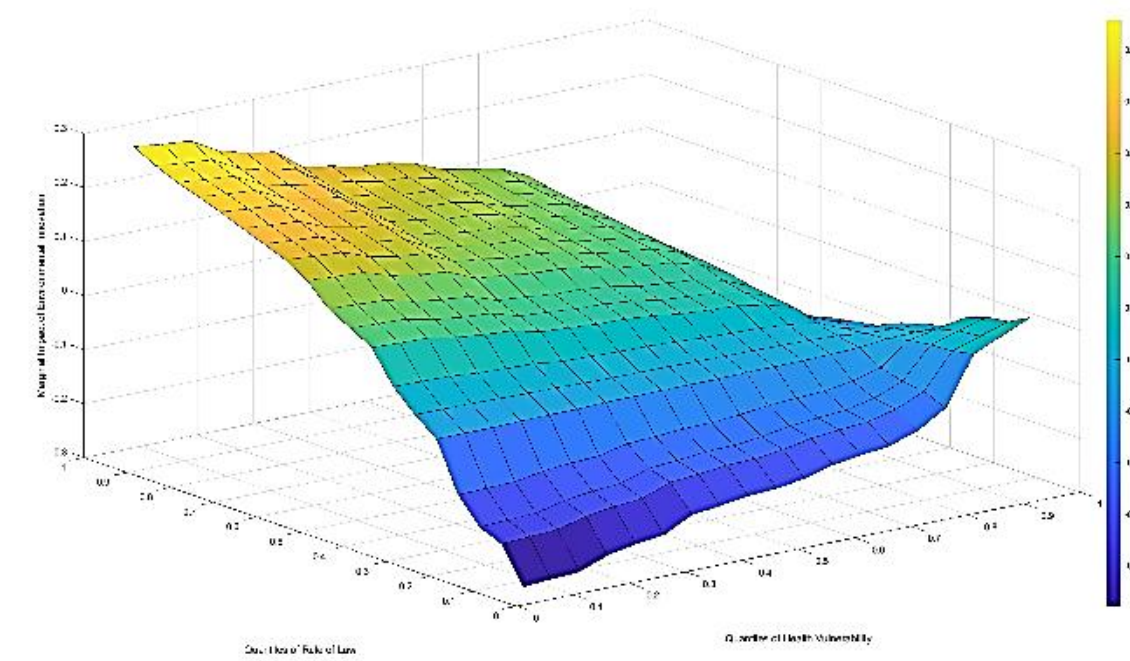

Governance Quality Indicator: Rule of Law

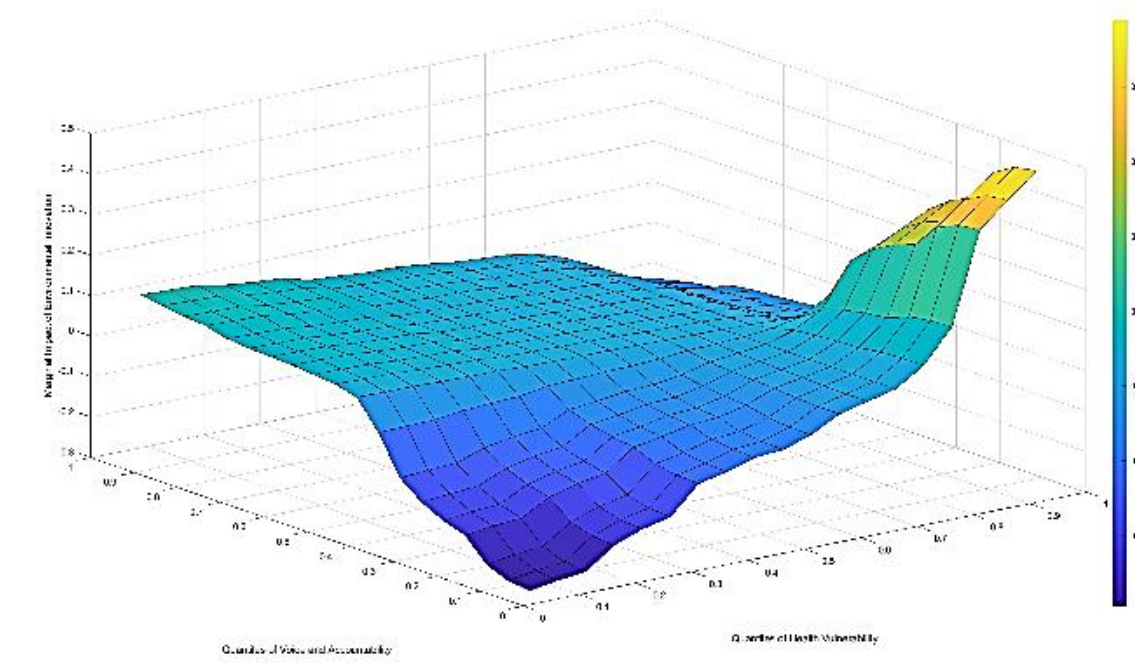

Governance Quality Indicator: Voice and Accountability

Figure S90: BRICS Policy Simulation Outcomes for the Marginal Impact of Environmental Innovation on SDG 7.3.1 under Health Vulnerability and Governance Quality, Related to Figure 4-5 and Table 2

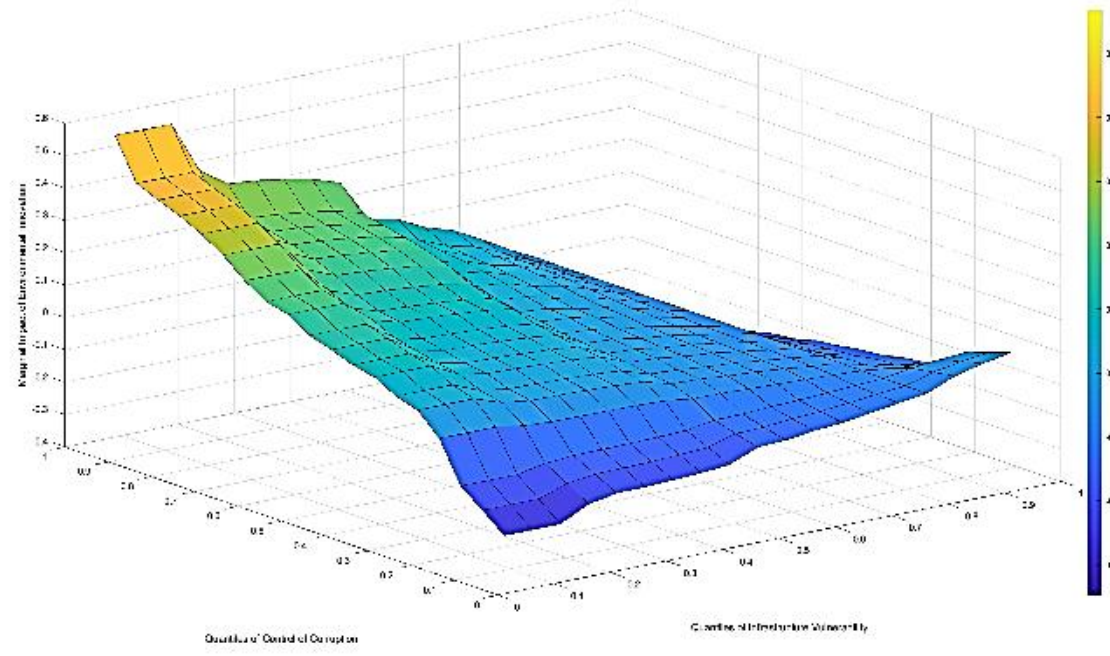

Governance Quality Indicator: Control of Corruption

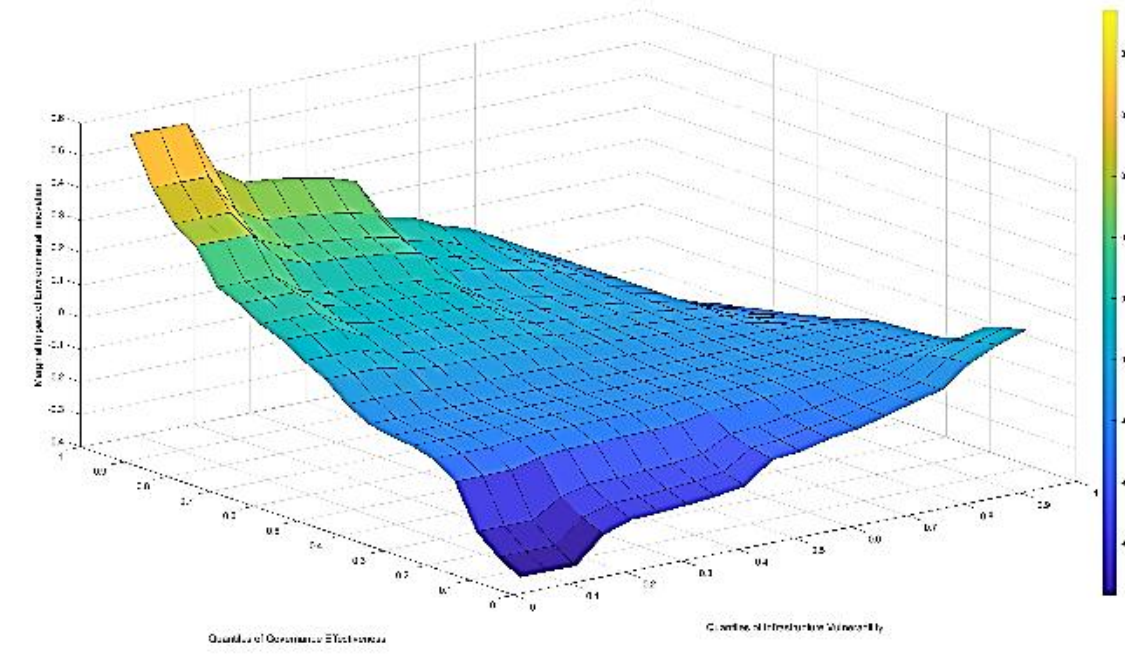

Governance Quality Indicator: Governance Effectiveness

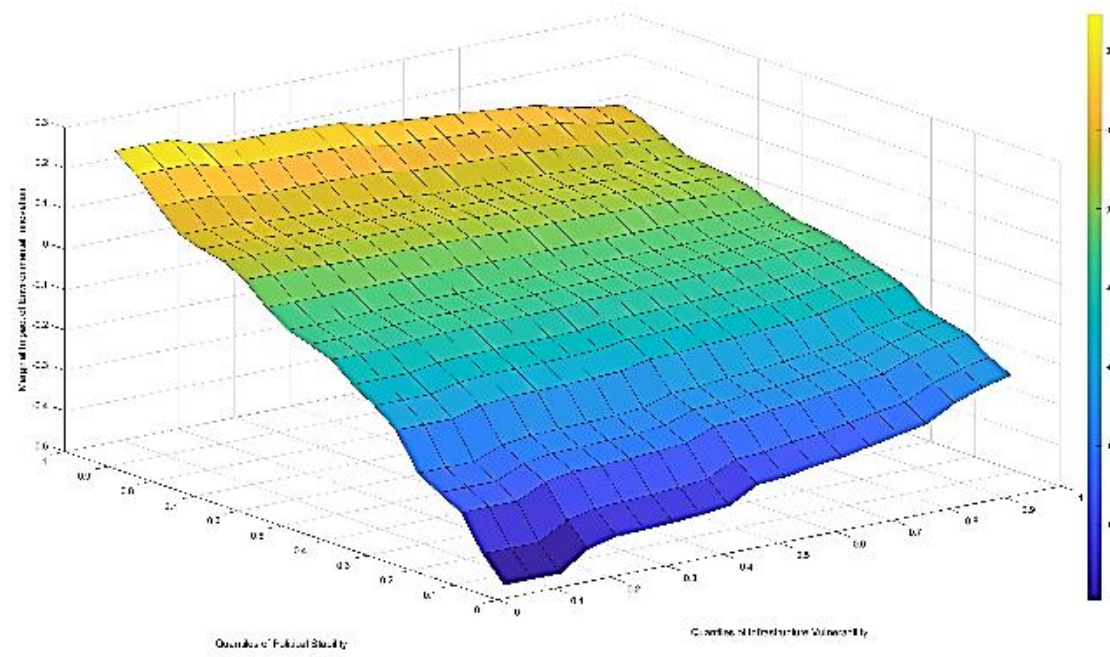

Governance Quality Indicator: Political Stability

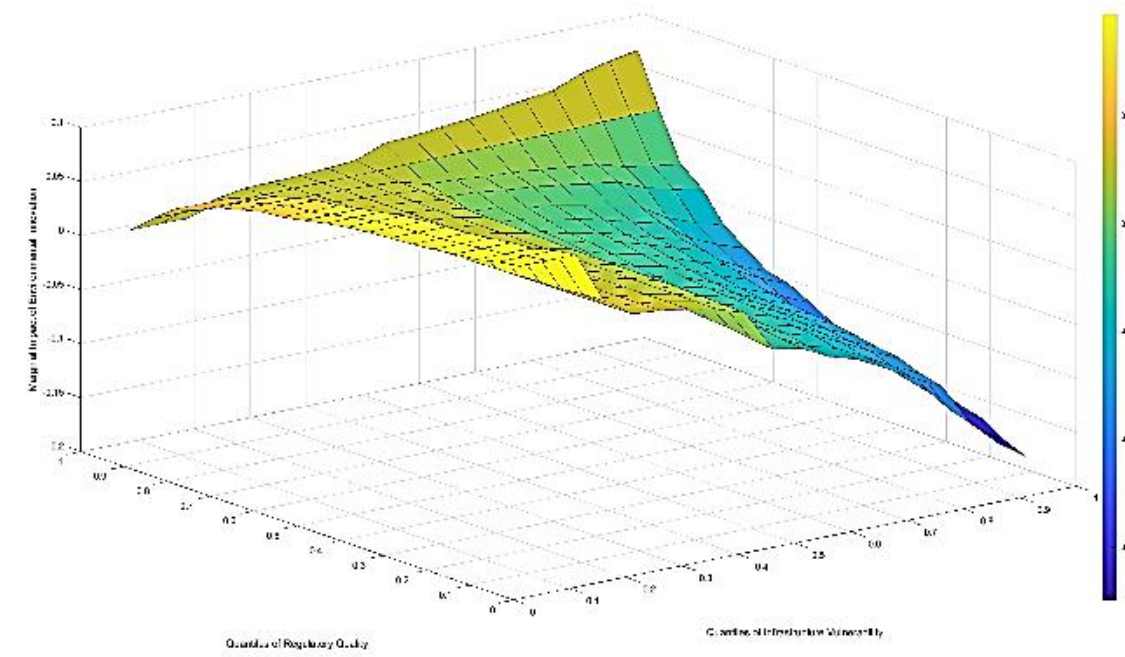

Governance Quality Indicator: Regulatory Quality

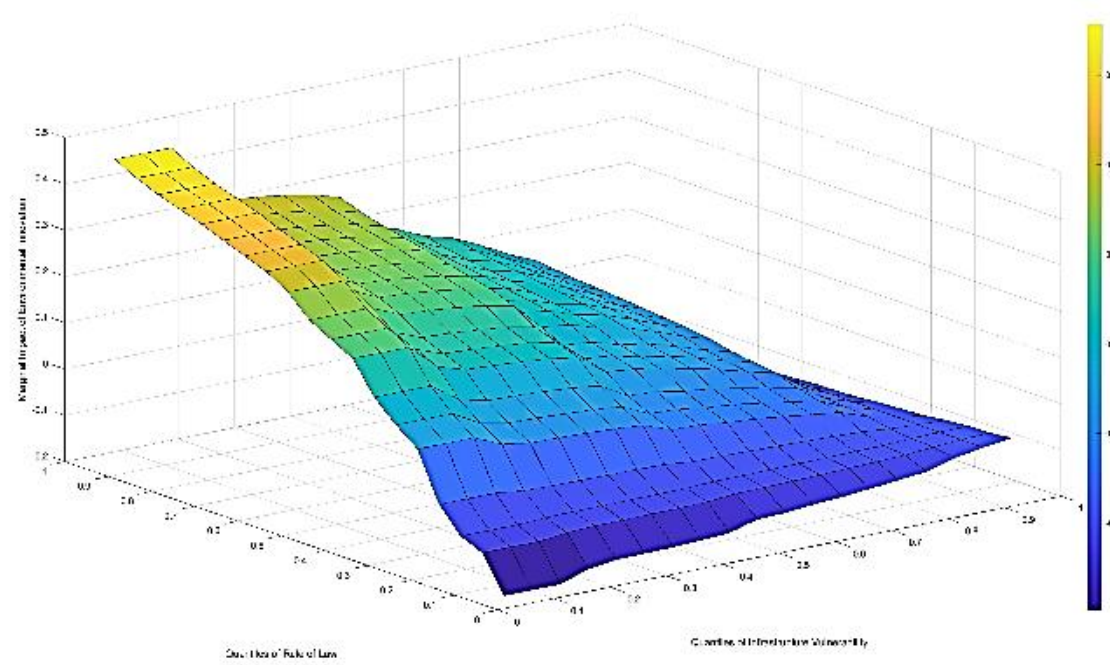

Governance Quality Indicator: Rule of Law

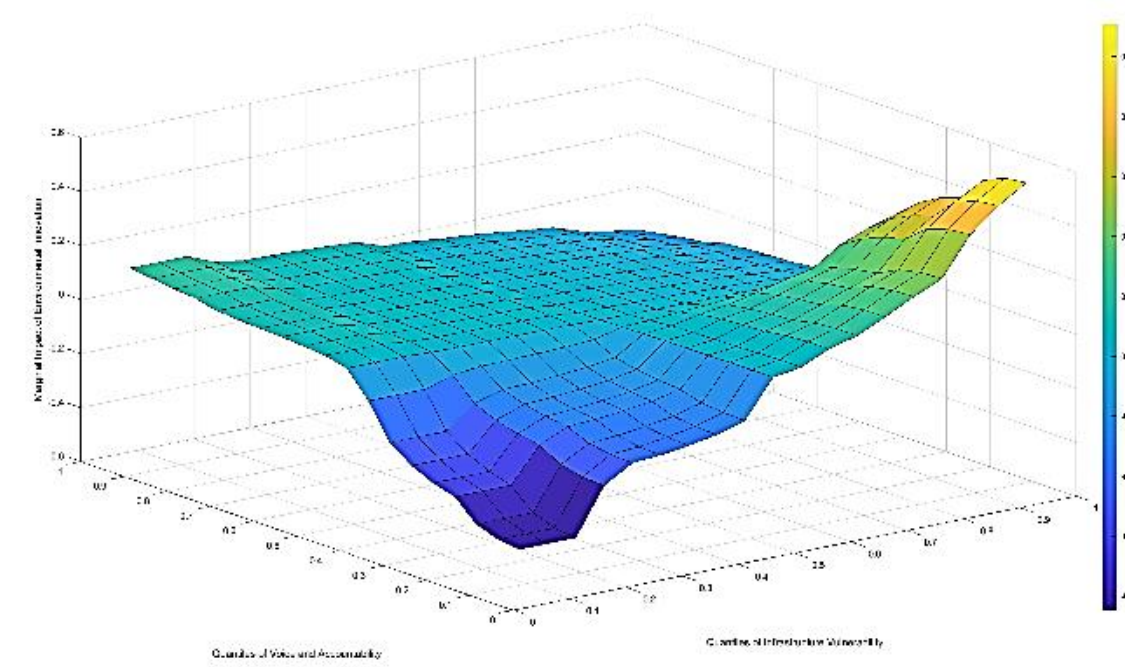

Governance Quality Indicator: Voice and Accountability

Figure S91: BRICS Policy Simulation Outcomes for the Marginal Impact of Environmental Innovation on SDG 7.3.1 under Infrastructure Vulnerability and Governance Quality, Related to Figure 4-5 and Table 2

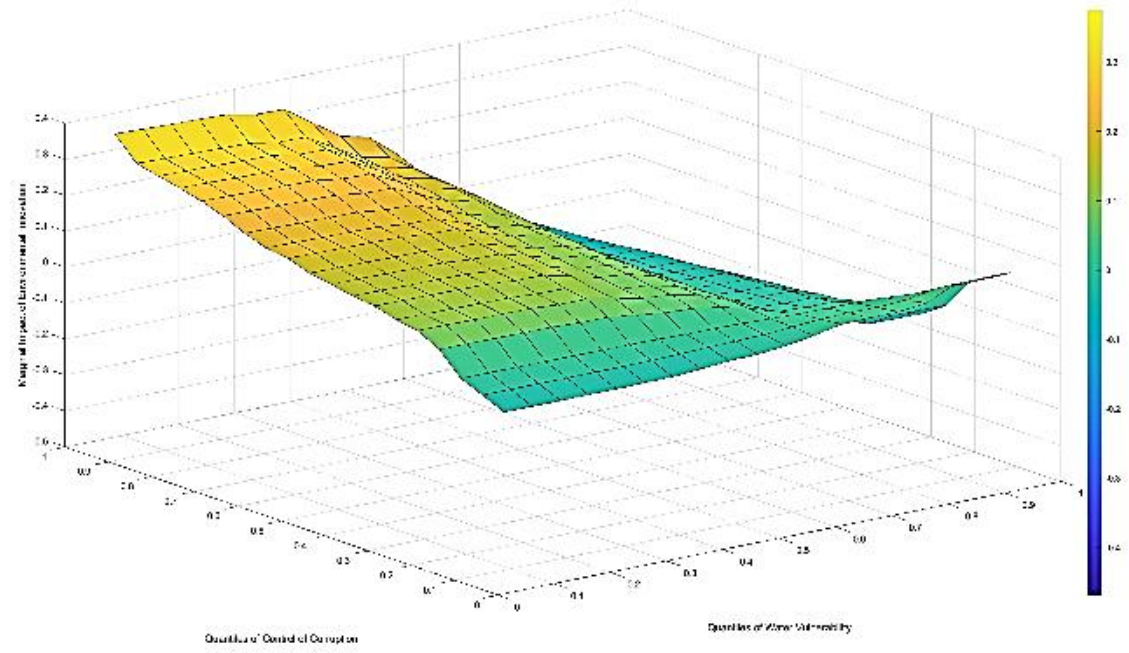

Governance Quality Indicator: Control of Corruption

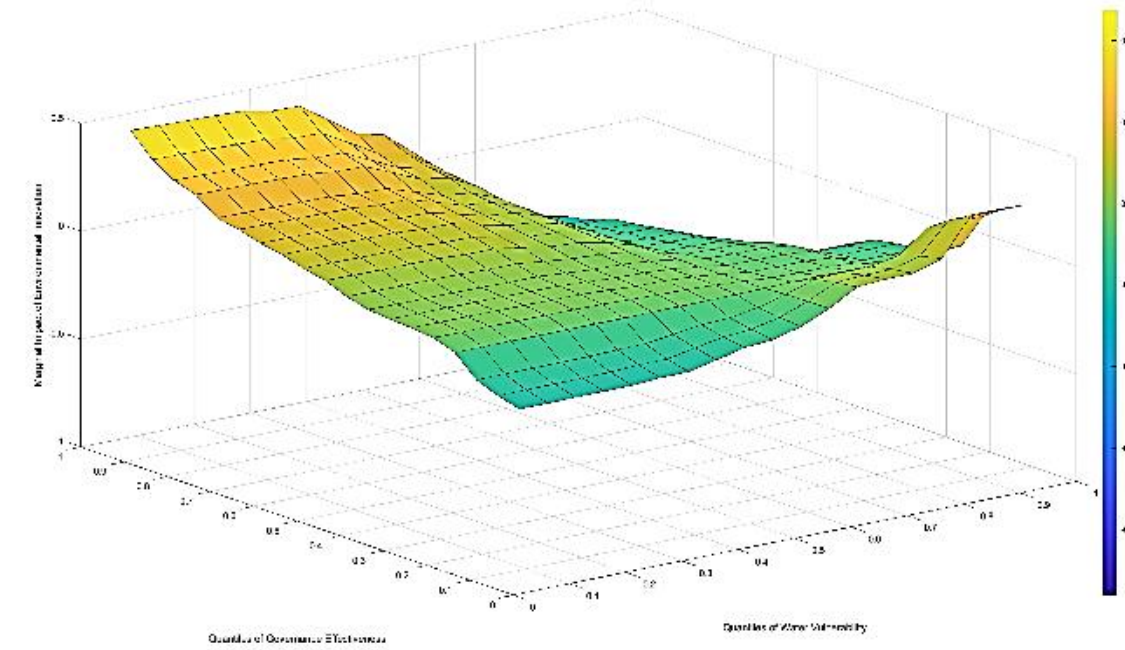

Governance Quality Indicator: Governance Effectiveness

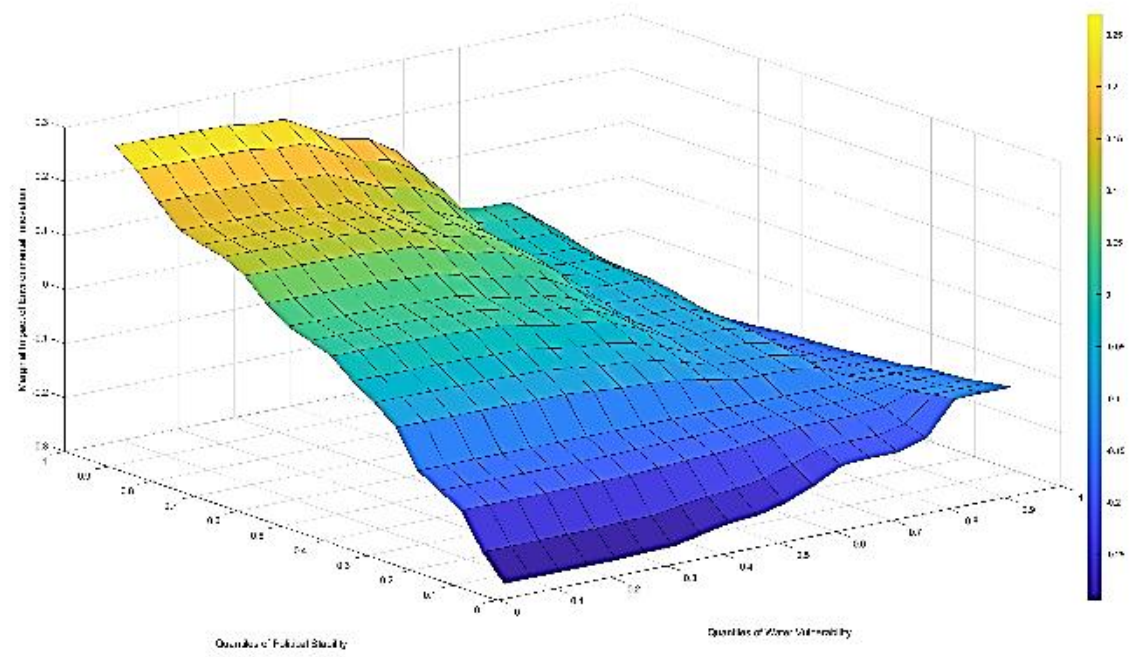

Governance Quality Indicator: Political Stability

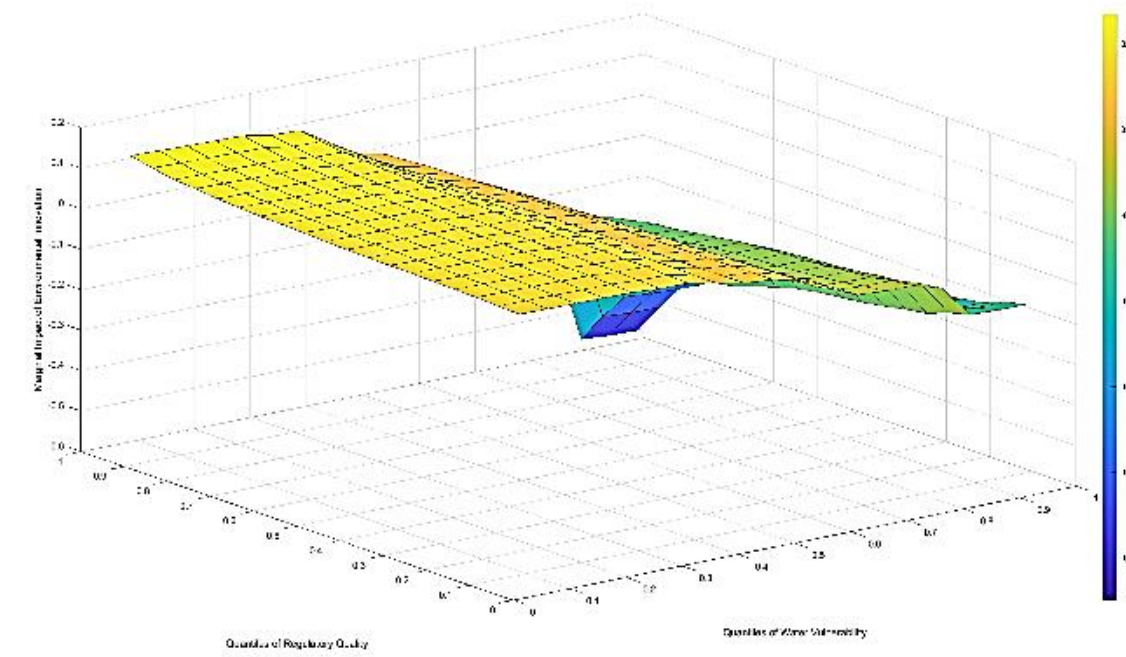

Governance Quality Indicator: Regulatory Quality

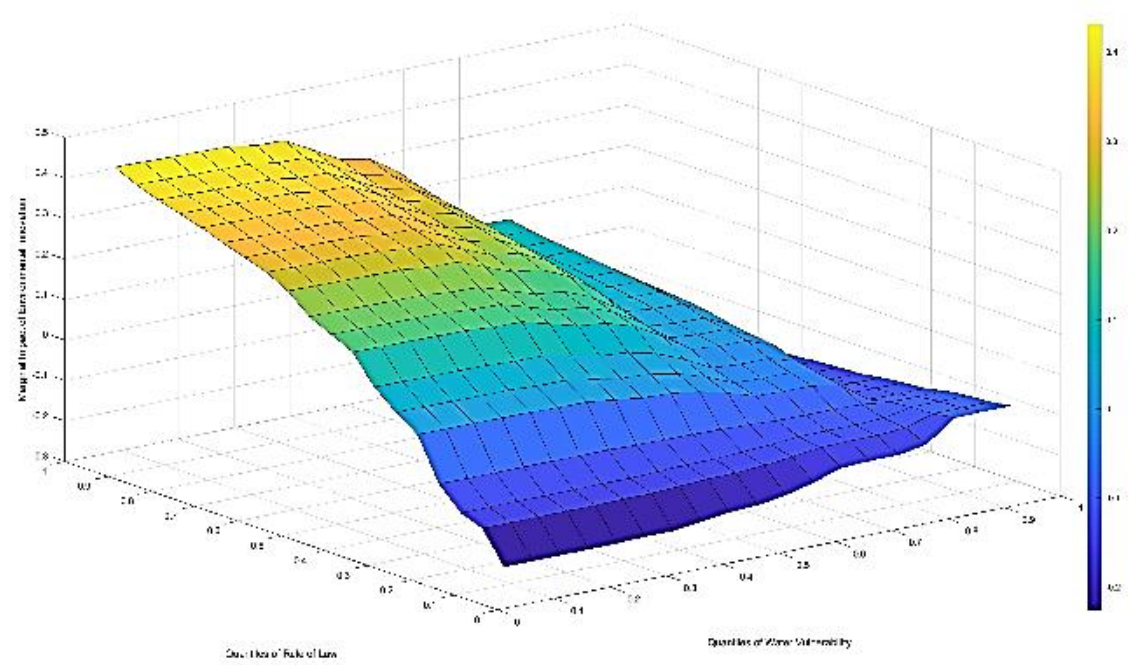

Governance Quality Indicator: Rule of Law

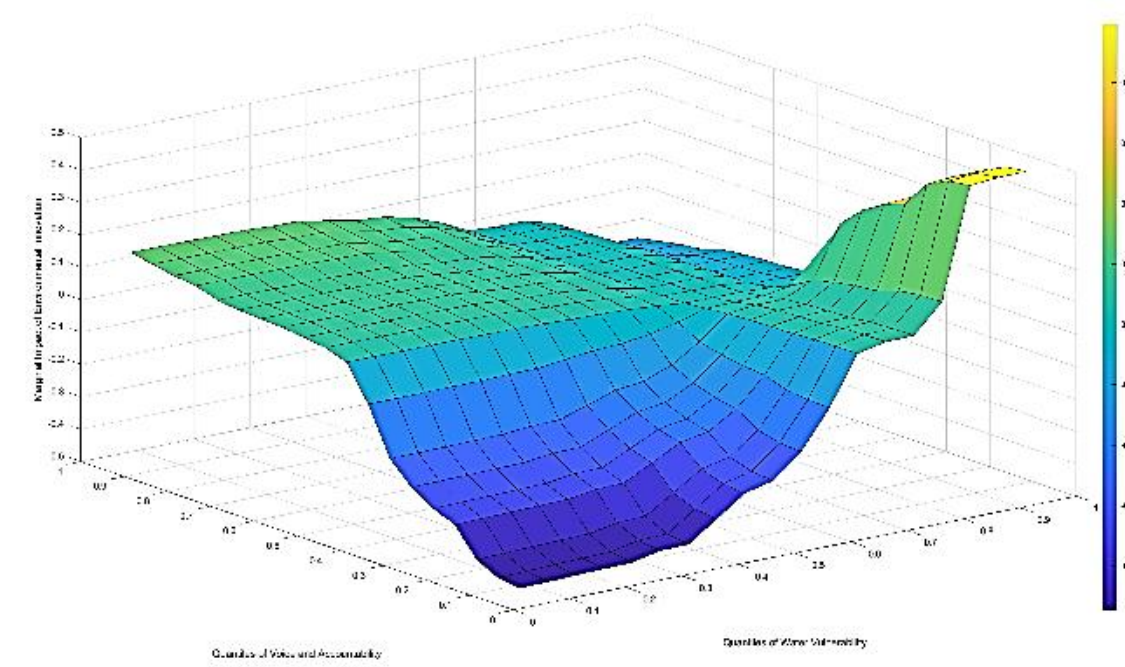

Governance Quality Indicator: Voice and Accountability

Figure S92: BRICS Policy Simulation Outcomes for the Marginal Impact of Environmental Innovation on SDG 7.3.1 under Water Vulnerability and Governance Quality, Related to Figure 4-5 and Table 2

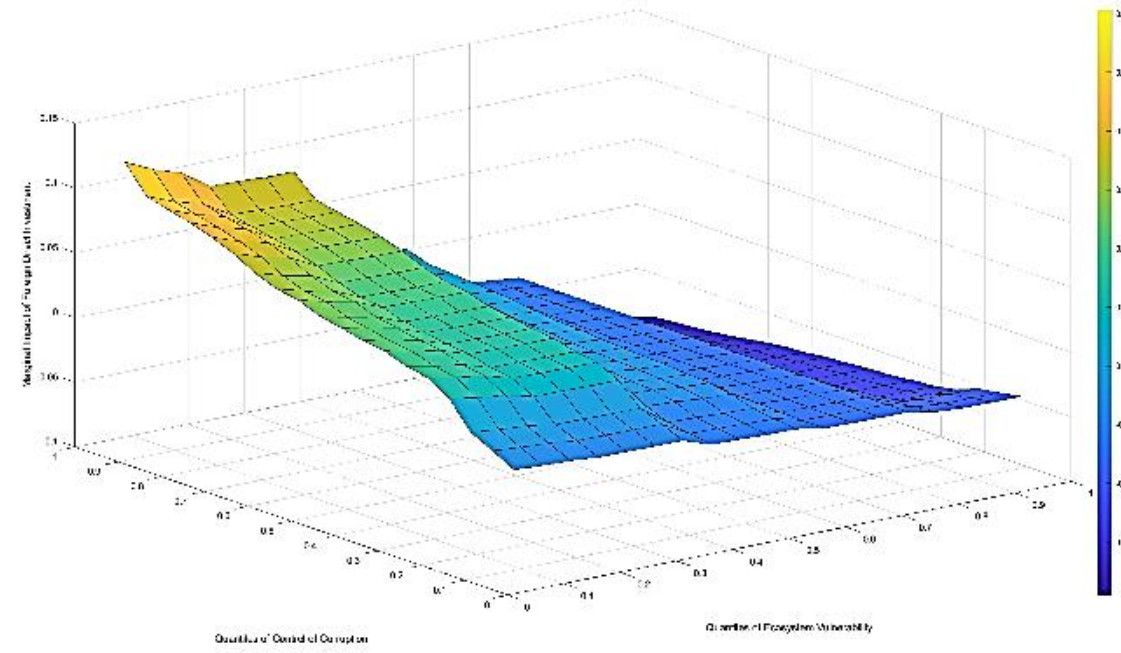

Governance Quality Indicator: Control of Corruption

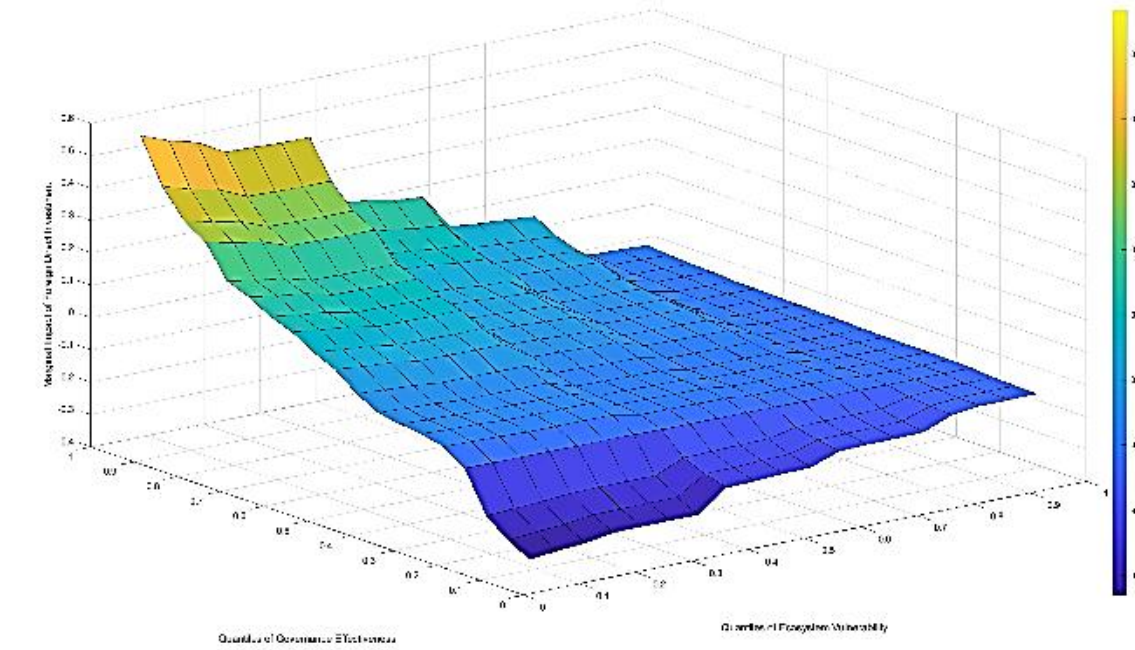

Governance Quality Indicator: Governance Effectiveness

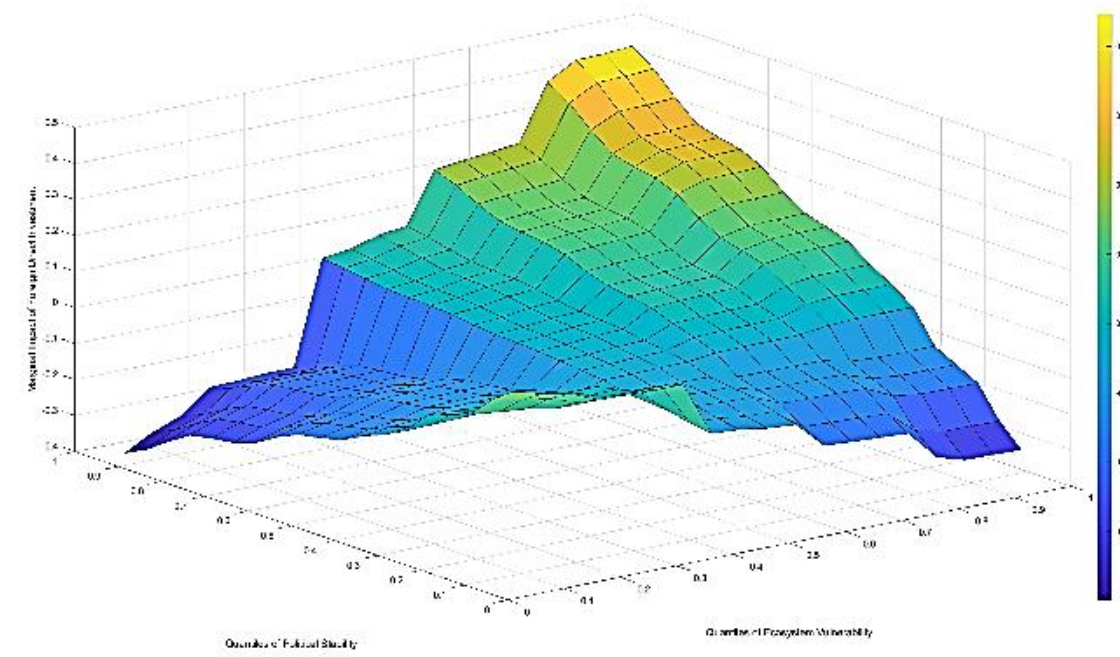

Governance Quality Indicator: Political Stability

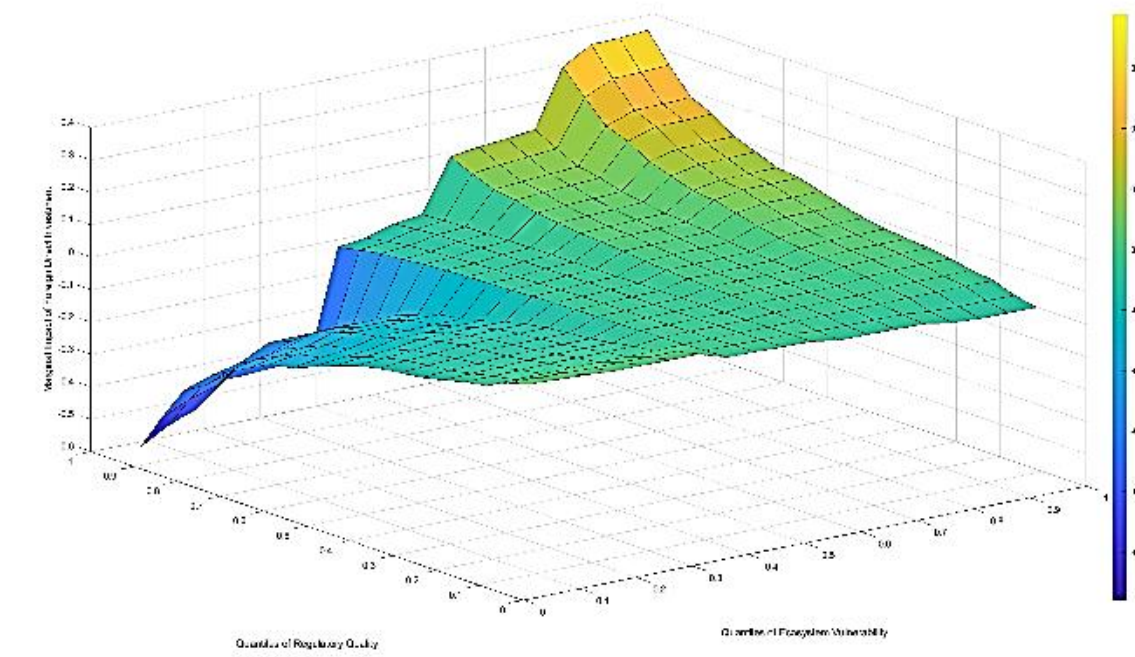

Governance Quality Indicator: Regulatory Quality

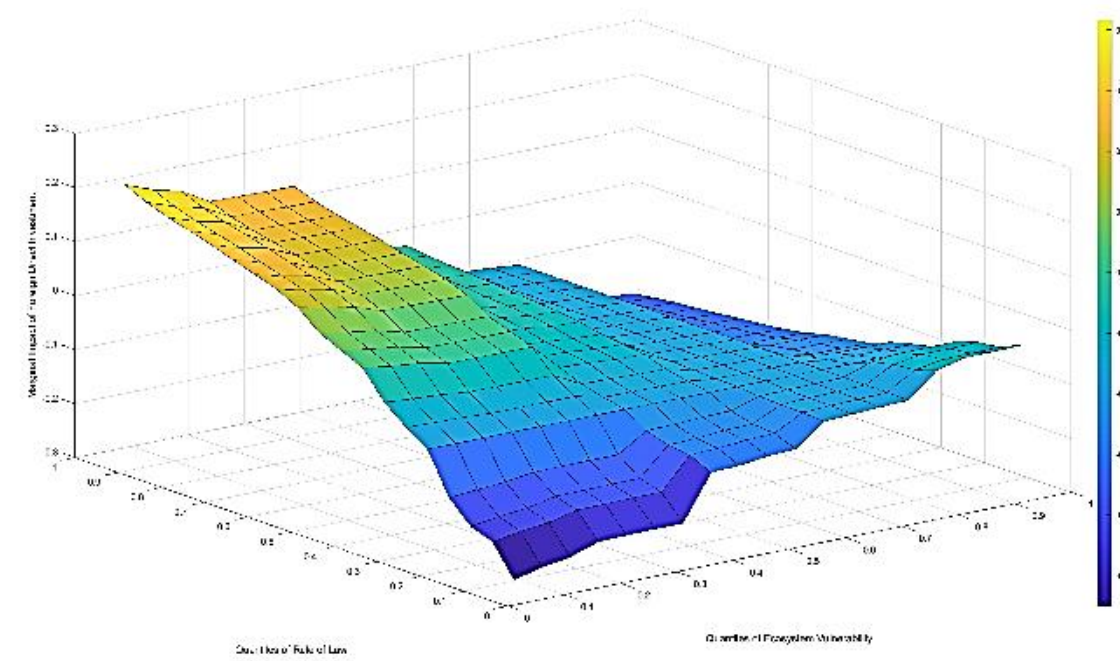

Governance Quality Indicator: Rule of Law

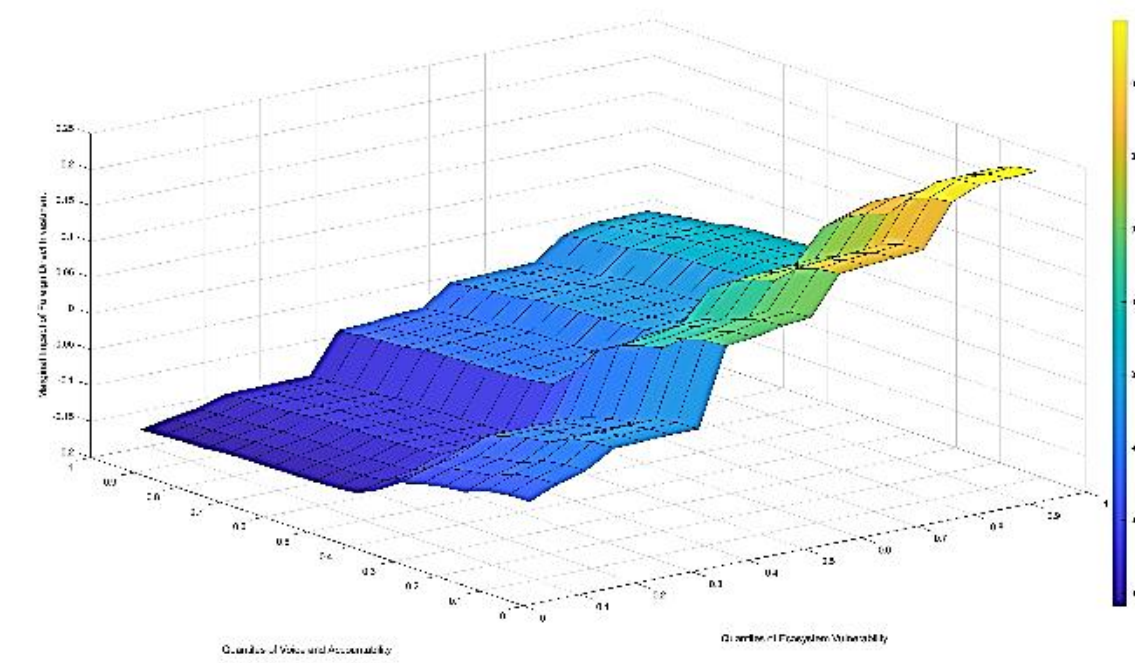

Governance Quality Indicator: Voice and Accountability

Figure S93: BRICS Policy Simulation Outcomes for the Marginal Impact of Foreign Direct Investment on SDG 7.3.1 under Ecosystem Vulnerability and Governance Quality, Related to Figure 4-5 and Table 2

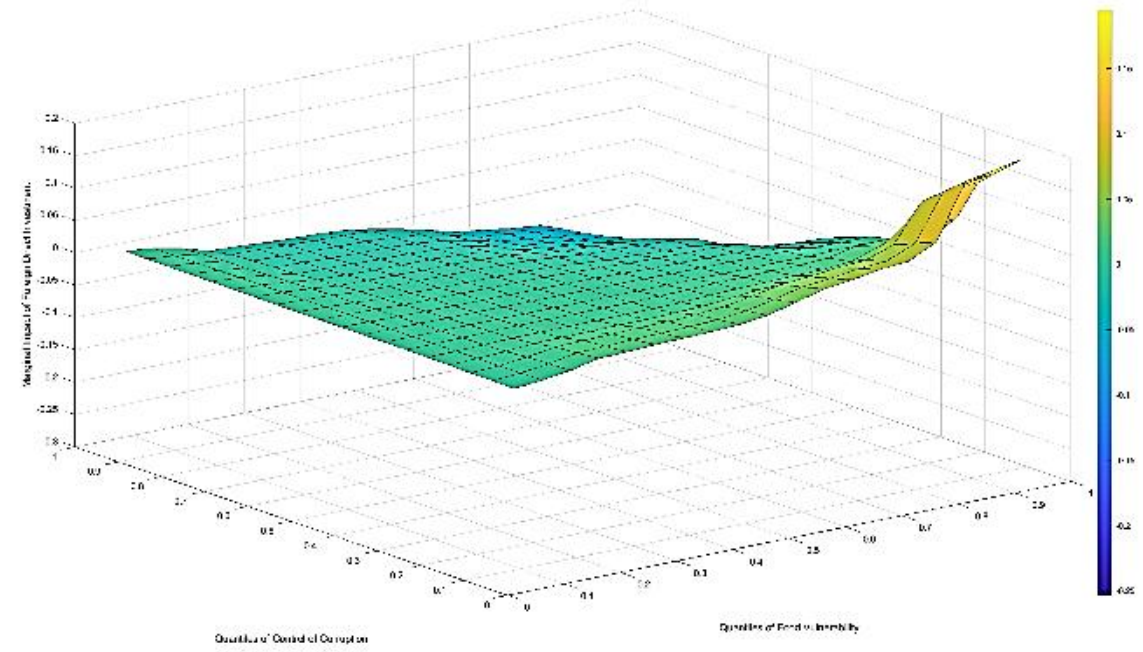

Governance Quality Indicator: Control of Corruption

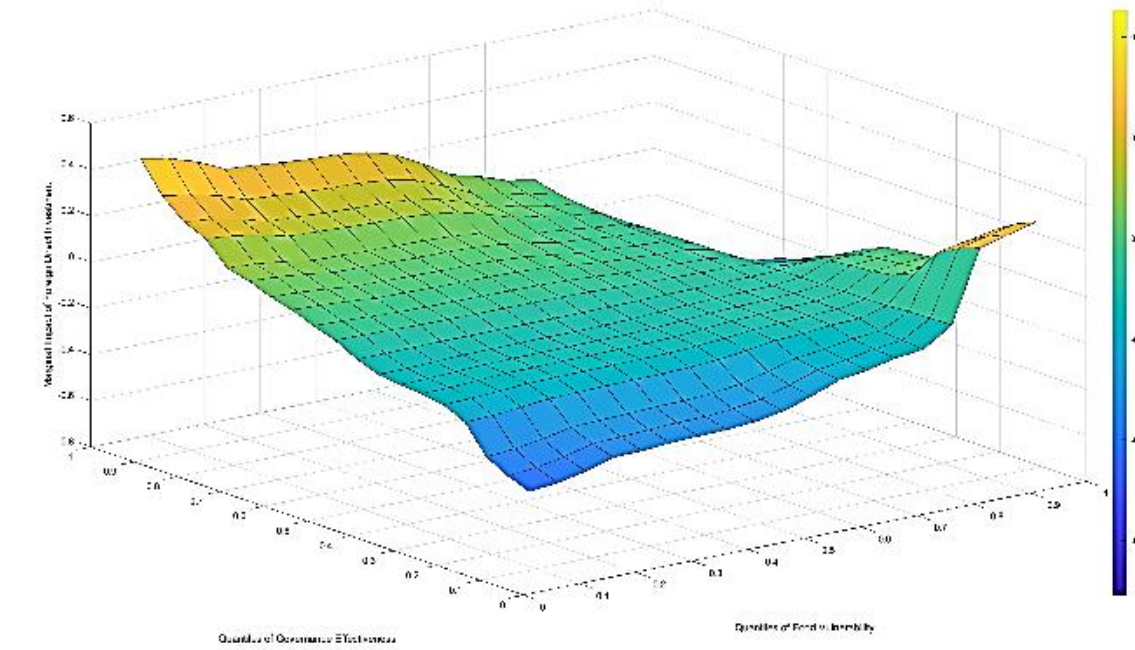

Governance Quality Indicator: Governance Effectiveness

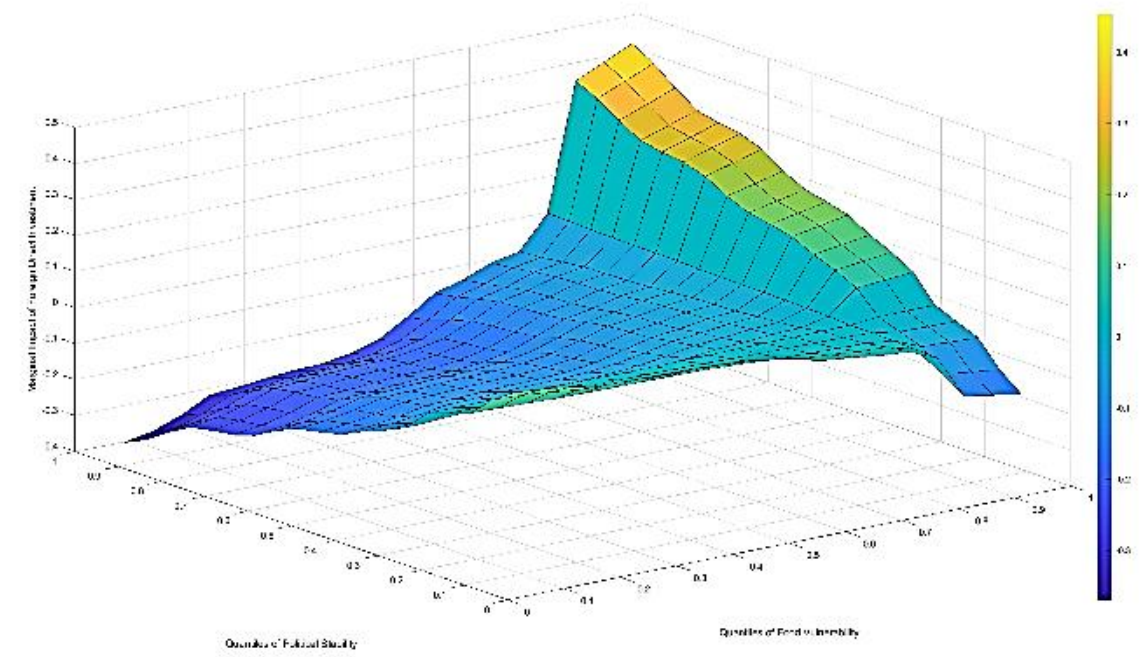

Governance Quality Indicator: Political Stability

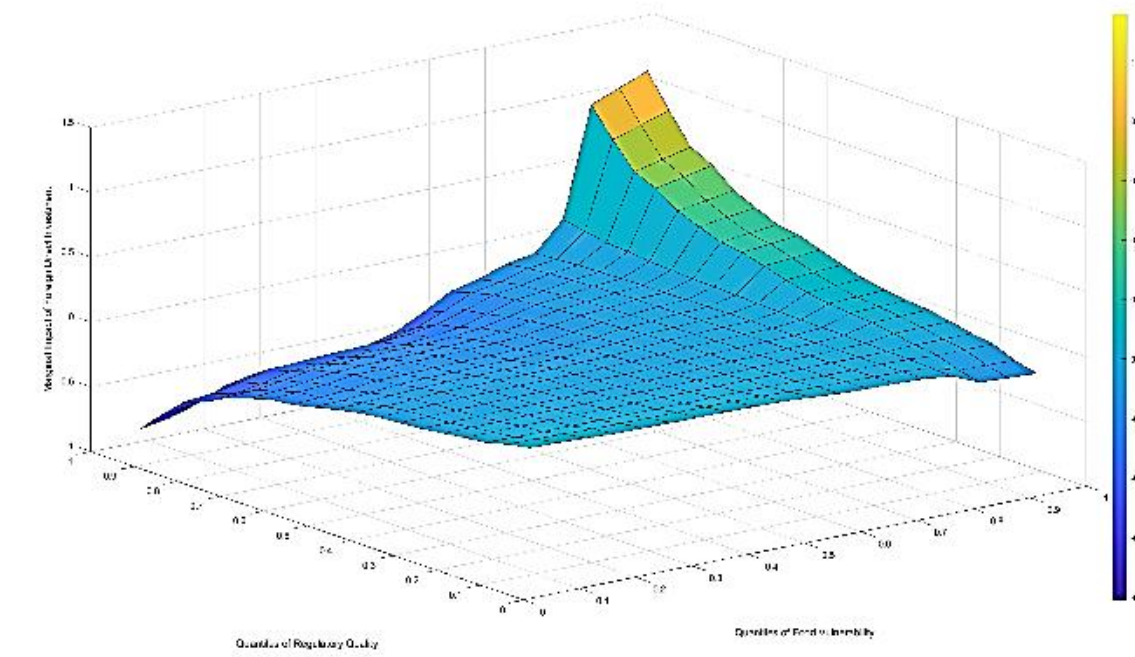

Governance Quality Indicator: Regulatory Quality

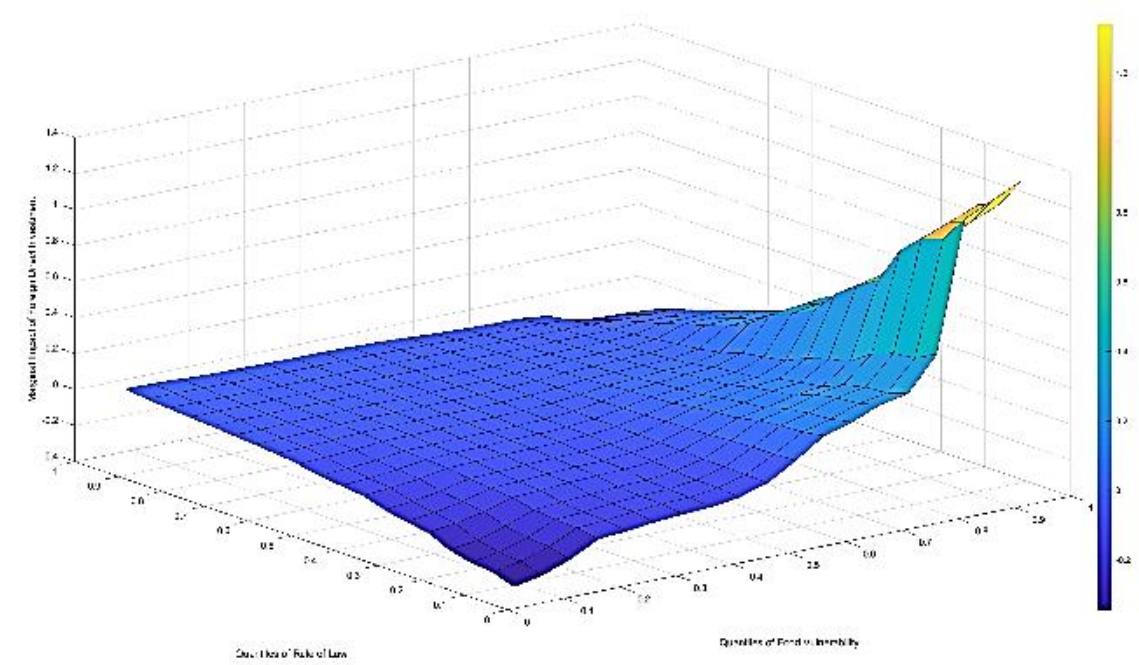

Governance Quality Indicator: Rule of Law

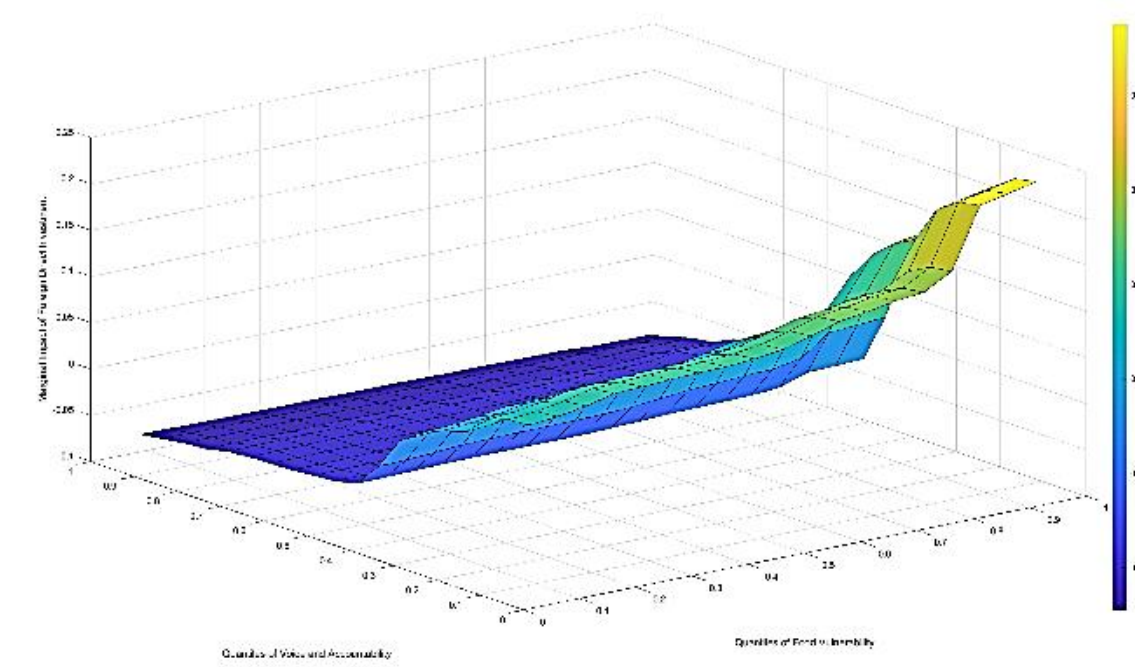

Governance Quality Indicator: Voice and Accountability

Figure S94: BRICS Policy Simulation Outcomes for the Marginal Impact of Foreign Direct Investment on SDG 7.3.1 under Food Vulnerability and Governance Quality, Related to Figure 4-5 and Table 2

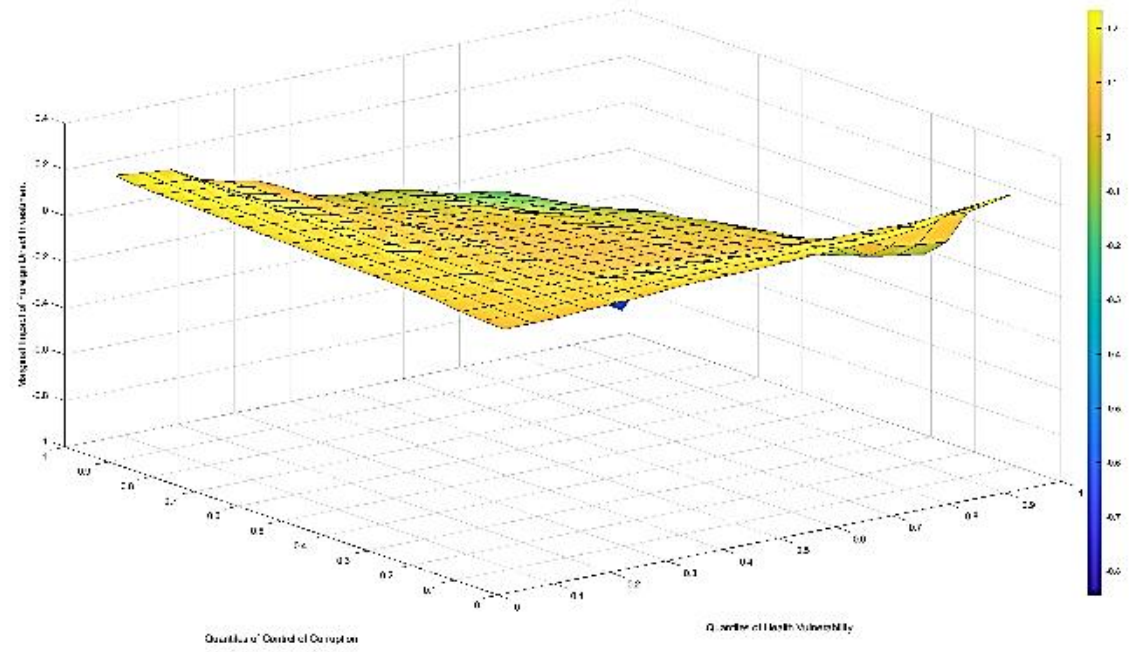

Governance Quality Indicator: Control of Corruption

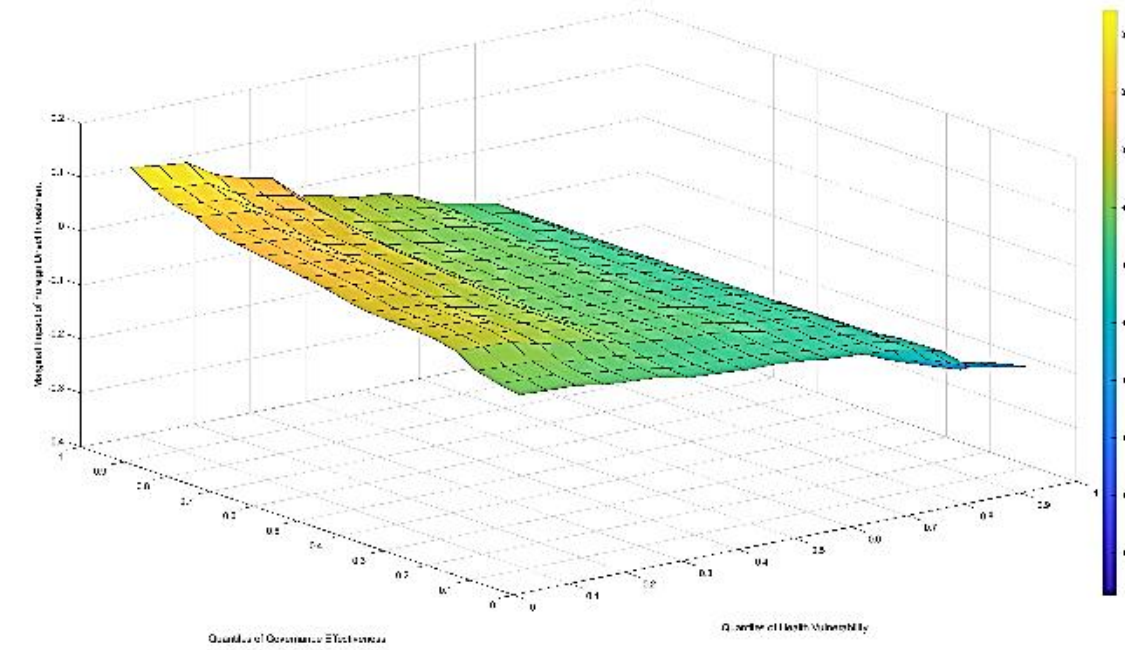

Governance Quality Indicator: Governance Effectiveness

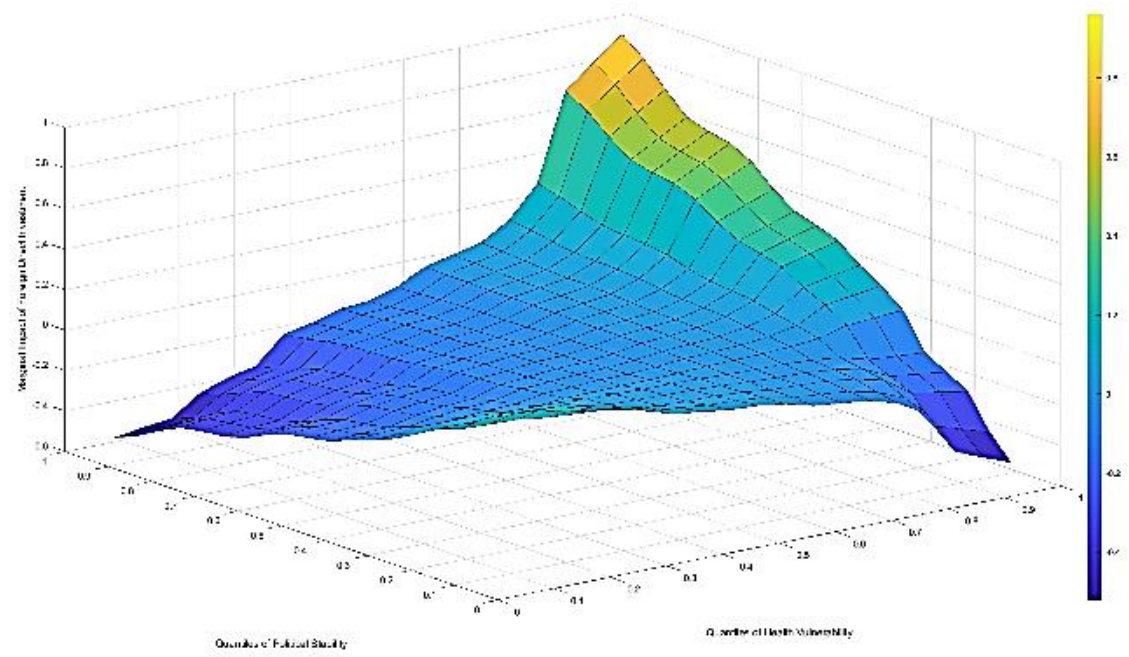

Governance Quality Indicator: Political Stability

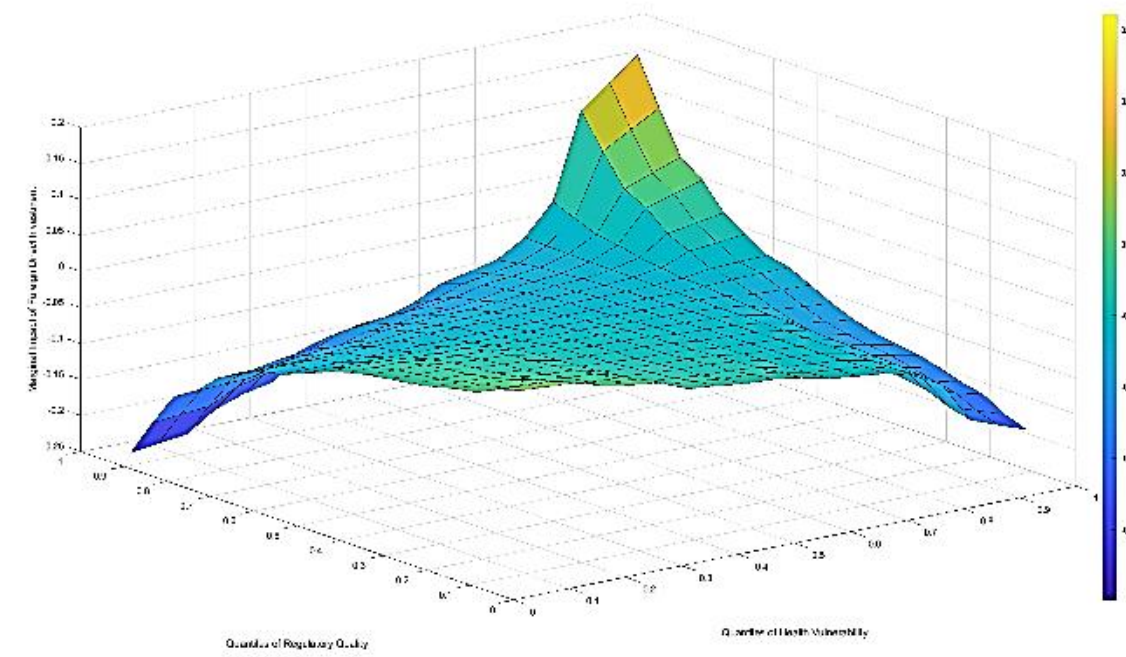

Governance Quality Indicator: Regulatory Quality

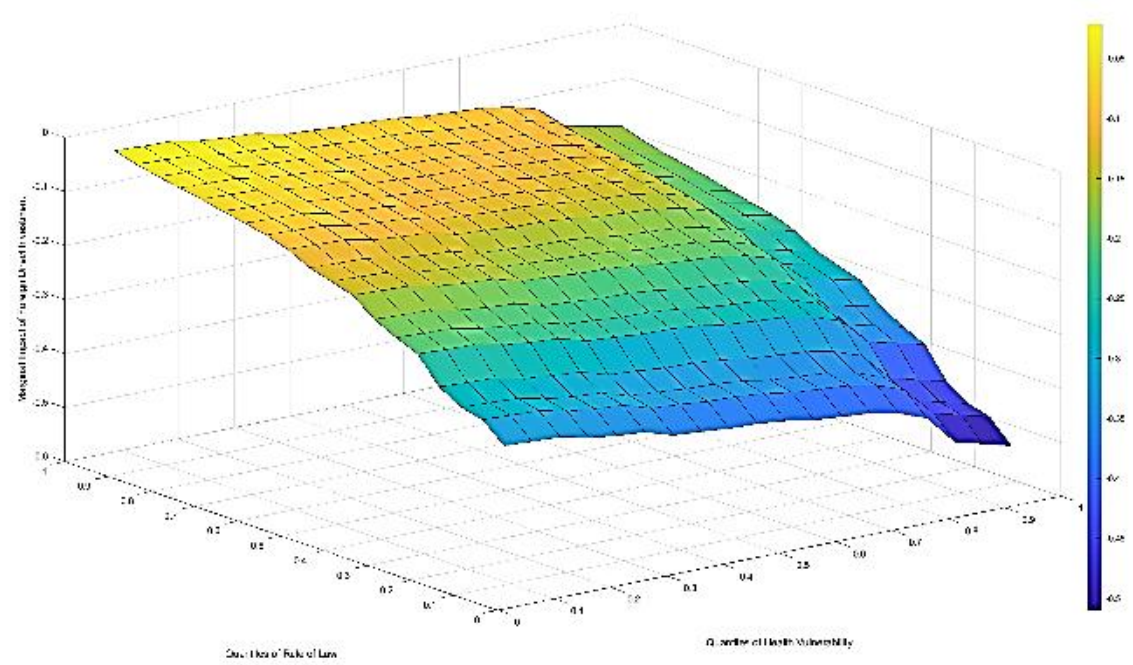

Governance Quality Indicator: Rule of Law

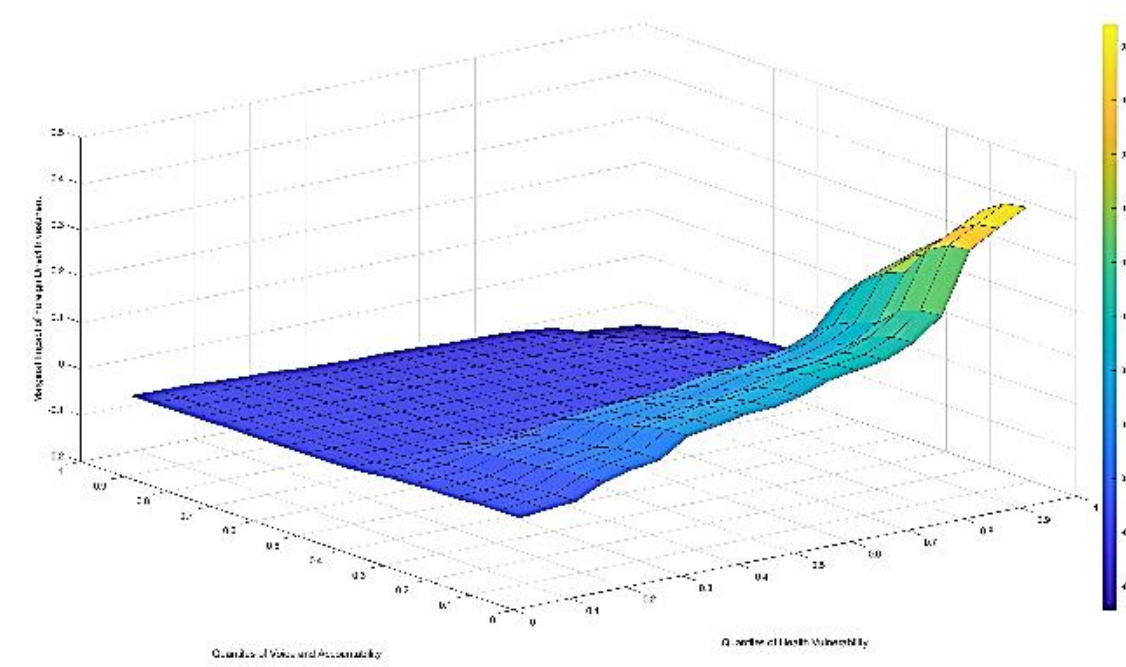

Governance Quality Indicator: Voice and Accountability

Figure S95: BRICS Policy Simulation Outcomes for the Marginal Impact of Foreign Direct Investment on SDG 7.3.1 under Health Vulnerability and Governance Quality, Related to Figure 4-5 and Table 2

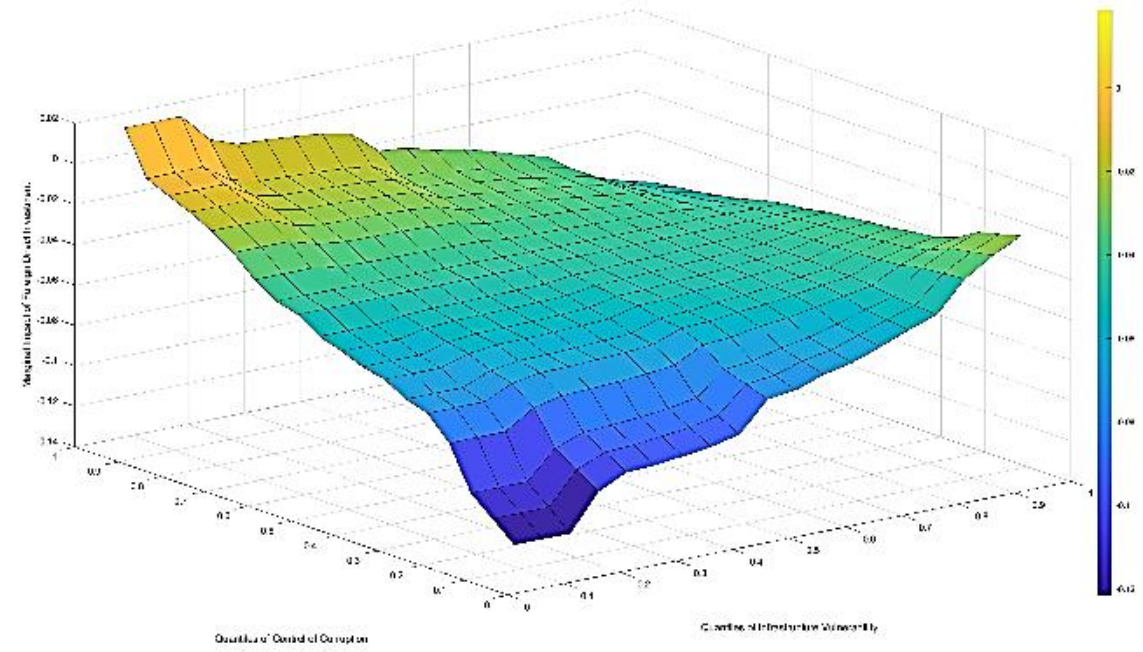

Governance Quality Indicator: Control of Corruption

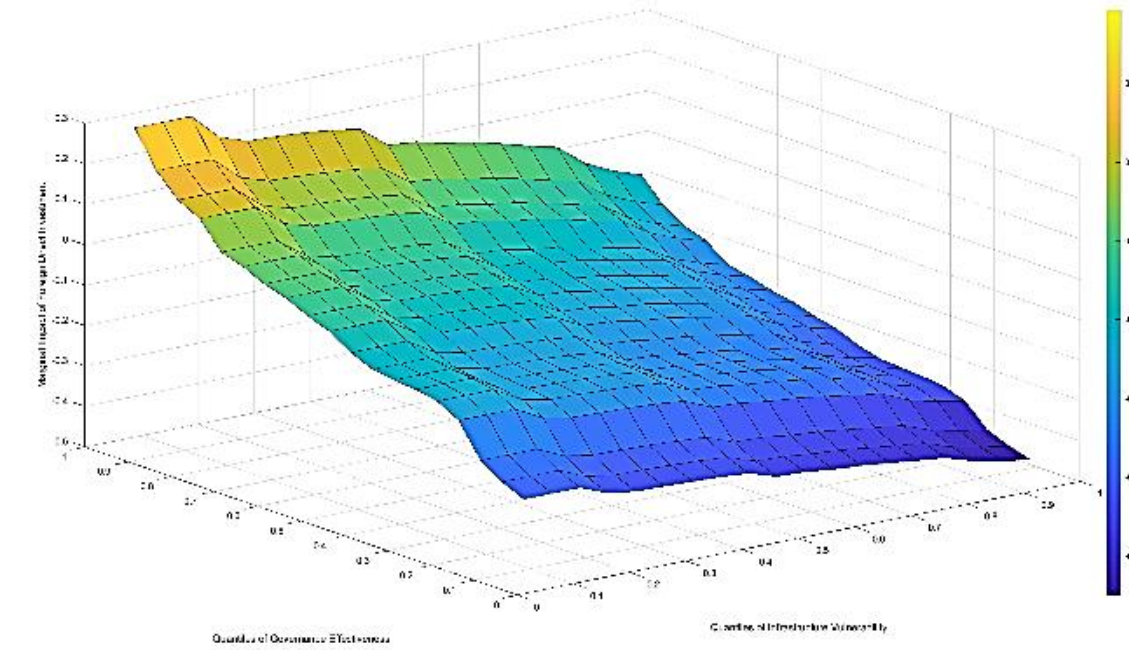

Governance Quality Indicator: Governance Effectiveness

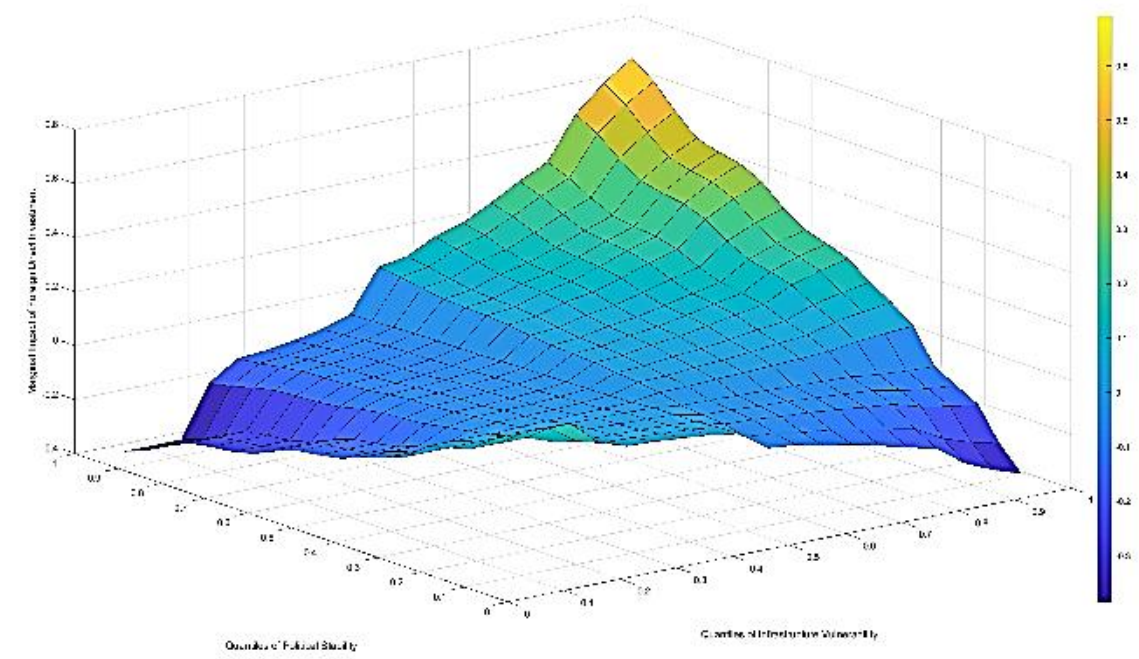

Governance Quality Indicator: Political Stability

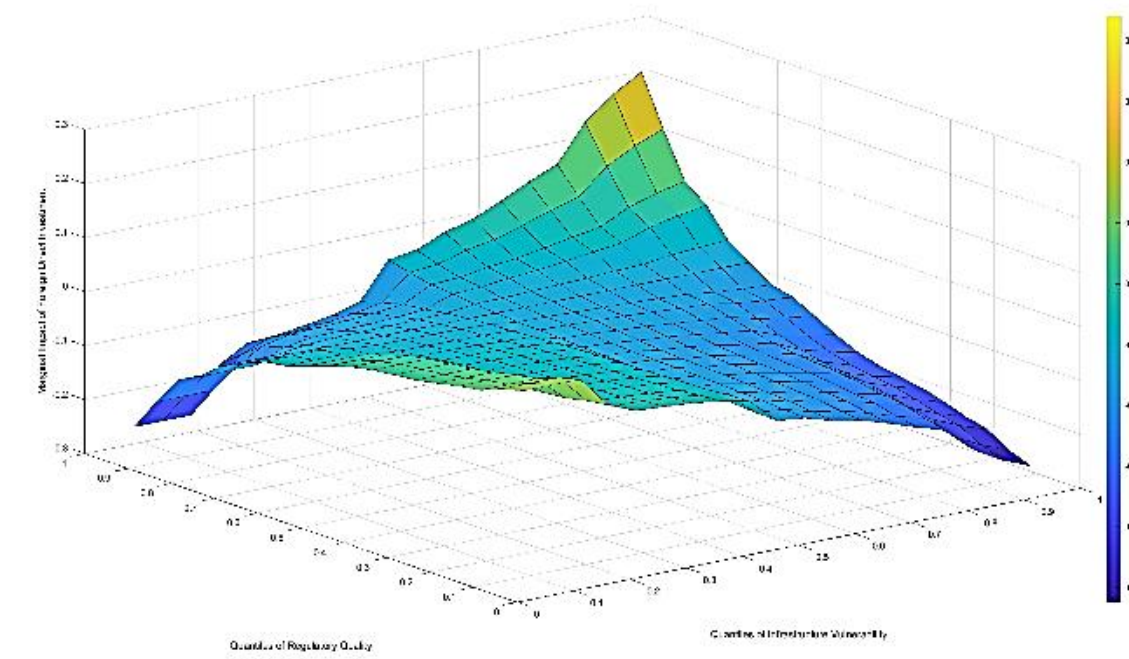

Governance Quality Indicator: Regulatory Quality

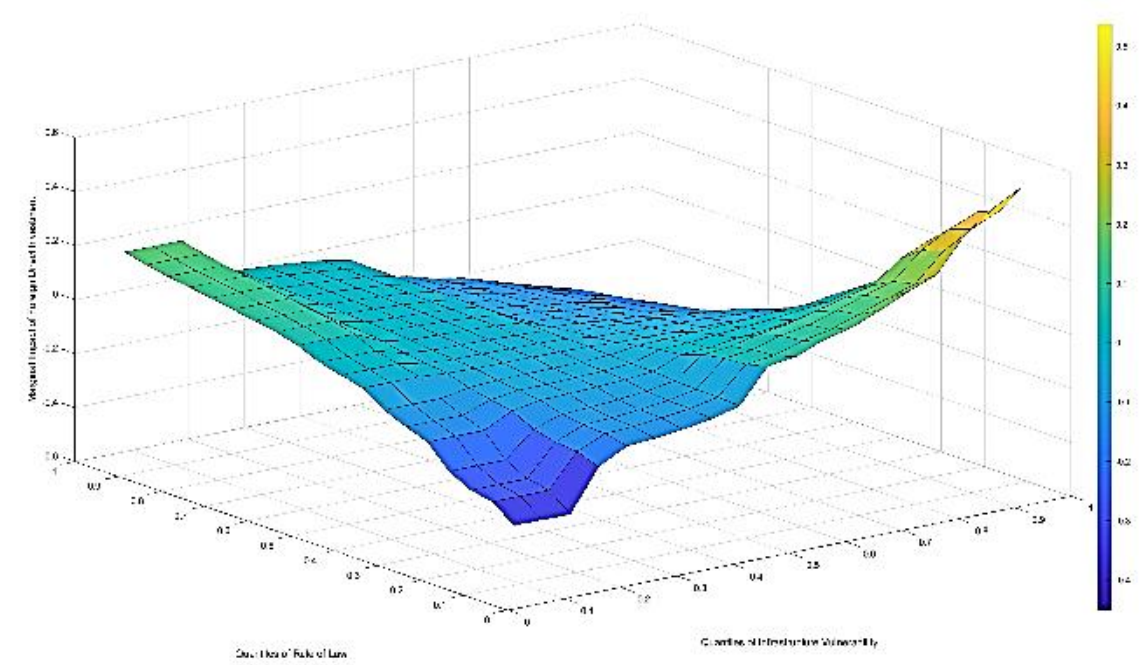

Governance Quality Indicator: Rule of Law

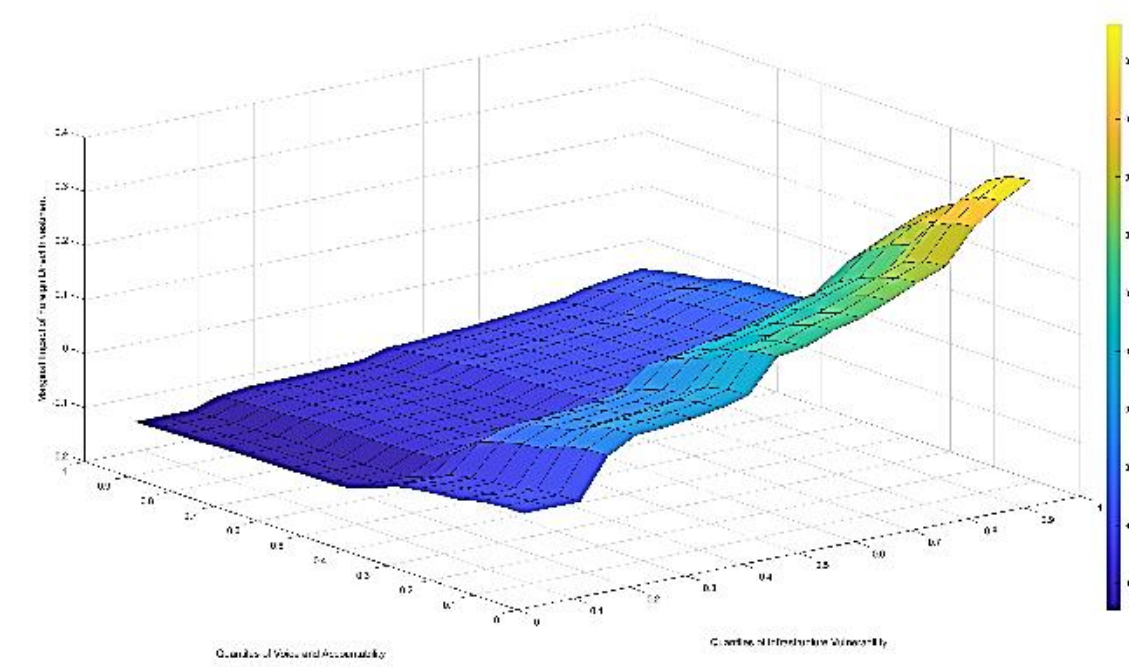

Governance Quality Indicator: Voice and Accountability

Figure S96: BRICS Policy Simulation Outcomes for the Marginal Impact of Foreign Direct Investment on SDG 7.3.1 under Infrastructure Vulnerability and Governance Quality, Related to Figure 4-5 and Table 2

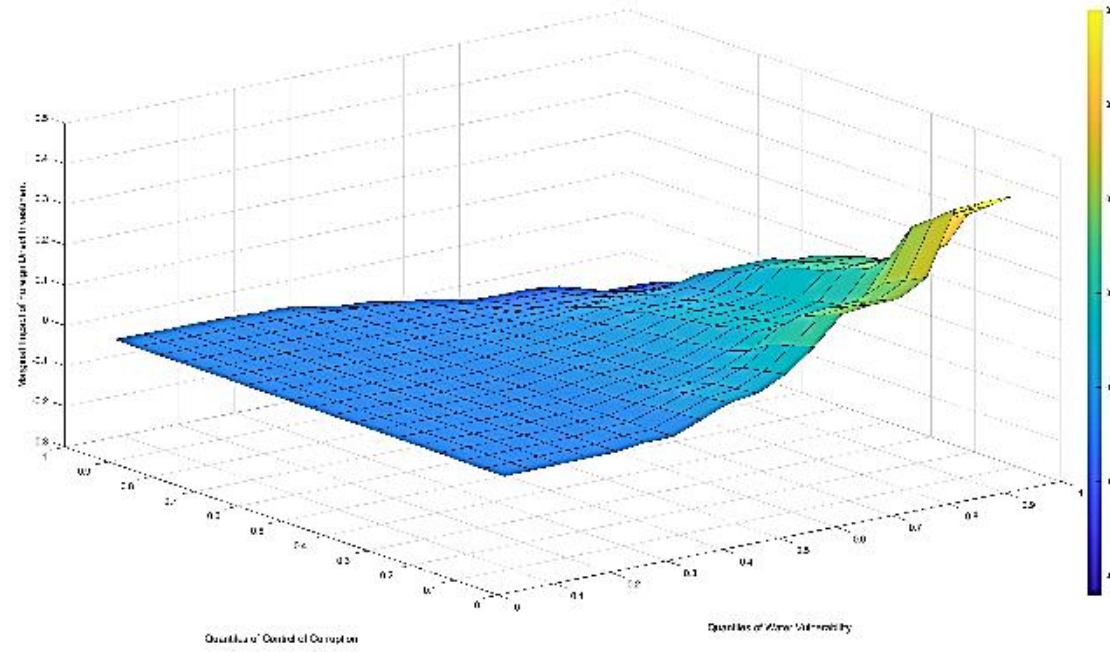

Governance Quality Indicator: Control of Corruption

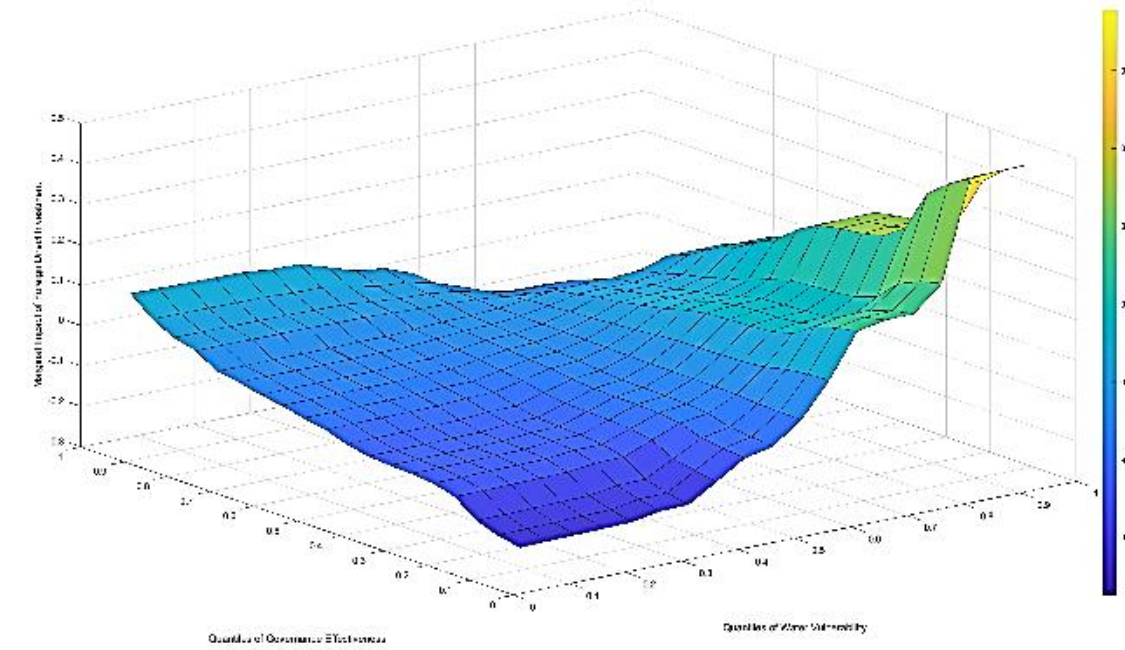

Governance Quality Indicator: Governance Effectiveness

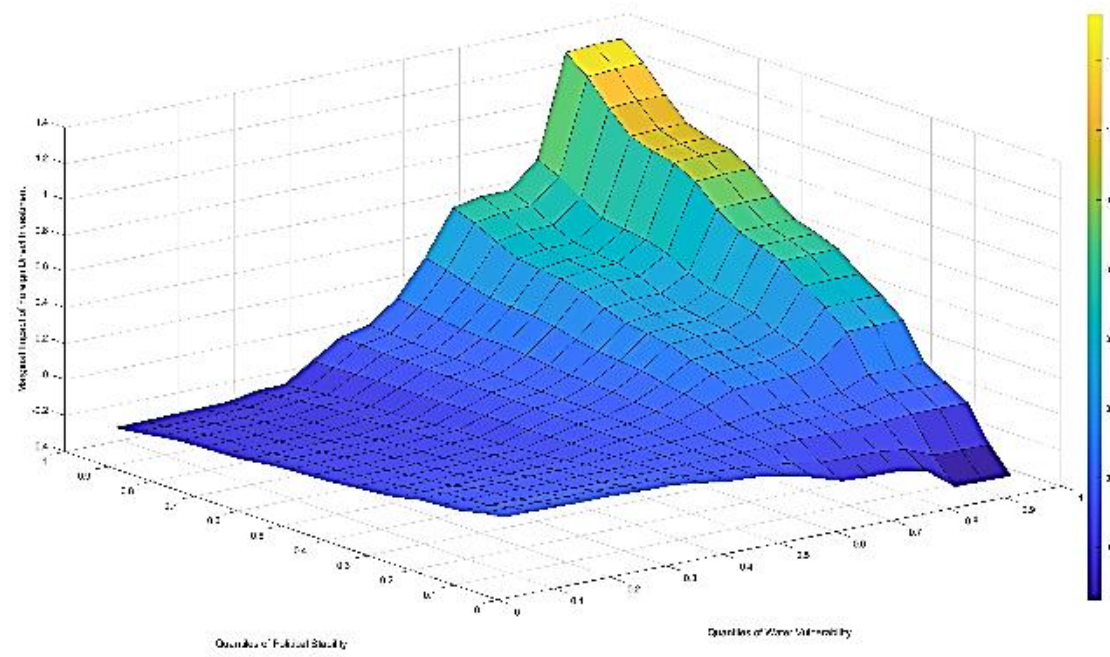

Governance Quality Indicator: Political Stability

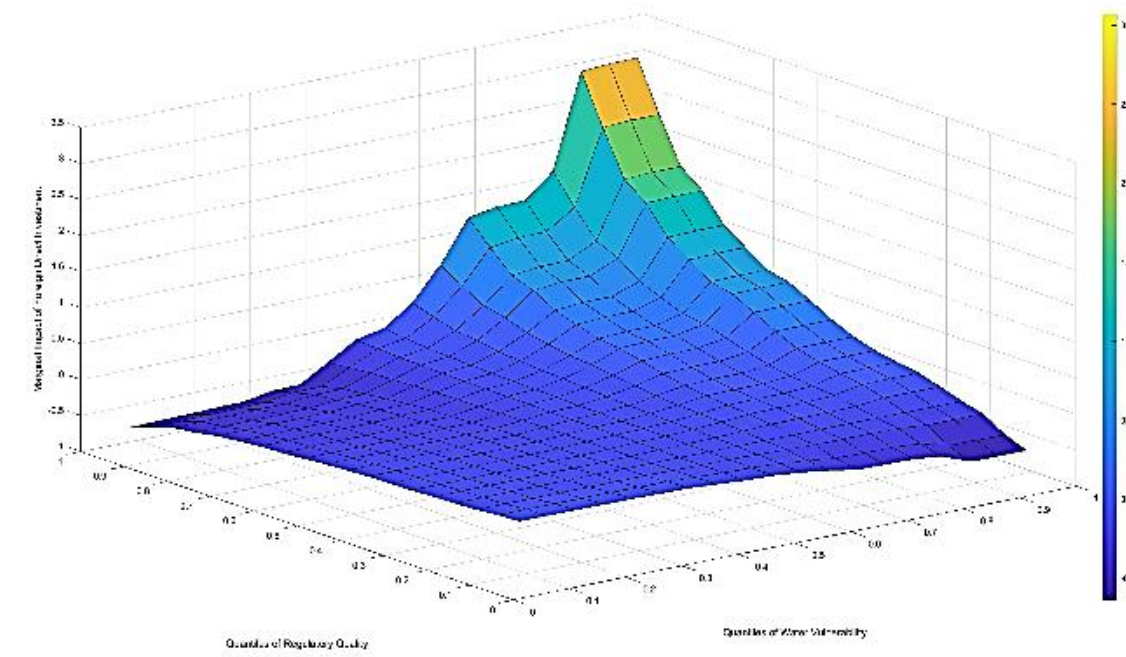

Governance Quality Indicator: Regulatory Quality

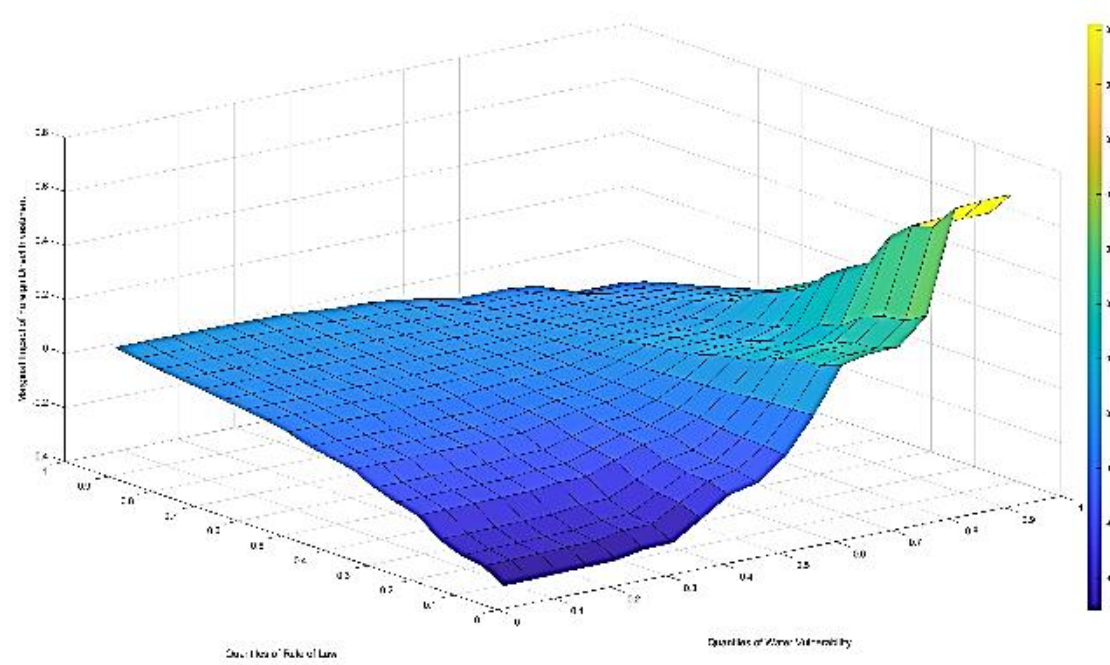

Governance Quality Indicator: Rule of Law

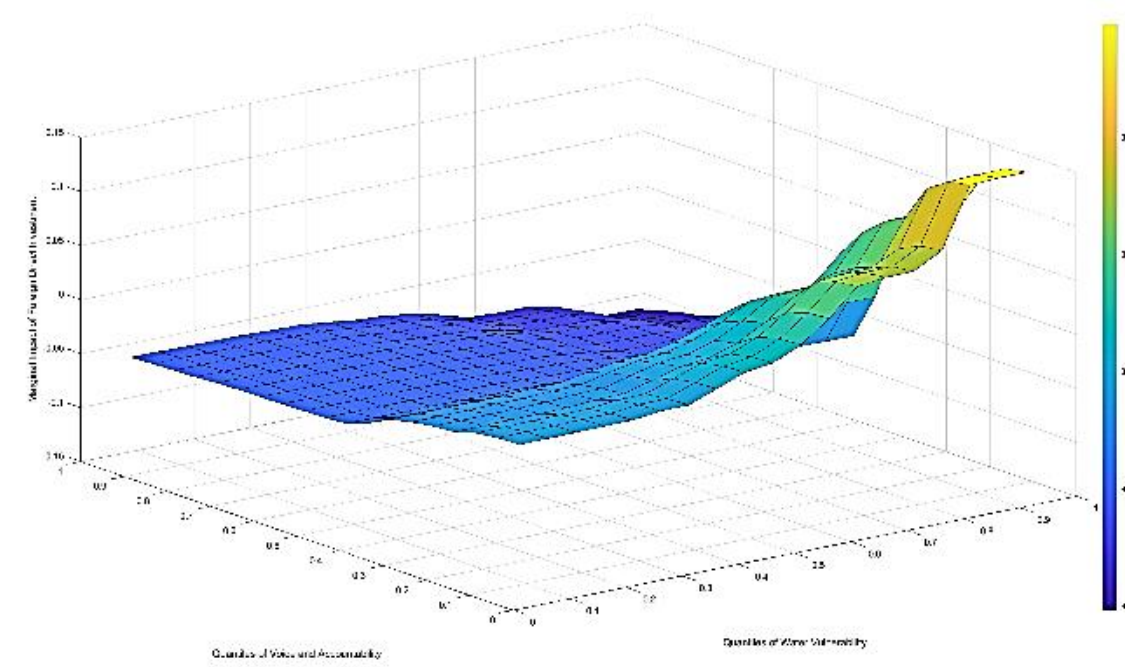

Governance Quality Indicator: Voice and Accountability

Figure S97: BRICS Policy Simulation Outcomes for the Marginal Impact of Foreign Direct Investment on SDG 7.3.1 under Water Vulnerability and Governance Quality, Related to Figure 4-5 and Table 2

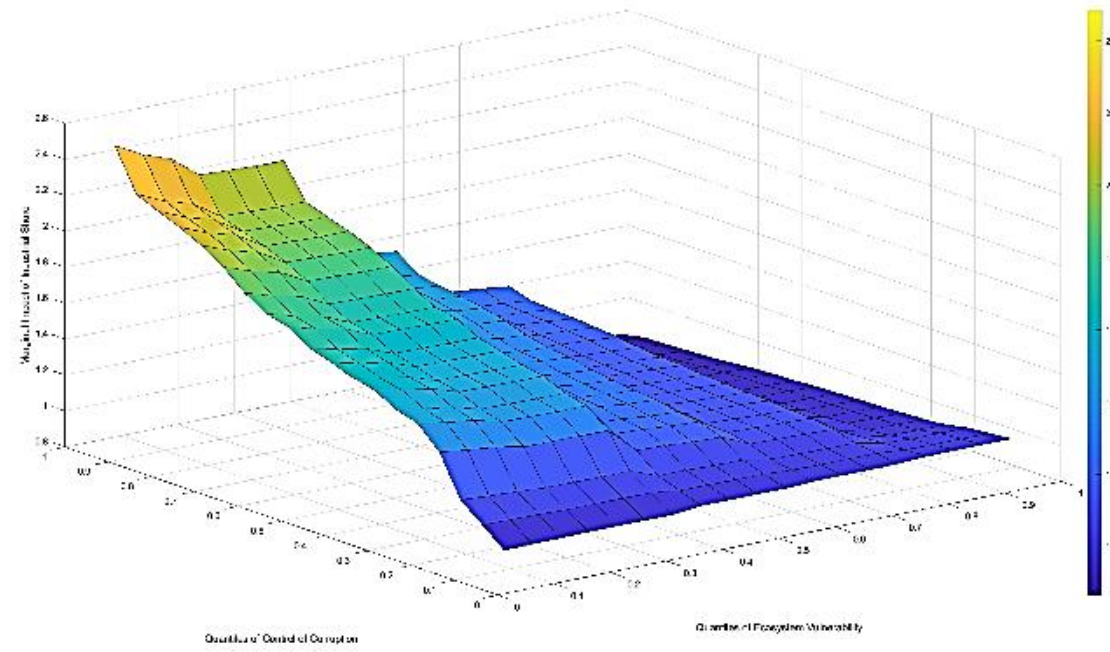

Governance Quality Indicator: Control of Corruption

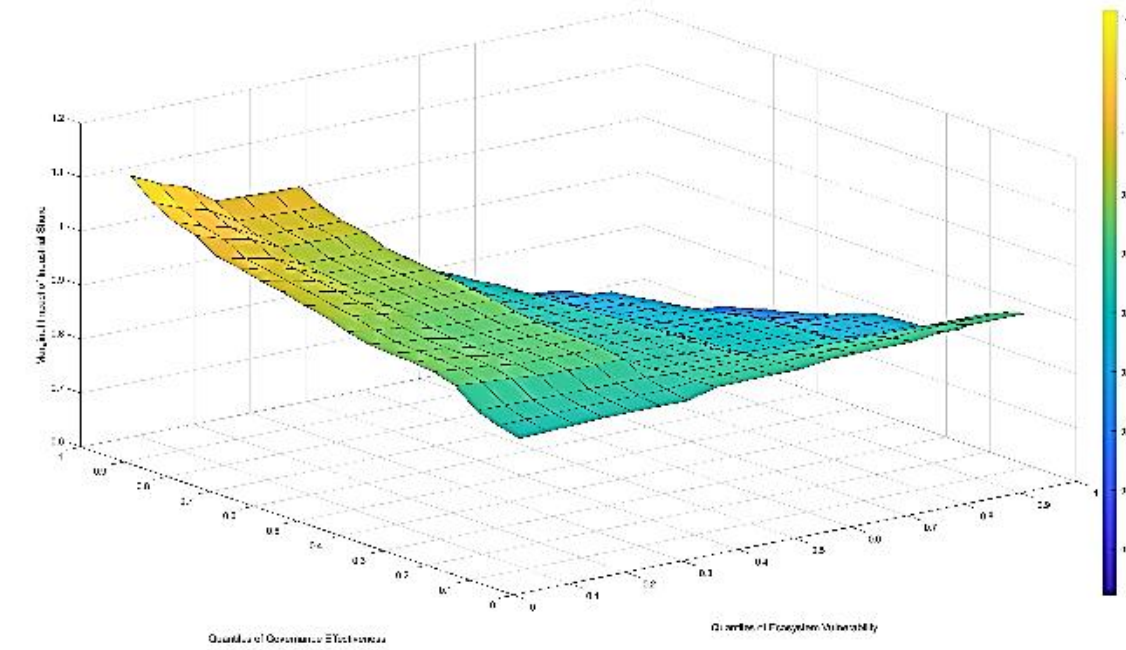

Governance Quality Indicator: Governance Effectiveness

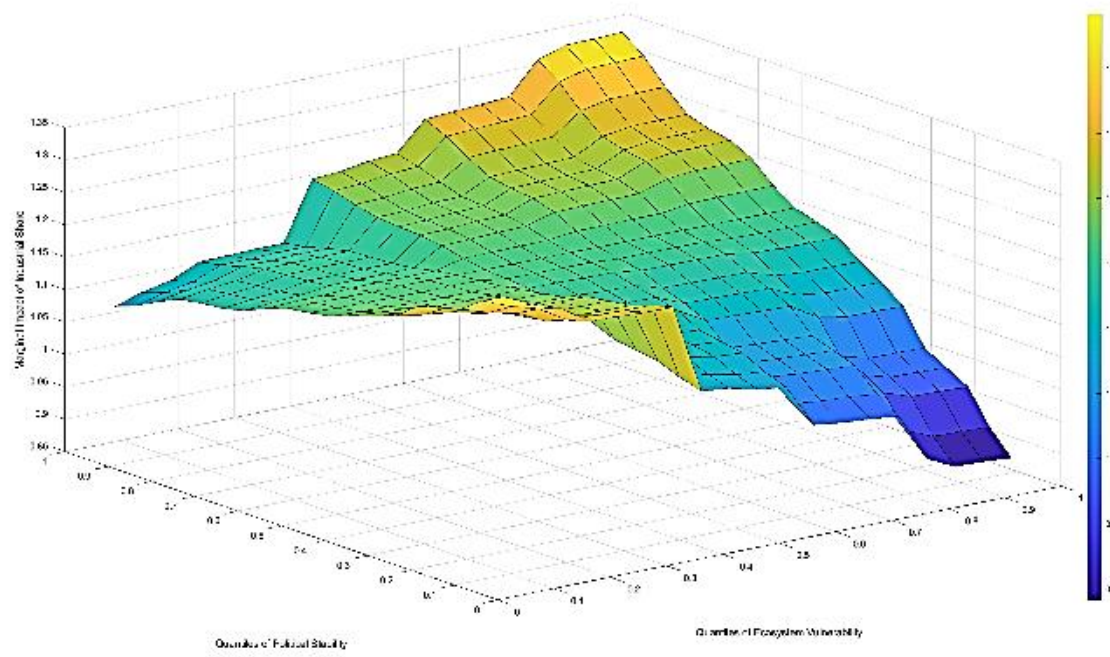

Governance Quality Indicator: Political Stability

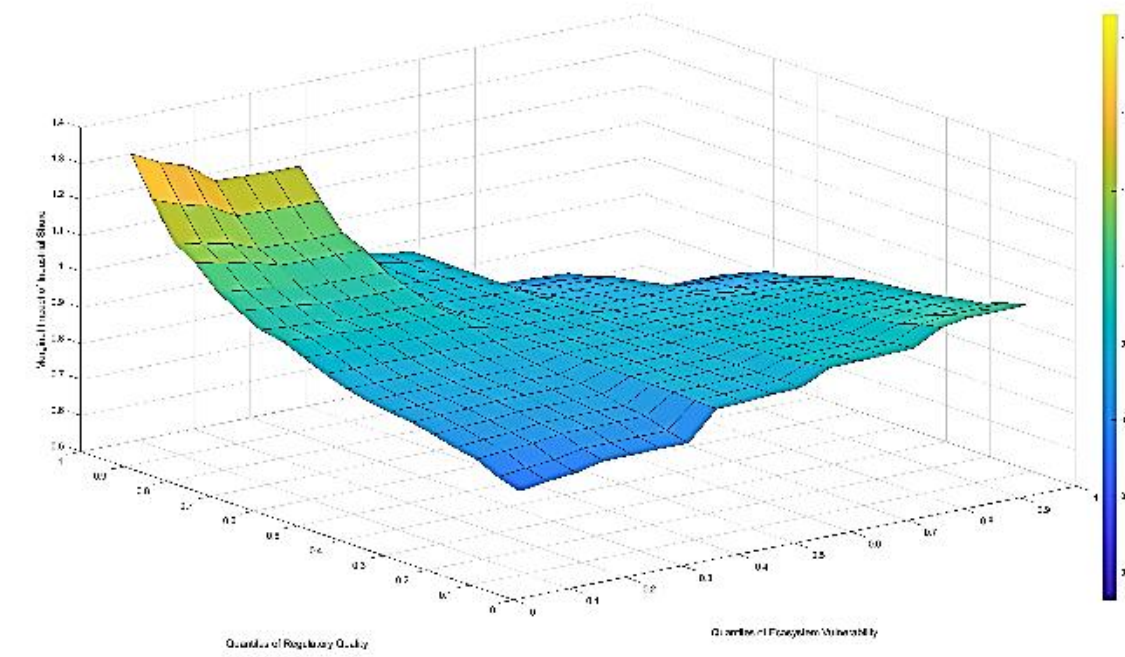

Governance Quality Indicator: Regulatory Quality

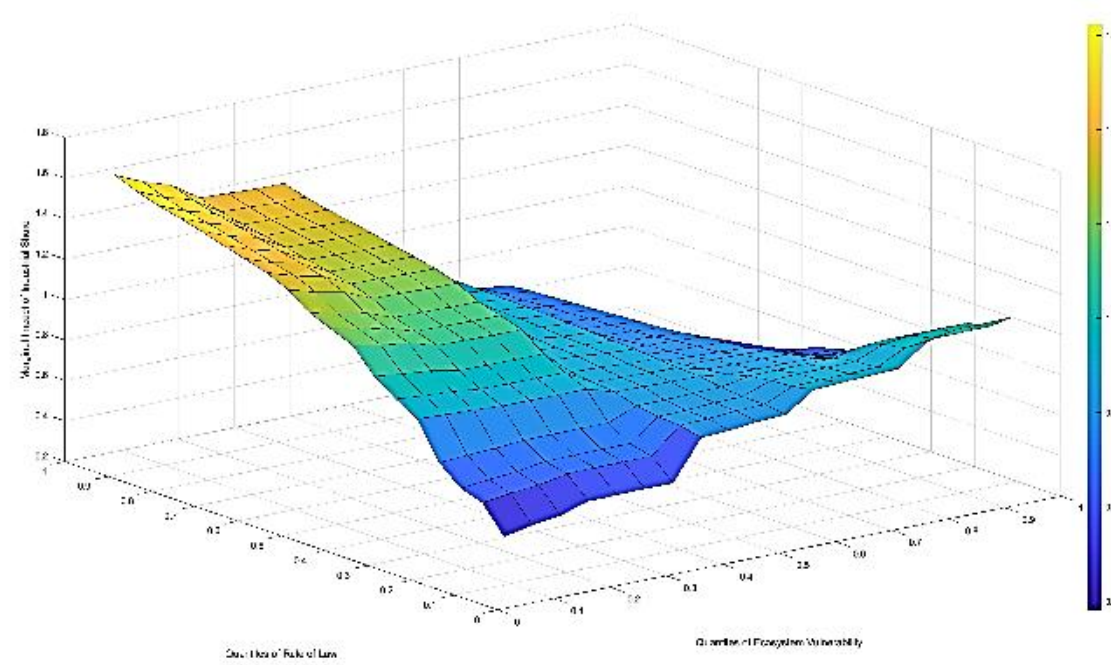

Governance Quality Indicator: Rule of Law

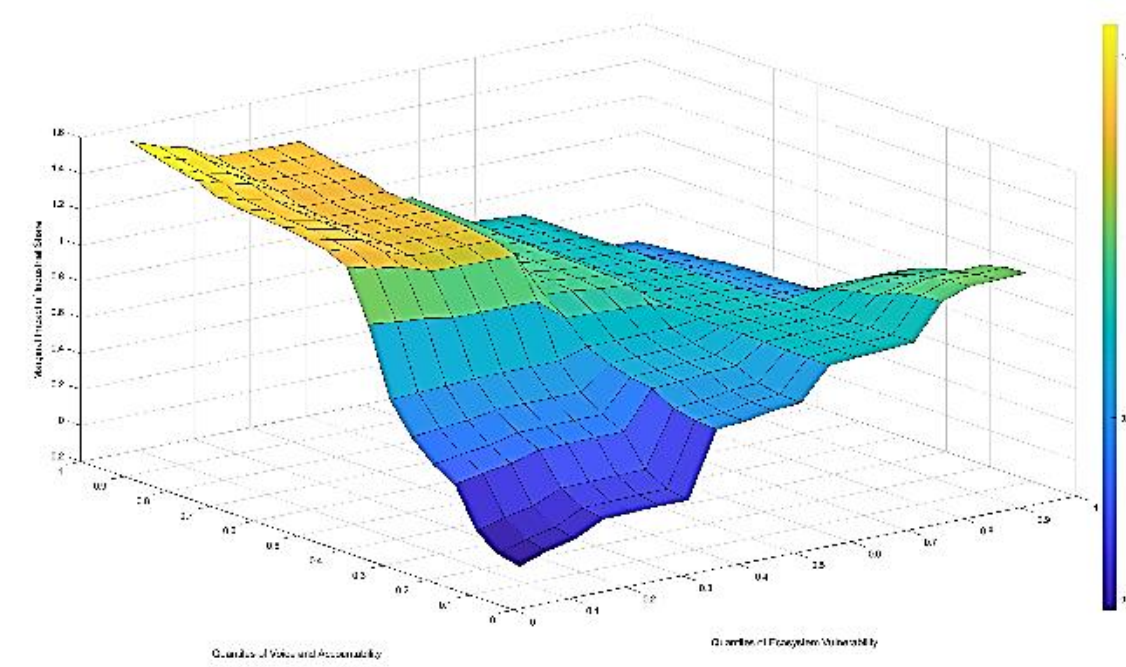

Governance Quality Indicator: Voice and Accountability

Figure S98: BRICS Policy Simulation Outcomes for the Marginal Impact of Industrial Share on SDG 7.3.1 under Ecosystem Vulnerability and Governance Quality, Related to Figure 4-5 and Table 2

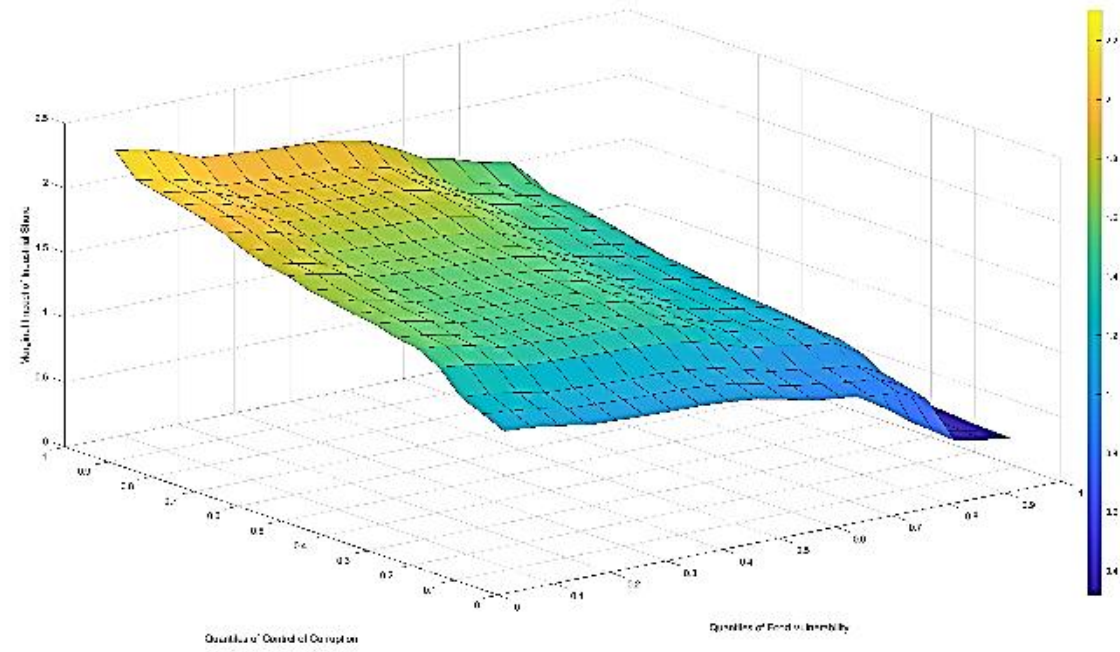

Governance Quality Indicator: Control of Corruption

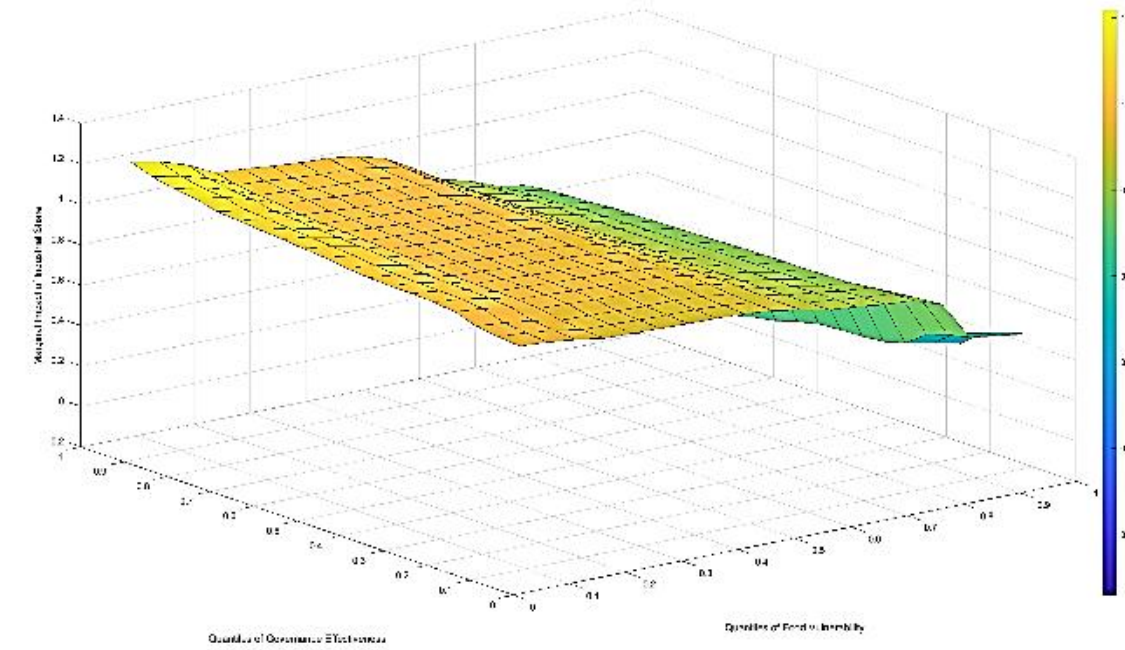

Governance Quality Indicator: Governance Effectiveness

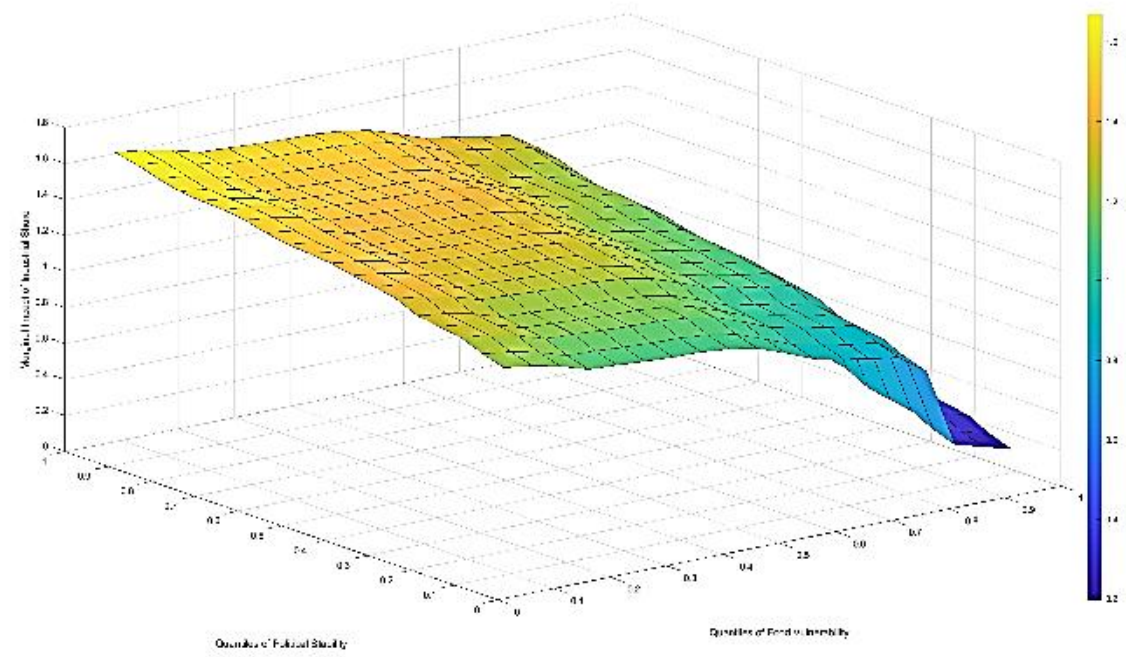

Governance Quality Indicator: Political Stability

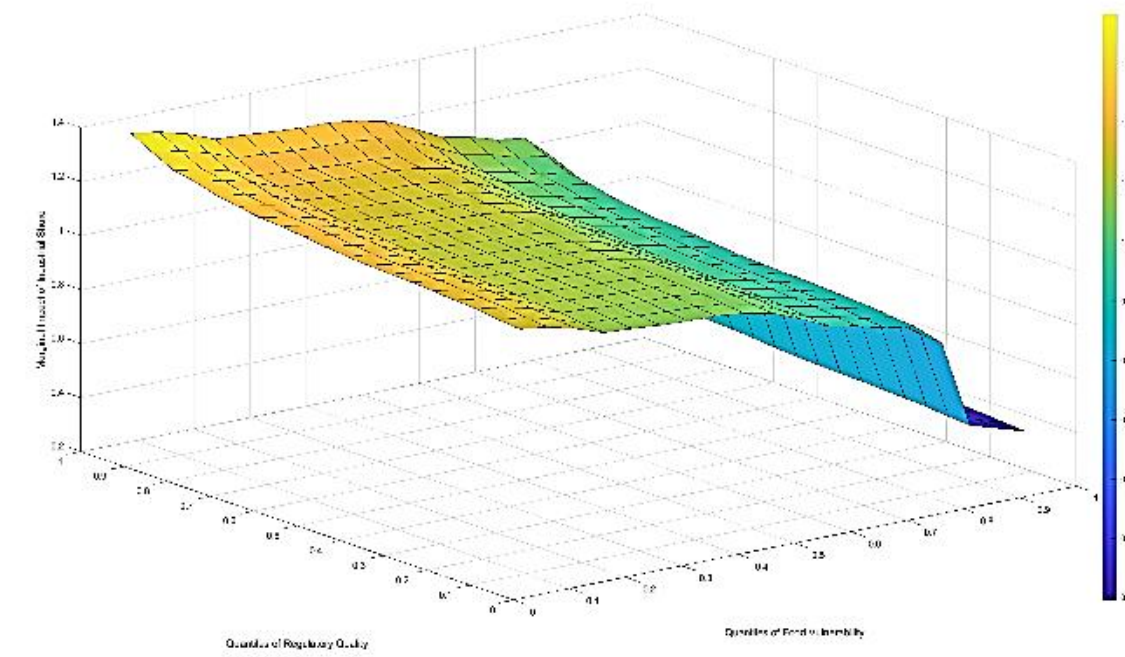

Governance Quality Indicator: Regulatory Quality

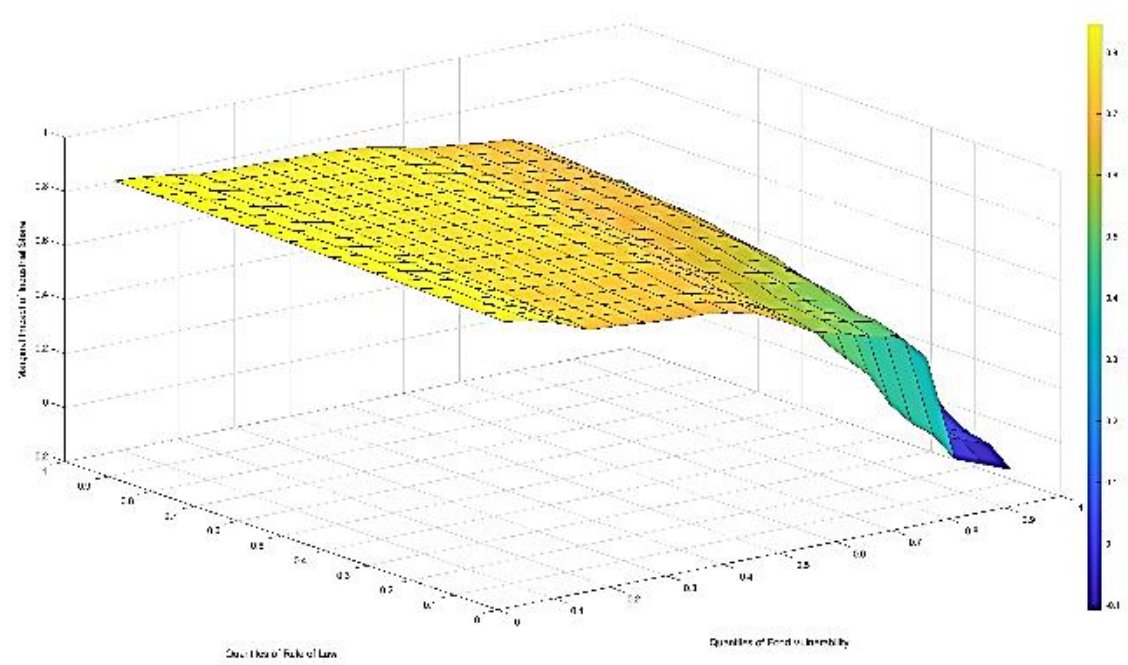

Governance Quality Indicator: Rule of Law

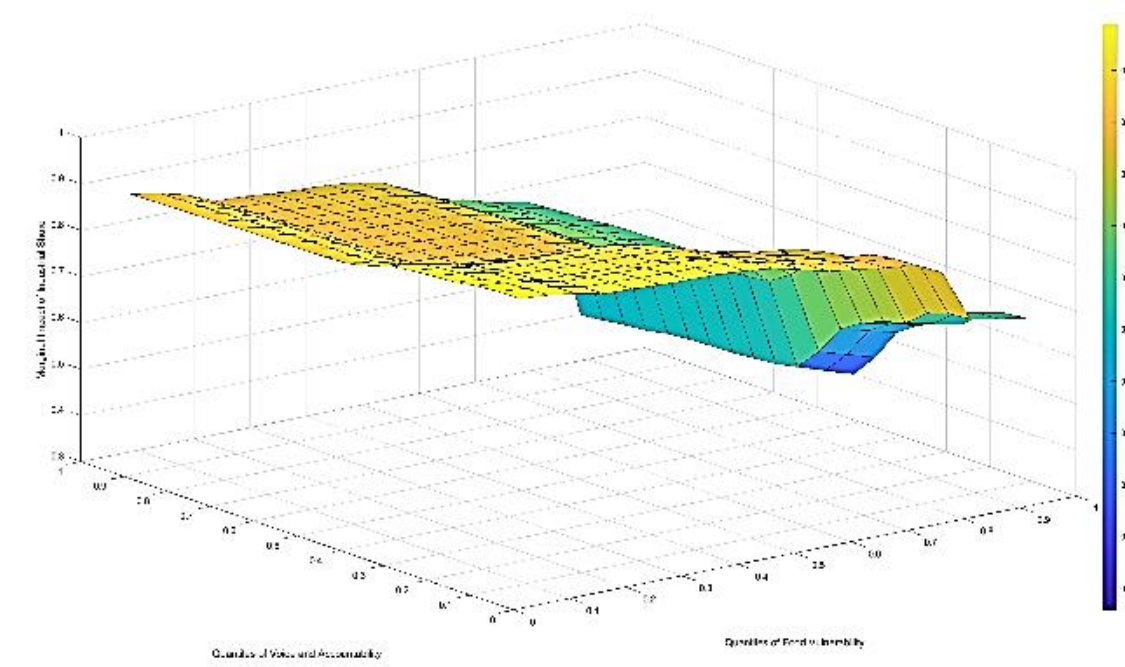

Governance Quality Indicator: Voice and Accountability

Figure S99: BRICS Policy Simulation Outcomes for the Marginal Impact of Industrial Share on SDG 7.3.1 under Food Vulnerability and Governance Quality, Related to Figure 4-5 and Table 2

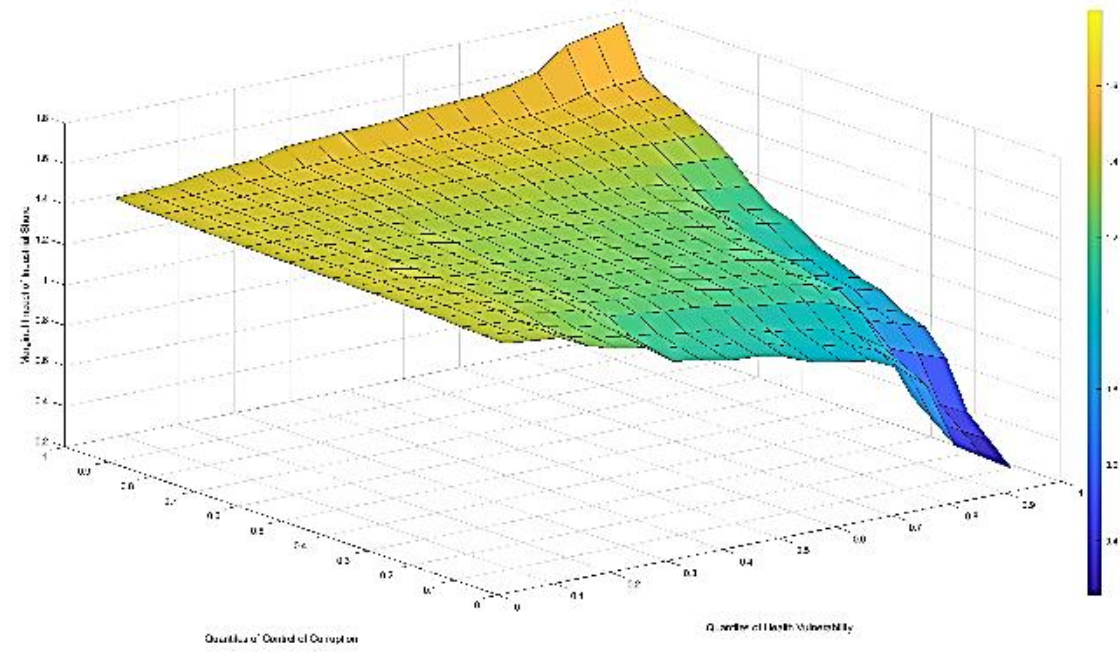

Governance Quality Indicator: Control of Corruption

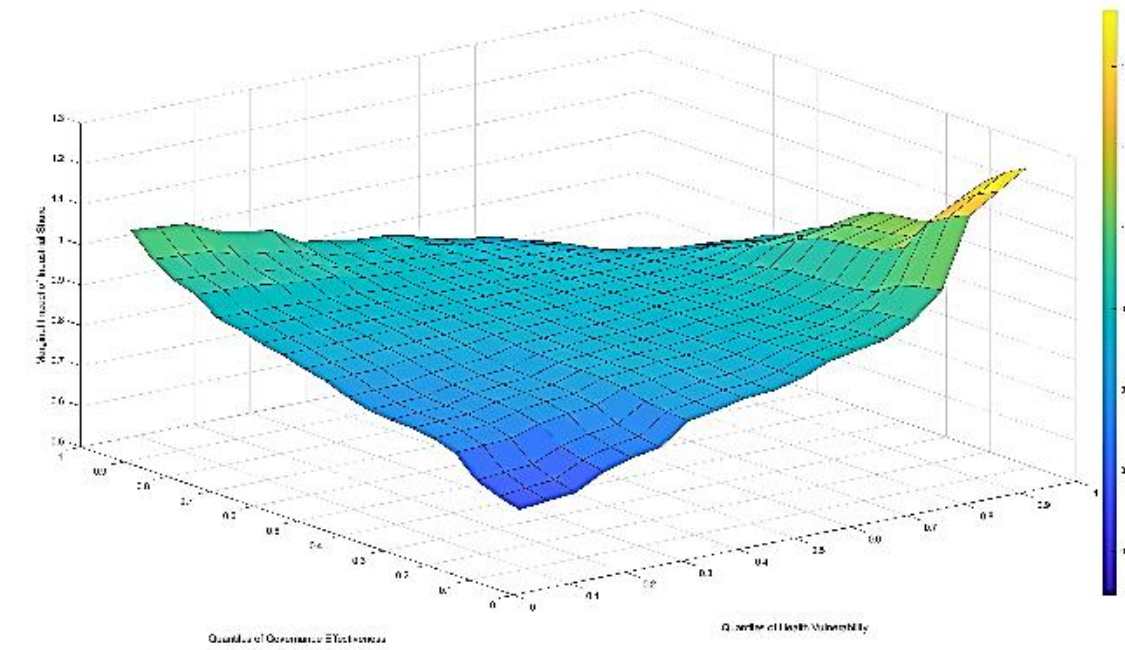

Governance Quality Indicator: Governance Effectiveness

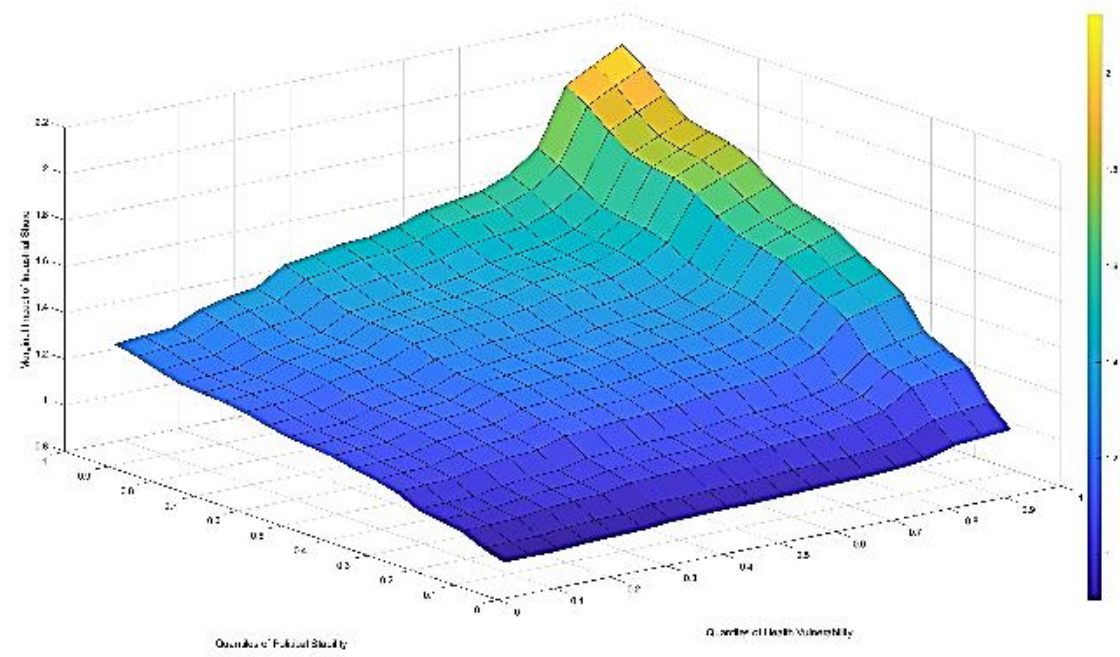

Governance Quality Indicator: Political Stability

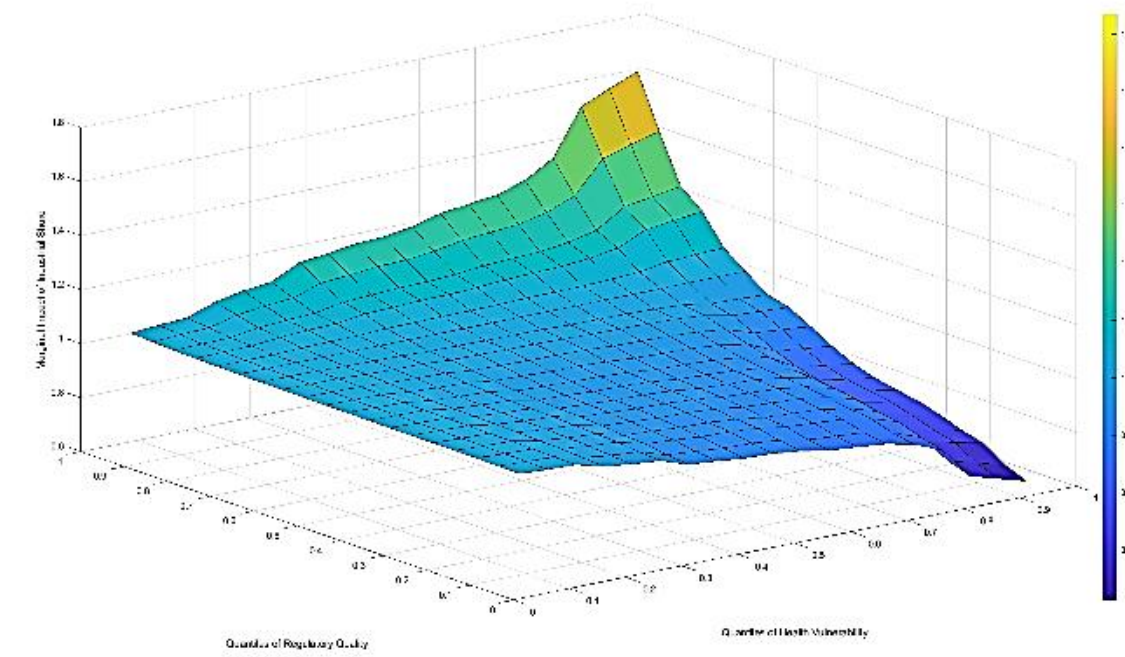

Governance Quality Indicator: Regulatory Quality

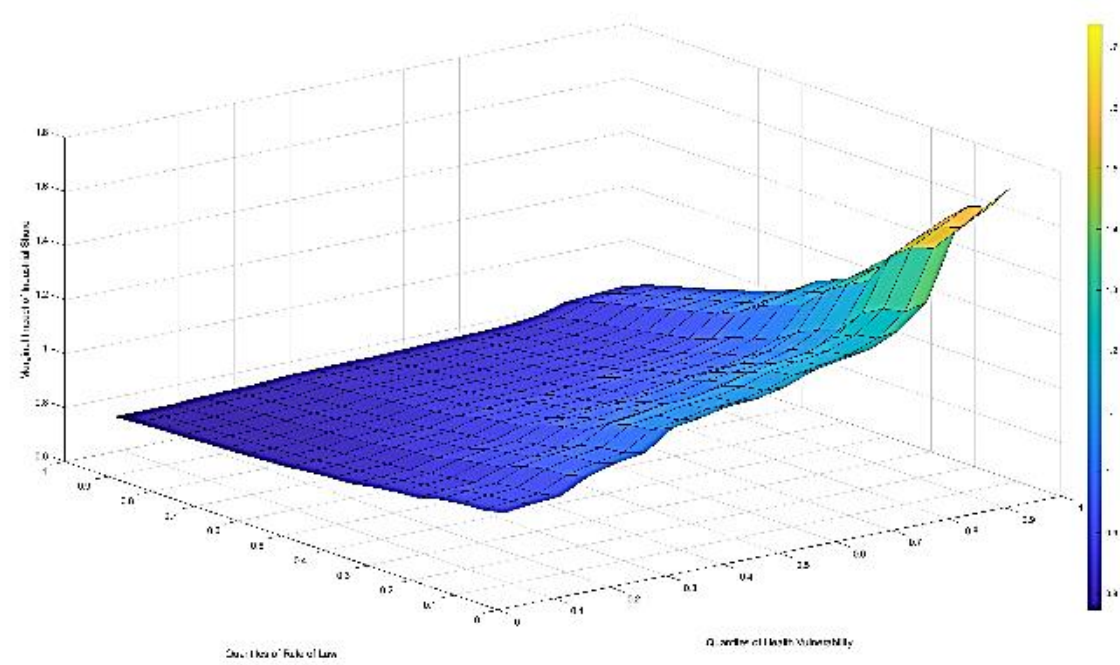

Governance Quality Indicator: Rule of Law

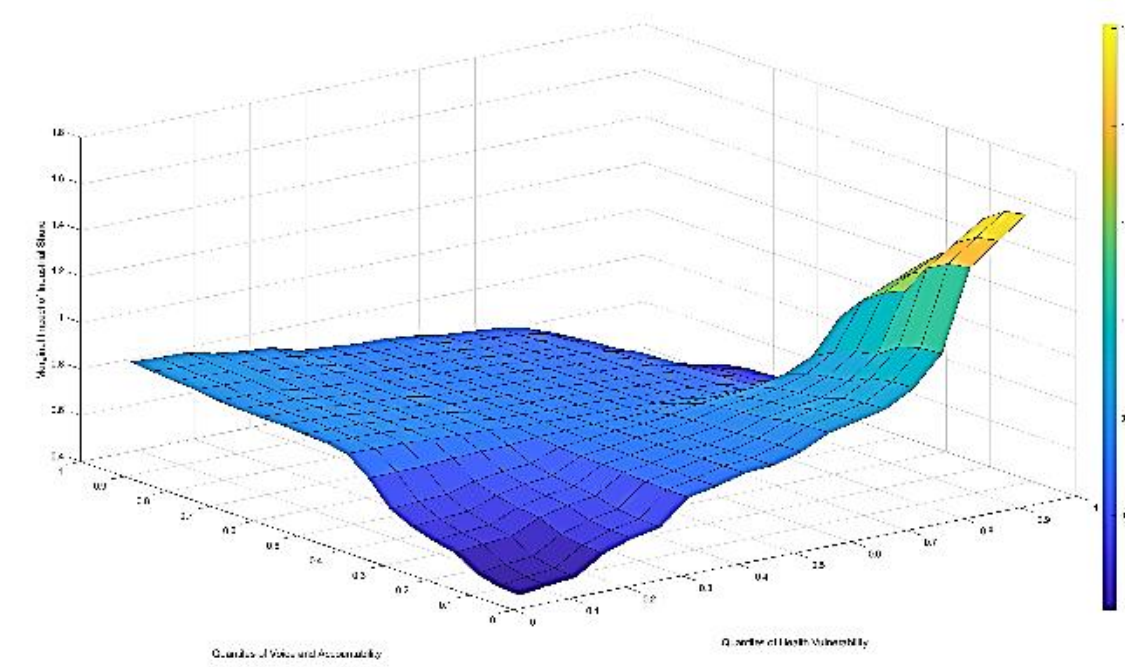

Governance Quality Indicator: Voice and Accountability

Figure S100: BRICS Policy Simulation Outcomes for the Marginal Impact of Industrial Share on SDG 7.3.1 under Health Vulnerability and Governance Quality, Related to Figure 4-5 and Table 2

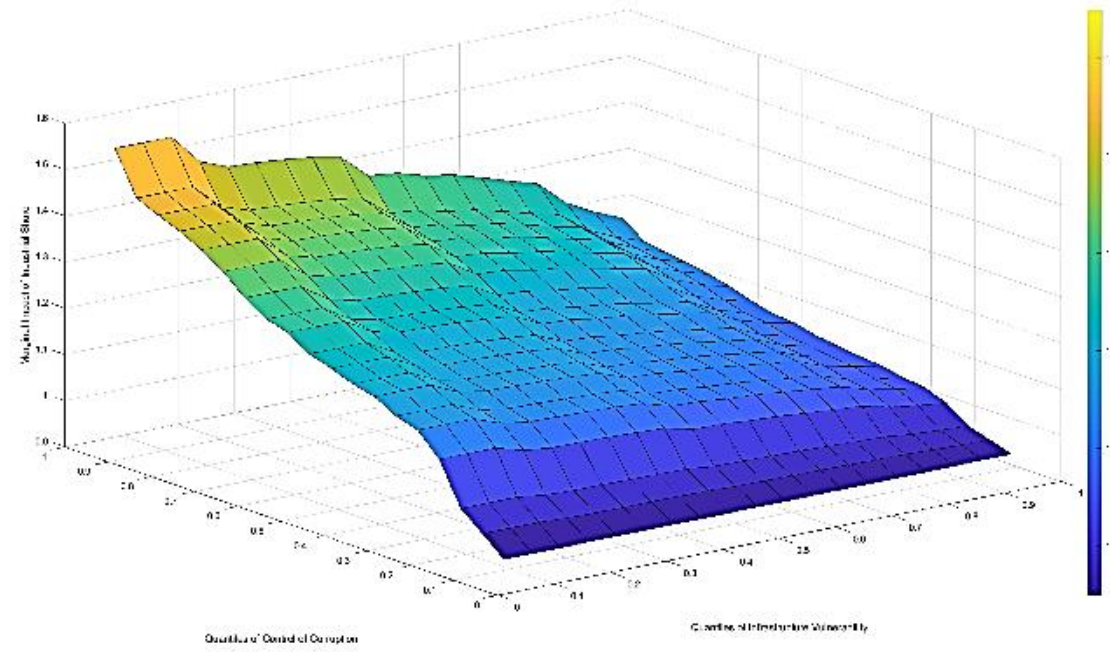

Governance Quality Indicator: Control of Corruption

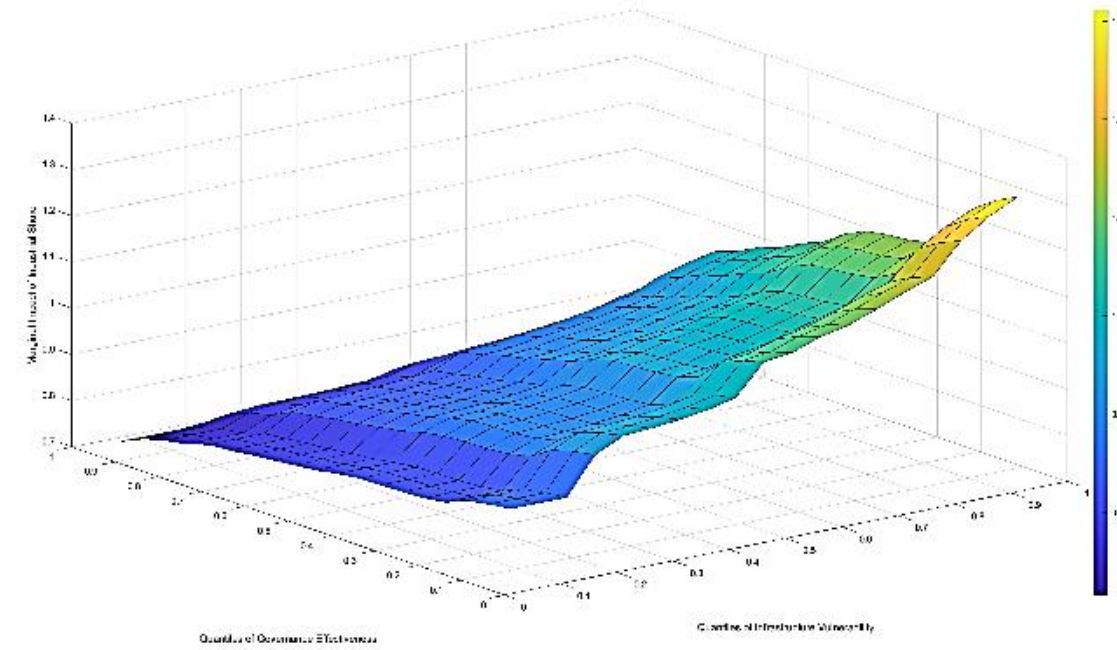

Governance Quality Indicator: Governance Effectiveness

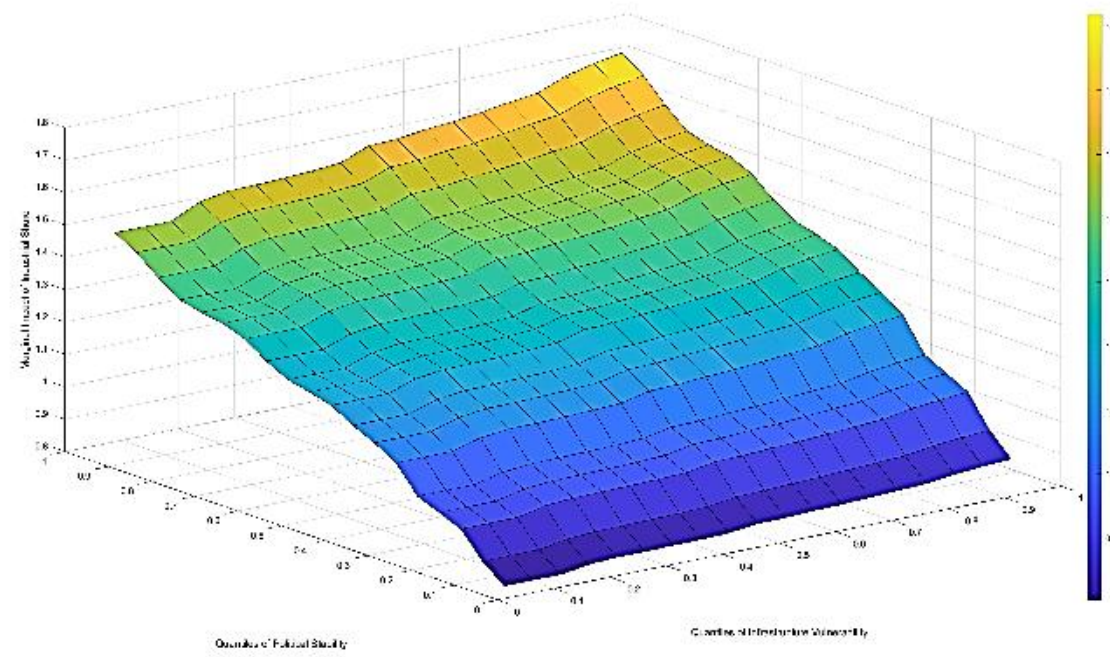

Governance Quality Indicator: Political Stability

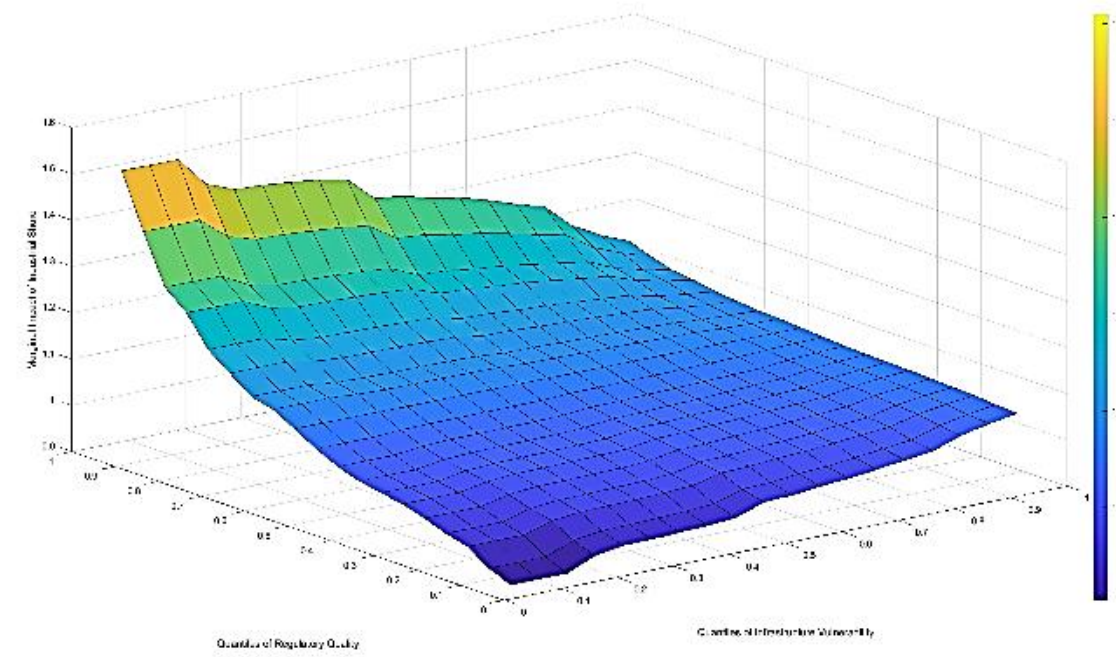

Governance Quality Indicator: Regulatory Quality

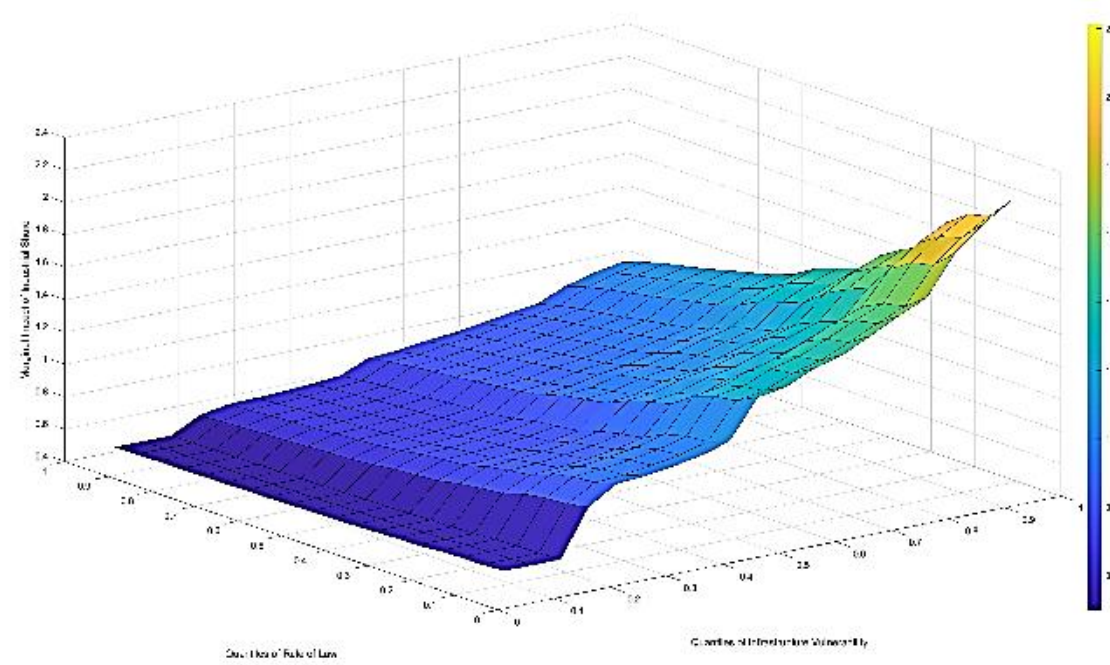

Governance Quality Indicator: Rule of Law

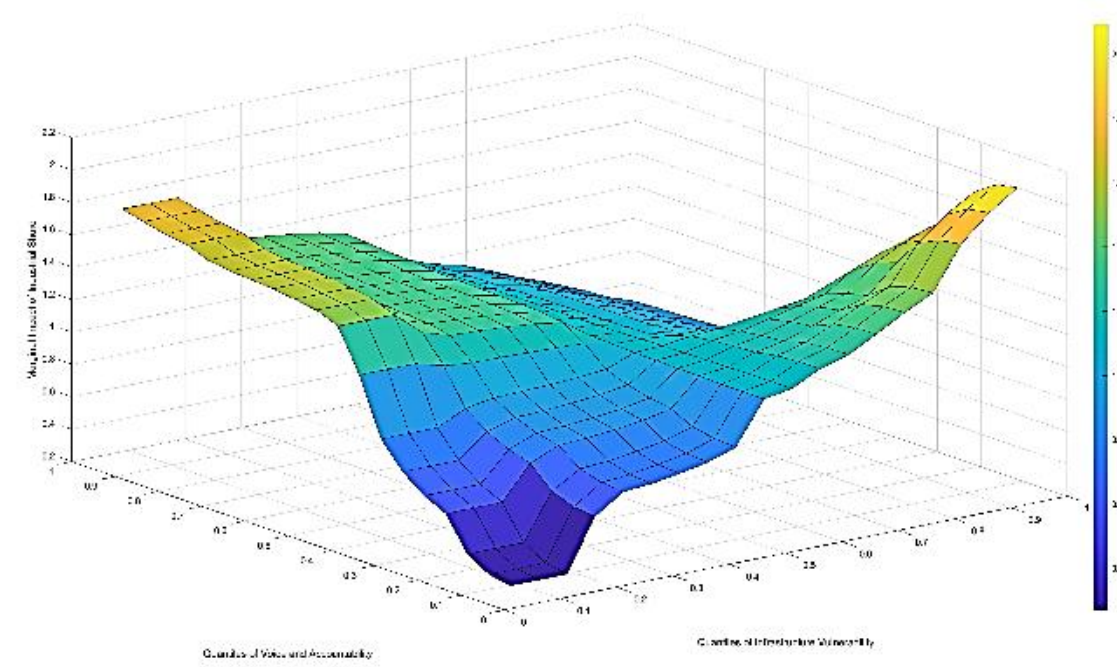

Governance Quality Indicator: Voice and Accountability

Figure S101: BRICS Policy Simulation Outcomes for the Marginal Impact of Industrial Share on SDG 7.3.1 under Infrastructure Vulnerability and Governance Quality, Related to Figure 4-5 and Table 2

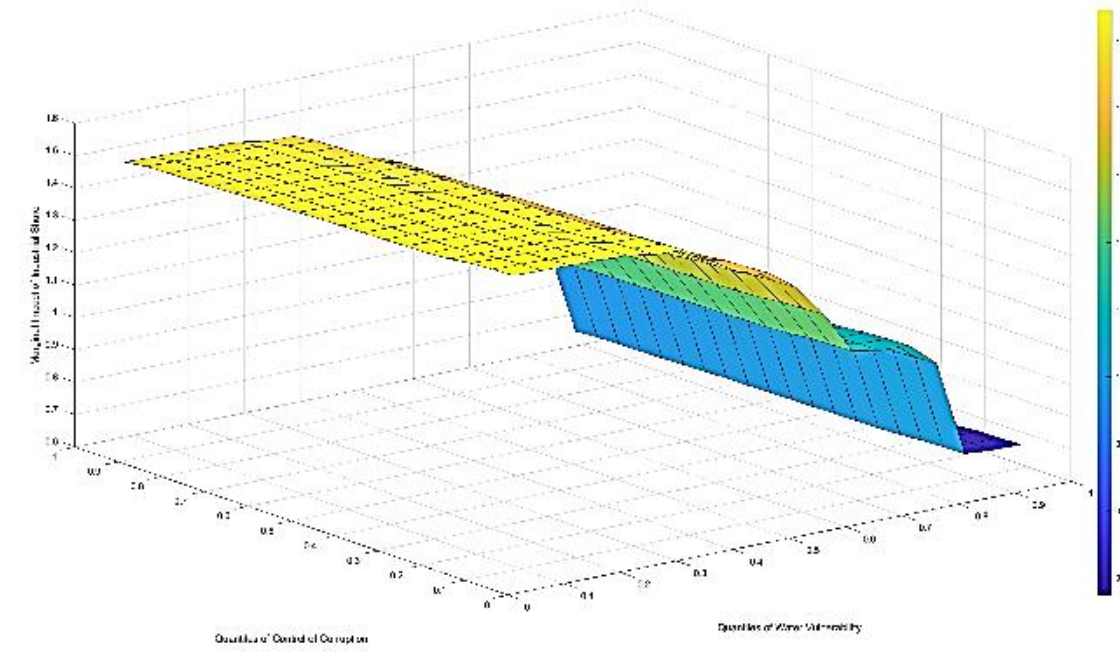

Governance Quality Indicator: Control of Corruption

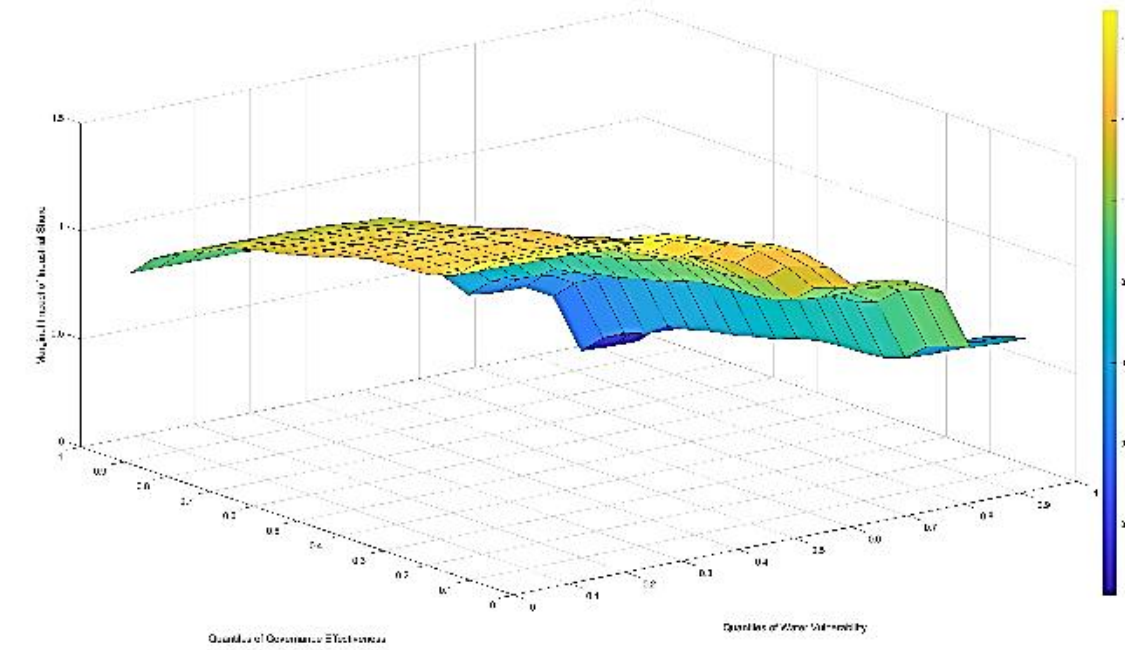

Governance Quality Indicator: Governance Effectiveness

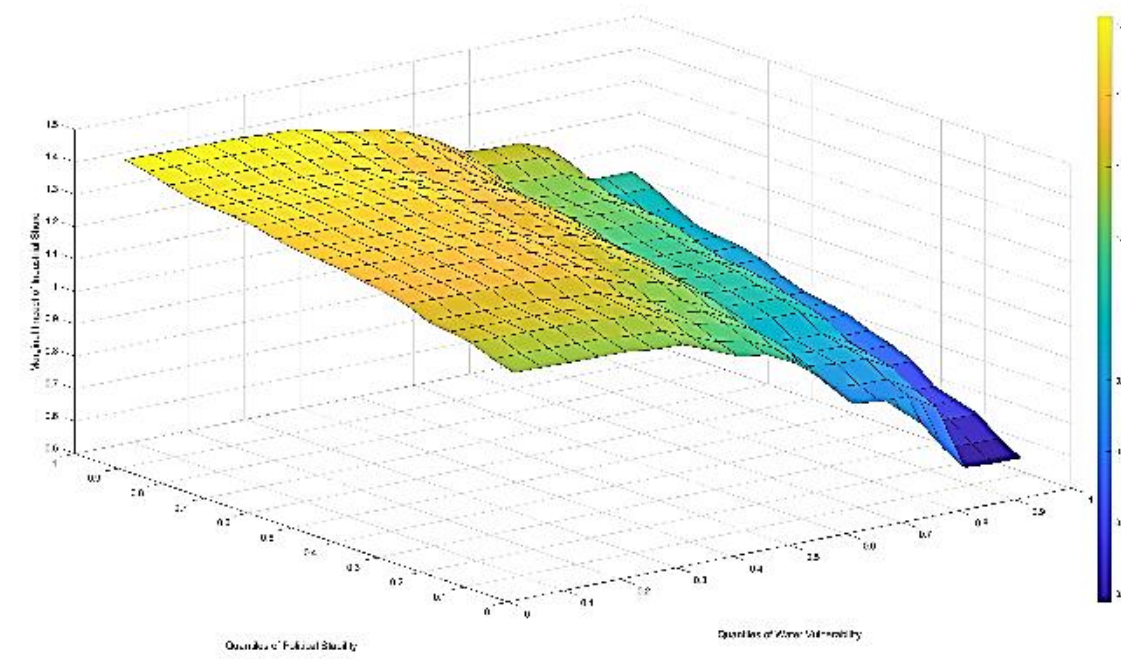

Governance Quality Indicator: Political Stability

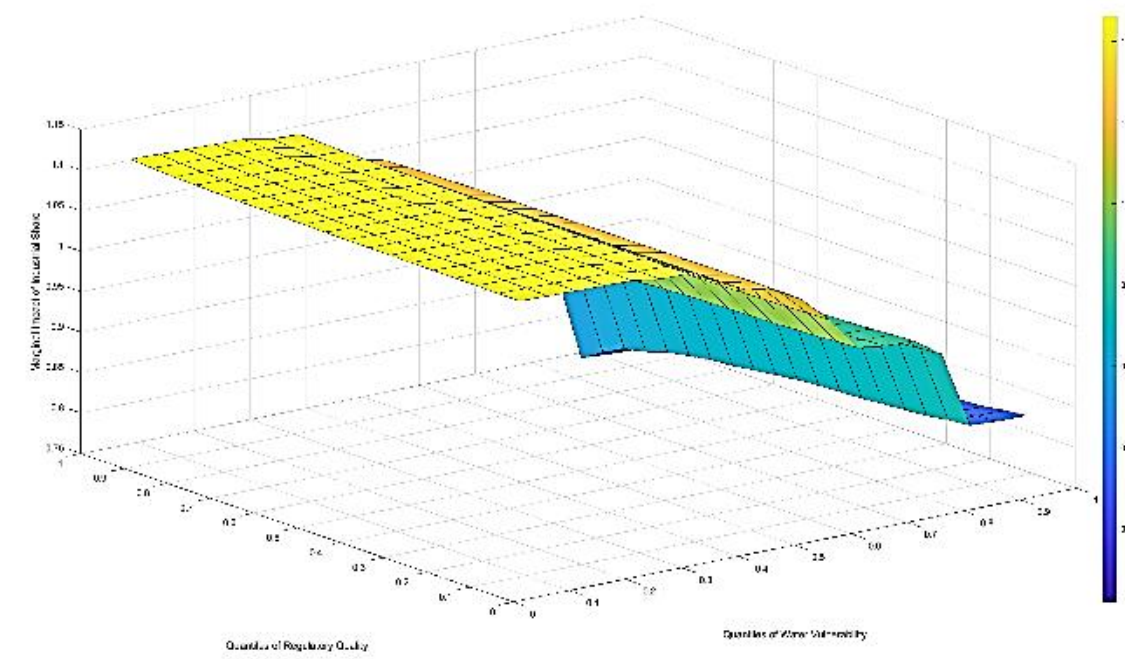

Governance Quality Indicator: Regulatory Quality

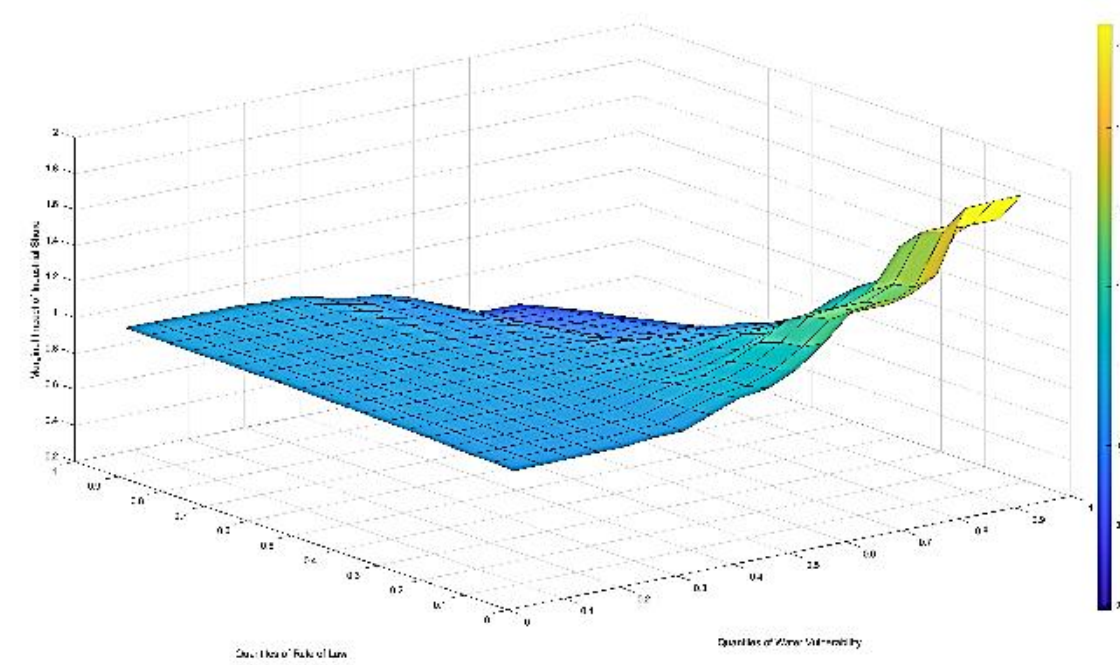

Governance Quality Indicator: Rule of Law

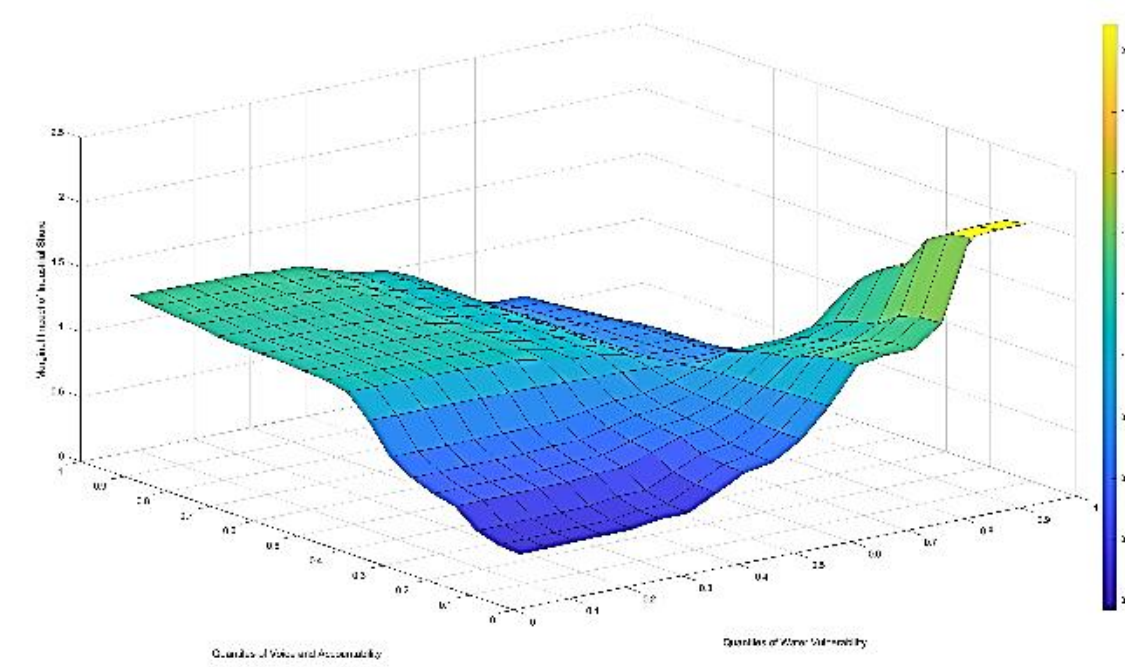

Governance Quality Indicator: Voice and Accountability

Figure S102: BRICS Policy Simulation Outcomes for the Marginal Impact of Industrial Share on SDG 7.3.1 under Water Vulnerability and Governance Quality, Related to Figure 4-5 and Table 2

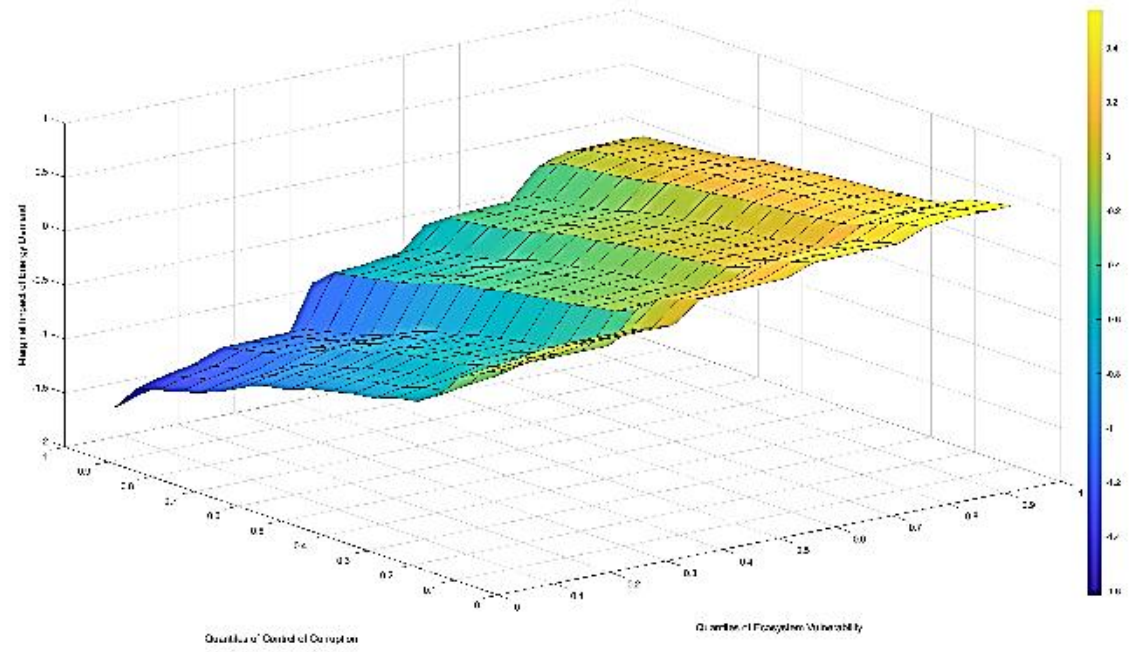

Governance Quality Indicator: Control of Corruption

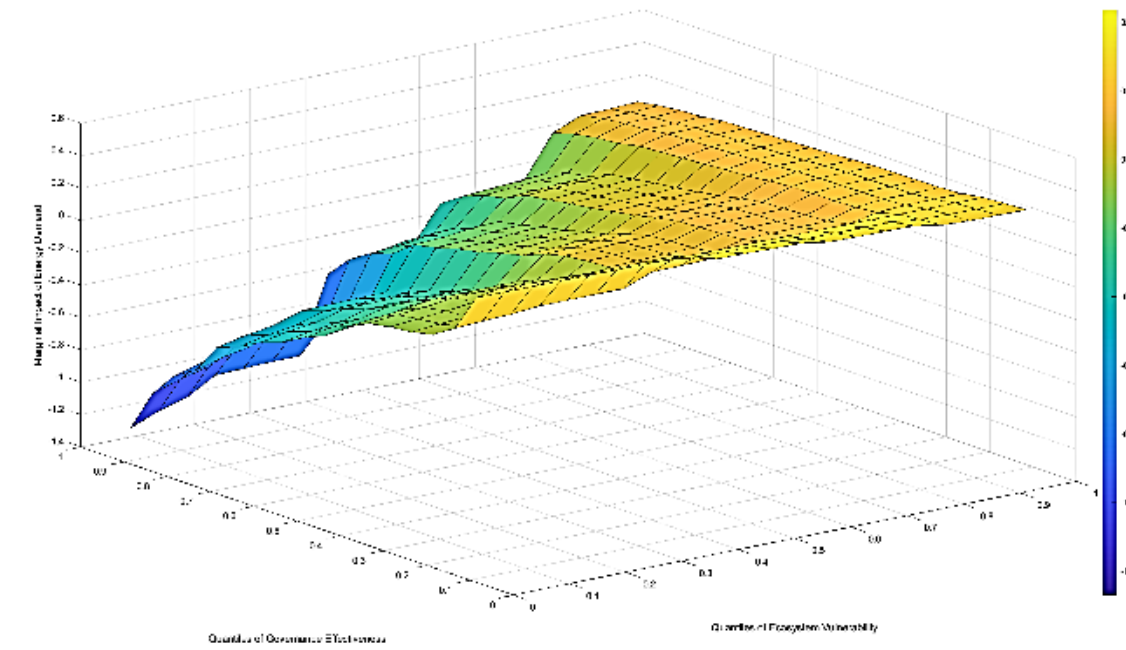

Governance Quality Indicator: Governance Effectiveness

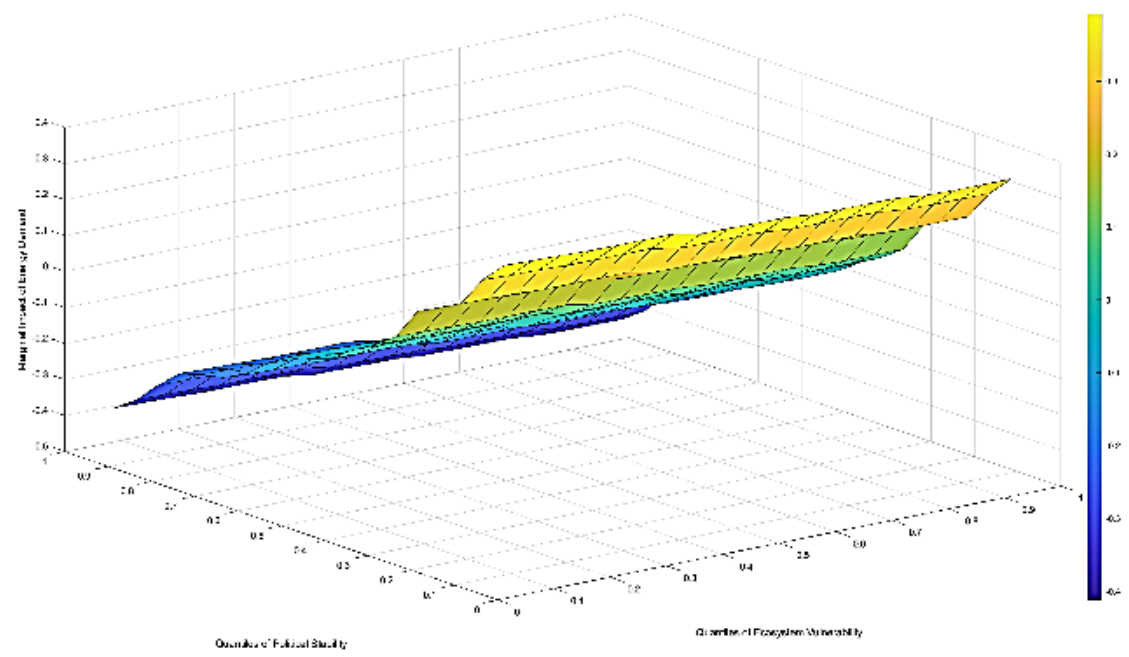

Governance Quality Indicator: Political Stability

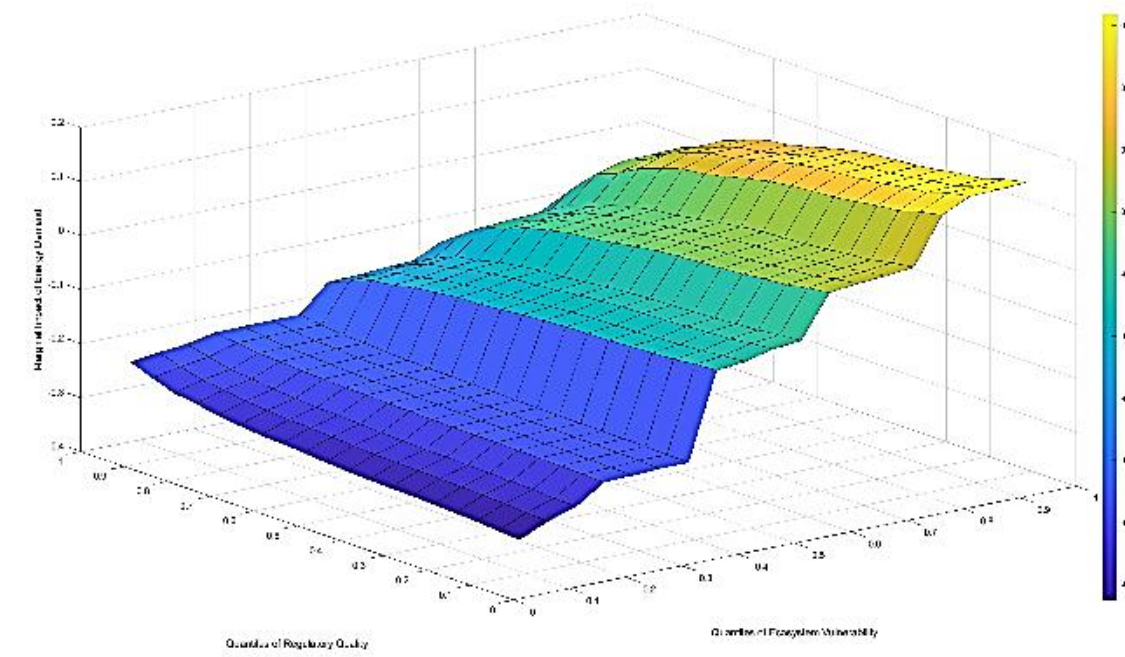

Governance Quality Indicator: Regulatory Quality

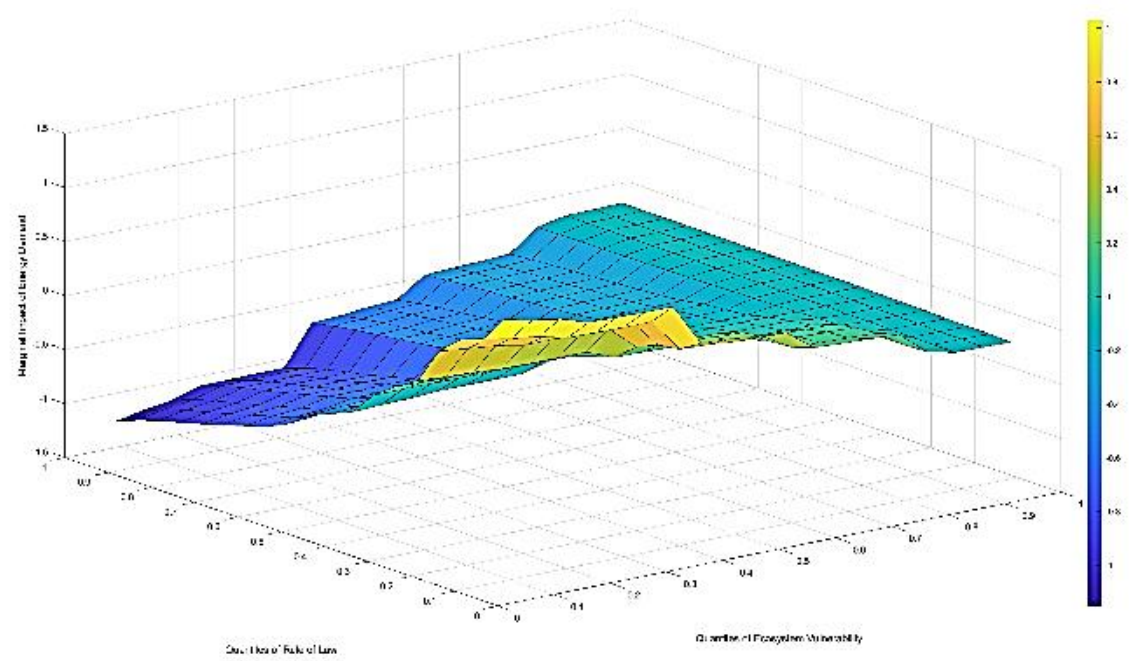

Governance Quality Indicator: Rule of Law

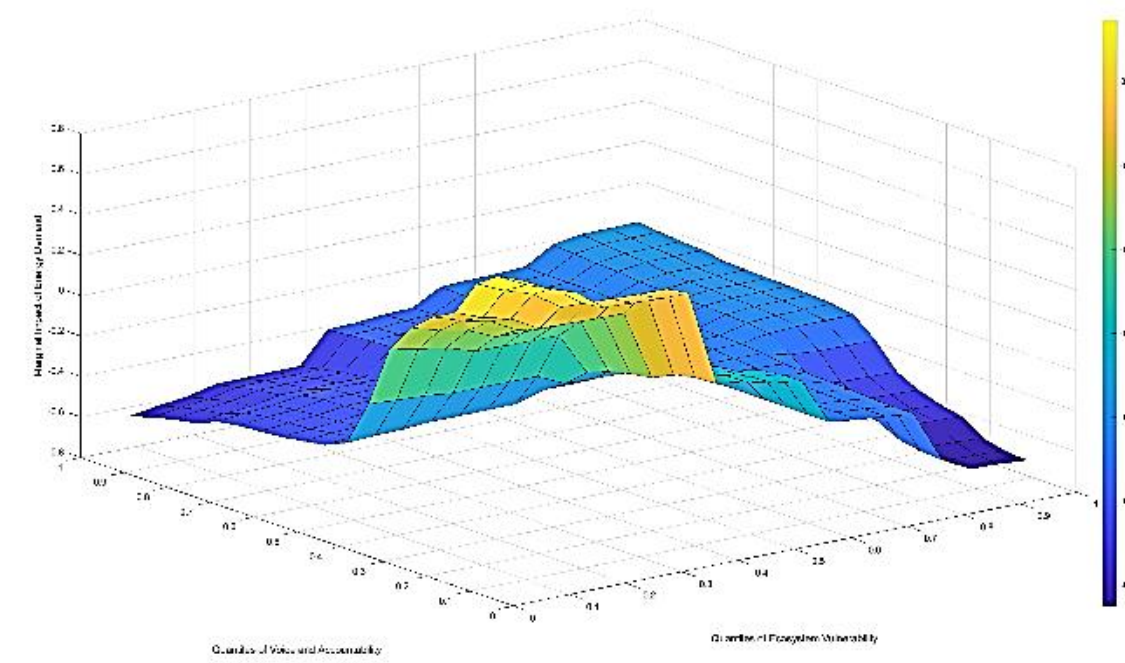

Governance Quality Indicator: Voice and Accountability

Figure S103: BRICS Policy Simulation Outcomes for the Marginal Impact of Energy Demand on SDG 7.3.1 under Ecosystem Vulnerability and Governance Quality, Related to Figure 4-5 and Table 2

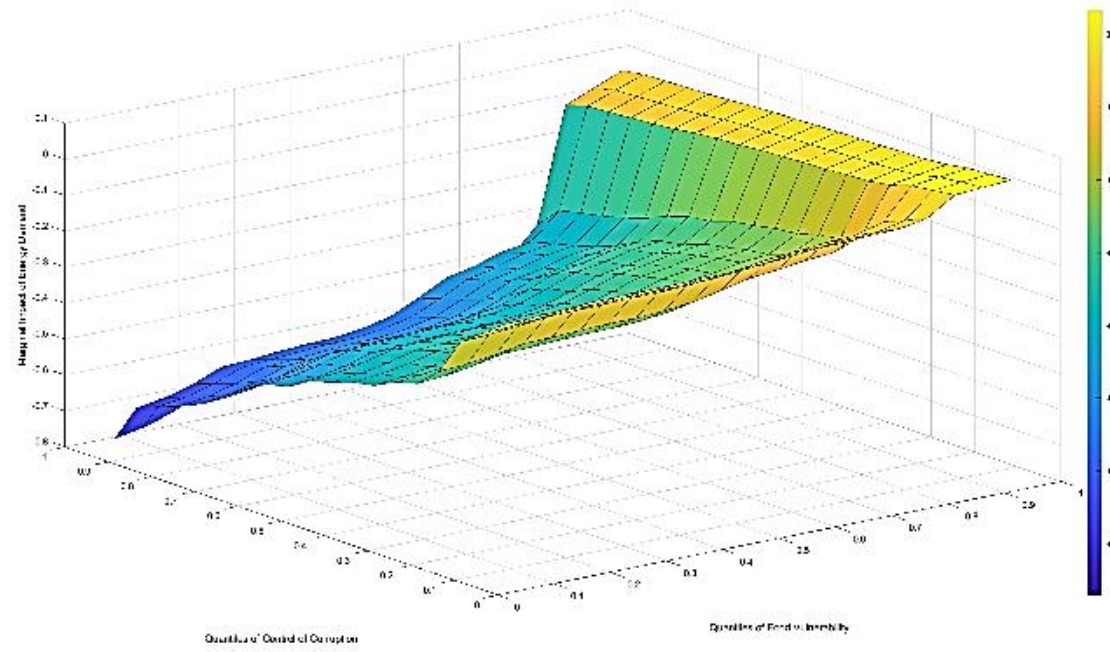

Governance Quality Indicator: Control of Corruption

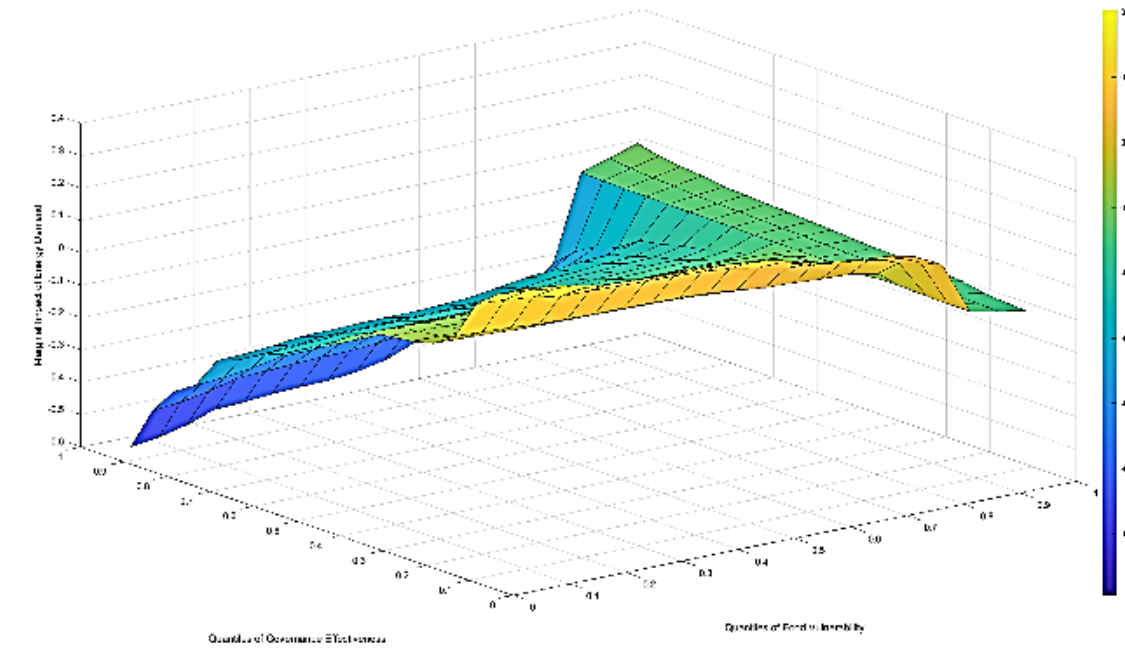

Governance Quality Indicator: Governance Effectiveness

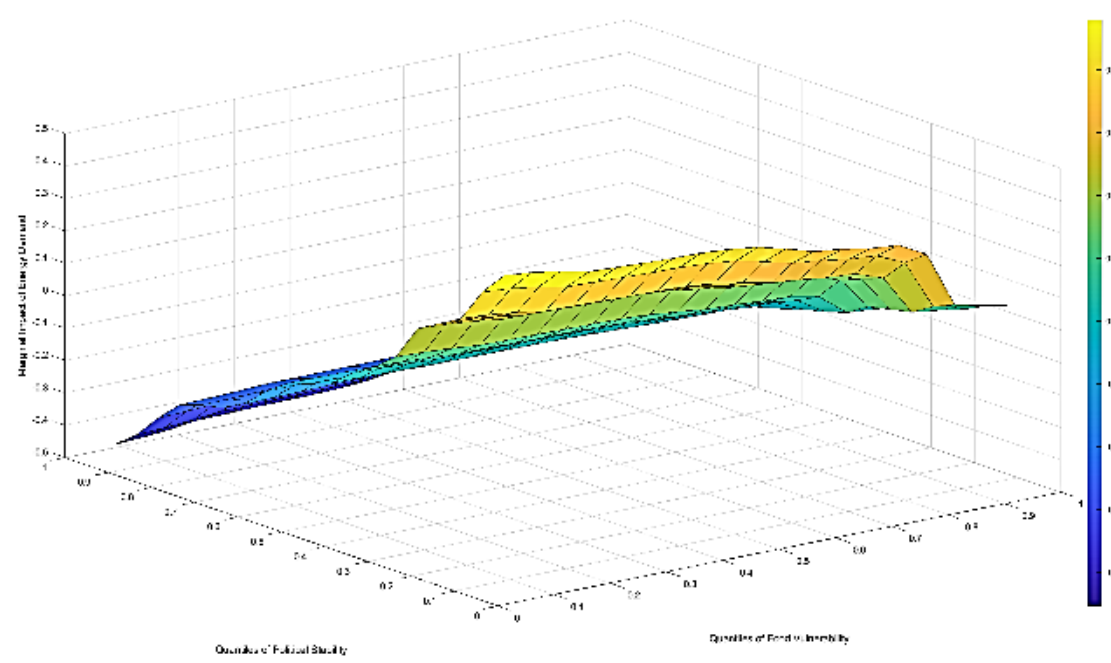

Governance Quality Indicator: Political Stability

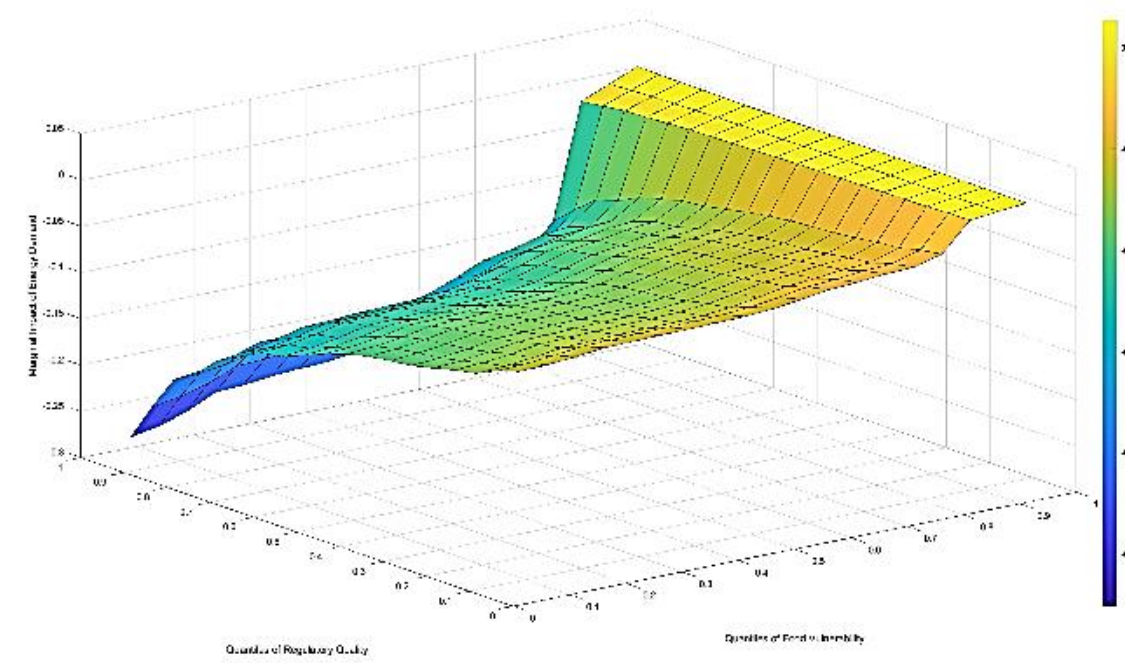

Governance Quality Indicator: Regulatory Quality

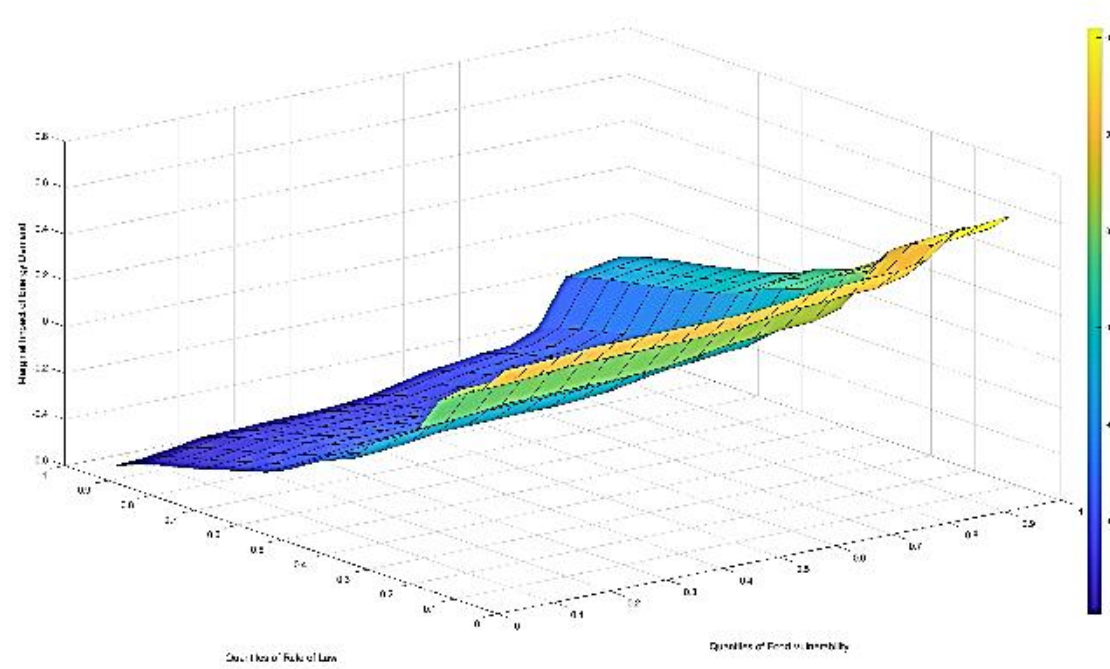

Governance Quality Indicator: Rule of Law

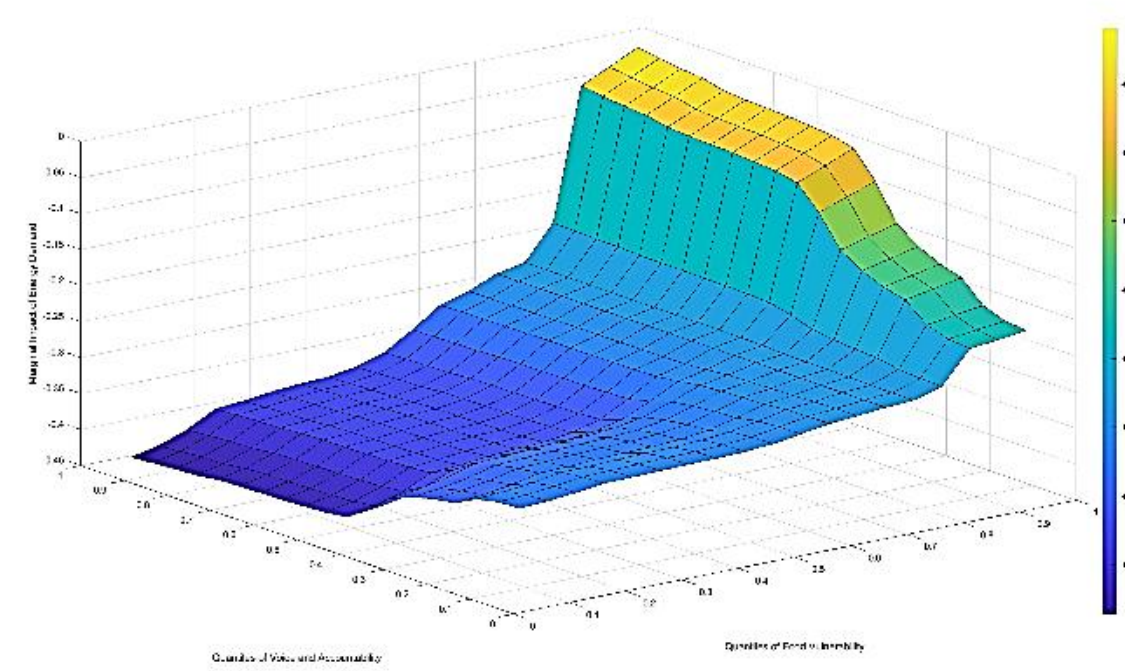

Governance Quality Indicator: Voice and Accountability

Figure S104: BRICS Policy Simulation Outcomes for the Marginal Impact of Energy Demand on SDG 7.3.1 under Food Vulnerability and Governance Quality, Related to Figure 4-5 and Table 2

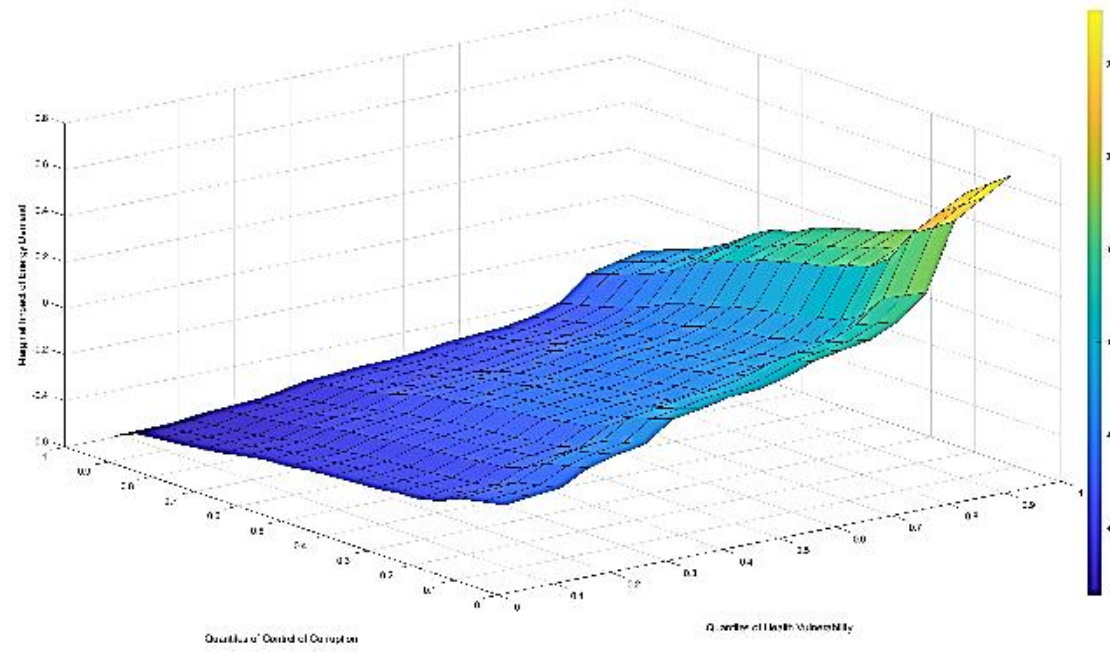

Governance Quality Indicator: Control of Corruption

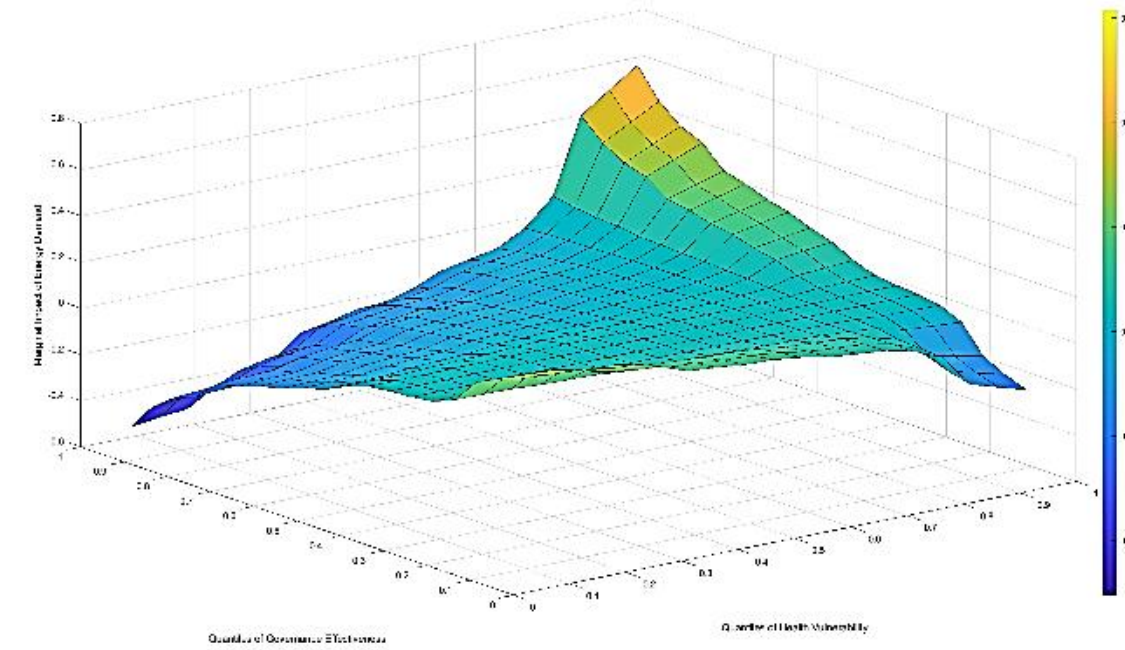

Governance Quality Indicator: Governance Effectiveness

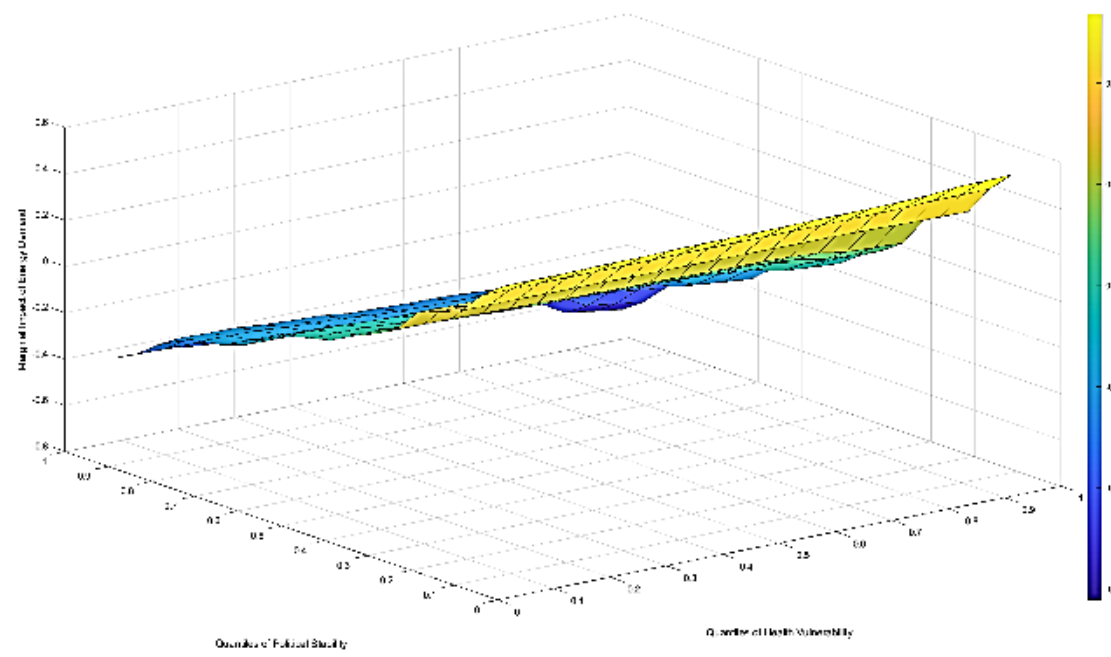

Governance Quality Indicator: Political Stability

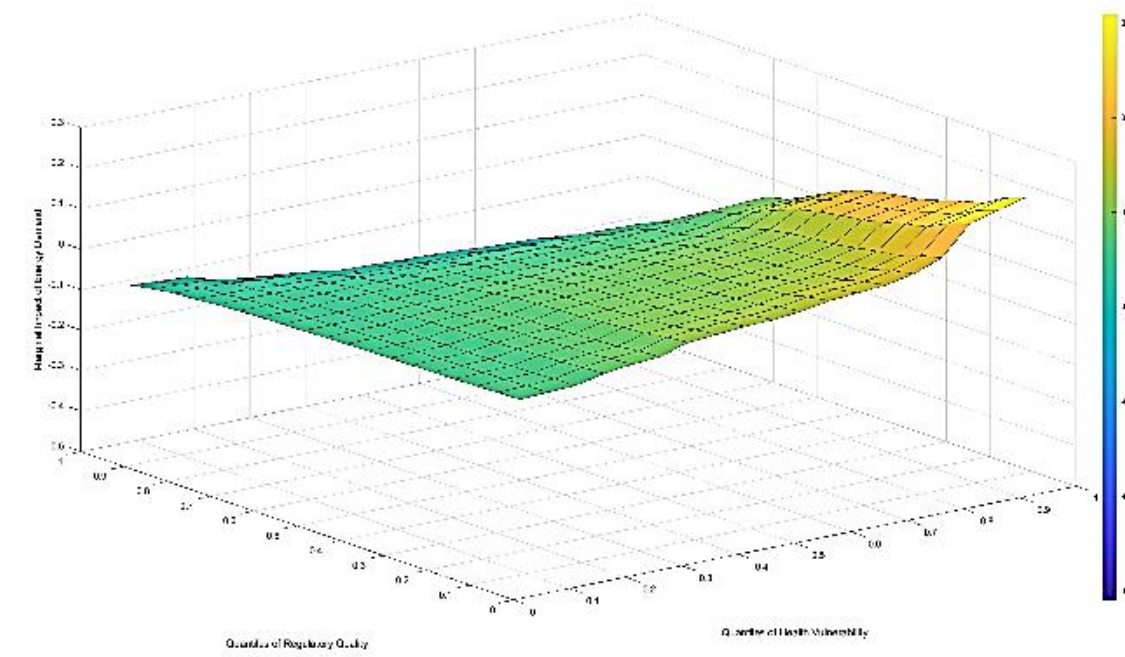

Governance Quality Indicator: Regulatory Quality

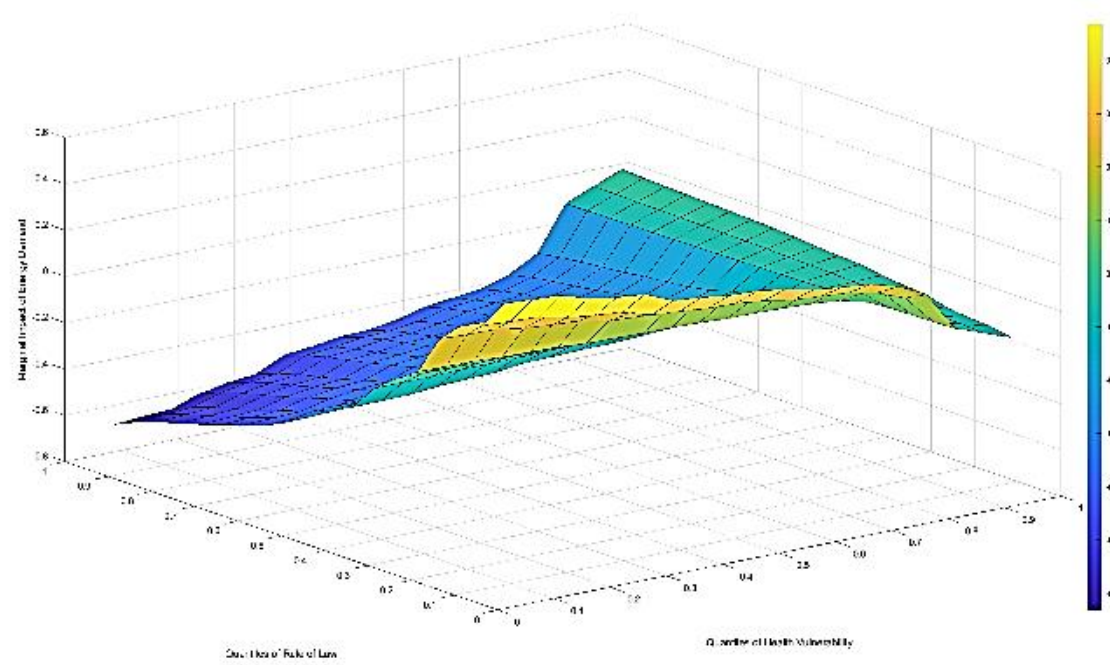

Governance Quality Indicator: Rule of Law

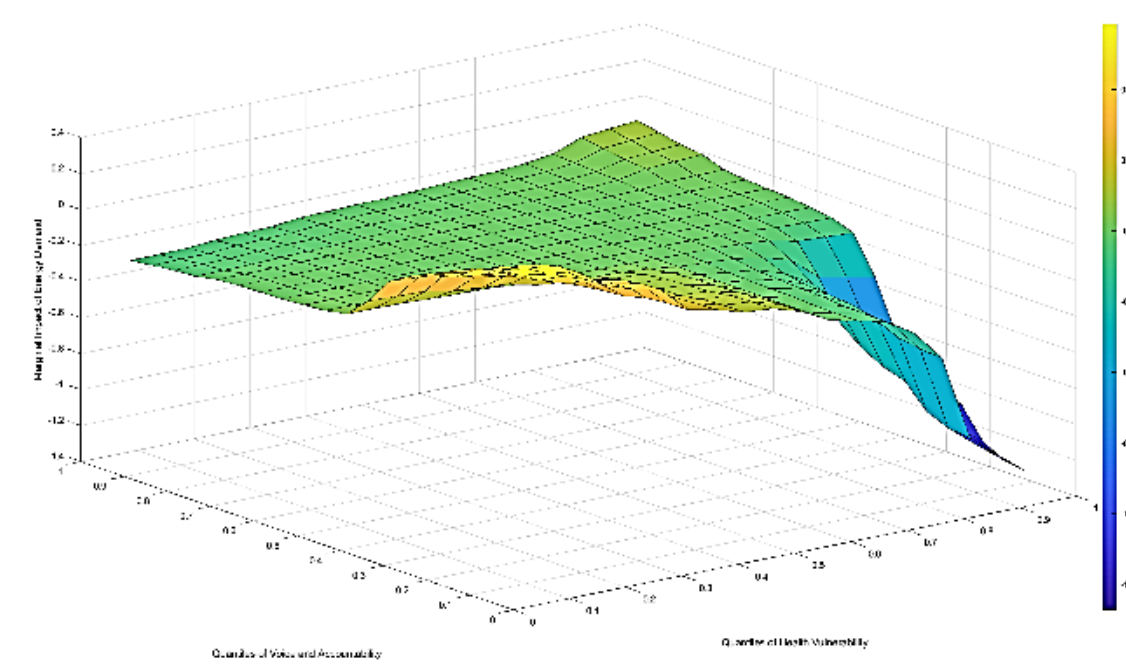

Governance Quality Indicator: Voice and Accountability

Figure S105: BRICS Policy Simulation Outcomes for the Marginal Impact of Energy Demand on SDG 7.3.1 under Health Vulnerability and Governance Quality, Related to Figure 4-5 and Table 2

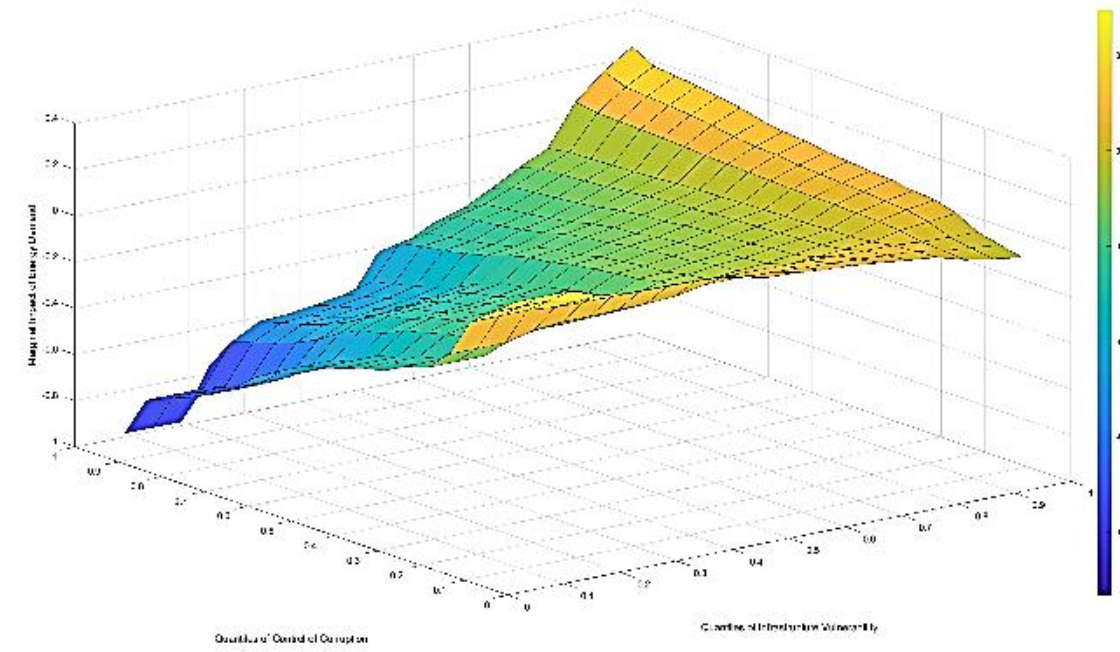

Governance Quality Indicator: Control of Corruption

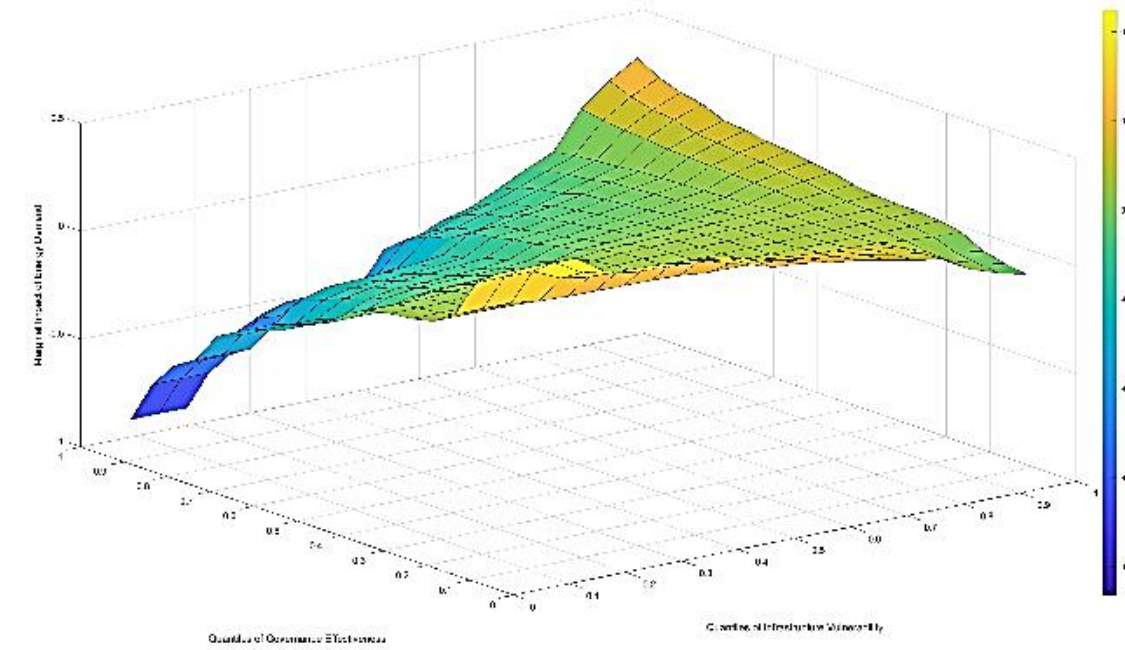

Governance Quality Indicator: Governance Effectiveness

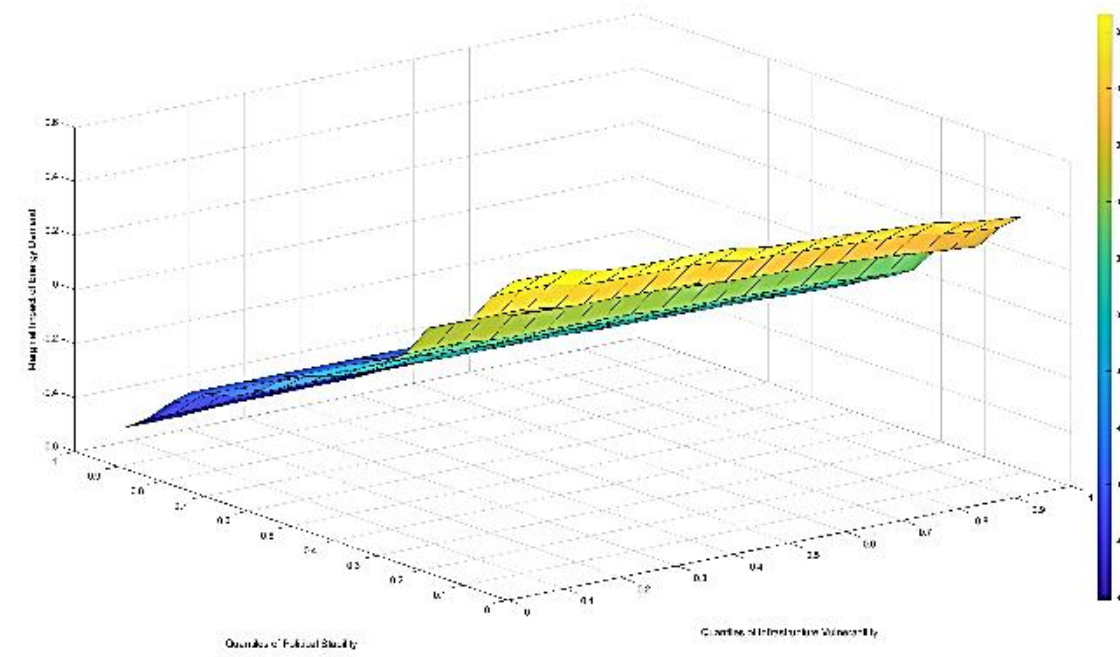

Governance Quality Indicator: Political Stability

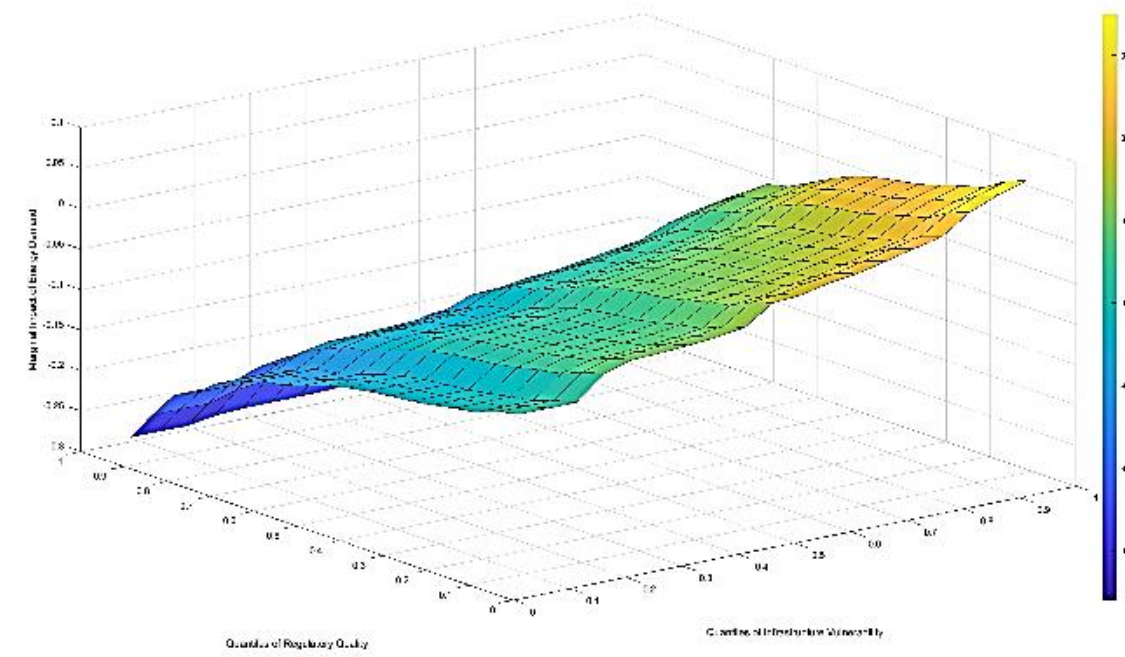

Governance Quality Indicator: Regulatory Quality

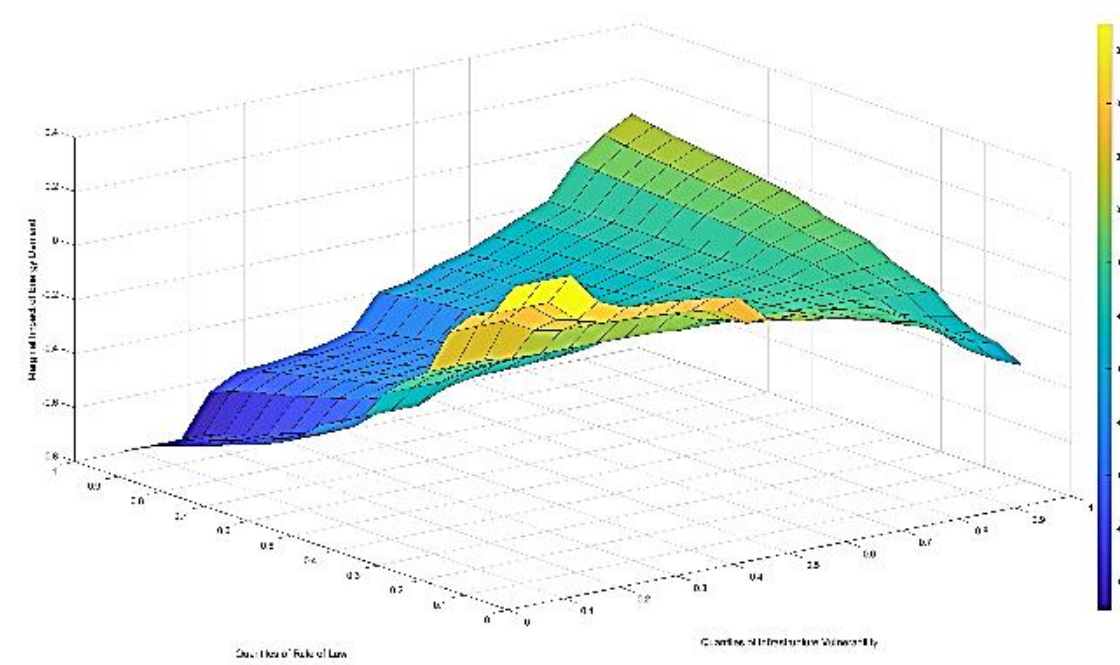

Governance Quality Indicator: Rule of Law

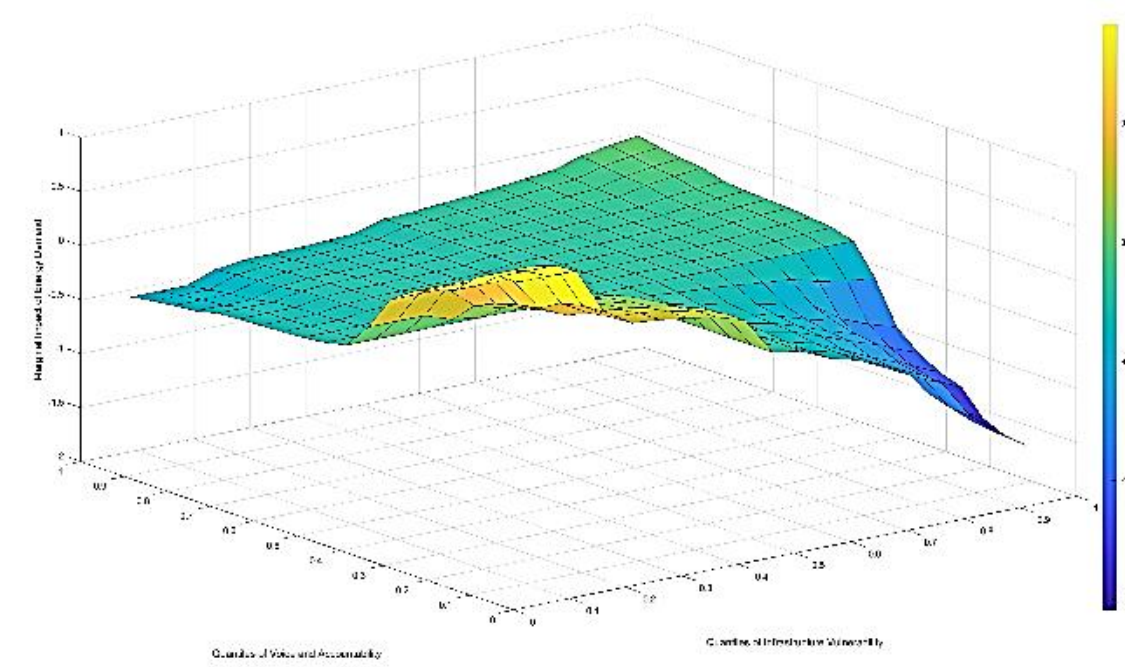

Governance Quality Indicator: Voice and Accountability

Figure S106: BRICS Policy Simulation Outcomes for the Marginal Impact of Energy Demand on SDG 7.3.1 under Infrastructure Vulnerability and Governance Quality, Related to Figure 4-5 and Table 2

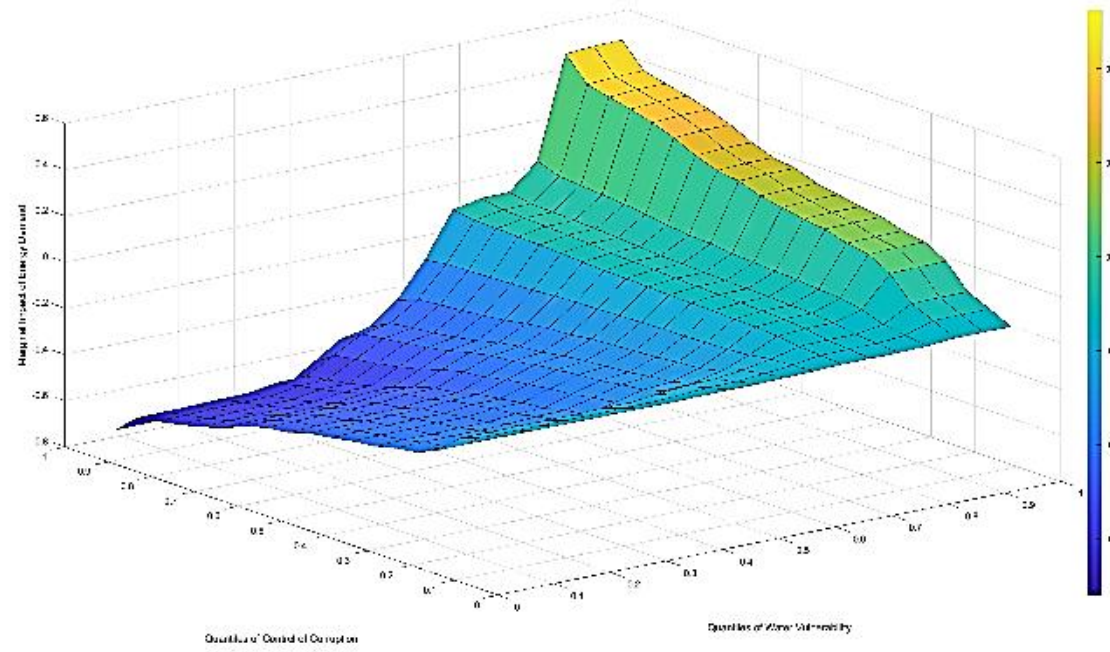

Governance Quality Indicator: Control of Corruption

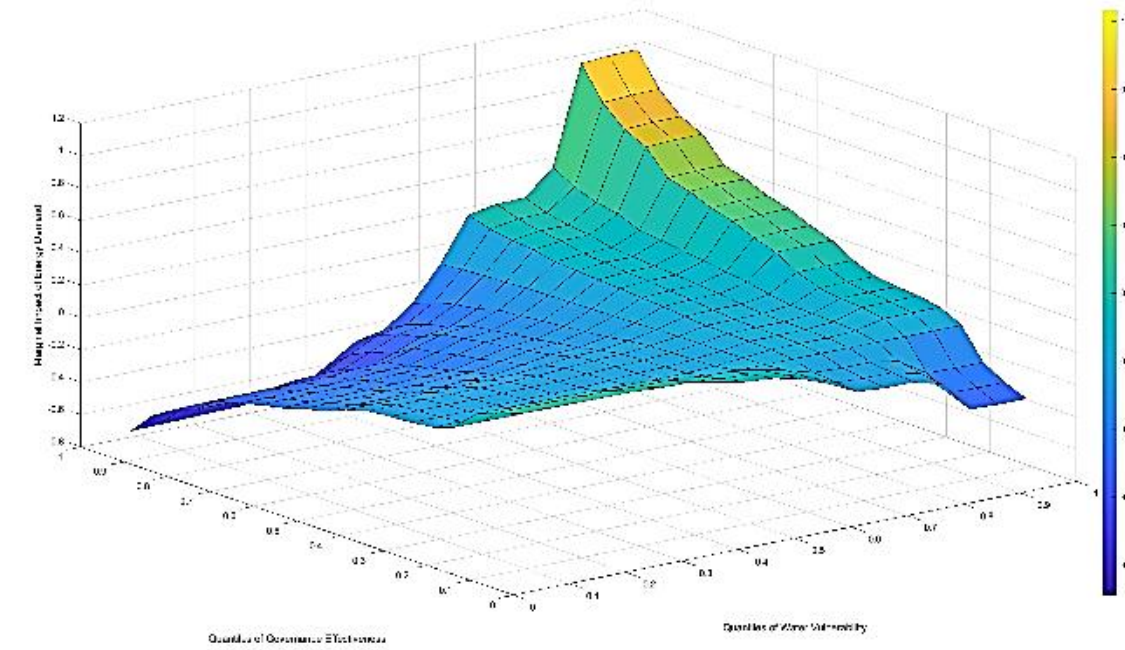

Governance Quality Indicator: Governance Effectiveness

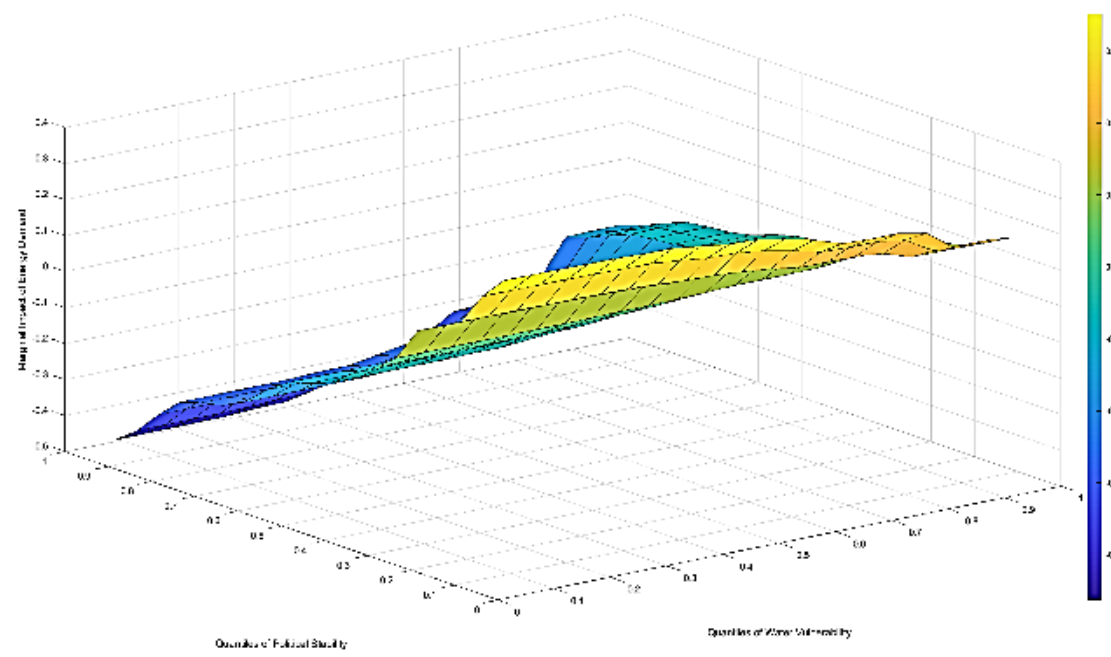

Governance Quality Indicator: Political Stability

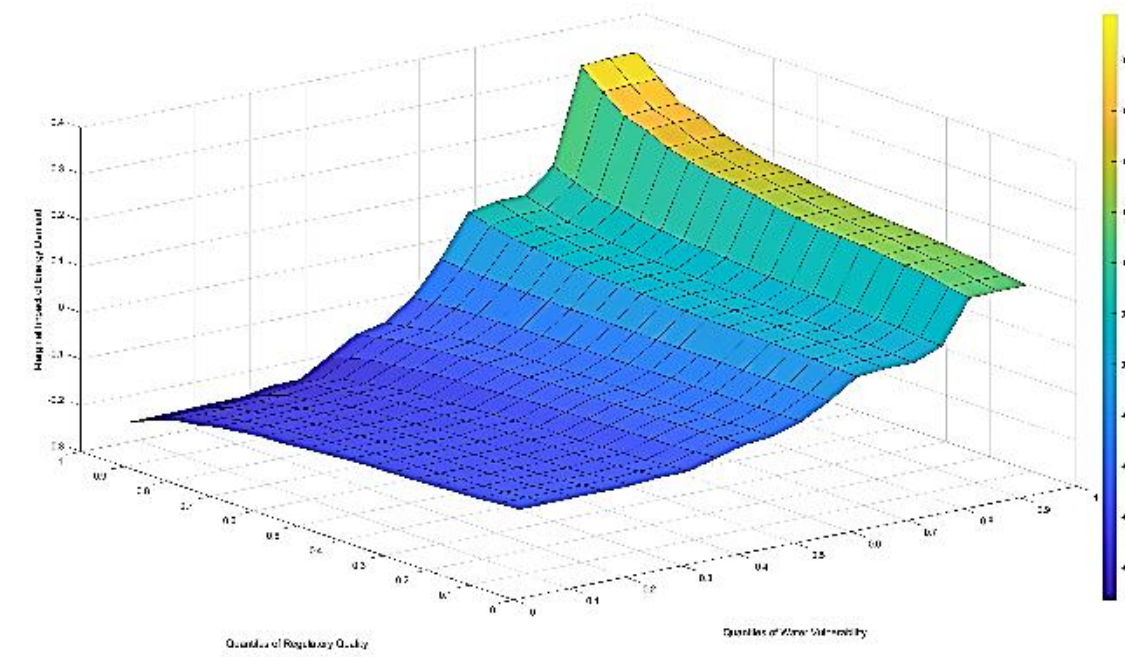

Governance Quality Indicator: Regulatory Quality

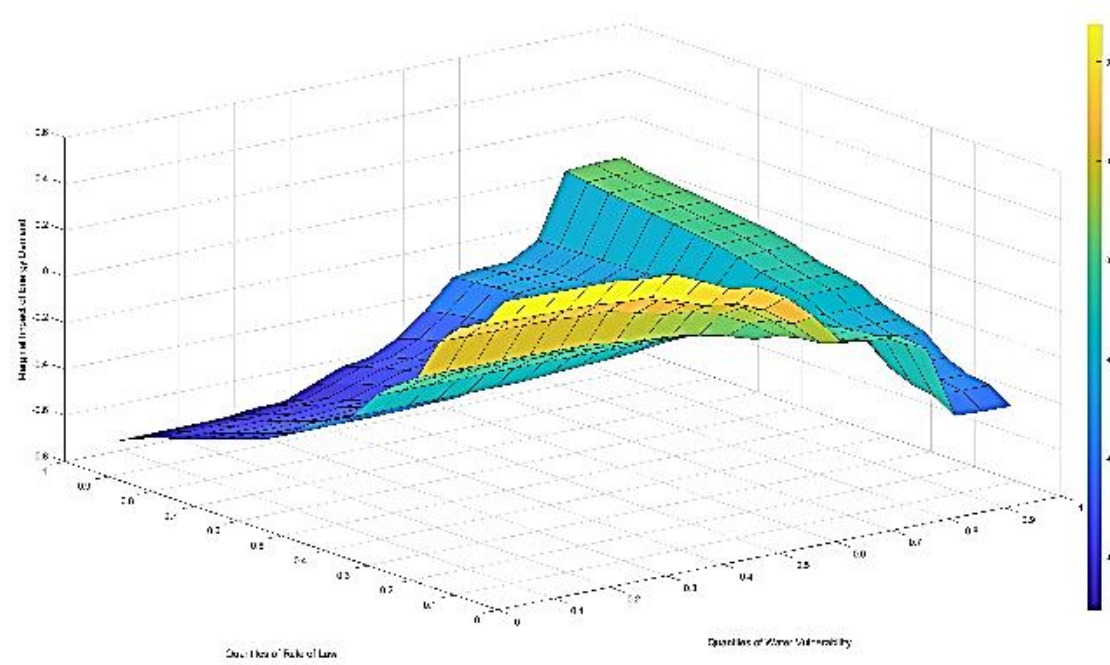

Governance Quality Indicator: Rule of Law

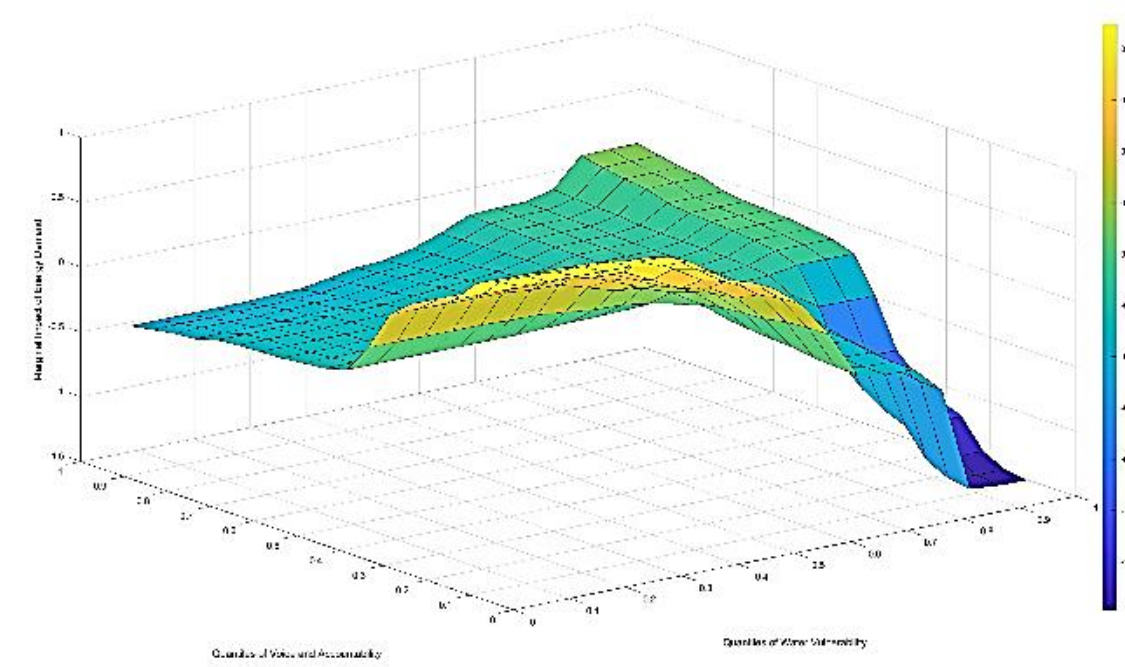

Governance Quality Indicator: Voice and Accountability

Figure S107: BRICS Policy Simulation Outcomes for the Marginal Impact of Energy Demand on SDG 7.3.1 under Water Vulnerability and Governance Quality, Related to Figure 4-5 and Table 2

Table S1: Variable description, Related to Key resource table, Related to Figure 2

Variable	Variable Description	Data Source
SDG 7 subgoals (Dependent Variables)		
SDG 7.1.1	SDG 7.1.1 Index	Sachs et al.[1]
SDG 7.1.2	SDG 7.1.2 Index	Sachs et al.[1]
SDG 7.2.1	SDG 7.2.1 Index	Sachs et al.[1]
SDG 7.3.1	Energy Intensity (MJ per 2017 USD PPP)	International Energy Agency[2]
SDG 7 subgoal Determinants		
Electricity Generation	Net Electricity Production per Capita (in GWh)	United Nations Statistics Division[3]<br>World Development Indicators[4]
Financial Access	Financial Institutions Access Index	World Bank[5]
Rural Population Growth	Rural Population Growth (Annual %)	World Development Indicators[4]
Income Inequality	Gini Coefficient of Pre-tax National Income	World Inequality Database[6]
Health Expenditure	Out-of-pocket Expenditure (% of Current Health Expenditure)	World Development Indicators[4]
Renewable Electricity Generation	Hydro, Wind, and Solar PV Electricity Generation (in GWh)	International Energy Agency[7]
Policy Stringency	Environmental Policy Stringency Index	OECD Statistics[8]
Per Capita Income	GDP per person employed (Constant 2021 PPP $)	World Development Indicators[4]
Urban Population Growth	Urban Population Growth (Annual %)	World Development Indicators[4]
Environmental Innovation	Total number of Environmental Patents	OECD Statistics[8]
Financial Development	Financial Development Index	World Bank[5]
Net Energy Import	Net Electricity Import (PJ)	United Nations Statistics Division[3]
Low Carbon Trade	Total trade in Low Carbon Technology Products (% of GDP)	International Monetary Fund[9]
Foreign Direct Investment	Foreign Direct Investment, Net Inflows (% of GDP)	World Development Indicators[4]
Industrial Share	Industry (including construction), Value Added (% of GDP)	World Development Indicators[4]
Energy Demand	Electricity Demand (TWh)	Ember[10]
Governance Quality (Moderator)		
Control of Corruption	perceptions of the extent to which public power is exercised for private gain, including both petty and grand forms of corruption, as well as "capture" of the state by elites and private interests	Worldwide Governance Indicators[11]
Governance Effectiveness	perceptions of the quality of public services, civil service and the degree of its independence from political pressures, policy formulation and implementation, and the credibility of the government's commitment to such policies	
Political Stability	perceptions of the likelihood of political instability and/or politically motivated violence, including terrorism.	
Regulatory Quality	perceptions of the ability of the government to formulate and implement sound policies and regulations that permit and promote private sector development	
Rule of Law	perceptions of the extent to which agents have confidence in and abide by the rules of society, and the quality of contract enforcement, property rights, the police, and the courts, as well as the likelihood of crime and violence	
Voice and Accountability	perceptions of the extent to which a country's citizens can participate in selecting their government, with freedom of expression, freedom of association, and free media	

Climate Vulnerability (Moderator)		
Ecosystem Vulnerability	Projected change in biome distribution, Projected change in marine biodiversity	ND-GAIN Database[12]
Food Vulnerability	Projected change in cereal yields, Projected population change	
Health Vulnerability	Projected change in vector-borne diseases, Projected change in deaths from climate-related diseases	
Infrastructure Vulnerability	Projected change in hydropower generation capacity, Projection of sea level rise impacts	
Water Vulnerability	Projected change in annual groundwater runoff, Projected change in annual groundwater recharge	

Table S2: Descriptive statistics, Related to Figure 2

Variable	N	Mean	Variance	Standard Deviation	Skewness	Kurtosis	p10	p90
SDG 7 subgoals (Dependent Variables)								
SDG 7.1.1	115	90.7928	156.9723	12.5289	-1.2880	3.6463	70.1860	100.0000
SDG 7.1.2	115	72.5208	546.4634	23.3766	-0.6624	2.1802	35.9180	96.0200
SDG 7.2.1	115	20.6405	764.4973	27.6495	1.2792	2.9696	0.3850	74.6150
SDG 7.3.1	115	6.8515	4.9922	2.2343	0.1871	2.0578	3.9000	10.0300
SDG 7 subgoal Determinants								
Electricity Generation	115	3505.3837	4451911.3348	2109.9553	0.2485	1.9182	728.1748	6896.0006
Financial Access	115	0.4178	0.0684	0.2615	0.2366	1.8127	0.0997	0.7566
Rural Population Growth	115	-0.5752	1.0044	1.0022	-0.1334	2.6122	-2.0430	0.8594
Income Inequality	115	0.6243	0.0049	0.0699	0.2212	1.8136	0.5439	0.7186
Health Expenditure	115	36.3234	387.5569	19.6865	0.2445	2.2688	7.7509	66.7600
Renewable Electricity Generation	115	358926.7391	234940000000.0000	484705.5356	2.6695	10.1710	5056.0000	772018.0000
Policy Stringency	115	1.1635	0.7050	0.8396	1.0649	3.0792	0.2500	2.7500
Per Capita Income	115	37250.6040	387575596.3747	19686.9397	0.4349	2.4871	11635.1824	69963.1105
Urban Population Growth	115	1.7809	1.3239	1.1506	-0.1778	2.4218	0.0804	3.1307
Environmental Innovation	115	7379.1701	292001068.2112	17088.0387	3.3106	13.5669	390.7300	21985.2000
Financial Development	115	0.5235	0.0071	0.0841	0.1477	1.9147	0.4198	0.6349
Net Energy Import	115	152.2740	71302832.2236	8444.1004	-0.2356	3.0299	-13767.5830	9586.7740
Low Carbon Trade	115	1.3365	0.8336	0.9130	1.5968	5.2696	0.5579	2.4341
Foreign Direct Investment	115	2.3082	2.0869	1.4446	1.1261	7.6468	0.6846	4.0035
Industrial Share	115	29.8445	60.7472	7.7941	0.9436	2.9256	22.4953	44.7934
Energy Demand	115	1500.3206	3751323.8363	1936.8335	2.2709	7.3196	230.0000	4700.2800
Governance Quality (Moderators)								
Control of Corruption	115	-0.3499	0.1537	0.3921	-0.0720	2.4839	-0.9408	0.0944
Governance Effectiveness	115	-0.0108	0.1106	0.3326	0.4070	2.8496	-0.4615	0.4386
Political Stability	115	-0.5584	0.1817	0.4262	-0.2470	2.3471	-1.1932	-0.0254
Regulatory Quality	115	-0.1116	0.1121	0.3348	1.0584	3.3775	-0.4641	0.4456
Rule of Law	115	-0.2887	0.1321	0.3634	-0.5615	2.0829	-0.8318	0.0977
Voice and Accountability	115	-0.2045	0.8008	0.8949	-0.5793	1.6277	-1.6314	0.6486
Climate Vulnerability (Moderators)								
Ecosystem Vulnerability	115	0.4850	0.0024	0.0488	0.0568	1.7159	0.4220	0.5476
Food Vulnerability	115	0.4438	0.0081	0.0899	1.1377	2.7911	0.3605	0.6153
Health Vulnerability	115	0.4657	0.0132	0.1151	0.8881	2.6414	0.3399	0.6681
Infrastructure Vulnerability	115	0.1595	0.0037	0.0611	0.2666	2.0420	0.0761	0.2556
Water Vulnerability	115	0.3563	0.0095	0.0972	0.7148	2.0062	0.2664	0.5228

Table S3: Multicollinearity Statistics for SDG 7.1.1 Determinants, Related to Figure 2

**Determinants**	**Models with Ecosystem Vulnerability**						**Models with Food Vulnerability**						**Models with Health Vulnerability**					
Electricity Generation	1.50	1.68	1.60	1.63	2.22	1.62	1.73	1.65	1.60	1.61	1.98	1.62	1.57	1.64	1.92	1.59	1.97	1.55
Financial Access	1.44	2.45	1.60	1.45	2.13	1.65	1.52	2.11	1.60	1.50	1.73	1.65	1.56	2.09	1.98	1.45	1.73	1.65
Rural Population Growth	1.50	1.35	1.82	1.12	1.05	1.48	1.48	1.24	1.83	1.12	1.12	1.45	1.35	1.24	1.07	1.12	1.12	1.45
Income Inequality	1.15	1.26	1.98	1.21	1.14	1.09	1.18	1.19	1.99	1.19	1.62	1.08	1.15	1.18	1.10	1.22	1.61	1.09
Health Expenditure	1.20	1.03	1.16	1.07	1.18	1.07	1.16	1.02	1.16	1.17	1.15	1.11	1.16	1.02	1.03	1.06	1.14	1.08
Ecosystem Vulnerability	1.20	1.82	1.00	1.08	1.17	1.10	-	-	-	-	-	-	-	-	-	-	-	-
Food Vulnerability	-	-	-	-	-	-	1.29	1.10	1.01	138	1.09	1.10	-	-	-	-	-	-
Health Vulnerability	-	-	-	-	-	-	-	-	-	-	-	-	1.14	1.06	1.08	1.10	1.06	1.03
Infrastructure Vulnerability	-	-	-	-	-	-	-	-	-	-	-	-	-	-	-	-	-	-
Water Vulnerability	-	-	-	-	-	-	-	-	-	-	-	-	-	-	-	-	-	-
Control of Corruption	1.14	-	-	-	-	-	1.12	-	-	-	-	-	1.12	-	-	-	-	-
Governance Effectiveness	-	2.64	-	-	-	-	-	1.60	-	-	-	-	-	1.54	-	-	-	-
Political Stability	-	-	2.67	-	-	-	-	-	2.69	-	-	-	-	-	1.04	-	-	-
Regulatory Quality	-	-	-	1.08	-	-	-	-	-	1.29	-	-	-	-	-	1.10	-	-
Rule of Law	-	-	-	-	1.20	-	-	-	-	-	2.64	-	-	-	-	-	2.57	-
Voice and Accountability	-	-	-	-	-	1.12	-	-	-	-	-	1.13	-	-	-	-	-	1.12
**Mean VIF**	**1.30**	**1.75**	**1.69**	**1.24**	**1.44**	**1.31**	**1.35**	**1.41**	**1.70**	**1.32**	**1.62**	**1.31**	**1.29**	**1.40**	**1.32**	**1.23**	**1.60**	**1.28**

**Determinants**	**Models with Infrastructure Vulnerability**						**Models with Water Vulnerability**					
Electricity Generation	2.04	1.98	1.64	2.33	1.98	1.56	1.51	1.64	1.60	1.62	1.97	1.60
Financial Access	2.62	2.66	1.90	2.38	1.73	1.65	1.46	2.08	1.60	1.45	1.73	1.66
Rural Population Growth	2.04	2.24	1.44	1.75	1.12	1.45	1.45	1.23	1.88	1.12	1.12	1.49
Income Inequality	2.18	1.61	1.42	1.36	1.62	1.13	1.15	1.18	2.05	1.25	1.61	1.12
Health Expenditure	1.23	1.06	1.06	1.07	1.15	1.07	1.17	1.02	1.17	1.08	1.14	1.22
Ecosystem Vulnerability	-	-	-	-	-	-	-	-	-	-	-	-
Food Vulnerability	-	-	-	-	-	-	-	-	-	-	-	-
Health Vulnerability	-	-	-	-	-	-	-	-	-	-	-	-
Infrastructure Vulnerability	1.75	1.80	1.77	1.76	1.09	1.12	-	-	-	-	-	-
Water Vulnerability	-	-	-	-	-	-	1.25	1.03	1.08	1.12	1.06	1.22
Control of Corruption	1.61	-	-	-	-	-	1.31	-	-	-	-	-
Governance Effectiveness	-	1.62	-	-	-	-	-	1.49	-	-	-	-
Political Stability	-	-	1.13	-	-	-	-	-	2.88	-	-	-
Regulatory Quality	-	-	-	1.21	-	-	-	-	-	1.08	-	-
Rule of Law	-	-	-	-	2.64	-	-	-	-	-	2.57	-
Voice and Accountability	-	-	-	-	-	1.13	-	-	-	-	-	1.15
**Mean VIF**	**1.92**	**1.85**	**1.48**	**1.70**	**1.62**	**1.30**	**1.33**	**1.38**	**1.75**	**1.25**	**1.60**	**1.35**

Table S4: Multicollinearity Statistics for SDG 7.1.2 Determinants, Related to Figure 2

Determinants	Models with Ecosystem Vulnerability						Models with Food Vulnerability						Models with Health Vulnerability					
Renewable Electricity Gen.	1.77	1.78	2.19	1.88	1.79	1.74	1.78	1.79	2.17	1.88	1.79	1.75	1.78	1.78	2.20	1.89	1.79	1.75
Financial Access	1.76	1.91	2.02	1.70	1.72	2.61	1.77	1.94	2.01	1.70	1.72	2.70	1.77	1.92	2.03	1.70	1.72	2.72
Policy Stringency	1.22	1.35	1.09	1.10	1.30	1.08	1.23	1.38	1.09	1.10	1.29	1.08	1.23	1.37	1.09	1.10	1.28	1.08
Per Capita Income	1.03	1.08	1.01	1.01	1.21	1.21	1.04	1.09	1.00	1.01	1.21	1.23	1.04	1.09	1.01	1.01	1.19	1.23
Urban Population Growth	1.06	1.20	1.41	1.02	1.05	1.05	1.07	1.22	1.39	1.02	1.05	1.06	1.07	1.21	1.42	1.02	1.05	1.06
Ecosystem Vulnerability	1.01	1.04	1.04	1.00	1.23	1.08	-	-	-	-	-	-	-	-	-	-	-	-
Food Vulnerability	-	-	-	-	-	-	1.10	1.15	1.00	1.05	1.23	1.19	-	-	-	-	-	-
Health Vulnerability	-	-	-	-	-	-	-	-	-	-	-	-	1.13	1.10	1.06	1.09	1.13	1.21
Infrastructure Vulnerability	-	-	-	-	-	-	-	-	-	-	-	-	-	-	-	-	-	-
Water Vulnerability	-	-	-	-	-	-	-	-	-	-	-	-	-	-	-	-	-	-
Control of Corruption	1.25	-	-	-	-	-	1.35	-	-	-	-	-	1.39	-	-	-	-	-
Governance Effectiveness	-	2.01	-	-	-	-	-	2.24	-	-	-	-	-	2.13	-	-	-	-
Political Stability	-	-	1.82	-	-	-	-	-	1.75	-	-	-	-	-	1.85	-	-	-
Regulatory Quality	-	-	-	1.20	-	-	-	-	-	1.26	-	-	-	-	-	1.32	-	-
Rule of Law	-	-	-	-	1.81	-	-	-	-	-	1.80	-	-	-	-	-	1.66	-
Voice and Accountability	-	-	-	-	-	2.06	-	-	-	-	-	2.26	-	-	-	-	-	2.30
**Mean VIF**	**1.30**	**1.48**	**1.51**	**1.27**	**1.44**	**1.55**	**1.33**	**1.54**	**1.49**	**1.29**	**1.44**	**1.61**	**1.34**	**1.51**	**1.52**	**1.30**	**1.40**	**1.62**

Determinants	Models with Infrastructure Vulnerability						Models with Water Vulnerability					
Renewable Electricity Gen.	1.77	1.78	2.20	1.88	1.78	1.74	1.83	1.81	2.56	2.12	1.81	1.26
Financial Access	1.76	1.91	2.03	1.70	1.71	2.55	1.83	2.00	2.28	1.70	1.72	1.93
Policy Stringency	1.22	1.35	1.09	1.10	1.26	1.08	1.35	1.47	1.10	1.13	1.35	1.09
Per Capita Income	1.04	1.08	1.01	1.01	1.17	1.19	1.07	1.12	1.01	1.02	1.25	1.32
Urban Population Growth	1.06	1.20	1.42	1.02	1.04	1.05	1.12	1.28	1.73	1.05	1.06	1.08
Ecosystem Vulnerability	-	-	-	-	-	-	-	-	-	-	-	-
Food Vulnerability	-	-	-	-	-	-	-	-	-	-	-	-
Health Vulnerability	-	-	-	-	-	-	-	-	-	-	-	-
Infrastructure Vulnerability	1.05	1.04	1.06	1.04	1.00	1.01	-	-	-	-	-	-
Water Vulnerability	-	-	-	-	-	-	2.00	1.47	1.85	2.48	1.52	1.65
Control of Corruption	1.30	-	-	-	-	-	2.47	-	-	-	-	-
Governance Effectiveness	-	2.02	-	-	-	-	-	2.85	-	-	-	-
Political Stability	-	-	1.86	-	-	-	-	-	3.23	-	-	-
Regulatory Quality	-	-	-	1.26	-	-	-	-	-	2.99	-	-
Rule of Law	-	-	-	-	1.47	-	-	-	-	-	2.23	-
Voice and Accountability	-	-	-	-	-	1.93	-	-	-	-	-	3.15
**Mean VIF**	**1.32**	**1.48**	**1.52**	**1.29**	**1.35**	**1.51**	**1.67**	**1.71**	**1.96**	**1.78**	**1.57**	**1.64**

Table S5: Multicollinearity Statistics for SDG 7.2.1 Determinants, Related to Figure 2

**Determinants**	**Models with Ecosystem Vulnerability**						**Models with Food Vulnerability**						**Models with Health Vulnerability**					
Policy Stringency	2.04	2.00	2.40	2.16	1.99	1.09	2.05	2.01	2.31	2.16	1.98	1.10	2.05	2.01	2.31	2.20	2.08	1.10
Environmental Innovation	2.59	2.73	2.55	2.49	2.73	1.48	2.60	2.74	2.54	2.49	2.67	1.49	2.61	2.76	2.54	2.49	2.48	1.49
Financial Development	1.25	1.19	1.52	1.33	1.36	1.31	1.25	1.19	1.45	1.33	1.36	1.29	1.25	1.19	1.45	1.35	1.23	1.31
Net Energy Import	2.07	1.21	1.23	1.27	1.03	1.01	2.11	1.22	1.19	1.28	1.08	1.01	2.19	1.23	1.19	1.31	2.53	1.01
Low Carbon Trade	1.26	1.29	1.30	1.23	1.22	1.12	1.26	1.29	1.28	1.23	1.22	1.18	1.26	1.30	1.28	1.23	1.41	1.13
Ecosystem Vulnerability	1.00	1.03	1.27	1.01	1.13	1.05	-	-	-	-	-	-	-	-	-	-	-	-
Food Vulnerability	-	-	-	-	-	-	1.04	1.07	1.00	1.03	1.14	1.09	-	-	-	-	-	-
Health Vulnerability	-	-	-	-	-	-	-	-	-	-	-	-	1.12	1.16	1.01	1.18	1.01	1.04
Infrastructure Vulnerability	-	-	-	-	-	-	-	-	-	-	-	-	-	-	-	-	-	-
Water Vulnerability	-	-	-	-	-	-	-	-	-	-	-	-	-	-	-	-	-	-
Control of Corruption	2.14	-	-	-	-	-	2.22	-	-	-	-	-	2.40	-	-	-	-	-
Governance Effectiveness	-	1.84	-	-	-	-	-	1.91	-	-	-	-	-	2.07	-	-	-	-
Political Stability	-	-	2.18	-	-	-	-	-	1.72	-	-	-	-	-	1.73	-	-	-
Regulatory Quality	-	-	-	1.56	-	-	-	-	-	1.62	-	-	-	-	-	1.85	-	-
Rule of Law	-	-	-	-	1.31	-	-	-	-	-	1.18	-	-	-	-	-	2.67	-
Voice and Accountability	-	-	-	-	-	1.07	-	-	-	-	-	1.06	-	-	-	-	-	1.06
**Mean VIF**	**1.76**	**1.61**	**1.78**	**1.58**	**1.54**	**1.16**	**1.79**	**1.63**	**1.64**	**1.59**	**1.52**	**1.18**	**1.84**	**1.67**	**1.64**	**1.66**	**1.92**	**1.16**

**Determinants**	**Models with Infrastructure Vulnerability**						**Models with Water Vulnerability**					
Policy Stringency	2.07	2.01	2.37	2.22	2.08	1.09	2.07	2.01	2.36	2.26	2.00	1.12
Environmental Innovation	2.63	2.76	2.55	2.49	2.48	1.48	2.63	2.77	2.54	2.49	2.69	1.58
Financial Development	1.26	1.19	1.50	1.37	1.23	1.28	1.26	1.19	1.49	1.40	1.36	1.45
Net Energy Import	2.39	1.23	1.21	1.35	2.55	1.01	2.38	1.24	1.21	1.38	1.03	1.01
Low Carbon Trade	1.26	1.29	1.29	1.23	1.41	1.13	1.26	1.30	1.29	1.23	1.21	1.13
Ecosystem Vulnerability	-	-	-	-	-	-	-	-	-	-	-	-
Food Vulnerability	-	-	-	-	-	-	-	-	-	-	-	-
Health Vulnerability	-	-	-	-	-	-	-	-	-	-	-	-
Infrastructure Vulnerability	1.31	1.15	1.19	1.32	1.02	1.03	-	-	-	-	-	-
Water Vulnerability	-	-	-	-	-	-	1.30	1.21	1.15	1.49	1.05	1.31
Control of Corruption	2.82	-	-	-	-	-	2.78	-	-	-	-	-
Governance Effectiveness	-	2.05	-	-	-	-	-	2.16	-	-	-	-
Political Stability	-	-	2.04	-	-	-	-	-	1.98	-	-	-
Regulatory Quality	-	-	-	2.07	-	-	-	-	-	2.33	-	-
Rule of Law	-	-	-	-	2.71	-	-	-	-	-	1.21	-
Voice and Accountability	-	-	-	-	-	1.07	-	-	-	-	-	1.18
**Mean VIF**	**1.96**	**1.67**	**1.74**	**1.72**	**1.93**	**1.16**	**1.95**	**1.70**	**1.72**	**1.80**	**1.51**	**1.25**

Table S6: Multicollinearity Statistics for SDG 7.3.1 Determinants, Related to Figure 2

**Determinants**	**Models with Ecosystem Vulnerability**						**Models with Food Vulnerability**						**Models with Health Vulnerability**					
Policy Stringency	1.48	1.58	1.47	1.66	1.95	1.82	1.42	1.60	1.42	1.55	1.91	1.66	1.28	1.51	1.31	1.40	1.90	1.49
Environmental Innovation	1.91	1.79	1.67	1.56	1.30	1.54	2.44	2.30	2.01	2.00	1.52	1.99	2.28	2.08	1.92	1.83	1.46	1.86
Foreign Direct Investment	1.14	1.15	1.14	1.13	1.05	1.15	1.14	1.16	1.14	1.14	1.06	1.15	1.12	1.15	1.12	1.12	1.06	1.17
Industrial Share	2.57	1.74	1.81	2.23	2.21	2.12	1.72	1.39	1.53	1.72	2.09	1.61	1.88	1.44	1.57	1.79	2.13	1.68
Energy Demand	1.24	1.25	1.27	1.24	1.48	1.55	1.38	1.41	1.39	1.39	1.70	1.62	1.21	1.25	1.23	1.21	1.52	1.42
Ecosystem Vulnerability	2.69	2.16	1.87	2.06	1.45	2.74	-	-	-	-	-	-	-	-	-	-	-	-
Food Vulnerability	-	-	-	-	-	-	2.64	2.38	2.30	2.28	1.62	2.61	-	-	-	-	-	-
Health Vulnerability	-	-	-	-	-	-	-	-	-	-	-	-	2.07	1.77	1.67	1.70	1.39	1.89
Infrastructure Vulnerability	-	-	-	-	-	-	-	-	-	-	-	-	-	-	-	-	-	-
Water Vulnerability	-	-	-	-	-	-	-	-	-	-	-	-	-	-	-	-	-	-
Control of Corruption	1.88	-	-	-	-	-	1.54	-	-	-	-	-	1.62	-	-	-	-	-
Governance Effectiveness	-	1.88	-	-	-	-	-	1.73	-	-	-	-	-	1.72	-	-	-	-
Political Stability	-	-	1.39	-	-	-	-	-	1.42	-	-	-	-	-	1.39	-	-	-
Regulatory Quality	-	-	-	1.63	-	-	-	-	-	1.50	-	-	-	-	-	1.51	-	-
Rule of Law	-	-	-	-	1.11	-	-	-	-	-	1.09	-	-	-	-	-	1.10	-
Voice and Accountability	-	-	-	-	-	1.16	-	-	-	-	-	1.15	-	-	-	-	-	1.13
**Mean VIF**	**1.84**	**1.65**	**1.52**	**1.65**	**1.51**	**1.73**	**1.76**	**1.71**	**1.60**	**1.66**	**1.57**	**1.69**	**1.64**	**1.56**	**1.46**	**1.51**	**1.51**	**1.52**

**Determinants**	**Models with Infrastructure Vulnerability**						**Models with Water Vulnerability**					
Policy Stringency	1.64	1.80	1.62	1.73	2.06	2.13	1.84	1.99	1.81	1.91	2.02	2.10
Environmental Innovation	1.58	1.56	1.56	1.39	1.29	1.53	2.05	1.95	1.81	1.72	1.39	1.76
Foreign Direct Investment	1.14	1.18	1.14	1.14	1.06	1.33	1.11	1.15	1.12	1.12	1.05	1.20
Industrial Share	1.73	1.40	1.53	1.72	2.07	1.61	2.21	1.71	1.77	2.01	2.32	2.03
Energy Demand	1.06	1.11	1.05	1.04	1.31	1.30	1.21	1.26	1.21	1.21	1.49	1.40
Ecosystem Vulnerability	-	-	-	-	-	-	-	-	-	-	-	-
Food Vulnerability	-	-	-	-	-	-	-	-	-	-	-	-
Health Vulnerability	-	-	-	-	-	-	-	-	-	-	-	-
Infrastructure Vulnerability	1.71	1.50	1.47	1.46	1.20	1.91	-	-	-	-	-	-
Water Vulnerability	-	-	-	-	-	-	2.69	2.48	2.51	2.43	1.60	2.99
Control of Corruption	1.53	-	-	-	-	-	1.45	-	-	-	-	-
Governance Effectiveness	-	1.68	-	-	-	-	-	1.66	-	-	-	-
Political Stability	-	-	1.40	-	-	-	-	-	1.43	-	-	-
Regulatory Quality	-	-	-	1.48	-	-	-	-	-	1.48	-	-
Rule of Law	-	-	-	-	1.09	-	-	-	-	-	1.09	-
Voice and Accountability	-	-	-	-	-	1.13	-	-	-	-	-	1.19
**Mean VIF**	**1.48**	**1.46**	**1.40**	**1.42**	**1.44**	**1.56**	**1.79**	**1.74**	**1.67**	**1.70**	**1.57**	**1.81**

Table S7: Long Run Coefficient Estimation using Generalized Least Square with Ecosystem Vulnerability for SDG 7.1.1 – With Control of Corruption, Governance Effectiveness, and Political Stability, Related to Figure 3

Variables	Model 1	Model 2	Model 1	Model 2	Model 1	Model 2
	Control of Corruption		Governance Effectiveness		Political Stability	
Electricity Generation	-0.0186	0.1173	0.0064	-0.0708	-0.0165	0.1915
	(0.0175)	(0.2589)	(0.0162)	(0.2288)	(0.0181)	(0.2872)
Financial Access	0.1483***	-0.4696	0.1118***	-1.3058*	0.1551***	-1.1516
	(0.0292)	(0.8799)	(0.0263)	(0.7312)	(0.0290)	(1.0364)
Rural Population Growth	-0.0517***	0.5758***	-0.0541***	0.7459***	-0.0463***	0.3793**
	(0.0057)	(0.1245)	(0.0054)	(0.0987)	(0.0055)	(0.1704)
Income Inequality	-0.1658	-5.5105**	-0.1249	-3.8438*	-0.2103	-3.7723
	(0.1487)	(2.6968)	(0.1442)	(1.9897)	(0.1398)	(3.0469)
Health Expenditure	0.0019**	-0.0232*	0.0023***	-0.0273***	0.0016**	-0.0527***
	(0.0008)	(0.0135)	(0.0008)	(0.0103)	(0.0007)	(0.0142)
Ecosystem Vulnerability	-0.6087**	-8.2051	-0.4490*	-11.0790*	-0.7271***	-8.6997
	(0.2444)	(5.5431)	(0.2462)	(5.7548)	(0.2407)	(6.0976)
Quality of Governance	-0.0396***	1.4574*	-0.0657***	0.2488	-0.0071	0.3933
	(0.0153)	(0.7864)	(0.0152)	(1.3456)	(0.0104)	(0.9836)
Electricity Generation * Ecosystem Vulnerability	-	-0.4052	-	0.1183	-	-0.4675
	-	(0.5474)	-	(0.4957)	-	(0.6678)
Financial Access * Ecosystem Vulnerability	-	1.1158	-	3.2505**	-	2.5596
	-	(1.8535)	-	(1.5659)	-	(2.2477)
Rural Population Growth * Ecosystem Vulnerability	-	-1.2840***	-	-1.6853***	-	-0.8371**
	-	(0.2785)	-	(0.2196)	-	(0.3828)
Income Inequality * Ecosystem Vulnerability	-	10.7224*	-	7.9516**	-	7.9200
	-	(5.5196)	-	(3.9850)	-	(6.2586)
Health Expenditure * Ecosystem Vulnerability	-	0.0495*	-	0.0615***	-	0.1150***
	-	(0.0282)	-	(0.0220)	-	(0.0311)
Electricity Generation * Quality of Governance	-	0.4333	-	1.2981***	-	0.2506
	-	(0.3393)	-	(0.3444)	-	(0.2192)
Financial Access * Quality of Governance	-	-0.1249	-	0.0611	-	0.7929
	-	(1.3503)	-	(1.9665)	-	(1.3921)
Rural Population Growth * Quality of Governance	-	0.1701	-	0.6986	-	0.0571
	-	(0.2122)	-	(0.4763)	-	(0.2491)
Income Inequality * Quality of Governance	-	-5.3180	-	-16.5689***	-	-0.1279
	-	(4.3610)	-	(4.5479)	-	(3.0992)
Health Expenditure * Quality of Governance	-	-0.0226*	-	-0.0042	-	-0.0514***
	-	(0.0135)	-	(0.0257)	-	(0.0175)
Electricity Generation * Quality of Governance * Ecosystem Vulnerability	-	-1.2105	-	-3.0728***	-	-0.6054
	-	(0.7602)	-	(0.8054)	-	(0.5233)
Financial Access * Quality of Governance * Ecosystem Vulnerability	-	0.3200	-	0.0376	-	-1.9124
	-	(2.9856)	-	(4.2686)	-	(3.1569)
Rural Population Growth * Quality of Governance * Ecosystem Vulnerability	-	-0.3477	-	-1.4464	-	-0.0557

	-	(0.4719)	-	(0.9895)	-	(0.5445)
Income Inequality * Quality of Governance * Ecosystem Vulnerability	-	9.8714	-	38.0490***	-	0.0101
	-	(9.7480)	-	(10.0560)	-	(6.2137)
Health Expenditure * Quality of Governance * Ecosystem Vulnerability	-	0.0500*	-	0.0124	-	0.1074***
	-	(0.0280)	-	(0.0520)	-	(0.0377)
Constant	4.8970***	9.2535***	4.6076***	9.7646***	4.9802***	8.8169***
	(0.2998)	(2.8779)	(0.3141)	(2.8683)	(0.2910)	(2.9356)
Wald-chi2	420.3	589.0	386.2	1108	266.9	518.3
Observations	115	115	115	115	115	115
Heteroskedasticity controlled	Yes	Yes	Yes	Yes	Yes	Yes
Autocorrelation controlled	Yes	Yes	Yes	Yes	Yes	Yes
Cross-sectional correlation controlled	Yes	Yes	Yes	Yes	Yes	Yes
Marginal Impact						
Electricity Generation	-0.0186	-0.0317	0.0064	-0.0339	-0.0165	-0.0078
	(0.0175)	(0.0348)	(0.0162)	(0.0413)	(0.0181)	(0.0353)
Financial Access	0.1483***	0.0626	0.1118***	0.2700***	0.1551***	0.1756***
	(0.0292)	(0.0443)	(0.0263)	(0.0595)	(0.0290)	(0.0567)
Rural Population Growth	-0.0517***	-0.0492***	-0.0541***	-0.0820***	-0.0463***	-0.0431***
	(0.0057)	(0.0094)	(0.0054)	(0.0148)	(0.0055)	(0.0105)
Income Inequality	-0.1658	-0.0742	-0.1249	0.2721	-0.2103	0.1372
	(0.1487)	(0.2121)	(0.1442)	(0.2385)	(0.1398)	(0.2278)
Health Expenditure	0.0019**	0.0005	0.0023***	0.0026**	0.0016**	0.0021*
	(0.0008)	(0.0012)	(0.0008)	(0.0013)	(0.0007)	(0.0012)
Note:<br>Standard errors in parentheses ()<br>*** p<0.01, ** p<0.05, * p<0.1 by Z-test						

Table S8: Long Run Coefficient Estimation using Generalized Least Square with Ecosystem Vulnerability for SDG 7.1.1 – With Regulatory Quality, Rule of Law, and Voice and Accountability, Related to Figure 3

Variables	Model 1	Model 2	Model 1	Model 2	Model 1	Model 2
	Regulatory Quality			Rule of Law	Voice and Accountability	
Electricity Generation	-0.0254	-0.1090	-0.0049	1.3004***	-0.0243	0.5682***
	(0.0179)	(0.2421)	(0.0186)	(0.3724)	(0.0163)	(0.2168)
Financial Access	0.1206***	-0.0124	0.1117***	-0.6304	0.0981***	-2.3063***
	(0.0349)	(0.7029)	(0.0323)	(1.1728)	(0.0269)	(0.7940)
Rural Population Growth	-0.0393***	0.3988***	-0.0478***	0.7106***	-0.0145**	0.4130***
	(0.0062)	(0.1236)	(0.0069)	(0.1512)	(0.0060)	(0.1285)
Income Inequality	-0.4133***	-4.3441*	-0.0256	-4.0424	0.2280	-5.7952***
	(0.1472)	(2.3114)	(0.1623)	(2.6588)	(0.1513)	(1.7052)
Health Expenditure	0.0011	-0.0142	0.0009	0.0208	0.0002	-0.0015
	(0.0008)	(0.0120)	(0.0009)	(0.0131)	(0.0007)	(0.0084)
Ecosystem Vulnerability	-0.9652***	-10.8228*	-0.2956	14.6123**	-1.0408***	-1.0939
	(0.2685)	(5.9778)	(0.2506)	(6.6560)	(0.2142)	(4.2242)
Quality of Governance	-0.0426**	-1.0553	-0.0785***	-1.4662	-0.0864***	0.0368
	(0.0173)	(1.4697)	(0.0224)	(0.9574)	(0.0133)	(0.3401)
Electricity Generation * Ecosystem Vulnerability	-	0.2407	-	-2.7624***	-	-1.1672***
	-	(0.5439)	-	(0.7569)	-	(0.4411)
Financial Access * Ecosystem Vulnerability	-	0.1294	-	1.9966	-	5.6146***
	-	(1.5347)	-	(2.4540)	-	(1.7530)
Rural Population Growth * Ecosystem Vulnerability	-	-0.8992***	-	-1.5919***	-	-0.9455***
	-	(0.2707)	-	(0.3247)	-	(0.2858)
Income Inequality * Ecosystem Vulnerability	-	9.3180*	-	9.3993*	-	12.6222***
	-	(4.7779)	-	(5.1551)	-	(3.6062)
Health Expenditure * Ecosystem Vulnerability	-	0.0345	-	-0.0389	-	0.0070
	-	(0.0260)	-	(0.0262)	-	(0.0175)
Electricity Generation * Quality of Governance	-	0.5748	-	1.4750***	-	0.1667
	-	(0.4096)	-	(0.3959)	-	(0.1498)
Financial Access * Quality of Governance	-	0.5968	-	-1.0489	-	-1.2291*
	-	(2.2566)	-	(1.7719)	-	(0.7379)
Rural Population Growth * Quality of Governance	-	0.1963	-	0.2999	-	0.6012***
	-	(0.3345)	-	(0.2589)	-	(0.1149)
Income Inequality * Quality of Governance	-	-5.5617	-	-16.7242***	-	0.1763
	-	(5.8144)	-	(4.8259)	-	(1.8725)
Health Expenditure * Quality of Governance	-	-0.0229	-	0.0117	-	0.0033
	-	(0.0188)	-	(0.0149)	-	(0.0063)
Electricity Generation * Quality of Governance * Ecosystem Vulnerability	-	-1.1268	-	-3.1158***	-	-0.3431
	-	(1.0320)	-	(0.8518)	-	(0.3142)
Financial Access * Quality of Governance * Ecosystem Vulnerability	-	-0.4376	-	3.2699	-	2.9276*
	-	(4.8931)	-	(3.8959)	-	(1.5780)
Rural Population Growth * Quality of Governance * Ecosystem Vulnerability	-	-0.3954	-	-0.6802	-	-1.3264***

	-	(0.7279)	-	(0.5439)	-	(0.2421)
Income Inequality * Quality of Governance * Ecosystem Vulnerability	-	12.9596	-	38.5258***	-	-1.2865
	-	(12.1408)	-	(10.3056)	-	(3.8670)
Health Expenditure * Quality of Governance * Ecosystem Vulnerability	-	0.0586	-	-0.0085	-	-0.0025
	-	(0.0400)	-	(0.0302)	-	(0.0129)
Constant	5.3391***	9.4833***	4.5908***	-2.8422	4.9995***	4.6619**
	(0.2962)	(2.8759)	(0.3116)	(3.5279)	(0.2419)	(2.1605)
Wald-chi2	292.3	354.0	123.3	511.1	123.0	1283
Observations	115	115	115	115	115	115
Heteroskedasticity controlled	Yes	Yes	Yes	Yes	Yes	Yes
Autocorrelation controlled	Yes	Yes	Yes	Yes	Yes	Yes
Cross-sectional correlation controlled	Yes	Yes	Yes	Yes	Yes	Yes
Marginal Impact						
Electricity Generation	-0.0254	0.0061	-0.0049	-0.0594	-0.0243	0.0013
	(0.0179)	(0.0367)	(0.0186)	(0.0387)	(0.0163)	(0.0319)
Financial Access	0.1206***	0.0081	0.1117***	0.2151***	0.0981***	0.3841***
	(0.0349)	(0.0503)	(0.0323)	(0.0564)	(0.0269)	(0.0707)
Rural Population Growth	-0.0393***	-0.0372***	-0.0478***	-0.0595***	-0.0145**	-0.0399***
	(0.0062)	(0.0094)	(0.0069)	(0.0124)	(0.0060)	(0.0122)
Income Inequality	-0.4133***	0.0761	-0.0256	0.3292	0.2280	0.4146
	(0.1472)	(0.2276)	(0.1623)	(0.2498)	(0.1513)	(0.2557)
Health Expenditure	0.0011	0.0019	0.0009	-0.0003	0.0002	0.0014
	(0.0008)	(0.0013)	(0.0009)	(0.0012)	(0.0007)	(0.0010)

Note:
Standard errors in parentheses ()
*** p<0.01, ** p<0.05, * p<0.1 by Z-test by Z-test

Table S9: Long Run Coefficient Estimation using Generalized Least Square with Food Vulnerability for SDG 7.1.1 – With Control of Corruption, Governance Effectiveness, and Political Stability, Related to Figure 3

Variables	Model 1	Model 2	Model 1	Model 2	Model 1	Model 2
	Control of Corruption		Governance Effectiveness		Political Stability	
Electricity Generation	-0.0408***	0.5506***	-0.0315**	0.0486	-0.0340**	1.0506***
	(0.0135)	(0.1872)	(0.0134)	(0.1843)	(0.0141)	(0.1170)
Financial Access	0.0462*	-1.4578**	0.0274	-2.0563***	0.0434*	-0.9918*
	(0.0251)	(0.5899)	(0.0236)	(0.4591)	(0.0263)	(0.5554)
Rural Population Growth	-0.0268***	0.4234***	-0.0287***	0.3123***	-0.0242***	-0.4228**
	(0.0047)	(0.1273)	(0.0055)	(0.1085)	(0.0044)	(0.1666)
Income Inequality	-0.0700	-7.9682***	-0.0620	-4.7865***	-0.0844	-2.6982*
	(0.0988)	(1.9605)	(0.0963)	(1.0904)	(0.0983)	(1.6277)
Health Expenditure	0.0011*	0.0057	0.0012**	-0.0170***	0.0012*	0.0465***
	(0.0006)	(0.0076)	(0.0006)	(0.0053)	(0.0006)	(0.0099)
Food Vulnerability	-1.8850***	-2.3720	-1.9114***	-10.2407**	-2.0031***	17.5406***
	(0.2756)	(2.9108)	(0.3005)	(4.2784)	(0.2816)	(3.2222)
Quality of Governance	-0.0226*	-0.0131	-0.0244*	-0.4805	0.0011	0.2891
	(0.0125)	(0.5912)	(0.0135)	(0.6307)	(0.0094)	(0.6106)
Electricity Generation * Food Vulnerability	-	-1.3839***	-	-0.1555	-	-2.8484***
	-	(0.4787)	-	(0.4519)	-	(0.3176)
Financial Access * Food Vulnerability	-	4.3040***	-	5.3739***	-	2.1476
	-	(1.4227)	-	(1.1652)	-	(1.4152)
Rural Population Growth * Food Vulnerability	-	-1.0250***	-	-0.7736***	-	1.0291**
	-	(0.3110)	-	(0.2691)	-	(0.4084)
Income Inequality * Food Vulnerability	-	18.3993***	-	10.8526***	-	7.3687*
	-	(4.7642)	-	(2.6933)	-	(3.8465)
Health Expenditure * Food Vulnerability	-	-0.0042	-	0.0454***	-	-0.1083***
	-	(0.0166)	-	(0.0125)	-	(0.0230)
Electricity Generation * Quality of Governance	-	0.5631**	-	0.5436**	-	-0.1342
	-	(0.2236)	-	(0.2611)	-	(0.1319)
Financial Access * Quality of Governance	-	-0.4532	-	-2.5584***	-	3.0242***
	-	(0.7385)	-	(0.9578)	-	(0.7554)
Rural Population Growth * Quality of Governance	-	-0.0341	-	-0.0257	-	-1.5508***
	-	(0.2606)	-	(0.3044)	-	(0.2525)
Income Inequality * Quality of Governance	-	-8.2568***	-	-6.5699*	-	-1.8174
	-	(3.1110)	-	(3.6512)	-	(1.6837)
Health Expenditure * Quality of Governance	-	0.0167	-	0.0171	-	0.0578***
	-	(0.0123)	-	(0.0156)	-	(0.0115)
Electricity Generation * Quality of Governance * Food Vulnerability	-	-1.3880**	-	-1.2292*	-	0.1044
	-	(0.5892)	-	(0.7266)	-	(0.3915)
Financial Access * Quality of Governance * Food Vulnerability	-	1.6257	-	6.6574***	-	-8.4510***
	-	(1.9430)	-	(2.5682)	-	(1.9934)
Rural Population Growth * Quality of Governance * Food Vulnerability	-	0.1960	-	0.1456	-	3.9526***

	-	(0.6701)	-	(0.7828)	-	(0.6266)
Income Inequality * Quality of Governance * Food Vulnerability	-	19.2619**	-	15.6868*	-	6.2524*
	-	(7.8199)	-	(9.3137)	-	(3.7399)
Health Expenditure * Quality of Governance * Food Vulnerability	-	-0.0263	-	-0.0256	-	-0.1322***
	-	(0.0289)	-	(0.0349)	-	(0.0250)
Constant	5.5919***	5.6878***	5.5314***	8.9946***	5.6019***	-1.9657
	(0.2315)	(1.3486)	(0.2549)	(1.8071)	(0.2375)	(1.3528)
Wald-chi2	507.7	3031	514.2	2192	310.2	4062
Observations	115	115	115	115	115	115
Heteroskedasticity controlled	Yes	Yes	Yes	Yes	Yes	Yes
Autocorrelation controlled	Yes	Yes	Yes	Yes	Yes	Yes
Cross-sectional correlation controlled	Yes	Yes	Yes	Yes	Yes	Yes
Marginal Impact						
Electricity Generation	-0.0408***	-0.0549*	-0.0315**	-0.0284	-0.0340**	-0.1662***
	(0.0135)	(0.0323)	(0.0134)	(0.0286)	(0.0141)	(0.0250)
Financial Access	0.0462*	0.3701***	0.0274	0.3679***	0.0434*	0.4999***
	(0.0251)	(0.0569)	(0.0236)	(0.0662)	(0.0263)	(0.0607)
Rural Population Growth	-0.0268***	-0.0487***	-0.0287***	-0.0305**	-0.0242***	-0.1418***
	(0.0047)	(0.0101)	(0.0055)	(0.0126)	(0.0044)	(0.0144)
Income Inequality	-0.0700	0.2325	-0.0620	0.1282	-0.0844	-0.0606
	(0.0988)	(0.1768)	(0.0963)	(0.1980)	(0.0983)	(0.1273)
Health Expenditure	0.0011*	0.0019**	0.0012**	0.0029***	0.0012*	0.0010*
	(0.0006)	(0.0008)	(0.0006)	(0.0008)	(0.0006)	(0.0005)

Note:
Standard errors in parentheses ()
*** p<0.01, ** p<0.05, * p<0.1 by Z-test by Z-test

Table S10: Long Run Coefficient Estimation using Generalized Least Square with Food Vulnerability for SDG 7.1.1 – With Regulatory Quality, Rule of Law, and Voice and Accountability, Related to Figure 3

Variables	Model 1	Model 2	Model 1	Model 2	Model 1	Model 2
	Regulatory Quality		Rule of Law		Voice and Accountability	
Electricity Generation	-0.0416***	0.5841***	-0.0223	1.0034***	-0.0244	0.4648***
	(0.0144)	(0.2007)	(0.0154)	(0.1659)	(0.0149)	(0.1570)
Financial Access	0.0391	-2.0759***	0.0735***	-3.7345***	0.0668**	-1.1716***
	(0.0280)	(0.5255)	(0.0282)	(0.3793)	(0.0276)	(0.4333)
Rural Population Growth	-0.0217***	0.3156***	-0.0315***	0.6816***	-0.0064	0.2873**
	(0.0049)	(0.1155)	(0.0065)	(0.0849)	(0.0052)	(0.1174)
Income Inequality	-0.1632	-6.4502***	-0.0700	-3.9459***	0.2598*	-5.5492***
	(0.1069)	(1.8548)	(0.1190)	(1.0795)	(0.1344)	(1.1708)
Health Expenditure	0.0008	0.0018	0.0015**	0.0130**	0.0005	-0.0070
	(0.0006)	(0.0094)	(0.0008)	(0.0060)	(0.0006)	(0.0058)
Food Vulnerability	-2.0751***	-0.9622	-1.4913***	8.7365***	-1.3598***	-1.6105
	(0.2962)	(4.9630)	(0.2816)	(2.8864)	(0.2826)	(3.5273)
Quality of Governance	-0.0096	-0.5134	-0.0221	-1.2214*	-0.0527***	-0.2372
	(0.0152)	(1.3528)	(0.0200)	(0.6685)	(0.0124)	(0.3489)
Electricity Generation * Food Vulnerability	-	-1.5160***	-	-2.2668***	-	-1.1613***
	-	(0.5501)	-	(0.3761)	-	(0.3840)
Financial Access * Food Vulnerability	-	5.6622***	-	9.8124***	-	3.4882***
	-	(1.3243)	-	(0.9209)	-	(1.1054)
Rural Population Growth * Food Vulnerability	-	-0.8079***	-	-1.6172***	-	-0.6938**
	-	(0.2811)	-	(0.2022)	-	(0.2885)
Income Inequality * Food Vulnerability	-	15.4887***	-	8.9668***	-	13.1307***
	-	(4.3277)	-	(2.3062)	-	(2.7482)
Health Expenditure * Food Vulnerability	-	0.0049	-	-0.0168	-	0.0166
	-	(0.0224)	-	(0.0120)	-	(0.0132)
Electricity Generation * Quality of Governance	-	-0.0042	-	1.1549***	-	0.2657**
	-	(0.2576)	-	(0.1636)	-	(0.1070)
Financial Access * Quality of Governance	-	1.6162	-	-4.6363***	-	-0.7286
	-	(1.1118)	-	(0.6663)	-	(0.4593)
Rural Population Growth * Quality of Governance	-	-0.4449	-	0.4393***	-	0.4825***
	-	(0.3848)	-	(0.1442)	-	(0.1102)
Income Inequality * Quality of Governance	-	-3.4216	-	-11.2024***	-	-1.1350
	-	(4.4065)	-	(1.9469)	-	(1.3929)
Health Expenditure * Quality of Governance	-	0.0401**	-	0.0029	-	-0.0094*
	-	(0.0186)	-	(0.0082)	-	(0.0052)
Electricity Generation * Quality of Governance * Food Vulnerability	-	0.1334	-	-2.6382***	-	-0.4906*
	-	(0.8057)	-	(0.4348)	-	(0.2799)
Financial Access * Quality of Governance * Food Vulnerability	-	-3.5245	-	12.4820***	-	2.0632*
	-	(2.8869)	-	(1.7469)	-	(1.1618)
Rural Population Growth * Quality of Governance * Food Vulnerability	-	1.2231	-	-0.9554***	-	-1.1647***

	-	(0.9633)	-	(0.3690)	-	(0.2702)
Income Inequality * Quality of Governance * Food Vulnerability	-	7.5807	-	28.0403***	-	1.1835
	-	(10.0180)	-	(4.8595)	-	(3.3440)
Health Expenditure * Quality of Governance * Food Vulnerability	-	-0.0785*	-	0.0184	-	0.0254**
	-	(0.0435)	-	(0.0180)	-	(0.0122)
Constant	5.7596***	5.0898**	5.2549***	0.2857	5.0638***	5.3490***
	(0.2489)	(2.0598)	(0.2411)	(1.4690)	(0.2377)	(1.5883)
Wald-chi2	244.7	1417	234.9	4174	99.45	1369
Observations	115	115	115	115	115	115
Heteroskedasticity controlled	Yes	Yes	Yes	Yes	Yes	Yes
Autocorrelation controlled	Yes	Yes	Yes	Yes	Yes	Yes
Cross-sectional correlation controlled	Yes	Yes	Yes	Yes	Yes	Yes
Marginal Impact						
Electricity Generation	-0.0416***	-0.0951*	-0.0223	-0.0514**	-0.0244	-0.0781*
	(0.0144)	(0.0489)	(0.0154)	(0.0238)	(0.0149)	(0.0424)
Financial Access	0.0391	0.4356***	0.0735***	0.6122***	0.0668**	0.4129***
	(0.0280)	(0.0729)	(0.0282)	(0.0509)	(0.0276)	(0.0839)
Rural Population Growth	-0.0217***	-0.0554***	-0.0315***	-0.0599***	-0.0064	-0.0557***
	(0.0049)	(0.0135)	(0.0065)	(0.0099)	(0.0052)	(0.0168)
Income Inequality	-0.1632	0.4216**	-0.0700	0.2421*	0.2598*	0.4463*
	(0.1069)	(0.1689)	(0.1190)	(0.1404)	(0.1344)	(0.2316)
Health Expenditure	0.0008	0.0035***	0.0015**	0.0027***	0.0005	0.0009
	(0.0006)	(0.0012)	(0.0008)	(0.0007)	(0.0006)	(0.0010)

Note:
Standard errors in parentheses ()
*** p<0.01, ** p<0.05, * p<0.1 by Z-test by Z-test

Table S11: Long Run Coefficient Estimation using Generalized Least Square with Health Vulnerability for SDG 7.1.1 – With Control of Corruption, Governance Effectiveness, and Political Stability, Related to Figure 3

Variables	Model 1	Model 2	Model 1	Model 2	Model 1	Model 2
	Control of Corruption		Governance Effectiveness		Political Stability	
Electricity Generation	-0.0415**	0.0209	-0.0232	-0.3908***	-0.0475***	0.2005**
	(0.0168)	(0.1124)	(0.0159)	(0.1059)	(0.0170)	(0.0995)
Financial Access	0.1116***	0.8077**	0.0924***	-0.1680	0.0982***	0.4393
	(0.0238)	(0.3413)	(0.0199)	(0.2280)	(0.0257)	(0.3462)
Rural Population Growth	-0.0356***	0.1374**	-0.0384***	0.0974*	-0.0258***	-0.1479**
	(0.0053)	(0.0691)	(0.0057)	(0.0520)	(0.0054)	(0.0721)
Income Inequality	0.0716	-5.2066***	0.0387	-3.7271***	0.0252	0.0546
	(0.1265)	(1.7377)	(0.1215)	(0.7069)	(0.1277)	(1.5729)
Health Expenditure	0.0027***	0.0093	0.0029***	-0.0028	0.0025***	0.0432***
	(0.0008)	(0.0067)	(0.0007)	(0.0045)	(0.0007)	(0.0074)
Health Vulnerability	-0.5820***	-3.7555	-0.5407***	-12.0928***	-0.7057***	8.1252***
	(0.1643)	(3.0075)	(0.1530)	(2.3760)	(0.1597)	(2.7763)
Quality of Governance	-0.0320**	0.9162	-0.0474***	0.7177	-0.0075	1.6546*
	(0.0151)	(0.7285)	(0.0145)	(0.7420)	(0.0113)	(0.9032)
Electricity Generation * Health Vulnerability	-	-0.2736	-	0.8481***	-	-0.8836***
	-	(0.2458)	-	(0.2408)	-	(0.2747)
Financial Access * Health Vulnerability	-	-1.4098*	-	0.6446	-	-1.0949
	-	(0.7808)	-	(0.5357)	-	(0.8417)
Rural Population Growth * Health Vulnerability	-	-0.3437**	-	-0.2171*	-	0.3087*
	-	(0.1615)	-	(0.1197)	-	(0.1645)
Income Inequality * Health Vulnerability	-	10.5315***	-	7.2286***	-	1.3184
	-	(3.5578)	-	(1.4826)	-	(3.3115)
Health Expenditure * Health Vulnerability	-	-0.0190	-	0.0111	-	-0.0873***
	-	(0.0123)	-	(0.0097)	-	(0.0155)
Electricity Generation * Quality of Governance	-	0.1034	-	0.4971***	-	-0.4608***
	-	(0.2047)	-	(0.1782)	-	(0.1212)
Financial Access * Quality of Governance	-	2.2436***	-	-0.4964	-	2.1696***
	-	(0.6386)	-	(0.4933)	-	(0.4854)
Rural Population Growth * Quality of Governance	-	-0.2132	-	0.0457	-	-0.6693***
	-	(0.1511)	-	(0.1383)	-	(0.1376)
Income Inequality * Quality of Governance	-	-4.7436**	-	-8.4915***	-	0.8944
	-	(2.3949)	-	(2.2565)	-	(1.7074)
Health Expenditure * Quality of Governance	-	0.0148*	-	0.0153	-	0.0459***
	-	(0.0083)	-	(0.0120)	-	(0.0092)
Electricity Generation * Quality of Governance * Health Vulnerability	-	-0.4541	-	-1.4261***	-	0.4488
	-	(0.4857)	-	(0.4765)	-	(0.3352)
Financial Access * Quality of Governance * Health Vulnerability	-	-5.4002***	-	1.5583	-	-5.9582***
	-	(1.6993)	-	(1.2220)	-	(1.2725)
Rural Population Growth * Quality of Governance * Health Vulnerability	-	0.5172	-	-0.2032	-	1.7155***

	-	(0.3938)	-	(0.3845)	-	(0.3211)
Income Inequality * Quality of Governance * Health Vulnerability	-	9.6029*	-	19.1990***	-	-0.2422
	-	(5.4920)	-	(5.3504)	-	(3.3691)
Health Expenditure * Quality of Governance * Health Vulnerability	-	-0.0279*	-	-0.0228	-	-0.0943***
	-	(0.0162)	-	(0.0211)	-	(0.0180)
Constant	4.9093***	7.2274***	4.7735***	10.2700***	5.0711***	1.9133
	(0.2353)	(1.6455)	(0.2242)	(1.1104)	(0.2326)	(1.3641)
Wald-chi2	247.0	1326	249.5	1684	132.2	2289
Observations	115	115	115	115	115	115
Heteroskedasticity controlled	Yes	Yes	Yes	Yes	Yes	Yes
Autocorrelation controlled	Yes	Yes	Yes	Yes	Yes	Yes
Cross-sectional correlation controlled	Yes	Yes	Yes	Yes	Yes	Yes
Marginal Impact						
Electricity Generation	-0.0415**	-0.0733**	-0.0232	-0.0048	-0.0475***	-0.0776**
	(0.0168)	(0.0304)	(0.0159)	(0.0227)	(0.0170)	(0.0304)
Financial Access	0.1116***	0.1906***	0.0924***	0.1415***	0.0982***	0.3624***
	(0.0238)	(0.0524)	(0.0199)	(0.0388)	(0.0257)	(0.0606)
Rural Population Growth	-0.0356***	-0.0271**	-0.0384***	-0.0047	-0.0258***	-0.1038***
	(0.0053)	(0.0110)	(0.0057)	(0.0100)	(0.0054)	(0.0157)
Income Inequality	0.0716	-0.1083	0.0387	-0.2203	0.0252	0.2361
	(0.1265)	(0.1803)	(0.1215)	(0.1450)	(0.1277)	(0.1539)
Health Expenditure	0.0027***	-0.0005	0.0029***	0.0022**	0.0025***	0.0029***
	(0.0008)	(0.0011)	(0.0007)	(0.0009)	(0.0007)	(0.0009)

Note:
Standard errors in parentheses ()
*** p<0.01, ** p<0.05, * p<0.1 by Z-test by Z-test

Table S12: Long Run Coefficient Estimation using Generalized Least Square with Health Vulnerability for SDG 7.1.1 – With Regulatory Quality, Rule of Law, and Voice and Accountability, Related to Figure 3

Variables	Model 1	Model 2	Model 1	Model 2	Model 1	Model 2
	Regulatory Quality		Rule of Law		Voice and Accountability	
Electricity Generation	-0.0460***	0.0917	-0.0417**	0.2089	-0.0199	-0.0387
	(0.0165)	(0.1156)	(0.0190)	(0.1479)	(0.0198)	(0.1191)
Financial Access	0.0976***	-0.0247	0.1317***	-0.7903**	0.0642**	0.1031
	(0.0265)	(0.3058)	(0.0272)	(0.3822)	(0.0281)	(0.2578)
Rural Population Growth	-0.0267***	0.1044	-0.0442***	0.0444	-0.0036	-0.0238
	(0.0053)	(0.0680)	(0.0061)	(0.0640)	(0.0060)	(0.0745)
Income Inequality	-0.0199	-3.2955***	-0.0305	-0.2592	0.3487**	-1.9433**
	(0.1283)	(1.1816)	(0.1356)	(1.1193)	(0.1607)	(0.8998)
Health Expenditure	0.0024***	0.0197***	0.0017*	0.0328***	0.0003	0.0072
	(0.0008)	(0.0061)	(0.0009)	(0.0067)	(0.0008)	(0.0045)
Health Vulnerability	-0.7145***	-1.7459	-0.4516**	3.1539	-0.3493**	-4.7995**
	(0.1631)	(3.0358)	(0.1784)	(2.8513)	(0.1769)	(2.3403)
Quality of Governance	-0.0105	0.1371	-0.0667***	-1.6068*	-0.0483***	-0.1982
	(0.0186)	(1.5101)	(0.0200)	(0.9417)	(0.0152)	(0.3447)
Electricity Generation * Health Vulnerability	-	-0.3752	-	-0.5050	-	0.1519
	-	(0.2756)	-	(0.3201)	-	(0.2766)
Financial Access * Health Vulnerability	-	0.3200	-	2.2580**	-	0.0667
	-	(0.7722)	-	(0.8927)	-	(0.6205)
Rural Population Growth * Health Vulnerability	-	-0.2633*	-	-0.1330	-	0.0901
	-	(0.1579)	-	(0.1509)	-	(0.1700)
Income Inequality * Health Vulnerability	-	8.2870***	-	2.3091	-	5.8059***
	-	(2.5936)	-	(2.0965)	-	(1.9059)
Health Expenditure * Health Vulnerability	-	-0.0334**	-	-0.0535***	-	-0.0058
	-	(0.0140)	-	(0.0121)	-	(0.0097)
Electricity Generation * Quality of Governance	-	-0.0601	-	0.4558***	-	-0.0740
	-	(0.1716)	-	(0.1680)	-	(0.0806)
Financial Access * Quality of Governance	-	2.0724***	-	-1.1060*	-	0.4282
	-	(0.6641)	-	(0.6512)	-	(0.2697)
Rural Population Growth * Quality of Governance	-	-0.2971	-	-0.0337	-	0.1103*
	-	(0.1940)	-	(0.0990)	-	(0.0563)
Income Inequality * Quality of Governance	-	-2.7361	-	-4.8096***	-	0.9683
	-	(3.3101)	-	(1.7331)	-	(1.1224)
Health Expenditure * Quality of Governance	-	0.0415***	-	0.0327***	-	0.0131***
	-	(0.0120)	-	(0.0087)	-	(0.0034)
Electricity Generation * Quality of Governance * Health Vulnerability	-	-0.0880	-	-1.0748***	-	0.1540
	-	(0.5831)	-	(0.3393)	-	(0.1882)
Financial Access * Quality of Governance * Health Vulnerability	-	-4.9680***	-	3.0984*	-	-0.7930
	-	(1.7184)	-	(1.6959)	-	(0.6006)
Rural Population Growth * Quality of Governance * Health Vulnerability	-	0.8304*	-	0.1118	-	-0.2988**

	-	(0.4403)	-	(0.2566)	-	(0.1339)
Income Inequality * Quality of Governance * Health Vulnerability	-	7.6630	-	15.4732***	-	-2.2263
	-	(7.2985)	-	(4.1129)	-	(2.4905)
Health Expenditure * Quality of Governance * Health Vulnerability	-	-0.0738***	-	-0.0446***	-	-0.0215***
	-	(0.0267)	-	(0.0169)	-	(0.0067)
Constant	5.0988***	5.4888***	4.9178***	2.4247	4.5987***	5.8817***
	(0.2279)	(1.4586)	(0.2606)	(1.5333)	(0.2367)	(1.1460)
Wald-chi2	124.2	1343	202.5	859.8	35.83	655.7
Observations	115	115	115	115	115	115
Heteroskedasticity controlled	Yes	Yes	Yes	Yes	Yes	Yes
Autocorrelation controlled	Yes	Yes	Yes	Yes	Yes	Yes
Cross-sectional correlation controlled	Yes	Yes	Yes	Yes	Yes	Yes
Marginal Impact						
Electricity Generation	-0.0460***	-0.0716*	-0.0417**	-0.0404	-0.0199	0.0383
	(0.0165)	(0.0385)	(0.0190)	(0.0325)	(0.0198)	(0.0472)
Financial Access	0.0976***	0.1615**	0.1317***	0.2418***	0.0642**	0.0923
	(0.0265)	(0.0680)	(0.0272)	(0.0602)	(0.0281)	(0.0654)
Rural Population Growth	-0.0267***	-0.0299**	-0.0442***	-0.0201	-0.0036	0.0128
	(0.0053)	(0.0116)	(0.0061)	(0.0128)	(0.0060)	(0.0108)
Income Inequality	-0.0199	0.4551***	-0.0305	0.5130***	0.3487**	0.6909***
	(0.1283)	(0.1726)	(0.1356)	(0.1932)	(0.1607)	(0.2071)
Health Expenditure	0.0024***	0.0035***	0.0017*	0.0033***	0.0003	0.0030**
	(0.0008)	(0.0011)	(0.0009)	(0.0012)	(0.0008)	(0.0013)

Note:
Standard errors in parentheses ()
*** p<0.01, ** p<0.05, * p<0.1 by Z-test by Z-test

Table S13: Long Run Coefficient Estimation using Generalized Least Square with Infrastructure Vulnerability for SDG 7.1.1 – With Control of Corruption, Governance Effectiveness, and Political Stability, Related to Figure 3

Variables	Model 1	Model 2	Model 1	Model 2	Model 1	Model 2
	Control of Corruption		Governance Effectiveness		Political Stability	
Electricity Generation	-0.0256	-0.1285*	0.0058	-0.2762***	-0.0194	0.0825
	(0.0191)	(0.0737)	(0.0195)	(0.0583)	(0.0211)	(0.0599)
Financial Access	0.1933***	0.0798	0.1575***	-0.1323	0.1992***	0.2130
	(0.0190)	(0.2015)	(0.0202)	(0.1211)	(0.0210)	(0.1822)
Rural Population Growth	-0.0475***	0.1443**	-0.0488***	0.0842**	-0.0510***	0.2097***
	(0.0085)	(0.0606)	(0.0084)	(0.0408)	(0.0083)	(0.0690)
Income Inequality	-0.0588	-2.4475***	-0.1028	-2.9786***	-0.0783	-0.9386
	(0.1407)	(0.7182)	(0.1436)	(0.4765)	(0.1485)	(0.7051)
Health Expenditure	0.0022***	-0.0013	0.0029***	-0.0003	0.0028***	-0.0056
	(0.0008)	(0.0041)	(0.0008)	(0.0029)	(0.0008)	(0.0046)
Infrastructure Vulnerability	-0.1234	-15.2446***	-0.1392	-25.2821***	-0.4104**	2.7315
	(0.1889)	(4.8226)	(0.1913)	(4.1267)	(0.1816)	(5.2225)
Quality of Governance	-0.0622***	0.5292	-0.0596***	3.9858***	-0.0074	-0.3255
	(0.0154)	(0.6898)	(0.0154)	(1.1670)	(0.0116)	(0.8200)
Electricity Generation * Infrastructure Vulnerability	-	0.5360	-	1.6780***	-	-1.1358**
	-	(0.4483)	-	(0.3825)	-	(0.4522)
Financial Access * Infrastructure Vulnerability	-	0.4320	-	0.9846	-	0.8959
	-	(1.3318)	-	(0.7838)	-	(1.3237)
Rural Population Growth * Infrastructure Vulnerability	-	-0.9364**	-	-0.4871*	-	-1.5654***
	-	(0.4097)	-	(0.2766)	-	(0.4550)
Income Inequality * Infrastructure Vulnerability	-	14.1642***	-	16.8143***	-	4.5568
	-	(4.2587)	-	(2.4387)	-	(4.4437)
Health Expenditure * Infrastructure Vulnerability	-	0.0238	-	0.0096	-	0.0447
	-	(0.0218)	-	(0.0170)	-	(0.0275)
Electricity Generation * Quality of Governance	-	0.0910	-	-0.0156	-	0.0040
	-	(0.0902)	-	(0.0912)	-	(0.0580)
Financial Access * Quality of Governance	-	-0.1364	-	-0.8317***	-	0.4356**
	-	(0.2421)	-	(0.2986)	-	(0.1959)
Rural Population Growth * Quality of Governance	-	0.1224	-	0.6102***	-	0.0024
	-	(0.0792)	-	(0.1389)	-	(0.1161)
Income Inequality * Quality of Governance	-	-1.7653*	-	-4.4187***	-	0.5207
	-	(0.9409)	-	(0.9600)	-	(0.7619)
Health Expenditure * Quality of Governance	-	-0.0029	-	-0.0071	-	-0.0095
	-	(0.0048)	-	(0.0085)	-	(0.0059)
Electricity Generation * Quality of Governance * Infrastructure Vulnerability	-	-0.9794*	-	-3.0527***	-	0.5657
	-	(0.5592)	-	(0.5780)	-	(0.4872)
Financial Access * Quality of Governance * Infrastructure Vulnerability	-	2.8314	-	7.4932***	-	-1.5303
	-	(1.8922)	-	(2.2160)	-	(1.6226)
Rural Population Growth * Quality of Governance * Infrastructure Vulnerability	-	-0.6282	-	-4.1165***	-	0.4546

	-	(0.5732)	-	(0.8742)	-	(0.7421)
Income Inequality * Quality of Governance * Infrastructure Vulnerability	-	7.0463	-	23.9999***	-	-7.7459*
	-	(6.9735)	-	(5.8376)	-	(4.4270)
Health Expenditure * Quality of Governance * Infrastructure Vulnerability	-	0.0541**	-	0.0648*	-	0.0526
	-	(0.0257)	-	(0.0387)	-	(0.0326)
Constant	4.5871***	7.2910***	4.3732***	8.8431***	4.5801***	4.7359***
	(0.2518)	(0.9267)	(0.2606)	(0.7686)	(0.2651)	(0.9213)
Wald-chi2	344.4	770.3	275.7	2346	213.1	1249
Observations	115	115	115	115	115	115
Heteroskedasticity controlled	Yes	Yes	Yes	Yes	Yes	Yes
Autocorrelation controlled	Yes	Yes	Yes	Yes	Yes	Yes
Cross-sectional correlation controlled	Yes	Yes	Yes	Yes	Yes	Yes
Marginal Impact						
Electricity Generation	-0.0256	-0.0258	0.0058	-0.0224	-0.0194	-0.1551***
	(0.0191)	(0.0256)	(0.0195)	(0.0265)	(0.0211)	(0.0319)
Financial Access	0.1933***	0.0546*	0.1575***	0.0681**	0.1992***	0.2594***
	(0.0190)	(0.0293)	(0.0202)	(0.0320)	(0.0210)	(0.0390)
Rural Population Growth	-0.0475***	-0.0164	-0.0488***	-0.0190*	-0.0510***	-0.0850***
	(0.0085)	(0.0108)	(0.0084)	(0.0100)	(0.0083)	(0.0142)
Income Inequality	-0.0588	0.0765	-0.1028	-0.1388	-0.0783	0.2405
	(0.1407)	(0.1308)	(0.1436)	(0.1550)	(0.1485)	(0.1609)
Health Expenditure	0.0022***	0.0008	0.0029***	0.0016*	0.0028***	0.0018**
	(0.0008)	(0.0009)	(0.0008)	(0.0010)	(0.0008)	(0.0009)

Note:
Standard errors in parentheses ()
*** p<0.01, ** p<0.05, * p<0.1 by Z-test by Z-test

Table S14: Long Run Coefficient Estimation using Generalized Least Square with Infrastructure Vulnerability for SDG 7.1.1 – With Regulatory Quality, Rule of Law, and Voice and Accountability, Related to Figure 3

Variables	Model 1	Model 2	Model 1	Model 2	Model 1	Model 2
	Regulatory Quality		Rule of Law		Voice and Accountability	
Electricity Generation	-0.0174	-0.0697	-0.0193	-0.0821	-0.0193	-0.0158
	(0.0216)	(0.0573)	(0.0189)	(0.0969)	(0.0216)	(0.0732)
Financial Access	0.1964***	-0.1716	0.1709***	-0.2791	0.1487***	-0.5638***
	(0.0252)	(0.1398)	(0.0202)	(0.2041)	(0.0281)	(0.1027)
Rural Population Growth	-0.0370***	0.1692***	-0.0471***	0.2947***	-0.0159**	0.1678***
	(0.0096)	(0.0434)	(0.0086)	(0.0515)	(0.0081)	(0.0324)
Income Inequality	-0.1389	-2.4971***	-0.0706	0.1597	0.2839*	-0.7501*
	(0.1614)	(0.5868)	(0.1407)	(0.9214)	(0.1664)	(0.3856)
Health Expenditure	0.0024***	-0.0014	0.0015*	0.0105**	0.0009	0.0027
	(0.0009)	(0.0033)	(0.0009)	(0.0053)	(0.0008)	(0.0023)
Infrastructure Vulnerability	-0.1704	-14.8302***	-0.1031	-0.8654	-0.6853***	-2.3681
	(0.2101)	(5.2003)	(0.2086)	(5.6017)	(0.1817)	(3.9801)
Quality of Governance	-0.0374*	-0.0966	-0.0778***	0.7833	-0.0754***	0.4999*
	(0.0196)	(1.1118)	(0.0209)	(1.1310)	(0.0152)	(0.2776)
Electricity Generation * Infrastructure Vulnerability	-	0.0907	-	-0.4138	-	-0.9074**
	-	(0.4654)	-	(0.4886)	-	(0.4059)
Financial Access * Infrastructure Vulnerability	-	1.7287*	-	2.8985**	-	4.5973***
	-	(0.9761)	-	(1.3171)	-	(0.7007)
Rural Population Growth * Infrastructure Vulnerability	-	-1.2505***	-	-1.9903***	-	-1.1728***
	-	(0.3099)	-	(0.3408)	-	(0.2455)
Income Inequality * Infrastructure Vulnerability	-	17.0825***	-	2.5921	-	8.6491***
	-	(3.5173)	-	(4.3642)	-	(2.3235)
Health Expenditure * Infrastructure Vulnerability	-	0.0310	-	-0.0317	-	0.0009
	-	(0.0204)	-	(0.0243)	-	(0.0126)
Electricity Generation * Quality of Governance	-	0.1220	-	-0.0772	-	-0.0233
	-	(0.1110)	-	(0.0961)	-	(0.0349)
Financial Access * Quality of Governance	-	0.0310	-	-0.4680**	-	-0.4190***
	-	(0.3738)	-	(0.2347)	-	(0.1171)
Rural Population Growth * Quality of Governance	-	0.3950***	-	0.3311***	-	0.1363***
	-	(0.1264)	-	(0.0652)	-	(0.0293)
Income Inequality * Quality of Governance	-	-0.2453	-	0.0398	-	-0.1273
	-	(1.3504)	-	(1.0996)	-	(0.4675)
Health Expenditure * Quality of Governance	-	-0.0129*	-	0.0098	-	0.0051**
	-	(0.0068)	-	(0.0060)	-	(0.0025)
Electricity Generation * Quality of Governance * Infrastructure Vulnerability	-	-0.9371	-	-1.1130**	-	-0.4201*
	-	(0.7848)	-	(0.5389)	-	(0.2488)
Financial Access * Quality of Governance * Infrastructure Vulnerability	-	0.8156	-	5.1120***	-	2.8190***
	-	(2.6098)	-	(1.8180)	-	(0.7009)
Rural Population Growth * Quality of Governance * Infrastructure Vulnerability	-	-2.2715***	-	-1.9239***	-	-0.9810***

	-	(0.8752)	-	(0.4591)	-	(0.1958)
Income Inequality * Quality of Governance * Infrastructure Vulnerability	-	0.7166	-	7.9883	-	1.0203
	-	(9.3025)	-	(6.3992)	-	(2.7471)
Health Expenditure * Quality of Governance * Infrastructure Vulnerability	-	0.1262***	-	-0.0038	-	-0.0180
	-	(0.0329)	-	(0.0272)	-	(0.0136)
Constant	4.5975***	7.0332***	4.5670***	5.2238***	4.4819***	5.6140***
	(0.2775)	(0.8038)	(0.2459)	(1.3468)	(0.2301)	(0.7900)
Wald-chi2	173.6	1664	157.8	1080	92.20	2412
Observations	115	115	115	115	115	115
Heteroskedasticity controlled	Yes	Yes	Yes	Yes	Yes	Yes
Autocorrelation controlled	Yes	Yes	Yes	Yes	Yes	Yes
Cross-sectional correlation controlled	Yes	Yes	Yes	Yes	Yes	Yes
Marginal Impact						
Electricity Generation	-0.0174	-0.0487	-0.0193	-0.0899***	-0.0193	-0.1452***
	(0.0216)	(0.0377)	(0.0189)	(0.0313)	(0.0216)	(0.0321)
Financial Access	0.1964***	0.0831**	0.1709***	0.1532***	0.1487***	0.1842***
	(0.0252)	(0.0377)	(0.0202)	(0.0350)	(0.0281)	(0.0353)
Rural Population Growth	-0.0370***	-0.0255*	-0.0471***	-0.0562***	-0.0159**	-0.0224**
	(0.0096)	(0.0133)	(0.0086)	(0.0119)	(0.0081)	(0.0107)
Income Inequality	-0.1389	0.2395	-0.0706	0.3036*	0.2839*	0.6298***
	(0.1614)	(0.1667)	(0.1407)	(0.1628)	(0.1664)	(0.1391)
Health Expenditure	0.0024***	0.0023**	0.0015*	0.0027***	0.0009	0.0022***
	(0.0009)	(0.0011)	(0.0009)	(0.0010)	(0.0008)	(0.0009)

Note:
Standard errors in parentheses ()
*** p<0.01, ** p<0.05, * p<0.1 by Z-test by Z-test

Table S15: Long Run Coefficient Estimation using Generalized Least Square with Water Vulnerability for SDG 7.1.1 – With Control of Corruption, Governance Effectiveness, and Political Stability, Related to Figure 3

Variables	Model 1	Model 2	Model 1	Model 2	Model 1	Model 2
	Control of Corruption		Governance Effectiveness		Political Stability	
Electricity Generation	-0.0062	-0.0259	0.0192	-0.2472	0.0008	-0.0223
	(0.0180)	(0.1217)	(0.0154)	(0.1537)	(0.0199)	(0.1200)
Financial Access	0.1935***	0.3988	0.1576***	0.0553	0.2349***	1.5569***
	(0.0228)	(0.5054)	(0.0206)	(0.3118)	(0.0213)	(0.5233)
Rural Population Growth	-0.0482***	0.1300**	-0.0533***	0.1724***	-0.0440***	-0.0238
	(0.0065)	(0.0617)	(0.0061)	(0.0507)	(0.0061)	(0.0665)
Income Inequality	0.0316	-9.1722***	-0.0145	-3.9674***	0.0662	-0.6386
	(0.1503)	(1.3988)	(0.1420)	(0.7464)	(0.1480)	(1.6586)
Health Expenditure	0.0026***	0.0158***	0.0027***	-0.0054	0.0024***	0.0249***
	(0.0008)	(0.0059)	(0.0007)	(0.0043)	(0.0008)	(0.0079)
Water Vulnerability	-0.0778	-12.1647***	-0.0313	-13.9960***	-0.0899	2.1092
	(0.1369)	(3.1354)	(0.1506)	(4.4758)	(0.1426)	(4.2375)
Quality of Governance	-0.0503***	1.3030*	-0.0723***	-2.3211*	-0.0167	-0.5857
	(0.0153)	(0.7537)	(0.0156)	(1.2094)	(0.0106)	(1.0411)
Electricity Generation * Water Vulnerability	-	-0.3690	-	0.6515	-	0.1525
	-	(0.3709)	-	(0.4847)	-	(0.4241)
Financial Access * Water Vulnerability	-	-0.6979	-	0.5973	-	-4.3793***
	-	(1.5989)	-	(1.0307)	-	(1.6827)
Rural Population Growth * Water Vulnerability	-	-0.4946**	-	-0.6447***	-	0.0597
	-	(0.2148)	-	(0.1751)	-	(0.2362)
Income Inequality * Water Vulnerability	-	26.6272***	-	11.7515***	-	2.3786
	-	(4.0244)	-	(1.8456)	-	(4.7684)
Health Expenditure * Water Vulnerability	-	-0.0406**	-	0.0250**	-	-0.0643***
	-	(0.0159)	-	(0.0121)	-	(0.0226)
Electricity Generation * Quality of Governance	-	0.3504*	-	-0.0670	-	-0.3463***
	-	(0.1991)	-	(0.1651)	-	(0.1214)
Financial Access * Quality of Governance	-	0.4865	-	2.7399***	-	2.1488***
	-	(0.8288)	-	(0.8302)	-	(0.7401)
Rural Population Growth * Quality of Governance	-	-0.0097	-	-0.3625	-	-0.1912
	-	(0.1467)	-	(0.2256)	-	(0.1259)
Income Inequality * Quality of Governance	-	-8.8888***	-	-2.1086	-	2.0958
	-	(2.3710)	-	(2.2405)	-	(1.8070)
Health Expenditure * Quality of Governance	-	0.0273***	-	0.0538***	-	0.0191*
	-	(0.0099)	-	(0.0139)	-	(0.0102)
Electricity Generation * Quality of Governance * Water Vulnerability	-	-1.5384**	-	0.6756	-	1.3632**
	-	(0.7767)	-	(0.7126)	-	(0.5537)
Financial Access * Quality of Governance * Water Vulnerability	-	-1.0142	-	-8.5575***	-	-6.8362***
	-	(2.8201)	-	(2.9134)	-	(2.5253)
Rural Population Growth * Quality of Governance * Water Vulnerability	-	-0.0085	-	1.2963*	-	0.8447**

	-	(0.5063)	-	(0.6888)	-	(0.4100)
Income Inequality * Quality of Governance * Water Vulnerability	-	26.1297***	-	11.1943*	-	-7.2529
	-	(7.8104)	-	(6.4945)	-	(4.9144)
Health Expenditure * Quality of Governance * Water Vulnerability	-	-0.0730**	-	-0.1460***	-	-0.0545**
	-	(0.0298)	-	(0.0390)	-	(0.0273)
Constant	4.3681***	9.9015***	4.2013***	9.2603***	4.2851***	3.3531**
	(0.2540)	(1.4019)	(0.2454)	(1.5899)	(0.2685)	(1.6521)
Wald-chi2	343.2	1697	331.0	2487	242.0	847.8
Observations	115	115	115	115	115	115
Heteroskedasticity controlled	Yes	Yes	Yes	Yes	Yes	Yes
Autocorrelation controlled	Yes	Yes	Yes	Yes	Yes	Yes
Cross-sectional correlation controlled	Yes	Yes	Yes	Yes	Yes	Yes
Marginal Impact						
Electricity Generation	-0.0062	-0.0910***	0.0192	-0.0106	0.0008	-0.0715**
	(0.0180)	(0.0253)	(0.0154)	(0.0324)	(0.0199)	(0.0361)
Financial Access	0.1935***	0.1045***	0.1576***	0.1901***	0.2349***	0.2856***
	(0.0228)	(0.0366)	(0.0206)	(0.0573)	(0.0213)	(0.0757)
Rural Population Growth	-0.0482***	-0.0418***	-0.0533***	-0.0461***	-0.0440***	-0.0797***
	(0.0065)	(0.0116)	(0.0061)	(0.0153)	(0.0061)	(0.0172)
Income Inequality	0.0316	0.2163	-0.0145	0.3056*	0.0662	0.6183***
	(0.1503)	(0.1556)	(0.1420)	(0.1626)	(0.1480)	(0.1954)
Health Expenditure	0.0026***	0.0007	0.0027***	0.0021**	0.0024***	0.0032***
	(0.0008)	(0.0009)	(0.0007)	(0.0009)	(0.0008)	(0.0011)

Note:
Standard errors in parentheses ()
*** p<0.01, ** p<0.05, * p<0.1 by Z-test by Z-test

Table S16: Long Run Coefficient Estimation using Generalized Least Square with Water Vulnerability for SDG 7.1.1 – With Regulatory Quality, Rule of Law, and Voice and Accountability, Related to Figure 3

Variables	Model 1	Model 2	Model 1	Model 2	Model 1	Model 2
		Regulatory Quality		Rule of Law		Voice and Accountability
Electricity Generation	-0.0109	-0.0381	0.0049	0.3668**	-0.0121	0.2672*
	(0.0194)	(0.1258)	(0.0186)	(0.1704)	(0.0179)	(0.1428)
Financial Access	0.2075***	0.5420	0.1646***	-1.3425***	0.1341***	-0.9973**
	(0.0295)	(0.4012)	(0.0290)	(0.4318)	(0.0274)	(0.3970)
Rural Population Growth	-0.0395***	0.0596	-0.0477***	0.2730***	-0.0114*	0.1778***
	(0.0069)	(0.0615)	(0.0081)	(0.0482)	(0.0063)	(0.0526)
Income Inequality	-0.1588	-5.5821***	0.0822	-1.7202*	0.3696**	-1.8564***
	(0.1595)	(1.0837)	(0.1619)	(0.9043)	(0.1583)	(0.7071)
Health Expenditure	0.0019**	0.0056	0.0009	0.0225***	0.0009	0.0066*
	(0.0008)	(0.0065)	(0.0009)	(0.0053)	(0.0008)	(0.0038)
Water Vulnerability	-0.1530	-11.6641***	0.0388	5.7839	-0.3853**	1.5221
	(0.1713)	(4.5143)	(0.1623)	(3.8417)	(0.1659)	(3.4075)
Quality of Governance	-0.0407**	-3.2050***	-0.0800***	-0.8634	-0.0817***	-0.4818
	(0.0178)	(1.2216)	(0.0231)	(0.8352)	(0.0179)	(0.2980)
Electricity Generation * Water Vulnerability	-	0.1275	-	-1.3517***	-	-0.8649*
	-	(0.4080)	-	(0.4459)	-	(0.4425)
Financial Access * Water Vulnerability	-	-1.2543	-	4.9626***	-	4.3065***
	-	(1.3231)	-	(1.3628)	-	(1.3511)
Rural Population Growth * Water Vulnerability	-	-0.3197	-	-0.8756***	-	-0.6562***
	-	(0.2073)	-	(0.1627)	-	(0.1818)
Income Inequality * Water Vulnerability	-	17.7311***	-	7.6618***	-	6.3874***
	-	(3.1672)	-	(2.2809)	-	(1.9036)
Health Expenditure * Water Vulnerability	-	-0.0025	-	-0.0523***	-	-0.0110
	-	(0.0200)	-	(0.0128)	-	(0.0100)
Electricity Generation * Quality of Governance	-	0.0379	-	0.4425***	-	0.1153*
	-	(0.2291)	-	(0.1444)	-	(0.0640)
Financial Access * Quality of Governance	-	3.0338**	-	-2.5815***	-	-0.3064
	-	(1.2638)	-	(0.7594)	-	(0.3232)
Rural Population Growth * Quality of Governance	-	0.0698	-	0.2191**	-	0.1324***
	-	(0.1656)	-	(0.1029)	-	(0.0483)
Income Inequality * Quality of Governance	-	0.9803	-	-4.3534**	-	-0.8945
	-	(2.8130)	-	(1.7363)	-	(0.8163)
Health Expenditure * Quality of Governance	-	0.0103	-	0.0197**	-	0.0065*
	-	(0.0141)	-	(0.0090)	-	(0.0040)
Electricity Generation * Quality of Governance * Water Vulnerability	-	0.7295	-	-1.7812***	-	-0.3088
	-	(0.8862)	-	(0.5142)	-	(0.2171)
Financial Access * Quality of Governance * Water Vulnerability	-	-8.7654**	-	9.6293***	-	1.8192*
	-	(4.2603)	-	(2.6345)	-	(1.0419)
Rural Population Growth * Quality of Governance * Water Vulnerability	-	0.0413	-	-0.5590*	-	-0.4476***

	-	(0.5576)	-	(0.3256)	-	(0.1437)
Income Inequality * Quality of Governance * Water Vulnerability	-	0.3205	-	20.5794***	-	3.1581
	-	(8.7750)	-	(5.3312)	-	(2.3358)
Health Expenditure * Quality of Governance * Water Vulnerability	-	-0.0088	-	-0.0305	-	-0.0083
	-	(0.0436)	-	(0.0261)	-	(0.0103)
Constant	4.5861***	7.9900***	4.2678***	2.4298	4.4099***	3.7839***
	(0.2777)	(1.5507)	(0.2533)	(1.6861)	(0.2254)	(1.3174)
Wald-chi2	231.8	1227	114.4	1729	78.67	1645
Observations	115	115	115	115	115	115
Heteroskedasticity controlled	Yes	Yes	Yes	Yes	Yes	Yes
Autocorrelation controlled	Yes	Yes	Yes	Yes	Yes	Yes
Cross-sectional correlation controlled	Yes	Yes	Yes	Yes	Yes	Yes
Marginal Impact						
Electricity Generation	-0.0109	-0.0337	0.0049	-0.0875**	-0.0121	-0.0420
	(0.0194)	(0.0367)	(0.0186)	(0.0405)	(0.0179)	(0.0448)
Financial Access	0.2075***	0.1985***	0.1646***	0.3330***	0.1341***	0.4670***
	(0.0295)	(0.0585)	(0.0290)	(0.0723)	(0.0274)	(0.0828)
Rural Population Growth	-0.0395***	-0.0642***	-0.0477***	-0.0536***	-0.0114*	-0.0504***
	(0.0069)	(0.0155)	(0.0081)	(0.0124)	(0.0063)	(0.0139)
Income Inequality	-0.1588	0.6098***	0.0822	0.4758***	0.3696**	0.3718*
	(0.1595)	(0.1977)	(0.1619)	(0.1390)	(0.1583)	(0.2067)
Health Expenditure	0.0019**	0.0040***	0.0009	0.0009	0.0009	0.0019*
	(0.0008)	(0.0012)	(0.0009)	(0.0008)	(0.0008)	(0.0010)

Note:
Standard errors in parentheses ()
*** p<0.01, ** p<0.05, * p<0.1 by Z-test by Z-test

Table S17: Long Run Coefficient Estimation using Generalized Least Square with Ecosystem Vulnerability for SDG 7.1.2 – With Control of Corruption, Governance Effectiveness, and Political Stability, Related to Figure 3

Variables	Model 1	Model 2	Model 1	Model 2	Model 1	Model 2
	Control of Corruption		Governance Effectiveness		Political Stability	
Renewable Electricity Generation	0.0319***	-0.2865**	0.0245***	-0.8025***	0.0281***	-0.5283***
	(0.0038)	(0.1333)	(0.0035)	(0.1350)	(0.0065)	(0.1790)
Financial Access	0.1438***	-3.2483**	0.1820***	-2.3645***	0.0851*	-2.4896**
	(0.0285)	(1.3107)	(0.0279)	(0.8904)	(0.0487)	(0.9912)
Policy Stringency	0.0010	0.0340	0.0058	0.1816	0.0120	-0.2108
	(0.0050)	(0.1439)	(0.0055)	(0.1398)	(0.0074)	(0.1868)
Per Capita Income	0.2980***	-0.5988*	0.2839***	-1.8515***	0.3290***	-0.8703***
	(0.0154)	(0.3282)	(0.0164)	(0.3784)	(0.0257)	(0.3345)
Urban Population Growth	-0.0094	-0.1905	-0.0115*	-0.1944	-0.0025	-0.1086
	(0.0058)	(0.1787)	(0.0068)	(0.1885)	(0.0082)	(0.1938)
Ecosystem Vulnerability	-1.9989***	-29.6548***	-1.7266***	-68.5158***	-2.3182***	-37.9103***
	(0.2230)	(7.9829)	(0.3981)	(8.1670)	(0.3442)	(8.8899)
Quality of Governance	-0.0128	-2.3267**	-0.0139	0.4031	0.0178*	3.3908***
	(0.0095)	(0.9546)	(0.0093)	(1.9082)	(0.0093)	(1.1666)
Renewable Electricity Generation * Ecosystem Vulnerability	-	0.5974**	-	1.6787***	-	1.0442***
	-	(0.2728)	-	(0.2794)	-	(0.3619)
Financial Access * Ecosystem Vulnerability	-	7.4636***	-	6.2749***	-	6.0999***
	-	(2.7498)	-	(1.8632)	-	(2.1468)
Policy Stringency * Ecosystem Vulnerability	-	-0.0981	-	-0.5317*	-	0.4272
	-	(0.3039)	-	(0.2905)	-	(0.3938)
Per Capita Income * Ecosystem Vulnerability	-	1.4819**	-	4.2875***	-	1.7521**
	-	(0.6709)	-	(0.7862)	-	(0.7013)
Urban Population Growth * Ecosystem Vulnerability	-	0.3878	-	0.4334	-	0.1977
	-	(0.3738)	-	(0.3890)	-	(0.4087)
Renewable Electricity Generation * Quality of Governance	-	0.4266**	-	0.6455**	-	-0.6779*
	-	(0.2038)	-	(0.2840)	-	(0.3490)
Financial Access * Quality of Governance	-	-0.4543	-	10.2607***	-	-1.2112
	-	(1.5920)	-	(2.0932)	-	(1.2002)
Policy Stringency * Quality of Governance	-	0.4304	-	-2.8594***	-	0.1560
	-	(0.3039)	-	(0.4634)	-	(0.2724)
Per Capita Income * Quality of Governance	-	-0.1473	-	-0.8922***	-	0.6052*
	-	(0.1952)	-	(0.1996)	-	(0.3531)
Urban Population Growth * Quality of Governance	-	-0.9021***	-	0.9765**	-	-0.1787
	-	(0.2681)	-	(0.4578)	-	(0.2513)
Renewable Electricity Generation * Quality of Governance * Ecosystem Vulnerability	-	-0.8121*	-	-1.4732***	-	1.2792*
	-	(0.4199)	-	(0.5568)	-	(0.7044)
Financial Access * Quality of Governance * Ecosystem Vulnerability	-	0.8461	-	-22.9370***	-	3.0318
	-	(3.5371)	-	(4.6798)	-	(2.7799)
Policy Stringency * Quality of Governance * Ecosystem Vulnerability	-	-0.7930	-	6.3714***	-	-0.2457

	-	(0.6632)	-	(0.9400)	-	(0.5575)
Per Capita Income * Quality of Governance * Ecosystem Vulnerability	-	0.6415	-	1.8893***	-	-1.8154**
	-	(0.4023)	-	(0.5529)	-	(0.8298)
Urban Population Growth * Quality of Governance * Ecosystem Vulnerability	-	2.0521***	-	-2.1175**	-	0.4071
	-	(0.5523)	-	(0.9589)	-	(0.5335)
Constant	1.7341***	17.3372***	1.8171***	34.8860***	1.6264***	23.0523***
	(0.2367)	(3.9035)	(0.3326)	(3.9960)	(0.3869)	(4.3800)
Wald-chi2	2775	3456	2869	8111	927.3	3691
Observations	115	115	115	115	115	115
Heteroskedasticity controlled	Yes	Yes	Yes	Yes	Yes	Yes
Autocorrelation controlled	Yes	Yes	Yes	Yes	Yes	Yes
Cross-sectional correlation controlled	Yes	Yes	Yes	Yes	Yes	Yes
Marginal Impact						
Renewable Electricity Generation	0.0319***	-0.0124*	0.0245***	0.0014	0.0281***	0.0030
	(0.0038)	(0.0068)	(0.0035)	(0.0088)	(0.0065)	(0.0088)
Financial Access	0.1438***	0.3909***	0.1820***	0.5190***	0.0851*	0.3067***
	(0.0285)	(0.0646)	(0.0279)	(0.0724)	(0.0487)	(0.0801)
Policy Stringency	0.0010	-0.0337***	0.0058	-0.0318***	0.0120	-0.0228*
	(0.0050)	(0.0111)	(0.0055)	(0.0113)	(0.0074)	(0.0131)
Per Capita Income	0.2980***	0.0658*	0.2839***	0.2414***	0.3290***	0.1433***
	(0.0154)	(0.0356)	(0.0164)	(0.0340)	(0.0257)	(0.0426)
Urban Population Growth	-0.0094	-0.0245***	-0.0115*	0.0007	-0.0025	-0.0255**
	(0.0058)	(0.0088)	(0.0068)	(0.0106)	(0.0082)	(0.0104)
Note:<br>Standard errors in parentheses ()<br>*** p<0.01, ** p<0.05, * p<0.1 by Z-test by Z-test						

Table S18: Long Run Coefficient Estimation using Generalized Least Square with Ecosystem Vulnerability for SDG 7.1.2 – With Regulatory Quality, Rule of Law, and Voice and Accountability, Related to Figure 3

Variables	Model 1	Model 2	Model 1	Model 2	Model 1	Model 2
	Regulatory Quality		Rule of Law		Voice and Accountability	
Renewable Electricity Generation	0.0287***	-0.5765***	0.0272***	-0.6548***	0.0464***	-0.0719
	(0.0071)	(0.1044)	(0.0031)	(0.1441)	(0.0046)	(0.1601)
Financial Access	0.1162***	-3.2537***	0.2045***	-0.0358	0.1296***	-4.9626***
	(0.0378)	(0.6245)	(0.0272)	(1.5228)	(0.0299)	(0.9587)
Policy Stringency	0.0056	-0.0537	0.0022	-0.3268*	0.0083	-0.2239*
	(0.0070)	(0.1194)	(0.0052)	(0.1839)	(0.0059)	(0.1262)
Per Capita Income	0.2958***	-0.9047***	0.2905***	-0.3898	0.2827***	-0.5809*
	(0.0224)	(0.3195)	(0.0149)	(0.3626)	(0.0154)	(0.3160)
Urban Population Growth	-0.0102	0.1005	-0.0055	0.3779	-0.0171***	0.0021
	(0.0076)	(0.1408)	(0.0062)	(0.2936)	(0.0065)	(0.1462)
Ecosystem Vulnerability	-1.6228***	-42.0553***	-1.9647***	-33.6167***	-1.4758***	-26.5155***
	(0.2711)	(7.6197)	(0.2436)	(9.0596)	(0.2300)	(7.9195)
Quality of Governance	0.0271*	-5.4413***	-0.0050	-4.6078***	0.0578***	-1.8500***
	(0.0163)	(1.3477)	(0.0120)	(1.2879)	(0.0107)	(0.3743)
Renewable Electricity Generation * Ecosystem Vulnerability	-	1.1962***	-	1.3558***	-	0.1203
	-	(0.2138)	-	(0.2938)	-	(0.3240)
Financial Access * Ecosystem Vulnerability	-	7.5981***	-	1.0746	-	10.9855***
	-	(1.3668)	-	(3.1631)	-	(2.0538)
Policy Stringency * Ecosystem Vulnerability	-	0.0517	-	0.6375*	-	0.4736*
	-	(0.2556)	-	(0.3726)	-	(0.2587)
Per Capita Income * Ecosystem Vulnerability	-	2.1353***	-	1.4704**	-	1.8831***
	-	(0.6567)	-	(0.7127)	-	(0.6290)
Urban Population Growth	-	-0.2699	-	-0.7845	-	-0.0540
	-	(0.2959)	-	(0.6083)	-	(0.3068)
Renewable Electricity Generation * Quality of Governance	-	0.5478***	-	1.0596***	-	0.4607***
	-	(0.1931)	-	(0.4086)	-	(0.1721)
Financial Access * Quality of Governance	-	0.6606	-	5.7351***	-	-3.5528***
	-	(1.3198)	-	(2.1306)	-	(0.9039)
Policy Stringency * Quality of Governance	-	-0.7850*	-	-0.7919*	-	0.3540**
	-	(0.4486)	-	(0.4774)	-	(0.1587)
Per Capita Income * Quality of Governance	-	-0.0374	-	-0.9409**	-	-0.1277
	-	(0.1940)	-	(0.4225)	-	(0.1990)
Urban Population Growth	-	-0.7411***	-	-0.4481	-	-0.0431
	-	(0.2174)	-	(0.4852)	-	(0.1551)
Renewable Electricity Generation * Quality of Governance * Ecosystem Vulnerability	-	-1.0249***	-	-2.2024***	-	-0.7265**
	-	(0.3919)	-	(0.8377)	-	(0.3366)
Financial Access * Quality of Governance * Ecosystem Vulnerability	-	-1.6050	-	-11.9692**	-	7.5486***
	-	(2.9067)	-	(4.6950)	-	(1.8908)
Policy Stringency * Quality of Governance * Ecosystem Vulnerability	-	1.6619*	-	1.7975*	-	-0.7645**

	-	(0.9263)	-	(1.0216)	-	(0.3185)
Per Capita Income * Quality of Governance * Ecosystem Vulnerability	-	0.9800**	-	2.8069***	-	0.3570
	-	(0.4253)	-	(0.8982)	-	(0.3926)
Urban Population Growth	-	1.7861***	-	1.0675	-	0.1518
	-	(0.4612)	-	(1.0079)	-	(0.3124)
Constant	1.6232***	23.3027***	1.8170***	17.0364***	1.5110***	13.9022***
	(0.3005)	(3.7595)	(0.2422)	(4.5809)	(0.2474)	(3.9589)
Wald-chi2	2338	9244	3255	3357	6798	13279
Observations	115	115	115	115	115	115
Heteroskedasticity controlled	Yes	Yes	Yes	Yes	Yes	Yes
Autocorrelation controlled	Yes	Yes	Yes	Yes	Yes	Yes
Cross-sectional correlation controlled	Yes	Yes	Yes	Yes	Yes	Yes
Marginal Impact						
Renewable Electricity Generation	0.0287***	-0.0006	0.0272***	-0.0166	0.0464***	-0.0374***
	(0.0071)	(0.0082)	(0.0031)	(0.0111)	(0.0046)	(0.0107)
Financial Access	0.1162***	0.4464***	0.2045***	0.3876***	0.1296***	0.3597***
	(0.0378)	(0.0589)	(0.0272)	(0.0755)	(0.0299)	(0.0705)
Policy Stringency	0.0056	-0.0333***	0.0022	-0.0230**	0.0083	0.0074
	(0.0070)	(0.0120)	(0.0052)	(0.0114)	(0.0059)	(0.0092)
Per Capita Income	0.2958***	0.0806**	0.2905***	0.2295***	0.2827***	0.3238***
	(0.0224)	(0.0329)	(0.0149)	(0.0471)	(0.0154)	(0.0429)
Urban Population Growth	-0.0102	-0.0468***	-0.0055	-0.0122	-0.0171***	-0.0300***
	(0.0076)	(0.0083)	(0.0062)	(0.0139)	(0.0065)	(0.0098)

Note:
Standard errors in parentheses ()
*** p<0.01, ** p<0.05, * p<0.1 by Z-test by Z-test

Table S19: Long Run Coefficient Estimation using Generalized Least Square with Food Vulnerability for SDG 7.1.2 – With Control of Corruption, Governance Effectiveness, and Political Stability, Related to Figure 3

Variables	Model 1	Model 2	Model 1	Model 2	Model 1	Model 2
	Control of Corruption		Governance Effectiveness		Political Stability	
Renewable Electricity Generation	0.0393***	-0.3466***	0.0276***	-0.0058	0.0322***	-0.3245***
	(0.0068)	(0.0856)	(0.0049)	(0.0620)	(0.0068)	(0.1085)
Financial Access	0.1265***	-1.1375	0.1659***	-2.0217***	0.1236***	-3.1448***
	(0.0350)	(0.6997)	(0.0260)	(0.5163)	(0.0412)	(0.5611)
Policy Stringency	0.0021	-0.0921	0.0039	-0.0845	0.0016	-0.0230
	(0.0070)	(0.0729)	(0.0058)	(0.0626)	(0.0079)	(0.0961)
Per Capita Income	0.3367***	-0.2270	0.3344***	-0.8668***	0.4124***	-0.1115
	(0.0241)	(0.2185)	(0.0145)	(0.2046)	(0.0275)	(0.1893)
Urban Population Growth	-0.0136	0.1294	-0.0066	-0.1902**	-0.0061	-0.0909
	(0.0083)	(0.1289)	(0.0076)	(0.0925)	(0.0090)	(0.1348)
Food Vulnerability	0.1678	-23.6272***	0.3698	-30.4909***	-1.1875***	-22.1289***
	(0.3331)	(4.6779)	(0.2973)	(4.6618)	(0.3339)	(4.6244)
Quality of Governance	-0.0127	-4.8205***	-0.0267***	-5.4936***	0.0358***	-1.1244*
	(0.0136)	(0.8600)	(0.0101)	(1.3208)	(0.0113)	(0.6651)
Renewable Electricity Generation * Food Vulnerability	-	0.8194***	-	0.0651	-	0.7918***
	-	(0.1953)	-	(0.1468)	-	(0.2512)
Financial Access * Food Vulnerability	-	4.0048**	-	6.2697***	-	8.8992***
	-	(1.7194)	-	(1.2951)	-	(1.4064)
Policy Stringency * Food Vulnerability	-	0.2040	-	0.1217	-	0.0228
	-	(0.1750)	-	(0.1521)	-	(0.2343)
Per Capita Income * Food Vulnerability	-	1.2174**	-	2.6675***	-	0.9062**
	-	(0.5039)	-	(0.4894)	-	(0.4515)
Urban Population Growth * Food Vulnerability	-	-0.3459	-	0.3567	-	0.1682
	-	(0.3059)	-	(0.2188)	-	(0.3284)
Renewable Electricity Generation * Quality of Governance	-	-0.9865***	-	-1.0106***	-	-0.3236**
	-	(0.1968)	-	(0.2078)	-	(0.1452)
Financial Access * Quality of Governance	-	1.5822*	-	-0.8991	-	-0.0255
	-	(0.9308)	-	(1.0547)	-	(0.5961)
Policy Stringency * Quality of Governance	-	0.5953***	-	0.4717***	-	0.2123*
	-	(0.2160)	-	(0.1797)	-	(0.1236)
Per Capita Income * Quality of Governance	-	1.3188***	-	1.4004***	-	0.4196***
	-	(0.2144)	-	(0.2320)	-	(0.1567)
Urban Population Growth * Quality of Governance	-	0.8244***	-	1.0763***	-	0.2521*
	-	(0.2537)	-	(0.2419)	-	(0.1376)
Renewable Electricity Generation * Quality of Governance * Food Vulnerability	-	2.4245***	-	2.4553***	-	0.7314**
	-	(0.4553)	-	(0.4792)	-	(0.3332)
Financial Access * Quality of Governance * Food Vulnerability	-	-4.4065*	-	2.1735	-	-0.0596
	-	(2.4076)	-	(2.7731)	-	(1.5737)
Policy Stringency * Quality of Governance * Food Vulnerability	-	-1.5176***	-	-1.1676***	-	-0.4736

	-	(0.5431)	-	(0.4402)	-	(0.2982)
Per Capita Income * Quality of Governance * Food Vulnerability	-	-2.1180***	-	-2.1456***	-	-0.7005*
	-	(0.4517)	-	(0.4941)	-	(0.3629)
Urban Population Growth * Quality of Governance * Food Vulnerability	-	-1.6601***	-	-2.3194***	-	-0.4656
	-	(0.6219)	-	(0.6031)	-	(0.3435)
Constant	0.2515	11.1874***	0.2387	14.1199***	0.1117	10.6080***
	(0.3792)	(1.9865)	(0.2761)	(1.9170)	(0.4185)	(1.8845)
Wald-chi2	577.8	15309	2851	25700	1312	6896
Observations	115	115	115	115	115	115
Heteroskedasticity controlled	Yes	Yes	Yes	Yes	Yes	Yes
Autocorrelation controlled	Yes	Yes	Yes	Yes	Yes	Yes
Cross-sectional correlation controlled	Yes	Yes	Yes	Yes	Yes	Yes
Marginal Impact						
Renewable Electricity Generation	0.0393***	0.0030	0.0276***	0.0382***	0.0322***	0.0149
	(0.0068)	(0.0077)	(0.0049)	(0.0079)	(0.0068)	(0.0092)
Financial Access	0.1265***	0.7395***	0.1659***	0.7745***	0.1236***	0.8349***
	(0.0350)	(0.0825)	(0.0260)	(0.0650)	(0.0412)	(0.0801)
Policy Stringency	0.0021	0.0150*	0.0039	-0.0376***	0.0016	-0.0066
	(0.0070)	(0.0084)	(0.0058)	(0.0079)	(0.0079)	(0.0101)
Per Capita Income	0.3367***	0.1658***	0.3344***	0.2983***	0.4124***	0.2410***
	(0.0241)	(0.0286)	(0.0145)	(0.0275)	(0.0275)	(0.0305)
Urban Population Growth	-0.0136	-0.0666***	-0.0066	-0.0475***	-0.0061	-0.0343***
	(0.0083)	(0.0107)	(0.0076)	(0.0099)	(0.0090)	(0.0117)

Note:
Standard errors in parentheses ()
*** p<0.01, ** p<0.05, * p<0.1 by Z-test by Z-test

Table S20: Long Run Coefficient Estimation using Generalized Least Square with Food Vulnerability for SDG 7.1.2 – With Regulatory Quality, Rule of Law, and Voice and Accountability, Related to Figure 3

Variables	Model 1	Model 2	Model 1	Model 2	Model 1	Model 2
	Regulatory Quality		Rule of Law		Voice and Accountability	
Renewable Electricity Generation	0.0242***	-0.1598*	0.0280***	-0.0971	0.0625***	0.2130**
	(0.0076)	(0.0912)	(0.0060)	(0.0874)	(0.0057)	(0.1085)
Financial Access	0.1072***	-2.8396***	0.1518***	-2.9154***	0.1670***	-2.5420***
	(0.0375)	(0.5537)	(0.0349)	(0.5583)	(0.0302)	(0.4778)
Policy Stringency	0.0052	-0.0562	0.0004	-0.2809***	-0.0067	-0.2599***
	(0.0076)	(0.0707)	(0.0072)	(0.0711)	(0.0067)	(0.0739)
Per Capita Income	0.3691***	-0.3855*	0.3613***	0.2344	0.3151***	-0.7530***
	(0.0243)	(0.2223)	(0.0207)	(0.2692)	(0.0196)	(0.2028)
Urban Population Growth	-0.0059	-0.2248**	-0.0129	0.1434	-0.0160**	-0.5775***
	(0.0084)	(0.1106)	(0.0081)	(0.1417)	(0.0078)	(0.1027)
Food Vulnerability	-0.4023	-25.9727***	0.0727	-8.2953	0.1635	-25.6063***
	(0.3446)	(5.0200)	(0.3375)	(6.5054)	(0.3047)	(4.3058)
Quality of Governance	0.0440***	-7.3222***	-0.0275*	-5.3588***	0.0796***	-1.4481***
	(0.0169)	(1.1901)	(0.0162)	(1.3026)	(0.0127)	(0.4537)
Renewable Electricity Generation * Food Vulnerability	-	0.4660**	-	0.2378	-	-0.5143**
	-	(0.2106)	-	(0.1975)	-	(0.2580)
Financial Access * Food Vulnerability	-	7.9748***	-	8.3279***	-	6.9929***
	-	(1.3704)	-	(1.3507)	-	(1.2273)
Policy Stringency * Food Vulnerability	-	0.0887	-	0.6455***	-	0.6293***
	-	(0.1678)	-	(0.1629)	-	(0.1723)
Per Capita Income * Food Vulnerability	-	1.6810***	-	0.1853	-	2.5347***
	-	(0.5272)	-	(0.5817)	-	(0.4759)
Urban Population Growth * Food Vulnerability	-	0.4240	-	-0.3108	-	1.3294***
	-	(0.2634)	-	(0.3336)	-	(0.2485)
Renewable Electricity Generation * Quality of Governance	-	-0.5706**	-	-0.6882***	-	-0.7186***
	-	(0.2291)	-	(0.2510)	-	(0.1655)
Financial Access * Quality of Governance	-	-0.0211	-	2.3819**	-	-0.3593
	-	(1.1826)	-	(0.9751)	-	(0.6571)
Policy Stringency * Quality of Governance	-	0.2600	-	0.3039	-	0.4015***
	-	(0.2136)	-	(0.2481)	-	(0.1265)
Per Capita Income * Quality of Governance	-	1.2397***	-	1.0304***	-	0.9673***
	-	(0.2316)	-	(0.3003)	-	(0.1874)
Urban Population Growth * Quality of Governance	-	0.3682	-	0.8881***	-	0.2666**
	-	(0.2368)	-	(0.1928)	-	(0.1276)
Renewable Electricity Generation * Quality of Governance * Food Vulnerability	-	1.4815***	-	1.8255***	-	1.8330***
	-	(0.5085)	-	(0.5908)	-	(0.4090)
Financial Access * Quality of Governance * Food Vulnerability	-	-1.1884	-	-6.7872***	-	0.3229
	-	(2.9059)	-	(2.5985)	-	(1.6778)
Policy Stringency * Quality of Governance * Food Vulnerability	-	-0.6407	-	-0.8467	-	-1.0259***

	-	(0.4976)	-	(0.6316)	-	(0.3153)
Per Capita Income * Quality of Governance * Food Vulnerability	-	-1.3997***	-	-1.4261**	-	-2.0806***
	-	(0.5070)	-	(0.6596)	-	(0.4410)
Urban Population Growth * Quality of Governance * Food Vulnerability	-	-0.5175	-	-1.8492***	-	-0.6430**
	-	(0.5778)	-	(0.4788)	-	(0.3127)
Constant	0.2834	11.4308***	0.1169	4.3079	0.2151	11.8337***
	(0.3707)	(2.1118)	(0.3404)	(3.0151)	(0.3112)	(1.9352)
Wald-chi2	1184	19443	1537	10579	1936	18037
Observations	115	115	115	115	115	115
Heteroskedasticity controlled	Yes	Yes	Yes	Yes	Yes	Yes
Autocorrelation controlled	Yes	Yes	Yes	Yes	Yes	Yes
Cross-sectional correlation controlled	Yes	Yes	Yes	Yes	Yes	Yes
Marginal Impact						
Renewable Electricity Generation	0.0242***	0.0355***	0.0280***	0.0101	0.0625***	0.0315***
	(0.0076)	(0.0084)	(0.0060)	(0.0116)	(0.0057)	(0.0075)
Financial Access	0.1072***	0.7627***	0.1518***	0.8256***	0.1670***	0.6176***
	(0.0375)	(0.0715)	(0.0349)	(0.0815)	(0.0302)	(0.0764)
Policy Stringency	0.0052	-0.0134	0.0004	0.0093	-0.0067	-0.0067
	(0.0076)	(0.0099)	(0.0072)	(0.0096)	(0.0067)	(0.0096)
Per Capita Income	0.3691***	0.2932***	0.3613***	0.1730***	0.3151***	0.2878***
	(0.0243)	(0.0267)	(0.0207)	(0.0290)	(0.0196)	(0.0376)
Urban Population Growth	-0.0059	-0.0514***	-0.0129	-0.0514***	-0.0160**	-0.0069
	(0.0084)	(0.0123)	(0.0081)	(0.0144)	(0.0078)	(0.0120)
Note:<br>Standard errors in parentheses ()<br>*** p<0.01, ** p<0.05, * p<0.1 by Z-test by Z-test						

Table S21: Long Run Coefficient Estimation using Generalized Least Square with Health Vulnerability for SDG 7.1.2 – With Control of Corruption, Governance Effectiveness, and Political Stability, Related to Figure 3

Variables	Model 1	Model 2	Model 1	Model 2	Model 1	Model 2
	Control of Corruption		Governance Effectiveness		Political Stability	
Renewable Electricity Generation	0.0419***	0.1135*	0.0210***	0.0119	0.0405***	0.1863***
	(0.0058)	(0.0624)	(0.0038)	(0.0455)	(0.0078)	(0.0593)
Financial Access	0.1221***	-1.2418**	0.1401***	-1.2678***	0.0876**	-1.0899***
	(0.0331)	(0.5046)	(0.0239)	(0.2738)	(0.0369)	(0.3546)
Policy Stringency	-0.0000	-0.4070***	0.0043	-0.4282***	0.0026	-0.2924***
	(0.0064)	(0.1035)	(0.0056)	(0.0624)	(0.0072)	(0.0777)
Per Capita Income	0.2868***	-0.3566***	0.3158***	-0.3171***	0.3963***	-0.6698***
	(0.0257)	(0.1375)	(0.0144)	(0.1120)	(0.0274)	(0.1211)
Urban Population Growth	-0.0144*	-0.0247	-0.0102	0.0482	-0.0074	-0.2927***
	(0.0081)	(0.1027)	(0.0073)	(0.0541)	(0.0095)	(0.0798)
Health Vulnerability	-0.3028*	-13.2993***	-0.3437**	-12.9076***	-0.8385***	-18.1611***
	(0.1595)	(3.4132)	(0.1364)	(2.7543)	(0.1740)	(3.0748)
Quality of Governance	-0.0101	-4.6534***	-0.0219**	-6.5246***	0.0292***	-0.9565
	(0.0120)	(0.8536)	(0.0099)	(1.1001)	(0.0105)	(0.7699)
Renewable Electricity Generation * Health Vulnerability	-	-0.2323*	-	-0.0621	-	-0.3824***
	-	(0.1366)	-	(0.1033)	-	(0.1367)
Financial Access * Health Vulnerability	-	3.6643***	-	3.8991***	-	3.6162***
	-	(1.1666)	-	(0.6861)	-	(0.9005)
Policy Stringency * Health Vulnerability	-	0.8704***	-	0.8915***	-	0.6135***
	-	(0.2196)	-	(0.1334)	-	(0.1708)
Per Capita Income * Health Vulnerability	-	1.2274***	-	0.9902***	-	1.8396***
	-	(0.2912)	-	(0.2619)	-	(0.2576)
Urban Population Growth * Health Vulnerability	-	0.0428	-	-0.1472	-	0.6452***
	-	(0.2393)	-	(0.1279)	-	(0.1915)
Renewable Electricity Generation * Quality of Governance	-	0.4745***	-	0.7024***	-	0.0825
	-	(0.1155)	-	(0.0980)	-	(0.1022)
Financial Access * Quality of Governance	-	0.4453	-	0.3454	-	-0.1329
	-	(0.6482)	-	(0.5624)	-	(0.3347)
Policy Stringency * Quality of Governance	-	-0.2634	-	-0.5159***	-	0.0349
	-	(0.1707)	-	(0.0951)	-	(0.1047)
Per Capita Income * Quality of Governance	-	-0.0929	-	-0.1845	-	0.0012
	-	(0.1242)	-	(0.1197)	-	(0.1210)
Urban Population Growth * Quality of Governance	-	-0.2367	-	-0.2264	-	-0.0847
	-	(0.1604)	-	(0.1626)	-	(0.0946)
Renewable Electricity Generation * Quality of Governance * Health Vulnerability	-	-0.8589***	-	-1.3034***	-	-0.1741
	-	(0.2581)	-	(0.2277)	-	(0.2310)
Financial Access * Quality of Governance * Health Vulnerability	-	-1.3444	-	-0.9750	-	0.3390
	-	(1.7174)	-	(1.5165)	-	(0.9443)
Policy Stringency * Quality of Governance * Health Vulnerability	-	0.5137	-	0.9983***	-	-0.0639

	-	(0.4012)	-	(0.2201)	-	(0.2271)
Per Capita Income * Quality of Governance * Health Vulnerability	-	0.9376***	-	1.4634***	-	0.1734
	-	(0.2382)	-	(0.1957)	-	(0.2597)
Urban Population Growth * Quality of Governance * Health Vulnerability	-	0.7612*	-	0.7514*	-	0.3022
	-	(0.4094)	-	(0.4306)	-	(0.2404)
Constant	0.9656***	8.0520***	0.8302***	8.8828***	0.0673	10.5010***
	(0.3399)	(1.5764)	(0.2112)	(1.1679)	(0.3609)	(1.4209)
Wald-chi2	519.0	5434	3960	11573	1425	5188
Observations	115	115	115	115	115	115
Heteroskedasticity controlled	Yes	Yes	Yes	Yes	Yes	Yes
Autocorrelation controlled	Yes	Yes	Yes	Yes	Yes	Yes
Cross-sectional correlation controlled	Yes	Yes	Yes	Yes	Yes	Yes
Marginal Impact						
Renewable Electricity Generation	0.0419***	-0.0296***	0.0210***	-0.0279***	0.0405***	0.0102
	(0.0058)	(0.0084)	(0.0038)	(0.0060)	(0.0078)	(0.0098)
Financial Access	0.1221***	0.5142***	0.1401***	0.5419***	0.0876**	0.5749***
	(0.0331)	(0.0832)	(0.0239)	(0.0693)	(0.0369)	(0.0738)
Policy Stringency	-0.0000	0.0121	0.0043	-0.0049	0.0026	-0.0085
	(0.0064)	(0.0097)	(0.0056)	(0.0072)	(0.0072)	(0.0089)
Per Capita Income	0.2868***	0.1044***	0.3158***	0.1497***	0.3963***	0.1383***
	(0.0257)	(0.0350)	(0.0144)	(0.0318)	(0.0274)	(0.0341)
Urban Population Growth	-0.0144*	-0.0382***	-0.0102	-0.0160*	-0.0074	-0.0284**
	(0.0081)	(0.0132)	(0.0073)	(0.0093)	(0.0095)	(0.0134)

Note:
Standard errors in parentheses ()
*** p<0.01, ** p<0.05, * p<0.1 by Z-test by Z-test

Table S22: Long Run Coefficient Estimation using Generalized Least Square with Health Vulnerability for SDG 7.1.2 – With Regulatory Quality, Rule of Law, and Voice and Accountability, Related to Figure 3

Variables	Model 1	Model 2	Model 1	Model 2	Model 1	Model 2
	Regulatory Quality		Rule of Law		Voice and Accountability	
Renewable Electricity Generation	0.0260***	0.1219***	0.0262***	0.1339**	0.0395***	0.3173***
	(0.0074)	(0.0408)	(0.0055)	(0.0546)	(0.0058)	(0.0429)
Financial Access	0.0649**	-1.0940***	0.1314***	-1.3403***	0.1794***	-1.0931***
	(0.0275)	(0.2658)	(0.0280)	(0.4532)	(0.0283)	(0.2551)
Policy Stringency	0.0056	-0.3106***	-0.0048	-0.3183***	-0.0132**	-0.0306
	(0.0065)	(0.0579)	(0.0066)	(0.0713)	(0.0062)	(0.0581)
Per Capita Income	0.3302***	-0.6000***	0.3454***	0.0256	0.3191***	-0.3003**
	(0.0216)	(0.1340)	(0.0186)	(0.1767)	(0.0191)	(0.1272)
Urban Population Growth	-0.0113	-0.1004	-0.0152*	0.1202	-0.0073	-0.0914
	(0.0078)	(0.0622)	(0.0080)	(0.0973)	(0.0084)	(0.0680)
Health Vulnerability	-0.7509***	-19.3999***	-0.4198***	-6.4819	-0.4514***	-9.4806***
	(0.1638)	(2.9949)	(0.1536)	(4.1669)	(0.1585)	(2.8639)
Quality of Governance	0.0388***	-9.0017***	-0.0336**	-6.2574***	0.0389***	-3.1415***
	(0.0133)	(1.1448)	(0.0139)	(1.2254)	(0.0130)	(0.4363)
Renewable Electricity Generation * Health Vulnerability	-	-0.2449***	-	-0.2511**	-	-0.6735***
	-	(0.0936)	-	(0.1170)	-	(0.0963)
Financial Access * Health Vulnerability	-	3.0834***	-	4.2358***	-	3.0521***
	-	(0.6411)	-	(1.0266)	-	(0.6061)
Policy Stringency * Health Vulnerability	-	0.6598***	-	0.6705***	-	0.1458
	-	(0.1224)	-	(0.1454)	-	(0.1174)
Per Capita Income * Health Vulnerability	-	1.9019***	-	0.7259**	-	1.5525***
	-	(0.3008)	-	(0.3367)	-	(0.2623)
Urban Population Growth * Health Vulnerability	-	0.1647	-	-0.2549	-	0.1691
	-	(0.1422)	-	(0.2221)	-	(0.1556)
Renewable Electricity Generation * Quality of Governance	-	0.3123***	-	0.0961	-	-0.1993***
	-	(0.1058)	-	(0.1651)	-	(0.0671)
Financial Access * Quality of Governance	-	-0.1696	-	0.5370	-	-0.4632
	-	(0.4880)	-	(0.6441)	-	(0.3102)
Policy Stringency * Quality of Governance	-	-0.3258*	-	0.0510	-	0.1690**
	-	(0.1724)	-	(0.1280)	-	(0.0741)
Per Capita Income * Quality of Governance	-	0.4983***	-	0.3397*	-	0.5370***
	-	(0.1431)	-	(0.1943)	-	(0.0938)
Urban Population Growth * Quality of Governance	-	-0.1368	-	0.1470	-	0.1430***
	-	(0.1107)	-	(0.1429)	-	(0.0495)
Renewable Electricity Generation * Quality of Governance * Health Vulnerability	-	-0.3576	-	0.0087	-	0.6183***
	-	(0.2425)	-	(0.3718)	-	(0.1617)
Financial Access * Quality of Governance * Health Vulnerability	-	-0.8325	-	-1.3644	-	0.8522
	-	(1.2495)	-	(1.6920)	-	(0.7253)
Policy Stringency * Quality of Governance * Health Vulnerability	-	0.6419*	-	-0.0758	-	-0.3723**

	-	(0.3656)	-	(0.2962)	-	(0.1637)
Per Capita Income * Quality of Governance * Health Vulnerability	-	0.4156*	-	0.3106	-	-0.7469***
	-	(0.2201)	-	(0.4249)	-	(0.1810)
Urban Population Growth * Quality of Governance * Health Vulnerability	-	0.6593**	-	-0.2801	-	-0.2054*
	-	(0.2802)	-	(0.3600)	-	(0.1159)
Constant	0.8464***	10.2720***	0.5324**	3.1087	0.6610**	4.2527***
	(0.2794)	(1.3769)	(0.2545)	(2.1541)	(0.2706)	(1.4448)
Wald-chi2	2131	11412	2344	4622	2959	11827
Observations	115	115	115	115	115	115
Heteroskedasticity controlled	Yes	Yes	Yes	Yes	Yes	Yes
Autocorrelation controlled	Yes	Yes	Yes	Yes	Yes	Yes
Cross-sectional correlation controlled	Yes	Yes	Yes	Yes	Yes	Yes
Marginal Impact						
Renewable Electricity Generation	0.0260***	-0.0077	0.0262***	-0.0117	0.0395***	0.0088
	(0.0074)	(0.0073)	(0.0055)	(0.0100)	(0.0058)	(0.0118)
Financial Access	0.0649**	0.4059***	0.1314***	0.6265***	0.1794***	0.3739***
	(0.0275)	(0.0583)	(0.0280)	(0.0825)	(0.0283)	(0.0618)
Policy Stringency	0.0056	-0.0017	-0.0048	-0.0125	-0.0132**	0.0242**
	(0.0065)	(0.0077)	(0.0066)	(0.0090)	(0.0062)	(0.0095)
Per Capita Income	0.3302***	0.2076***	0.3454***	0.2316***	0.3191***	0.3560***
	(0.0216)	(0.0292)	(0.0186)	(0.0364)	(0.0191)	(0.0338)
Urban Population Growth	-0.0113	-0.0440***	-0.0152*	-0.0103	-0.0073	-0.0300***
	(0.0078)	(0.0119)	(0.0080)	(0.0153)	(0.0084)	(0.0110)

Note:
Standard errors in parentheses ()
*** p<0.01, ** p<0.05, * p<0.1 by Z-test by Z-test

Table S23: Long Run Coefficient Estimation using Generalized Least Square with Infrastructure Vulnerability for SDG 7.1.2 – With Control of Corruption, Governance Effectiveness, and Political Stability, Related to Figure 3

Variables	Model 1	Model 2	Model 1	Model 2	Model 1	Model 2
	Control of Corruption		Governance Effectiveness		Political Stability	
Renewable Electricity Generation	0.0343***	0.0180	0.0330***	-0.0468	0.0463***	-0.0820**
	(0.0087)	(0.0372)	(0.0046)	(0.0295)	(0.0090)	(0.0395)
Financial Access	0.1051**	-0.2398	0.1772***	0.1490	0.1532***	-0.1522
	(0.0464)	(0.2920)	(0.0366)	(0.1644)	(0.0574)	(0.1573)
Policy Stringency	0.0065	-0.0382	-0.0000	-0.2011***	-0.0041	-0.0874***
	(0.0073)	(0.0389)	(0.0071)	(0.0287)	(0.0093)	(0.0330)
Per Capita Income	0.3488***	-0.2007**	0.3422***	-0.4290***	0.4763***	-0.3402***
	(0.0255)	(0.0885)	(0.0173)	(0.1001)	(0.0314)	(0.0730)
Urban Population Growth	-0.0092	-0.0418	-0.0066	0.0406	-0.0026	-0.0269
	(0.0083)	(0.0539)	(0.0078)	(0.0388)	(0.0105)	(0.0373)
Infrastructure Vulnerability	-0.5929***	-36.3296***	-0.8317***	-46.9238***	-0.8914***	-51.7205***
	(0.1826)	(5.7518)	(0.1967)	(6.3870)	(0.2757)	(5.2207)
Quality of Governance	-0.0103	-1.6471*	-0.0441***	4.3013***	0.0348**	-0.3036
	(0.0162)	(0.9470)	(0.0133)	(1.3278)	(0.0135)	(0.7107)
Renewable Electricity Generation * Infrastructure Vulnerability	-	0.0120	-	0.2689	-	1.0014***
	-	(0.2593)	-	(0.2078)	-	(0.2891)
Financial Access * Infrastructure Vulnerability	-	4.6000**	-	1.4375	-	2.2031*
	-	(2.0772)	-	(1.0981)	-	(1.1400)
Policy Stringency * Infrastructure Vulnerability	-	0.2082	-	1.1984***	-	0.5824***
	-	(0.2548)	-	(0.1627)	-	(0.2138)
Per Capita Income * Infrastructure Vulnerability	-	3.0206***	-	3.8842***	-	3.4585***
	-	(0.5468)	-	(0.6669)	-	(0.4866)
Urban Population Growth * Infrastructure Vulnerability	-	0.3521	-	-0.3725	-	0.1156
	-	(0.3904)	-	(0.2722)	-	(0.2670)
Renewable Electricity Generation * Quality of Governance	-	0.0260	-	0.1484*	-	-0.0284
	-	(0.0824)	-	(0.0765)	-	(0.0611)
Financial Access * Quality of Governance	-	-0.3844	-	1.1816***	-	0.0038
	-	(0.3478)	-	(0.1854)	-	(0.1358)
Policy Stringency * Quality of Governance	-	0.1777**	-	-0.2671***	-	-0.0629
	-	(0.0740)	-	(0.0989)	-	(0.0536)
Per Capita Income * Quality of Governance	-	0.1526*	-	-0.5895***	-	0.0604
	-	(0.0856)	-	(0.0971)	-	(0.0602)
Urban Population Growth * Quality of Governance	-	-0.1940**	-	0.1472**	-	0.0633
	-	(0.0909)	-	(0.0680)	-	(0.0404)
Renewable Electricity Generation * Quality of Governance * Infrastructure Vulnerability	-	-0.1390	-	-0.6146	-	0.2401
	-	(0.5921)	-	(0.4867)	-	(0.4261)
Financial Access * Quality of Governance * Infrastructure Vulnerability	-	6.5236**	-	-5.8471***	-	-0.6834
	-	(3.2874)	-	(1.9180)	-	(1.4943)
Policy Stringency * Quality of Governance * Infrastructure Vulnerability	-	-1.1204**	-	1.9329***	-	0.4216

	-	(0.5042)	-	(0.5839)	-	(0.3375)
Per Capita Income * Quality of Governance * Infrastructure Vulnerability	-	-0.4233	-	0.5615	-	-0.2432
	-	(0.5771)	-	(0.5626)	-	(0.5302)
Urban Population Growth * Quality of Governance * Infrastructure Vulnerability	-	2.3633***	-	-0.9972**	-	-0.3414
	-	(0.8224)	-	(0.4554)	-	(0.3384)
Constant	0.2913	6.7347***	0.3937*	9.7883***	-1.1242***	9.4168***
	(0.3095)	(1.0183)	(0.2034)	(1.0832)	(0.3761)	(0.9004)
Wald-chi2	309.5	7758	1746	13807	617.8	11401
Observations	115	115	115	115	115	115
Heteroskedasticity controlled	Yes	Yes	Yes	Yes	Yes	Yes
Autocorrelation controlled	Yes	Yes	Yes	Yes	Yes	Yes
Cross-sectional correlation controlled	Yes	Yes	Yes	Yes	Yes	Yes
Marginal Impact						
Renewable Electricity Generation	0.0343***	0.0178**	0.0330***	-0.0084	0.0463***	0.0705***
	(0.0087)	(0.0085)	(0.0046)	(0.0080)	(0.0090)	(0.0107)
Financial Access	0.1051**	0.3016***	0.1772***	0.3387***	0.1532***	0.2626***
	(0.0464)	(0.0545)	(0.0366)	(0.0458)	(0.0574)	(0.0489)
Policy Stringency	0.0065	-0.0110	-0.0000	0.0018	-0.0041	0.0002
	(0.0073)	(0.0101)	(0.0071)	(0.0094)	(0.0093)	(0.0083)
Per Capita Income	0.3488***	0.2489***	0.3422***	0.1994***	0.4763***	0.2010***
	(0.0255)	(0.0333)	(0.0173)	(0.0305)	(0.0314)	(0.0340)
Urban Population Growth	-0.0092	-0.0362***	-0.0066	-0.0250**	-0.0026	-0.0111
	(0.0083)	(0.0121)	(0.0078)	(0.0120)	(0.0105)	(0.0118)

Note:
Standard errors in parentheses ()
*** p<0.01, ** p<0.05, * p<0.1 by Z-test by Z-test

Table S24: Long Run Coefficient Estimation using Generalized Least Square with Infrastructure Vulnerability for SDG 7.1.2 – With Regulatory Quality, Rule of Law, and Voice and Accountability, Related to Figure 3

Variables	Model 1	Model 2	Model 1	Model 2	Model 1	Model 2
	Regulatory Quality		Rule of Law		Voice and Accountability	
Renewable Electricity Generation	0.0393***	0.0197	0.0448***	-0.0461	0.0608***	0.0057
	(0.0110)	(0.0258)	(0.0072)	(0.0314)	(0.0074)	(0.0370)
Financial Access	0.0749	-0.0183	0.2357***	0.1352	0.1555***	-0.3371**
	(0.0526)	(0.1373)	(0.0560)	(0.2197)	(0.0429)	(0.1312)
Policy Stringency	0.0080	-0.0983***	-0.0011	-0.0168	-0.0001	-0.0865***
	(0.0096)	(0.0269)	(0.0093)	(0.0340)	(0.0086)	(0.0277)
Per Capita Income	0.3801***	-0.2913***	0.4501***	-0.1597*	0.3261***	-0.0398
	(0.0289)	(0.0674)	(0.0305)	(0.0882)	(0.0204)	(0.0820)
Urban Population Growth	0.0005	-0.0288	0.0010	-0.0055	0.0013	-0.0990***
	(0.0093)	(0.0311)	(0.0110)	(0.0466)	(0.0105)	(0.0307)
Infrastructure Vulnerability	-0.6928***	-43.3261***	-1.0251***	-36.1919***	-0.5972**	-32.9236***
	(0.2603)	(4.7632)	(0.2814)	(5.9298)	(0.2473)	(5.0319)
Quality of Governance	0.0436*	-0.7816	-0.0418	-1.3211	0.0849***	-1.1468***
	(0.0224)	(1.0347)	(0.0259)	(1.0027)	(0.0181)	(0.3473)
Renewable Electricity Generation * Infrastructure Vulnerability	-	0.0245	-	0.5427**	-	0.0900
	-	(0.1740)	-	(0.2191)	-	(0.2413)
Financial Access * Infrastructure Vulnerability	-	1.7799*	-	2.4041*	-	4.0392***
	-	(1.0292)	-	(1.4259)	-	(0.9209)
Policy Stringency * Infrastructure Vulnerability	-	0.6673***	-	0.2081	-	0.6809***
	-	(0.1603)	-	(0.1927)	-	(0.1672)
Per Capita Income * Infrastructure Vulnerability	-	3.8522***	-	2.5532***	-	2.5973***
	-	(0.4402)	-	(0.5019)	-	(0.5076)
Urban Population Growth * Infrastructure Vulnerability	-	0.1190	-	0.0656	-	0.7260***
	-	(0.2206)	-	(0.3383)	-	(0.2079)
Renewable Electricity Generation * Quality of Governance	-	-0.2853***	-	0.0271	-	-0.0763
	-	(0.0638)	-	(0.0879)	-	(0.0530)
Financial Access * Quality of Governance	-	0.1774	-	0.1311	-	-0.3848***
	-	(0.2766)	-	(0.2485)	-	(0.1133)
Policy Stringency * Quality of Governance	-	-0.1140	-	0.1806***	-	0.1312***
	-	(0.0797)	-	(0.0666)	-	(0.0371)
Per Capita Income * Quality of Governance	-	0.4031***	-	0.0870	-	0.2310***
	-	(0.0876)	-	(0.0957)	-	(0.0597)
Urban Population Growth * Quality of Governance	-	0.0303	-	-0.1156	-	0.0080
	-	(0.0736)	-	(0.0978)	-	(0.0294)
Renewable Electricity Generation * Quality of Governance * Infrastructure Vulnerability	-	2.3114***	-	-0.3290	-	1.0262***
	-	(0.4477)	-	(0.6635)	-	(0.3083)
Financial Access * Quality of Governance * Infrastructure Vulnerability	-	0.5116	-	3.2063	-	3.4909***
	-	(2.4953)	-	(2.5150)	-	(0.7259)
Policy Stringency * Quality of Governance * Infrastructure Vulnerability	-	0.9422*	-	-0.8273*	-	-0.7373***

	-	(0.5144)	-	(0.4414)	-	(0.2040)
Per Capita Income * Quality of Governance * Infrastructure Vulnerability	-	-2.8501***	-	0.0138	-	-1.5220***
	-	(0.4298)	-	(0.7212)	-	(0.3643)
Urban Population Growth * Quality of Governance * Infrastructure Vulnerability	-	0.0856	-	1.7479**	-	0.1821
	-	(0.6146)	-	(0.8839)	-	(0.1693)
Constant	-0.1446	7.5467***	-0.8405**	6.7922***	0.2282	5.3217***
	(0.3120)	(0.8145)	(0.3619)	(1.0895)	(0.2461)	(0.8933)
Wald-chi2	751.6	16279	742.1	4640	3242	31989
Observations	115	115	115	115	115	115
Heteroskedasticity controlled	Yes	Yes	Yes	Yes	Yes	Yes
Autocorrelation controlled	Yes	Yes	Yes	Yes	Yes	Yes
Cross-sectional correlation controlled	Yes	Yes	Yes	Yes	Yes	Yes
Marginal Impact						
Renewable Electricity Generation	0.0393***	0.0057	0.0448***	0.0432***	0.0608***	0.0099
	(0.0110)	(0.0067)	(0.0072)	(0.0099)	(0.0074)	(0.0116)
Financial Access	0.0749	0.2348***	0.2357***	0.3772***	0.1555***	0.2981***
	(0.0526)	(0.0415)	(0.0560)	(0.0504)	(0.0429)	(0.0559)
Policy Stringency	0.0080	0.0006	-0.0011	-0.0090	-0.0001	0.0138**
	(0.0096)	(0.0086)	(0.0093)	(0.0079)	(0.0086)	(0.0064)
Per Capita Income	0.3801***	0.3394***	0.4501***	0.2219***	0.3261***	0.3655***
	(0.0289)	(0.0296)	(0.0305)	(0.0328)	(0.0204)	(0.0341)
Urban Population Growth	0.0005	-0.0151	0.0010	-0.0181	0.0013	0.0106
	(0.0093)	(0.0112)	(0.0110)	(0.0134)	(0.0105)	(0.0087)

Note:
Standard errors in parentheses ()
*** p<0.01, ** p<0.05, * p<0.1 by Z-test by Z-test

Table S25: Long Run Coefficient Estimation using Generalized Least Square with Water Vulnerability for SDG 7.1.2 – With Control of Corruption, Governance Effectiveness, and Political Stability, Related to Figure 3

Variables	Model 1	Model 2	Model 1	Model 2	Model 1	Model 2
	Control of Corruption		Governance Effectiveness		Political Stability	
Renewable Electricity Generation	0.0521***	0.3131***	0.0464***	0.0386	0.0472***	0.2949***
	(0.0045)	(0.0692)	(0.0050)	(0.0457)	(0.0069)	(0.0668)
Financial Access	0.0914***	0.5231	0.1454***	0.5788*	0.0905**	0.7834**
	(0.0327)	(0.5838)	(0.0325)	(0.3174)	(0.0427)	(0.3617)
Policy Stringency	0.0075	-0.1828**	0.0033	-0.1304***	0.0182**	-0.0146
	(0.0055)	(0.0736)	(0.0060)	(0.0352)	(0.0071)	(0.0616)
Per Capita Income	0.2671***	-1.4248***	0.2969***	-1.3653***	0.3139***	-1.2490***
	(0.0226)	(0.1597)	(0.0181)	(0.1394)	(0.0269)	(0.1413)
Urban Population Growth	-0.0080	-0.2239**	-0.0068	-0.1749***	-0.0042	-0.2211***
	(0.0069)	(0.0941)	(0.0072)	(0.0552)	(0.0083)	(0.0736)
Water Vulnerability	-1.4708***	-40.9454***	-1.1849***	-48.4180***	-2.0761***	-35.4513***
	(0.3483)	(4.1183)	(0.3262)	(3.7647)	(0.2696)	(3.9559)
Quality of Governance	-0.0221**	-3.1661***	-0.0232**	-4.5068***	0.0136*	-0.3743
	(0.0108)	(1.0404)	(0.0101)	(1.3081)	(0.0081)	(0.9222)
Renewable Electricity Generation * Water Vulnerability	-	-0.9214***	-	-0.0965	-	-0.8381***
	-	(0.2144)	-	(0.1322)	-	(0.2033)
Financial Access * Water Vulnerability	-	-1.4221	-	-1.0488	-	-2.0432*
	-	(1.9035)	-	(1.0036)	-	(1.1814)
Policy Stringency * Water Vulnerability	-	0.5858**	-	0.3051***	-	0.0429
	-	(0.2433)	-	(0.1095)	-	(0.2032)
Per Capita Income * Water Vulnerability	-	4.8261***	-	4.6750***	-	4.3380***
	-	(0.4362)	-	(0.3920)	-	(0.4218)
Urban Population Growth * Water Vulnerability	-	0.6761**	-	0.4701***	-	0.6186**
	-	(0.2997)	-	(0.1675)	-	(0.2476)
Renewable Electricity Generation * Quality of Governance	-	0.6173***	-	0.6961***	-	0.0861
	-	(0.1575)	-	(0.1242)	-	(0.1004)
Financial Access * Quality of Governance	-	0.2316	-	2.3697***	-	0.7392
	-	(1.1541)	-	(0.6957)	-	(0.5175)
Policy Stringency * Quality of Governance	-	0.0172	-	-0.4672***	-	0.3001***
	-	(0.1902)	-	(0.1237)	-	(0.0948)
Per Capita Income * Quality of Governance	-	-0.4400***	-	-0.4501***	-	-0.1543
	-	(0.1515)	-	(0.1404)	-	(0.1051)
Urban Population Growth	-	-0.3346	-	-0.0060	-	0.0921
	-	(0.2177)	-	(0.1382)	-	(0.1147)
Renewable Electricity Generation * Quality of Governance * Water Vulnerability	-	-1.7819***	-	-2.0838***	-	-0.2566
	-	(0.5041)	-	(0.3813)	-	(0.3024)
Financial Access * Quality of Governance * Water Vulnerability	-	-0.6253	-	-8.1739***	-	-2.5791
	-	(4.2062)	-	(2.5873)	-	(1.9194)
Policy Stringency * Quality of Governance * Water Vulnerability	-	-0.0910	-	1.6356***	-	-0.8097***

	-	(0.6525)	-	(0.3644)	-	(0.3052)
Per Capita Income * Quality of Governance * Water Vulnerability	-	2.0987***	-	2.5766***	-	0.5881*
	-	(0.4790)	-	(0.4844)	-	(0.3413)
Urban Population Growth * Quality of Governance * Water Vulnerability	-	1.2858*	-	0.2489	-	-0.2446
	-	(0.7515)	-	(0.4315)	-	(0.4072)
Constant	1.3919***	16.0237***	1.0081***	18.4176***	1.1118***	13.9672***
	(0.2890)	(1.5304)	(0.2393)	(1.4279)	(0.3402)	(1.4020)
Wald-chi2	557.0	6763	958.8	23980	619.1	9287
Observations	115	115	115	115	115	115
Heteroskedasticity controlled	Yes	Yes	Yes	Yes	Yes	Yes
Autocorrelation controlled	Yes	Yes	Yes	Yes	Yes	Yes
Cross-sectional correlation controlled	Yes	Yes	Yes	Yes	Yes	Yes
Marginal Impact						
Renewable Electricity Generation	0.0521***	-0.0124	0.0464***	-0.0150**	0.0472***	0.0041
	(0.0045)	(0.0089)	(0.0050)	(0.0069)	(0.0069)	(0.0105)
Financial Access	0.0914***	0.0122	0.1454***	0.1333**	0.0905**	0.2044**
	(0.0327)	(0.0830)	(0.0325)	(0.0680)	(0.0427)	(0.0894)
Policy Stringency	0.0075	0.0310**	0.0033	-0.0074	0.0182**	0.0095
	(0.0055)	(0.0145)	(0.0060)	(0.0106)	(0.0071)	(0.0122)
Per Capita Income	0.2671***	0.1909***	0.2969***	0.3197***	0.3139***	0.2546***
	(0.0226)	(0.0405)	(0.0181)	(0.0290)	(0.0269)	(0.0342)
Urban Population Growth	-0.0080	-0.0238	-0.0068	-0.0060	-0.0042	0.0011
	(0.0069)	(0.0160)	(0.0072)	(0.0097)	(0.0083)	(0.0164)

Note:
Standard errors in parentheses ()
*** p<0.01, ** p<0.05, * p<0.1 by Z-test by Z-test

Table S26: Long Run Coefficient Estimation using Generalized Least Square with Water Vulnerability for SDG 7.1.2 – With Regulatory Quality, Rule of Law, and Voice and Accountability, Related to Figure 3

Variables	Model 1	Model 2	Model 1	Model 2	Model 1	Model 2
	Regulatory Quality		Rule of Law		Voice and Accountability	
Renewable Electricity Generation	0.0478***	0.2038***	0.0499***	0.3035***	0.0561***	0.0956*
	(0.0075)	(0.0653)	(0.0046)	(0.0646)	(0.0046)	(0.0574)
Financial Access	0.0966**	0.9401***	0.1288***	1.2789***	0.1143***	-0.6698**
	(0.0379)	(0.3637)	(0.0302)	(0.4216)	(0.0290)	(0.3265)
Policy Stringency	0.0170**	-0.3002***	0.0025	-0.1647***	0.0129**	-0.1185***
	(0.0066)	(0.0469)	(0.0058)	(0.0404)	(0.0059)	(0.0363)
Per Capita Income	0.2598***	-1.5908***	0.3080***	-1.1219***	0.2768***	-1.0301***
	(0.0221)	(0.1495)	(0.0200)	(0.1822)	(0.0159)	(0.1521)
Urban Population Growth	-0.0018	-0.4208***	-0.0073	0.0180	-0.0102	-0.1075**
	(0.0075)	(0.0755)	(0.0069)	(0.0913)	(0.0064)	(0.0463)
Water Vulnerability	-2.0369***	-51.9722***	-1.3649***	-32.5259***	-1.3441***	-38.5575***
	(0.2373)	(4.0158)	(0.2680)	(4.1474)	(0.1861)	(3.6432)
Quality of Governance	0.0153	-8.9711***	-0.0349**	-3.4443***	0.0406***	-2.9752***
	(0.0142)	(1.3311)	(0.0141)	(1.1213)	(0.0148)	(0.3111)
Renewable Electricity Generation * Water Vulnerability	-	-0.6302***	-	-0.9024***	-	-0.3893**
	-	(0.1972)	-	(0.1947)	-	(0.1849)
Financial Access * Water Vulnerability	-	-3.0330**	-	-3.2382**	-	2.6343**
	-	(1.1802)	-	(1.3980)	-	(1.1497)
Policy Stringency * Water Vulnerability	-	0.9670***	-	0.6162***	-	0.3724***
	-	(0.1453)	-	(0.1251)	-	(0.1123)
Per Capita Income * Water Vulnerability	-	5.4180***	-	4.1854***	-	4.0022***
	-	(0.4163)	-	(0.4800)	-	(0.4638)
Urban Population Growth * Water Vulnerability	-	1.2420***	-	-0.0469	-	0.2973*
	-	(0.2381)	-	(0.2802)	-	(0.1552)
Renewable Electricity Generation * Quality of Governance	-	0.8450***	-	0.5602***	-	0.2426***
	-	(0.1656)	-	(0.1744)	-	(0.0819)
Financial Access * Quality of Governance	-	1.3331	-	2.0394**	-	0.2098
	-	(0.9882)	-	(1.0287)	-	(0.2519)
Policy Stringency * Quality of Governance	-	-0.3481	-	-0.2119	-	0.2496***
	-	(0.2139)	-	(0.1435)	-	(0.0406)
Per Capita Income * Quality of Governance	-	-0.0723	-	-0.3853**	-	0.0036
	-	(0.2031)	-	(0.1858)	-	(0.1101)
Urban Population Growth * Quality of Governance	-	-0.3172*	-	0.0053	-	-0.2431***
	-	(0.1902)	-	(0.2080)	-	(0.0484)
Renewable Electricity Generation * Quality of Governance * Water Vulnerability	-	-2.3475***	-	-1.6929***	-	-0.3546
	-	(0.5327)	-	(0.5376)	-	(0.2429)
Financial Access * Quality of Governance * Water Vulnerability	-	-7.0544**	-	-6.3875*	-	-0.4412
	-	(3.5530)	-	(3.8444)	-	(0.8445)
Policy Stringency * Quality of Governance * Water Vulnerability	-	1.3931**	-	0.9642**	-	-0.6528***

	-	(0.6616)	-	(0.4731)	-	(0.1130)
Per Capita Income * Quality of Governance * Water Vulnerability	-	2.5233***	-	2.0545***	-	0.3535
	-	(0.5605)	-	(0.5940)	-	(0.2964)
Urban Population Growth * Quality of Governance * Water Vulnerability	-	1.6926***	-	0.2573	-	0.7748***
	-	(0.6334)	-	(0.6704)	-	(0.1312)
Constant	1.6457***	19.5406***	0.9305***	12.1820***	1.1722***	14.5500***
	(0.2644)	(1.5060)	(0.2503)	(1.6677)	(0.2063)	(1.3188)
Wald-chi2	1007	15978	827.2	4475	4917	22640
Observations	115	115	115	115	115	115
Heteroskedasticity controlled	Yes	Yes	Yes	Yes	Yes	Yes
Autocorrelation controlled	Yes	Yes	Yes	Yes	Yes	Yes
Cross-sectional correlation controlled	Yes	Yes	Yes	Yes	Yes	Yes
Marginal Impact						
Renewable Electricity Generation	0.0478***	0.0033	0.0499***	-0.0324***	0.0561***	-0.0669***
	(0.0075)	(0.0091)	(0.0046)	(0.0109)	(0.0046)	(0.0152)
Financial Access	0.0966**	0.0664	0.1288***	0.0922	0.1143***	0.2580***
	(0.0379)	(0.0558)	(0.0302)	(0.0939)	(0.0290)	(0.0829)
Policy Stringency	0.0170**	0.0129	0.0025	0.0321***	0.0129**	0.0108
	(0.0066)	(0.0107)	(0.0058)	(0.0117)	(0.0059)	(0.0080)
Per Capita Income	0.2598***	0.2204***	0.3080***	0.3018***	0.2768***	0.3693***
	(0.0221)	(0.0274)	(0.0200)	(0.0378)	(0.0159)	(0.0357)
Urban Population Growth	-0.0018	-0.0283***	-0.0073	-0.0227	-0.0102	-0.0084
	(0.0075)	(0.0107)	(0.0069)	(0.0160)	(0.0064)	(0.0112)

Note:
Standard errors in parentheses ()
*** p<0.01, ** p<0.05, * p<0.1 by Z-test by Z-test

Table S27: Long Run Coefficient Estimation using Generalized Least Square with Ecosystem Vulnerability for SDG 7.2.1 – With Control of Corruption, Governance Effectiveness, and Political Stability, Related to Figure 3

Variables	Model 1	Model 2	Model 1	Model 2	Model 1	Model 2
	Control of Corruption		Governance Effectiveness		Political Stability	
Policy Stringency	0.1157	-2.2690	0.1153	-3.9703**	0.0739	-8.1403***
	(0.0979)	(2.3053)	(0.1066)	(1.7660)	(0.1142)	(2.0725)
Environmental Innovation	-0.2960***	-3.1293	-0.2828***	-4.2594**	-0.2112**	-7.0795***
	(0.0859)	(2.6281)	(0.0826)	(2.0070)	(0.1073)	(2.5866)
Financial Development	2.2633***	50.0798***	2.1973***	42.1692***	1.9969**	63.4746***
	(0.6901)	(17.4738)	(0.7354)	(12.7296)	(0.8292)	(17.4865)
Net Energy Import	6.3118***	4.2145	6.9982***	19.1268**	6.5374***	61.1766***
	(0.7443)	(19.1804)	(0.6540)	(8.3295)	(0.7075)	(17.1303)
Low Carbon Trade	-0.1031	2.8134	-0.1163	2.9528	-0.0589	10.0418***
	(0.1001)	(3.5598)	(0.1052)	(1.8820)	(0.1084)	(3.1481)
Ecosystem Vulnerability	-12.7632***	-20.5053	-15.0266***	-26.1323	-8.5762***	-20.8298
	(2.1823)	(26.7063)	(2.1606)	(26.3814)	(2.5657)	(27.5838)
Quality of Governance	-0.3198*	0.3809	-0.3790**	-3.7935	0.0001	-1.6345
	(0.1773)	(2.9909)	(0.1493)	(2.4516)	(0.1321)	(1.8891)
Policy Stringency * Ecosystem Vulnerability	-	4.9040	-	8.1156**	-	18.3607***
	-	(4.8453)	-	(3.6958)	-	(4.3616)
Environmental Innovation * Ecosystem Vulnerability	-	5.8916	-	8.2166**	-	14.7096***
	-	(5.4120)	-	(4.0815)	-	(5.3725)
Financial Development * Ecosystem Vulnerability	-	-103.6655***	-	-86.8768***	-	-135.4462***
	-	(36.4963)	-	(26.8973)	-	(36.8599)
Net Energy Import * Ecosystem Vulnerability	-	5.2965	-	-23.6797	-	-119.7306***
	-	(42.4470)	-	(19.0843)	-	(39.4271)
Low Carbon Trade * Ecosystem Vulnerability	-	-7.1353	-	-7.3812*	-	-20.5125***
	-	(7.2634)	-	(3.8239)	-	(6.3761)
Policy Stringency * Quality of Governance	-	-2.2819	-	-14.8546***	-	-10.2318***
	-	(4.8666)	-	(4.8583)	-	(3.2821)
Environmental Innovation * Quality of Governance	-	-2.0798	-	-4.9207**	-	-4.5919**
	-	(2.7151)	-	(2.4935)	-	(1.9264)
Financial Development * Quality of Governance	-	40.1909	-	85.6976***	-	44.2967**
	-	(30.0064)	-	(28.3506)	-	(21.4089)
Net Energy Import * Quality of Governance	-	1.2672	-	40.7305**	-	55.2834***
	-	(24.6767)	-	(18.9987)	-	(17.5775)
Low Carbon Trade * Quality of Governance	-	1.1847	-	-12.1935*	-	8.3896**
	-	(4.8243)	-	(6.7212)	-	(3.9772)
Policy Stringency * Quality of Governance * Ecosystem Vulnerability	-	4.5996	-	29.2182***	-	22.6185***
	-	(10.4347)	-	(9.7474)	-	(6.6733)
Environmental Innovation * Quality of Governance * Ecosystem Vulnerability	-	5.3056	-	12.8443**	-	10.6432***
	-	(5.8441)	-	(5.3318)	-	(4.1049)
Financial Development * Quality of Governance * Ecosystem Vulnerability	-	-90.5066	-	-182.2497***	-	-96.1673**

	-	(64.8447)	-	(60.3556)	-	(44.3888)
Net Energy Import * Quality of Governance * Ecosystem Vulnerability	-	-12.5842	-	-108.8425**	-	-129.1064***
	-	(55.5412)	-	(42.5514)	-	(40.2947)
Low Carbon Trade * Quality of Governance * Ecosystem Vulnerability	-	-3.4207	-	26.4493*	-	-15.8621**
	-	(10.0336)	-	(13.5700)	-	(8.0324)
Constant	5.1382***	10.8671	6.2065***	13.5535	3.2056**	9.9761
	(1.1833)	(13.1604)	(1.1717)	(12.7742)	(1.4502)	(13.4832)
Wald-chi2	127.7	1152	177.6	1602	141.8	1395
Observations	115	115	115	115	115	115
Heteroskedasticity controlled	Yes	Yes	Yes	Yes	Yes	Yes
Autocorrelation controlled	Yes	Yes	Yes	Yes	Yes	Yes
Cross-sectional correlation controlled	Yes	Yes	Yes	Yes	Yes	Yes
Marginal Impact						
Policy Stringency	0.1157	0.1508	0.1153	0.1879	0.0739	0.2244
	(0.0979)	(0.1787)	(0.1066)	(0.1549)	(0.1142)	(0.1599)
Environmental Innovation	-0.2960***	-0.4175**	-0.2828***	-0.1943	-0.2112**	-0.3242*
	(0.0859)	(0.1830)	(0.0826)	(0.1525)	(0.1073)	(0.1683)
Financial Development	2.2633***	0.6367	2.1973***	-1.2751	1.9969**	-0.3606
	(0.6901)	(1.1127)	(0.7354)	(0.9827)	(0.8292)	(0.9479)
Net Energy Import	6.3118***	8.4105***	6.9982***	6.9722***	6.5374***	7.9337***
	(0.7443)	(1.5297)	(0.6540)	(1.3486)	(0.7075)	(1.0913)
Low Carbon Trade	-0.1031	-0.4986***	-0.1163	-0.4389***	-0.0589	-0.2055*
	(0.1001)	(0.1236)	(0.1052)	(0.1234)	(0.1084)	(0.1191)

Note:
Standard errors in parentheses ()
*** p<0.01, ** p<0.05, * p<0.1 by Z-test by Z-test

Table S28: Long Run Coefficient Estimation using Generalized Least Square with Ecosystem Vulnerability for SDG 7.2.1 – With Regulatory Quality, Rule of Law, and Voice and Accountability, Related to Figure 3

Variables	Model 1	Model 2	Model 1	Model 2	Model 1	Model 2
	Regulatory Quality		Rule of Law		Voice and Accountability	
Policy Stringency	0.1575**	-0.9634	0.0698	-4.0672**	0.0640	1.3884
	(0.0671)	(1.8258)	(0.0586)	(1.7511)	(0.0623)	(1.5577)
Environmental Innovation	-0.1636**	-2.7769	-0.0655	-14.7641***	-0.0127	-3.4067
	(0.0708)	(2.6343)	(0.0693)	(2.2096)	(0.0742)	(2.2275)
Financial Development	0.9961*	53.5937***	0.1768	91.1336***	0.6325	21.0129*
	(0.5117)	(14.8098)	(0.5741)	(12.3073)	(0.5739)	(12.1343)
Net Energy Import	4.7727***	20.6370	2.9144***	40.4801**	3.0100***	28.3339**
	(0.5898)	(14.1181)	(0.6218)	(16.0752)	(0.6237)	(12.9582)
Low Carbon Trade	-0.0690	-0.2152	-0.0686	2.6601	-0.0945	5.2669**
	(0.0749)	(2.4675)	(0.0718)	(3.2806)	(0.0746)	(2.3670)
Ecosystem Vulnerability	-5.0128*	12.4553	5.1314**	-96.0213***	7.6005***	-24.4123
	(2.6465)	(29.4221)	(2.4483)	(30.9145)	(2.0637)	(27.6953)
Quality of Governance	-0.1584	-4.3644	0.3045*	7.2381***	0.4392***	1.0006
	(0.1257)	(3.3268)	(0.1614)	(2.7291)	(0.1370)	(1.1122)
Policy Stringency * Ecosystem Vulnerability	-	2.7401	-	8.1383**	-	-2.6100
	-	(3.7476)	-	(3.5628)	-	(3.2209)
Environmental Innovation * Ecosystem Vulnerability	-	5.2552	-	29.9471***	-	6.9626
	-	(5.4001)	-	(4.4801)	-	(4.5113)
Financial Development * Ecosystem Vulnerability	-	-112.8067***	-	-181.0444***	-	-40.0561
	-	(30.7082)	-	(25.4087)	-	(26.0833)
Net Energy Import * Ecosystem Vulnerability	-	-29.9848	-	-78.9034**	-	-38.6350
	-	(31.6902)	-	(33.0973)	-	(27.1893)
Low Carbon Trade * Ecosystem Vulnerability	-	0.0714	-	-6.6213	-	-12.9951***
	-	(5.0288)	-	(6.6320)	-	(4.8459)
Policy Stringency * Quality of Governance	-	-4.5538	-	-11.4346**	-	-0.0407
	-	(6.4197)	-	(5.5013)	-	(2.9354)
Environmental Innovation * Quality of Governance	-	-5.2513	-	-11.7497***	-	-2.4802
	-	(4.1546)	-	(2.8740)	-	(1.6154)
Financial Development * Quality of Governance	-	95.1226**	-	146.2029***	-	38.5334**
	-	(40.6445)	-	(30.6894)	-	(16.4257)
Net Energy Import * Quality of Governance	-	29.0686	-	-41.1654*	-	-21.4246
	-	(35.6739)	-	(21.6296)	-	(13.0350)
Low Carbon Trade * Quality of Governance	-	-9.9637*	-	-0.2303	-	0.8772
	-	(5.6117)	-	(4.5181)	-	(2.1616)
Policy Stringency * Quality of Governance * Ecosystem Vulnerability	-	8.8983	-	22.3689**	-	0.1418
	-	(12.9177)	-	(11.3507)	-	(5.8809)
Environmental Innovation * Quality of Governance * Ecosystem Vulnerability	-	13.2397	-	22.9141***	-	4.5809
	-	(8.6749)	-	(5.9285)	-	(3.2858)
Financial Development * Quality of Governance * Ecosystem Vulnerability	-	-204.3183**	-	-299.9841***	-	-78.2187**

	-	(83.8350)	-	(65.7105)	-	(34.0019)
Net Energy Import * Quality of Governance * Ecosystem Vulnerability	-	-74.5920	-	72.5636	-	49.3995*
	-	(80.8588)	-	(46.1831)	-	(27.6165)
Low Carbon Trade * Quality of Governance * Ecosystem Vulnerability	-	21.9946*	-	-0.3316	-	-3.0012
	-	(11.5604)	-	(9.1847)	-	(4.3668)
Constant	1.7301	-5.1589	-2.3153*	47.2744***	-3.8180***	9.4919
	(1.3369)	(14.4964)	(1.2417)	(15.2417)	(1.1366)	(13.6910)
Wald-chi2	197.1	569.1	241.7	2126	1001	1407
Observations	115	115	115	115	115	115
Heteroskedasticity controlled	Yes	Yes	Yes	Yes	Yes	Yes
Autocorrelation controlled	Yes	Yes	Yes	Yes	Yes	Yes
Cross-sectional correlation controlled	Yes	Yes	Yes	Yes	Yes	Yes
Marginal Impact						
Policy Stringency	0.1575**	0.3798*	0.0698	0.2690	0.0640	0.1172
	(0.0671)	(0.2022)	(0.0586)	(0.1753)	(0.0623)	(0.1618)
Environmental Innovation	-0.1636**	-0.3772***	-0.0655	0.1683	-0.0127	0.0330
	(0.0708)	(0.1457)	(0.0693)	(0.1510)	(0.0742)	(0.2053)
Financial Development	0.9961*	-0.3878	0.1768	0.1747	0.6325	1.2891
	(0.5117)	(1.0936)	(0.5741)	(1.0693)	(0.5739)	(1.2214)
Net Energy Import	4.7727***	6.9918***	2.9144***	4.6547***	3.0100***	9.1907***
	(0.5898)	(1.3531)	(0.6218)	(1.2598)	(0.6237)	(1.5346)
Low Carbon Trade	-0.0690	-0.2894**	-0.0686	-0.4412***	-0.0945	-0.9236***
	(0.0749)	(0.1174)	(0.0718)	(0.1188)	(0.0746)	(0.1274)
Note:<br>Standard errors in parentheses ()<br>*** p<0.01, ** p<0.05, * p<0.1 by Z-test by Z-test						

Table S29: Long Run Coefficient Estimation using Generalized Least Square with Food Vulnerability for SDG 7.2.1 – With Control of Corruption, Governance Effectiveness, and Political Stability, Related to Figure 3

Variables	Model 1	Model 2	Model 1	Model 2	Model 1	Model 2
	Control of Corruption		Governance Effectiveness		Political Stability	
Policy Stringency	0.1954**	0.0979	0.1505	1.3320*	0.2078**	-3.5332**
	(0.0977)	(1.4221)	(0.0945)	(0.7317)	(0.0911)	(1.5282)
Environmental Innovation	-0.0665	-4.6524***	-0.0541	-3.2152***	-0.0476	-5.2141***
	(0.0964)	(1.3752)	(0.0868)	(0.7228)	(0.0896)	(1.6981)
Financial Development	1.9733**	56.8405***	2.4766***	17.5689***	1.4439**	68.1879***
	(0.7819)	(10.8446)	(0.7987)	(6.1104)	(0.7214)	(12.7452)
Net Energy Import	3.4707***	16.4640	3.3762***	15.7925***	3.1469***	11.7871
	(0.7490)	(12.0381)	(0.6711)	(4.0239)	(0.5787)	(15.8958)
Low Carbon Trade	-0.0229	-0.8536	-0.0346	0.5875	0.0104	7.9448***
	(0.0981)	(2.1953)	(0.1004)	(0.8816)	(0.0917)	(2.9939)
Food Vulnerability	6.2258***	-6.6952	6.7744***	-16.9482	5.6844***	-3.3647
	(1.0403)	(12.5272)	(0.9140)	(11.7284)	(1.0895)	(13.7771)
Quality of Governance	-0.3045	4.7446*	-0.4278***	-4.9144**	-0.0299	3.5505*
	(0.2058)	(2.4348)	(0.1525)	(2.2583)	(0.1018)	(1.8703)
Policy Stringency * Food Vulnerability	-	0.1651	-	-2.4423	-	8.9840***
	-	(3.1441)	-	(1.6109)	-	(3.3968)
Environmental Innovation * Food Vulnerability	-	9.8625***	-	7.3689***	-	9.7322**
	-	(3.1850)	-	(1.6337)	-	(4.2004)
Financial Development * Food Vulnerability	-	-124.3817***	-	-29.5603**	-	-151.4103***
	-	(25.0675)	-	(13.6591)	-	(29.6526)
Net Energy Import * Food Vulnerability	-	-26.6571	-	-30.1717***	-	-9.6324
	-	(30.2639)	-	(10.6189)	-	(43.3287)
Low Carbon Trade * Food Vulnerability	-	0.7637	-	-2.1777	-	-18.8508***
	-	(4.9166)	-	(2.0491)	-	(6.7645)
Policy Stringency * Quality of Governance	-	-1.1447	-	-8.8423***	-	-4.2580**
	-	(3.5445)	-	(2.3116)	-	(2.0364)
Environmental Innovation * Quality of Governance	-	-5.2619**	-	-2.5955	-	-3.6251**
	-	(2.5451)	-	(1.7386)	-	(1.6097)
Financial Development * Quality of Governance	-	91.5255***	-	40.4099***	-	57.5298***
	-	(22.2640)	-	(14.5284)	-	(13.6994)
Net Energy Import * Quality of Governance	-	6.8925	-	42.1497**	-	0.9603
	-	(21.1018)	-	(17.6737)	-	(16.4795)
Low Carbon Trade * Quality of Governance	-	-5.5478	-	-5.8141	-	6.5795**
	-	(3.6963)	-	(3.9710)	-	(3.0396)
Policy Stringency * Quality of Governance * Food Vulnerability	-	2.2877	-	18.6035***	-	10.1407**
	-	(8.2006)	-	(4.8494)	-	(4.2690)
Environmental Innovation * Quality of Governance * Food Vulnerability	-	11.6473*	-	8.7486**	-	6.4406*
	-	(6.2486)	-	(4.3102)	-	(3.8787)
Financial Development * Quality of Governance * Food Vulnerability	-	-232.8937***	-	-100.3180***	-	-132.5898***

	-	(53.6129)	-	(33.6260)	-	(30.3627)
Net Energy Import * Quality of Governance * Food Vulnerability	-	-14.1624	-	-113.1184**	-	3.1681
	-	(56.9872)	-	(47.5775)	-	(44.4042)
Low Carbon Trade * Quality of Governance * Food Vulnerability	-	13.3391	-	16.0142*	-	-14.1709**
	-	(8.6069)	-	(9.2433)	-	(6.5587)
Constant	-4.0430***	3.6752	-4.2927***	5.3424	-3.4483***	4.6004
	(0.8690)	(5.3817)	(0.8699)	(5.2671)	(0.8196)	(5.7461)
Wald-chi2	377.1	960.4	487.7	1546	379.7	1490
Observations	115	115	115	115	115	115
Heteroskedasticity controlled	Yes	Yes	Yes	Yes	Yes	Yes
Autocorrelation controlled	Yes	Yes	Yes	Yes	Yes	Yes
Cross-sectional correlation controlled	Yes	Yes	Yes	Yes	Yes	Yes
Marginal Impact						
Policy Stringency	0.1954**	0.2326	0.1505	0.3755***	0.2078**	0.1594
	(0.0977)	(0.1671)	(0.0945)	(0.1361)	(0.0911)	(0.1790)
Environmental Innovation	-0.0665	-0.1600	-0.0541	0.0984	-0.0476	-0.5677***
	(0.0964)	(0.1403)	(0.0868)	(0.1047)	(0.0896)	(0.1228)
Financial Development	1.9733**	4.1260***	2.4766***	3.8401***	1.4439**	3.8039***
	(0.7819)	(1.0013)	(0.7987)	(0.9062)	(0.7214)	(1.0223)
Net Energy Import	3.4707***	4.3197***	3.3762***	1.7517*	3.1469***	6.1407***
	(0.7490)	(1.4088)	(0.6711)	(0.9768)	(0.5787)	(1.3507)
Low Carbon Trade	-0.0229	-0.5503***	-0.0346	-0.2888***	0.0104	-0.3613**
	(0.0981)	(0.1243)	(0.1004)	(0.1121)	(0.0917)	(0.1415)

Note:
Standard errors in parentheses ()
*** p<0.01, ** p<0.05, * p<0.1 by Z-test by Z-test

Table S30: Long Run Coefficient Estimation using Generalized Least Square with Food Vulnerability for SDG 7.2.1 – With Regulatory Quality, Rule of Law, and Voice and Accountability, Related to Figure 3

Variables	Model 1	Model 2	Model 1	Model 2	Model 1	Model 2
	Regulatory Quality		Rule of Law		Voice and Accountability	
Policy Stringency	0.2087**	-0.8195	0.1880**	2.0718*	0.1881**	0.7878
	(0.0895)	(1.2982)	(0.0945)	(1.2115)	(0.0762)	(0.8277)
Environmental Innovation	-0.0944	-4.0097***	0.0250	-3.8233***	0.0715	-1.4983*
	(0.0892)	(1.3280)	(0.0980)	(1.0458)	(0.1090)	(0.8890)
Financial Development	1.5289**	31.9527***	1.6785**	26.4577***	0.8707	-4.0788
	(0.6845)	(8.8492)	(0.7653)	(8.0222)	(0.5931)	(8.2171)
Net Energy Import	3.6018***	6.2690	2.6807***	4.7806	3.0259***	17.8907**
	(0.5211)	(10.1207)	(0.6794)	(7.6842)	(0.5831)	(7.0966)
Low Carbon Trade	-0.0125	7.0970***	-0.0069	3.3480**	-0.0016	-0.0153
	(0.0892)	(2.3330)	(0.0944)	(1.3940)	(0.0811)	(1.2335)
Food Vulnerability	4.2912***	-22.3837	5.9702***	-22.1349	3.0125***	-14.7352
	(1.0283)	(15.3801)	(1.0886)	(17.5860)	(0.9900)	(12.7618)
Quality of Governance	-0.1822	3.4780	0.3322	1.0830	0.3824**	0.0479
	(0.1804)	(2.9759)	(0.2173)	(3.4034)	(0.1821)	(1.1527)
Policy Stringency * Food Vulnerability	-	2.1644	-	-4.2530*	-	-1.1249
	-	(2.9488)	-	(2.5273)	-	(1.9092)
Environmental Innovation * Food Vulnerability	-	8.2047***	-	8.3551***	-	1.5604
	-	(3.1559)	-	(2.2753)	-	(2.0911)
Financial Development * Food Vulnerability	-	-66.6953***	-	-49.4828***	-	30.3052
	-	(20.3012)	-	(16.9441)	-	(20.7155)
Net Energy Import * Food Vulnerability	-	-1.9969	-	-2.6906	-	-19.0768
	-	(26.8738)	-	(17.3366)	-	(16.9377)
Low Carbon Trade * Food Vulnerability	-	-16.6313***	-	-7.9412**	-	-2.2763
	-	(5.3519)	-	(3.0951)	-	(2.7456)
Policy Stringency * Quality of Governance	-	-10.6993***	-	10.2222***	-	3.8553**
	-	(3.4325)	-	(3.7824)	-	(1.5553)
Environmental Innovation * Quality of Governance	-	-2.9093	-	-5.4877**	-	-6.6452***
	-	(3.1638)	-	(2.1955)	-	(1.2281)
Financial Development * Quality of Governance	-	67.5971***	-	71.1690***	-	89.9167***
	-	(22.7512)	-	(23.5198)	-	(15.3345)
Net Energy Import * Quality of Governance	-	4.3145	-	-16.6928	-	12.6998
	-	(29.1961)	-	(13.5406)	-	(8.9312)
Low Carbon Trade * Quality of Governance	-	10.0832*	-	-2.0832	-	-1.9323
	-	(5.1755)	-	(2.6043)	-	(1.2540)
Policy Stringency * Quality of Governance * Food Vulnerability	-	22.6499***	-	-25.3130***	-	-9.0998**
	-	(7.5836)	-	(8.9103)	-	(3.8099)
Environmental Innovation * Quality of Governance * Food Vulnerability	-	6.0882	-	12.8515***	-	16.4801***
	-	(7.6837)	-	(4.9366)	-	(3.1290)
Financial Development * Quality of Governance * Food Vulnerability	-	-171.4746***	-	-175.0425***	-	-231.6026***

	-	(51.2202)	-	(60.6630)	-	(38.7046)
Net Energy Import * Quality of Governance * Food Vulnerability	-	-3.5231	-	42.7173	-	-21.1081
	-	(79.6737)	-	(35.5285)	-	(24.6256)
Low Carbon Trade * Quality of Governance * Food Vulnerability	-	-20.3794*	-	6.0847	-	3.3076
	-	(11.4991)	-	(6.2141)	-	(2.9205)
Constant	-2.7486***	10.2340	-3.8961***	7.7173	-2.5986***	6.0465
	(0.8034)	(6.5180)	(0.8613)	(8.0445)	(0.7250)	(5.5921)
Wald-chi2	377.3	1111	458.0	864.1	430.6	2025
Observations	115	115	115	115	115	115
Heteroskedasticity controlled	Yes	Yes	Yes	Yes	Yes	Yes
Autocorrelation controlled	Yes	Yes	Yes	Yes	Yes	Yes
Cross-sectional correlation controlled	Yes	Yes	Yes	Yes	Yes	Yes
Marginal Impact						
Policy Stringency	0.2087**	0.1861	0.1880**	-0.0354	0.1881**	-0.0028
	(0.0895)	(0.1682)	(0.0945)	(0.1647)	(0.0762)	(0.1348)
Environmental Innovation	-0.0944	-0.3523***	0.0250	0.0825	0.0715	-0.3470*
	(0.0892)	(0.1230)	(0.0980)	(0.1323)	(0.1090)	(0.2015)
Financial Development	1.5289**	3.5066***	1.6785**	2.8383**	0.8707	3.6353***
	(0.6845)	(1.0188)	(0.7653)	(1.1497)	(0.5931)	(1.1597)
Net Energy Import	3.6018***	5.0800***	2.6807***	3.7958***	3.0259***	7.9793***
	(0.5211)	(1.4615)	(0.6794)	(1.3154)	(0.5831)	(1.5428)
Low Carbon Trade	-0.0125	-0.3760***	-0.0069	-0.2319*	-0.0016	-0.8111***
	(0.0892)	(0.1248)	(0.0944)	(0.1333)	(0.0811)	(0.1348)

Note:
Standard errors in parentheses ()
*** p<0.01, ** p<0.05, * p<0.1 by Z-test by Z-test

Table S31: Long Run Coefficient Estimation using Generalized Least Square with Health Vulnerability for SDG 7.2.1 – With Control of Corruption, Governance Effectiveness, and Political Stability, Related to Figure 3

Variables	Model 1	Model 2	Model 1	Model 2	Model 1	Model 2
	Control of Corruption		Governance Effectiveness		Political Stability	
Policy Stringency	0.2120**	0.2309	0.1932**	-0.1278	0.2011**	-0.7847
	(0.0944)	(1.2011)	(0.0952)	(0.9346)	(0.0884)	(1.1804)
Environmental Innovation	-0.0710	0.1680	-0.0903	-1.2349**	-0.0778	0.3818
	(0.1038)	(0.7868)	(0.0970)	(0.5949)	(0.0947)	(0.8761)
Financial Development	2.4004***	18.0698**	2.8978***	4.8811	1.9783***	17.3680**
	(0.7706)	(7.8734)	(0.7982)	(5.4995)	(0.7485)	(7.0438)
Net Energy Import	3.4847***	-14.0082	3.5367***	6.6363**	3.2355***	-18.1515**
	(0.7926)	(8.9248)	(0.7187)	(3.3745)	(0.5864)	(9.1740)
Low Carbon Trade	-0.1258	1.9819	-0.1497	1.2994*	-0.1096	3.5042**
	(0.1005)	(1.2821)	(0.1027)	(0.7806)	(0.0976)	(1.3822)
Health Vulnerability	4.8535***	3.0836	4.9558***	-8.9500	4.9554***	2.1396
	(0.7341)	(9.0025)	(0.6827)	(9.8424)	(0.7199)	(7.6173)
Quality of Governance	-0.2865	0.1272	-0.2699	-6.2302**	-0.0937	-1.5387
	(0.2129)	(2.1602)	(0.1679)	(3.1263)	(0.1102)	(1.8283)
Policy Stringency * Health Vulnerability	-	-0.6765	-	0.8178	-	1.8571
	-	(2.4085)	-	(1.8849)	-	(2.3851)
Environmental Innovation * Health Vulnerability	-	-0.9422	-	2.4509*	-	-2.4449
	-	(1.8775)	-	(1.2527)	-	(2.1886)
Financial Development * Health Vulnerability	-	-27.6934	-	1.1364	-	-27.2965*
	-	(17.0610)	-	(11.4721)	-	(14.8013)
Net Energy Import * Health Vulnerability	-	45.5497**	-	-6.9380	-	66.1425**
	-	(22.9826)	-	(9.0565)	-	(25.6958)
Low Carbon Trade * Health Vulnerability	-	-5.5251**	-	-3.6648**	-	-7.8498***
	-	(2.7478)	-	(1.6054)	-	(2.8144)
Policy Stringency * Quality of Governance	-	0.8748	-	-3.4586*	-	-0.8604
	-	(2.4900)	-	(2.0823)	-	(1.7707)
Environmental Innovation * Quality of Governance	-	1.1061	-	-1.3391	-	0.9389
	-	(1.6705)	-	(1.5183)	-	(0.9022)
Financial Development * Quality of Governance	-	14.9887	-	17.2092	-	9.4931
	-	(13.1008)	-	(13.1176)	-	(7.7435)
Net Energy Import * Quality of Governance	-	-31.3434*	-	21.0859	-	-19.6020**
	-	(18.2387)	-	(15.2543)	-	(9.5319)
Low Carbon Trade * Quality of Governance	-	0.9127	-	0.7734	-	2.7182*
	-	(2.0609)	-	(3.1540)	-	(1.4976)
Policy Stringency * Quality of Governance * Health Vulnerability	-	-3.1568	-	4.4302	-	2.1034
	-	(5.3738)	-	(3.8825)	-	(3.3353)
Environmental Innovation * Quality of Governance * Health Vulnerability	-	-3.2553	-	6.8032*	-	-3.4008
	-	(4.2181)	-	(3.6175)	-	(2.1044)
Financial Development * Quality of Governance * Health Vulnerability	-	-36.2137	-	-50.3782*	-	-16.2113

	-	(30.5984)	-	(28.6208)	-	(14.9754)
Net Energy Import * Quality of Governance * Health Vulnerability	-	82.3609	-	-61.7028	-	60.7520**
	-	(51.2383)	-	(42.7953)	-	(26.0595)
Low Carbon Trade * Quality of Governance * Health Vulnerability	-	-2.5092	-	-0.7861	-	-5.8714**
	-	(4.6345)	-	(6.8968)	-	(2.9045)
Constant	-3.5084***	-4.0483	-3.4239***	2.1117	-3.0581***	-3.5542
	(0.8289)	(4.2476)	(0.8546)	(4.7877)	(0.8101)	(3.4099)
Wald-chi2	498.1	1037	500.4	1390	845.7	2461
Observations	115	115	115	115	115	115
Heteroskedasticity controlled	Yes	Yes	Yes	Yes	Yes	Yes
Autocorrelation controlled	Yes	Yes	Yes	Yes	Yes	Yes
Cross-sectional correlation controlled	Yes	Yes	Yes	Yes	Yes	Yes
Marginal Impact						
Policy Stringency	0.2120**	0.0917	0.1932**	0.3017*	0.2011**	-0.0199
	(0.0944)	(0.1544)	(0.0952)	(0.1780)	(0.0884)	(0.1541)
Environmental Innovation	-0.0710	-0.1608	-0.0903	-0.0618	-0.0778	-0.3425***
	(0.1038)	(0.1475)	(0.0970)	(0.1175)	(0.0947)	(0.1054)
Financial Development	2.4004***	5.4568***	2.8978***	5.0970***	1.9783***	3.8289***
	(0.7706)	(1.0499)	(0.7982)	(1.0894)	(0.7485)	(1.0370)
Net Energy Import	3.4847***	5.5978***	3.5367***	3.0212***	3.2355***	6.8303***
	(0.7926)	(1.6232)	(0.7187)	(1.1417)	(0.5864)	(1.1568)
Low Carbon Trade	-0.1258	-0.5274***	-0.1497	-0.4177***	-0.1096	-0.0489
	(0.1005)	(0.1147)	(0.1027)	(0.1082)	(0.0976)	(0.1121)

Note:
Standard errors in parentheses ()
*** p<0.01, ** p<0.05, * p<0.1 by Z-test

Table S32: Long Run Coefficient Estimation using Generalized Least Square with Health Vulnerability for SDG 7.2.1 – With Regulatory Quality, Rule of Law, and Voice and Accountability, Related to Figure 3

Variables	Model 1	Model 2	Model 1	Model 2	Model 1	Model 2
	Regulatory Quality		Rule of Law		Voice and Accountability	
Policy Stringency	0.2077**	1.1531	0.1690*	1.0256	0.1796**	-0.4102
	(0.0897)	(1.0541)	(0.0936)	(1.0249)	(0.0794)	(0.8120)
Environmental Innovation	-0.1088	-1.4418*	0.0351	-1.8246***	0.0412	-0.1332
	(0.0987)	(0.7374)	(0.1053)	(0.6218)	(0.0892)	(0.6907)
Financial Development	1.7702**	12.7011**	2.6358***	18.5841***	1.8040***	4.4383
	(0.7053)	(6.4372)	(0.8056)	(6.1334)	(0.6986)	(4.4885)
Net Energy Import	3.8212***	1.1803	2.7066***	2.7095	2.6541***	11.9780**
	(0.5604)	(6.0993)	(0.7162)	(5.3267)	(0.5691)	(5.3999)
Low Carbon Trade	-0.0978	1.5615	-0.1690*	0.6587	-0.0113	-1.8514***
	(0.0961)	(1.1812)	(0.1010)	(0.8840)	(0.0922)	(0.6974)
Health Vulnerability	3.6176***	-6.8516	4.8763***	-5.8755	3.8055***	7.7338
	(0.6847)	(9.4606)	(0.7412)	(9.0001)	(0.5831)	(8.8185)
Quality of Governance	-0.2197	-0.5147	0.4489*	2.7031	0.5119***	-0.4987
	(0.1919)	(2.8058)	(0.2387)	(2.9221)	(0.1408)	(1.3219)
Policy Stringency * Health Vulnerability	-	-1.8776	-	-1.9858	-	1.0820
	-	(2.1764)	-	(1.7992)	-	(1.5158)
Environmental Innovation * Health Vulnerability	-	3.1270*	-	3.5510***	-	-0.7502
	-	(1.7837)	-	(1.1934)	-	(1.2496)
Financial Development * Health Vulnerability	-	-18.9901	-	-30.2754***	-	5.3078
	-	(13.9616)	-	(10.7232)	-	(9.7288)
Net Energy Import * Health Vulnerability	-	2.2100	-	4.7233	-	-11.5663
	-	(16.9078)	-	(10.1637)	-	(10.8781)
Low Carbon Trade * Health Vulnerability	-	-3.6045	-	-1.7015	-	2.0029
	-	(2.5252)	-	(1.6925)	-	(1.3358)
Policy Stringency * Quality of Governance	-	2.6895	-	6.5048***	-	2.3134**
	-	(2.5316)	-	(2.1556)	-	(1.0581)
Environmental Innovation * Quality of Governance	-	-2.6785	-	-5.1867***	-	-2.7400***
	-	(1.7931)	-	(1.2098)	-	(0.6537)
Financial Development * Quality of Governance	-	34.0896**	-	52.0920***	-	33.0084***
	-	(16.6839)	-	(15.8880)	-	(8.3502)
Net Energy Import * Quality of Governance	-	7.5877	-	6.2817	-	8.9206
	-	(16.4152)	-	(12.7470)	-	(7.0788)
Low Carbon Trade * Quality of Governance	-	-0.6058	-	-0.9021	-	-2.5677***
	-	(2.7708)	-	(1.5893)	-	(0.8177)
Policy Stringency * Quality of Governance * Health Vulnerability	-	-6.1774	-	-14.6809***	-	-4.7526**
	-	(5.1498)	-	(4.3331)	-	(2.3172)
Environmental Innovation * Quality of Governance * Health Vulnerability	-	7.6475*	-	11.9126***	-	6.9811***
	-	(4.2064)	-	(2.6804)	-	(1.8641)
Financial Development * Quality of Governance * Health Vulnerability	-	-85.3677**	-	-132.9519***	-	-82.7552***

	-	(35.9051)	-	(39.1474)	-	(20.1453)
Net Energy Import * Quality of Governance * Health Vulnerability	-	-31.3753	-	-13.1708	-	-18.9759
	-	(47.2415)	-	(36.2272)	-	(19.9670)
Low Carbon Trade * Quality of Governance * Health Vulnerability	-	2.4953	-	1.5202	-	3.8710**
	-	(5.9636)	-	(3.3186)	-	(1.6883)
Constant	-2.3924***	1.1930	-3.7359***	0.3341	-2.9252***	-4.0478
	(0.7864)	(4.1377)	(0.8331)	(4.8383)	(0.7204)	(4.9086)
Wald-chi2	717.7	1072	698.0	1472	815.9	3549
Observations	115	115	115	115	115	115
Heteroskedasticity controlled	Yes	Yes	Yes	Yes	Yes	Yes
Autocorrelation controlled	Yes	Yes	Yes	Yes	Yes	Yes
Cross-sectional correlation controlled	Yes	Yes	Yes	Yes	Yes	Yes
Marginal Impact						
Policy Stringency	0.2077**	0.3124*	0.1690*	-0.1721	0.1796**	-0.1054
	(0.0897)	(0.1636)	(0.0936)	(0.1670)	(0.0794)	(0.1486)
Environmental Innovation	-0.1088	-0.0998	0.0351	0.0241	0.0412	-0.3247
	(0.0987)	(0.1305)	(0.1053)	(0.1393)	(0.0892)	(0.2377)
Financial Development	1.7702**	4.6660***	2.6358***	3.9809***	1.8040***	4.9301***
	(0.7053)	(0.9822)	(0.8056)	(1.0695)	(0.6986)	(1.1750)
Net Energy Import	3.8212***	3.0583*	2.7066***	4.5356***	2.6541***	5.8609***
	(0.5604)	(1.5868)	(0.7162)	(1.2949)	(0.5691)	(1.5036)
Low Carbon Trade	-0.0978	-0.1844*	-0.1690*	-0.0395	-0.0113	-0.6166***
	(0.0961)	(0.1012)	(0.1010)	(0.1342)	(0.0922)	(0.1415)

Note:
Standard errors in parentheses ()
*** p<0.01, ** p<0.05, * p<0.1 by Z-test

Table S33: Long Run Coefficient Estimation using Generalized Least Square with Infrastructure Vulnerability for SDG 7.2.1 – With Control of Corruption, Governance Effectiveness, and Political Stability, Related to Figure 3

Variables	Model 1	Model 2	Model 1	Model 2	Model 1	Model 2
	Control of Corruption		Governance Effectiveness		Political Stability	
Policy Stringency	0.3040***	-0.3166	0.3065***	0.5433	0.2855***	-0.4276
	(0.0986)	(0.6682)	(0.0965)	(0.4904)	(0.0936)	(0.5847)
Environmental Innovation	-0.2098**	-0.5043	-0.2165**	-0.5641	-0.2016**	-0.2882
	(0.0982)	(0.5013)	(0.0904)	(0.4001)	(0.0877)	(0.4882)
Financial Development	1.8483**	10.2262**	2.1723***	5.2225*	1.4471*	7.9343*
	(0.7982)	(4.3438)	(0.7902)	(2.8484)	(0.7708)	(4.5700)
Net Energy Import	3.1500***	-4.2013	3.0182***	4.1953**	3.2363***	0.6999
	(0.8262)	(4.5389)	(0.7406)	(1.7146)	(0.5574)	(3.0698)
Low Carbon Trade	-0.0650	-1.1743*	-0.0704	0.1463	-0.0957	-0.7301
	(0.1008)	(0.6643)	(0.1008)	(0.3869)	(0.0939)	(0.7483)
Infrastructure Vulnerability	10.3153***	1.1011	10.9656***	5.9420	9.0386***	19.0120
	(1.6571)	(17.8274)	(1.5210)	(17.6899)	(1.4963)	(16.2603)
Quality of Governance	-0.2555	3.1266	-0.1709	-1.3239	-0.1204	1.7826
	(0.2194)	(2.7247)	(0.1734)	(2.2200)	(0.1155)	(1.7159)
Policy Stringency * Infrastructure Vulnerability	-	2.1540	-	-0.9213	-	4.7512
	-	(4.5470)	-	(2.6198)	-	(3.5234)
Environmental Innovation * Infrastructure Vulnerability	-	-0.0241	-	2.3132	-	-2.6540
	-	(3.5383)	-	(2.2081)	-	(3.6053)
Financial Development * Infrastructure Vulnerability	-	-31.5010	-	-10.1148	-	-14.9918
	-	(28.3532)	-	(15.7560)	-	(28.8173)
Net Energy Import * Infrastructure Vulnerability	-	46.9893	-	-8.0364	-	26.2264
	-	(34.0947)	-	(14.1265)	-	(30.6403)
Low Carbon Trade * Infrastructure Vulnerability	-	5.0308	-	-2.8120	-	2.2066
	-	(4.6852)	-	(2.3227)	-	(4.9618)
Policy Stringency * Quality of Governance	-	-1.4902	-	-1.8347	-	-1.2905
	-	(1.5467)	-	(1.5126)	-	(0.9425)
Environmental Innovation * Quality of Governance	-	0.3324	-	0.3333	-	-0.1869
	-	(0.6801)	-	(0.6769)	-	(0.4078)
Financial Development * Quality of Governance	-	1.7039	-	-1.5452	-	4.2363
	-	(7.2099)	-	(8.3829)	-	(5.6198)
Net Energy Import * Quality of Governance	-	-14.5240*	-	-0.9274	-	-4.0645
	-	(8.0893)	-	(5.7676)	-	(3.7347)
Low Carbon Trade * Quality of Governance	-	-1.4690	-	-1.0039	-	-0.9152
	-	(1.0544)	-	(1.4645)	-	(0.8286)
Policy Stringency * Quality of Governance * Infrastructure Vulnerability	-	5.7440	-	6.3260	-	8.1379
	-	(11.4794)	-	(7.8492)	-	(5.0485)
Environmental Innovation * Quality of Governance * Infrastructure Vulnerability	-	-5.4540	-	1.6935	-	-3.2276
	-	(6.3692)	-	(4.7365)	-	(2.8734)
Financial Development * Quality of Governance * Infrastructure Vulnerability	-	-13.4127	-	-3.0990	-	-3.2333

	-	(54.4538)	-	(50.1671)	-	(31.3605)
Net Energy Import * Quality of Governance * Infrastructure Vulnerability	-	92.6206	-	-21.5676	-	36.2334
	-	(76.4087)	-	(49.6245)	-	(35.7314)
Low Carbon Trade * Quality of Governance * Infrastructure Vulnerability	-	9.7343	-	7.8130	-	4.5157
	-	(8.0378)	-	(9.0737)	-	(5.1297)
Constant	-1.5338**	0.5230	-1.5042**	-1.3305	-1.0537	-2.0278
	(0.7056)	(3.5406)	(0.7204)	(3.4644)	(0.6949)	(3.0773)
Wald-chi2	413.5	1472	476.2	1118	795.3	1290
Observations	115	115	115	115	115	115
Heteroskedasticity controlled	Yes	Yes	Yes	Yes	Yes	Yes
Autocorrelation controlled	Yes	Yes	Yes	Yes	Yes	Yes
Cross-sectional correlation controlled	Yes	Yes	Yes	Yes	Yes	Yes
Marginal Impact						
Policy Stringency	0.3040***	0.2606	0.3065***	0.4452***	0.2855***	0.2703**
	(0.0986)	(0.1592)	(0.0965)	(0.1688)	(0.0936)	(0.1319)
Environmental Innovation	-0.2098**	-0.3512*	-0.2165**	-0.1910	-0.2016**	-0.2975**
	(0.0982)	(0.1822)	(0.0904)	(0.1591)	(0.0877)	(0.1397)
Financial Development	1.8483**	5.2775***	2.1723***	3.6117***	1.4471*	3.4876***
	(0.7982)	(0.9957)	(0.7902)	(1.0459)	(0.7708)	(0.8610)
Net Energy Import	3.1500***	3.7360***	3.0182***	2.8246***	3.2363***	3.6769***
	(0.8262)	(1.2231)	(0.7406)	(1.0216)	(0.5574)	(0.9979)
Low Carbon Trade	-0.0650	-0.3455**	-0.0704	-0.2555**	-0.0957	-0.3002***
	(0.1008)	(0.1438)	(0.1008)	(0.1300)	(0.0939)	(0.1025)
Note:<br>Standard errors in parentheses ()<br>*** p<0.01, ** p<0.05, * p<0.1 by Z-test						

Table S34: Long Run Coefficient Estimation using Generalized Least Square with Infrastructure Vulnerability for SDG 7.2.1 – With Regulatory Quality, Rule of Law, and Voice and Accountability, Related to Figure 3

Variables	Model 1	Model 2	Model 1	Model 2	Model 1	Model 2
	Regulatory Quality		Rule of Law		Voice and Accountability	
Policy Stringency	0.2581***	1.1683***	0.2772***	0.4264	0.2289***	1.0154*
	(0.0932)	(0.3729)	(0.0989)	(0.5416)	(0.0734)	(0.5539)
Environmental Innovation	-0.2043**	-1.0256***	-0.1508	-1.0683**	0.0787	-0.1732
	(0.0917)	(0.3411)	(0.1024)	(0.5018)	(0.0889)	(0.7041)
Financial Development	1.3085*	6.0803***	2.2673***	6.8856*	1.4146**	7.3162**
	(0.7342)	(2.2576)	(0.7705)	(4.0810)	(0.6077)	(3.1986)
Net Energy Import	3.6639***	-1.0541	2.4426***	7.5676**	2.0832***	1.0341
	(0.6027)	(2.0214)	(0.6801)	(3.8564)	(0.5753)	(3.9537)
Low Carbon Trade	-0.0680	-0.2565	-0.1052	-0.8671	-0.0128	-0.7997
	(0.0938)	(0.4320)	(0.0991)	(0.5791)	(0.0785)	(0.4872)
Infrastructure Vulnerability	6.9752***	-14.4458	10.7504***	4.8881	6.6242***	-2.6875
	(1.5616)	(11.7891)	(1.5968)	(23.0002)	(1.0598)	(24.5843)
Quality of Governance	-0.0823	-5.1208*	0.2577	0.8683	0.6882***	2.1747
	(0.2015)	(2.7110)	(0.2498)	(3.4554)	(0.1330)	(1.3937)
Policy Stringency * Infrastructure Vulnerability	-	-3.9166*	-	-1.0037	-	-3.9098
	-	(2.1468)	-	(2.6813)	-	(3.0374)
Environmental Innovation * Infrastructure Vulnerability	-	5.6464**	-	3.8080	-	0.3327
	-	(2.4136)	-	(2.6198)	-	(3.3463)
Financial Development * Infrastructure Vulnerability	-	-19.5194	-	-28.5379	-	-0.8933
	-	(14.4193)	-	(19.5225)	-	(18.7506)
Net Energy Import * Infrastructure Vulnerability	-	0.0200	-	-17.9874	-	14.9544
	-	(19.7393)	-	(21.5198)	-	(19.5763)
Low Carbon Trade * Infrastructure Vulnerability	-	1.8737	-	4.1849	-	-0.1511
	-	(2.9595)	-	(3.2474)	-	(3.1137)
Policy Stringency * Quality of Governance	-	-1.1728	-	0.7193	-	-2.4800***
	-	(0.9662)	-	(1.3459)	-	(0.7864)
Environmental Innovation * Quality of Governance	-	0.7045	-	-1.7704**	-	0.4987
	-	(0.7184)	-	(0.7699)	-	(0.4673)
Financial Development * Quality of Governance	-	9.4905	-	14.5675**	-	-3.6800
	-	(6.0977)	-	(7.1882)	-	(4.4566)
Net Energy Import * Quality of Governance	-	-9.7099*	-	9.4619	-	-5.2364*
	-	(5.8057)	-	(6.1339)	-	(3.1393)
Low Carbon Trade * Quality of Governance	-	-0.9371	-	-0.7826	-	-0.2717
	-	(0.9456)	-	(0.8947)	-	(0.4986)
Policy Stringency * Quality of Governance * Infrastructure Vulnerability	-	1.1490	-	-6.7715	-	13.2415***
	-	(6.5082)	-	(8.8433)	-	(4.5039)
Environmental Innovation * Quality of Governance * Infrastructure Vulnerability	-	3.1531	-	15.4141***	-	-2.8129
	-	(5.7720)	-	(5.1270)	-	(2.9283)
Financial Development * Quality of Governance * Infrastructure Vulnerability	-	-47.8083	-	-154.3923***	-	17.2959

	-	(38.2832)	-	(58.0260)	-	(30.7462)
Net Energy Import * Quality of Governance * Infrastructure Vulnerability	-	-0.2313	-	-64.9882	-	24.8683
	-	(62.8180)	-	(54.4352)	-	(20.0646)
Low Carbon Trade * Quality of Governance * Infrastructure Vulnerability	-	11.3215	-	3.0684	-	-1.4088
	-	(7.0304)	-	(6.0283)	-	(3.0142)
Constant	-0.8201	2.9129	-1.6714**	0.2916	-2.0651***	-2.1158
	(0.6873)	(2.2657)	(0.6830)	(4.6289)	(0.6236)	(5.1422)
Wald-chi2	486.2	3635	592.1	1155	869.4	1554
Observations	115	115	115	115	115	115
Heteroskedasticity controlled	Yes	Yes	Yes	Yes	Yes	Yes
Autocorrelation controlled	Yes	Yes	Yes	Yes	Yes	Yes
Cross-sectional correlation controlled	Yes	Yes	Yes	Yes	Yes	Yes
Marginal Impact						
Policy Stringency	0.2581***	0.6498***	0.2772***	0.2774	0.2289***	0.5658***
	(0.0932)	(0.1253)	(0.0989)	(0.1832)	(0.0734)	(0.1785)
Environmental Innovation	-0.2043**	-0.2714**	-0.1508	-0.4477**	0.0787	-0.1513
	(0.0917)	(0.1185)	(0.1024)	(0.1798)	(0.0889)	(0.2810)
Financial Development	1.3085*	2.9357***	2.2673***	3.1147***	1.4146**	7.4910***
	(0.7342)	(0.7313)	(0.7705)	(0.9485)	(0.6077)	(1.3160)
Net Energy Import	3.6639***	0.0377	2.4426***	4.0658***	2.0832***	3.8645**
	(0.6027)	(1.0494)	(0.6801)	(1.2764)	(0.5753)	(1.9604)
Low Carbon Trade	-0.0680	-0.0965	-0.1052	-0.0727	-0.0128	-0.7328***
	(0.0938)	(0.0954)	(0.0991)	(0.1418)	(0.0785)	(0.1599)

Note:
Standard errors in parentheses ()
*** $p<0.01$, ** $p<0.05$, * $p<0.1$ by Z-test

Table S35: Long Run Coefficient Estimation using Generalized Least Square with Water Vulnerability for SDG 7.2.1 – With Control of Corruption, Governance Effectiveness, and Political Stability, Related to Figure 3

Variables	Model 1	Model 2	Model 1	Model 2	Model 1	Model 2
	Control of Corruption		Governance Effectiveness		Political Stability	
Policy Stringency	0.1676*	-0.4308	0.1334	0.4322	0.1611*	-1.9267**
	(0.0892)	(1.0207)	(0.0962)	(0.5819)	(0.0863)	(0.9759)
Environmental Innovation	-0.1165	-1.6801	-0.1353	-1.7023**	-0.1095	-2.8499**
	(0.0883)	(1.0407)	(0.0882)	(0.6858)	(0.0872)	(1.2634)
Financial Development	2.0938***	28.5224***	2.5498***	11.1766**	1.6744**	31.7427***
	(0.6810)	(8.2374)	(0.7296)	(4.8305)	(0.6772)	(9.3752)
Net Energy Import	2.6368***	2.0372	3.0567***	10.1881***	2.5201***	31.0288***
	(0.7783)	(10.7326)	(0.7081)	(3.4152)	(0.5971)	(10.8708)
Low Carbon Trade	-0.0109	-0.6849	-0.0784	0.5664	0.0298	-0.9753
	(0.0930)	(1.2559)	(0.0986)	(0.6303)	(0.0899)	(1.2785)
Water Vulnerability	7.1091***	-5.1822	6.4721***	-6.8569	7.3441***	3.3439
	(1.0848)	(13.1585)	(0.9716)	(13.1038)	(1.1452)	(14.8649)
Quality of Governance	-0.1015	0.9227	-0.1747	-5.4484**	0.0569	-2.0756
	(0.1898)	(3.1029)	(0.1453)	(2.7063)	(0.0966)	(2.3244)
Policy Stringency * Water Vulnerability	-	1.2338	-	-0.6508	-	7.8286***
	-	(3.2361)	-	(1.5765)	-	(2.8913)
Environmental Innovation * Water Vulnerability	-	3.7153	-	4.7707**	-	9.1112**
	-	(3.2534)	-	(1.8800)	-	(4.1032)
Financial Development * Water Vulnerability	-	-70.6845***	-	-18.3449	-	-85.5994***
	-	(24.6673)	-	(12.9125)	-	(27.7326)
Net Energy Import * Water Vulnerability	-	14.4333	-	-22.4058**	-	-89.8518**
	-	(36.1038)	-	(11.4002)	-	(38.9630)
Low Carbon Trade * Water Vulnerability	-	-1.3289	-	-2.7777	-	0.2515
	-	(3.8291)	-	(1.7034)	-	(3.5728)
Policy Stringency * Quality of Governance	-	-3.5908	-	-4.7780**	-	-2.5737
	-	(2.9042)	-	(2.0181)	-	(1.5981)
Environmental Innovation * Quality of Governance	-	0.2441	-	-0.2992	-	-1.5457
	-	(1.5687)	-	(1.2057)	-	(0.9749)
Financial Development * Quality of Governance	-	33.7326*	-	20.4877	-	19.4580*
	-	(17.7647)	-	(13.7161)	-	(10.3545)
Net Energy Import * Quality of Governance	-	-21.0545	-	7.0940	-	22.7261*
	-	(25.3449)	-	(13.8487)	-	(12.0547)
Low Carbon Trade * Quality of Governance	-	-1.2216	-	-2.1167	-	-1.7692
	-	(2.1891)	-	(2.3295)	-	(1.5529)
Policy Stringency * Quality of Governance * Water Vulnerability	-	7.5411	-	10.1432*	-	9.1308**
	-	(9.3782)	-	(5.2751)	-	(4.1392)
Environmental Innovation * Quality of Governance * Water Vulnerability	-	-0.6207	-	4.1162	-	6.4606*
	-	(5.9611)	-	(4.5317)	-	(3.3387)
Financial Development * Quality of Governance * Water Vulnerability	-	-120.1657**	-	-66.4115	-	-59.7688**

	-	(58.3102)	-	(42.9958)	-	(28.9464)
Net Energy Import * Quality of Governance * Water Vulnerability	-	74.7964	-	-31.8909	-	-80.2916*
	-	(91.2042)	-	(47.8633)	-	(42.7127)
Low Carbon Trade * Quality of Governance * Water Vulnerability	-	0.6041	-	6.7592	-	2.4034
	-	(7.0180)	-	(6.6179)	-	(4.2705)
Constant	-3.0681***	1.2535	-2.8154***	0.6940	-2.8411***	-2.9070
	(0.6584)	(4.7937)	(0.7199)	(4.9041)	(0.6569)	(5.2329)
Wald-chi2	377.9	744.9	424.0	830.3	396.3	827.7
Observations	115	115	115	115	115	115
Heteroskedasticity controlled	Yes	Yes	Yes	Yes	Yes	Yes
Autocorrelation controlled	Yes	Yes	Yes	Yes	Yes	Yes
Cross-sectional correlation controlled	Yes	Yes	Yes	Yes	Yes	Yes
Marginal Impact						
Policy Stringency	0.1676*	0.3392*	0.1334	0.3093*	0.1611*	0.3110
	(0.0892)	(0.1940)	(0.0962)	(0.1794)	(0.0863)	(0.2064)
Environmental Innovation	-0.1165	-0.3656**	-0.1353	0.0239	-0.1095	-0.1477
	(0.0883)	(0.1817)	(0.0882)	(0.1447)	(0.0872)	(0.1567)
Financial Development	2.0938***	6.2910***	2.5498***	4.0438***	1.6744**	3.3972***
	(0.6810)	(1.0981)	(0.7296)	(0.9782)	(0.6772)	(1.2357)
Net Energy Import	2.6368***	5.3620***	3.0567***	1.9483*	2.5201***	3.8133***
	(0.7783)	(1.3975)	(0.7081)	(1.1065)	(0.5971)	(1.0630)
Low Carbon Trade	-0.0109	-0.8051***	-0.0784	-0.3621***	0.0298	-0.4212***
	(0.0930)	(0.1398)	(0.0986)	(0.1257)	(0.0899)	(0.1428)

Note:
Standard errors in parentheses ()
*** $p<0.01$, ** $p<0.05$, * $p<0.1$ by Z-test

Table S36: Long Run Coefficient Estimation using Generalized Least Square with Water Vulnerability for SDG 7.2.1 – With Regulatory Quality, Rule of Law, and Voice and Accountability, Related to Figure 3

Variables	Model 1	Model 2	Model 1	Model 2	Model 1	Model 2
	Regulatory Quality		Rule of Law		Voice and Accountability	
Policy Stringency	0.1829**	0.9340	0.1573*	0.0717	0.1609**	-0.4048
	(0.0829)	(0.6564)	(0.0832)	(0.6606)	(0.0702)	(0.7325)
Environmental Innovation	-0.1287	-2.1646**	-0.0539	-2.0274***	0.0395	-0.5491
	(0.0812)	(0.9120)	(0.0925)	(0.7554)	(0.0913)	(0.9040)
Financial Development	1.6200***	13.8216**	1.6838***	20.1413***	1.1586**	6.8698
	(0.6147)	(5.7533)	(0.6478)	(5.4938)	(0.5646)	(4.8458)
Net Energy Import	3.3956***	8.5226	2.2149***	6.4538	2.2486***	19.1219***
	(0.5313)	(6.8940)	(0.6274)	(6.8056)	(0.5869)	(5.4090)
Low Carbon Trade	-0.0116	-0.2766	-0.0071	-0.9410	0.0294	-1.3594*
	(0.0838)	(1.0405)	(0.0872)	(0.8604)	(0.0780)	(0.7597)
Water Vulnerability	2.5173*	-11.9913	6.9608***	-9.0279	5.1628***	1.3633
	(1.3851)	(11.4686)	(1.0483)	(14.7176)	(0.8196)	(13.8069)
Quality of Governance	-0.0435	-4.1990	0.2869	-1.0125	0.4752***	0.7564
	(0.1673)	(3.5036)	(0.1818)	(2.7351)	(0.1536)	(1.3343)
Policy Stringency * Water Vulnerability	-	-1.5472	-	-0.5375	-	1.3635
	-	(1.8345)	-	(1.7472)	-	(1.8211)
Environmental Innovation * Water Vulnerability	-	6.0392**	-	5.6755***	-	1.6779
	-	(2.8736)	-	(1.9962)	-	(2.1236)
Financial Development * Water Vulnerability	-	-32.6725*	-	-40.6526***	-	-7.4076
	-	(16.7208)	-	(13.8554)	-	(13.8677)
Net Energy Import * Water Vulnerability	-	-22.5097	-	-12.8602	-	-33.1628**
	-	(24.2354)	-	(17.6690)	-	(14.2089)
Low Carbon Trade * Water Vulnerability	-	0.5751	-	1.4370	-	0.1465
	-	(3.0949)	-	(2.3929)	-	(2.1813)
Policy Stringency * Quality of Governance	-	-2.7684	-	0.8943	-	1.6249
	-	(1.9433)	-	(2.1705)	-	(1.2497)
Environmental Innovation * Quality of Governance	-	-0.9174	-	-1.1695	-	-0.8472
	-	(1.7824)	-	(1.2611)	-	(0.7683)
Financial Development * Quality of Governance	-	26.9956*	-	17.1615	-	4.2352
	-	(14.5384)	-	(16.1281)	-	(8.4170)
Net Energy Import * Quality of Governance	-	8.1764	-	-8.9350	-	2.9611
	-	(21.7899)	-	(16.0223)	-	(5.8017)
Low Carbon Trade * Quality of Governance	-	-2.8713	-	-1.9826	-	-2.3670***
	-	(2.5983)	-	(1.5232)	-	(0.8128)
Policy Stringency * Quality of Governance * Water Vulnerability	-	5.7768	-	-6.0009	-	-4.7901
	-	(5.6005)	-	(6.1929)	-	(3.2102)
Environmental Innovation * Quality of Governance * Water Vulnerability	-	6.7763	-	4.0221	-	2.1353
	-	(6.6254)	-	(3.8455)	-	(2.1809)
Financial Development * Quality of Governance * Water Vulnerability	-	-91.7088**	-	-40.3750	-	-10.1060

	-	(44.3973)	-	(51.4688)	-	(24.3814)
Net Energy Import * Quality of Governance * Water Vulnerability	-	-48.3816	-	21.2750	-	-0.5063
	-	(79.0135)	-	(55.4898)	-	(18.6892)
Low Carbon Trade * Quality of Governance * Water Vulnerability	-	9.7248	-	4.3973	-	3.7381*
	-	(8.2850)	-	(4.3561)	-	(2.1898)
Constant	-1.5524**	4.0788	-2.9531***	1.0801	-2.6421***	-3.4081
	(0.6283)	(4.2879)	(0.6027)	(5.5747)	(0.6105)	(5.8481)
Wald-chi2	175.0	1335	482.1	788.5	795.9	1033
Observations	115	115	115	115	115	115
Heteroskedasticity controlled	Yes	Yes	Yes	Yes	Yes	Yes
Autocorrelation controlled	Yes	Yes	Yes	Yes	Yes	Yes
Cross-sectional correlation controlled	Yes	Yes	Yes	Yes	Yes	Yes
Marginal Impact						
Policy Stringency	0.1829**	0.4005**	0.1573*	0.1441	0.1609**	0.0981
	(0.0829)	(0.1780)	(0.0832)	(0.2080)	(0.0702)	(0.2070)
Environmental Innovation	-0.1287	-0.2522*	-0.0539	-0.0176	0.0395	0.0662
	(0.0812)	(0.1342)	(0.0925)	(0.1700)	(0.0913)	(0.2711)
Financial Development	1.6200***	3.7924***	1.6838***	4.2155***	1.1586**	4.1017***
	(0.6147)	(0.9892)	(0.6478)	(1.0113)	(0.5646)	(1.3450)
Net Energy Import	3.3956***	2.0299*	2.2149***	2.6004**	2.2486***	6.7376***
	(0.5313)	(1.0494)	(0.6274)	(1.2758)	(0.5869)	(1.7051)
Low Carbon Trade	-0.0116	-0.2416**	-0.0071	-0.2392*	0.0294	-1.0958***
	(0.0838)	(0.1224)	(0.0872)	(0.1452)	(0.0780)	(0.1492)

Note:
Standard errors in parentheses ()
*** p<0.01, ** p<0.05, * p<0.1 by Z-test

Table S37: Long Run Coefficient Estimation using Generalized Least Square with Ecosystem Vulnerability for SDG 7.3.1 – With Control of Corruption, Governance Effectiveness, and Political Stability, Related to Figure 3

Variables	Model 1	Model 2	Model 1	Model 2	Model 1	Model 2
	Control of Corruption		Governance Effectiveness		Political Stability	
Policy Stringency	-0.0198	1.7046***	-0.0379**	0.9441***	-0.0408**	0.8080
	(0.0177)	(0.5143)	(0.0175)	(0.2869)	(0.0176)	(0.5644)
Environmental Innovation	-0.0556**	2.0399***	-0.0527**	1.4012***	-0.0779***	0.9379
	(0.0273)	(0.6236)	(0.0259)	(0.3684)	(0.0253)	(0.6356)
Foreign Direct Investment	-0.0232	0.5905	-0.0489	0.6800	-0.0471	-2.8751*
	(0.0364)	(1.4187)	(0.0394)	(0.7236)	(0.0381)	(1.6585)
Industrial Share	0.4885***	6.0450***	0.7693***	1.6085	0.6478***	0.3335
	(0.1027)	(1.4002)	(0.0968)	(1.0991)	(0.0981)	(1.7407)
Energy Demand	-0.0625	-5.3754***	-0.0322	-2.0243***	0.0142	-0.2247
	(0.0472)	(0.6831)	(0.0408)	(0.4316)	(0.0417)	(1.0241)
Ecosystem Vulnerability	0.0331	-4.8063	-0.6220*	-0.1056	-0.0788	6.6664
	(0.3791)	(7.0677)	(0.3446)	(5.7349)	(0.3526)	(7.6479)
Quality of Governance	-0.0730**	0.2423	-0.0435	2.2472***	-0.0210	0.7663
	(0.0337)	(1.0267)	(0.0274)	(0.6910)	(0.0208)	(0.6381)
Policy Stringency * Ecosystem Vulnerability	-	-3.3096***	-	-1.9329***	-	-1.5583
	-	(1.0521)	-	(0.5689)	-	(1.1565)
Environmental Innovation * Ecosystem Vulnerability	-	-3.7872***	-	-2.8894***	-	-1.6316
	-	(1.2465)	-	(0.7114)	-	(1.2735)
Foreign Direct Investment * Ecosystem Vulnerability	-	-1.2017	-	-1.4462	-	6.0075*
	-	(2.8511)	-	(1.4702)	-	(3.3557)
Industrial Share * Ecosystem Vulnerability	-	-9.3780***	-	-1.5231	-	1.8091
	-	(2.6875)	-	(2.1130)	-	(3.5616)
Energy Demand * Ecosystem Vulnerability	-	9.9040***	-	4.0503***	-	-0.2917
	-	(1.3161)	-	(0.8306)	-	(2.0559)
Policy Stringency * Quality of Governance	-	1.9500*	-	1.2806	-	0.0456
	-	(1.0878)	-	(0.9190)	-	(0.7562)
Environmental Innovation * Quality of Governance	-	0.5484	-	4.1587***	-	1.3700
	-	(1.4261)	-	(1.3337)	-	(0.8575)
Foreign Direct Investment * Quality of Governance	-	0.5234	-	3.2025	-	-3.4397
	-	(2.1802)	-	(3.1156)	-	(2.1039)
Industrial Share * Quality of Governance	-	5.1305**	-	1.7254	-	-1.7405
	-	(2.3348)	-	(2.4933)	-	(1.8986)
Energy Demand * Quality of Governance	-	-3.4997**	-	-5.8886***	-	-0.5884
	-	(1.4223)	-	(1.5911)	-	(1.2501)
Policy Stringency * Quality of Governance * Ecosystem Vulnerability	-	-3.7165	-	-1.9352	-	-0.0310
	-	(2.2606)	-	(1.8318)	-	(1.5312)
Environmental Innovation * Quality of Governance * Ecosystem Vulnerability	-	-0.2104	-	-7.7700***	-	-2.0648
	-	(2.8955)	-	(2.6900)	-	(1.6753)
Foreign Direct Investment * Quality of Governance * Ecosystem Vulnerability	-	-1.0132	-	-5.7630	-	7.2999*

	-	(4.5297)	-	(6.2454)	-	(4.2164)
Industrial Share * Quality of Governance * Ecosystem Vulnerability	-	-9.5805**	-	-3.6343	-	3.7906
	-	(4.6932)	-	(4.9134)	-	(3.8569)
Energy Demand * Quality of Governance * Ecosystem Vulnerability	-	5.5148*	-	10.4765***	-	0.0309
	-	(2.8339)	-	(3.2227)	-	(2.4439)
Constant	1.1182***	1.3579	0.3653	-0.5018	0.3811	-4.0423
	(0.3533)	(3.7499)	(0.2455)	(2.9310)	(0.2636)	(3.7466)
Wald-chi2	58.12	2626	232.9	1602	131.5	1310
Observations	115	115	115	115	115	115
Heteroskedasticity controlled	Yes	Yes	Yes	Yes	Yes	Yes
Autocorrelation controlled	Yes	Yes	Yes	Yes	Yes	Yes
Cross-sectional correlation controlled	Yes	Yes	Yes	Yes	Yes	Yes
Marginal Impact						
Policy Stringency	-0.0198	0.0288	-0.0379**	-0.0113	-0.0408**	0.0354
	(0.0177)	(0.0263)	(0.0175)	(0.0237)	(0.0176)	(0.0261)
Environmental Innovation	-0.0556**	0.0460	-0.0527**	-0.0615	-0.0779***	-0.0476
	(0.0273)	(0.0400)	(0.0259)	(0.0426)	(0.0253)	(0.0373)
Foreign Direct Investment	-0.0232	-0.0087	-0.0489	-0.0682	-0.0471	-0.0590
	(0.0364)	(0.0609)	(0.0394)	(0.0513)	(0.0381)	(0.0536)
Industrial Share	0.4885***	1.2784***	0.7693***	0.8435***	0.6478***	1.1348***
	(0.1027)	(0.1043)	(0.0968)	(0.1096)	(0.0981)	(0.1142)
Energy Demand	-0.0625	-0.2552***	-0.0322	0.0258	0.0142	-0.0462
	(0.0472)	(0.0666)	(0.0408)	(0.0611)	(0.0417)	(0.0575)

Note:
Standard errors in parentheses ()
*** p<0.01, ** p<0.05, * p<0.1 by Z-test

Table S38: Long Run Coefficient Estimation using Generalized Least Square with Ecosystem Vulnerability for SDG 7.3.1 – With Regulatory Quality, Rule of Law, and Voice and Accountability, Related to Figure 3

Variables	Model 1	Model 2	Model 1	Model 2	Model 1	Model 2
	Regulatory Quality		Rule of Law		Voice and Accountability	
Policy Stringency	-0.0419**	0.8172**	-0.0202	1.0688***	-0.0140	0.1183
	(0.0180)	(0.3681)	(0.0172)	(0.3097)	(0.0164)	(0.2961)
Environmental Innovation	-0.0772***	0.9190**	-0.0658***	3.6387***	0.0072	0.5490
	(0.0275)	(0.4500)	(0.0248)	(0.3614)	(0.0229)	(0.4155)
Foreign Direct Investment	-0.0606	-1.4148	-0.0234	1.1155	-0.0378	-0.7808
	(0.0408)	(0.9470)	(0.0391)	(0.9828)	(0.0353)	(0.7543)
Industrial Share	0.8882***	1.6507	0.5150***	4.6382***	0.6023***	2.6011**
	(0.0955)	(1.2263)	(0.0971)	(0.9585)	(0.0848)	(1.1139)
Energy Demand	-0.0027	-1.3067*	-0.0828**	-3.3503***	-0.2718***	0.1074
	(0.0459)	(0.6805)	(0.0392)	(0.6154)	(0.0512)	(1.0210)
Ecosystem Vulnerability	-1.0446***	1.8813	-0.6922*	36.9350***	0.6557**	24.3579*
	(0.3239)	(6.6477)	(0.3846)	(7.8795)	(0.3284)	(13.6763)
Quality of Governance	0.0562	0.7817	-0.1139***	1.1356	-0.2272***	-0.3855
	(0.0380)	(0.8192)	(0.0387)	(0.8520)	(0.0360)	(0.3596)
Policy Stringency * Ecosystem Vulnerability	-	-1.6930**	-	-2.0230***	-	-0.2734
	-	(0.7483)	-	(0.6137)	-	(0.5907)
Environmental Innovation * Ecosystem Vulnerability	-	-1.8705**	-	-6.9103***	-	-1.0638
	-	(0.8959)	-	(0.6760)	-	(0.8029)
Foreign Direct Investment * Ecosystem Vulnerability	-	2.8343	-	-2.3314	-	1.5585
	-	(1.9124)	-	(1.9546)	-	(1.5385)
Industrial Share * Ecosystem Vulnerability	-	-1.5351	-	-7.6005***	-	-3.5918
	-	(2.4718)	-	(1.8177)	-	(2.2570)
Energy Demand * Ecosystem Vulnerability	-	2.4787*	-	5.9592***	-	-0.7189
	-	(1.3471)	-	(1.1703)	-	(2.0056)
Policy Stringency * Quality of Governance	-	2.1946	-	1.5859**	-	1.3971***
	-	(1.4307)	-	(0.7679)	-	(0.5376)
Environmental Innovation * Quality of Governance	-	-2.7128*	-	4.6906***	-	1.0657
	-	(1.5585)	-	(0.9675)	-	(0.6809)
Foreign Direct Investment * Quality of Governance	-	-3.4015	-	2.2447	-	0.1681
	-	(2.2736)	-	(1.8279)	-	(0.8936)
Industrial Share * Quality of Governance	-	3.2384	-	6.1913***	-	3.5645**
	-	(2.8197)	-	(1.9125)	-	(1.5533)
Energy Demand * Quality of Governance	-	0.9639	-	-8.3319***	-	-2.9994***
	-	(1.6808)	-	(1.3779)	-	(1.1623)
Policy Stringency * Quality of Governance * Ecosystem Vulnerability	-	-4.3002	-	-2.8665*	-	-2.7918***
	-	(2.8899)	-	(1.5513)	-	(1.0616)
Environmental Innovation * Quality of Governance * Ecosystem Vulnerability	-	5.4574*	-	-8.5055***	-	-2.0387
	-	(3.1027)	-	(1.9094)	-	(1.3090)
Foreign Direct Investment * Quality of Governance * Ecosystem Vulnerability	-	6.9100	-	-4.3443	-	-0.4920

	-	(4.5723)	-	(3.8952)	-	(1.8209)
Industrial Share * Quality of Governance * Ecosystem Vulnerability	-	-6.5997	-	-12.4375***	-	-6.9364**
	-	(5.7823)	-	(3.9273)	-	(3.0207)
Energy Demand * Quality of Governance * Ecosystem Vulnerability	-	-2.1485	-	15.2764***	-	5.8657***
	-	(3.3719)	-	(2.7422)	-	(2.2437)
Constant	0.1591	-1.3581	1.5597***	-18.2553***	1.3406***	-11.3578*
	(0.2547)	(3.3427)	(0.3250)	(4.0618)	(0.2756)	(6.8232)
Wald-chi2	323.8	857.2	170.8	4072	286.3	1088
Observations	115	115	115	115	115	115
Heteroskedasticity controlled	Yes	Yes	Yes	Yes	Yes	Yes
Autocorrelation controlled	Yes	Yes	Yes	Yes	Yes	Yes
Cross-sectional correlation controlled	Yes	Yes	Yes	Yes	Yes	Yes
Marginal Impact						
Policy Stringency	-0.0419**	-0.0101	-0.0202	0.0030	-0.0140	-0.0293
	(0.0180)	(0.0226)	(0.0172)	(0.0247)	(0.0164)	(0.0258)
Environmental Innovation	-0.0772***	0.0117	-0.0658***	0.0404	0.0072	0.0128
	(0.0275)	(0.0400)	(0.0248)	(0.0424)	(0.0229)	(0.0440)
Foreign Direct Investment	-0.0606	-0.0442	-0.0234	-0.0977*	-0.0378	-0.0117
	(0.0408)	(0.0487)	(0.0391)	(0.0545)	(0.0353)	(0.0509)
Industrial Share	0.8882***	0.9112***	0.5150***	0.7839***	0.6023***	0.8026***
	(0.0955)	(0.1048)	(0.0971)	(0.0978)	(0.0848)	(0.1151)
Energy Demand	-0.0027	-0.0930	-0.0828**	-0.0434	-0.2718***	-0.1964**
	(0.0459)	(0.0630)	(0.0392)	(0.0638)	(0.0512)	(0.0867)

Note:
Standard errors in parentheses ()
*** $p<0.01$, ** $p<0.05$, * $p<0.1$ by Z-test

Table S39: Long Run Coefficient Estimation using Generalized Least Square with Food Vulnerability for SDG 7.3.1 – With Control of Corruption, Governance Effectiveness, and Political Stability, Related to Figure 3

Variables	Model 1	Model 2	Model 1	Model 2	Model 1	Model 2
	Control of Corruption		Governance Effectiveness		Political Stability	
Policy Stringency	-0.0301*	0.4841*	-0.0455***	0.2313**	-0.0390**	-0.2850
	(0.0166)	(0.2875)	(0.0165)	(0.0968)	(0.0169)	(0.3661)
Environmental Innovation	-0.0470*	0.3950	-0.0423*	-0.0287	-0.0753***	0.5936
	(0.0251)	(0.2744)	(0.0238)	(0.1521)	(0.0242)	(0.3825)
Foreign Direct Investment	-0.0133	0.2126	-0.0238	0.1643	-0.0414	-1.3873
	(0.0375)	(0.6593)	(0.0387)	(0.2405)	(0.0385)	(1.1649)
Industrial Share	0.7038***	4.2172***	0.6968***	2.2221***	0.7174***	2.8769***
	(0.0872)	(0.6036)	(0.0833)	(0.4861)	(0.0885)	(0.8872)
Energy Demand	-0.0570	-1.4099***	-0.0573	-0.2055	-0.0120	-0.6911
	(0.0435)	(0.3980)	(0.0363)	(0.2475)	(0.0413)	(0.5848)
Food Vulnerability	-0.9847***	11.4657***	-1.2029***	9.1963**	-1.0152***	13.2262**
	(0.1548)	(3.3658)	(0.1210)	(3.6749)	(0.1596)	(5.2783)
Quality of Governance	-0.0436	-1.0856*	-0.0139	1.4969**	-0.0272	0.3291
	(0.0323)	(0.5851)	(0.0255)	(0.6351)	(0.0214)	(0.5345)
Policy Stringency * Food Vulnerability	-	-0.9641	-	-0.6058***	-	0.6537
	-	(0.6464)	-	(0.2027)	-	(0.8188)
Environmental Innovation * Food Vulnerability	-	-0.7097	-	-0.0311	-	-1.2354
	-	(0.6266)	-	(0.3366)	-	(0.9118)
Foreign Direct Investment * Food Vulnerability	-	-0.5859	-	-0.5047	-	2.9141
	-	(1.4428)	-	(0.5099)	-	(2.5709)
Industrial Share * Food Vulnerability	-	-6.0939***	-	-3.2077***	-	-3.4376*
	-	(1.3242)	-	(0.9581)	-	(2.0341)
Energy Demand * Food Vulnerability	-	2.2291***	-	0.2803	-	0.7625
	-	(0.8601)	-	(0.5358)	-	(1.3376)
Policy Stringency * Quality of Governance	-	0.9106	-	0.4412	-	-0.5215
	-	(0.7089)	-	(0.3517)	-	(0.4416)
Environmental Innovation * Quality of Governance	-	0.1337	-	0.8302	-	1.1933***
	-	(0.6576)	-	(0.5858)	-	(0.4584)
Foreign Direct Investment * Quality of Governance	-	0.4634	-	3.0229**	-	-1.4870
	-	(1.3167)	-	(1.3377)	-	(1.2483)
Industrial Share * Quality of Governance	-	1.8920	-	1.3159	-	0.2413
	-	(1.3180)	-	(0.9436)	-	(0.9179)
Energy Demand * Quality of Governance	-	-1.1507	-	-2.0015**	-	-1.3323**
	-	(0.8602)	-	(0.8380)	-	(0.6421)
Policy Stringency * Quality of Governance * Food Vulnerability	-	-1.7299	-	-0.5872	-	1.2047
	-	(1.6441)	-	(0.7391)	-	(0.9467)
Environmental Innovation * Quality of Governance * Food Vulnerability	-	0.0713	-	-1.3426	-	-2.0567**
	-	(1.5530)	-	(1.4620)	-	(1.0346)
Foreign Direct Investment * Quality of Governance * Food Vulnerability	-	-1.2896	-	-6.3182**	-	3.1068

	-	(3.0918)	-	(3.0003)	-	(2.6327)
Industrial Share * Quality of Governance * Food Vulnerability	-	-2.8820	-	-3.0622	-	0.2798
	-	(3.0854)	-	(1.9707)	-	(1.9737)
Energy Demand * Quality of Governance * Food Vulnerability	-	1.7612	-	3.3576*	-	1.7751
	-	(1.9212)	-	(2.0288)	-	(1.4216)
Constant	0.7854***	-6.1626***	0.9104***	-3.9352**	0.6858***	-6.4976***
	(0.2407)	(1.5143)	(0.2305)	(1.6631)	(0.2567)	(2.2784)
Wald-chi2	289.4	3231	533.6	2520	321.0	1825
Observations	115	115	115	115	115	115
Heteroskedasticity controlled	Yes	Yes	Yes	Yes	Yes	Yes
Autocorrelation controlled	Yes	Yes	Yes	Yes	Yes	Yes
Cross-sectional correlation controlled	Yes	Yes	Yes	Yes	Yes	Yes
Marginal Impact						
Policy Stringency	-0.0301*	-0.0061	-0.0455***	-0.0433**	-0.0390**	-0.0211
	(0.0166)	(0.0163)	(0.0165)	(0.0204)	(0.0169)	(0.0226)
Environmental Innovation	-0.0470*	0.0226	-0.0423*	-0.0538	-0.0753***	-0.0790***
	(0.0251)	(0.0332)	(0.0238)	(0.0365)	(0.0242)	(0.0287)
Foreign Direct Investment	-0.0133	-0.0184	-0.0238	-0.1033**	-0.0414	-0.0823
	(0.0375)	(0.0499)	(0.0387)	(0.0412)	(0.0385)	(0.0525)
Industrial Share	0.7038***	1.2775***	0.6968***	0.7790***	0.7174***	1.1427***
	(0.0872)	(0.0786)	(0.0833)	(0.1081)	(0.0885)	(0.1006)
Energy Demand	-0.0570	-0.2788***	-0.0573	-0.0537	-0.0120	-0.0765
	(0.0435)	(0.0621)	(0.0363)	(0.0607)	(0.0413)	(0.0479)

Note:
Standard errors in parentheses ()
*** p<0.01, ** p<0.05, * p<0.1 by Z-test

Table S40: Long Run Coefficient Estimation using Generalized Least Square with Food Vulnerability for SDG 7.3.1 – With Regulatory Quality, Rule of Law, and Voice and Accountability, Related to Figure 3

Variables	Model 1	Model 2	Model 1	Model 2	Model 1	Model 2
	Regulatory Quality		Rule of Law		Voice and Accountability	
Policy Stringency	-0.0519***	-0.0354	-0.0397**	0.0945	-0.0163	0.1592
	(0.0163)	(0.2215)	(0.0157)	(0.1488)	(0.0151)	(0.1296)
Environmental Innovation	-0.0535**	0.2447	-0.0289	0.8038***	-0.0027	0.2874
	(0.0235)	(0.1774)	(0.0227)	(0.1837)	(0.0214)	(0.2002)
Foreign Direct Investment	-0.0263	-1.2865*	-0.0200	-0.1493	-0.0199	-0.0790
	(0.0379)	(0.6616)	(0.0389)	(0.3019)	(0.0355)	(0.3223)
Industrial Share	0.7238***	2.3057***	0.6868***	1.4354**	0.6655***	1.4712***
	(0.0825)	(0.5695)	(0.0819)	(0.5755)	(0.0778)	(0.5649)
Energy Demand	-0.0334	-0.3746	-0.0867**	-1.0396***	-0.2360***	-0.8399**
	(0.0381)	(0.3757)	(0.0354)	(0.3737)	(0.0468)	(0.4131)
Food Vulnerability	-1.1425***	9.0644**	-1.1791***	6.6721	-0.4475***	1.4734
	(0.1203)	(4.0034)	(0.1286)	(4.4590)	(0.1474)	(4.3339)
Quality of Governance	0.0373	0.1208	-0.0644**	1.7383**	-0.1820***	-0.0334
	(0.0340)	(0.6715)	(0.0319)	(0.8538)	(0.0338)	(0.3656)
Policy Stringency * Food Vulnerability	-	0.0337	-	-0.2092	-	-0.3975
	-	(0.4909)	-	(0.2999)	-	(0.3015)
Environmental Innovation * Food Vulnerability	-	-0.6839*	-	-1.5934***	-	-0.5771
	-	(0.3810)	-	(0.3525)	-	(0.4506)
Foreign Direct Investment * Food Vulnerability	-	2.7419*	-	0.3308	-	0.1575
	-	(1.4662)	-	(0.6504)	-	(0.8544)
Industrial Share * Food Vulnerability	-	-2.9528**	-	-1.6662	-	-1.6062
	-	(1.2175)	-	(1.0790)	-	(1.2634)
Energy Demand * Food Vulnerability	-	0.6191	-	1.6087**	-	1.2319
	-	(0.8208)	-	(0.7741)	-	(0.9563)
Policy Stringency * Quality of Governance	-	-0.0225	-	-0.3041	-	-0.0078
	-	(0.6663)	-	(0.6659)	-	(0.3169)
Environmental Innovation * Quality of Governance	-	0.4157	-	1.1160**	-	0.1399
	-	(0.5279)	-	(0.5366)	-	(0.4344)
Foreign Direct Investment * Quality of Governance	-	-3.3045*	-	2.5272*	-	0.0218
	-	(1.7506)	-	(1.3933)	-	(0.8987)
Industrial Share * Quality of Governance	-	0.2916	-	-0.6379	-	0.1063
	-	(1.6639)	-	(1.3960)	-	(1.0947)
Energy Demand * Quality of Governance	-	-0.4409	-	-1.1858	-	-0.2287
	-	(1.0397)	-	(0.8390)	-	(0.8701)
Policy Stringency * Quality of Governance * Food Vulnerability	-	0.2784	-	1.1330	-	-0.0051
	-	(1.4452)	-	(1.6038)	-	(0.7668)
Environmental Innovation * Quality of Governance * Food Vulnerability	-	-0.9192	-	-1.6748	-	-0.2764
	-	(1.1764)	-	(1.2744)	-	(1.0233)
Foreign Direct Investment * Quality of Governance * Food Vulnerability	-	7.1205*	-	-6.1920*	-	-0.2449

	-	(3.8361)	-	(3.6653)	-	(2.2840)
Industrial Share * Quality of Governance * Food Vulnerability	-	-0.2529	-	1.8612	-	-0.3743
	-	(3.5562)	-	(3.4521)	-	(2.6998)
Energy Demand * Quality of Governance * Food Vulnerability	-	0.7106	-	0.8048	-	0.5067
	-	(2.2737)	-	(1.8936)	-	(2.0508)
Constant	0.7264***	-4.3064**	1.0073***	-2.0093	1.4970***	0.4607
	(0.2451)	(1.7461)	(0.2260)	(2.0257)	(0.2599)	(1.9967)
Wald-chi2	630.1	1666	615.7	2898	597.0	2763
Observations	115	115	115	115	115	115
Heteroskedasticity controlled	Yes	Yes	Yes	Yes	Yes	Yes
Autocorrelation controlled	Yes	Yes	Yes	Yes	Yes	Yes
Cross-sectional correlation controlled	Yes	Yes	Yes	Yes	Yes	Yes
Marginal Impact						
Policy Stringency	-0.0519***	-0.0321*	-0.0397**	-0.0329*	-0.0163	-0.0154
	(0.0163)	(0.0189)	(0.0157)	(0.0197)	(0.0151)	(0.0189)
Environmental Innovation	-0.0535**	-0.0586	-0.0289	-0.0449	-0.0027	0.0177
	(0.0235)	(0.0356)	(0.0227)	(0.0287)	(0.0214)	(0.0357)
Foreign Direct Investment	-0.0263	-0.0620	-0.0200	-0.0639	-0.0199	-0.0002
	(0.0379)	(0.0427)	(0.0389)	(0.0528)	(0.0355)	(0.0551)
Industrial Share	0.7238***	0.9755***	0.6868***	0.6792***	0.6655***	0.7570***
	(0.0825)	(0.0915)	(0.0819)	(0.0908)	(0.0778)	(0.0965)
Energy Demand	-0.0334	-0.0866	-0.0867**	-0.0701	-0.2360***	-0.2740***
	(0.0381)	(0.0588)	(0.0354)	(0.0522)	(0.0468)	(0.0740)
Note:<br>Standard errors in parentheses ()<br>*** p<0.01, ** p<0.05, * p<0.1 by Z-test						

Table S41: Long Run Coefficient Estimation using Generalized Least Square with Health Vulnerability for SDG 7.3.1 – With Control of Corruption, Governance Effectiveness, and Political Stability, Related to Figure 3

Variables	Model 1	Model 2	Model 1	Model 2	Model 1	Model 2
	Control of Corruption		Governance Effectiveness		Political Stability	
Policy Stringency	-0.0393**	0.4035	-0.0502***	0.2131*	-0.0429**	-0.3820
	(0.0176)	(0.2511)	(0.0177)	(0.1131)	(0.0178)	(0.3453)
Environmental Innovation	-0.0631**	0.2792	-0.0570**	0.0829	-0.0909***	0.2229
	(0.0261)	(0.2387)	(0.0251)	(0.1240)	(0.0255)	(0.2522)
Foreign Direct Investment	-0.0132	0.8807	-0.0236	0.3134	-0.0333	-1.8063*
	(0.0396)	(0.6241)	(0.0417)	(0.2045)	(0.0404)	(1.0370)
Industrial Share	0.7932***	1.3994**	0.7645***	0.7534*	0.7638***	0.4857
	(0.0905)	(0.5886)	(0.0884)	(0.4214)	(0.0942)	(0.8504)
Energy Demand	-0.0330	-0.8594**	-0.0345	-0.3076	0.0127	-0.1470
	(0.0447)	(0.3696)	(0.0381)	(0.1950)	(0.0434)	(0.4103)
Health Vulnerability	-0.7126***	-2.5629	-0.8355***	-2.3386	-0.7848***	-4.7284
	(0.1066)	(3.3756)	(0.0923)	(2.8996)	(0.1102)	(3.4509)
Quality of Governance	-0.0466	-0.2097	-0.0326	1.5358**	-0.0205	0.4073
	(0.0340)	(0.8337)	(0.0275)	(0.6125)	(0.0226)	(0.5279)
Policy Stringency * Health Vulnerability	-	-0.7382	-	-0.5497**	-	0.8618
	-	(0.5292)	-	(0.2287)	-	(0.7243)
Environmental Innovation * Health Vulnerability	-	-0.5150	-	-0.3595	-	-0.2619
	-	(0.5099)	-	(0.2455)	-	(0.5547)
Foreign Direct Investment * Health Vulnerability	-	-2.1415	-	-0.8560*	-	3.9192*
	-	(1.4049)	-	(0.4563)	-	(2.2560)
Industrial Share * Health Vulnerability	-	0.0804	-	0.2693	-	2.3070
	-	(1.2100)	-	(0.8106)	-	(1.8838)
Energy Demand * Health Vulnerability	-	1.1234	-	0.6841*	-	-0.6241
	-	(0.7676)	-	(0.3833)	-	(0.9077)
Policy Stringency * Quality of Governance	-	0.3371	-	0.7624**	-	-0.6561
	-	(0.5901)	-	(0.3705)	-	(0.4397)
Environmental Innovation * Quality of Governance	-	0.0342	-	0.9119**	-	0.6965**
	-	(0.4837)	-	(0.3911)	-	(0.3169)
Foreign Direct Investment * Quality of Governance	-	0.9020	-	0.4698	-	-2.1512*
	-	(1.2192)	-	(1.2014)	-	(1.1104)
Industrial Share * Quality of Governance	-	-1.0141	-	1.2420*	-	-0.2316
	-	(0.9205)	-	(0.6594)	-	(0.7142)
Energy Demand * Quality of Governance	-	0.3215	-	-2.0617***	-	-0.4773
	-	(0.6854)	-	(0.6028)	-	(0.4972)
Policy Stringency * Quality of Governance * Health Vulnerability	-	-0.4566	-	-1.1996	-	1.4427
	-	(1.2869)	-	(0.7735)	-	(0.8834)
Environmental Innovation * Quality of Governance * Health Vulnerability	-	0.2991	-	-2.0761**	-	-0.6581
	-	(1.1266)	-	(0.9714)	-	(0.6319)
Foreign Direct Investment * Quality of Governance * Health Vulnerability	-	-2.4881	-	-0.9329	-	4.6806**

	-	(2.9947)	-	(2.7862)	-	(2.2686)
Industrial Share * Quality of Governance * Health Vulnerability	-	3.1607	-	-2.7346*	-	1.5119
	-	(2.3014)	-	(1.4976)	-	(1.4734)
Energy Demand * Quality of Governance * Health Vulnerability	-	-1.5926	-	4.0488***	-	-0.5669
	-	(1.5540)	-	(1.4682)	-	(1.0161)
Constant	0.3392	0.1495	0.4958**	0.6969	0.3791	0.9752
	(0.2213)	(1.7670)	(0.2230)	(1.4057)	(0.2490)	(1.6634)
Wald-chi2	321.8	2975	441.9	1651	329.5	4286
Observations	115	115	115	115	115	115
Heteroskedasticity controlled	Yes	Yes	Yes	Yes	Yes	Yes
Autocorrelation controlled	Yes	Yes	Yes	Yes	Yes	Yes
Cross-sectional correlation controlled	Yes	Yes	Yes	Yes	Yes	Yes
Marginal Impact						
Policy Stringency	-0.0393**	0.0114	-0.0502***	-0.0541***	-0.0429**	-0.0125
	(0.0176)	(0.0214)	(0.0177)	(0.0203)	(0.0178)	(0.0221)
Environmental Innovation	-0.0631**	-0.0182	-0.0570**	-0.0996***	-0.0909***	-0.1064***
	(0.0261)	(0.0352)	(0.0251)	(0.0338)	(0.0255)	(0.0254)
Foreign Direct Investment	-0.0132	-0.0524	-0.0236	-0.0926**	-0.0333	-0.0717
	(0.0396)	(0.0564)	(0.0417)	(0.0456)	(0.0404)	(0.0495)
Industrial Share	0.7932***	1.3091***	0.7645***	0.8585***	0.7638***	1.2722***
	(0.0905)	(0.0972)	(0.0884)	(0.0980)	(0.0942)	(0.0906)
Energy Demand	-0.0330	-0.2055***	-0.0345	0.0435	0.0127	-0.0146
	(0.0447)	(0.0638)	(0.0381)	(0.0535)	(0.0434)	(0.0399)

Note:
Standard errors in parentheses ()
*** p<0.01, ** p<0.05, * p<0.1 by Z-test

Table S42: Long Run Coefficient Estimation using Generalized Least Square with Health Vulnerability for SDG 7.3.1 – With Regulatory Quality, Rule of Law, and Voice and Accountability, Related to Figure 3

Variables	Model 1	Model 2	Model 1	Model 2	Model 1	Model 2
	Regulatory Quality		Rule of Law		Voice and Accountability	
Policy Stringency	-0.0586***	0.0067	-0.0481***	0.2359	-0.0220	0.0478
	(0.0175)	(0.1979)	(0.0169)	(0.1569)	(0.0158)	(0.1192)
Environmental Innovation	-0.0688***	0.0044	-0.0413*	0.5303***	0.0004	-0.0889
	(0.0252)	(0.1616)	(0.0244)	(0.1598)	(0.0225)	(0.1554)
Foreign Direct Investment	-0.0207	-0.1591	-0.0227	0.0815	-0.0142	-0.1263
	(0.0395)	(0.5131)	(0.0414)	(0.2973)	(0.0366)	(0.2139)
Industrial Share	0.7486***	1.0058**	0.7721***	0.6085	0.6422***	0.6636
	(0.0878)	(0.5061)	(0.0870)	(0.5378)	(0.0804)	(0.4402)
Energy Demand	-0.0047	-0.0552	-0.0685*	-0.9604***	-0.2442***	0.2432
	(0.0404)	(0.2061)	(0.0379)	(0.2783)	(0.0473)	(0.3402)
Health Vulnerability	-0.8064***	0.3763	-0.7785***	-3.5467	-0.3358***	4.0826
	(0.0944)	(2.8847)	(0.0986)	(3.5893)	(0.1014)	(3.5248)
Quality of Governance	0.0299	-0.0354	-0.0785**	2.8064***	-0.1963***	0.0038
	(0.0361)	(0.7534)	(0.0345)	(0.8639)	(0.0329)	(0.4104)
Policy Stringency * Health Vulnerability	-	-0.0850	-	-0.4464	-	-0.1517
	-	(0.4091)	-	(0.3032)	-	(0.2345)
Environmental Innovation * Health Vulnerability	-	-0.1503	-	-0.9649***	-	0.2306
	-	(0.3281)	-	(0.2757)	-	(0.3075)
Foreign Direct Investment * Health Vulnerability	-	0.1895	-	-0.4195	-	0.2378
	-	(1.1566)	-	(0.6471)	-	(0.4916)
Industrial Share * Health Vulnerability	-	0.0758	-	0.5428	-	0.1419
	-	(1.0533)	-	(0.9914)	-	(0.8722)
Energy Demand * Health Vulnerability	-	-0.0075	-	1.3985***	-	-0.9587
	-	(0.4329)	-	(0.5170)	-	(0.6887)
Policy Stringency * Quality of Governance	-	-0.0439	-	0.6633	-	0.1077
	-	(0.6229)	-	(0.4664)	-	(0.2013)
Environmental Innovation * Quality of Governance	-	0.0169	-	1.1553***	-	0.5629**
	-	(0.4332)	-	(0.3984)	-	(0.2511)
Foreign Direct Investment * Quality of Governance	-	-0.7364	-	0.2335	-	0.1944
	-	(1.3156)	-	(0.8755)	-	(0.3585)
Industrial Share * Quality of Governance	-	-0.7963	-	0.4904	-	0.8290
	-	(1.0088)	-	(0.8864)	-	(0.5973)
Energy Demand * Quality of Governance	-	0.4263	-	-2.0634***	-	-1.0168*
	-	(0.6594)	-	(0.6483)	-	(0.5353)
Policy Stringency * Quality of Governance * Health Vulnerability	-	0.4162	-	-1.1617	-	-0.2954
	-	(1.2785)	-	(1.0039)	-	(0.4298)
Environmental Innovation * Quality of Governance * Health Vulnerability	-	0.0423	-	-1.9052**	-	-1.1744**
	-	(0.9297)	-	(0.8404)	-	(0.5135)
Foreign Direct Investment * Quality of Governance * Health Vulnerability	-	1.4863	-	0.1151	-	-0.6114

	-	(2.9689)	-	(2.2609)	-	(0.8757)
Industrial Share * Quality of Governance * Health Vulnerability	-	2.4431	-	-1.8403	-	-1.9299
	-	(2.2587)	-	(2.0968)	-	(1.3307)
Energy Demand * Quality of Governance * Health Vulnerability	-	-1.3987	-	3.1721**	-	2.2042**
	-	(1.4326)	-	(1.4054)	-	(1.1109)
Constant	0.4296*	-0.8028	0.5303**	2.0512	1.5603***	-1.1456
	(0.2397)	(1.4082)	(0.2172)	(1.8026)	(0.2699)	(1.7270)
Wald-chi2	440.3	1800	538.4	2425	533.3	2146
Observations	115	115	115	115	115	115
Heteroskedasticity controlled	Yes	Yes	Yes	Yes	Yes	Yes
Autocorrelation controlled	Yes	Yes	Yes	Yes	Yes	Yes
Cross-sectional correlation controlled	Yes	Yes	Yes	Yes	Yes	Yes
Marginal Impact						
Policy Stringency	-0.0586***	-0.0505**	-0.0481***	-0.0365	-0.0220	-0.0279
	(0.0175)	(0.0213)	(0.0169)	(0.0251)	(0.0158)	(0.0208)
Environmental Innovation	-0.0688***	-0.0698*	-0.0413*	-0.0444	0.0004	-0.0289
	(0.0252)	(0.0359)	(0.0244)	(0.0358)	(0.0225)	(0.0376)
Foreign Direct Investment	-0.0207	-0.0690	-0.0227	-0.1939***	-0.0142	-0.0201
	(0.0395)	(0.0467)	(0.0414)	(0.0589)	(0.0366)	(0.0485)
Industrial Share	0.7486***	0.9980***	0.7721***	0.9210***	0.6422***	0.6714***
	(0.0878)	(0.1002)	(0.0870)	(0.0967)	(0.0804)	(0.1022)
Energy Demand	-0.0047	-0.0307	-0.0685*	-0.0601	-0.2442***	-0.1224
	(0.0404)	(0.0560)	(0.0379)	(0.0570)	(0.0473)	(0.0797)

Note:
Standard errors in parentheses ()
*** p<0.01, ** p<0.05, * p<0.1 by Z-test

Table S43: Long Run Coefficient Estimation using Generalized Least Square with Infrastructure Vulnerability for SDG 7.3.1 – With Control of Corruption, Governance Effectiveness, and Political Stability, Related to Figure 3

Variables	Model 1	Model 2	Model 1	Model 2	Model 1	Model 2
	Control of Corruption		Governance Effectiveness		Political Stability	
Policy Stringency	-0.0469***	0.2163**	-0.0529***	0.2219***	-0.0490***	-0.0138
	(0.0180)	(0.0959)	(0.0178)	(0.0830)	(0.0178)	(0.1063)
Environmental Innovation	-0.0434	0.6468***	-0.0287	0.1198	-0.0636***	0.2703
	(0.0265)	(0.1882)	(0.0257)	(0.0928)	(0.0247)	(0.1951)
Foreign Direct Investment	-0.0156	0.0033	-0.0249	0.0749	-0.0153	-0.7729*
	(0.0367)	(0.2871)	(0.0390)	(0.1437)	(0.0382)	(0.4623)
Industrial Share	0.6971***	1.5308***	0.7640***	0.6900*	0.7574***	1.3638***
	(0.0878)	(0.4263)	(0.0863)	(0.3532)	(0.0865)	(0.5255)
Energy Demand	-0.0046	-1.0367***	-0.0252	-0.2604**	0.0250	-0.4805**
	(0.0433)	(0.2263)	(0.0377)	(0.1107)	(0.0388)	(0.2104)
Infrastructure Vulnerability	-1.7863***	-1.8518	-1.9323***	-7.0168	-1.7160***	-5.2471
	(0.2066)	(6.0178)	(0.1826)	(4.9218)	(0.1900)	(5.8072)
Quality of Governance	-0.0058	-0.5588	-0.0315	1.7163***	0.0223	-0.3849
	(0.0339)	(0.6191)	(0.0272)	(0.5682)	(0.0215)	(0.4714)
Policy Stringency * Infrastructure Vulnerability	-	-0.9866	-	-1.4697***	-	0.4620
	-	(0.6608)	-	(0.4586)	-	(0.7235)
Environmental Innovation * Infrastructure Vulnerability	-	-3.4475***	-	-0.8990**	-	-0.6588
	-	(1.1395)	-	(0.4521)	-	(1.1687)
Foreign Direct Investment * Infrastructure Vulnerability	-	-0.2089	-	-1.3310	-	5.3614*
	-	(2.0510)	-	(0.9935)	-	(3.0164)
Industrial Share * Infrastructure Vulnerability	-	-1.5108	-	1.3609	-	1.2243
	-	(2.2867)	-	(1.7481)	-	(3.0655)
Energy Demand * Infrastructure Vulnerability	-	4.7266***	-	1.4601***	-	0.1754
	-	(1.3748)	-	(0.5283)	-	(1.4368)
Policy Stringency * Quality of Governance	-	0.3167	-	0.3270	-	-0.0579
	-	(0.2393)	-	(0.2352)	-	(0.1690)
Environmental Innovation * Quality of Governance	-	0.9839***	-	1.3510***	-	0.6792***
	-	(0.3478)	-	(0.2396)	-	(0.2234)
Foreign Direct Investment * Quality of Governance	-	0.1627	-	0.6065	-	-0.8983
	-	(0.5039)	-	(0.5875)	-	(0.5499)
Industrial Share * Quality of Governance	-	0.5737	-	0.0252	-	0.4607
	-	(0.5795)	-	(0.5975)	-	(0.4404)
Energy Demand * Quality of Governance	-	-1.3216***	-	-1.7949***	-	-0.8327***
	-	(0.3952)	-	(0.3422)	-	(0.2523)
Policy Stringency * Quality of Governance * Infrastructure Vulnerability	-	-1.0188	-	-0.8025	-	1.0239
	-	(1.7165)	-	(1.2849)	-	(0.9975)
Environmental Innovation * Quality of Governance * Infrastructure Vulnerability	-	-4.6721**	-	-6.4368***	-	-1.6003
	-	(2.3543)	-	(1.3605)	-	(1.2346)
Foreign Direct Investment * Quality of Governance * Infrastructure Vulnerability	-	-0.7325	-	-1.0129	-	6.3193**

	-	(4.0199)	-	(3.6964)	-	(3.2216)
Industrial Share * Quality of Governance * Infrastructure Vulnerability	-	-1.5170	-	-2.0183	-	0.6453
	-	(3.4897)	-	(3.1866)	-	(2.4254)
Energy Demand * Quality of Governance * Infrastructure Vulnerability	-	5.9176**	-	8.0435***	-	0.7108
	-	(2.7419)	-	(2.2994)	-	(1.4943)
Constant	0.3098	-1.0153	0.1602	0.4139	0.0628	-0.8202
	(0.2164)	(1.0875)	(0.2028)	(0.9324)	(0.2134)	(0.9969)
Wald-chi2	357.5	1724	558.8	2707	495.8	3264
Observations	115	115	115	115	115	115
Heteroskedasticity controlled	Yes	Yes	Yes	Yes	Yes	Yes
Autocorrelation controlled	Yes	Yes	Yes	Yes	Yes	Yes
Cross-sectional correlation controlled	Yes	Yes	Yes	Yes	Yes	Yes
Marginal Impact						
Policy Stringency	-0.0469***	-0.0008	-0.0529***	-0.0198	-0.0490***	-0.0060
	(0.0180)	(0.0236)	(0.0178)	(0.0211)	(0.0178)	(0.0201)
Environmental Innovation	-0.0434	-0.0133	-0.0287	-0.0677**	-0.0636***	-0.0606*
	(0.0265)	(0.0360)	(0.0257)	(0.0322)	(0.0247)	(0.0355)
Foreign Direct Investment	-0.0156	-0.0502	-0.0249	-0.1486***	-0.0153	-0.0223
	(0.0367)	(0.0604)	(0.0390)	(0.0552)	(0.0382)	(0.0475)
Industrial Share	0.6971***	1.1651***	0.7640***	0.8975***	0.7574***	1.2399***
	(0.0878)	(0.0986)	(0.0863)	(0.0989)	(0.0865)	(0.1017)
Energy Demand	-0.0046	-0.1167**	-0.0252	0.0288	0.0250	-0.0556
	(0.0433)	(0.0538)	(0.0377)	(0.0509)	(0.0388)	(0.0420)

Note:
Standard errors in parentheses ()
*** p<0.01, ** p<0.05, * p<0.1 by Z-test

Table S44: Long Run Coefficient Estimation using Generalized Least Square with Infrastructure Vulnerability for SDG 7.3.1 – With Regulatory Quality, Rule of Law, and Voice and Accountability, Related to Figure 3

Variables	Model 1	Model 2	Model 1	Model 2	Model 1	Model 2
	Regulatory Quality		Rule of Law		Voice and Accountability	
Policy Stringency	-0.0604***	0.0726	-0.0517***	0.1996***	-0.0274*	-0.0461
	(0.0176)	(0.0809)	(0.0177)	(0.0764)	(0.0156)	(0.0871)
Environmental Innovation	-0.0522**	0.1066	-0.0279	0.5697***	0.0171	-0.0866
	(0.0258)	(0.1535)	(0.0244)	(0.1194)	(0.0218)	(0.1260)
Foreign Direct Investment	-0.0303	-0.0768	-0.0267	0.2936*	-0.0132	-0.1768
	(0.0382)	(0.2881)	(0.0396)	(0.1590)	(0.0353)	(0.1518)
Industrial Share	0.7989***	1.2001***	0.7782***	0.2451	0.6321***	1.5022***
	(0.0856)	(0.3816)	(0.0876)	(0.4255)	(0.0775)	(0.3438)
Energy Demand	0.0144	-0.2129	-0.0374	-0.8833***	-0.2364***	0.0872
	(0.0408)	(0.1678)	(0.0386)	(0.1851)	(0.0479)	(0.2425)
Infrastructure Vulnerability	-1.8539***	0.9779	-1.8061***	-15.0680**	-0.9860***	14.0897*
	(0.1917)	(6.1787)	(0.2196)	(6.8244)	(0.2127)	(8.3881)
Quality of Governance	0.0403	-0.1699	-0.0470	1.9434**	-0.1951***	-0.0276
	(0.0345)	(0.7492)	(0.0381)	(0.8251)	(0.0319)	(0.5252)
Policy Stringency * Infrastructure Vulnerability	-	-0.4575	-	-1.2209***	-	0.0964
	-	(0.5140)	-	(0.4018)	-	(0.4928)
Environmental Innovation * Infrastructure Vulnerability	-	-0.6414	-	-2.5696***	-	0.6389
	-	(0.8747)	-	(0.5521)	-	(0.6954)
Foreign Direct Investment * Infrastructure Vulnerability	-	0.0944	-	-2.3243**	-	1.0105
	-	(2.0215)	-	(1.0224)	-	(1.0364)
Industrial Share * Infrastructure Vulnerability	-	-0.4132	-	3.4488*	-	-2.3098
	-	(2.1527)	-	(1.9294)	-	(1.9376)
Energy Demand * Infrastructure Vulnerability	-	0.5525	-	3.5402***	-	-1.8253
	-	(1.0475)	-	(0.9679)	-	(1.4334)
Policy Stringency * Quality of Governance	-	0.2140	-	0.5890***	-	0.2675**
	-	(0.2796)	-	(0.1813)	-	(0.1217)
Environmental Innovation * Quality of Governance	-	-0.1992	-	0.8486***	-	0.4367**
	-	(0.4668)	-	(0.2430)	-	(0.2196)
Foreign Direct Investment * Quality of Governance	-	-0.5545	-	1.0196***	-	0.0235
	-	(0.7500)	-	(0.3238)	-	(0.1954)
Industrial Share * Quality of Governance	-	0.7144	-	0.3594	-	1.1772***
	-	(0.8582)	-	(0.5113)	-	(0.4276)
Energy Demand * Quality of Governance	-	-0.1240	-	-1.5645***	-	-1.0890***
	-	(0.4573)	-	(0.3189)	-	(0.3743)
Policy Stringency * Quality of Governance * Infrastructure Vulnerability	-	-0.9141	-	-3.5196***	-	-1.6470**
	-	(1.9352)	-	(1.0972)	-	(0.6713)
Environmental Innovation * Quality of Governance * Infrastructure Vulnerability	-	1.5801	-	-3.3296**	-	-2.7019**
	-	(2.7456)	-	(1.3534)	-	(1.2487)
Foreign Direct Investment * Quality of Governance * Infrastructure Vulnerability	-	3.6710	-	-7.2083***	-	-0.7414

	-	(5.3471)	-	(2.5909)	-	(1.2764)
Industrial Share * Quality of Governance * Infrastructure Vulnerability	-	-2.4059	-	-5.4632**	-	-7.1422***
	-	(4.8194)	-	(2.7462)	-	(2.2839)
Energy Demand * Quality of Governance * Infrastructure Vulnerability	-	-0.5988	-	7.3661***	-	6.6759***
	-	(2.9347)	-	(1.8685)	-	(2.2102)
Constant	-0.0495	-1.2185	0.1562	2.5681*	1.4350***	-2.8742**
	(0.2136)	(1.0539)	(0.2099)	(1.3556)	(0.2742)	(1.4244)
Wald-chi2	624.2	1864	592.9	2404	786.9	1946
Observations	115	115	115	115	115	115
Heteroskedasticity controlled	Yes	Yes	Yes	Yes	Yes	Yes
Autocorrelation controlled	Yes	Yes	Yes	Yes	Yes	Yes
Cross-sectional correlation controlled	Yes	Yes	Yes	Yes	Yes	Yes
Marginal Impact						
Policy Stringency	-0.0604***	-0.0046	-0.0517***	-0.0515**	-0.0274*	-0.0440
	(0.0176)	(0.0219)	(0.0177)	(0.0259)	(0.0156)	(0.0274)
Environmental Innovation	-0.0522**	-0.0074	-0.0279	0.0224	0.0171	-0.0060
	(0.0258)	(0.0408)	(0.0244)	(0.0323)	(0.0218)	(0.0383)
Foreign Direct Investment	-0.0303	-0.0788	-0.0267	-0.1386***	-0.0132	-0.0018
	(0.0382)	(0.0501)	(0.0396)	(0.0538)	(0.0353)	(0.0556)
Industrial Share	0.7989***	1.1062***	0.7782***	0.8679***	0.6321***	1.0728***
	(0.0856)	(0.0981)	(0.0876)	(0.1033)	(0.0775)	(0.1178)
Energy Demand	0.0144	-0.0980*	-0.0374	-0.1049**	-0.2364***	-0.1492**
	(0.0408)	(0.0554)	(0.0386)	(0.0471)	(0.0479)	(0.0651)
Note:<br>Standard errors in parentheses ()<br>*** p<0.01, ** p<0.05, * p<0.1 by Z-test						

Table S45: Long Run Coefficient Estimation using Generalized Least Square with Water Vulnerability for SDG 7.3.1 – With Control of Corruption, Governance Effectiveness, and Political Stability, Related to Figure 3

Variables	Model 1	Model 2	Model 1	Model 2	Model 1	Model 2
	Control of Corruption		Governance Effectiveness		Political Stability	
Policy Stringency	-0.0287*	0.3694**	-0.0409**	0.3654***	-0.0346**	0.0489
	(0.0166)	(0.1621)	(0.0162)	(0.1112)	(0.0171)	(0.1558)
Environmental Innovation	-0.0488**	0.9385***	-0.0382	0.5200***	-0.0719***	0.7009***
	(0.0248)	(0.2349)	(0.0233)	(0.1409)	(0.0241)	(0.2480)
Foreign Direct Investment	-0.0068	0.0203	-0.0098	-0.2997	-0.0298	-1.8217***
	(0.0364)	(0.4693)	(0.0367)	(0.2413)	(0.0380)	(0.5320)
Industrial Share	0.7389***	2.3443***	0.7237***	2.0063***	0.7486***	1.7769***
	(0.0865)	(0.4855)	(0.0814)	(0.4187)	(0.0894)	(0.5646)
Energy Demand	-0.0208	-1.6036***	-0.0179	-0.6936***	0.0170	-0.7394***
	(0.0412)	(0.3181)	(0.0341)	(0.1749)	(0.0383)	(0.2812)
Water Vulnerability	-1.0924***	4.4416	-1.3427***	9.6527***	-1.1266***	6.9703*
	(0.1494)	(4.1892)	(0.1192)	(3.6627)	(0.1499)	(4.1974)
Quality of Governance	-0.0474	0.3028	-0.0448*	2.7904***	-0.0153	0.3855
	(0.0318)	(0.9152)	(0.0255)	(0.7698)	(0.0214)	(0.5326)
Policy Stringency * Water Vulnerability	-	-0.9457*	-	-1.0255***	-	0.0082
	-	(0.5057)	-	(0.2868)	-	(0.4695)
Environmental Innovation * Water Vulnerability	-	-2.4568***	-	-1.5458***	-	-1.6968**
	-	(0.6678)	-	(0.3527)	-	(0.6845)
Foreign Direct Investment * Water Vulnerability	-	-0.1952	-	0.8313	-	5.9191***
	-	(1.4446)	-	(0.6928)	-	(1.6379)
Industrial Share * Water Vulnerability	-	-3.1779**	-	-3.1313***	-	-1.4044
	-	(1.3460)	-	(0.9976)	-	(1.5845)
Energy Demand * Water Vulnerability	-	3.7873***	-	1.9175***	-	1.1508
	-	(0.9222)	-	(0.4265)	-	(0.8477)
Policy Stringency * Quality of Governance	-	0.5730	-	0.5742	-	-0.0441
	-	(0.4540)	-	(0.3610)	-	(0.1884)
Environmental Innovation * Quality of Governance	-	1.0537**	-	2.3841***	-	0.9158***
	-	(0.5178)	-	(0.5649)	-	(0.2599)
Foreign Direct Investment * Quality of Governance	-	0.5022	-	1.2453	-	-1.6840***
	-	(0.9772)	-	(1.0101)	-	(0.5711)
Industrial Share * Quality of Governance	-	-0.0692	-	-0.6178	-	0.0082
	-	(0.8979)	-	(0.8877)	-	(0.5437)
Energy Demand * Quality of Governance	-	-1.3749**	-	-2.9438***	-	-0.9836***
	-	(0.6142)	-	(0.6973)	-	(0.3488)
Policy Stringency * Quality of Governance * Water Vulnerability	-	-1.3510	-	-0.9695	-	0.3575
	-	(1.3669)	-	(0.9438)	-	(0.5118)
Environmental Innovation * Quality of Governance * Water Vulnerability	-	-2.8092*	-	-6.6868***	-	-1.8509***
	-	(1.5411)	-	(1.5989)	-	(0.6634)
Foreign Direct Investment * Quality of Governance * Water Vulnerability	-	-1.8650	-	-3.6753	-	5.6422***

	-	(3.2202)	-	(3.1504)	-	(1.6242)
Industrial Share * Quality of Governance * Water Vulnerability	-	0.0822	-	0.0927	-	0.6606
	-	(3.0003)	-	(2.4742)	-	(1.4612)
Energy Demand * Quality of Governance * Water Vulnerability	-	3.5939*	-	7.9220***	-	1.3985
	-	(1.9714)	-	(2.1041)	-	(0.9069)
Constant	0.3924*	-2.5197	0.4759**	-4.1462***	0.3092	-3.4486**
	(0.2105)	(1.7392)	(0.1994)	(1.4250)	(0.2229)	(1.5880)
Wald-chi2	303.5	2373	561.5	3735	328.5	3317
Observations	115	115	115	115	115	115
Heteroskedasticity controlled	Yes	Yes	Yes	Yes	Yes	Yes
Autocorrelation controlled	Yes	Yes	Yes	Yes	Yes	Yes
Cross-sectional correlation controlled	Yes	Yes	Yes	Yes	Yes	Yes
Marginal Impact						
Policy Stringency	-0.0287*	-0.0021	-0.0409**	-0.0117	-0.0346**	-0.0014
	(0.0166)	(0.0235)	(0.0162)	(0.0204)	(0.0171)	(0.0207)
Environmental Innovation	-0.0488**	0.0394	-0.0382	-0.0943***	-0.0719***	-0.0119
	(0.0248)	(0.0337)	(0.0233)	(0.0355)	(0.0241)	(0.0366)
Foreign Direct Investment	-0.0068	0.0040	-0.0098	-0.0378	-0.0298	-0.0014
	(0.0364)	(0.0545)	(0.0367)	(0.0558)	(0.0380)	(0.0436)
Industrial Share	0.7389***	1.2262***	0.7237***	0.8978***	0.7486***	1.1280***
	(0.0865)	(0.0915)	(0.0814)	(0.1135)	(0.0894)	(0.0987)
Energy Demand	-0.0208	-0.2145***	-0.0179	0.0662	0.0170	-0.0848**
	(0.0412)	(0.0560)	(0.0341)	(0.0612)	(0.0383)	(0.0395)

Note:
Standard errors in parentheses ()
*** p<0.01, ** p<0.05, * p<0.1 by Z-test

Table S46: Long Run Coefficient Estimation using Generalized Least Square with Water Vulnerability for SDG 7.3.1 – With Regulatory Quality, Rule of Law, and Voice and Accountability, Related to Figure 3

Variables	Model 1	Model 2	Model 1	Model 2	Model 1	Model 2
	Regulatory Quality		Rule of Law		Voice and Accountability	
Policy Stringency	-0.0504***	-0.0688	-0.0396**	0.2389**	-0.0189	0.0381
	(0.0165)	(0.1245)	(0.0156)	(0.0930)	(0.0150)	(0.1197)
Environmental Innovation	-0.0594**	0.5411***	-0.0359	0.8634***	-0.0082	-0.1316
	(0.0238)	(0.1561)	(0.0223)	(0.1408)	(0.0220)	(0.1691)
Foreign Direct Investment	-0.0170	-1.7408***	-0.0049	-0.0326	-0.0089	-0.0507
	(0.0368)	(0.4471)	(0.0363)	(0.2073)	(0.0348)	(0.1902)
Industrial Share	0.7534***	1.4284***	0.7083***	1.3338***	0.6795***	1.2895***
	(0.0828)	(0.4605)	(0.0802)	(0.4729)	(0.0785)	(0.4221)
Energy Demand	0.0122	-0.5887**	-0.0314	-1.1453***	-0.2037***	0.2064
	(0.0371)	(0.2399)	(0.0343)	(0.2355)	(0.0501)	(0.3569)
Water Vulnerability	-1.2474***	2.8609	-1.2668***	4.5855	-0.5926***	7.0111
	(0.1291)	(4.0091)	(0.1296)	(4.3230)	(0.1471)	(4.6613)
Quality of Governance	0.0066	0.2702	-0.0627**	2.2369***	-0.1764***	0.3418
	(0.0347)	(0.7367)	(0.0315)	(0.7567)	(0.0334)	(0.3841)
Policy Stringency * Water Vulnerability	-	0.1735	-	-0.5768***	-	-0.1917
	-	(0.3597)	-	(0.2195)	-	(0.2845)
Environmental Innovation * Water Vulnerability	-	-1.6592***	-	-1.9382***	-	0.3702
	-	(0.4012)	-	(0.3058)	-	(0.3939)
Foreign Direct Investment * Water Vulnerability	-	5.3203***	-	0.0508	-	0.0436
	-	(1.3945)	-	(0.5405)	-	(0.5155)
Industrial Share * Water Vulnerability	-	-1.1985	-	-1.4538	-	-1.0577
	-	(1.2296)	-	(1.0363)	-	(0.9999)
Energy Demand * Water Vulnerability	-	1.5538**	-	2.2180***	-	-0.9418
	-	(0.6723)	-	(0.5588)	-	(0.8732)
Policy Stringency * Quality of Governance	-	0.0494	-	0.6130*	-	0.5386**
	-	(0.3459)	-	(0.3306)	-	(0.2179)
Environmental Innovation * Quality of Governance	-	0.4665	-	1.2903***	-	0.8065***
	-	(0.4154)	-	(0.3754)	-	(0.2951)
Foreign Direct Investment * Quality of Governance	-	-4.2448***	-	1.4918**	-	0.0725
	-	(1.0404)	-	(0.5818)	-	(0.2933)
Industrial Share * Quality of Governance	-	0.0929	-	1.4844*	-	1.6665**
	-	(0.8983)	-	(0.7772)	-	(0.7042)
Energy Demand * Quality of Governance	-	-0.3734	-	-2.6528***	-	-1.8319***
	-	(0.5375)	-	(0.4901)	-	(0.5232)
Policy Stringency * Quality of Governance * Water Vulnerability	-	0.3088	-	-1.5145*	-	-1.4312***
	-	(1.0439)	-	(0.8569)	-	(0.5460)
Environmental Innovation * Quality of Governance * Water Vulnerability	-	-1.5519	-	-2.6050***	-	-2.0412***
	-	(1.1148)	-	(0.9094)	-	(0.7240)
Foreign Direct Investment * Quality of Governance * Water Vulnerability	-	14.1479***	-	-4.2802**	-	-0.3409

	-	(3.4762)	-	(1.7649)	-	(0.7820)
Industrial Share * Quality of Governance * Water Vulnerability	-	-0.3157	-	-5.4039***	-	-4.7363***
	-	(2.8042)	-	(2.0805)	-	(1.7986)
Energy Demand * Quality of Governance * Water Vulnerability	-	1.0746	-	5.9326***	-	4.7666***
	-	(1.6662)	-	(1.2438)	-	(1.3024)
Constant	0.3095	-1.8329	0.5494***	-1.4260	1.2947***	-2.7497
	(0.2180)	(1.5825)	(0.2004)	(1.7878)	(0.2783)	(1.8692)
Wald-chi2	552.4	3229	639.0	5027	643.7	2084
Observations	115	115	115	115	115	115
Heteroskedasticity controlled	Yes	Yes	Yes	Yes	Yes	Yes
Autocorrelation controlled	Yes	Yes	Yes	Yes	Yes	Yes
Cross-sectional correlation controlled	Yes	Yes	Yes	Yes	Yes	Yes
Marginal Impact						
Policy Stringency	-0.0504***	-0.0280	-0.0396**	-0.0119	-0.0189	-0.0360
	(0.0165)	(0.0188)	(0.0156)	(0.0283)	(0.0150)	(0.0276)
Environmental Innovation	-0.0594**	-0.0239	-0.0359	0.0269	-0.0082	-0.0157
	(0.0238)	(0.0355)	(0.0223)	(0.0334)	(0.0220)	(0.0408)
Foreign Direct Investment	-0.0170	-0.0850**	-0.0049	-0.0728	-0.0089	-0.0251
	(0.0368)	(0.0420)	(0.0363)	(0.0473)	(0.0348)	(0.0442)
Industrial Share	0.7534***	1.0069***	0.7083***	0.8574***	0.6795***	0.9173***
	(0.0828)	(0.0953)	(0.0802)	(0.0969)	(0.0785)	(0.1077)
Energy Demand	0.0122	-0.0476	-0.0314	-0.1053**	-0.2037***	-0.1022
	(0.0371)	(0.0513)	(0.0343)	(0.0504)	(0.0501)	(0.0709)

Note:
Standard errors in parentheses ()
*** p<0.01, ** p<0.05, * p<0.1 by Z-test

Table S47: Country-wise Policy Simulation Outcomes (Marginal Impacts) for 7.1.1 under Climate Vulnerability (Ecosystem, Food, and Health) and Governance Quality, Related to Figure 6

	Brazil	China	India	Russia	S. Africa	Brazil	China	India	Russia	S. Africa	Brazil	China	India	Russia	S. Africa
**Variables**	**CORR/EV**					**CORR/FV**					**CORR/HELV**				
Electricity Generation	0.1118	0.0188	0.6907	0.1982	0.0914	0.0858	0.0001	0.7599	-0.1167	0.1865	0.0018	0.0114	0.4414	0.0500	-0.0343
Financial Access	0.1831	0.0229	0.6907	0.0032	0.0387	0.0921	0.0539	2.9080	-0.0344	-0.1943	0.0802	-0.0070	0.4598	-0.0352	0.1053
Rural Population Growth	-0.0571	-0.0120	-0.4402	0.0220	-0.0010	-0.0302	-0.0016	-0.4449	-0.0305	-0.1307	0.0096	-0.0014	-0.3102	-0.0027	-0.1241
Income Inequality	-0.9979	0.0567	3.8694	0.0375	0.3636	-0.5816	0.1080	4.2079	0.3070	-0.1065	-0.1039	0.3836	-0.9076	0.1058	0.7693
Health Expenditure	-0.0037	-0.0008	-0.0076	0.0000	-0.0056	-0.0018	-0.0002	-0.0199	-0.0022	-0.0049	-0.0001	-0.0008	-0.0005	-0.0001	-0.0066
**Variables**	**GOVEFF/EV**					**GOVEFF/FV**					**GOVEFF/HELV**				
Electricity Generation	0.0862	0.0244	0.5816	0.0820	-0.0296	0.0680	0.0070	0.6997	-0.0841	0.3227	-0.0027	0.0147	0.4436	0.0346	0.1967
Financial Access	0.2347	-0.0017	1.9529	0.0048	0.1921	0.1203	0.0425	2.3054	-0.0246	-0.2280	0.0992	-0.0308	0.4563	-0.0242	0.2845
Rural Population Growth	-0.0353	-0.0240	-0.5704	0.0027	0.0103	-0.0317	-0.0059	-0.5031	-0.0154	-0.1018	0.0030	-0.0066	-0.4149	-0.0050	-0.0266
Income Inequality	-0.9839	-0.2479	3.6541	0.0564	0.9606	-0.5175	0.0929	5.0755	0.2895	0.9350	-0.2658	0.2919	-1.2536	0.0690	1.3641
Health Expenditure	-0.0024	-0.0008	-0.0189	-0.0011	-0.0188	-0.0020	-0.0007	-0.0244	-0.0020	-0.0051	-0.0011	-0.0001	0.0049	0.0002	-0.0044
**Variables**	**POLST/EV**					**POLST/FV**					**POLST/HELV**				
Electricity Generation	0.1061	0.0147	0.6982	-0.0068	0.0796	0.0610	0.0009	0.7731	-0.1596	0.6151	0.0079	0.0044	0.4871	-0.0275	-0.4150
Financial Access	0.1916	0.0658	2.3391	0.0153	0.2322	0.1090	0.0403	2.9241	-0.0261	0.0097	0.1397	-0.0051	-0.4935	-0.0162	0.5270
Rural Population Growth	-0.0356	-0.0151	-0.6105	0.0069	0.0529	-0.0230	-0.0008	-0.5259	-0.0120	-0.0781	-0.0106	-0.0018	-0.4812	-0.0117	0.0453
Income Inequality	-0.9746	0.3368	3.9895	0.0491	0.7572	-0.3686	0.0456	3.8519	0.3334	0.8303	-0.3320	-0.0568	-0.6074	0.1209	1.5028
Health Expenditure	-0.0032	-0.0008	-0.0167	-0.0003	-0.0131	-0.0018	-0.0002	-0.0232	-0.0022	-0.0159	-0.0011	-0.0001	0.0019	0.0009	-0.0183
**Variables**	**REG/EV**					**REG/FV**					**REG/HELV**				
Electricity Generation	0.0978	0.0140	0.6139	0.1066	0.1680	0.0561	0.0018	0.7281	0.0203	0.3556	0.0127	0.0088	0.6277	0.1172	0.4159
Financial Access	0.1966	0.0219	2.2882	-0.0490	0.3657	0.0335	0.0303	2.6871	-0.0140	0.1749	0.1039	-0.0242	0.8470	-0.0272	0.4647
Rural Population Growth	-0.0524	-0.0102	-0.7473	-0.0362	0.0317	-0.0214	-0.0006	-0.6531	-0.0180	-0.0647	-0.0102	0.0009	-0.6336	-0.0016	-0.0163
Income Inequality	-1.0484	0.3765	3.6158	0.2920	0.8296	-0.3418	0.0647	5.1480	0.1890	0.6763	-0.1822	-0.0035	-1.5292	0.0075	1.6992
Health Expenditure	-0.0033	-0.0008	-0.0137	-0.0043	-0.0129	-0.0021	0.0000	-0.0363	-0.0013	-0.0136	-0.0016	0.0004	0.0042	0.0003	-0.0209
**Variables**	**RULE/EV**					**RULE/FV**					**RULE/HELV**				
Electricity Generation	0.0756	0.0146	0.8384	0.1106	0.0689	0.0422	0.0055	0.7936	-0.0844	0.4706	0.0288	0.0235	0.2498	0.0507	-0.1772
Financial Access	0.0372	0.0095	3.1445	-0.0030	0.2365	0.0111	0.0468	3.2253	-0.0174	0.0837	0.0963	-0.0230	-2.0520	-0.0279	0.3941
Rural Population Growth	-0.0467	0.0035	-0.5929	0.0090	0.0297	-0.0130	-0.0025	-0.5101	-0.0090	-0.0501	0.0101	0.0014	-0.3567	0.0006	0.1367
Income Inequality	-0.6779	-0.3945	7.7318	0.1285	0.6822	-0.1690	0.0897	8.0853	0.2805	0.0099	-0.2447	0.4360	2.9657	0.0683	1.1321
Health Expenditure	-0.0025	-0.0008	-0.0163	-0.0008	-0.0123	-0.0018	-0.0001	-0.0341	-0.0018	-0.0058	-0.0018	-0.0006	0.0019	0.0006	-0.0136
**Variables**	**VOICE/EV**					**VOICE/FV**					**VOICE/HELV**				
Electricity Generation	0.0969	0.0148	0.7213	-0.0054	0.0021	0.0999	0.0005	0.7207	0.1067	0.3097	0.0552	0.0070	0.5915	0.0192	-0.4941
Financial Access	0.3920	0.0497	2.9740	-0.0035	0.2462	0.1779	0.0094	3.0891	0.0024	-0.1060	0.0888	-0.0159	1.1022	-0.0295	0.5632
Rural Population Growth	-0.0550	-0.0164	-0.5800	0.0108	0.0083	-0.0585	-0.0001	-0.5022	0.0019	-0.0997	-0.0371	0.0003	-0.6505	0.0041	-0.0717
Income Inequality	-1.4774	0.4141	4.9257	0.0887	0.8190	-0.3592	0.0181	4.4529	0.1058	0.9055	-0.3071	-0.0838	-0.8918	0.0701	1.4543
Health Expenditure	-0.0031	-0.0008	-0.0151	-0.0014	-0.0143	-0.0021	-0.0001	-0.0202	-0.0007	-0.0232	-0.0009	-0.0005	0.0026	-0.0002	-0.0198

Note:
EV: Ecosystem Vulnerability; FV: Food Vulnerability; HELV: Health Vulnerability
CORR: Control of Corruption; GOVEFF: Governance Effectiveness; POLST: Political Stability; REG: Regulatory Quality; RULE: Rule of Law; VOICE: Voice and Accountability

Table S48: Country-wise Policy Simulation Outcomes (Marginal Impacts) for 7.1.1 under Climate Vulnerability (Infrastructure and Water) and Governance Quality, Related to Figure 6

	Brazil	China	India	Russia	S. Africa	Brazil	China	India	Russia	S. Africa
**Variables**	**CORR/IV**					**CORR/WV**				
Electricity Generation	0.1251	0.0153	0.2524	0.0055	0.0158	-0.0714	0.0196	0.6064	0.0980	0.1205
Financial Access	0.1508	0.0337	0.7035	-0.0030	-0.0089	0.0389	0.0776	3.0332	-0.0134	-0.2202
Rural Population Growth	0.0066	0.0008	-0.1597	-0.0028	0.0004	-0.0037	-0.0142	-0.3026	0.0174	-0.1290
Income Inequality	-0.7324	0.2524	1.7106	0.0471	-0.0014	-0.0591	0.2491	3.1720	0.0581	-1.5392
Health Expenditure	-0.0039	-0.0011	-0.0050	-0.0003	-0.0002	0.0005	-0.0011	-0.0112	-0.0002	0.0217
**Variables**	**GOVEFF/IV**					**GOVEFF/WV**				
Electricity Generation	0.0790	0.0227	0.2348	0.0034	-0.0027	-0.0302	0.0235	0.9368	0.1748	0.0927
Financial Access	0.1863	0.0156	0.6405	-0.0050	-0.0186	0.0173	0.0480	3.7219	0.0002	-0.4720
Rural Population Growth	-0.0079	-0.0069	-0.1907	-0.0040	-0.0041	-0.0085	-0.0126	-0.4427	0.0047	-0.0846
Income Inequality	-0.6933	0.1461	1.0084	0.0524	0.0119	-0.2037	0.1757	4.5130	0.0687	-1.1722
Health Expenditure	-0.0015	-0.0007	-0.0021	-0.0004	-0.0003	-0.0010	-0.0007	-0.0111	-0.0007	0.0063
**Variables**	**POLST/IV**					**POLST/WV**				
Electricity Generation	0.1188	0.0037	0.1813	0.0003	0.0235	-0.0439	0.0154	0.7248	0.1187	0.5964
Financial Access	0.1282	0.0190	0.4110	-0.0031	-0.0039	0.0576	0.0439	3.4819	0.0174	-0.0503
Rural Population Growth	0.0001	0.0002	-0.1468	-0.0031	-0.0081	-0.0068	-0.0146	-0.3600	0.0009	-0.0239
Income Inequality	-0.6440	0.0116	1.1675	0.0308	-0.0099	-0.0965	0.0849	3.9588	0.1454	-0.5563
Health Expenditure	-0.0032	-0.0007	-0.0035	-0.0003	0.0001	-0.0005	-0.0008	-0.0157	0.0013	0.0066
**Variables**	**REG/IV**					**REG/WV**				
Electricity Generation	0.1087	0.0083	0.2253	0.0131	0.0173	-0.0295	0.0168	0.8641	0.1098	0.3871
Financial Access	0.1113	0.0046	0.7098	-0.0049	0.0071	0.0609	0.0610	5.1857	-0.0347	-0.1935
Rural Population Growth	-0.0198	0.0004	-0.1675	-0.0020	-0.0058	-0.0104	-0.0112	-0.4902	-0.0195	0.0361
Income Inequality	-0.5355	0.0270	1.1340	0.0516	0.0224	0.0130	0.0883	5.2887	0.0629	-0.4063
Health Expenditure	-0.0031	-0.0001	-0.0017	-0.0004	-0.0006	-0.0012	-0.0008	-0.0164	-0.0018	-0.0007
**Variables**	**RULE/IV**					**RULE/WV**				
Electricity Generation	0.1140	0.0194	0.2185	0.0157	0.0222	-0.0095	0.0231	0.7658	0.1308	0.5110
Financial Access	0.2506	0.0117	0.4435	-0.0092	-0.0047	-0.0109	0.0417	5.1359	-0.0304	0.0916
Rural Population Growth	0.0240	-0.0044	-0.1893	-0.0026	-0.0060	-0.0059	-0.0035	-0.4445	0.0031	-0.0307
Income Inequality	-1.2732	0.2630	1.2112	0.0553	-0.0388	0.1161	0.1838	3.1587	0.1016	-0.7148
Health Expenditure	-0.0036	-0.0011	-0.0010	-0.0004	0.0003	-0.0011	-0.0015	-0.0128	-0.0015	0.0188
**Variables**	**VOICE/IV**					**VOICE/WV**				
Electricity Generation	0.0968	0.0049	0.2227	0.0300	0.0144	0.0043	0.0170	0.7577	0.1022	0.4345
Financial Access	0.0820	0.0143	0.7993	-0.0187	-0.0116	-0.0746	0.0538	4.8624	-0.0157	-0.2256
Rural Population Growth	-0.0492	0.0033	-0.1805	-0.0024	-0.0057	-0.0381	-0.0159	-0.5078	0.0053	-0.0310
Income Inequality	-0.6911	-0.0728	1.0114	0.0512	-0.0035	0.2192	0.1473	8.5451	0.0025	-1.0411
Health Expenditure	-0.0026	-0.0007	-0.0050	-0.0004	-0.0001	-0.0005	-0.0010	-0.0147	-0.0009	0.0049

Note:
IV: Infrastructure Vulnerability; WV: Water Vulnerability
CORR: Control of Corruption; GOVEFF: Governance Effectiveness; POLST: Political Stability; REG: Regulatory Quality; RULE: Rule of Law; VOICE: Voice and Accountability

Table S49: Country-wise Policy Simulation Outcomes (Changes in Marginal Impacts) for 7.1.1 under Climate Vulnerability (Ecosystem, Food, and Health) and Governance Quality, Related to Figure 7

	Brazil	China	India	Russia	S. Africa	Brazil	China	India	Russia	S. Africa	Brazil	China	India	Russia	S. Africa
**Variables**	CORR/EV					CORR/FV					CORR/HELV				
Electricity Generation	0.86	1.14	-0.10	1.79	-0.27	0.19	-1.02	0.49	-0.08	-0.36	-0.97	-0.22	0.22	-0.71	-1.23
Financial Access	-123.07	24.44	-17.18	-1.20	-1.18	28.71	-6.92	-13.17	-2.74	-0.46	31.08	-2.00	-2.12	-0.20	-1.36
Rural Population Growth	-4.86	-6.00	3.71	1.08	-1.14	-2.91	-0.64	1.51	3.84	-20.22	-0.36	-1.50	0.73	-0.52	-30.55
Income Inequality	4.52	-15.18	-2.86	-0.67	2.07	1.83	-15.40	-73.43	1.52	-0.66	-0.46	8.94	-0.19	263.50	2.90
Health Expenditure	1.18	0.33	1.81	-1.00	-0.50	0.20	-0.33	-40.80	10.00	-0.78	-0.93	0.33	-1.16	-1.20	-0.70
**Variables**	GOVEFF/EV					GOVEFF/FV					GOVEFF/HELV				
Electricity Generation	0.38	1.46	-0.27	0.46	-1.13	-0.22	-4.18	-0.14	-0.40	0.49	-1.04	-0.01	-0.03	-0.82	-0.05
Financial Access	92.88	3.25	-8.98	-1.27	-1.83	8.70	-5.21	-3.45	-2.02	-0.18	11.10	-8.16	-1.67	-0.54	-2.11
Rural Population Growth	-3.37	7.89	4.54	-0.04	8.36	-2.92	-0.40	1.72	0.43	52.58	-0.80	1.87	1.25	-3.00	2659.00
Income Inequality	3.98	4.09	-2.61	0.13	0.41	1.07	-2.29	-6.75	1.56	0.13	0.33	12.27	-0.32	2.42	0.95
Health Expenditure	0.71	0.33	4.56	1.20	-0.24	0.82	1.33	8.76	19.00	-0.84	-0.31	-0.83	0.40	-0.33	-0.84
**Variables**	POLST/EV					POLST/FV					POLST/HELV				
Electricity Generation	0.60	1.67	-0.17	-1.23	-0.80	-0.11	-1.12	-0.04	0.03	0.39	-0.88	-0.53	-0.04	-1.15	-2.02
Financial Access	-44.55	20.93	-24.65	-1.61	-5.82	-110.00	-5.80	-5.50	-3.19	-1.05	13969.00	-2.19	-0.24	-0.74	-4.00
Rural Population Growth	-3.28	-5.44	4.32	0.13	2.62	-2.45	-0.82	1.77	1.50	-8.81	-1.68	-1.26	1.45	15.71	3.44
Income Inequality	4.00	13.52	-2.47	0.85	-0.12	0.81	2.80	-4.23	3.00	0.03	0.66	-1.36	-0.71	-3.62	1.04
Health Expenditure	1.13	0.14	5.19	-0.50	1.62	0.20	-0.33	28.00	10.00	-0.15	-0.31	-0.83	-0.44	0.80	0.03
**Variables**	REG/EV					REG/FV					REG/HELV				
Electricity Generation	0.66	0.44	-0.41	-0.64	0.22	-0.20	-1.45	-0.30	-1.18	0.97	-0.79	-0.37	-0.12	-0.28	2.39
Financial Access	-281.86	-2191.00	-4.44	-0.06	-3.91	8.31	-4.00	-3.10	-1.60	-1.88	24.98	59.50	-1.71	-0.23	-3.48
Rural Population Growth	-4.42	-2.96	3.89	-6.25	6.73	-2.34	-0.76	1.85	0.75	-36.94	-1.66	-0.90	1.56	-0.77	-9.58
Income Inequality	4.64	10.88	-1.84	-3.47	0.46	0.65	0.53	-2.79	1.20	0.45	-0.08	-1.02	-0.61	-5.17	2.06
Health Expenditure	0.94	0.33	0.80	9.75	-0.14	0.40	-1.00	4.76	5.50	-0.41	0.00	-1.67	-5.67	-0.25	-0.05
**Variables**	RULE/EV					RULE/FV					RULE/HELV				
Electricity Generation	0.23	1.47	0.13	-0.18	-0.61	-0.44	-1.81	0.22	-0.24	0.75	-0.55	1.80	-0.39	-0.82	-2.00
Financial Access	-11.33	22.75	-25.80	-0.95	-3.12	4.05	-6.09	-7.68	4.44	-1.38	30.06	-7.97	2.70	-0.73	-3.04
Rural Population Growth	-4.05	-9.75	5.10	-0.40	4.50	-1.80	-0.70	1.77	10.25	-10.63	-0.34	-1.67	0.87	-0.94	21.41
Income Inequality	2.59	-52.23	-4.88	1.00	0.23	-0.20	-3.13	-20.20	0.95	-0.98	0.25	12.97	-2.83	0.40	1.02
Health Expenditure	0.79	0.14	5.27	0.14	0.17	0.38	-0.75	55.83	5.00	-0.72	0.20	-0.25	-0.47	1.00	-0.29
**Variables**	VOICE/EV					VOICE/FV					VOICE/HELV				
Electricity Generation	0.42	2.08	-0.04	-1.03	-0.99	0.23	-1.06	0.29	17.40	-0.08	-0.20	-0.37	0.55	-0.89	-2.83
Financial Access	-23.02	25.16	-20.77	-0.83	-112.91	-13.80	-1.76	-14.33	-0.78	-0.30	-7.08	-4.12	-3.65	-0.31	-5.19
Rural Population Growth	-4.77	-6.29	4.66	0.77	0.11	-4.75	-0.98	0.63	-1.56	-27.95	-3.54	-0.95	1.47	-2.03	-16.93
Income Inequality	7.57	26.24	-3.38	1.59	0.30	0.86	2.62	-4.18	0.29	0.50	0.72	-1.52	-0.56	5.68	1.53
Health Expenditure	1.21	0.14	4.59	2.50	0.96	0.62	-0.67	13.43	69.00	0.13	-0.31	-0.29	0.24	-1.50	0.06

Note:
EV: Ecosystem Vulnerability; FV: Food Vulnerability; HELV: Health Vulnerability
CORR: Control of Corruption; GOVEFF: Governance Effectiveness; POLST: Political Stability; REG: Regulatory Quality; RULE: Rule of Law; VOICE: Voice and Accountability

Table S50: Country-wise Policy Simulation Outcomes (Changes in Marginal Impacts) for 7.1.1 under Climate Vulnerability (Infrastructure and Water) and Governance Quality, Related to Figure 7

	Brazil	China	India	Russia	S. Africa	Brazil	China	India	Russia	S. Africa
**Variables**			**CORR/IV**					**CORR/WV**		
Electricity Generation	1.20	0.07	-0.05	-1.46	0.98	4.58	0.53	0.06	0.42	-0.49
Financial Access	376.00	17.72	-2.61	-1.44	889.00	-1.53	44.65	-8.32	-1.84	-0.29
Rural Population Growth	-0.57	-0.81	0.59	-0.50	-1.33	-1.29	-4.09	0.99	-10.16	-31.00
Income Inequality	2.26	3.14	3.36	0.33	-0.98	-0.66	2.36	-7.00	-0.46	-11.43
Health Expenditure	1.44	0.83	-2.56	-2.00	19.00	-1.42	0.83	-113.00	-0.71	-1.94
**Variables**			**GOVEFF/IV**					**GOVEFF/WV**		
Electricity Generation	0.47	0.24	-0.28	-1.36	-1.33	3.87	0.74	0.16	1.84	-0.59
Financial Access	61.10	3.46	-2.16	-1.57	6.75	-1.27	-69.57	-5.78	-0.98	0.67
Rural Population Growth	-1.52	2.00	0.94	-0.27	2.42	-1.66	13.00	1.65	-1.67	46.00
Income Inequality	1.71	11.38	5.90	0.07	-1.19	0.17	3.07	-4.37	2.82	-2.74
Health Expenditure	0.07	0.00	-1.68	-2.33	0.50	-0.29	0.17	36.00	0.17	-1.20
**Variables**			**POLST/IV**					**POLST/WV**		
Electricity Generation	0.84	-0.54	-0.42	-1.03	46.00	-25.39	0.50	-0.11	1.23	0.35
Financial Access	-59.27	7.26	-1.71	-1.36	3.88	-1.96	3.06	-5.39	11.43	-0.74
Rural Population Growth	-0.99	-0.97	0.54	-0.28	4.79	-1.51	-2.72	1.06	-1.10	-3.52
Income Inequality	2.00	-0.93	3.93	-0.43	-0.87	-0.46	-0.51	-3.83	-4.41	-1.78
Health Expenditure	1.13	0.17	-2.13	-2.50	9.00	-0.64	0.33	38.25	-4.25	-1.34
**Variables**			**REG/IV**					**REG/WV**		
Electricity Generation	0.91	-0.38	-0.35	-2.32	6.21	2.60	0.08	-0.12	1.54	1.34
Financial Access	73.20	-2.92	-2.19	-1.62	-24.67	-1.90	9.52	-5.74	3469.00	-0.05
Rural Population Growth	-2.26	-0.96	0.61	-0.51	3.46	-1.82	-2.01	0.95	2.61	29.08
Income Inequality	1.40	-0.86	39.50	0.00	-1.36	-1.08	-0.59	-2.84	1.56	-1.79
Health Expenditure	0.94	-0.80	-1.61	-3.00	1.00	-0.08	0.60	2.73	3.50	-0.97
**Variables**			**RULE/IV**					**RULE/WV**		
Electricity Generation	0.93	1.49	-0.13	-2.25	1.44	0.36	1.82	0.30	0.54	1.51
Financial Access	-209.83	116.00	-2.13	-1.97	6.83	-0.83	9.43	-16.26	1.01	-1.39
Rural Population Growth	0.54	1.59	0.67	-0.42	6.50	-1.46	6.00	1.21	-2.29	-10.59
Income Inequality	4.65	6.17	1.81	0.10	-0.39	-1.67	2.43	-5.92	1.48	-2.45
Health Expenditure	1.57	0.38	-1.30	-3.00	29.00	-0.21	1.14	-129.00	2.00	-1.77
**Variables**			**VOICE/IV**					**VOICE/WV**		
Electricity Generation	0.59	-0.55	-0.25	7.57	-13.00	-1.40	0.73	0.21	-0.39	0.35
Financial Access	-3.38	22.83	-2.67	-3.79	0.87	0.18	5.73	-13.66	-0.11	0.35
Rural Population Growth	-4.26	-0.54	0.37	-0.33	3.75	-3.95	-2.83	0.80	0.51	-11.69
Income Inequality	1.92	-1.41	-70.27	0.05	-0.95	-2.23	-0.25	-6.58	-0.87	-3.04
Health Expenditure	0.73	0.00	-2.92	-3.00	0.00	-0.64	0.67	9.50	1.25	-1.23

Note:
IV: Infrastructure Vulnerability; WV: Water Vulnerability
CORR: Control of Corruption; GOVEFF: Governance Effectiveness; POLST: Political Stability; REG: Regulatory Quality; RULE: Rule of Law; VOICE: Voice and Accountability

Table S51: Country-wise Policy Simulation Outcomes (Marginal Impacts) for 7.1.2 under Climate Vulnerability (Ecosystem, Food, and Health) and Governance Quality, Related to Figure 6

	Brazil	China	India	Russia	S. Africa	Brazil	China	India	Russia	S. Africa	Brazil	China	India	Russia	S. Africa
**Variables**	**CORR/EV**					**CORR/FV**					**CORR/HELV**				
Renewable Electricity Generation	0.0940	0.3324	0.6544	-0.2117	0.1121	0.0554	0.2964	0.7592	-0.1239	0.0342	0.0429	0.0659	0.1703	-0.2447	0.1700
Financial Access	0.2993	0.9143	6.4465	-0.0646	0.8299	0.0692	0.7655	5.2777	-0.0847	-0.0730	0.0907	-0.0672	-0.1274	-0.1407	0.8571
Policy Stringency	0.0141	0.2067	0.3509	-0.0824	0.0765	0.0079	0.0283	0.5278	-0.1292	-0.1263	-0.0078	-0.0003	0.1433	-0.1720	0.2663
Per Capita Income	0.2245	0.4662	1.4260	-0.3297	0.7200	0.2063	0.4493	1.2649	-0.4203	0.3881	0.0492	0.1342	1.1192	-0.4263	0.8024
Urban Population Growth	-0.0812	-0.3095	-1.0111	0.1068	-0.1776	-0.0735	-0.2056	-0.8965	-0.0204	0.2166	-0.0194	0.0872	-0.2229	-0.0356	0.2377
**Variables**	**GOVEFF/EV**					**GOVEFF/FV**					**GOVEFF/HELV**				
Renewable Electricity Generation	0.1220	0.3403	0.7575	-0.1572	-0.0295	0.0517	0.2577	0.8526	-0.1201	-0.0355	0.0518	0.1084	0.1020	-0.1928	-0.0301
Financial Access	0.4035	0.5722	4.4057	0.0147	0.8093	0.0948	0.6664	4.3278	-0.1034	0.0158	0.0874	0.0665	1.4084	-0.1251	0.7506
Policy Stringency	0.0091	0.1533	0.5068	-0.1366	0.0988	0.0209	0.0511	0.5058	-0.1082	-0.0642	-0.0243	0.0310	0.0906	-0.1516	0.2386
Per Capita Income	0.2242	0.5273	1.1680	-0.2410	0.9614	0.1616	0.4709	1.2118	-0.3719	0.6174	0.0624	0.2011	0.8528	-0.3417	0.7187
Urban Population Growth	-0.0863	-0.2783	-1.1601	0.0917	0.1752	-0.0508	-0.1249	-1.0835	0.0155	0.1652	-0.0063	0.0380	-0.1513	-0.0017	0.2458
**Variables**	**POLST/EV**					**POLST/FV**					**POLST/HELV**				
Renewable Electricity Generation	0.1153	0.3119	0.9004	-0.2270	0.0780	0.0546	0.2259	0.7451	-0.2138	0.0653	0.0522	0.1528	0.0769	-0.2255	0.2037
Financial Access	0.3569	1.6235	5.2550	0.0408	1.0192	0.0593	0.9556	4.7693	-0.0992	0.2793	0.1341	0.1522	0.4159	-0.1413	1.3676
Policy Stringency	0.0113	0.2630	0.5297	-0.0840	0.1608	0.0231	0.0471	0.5594	-0.1067	-0.0049	-0.0279	0.0251	0.0620	-0.1173	0.4070
Per Capita Income	0.2440	0.4494	1.2733	-0.2416	0.8487	0.1673	0.3774	1.1175	-0.5287	0.2564	0.0539	0.2492	0.6713	-0.3886	1.1754
Urban Population Growth	-0.0866	-0.2514	-1.4825	0.1277	0.0949	-0.0379	-0.0749	-1.6304	0.0224	0.2771	-0.0136	-0.0422	-0.1711	0.0174	0.2804
**Variables**	**REG/EV**					**REG/FV**					**REG/HELV**				
Renewable Electricity Generation	0.1155	0.3114	0.9852	-0.2062	-0.0239	0.0504	0.2246	0.8276	-0.2195	0.0088	0.0442	0.1532	0.0675	-0.2424	0.1308
Financial Access	0.3613	0.9846	4.8295	0.0272	0.7209	-0.0448	-0.2256	4.6151	-0.0592	0.3274	0.1190	0.1302	3.0325	-0.0597	1.1374
Policy Stringency	0.0443	0.2323	0.6062	-0.0794	0.0625	0.0207	0.0634	0.5640	-0.0907	-0.1170	-0.0142	0.0288	0.0581	-0.1368	0.2475
Per Capita Income	0.2604	0.4640	1.1980	-0.3046	0.4979	0.1992	0.3646	1.1314	-0.3525	0.3338	0.0565	0.2304	0.7184	-0.3980	1.0231
Urban Population Growth	-0.0892	-0.2637	-1.4595	0.1090	0.1801	-0.0265	-0.0852	-2.3180	0.0396	0.2871	-0.0256	-0.0666	-0.1448	0.0206	0.2481
**Variables**	**RULE/EV**					**RULE/FV**					**RULE/HELV**				
Renewable Electricity Generation	0.0585	0.3491	0.9190	-0.2603	0.1053	0.0541	0.2479	0.7353	-0.1987	0.0627	0.0444	0.0866	0.2565	-0.2479	0.2032
Financial Access	0.2540	0.7207	6.7368	0.0372	0.9353	-0.0273	0.5704	5.6937	0.0352	0.1518	0.0885	0.0975	-2.9202	-0.1174	1.2247
Policy Stringency	0.0450	0.1542	0.5374	-0.0841	0.0684	0.0221	0.0641	0.4844	-0.0751	-0.1036	-0.0242	0.0350	0.1428	-0.1131	0.4186
Per Capita Income	0.2971	0.5531	1.3507	-0.2423	0.9001	0.1666	0.5003	1.2186	-0.4640	0.6151	0.0700	0.1751	0.8744	-0.3248	1.1130
Urban Population Growth	-0.0735	-0.2258	-1.5725	0.1724	-0.0228	-0.0379	-0.1460	-1.8696	0.1126	0.3799	-0.0230	0.0466	-0.2922	0.0502	0.4062
**Variables**	**VOICE/EV**					**VOICE/FV**					**VOICE/HELV**				
Renewable Electricity Generation	0.1350	0.3243	0.7766	-0.1264	0.1331	0.0710	0.2543	0.8235	-0.1976	0.0943	0.0460	0.1573	0.0889	-0.1487	0.2151
Financial Access	0.6784	1.4289	5.8874	-0.0627	1.0313	0.1844	0.2603	5.3775	-0.0554	0.2205	0.1072	0.2083	1.6341	-0.0371	1.2949
Policy Stringency	0.0449	0.2705	0.4479	-0.0901	0.2190	0.0258	0.0572	0.5385	-0.0899	0.0262	-0.0153	0.0356	0.0727	-0.1034	0.3496
Per Capita Income	0.2845	0.4636	1.2131	-0.2227	0.8626	0.2042	0.4117	1.1326	-0.5241	0.3073	0.0718	0.2722	0.7973	-0.3785	1.0597
Urban Population Growth	-0.1158	-0.2913	-1.2863	0.1881	0.0371	-0.0993	-0.1109	-2.8423	0.1353	0.2274	-0.0892	-0.0218	-0.0500	0.0923	0.0697

Note:
EV: Ecosystem Vulnerability; FV: Food Vulnerability; HELV: Health Vulnerability
CORR: Control of Corruption; GOVEFF: Governance Effectiveness; POLST: Political Stability; REG: Regulatory Quality; RULE: Rule of Law; VOICE: Voice and Accountability

Table S52: Country-wise Policy Simulation Outcomes (Marginal Impacts) for 7.1.2 under Climate Vulnerability (Infrastructure and Water) and Governance Quality, Related to Figure 6

	Brazil	China	India	Russia	S. Africa	Brazil	China	India	Russia	S. Africa
**Variables**	**CORR/IV**					**CORR/WV**				
Renewable Electricity Generation	0.0940	0.2426	0.4813	-0.4455	0.0876	0.0041	0.3380	0.5136	-0.0484	0.0337
Financial Access	0.2036	0.3799	3.2383	-0.5806	0.8458	-0.0636	1.3580	5.8240	0.0200	-0.0916
Policy Stringency	0.0010	-0.0201	0.3610	-0.1845	-0.0094	0.0148	0.1584	0.4320	-0.0417	-0.1953
Per Capita Income	0.2378	0.3564	0.9984	0.9219	0.6298	0.0507	0.4553	1.0740	-0.1652	0.2916
Urban Population Growth	-0.0603	-0.0948	-0.5078	-0.0437	-0.1584	-0.0191	-0.2758	-0.6478	0.0420	0.2104
**Variables**	**GOVEFF/IV**					**GOVEFF/WV**				
Renewable Electricity Generation	0.0607	0.1942	0.5066	0.3950	0.0079	-0.0012	0.3420	0.5708	-0.0531	-0.0979
Financial Access	0.2651	0.4727	3.6644	-0.2588	0.5234	-0.0713	0.9698	6.9319	-0.0118	0.0153
Policy Stringency	-0.0120	0.0268	0.3424	-0.4461	-0.1082	0.0143	0.1339	0.4477	-0.0298	-0.1762
Per Capita Income	0.1556	0.3156	1.0996	0.9727	0.4540	-0.0512	0.4580	1.1820	-0.1099	0.6549
Urban Population Growth	-0.0627	-0.0007	-0.4603	0.0104	-0.0679	-0.0376	-0.2493	-0.5289	0.0525	0.2088
**Variables**	**POLST/IV**					**POLST/WV**				
Renewable Electricity Generation	0.0804	0.2005	0.4720	-0.7011	0.1057	0.0002	0.2684	0.4544	-0.0781	-0.0752
Financial Access	0.1904	0.3761	3.4937	-0.4646	1.0636	-0.0297	0.9732	5.6912	-0.0013	0.2668
Policy Stringency	-0.0080	0.0398	0.3826	-0.3721	0.1862	0.0141	0.1667	0.3668	-0.0285	-0.2160
Per Capita Income	0.2219	0.3391	1.0176	1.2715	0.7950	-0.0243	0.3882	1.0293	-0.1682	0.3468
Urban Population Growth	-0.0609	-0.0371	-0.4629	0.0359	-0.2517	-0.0285	-0.1778	-0.5913	0.0609	0.2636
**Variables**	**REG/IV**					**REG/WV**				
Renewable Electricity Generation	0.0871	0.1871	0.5862	-0.5990	0.0308	0.0104	0.2850	0.5196	-0.0932	-0.0433
Financial Access	0.1644	0.3729	4.0398	-0.2328	0.6934	-0.0515	1.2791	7.9123	0.0222	-0.0505
Policy Stringency	0.0008	0.0261	0.4737	-0.2971	0.0430	0.0160	0.1934	0.5502	-0.0282	-0.1720
Per Capita Income	0.2473	0.3068	1.0642	0.2229	0.4426	0.0624	0.3921	0.9963	-0.1923	0.3055
Urban Population Growth	-0.0623	-0.0503	-0.5577	0.0042	-0.0817	-0.0253	-0.2467	-0.6271	0.0502	0.3004
**Variables**	**RULE/IV**					**RULE/WV**				
Renewable Electricity Generation	0.1163	0.2178	0.3894	-0.4874	0.0290	0.0110	0.3175	0.5830	-0.0981	-0.0439
Financial Access	0.3594	0.4027	0.9432	-0.3512	0.8899	-0.1162	0.8813	7.8748	0.0581	0.1878
Policy Stringency	0.0246	0.0299	0.2309	-0.3164	0.1051	0.0157	0.0856	0.4557	-0.0251	-0.2143
Per Capita Income	0.3826	0.3653	0.9245	0.7639	0.9787	-0.0024	0.4539	1.1067	-0.1492	0.3912
Urban Population Growth	-0.0771	-0.0592	-0.5271	-0.0020	-0.3275	-0.0297	-0.2409	-0.8017	0.0663	0.3793
**Variables**	**VOICE/IV**					**VOICE/WV**				
Renewable Electricity Generation	0.0961	0.2001	0.5511	-0.2490	0.1079	0.0022	0.2949	0.4854	-0.0930	-0.0147
Financial Access	0.2072	0.4024	4.3424	0.1885	1.0520	-0.1290	1.1591	7.6254	0.0184	0.1949
Policy Stringency	0.0306	0.0438	0.3456	-0.1667	0.2425	0.0187	0.2293	0.3887	-0.0223	-0.1039
Per Capita Income	0.2795	0.3526	1.0336	-0.4658	0.7882	-0.0121	0.4052	0.8860	-0.1325	0.4295
Urban Population Growth	-0.0935	-0.0509	-0.5847	0.1459	-0.2877	-0.0640	-0.2131	-0.6869	0.0799	0.2212

Note:
IV: Infrastructure Vulnerability; WV: Water Vulnerability
CORR: Control of Corruption; GOVEFF: Governance Effectiveness; POLST: Political Stability; REG: Regulatory Quality; RULE: Rule of Law; VOICE: Voice and Accountability

Table S53: Country-wise Policy Simulation Outcomes (Changes in Marginal Impacts) for 7.1.2 under Climate Vulnerability (Ecosystem, Food, and Health) and Governance Quality, Related to Figure 7

	Brazil	China	India	Russia	S. Africa	Brazil	China	India	Russia	S. Africa	Brazil	China	India	Russia	S. Africa
**Variables**	CORR/EV					CORR/FV					CORR/HELV				
Renewable Electricity Generation	2.12	-30.16	1.72	0.23	2.56	0.62	-13.78	2.50	-0.43	-0.19	0.58	3.92	-0.22	-0.04	0.60
Financial Access	1.60	2.89	3.36	-0.17	2.49	-0.31	1.14	1.84	-0.36	-1.33	0.25	-1.43	-1.07	-0.30	1.18
Policy Stringency	1.35	9.39	1.51	0.40	-4.23	-0.01	1.02	2.43	0.22	1.43	3.11	-1.01	-0.14	1.31	-7.56
Per Capita Income	0.99	0.41	2.72	0.37	0.29	0.73	0.69	4.18	-16.51	-0.60	-0.16	-0.24	-4.14	-5.12	0.94
Urban Population Growth	3.27	12.63	-12.53	1.89	443.00	3.51	4.27	-19.99	-1.38	17.05	-0.04	-5.30	62.69	-1.56	8.43
**Variables**	GOVEFF/EV					GOVEFF/FV					GOVEFF/HELV				
Renewable Electricity Generation	2.34	-84.00	2.41	-0.03	-2.52	0.45	56.27	4.63	-0.40	-2.99	0.93	2.49	-0.51	-0.15	-1.49
Financial Access	2.10	1.45	1.75	-1.17	1.07	-0.12	0.80	1.26	-0.39	-0.96	0.28	-0.62	-0.26	-0.33	0.15
Policy Stringency	-0.52	13.60	4.42	0.68	-3.98	0.06	25.89	3.75	0.27	0.16	6.84	1.31	-0.26	1.02	-5.16
Per Capita Income	0.70	0.50	1.85	1.00	0.58	0.35	0.77	2.79	-4.29	-0.25	0.31	0.20	-3.34	-4.26	0.68
Urban Population Growth	6.02	83.33	-15.78	1.11	-93.21	5.43	2.51	-13.70	-0.75	18.21	-0.70	-2.57	-90.00	-1.03	12.43
**Variables**	POLST/EV					POLST/FV					POLST/HELV				
Renewable Electricity Generation	2.39	-15.64	2.32	0.46	2.13	0.55	-9.16	2.14	0.06	-0.04	0.95	253.67	-0.64	-0.01	1.20
Financial Access	2.06	8.31	1.81	-1.80	1.63	-0.44	3.03	1.26	-0.12	-0.34	0.94	0.13	-0.78	-0.14	1.29
Policy Stringency	-0.34	11.23	3.15	-0.26	-5.77	0.19	1.15	2.85	-0.12	-0.93	8.62	0.02	-0.60	0.15	-7.99
Per Capita Income	0.79	0.24	3.94	0.48	0.29	0.39	0.24	5.81	-13.13	-0.74	0.12	0.14	-2.77	-5.22	0.75
Urban Population Growth	5.10	42.34	-30.89	2.25	-56.82	3.46	1.91	-71.58	-0.55	30.13	-0.34	1.20	8.25	-0.71	16.53
**Variables**	REG/EV					REG/FV					REG/HELV				
Renewable Electricity Generation	3.13	15.39	2.89	0.26	-3.66	0.58	-13.98	3.67	0.31	-0.89	0.68	19.99	-0.67	0.28	0.47
Financial Access	2.81	4.43	2.53	-1.30	1.18	-1.53	-1.65	1.68	-0.42	-0.45	0.75	-0.12	0.71	-0.59	0.67
Policy Stringency	4.03	13.25	3.30	-0.23	-3.12	1.23	4.82	3.06	-0.12	0.84	6.89	0.46	-0.62	0.48	-5.12
Per Capita Income	0.80	0.35	2.14	4.05	-0.19	0.31	0.34	2.48	12.51	-0.69	-0.13	0.23	-3.01	-10.52	0.32
Urban Population Growth	4.61	-24.76	-20.88	0.87	-53.97	0.74	2.05	-54.66	-0.36	22.34	0.30	2.54	8.34	-0.70	12.27
**Variables**	RULE/EV					RULE/FV					RULE/HELV				
Renewable Electricity Generation	0.46	-45.19	2.44	1.99	4.72	0.49	-7.39	2.13	0.05	25.13	0.66	-11.56	0.07	0.05	2.92
Financial Access	1.05	2.24	3.61	-0.47	1.60	-1.26	0.81	1.86	-1.36	-0.06	0.29	-0.25	-2.48	-0.38	1.52
Policy Stringency	1.46	11.54	3.55	-0.15	-3.05	0.08	2.79	2.47	-0.39	1.66	6.56	0.32	-0.09	0.10	-8.91
Per Capita Income	1.11	0.54	3.21	-0.28	0.26	0.53	0.80	7.84	-8.61	-0.45	0.84	-0.15	-2.77	-4.02	0.31
Urban Population Growth	4.40	563.50	-24.37	6.09	75.00	3.99	7.02	-123.20	1.03	36.25	0.07	-5.52	7.67	-0.23	20.49
**Variables**	VOICE/EV					VOICE/FV					VOICE/HELV				
Renewable Electricity Generation	2.38	539.50	-11.11	-0.18	3.24	0.94	-42.02	-10.83	-0.02	0.37	0.58	7.19	-2.42	-0.33	1.25
Financial Access	4.04	6.70	48.31	0.32	1.95	0.80	-0.13	18.23	-0.48	0.68	1.93	0.49	3.21	-0.76	2.09
Policy Stringency	1.45	20.81	-20.73	-0.21	-7.28	0.28	4.84	-28.20	-0.28	-1.53	4.28	0.87	3.75	-0.01	-7.17
Per Capita Income	1.27	0.29	-0.12	0.16	0.27	0.74	0.47	-0.14	28.78	-0.68	0.57	0.49	0.15	40.59	0.44
Urban Population Growth	6.67	363.13	-6.75	4.13	52.00	12.07	2.95	-14.67	1.93	13.30	3.62	0.15	-1.33	0.99	2.13

Note:
EV: Ecosystem Vulnerability; FV: Food Vulnerability; HELV: Health Vulnerability
CORR: Control of Corruption; GOVEFF: Governance Effectiveness; POLST: Political Stability; REG: Regulatory Quality; RULE: Rule of Law; VOICE: Voice and Accountability

Table S54: Country-wise Policy Simulation Outcomes (Changes in Marginal Impacts) for 7.1.2 under Climate Vulnerability (Infrastructure and Water) and Governance Quality, Related to Figure 7

	Brazil	China	India	Russia	S. Africa	Brazil	China	India	Russia	S. Africa
**Variables**			CORR/IV					CORR/WV		
Renewable Electricity Generation	1.85	3.26	0.79	0.65	1.35	-1.17	-14.31	0.92	-0.36	-1.47
Financial Access	1.03	0.18	0.96	3.20	1.63	-0.50	2.84	1.78	-1.31	-0.39
Policy Stringency	-0.90	2.65	2.79	1.38	-0.53	4.69	11.67	2.33	-0.31	8.77
Per Capita Income	0.71	1.73	10.84	296.39	0.25	-3.91	0.74	10.79	-2.81	-0.69
Urban Population Growth	3.50	4.48	-35.78	-1.90	20.12	0.87	5.49	-240.93	-0.17	-27.30
**Variables**			GOVEFF/IV					GOVEFF/WV		
Renewable Electricity Generation	0.64	2.74	1.13	-2.60	-0.68	-0.98	34.63	1.07	-0.35	0.22
Financial Access	1.14	0.47	1.21	0.76	0.38	-0.67	1.63	2.05	-0.85	-1.19
Policy Stringency	-1.56	-11.31	5.04	5.81	4.03	1.55	-38.19	4.13	-0.44	6.47
Per Capita Income	0.02	1.50	4.98	24.94	0.36	-0.25	0.77	16.33	-2.13	-0.31
Urban Population Growth	6.04	-0.97	-77.72	-0.79	6.46	7.74	5.58	-69.69	0.03	-23.70
**Variables**			POLST/IV					POLST/WV		
Renewable Electricity Generation	1.41	9.55	0.52	1.89	0.98	-1.00	-8.94	0.68	0.13	1.11
Financial Access	0.69	0.44	0.65	3.71	1.86	-0.87	3.35	1.67	-0.98	19.21
Policy Stringency	-1.40	3.10	3.86	2.64	-8.08	2.92	6.98	2.03	-0.55	6.08
Per Capita Income	0.39	0.63	-23.66	173.18	0.51	-0.63	0.23	13.62	-2.84	-0.63
Urban Population Growth	4.80	0.63	15.96	-0.13	31.27	3.67	6.17	57.54	0.17	-38.66
**Variables**			REG/IV					REG/WV		
Renewable Electricity Generation	1.87	2.71	1.32	2.46	-0.29	-1.27	-14.64	1.13	0.62	0.48
Financial Access	0.95	0.14	1.42	1.37	0.70	-0.72	2.77	3.11	-1.37	-1.33
Policy Stringency	-0.93	-6.02	4.61	1.95	-2.72	11.31	17.78	7.95	-0.53	4.55
Per Capita Income	0.50	1.31	9.08	-6.98	-0.21	-3.89	0.41	3.29	-4.42	-0.70
Urban Population Growth	4.51	1.23	34.08	-0.93	11.19	2.24	8.00	-61.88	-0.14	-54.64
**Variables**			RULE/IV					RULE/WV		
Renewable Electricity Generation	1.93	6.86	0.32	1.09	0.18	-1.23	-6.95	1.14	0.43	-0.21
Financial Access	2.01	0.38	-0.48	2.80	1.90	-0.47	1.96	2.68	-1.79	-11.61
Policy Stringency	0.17	5.50	2.51	2.00	-5.38	2.08	3.51	2.61	-0.61	6.29
Per Capita Income	1.34	1.28	-14.82	-63.61	0.48	-0.96	0.42	19.34	-2.42	-0.63
Urban Population Growth	6.79	4.06	12.69	-1.04	78.88	4.21	14.25	130.43	0.27	-211.72
**Variables**			VOICE/IV					VOICE/WV		
Renewable Electricity Generation	1.90	2.55	-67.40	0.05	0.75	-1.04	-37.41	-17.23	0.37	2.42
Financial Access	0.32	0.19	8.70	-3.01	3.13	-0.39	3.09	12.59	-1.37	-5.64
Policy Stringency	0.44	-9.94	-15.16	0.52	-9.48	3.45	29.57	-23.09	-0.66	1.95
Per Capita Income	0.65	2.06	-0.01	5.39	0.35	-0.85	0.42	-0.21	-3.76	-0.55
Urban Population Growth	8.44	1.35	-4.88	2.94	-1439.50	7.77	6.64	-4.99	0.83	62.20

Note:
IV: Infrastructure Vulnerability; WV: Water Vulnerability
CORR: Control of Corruption; GOVEFF: Governance Effectiveness; POLST: Political Stability; REG: Regulatory Quality; RULE: Rule of Law; VOICE: Voice and Accountability

Table S55: Country-wise Policy Simulation Outcomes (Marginal Impacts) for 7.2.1 under Climate Vulnerability (Ecosystem, Food, and Health) and Governance Quality, Related to Figure 6

	Brazil	China	India	Russia	S. Africa	Brazil	China	India	Russia	S. Africa	Brazil	China	India	Russia	S. Africa
**Variables**	**CORR/EV**					**CORR/FV**					**CORR/HELV**				
Environmental Innovation	-0.0009	1.4711	0.1822	-1.1364	-1.7103	0.0212	0.5164	0.1429	-2.4192	-0.7985	-0.2150	-0.1338	0.2551	-1.2074	-0.9304
Policy Stringency	0.0431	0.9918	0.1128	0.8935	-0.1202	0.0399	-0.4273	0.1108	-1.0831	0.6370	0.0399	-0.7074	0.0017	-0.9595	1.1384
Financial Development	0.4588	14.3902	-0.3785	5.4422	8.7540	0.9810	5.7635	-0.4513	3.1424	3.9957	0.9852	-1.8230	-0.5031	-0.4303	4.7892
Net Energy Import	-5.2222	6.8858	2.0194	6.0440	-25.5721	-4.7775	2.6375	1.8543	8.8161	-15.7888	-5.4342	0.3006	-1.7943	9.4332	19.0114
Low Carbon Trade	-0.0090	-1.1014	0.0505	-1.2718	-0.0956	0.0873	-0.1405	0.0775	-1.6844	-0.1110	0.0455	0.8072	-0.1754	-0.0494	-0.0784
**Variables**	**GOVEFF/EV**					**GOVEFF/FV**					**GOVEFF/HELV**				
Environmental Innovation	0.0891	1.0215	0.1204	-0.8940	-1.0448	0.0186	0.4722	0.2469	-1.3197	-0.7256	-0.1064	-0.3276	0.2281	-1.2507	-0.4705
Policy Stringency	-0.0492	0.2269	0.1271	-1.1372	-0.0528	-0.0237	-0.3310	0.1728	-0.5746	0.4406	-0.0554	-0.2224	0.1489	-0.6301	0.6884
Financial Development	0.4347	9.1870	-1.5596	0.7436	3.1389	0.7864	-1.7963	-0.9025	1.9517	2.9557	0.4680	-6.1168	-0.1409	-3.5452	3.2908
Net Energy Import	-4.0266	4.2714	0.5959	10.6848	-33.0620	-3.7220	3.6432	1.6514	4.3058	-40.6022	-5.0577	4.5333	-2.3367	7.1554	-39.0233
Low Carbon Trade	0.0163	-0.8985	-0.4370	0.1060	0.1367	0.0532	-0.2557	-0.2200	-0.1054	-0.0364	0.0301	0.3221	-0.2349	0.1839	0.0758
**Variables**	**POLST/EV**					**POLST/FV**					**POLST/HELV**				
Environmental Innovation	0.0389	1.1999	0.1456	-1.1646	-2.2863	0.0655	0.4748	0.1847	-1.4220	-0.9601	-0.1742	0.4368	-0.0322	-1.5213	-2.0924
Policy Stringency	-0.0525	1.5019	0.1756	-0.3866	0.5641	-0.0373	-0.2420	0.1693	-0.7166	1.0036	-0.0757	-0.5916	0.1574	-0.8272	1.2298
Financial Development	0.3886	12.8422	0.7268	1.4781	6.5713	1.1570	3.7811	0.2204	2.2618	4.0751	0.7381	2.0198	-0.0251	-0.5714	7.1258
Net Energy Import	-3.8642	6.8350	1.8295	11.9030	-4.0273	-3.4706	4.5564	1.6770	5.5561	-11.8856	-5.0340	1.3521	-4.6142	6.9873	71.5835
Low Carbon Trade	0.1459	-1.3884	0.3280	0.1017	0.6797	0.0095	0.0240	0.1864	-0.2233	0.0088	-0.0364	0.9659	-0.1243	0.6895	0.4311
**Variables**	**REG/EV**					**REG/FV**					**REG/HELV**				
Environmental Innovation	0.0894	1.2395	0.1294	-1.9098	-0.3152	0.0085	0.3127	0.3513	-1.1779	-0.6711	-0.1865	-0.0419	-0.0796	-1.3252	-0.8062
Policy Stringency	0.0054	1.2258	0.1009	0.7218	0.4501	0.0004	-0.6124	0.2365	0.2982	0.5265	-0.0090	-0.3564	0.0397	-0.1168	1.0989
Financial Development	0.4483	13.1908	-1.7410	0.3331	3.8636	1.1914	1.9653	-0.6663	-0.0277	3.5563	1.1199	0.9649	-0.9767	-3.8816	6.5884
Net Energy Import	-4.4064	6.8774	0.7245	8.5579	-34.6806	-3.8499	4.3001	2.0666	-0.5739	-6.8727	-6.1413	2.1524	-0.7259	4.0520	1.3129
Low Carbon Trade	0.0520	-1.3443	-0.1183	1.2495	0.4265	0.0285	-0.1921	-0.0554	0.5014	0.0183	-0.0279	0.8940	-0.2966	1.5639	0.4424
**Variables**	**RULE/EV**					**RULE/FV**					**RULE/HELV**				
Environmental Innovation	0.0330	0.9163	0.4651	-0.9440	-2.2661	-0.0123	0.5175	0.4462	-1.1928	-0.8748	-0.2455	0.0036	0.3352	-1.0314	-1.4903
Policy Stringency	0.0498	0.3431	0.1787	-0.3819	0.0696	-0.0071	-0.3123	0.2020	-0.3456	0.6736	-0.0875	-0.3397	0.1591	0.1914	1.2044
Financial Development	0.2388	6.4740	0.0428	-0.3901	8.2984	1.1692	2.6902	1.0526	0.2151	3.2971	0.8527	-0.8523	-0.8859	0.2527	6.2080
Net Energy Import	-4.6007	4.3963	3.0507	6.7981	-50.2474	-2.8834	0.7483	2.6700	-0.1355	13.6242	-5.5163	-0.7846	-3.4047	4.2685	66.7816
Low Carbon Trade	-0.0741	-0.7626	0.4225	0.2214	0.1685	-0.0468	-0.0630	-0.2918	0.3781	0.0560	-0.1417	0.5762	-0.0693	0.6584	0.4723
**Variables**	**VOICE/EV**					**VOICE/FV**					**VOICE/HELV**				
Environmental Innovation	0.0752	1.2669	0.1313	-1.5360	-2.1747	0.0130	0.2715	0.2442	-1.4371	-1.1836	-0.1586	-0.1928	0.0651	-1.9017	-1.2724
Policy Stringency	0.0269	1.6922	0.1376	-2.0884	0.9834	0.0968	-0.0012	0.1809	-0.9779	1.3769	0.0552	-0.3057	0.1669	-1.9158	1.2229
Financial Development	0.3851	12.8263	-1.3426	-2.6604	7.7080	0.7469	0.7090	-0.4272	-1.1825	5.2928	-1.0466	-2.6775	-0.7111	-0.6346	6.1851
Net Energy Import	-4.8280	7.2237	1.9522	9.9534	38.5026	-4.3323	3.6906	1.9680	5.9603	-35.2267	-4.9013	-2.3705	-1.7159	4.2387	54.7464
Low Carbon Trade	-0.0212	-1.2833	0.0154	0.5989	0.4566	-0.2519	-0.2071	-0.0571	0.7891	-0.0354	-0.1761	0.8037	-0.4001	1.7091	0.1084

Note:
EV: Ecosystem Vulnerability; FV: Food Vulnerability; HELV: Health Vulnerability
CORR: Control of Corruption; GOVEFF: Governance Effectiveness; POLST: Political Stability; REG: Regulatory Quality; RULE: Rule of Law; VOICE: Voice and Accountability

Table S56: Country-wise Policy Simulation Outcomes (Marginal Impacts) for 7.2.1 under Climate Vulnerability (Infrastructure and Water) and Governance Quality, Related to Figure 6

	Brazil	China	India	Russia	S. Africa	Brazil	China	India	Russia	S. Africa
**Variables**			**CORR/IV**					**CORR/WV**		
Environmental Innovation	0.2004	1.0172	0.1857	3.9500	-2.0898	0.0380	1.0883	0.1466	-0.5567	-0.6437
Policy Stringency	0.0409	-0.4870	0.0522	-4.1483	-0.7465	0.0479	0.4034	0.1079	0.7613	0.3439
Financial Development	0.8153	6.3069	-0.5088	-9.6480	6.5459	0.6490	12.0308	-0.3357	2.1184	3.1558
Net Energy Import	-6.6888	11.0825	0.2479	4.6224	-26.6477	-4.5260	6.9451	-0.3889	3.0557	-14.6432
Low Carbon Trade	-0.1333	0.2340	-0.0809	-7.0841	-0.2948	0.1845	-0.1372	-0.0574	-0.2137	0.0884
**Variables**			**GOVEFF/IV**					**GOVEFF/WV**		
Environmental Innovation	0.1916	0.5974	0.0372	7.2392	-1.2533	-0.0141	0.9242	0.3513	-0.9499	-0.5444
Policy Stringency	-0.0495	-0.1727	0.0696	-7.8930	-1.5624	-0.0241	0.2557	0.1890	0.5485	0.5642
Financial Development	0.6698	-6.7865	-0.3642	13.0432	4.1001	0.7842	10.2238	-0.4940	1.8225	2.5386
Net Energy Import	-5.0061	14.1561	-0.0684	1.0095	-35.7535	-3.6111	5.9545	1.3041	-2.4772	-21.8255
Low Carbon Trade	-0.2134	-0.0363	-0.1313	-4.4981	-0.0462	0.1496	-0.3865	-0.1081	0.0000	0.1675
**Variables**			**POLST/IV**					**POLST/WV**		
Environmental Innovation	0.0967	0.6680	0.1133	6.7078	-2.4061	-0.1153	1.0785	0.1003	-1.5059	-0.3123
Policy Stringency	-0.0624	-0.1214	0.0415	-5.8693	0.5258	-0.0314	0.4075	0.1057	0.2223	0.2045
Financial Development	0.5633	2.9472	-0.0683	-14.9106	6.2466	0.8553	11.0395	0.1553	1.1802	2.5858
Net Energy Import	-5.4076	14.6486	-0.1490	5.5202	-19.1554	-3.2819	7.1037	0.9510	-3.5993	4.2535
Low Carbon Trade	-0.1117	0.2518	-0.0362	-7.8430	0.3291	0.0916	0.1292	-0.0321	-0.1270	0.2373
**Variables**			**REG/IV**					**REG/WV**		
Environmental Innovation	0.1969	0.1827	0.0806	0.0591	-0.6418	0.1316	1.1457	0.0852	-1.9714	-0.1855
Policy Stringency	0.0344	-0.1133	-0.0161	-3.1612	0.6399	0.0189	0.7111	0.1258	0.6768	0.2311
Financial Development	0.8752	1.4544	-0.9008	-5.0535	6.4500	0.5152	13.2508	-0.8769	-0.2811	0.8148
Net Energy Import	-6.1350	9.9162	0.2331	2.0515	-15.2204	-3.0101	7.1814	0.5330	-1.1852	-10.0420
Low Carbon Trade	-0.0934	0.2711	-0.0293	-2.7951	0.4048	0.0827	-0.1719	0.0004	0.5134	0.1806
**Variables**			**RULE/IV**					**RULE/WV**		
Environmental Innovation	0.1701	1.2943	0.2310	-3.0321	-2.2650	-0.2155	0.3426	0.2468	-0.5122	-0.3928
Policy Stringency	0.0336	-0.1026	0.1726	-2.6534	0.4042	-0.0387	-0.5340	0.1956	0.1359	0.1247
Financial Development	0.7931	5.0619	-0.3917	-3.0044	7.3423	0.9364	4.9466	0.5455	1.3540	1.2160
Net Energy Import	-5.8104	12.1031	-0.0732	4.7003	-3.5339	-3.1895	4.7435	1.6075	-1.5334	-11.0721
Low Carbon Trade	-0.1344	0.1103	-0.1087	-2.0345	0.1034	-0.0619	0.8735	-0.0914	0.3659	0.1123
**Variables**			**VOICE/IV**					**VOICE/WV**		
Environmental Innovation	0.1896	-0.0269	0.1352	-0.9844	-2.6768	-0.1413	1.0049	-0.0163	-1.4432	-0.6217
Policy Stringency	0.0496	0.0127	0.0762	-1.2684	1.3706	0.0808	0.9482	0.1267	0.2849	1.5686
Financial Development	0.8473	-2.2770	-0.3832	0.8001	5.9555	0.8317	12.4306	-0.7295	-2.2074	2.9464
Net Energy Import	-5.5766	11.4000	0.6255	0.2448	32.6294	-5.7987	5.7773	0.1720	-0.0102	-3.3196
Low Carbon Trade	-0.2503	0.1249	-0.1264	-0.9699	0.4844	-0.1508	-0.3629	-0.0298	0.0852	0.2308

Note:
IV: Infrastructure Vulnerability; WV: Water Vulnerability
CORR: Control of Corruption; GOVEFF: Governance Effectiveness; POLST: Political Stability; REG: Regulatory Quality; RULE: Rule of Law; VOICE: Voice and Accountability

Table S57: Country-wise Policy Simulation Outcomes (Changes in Marginal Impacts) for 7.2.1 under Climate Vulnerability (Ecosystem, Food, and Health) and Governance Quality, Related to Figure 7

	Brazil	China	India	Russia	S. Africa	Brazil	China	India	Russia	S. Africa	Brazil	China	India	Russia	S. Africa
**Variables**	**CORR/EV**					**CORR/FV**					**CORR/HELV**				
Environmental Innovation	-1.02	0.94	-2.32	-0.08	0.72	-0.54	0.08	9.83	0.67	0.10	-4.60	-1.08	-16.94	-0.40	0.09
Policy Stringency	-1.85	-3.69	-0.27	3.71	-1.56	-1.66	-0.27	-0.03	2.51	1.32	-0.03	19.27	-0.98	2.09	3.71
Financial Development	2.37	2.79	-2.54	2.40	0.42	12.70	2.38	-3.10	0.33	-0.09	0.50	-1.48	-3.26	-1.15	-0.19
Net Energy Import	0.17	7.95	1.44	-0.10	-0.50	0.17	-21.49	5.34	-20.54	-0.62	0.78	0.38	4.99	-3.23	-1.41
Low Carbon Trade	-0.88	0.59	-1.26	-13.38	-1.34	-2.53	-0.43	-1.35	-11.60	-1.42	-1.32	-2.70	-0.20	-1.18	-1.27
**Variables**	**GOVEFF/EV**					**GOVEFF/FV**					**GOVEFF/HELV**				
Environmental Innovation	0.48	0.44	-1.26	-0.53	0.34	-0.72	0.14	-10.57	-0.39	0.13	-2.99	-1.17	-7.03	-0.48	-0.20
Policy Stringency	-0.43	-1.38	-0.47	-2.87	-1.26	-0.73	-0.38	0.03	-2.31	0.74	-2.41	-0.22	-0.03	-2.93	2.01
Financial Development	19.22	0.37	56.55	-0.62	-0.43	42.69	-8.43	-13.21	-0.28	-0.25	-0.24	-2.11	14.32	-2.15	-0.30
Net Energy Import	-0.08	2.16	-0.67	3.41	-0.36	-0.08	-15.41	3.37	-2.46	-0.06	0.57	-2.55	7.23	-2.38	-0.13
Low Carbon Trade	-1.43	0.25	2.35	0.12	-0.50	-2.81	0.13	0.22	-1.90	-1.15	-1.21	-1.83	0.19	-0.06	-0.71
**Variables**	**POLST/EV**					**POLST/FV**					**POLST/HELV**				
Environmental Innovation	-0.18	1.17	-1.54	-0.15	3.77	0.01	-0.04	5.67	-0.09	0.93	-5.06	-0.79	-9.47	-0.23	3.04
Policy Stringency	-0.45	-4.27	-0.14	-2.58	0.75	-0.62	-0.58	0.26	5.36	2.19	-3.86	-9.49	0.38	3.91	2.78
Financial Development	16.27	2.83	3.06	-0.32	-0.11	11.97	1.31	1.08	-0.27	-0.20	0.24	-2.34	-1.28	-1.15	-0.02
Net Energy Import	-0.04	4.59	0.97	1.45	-0.92	-0.08	-12.87	-46.45	-9.56	-0.71	0.65	-1.30	3.28	-2.51	-2.50
Low Carbon Trade	-5.71	0.83	-3.33	-0.08	0.54	-1.51	-1.10	-2.10	-2.63	-0.97	-0.73	-3.86	-0.33	1.75	0.01
**Variables**	**REG/EV**					**REG/FV**					**REG/HELV**				
Environmental Innovation	0.51	-9.07	-1.43	1.42	-0.42	-0.87	-0.24	3.36	0.60	0.13	-6.10	-1.02	-2.04	0.66	0.35
Policy Stringency	-1.06	-3.22	-0.37	0.51	0.55	-1.00	0.11	2.58	-0.17	0.76	-1.24	-3.09	-0.27	-1.46	2.71
Financial Development	20.05	1.54	32.04	-0.76	0.06	29.32	0.08	3.23	-1.02	-0.13	0.54	-1.82	4.18	-2.94	0.33
Net Energy Import	0.02	1.19	-0.54	0.31	-0.27	-0.04	-21.59	3.53	-1.16	-0.83	1.12	-1.57	2.64	0.83	-1.03
Low Carbon Trade	-2.42	0.32	-0.20	9.86	-0.04	-2.20	-0.33	-0.71	2.96	-0.95	-0.81	-3.45	0.43	9.04	0.02
**Variables**	**RULE/EV**					**RULE/FV**					**RULE/HELV**				
Environmental Innovation	-0.32	0.11	-2.46	-0.16	1.37	-1.21	0.08	15.71	0.26	0.27	-6.78	-1.00	16.74	-0.41	0.86
Policy Stringency	-1.54	-1.44	-0.27	-2.43	-0.76	-0.92	-0.47	0.18	0.00	0.99	-4.40	-0.33	-0.04	-1.65	2.70
Financial Development	0.44	0.29	-0.87	-1.30	0.20	4.12	0.49	2.61	-0.92	-0.11	0.26	-1.31	-4.04	-0.91	-0.04
Net Energy Import	0.20	0.46	1.02	0.05	-0.07	-0.22	-10.03	3.80	-0.94	-1.35	0.81	-2.16	52.53	-1.97	-2.42
Low Carbon Trade	0.49	-0.07	-3.50	1.67	-0.54	0.01	-0.75	0.35	1.46	-0.82	-0.04	-2.03	-0.71	1.74	0.26
**Variables**	**VOICE/EV**					**VOICE/FV**					**VOICE/HELV**				
Environmental Innovation	-4.33	0.70	-1.25	0.20	2.10	-1.32	-0.47	-14.20	-0.08	0.79	32.04	-1.09	-2.27	-0.15	0.81
Policy Stringency	-1.52	-6.47	-0.45	-11.04	2.16	-2.91	-1.00	0.14	0.93	3.55	0.32	-2.01	-0.10	3.01	2.88
Financial Development	0.16	6.89	11.81	-2.78	0.04	1.51	0.27	-23.48	-1.69	0.16	-2.44	-0.45	-3.32	-1.30	-0.11
Net Energy Import	0.14	11.86	-0.03	0.52	-1.73	0.04	-66.09	2.77	7.54	-0.14	0.43	-0.57	3.78	-2.70	-2.16
Low Carbon Trade	-0.52	0.82	-1.24	4.54	0.16	4.50	-0.24	-0.57	4.36	-1.12	0.34	-3.42	1.11	5.96	-0.71

Note:
EV: Ecosystem Vulnerability; FV: Food Vulnerability; HELV: Health Vulnerability
CORR: Control of Corruption; GOVEFF: Governance Effectiveness; POLST: Political Stability; REG: Regulatory Quality; RULE: Rule of Law; VOICE: Voice and Accountability

Table S58: Country-wise Policy Simulation Outcomes (Changes in Marginal Impacts) for 7.2.1 under Climate Vulnerability (Infrastructure and Water) and Governance Quality, Related to Figure 7

	Brazil	China	India	Russia	S. Africa	Brazil	China	India	Russia	S. Africa
**Variables**	CORR/IV					CORR/WV				
Environmental Innovation	6.08	-0.29	115.06	-3.47	1.11	-0.68	-0.35	13.66	-0.21	0.36
Policy Stringency	67.17	1.53	-0.46	23.52	-4.67	-2.14	-9.17	-0.06	-0.51	0.06
Financial Development	0.84	16.44	-5.77	-7.74	0.03	0.91	5.56	-2.53	-0.39	-0.06
Net Energy Import	1.60	-2.35	-4.54	-10.78	-0.46	0.22	-3.80	-2.17	-1.69	-0.65
Low Carbon Trade	0.46	-1.57	-0.59	-62.23	-2.09	-2.93	-0.68	-0.74	-2.56	-0.75
**Variables**	GOVEFF/IV					GOVEFF/WV				
Environmental Innovation	3.88	-0.60	-2.33	-4.08	0.71	-1.13	-0.53	12.16	-0.07	0.28
Policy Stringency	1.06	0.32	-0.46	-11.87	-8.65	-0.70	-1.82	0.17	-0.60	0.76
Financial Development	0.81	1.11	0.99	5.54	-0.25	4.14	0.94	3.33	-0.56	-0.22
Net Energy Import	0.88	-2.20	-0.83	-1.28	-0.31	-0.07	-2.83	4.75	-0.40	-0.49
Low Carbon Trade	2.18	-0.90	-0.21	-101.40	-1.17	-3.94	0.01	-0.50	-1.00	-0.52
**Variables**	POLST/IV					POLST/WV				
Environmental Innovation	2.10	-0.49	3.21	-5.30	3.25	11.27	-0.46	2.08	1.87	0.79
Policy Stringency	0.91	-0.59	-0.58	-350.36	0.68	-0.72	2.18	-0.20	-0.80	-0.45
Financial Development	0.63	-6.72	-0.24	-6.63	-0.18	-6.29	-14.89	0.67	-0.72	-0.37
Net Energy Import	1.17	-2.56	-0.82	-5.98	-0.63	-0.19	-2.52	-4.29	0.23	-1.10
Low Carbon Trade	0.76	-1.60	-0.76	-95.49	-0.25	6.16	-1.38	-0.82	-2.19	-0.46
**Variables**	REG/IV					REG/WV				
Environmental Innovation	3.69	-0.88	-0.31	-1.08	0.05	0.55	-0.44	-0.09	8.46	-0.30
Policy Stringency	-3.23	-0.44	-0.28	-12.55	1.23	-1.22	1.79	0.63	-0.47	-0.34
Financial Development	1.42	-2.13	0.59	-4.06	0.36	4.29	-14.32	4.19	-1.08	-0.66
Net Energy Import	1.36	-1.95	-1.41	-0.21	-0.68	-0.23	-2.26	1.33	0.19	-0.75
Low Carbon Trade	0.26	-1.73	-0.83	-24.73	-0.08	-3.14	-0.49	-1.00	3.78	-0.58
**Variables**	RULE/IV					RULE/WV				
Environmental Innovation	3.63	-0.20	8.75	1.28	1.37	0.72	-0.83	4.67	0.11	0.10
Policy Stringency	-2.24	-0.74	0.19	12.26	0.36	-0.70	0.13	0.10	-0.88	-0.63
Financial Development	1.18	16.66	-3.95	-2.66	0.05	-5.92	1.22	1.08	-0.67	-0.66
Net Energy Import	1.26	-2.66	-0.58	-20.77	-0.93	-0.26	-6.32	3.48	-0.47	-0.74
Low Carbon Trade	0.95	-1.24	-0.47	-23.43	-0.72	-3.39	-2.62	-0.61	2.64	-0.69
**Variables**	VOICE/IV					VOICE/WV				
Environmental Innovation	1.60	-1.02	-5.02	-0.47	2.58	-22.74	-0.50	-2.20	0.97	0.98
Policy Stringency	-2.68	-1.23	-0.51	2.44	3.51	-2.78	1.81	-0.38	-0.73	3.30
Financial Development	2.05	-0.44	-6.85	2.84	-0.21	0.89	-4.49	-3.51	-1.66	-0.22
Net Energy Import	1.48	-2.11	-2.48	-0.91	-1.62	0.46	-2.20	-0.39	-0.99	-0.92
Low Carbon Trade	2.60	-1.28	-0.17	-23.77	0.23	1.54	0.05	-0.86	0.08	-0.43

Note:
IV: Infrastructure Vulnerability; WV: Water Vulnerability
CORR: Control of Corruption; GOVEFF: Governance Effectiveness; POLST: Political Stability; REG: Regulatory Quality; RULE: Rule of Law; VOICE: Voice and Accountability

Table S59: Country-wise Policy Simulation Outcomes (Marginal Impacts) for 7.3.1 under Climate Vulnerability (Ecosystem, Food, and Health) and Governance Quality, Related to Figure 6

	Brazil	China	India	Russia	S. Africa	Brazil	China	India	Russia	S. Africa	Brazil	China	India	Russia	S. Africa
**Variables**	CORR/EV					CORR/FV					CORR/HELV				
Policy Stringency	0.0323	-0.2147	-0.1289	-0.2320	0.1813	0.0201	0.0732	-0.1775	-0.3307	0.0317	0.0214	0.0704	-0.0865	-0.2091	-0.1260
Environmental Innovation	0.0148	-0.2382	-0.2465	-0.1140	0.1308	0.0050	-0.1525	-0.1533	0.0493	-0.0595	-0.0006	0.0407	-0.1613	-0.1105	-0.0337
Foreign Direct Investment	-0.0047	1.7806	-0.2865	0.2599	-0.0992	0.0578	0.6829	-0.1529	-0.1148	0.1459	-0.0082	0.5921	0.1080	-0.3826	0.0564
Industrial Share	0.0757	2.5470	0.8880	0.4920	1.2527	0.0347	1.6088	0.3453	0.8281	0.3408	0.0608	1.1326	0.0996	1.0485	0.5601
Energy Demand	0.0003	-0.4977	-0.3532	-0.2961	1.0801	0.0340	-0.4862	-0.3486	-0.9483	0.1705	-0.0483	0.1798	-0.0871	-0.6732	0.1034
**Variables**	GOVEFF/EV					GOVEFF/FV					GOVEFF/HELV				
Policy Stringency	0.0692	-0.1717	-0.1807	0.0163	-0.0480	0.0652	0.0259	-0.1890	-0.1982	0.0462	0.0582	0.0287	-0.1337	-0.2351	-0.1282
Environmental Innovation	0.0660	-0.2027	-0.2256	-0.0127	0.0891	0.0344	-0.1980	-0.2205	-0.1087	-0.0320	-0.0948	-0.0424	-0.1265	-0.1300	-0.0392
Foreign Direct Investment	0.0498	1.5163	0.3477	0.0262	0.0195	0.0607	0.6036	0.1891	0.0188	0.0757	-0.0361	0.5780	0.1503	-0.3988	0.0835
Industrial Share	-0.1389	2.1657	0.9081	0.4118	1.1528	-0.1493	1.5087	0.3762	0.5776	0.1229	0.0833	0.9245	0.2247	0.8603	1.1169
Energy Demand	0.0553	-0.5536	-0.3095	-1.1013	0.8648	0.1212	-0.5542	-0.3610	-1.2516	0.3134	-0.1201	-0.0044	0.0682	-0.7592	-0.2696
**Variables**	POLST/EV					POLST/FV					POLST/HELV				
Policy Stringency	0.0635	-0.2021	-0.1965	-0.0840	-0.0293	0.0657	0.0544	-0.2210	-0.2078	0.0543	0.0627	-0.0053	-0.1576	-0.1543	-0.1919
Environmental Innovation	0.0236	-0.1837	-0.2437	-0.0623	0.2212	0.0210	-0.0937	-0.1611	-0.1050	-0.0095	-0.0558	0.0208	-0.0422	-0.1106	0.2439
Foreign Direct Investment	0.0502	2.3946	-0.2363	0.0495	0.1922	0.0514	0.8704	-0.5736	0.0089	0.0418	-0.0427	0.6114	-0.0824	-0.2281	0.1336
Industrial Share	-0.1974	2.5391	0.9255	0.4281	1.0891	-0.2361	1.5581	0.3063	0.5482	0.3761	-0.0078	0.8518	0.0065	0.8019	1.3069
Energy Demand	0.0188	-0.4015	-0.3599	-0.8805	-0.2087	0.0853	-0.2468	-0.4281	-1.1625	-0.1520	-0.0560	0.0095	0.0955	-0.9324	-1.6255
**Variables**	REG/EV					REG/FV					REG/HELV				
Policy Stringency	0.0431	-0.2248	-0.1849	-0.1438	-0.0985	0.0470	0.0229	-0.2274	-0.2481	-0.0224	0.0360	0.0036	-0.1093	-0.1201	-0.1941
Environmental Innovation	0.0292	-0.2113	-0.2515	0.1550	-0.1564	0.0059	-0.1311	-0.2612	-0.0530	-0.0617	-0.0382	-0.0086	0.0069	-0.0394	-0.0226
Foreign Direct Investment	-0.0020	2.4674	-0.1230	-0.1546	0.0973	0.0607	0.7996	-0.1134	-0.1210	0.0389	-0.0364	0.4071	-0.0151	-0.3341	0.1564
Industrial Share	-0.0988	2.5401	0.9317	0.7731	1.2753	-0.1260	1.5905	0.3720	0.7241	0.1782	0.0644	0.8608	0.0494	0.7658	1.8547
Energy Demand	0.0248	-0.4490	-0.3317	-0.6520	-0.1345	0.0836	-0.3094	-0.3855	-1.0702	0.2339	-0.0610	0.0233	-0.0847	-0.7344	-0.6035
**Variables**	RULE/EV					RULE/FV					RULE/HELV				
Policy Stringency	0.0590	-0.1547	-0.1916	-0.0321	0.0096	0.0568	0.0183	-0.2029	-0.0564	0.0465	0.0495	0.0171	-0.1288	0.0977	-0.1604
Environmental Innovation	0.0556	-0.1819	-0.3842	-0.0311	0.2986	0.0711	-0.1860	-0.3291	0.1313	-0.0385	-0.0253	-0.0628	-0.2100	-0.0927	0.0899
Foreign Direct Investment	0.0959	1.0226	-0.8395	-0.2225	0.1864	0.1325	0.1676	-0.1897	-0.1315	0.0179	0.0579	0.2895	0.1119	-0.3932	-0.0102
Industrial Share	-0.1964	1.4191	1.1500	0.5746	1.8917	-0.1987	0.9680	0.4771	0.3580	0.0164	-0.0700	0.6688	0.1849	0.5734	1.2739
Energy Demand	0.1180	-0.4902	-0.4454	-0.6025	0.8581	0.1826	-0.4805	-0.4428	-0.4624	-0.0193	-0.0281	0.0636	-0.0974	-0.9351	-0.7964
**Variables**	VOICE/EV					VOICE/FV					VOICE/HELV				
Policy Stringency	0.0663	-0.2299	-0.1802	0.1112	-0.1100	0.0954	0.0045	-0.2118	-0.0122	-0.1913	0.0641	-0.0342	-0.1575	0.0753	-0.2040
Environmental Innovation	0.0206	-0.2053	-0.2590	0.0989	0.2356	0.0449	-0.1216	-0.2231	0.1022	0.0319	-0.0832	0.0254	-0.1778	0.1030	0.0919
Foreign Direct Investment	0.0370	2.4105	-0.2850	0.1324	0.2851	0.0340	0.9963	-0.2151	0.1559	0.1737	-0.0047	0.6230	0.0606	0.0272	0.3181
Industrial Share	-0.1377	2.7917	1.0636	0.2332	2.0555	-0.1132	1.7450	0.6959	0.3594	0.4409	-0.0220	1.1143	0.1035	0.3329	1.2479
Energy Demand	0.0393	-0.4453	-0.3941	-1.2711	-0.6755	0.1091	-0.3239	-0.3870	-0.7882	0.4467	-0.0364	0.0098	-0.0188	-0.6344	-0.9634

Note:
EV: Ecosystem Vulnerability; FV: Food Vulnerability; HELV: Health Vulnerability
CORR: Control of Corruption; GOVEFF: Governance Effectiveness; POLST: Political Stability; REG: Regulatory Quality; RULE: Rule of Law; VOICE: Voice and Accountability

Table S60: Country-wise Policy Simulation Outcomes (Marginal Impacts) for 7.3.1 under Climate Vulnerability (Infrastructure and Water) and Governance Quality, Related to Figure 6

	Brazil	China	India	Russia	S. Africa	Brazil	China	India	Russia	S. Africa
**Variables**	**CORR/IV**					**CORR/WV**				
Policy Stringency	0.0231	0.1114	-0.1325	-3.4889	0.1785	0.0189	-0.1412	-0.1545	-0.1214	0.0743
Environmental Innovation	-0.0073	-0.1636	-0.1745	-3.1688	0.1996	0.0052	-0.2035	-0.1581	0.1796	-0.0937
Foreign Direct Investment	-0.0194	0.6358	-0.0437	0.0656	-0.0743	0.0198	1.2232	-0.0952	-0.3726	0.0574
Industrial Share	0.1358	1.4658	0.0150	-2.1444	0.8055	0.1838	1.8440	0.1970	0.4382	0.6451
Energy Demand	-0.0230	-0.3507	-0.2120	-5.8081	0.9159	0.0988	-0.4692	-0.2953	-0.4703	0.2177
**Variables**	**GOVEFF/IV**					**GOVEFF/WV**				
Policy Stringency	0.0591	0.0640	-0.1502	-3.8322	0.4649	0.0535	-0.1391	-0.1849	-0.0010	0.0594
Environmental Innovation	-0.0067	-0.1351	-0.1381	-2.3009	0.1334	0.0527	-0.1871	-0.2003	0.1462	-0.0577
Foreign Direct Investment	-0.0586	0.6148	0.0447	-0.6183	-0.0227	0.0128	0.9710	0.0157	-0.2253	0.0465
Industrial Share	-0.0337	1.1706	0.3555	8.9056	0.6388	0.0356	1.5350	0.2038	-0.1357	0.4655
Energy Demand	0.0248	-0.1425	-0.1713	-7.2490	0.9728	0.2231	-0.3779	-0.3459	-0.3795	0.2792
**Variables**	**POLST/IV**					**POLST/WV**				
Policy Stringency	0.0677	-0.0361	-0.1503	-3.0478	0.0340	0.0577	-0.1477	-0.1690	0.0022	0.2107
Environmental Innovation	0.0002	-0.0975	-0.1528	-3.3733	0.2208	-0.0401	-0.1384	-0.1593	0.1591	-0.1035
Foreign Direct Investment	-0.0352	0.8061	-0.0818	-0.4398	0.1484	-0.0277	1.1051	-0.1871	-0.2570	0.0643
Industrial Share	-0.0227	1.4730	-0.0575	-3.7944	0.6131	-0.0007	1.7954	0.0035	-0.0982	0.2225
Energy Demand	0.0142	-0.2432	-0.1554	-6.6890	0.7129	0.1400	-0.2997	-0.3525	-0.5521	-0.3383
**Variables**	**REG/IV**					**REG/WV**				
Policy Stringency	0.0325	-0.0003	-0.1481	-2.1830	-0.0530	0.0376	-0.1474	-0.2055	-0.0090	0.0610
Environmental Innovation	-0.0284	-0.0944	-0.1499	-1.9886	-0.0801	-0.0304	-0.1566	-0.1179	0.2342	-0.1598
Foreign Direct Investment	-0.0797	0.5303	-0.1299	-0.0042	0.0401	-0.0317	1.0065	-0.3396	-0.3117	0.0318
Industrial Share	0.0212	1.3987	0.0218	-0.7201	0.7517	0.0752	2.0271	0.0871	0.2016	1.1204
Energy Demand	-0.0152	-0.1729	-0.1634	-2.8884	-0.3408	0.1344	-0.3069	-0.2974	-0.5265	-0.0800
**Variables**	**RULE/IV**					**RULE/WV**				
Policy Stringency	0.0402	0.0394	-0.1597	-2.2854	0.0246	0.0568	-0.0547	-0.1980	0.2097	0.1249
Environmental Innovation	0.0096	-0.2351	-0.2072	-1.3006	0.1846	-0.0537	-0.1680	-0.2240	0.2217	-0.0746
Foreign Direct Investment	-0.0610	0.1770	-0.0260	-0.1059	0.0370	0.1011	0.5523	0.3112	-0.4088	0.0078
Industrial Share	0.0201	0.9598	0.0976	-3.7684	1.0853	-0.0338	0.4824	0.2844	0.2609	0.5542
Energy Demand	0.0293	-0.3070	-0.3398	-2.9517	0.8141	0.0924	-0.2290	-0.4109	-0.5688	-0.1717
**Variables**	**VOICE/IV**					**VOICE/WV**				
Policy Stringency	0.0488	-0.0338	-0.1726	0.0507	-0.1141	0.0834	-0.2161	-0.1907	0.0983	-0.2017
Environmental Innovation	-0.0066	-0.0625	-0.1835	0.2261	0.2335	0.0670	-0.1402	-0.1267	0.1641	-0.0688
Foreign Direct Investment	-0.0320	0.8514	-0.3038	-0.0834	0.1709	0.0492	1.0581	-0.3528	-0.0236	0.2542
Industrial Share	-0.0641	1.6452	0.0436	0.5695	0.8626	-0.0621	1.5601	-0.0064	0.1551	1.2210
Energy Demand	0.0923	-0.1921	-0.2673	-0.1682	0.2687	0.4770	-0.3227	-0.3206	-0.5171	-0.1852

Note:
IV: Infrastructure Vulnerability; WV: Water Vulnerability
CORR: Control of Corruption; GOVEFF: Governance Effectiveness; POLST: Political Stability; REG: Regulatory Quality; RULE: Rule of Law; VOICE: Voice and Accountability

Table S61: Country-wise Policy Simulation Outcomes (Changes in Marginal Impacts) for 7.3.1 under Climate Vulnerability (Ecosystem, Food, and Health) and Governance Quality, Related to Figure 7

	Brazil	China	India	Russia	S. Africa	Brazil	China	India	Russia	S. Africa	Brazil	China	India	Russia	S. Africa
**Variables**	**CORR/EV**					**CORR/FV**					**CORR/HELV**				
Policy Stringency	-0.24	-25.40	1.44	0.07	4.85	-0.32	-0.02	2.42	1.50	0.94	-0.12	1.89	0.41	2.33	-5.47
Environmental Innovation	-50.33	-9.02	1.14	3.03	1.46	-0.81	-2.98	0.45	-1.91	2.65	-1.27	-0.62	0.73	-0.35	-1.83
Foreign Direct Investment	-1.05	6.00	4.78	-3.18	1.83	-0.13	1.48	2.38	0.30	8.23	-1.12	0.50	-3.19	0.45	-2.37
Industrial Share	5.20	1.89	1.33	1.75	0.14	-0.25	0.04	-0.05	0.42	-0.05	-0.30	0.58	-0.71	0.74	-0.47
Energy Demand	-1.01	0.24	-28.38	-3.53	0.81	-2.73	1.40	38.17	-8.42	-0.36	-0.59	-1.56	-2.35	0.49	-0.70
**Variables**	**GOVEFF/EV**					**GOVEFF/FV**					**GOVEFF/HELV**				
Policy Stringency	0.25	-5.26	1.13	-5.41	-3.03	0.34	-0.56	1.14	-7.91	14.40	1.51	-0.11	0.41	-6.19	-13.21
Environmental Innovation	-4.14	-3.81	49.13	-0.71	1.68	-3.46	-3.77	1.16	0.00	-0.52	6.35	-1.39	0.50	-0.19	3.61
Foreign Direct Investment	-0.34	5.60	-3.79	-1.15	-2.02	0.41	0.95	-2.47	-1.10	1.34	-1.99	0.56	-2.31	0.51	-2.75
Industrial Share	1.53	0.81	1.12	-0.11	-0.09	1.74	0.03	-0.07	-0.18	-0.43	-0.22	0.17	-0.41	0.20	-0.03
Energy Demand	1.75	0.13	6.66	0.74	0.92	7.91	2.33	-12.88	1.72	0.66	-0.20	-0.99	-0.58	0.04	-2.46
**Variables**	**POLST/EV**					**POLST/FV**					**POLST/HELV**				
Policy Stringency	0.35	-13.25	1.20	-16.85	-3.57	0.77	-0.12	1.70	-44.29	-11.44	2.65	-1.21	0.70	-4.06	-80.96
Environmental Innovation	-1.75	-3.35	1.24	0.19	1.32	-2.14	-2.17	0.33	0.78	-0.74	1.64	-0.81	-0.58	-0.17	-88.11
Foreign Direct Investment	-0.31	8.86	0.68	-1.28	-6.70	0.56	2.18	3.82	-1.05	1.32	-2.15	0.63	-0.26	-0.14	-3.67
Industrial Share	2.24	1.72	1.09	-0.10	-0.28	2.94	0.04	-0.25	-0.20	0.33	-1.09	0.25	-0.98	0.10	0.09
Energy Demand	-0.25	-0.06	-5.36	0.42	-2.12	1.39	0.04	-7.88	0.88	-1.89	-0.59	-1.03	-0.54	0.06	19.32
**Variables**	**REG/EV**					**REG/FV**					**REG/HELV**				
Policy Stringency	-0.06	-13.22	2.71	-2.60	-7.52	0.81	-0.65	5.48	-38.03	0.07	0.91	-0.86	0.97	-3.48	-47.21
Environmental Innovation	-2.05	-3.35	2.80	2.10	-22.72	-1.30	-2.59	0.84	-0.29	31.47	0.86	-1.09	-1.06	-0.76	-3.07
Foreign Direct Investment	-1.03	7.90	-0.03	0.13	-3.85	1.40	1.63	0.21	-0.26	0.94	-1.97	0.07	-0.86	0.26	-3.57
Industrial Share	1.42	1.53	1.00	0.29	0.08	4.60	0.01	-0.15	0.04	-0.41	-0.25	0.33	-0.88	0.06	0.53
Energy Demand	-0.18	0.02	17.53	1.14	-1.48	0.22	0.35	-32.60	1.27	-0.04	-0.49	-1.07	-1.57	0.02	-9.56
**Variables**	**RULE/EV**					**RULE/FV**					**RULE/HELV**				
Policy Stringency	0.39	-4.78	1.12	-5.86	0.96	0.91	-0.67	1.57	-3.95	-4.27	2.06	-0.41	0.36	1.05	266.33
Environmental Innovation	-2.98	-3.51	4.47	-0.25	2.14	-5.34	-4.25	1.66	2.84	-0.44	0.30	-1.57	0.88	-0.43	4.99
Foreign Direct Investment	0.18	2.43	5.57	0.17	-4.35	1.94	-0.27	0.95	-0.23	1.08	0.31	-0.26	-1.94	0.48	-0.85
Industrial Share	2.13	0.29	1.51	0.30	0.22	2.13	-0.30	0.11	-0.28	-0.93	-1.96	-0.06	-0.56	-0.21	0.01
Energy Demand	1.28	-0.02	119.38	-0.05	1.76	1.54	3.47	-52.49	0.08	-1.14	-0.74	-1.19	-1.84	0.29	-9.55
**Variables**	**VOICE/EV**					**VOICE/FV**					**VOICE/HELV**				
Policy Stringency	0.19	-9.68	0.23	0.15	-8.24	0.90	-0.93	0.59	-1.09	-160.42	1.74	-2.26	0.11	-0.32	-29.73
Environmental Innovation	-2.45	-3.33	-53.86	1.72	0.99	-2.89	-2.46	1.88	0.12	-2.38	2.76	-0.77	1.59	3.22	1.30
Foreign Direct Investment	-0.50	8.56	0.60	-3.71	-5.22	-0.22	2.39	0.38	0.67	77.95	-1.12	0.66	-1.37	-1.37	-4.93
Industrial Share	1.57	1.81	1.68	-0.25	0.23	1.12	0.12	0.81	-0.17	0.02	-1.22	0.61	-0.73	0.08	-0.11
Energy Demand	2.28	-0.04	37.26	71.63	-1.98	3.09	0.33	-10.63	-2.44	-0.02	-0.73	-1.03	-1.14	13.29	-3.32

Note:
EV: Ecosystem Vulnerability; FV: Food Vulnerability; HELV: Health Vulnerability
CORR: Control of Corruption; GOVEFF: Governance Effectiveness; POLST: Political Stability; REG: Regulatory Quality; RULE: Rule of Law; VOICE: Voice and Accountability

Table S62: Country-wise Policy Simulation Outcomes (Changes in Marginal Impacts) for 7.3.1 under Climate Vulnerability (Infrastructure and Water) and Governance Quality, Related to Figure 7

	Brazil	China	India	Russia	S. Africa	Brazil	China	India	Russia	S. Africa
**Variables**			CORR/IV					CORR/WV		
Policy Stringency	-0.30	2.11	1.53	38.56	4.31	-0.47	-8.10	1.79	-0.30	1.72
Environmental Innovation	-1.78	-2.36	0.62	16.33	1.98	0.24	-3.26	0.29	-2.01	-469.50
Foreign Direct Investment	-1.31	1.31	-0.14	-1.17	1.31	-0.75	2.55	0.56	0.14	-3.22
Industrial Share	3.14	0.17	-0.96	-5.79	-0.28	5.59	0.53	-0.54	-0.05	-0.26
Energy Demand	-0.61	-0.09	-20.45	6.90	0.72	-2.83	0.20	-13.41	-0.47	-0.53
**Variables**			GOVEFF/IV					GOVEFF/WV		
Policy Stringency	0.24	0.65	0.85	-51.76	34.49	0.45	-5.14	1.49	-0.92	5.32
Environmental Innovation	-0.48	-2.12	0.32	7.28	-5.76	0.24	-2.96	0.25	-1.55	-0.07
Foreign Direct Investment	-2.45	1.29	-1.35	0.53	-0.32	-0.58	1.99	-1.13	-0.34	-3.17
Industrial Share	-0.38	-0.07	-0.12	13.58	-0.43	-1.55	0.16	-0.65	-1.20	-0.50
Energy Demand	0.52	-0.64	-3.25	10.82	0.24	1.35	-0.13	-4.51	-0.63	-0.37
**Variables**			POLST/IV					POLST/WV		
Policy Stringency	0.75	-2.09	0.89	185.98	-10.19	0.56	-10.23	0.89	-1.08	-55.03
Environmental Innovation	-1.01	-1.82	0.25	16.18	7.00	22.59	-2.43	0.04	-2.22	1.65
Foreign Direct Investment	-2.14	2.19	-0.35	0.15	-2.91	-1.74	3.05	0.23	-0.20	-2.62
Industrial Share	-0.63	0.25	-1.14	-8.32	-0.54	-0.99	0.65	-0.99	-1.17	-0.78
Energy Demand	-0.13	-0.39	-2.45	7.09	-0.25	2.22	-0.29	-4.16	-0.54	-2.02
**Variables**			REG/IV					REG/WV		
Policy Stringency	0.11	-1.01	4.51	58.81	-6.10	0.20	-9.05	1.73	-0.80	12.26
Environmental Innovation	-0.05	-1.73	0.02	9.02	1.42	0.85	-2.39	-0.27	-2.45	2.32
Foreign Direct Investment	-4.69	0.95	0.28	-0.99	-1.97	-1.84	2.08	1.28	-0.03	-1.98
Industrial Share	-1.76	0.13	-0.95	-2.56	-0.28	-2.92	0.66	-0.85	-0.65	0.16
Energy Demand	-1.55	-0.57	-3.44	2.25	-1.63	2.03	-0.29	-4.01	-0.53	-1.30
**Variables**			RULE/IV					RULE/WV		
Policy Stringency	0.28	0.06	0.91	258.70	-3.48	0.70	-3.74	1.31	-3.94	311.25
Environmental Innovation	-1.43	-2.93	0.64	9.34	-62.53	1.15	-2.81	0.47	-3.47	0.69
Foreign Direct Investment	-2.46	-0.34	-0.78	-0.74	-1.49	0.94	0.70	-3.35	0.07	-1.28
Industrial Share	-1.31	-0.22	-0.78	-10.53	-0.17	-0.48	-0.59	-0.48	-0.29	-0.42
Energy Demand	-0.39	-0.20	-6.73	2.07	0.04	0.97	-0.43	-6.21	-0.57	-1.56
**Variables**			VOICE/IV					VOICE/WV		
Policy Stringency	-0.02	-1.87	0.26	-0.46	-24.29	1.37	-8.30	0.37	0.11	-75.70
Environmental Innovation	-0.69	-1.50	1.48	-17.87	7.40	2.40	-2.28	-0.04	21.79	4.98
Foreign Direct Investment	-1.83	2.23	0.84	-0.35	-2.96	0.64	2.50	1.24	-0.70	-5.21
Industrial Share	0.24	0.34	-0.88	0.73	-0.40	-0.12	0.32	-1.01	-0.52	0.02
Energy Demand	1.94	-0.53	-4.40	0.31	-0.79	1.82	-0.30	-4.02	3.71	-1.27

Note:
IV: Infrastructure Vulnerability; WV: Water Vulnerability
CORR: Control of Corruption; GOVEFF: Governance Effectiveness; POLST: Political Stability; REG: Regulatory Quality; RULE: Rule of Law; VOICE: Voice and Accountability

Table S63: Long Run Coefficient Estimation using Panel-Corrected Standard Errors with Ecosystem Vulnerability for SDG 7.1.1 – With Control of Corruption, Governance Effectiveness, and Political Stability, Related to Figure 3

Variables	Model 1	Model 2	Model 1	Model 2	Model 1	Model 2
	Control of Corruption		Governance Effectiveness		Political Stability	
Electricity Generation	0.0702*	0.6449	0.1179***	0.1356	0.0912**	0.6435**
	(0.0416)	(0.4193)	(0.0395)	(0.2816)	(0.0408)	(0.3273)
Financial Access	0.0837*	0.2725	0.1021**	-2.0759**	0.0741	-1.7915
	(0.0497)	(1.3086)	(0.0481)	(0.9125)	(0.0513)	(1.2083)
Rural Population Growth	-0.0834***	0.8002***	-0.0859***	0.8146***	-0.0682***	0.7091***
	(0.0098)	(0.1628)	(0.0098)	(0.1319)	(0.0095)	(0.1911)
Income Inequality	0.6681*	-12.3693***	0.7911**	-5.5532**	0.4806	-6.4243
	(0.3799)	(3.9637)	(0.3883)	(2.6345)	(0.3790)	(4.5471)
Health Expenditure	0.0038*	0.0002	0.0056***	-0.0041	0.0047**	-0.0313*
	(0.0021)	(0.0209)	(0.0020)	(0.0145)	(0.0019)	(0.0186)
Ecosystem Vulnerability	-0.5573	-5.4496	-0.2741	-8.4224	-0.8697**	-2.2692
	(0.3563)	(8.2565)	(0.4087)	(7.3954)	(0.3606)	(7.4938)
Quality of Governance	-0.0787***	1.8185	-0.0606*	-0.3507	0.0086	1.5439
	(0.0302)	(1.1886)	(0.0319)	(1.7825)	(0.0199)	(1.4593)
Electricity Generation * Ecosystem Vulnerability	-	-1.6039*	-	-0.3467	-	-1.6697**
	-	(0.8824)	-	(0.6075)	-	(0.7905)
Financial Access * Ecosystem Vulnerability	-	-0.2517	-	4.9796**	-	3.8479
	-	(2.7432)	-	(1.9408)	-	(2.6186)
Rural Population Growth * Ecosystem Vulnerability	-	-1.7769***	-	-1.8317***	-	-1.5747***
	-	(0.3611)	-	(0.2885)	-	(0.4273)
Income Inequality * Ecosystem Vulnerability	-	25.3849***	-	11.7474**	-	12.9624
	-	(8.0237)	-	(5.2349)	-	(9.0960)
Health Expenditure * Ecosystem Vulnerability	-	0.0018	-	0.0158	-	0.0635
	-	(0.0432)	-	(0.0306)	-	(0.0414)
Electricity Generation * Quality of Governance	-	0.7900	-	1.2271***	-	0.4127
	-	(0.5406)	-	(0.4301)	-	(0.2773)
Financial Access * Quality of Governance	-	1.2210	-	0.4069	-	0.9874
	-	(2.0006)	-	(2.4600)	-	(1.6178)
Rural Population Growth * Quality of Governance	-	0.2198	-	0.9082	-	0.3112
	-	(0.2954)	-	(0.6518)	-	(0.2808)
Income Inequality * Quality of Governance	-	-12.5692*	-	-16.6427***	-	-3.4403
	-	(6.9520)	-	(5.9874)	-	(4.5807)
Health Expenditure * Quality of Governance	-	0.0023	-	0.0377	-	-0.0473*
	-	(0.0204)	-	(0.0375)	-	(0.0272)
Electricity Generation * Quality of Governance * Ecosystem Vulnerability	-	-2.0499*	-	-2.8316***	-	-1.1551*
	-	(1.1862)	-	(1.0022)	-	(0.6650)
Financial Access * Quality of Governance * Ecosystem Vulnerability	-	-2.5814	-	-1.0142	-	-2.5861
	-	(4.4000)	-	(5.3411)	-	(3.6643)

Rural Population Growth * Quality of Governance * Ecosystem Vulnerability	-	-0.4359	-	-1.8567	-	-0.6110
	-	(0.6537)	-	(1.3350)	-	(0.6027)
Income Inequality * Quality of Governance * Ecosystem Vulnerability	-	24.9872	-	38.7981***	-	6.4301
	-	(15.5395)	-	(13.1829)	-	(9.1325)
Health Expenditure * Quality of Governance * Ecosystem Vulnerability	-	-0.0001	-	-0.0685	-	0.0890
	-	(0.0412)	-	(0.0745)	-	(0.0587)
Constant	3.5469***	8.1506*	2.9048***	8.5130**	3.6599***	6.9763*
	(0.6713)	(4.3179)	(0.6997)	(3.7227)	(0.6634)	(3.7784)
Wald-chi2	157.8	555.5	176.3	943.0	111.2	433.5
Observations	115	115	115	115	115	115
Heteroskedasticity controlled	Yes	Yes	Yes	Yes	Yes	Yes
Autocorrelation controlled	Yes	Yes	Yes	Yes	Yes	Yes
Cross-sectional correlation controlled	Yes	Yes	Yes	Yes	Yes	Yes
Marginal Impact						
Electricity Generation	0.0702*	-0.0720	0.1179***	-0.0518	0.0912**	-0.0774
	(0.0416)	(0.0523)	(0.0395)	(0.0538)	(0.0408)	(0.0508)
Financial Access	0.0837*	0.1480***	0.1021**	0.3324***	0.0741	0.2381***
	(0.0497)	(0.0552)	(0.0481)	(0.0691)	(0.0513)	(0.0670)
Rural Population Growth	-0.0834***	-0.0667***	-0.0859***	-0.0874***	-0.0682***	-0.0594***
	(0.0098)	(0.0133)	(0.0098)	(0.0190)	(0.0095)	(0.0137)
Income Inequality	0.6681*	0.2276	0.7911**	0.4059	0.4806	0.0054
	(0.3799)	(0.3088)	(0.3883)	(0.2999)	(0.3790)	(0.2919)
Health Expenditure	0.0038*	0.0003	0.0056***	0.0030*	0.0047**	0.0013
	(0.0021)	(0.0016)	(0.0020)	(0.0015)	(0.0019)	(0.0014)

Note:
Standard errors in parentheses ()
*** p<0.01, ** p<0.05, * p<0.1 by Z-test

Table S64: Long Run Coefficient Estimation using Panel-Corrected Standard Errors with Ecosystem Vulnerability for SDG 7.1.1 – With Regulatory Quality, Rule of Law, and Voice and Accountability, Related to Figure 3

Variables	Model 1	Model 2	Model 1	Model 2	Model 1	Model 2
	Regulatory Quality		Rule of Law		Voice and Accountability	
Electricity Generation	0.0696*	0.0586	0.0311	1.4085***	-0.0166	0.3871
	(0.0374)	(0.3318)	(0.0380)	(0.4598)	(0.0399)	(0.2734)
Financial Access	0.0411	-0.6429	0.2110***	-0.1965	0.0936*	-2.4403**
	(0.0578)	(0.8512)	(0.0553)	(1.5867)	(0.0569)	(1.0891)
Rural Population Growth	-0.0682***	0.5248***	-0.0741***	0.3717	-0.0165*	0.2195
	(0.0089)	(0.1513)	(0.0089)	(0.2286)	(0.0090)	(0.1878)
Income Inequality	0.5042	-8.6543***	0.7002**	-11.8719***	1.0575***	-7.9374***
	(0.3682)	(3.2412)	(0.3381)	(3.3951)	(0.3442)	(2.4359)
Health Expenditure	0.0031*	-0.0058	0.0023	0.0388**	0.0009	0.0008
	(0.0019)	(0.0176)	(0.0018)	(0.0189)	(0.0018)	(0.0109)
Ecosystem Vulnerability	-0.9752***	-12.3229	0.4558	9.4808	-1.1371***	-6.3755
	(0.3758)	(8.3957)	(0.4491)	(8.7543)	(0.3785)	(5.4941)
Quality of Governance	-0.0879***	0.8925	-0.1865***	-2.1026	-0.1376***	-0.1322
	(0.0329)	(2.1209)	(0.0429)	(1.2811)	(0.0267)	(0.4733)
Electricity Generation * Ecosystem Vulnerability	-	-0.2813	-	-2.9814***	-	-0.7716
	-	(0.7471)	-	(0.9369)	-	(0.5724)
Financial Access * Ecosystem Vulnerability	-	1.5546	-	1.6580	-	6.0974**
	-	(1.8529)	-	(3.3351)	-	(2.4092)
Rural Population Growth * Ecosystem Vulnerability	-	-1.1811***	-	-0.8962*	-	-0.5133
	-	(0.3305)	-	(0.4831)	-	(0.4163)
Income Inequality * Ecosystem Vulnerability	-	18.0713***	-	25.3429***	-	16.9656***
	-	(6.6104)	-	(6.6519)	-	(5.3089)
Health Expenditure * Ecosystem Vulnerability	-	0.0157	-	-0.0760**	-	0.0033
	-	(0.0378)	-	(0.0382)	-	(0.0234)
Electricity Generation * Quality of Governance	-	0.8913*	-	1.8738***	-	0.1259
	-	(0.5389)	-	(0.5152)	-	(0.2174)
Financial Access * Quality of Governance	-	-0.1819	-	-1.1982	-	-1.2090
	-	(2.6804)	-	(2.5187)	-	(1.0600)
Rural Population Growth * Quality of Governance	-	0.3261	-	-0.4891	-	0.6106***
	-	(0.4238)	-	(0.4020)	-	(0.1501)
Income Inequality * Quality of Governance	-	-10.4576	-	-24.9299***	-	0.9162
	-	(8.4319)	-	(6.5238)	-	(2.8951)
Health Expenditure * Quality of Governance	-	-0.0373	-	0.0395*	-	0.0046
	-	(0.0282)	-	(0.0209)	-	(0.0078)
Electricity Generation * Quality of Governance * Ecosystem Vulnerability	-	-2.0591	-	-3.8002***	-	-0.1874
	-	(1.4023)	-	(1.1111)	-	(0.4726)
Financial Access * Quality of Governance * Ecosystem Vulnerability	-	0.8983	-	4.4980	-	2.9423
	-	(5.7582)	-	(5.5567)	-	(2.2788)
Rural Population Growth * Quality of Governance * Ecosystem Vulnerability	-	-0.6455	-	0.9911	-	-1.3276***

	-	(0.9111)	-	(0.8319)	-	(0.3133)
Income Inequality * Quality of Governance * Ecosystem Vulnerability	-	21.0854	-	55.1241***	-	-3.2711
	-	(17.4465)	-	(14.0424)	-	(6.0625)
Health Expenditure * Quality of Governance * Ecosystem Vulnerability	-	0.0749	-	-0.0641	-	-0.0050
	-	(0.0603)	-	(0.0415)	-	(0.0165)
Constant	3.9236***	10.9485***	3.3234***	-0.4100	4.4122***	7.1342***
	(0.6405)	(4.0719)	(0.6042)	(4.5549)	(0.5829)	(2.7311)
Wald-chi2	121.6	379.5	161.3	605.5	109.1	983.3
Observations	115	115	115	115	115	115
Heteroskedasticity controlled	Yes	Yes	Yes	Yes	Yes	Yes
Autocorrelation controlled	Yes	Yes	Yes	Yes	Yes	Yes
Cross-sectional correlation controlled	Yes	Yes	Yes	Yes	Yes	Yes
Marginal Impact						
Electricity Generation	0.0696*	-0.0631	0.0311	-0.0837*	-0.0166	0.0053
	(0.0374)	(0.0532)	(0.0380)	(0.0503)	(0.0399)	(0.0421)
Financial Access	0.0411	0.0814	0.2110***	0.3680***	0.0936*	0.4786***
	(0.0578)	(0.0575)	(0.0553)	(0.0745)	(0.0569)	(0.0958)
Rural Population Growth	-0.0682***	-0.0486***	-0.0741***	-0.0508***	-0.0165*	-0.0256
	(0.0089)	(0.0123)	(0.0089)	(0.0160)	(0.0090)	(0.0172)
Income Inequality	0.5042	0.1062	0.7002**	0.4401	1.0575***	0.4198
	(0.3682)	(0.2942)	(0.3381)	(0.3325)	(0.3442)	(0.3713)
Health Expenditure	0.0031*	0.0018	0.0023	-0.0011	0.0009	0.0019
	(0.0019)	(0.0015)	(0.0018)	(0.0016)	(0.0018)	(0.0013)

Note:
Standard errors in parentheses ()
*** p<0.01, ** p<0.05, * p<0.1 by Z-test

Table S65: Long Run Coefficient Estimation using Panel-Corrected Standard Errors with Food Vulnerability for SDG 7.1.1 – With Control of Corruption, Governance Effectiveness, and Political Stability, Related to Figure 3

Variables	Model 1	Model 2	Model 1	Model 2	Model 1	Model 2
	Control of Corruption		Governance Effectiveness		Political Stability	
Electricity Generation	0.0635	0.6002**	0.1094***	0.1644	0.0771**	0.8476***
	(0.0407)	(0.2627)	(0.0371)	(0.2627)	(0.0385)	(0.1735)
Financial Access	0.0901**	0.2421	0.0988**	-2.2395***	0.1012**	-0.7485
	(0.0425)	(0.9455)	(0.0424)	(0.7603)	(0.0436)	(0.7038)
Rural Population Growth	-0.0663***	0.2693	-0.0706***	0.3511**	-0.0496***	-0.4835*
	(0.0104)	(0.1836)	(0.0125)	(0.1573)	(0.0092)	(0.2867)
Income Inequality	0.8832**	-10.9887***	0.9398**	-3.1160*	0.7650**	-3.5013
	(0.3574)	(3.0140)	(0.3681)	(1.6005)	(0.3502)	(2.2791)
Health Expenditure	0.0046**	0.0175	0.0063***	-0.0030	0.0055***	0.0458***
	(0.0021)	(0.0109)	(0.0020)	(0.0074)	(0.0019)	(0.0158)
Food Vulnerability	-0.5774**	-3.3799	-0.4378	-4.1976	-0.7559***	15.0017***
	(0.2732)	(5.1210)	(0.3145)	(5.8775)	(0.2884)	(5.4223)
Quality of Governance	-0.0742***	0.2710	-0.0409	-1.5114	-0.0006	0.6062
	(0.0283)	(0.8547)	(0.0310)	(1.0788)	(0.0196)	(0.9169)
Electricity Generation * Food Vulnerability	-	-1.5694**	-	-0.3461	-	-2.4622***
	-	(0.6667)	-	(0.6439)	-	(0.5076)
Financial Access * Food Vulnerability	-	0.2187	-	6.0661***	-	1.9706
	-	(2.3350)	-	(1.9231)	-	(1.8549)
Rural Population Growth * Food Vulnerability	-	-0.6528	-	-0.8936**	-	1.1753*
	-	(0.4468)	-	(0.3903)	-	(0.7024)
Income Inequality * Food Vulnerability	-	25.6966***	-	6.5305*	-	8.2564
	-	(7.2906)	-	(3.9668)	-	(5.4767)
Health Expenditure * Food Vulnerability	-	-0.0327	-	0.0149	-	-0.1111***
	-	(0.0255)	-	(0.0174)	-	(0.0386)
Electricity Generation * Quality of Governance	-	0.7843**	-	-0.0371	-	-0.2133
	-	(0.3120)	-	(0.3081)	-	(0.1787)
Financial Access * Quality of Governance	-	1.5478	-	-1.4533	-	3.1748***
	-	(0.9490)	-	(1.2156)	-	(0.9954)
Rural Population Growth * Quality of Governance	-	-0.4797	-	-0.1093	-	-1.5322***
	-	(0.3821)	-	(0.4813)	-	(0.4432)
Income Inequality * Quality of Governance	-	-14.0804***	-	0.7422	-	-2.4019
	-	(4.7144)	-	(4.7787)	-	(2.4997)
Health Expenditure * Quality of Governance	-	0.0505***	-	0.0299	-	0.0580***
	-	(0.0175)	-	(0.0227)	-	(0.0178)
Electricity Generation * Quality of Governance * Food Vulnerability	-	-2.0746**	-	0.4506	-	0.3147
	-	(0.8321)	-	(0.8821)	-	(0.5329)
Financial Access * Quality of Governance * Food Vulnerability	-	-3.4764	-	4.0845	-	-8.5644***
	-	(2.5295)	-	(3.3113)	-	(2.7116)
Rural Population Growth * Quality of Governance * Food Vulnerability	-	1.3493	-	0.4109	-	3.9445***

	-	(0.9884)	-	(1.2304)	-	(1.1027)
Income Inequality * Quality of Governance * Food Vulnerability	-	34.1633***	-	-2.3819	-	6.5047
	-	(11.8066)	-	(12.0479)	-	(5.8280)
Health Expenditure * Quality of Governance * Food Vulnerability	-	-0.1072**	-	-0.0432	-	-0.1415***
	-	(0.0418)	-	(0.0515)	-	(0.0408)
Constant	3.4292***	6.2774***	2.9259***	6.1834**	3.4737***	-0.3124
	(0.6006)	(2.2822)	(0.5877)	(2.4942)	(0.5752)	(2.1287)
Wald-chi2	199.6	747.1	220.2	840.0	144.9	1053
Observations	115	115	115	115	115	115
Heteroskedasticity controlled	Yes	Yes	Yes	Yes	Yes	Yes
Autocorrelation controlled	Yes	Yes	Yes	Yes	Yes	Yes
Cross-sectional correlation controlled	Yes	Yes	Yes	Yes	Yes	Yes
Marginal Impact						
Electricity Generation	0.0635	-0.0633	0.1094***	0.0119	0.0771**	-0.2090***
	(0.0407)	(0.0447)	(0.0371)	(0.0435)	(0.0385)	(0.0429)
Financial Access	0.0901**	0.3128***	0.0988**	0.4756***	0.1012**	0.6104***
	(0.0425)	(0.1018)	(0.0424)	(0.1029)	(0.0436)	(0.0888)
Rural Population Growth	-0.0663***	-0.0526***	-0.0706***	-0.0437**	-0.0496***	-0.1458***
	(0.0104)	(0.0159)	(0.0125)	(0.0172)	(0.0092)	(0.0258)
Income Inequality	0.8832**	0.2801	0.9398**	-0.2296	0.7650**	-0.2098
	(0.3574)	(0.2411)	(0.3681)	(0.2643)	(0.3502)	(0.1955)
Health Expenditure	0.0046**	0.0013	0.0063***	0.0032***	0.0055***	0.0014
	(0.0021)	(0.0012)	(0.0020)	(0.0011)	(0.0019)	(0.0009)

Note:
Standard errors in parentheses ()
*** p<0.01, ** p<0.05, * p<0.1 by Z-test

Table S66: Long Run Coefficient Estimation using Panel-Corrected Standard Errors with Food Vulnerability for SDG 7.1.1 – With Regulatory Quality, Rule of Law, and Voice and Accountability, Related to Figure 3

Variables	Model 1	Model 2	Model 1	Model 2	Model 1	Model 2
	Regulatory Quality		Rule of Law		Voice and Accountability	
Electricity Generation	0.0640*	0.6797**	0.0269	0.4689*	0.0040	0.4442*
	(0.0356)	(0.2943)	(0.0380)	(0.2616)	(0.0380)	(0.2528)
Financial Access	0.0861*	-2.1838***	0.1495***	-1.9342***	0.1620***	-0.9847*
	(0.0461)	(0.7741)	(0.0456)	(0.7440)	(0.0527)	(0.5838)
Rural Population Growth	-0.0484***	0.4243**	-0.0695***	0.5902***	-0.0127	0.2629
	(0.0091)	(0.1697)	(0.0125)	(0.1824)	(0.0091)	(0.1810)
Income Inequality	0.7635**	-2.4549	0.6487**	-4.6783**	1.0870***	-5.1717***
	(0.3442)	(2.8505)	(0.3251)	(2.1557)	(0.3354)	(1.5191)
Health Expenditure	0.0042**	0.0104	0.0026	0.0048	0.0017	-0.0014
	(0.0018)	(0.0119)	(0.0020)	(0.0101)	(0.0018)	(0.0090)
Food Vulnerability	-0.7242**	6.6525	-0.1288	0.6375	-0.4614	-1.0663
	(0.2899)	(7.0035)	(0.3545)	(5.1582)	(0.3451)	(5.8129)
Quality of Governance	-0.0718**	-2.2300	-0.1566***	-0.1486	-0.1130***	-0.6308
	(0.0305)	(2.1179)	(0.0445)	(1.2373)	(0.0293)	(0.5339)
Electricity Generation * Food Vulnerability	-	-1.6298**	-	-1.1371*	-	-1.0319*
	-	(0.8295)	-	(0.5951)	-	(0.6113)
Financial Access * Food Vulnerability	-	6.0253***	-	5.9394***	-	3.2948**
	-	(1.9507)	-	(1.8176)	-	(1.5023)
Rural Population Growth * Food Vulnerability	-	-1.0831***	-	-1.4273***	-	-0.6319
	-	(0.4143)	-	(0.4299)	-	(0.4451)
Income Inequality * Food Vulnerability	-	6.0193	-	9.8261**	-	12.0887***
	-	(6.6700)	-	(4.7748)	-	(3.6550)
Health Expenditure * Food Vulnerability	-	-0.0151	-	-0.0029	-	0.0048
	-	(0.0290)	-	(0.0209)	-	(0.0211)
Electricity Generation * Quality of Governance	-	-0.3335	-	0.9453***	-	0.3142*
	-	(0.3600)	-	(0.2972)	-	(0.1624)
Financial Access * Quality of Governance	-	2.0521	-	-3.1449**	-	-0.4622
	-	(1.4658)	-	(1.3411)	-	(0.6929)
Rural Population Growth * Quality of Governance	-	-0.8584	-	0.2900	-	0.5856***
	-	(0.6139)	-	(0.3083)	-	(0.1479)
Income Inequality * Quality of Governance	-	2.6081	-	-12.1653***	-	-1.1679
	-	(6.9267)	-	(3.2788)	-	(2.0296)
Health Expenditure * Quality of Governance	-	0.0608**	-	0.0166	-	-0.0171**
	-	(0.0258)	-	(0.0152)	-	(0.0081)
Electricity Generation * Quality of Governance * Food Vulnerability	-	1.2598	-	-2.3692***	-	-0.4717
	-	(1.1947)	-	(0.7470)	-	(0.4108)
Financial Access * Quality of Governance * Food Vulnerability	-	-4.9705	-	9.3847***	-	1.5375
	-	(3.8878)	-	(3.4767)	-	(1.7464)
Rural Population Growth * Quality of Governance * Food Vulnerability	-	2.3172	-	-0.5670	-	-1.4279***

	-	(1.5447)	-	(0.7939)	-	(0.3660)
Income Inequality * Quality of Governance * Food Vulnerability	-	-5.0385	-	29.2753***	-	0.8540
	-	(15.6890)	-	(8.2870)	-	(4.8930)
Health Expenditure * Quality of Governance * Food Vulnerability	-	-0.1173*	-	-0.0227	-	0.0452**
	-	(0.0630)	-	(0.0353)	-	(0.0202)
Constant	3.6093***	1.4669	3.6928***	4.3854*	3.8298***	4.8303*
	(0.5523)	(2.8384)	(0.5350)	(2.5948)	(0.5096)	(2.5492)
Wald-chi2	140.8	399.8	160.5	715.4	84.50	974.7
Observations	115	115	115	115	115	115
Heteroskedasticity controlled	Yes	Yes	Yes	Yes	Yes	Yes
Autocorrelation controlled	Yes	Yes	Yes	Yes	Yes	Yes
Cross-sectional correlation controlled	Yes	Yes	Yes	Yes	Yes	Yes
Marginal Impact						
Electricity Generation	0.0640*	-0.0704	0.0269	-0.0531	0.0040	-0.0522
	(0.0356)	(0.0734)	(0.0380)	(0.0406)	(0.0380)	(0.0552)
Financial Access	0.0861*	0.5137***	0.1495***	0.5971***	0.1620***	0.4883***
	(0.0461)	(0.1050)	(0.0456)	(0.1011)	(0.0527)	(0.1161)
Rural Population Growth	-0.0484***	-0.0782***	-0.0695***	-0.0658***	-0.0127	-0.0593**
	(0.0091)	(0.0221)	(0.0125)	(0.0187)	(0.0091)	(0.0232)
Income Inequality	0.7635**	0.1813	0.6487**	0.0358	1.0870***	0.3860
	(0.3442)	(0.2384)	(0.3251)	(0.2494)	(0.3354)	(0.2840)
Health Expenditure	0.0042**	0.0029*	0.0026	0.0012	0.0017	0.0017
	(0.0018)	(0.0017)	(0.0020)	(0.0013)	(0.0018)	(0.0013)
Note:<br>Standard errors in parentheses ()<br>*** p<0.01, ** p<0.05, * p<0.1 by Z-test						

Table S67: Long Run Coefficient Estimation using Panel-Corrected Standard Errors with Health Vulnerability for SDG 7.1.1 – With Control of Corruption, Governance Effectiveness, and Political Stability, Related to Figure 3

Variables	Model 1	Model 2	Model 1	Model 2	Model 1	Model 2
	Control of Corruption		Governance Effectiveness		Political Stability	
Electricity Generation	0.0327	0.1355	0.0552	-0.3187**	0.0398	0.1990
	(0.0395)	(0.1650)	(0.0366)	(0.1468)	(0.0377)	(0.1266)
Financial Access	0.0654	1.1576***	0.0641	-0.0937	0.0685	1.2100***
	(0.0438)	(0.4281)	(0.0447)	(0.3241)	(0.0448)	(0.4125)
Rural Population Growth	-0.0654***	0.2042**	-0.0611***	0.1516**	-0.0511***	-0.1588*
	(0.0094)	(0.1022)	(0.0108)	(0.0733)	(0.0086)	(0.0962)
Income Inequality	0.8863**	-7.1092***	0.9321**	-3.8472***	0.7873**	0.3595
	(0.3578)	(2.3707)	(0.3721)	(1.0758)	(0.3497)	(1.9713)
Health Expenditure	0.0051**	0.0072	0.0065***	-0.0021	0.0060***	0.0439***
	(0.0021)	(0.0085)	(0.0020)	(0.0064)	(0.0019)	(0.0100)
Health Vulnerability	-0.7529***	-4.0800	-0.8238***	-10.6976***	-0.9043***	9.6517**
	(0.2413)	(4.0861)	(0.2889)	(3.6493)	(0.2347)	(3.7549)
Quality of Governance	-0.0565*	1.5208	-0.0136	0.8696	0.0120	1.4367
	(0.0298)	(0.9824)	(0.0319)	(1.2787)	(0.0197)	(1.2099)
Electricity Generation * Health Vulnerability	-	-0.5824	-	0.6769*	-	-0.9348**
	-	(0.3802)	-	(0.3507)	-	(0.3847)
Financial Access * Health Vulnerability	-	-2.1570**	-	0.5161	-	-2.7396***
	-	(0.9871)	-	(0.7711)	-	(1.0518)
Rural Population Growth * Health Vulnerability	-	-0.4895**	-	-0.3516**	-	0.3093
	-	(0.2353)	-	(0.1697)	-	(0.2165)
Income Inequality * Health Vulnerability	-	14.9191***	-	7.5335***	-	0.8340
	-	(4.9003)	-	(2.3281)	-	(4.1167)
Health Expenditure * Health Vulnerability	-	-0.0164	-	0.0081	-	-0.0920***
	-	(0.0165)	-	(0.0144)	-	(0.0219)
Electricity Generation * Quality of Governance	-	0.2808	-	0.3043	-	-0.5040***
	-	(0.2804)	-	(0.2609)	-	(0.1587)
Financial Access * Quality of Governance	-	2.5573***	-	-0.4072	-	3.0763***
	-	(0.8063)	-	(0.6330)	-	(0.6317)
Rural Population Growth * Quality of Governance	-	-0.1933	-	0.0022	-	-0.6678***
	-	(0.1780)	-	(0.2031)	-	(0.1893)
Income Inequality * Quality of Governance	-	-8.0378**	-	-6.8064**	-	1.0820
	-	(3.5775)	-	(3.4515)	-	(2.2706)
Health Expenditure * Quality of Governance	-	0.0206**	-	0.0237	-	0.0428***
	-	(0.0099)	-	(0.0165)	-	(0.0129)
Electricity Generation * Quality of Governance * Health Vulnerability	-	-1.0911	-	-0.9597	-	0.6256
	-	(0.7013)	-	(0.7128)	-	(0.4340)
Financial Access * Quality of Governance * Health Vulnerability	-	-6.1505***	-	1.6280	-	-8.0508***
	-	(2.1610)	-	(1.5579)	-	(1.7248)
Rural Population Growth * Quality of Governance * Health Vulnerability	-	0.5033	-	-0.0689	-	1.7495***

	-	(0.4683)	-	(0.5728)	-	(0.4459)
Income Inequality * Quality of Governance * Health Vulnerability	-	17.9966**	-	14.2094*	-	-0.5758
	-	(8.3095)	-	(8.1668)	-	(4.6182)
Health Expenditure * Quality of Governance * Health Vulnerability	-	-0.0424**	-	-0.0394	-	-0.0943***
	-	(0.0198)	-	(0.0294)	-	(0.0261)
Constant	3.7639***	7.5345***	3.5636***	9.6801***	3.8412***	1.3543
	(0.5713)	(2.1424)	(0.5521)	(1.6285)	(0.5489)	(1.7473)
Wald-chi2	171.6	803.3	174.6	758.9	126.7	824.7
Observations	115	115	115	115	115	115
Heteroskedasticity controlled	Yes	Yes	Yes	Yes	Yes	Yes
Autocorrelation controlled	Yes	Yes	Yes	Yes	Yes	Yes
Cross-sectional correlation controlled	Yes	Yes	Yes	Yes	Yes	Yes
Marginal Impact						
Electricity Generation	0.0327	-0.0673*	0.0552	-0.0091	0.0398	-0.1276***
	(0.0395)	(0.0407)	(0.0366)	(0.0403)	(0.0377)	(0.0460)
Financial Access	0.0654	0.1973***	0.0641	0.1552***	0.0685	0.4384***
	(0.0438)	(0.0709)	(0.0447)	(0.0561)	(0.0448)	(0.0710)
Rural Population Growth	-0.0654***	-0.0330**	-0.0611***	-0.0123	-0.0511***	-0.1247***
	(0.0094)	(0.0137)	(0.0108)	(0.0126)	(0.0086)	(0.0228)
Income Inequality	0.8863**	-0.0963	0.9321**	-0.2292	0.7873**	0.3026
	(0.3578)	(0.2081)	(0.3721)	(0.1900)	(0.3497)	(0.2092)
Health Expenditure	0.0051**	-0.0012	0.0065***	0.0013	0.0060***	0.0032**
	(0.0021)	(0.0015)	(0.0020)	(0.0015)	(0.0019)	(0.0013)

Note:
Standard errors in parentheses ()
*** p<0.01, ** p<0.05, * p<0.1 by Z-test

Table S68: Long Run Coefficient Estimation using Panel-Corrected Standard Errors with Health Vulnerability for SDG 7.1.1 – With Regulatory Quality, Rule of Law, and Voice and Accountability, Related to Figure 3

Variables	Model 1	Model 2	Model 1	Model 2	Model 1	Model 2
	Regulatory Quality		Rule of Law		Voice and Accountability	
Electricity Generation	0.0351	0.1066	0.0088	0.1240	-0.0363	-0.1688
	(0.0357)	(0.1503)	(0.0371)	(0.1964)	(0.0377)	(0.1760)
Financial Access	0.0682	0.4302	0.1187***	-0.6326	0.1384***	0.4136
	(0.0457)	(0.3265)	(0.0448)	(0.5253)	(0.0486)	(0.3714)
Rural Population Growth	-0.0542***	0.1918**	-0.0656***	0.1002	-0.0122	0.0280
	(0.0084)	(0.0944)	(0.0093)	(0.1120)	(0.0093)	(0.1304)
Income Inequality	0.8001**	-3.1176*	0.7021**	-4.0681**	1.2329***	-3.7750***
	(0.3461)	(1.6553)	(0.3325)	(1.6808)	(0.3350)	(1.2200)
Health Expenditure	0.0049***	0.0111	0.0031	0.0104	0.0022	-0.0063
	(0.0018)	(0.0085)	(0.0020)	(0.0101)	(0.0018)	(0.0066)
Health Vulnerability	-0.8110***	-1.5057	-0.3881	-2.0423	-0.6891***	-9.7903***
	(0.2392)	(4.3159)	(0.2647)	(4.0925)	(0.2274)	(3.7142)
Quality of Governance	-0.0515	-0.6337	-0.1398***	-1.0280	-0.1203***	0.1996
	(0.0325)	(2.2365)	(0.0386)	(1.2113)	(0.0267)	(0.4482)
Electricity Generation * Health Vulnerability	-	-0.3748	-	-0.4692	-	0.4132
	-	(0.3980)	-	(0.4215)	-	(0.4107)
Financial Access * Health Vulnerability	-	-0.5964	-	2.4073*	-	-0.4263
	-	(0.8177)	-	(1.2314)	-	(0.9060)
Rural Population Growth * Health Vulnerability	-	-0.4808**	-	-0.3067	-	0.0045
	-	(0.2232)	-	(0.2621)	-	(0.2999)
Income Inequality * Health Vulnerability	-	7.5226**	-	8.9401***	-	9.9708***
	-	(3.5403)	-	(3.1607)	-	(2.5430)
Health Expenditure * Health Vulnerability	-	-0.0186	-	-0.0197	-	0.0189
	-	(0.0199)	-	(0.0183)	-	(0.0144)
Electricity Generation * Quality of Governance	-	-0.0389	-	0.8837***	-	-0.1414
	-	(0.2520)	-	(0.2672)	-	(0.1231)
Financial Access * Quality of Governance	-	3.3234***	-	-2.1092*	-	1.1952***
	-	(0.9008)	-	(1.0908)	-	(0.3694)
Rural Population Growth * Quality of Governance	-	-0.0512	-	0.1543	-	0.2482***
	-	(0.2859)	-	(0.1677)	-	(0.0858)
Income Inequality * Quality of Governance	-	-1.2145	-	-9.9872***	-	1.2054
	-	(4.8071)	-	(2.6536)	-	(1.6645)
Health Expenditure * Quality of Governance	-	0.0157	-	0.0027	-	0.0041
	-	(0.0158)	-	(0.0136)	-	(0.0049)
Electricity Generation * Quality of Governance * Health Vulnerability	-	0.1582	-	-2.0420***	-	0.2526
	-	(0.8270)	-	(0.5435)	-	(0.2792)
Financial Access * Quality of Governance * Health Vulnerability	-	-7.8050***	-	6.4134**	-	-2.3201***
	-	(2.3749)	-	(2.8337)	-	(0.8304)
Rural Population Growth * Quality of Governance * Health Vulnerability	-	0.2687	-	-0.4001	-	-0.6960***
	-	(0.6527)	-	(0.4272)	-	(0.2032)

Income Inequality * Quality of Governance * Health Vulnerability	-	3.4001	-	24.9551***	-	-3.6590
	-	(10.3465)	-	(6.4272)	-	(3.7415)
Health Expenditure * Quality of Governance * Health Vulnerability	-	-0.0261	-	0.0055	-	-0.0085
	-	(0.0367)	-	(0.0275)	-	(0.0094)
Constant	3.8515***	5.3740***	3.9264***	5.9176***	4.1653***	8.2469***
	(0.5381)	(1.9747)	(0.5292)	(2.2591)	(0.4780)	(1.7429)
Wald-chi2	130.6	673.8	158.9	864.1	100.0	769.7
Observations	115	115	115	115	115	115
Heteroskedasticity controlled	Yes	Yes	Yes	Yes	Yes	Yes
Autocorrelation controlled	Yes	Yes	Yes	Yes	Yes	Yes
Cross-sectional correlation controlled	Yes	Yes	Yes	Yes	Yes	Yes
Marginal Impact						
Electricity Generation	0.0351	-0.0722	0.0088	-0.1264**	-0.0363	0.0379
	(0.0357)	(0.0541)	(0.0371)	(0.0506)	(0.0377)	(0.0643)
Financial Access	0.0682	0.2034***	0.1187***	0.3963***	0.1384***	0.1044
	(0.0457)	(0.0724)	(0.0448)	(0.0910)	(0.0486)	(0.0928)
Rural Population Growth	-0.0542***	-0.0409**	-0.0656***	-0.0434**	-0.0122	0.0195
	(0.0084)	(0.0174)	(0.0093)	(0.0188)	(0.0093)	(0.0178)
Income Inequality	0.8001**	0.3376	0.7021**	0.2504	1.2329***	0.8329***
	(0.3461)	(0.2078)	(0.3325)	(0.2636)	(0.3350)	(0.2374)
Health Expenditure	0.0049***	0.0021	0.0031	-0.0001	0.0022	0.0021
	(0.0018)	(0.0016)	(0.0020)	(0.0018)	(0.0018)	(0.0017)

Note:
Standard errors in parentheses ()
*** p<0.01, ** p<0.05, * p<0.1 by Z-test

Table S69: Long Run Coefficient Estimation using Panel-Corrected Standard Errors with Infrastructure Vulnerability for SDG 7.1.1 – With Control of Corruption, Governance Effectiveness, and Political Stability, Related to Figure 3

Variables	Model 1	Model 2	Model 1	Model 2	Model 1	Model 2
	Control of Corruption		Governance Effectiveness		Political Stability	
Electricity Generation	0.0252	-0.0966	0.0409	-0.2279***	0.0334	0.0907
	(0.0410)	(0.0927)	(0.0398)	(0.0678)	(0.0410)	(0.0682)
Financial Access	0.1326***	-0.0127	0.1416***	-0.2951**	0.1489***	0.1007
	(0.0383)	(0.2458)	(0.0386)	(0.1325)	(0.0381)	(0.2076)
Rural Population Growth	-0.0923***	0.2427***	-0.0863***	0.1046**	-0.0813***	0.2398***
	(0.0099)	(0.0738)	(0.0098)	(0.0473)	(0.0108)	(0.0746)
Income Inequality	0.6513*	-3.0883***	0.5790*	-2.8493***	0.5155	-1.3438*
	(0.3376)	(0.8465)	(0.3355)	(0.5537)	(0.3292)	(0.7798)
Health Expenditure	0.0042**	-0.0027	0.0049***	0.0003	0.0047***	-0.0056
	(0.0019)	(0.0045)	(0.0018)	(0.0034)	(0.0018)	(0.0049)
Infrastructure Vulnerability	-1.0007***	-16.2761***	-1.0863***	-23.1999***	-1.1765***	-0.6038
	(0.2361)	(6.2223)	(0.2814)	(5.1456)	(0.2510)	(6.3317)
Quality of Governance	-0.0568*	0.6247	-0.0150	4.4120***	0.0017	-0.3769
	(0.0292)	(0.8425)	(0.0297)	(1.3237)	(0.0199)	(0.9560)
Electricity Generation * Infrastructure Vulnerability	-	0.0546	-	1.3190***	-	-1.2136**
	-	(0.6039)	-	(0.4758)	-	(0.5249)
Financial Access * Infrastructure Vulnerability	-	0.8761	-	2.0320**	-	1.5964
	-	(1.6617)	-	(0.8755)	-	(1.5467)
Rural Population Growth * Infrastructure Vulnerability	-	-1.6411***	-	-0.6564**	-	-1.7719***
	-	(0.5070)	-	(0.3291)	-	(0.4933)
Income Inequality * Infrastructure Vulnerability	-	20.0056***	-	16.7016***	-	9.0494*
	-	(5.2831)	-	(3.0455)	-	(5.0929)
Health Expenditure * Infrastructure Vulnerability	-	0.0354	-	0.0111	-	0.0523*
	-	(0.0239)	-	(0.0211)	-	(0.0312)
Electricity Generation * Quality of Governance	-	0.1794	-	-0.0506	-	0.1141
	-	(0.1235)	-	(0.1091)	-	(0.0711)
Financial Access * Quality of Governance	-	-0.1507	-	-0.9746***	-	0.3546
	-	(0.2934)	-	(0.3329)	-	(0.2291)
Rural Population Growth * Quality of Governance	-	0.2461***	-	0.6374***	-	0.0640
	-	(0.0896)	-	(0.1583)	-	(0.1271)
Income Inequality * Quality of Governance	-	-2.6019**	-	-4.1208***	-	-0.4250
	-	(1.1911)	-	(1.1608)	-	(0.8757)
Health Expenditure * Quality of Governance	-	-0.0085*	-	-0.0107	-	-0.0143**
	-	(0.0051)	-	(0.0095)	-	(0.0065)
Electricity Generation * Quality of Governance * Infrastructure Vulnerability	-	-1.8422**	-	-3.0977***	-	-0.3208
	-	(0.7359)	-	(0.6865)	-	(0.5593)
Financial Access * Quality of Governance * Infrastructure Vulnerability	-	2.2844	-	8.5086***	-	-0.8777
	-	(2.3967)	-	(2.4679)	-	(1.9510)
Rural Population Growth * Quality of Governance * Infrastructure Vulnerability	-	-1.6460**	-	-4.3930***	-	-0.0905

	-	(0.6620)	-	(0.9912)	-	(0.8392)
Income Inequality * Quality of Governance * Infrastructure Vulnerability	-	15.3001	-	21.0365***	-	0.3089
	-	(9.7869)	-	(7.5655)	-	(5.1300)
Health Expenditure * Quality of Governance * Infrastructure Vulnerability	-	0.0828***	-	0.0849**	-	0.0809**
	-	(0.0268)	-	(0.0418)	-	(0.0374)
Constant	3.7697***	7.6034***	3.6959***	8.4733***	3.8175***	5.0455***
	(0.5676)	(1.1143)	(0.5553)	(0.8902)	(0.5502)	(1.0467)
Wald-chi2	168.4	967.0	141.2	1322	121.1	976.0
Observations	115	115	115	115	115	115
Heteroskedasticity controlled	Yes	Yes	Yes	Yes	Yes	Yes
Autocorrelation controlled	Yes	Yes	Yes	Yes	Yes	Yes
Cross-sectional correlation controlled	Yes	Yes	Yes	Yes	Yes	Yes
Marginal Impact						
Electricity Generation	0.0252	-0.0584*	0.0409	-0.0312	0.0334	-0.1358***
	(0.0410)	(0.0337)	(0.0398)	(0.0330)	(0.0410)	(0.0385)
Financial Access	0.1326***	0.0654	0.1416***	0.0786**	0.1489***	0.2414***
	(0.0383)	(0.0421)	(0.0386)	(0.0357)	(0.0381)	(0.0422)
Rural Population Growth	-0.0923***	-0.0227	-0.0863***	-0.0271**	-0.0813***	-0.0699***
	(0.0099)	(0.0150)	(0.0098)	(0.0126)	(0.0108)	(0.0171)
Income Inequality	0.6513*	0.2466	0.5790*	-0.0443	0.5155	0.3073
	(0.3376)	(0.1650)	(0.3355)	(0.1710)	(0.3292)	(0.1934)
Health Expenditure	0.0042**	0.0017	0.0049***	0.0026**	0.0047***	0.0030***
	(0.0019)	(0.0011)	(0.0018)	(0.0011)	(0.0018)	(0.0011)

Note:
Standard errors in parentheses ()
*** p<0.01, ** p<0.05, * p<0.1 by Z-test

Table S70: Long Run Coefficient Estimation using Panel-Corrected Standard Errors with Infrastructure Vulnerability for SDG 7.1.1 – With Regulatory Quality, Rule of Law, and Voice and Accountability, Related to Figure 3

Variables	Model 1	Model 2	Model 1	Model 2	Model 1	Model 2
	Regulatory Quality		Rule of Law		Voice and Accountability	
Electricity Generation	0.0198	-0.0637	0.0124	-0.1866	-0.0522	-0.1693
	(0.0376)	(0.0713)	(0.0384)	(0.1308)	(0.0402)	(0.1224)
Financial Access	0.1328***	-0.1446	0.1535***	-0.3754	0.1851***	-0.5218***
	(0.0422)	(0.1603)	(0.0357)	(0.2490)	(0.0412)	(0.1675)
Rural Population Growth	-0.0754***	0.1966***	-0.0781***	0.3582***	-0.0308***	0.1394**
	(0.0105)	(0.0539)	(0.0095)	(0.0612)	(0.0106)	(0.0550)
Income Inequality	0.4888	-2.4744***	0.5824*	-0.5749	1.0919***	-2.5950***
	(0.3209)	(0.6974)	(0.3222)	(1.1197)	(0.3089)	(0.5963)
Health Expenditure	0.0033**	-0.0002	0.0028	0.0063	0.0015	-0.0065*
	(0.0017)	(0.0038)	(0.0018)	(0.0068)	(0.0016)	(0.0037)
Infrastructure Vulnerability	-1.1653***	-14.2891**	-0.5022	-5.5738	-1.2083***	-17.5211***
	(0.2541)	(6.8667)	(0.3198)	(7.4581)	(0.2487)	(6.5307)
Quality of Governance	-0.0681**	0.9452	-0.1296***	2.4842*	-0.1268***	1.0262**
	(0.0316)	(1.3277)	(0.0425)	(1.5034)	(0.0243)	(0.4191)
Electricity Generation * Infrastructure Vulnerability	-	0.0281	-	-0.3026	-	0.0044
	-	(0.6176)	-	(0.6319)	-	(0.6425)
Financial Access * Infrastructure Vulnerability	-	1.5240	-	3.3573**	-	4.7910***
	-	(1.1193)	-	(1.5760)	-	(1.1476)
Rural Population Growth * Infrastructure Vulnerability	-	-1.4093***	-	-2.4459***	-	-0.8057*
	-	(0.3873)	-	(0.3975)	-	(0.4185)
Income Inequality * Infrastructure Vulnerability	-	17.3775***	-	6.7592	-	20.0686***
	-	(4.4784)	-	(5.1849)	-	(3.5021)
Health Expenditure * Infrastructure Vulnerability	-	0.0240	-	-0.0138	-	0.0423**
	-	(0.0249)	-	(0.0316)	-	(0.0202)
Electricity Generation * Quality of Governance	-	0.1584	-	-0.2780**	-	-0.0620
	-	(0.1384)	-	(0.1406)	-	(0.0529)
Financial Access * Quality of Governance	-	0.0399	-	-0.4748	-	-0.2057
	-	(0.4137)	-	(0.2962)	-	(0.1909)
Rural Population Growth * Quality of Governance	-	0.4905***	-	0.3671***	-	0.1385***
	-	(0.1529)	-	(0.0815)	-	(0.0476)
Income Inequality * Quality of Governance	-	-1.7733	-	0.4672	-	-0.2756
	-	(1.7349)	-	(1.3569)	-	(0.7126)
Health Expenditure * Quality of Governance	-	-0.0220***	-	0.0043	-	-0.0013
	-	(0.0076)	-	(0.0079)	-	(0.0039)
Electricity Generation * Quality of Governance * Infrastructure Vulnerability	-	-1.8347*	-	-0.8440	-	-0.3294
	-	(1.0243)	-	(0.7152)	-	(0.3961)
Financial Access * Quality of Governance * Infrastructure Vulnerability	-	0.5969	-	5.0661**	-	1.7687
	-	(2.9865)	-	(2.3034)	-	(1.1504)
Rural Population Growth * Quality of Governance * Infrastructure Vulnerability	-	-3.1531***	-	-2.2605***	-	-1.1655***

	-	(1.0769)	-	(0.5799)	-	(0.3165)
Income Inequality * Quality of Governance * Infrastructure Vulnerability	-	9.0369	-	1.7862	-	-2.1654
	-	(12.5175)	-	(8.4291)	-	(4.6343)
Health Expenditure * Quality of Governance * Infrastructure Vulnerability	-	0.1575***	-	0.0106	-	0.0106
	-	(0.0369)	-	(0.0374)	-	(0.0215)
Constant	3.9909***	6.9314***	3.8643***	6.7537***	4.2439***	8.2122***
	(0.5143)	(0.9799)	(0.5348)	(1.7798)	(0.4717)	(1.3305)
Wald-chi2	107.3	1201	153.2	1819	112.3	2299
Observations	115	115	115	115	115	115
Heteroskedasticity controlled	Yes	Yes	Yes	Yes	Yes	Yes
Autocorrelation controlled	Yes	Yes	Yes	Yes	Yes	Yes
Cross-sectional correlation controlled	Yes	Yes	Yes	Yes	Yes	Yes
Marginal Impact						
Electricity Generation	0.0198	-0.0374	0.0124	-0.1274***	-0.0522	-0.1476***
	(0.0376)	(0.0470)	(0.0384)	(0.0383)	(0.0402)	(0.0493)
Financial Access	0.1328***	0.0812**	0.1535***	0.1335***	0.1851***	0.2400***
	(0.0422)	(0.0360)	(0.0357)	(0.0457)	(0.0412)	(0.0495)
Rural Population Growth	-0.0754***	-0.0151	-0.0781***	-0.0649***	-0.0308***	0.0119
	(0.0105)	(0.0165)	(0.0095)	(0.0144)	(0.0106)	(0.0179)
Income Inequality	0.4888	0.3009	0.5824*	0.3106	1.0919***	0.7168***
	(0.3209)	(0.1831)	(0.3222)	(0.1892)	(0.3089)	(0.2071)
Health Expenditure	0.0033**	0.0027**	0.0028	0.0025**	0.0015	0.0003
	(0.0017)	(0.0013)	(0.0018)	(0.0012)	(0.0016)	(0.0014)

Note:
Standard errors in parentheses ()
*** p<0.01, ** p<0.05, * p<0.1 by Z-test

Table S71: Long Run Coefficient Estimation using Panel-Corrected Standard Errors with Water Vulnerability for SDG 7.1.1 – With Control of Corruption, Governance Effectiveness, and Political Stability, Related to Figure 3

Variables	Model 1	Model 2	Model 1	Model 2	Model 1	Model 2
	Control of Corruption		Governance Effectiveness		Political Stability	
Electricity Generation	0.0809**	0.1449	0.1311***	0.1396	0.1140***	0.0436
	(0.0404)	(0.1882)	(0.0362)	(0.1878)	(0.0392)	(0.1578)
Financial Access	0.1417***	1.2889*	0.1545***	-0.7650**	0.1640***	1.9333***
	(0.0424)	(0.7078)	(0.0413)	(0.3850)	(0.0430)	(0.6364)
Rural Population Growth	-0.0835***	0.1078	-0.0883***	0.2481***	-0.0675***	0.0505
	(0.0104)	(0.0849)	(0.0103)	(0.0711)	(0.0103)	(0.0836)
Income Inequality	0.7408**	-7.2364***	0.8030**	-2.4977***	0.6008*	-1.7101
	(0.3627)	(1.9276)	(0.3638)	(0.9415)	(0.3633)	(2.0812)
Health Expenditure	0.0038*	0.0256***	0.0050***	0.0071	0.0052***	0.0302***
	(0.0020)	(0.0081)	(0.0019)	(0.0058)	(0.0019)	(0.0091)
Water Vulnerability	-0.0234	-7.1592	0.2545	-2.6287	-0.0554	3.9356
	(0.1663)	(4.8052)	(0.1876)	(5.6436)	(0.1770)	(5.7175)
Quality of Governance	-0.0877***	-0.9410	-0.0892***	-4.9664***	0.0097	0.0810
	(0.0300)	(0.9155)	(0.0305)	(1.4180)	(0.0198)	(1.1778)
Electricity Generation * Water Vulnerability	-	-0.3941	-	-0.5260	-	-0.2172
	-	(0.5858)	-	(0.5999)	-	(0.5696)
Financial Access * Water Vulnerability	-	-3.7011	-	3.2856***	-	-5.5941***
	-	(2.2553)	-	(1.2694)	-	(2.0855)
Rural Population Growth * Water Vulnerability	-	-0.3945	-	-0.9149***	-	-0.2147
	-	(0.2973)	-	(0.2412)	-	(0.2989)
Income Inequality * Water Vulnerability	-	21.9781***	-	8.5730***	-	5.3965
	-	(5.7997)	-	(2.4352)	-	(6.2792)
Health Expenditure * Water Vulnerability	-	-0.0591***	-	-0.0061	-	-0.0853***
	-	(0.0224)	-	(0.0163)	-	(0.0277)
Electricity Generation * Quality of Governance	-	0.2137	-	-0.2253	-	-0.3474**
	-	(0.3199)	-	(0.2281)	-	(0.1685)
Financial Access * Quality of Governance	-	2.5228**	-	3.7545***	-	2.9035***
	-	(1.2033)	-	(0.9463)	-	(0.9666)
Rural Population Growth * Quality of Governance	-	-0.3134	-	-0.4875*	-	-0.1534
	-	(0.1998)	-	(0.2958)	-	(0.1668)
Income Inequality * Quality of Governance	-	-6.4758*	-	3.1017	-	0.4444
	-	(3.5486)	-	(2.8491)	-	(2.1768)
Health Expenditure * Quality of Governance	-	0.0495***	-	0.0599***	-	0.0241*
	-	(0.0137)	-	(0.0186)	-	(0.0126)
Electricity Generation * Quality of Governance * Water Vulnerability	-	-0.3347	-	1.9890**	-	1.2432*
	-	(1.1997)	-	(0.9288)	-	(0.7180)
Financial Access * Quality of Governance * Water Vulnerability	-	-8.3686**	-	-12.9638***	-	-9.6754***
	-	(4.1056)	-	(3.3179)	-	(3.3400)
Rural Population Growth * Quality of Governance * Water Vulnerability	-	1.0069	-	1.7912*	-	0.7336

	-	(0.7025)	-	(0.9150)	-	(0.5590)
Income Inequality * Quality of Governance * Water Vulnerability	-	19.6408	-	-2.6653	-	-3.1217
	-	(12.0081)	-	(8.7787)	-	(6.4631)
Health Expenditure * Quality of Governance * Water Vulnerability	-	-0.1261***	-	-0.1481***	-	-0.0794**
	-	(0.0408)	-	(0.0523)	-	(0.0351)
Constant	3.1248***	6.6005***	2.5684***	5.1608***	2.9486***	3.2834
	(0.5832)	(1.9512)	(0.5636)	(1.9654)	(0.5664)	(2.0694)
Wald-chi2	186.4	710.1	200.9	1339	119.9	391.2
Observations	115	115	115	115	115	115
Heteroskedasticity controlled	Yes	Yes	Yes	Yes	Yes	Yes
Autocorrelation controlled	Yes	Yes	Yes	Yes	Yes	Yes
Cross-sectional correlation controlled	Yes	Yes	Yes	Yes	Yes	Yes
Marginal Impact						
Electricity Generation	0.0809**	-0.0292	0.1311***	-0.0341	0.1140***	-0.1106**
	(0.0404)	(0.0344)	(0.0362)	(0.0401)	(0.0392)	(0.0513)
Financial Access	0.1417***	0.1152**	0.1545***	0.2917***	0.1640***	0.4262***
	(0.0424)	(0.0504)	(0.0413)	(0.0713)	(0.0430)	(0.0989)
Rural Population Growth	-0.0835***	-0.0467***	-0.0883***	-0.0625***	-0.0675***	-0.1001***
	(0.0104)	(0.0171)	(0.0103)	(0.0203)	(0.0103)	(0.0237)
Income Inequality	0.7408**	0.4483**	0.8030**	0.5082***	0.6008*	0.6444***
	(0.3627)	(0.1972)	(0.3638)	(0.1935)	(0.3633)	(0.2320)
Health Expenditure	0.0038*	0.0027**	0.0050***	0.0034***	0.0052***	0.0036***
	(0.0020)	(0.0012)	(0.0019)	(0.0011)	(0.0019)	(0.0013)

Note:
Standard errors in parentheses ()
*** p<0.01, ** p<0.05, * p<0.1 by Z-test

Table S72: Long Run Coefficient Estimation using Panel-Corrected Standard Errors with Water Vulnerability for SDG 7.1.1 – With Regulatory Quality, Rule of Law, and Voice and Accountability, Related to Figure 3

Variables	Model 1	Model 2	Model 1	Model 2	Model 1	Model 2
	Regulatory Quality		Rule of Law		Voice and Accountability	
Electricity Generation	0.0907**	-0.0791	0.0331	0.2984	-0.0000	-0.0229
	(0.0354)	(0.1679)	(0.0372)	(0.2491)	(0.0396)	(0.2248)
Financial Access	0.1299***	1.0451**	0.2139***	-0.2563	0.1758***	-0.1130
	(0.0482)	(0.4845)	(0.0427)	(0.7488)	(0.0472)	(0.7266)
Rural Population Growth	-0.0674***	0.0303	-0.0709***	0.2211**	-0.0156*	0.1291
	(0.0097)	(0.0858)	(0.0094)	(0.1076)	(0.0092)	(0.0852)
Income Inequality	0.6318*	-6.0117***	0.5954*	-3.6574***	1.1594***	-1.8465*
	(0.3559)	(1.4061)	(0.3162)	(1.3807)	(0.3355)	(1.0190)
Health Expenditure	0.0038**	0.0133	0.0012	0.0155*	0.0016	0.0061
	(0.0018)	(0.0083)	(0.0019)	(0.0086)	(0.0018)	(0.0050)
Water Vulnerability	-0.1639	-11.6264*	0.4222*	0.6710	-0.3124	-5.0009
	(0.1894)	(6.2067)	(0.2219)	(5.8986)	(0.2210)	(5.3780)
Quality of Governance	-0.0858**	-4.2217***	-0.1886***	-1.3461	-0.1375***	-0.9785**
	(0.0336)	(1.3641)	(0.0422)	(1.1952)	(0.0290)	(0.3989)
Electricity Generation * Water Vulnerability	-	0.2384	-	-1.0774	-	0.1768
	-	(0.5567)	-	(0.7389)	-	(0.7431)
Financial Access * Water Vulnerability	-	-2.8804*	-	2.1359	-	1.4968
	-	(1.5929)	-	(2.4753)	-	(2.4827)
Rural Population Growth * Water Vulnerability	-	-0.2470	-	-0.7543**	-	-0.4887*
	-	(0.2855)	-	(0.3470)	-	(0.2956)
Income Inequality * Water Vulnerability	-	18.5820***	-	12.6302***	-	6.4448**
	-	(4.2341)	-	(3.5178)	-	(3.1956)
Health Expenditure * Water Vulnerability	-	-0.0256	-	-0.0341	-	-0.0095
	-	(0.0264)	-	(0.0216)	-	(0.0144)
Electricity Generation * Quality of Governance	-	0.0236	-	0.5171*	-	0.0617
	-	(0.3197)	-	(0.2748)	-	(0.1005)
Financial Access * Quality of Governance	-	4.6531***	-	-0.9347	-	0.5111
	-	(1.6510)	-	(1.3932)	-	(0.5820)
Rural Population Growth * Quality of Governance	-	0.1795	-	0.0155	-	0.1518**
	-	(0.2228)	-	(0.1953)	-	(0.0667)
Income Inequality * Quality of Governance	-	1.1211	-	-6.3207**	-	0.0281
	-	(3.7064)	-	(2.9235)	-	(1.1945)
Health Expenditure * Quality of Governance	-	0.0196	-	0.0260*	-	0.0018
	-	(0.0176)	-	(0.0155)	-	(0.0049)
Electricity Generation * Quality of Governance * Water Vulnerability	-	1.1767	-	-1.6688*	-	0.1215
	-	(1.1981)	-	(0.9385)	-	(0.3446)
Financial Access * Quality of Governance * Water Vulnerability	-	-14.5304***	-	4.8010	-	-0.7026
	-	(5.6044)	-	(4.8816)	-	(1.9009)
Rural Population Growth * Quality of Governance * Water Vulnerability	-	-0.2163	-	0.0890	-	-0.5057***
	-	(0.7480)	-	(0.6170)	-	(0.1928)

Income Inequality * Quality of Governance * Water Vulnerability	-	0.2539	-	24.6606**	-	-0.9168
	-	(12.0526)	-	(9.6090)	-	(3.6702)
Health Expenditure * Quality of Governance * Water Vulnerability	-	-0.0404	-	-0.0525	-	0.0054
	-	(0.0569)	-	(0.0455)	-	(0.0134)
Constant	3.2005***	8.1122***	3.4840***	4.1162*	3.7167***	5.5410***
	(0.5386)	(2.0373)	(0.5031)	(2.3608)	(0.5123)	(1.8543)
Wald-chi2	127.0	573.8	163.2	609.7	89.03	758.3
Observations	115	115	115	115	115	115
Heteroskedasticity controlled	Yes	Yes	Yes	Yes	Yes	Yes
Autocorrelation controlled	Yes	Yes	Yes	Yes	Yes	Yes
Cross-sectional correlation controlled	Yes	Yes	Yes	Yes	Yes	Yes
Marginal Impact						
Electricity Generation	0.0907**	-0.0561	0.0331	-0.0895	-0.0000	0.0186
	(0.0354)	(0.0458)	(0.0372)	(0.0557)	(0.0396)	(0.0640)
Financial Access	0.1299***	0.2322***	0.2139***	0.3568***	0.1758***	0.3670**
	(0.0482)	(0.0531)	(0.0427)	(0.1247)	(0.0472)	(0.1429)
Rural Population Growth	-0.0674***	-0.0669***	-0.0709***	-0.0599***	-0.0156*	-0.0391*
	(0.0097)	(0.0197)	(0.0094)	(0.0218)	(0.0092)	(0.0217)
Income Inequality	0.6318*	0.4710**	0.5954*	0.5217**	1.1594***	0.5109*
	(0.3559)	(0.2236)	(0.3162)	(0.2154)	(0.3355)	(0.3103)
Health Expenditure	0.0038**	0.0040***	0.0012	0.0004	0.0016	0.0020
	(0.0018)	(0.0014)	(0.0019)	(0.0012)	(0.0018)	(0.0012)

Note:
Standard errors in parentheses ()
*** p<0.01, ** p<0.05, * p<0.1 by Z-test

Table S73: Long Run Coefficient Estimation using Panel-Corrected Standard Errors with Ecosystem Vulnerability for SDG 7.1.2 – With Control of Corruption, Governance Effectiveness, and Political Stability, Related to Figure 3

Variables	Model 1	Model 2	Model 1	Model 2	Model 1	Model 2
	Control of Corruption		Governance Effectiveness		Political Stability	
Renewable Electricity Generation	0.0429***	-0.8114***	0.0422***	-0.9336***	0.0491***	-0.9233***
	(0.0120)	(0.2912)	(0.0120)	(0.1902)	(0.0122)	(0.2768)
Financial Access	-0.0451	-0.4608	-0.0521	-3.0481***	-0.1227	-3.1496**
	(0.0926)	(2.7013)	(0.0929)	(1.1507)	(0.1026)	(1.3723)
Policy Stringency	0.0018	-0.7630**	0.0024	0.0115	-0.0010	-0.3862
	(0.0143)	(0.3503)	(0.0142)	(0.1813)	(0.0154)	(0.2863)
Per Capita Income	0.4199***	-1.2685*	0.4222***	-1.8988***	0.4577***	-0.7404*
	(0.0428)	(0.6788)	(0.0441)	(0.5439)	(0.0435)	(0.4252)
Urban Population Growth	0.0000	-1.1080***	0.0007	-0.4519	0.0051	-0.1785
	(0.0120)	(0.4054)	(0.0120)	(0.2760)	(0.0131)	(0.2654)
Ecosystem Vulnerability	-2.1205***	-61.4548***	-2.1316***	-75.1596***	-2.1920***	-44.7218***
	(0.6057)	(16.0407)	(0.6246)	(11.3075)	(0.6306)	(12.5852)
Quality of Governance	0.0185	-0.0526	0.0045	-1.7162	0.0365*	6.5261***
	(0.0276)	(1.9711)	(0.0311)	(2.6432)	(0.0205)	(1.9201)
Renewable Electricity Generation * Ecosystem Vulnerability	-	1.6416***	-	1.9699***	-	1.8453***
	-	(0.5958)	-	(0.3915)	-	(0.5573)
Financial Access * Ecosystem Vulnerability	-	1.4989	-	8.0214***	-	7.5835**
	-	(5.6591)	-	(2.3988)	-	(2.9963)
Policy Stringency * Ecosystem Vulnerability	-	1.5458**	-	-0.2506	-	0.7839
	-	(0.7359)	-	(0.3757)	-	(0.6065)
Per Capita Income * Ecosystem Vulnerability	-	3.0601**	-	4.5274***	-	1.3400
	-	(1.4013)	-	(1.1207)	-	(0.8759)
Urban Population Growth * Ecosystem Vulnerability	-	2.2768***	-	0.9965*	-	0.3017
	-	(0.8456)	-	(0.5687)	-	(0.5612)
Renewable Electricity Generation * Quality of Governance	-	0.4586	-	0.9808***	-	-1.4337**
	-	(0.3993)	-	(0.3715)	-	(0.5624)
Financial Access * Quality of Governance	-	4.1416	-	13.2459***	-	-2.0051
	-	(3.3779)	-	(2.6065)	-	(1.7866)
Policy Stringency * Quality of Governance	-	-0.7803	-	-4.2644***	-	0.7075*
	-	(0.6461)	-	(0.5879)	-	(0.4248)
Per Capita Income * Quality of Governance	-	-0.2711	-	-1.0115***	-	1.2106**
	-	(0.3465)	-	(0.2404)	-	(0.5596)
Urban Population Growth * Quality of Governance	-	-1.6482***	-	1.0565*	-	0.1648
	-	(0.5581)	-	(0.6247)	-	(0.4074)
Renewable Electricity Generation * Quality of Governance * Ecosystem Vulnerability	-	-1.0103	-	-2.1610***	-	2.7900**
	-	(0.8191)	-	(0.7206)	-	(1.1335)
Financial Access * Quality of Governance * Ecosystem Vulnerability	-	-9.3283	-	-30.2468***	-	4.8361
	-	(7.4526)	-	(5.8036)	-	(4.1609)
Policy Stringency * Quality of Governance * Ecosystem Vulnerability	-	1.9096	-	9.1939***	-	-1.2586
	-	(1.4150)	-	(1.1743)	-	(0.8767)

Per Capita Income * Quality of Governance * Ecosystem Vulnerability	-	0.5986	-	2.5674***	-	-3.6298***
	-	(0.8095)	-	(0.6922)	-	(1.3474)
Urban Population Growth * Quality of Governance * Ecosystem Vulnerability	-	3.4515***	-	-2.1995*	-	-0.3983
	-	(1.1641)	-	(1.3211)	-	(0.8742)
Constant	0.4162	31.9371***	0.4010	37.2112***	0.0251	27.1710***
	(0.6426)	(7.8182)	(0.6706)	(5.6291)	(0.6579)	(6.3723)
Wald-chi2	274.6	2192	275.2	8041	356.0	2545
Observations	115	115	115	115	115	115
Heteroskedasticity controlled	Yes	Yes	Yes	Yes	Yes	Yes
Autocorrelation controlled	Yes	Yes	Yes	Yes	Yes	Yes
Cross-sectional correlation controlled	Yes	Yes	Yes	Yes	Yes	Yes
Marginal Impact						
Renewable Electricity Generation	0.0429***	-0.0095	0.0422***	0.0065	0.0491***	0.0009
	(0.0120)	(0.0135)	(0.0120)	(0.0129)	(0.0122)	(0.0139)
Financial Access	-0.0451	0.3519***	-0.0521	0.6346***	-0.1227	0.3108***
	(0.0926)	(0.1187)	(0.0929)	(0.0968)	(0.1026)	(0.1138)
Policy Stringency	0.0018	-0.0545***	0.0024	-0.0444***	-0.0010	-0.0532***
	(0.0143)	(0.0189)	(0.0142)	(0.0155)	(0.0154)	(0.0181)
Per Capita Income	0.4199***	0.2119***	0.4222***	0.3132***	0.4577***	0.2369***
	(0.0428)	(0.0754)	(0.0441)	(0.0480)	(0.0435)	(0.0660)
Urban Population Growth	0.0000	0.0049	0.0007	0.0152	0.0051	-0.0142
	(0.0120)	(0.0167)	(0.0120)	(0.0144)	(0.0131)	(0.0162)

Note:
Standard errors in parentheses ()
*** p<0.01, ** p<0.05, * p<0.1 by Z-test

Table S74: Long Run Coefficient Estimation using Panel-Corrected Standard Errors with Ecosystem Vulnerability for SDG 7.1.2 – With Regulatory Quality, Rule of Law, and Voice and Accountability, Related to Figure 3

Variables	Model 1	Model 2	Model 1	Model 2	Model 1	Model 2
	Regulatory Quality		Rule of Law		Voice and Accountability	
Renewable Electricity Generation	0.0476***	-0.7163***	0.0426***	-0.4559**	0.0708***	0.3285*
	(0.0124)	(0.1780)	(0.0115)	(0.2011)	(0.0138)	(0.1872)
Financial Access	-0.0697	-1.6337	-0.0648	1.2251	-0.2570**	-5.5684***
	(0.0989)	(1.0246)	(0.0983)	(2.0198)	(0.1250)	(1.1174)
Policy Stringency	0.0006	-0.7952***	0.0015	-0.9675***	-0.0067	-0.5644***
	(0.0150)	(0.1889)	(0.0149)	(0.2378)	(0.0189)	(0.1486)
Per Capita Income	0.4380***	-2.3574***	0.4480***	0.3990	0.5325***	-0.0063
	(0.0428)	(0.6210)	(0.0456)	(0.5291)	(0.0518)	(0.3546)
Urban Population Growth	0.0048	-0.6677**	0.0073	-0.0628	0.0345**	0.0138
	(0.0124)	(0.3047)	(0.0129)	(0.4254)	(0.0157)	(0.1739)
Ecosystem Vulnerability	-2.1604***	-77.6478***	-1.9203***	-18.2033	-2.6900***	-7.2389
	(0.6286)	(13.6189)	(0.6579)	(12.9336)	(0.6829)	(8.9330)
Quality of Governance	0.0336	-2.7039	-0.0517	-8.4763***	0.0462	-2.4861***
	(0.0364)	(2.3074)	(0.0437)	(1.9992)	(0.0301)	(0.4652)
Renewable Electricity Generation * Ecosystem Vulnerability	-	1.4448***	-	0.9386**	-	-0.6926*
	-	(0.3590)	-	(0.4108)	-	(0.3810)
Financial Access * Ecosystem Vulnerability	-	4.3959*	-	-1.9082	-	12.3031***
	-	(2.2432)	-	(4.2359)	-	(2.4175)
Policy Stringency * Ecosystem Vulnerability	-	1.5769***	-	1.9679***	-	1.1722***
	-	(0.3952)	-	(0.4856)	-	(0.3042)
Per Capita Income * Ecosystem Vulnerability	-	5.0732***	-	0.3461	-	0.9413
	-	(1.2547)	-	(1.0175)	-	(0.7082)
Urban Population Growth	-	1.3175**	-	0.1038	-	-0.0745
	-	(0.6352)	-	(0.8842)	-	(0.3669)
Renewable Electricity Generation * Quality of Governance	-	0.5213	-	1.0571*	-	0.3542*
	-	(0.3262)	-	(0.5926)	-	(0.1920)
Financial Access * Quality of Governance	-	5.2396**	-	10.2196***	-	-4.2406***
	-	(2.4024)	-	(3.0039)	-	(1.0887)
Policy Stringency * Quality of Governance	-	-3.2952***	-	-1.7643***	-	0.3940**
	-	(0.8002)	-	(0.6501)	-	(0.1852)
Per Capita Income * Quality of Governance	-	-0.1700	-	-0.5784	-	0.0927
	-	(0.3031)	-	(0.5893)	-	(0.2217)
Urban Population Growth	-	-0.8062**	-	-0.9352	-	-0.0728
	-	(0.4063)	-	(0.7464)	-	(0.1704)
Renewable Electricity Generation * Quality of Governance * Ecosystem Vulnerability	-	-1.0639	-	-2.2117*	-	-0.5058
	-	(0.6569)	-	(1.2128)	-	(0.3751)
Financial Access * Quality of Governance * Ecosystem Vulnerability	-	-11.6980**	-	-22.3491***	-	9.0482***
	-	(5.2563)	-	(6.6972)	-	(2.3057)
Policy Stringency * Quality of Governance * Ecosystem Vulnerability	-	7.0252***	-	3.8569***	-	-0.8461**

	-	(1.6505)	-	(1.3893)	-	(0.3776)
Per Capita Income * Quality of Governance * Ecosystem Vulnerability	-	0.8172	-	2.8469**	-	0.0210
	-	(0.7073)	-	(1.2950)	-	(0.4375)
Urban Population Growth	-	1.8248**	-	2.0247	-	0.2176
	-	(0.8661)	-	(1.5580)	-	(0.3444)
Constant	0.1931	41.0161***	0.0062	7.0543	-0.7668	3.2168
	(0.6536)	(6.8883)	(0.7197)	(6.7190)	(0.7947)	(4.4523)
Wald-chi2	331.4	4793	309.7	3311	679.5	8371
Observations	115	115	115	115	115	115
Heteroskedasticity controlled	Yes	Yes	Yes	Yes	Yes	Yes
Autocorrelation controlled	Yes	Yes	Yes	Yes	Yes	Yes
Cross-sectional correlation controlled	Yes	Yes	Yes	Yes	Yes	Yes
Marginal Impact						
Renewable Electricity Generation	0.0476***	-0.0148	0.0426***	-0.0180	0.0708***	-0.0307**
	(0.0124)	(0.0145)	(0.0115)	(0.0164)	(0.0138)	(0.0130)
Financial Access	-0.0697	0.5627***	-0.0648	0.2584**	-0.2570**	0.3882***
	(0.0989)	(0.0987)	(0.0983)	(0.1296)	(0.1250)	(0.0888)
Policy Stringency	0.0006	-0.0527***	0.0015	-0.0059	-0.0067	0.0055
	(0.0150)	(0.0200)	(0.0149)	(0.0170)	(0.0189)	(0.0110)
Per Capita Income	0.4380***	0.0765	0.4480***	0.3632***	0.5325***	0.4292***
	(0.0428)	(0.0644)	(0.0456)	(0.0711)	(0.0518)	(0.0503)
Urban Population Growth	0.0048	-0.0401***	0.0073	-0.0060	0.0345**	-0.0285**
	(0.0124)	(0.0140)	(0.0129)	(0.0209)	(0.0157)	(0.0124)

Note:
Standard errors in parentheses ()
*** p<0.01, ** p<0.05, * p<0.1 by Z-test

Table S75: Long Run Coefficient Estimation using Panel-Corrected Standard Errors with Food Vulnerability for SDG 7.1.2 – With Control of Corruption, Governance Effectiveness, and Political Stability, Related to Figure 3

Variables	Model 1	Model 2	Model 1	Model 2	Model 1	Model 2
	Control of Corruption		Governance Effectiveness		Political Stability	
Renewable Electricity Generation	0.0336**	-0.2981***	0.0357***	0.0682	0.0403***	-0.2274
	(0.0137)	(0.1059)	(0.0125)	(0.0784)	(0.0123)	(0.1399)
Financial Access	0.0205	-0.7049	0.0043	-1.5803**	-0.0347	-2.9014***
	(0.0754)	(1.0212)	(0.0812)	(0.6389)	(0.0858)	(0.8178)
Policy Stringency	0.0040	-0.1086	0.0009	-0.1322	-0.0023	-0.1151
	(0.0142)	(0.1020)	(0.0149)	(0.0806)	(0.0155)	(0.1362)
Per Capita Income	0.4241***	-0.4783	0.4419***	-0.9928***	0.4662***	-0.5469**
	(0.0464)	(0.2983)	(0.0479)	(0.2584)	(0.0495)	(0.2573)
Urban Population Growth	-0.0030	0.0359	0.0005	-0.1598	0.0017	-0.2273
	(0.0119)	(0.1658)	(0.0128)	(0.1130)	(0.0135)	(0.1810)
Food Vulnerability	-0.8102	-29.3957***	-1.0717**	-31.1828***	-1.0661**	-31.0003***
	(0.5412)	(6.7167)	(0.4868)	(5.9256)	(0.4557)	(6.3527)
Quality of Governance	-0.0046	-4.4382***	0.0097	-4.9382***	0.0341*	-0.9728
	(0.0258)	(1.1155)	(0.0304)	(1.7518)	(0.0201)	(0.9393)
Renewable Electricity Generation * Food Vulnerability	-	0.7325***	-	-0.1041	-	0.5846*
	-	(0.2420)	-	(0.1870)	-	(0.3273)
Financial Access * Food Vulnerability	-	2.8801	-	5.1695***	-	8.3336***
	-	(2.5023)	-	(1.6032)	-	(2.0470)
Policy Stringency * Food Vulnerability	-	0.2166	-	0.2170	-	0.2293
	-	(0.2435)	-	(0.1983)	-	(0.3315)
Per Capita Income * Food Vulnerability	-	1.8918***	-	2.9744***	-	1.9613***
	-	(0.6903)	-	(0.6229)	-	(0.6098)
Urban Population Growth * Food Vulnerability	-	-0.1026	-	0.2928	-	0.5076
	-	(0.3905)	-	(0.2691)	-	(0.4364)
Renewable Electricity Generation * Quality of Governance	-	-0.9252***	-	-1.0627***	-	-0.2595
	-	(0.2402)	-	(0.2516)	-	(0.1962)
Financial Access * Quality of Governance	-	1.3728	-	-0.6436	-	-0.2937
	-	(1.1853)	-	(1.3371)	-	(0.8757)
Policy Stringency * Quality of Governance	-	0.4838*	-	0.5304**	-	0.0927
	-	(0.2816)	-	(0.2224)	-	(0.1668)
Per Capita Income * Quality of Governance	-	1.2712***	-	1.3726***	-	0.3847*
	-	(0.2559)	-	(0.2809)	-	(0.2136)
Urban Population Growth * Quality of Governance	-	0.7012**	-	1.1228***	-	0.1692
	-	(0.3025)	-	(0.3136)	-	(0.1875)
Renewable Electricity Generation * Quality of Governance * Food Vulnerability	-	2.2583***	-	2.5784***	-	0.5732
	-	(0.5600)	-	(0.5832)	-	(0.4511)
Financial Access * Quality of Governance * Food Vulnerability	-	-3.8358	-	1.9041	-	0.6694
	-	(3.1244)	-	(3.5596)	-	(2.3053)
Policy Stringency * Quality of Governance * Food Vulnerability	-	-1.1888*	-	-1.2938**	-	-0.1610

	-	(0.7066)	-	(0.5475)	-	(0.3997)
Per Capita Income * Quality of Governance * Food Vulnerability	-	-2.0831***	-	-2.2224***	-	-0.6610
	-	(0.5416)	-	(0.5910)	-	(0.4892)
Urban Population Growth * Quality of Governance * Food Vulnerability	-	-1.3553*	-	-2.4259***	-	-0.2355
	-	(0.7379)	-	(0.7709)	-	(0.4672)
Constant	-0.2257	13.1451***	-0.3122	14.3483***	-0.5843	14.1198***
	(0.7012)	(2.8570)	(0.7063)	(2.4086)	(0.7191)	(2.5956)
Wald-chi2	238.2	15022	393.7	19697	530.8	5523
Observations	115	115	115	115	115	115
Heteroskedasticity controlled	Yes	Yes	Yes	Yes	Yes	Yes
Autocorrelation controlled	Yes	Yes	Yes	Yes	Yes	Yes
Cross-sectional correlation controlled	Yes	Yes	Yes	Yes	Yes	Yes
Marginal Impact						
Renewable Electricity Generation	0.0336**	0.0160	0.0357***	0.0379***	0.0403***	0.0259**
	(0.0137)	(0.0099)	(0.0125)	(0.0103)	(0.0123)	(0.0114)
Financial Access	0.0205	0.6616***	0.0043	0.7244***	-0.0347	0.7850***
	(0.0754)	(0.1157)	(0.0812)	(0.0804)	(0.0858)	(0.1132)
Policy Stringency	0.0040	-0.0056	0.0009	-0.0438***	-0.0023	-0.0227*
	(0.0142)	(0.0113)	(0.0149)	(0.0105)	(0.0155)	(0.0124)
Per Capita Income	0.4241***	0.2252***	0.4419***	0.3087***	0.4662***	0.2830***
	(0.0464)	(0.0369)	(0.0479)	(0.0363)	(0.0495)	(0.0410)
Urban Population Growth	-0.0030	-0.0541***	0.0005	-0.0461***	0.0017	-0.0344**
	(0.0119)	(0.0137)	(0.0128)	(0.0127)	(0.0135)	(0.0154)

Note:
Standard errors in parentheses ()
*** p<0.01, ** p<0.05, * p<0.1 by Z-test

Table S76: Long Run Coefficient Estimation using Panel-Corrected Standard Errors with Food Vulnerability for SDG 7.1.2 – With Regulatory Quality, Rule of Law, and Voice and Accountability, Related to Figure 3

Variables	Model 1	Model 2	Model 1	Model 2	Model 1	Model 2
	Regulatory Quality		Rule of Law		Voice and Accountability	
Renewable Electricity Generation	0.0384***	-0.0470	0.0348***	-0.0595	0.0579***	0.1898
	(0.0130)	(0.1060)	(0.0123)	(0.1079)	(0.0133)	(0.1271)
Financial Access	0.0138	-2.3183***	0.0135	-2.4556***	-0.0805	-2.5048***
	(0.0791)	(0.6694)	(0.0799)	(0.7755)	(0.0847)	(0.5952)
Policy Stringency	0.0019	-0.1359*	0.0028	-0.3032***	0.0096	-0.2497***
	(0.0149)	(0.0812)	(0.0143)	(0.0933)	(0.0168)	(0.0884)
Per Capita Income	0.4363***	-0.6981***	0.4436***	0.1048	0.4416***	-0.6991***
	(0.0464)	(0.2635)	(0.0473)	(0.3418)	(0.0496)	(0.2508)
Urban Population Growth	0.0003	-0.2813**	0.0009	0.0443	0.0105	-0.5976***
	(0.0125)	(0.1283)	(0.0124)	(0.1835)	(0.0131)	(0.1278)
Food Vulnerability	-1.0531**	-30.3933***	-0.8433*	-11.5597	-1.7171***	-26.1173***
	(0.4862)	(5.9966)	(0.4823)	(8.5853)	(0.4551)	(5.4926)
Quality of Governance	0.0403	-7.5177***	-0.0625	-6.0671***	0.0971***	-1.7188***
	(0.0316)	(1.4030)	(0.0385)	(1.8588)	(0.0265)	(0.5264)
Renewable Electricity Generation * Food Vulnerability	-	0.2048	-	0.1740	-	-0.4385
	-	(0.2456)	-	(0.2430)	-	(0.3002)
Financial Access * Food Vulnerability	-	6.6621***	-	7.2175***	-	6.8159***
	-	(1.6708)	-	(1.8552)	-	(1.5432)
Policy Stringency * Food Vulnerability	-	0.2624	-	0.6689***	-	0.5903***
	-	(0.1943)	-	(0.2150)	-	(0.2054)
Per Capita Income * Food Vulnerability	-	2.4304***	-	0.5874	-	2.5186***
	-	(0.6275)	-	(0.7353)	-	(0.5888)
Urban Population Growth * Food Vulnerability	-	0.5578*	-	-0.0941	-	1.3827***
	-	(0.3072)	-	(0.4307)	-	(0.3082)
Renewable Electricity Generation * Quality of Governance	-	-0.3951	-	-0.7184**	-	-0.5940***
	-	(0.2800)	-	(0.2893)	-	(0.1840)
Financial Access * Quality of Governance	-	0.1224	-	1.7089	-	-0.5600
	-	(1.4439)	-	(1.1847)	-	(0.7391)
Policy Stringency * Quality of Governance	-	-0.1079	-	0.3895	-	0.4002***
	-	(0.2497)	-	(0.3056)	-	(0.1491)
Per Capita Income * Quality of Governance	-	1.1246***	-	1.1922***	-	0.8811***
	-	(0.2798)	-	(0.3580)	-	(0.2104)
Urban Population Growth * Quality of Governance	-	0.2330	-	0.5987**	-	0.1272
	-	(0.3019)	-	(0.2724)	-	(0.1411)
Renewable Electricity Generation * Quality of Governance * Food Vulnerability	-	1.0863*	-	1.8987***	-	1.5440***
	-	(0.6273)	-	(0.6835)	-	(0.4545)
Financial Access * Quality of Governance * Food Vulnerability	-	-1.4620	-	-4.9501	-	0.8457
	-	(3.6128)	-	(3.1692)	-	(1.8941)
Policy Stringency * Quality of Governance * Food Vulnerability	-	0.2096	-	-1.0508	-	-1.0453***

	-	(0.5882)	-	(0.7788)	-	(0.3726)
Per Capita Income * Quality of Governance * Food Vulnerability	-	-1.1290*	-	-1.6665**	-	-1.8268***
	-	(0.6049)	-	(0.7446)	-	(0.4954)
Urban Population Growth * Quality of Governance * Food Vulnerability	-	-0.1361	-	-1.1316*	-	-0.2945
	-	(0.7341)	-	(0.6621)	-	(0.3464)
Constant	-0.2956	13.2369***	-0.4439	5.1719	-0.2593	11.4679***
	(0.6880)	(2.4933)	(0.6996)	(4.0012)	(0.7175)	(2.4737)
Wald-chi2	384.7	21909	310.9	10088	677.8	17110
Observations	115	115	115	115	115	115
Heteroskedasticity controlled	Yes	Yes	Yes	Yes	Yes	Yes
Autocorrelation controlled	Yes	Yes	Yes	Yes	Yes	Yes
Cross-sectional correlation controlled	Yes	Yes	Yes	Yes	Yes	Yes
Marginal Impact						
Renewable Electricity Generation	0.0384***	0.0328***	0.0348***	0.0202	0.0579***	0.0323***
	(0.0130)	(0.0101)	(0.0123)	(0.0137)	(0.0133)	(0.0091)
Financial Access	0.0138	0.6992***	0.0135	0.7886***	-0.0805	0.5888***
	(0.0791)	(0.0870)	(0.0799)	(0.0996)	(0.0847)	(0.0999)
Policy Stringency	0.0019	-0.0180	0.0028	-0.0054	0.0096	-0.0124
	(0.0149)	(0.0113)	(0.0143)	(0.0117)	(0.0168)	(0.0116)
Per Capita Income	0.4363***	0.3124***	0.4436***	0.2012***	0.4416***	0.3384***
	(0.0464)	(0.0326)	(0.0473)	(0.0364)	(0.0496)	(0.0445)
Urban Population Growth	0.0003	-0.0528***	0.0009	-0.0482***	0.0105	0.0062
	(0.0125)	(0.0151)	(0.0124)	(0.0177)	(0.0131)	(0.0140)

Note:
Standard errors in parentheses ()
*** p<0.01, ** p<0.05, * p<0.1 by Z-test

Table S77: Long Run Coefficient Estimation using Panel-Corrected Standard Errors with Health Vulnerability for SDG 7.1.2 – With Control of Corruption, Governance Effectiveness, and Political Stability, Related to Figure 3

Variables	Model 1	Model 2	Model 1	Model 2	Model 1	Model 2
	Control of Corruption		Governance Effectiveness		Political Stability	
Renewable Electricity Generation	0.0305**	0.1421*	0.0366***	0.0498	0.0410***	0.1827**
	(0.0148)	(0.0746)	(0.0133)	(0.0468)	(0.0129)	(0.0875)
Financial Access	0.0346	-1.1233*	0.0274	-1.1301***	-0.0018	-1.0142**
	(0.0706)	(0.6779)	(0.0776)	(0.3550)	(0.0809)	(0.4907)
Policy Stringency	0.0000	-0.4643***	-0.0061	-0.3584***	-0.0087	-0.2829**
	(0.0142)	(0.1186)	(0.0146)	(0.0704)	(0.0146)	(0.1154)
Per Capita Income	0.3953***	-0.4166**	0.4105***	-0.4854***	0.4261***	-0.6167***
	(0.0422)	(0.1778)	(0.0457)	(0.1515)	(0.0467)	(0.1701)
Urban Population Growth	-0.0027	-0.0158	0.0029	-0.0336	0.0037	-0.2675**
	(0.0133)	(0.1140)	(0.0141)	(0.0691)	(0.0144)	(0.1089)
Health Vulnerability	-0.7402***	-14.7106***	-0.9050***	-16.3068***	-0.9043***	-18.1752***
	(0.2851)	(4.5346)	(0.2904)	(3.6435)	(0.2900)	(4.2005)
Quality of Governance	0.0014	-5.7288***	0.0063	-6.8088***	0.0320*	-1.4524
	(0.0260)	(0.9860)	(0.0301)	(1.5248)	(0.0192)	(1.0631)
Renewable Electricity Generation * Health Vulnerability	-	-0.2771*	-	-0.1328	-	-0.3552*
	-	(0.1625)	-	(0.1087)	-	(0.1985)
Financial Access * Health Vulnerability	-	3.4044**	-	3.6598***	-	3.4141***
	-	(1.5261)	-	(0.8641)	-	(1.2532)
Policy Stringency * Health Vulnerability	-	0.9679***	-	0.7304***	-	0.5902**
	-	(0.2545)	-	(0.1540)	-	(0.2502)
Per Capita Income * Health Vulnerability	-	1.4197***	-	1.4244***	-	1.8270***
	-	(0.3909)	-	(0.3616)	-	(0.3596)
Urban Population Growth * Health Vulnerability	-	0.0395	-	0.0432	-	0.6265**
	-	(0.2630)	-	(0.1649)	-	(0.2646)
Renewable Electricity Generation * Quality of Governance	-	0.4748***	-	0.4046***	-	0.1499
	-	(0.1490)	-	(0.1209)	-	(0.1526)
Financial Access * Quality of Governance	-	0.7528	-	0.4454	-	0.3593
	-	(0.7817)	-	(0.6960)	-	(0.4902)
Policy Stringency * Quality of Governance	-	-0.3463	-	-0.3509***	-	0.0228
	-	(0.2157)	-	(0.1182)	-	(0.1471)
Per Capita Income * Quality of Governance	-	-0.0039	-	0.1330	-	-0.0462
	-	(0.1377)	-	(0.1469)	-	(0.1782)
Urban Population Growth * Quality of Governance	-	-0.0974	-	-0.0289	-	-0.0498
	-	(0.1931)	-	(0.2130)	-	(0.1265)
Renewable Electricity Generation * Quality of Governance * Health Vulnerability	-	-0.8236**	-	-0.6503**	-	-0.2913
	-	(0.3292)	-	(0.2785)	-	(0.3378)
Financial Access * Quality of Governance * Health Vulnerability	-	-2.5066	-	-1.4245	-	-1.0057
	-	(2.0220)	-	(1.9109)	-	(1.3846)
Policy Stringency * Quality of Governance * Health Vulnerability	-	0.7012	-	0.6750**	-	-0.0422

	-	(0.4994)	-	(0.2671)	-	(0.3147)
Per Capita Income * Quality of Governance * Health Vulnerability	-	0.9437***	-	0.8441***	-	0.3513
	-	(0.2802)	-	(0.2277)	-	(0.3834)
Urban Population Growth * Quality of Governance * Health Vulnerability	-	0.4842	-	0.2711	-	0.2461
	-	(0.4891)	-	(0.5527)	-	(0.3216)
Constant	0.0931	8.3217***	-0.0593	10.0454***	-0.2425	9.8939***
	(0.5469)	(2.0505)	(0.5939)	(1.5327)	(0.6078)	(1.9710)
Wald-chi2	267.3	4245	443.4	7847	543.9	3220
Observations	115	115	115	115	115	115
Heteroskedasticity controlled	Yes	Yes	Yes	Yes	Yes	Yes
Autocorrelation controlled	Yes	Yes	Yes	Yes	Yes	Yes
Cross-sectional correlation controlled	Yes	Yes	Yes	Yes	Yes	Yes
Marginal Impact						
Renewable Electricity Generation	0.0305**	-0.0273***	0.0366***	-0.0181**	0.0410***	0.0139
	(0.0148)	(0.0094)	(0.0133)	(0.0082)	(0.0129)	(0.0118)
Financial Access	0.0346	0.5815***	0.0274	0.5659***	-0.0018	0.6528***
	(0.0706)	(0.0862)	(0.0776)	(0.0739)	(0.0809)	(0.0995)
Policy Stringency	0.0000	0.0006	-0.0061	-0.0127	-0.0087	-0.0091
	(0.0142)	(0.0099)	(0.0146)	(0.0094)	(0.0146)	(0.0118)
Per Capita Income	0.3953***	0.1018***	0.4105***	0.1787***	0.4261***	0.1630***
	(0.0422)	(0.0395)	(0.0457)	(0.0392)	(0.0467)	(0.0469)
Urban Population Growth	-0.0027	-0.0373**	0.0029	-0.0125	0.0037	-0.0159
	(0.0133)	(0.0157)	(0.0141)	(0.0121)	(0.0144)	(0.0183)

Note:
Standard errors in parentheses ()
*** p<0.01, ** p<0.05, * p<0.1 by Z-test

Table S78: Long Run Coefficient Estimation using Panel-Corrected Standard Errors with Health Vulnerability for SDG 7.1.2 – With Regulatory Quality, Rule of Law, and Voice and Accountability, Related to Figure 3

Variables	Model 1	Model 2	Model 1	Model 2	Model 1	Model 2
	Regulatory Quality		Rule of Law		Voice and Accountability	
Renewable Electricity Generation	0.0389***	0.0719	0.0339***	0.1712**	0.0556***	0.3747***
	(0.0140)	(0.0488)	(0.0128)	(0.0692)	(0.0147)	(0.0623)
Financial Access	0.0389	-1.2057***	0.0298	-1.1663**	-0.0111	-0.9267***
	(0.0740)	(0.3210)	(0.0773)	(0.5608)	(0.0794)	(0.3350)
Policy Stringency	-0.0043	-0.2934***	-0.0018	-0.3752***	-0.0073	-0.1204
	(0.0147)	(0.0703)	(0.0141)	(0.0877)	(0.0155)	(0.0747)
Per Capita Income	0.3994***	-0.5356***	0.4120***	0.1963	0.4108***	-0.3826**
	(0.0438)	(0.1561)	(0.0444)	(0.2309)	(0.0458)	(0.1739)
Urban Population Growth	0.0020	-0.1500**	0.0032	0.1653	0.0122	-0.1683**
	(0.0139)	(0.0733)	(0.0136)	(0.1221)	(0.0153)	(0.0831)
Health Vulnerability	-0.9171***	-19.7030***	-0.7952***	-2.8152	-1.1550***	-11.0016***
	(0.2883)	(3.4712)	(0.2681)	(5.5191)	(0.2865)	(3.7935)
Quality of Governance	0.0470	-10.7107***	-0.0662*	-7.8946***	0.0655**	-3.8606***
	(0.0316)	(1.4670)	(0.0383)	(1.6011)	(0.0278)	(0.5054)
Renewable Electricity Generation * Health Vulnerability	-	-0.1291	-	-0.3229**	-	-0.8145***
	-	(0.1125)	-	(0.1468)	-	(0.1361)
Financial Access * Health Vulnerability	-	3.4981***	-	3.7484***	-	2.5648***
	-	(0.7710)	-	(1.2591)	-	(0.8022)
Policy Stringency * Health Vulnerability	-	0.6316***	-	0.7781***	-	0.3292**
	-	(0.1472)	-	(0.1805)	-	(0.1504)
Per Capita Income * Health Vulnerability	-	1.7683***	-	0.4707	-	1.8386***
	-	(0.3478)	-	(0.4400)	-	(0.3619)
Urban Population Growth * Health Vulnerability	-	0.2808*	-	-0.3587	-	0.3563*
	-	(0.1699)	-	(0.2782)	-	(0.1934)
Renewable Electricity Generation * Quality of Governance	-	0.3225***	-	0.0974	-	-0.3338***
	-	(0.1250)	-	(0.2150)	-	(0.0860)
Financial Access * Quality of Governance	-	0.2888	-	0.9170	-	-0.7449*
	-	(0.6651)	-	(0.7811)	-	(0.4111)
Policy Stringency * Quality of Governance	-	-0.4333**	-	0.1095	-	0.2735***
	-	(0.2150)	-	(0.1728)	-	(0.0923)
Per Capita Income * Quality of Governance	-	0.6446***	-	0.4610*	-	0.7623***
	-	(0.1793)	-	(0.2490)	-	(0.1134)
Urban Population Growth * Quality of Governance	-	-0.0825	-	0.1769	-	0.1356*
	-	(0.1422)	-	(0.1901)	-	(0.0707)
Renewable Electricity Generation * Quality of Governance * Health Vulnerability	-	-0.3709	-	0.0230	-	0.9605***
	-	(0.2880)	-	(0.4818)	-	(0.2078)
Financial Access * Quality of Governance * Health Vulnerability	-	-2.4989	-	-2.6133	-	1.5554
	-	(1.7210)	-	(2.0796)	-	(0.9893)
Policy Stringency * Quality of Governance * Health Vulnerability	-	0.8621*	-	-0.2280	-	-0.6522***

	-	(0.4583)	-	(0.3966)	-	(0.2085)
Per Capita Income * Quality of Governance * Health Vulnerability	-	0.4588*	-	0.3904	-	-1.1482***
	-	(0.2575)	-	(0.5384)	-	(0.2389)
Urban Population Growth * Quality of Governance * Health Vulnerability	-	0.6065*	-	-0.3537	-	-0.1752
	-	(0.3633)	-	(0.4877)	-	(0.1658)
Constant	0.0343	10.2856***	-0.1183	0.8410	-0.1570	4.5421**
	(0.5645)	(1.6009)	(0.5732)	(2.8540)	(0.6009)	(1.9186)
Wald-chi2	442.0	11143	373.5	5146	803.0	10697
Observations	115	115	115	115	115	115
Heteroskedasticity controlled	Yes	Yes	Yes	Yes	Yes	Yes
Autocorrelation controlled	Yes	Yes	Yes	Yes	Yes	Yes
Cross-sectional correlation controlled	Yes	Yes	Yes	Yes	Yes	Yes
Marginal Impact						
Renewable Electricity Generation	0.0389***	-0.0041	0.0339***	-0.0098	0.0556***	0.0083
	(0.0140)	(0.0094)	(0.0128)	(0.0124)	(0.0147)	(0.0155)
Financial Access	0.0389	0.5263***	0.0298	0.6004***	-0.0111	0.3305***
	(0.0740)	(0.0695)	(0.0773)	(0.0935)	(0.0794)	(0.0813)
Policy Stringency	-0.0043	0.0025	-0.0018	-0.0195*	-0.0073	0.0146
	(0.0147)	(0.0094)	(0.0141)	(0.0109)	(0.0155)	(0.0119)
Per Capita Income	0.3994***	0.1912***	0.4120***	0.2397***	0.4108***	0.3840***
	(0.0438)	(0.0347)	(0.0444)	(0.0430)	(0.0458)	(0.0463)
Urban Population Growth	0.0020	-0.0428***	0.0032	-0.0141	0.0122	-0.0200
	(0.0139)	(0.0143)	(0.0136)	(0.0180)	(0.0153)	(0.0154)

Note:
Standard errors in parentheses ()
*** p<0.01, ** p<0.05, * p<0.1 by Z-test

Table S79: Long Run Coefficient Estimation using Panel-Corrected Standard Errors with Infrastructure Vulnerability for SDG 7.1.2 – With Control of Corruption, Governance Effectiveness, and Political Stability, Related to Figure 3

Variables	Model 1	Model 2	Model 1	Model 2	Model 1	Model 2
	Control of Corruption		Governance Effectiveness		Political Stability	
Renewable Electricity Generation	0.0467***	0.0996*	0.0607***	-0.0067	0.0636***	-0.0096
	(0.0150)	(0.0547)	(0.0131)	(0.0342)	(0.0131)	(0.0640)
Financial Access	0.0351	-0.5299	0.0136	0.2474	-0.0116	-0.1574
	(0.0707)	(0.4266)	(0.0838)	(0.1894)	(0.0848)	(0.2716)
Policy Stringency	0.0014	-0.0931*	-0.0074	-0.1829***	-0.0086	-0.0563
	(0.0129)	(0.0515)	(0.0139)	(0.0348)	(0.0141)	(0.0613)
Per Capita Income	0.4117***	-0.2293*	0.4547***	-0.4620***	0.4649***	-0.1254
	(0.0397)	(0.1260)	(0.0413)	(0.1166)	(0.0406)	(0.1229)
Urban Population Growth	-0.0019	-0.0142	0.0094	0.0517	0.0095	0.0325
	(0.0114)	(0.0645)	(0.0127)	(0.0475)	(0.0128)	(0.0666)
Infrastructure Vulnerability	-1.1788***	-31.8899***	-1.5056***	-48.2110***	-1.4950***	-28.3685***
	(0.3474)	(8.4143)	(0.4022)	(7.2815)	(0.4118)	(7.4711)
Quality of Governance	0.0024	-0.8431	0.0042	6.1216***	0.0285	1.1204
	(0.0251)	(1.2907)	(0.0297)	(1.5844)	(0.0190)	(1.3259)
Renewable Electricity Generation * Infrastructure Vulnerability	-	-0.5668	-	0.0199	-	0.2867
	-	(0.3768)	-	(0.2412)	-	(0.4484)
Financial Access * Infrastructure Vulnerability	-	6.1396**	-	0.5109	-	3.8157*
	-	(2.9699)	-	(1.2588)	-	(2.1341)
Policy Stringency * Infrastructure Vulnerability	-	0.6460*	-	1.1240***	-	0.3620
	-	(0.3479)	-	(0.1880)	-	(0.3746)
Per Capita Income * Infrastructure Vulnerability	-	3.1804***	-	4.3467***	-	2.0472***
	-	(0.7871)	-	(0.7769)	-	(0.6774)
Urban Population Growth * Infrastructure Vulnerability	-	0.1537	-	-0.3641	-	-0.2780
	-	(0.4694)	-	(0.3362)	-	(0.5206)
Renewable Electricity Generation * Quality of Governance	-	-0.0070	-	0.1578*	-	-0.1433
	-	(0.1262)	-	(0.0932)	-	(0.1189)
Financial Access * Quality of Governance	-	-0.6789	-	1.3832***	-	0.0387
	-	(0.4873)	-	(0.2123)	-	(0.2603)
Policy Stringency * Quality of Governance	-	0.1727*	-	-0.3178***	-	0.1781
	-	(0.0963)	-	(0.1136)	-	(0.1107)
Per Capita Income * Quality of Governance	-	0.1321	-	-0.7730***	-	0.0497
	-	(0.1243)	-	(0.1132)	-	(0.1177)
Urban Population Growth * Quality of Governance	-	-0.3042**	-	0.2528***	-	-0.0081
	-	(0.1210)	-	(0.0768)	-	(0.0838)
Renewable Electricity Generation * Quality of Governance * Infrastructure Vulnerability	-	0.0353	-	-0.8563	-	0.8926
	-	(0.9014)	-	(0.5905)	-	(0.8383)
Financial Access * Quality of Governance * Infrastructure Vulnerability	-	9.7794**	-	-6.4515***	-	1.5533
	-	(4.4923)	-	(2.2478)	-	(2.9484)
Policy Stringency * Quality of Governance * Infrastructure Vulnerability	-	-1.1798*	-	2.6722***	-	-0.9254

	-	(0.6827)	-	(0.6622)	-	(0.6363)
Per Capita Income * Quality of Governance * Infrastructure Vulnerability	-	-0.8195	-	0.7694	-	-1.1215
	-	(0.8566)	-	(0.6925)	-	(0.9963)
Urban Population Growth * Quality of Governance * Infrastructure Vulnerability	-	3.0488***	-	-1.7603***	-	0.2779
	-	(1.1102)	-	(0.5032)	-	(0.7869)
Constant	-0.4269	6.2209***	-0.9869**	9.5626***	-1.1015**	6.1580***
	(0.4685)	(1.4501)	(0.4930)	(1.2333)	(0.4884)	(1.6104)
Wald-chi2	190.7	8289	361.8	17879	406.8	5406
Observations	115	115	115	115	115	115
Heteroskedasticity controlled	Yes	Yes	Yes	Yes	Yes	Yes
Autocorrelation controlled	Yes	Yes	Yes	Yes	Yes	Yes
Cross-sectional correlation controlled	Yes	Yes	Yes	Yes	Yes	Yes
Marginal Impact						
Renewable Electricity Generation	0.0467***	0.0099	0.0607***	-0.0092	0.0636***	0.0305**
	(0.0150)	(0.0111)	(0.0131)	(0.0091)	(0.0131)	(0.0124)
Financial Access	0.0351	0.1971***	0.0136	0.2843***	-0.0116	0.2806***
	(0.0707)	(0.0685)	(0.0838)	(0.0522)	(0.0848)	(0.0916)
Policy Stringency	0.0014	0.0086	-0.0074	0.0120	-0.0086	-0.0092
	(0.0129)	(0.0137)	(0.0139)	(0.0112)	(0.0141)	(0.0159)
Per Capita Income	0.4117***	0.2728***	0.4547***	0.2432***	0.4649***	0.2810***
	(0.0397)	(0.0435)	(0.0413)	(0.0353)	(0.0406)	(0.0522)
Urban Population Growth	-0.0019	-0.0360**	0.0094	-0.0172	0.0095	-0.0339*
	(0.0114)	(0.0176)	(0.0127)	(0.0142)	(0.0128)	(0.0186)

Note:
Standard errors in parentheses ()
*** p<0.01, ** p<0.05, * p<0.1 by Z-test

Table S80: Long Run Coefficient Estimation using Panel-Corrected Standard Errors with Infrastructure Vulnerability for SDG 7.1.2 – With Regulatory Quality, Rule of Law, and Voice and Accountability, Related to Figure 3

Variables	Model 1	Model 2	Model 1	Model 2	Model 1	Model 2
	Regulatory Quality		Rule of Law		Voice and Accountability	
Renewable Electricity Generation	0.0636***	0.0451	0.0645***	0.0226	0.1079***	0.0339
	(0.0139)	(0.0344)	(0.0124)	(0.0456)	(0.0149)	(0.0452)
Financial Access	0.0280	-0.0082	-0.0113	0.1900	-0.1254	-0.3982**
	(0.0802)	(0.1857)	(0.0930)	(0.3008)	(0.0970)	(0.1614)
Policy Stringency	-0.0056	-0.1491***	-0.0117	-0.0991*	-0.0112	-0.1046***
	(0.0138)	(0.0406)	(0.0151)	(0.0506)	(0.0165)	(0.0350)
Per Capita Income	0.4419***	-0.3476***	0.4885***	-0.0658	0.5204***	0.0707
	(0.0390)	(0.0918)	(0.0448)	(0.1383)	(0.0470)	(0.0955)
Urban Population Growth	0.0077	-0.0773*	0.0235*	0.1071*	0.0482***	-0.0446
	(0.0124)	(0.0401)	(0.0142)	(0.0629)	(0.0162)	(0.0354)
Infrastructure Vulnerability	-1.5269***	-46.4770***	-1.5605***	-22.4531***	-2.3528***	-26.7206***
	(0.3938)	(6.1612)	(0.4421)	(8.3259)	(0.4431)	(6.1804)
Quality of Governance	0.0495	-0.8324	-0.0196	-0.5538	0.0995***	-1.4957***
	(0.0323)	(1.4721)	(0.0434)	(1.7567)	(0.0286)	(0.4141)
Renewable Electricity Generation * Infrastructure Vulnerability	-	-0.1806	-	-0.0327	-	-0.1543
	-	(0.2304)	-	(0.2920)	-	(0.3003)
Financial Access * Infrastructure Vulnerability	-	1.6686	-	2.2028	-	3.7769***
	-	(1.4212)	-	(2.0180)	-	(1.1418)
Policy Stringency * Infrastructure Vulnerability	-	0.9635***	-	0.6897**	-	0.7981***
	-	(0.2325)	-	(0.2847)	-	(0.2093)
Per Capita Income * Infrastructure Vulnerability	-	4.3385***	-	2.0114***	-	2.3600***
	-	(0.5870)	-	(0.7374)	-	(0.5857)
Urban Population Growth * Infrastructure Vulnerability	-	0.4424	-	-0.7542*	-	0.3637
	-	(0.2915)	-	(0.4430)	-	(0.2383)
Renewable Electricity Generation * Quality of Governance	-	-0.2855***	-	-0.0960	-	-0.0443
	-	(0.0894)	-	(0.1316)	-	(0.0626)
Financial Access * Quality of Governance	-	0.1840	-	-0.0758	-	-0.3646**
	-	(0.3732)	-	(0.4186)	-	(0.1498)
Policy Stringency * Quality of Governance	-	-0.0863	-	0.1748*	-	0.1742***
	-	(0.1153)	-	(0.0961)	-	(0.0418)
Per Capita Income * Quality of Governance	-	0.4153***	-	0.1631	-	0.2259***
	-	(0.1476)	-	(0.1527)	-	(0.0698)
Urban Population Growth * Quality of Governance	-	-0.0960	-	-0.1531	-	-0.0223
	-	(0.1154)	-	(0.1489)	-	(0.0371)
Renewable Electricity Generation * Quality of Governance * Infrastructure Vulnerability	-	2.2296***	-	0.3791	-	0.9621***
	-	(0.6404)	-	(0.9557)	-	(0.3624)
Financial Access * Quality of Governance * Infrastructure Vulnerability	-	1.1293	-	7.2465*	-	3.2057***
	-	(3.5601)	-	(4.3657)	-	(0.9457)
Policy Stringency * Quality of Governance * Infrastructure Vulnerability	-	0.9677	-	-1.0199	-	-1.0078***

	-	(0.7258)	-	(0.6273)	-	(0.2324)
Per Capita Income * Quality of Governance * Infrastructure Vulnerability	-	-2.8697***	-	-1.0149	-	-1.4384***
	-	(0.6319)	-	(1.0681)	-	(0.4327)
Urban Population Growth * Quality of Governance * Infrastructure Vulnerability	-	0.9543	-	2.1705	-	0.3101
	-	(0.9590)	-	(1.3681)	-	(0.2116)
Constant	-0.8840*	7.9291***	-1.3879***	4.8210***	-2.0779***	3.7816***
	(0.4685)	(1.1249)	(0.5341)	(1.6922)	(0.5606)	(1.1098)
Wald-chi2	362.5	15489	494.9	6899	934.0	30201
Observations	115	115	115	115	115	115
Heteroskedasticity controlled	Yes	Yes	Yes	Yes	Yes	Yes
Autocorrelation controlled	Yes	Yes	Yes	Yes	Yes	Yes
Cross-sectional correlation controlled	Yes	Yes	Yes	Yes	Yes	Yes
Marginal Impact						
Renewable Electricity Generation	0.0636***	0.0002	0.0645***	0.0328**	0.1079***	-0.0059
	(0.0139)	(0.0093)	(0.0124)	(0.0130)	(0.0149)	(0.0145)
Financial Access	0.0280	0.2131***	-0.0113	0.3292***	-0.1254	0.1982***
	(0.0802)	(0.0600)	(0.0930)	(0.0820)	(0.0970)	(0.0702)
Policy Stringency	-0.0056	-0.0066	-0.0117	-0.0066	-0.0112	0.0124
	(0.0138)	(0.0125)	(0.0151)	(0.0118)	(0.0165)	(0.0081)
Per Capita Income	0.4419***	0.3597***	0.4885***	0.2407***	0.5204***	0.4371***
	(0.0390)	(0.0426)	(0.0448)	(0.0480)	(0.0470)	(0.0434)
Urban Population Growth	0.0077	-0.0166	0.0235*	-0.0391**	0.0482***	0.0102
	(0.0124)	(0.0134)	(0.0142)	(0.0180)	(0.0162)	(0.0107)

Note:
Standard errors in parentheses ()
*** p<0.01, ** p<0.05, * p<0.1 by Z-test

Table S81: Long Run Coefficient Estimation using Panel-Corrected Standard Errors with Water Vulnerability for SDG 7.1.2 – With Control of Corruption, Governance Effectiveness, and Political Stability, Related to Figure 3

Variables	Model 1	Model 2	Model 1	Model 2	Model 1	Model 2
	Control of Corruption		Governance Effectiveness		Political Stability	
Renewable Electricity Generation	0.0518***	0.2719***	0.0556***	-0.0109	0.0583***	0.3984***
	(0.0166)	(0.0908)	(0.0156)	(0.0583)	(0.0146)	(0.0975)
Financial Access	-0.0067	0.4358	-0.0247	0.7121*	-0.0638	0.8406*
	(0.0673)	(0.7786)	(0.0684)	(0.4145)	(0.0720)	(0.5065)
Policy Stringency	0.0167	-0.2294**	0.0180	-0.1370***	0.0205	-0.0471
	(0.0129)	(0.0964)	(0.0135)	(0.0410)	(0.0145)	(0.0982)
Per Capita Income	0.3632***	-1.6971***	0.3694***	-1.5585***	0.3886***	-1.6073***
	(0.0424)	(0.2108)	(0.0428)	(0.1785)	(0.0414)	(0.2218)
Urban Population Growth	0.0048	-0.3184***	0.0079	-0.2511***	0.0111	-0.3705***
	(0.0122)	(0.1233)	(0.0125)	(0.0718)	(0.0131)	(0.1208)
Water Vulnerability	-1.7068***	-48.9163***	-1.8129***	-54.5235***	-1.8298***	-42.5859***
	(0.5899)	(5.6237)	(0.5223)	(4.9209)	(0.4679)	(6.0729)
Quality of Governance	-0.0133	-2.3168*	0.0098	-3.2736*	0.0164	0.6406
	(0.0245)	(1.2713)	(0.0271)	(1.7578)	(0.0158)	(1.3941)
Renewable Electricity Generation * Water Vulnerability	-	-0.8333***	-	0.0087	-	-1.1865***
	-	(0.2796)	-	(0.1675)	-	(0.2933)
Financial Access * Water Vulnerability	-	-0.8238	-	-1.0768	-	-1.8822
	-	(2.4654)	-	(1.3274)	-	(1.6698)
Policy Stringency * Water Vulnerability	-	0.6917**	-	0.2988**	-	0.1677
	-	(0.3152)	-	(0.1299)	-	(0.3181)
Per Capita Income * Water Vulnerability	-	5.4254***	-	5.1276***	-	5.3598***
	-	(0.5705)	-	(0.5113)	-	(0.6323)
Urban Population Growth * Water Vulnerability	-	0.9236**	-	0.6983***	-	1.1468***
	-	(0.3870)	-	(0.2174)	-	(0.4034)
Renewable Electricity Generation * Quality of Governance	-	0.5823***	-	0.5275***	-	0.0885
	-	(0.1906)	-	(0.1655)	-	(0.1572)
Financial Access * Quality of Governance	-	0.0780	-	2.4197***	-	0.3478
	-	(1.4795)	-	(0.8765)	-	(0.7551)
Policy Stringency * Quality of Governance	-	0.1202	-	-0.4494***	-	0.3585**
	-	(0.2490)	-	(0.1527)	-	(0.1508)
Per Capita Income * Quality of Governance	-	-0.4729***	-	-0.3874**	-	-0.2186
	-	(0.1776)	-	(0.1698)	-	(0.1640)
Urban Population Growth	-	-0.2967	-	0.0483	-	-0.0053
	-	(0.2708)	-	(0.1760)	-	(0.1596)
Renewable Electricity Generation * Quality of Governance * Water Vulnerability	-	-1.6542***	-	-1.5662***	-	-0.3447
	-	(0.6064)	-	(0.5056)	-	(0.4652)
Financial Access * Quality of Governance * Water Vulnerability	-	-0.2033	-	-7.6297**	-	-0.9359
	-	(5.3213)	-	(3.2571)	-	(2.8705)
Policy Stringency * Quality of Governance * Water Vulnerability	-	-0.2787	-	1.5269***	-	-0.8715*

	-	(0.8379)	-	(0.4511)	-	(0.4786)
Per Capita Income * Quality of Governance * Water Vulnerability	-	1.9263***	-	1.9954***	-	0.5364
	-	(0.5531)	-	(0.6433)	-	(0.5737)
Urban Population Growth * Quality of Governance * Water Vulnerability	-	1.0961	-	0.0277	-	0.1660
	-	(0.9314)	-	(0.5558)	-	(0.5681)
Constant	0.4123	19.5611***	0.3454	21.0201***	0.1383	16.5856***
	(0.5323)	(2.0834)	(0.5387)	(1.8483)	(0.5232)	(2.3121)
Wald-chi2	243.5	6165	327.4	24789	426.9	6784
Observations	115	115	115	115	115	115
Heteroskedasticity controlled	Yes	Yes	Yes	Yes	Yes	Yes
Autocorrelation controlled	Yes	Yes	Yes	Yes	Yes	Yes
Cross-sectional correlation controlled	Yes	Yes	Yes	Yes	Yes	Yes
Marginal Impact						
Renewable Electricity Generation	0.0518***	-0.0256**	0.0556***	-0.0224**	0.0583***	0.0013
	(0.0166)	(0.0110)	(0.0156)	(0.0088)	(0.0146)	(0.0147)
Financial Access	-0.0067	0.1400	-0.0247	0.2592***	-0.0638	0.1796
	(0.0673)	(0.0962)	(0.0684)	(0.0918)	(0.0720)	(0.1427)
Policy Stringency	0.0167	0.0091	0.0180	-0.0171	0.0205	0.0022
	(0.0129)	(0.0181)	(0.0135)	(0.0138)	(0.0145)	(0.0216)
Per Capita Income	0.3632***	0.1649***	0.3694***	0.2839***	0.3886***	0.3076***
	(0.0424)	(0.0474)	(0.0428)	(0.0369)	(0.0414)	(0.0488)
Urban Population Growth	0.0048	-0.0201	0.0079	-0.0026	0.0111	0.0049
	(0.0122)	(0.0192)	(0.0125)	(0.0124)	(0.0131)	(0.0214)

Note:
Standard errors in parentheses ()
*** p<0.01, ** p<0.05, * p<0.1 by Z-test

Table S82: Long Run Coefficient Estimation using Panel-Corrected Standard Errors with Water Vulnerability for SDG 7.1.2 – With Regulatory Quality, Rule of Law, and Voice and Accountability, Related to Figure 3

Variables	Model 1	Model 2	Model 1	Model 2	Model 1	Model 2
	Regulatory Quality		Rule of Law		Voice and Accountability	
Renewable Electricity Generation	0.0567***	0.2188***	0.0524***	0.3005***	0.0922***	0.0933
	(0.0154)	(0.0755)	(0.0152)	(0.0792)	(0.0129)	(0.0664)
Financial Access	-0.0286	0.7586*	-0.0107	1.3664***	-0.2922***	-0.5540
	(0.0700)	(0.4356)	(0.0687)	(0.5172)	(0.0903)	(0.3678)
Policy Stringency	0.0188	-0.3841***	0.0175	-0.1985***	0.0453**	-0.1370***
	(0.0138)	(0.0578)	(0.0130)	(0.0494)	(0.0208)	(0.0411)
Per Capita Income	0.3744***	-1.6149***	0.3728***	-1.2107***	0.4709***	-0.8579***
	(0.0413)	(0.1824)	(0.0423)	(0.2440)	(0.0522)	(0.1729)
Urban Population Growth	0.0092	-0.4988***	0.0060	0.1045	0.0477***	-0.0986*
	(0.0127)	(0.0938)	(0.0121)	(0.1194)	(0.0141)	(0.0563)
Water Vulnerability	-1.8022***	-53.4768***	-1.6123***	-33.4674***	-2.4274***	-35.3977***
	(0.5039)	(4.7183)	(0.5189)	(5.6749)	(0.3445)	(4.0268)
Quality of Governance	0.0141	-8.4924***	-0.0580*	-3.0472**	0.0770***	-3.3985***
	(0.0291)	(1.7251)	(0.0352)	(1.5236)	(0.0244)	(0.3511)
Renewable Electricity Generation * Water Vulnerability	-	-0.6723***	-	-0.9078***	-	-0.3911*
	-	(0.2263)	-	(0.2383)	-	(0.2141)
Financial Access * Water Vulnerability	-	-2.1580	-	-3.2031*	-	2.1284*
	-	(1.4447)	-	(1.7163)	-	(1.2757)
Policy Stringency * Water Vulnerability	-	1.1279***	-	0.6776***	-	0.4160***
	-	(0.1734)	-	(0.1523)	-	(0.1269)
Per Capita Income * Water Vulnerability	-	5.5661***	-	4.3642***	-	3.7219***
	-	(0.5094)	-	(0.6293)	-	(0.5136)
Urban Population Growth * Water Vulnerability	-	1.4738***	-	-0.3360	-	0.3081*
	-	(0.2965)	-	(0.3660)	-	(0.1864)
Renewable Electricity Generation * Quality of Governance	-	0.8349***	-	0.5434**	-	0.2489***
	-	(0.2095)	-	(0.2111)	-	(0.0928)
Financial Access * Quality of Governance	-	0.8275	-	2.7181**	-	0.3230
	-	(1.1812)	-	(1.2443)	-	(0.2813)
Policy Stringency * Quality of Governance	-	-0.3675	-	-0.2604	-	0.2742***
	-	(0.2569)	-	(0.1773)	-	(0.0499)
Per Capita Income * Quality of Governance	-	-0.0602	-	-0.4705**	-	0.0453
	-	(0.2566)	-	(0.2388)	-	(0.1184)
Urban Population Growth * Quality of Governance	-	-0.5354**	-	0.2738	-	-0.2391***
	-	(0.2394)	-	(0.2707)	-	(0.0603)
Renewable Electricity Generation * Quality of Governance * Water Vulnerability	-	-2.4306***	-	-1.6722**	-	-0.3298
	-	(0.6702)	-	(0.6502)	-	(0.2696)
Financial Access * Quality of Governance * Water Vulnerability	-	-4.9518	-	-8.8060*	-	-0.8652
	-	(4.2795)	-	(4.6623)	-	(0.9314)
Policy Stringency * Quality of Governance * Water Vulnerability	-	1.4983*	-	1.1675**	-	-0.7194***

	-	(0.7931)	-	(0.5731)	-	(0.1412)
Per Capita Income * Quality of Governance * Water Vulnerability	-	2.4478***	-	2.2629***	-	0.2942
	-	(0.7116)	-	(0.7095)	-	(0.3221)
Urban Population Growth * Quality of Governance * Water Vulnerability	-	2.3058***	-	-0.6529	-	0.7941***
	-	(0.8031)	-	(0.8781)	-	(0.1621)
Constant	0.2764	19.7431***	0.2604	12.8532***	-0.8932	12.7041***
	(0.5186)	(1.8095)	(0.5353)	(2.3175)	(0.6837)	(1.4942)
Wald-chi2	359.6	17597	266.1	6148	1200	22865
Observations	115	115	115	115	115	115
Heteroskedasticity controlled	Yes	Yes	Yes	Yes	Yes	Yes
Autocorrelation controlled	Yes	Yes	Yes	Yes	Yes	Yes
Cross-sectional correlation controlled	Yes	Yes	Yes	Yes	Yes	Yes
Marginal Impact						
Renewable Electricity Generation	0.0567***	0.0087	0.0524***	-0.0344**	0.0922***	-0.0729***
	(0.0154)	(0.0117)	(0.0152)	(0.0136)	(0.0129)	(0.0176)
Financial Access	-0.0286	0.1471*	-0.0107	0.2066*	-0.2922***	0.2014**
	(0.0700)	(0.0752)	(0.0687)	(0.1199)	(0.0903)	(0.0940)
Policy Stringency	0.0188	-0.0168	0.0175	0.0165	0.0453**	0.0076
	(0.0138)	(0.0147)	(0.0130)	(0.0154)	(0.0208)	(0.0096)
Per Capita Income	0.3744***	0.2515***	0.3728***	0.2832***	0.4709***	0.4374***
	(0.0413)	(0.0364)	(0.0423)	(0.0436)	(0.0522)	(0.0420)
Urban Population Growth	0.0092	-0.0302**	0.0060	-0.0375*	0.0477***	0.0021
	(0.0127)	(0.0143)	(0.0121)	(0.0199)	(0.0141)	(0.0136)

Note:
Standard errors in parentheses ()
*** p<0.01, ** p<0.05, * p<0.1 by Z-test

Table S83: Long Run Coefficient Estimation using Panel-Corrected Standard Errors with Ecosystem Vulnerability for SDG 7.2.1 – With Control of Corruption, Governance Effectiveness, and Political Stability, Related to Figure 3

Variables	Model 1	Model 2	Model 1	Model 2	Model 1	Model 2
	Control of Corruption		Governance Effectiveness		Political Stability	
Policy Stringency	0.3014*	-6.4879**	0.3499**	-8.4293***	0.3091*	-10.1277***
	(0.1664)	(3.0004)	(0.1686)	(2.0485)	(0.1673)	(2.4693)
Environmental Innovation	-0.6957***	-1.7478	-0.5455***	-1.3282	-0.6195***	-6.7868**
	(0.1535)	(3.1498)	(0.1254)	(2.2815)	(0.1358)	(2.9819)
Financial Development	0.7959	31.7665	0.7595	24.9057*	0.3908	65.0628***
	(1.2408)	(21.9874)	(1.2143)	(14.0796)	(1.2874)	(19.6345)
Net Energy Import	9.7827***	14.3011	9.0324***	22.3543**	9.0059***	61.4262***
	(1.2041)	(24.7546)	(0.9434)	(10.4538)	(1.1104)	(20.8267)
Low Carbon Trade	-0.4033***	7.9039	-0.4084***	1.8608	-0.3150**	7.7821*
	(0.1497)	(4.9877)	(0.1500)	(2.2601)	(0.1447)	(4.0649)
Ecosystem Vulnerability	-16.8964***	-8.0810	-16.1184***	-3.8530	-19.0890***	-22.4585
	(2.7355)	(33.2266)	(2.6360)	(30.9265)	(3.4781)	(33.5367)
Quality of Governance	-0.8683**	-0.6868	-0.8972***	-8.1966***	-0.4890**	-1.1328
	(0.3744)	(3.8492)	(0.2842)	(3.0564)	(0.2334)	(2.4259)
Policy Stringency * Ecosystem Vulnerability	-	13.2361**	-	17.3975***	-	22.1859***
	-	(6.2398)	-	(4.2937)	-	(5.1676)
Environmental Innovation * Ecosystem Vulnerability	-	3.2981	-	2.0936	-	13.7220**
	-	(6.4216)	-	(4.6220)	-	(6.1820)
Financial Development * Ecosystem Vulnerability	-	-66.5262	-	-50.4083*	-	-136.2966***
	-	(46.0144)	-	(29.6681)	-	(41.5072)
Net Energy Import * Ecosystem Vulnerability	-	-17.7945	-	-32.2459	-	-120.0994**
	-	(54.0138)	-	(23.8165)	-	(47.2672)
Low Carbon Trade * Ecosystem Vulnerability	-	-17.4703*	-	-4.9245	-	-15.9170*
	-	(10.1335)	-	(4.5700)	-	(8.2313)
Policy Stringency * Quality of Governance	-	-6.3894	-	-11.0316**	-	-10.5456**
	-	(6.9604)	-	(5.6250)	-	(4.2986)
Environmental Innovation * Quality of Governance	-	-0.5110	-	-3.0617	-	-3.3946
	-	(3.7482)	-	(2.9159)	-	(2.3930)
Financial Development * Quality of Governance	-	14.5427	-	66.7153**	-	34.0624
	-	(41.5332)	-	(32.9801)	-	(26.9286)
Net Energy Import * Quality of Governance	-	21.0241	-	37.4452	-	55.5540**
	-	(31.8076)	-	(23.1216)	-	(22.3363)
Low Carbon Trade * Quality of Governance	-	6.0948	-	-17.2430**	-	5.1201
	-	(6.7131)	-	(8.1681)	-	(5.2504)
Policy Stringency * Quality of Governance * Ecosystem Vulnerability	-	12.1823	-	19.1795*	-	23.0639***
	-	(14.8180)	-	(11.1749)	-	(8.5454)
Environmental Innovation * Quality of Governance * Ecosystem Vulnerability	-	2.9221	-	10.9201*	-	7.8998
	-	(7.9939)	-	(6.2869)	-	(5.0415)
Financial Development * Quality of Governance * Ecosystem Vulnerability	-	-42.3534	-	-143.1535**	-	-73.9057

	-	(90.0888)	-	(69.6949)	-	(55.6356)
Net Energy Import * Quality of Governance * Ecosystem Vulnerability	-	-55.1939	-	-104.0505**	-	-127.6442**
	-	(71.5858)	-	(51.5413)	-	(50.5274)
Low Carbon Trade * Quality of Governance * Ecosystem Vulnerability	-	-13.3641	-	35.2702**	-	-9.6973
	-	(13.9289)	-	(16.5199)	-	(10.6721)
Constant	9.8913***	5.0414	9.0092***	3.2476	10.8631***	11.3258
	(1.8063)	(16.3971)	(1.7053)	(14.9901)	(2.0897)	(16.3311)
Wald-chi2	146.4	928.9	164.2	1121	118.9	945.1
Observations	115	115	115	115	115	115
Heteroskedasticity controlled	Yes	Yes	Yes	Yes	Yes	Yes
Autocorrelation controlled	Yes	Yes	Yes	Yes	Yes	Yes
Cross-sectional correlation controlled	Yes	Yes	Yes	Yes	Yes	Yes
Marginal Impact						
Policy Stringency	0.3014*	0.1621	0.3499**	0.1676	0.3091*	0.1442
	(0.1664)	(0.2470)	(0.1686)	(0.1941)	(0.1673)	(0.2209)
Environmental Innovation	-0.6957***	-0.4504*	-0.5455***	-0.2567	-0.6195***	-0.4206**
	(0.1535)	(0.2338)	(0.1254)	(0.1755)	(0.1358)	(0.1880)
Financial Development	0.7959	1.3851	0.7595	-0.5648	0.3908	0.3758
	(1.2408)	(1.4243)	(1.2143)	(1.1591)	(1.2874)	(1.2108)
Net Energy Import	9.7827***	7.3974***	9.0324***	6.0912***	9.0059***	7.4492***
	(1.2041)	(1.7745)	(0.9434)	(1.6108)	(1.1104)	(1.3194)
Low Carbon Trade	-0.4033***	-0.5019***	-0.4084***	-0.2661*	-0.3150**	-0.1153
	(0.1497)	(0.1518)	(0.1500)	(0.1406)	(0.1447)	(0.1473)

Note:
Standard errors in parentheses ()
*** p<0.01, ** p<0.05, * p<0.1 by Z-test

Table S84: Long Run Coefficient Estimation using Panel-Corrected Standard Errors with Ecosystem Vulnerability for SDG 7.2.1 – With Regulatory Quality, Rule of Law, and Voice and Accountability, Related to Figure 3

Variables	Model 1	Model 2	Model 1	Model 2	Model 1	Model 2
	Regulatory Quality		Rule of Law		Voice and Accountability	
Policy Stringency	0.2956	-4.1339*	0.2942*	-2.5764	0.1705	0.1678
	(0.1803)	(2.1267)	(0.1740)	(2.0472)	(0.1738)	(1.7342)
Environmental Innovation	-0.5977***	-2.7904	-0.4252***	-16.5991***	0.1770	-4.7202*
	(0.1547)	(3.1458)	(0.1530)	(2.5804)	(0.2180)	(2.7842)
Financial Development	0.0812	60.6767***	0.1516	95.8329***	0.0087	25.3403
	(1.2916)	(15.3317)	(1.3454)	(13.5966)	(1.2351)	(15.4951)
Net Energy Import	9.1795***	41.8658**	7.3966***	24.7527	6.2035***	40.4970**
	(0.9674)	(17.1194)	(1.0314)	(18.8906)	(0.9673)	(16.7798)
Low Carbon Trade	-0.5522***	2.6390	-0.3625**	3.1524	-0.3102**	7.9228**
	(0.1592)	(3.2725)	(0.1529)	(3.8219)	(0.1350)	(3.2147)
Ecosystem Vulnerability	-18.0230***	33.9221	-18.3045***	-128.5911***	-19.2354***	-20.3567
	(2.7484)	(35.4057)	(3.1534)	(34.6369)	(2.8873)	(35.5992)
Quality of Governance	-0.4230	-4.6899	0.7053*	7.2119**	1.1707***	0.0797
	(0.3978)	(4.0130)	(0.4204)	(2.9598)	(0.2514)	(1.3910)
Policy Stringency * Ecosystem Vulnerability	-	9.3515**	-	4.9766	-	-0.1550
	-	(4.4112)	-	(4.1803)	-	(3.5961)
Environmental Innovation * Ecosystem Vulnerability	-	5.2886	-	33.4721***	-	9.2619*
	-	(6.4500)	-	(5.2190)	-	(5.5711)
Financial Development * Ecosystem Vulnerability	-	-129.7922***	-	-190.2791***	-	-47.4786
	-	(32.3410)	-	(28.0992)	-	(33.3614)
Net Energy Import * Ecosystem Vulnerability	-	-75.2257*	-	-46.6010	-	-63.8030*
	-	(38.6952)	-	(38.5796)	-	(34.7707)
Low Carbon Trade * Ecosystem Vulnerability	-	-5.5194	-	-7.5742	-	-18.2822***
	-	(6.6712)	-	(7.7175)	-	(6.5232)
Policy Stringency * Quality of Governance	-	-0.7738	-	-6.5687	-	-1.2305
	-	(7.7993)	-	(6.5899)	-	(3.8295)
Environmental Innovation * Quality of Governance	-	-9.5284*	-	-11.4858***	-	-3.7073*
	-	(5.1078)	-	(3.0957)	-	(2.0208)
Financial Development * Quality of Governance	-	112.4289**	-	154.5832***	-	39.8755*
	-	(49.6609)	-	(33.6801)	-	(20.3656)
Net Energy Import * Quality of Governance	-	82.4649**	-	-59.4192**	-	-4.9804
	-	(41.8599)	-	(25.2477)	-	(15.8853)
Low Carbon Trade * Quality of Governance	-	-4.9004	-	0.3835	-	3.5810
	-	(7.4839)	-	(5.2198)	-	(3.0138)
Policy Stringency * Quality of Governance * Ecosystem Vulnerability	-	1.1603	-	11.8299	-	2.4019
	-	(15.6623)	-	(13.5238)	-	(7.6577)
Environmental Innovation * Quality of Governance * Ecosystem Vulnerability	-	23.5465**	-	22.4419***	-	7.7164*
	-	(10.6274)	-	(6.4013)	-	(4.0865)
Financial Development * Quality of Governance * Ecosystem Vulnerability	-	-253.0572**	-	-319.3530***	-	-84.0242**

	-	(102.3854)	-	(71.5090)	-	(42.0331)
Net Energy Import * Quality of Governance * Ecosystem Vulnerability	-	-190.2165**	-	113.2710**	-	12.7263
	-	(95.4144)	-	(53.2842)	-	(33.2577)
Low Carbon Trade * Quality of Governance * Ecosystem Vulnerability	-	11.5904	-	-1.7991	-	-8.5469
	-	(15.4415)	-	(10.5772)	-	(6.0453)
Constant	10.8213***	-14.4556	10.4798***	63.7591***	7.2217***	8.6841
	(1.9713)	(17.4234)	(1.9475)	(17.0470)	(1.8533)	(17.7189)
Wald-chi2	193.5	821.5	134.5	2130	211.3	1937
Observations	115	115	115	115	115	115
Heteroskedasticity controlled	Yes	Yes	Yes	Yes	Yes	Yes
Autocorrelation controlled	Yes	Yes	Yes	Yes	Yes	Yes
Cross-sectional correlation controlled	Yes	Yes	Yes	Yes	Yes	Yes
Marginal Impact						
Policy Stringency	0.2956	0.4231*	0.2942*	0.1936	0.1705	0.1115
	(0.1803)	(0.2438)	(0.1740)	(0.2077)	(0.1738)	(0.2100)
Environmental Innovation	-0.5977***	-0.4692**	-0.4252***	0.0281	0.1770	-0.2184
	(0.1547)	(0.1879)	(0.1530)	(0.1720)	(0.2180)	(0.2327)
Financial Development	0.0812	-0.7682	0.1516	0.4975	0.0087	2.3052
	(1.2916)	(1.3199)	(1.3454)	(1.2033)	(1.2351)	(1.4154)
Net Energy Import	9.1795***	6.7393***	7.3966***	4.5636***	6.2035***	9.3404***
	(0.9674)	(1.5840)	(1.0314)	(1.4196)	(0.9673)	(1.8592)
Low Carbon Trade	-0.5522***	-0.1341	-0.3625**	-0.3972***	-0.3102**	-0.8470***
	(0.1592)	(0.1430)	(0.1529)	(0.1368)	(0.1350)	(0.1592)

Note:
Standard errors in parentheses ()
*** p<0.01, ** p<0.05, * p<0.1 by Z-test

Table S85: Long Run Coefficient Estimation using Panel-Corrected Standard Errors with Food Vulnerability for SDG 7.2.1 – With Control of Corruption, Governance Effectiveness, and Political Stability, Related to Figure 3

Variables	Model 1	Model 2	Model 1	Model 2	Model 1	Model 2
	Control of Corruption		Governance Effectiveness		Political Stability	
Policy Stringency	0.2185	1.3017	0.2265	1.3651	0.2336	-3.1249
	(0.1885)	(1.7071)	(0.1945)	(1.1444)	(0.1894)	(1.9368)
Environmental Innovation	-0.5904***	-7.7297***	-0.4751***	-7.0240***	-0.5001***	-5.9516***
	(0.1711)	(1.6556)	(0.1400)	(1.0516)	(0.1507)	(1.8830)
Financial Development	4.0249***	77.6696***	4.2912***	14.8667*	3.7029***	85.1731***
	(1.2744)	(12.7686)	(1.2714)	(8.9233)	(1.2807)	(15.8812)
Net Energy Import	6.3688***	18.0513	6.2402***	21.1846***	5.4185***	-1.5682
	(1.2591)	(14.7481)	(0.9832)	(6.7778)	(1.0949)	(18.6392)
Low Carbon Trade	-0.3040*	0.8503	-0.3724**	3.6137***	-0.2713*	8.4536**
	(0.1558)	(2.7596)	(0.1580)	(1.3578)	(0.1545)	(3.4397)
Food Vulnerability	1.2352	-29.4776*	1.5689	-79.7216***	1.2498	-16.1410
	(1.3863)	(15.0683)	(1.3981)	(16.4557)	(1.6193)	(18.4277)
Quality of Governance	-0.6105	9.1690***	-0.8973***	-2.4378	-0.0922	4.7310**
	(0.4098)	(2.8564)	(0.3154)	(3.5270)	(0.2399)	(2.0964)
Policy Stringency * Food Vulnerability	-	-2.5116	-	-3.0880	-	8.0436*
	-	(3.8026)	-	(2.5881)	-	(4.3547)
Environmental Innovation * Food Vulnerability	-	15.8409***	-	15.4193***	-	10.5004**
	-	(3.8215)	-	(2.4424)	-	(4.6295)
Financial Development * Food Vulnerability	-	-175.0341***	-	-23.5850	-	-190.1197***
	-	(30.0644)	-	(20.9357)	-	(37.6910)
Net Energy Import * Food Vulnerability	-	-21.1991	-	-34.6652*	-	31.1333
	-	(37.1623)	-	(18.5178)	-	(50.0945)
Low Carbon Trade * Food Vulnerability	-	-3.3972	-	-10.2369***	-	-20.1199**
	-	(6.1473)	-	(3.1882)	-	(7.8128)
Policy Stringency * Quality of Governance	-	0.8155	-	-11.1398***	-	-3.5961
	-	(4.2497)	-	(3.2320)	-	(2.6117)
Environmental Innovation * Quality of Governance	-	-8.5850***	-	-5.7701**	-	-3.0566*
	-	(2.9900)	-	(2.3427)	-	(1.8380)
Financial Development * Quality of Governance	-	136.4760***	-	68.2577***	-	64.6181***
	-	(26.9704)	-	(19.0458)	-	(16.9507)
Net Energy Import * Quality of Governance	-	10.2441	-	83.4865***	-	-11.3857
	-	(25.1286)	-	(25.9482)	-	(20.8055)
Low Carbon Trade * Quality of Governance	-	-5.5883	-	-12.8044**	-	5.1212
	-	(4.5159)	-	(5.5848)	-	(3.6775)
Policy Stringency * Quality of Governance * Food Vulnerability	-	-2.0206	-	25.1808***	-	9.1237*
	-	(9.9072)	-	(6.8026)	-	(5.5298)
Environmental Innovation * Quality of Governance * Food Vulnerability	-	18.3026**	-	17.8762***	-	4.3130
	-	(7.2679)	-	(5.9720)	-	(4.3821)
Financial Development * Quality of Governance * Food Vulnerability	-	-353.2803***	-	-187.2987***	-	-151.8004***

	-	(65.5736)	-	(45.4137)	-	(38.4327)
Net Energy Import * Quality of Governance * Food Vulnerability	-	-10.8447	-	-225.7144***	-	39.6955
	-	(67.2162)	-	(70.1232)	-	(55.1904)
Low Carbon Trade * Quality of Governance * Food Vulnerability	-	13.1129	-	32.6974**	-	-10.7862
	-	(10.5116)	-	(13.0885)	-	(7.9763)
Constant	0.3163	15.6766**	-0.4810	34.6443***	0.3452	10.6654
	(1.4305)	(6.2317)	(1.4270)	(6.9400)	(1.4840)	(7.5348)
Wald-chi2	85.91	739.0	102.4	697.8	75.11	928.1
Observations	115	115	115	115	115	115
Heteroskedasticity controlled	Yes	Yes	Yes	Yes	Yes	Yes
Autocorrelation controlled	Yes	Yes	Yes	Yes	Yes	Yes
Cross-sectional correlation controlled	Yes	Yes	Yes	Yes	Yes	Yes
Marginal Impact						
Policy Stringency	0.2185	0.2011	0.2265	0.1581	0.2336	0.0487
	(0.1885)	(0.2064)	(0.1945)	(0.1833)	(0.1894)	(0.2233)
Environmental Innovation	-0.5904***	-0.4074**	-0.4751***	-0.0873	-0.5001***	-0.7208***
	(0.1711)	(0.1667)	(0.1400)	(0.1501)	(0.1507)	(0.1469)
Financial Development	4.0249***	4.5872***	4.2912***	3.3400***	3.7029***	4.7131***
	(1.2744)	(1.1331)	(1.2714)	(1.1066)	(1.2807)	(1.1582)
Net Energy Import	6.3688***	6.6650***	6.2402***	4.5096**	5.4185***	8.1463***
	(1.2591)	(1.7464)	(0.9832)	(1.7614)	(1.0949)	(1.7159)
Low Carbon Trade	-0.3040*	-0.6456***	-0.3724**	-0.7355***	-0.2713*	-0.4935***
	(0.1558)	(0.1527)	(0.1580)	(0.1557)	(0.1545)	(0.1654)

Note:
Standard errors in parentheses ()
*** p<0.01, ** p<0.05, * p<0.1 by Z-test

Table S86: Long Run Coefficient Estimation using Panel-Corrected Standard Errors with Food Vulnerability for SDG 7.2.1 – With Regulatory Quality, Rule of Law, and Voice and Accountability, Related to Figure 3

Variables	Model 1	Model 2	Model 1	Model 2	Model 1	Model 2
	Regulatory Quality		Rule of Law		Voice and Accountability	
Policy Stringency	0.2029	-1.6938	0.2199	2.0513	0.2044	0.7726
	(0.2053)	(1.5190)	(0.1964)	(1.5208)	(0.1835)	(1.0019)
Environmental Innovation	-0.4351**	-6.0595***	-0.4689***	-6.5037***	0.1308	-2.5825**
	(0.1726)	(1.5619)	(0.1652)	(1.4420)	(0.2330)	(1.0570)
Financial Development	3.9066***	47.1899***	3.7846***	30.7457***	2.4456*	-14.8666
	(1.2807)	(9.4925)	(1.3241)	(9.3436)	(1.2609)	(10.1502)
Net Energy Import	5.4239***	10.0071	5.3153***	1.6858	3.7402***	26.1354***
	(1.0168)	(12.2435)	(1.1297)	(10.8790)	(1.0707)	(9.0548)
Low Carbon Trade	-0.4338***	9.6407***	-0.3343**	4.6138**	-0.2460*	1.6105
	(0.1632)	(2.7608)	(0.1605)	(2.0797)	(0.1439)	(1.5428)
Food Vulnerability	1.1729	-37.5896**	1.1869	-75.2793***	-2.9872*	-27.3806*
	(1.3970)	(18.2699)	(1.4937)	(23.1151)	(1.6746)	(15.9728)
Quality of Governance	0.2065	5.7196*	0.0938	-1.9712	1.1903***	0.3297
	(0.3869)	(3.4312)	(0.4478)	(4.3795)	(0.3150)	(1.5217)
Policy Stringency * Food Vulnerability	-	3.9968	-	-4.6921	-	-1.3987
	-	(3.4594)	-	(3.3036)	-	(2.4103)
Environmental Innovation * Food Vulnerability	-	12.3317***	-	14.5363***	-	3.2643
	-	(3.7071)	-	(3.1943)	-	(2.4949)
Financial Development * Food Vulnerability	-	-103.6908***	-	-63.7370***	-	58.1012**
	-	(22.2639)	-	(21.6119)	-	(26.0252)
Net Energy Import * Food Vulnerability	-	-5.0076	-	6.8802	-	-35.2076*
	-	(32.4803)	-	(25.7134)	-	(21.3497)
Low Carbon Trade * Food Vulnerability	-	-22.7452***	-	-12.6444***	-	-6.2043*
	-	(6.3384)	-	(4.6264)	-	(3.4269)
Policy Stringency * Quality of Governance	-	-12.7272***	-	12.7566**	-	1.8966
	-	(4.0433)	-	(5.2984)	-	(1.8480)
Environmental Innovation * Quality of Governance	-	-3.5492	-	-6.1527**	-	-8.1031***
	-	(3.7634)	-	(2.8878)	-	(1.4981)
Financial Development * Quality of Governance	-	80.6347***	-	59.5552*	-	103.6017***
	-	(27.7462)	-	(32.2846)	-	(18.7168)
Net Energy Import * Quality of Governance	-	6.9713	-	-9.1119	-	22.6878**
	-	(35.1908)	-	(19.3042)	-	(11.2563)
Low Carbon Trade * Quality of Governance	-	10.6850*	-	0.1348	-	-0.4937
	-	(6.2303)	-	(3.8154)	-	(1.7514)
Policy Stringency * Quality of Governance * Food Vulnerability	-	28.2159***	-	-32.8476***	-	-4.2737
	-	(8.9118)	-	(12.5581)	-	(4.6239)
Environmental Innovation * Quality of Governance * Food Vulnerability	-	7.0580	-	17.9110***	-	20.0928***
	-	(9.0021)	-	(6.7734)	-	(3.8131)
Financial Development * Quality of Governance * Food Vulnerability	-	-214.0444***	-	-149.0586*	-	-270.1736***

	-	(63.4079)	-	(82.6811)	-	(47.4025)
Net Energy Import * Quality of Governance * Food Vulnerability	-	-3.9155	-	3.5852	-	-44.7340
	-	(95.8537)	-	(51.4601)	-	(31.7728)
Low Carbon Trade * Quality of Governance * Food Vulnerability	-	-20.8691	-	-2.1975	-	-0.1399
	-	(13.8890)	-	(8.9974)	-	(4.1047)
Constant	0.1199	18.2699**	0.3286	33.1992***	-0.6966	13.2085*
	(1.5903)	(7.6372)	(1.4365)	(10.0257)	(1.3960)	(6.8531)
Wald-chi2	100.8	826.5	87.11	628.8	97.31	2549
Observations	115	115	115	115	115	115
Heteroskedasticity controlled	Yes	Yes	Yes	Yes	Yes	Yes
Autocorrelation controlled	Yes	Yes	Yes	Yes	Yes	Yes
Cross-sectional correlation controlled	Yes	Yes	Yes	Yes	Yes	Yes
Marginal Impact						
Policy Stringency	0.2029	0.0689	0.2199	-0.1693	0.2044	-0.0026
	(0.2053)	(0.1937)	(0.1964)	(0.2301)	(0.1835)	(0.1847)
Environmental Innovation	-0.4351**	-0.5482***	-0.4689***	-0.2085	0.1308	-0.5742***
	(0.1726)	(0.1450)	(0.1652)	(0.1778)	(0.2330)	(0.2141)
Financial Development	3.9066***	3.0284***	3.7846***	1.3486	2.4456*	4.4930***
	(1.2807)	(1.1396)	(1.3241)	(1.5003)	(1.2609)	(1.4716)
Net Energy Import	5.4239***	7.2051***	5.3153***	6.9834***	3.7402***	8.3126***
	(1.0168)	(1.7482)	(1.1297)	(1.9574)	(1.0707)	(1.6547)
Low Carbon Trade	-0.4338***	-0.5882***	-0.3343**	-0.8001***	-0.2460*	-1.0346***
	(0.1632)	(0.1432)	(0.1605)	(0.1936)	(0.1439)	(0.1560)

Note:
Standard errors in parentheses ()
*** p<0.01, ** p<0.05, * p<0.1 by Z-test

Table S87: Long Run Coefficient Estimation using Panel-Corrected Standard Errors with Health Vulnerability for SDG 7.2.1 – With Control of Corruption, Governance Effectiveness, and Political Stability, Related to Figure 3

Variables	Model 1	Model 2	Model 1	Model 2	Model 1	Model 2
	Control of Corruption		Governance Effectiveness		Political Stability	
Policy Stringency	0.2086	-0.1133	0.2280	-1.3317	0.1845	-2.7130
	(0.1842)	(1.7651)	(0.1882)	(1.3942)	(0.1987)	(2.3996)
Environmental Innovation	-0.5914***	1.3489	-0.4648***	-0.5310	-0.4694***	1.7723
	(0.1705)	(1.3024)	(0.1394)	(0.9086)	(0.1496)	(1.3754)
Financial Development	4.6220***	15.9611	4.8358***	-11.6929	4.6015***	28.9109**
	(1.3447)	(13.2431)	(1.3435)	(8.0202)	(1.3835)	(14.2239)
Net Energy Import	6.3175***	-25.8007*	6.1184***	3.9410	5.4618***	-35.0740**
	(1.2668)	(14.8271)	(0.9851)	(5.6356)	(1.0306)	(16.0363)
Low Carbon Trade	-0.3509**	-5.5925**	-0.4171***	-0.6910	-0.4445***	-6.9249***
	(0.1555)	(2.3270)	(0.1570)	(1.1070)	(0.1623)	(2.5734)
Health Vulnerability	1.7156	-12.2823	1.8119	-26.4441*	1.6692	-17.8361
	(1.2170)	(15.0695)	(1.2082)	(15.3061)	(1.4004)	(15.2646)
Quality of Governance	-0.6804	4.1453	-0.9495***	-3.2970	-0.0971	1.5894
	(0.4162)	(3.3329)	(0.3234)	(4.3794)	(0.2526)	(2.9324)
Policy Stringency * Health Vulnerability	-	-1.0562	-	2.7428	-	5.0218
	-	(3.7083)	-	(2.8894)	-	(4.9833)
Environmental Innovation * Health Vulnerability	-	-5.2246*	-	0.1713	-	-6.6753**
	-	(3.0813)	-	(1.9688)	-	(3.3146)
Financial Development * Health Vulnerability	-	-27.0920	-	34.3379*	-	-52.4280*
	-	(29.5972)	-	(17.5472)	-	(31.0443)
Net Energy Import * Health Vulnerability	-	96.7175**	-	9.2795	-	127.3160***
	-	(38.5373)	-	(15.7271)	-	(44.3026)
Low Carbon Trade * Health Vulnerability	-	8.6351*	-	-0.1450	-	11.9792**
	-	(5.1253)	-	(2.3155)	-	(5.3682)
Policy Stringency * Quality of Governance	-	-0.6441	-	-1.8256	-	-2.7036
	-	(3.9554)	-	(2.9088)	-	(3.2791)
Environmental Innovation * Quality of Governance	-	4.3745	-	-1.6646	-	3.2337**
	-	(2.7584)	-	(2.1230)	-	(1.4970)
Financial Development * Quality of Governance	-	28.8364	-	19.5384	-	15.8151
	-	(23.2224)	-	(16.7743)	-	(15.4930)
Net Energy Import * Quality of Governance	-	-65.0390**	-	33.5122	-	-32.9775*
	-	(30.7871)	-	(24.2816)	-	(17.7229)
Low Carbon Trade * Quality of Governance	-	-9.0127**	-	-6.8636	-	-8.3699***
	-	(3.6103)	-	(4.3692)	-	(2.9487)
Policy Stringency * Quality of Governance * Health Vulnerability	-	-1.8347	-	2.0788	-	6.4809
	-	(9.0791)	-	(5.6892)	-	(6.3244)
Environmental Innovation * Quality of Governance * Health Vulnerability	-	-12.0010*	-	10.1602*	-	-9.6470***
	-	(7.0931)	-	(5.4465)	-	(3.2891)
Financial Development * Quality of Governance * Health Vulnerability	-	-94.8052*	-	-90.1227**	-	-35.4053

	-	(55.6051)	-	(38.0481)	-	(31.8535)
Net Energy Import * Quality of Governance * Health Vulnerability	-	194.7286**	-	-104.7005	-	107.1687**
	-	(87.0593)	-	(68.6404)	-	(48.3168)
Low Carbon Trade * Quality of Governance * Health Vulnerability	-	16.3282*	-	13.8350	-	14.9818**
	-	(8.3481)	-	(9.6504)	-	(6.0165)
Constant	-0.1605	5.8651	-0.8701	12.4808*	-0.3005	6.4290
	(1.3999)	(6.6360)	(1.3788)	(7.1241)	(1.4978)	(6.8173)
Wald-chi2	92.36	693.3	110.9	656.7	110.7	503.6
Observations	115	115	115	115	115	115
Heteroskedasticity controlled	Yes	Yes	Yes	Yes	Yes	Yes
Autocorrelation controlled	Yes	Yes	Yes	Yes	Yes	Yes
Cross-sectional correlation controlled	Yes	Yes	Yes	Yes	Yes	Yes
Marginal Impact						
Policy Stringency	0.2086	-0.0997	0.2280	-0.0293	0.1845	-0.6533**
	(0.1842)	(0.2453)	(0.1882)	(0.2253)	(0.1987)	(0.2564)
Environmental Innovation	-0.5914***	-0.7826***	-0.4648***	-0.4075**	-0.4694***	-0.4796***
	(0.1705)	(0.2032)	(0.1394)	(0.1616)	(0.1496)	(0.1837)
Financial Development	4.6220***	7.7284***	4.8358***	3.8600***	4.6015***	5.4350***
	(1.3447)	(1.5651)	(1.3435)	(1.4622)	(1.3835)	(1.5501)
Net Energy Import	6.3175***	12.2694***	6.1184***	7.6357***	5.4618***	13.0551***
	(1.2668)	(2.2221)	(0.9851)	(2.0018)	(1.0306)	(2.1066)
Low Carbon Trade	-0.3509**	-0.9102***	-0.4171***	-0.6493***	-0.4445***	-0.8072***
	(0.1555)	(0.1824)	(0.1570)	(0.1359)	(0.1623)	(0.1854)

Note:
Standard errors in parentheses ()
*** p<0.01, ** p<0.05, * p<0.1 by Z-test

Table S88: Long Run Coefficient Estimation using Panel-Corrected Standard Errors with Health Vulnerability for SDG 7.2.1 – With Regulatory Quality, Rule of Law, and Voice and Accountability, Related to Figure 3

Variables	Model 1	Model 2	Model 1	Model 2	Model 1	Model 2
	Regulatory Quality		Rule of Law		Voice and Accountability	
Policy Stringency	0.1795	-1.2794	0.1625	-1.2811	0.0490	0.7194
	(0.2000)	(1.6820)	(0.2066)	(1.6209)	(0.2107)	(1.0752)
Environmental Innovation	-0.4191**	0.8178	-0.4712***	0.2465	0.1294	-1.5171*
	(0.1733)	(1.1883)	(0.1702)	(1.1234)	(0.2417)	(0.8710)
Financial Development	4.6800***	18.0172**	5.0994***	27.3947***	3.5925***	-6.4234
	(1.3545)	(9.1910)	(1.4446)	(8.6871)	(1.3238)	(5.7929)
Net Energy Import	5.2035***	-10.8341	5.6969***	-6.4095	3.9798***	28.4254***
	(1.0308)	(10.2079)	(1.0699)	(7.8495)	(0.9568)	(6.6712)
Low Carbon Trade	-0.4805***	-7.6044***	-0.5208***	-3.4000*	-0.4331***	-2.0114*
	(0.1640)	(1.9651)	(0.1679)	(1.7495)	(0.1564)	(1.1361)
Health Vulnerability	1.9187	-9.7373	2.2654*	8.0586	-0.4351	-9.5683
	(1.2225)	(14.2587)	(1.3418)	(17.3169)	(1.2948)	(11.5419)
Quality of Governance	0.1364	0.2871	-0.2722	-2.8739	0.9686***	-3.6860**
	(0.3940)	(4.6083)	(0.4922)	(4.9612)	(0.2900)	(1.7214)
Policy Stringency * Health Vulnerability	-	2.1102	-	1.9796	-	-1.4788
	-	(3.4909)	-	(2.9316)	-	(2.0653)
Environmental Innovation * Health Vulnerability	-	-3.5304	-	-1.0643	-	1.2254
	-	(2.8956)	-	(2.2957)	-	(1.7620)
Financial Development * Health Vulnerability	-	-31.5900	-	-47.1887***	-	30.1317**
	-	(20.4791)	-	(16.0241)	-	(14.1010)
Net Energy Import * Health Vulnerability	-	56.8649**	-	29.5200*	-	-31.5460**
	-	(28.3756)	-	(16.2872)	-	(14.8151)
Low Carbon Trade * Health Vulnerability	-	14.9810***	-	4.4183	-	1.1493
	-	(4.2864)	-	(3.6038)	-	(2.3922)
Policy Stringency * Quality of Governance	-	-1.5337	-	0.6597	-	3.3509**
	-	(4.4015)	-	(3.6239)	-	(1.5079)
Environmental Innovation * Quality of Governance	-	2.9958	-	-5.7470***	-	-2.9334***
	-	(2.9142)	-	(1.8754)	-	(0.8268)
Financial Development * Quality of Governance	-	46.4149*	-	52.3725**	-	33.3660***
	-	(26.4851)	-	(23.6984)	-	(11.7159)
Net Energy Import * Quality of Governance	-	-21.4769	-	35.3407*	-	12.9913
	-	(27.3968)	-	(20.1223)	-	(9.5339)
Low Carbon Trade * Quality of Governance	-	-22.2050***	-	-3.1776	-	-2.5660**
	-	(4.6857)	-	(2.5924)	-	(1.2489)
Policy Stringency * Quality of Governance * Health Vulnerability	-	2.9784	-	-1.3552	-	-7.3010**
	-	(8.8940)	-	(7.4485)	-	(3.3937)
Environmental Innovation * Quality of Governance * Health Vulnerability	-	-6.3812	-	17.7881***	-	8.1487***
	-	(6.7395)	-	(4.2859)	-	(2.2741)
Financial Development * Quality of Governance * Health Vulnerability	-	-139.7714**	-	-124.1395**	-	-89.9101***

	-	(57.2880)	-	(60.1797)	-	(28.2945)
Net Energy Import * Quality of Governance * Health Vulnerability	-	81.8118	-	-121.6199**	-	-18.9702
	-	(78.5693)	-	(59.6964)	-	(27.6020)
Low Carbon Trade * Quality of Governance * Health Vulnerability	-	47.2984***	-	1.9649	-	3.0132
	-	(10.2889)	-	(5.4761)	-	(2.6602)
Constant	-0.5851	3.8177	-0.8293	-4.7979	-2.1388	5.0402
	(1.5566)	(6.1615)	(1.4636)	(8.7870)	(1.5405)	(5.7560)
Wald-chi2	114.4	447.6	123.9	550.7	166.0	1849
Observations	115	115	115	115	115	115
Heteroskedasticity controlled	Yes	Yes	Yes	Yes	Yes	Yes
Autocorrelation controlled	Yes	Yes	Yes	Yes	Yes	Yes
Cross-sectional correlation controlled	Yes	Yes	Yes	Yes	Yes	Yes
Marginal Impact						
Policy Stringency	0.1795	-0.2865	0.1625	-0.4015*	0.0490	-0.2337
	(0.2000)	(0.2399)	(0.2066)	(0.2409)	(0.2107)	(0.2107)
Environmental Innovation	-0.4191**	-0.8159***	-0.4712***	-0.5348**	0.1294	-0.8162***
	(0.1733)	(0.2100)	(0.1702)	(0.2084)	(0.2417)	(0.2812)
Financial Development	4.6800***	5.6787***	5.0994***	3.8703**	3.5925***	5.9688***
	(1.3545)	(1.4569)	(1.4446)	(1.5469)	(1.3238)	(1.5554)
Net Energy Import	5.2035***	13.6244***	5.6969***	10.4325***	3.9798***	12.1705***
	(1.0308)	(2.4792)	(1.0699)	(2.2175)	(0.9568)	(1.7544)
Low Carbon Trade	-0.4805***	-0.7054***	-0.5208***	-0.6397***	-0.4331***	-1.1250***
	(0.1640)	(0.1517)	(0.1679)	(0.2329)	(0.1564)	(0.1907)

Note:
Standard errors in parentheses ()
*** p<0.01, ** p<0.05, * p<0.1 by Z-test

Table S89: Long Run Coefficient Estimation using Panel-Corrected Standard Errors with Infrastructure Vulnerability for SDG 7.2.1 – With Control of Corruption, Governance Effectiveness, and Political Stability, Related to Figure 3

Variables	Model 1	Model 2	Model 1	Model 2	Model 1	Model 2
	Control of Corruption		Governance Effectiveness		Political Stability	
Policy Stringency	0.2117	-1.3017	0.2367	-0.5254	0.1764	-1.8058
	(0.1763)	(1.1942)	(0.1785)	(0.7764)	(0.1916)	(1.2407)
Environmental Innovation	-0.6273***	-0.4850	-0.5166***	-0.8057	-0.4889***	1.3892
	(0.1568)	(0.7830)	(0.1285)	(0.6609)	(0.1315)	(0.9427)
Financial Development	5.1107***	-3.0798	5.1482***	-0.9702	5.3727***	-4.3939
	(1.2405)	(6.9748)	(1.2232)	(4.2125)	(1.2615)	(8.1935)
Net Energy Import	4.9476***	4.5062	4.8011***	3.1316	3.8359***	4.2435
	(1.4372)	(6.6829)	(1.1709)	(2.8526)	(1.2540)	(5.4389)
Low Carbon Trade	-0.3015**	-3.9053***	-0.3444**	-0.7914	-0.4195***	-3.4778***
	(0.1522)	(1.3380)	(0.1542)	(0.5835)	(0.1606)	(1.2276)
Infrastructure Vulnerability	9.0681***	-29.9197	8.6206***	-29.5351	10.1748***	44.4406
	(3.3084)	(26.4281)	(3.2129)	(30.1951)	(3.6703)	(32.7582)
Quality of Governance	-0.6226	1.6594	-0.8174**	-3.3914	0.0478	4.8535*
	(0.4118)	(4.3432)	(0.3181)	(3.3058)	(0.2552)	(2.8118)
Policy Stringency * Infrastructure Vulnerability	-	6.9198	-	3.3848	-	13.1169*
	-	(7.7597)	-	(4.4155)	-	(7.7041)
Environmental Innovation * Infrastructure Vulnerability	-	-3.7058	-	0.4865	-	-16.0001**
	-	(5.6270)	-	(3.7481)	-	(6.4919)
Financial Development * Infrastructure Vulnerability	-	56.2432	-	34.8248	-	81.6876
	-	(46.3329)	-	(25.2337)	-	(51.1907)
Net Energy Import * Infrastructure Vulnerability	-	27.4229	-	21.4668	-	23.5968
	-	(51.1004)	-	(25.6954)	-	(53.4977)
Low Carbon Trade * Infrastructure Vulnerability	-	15.5085*	-	1.8259	-	15.2941*
	-	(9.3766)	-	(3.7110)	-	(8.1012)
Policy Stringency * Quality of Governance	-	-3.1506	-	-2.7393	-	-2.0355
	-	(2.4829)	-	(2.1214)	-	(1.8777)
Environmental Innovation * Quality of Governance	-	1.6989	-	1.1400	-	0.7259
	-	(1.0682)	-	(0.9618)	-	(0.7046)
Financial Development * Quality of Governance	-	-10.0926	-	-4.1737	-	-7.7692
	-	(10.8328)	-	(11.9850)	-	(8.9321)
Net Energy Import * Quality of Governance	-	-2.5707	-	0.8621	-	-1.3670
	-	(12.2158)	-	(8.6886)	-	(7.2817)
Low Carbon Trade * Quality of Governance	-	-4.4793**	-	-3.8392*	-	-3.7608***
	-	(1.9090)	-	(2.1254)	-	(1.4402)
Policy Stringency * Quality of Governance * Infrastructure Vulnerability	-	14.1905	-	10.8879	-	13.4560
	-	(18.9888)	-	(11.4135)	-	(10.5236)
Environmental Innovation * Quality of Governance * Infrastructure Vulnerability	-	-11.2965	-	4.8557	-	-12.4316**
	-	(9.9743)	-	(7.2609)	-	(4.8994)
Financial Development * Quality of Governance * Infrastructure Vulnerability	-	35.3224	-	-25.0052	-	72.5268

	-	(84.9989)	-	(74.7002)	-	(52.9773)
Net Energy Import * Quality of Governance * Infrastructure Vulnerability	-	46.7691	-	-82.9226	-	46.6722
	-	(114.1379)	-	(75.9682)	-	(70.6380)
Low Carbon Trade * Quality of Governance * Infrastructure Vulnerability	-	17.7896	-	21.8646*	-	17.7063*
	-	(14.8838)	-	(13.1706)	-	(9.3057)
Constant	-0.1914	8.4874*	-0.6575	6.8049	-0.5678	-4.2370
	(1.0750)	(4.9788)	(1.0410)	(5.5082)	(1.1113)	(6.0478)
Wald-chi2	112.6	871.0	127.9	438.9	144.8	465.3
Observations	115	115	115	115	115	115
Heteroskedasticity controlled	Yes	Yes	Yes	Yes	Yes	Yes
Autocorrelation controlled	Yes	Yes	Yes	Yes	Yes	Yes
Cross-sectional correlation controlled	Yes	Yes	Yes	Yes	Yes	Yes
Marginal Impact						
Policy Stringency	0.2117	0.1936	0.2367	0.0941	0.1764	0.1322
	(0.1763)	(0.2605)	(0.1785)	(0.2523)	(0.1916)	(0.2428)
Environmental Innovation	-0.6273***	-1.1047***	-0.5166***	-0.7182***	-0.4889***	-0.3757*
	(0.1568)	(0.2713)	(0.1285)	(0.2406)	(0.1315)	(0.2270)
Financial Development	5.1107***	7.6532***	5.1482***	4.5149***	5.3727***	6.0165***
	(1.2405)	(1.1544)	(1.2232)	(1.4836)	(1.2615)	(1.3615)
Net Energy Import	4.9476***	7.4368***	4.8011***	6.1661***	3.8359***	4.2935***
	(1.4372)	(1.7942)	(1.1709)	(1.8128)	(1.2540)	(1.5333)
Low Carbon Trade	-0.3015**	-0.7554***	-0.3444**	-0.3584**	-0.4195***	-0.6368***
	(0.1522)	(0.2181)	(0.1542)	(0.1802)	(0.1606)	(0.1622)

Note:
Standard errors in parentheses ()
*** p<0.01, ** p<0.05, * p<0.1 by Z-test

Table S90: Long Run Coefficient Estimation using Panel-Corrected Standard Errors with Infrastructure Vulnerability for SDG 7.2.1 – With Regulatory Quality, Rule of Law, and Voice and Accountability, Related to Figure 3

Variables	Model 1	Model 2	Model 1	Model 2	Model 1	Model 2
	Regulatory Quality		Rule of Law		Voice and Accountability	
Policy Stringency	0.1828	0.1242	0.1896	-0.6344	0.0963	1.0736
	(0.1903)	(0.9124)	(0.1949)	(0.9515)	(0.1967)	(0.8436)
Environmental Innovation	-0.4525***	1.5930*	-0.6014***	0.3428	-0.0315	-1.0476
	(0.1562)	(0.9411)	(0.1689)	(0.8478)	(0.2207)	(0.7800)
Financial Development	5.2731***	-5.4292	5.8521***	10.3103*	4.7708***	0.4119
	(1.2407)	(5.4941)	(1.3056)	(6.1873)	(1.2319)	(3.5574)
Net Energy Import	3.6606***	8.0094*	4.5574***	7.9933	2.8178**	19.3959***
	(1.2525)	(4.5207)	(1.1643)	(5.3751)	(1.1362)	(4.4696)
Low Carbon Trade	-0.4250***	-2.7525**	-0.4386***	-3.2510***	-0.3214**	-1.8568**
	(0.1576)	(1.2716)	(0.1609)	(0.9832)	(0.1494)	(0.7245)
Infrastructure Vulnerability	10.1853***	41.8495	11.8093***	53.1105	6.9708**	-27.3915
	(3.3848)	(28.6952)	(3.5817)	(38.8775)	(3.3458)	(29.2696)
Quality of Governance	0.2445	-13.3815**	-0.6197	-3.0687	0.7617***	1.1549
	(0.3841)	(6.0969)	(0.5002)	(5.1730)	(0.2572)	(1.9000)
Policy Stringency * Infrastructure Vulnerability	-	0.5789	-	3.9563	-	-5.1545
	-	(5.1722)	-	(4.4815)	-	(4.4671)
Environmental Innovation * Infrastructure Vulnerability	-	-13.3204**	-	-5.3668	-	1.9418
	-	(6.3572)	-	(4.4457)	-	(3.9751)
Financial Development * Infrastructure Vulnerability	-	71.5175**	-	-31.1459	-	47.7709**
	-	(34.6051)	-	(30.4198)	-	(22.3476)
Net Energy Import * Infrastructure Vulnerability	-	4.2378	-	-11.9464	-	-40.5771*
	-	(42.7682)	-	(31.9704)	-	(24.2802)
Low Carbon Trade * Infrastructure Vulnerability	-	14.1389	-	13.9249**	-	1.9490
	-	(8.7249)	-	(5.9983)	-	(4.8040)
Policy Stringency * Quality of Governance	-	2.4497	-	-2.3065	-	-2.8430***
	-	(2.5520)	-	(2.0191)	-	(1.0350)
Environmental Innovation * Quality of Governance	-	4.2100***	-	-1.8448*	-	-0.1276
	-	(1.5613)	-	(1.0289)	-	(0.5959)
Financial Development * Quality of Governance	-	-19.5332	-	24.9734**	-	-3.7138
	-	(14.9437)	-	(9.8317)	-	(5.1797)
Net Energy Import * Quality of Governance	-	4.3964	-	17.6419**	-	0.4778
	-	(13.5275)	-	(8.3426)	-	(3.7138)
Low Carbon Trade * Quality of Governance	-	-5.2784*	-	-2.6251*	-	-0.9554
	-	(2.9543)	-	(1.4130)	-	(0.7289)
Policy Stringency * Quality of Governance * Infrastructure Vulnerability	-	-25.1012	-	12.7547	-	14.2001**
	-	(16.7395)	-	(12.7458)	-	(5.8070)
Environmental Innovation * Quality of Governance * Infrastructure Vulnerability	-	-15.8324	-	26.2142***	-	3.1180
	-	(12.7644)	-	(7.3150)	-	(3.6539)
Financial Development * Quality of Governance * Infrastructure Vulnerability	-	88.6660	-	-227.8248***	-	-1.9057

	-	(88.7029)	-	(75.4067)	-	(35.6320)
Net Energy Import * Quality of Governance * Infrastructure Vulnerability	-	51.3696	-	-199.2727***	-	-7.1987
	-	(139.1794)	-	(75.7806)	-	(26.1221)
Low Carbon Trade * Quality of Governance * Infrastructure Vulnerability	-	30.6051	-	6.3318	-	-0.7596
	-	(22.5463)	-	(9.8735)	-	(4.5773)
Constant	-0.7050	-7.1925	-0.7754	-7.2979	-2.5646*	2.9824
	(1.2286)	(5.4639)	(1.0863)	(7.3666)	(1.3749)	(5.5129)
Wald-chi2	142.2	683.4	151.0	702.7	183.0	1152
Observations	115	115	115	115	115	115
Heteroskedasticity controlled	Yes	Yes	Yes	Yes	Yes	Yes
Autocorrelation controlled	Yes	Yes	Yes	Yes	Yes	Yes
Cross-sectional correlation controlled	Yes	Yes	Yes	Yes	Yes	Yes
Marginal Impact						
Policy Stringency	0.1828	0.4829*	0.1896	0.2506	0.0963	0.4755**
	(0.1903)	(0.2714)	(0.1949)	(0.2601)	(0.1967)	(0.2402)
Environmental Innovation	-0.4525***	-0.6611**	-0.6014***	-0.8273***	-0.0315	-0.7902***
	(0.1562)	(0.2818)	(0.1689)	(0.2545)	(0.2207)	(0.2946)
Financial Development	5.2731***	6.2515***	5.8521***	5.4909***	4.7708***	8.8389***
	(1.2407)	(1.4304)	(1.3056)	(1.2984)	(1.2319)	(1.4412)
Net Energy Import	3.6606***	7.0902***	4.5574***	7.4307***	2.8178**	13.0072***
	(1.2525)	(2.0729)	(1.1643)	(1.8179)	(1.1362)	(2.3691)
Low Carbon Trade	-0.4250***	-0.5663***	-0.4386***	-0.4765**	-0.3214**	-1.3314***
	(0.1576)	(0.1933)	(0.1609)	(0.2409)	(0.1494)	(0.2027)

Note:
Standard errors in parentheses ()
*** p<0.01, ** p<0.05, * p<0.1 by Z-test

Table S91: Long Run Coefficient Estimation using Panel-Corrected Standard Errors with Water Vulnerability for SDG 7.2.1 – With Control of Corruption, Governance Effectiveness, and Political Stability, Related to Figure 3

Variables	Model 1	Model 2	Model 1	Model 2	Model 1	Model 2
	Control of Corruption		Governance Effectiveness		Political Stability	
Policy Stringency	0.2687	-1.4068	0.2900	-1.0901	0.2763	-4.4022***
	(0.1844)	(1.4017)	(0.1906)	(0.7609)	(0.1854)	(1.2174)
Environmental Innovation	-0.6014***	-4.5094***	-0.4991***	-3.4692***	-0.5204***	-4.5385***
	(0.1643)	(1.3996)	(0.1370)	(0.9801)	(0.1447)	(1.6100)
Financial Development	3.3333***	42.1585***	3.4101***	14.0218**	3.0797**	46.5052***
	(1.2814)	(10.1295)	(1.2650)	(6.4006)	(1.3055)	(11.3132)
Net Energy Import	6.7224***	14.2860	6.6266***	13.8938**	5.8330***	37.8927***
	(1.3006)	(14.8352)	(1.0102)	(5.6480)	(1.1130)	(13.8969)
Low Carbon Trade	-0.2945*	-1.2547	-0.3657**	0.5530	-0.2611*	-1.4155
	(0.1545)	(1.7671)	(0.1572)	(0.9521)	(0.1525)	(1.5643)
Water Vulnerability	-0.9805	-36.9281**	-0.9093	-44.0050**	-0.9183	-18.5265
	(1.5515)	(16.9154)	(1.4814)	(18.2719)	(1.7834)	(20.6003)
Quality of Governance	-0.6058	-1.9645	-0.8424***	-9.1583**	-0.1520	-0.8684
	(0.4131)	(4.0752)	(0.3099)	(3.9798)	(0.2411)	(2.9606)
Policy Stringency * Water Vulnerability	-	4.8370	-	3.1787	-	15.9589***
	-	(4.4652)	-	(2.2980)	-	(3.6903)
Environmental Innovation * Water Vulnerability	-	11.2461**	-	9.1507***	-	13.6029***
	-	(4.4813)	-	(2.8016)	-	(5.2089)
Financial Development * Water Vulnerability	-	-116.2454***	-	-27.9615	-	-130.5192***
	-	(31.6415)	-	(18.5482)	-	(35.4470)
Net Energy Import * Water Vulnerability	-	-17.0674	-	-27.4232	-	-111.8645**
	-	(50.8804)	-	(19.6981)	-	(50.0003)
Low Carbon Trade * Water Vulnerability	-	0.5242	-	-3.3081	-	2.0759
	-	(5.4150)	-	(2.6282)	-	(4.4357)
Policy Stringency * Quality of Governance	-	-6.7345*	-	-6.2452**	-	-5.2679***
	-	(3.7260)	-	(2.6496)	-	(1.9296)
Environmental Innovation * Quality of Governance	-	-1.9035	-	-1.3656	-	-2.2762*
	-	(2.2422)	-	(1.7347)	-	(1.2299)
Financial Development * Quality of Governance	-	68.3814***	-	49.7865**	-	26.3215**
	-	(23.8860)	-	(19.6719)	-	(13.2035)
Net Energy Import * Quality of Governance	-	4.0702	-	17.1801	-	35.9247**
	-	(35.4542)	-	(21.0898)	-	(16.5257)
Low Carbon Trade * Quality of Governance	-	-1.9995	-	-4.3370	-	-1.7893
	-	(2.9894)	-	(3.2612)	-	(1.9816)
Policy Stringency * Quality of Governance * Water Vulnerability	-	18.4237	-	15.2440**	-	18.5399***
	-	(12.3264)	-	(7.3262)	-	(5.1135)
Environmental Innovation * Quality of Governance * Water Vulnerability	-	7.9793	-	11.5913*	-	8.3997**
	-	(8.5042)	-	(6.7078)	-	(4.2408)
Financial Development * Quality of Governance * Water Vulnerability	-	-246.0851***	-	-180.8089***	-	-86.4333**

	-	(79.9897)	-	(63.8215)	-	(38.9871)
Net Energy Import * Quality of Governance * Water Vulnerability	-	-0.6189	-	-81.1293	-	-123.8993**
	-	(128.6689)	-	(73.4630)	-	(59.0859)
Low Carbon Trade * Quality of Governance * Water Vulnerability	-	3.1096	-	12.2051	-	3.7635
	-	(9.7528)	-	(9.4420)	-	(5.6424)
Constant	1.4167	13.4207**	0.9096	15.3242**	1.4074	5.0716
	(1.1720)	(5.8950)	(1.1250)	(6.2099)	(1.1824)	(6.9874)
Wald-chi2	59.01	901.8	74.87	461.8	56.04	532.0
Observations	115	115	115	115	115	115
Heteroskedasticity controlled	Yes	Yes	Yes	Yes	Yes	Yes
Autocorrelation controlled	Yes	Yes	Yes	Yes	Yes	Yes
Cross-sectional correlation controlled	Yes	Yes	Yes	Yes	Yes	Yes
Marginal Impact						
Policy Stringency	0.2687	0.4106	0.2900	0.1962	0.2763	0.1873
	(0.1844)	(0.2559)	(0.1906)	(0.2513)	(0.1854)	(0.2563)
Environmental Innovation	-0.6014***	-0.8164***	-0.4991***	-0.1287	-0.5204***	-0.2504
	(0.1643)	(0.2250)	(0.1370)	(0.2026)	(0.1447)	(0.2016)
Financial Development	3.3333***	7.0329***	3.4101***	2.4997*	3.0797**	4.1304***
	(1.2814)	(1.3957)	(1.2650)	(1.3615)	(1.3055)	(1.4722)
Net Energy Import	6.7224***	6.8568***	6.6266***	3.4792*	5.8330***	4.9622***
	(1.3006)	(1.6549)	(1.0102)	(1.7774)	(1.1130)	(1.4025)
Low Carbon Trade	-0.2945*	-0.7501***	-0.3657**	-0.5097***	-0.2611*	-0.4964***
	(0.1545)	(0.1791)	(0.1572)	(0.1459)	(0.1525)	(0.1772)

Note:
Standard errors in parentheses ()
*** p<0.01, ** p<0.05, * p<0.1 by Z-test

Table S92: Long Run Coefficient Estimation using Panel-Corrected Standard Errors with Water Vulnerability for SDG 7.2.1 – With Regulatory Quality, Rule of Law, and Voice and Accountability, Related to Figure 3

Variables	Model 1	Model 2	Model 1	Model 2	Model 1	Model 2
	Regulatory Quality		Rule of Law		Voice and Accountability	
Policy Stringency	0.2458	-0.8170	0.2651	-0.0531	0.2026	-0.2178
	(0.2022)	(0.9899)	(0.1911)	(0.8770)	(0.1864)	(0.8633)
Environmental Innovation	-0.4568***	-4.0345***	-0.4543***	-4.2398***	0.1588	-1.4236
	(0.1623)	(1.4336)	(0.1701)	(1.0535)	(0.2375)	(1.0163)
Financial Development	3.3769***	42.5319***	3.0473**	28.9205***	2.4077*	4.2831
	(1.3053)	(7.6166)	(1.3230)	(6.1005)	(1.2536)	(5.5609)
Net Energy Import	5.6847***	22.0820*	5.3669***	6.5593	3.9607***	31.2039***
	(1.0537)	(11.4402)	(1.1295)	(8.6507)	(1.0493)	(6.4203)
Low Carbon Trade	-0.4319***	-0.5781	-0.3074*	-2.5774*	-0.2482*	-0.0163
	(0.1617)	(1.6126)	(0.1581)	(1.3273)	(0.1430)	(1.0330)
Water Vulnerability	-0.3097	-8.4447	-0.9202	-50.5901***	-3.0927**	-12.3232
	(1.5941)	(18.9083)	(1.5348)	(18.8237)	(1.5463)	(16.4441)
Quality of Governance	0.2164	-7.4409	0.2760	1.8704	1.0934***	-1.0996
	(0.4141)	(5.2178)	(0.4275)	(3.6600)	(0.2789)	(1.6056)
Policy Stringency * Water Vulnerability	-	4.1453	-	-0.5239	-	0.8918
	-	(2.9561)	-	(2.3663)	-	(2.1817)
Environmental Innovation * Water Vulnerability	-	10.9029**	-	11.7431***	-	3.9174
	-	(4.6293)	-	(2.9146)	-	(2.4847)
Financial Development * Water Vulnerability	-	-123.9308***	-	-69.1081***	-	2.0788
	-	(24.2994)	-	(17.1623)	-	(16.7303)
Net Energy Import * Water Vulnerability	-	-53.6468	-	-11.4363	-	-54.4943***
	-	(41.0957)	-	(23.5245)	-	(17.0675)
Low Carbon Trade * Water Vulnerability	-	1.1606	-	4.0763	-	-4.2173
	-	(4.8482)	-	(3.7247)	-	(2.9828)
Policy Stringency * Quality of Governance	-	-4.5250	-	-0.1486	-	1.4353
	-	(2.9639)	-	(3.0494)	-	(1.5923)
Environmental Innovation * Quality of Governance	-	-5.2710*	-	-0.3223	-	-1.0227
	-	(3.0600)	-	(1.6445)	-	(0.9425)
Financial Development * Quality of Governance	-	90.5094***	-	16.8480	-	2.3874
	-	(24.5428)	-	(20.8316)	-	(10.4251)
Net Energy Import * Quality of Governance	-	41.4147	-	-32.3903	-	2.1278
	-	(38.7883)	-	(21.4685)	-	(6.8278)
Low Carbon Trade * Quality of Governance	-	-3.4420	-	-3.1848	-	-1.1518
	-	(4.1463)	-	(2.1628)	-	(1.0511)
Policy Stringency * Quality of Governance * Water Vulnerability	-	13.0007	-	-5.3378	-	-4.3602
	-	(8.6259)	-	(8.6476)	-	(4.0424)
Environmental Innovation * Quality of Governance * Water Vulnerability	-	23.3448**	-	1.4529	-	3.0576
	-	(11.2896)	-	(5.2843)	-	(2.7116)
Financial Development * Quality of Governance * Water Vulnerability	-	-324.1357***	-	-44.9242	-	-6.3569

	-	(77.3729)	-	(67.7906)	-	(30.2249)
Net Energy Import * Quality of Governance * Water Vulnerability	-	-146.8687	-	90.0779	-	5.5995
	-	(140.9754)	-	(76.0300)	-	(21.6051)
Low Carbon Trade * Quality of Governance * Water Vulnerability	-	10.9887	-	5.0249	-	-0.0916
	-	(13.3633)	-	(6.1809)	-	(2.8337)
Constant	1.0125	4.2594	1.3954	17.5480***	-1.2317	-0.0372
	(1.3488)	(6.6267)	(1.1397)	(6.5196)	(1.3245)	(6.6466)
Wald-chi2	82.12	620.5	65.17	528.8	102.4	1125
Observations	115	115	115	115	115	115
Heteroskedasticity controlled	Yes	Yes	Yes	Yes	Yes	Yes
Autocorrelation controlled	Yes	Yes	Yes	Yes	Yes	Yes
Cross-sectional correlation controlled	Yes	Yes	Yes	Yes	Yes	Yes
Marginal Impact						
Policy Stringency	0.2458	0.5093**	0.2651	0.2677	0.2026	0.1245
	(0.2022)	(0.2588)	(0.1911)	(0.2676)	(0.1864)	(0.2470)
Environmental Innovation	-0.4568***	-0.7389***	-0.4543***	-0.0892	0.1588	-0.0418
	(0.1623)	(0.2246)	(0.1701)	(0.2265)	(0.2375)	(0.2974)
Financial Development	3.3769***	4.6205***	3.0473**	3.3427**	2.4077*	4.9993***
	(1.3053)	(1.4229)	(1.3230)	(1.5020)	(1.2536)	(1.5463)
Net Energy Import	5.6847***	5.7523***	5.3669***	3.9985**	3.9607***	10.9444***
	(1.0537)	(1.7384)	(1.1295)	(1.7890)	(1.0493)	(2.0093)
Low Carbon Trade	-0.4319***	-0.3346*	-0.3074*	-0.6428***	-0.2482*	-1.2766***
	(0.1617)	(0.1835)	(0.1581)	(0.1956)	(0.1430)	(0.1793)

Note:
Standard errors in parentheses ()
*** p<0.01, ** p<0.05, * p<0.1 by Z-test

Table S93: Long Run Coefficient Estimation using Panel-Corrected Standard Errors with Ecosystem Vulnerability for SDG 7.3.1 – With Control of Corruption, Governance Effectiveness, and Political Stability, Related to Figure 3

Variables	Model 1	Model 2	Model 1	Model 2	Model 1	Model 2
	Control of Corruption		Governance Effectiveness		Political Stability	
Policy Stringency	-0.0189	1.6132***	-0.0110	2.0113***	-0.0219	1.1057
	(0.0250)	(0.6194)	(0.0286)	(0.4332)	(0.0265)	(0.7150)
Environmental Innovation	-0.0208	3.2632***	-0.0449	1.5014***	-0.0471	1.0408
	(0.0379)	(0.6520)	(0.0418)	(0.5065)	(0.0382)	(0.8648)
Foreign Direct Investment	-0.0596	3.3335*	-0.0960*	0.3266	-0.0883*	-0.8717
	(0.0489)	(1.9002)	(0.0576)	(1.1859)	(0.0535)	(2.0904)
Industrial Share	0.8841***	3.2463**	1.2217***	4.3274***	1.0496***	2.4133
	(0.1441)	(1.5107)	(0.1661)	(1.2662)	(0.1577)	(1.8737)
Energy Demand	-0.0991	-5.9280***	-0.0975	-1.4066**	-0.0686	0.0429
	(0.0695)	(0.9089)	(0.0679)	(0.6723)	(0.0658)	(1.3407)
Ecosystem Vulnerability	0.6619	-9.6776	-0.0801	30.6573***	0.1293	29.5665***
	(0.4852)	(9.6743)	(0.4578)	(6.7654)	(0.4730)	(9.3787)
Quality of Governance	-0.0953**	0.6990	-0.0260	4.6665***	-0.0358	1.0191
	(0.0482)	(1.4888)	(0.0473)	(1.0590)	(0.0342)	(0.8161)
Policy Stringency * Ecosystem Vulnerability	-	-3.0538**		-4.0068***	-	-2.1611
	-	(1.2723)		(0.8673)	-	(1.4759)
Environmental Innovation * Ecosystem Vulnerability	-	-6.1724***		-3.0780***	-	-1.9401
	-	(1.3052)		(0.9827)	-	(1.7528)
Foreign Direct Investment * Ecosystem Vulnerability	-	-6.7234*		-0.6391	-	1.8148
	-	(3.8242)		(2.4316)	-	(4.2591)
Industrial Share * Ecosystem Vulnerability	-	-3.9805		-6.9438***	-	-2.5019
	-	(2.8678)		(2.5239)	-	(3.9884)
Energy Demand * Ecosystem Vulnerability	-	10.9276***		2.8557**	-	-0.6559
	-	(1.7654)		(1.3091)	-	(2.7263)
Policy Stringency * Quality of Governance	-	1.1300		3.8535***	-	0.4217
	-	(1.2860)		(1.4673)	-	(0.9606)
Environmental Innovation * Quality of Governance	-	3.1076*		6.0104***	-	1.3408
	-	(1.6671)		(2.0643)	-	(1.0987)
Foreign Direct Investment * Quality of Governance	-	4.6668*		7.6061	-	-1.0822
	-	(2.8193)		(4.6733)	-	(2.7008)
Industrial Share * Quality of Governance	-	0.5641		8.1532**	-	-0.6696
	-	(2.8123)		(3.6427)	-	(2.2246)
Energy Demand * Quality of Governance	-	-4.1826**		-12.1875***	-	-1.3778
	-	(2.0063)		(2.5384)	-	(1.6165)
Policy Stringency * Quality of Governance * Ecosystem Vulnerability	-	-1.8991		-7.0943**	-	-0.7374
	-	(2.6762)		(2.9334)	-	(1.9423)
Environmental Innovation * Quality of Governance * Ecosystem Vulnerability	-	-5.1117		-11.4786***	-	-2.1595
	-	(3.3880)		(4.1568)	-	(2.1629)
Foreign Direct Investment * Quality of Governance * Ecosystem Vulnerability	-	-9.1323		-15.0233	-	2.3172

	-	(5.8242)		(9.3604)	-	(5.4521)
Industrial Share * Quality of Governance * Ecosystem Vulnerability	-	-0.5253		-17.9286**	-	1.6267
	-	(5.6654)		(7.3123)	-	(4.6178)
Energy Demand * Quality of Governance * Ecosystem Vulnerability	-	6.5071		23.1463***	-	1.7326
	-	(4.0173)		(5.1589)	-	(3.2059)
Constant	-0.5870*	4.1291	-1.1618***	-16.1357***	-0.8728***	-15.3446***
	(0.3323)	(5.1515)	(0.3140)	(3.3994)	(0.3323)	(4.5547)
Wald-chi2	74.59	1822	130.6	938.7	100.9	982.6
Observations	115	115	115	115	115	115
Heteroskedasticity controlled	Yes	Yes	Yes	Yes	Yes	Yes
Autocorrelation controlled	Yes	Yes	Yes	Yes	Yes	Yes
Cross-sectional correlation controlled	Yes	Yes	Yes	Yes	Yes	Yes
Marginal Impact						
Policy Stringency	-0.0189	0.0493	-0.0110	0.0115	-0.0219	0.0260
	(0.0250)	(0.0331)	(0.0286)	(0.0312)	(0.0265)	(0.0347)
Environmental Innovation	-0.0208	0.0236	-0.0449	-0.0807	-0.0471	-0.0517
	(0.0379)	(0.0518)	(0.0418)	(0.0624)	(0.0382)	(0.0499)
Foreign Direct Investment	-0.0596	-0.0573	-0.0960*	-0.0975	-0.0883*	-0.0279
	(0.0489)	(0.0761)	(0.0576)	(0.0759)	(0.0535)	(0.0648)
Industrial Share	0.8841***	1.2050***	1.2217***	0.8338***	1.0496***	1.1242***
	(0.1441)	(0.1178)	(0.1661)	(0.1321)	(0.1577)	(0.1241)
Energy Demand	-0.0991	-0.2358***	-0.0975	0.1592*	-0.0686	0.0152
	(0.0695)	(0.0889)	(0.0679)	(0.0933)	(0.0658)	(0.0708)

Note:
Standard errors in parentheses ()
*** p<0.01, ** p<0.05, * p<0.1 by Z-test

Table S94: Long Run Coefficient Estimation using Panel-Corrected Standard Errors with Ecosystem Vulnerability for SDG 7.3.1 – With Regulatory Quality, Rule of Law, and Voice and Accountability, Related to Figure 3

Variables	Model 1	Model 2	Model 1	Model 2	Model 1	Model 2
	Regulatory Quality		Rule of Law		Voice and Accountability	
Policy Stringency	-0.0041	0.9075*	-0.0140	1.6411***	0.0176	0.2847
	(0.0298)	(0.5057)	(0.0264)	(0.4002)	(0.0268)	(0.4131)
Environmental Innovation	-0.0470	0.7579	-0.0205	3.4096***	0.0064	0.4952
	(0.0442)	(0.5846)	(0.0391)	(0.4514)	(0.0331)	(0.5834)
Foreign Direct Investment	-0.1074*	-2.3840*	-0.0541	2.1316	-0.0712	-1.4288
	(0.0614)	(1.3435)	(0.0527)	(1.4129)	(0.0537)	(1.0319)
Industrial Share	1.3372***	4.2262***	0.9235***	5.6751***	0.7838***	3.8517***
	(0.1694)	(1.4371)	(0.1470)	(1.0896)	(0.1433)	(1.4609)
Energy Demand	-0.1251	-0.0834	-0.1151*	-3.6291***	-0.3900***	1.5754
	(0.0787)	(0.9341)	(0.0699)	(0.7725)	(0.0797)	(1.3171)
Ecosystem Vulnerability	-0.3437	32.3788***	1.0422**	40.4929***	1.3439***	52.5982***
	(0.4671)	(9.0679)	(0.5063)	(10.2579)	(0.3804)	(16.8110)
Quality of Governance	-0.0447	1.2074	-0.1809***	2.1708*	-0.3607***	-0.1223
	(0.0658)	(1.2193)	(0.0572)	(1.1080)	(0.0640)	(0.5962)
Policy Stringency * Ecosystem Vulnerability	-	-1.8894*	-	-3.1897***	-	-0.5922
	-	(1.0236)	-	(0.8088)	-	(0.8198)
Environmental Innovation * Ecosystem Vulnerability	-	-1.5677	-	-6.3861***	-	-0.9426
	-	(1.1608)	-	(0.8624)	-	(1.1213)
Foreign Direct Investment * Ecosystem Vulnerability	-	4.7259*	-	-4.3891	-	2.7676
	-	(2.7141)	-	(2.8338)	-	(2.0982)
Industrial Share * Ecosystem Vulnerability	-	-6.4730**	-	-9.9691***	-	-6.4884**
	-	(2.9493)	-	(2.1593)	-	(2.9917)
Energy Demand * Ecosystem Vulnerability	-	0.1067	-	6.4598***	-	-3.5673
	-	(1.8485)	-	(1.4764)	-	(2.5758)
Policy Stringency * Quality of Governance	-	3.2650	-	2.6018***	-	1.9848***
	-	(2.0289)	-	(0.9748)	-	(0.7007)
Environmental Innovation * Quality of Governance	-	-1.6281	-	4.2808***	-	1.8028*
	-	(2.1582)	-	(1.2700)	-	(1.0080)
Foreign Direct Investment * Quality of Governance	-	-5.1746	-	2.8057	-	-1.1650
	-	(3.1930)	-	(2.5050)	-	(1.1815)
Industrial Share * Quality of Governance	-	5.4360	-	10.3441***	-	3.8614*
	-	(4.0453)	-	(2.3701)	-	(2.0099)
Energy Demand * Quality of Governance	-	-1.4721	-	-10.3349***	-	-3.8897**
	-	(2.3295)	-	(1.7601)	-	(1.5894)
Policy Stringency * Quality of Governance * Ecosystem Vulnerability	-	-6.3855	-	-4.9378**	-	-3.8954***
	-	(4.0895)	-	(1.9960)	-	(1.3854)
Environmental Innovation * Quality of Governance * Ecosystem Vulnerability	-	3.4840	-	-7.5007***	-	-3.2760*
	-	(4.2848)	-	(2.5416)	-	(1.9394)
Foreign Direct Investment * Quality of Governance * Ecosystem Vulnerability	-	10.3264	-	-5.2671	-	2.4196

	-	(6.3939)	-	(5.3543)	-	(2.4132)
Industrial Share * Quality of Governance * Ecosystem Vulnerability	-	-11.1068	-	-21.1957***	-	-7.5098*
	-	(8.2979)	-	(5.0070)	-	(3.9474)
Energy Demand * Quality of Governance * Ecosystem Vulnerability	-	2.4402	-	18.9844***	-	7.2692**
	-	(4.6549)	-	(3.5745)	-	(3.0598)
Constant	-1.2303***	-17.1132***	-0.8273***	-19.6009***	1.0944**	-25.1301***
	(0.3347)	(4.5065)	(0.3164)	(5.2894)	(0.4810)	(8.3318)
Wald-chi2	156.2	491.1	106.6	2589	250.4	725.8
Observations	115	115	115	115	115	115
Heteroskedasticity controlled	Yes	Yes	Yes	Yes	Yes	Yes
Autocorrelation controlled	Yes	Yes	Yes	Yes	Yes	Yes
Cross-sectional correlation controlled	Yes	Yes	Yes	Yes	Yes	Yes
Marginal Impact						
Policy Stringency	-0.0041	-0.0187	-0.0140	-0.0142	0.0176	-0.0308
	(0.0298)	(0.0325)	(0.0264)	(0.0305)	(0.0268)	(0.0341)
Environmental Innovation	-0.0470	-0.0141	-0.0205	0.0531	0.0064	-0.0131
	(0.0442)	(0.0582)	(0.0391)	(0.0499)	(0.0331)	(0.0653)
Foreign Direct Investment	-0.1074*	-0.0878	-0.0541	-0.1214*	-0.0712	-0.0830
	(0.0614)	(0.0636)	(0.0527)	(0.0723)	(0.0537)	(0.0674)
Industrial Share	1.3372***	1.0969***	0.9235***	0.6132***	0.7838***	0.6434***
	(0.1694)	(0.1241)	(0.1470)	(0.1092)	(0.1433)	(0.1514)
Energy Demand	-0.1251	-0.0028	-0.1151*	0.0161	-0.3900***	-0.0637
	(0.0787)	(0.0887)	(0.0699)	(0.0765)	(0.0797)	(0.1219)
Note:<br>Standard errors in parentheses ()<br>*** p<0.01, ** p<0.05, * p<0.1 by Z-test						

Table S95: Long Run Coefficient Estimation using Panel-Corrected Standard Errors with Food Vulnerability for SDG 7.3.1 – With Control of Corruption, Governance Effectiveness, and Political Stability, Related to Figure 3

Variables	Model 1	Model 2	Model 1	Model 2	Model 1	Model 2
	Control of Corruption		Governance Effectiveness		Political Stability	
Policy Stringency	0.0120	-0.0146	0.0139	0.2263	0.0035	-0.7465
	(0.0281)	(0.4171)	(0.0294)	(0.1838)	(0.0278)	(0.6033)
Environmental Innovation	0.0245	1.0249**	-0.0197	-0.1102	-0.0165	0.1056
	(0.0427)	(0.4570)	(0.0429)	(0.2742)	(0.0402)	(0.6371)
Foreign Direct Investment	-0.0776	0.5323	-0.1073*	-0.3835	-0.1072**	-2.3811
	(0.0533)	(0.9437)	(0.0574)	(0.5069)	(0.0545)	(1.7236)
Industrial Share	1.1733***	3.4225***	1.3061***	3.9188***	1.1806***	1.1920
	(0.1419)	(0.9878)	(0.1486)	(0.7238)	(0.1443)	(1.4502)
Energy Demand	-0.2317***	-1.5854**	-0.1689**	0.5747	-0.1564**	1.6501*
	(0.0786)	(0.6866)	(0.0685)	(0.4870)	(0.0691)	(0.9907)
Food Vulnerability	-0.7416***	12.8781**	-0.7417***	30.9262***	-0.8417***	25.9144***
	(0.2126)	(5.6040)	(0.1888)	(7.2140)	(0.2284)	(8.3355)
Quality of Governance	-0.1408***	-1.0994	-0.0268	2.8355***	-0.0632*	-0.1890
	(0.0533)	(0.8785)	(0.0493)	(1.0599)	(0.0381)	(0.7553)
Policy Stringency * Food Vulnerability	-	0.1918	-	-0.4765	-	1.7652
	-	(0.9408)	-	(0.3975)	-	(1.3485)
Environmental Innovation * Food Vulnerability	-	-2.0681**	-	0.0534	-	-0.3238
	-	(1.0393)	-	(0.6366)	-	(1.5217)
Foreign Direct Investment * Food Vulnerability	-	-1.2772	-	0.7770	-	5.0487
	-	(2.0657)	-	(1.1335)	-	(3.8149)
Industrial Share * Food Vulnerability	-	-4.2243*	-	-6.3878***	-	0.8345
	-	(2.2399)	-	(1.5976)	-	(3.3977)
Energy Demand * Food Vulnerability	-	2.5102*	-	-1.4254	-	-4.4062*
	-	(1.5175)	-	(1.0897)	-	(2.2638)
Policy Stringency * Quality of Governance	-	-0.4736	-	0.9767	-	-0.7580
	-	(1.0223)	-	(0.7180)	-	(0.7074)
Environmental Innovation * Quality of Governance	-	1.5768	-	2.3980**	-	0.9820
	-	(1.0473)	-	(1.1319)	-	(0.7175)
Foreign Direct Investment * Quality of Governance	-	1.6836	-	4.8961**	-	-2.4085
	-	(1.8568)	-	(2.4350)	-	(1.8801)
Industrial Share * Quality of Governance	-	0.0734	-	3.3259*	-	-1.8405
	-	(2.3483)	-	(1.8498)	-	(1.5033)
Energy Demand * Quality of Governance	-	-1.6752	-	-5.1900***	-	0.0591
	-	(1.6511)	-	(1.6913)	-	(1.0487)
Policy Stringency * Quality of Governance * Food Vulnerability	-	1.7194	-	-1.7133	-	1.8658
	-	(2.3907)	-	(1.6149)	-	(1.5336)
Environmental Innovation * Quality of Governance * Food Vulnerability	-	-3.0680	-	-4.9766*	-	-1.7160
	-	(2.4746)	-	(2.8336)	-	(1.6624)
Foreign Direct Investment * Quality of Governance * Food Vulnerability	-	-3.9516	-	-10.9568**	-	4.9432

	-	(4.3776)	-	(5.4753)	-	(4.0040)
Industrial Share * Quality of Governance * Food Vulnerability	-	2.0020	-	-7.7646*	-	5.6297*
	-	(5.5355)	-	(4.1120)	-	(3.3677)
Energy Demand * Quality of Governance * Food Vulnerability	-	2.3531	-	10.3422**	-	-1.3793
	-	(3.7025)	-	(4.1153)	-	(2.3656)
Constant	-0.3977	-6.8441***	-0.8864***	-14.5385***	-0.5508	-12.6252***
	(0.3225)	(2.4959)	(0.3366)	(3.0739)	(0.3479)	(3.5764)
Wald-chi2	121.3	1340	169.5	696.2	136.0	871.8
Observations	115	115	115	115	115	115
Heteroskedasticity controlled	Yes	Yes	Yes	Yes	Yes	Yes
Autocorrelation controlled	Yes	Yes	Yes	Yes	Yes	Yes
Cross-sectional correlation controlled	Yes	Yes	Yes	Yes	Yes	Yes
Marginal Impact						
Policy Stringency	0.0120	-0.0186	0.0139	0.0012	0.0035	-0.0315
	(0.0281)	(0.0266)	(0.0294)	(0.0321)	(0.0278)	(0.0331)
Environmental Innovation	0.0245	0.0099	-0.0197	-0.1209*	-0.0165	-0.1342***
	(0.0427)	(0.0470)	(0.0429)	(0.0692)	(0.0402)	(0.0418)
Foreign Direct Investment	-0.0776	-0.0380	-0.1073*	-0.1103*	-0.1072**	-0.0981
	(0.0533)	(0.0689)	(0.0574)	(0.0639)	(0.0545)	(0.0714)
Industrial Share	1.1733***	1.2252***	1.3061***	1.0344***	1.1806***	1.1064***
	(0.1419)	(0.1001)	(0.1486)	(0.1366)	(0.1443)	(0.1256)
Energy Demand	-0.2317***	-0.2338***	-0.1689**	0.0159	-0.1564**	0.0249
	(0.0786)	(0.0865)	(0.0685)	(0.1086)	(0.0691)	(0.0699)

Note:
Standard errors in parentheses ()
*** p<0.01, ** p<0.05, * p<0.1 by Z-test

Table S96: Long Run Coefficient Estimation using Panel-Corrected Standard Errors with Food Vulnerability for SDG 7.3.1 – With Regulatory Quality, Rule of Law, and Voice and Accountability, Related to Figure 3

Variables	Model 1	Model 2	Model 1	Model 2	Model 1	Model 2
	Regulatory Quality		Rule of Law		Voice and Accountability	
Policy Stringency	0.0130	-0.2159	0.0208	-0.0634	0.0289	0.3154
	(0.0290)	(0.4080)	(0.0300)	(0.2304)	(0.0274)	(0.2297)
Environmental Innovation	-0.0121	-0.1290	0.0087	1.2403***	0.0242	0.1479
	(0.0430)	(0.3213)	(0.0416)	(0.2965)	(0.0358)	(0.3956)
Foreign Direct Investment	-0.1085*	-2.8350**	-0.0839	-0.4942	-0.0767	-0.1867
	(0.0583)	(1.1743)	(0.0576)	(0.5656)	(0.0565)	(0.5625)
Industrial Share	1.3040***	3.3337***	1.2660***	1.2735*	0.9638***	3.6691***
	(0.1463)	(0.9593)	(0.1422)	(0.7188)	(0.1374)	(0.9775)
Energy Demand	-0.1952**	0.9906	-0.2228***	-1.1738*	-0.4016***	0.2157
	(0.0774)	(0.6897)	(0.0718)	(0.6117)	(0.0829)	(0.8294)
Food Vulnerability	-0.7971***	29.5073***	-0.4988**	12.5126	0.1138	30.8108***
	(0.2093)	(7.1854)	(0.2027)	(7.8924)	(0.2338)	(8.0289)
Quality of Governance	-0.0718	-1.1621	-0.1537***	5.3038***	-0.3118***	-1.0399
	(0.0640)	(0.9774)	(0.0567)	(1.1425)	(0.0741)	(0.6402)
Policy Stringency * Food Vulnerability	-	0.4770	-	0.2723	-	-0.6634
	-	(0.9030)	-	(0.4927)	-	(0.5527)
Environmental Innovation * Food Vulnerability	-	0.1098	-	-2.2851***	-	-0.1004
	-	(0.7064)	-	(0.5888)	-	(0.9105)
Foreign Direct Investment * Food Vulnerability	-	6.2045**	-	1.0350	-	-0.1731
	-	(2.6103)	-	(1.2777)	-	(1.4540)
Industrial Share * Food Vulnerability	-	-4.9375**	-	-1.4335	-	-6.4650***
	-	(2.1472)	-	(1.5737)	-	(2.3332)
Energy Demand * Food Vulnerability	-	-2.4578	-	1.3808	-	-1.1613
	-	(1.5260)	-	(1.3050)	-	(1.9194)
Policy Stringency * Quality of Governance	-	0.0479	-	0.1358	-	0.0399
	-	(1.2953)	-	(1.1906)	-	(0.6312)
Environmental Innovation * Quality of Governance	-	0.7311	-	2.9282***	-	0.5920
	-	(0.9504)	-	(0.9186)	-	(0.8822)
Foreign Direct Investment * Quality of Governance	-	-5.6305*	-	0.9431	-	-2.4573
	-	(2.9693)	-	(2.3261)	-	(1.5479)
Industrial Share * Quality of Governance	-	-0.9938	-	5.8358**	-	0.1723
	-	(3.0568)	-	(2.4870)	-	(1.9932)
Energy Demand * Quality of Governance	-	0.0344	-	-6.9317***	-	-0.4059
	-	(1.9355)	-	(1.4605)	-	(1.6547)
Policy Stringency * Quality of Governance * Food Vulnerability	-	0.2503	-	0.3230	-	0.1311
	-	(2.8298)	-	(2.8815)	-	(1.5339)
Environmental Innovation * Quality of Governance * Food Vulnerability	-	-1.3814	-	-5.2235**	-	-1.0176
	-	(2.1490)	-	(2.2123)	-	(2.0787)
Foreign Direct Investment * Quality of Governance * Food Vulnerability	-	12.0689*	-	-1.7617	-	6.2160

	-	(6.4755)	-	(6.1402)	-	(3.9110)
Industrial Share * Quality of Governance * Food Vulnerability	-	3.8079	-	-15.2147**	-	0.5339
	-	(6.6855)	-	(6.2494)	-	(4.9855)
Energy Demand * Quality of Governance * Food Vulnerability	-	-0.8112	-	13.1622***	-	0.3170
	-	(4.2829)	-	(3.4016)	-	(3.8880)
Constant	-0.7408**	-13.7947***	-0.7661**	-3.9710	1.0369**	-13.1001***
	(0.3558)	(3.0798)	(0.3212)	(3.3972)	(0.5149)	(3.6071)
Wald-chi2	169.5	571.9	177.7	1083	236.6	571.2
Observations	115	115	115	115	115	115
Heteroskedasticity controlled	Yes	Yes	Yes	Yes	Yes	Yes
Autocorrelation controlled	Yes	Yes	Yes	Yes	Yes	Yes
Cross-sectional correlation controlled	Yes	Yes	Yes	Yes	Yes	Yes
Marginal Impact						
Policy Stringency	0.0130	-0.0222	0.0208	-0.0166	0.0289	0.0056
	(0.0290)	(0.0271)	(0.0300)	(0.0354)	(0.0274)	(0.0293)
Environmental Innovation	-0.0121	-0.0918*	0.0087	-0.0557	0.0242	0.0378
	(0.0430)	(0.0506)	(0.0416)	(0.0462)	(0.0358)	(0.0638)
Foreign Direct Investment	-0.1085*	-0.0651	-0.0839	-0.1170	-0.0767	-0.1006
	(0.0583)	(0.0604)	(0.0576)	(0.0878)	(0.0565)	(0.0864)
Industrial Share	1.3040***	1.0599***	1.2660***	0.5944***	0.9638***	0.7353***
	(0.1463)	(0.1161)	(0.1422)	(0.1209)	(0.1374)	(0.1550)
Energy Demand	-0.1952**	-0.0630	-0.2228***	0.0198	-0.4016***	-0.2340*
	(0.0774)	(0.0824)	(0.0718)	(0.0771)	(0.0829)	(0.1283)

Note:
Standard errors in parentheses ()
*** p<0.01, ** p<0.05, * p<0.1 by Z-test

Table S97: Long Run Coefficient Estimation using Panel-Corrected Standard Errors with Health Vulnerability for SDG 7.3.1 – With Control of Corruption, Governance Effectiveness, and Political Stability, Related to Figure 3

Variables	Model 1	Model 2	Model 1	Model 2	Model 1	Model 2
	Control of Corruption		Governance Effectiveness		Political Stability	
Policy Stringency	0.0093	0.3549	0.0108	0.3422*	-0.0021	-0.3619
	(0.0272)	(0.3472)	(0.0283)	(0.2078)	(0.0270)	(0.5371)
Environmental Innovation	0.0258	0.2573	-0.0132	-0.0596	-0.0153	-0.5073
	(0.0419)	(0.3634)	(0.0420)	(0.2083)	(0.0395)	(0.4203)
Foreign Direct Investment	-0.0729	1.4282*	-0.0980*	0.5501	-0.0972*	-1.9804
	(0.0534)	(0.8676)	(0.0558)	(0.3824)	(0.0531)	(1.5286)
Industrial Share	1.2511***	0.9421	1.3466***	1.3005*	1.2236***	-0.7792
	(0.1379)	(0.7115)	(0.1424)	(0.7004)	(0.1394)	(1.1336)
Energy Demand	-0.2401***	-0.6984	-0.1791***	-0.1829	-0.1570**	1.1635**
	(0.0745)	(0.5230)	(0.0664)	(0.3072)	(0.0666)	(0.5618)
Health Vulnerability	-0.7003***	-4.9847	-0.7535***	-1.0721	-0.7737***	-9.1540**
	(0.1593)	(4.6079)	(0.1457)	(5.0124)	(0.1688)	(4.1843)
Quality of Governance	-0.1390***	-0.9141	-0.0293	3.4596***	-0.0605*	0.7189
	(0.0520)	(1.1532)	(0.0494)	(1.1005)	(0.0365)	(0.7687)
Policy Stringency * Health Vulnerability	-	-0.5222	-	-0.6073	-	1.0199
	-	(0.7383)	-	(0.4320)	-	(1.1315)
Environmental Innovation * Health Vulnerability	-	-0.2650	-	0.0022	-	1.5760*
	-	(0.7656)	-	(0.4359)	-	(0.9362)
Foreign Direct Investment * Health Vulnerability	-	-3.4575*	-	-1.5766*	-	4.4155
	-	(1.9504)	-	(0.8808)	-	(3.3508)
Industrial Share * Health Vulnerability	-	1.8166	-	0.1782	-	6.1944**
	-	(1.4806)	-	(1.4740)	-	(2.5488)
Energy Demand * Health Vulnerability	-	0.3430	-	0.1827	-	-4.0902***
	-	(1.1061)	-	(0.6262)	-	(1.2537)
Policy Stringency * Quality of Governance	-	0.3139	-	1.4976**	-	-0.3345
	-	(0.7593)	-	(0.6522)	-	(0.6643)
Environmental Innovation * Quality of Governance	-	-0.2276	-	0.7591	-	-0.0825
	-	(0.6718)	-	(0.6980)	-	(0.4904)
Foreign Direct Investment * Quality of Governance	-	2.1219	-	1.5580	-	-2.2014
	-	(1.7026)	-	(2.1127)	-	(1.6460)
Industrial Share * Quality of Governance	-	-2.0910*	-	2.4842**	-	-1.1606
	-	(1.2458)	-	(1.1951)	-	(1.1168)
Energy Demand * Quality of Governance	-	1.2128	-	-2.9706***	-	0.7898
	-	(0.9465)	-	(1.1433)	-	(0.7138)
Policy Stringency * Quality of Governance * Health Vulnerability	-	-0.2458	-	-3.0030**	-	0.8492
	-	(1.6796)	-	(1.3966)	-	(1.3537)
Environmental Innovation * Quality of Governance * Health Vulnerability	-	1.2897	-	-1.2566	-	1.4064
	-	(1.5587)	-	(1.7708)	-	(1.0144)
Foreign Direct Investment * Quality of Governance * Health Vulnerability	-	-4.8523	-	-2.9679	-	5.0763

	-	(4.1949)	-	(4.9481)	-	(3.4181)
Industrial Share * Quality of Governance * Health Vulnerability	-	6.4971**	-	-6.7695**	-	3.6989
	-	(3.1025)	-	(2.8091)	-	(2.3123)
Energy Demand * Quality of Governance * Health Vulnerability	-	-4.3540**	-	5.5577**	-	-3.9663***
	-	(2.1302)	-	(2.7993)	-	(1.4731)
Constant	-0.6142**	0.6216	-0.9803***	-1.2215	-0.7092**	2.0772
	(0.2910)	(2.4710)	(0.3085)	(2.3487)	(0.3112)	(2.0365)
Wald-chi2	144.3	1454	188.1	581.0	151.1	1508
Observations	115	115	115	115	115	115
Heteroskedasticity controlled	Yes	Yes	Yes	Yes	Yes	Yes
Autocorrelation controlled	Yes	Yes	Yes	Yes	Yes	Yes
Cross-sectional correlation controlled	Yes	Yes	Yes	Yes	Yes	Yes
Marginal Impact						
Policy Stringency	0.0093	0.0394	0.0108	0.0356	-0.0021	0.0655*
	(0.0272)	(0.0298)	(0.0283)	(0.0332)	(0.0270)	(0.0352)
Environmental Innovation	0.0258	0.0166	-0.0132	-0.0700	-0.0153	-0.1155***
	(0.0419)	(0.0474)	(0.0420)	(0.0550)	(0.0395)	(0.0374)
Foreign Direct Investment	-0.0729	-0.1837**	-0.0980*	-0.2085***	-0.0972*	-0.0958
	(0.0534)	(0.0751)	(0.0558)	(0.0795)	(0.0531)	(0.0729)
Industrial Share	1.2511***	1.5278***	1.3466***	1.3395***	1.2236***	1.7328***
	(0.1379)	(0.1126)	(0.1424)	(0.1344)	(0.1394)	(0.1072)
Energy Demand	-0.2401***	-0.2982***	-0.1791***	-0.0516	-0.1570**	-0.0877
	(0.0745)	(0.0815)	(0.0664)	(0.0874)	(0.0666)	(0.0547)

Note:
Standard errors in parentheses ()
*** p<0.01, ** p<0.05, * p<0.1 by Z-test

Table S98: Long Run Coefficient Estimation using Panel-Corrected Standard Errors with Health Vulnerability for SDG 7.3.1 – With Regulatory Quality, Rule of Law, and Voice and Accountability, Related to Figure 3

Variables	Model 1	Model 2	Model 1	Model 2	Model 1	Model 2
	Regulatory Quality		Rule of Law		Voice and Accountability	
Policy Stringency	0.0021	0.0306	0.0195	0.3202	0.0312	0.0520
	(0.0270)	(0.3626)	(0.0291)	(0.2093)	(0.0275)	(0.2069)
Environmental Innovation	-0.0074	-0.3890	0.0090	0.8496***	0.0282	-0.1055
	(0.0407)	(0.2858)	(0.0407)	(0.2102)	(0.0363)	(0.2716)
Foreign Direct Investment	-0.0910*	0.6002	-0.0824	0.0107	-0.0786	0.0808
	(0.0548)	(0.8017)	(0.0562)	(0.4293)	(0.0544)	(0.3654)
Industrial Share	1.2706***	1.9468***	1.3216***	-0.2816	1.0390***	1.1304*
	(0.1405)	(0.7266)	(0.1391)	(0.5593)	(0.1421)	(0.6526)
Energy Demand	-0.1844**	0.4095	-0.2266***	-1.4978***	-0.3826***	0.7440
	(0.0717)	(0.3243)	(0.0685)	(0.3514)	(0.0829)	(0.5974)
Health Vulnerability	-0.7439***	5.1700	-0.6064***	-11.7815***	-0.2752*	11.2570**
	(0.1575)	(4.0748)	(0.1541)	(4.3066)	(0.1666)	(5.7135)
Quality of Governance	-0.0629	-1.6444	-0.1263**	5.2428***	-0.2554***	-0.0789
	(0.0599)	(1.1342)	(0.0559)	(1.0561)	(0.0677)	(0.7848)
Policy Stringency * Health Vulnerability	-	0.0100	-	-0.4773	-	0.0472
	-	(0.7522)	-	(0.4228)	-	(0.4192)
Environmental Innovation * Health Vulnerability	-	0.8212	-	-1.3288***	-	0.4418
	-	(0.5906)	-	(0.3762)	-	(0.5402)
Foreign Direct Investment * Health Vulnerability	-	-1.6683	-	-0.3183	-	-0.4273
	-	(1.8115)	-	(0.9432)	-	(0.8487)
Industrial Share * Health Vulnerability	-	-0.6271	-	2.6634**	-	0.1715
	-	(1.6263)	-	(1.1424)	-	(1.3923)
Energy Demand * Health Vulnerability	-	-1.4179**	-	1.9949***	-	-2.3018*
	-	(0.6998)	-	(0.6811)	-	(1.2082)
Policy Stringency * Quality of Governance	-	1.0331	-	1.1412*	-	0.7624**
	-	(1.1596)	-	(0.6536)	-	(0.3819)
Environmental Innovation * Quality of Governance	-	-1.2993*	-	2.1697***	-	1.0153**
	-	(0.7682)	-	(0.5395)	-	(0.4476)
Foreign Direct Investment * Quality of Governance	-	1.4375	-	-0.7094	-	0.2543
	-	(2.1007)	-	(1.2009)	-	(0.5776)
Industrial Share * Quality of Governance	-	0.0848	-	3.5908***	-	2.2555**
	-	(1.7692)	-	(1.2947)	-	(0.9584)
Energy Demand * Quality of Governance	-	1.4054	-	-5.1874***	-	-2.3014***
	-	(1.0598)	-	(0.8761)	-	(0.8734)
Policy Stringency * Quality of Governance * Health Vulnerability	-	-1.6801	-	-2.0278	-	-1.5093*
	-	(2.4094)	-	(1.4488)	-	(0.8228)
Environmental Innovation * Quality of Governance * Health Vulnerability	-	3.3754**	-	-4.0657***	-	-1.8250**
	-	(1.6797)	-	(1.1680)	-	(0.9067)
Foreign Direct Investment * Quality of Governance * Health Vulnerability	-	-3.5054	-	2.9537	-	-0.5465

	-	(4.7299)	-	(3.1620)	-	(1.3803)
Industrial Share * Quality of Governance * Health Vulnerability	-	2.2058	-	-10.4113***	-	-4.5057**
	-	(4.0431)	-	(3.1127)	-	(2.1600)
Energy Demand * Quality of Governance * Health Vulnerability	-	-4.4575*	-	10.0035***	-	4.3612**
	-	(2.2748)	-	(2.0137)	-	(1.7889)
Constant	-0.7364**	-4.3773**	-0.8610***	6.0327***	0.8140	-6.0032**
	(0.3230)	(1.9225)	(0.2992)	(2.0542)	(0.5330)	(2.7817)
Wald-chi2	161.3	672.7	205.2	1122	253.9	625.8
Observations	115	115	115	115	115	115
Heteroskedasticity controlled	Yes	Yes	Yes	Yes	Yes	Yes
Autocorrelation controlled	Yes	Yes	Yes	Yes	Yes	Yes
Cross-sectional correlation controlled	Yes	Yes	Yes	Yes	Yes	Yes
Marginal Impact						
Policy Stringency	0.0021	0.0107	0.0195	-0.0098	0.0312	0.0051
	(0.0270)	(0.0313)	(0.0291)	(0.0369)	(0.0275)	(0.0356)
Environmental Innovation	-0.0074	-0.0439	0.0090	0.0488	0.0282	-0.0022
	(0.0407)	(0.0530)	(0.0407)	(0.0481)	(0.0363)	(0.0652)
Foreign Direct Investment	-0.0910*	-0.1478**	-0.0824	-0.2556***	-0.0786	-0.1387*
	(0.0548)	(0.0721)	(0.0562)	(0.0829)	(0.0544)	(0.0790)
Industrial Share	1.2706***	1.5261***	1.3216***	1.0604***	1.0390***	1.0087***
	(0.1405)	(0.1300)	(0.1391)	(0.1255)	(0.1421)	(0.1817)
Energy Demand	-0.1844**	-0.1668*	-0.2266***	-0.1647**	-0.3826***	-0.1087
	(0.0717)	(0.0881)	(0.0685)	(0.0767)	(0.0829)	(0.1337)

Note:
Standard errors in parentheses ()
*** p<0.01, ** p<0.05, * p<0.1 by Z-test

Table S99: Long Run Coefficient Estimation using Panel-Corrected Standard Errors with Infrastructure Vulnerability for SDG 7.3.1 – With Control of Corruption, Governance Effectiveness, and Political Stability, Related to Figure 3

Variables	Model 1	Model 2	Model 1	Model 2	Model 1	Model 2
	Control of Corruption		Governance Effectiveness		Political Stability	
Policy Stringency	0.0036	0.1991	0.0109	0.4585***	-0.0016	0.2141
	(0.0264)	(0.1265)	(0.0281)	(0.1252)	(0.0268)	(0.1767)
Environmental Innovation	0.0385	0.8128***	0.0241	0.1597	0.0183	0.0533
	(0.0423)	(0.2572)	(0.0437)	(0.1501)	(0.0407)	(0.3314)
Foreign Direct Investment	-0.0618	-0.1243	-0.0842	0.0773	-0.0845*	-1.1259
	(0.0505)	(0.3910)	(0.0530)	(0.2209)	(0.0506)	(0.7573)
Industrial Share	1.1465***	2.6138***	1.2799***	2.2280***	1.1671***	2.8060***
	(0.1307)	(0.4929)	(0.1320)	(0.4923)	(0.1303)	(0.7687)
Energy Demand	-0.1982***	-1.5255***	-0.1817***	-0.4551***	-0.1562**	-0.6548**
	(0.0697)	(0.2821)	(0.0636)	(0.1692)	(0.0624)	(0.3272)
Infrastructure Vulnerability	-1.7321***	2.8737	-1.9854***	14.1147*	-2.0087***	7.4122
	(0.3719)	(8.7238)	(0.3499)	(7.7398)	(0.3744)	(8.3370)
Quality of Governance	-0.0984*	-1.7201**	-0.0307	1.3146	-0.0622*	-0.1904
	(0.0505)	(0.7703)	(0.0483)	(1.0264)	(0.0342)	(0.6976)
Policy Stringency * Infrastructure Vulnerability	-	-0.5333	-	-2.3529***	-	-1.0857
	-	(0.8693)	-	(0.7339)	-	(1.2553)
Environmental Innovation * Infrastructure Vulnerability	-	-4.3647***	-	-0.7543	-	1.0794
	-	(1.5623)	-	(0.7112)	-	(2.0151)
Foreign Direct Investment * Infrastructure Vulnerability	-	0.6582	-	-1.3656	-	7.9084
	-	(2.7877)	-	(1.5788)	-	(5.0996)
Industrial Share * Infrastructure Vulnerability	-	-5.7533**	-	-5.4563**	-	-7.0699
	-	(2.8685)	-	(2.6601)	-	(4.6376)
Energy Demand * Infrastructure Vulnerability	-	7.0747***	-	1.7169**	-	0.6859
	-	(1.7964)	-	(0.8438)	-	(2.2869)
Policy Stringency * Quality of Governance	-	0.2269	-	0.5510	-	0.1474
	-	(0.3190)	-	(0.3694)	-	(0.2725)
Environmental Innovation * Quality of Governance	-	1.3971***	-	1.3627***	-	0.4754
	-	(0.4831)	-	(0.3861)	-	(0.3628)
Foreign Direct Investment * Quality of Governance	-	0.0998	-	0.7781	-	-1.2432
	-	(0.6641)	-	(1.0345)	-	(0.8692)
Industrial Share * Quality of Governance	-	1.5877**	-	2.5326***	-	1.3829**
	-	(0.7424)	-	(0.9335)	-	(0.6787)
Energy Demand * Quality of Governance	-	-2.1005***	-	-3.1018***	-	-1.1276***
	-	(0.5411)	-	(0.5365)	-	(0.3576)
Policy Stringency * Quality of Governance * Infrastructure Vulnerability	-	0.5262	-	-2.8142	-	-0.6117
	-	(2.2786)	-	(2.0613)	-	(1.7160)
Environmental Innovation * Quality of Governance * Infrastructure Vulnerability	-	-7.4213**	-	-7.5676***	-	-0.0305
	-	(3.2851)	-	(2.2906)	-	(2.0830)
Foreign Direct Investment * Quality of Governance * Infrastructure Vulnerability	-	0.8191	-	-2.7289	-	8.4344

	-	(5.4014)	-	(6.9157)	-	(5.3003)
Industrial Share * Quality of Governance * Infrastructure Vulnerability	-	-4.7440	-	-16.4033***	-	-5.2147
	-	(4.5481)	-	(4.8814)	-	(3.9747)
Energy Demand * Quality of Governance * Infrastructure Vulnerability	-	10.1846***	-	17.1908***	-	2.1176
	-	(3.7605)	-	(3.7318)	-	(2.2910)
Constant	-0.6719**	-2.6696*	-1.0545***	-4.0659***	-0.8192***	-3.1293**
	(0.2882)	(1.5394)	(0.2906)	(1.3505)	(0.2909)	(1.4383)
Wald-chi2	156.8	1837	229.8	1273	185.9	1968
Observations	115	115	115	115	115	115
Heteroskedasticity controlled	Yes	Yes	Yes	Yes	Yes	Yes
Autocorrelation controlled	Yes	Yes	Yes	Yes	Yes	Yes
Cross-sectional correlation controlled	Yes	Yes	Yes	Yes	Yes	Yes
Marginal Impact						
Policy Stringency	0.0036	0.0083	0.0109	0.0643*	-0.0016	0.0173
	(0.0264)	(0.0302)	(0.0281)	(0.0331)	(0.0268)	(0.0292)
Environmental Innovation	0.0385	-0.0005	0.0241	-0.0100	0.0183	-0.0371
	(0.0423)	(0.0508)	(0.0437)	(0.0602)	(0.0407)	(0.0555)
Foreign Direct Investment	-0.0618	-0.0953	-0.0842	-0.1615*	-0.0845*	0.0207
	(0.0505)	(0.0729)	(0.0530)	(0.0944)	(0.0506)	(0.0708)
Industrial Share	1.1465***	1.3783***	1.2799***	1.2551***	1.1671***	1.4063***
	(0.1307)	(0.0889)	(0.1320)	(0.1351)	(0.1303)	(0.1193)
Energy Demand	-0.1982***	-0.1723**	-0.1817***	-0.0688	-0.1562**	-0.1188*
	(0.0697)	(0.0706)	(0.0636)	(0.0879)	(0.0624)	(0.0622)

Note:
Standard errors in parentheses ()
*** p<0.01, ** p<0.05, * p<0.1 by Z-test

Table S100: Long Run Coefficient Estimation using Panel-Corrected Standard Errors with Infrastructure Vulnerability for SDG 7.3.1 – With Regulatory Quality, Rule of Law, and Voice and Accountability, Related to Figure 3

Variables	Model 1	Model 2	Model 1	Model 2	Model 1	Model 2
	Regulatory Quality		Rule of Law		Voice and Accountability	
Policy Stringency	0.0070	0.1539	0.0148	0.2491**	0.0315	-0.1115
	(0.0275)	(0.1310)	(0.0288)	(0.1079)	(0.0280)	(0.1241)
Environmental Innovation	0.0261	0.0479	0.0248	0.7253***	0.0438	-0.2760
	(0.0430)	(0.2282)	(0.0403)	(0.1868)	(0.0365)	(0.1764)
Foreign Direct Investment	-0.0831	-0.2877	-0.0798	0.1198	-0.0769	-0.2068
	(0.0535)	(0.4439)	(0.0538)	(0.2269)	(0.0538)	(0.1934)
Industrial Share	1.2642***	2.2985***	1.2865***	1.0642	1.0802***	1.7632***
	(0.1308)	(0.5448)	(0.1315)	(0.6591)	(0.1338)	(0.4560)
Energy Demand	-0.1926***	-0.3201	-0.1977***	-1.4337***	-0.3568***	0.6543*
	(0.0678)	(0.2521)	(0.0637)	(0.2660)	(0.0805)	(0.3404)
Infrastructure Vulnerability	-1.9981***	10.5346	-1.7936***	-12.8967	-1.1249***	32.3418***
	(0.3604)	(9.8089)	(0.4133)	(11.3228)	(0.3944)	(11.2526)
Quality of Governance	-0.0564	-1.3942	-0.0593	3.3034**	-0.2093***	1.1009
	(0.0568)	(1.0424)	(0.0608)	(1.3669)	(0.0631)	(0.8302)
Policy Stringency * Infrastructure Vulnerability	-	-0.7552	-	-1.2782**	-	0.8236
	-	(0.8461)	-	(0.5926)	-	(0.7017)
Environmental Innovation * Infrastructure Vulnerability	-	-0.0431	-	-3.0602***	-	2.2266**
	-	(1.3685)	-	(0.8554)	-	(0.9745)
Foreign Direct Investment * Infrastructure Vulnerability	-	1.3937	-	-1.3348	-	0.6764
	-	(3.1466)	-	(1.4678)	-	(1.3288)
Industrial Share * Infrastructure Vulnerability	-	-5.3137*	-	-0.4826	-	-2.9821
	-	(3.2272)	-	(3.1131)	-	(2.5997)
Energy Demand * Infrastructure Vulnerability	-	0.8215	-	5.8296***	-	-6.0049***
	-	(1.6668)	-	(1.4449)	-	(1.9942)
Policy Stringency * Quality of Governance	-	0.5358	-	0.4403*	-	0.5581***
	-	(0.4479)	-	(0.2655)	-	(0.1843)
Environmental Innovation * Quality of Governance	-	0.2275	-	1.1413***	-	1.2400***
	-	(0.7169)	-	(0.3569)	-	(0.3289)
Foreign Direct Investment * Quality of Governance	-	-1.0116	-	0.7743	-	0.2349
	-	(1.2308)	-	(0.5082)	-	(0.2623)
Industrial Share * Quality of Governance	-	1.9157	-	1.4626**	-	1.9025***
	-	(1.2526)	-	(0.7108)	-	(0.6199)
Energy Demand * Quality of Governance	-	-0.9985	-	-2.6571***	-	-2.5266***
	-	(0.7111)	-	(0.4291)	-	(0.5234)
Policy Stringency * Quality of Governance * Infrastructure Vulnerability	-	-2.5059	-	-1.9664	-	-2.8841***
	-	(3.0855)	-	(1.6332)	-	(0.9987)
Environmental Innovation * Quality of Governance * Infrastructure Vulnerability	-	0.8736	-	-4.8156**	-	-7.0834***
	-	(4.2861)	-	(2.0771)	-	(1.8781)
Foreign Direct Investment * Quality of Governance * Infrastructure Vulnerability	-	7.6899	-	-4.3217	-	-1.6043

	-	(8.6571)	-	(4.6460)	-	(1.6924)
Industrial Share * Quality of Governance * Infrastructure Vulnerability	-	-7.5367	-	-11.9487***	-	-12.6350***
	-	(7.1178)	-	(3.9302)	-	(3.2262)
Energy Demand * Quality of Governance * Infrastructure Vulnerability	-	2.4550	-	12.1344***	-	14.3619***
	-	(4.6171)	-	(2.7242)	-	(3.1151)
Constant	-0.9432***	-3.7519**	-1.0273***	2.0721	0.4453	-6.2679***
	(0.3059)	(1.6215)	(0.2930)	(2.2720)	(0.5366)	(2.0079)
Wald-chi2	222.0	781.2	247.2	1876	316.9	1351
Observations	115	115	115	115	115	115
Heteroskedasticity controlled	Yes	Yes	Yes	Yes	Yes	Yes
Autocorrelation controlled	Yes	Yes	Yes	Yes	Yes	Yes
Cross-sectional correlation controlled	Yes	Yes	Yes	Yes	Yes	Yes
Marginal Impact						
Policy Stringency	0.0070	0.0276	0.0148	-0.0184	0.0315	-0.0217
	(0.0275)	(0.0342)	(0.0288)	(0.0372)	(0.0280)	(0.0367)
Environmental Innovation	0.0261	-0.0031	0.0248	0.0632	0.0438	0.0038
	(0.0430)	(0.0616)	(0.0403)	(0.0521)	(0.0365)	(0.0518)
Foreign Direct Investment	-0.0831	-0.1179	-0.0798	-0.1771**	-0.0769	-0.1066
	(0.0535)	(0.0743)	(0.0538)	(0.0895)	(0.0538)	(0.0754)
Industrial Share	1.2642***	1.3992***	1.2865***	0.9509***	1.0802***	1.2164***
	(0.1308)	(0.1217)	(0.1315)	(0.1317)	(0.1338)	(0.1543)
Energy Demand	-0.1926***	-0.1304	-0.1977***	-0.1286*	-0.3568***	-0.1482
	(0.0678)	(0.0803)	(0.0637)	(0.0748)	(0.0805)	(0.0932)

Note:
Standard errors in parentheses ()
*** p<0.01, ** p<0.05, * p<0.1 by Z-test

Table S101: Long Run Coefficient Estimation using Panel-Corrected Standard Errors with Water Vulnerability for SDG 7.3.1 – With Control of Corruption, Governance Effectiveness, and Political Stability, Related to Figure 3

Variables	Model 1	Model 2	Model 1	Model 2	Model 1	Model 2
	Control of Corruption		Governance Effectiveness		Political Stability	
Policy Stringency	0.0107	0.5432**	0.0148	0.7267***	0.0035	0.3471
	(0.0282)	(0.2292)	(0.0299)	(0.1432)	(0.0282)	(0.2611)
Environmental Innovation	0.0234	1.1899***	-0.0160	0.7067***	-0.0172	0.5398
	(0.0429)	(0.2734)	(0.0438)	(0.1859)	(0.0403)	(0.3591)
Foreign Direct Investment	-0.0746	0.6310	-0.1039*	-0.3893	-0.1039*	-1.3508
	(0.0535)	(0.7239)	(0.0574)	(0.3599)	(0.0548)	(0.9560)
Industrial Share	1.2123***	1.7049***	1.3514***	2.3124***	1.2380***	2.7129***
	(0.1462)	(0.5842)	(0.1531)	(0.4895)	(0.1489)	(0.7032)
Energy Demand	-0.2050***	-1.9717***	-0.1494**	-0.4723**	-0.1290**	-0.5700
	(0.0743)	(0.4297)	(0.0662)	(0.2202)	(0.0646)	(0.4071)
Water Vulnerability	-0.6899***	-0.2629	-0.7412***	22.3134***	-0.7985***	17.6023***
	(0.2092)	(6.2536)	(0.1922)	(4.4110)	(0.2266)	(5.8469)
Quality of Governance	-0.1393***	0.7001	-0.0332	4.8211***	-0.0601	0.5682
	(0.0530)	(1.3837)	(0.0503)	(1.1427)	(0.0379)	(0.8531)
Policy Stringency * Water Vulnerability	-	-1.3551*	-	-1.9067***	-	-0.8237
	-	(0.7267)	-	(0.3760)	-	(0.7985)
Environmental Innovation * Water Vulnerability	-	-3.1557***	-	-2.2085***	-	-1.1042
	-	(0.8080)	-	(0.4675)	-	(1.0165)
Foreign Direct Investment * Water Vulnerability	-	-2.0199	-	1.2466	-	4.5364
	-	(2.2204)	-	(1.0773)	-	(3.0088)
Industrial Share * Water Vulnerability	-	-1.5252	-	-4.3649***	-	-3.6731
	-	(1.7099)	-	(1.2237)	-	(2.2844)
Energy Demand * Water Vulnerability	-	4.7583***	-	1.6444***	-	0.3831
	-	(1.2965)	-	(0.5484)	-	(1.2712)
Policy Stringency * Quality of Governance	-	0.5914	-	1.3636***	-	0.1865
	-	(0.5892)	-	(0.5185)	-	(0.3506)
Environmental Innovation * Quality of Governance	-	1.8262***	-	4.4139***	-	0.7809*
	-	(0.6425)	-	(0.7898)	-	(0.4052)
Foreign Direct Investment * Quality of Governance	-	1.8327	-	4.5257***	-	-1.1504
	-	(1.4123)	-	(1.4158)	-	(0.9487)
Industrial Share * Quality of Governance	-	-0.4243	-	0.6590	-	0.5761
	-	(1.2241)	-	(1.2737)	-	(0.7894)
Energy Demand * Quality of Governance	-	-2.2230**	-	-6.5091***	-	-1.2467**
	-	(0.9016)	-	(0.9279)	-	(0.4887)
Policy Stringency * Quality of Governance * Water Vulnerability	-	-1.2521	-	-3.1333**	-	-0.3806
	-	(1.8200)	-	(1.4122)	-	(0.9633)
Environmental Innovation * Quality of Governance * Water Vulnerability	-	-4.6432**	-	-12.9791***	-	-1.4019
	-	(1.9569)	-	(2.2659)	-	(1.0345)
Foreign Direct Investment * Quality of Governance * Water Vulnerability	-	-5.8092	-	-14.8331***	-	4.0528

	-	(4.7015)	-	(4.4738)	-	(2.7885)
Industrial Share * Quality of Governance * Water Vulnerability	-	1.4655	-	-4.7591	-	-0.5661
	-	(4.3126)	-	(3.7101)	-	(2.3707)
Energy Demand * Quality of Governance * Water Vulnerability	-	5.2641*	-	18.6326***	-	1.8273
	-	(2.9445)	-	(2.8646)	-	(1.3329)
Constant	-0.7844**	-0.5777	-1.2668***	-8.6970***	-1.0123***	-7.3157***
	(0.3060)	(2.6316)	(0.3277)	(1.6393)	(0.3259)	(2.1268)
Wald-chi2	116.7	1769	161.6	1356	132.7	1966
Observations	115	115	115	115	115	115
Heteroskedasticity controlled	Yes	Yes	Yes	Yes	Yes	Yes
Autocorrelation controlled	Yes	Yes	Yes	Yes	Yes	Yes
Cross-sectional correlation controlled	Yes	Yes	Yes	Yes	Yes	Yes
Marginal Impact						
Policy Stringency	0.0107	0.0072	0.0148	0.0149	0.0035	0.0324
	(0.0282)	(0.0301)	(0.0299)	(0.0288)	(0.0282)	(0.0312)
Environmental Innovation	0.0234	-0.0032	-0.0160	-0.2012***	-0.0172	0.0156
	(0.0429)	(0.0447)	(0.0438)	(0.0546)	(0.0403)	(0.0521)
Foreign Direct Investment	-0.0746	-0.0166	-0.1039*	-0.0779	-0.1039*	0.0251
	(0.0535)	(0.0688)	(0.0574)	(0.0814)	(0.0548)	(0.0652)
Industrial Share	1.2123***	1.1300***	1.3514***	0.7232***	1.2380***	1.2058***
	(0.1462)	(0.1071)	(0.1531)	(0.1443)	(0.1489)	(0.1147)
Energy Demand	-0.2050***	-0.1449**	-0.1494**	0.2892***	-0.1290**	-0.1353**
	(0.0743)	(0.0695)	(0.0662)	(0.0892)	(0.0646)	(0.0540)

Note:
Standard errors in parentheses ()
*** p<0.01, ** p<0.05, * p<0.1 by Z-test

Table S102: Long Run Coefficient Estimation using Panel-Corrected Standard Errors with Water Vulnerability for SDG 7.3.1 – With Regulatory Quality, Rule of Law, and Voice and Accountability, Related to Figure 3

Variables	Model 1	Model 2	Model 1	Model 2	Model 1	Model 2
	Regulatory Quality		Rule of Law		Voice and Accountability	
Policy Stringency	0.0109	-0.0269	0.0179	0.3672***	0.0308	-0.0567
	(0.0290)	(0.2066)	(0.0298)	(0.1292)	(0.0276)	(0.1765)
Environmental Innovation	-0.0077	0.2059	0.0074	1.1467***	0.0245	-0.1974
	(0.0436)	(0.2437)	(0.0418)	(0.1867)	(0.0355)	(0.2612)
Foreign Direct Investment	-0.1017*	-1.7962**	-0.0800	0.0447	-0.0790	-0.4804*
	(0.0573)	(0.8141)	(0.0573)	(0.3286)	(0.0565)	(0.2838)
Industrial Share	1.3239***	2.1554***	1.2757***	1.5915***	0.9752***	1.5764***
	(0.1507)	(0.6170)	(0.1463)	(0.5048)	(0.1442)	(0.5776)
Energy Demand	-0.1699**	0.3281	-0.2008***	-1.4294***	-0.4046***	0.7920
	(0.0738)	(0.4006)	(0.0701)	(0.2999)	(0.0844)	(0.5389)
Water Vulnerability	-0.7918***	18.3294***	-0.4447**	9.4092	0.0625	18.9464***
	(0.2151)	(5.2726)	(0.2049)	(6.2711)	(0.2182)	(7.3211)
Quality of Governance	-0.0770	-0.0189	-0.1519***	3.1229***	-0.3068***	1.1605*
	(0.0647)	(1.4126)	(0.0571)	(1.1307)	(0.0714)	(0.7023)
Policy Stringency * Water Vulnerability	-	0.1671	-	-0.7850**	-	0.1008
	-	(0.6145)	-	(0.3355)	-	(0.4193)
Environmental Innovation * Water Vulnerability	-	-0.6785	-	-2.4917***	-	0.6757
	-	(0.6507)	-	(0.4128)	-	(0.6237)
Foreign Direct Investment * Water Vulnerability	-	5.5237**	-	-0.1697	-	1.2504
	-	(2.5334)	-	(0.8976)	-	(0.7851)
Industrial Share * Water Vulnerability	-	-2.1064	-	-2.2383*	-	-1.9313
	-	(1.9023)	-	(1.1740)	-	(1.4302)
Energy Demand * Water Vulnerability	-	-1.2511	-	2.6031***	-	-2.6626**
	-	(1.1532)	-	(0.7526)	-	(1.3263)
Policy Stringency * Quality of Governance	-	0.1408	-	0.6669	-	0.9824***
	-	(0.7268)	-	(0.4320)	-	(0.3371)
Environmental Innovation * Quality of Governance	-	0.7853	-	1.8964***	-	1.5880***
	-	(0.7484)	-	(0.5264)	-	(0.4771)
Foreign Direct Investment * Quality of Governance	-	-4.1967**	-	0.7201	-	0.0505
	-	(1.9214)	-	(0.8503)	-	(0.4068)
Industrial Share * Quality of Governance	-	-1.0660	-	2.7076***	-	2.1857**
	-	(1.6444)	-	(1.0134)	-	(1.0166)
Energy Demand * Quality of Governance	-	-0.2136	-	-4.0134***	-	-3.1205***
	-	(0.9510)	-	(0.6442)	-	(0.7515)
Policy Stringency * Quality of Governance * Water Vulnerability	-	0.5609	-	-1.3936	-	-2.4615***
	-	(2.1889)	-	(1.1580)	-	(0.8482)
Environmental Innovation * Quality of Governance * Water Vulnerability	-	-1.9189	-	-3.9580***	-	-3.8668***
	-	(2.0349)	-	(1.3196)	-	(1.1753)
Foreign Direct Investment * Quality of Governance * Water Vulnerability	-	13.7504**	-	-1.3755	-	-0.0614

	-	(6.4253)	-	(2.6836)	-	(1.1046)
Industrial Share * Quality of Governance * Water Vulnerability	-	5.5485	-	-8.9932***	-	-6.3863**
	-	(5.4317)	-	(2.9826)	-	(2.5988)
Energy Demand * Quality of Governance * Water Vulnerability	-	-1.1837	-	8.9709***	-	7.7068***
	-	(3.0093)	-	(1.7346)	-	(1.8685)
Constant	-1.0841***	-8.3251***	-0.9987***	-2.8204	1.0450*	-7.1253**
	(0.3358)	(2.0035)	(0.3126)	(2.4961)	(0.5797)	(2.9031)
Wald-chi2	153.9	1150	164.9	2574	239.1	1293
Observations	115	115	115	115	115	115
Heteroskedasticity controlled	Yes	Yes	Yes	Yes	Yes	Yes
Autocorrelation controlled	Yes	Yes	Yes	Yes	Yes	Yes
Cross-sectional correlation controlled	Yes	Yes	Yes	Yes	Yes	Yes
Marginal Impact						
Policy Stringency	0.0109	-0.0114	0.0179	0.0162	0.0308	-0.0421
	(0.0290)	(0.0287)	(0.0298)	(0.0369)	(0.0276)	(0.0405)
Environmental Innovation	-0.0077	-0.0267	0.0074	0.0558	0.0245	0.0007
	(0.0436)	(0.0536)	(0.0418)	(0.0454)	(0.0355)	(0.0574)
Foreign Direct Investment	-0.1017*	-0.0531	-0.0800	-0.1040	-0.0790	-0.0407
	(0.0573)	(0.0701)	(0.0573)	(0.0656)	(0.0565)	(0.0603)
Industrial Share	1.3239***	1.2441***	1.2757***	0.7948***	0.9752***	0.9072***
	(0.1507)	(0.1171)	(0.1463)	(0.1076)	(0.1442)	(0.1339)
Energy Demand	-0.1699**	-0.0342	-0.2008***	-0.1238*	-0.4046***	-0.0807
	(0.0738)	(0.0803)	(0.0701)	(0.0675)	(0.0844)	(0.1015)
Note:<br>Standard errors in parentheses ()<br>*** p<0.01, ** p<0.05, * p<0.1 by Z-test						

Table S103: Results of Dumitrescu and Hurlin [13] Causality test, Related to Figure 3

From	To	Z-bar tilde	p-value
SDG 7.1.1	Electricity Generation	1.2860	0.1985
	Financial Access	2.7908	0.0053
	Rural Population Growth	1.2946	0.1955
	Income Inequality	-0.9170	0.3592
	Health Expenditure	3.9931	0.0001
	Ecosystem Vulnerability	0.8897	0.3736
	Food Vulnerability	1.2085	0.2268
	Health Vulnerability	0.9392	0.3476
	Infrastructure Vulnerability	1.1874	0.2351
	Water Vulnerability	1.4051	0.1600
	Control of Corruption	1.0509	0.2933
	Governance Effectiveness	1.2123	0.2254
	Political Stability	1.5324	0.1254
	Regulatory Quality	0.7261	0.4678
	Rule of Law	-0.1431	0.8862
	Voice and Accountability	0.1425	0.8867
SDG 7.1.2	Renewable Electricity Gen.	3.4893	0.0005
	Financial Access	1.1084	0.2677
	Policy Stringency	1.3516	0.1765
	Per Capita Income	0.9651	0.3345
	Urban Population Growth	2.5493	0.0108
	Ecosystem Vulnerability	-0.0185	0.9853
	Food Vulnerability	1.4045	0.1602
	Health Vulnerability	1.9472	0.0515
	Infrastructure Vulnerability	1.3961	0.1627
	Water Vulnerability	1.5137	0.1301
	Control of Corruption	1.1576	0.2470
	Governance Effectiveness	1.7822	0.0747
	Political Stability	0.5777	0.5635
	Regulatory Quality	4.0288	0.0001
	Rule of Law	0.6530	0.5137
	Voice and Accountability	1.0001	0.3173
SDG 7.2.1	Policy Stringency	-0.1516	0.8795
	Environmental Innovation	0.7907	0.4291
	Financial Development	-0.8198	0.4123
	Net Energy Import	0.3554	0.7223
	Low Carbon Trade	0.9955	0.3195
	Ecosystem Vulnerability	-0.0698	0.9443
	Food Vulnerability	1.1373	0.2554
	Health Vulnerability	1.1149	0.2649
	Infrastructure Vulnerability	-0.2119	0.8322
	Water Vulnerability	1.1261	0.2601
	Control of Corruption	1.2767	0.2017
	Governance Effectiveness	1.3644	0.1725
	Political Stability	0.7626	0.4457
	Regulatory Quality	1.9465	0.0516
	Rule of Law	-0.1298	0.8967
	Voice and Accountability	0.8544	0.3929
SDG 7.3.1	Policy Stringency	0.6797	0.4967
	Environmental Innovation	-0.6104	0.5416
	Foreign Direct Investment	1.4008	0.1613
	Industrial Share	1.1190	0.2631
	Energy Demand	-0.3637	0.7161
	Ecosystem Vulnerability	0.7565	0.4494
	Food Vulnerability	-0.1004	0.9200
	Health Vulnerability	1.2480	0.2120
	Infrastructure Vulnerability	0.8063	0.4201
	Water Vulnerability	1.3730	0.1697
	Control of Corruption	0.9556	0.3393
	Governance Effectiveness	0.1339	0.8935
	Political Stability	0.0080	0.9936
	Regulatory Quality	0.8404	0.4007
	Rule of Law	0.8260	0.4088
	Voice and Accountability	0.7866	0.4315

References

1. Sachs, J.D., Lafortune, G., Fuller, G., and Drumm, E. (2023). Sustainable Development Report 2023: Implementing the SDG Stimulus (Dublin University Press) https://doi.org/10.25546/102924.
2. International Energy Agency (2023). Energy intensity. SDG7 Database. https://www.iea.org/data-and-statistics/data-product/sdg7-database#energy-intensity.
3. United Nations Statistics Division (2024). Total Electricity. Energy Stat. Database. https://data.un.org/Data.aspx?d=EDATA&f=cmID%3AEL.
4. World Bank (2023). World Development Indicators. https://data.worldbank.org/indicator.
5. World Bank (2022). Global Financial Development Database. https://www.worldbank.org/en/publication/gfdr/data/global-financial-development-database.
6. World Inequality Lab (2024). World Inequality Database. https://wid.world/.
7. International Energy Agency (2023). Energy Statistics Data Browser. https://www.iea.org/data-and-statistics/data-tools/energy-statistics-data-browser?country=WORLD&fuel=Renewables and waste&indicator=RenewGenBySource.
8. OECD (2025). OECD Statistics. Organ. Econ. Co-operation Dev. https://stats.oecd.org/.
9. International Monetary Fund (2023). Trade in Low Carbon Technology Products. https://climatedata.imf.org/datasets/1d33174e9e46429d9e570d539556f66a_0/about?deliveryName=DM15392.
10. Ember (2023). Electricity Data Explorer. https://ember-energy.org/data/electricity-data-explorer/.
11. World Bank (2023). Worldwide Governance Indicators. https://databank.worldbank.org/source/worldwide-governance-indicators.
12. ND-GAIN (2023). Country Index 2023: Vulnerability and Readiness Scores. Notre Dame Glob. Adapt. Initiat. https://gain.nd.edu/our-work/country-index/.
13. Dumitrescu, E.I., and Hurlin, C. (2012). Testing for Granger non-causality in heterogeneous panels. Econ. Model. *29*, 1450–1460. https://doi.org/10.1016/j.econmod.2012.02.014.
